# LivAge: An Online Aging Clock for Murine Transcriptomic Age Estimation

**DOI:** 10.1111/acel.70677

**Published:** 2026-08-21

**Authors:** Víctor Celemín‐Capaldi, Guillermina Bea, David Roiz‐Valle, Alejandro P. Ugalde, Clea Bárcena, Pedro M. Quirós, José M. P. Freije

**Affiliations:** ^1^ Departamento de Bioquímica y Biología Molecular Instituto Universitario de Oncología (IUOPA), Universidad de Oviedo Oviedo Spain; ^2^ Instituto de Investigación Sanitaria del Principado de Asturias, ISPA Oviedo Spain; ^3^ Centro de Investigación Biomédica en Red de Cáncer (CIBERONC) Madrid Spain

**Keywords:** aging, aging clocks, biomarkers, machine learning, progeria, transcriptome

## Abstract

The increase in life expectancy over the past century has been accompanied by the recognition of age as the primary risk factor for a wide range of pathologies, including cardiovascular and neurodegenerative diseases, as well as cancer. Thus, the development of interventions that slow the underlying biological processes of aging could have beneficial effects on the prevention or progression of these diseases. To evaluate such geroprotective interventions, quantifying biological damage via aging clocks—particularly those based on transcriptomic biomarkers—has become highly relevant, as they provide estimations with strong biological interpretability. However, the limited availability of transcriptomic clocks for murine experimental models has hindered the implementation of these tools in aging research and their use in evaluating interventions that may have geroprotective effects. Here, we have developed an accurate, accessible, and ready‐to‐use murine transcriptomic clock that provides robust age predictions for healthy mice using hepatic RNA‐seq data. Applying the clock to accelerated‐aging models revealed an increased transcriptomic age in progeroid syndromes, demonstrating its ability to capture aging‐related biological processes. Finally, considering the main interest of these tools, we show that well‐established interventions with geroprotective potential, both genetic (Snell Dwarf, Ames Dwarf, and growth hormone receptor‐deficient mice) and environmental (caloric, protein, or methionine restriction, as well as rapamycin supplementation in specific contexts), reduce the transcriptomic age estimated by the clock. These findings indicate that the proposed transcriptomic clock could be a valuable tool for studying the biology of aging and for designing and evaluating potential geroprotective interventions.

## Graphical abstract

We present LivAge, an online aging clock that estimates murine transcriptomic age from hepatic RNA‐seq data. Externally validated, it detects accelerated aging in progeroid models and quantifies the effect of geroprotective interventions, providing an accurate, accessible, and ready‐to‐use tool for aging research.

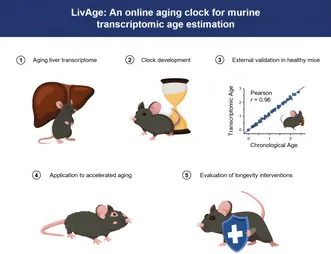

## 1 Introduction

Aging can be defined as the “process resulting from accumulation of consequences of life, such as molecular and cellular damage, that leads to increased risk of functional decline, chronic diseases, and ultimately mortality” (Moqri et al. 2024). A conceptual and integrative view of this biological phenomenon led to the proposal of hallmarks of aging: processes that manifest during physiological aging, whose experimental accentuation accelerates aging, while their experimental attenuation decelerates it; and which can be classified as primary, antagonistic, and integrative (López‐Otín et al. 2023). The progressive development of these hallmarks causes a gradual deterioration in the integrity and adaptability of cells, tissues, and organs, boosting the manifestation and progression of most human diseases at advanced chronological ages (Balachandran et al. 2025). Consequently, the rise in lifespan over the past century has highlighted aging as the main risk factor for many age‐associated pathologies, including cancer, cardiovascular diseases, and neurodegenerative disorders (Niccoli and Partridge 2012). In this context, prophylactic or therapeutic interventions—whether pharmacological, dietary, or related to lifestyle—that slow the progression of the biological hallmarks of aging, rather than targeting individual age‐related diseases, may protect against or alleviate a wide range of pathologies, as proposed by the geroscience hypothesis (Kroemer et al. 2025; Sierra and Kohanski 2017).

The design and evaluation of interventions aimed at mitigating the biological processes of aging require tools that can quantify age‐dependent molecular, cellular, and physiological damage (Moqri et al. 2024, 2023). In recent years, significant progress has been made in the development of so‐called “aging clocks”, computational machine learning models whose outputs are intended to reflect the level of aging of an organism (Galkin et al. 2020). In this way, the changes in DNA methylation patterns that occur with aging have been used to build epigenetic clocks, available for both humans (Hannum et al. 2013; Levine et al. 2018; Rutledge et al. 2022; Horvath 2013) and mice (Lu et al. 2022; Meer et al. 2018; Petkovich et al. 2017; Stubbs et al. 2017; Thompson et al. 2018). However, despite their remarkable development and usefulness, one of the most relevant challenges of epigenetic clocks is the biological interpretability of the methylation changes: it is often unclear whether they represent causal drivers of aging, neutral effects, or adaptive responses, especially considering the quasi‐stochastic epigenetic drift that occurs with advancing age (Ying et al. 2024; Teschendorff and Horvath 2025).

In this context, transcriptomic aging clocks—by directly employing age‐associated changes in gene expression—have a closer relation with cellular function, which would facilitate the biological interpretability of predictions (Rutledge et al. 2022). Thus, human transcriptomic clocks have been developed for blood cells (Peters et al. 2015), dermal fibroblasts (Fleischer et al. 2018; Meyer and Schumacher 2021) and other tissues (Ren and Kuan 2020; Shokhirev and Johnson 2021). Moreover, some authors have proposed the use of other types of computational tools far from the frequent linear regression models, such as neural networks (Holzscheck et al. 2021; Qi et al. 2025), or sample matching based on the correlation between expression profiles (Bulteau and Francesconi 2022). Despite this progress, the development and adoption of transcriptomic clocks is not comparable to that of epigenetic clocks. Some possible reasons include their lower performance, which in some cases is accompanied by a lack of validation using external samples and standardized protocols, as well as the lack of user‐friendly software (Qi et al. 2025). Furthermore, the higher sensitivity of gene expression to environmental factors, despite being of interest in the evaluation of interventions with geroprotective potential, also introduces additional variability and noise that are less present in methylation data (Salignon et al. 2025). Finally, the generation of transcriptomic aging clocks for experimental models of aging, such as *Mus musculus* , is even more limited, which hinders the study of aging risk factors and, especially, the evaluation of geroprotective interventions.

To address these limitations and explore the implementation of these tools in *M. musculus* , we present an aging clock capable of detecting the transcriptomic age of murine liver samples with high precision and accuracy. Using a strategy based on differential expression analysis of liver RNA‐seq data from multiple studies, we developed an accessible, ready‐to‐use, and web‐based platform for calculating transcriptomic ages. We demonstrate the utility of the clock by applying it to accelerated‐aging mouse models, detecting an increase in the estimated age in progeroid syndromes, confirming the clock's ability to capture age‐related biological processes. Moreover, we show that several genetic and environmental interventions can reduce the estimated transcriptomic age of murine models, thereby reinforcing their geroprotective potential. Consequently, our clock offers an interactive and statistically robust tool for predicting the transcriptomic age of murine liver samples and for evaluating interventions with geroprotective potential.

## 2 Results

### 2.1 Generation of a Transcriptomic Atlas of Liver Aging in Mice

**FIGURE 1:**
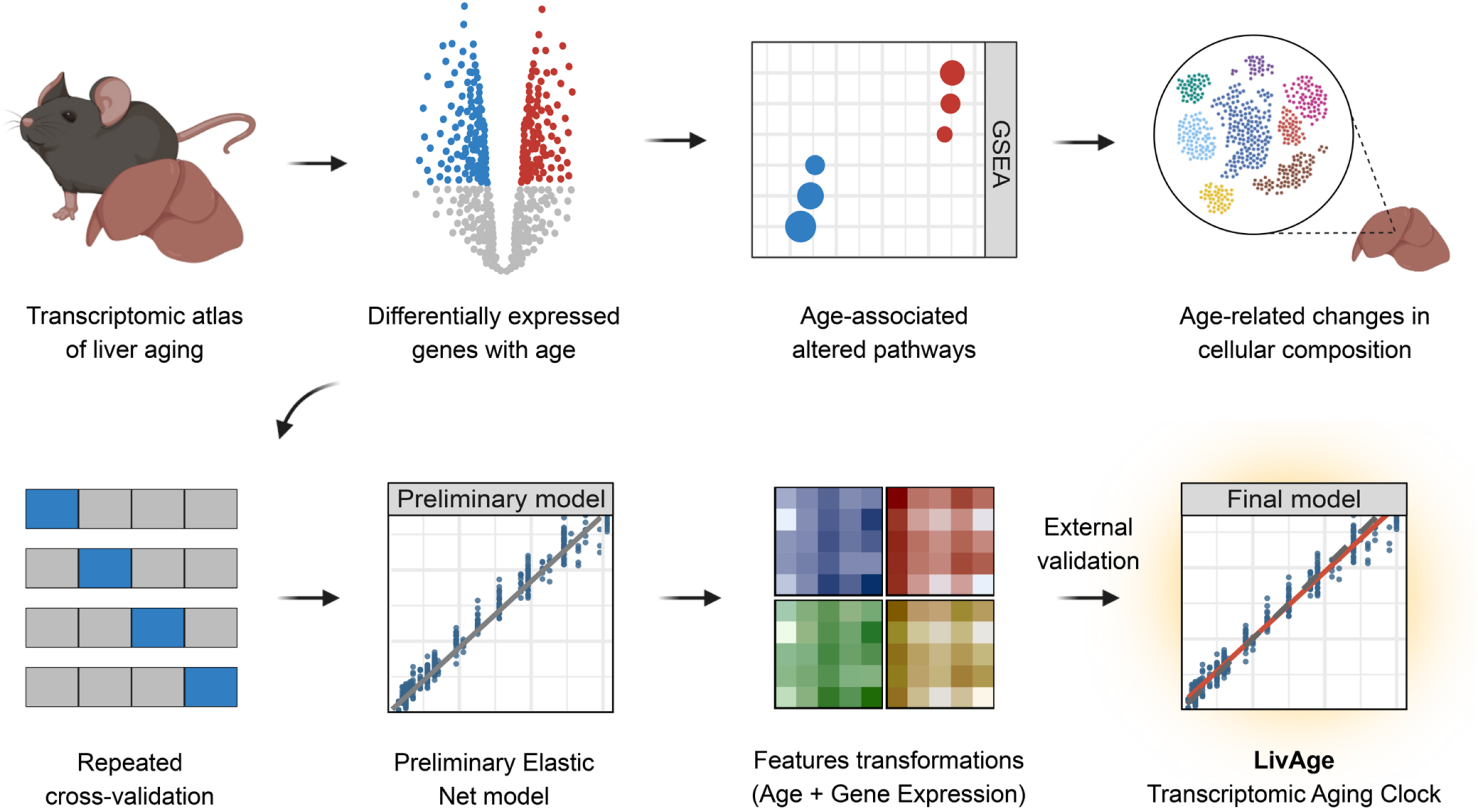
Workflow for the development of the LivAge transcriptomic clock. RNA‐seq samples from livers of different chronological ages were first compiled into a transcriptomic atlas of aging. Differentially expressed genes with age were selected and used for functional analyses, including gene set enrichment analysis (GSEA) and cellular composition estimation via deconvolution. Moreover, genes with the strongest age‐related changes were then used to perform repeated cross‐validation on the atlas data to train a preliminary elastic net regression model. Numeric transformations of the features (age and gene expression) were subsequently applied to optimize predictive performance to construct the final transcriptomic clock. Finally, the model was validated on an independent external dataset.

With the aim of training and evaluating a transcriptomic aging clock, we first downloaded and processed publicly available RNA‐seq data from liver samples of different chronological ages, generating a transcriptomic atlas of liver aging in mice. This dataset served as the basis for the construction of the murine transcriptomic clock, together with the selection of genes differentially expressed with advancing age, the implementation of a repeated cross‐validated elastic net model, and variable transformation (Figure 1). Accordingly, murine transcriptomic studies providing raw sequencing files (FASTQ format) and associated metadata on age and experimental conditions were collected. Only healthy samples from control groups, free of any reported genetic modifications or environmental interventions, were retained for further analysis. After ensuring optimal quality of the data, we proceeded with alignment and quantification of the raw reads, yielding gene counts that were subsequently normalized and filtered to remove low expression genes, ensuring robust downstream analysis (see Methods). The resulting transcriptomic repository, derived from 23 independent studies, comprised 432 liver samples from mice of C57BL/6 genetic background ranging in age from 1 to 30 months, including 365 males and 67 females. These samples served as the basis for analyzing molecular signatures of aging and for developing a murine transcriptomic clock (Figure S1a,b and Table S1).

**FIGURE 2:**
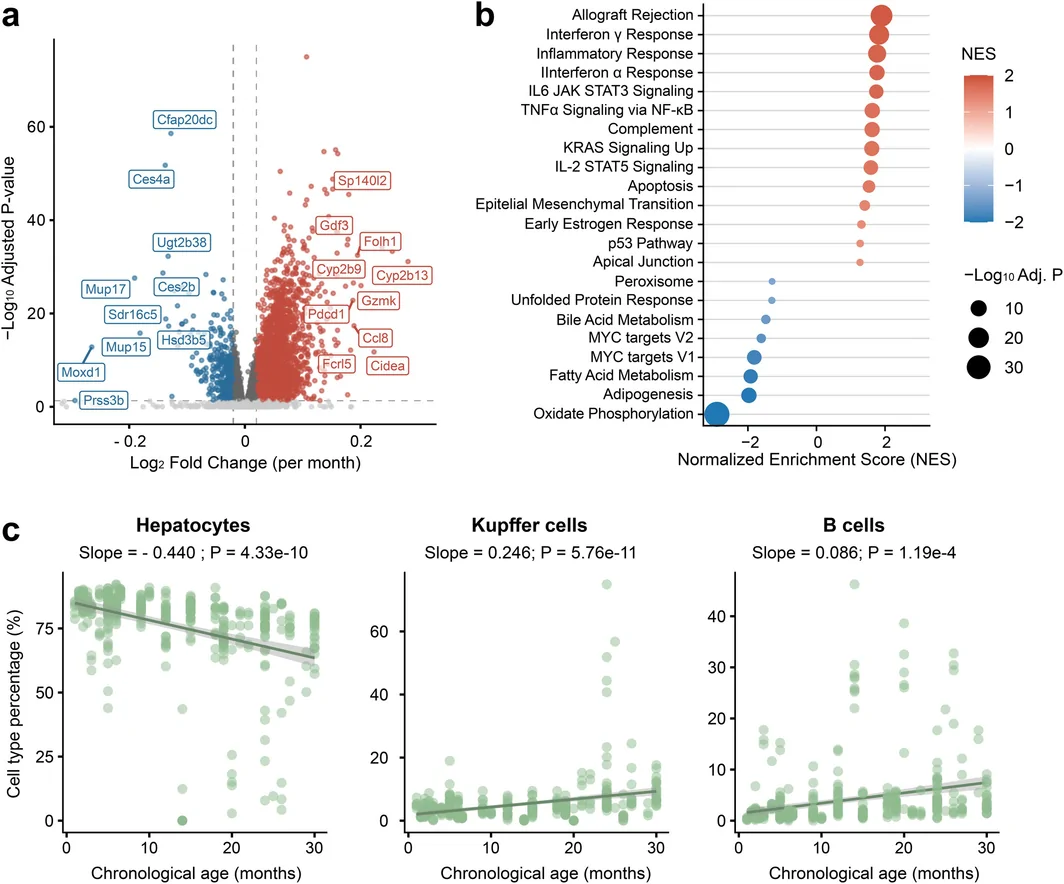
Differential expression analysis in a transcriptomic atlas of liver aging in mice. (a) Volcano plot showing differential gene expression across aging. Each dot represents a gene, plotted according to log2 fold change (*X*‐axis) and –log10 adjusted *p*‐value (*Y* axis). The statistical significance threshold was set at an adjusted *p*‐value of 0.05, while the fold‐change threshold was set at an absolute log2 fold change per month of 0.02. In this regard, genes significantly upregulated are shown in red, while significantly downregulated genes are shown in blue. Genes showing minimal expression changes are shown in black, and non‐significant genes are shown in gray. Selected differentially expressed genes are highlighted and labeled. (b) Gene Set Enrichment Analysis (GSEA) of Hallmark Pathways. The *X*‐axis represents the Normalized Enrichment Score (NES), where positive values (Red) indicate a significant activation of the pathways and negative values (Blue) indicate a significant repression, whereas the *Y*‐axis lists the biological pathways analyzed. The bubble plot displays the results of the GSEA performed on the MSigDB Hallmark gene set collection and is proportional to the statistical significance of the enrichment, represented as the transformed value of –log10 adjusted *p*‐value; a larger bubble size signifies higher statistical significance. (c) Changes in liver cell composition during aging. Scatter plots displaying the relative proportions of hepatocytes (left), Kupffer cells (middle), and B cells (right) in each sample, as estimated by cell type deconvolution of bulk RNA‐seq data. The *X*‐axis represents the chronological age of the samples (in months), and the *Y*‐axis shows the predicted percentage of each cell type. Each green dot corresponds to an individual RNA‐seq sample, while the solid line represents the fitted linear regression, with the shaded area indicating the 95% confidence interval. For each cell type, the slope of the regression and the corresponding *p*‐value are indicated.

To identify transcriptomic features of aging within our large dataset, we performed a differential expression analysis. This allowed us to confirm the ability to detect in our transcriptomic repository specific changes in gene expression and molecular pathways typically associated with aging. Using an FDR threshold of 0.05 and a log_2_ fold change per month of chronological age cutoff greater than 0.02 or less than −0.02, we detected 3350 genes whose expression significantly changed with chronological age after adjusting for study and sex (Figure 2a). Among the upregulated genes, the cytotoxic effector *Gzmk*, the inflammatory and immune‐related gene *Ccl8*, and the lipid droplet gene *Cidea* were notably upregulated. In contrast, multiple members of the Major Urinary Protein (MUP) family (*Mup6*, *Mup11*, *Mup13*, *Mup15*, and *Mup17*), together with the carboxylesterases *Ces2b* and *Ces4a* and the metabolic enzyme *Hsd3b5*, were among the most significantly downregulated genes (Data S1).

Next, we performed a Gene Set Enrichment Analysis (GSEA) to identify biologically related gene sets that were overrepresented among genes with aging‐associated changes (Figure 2b and Data S2). In this regard, we could observe that pathways with a positive association (positive normalized enrichment score, NES) were primarily involved in immunity and inflammation, highlighting pathways such as Allograft Rejection (NES = 1.90; FDR = 3.02 × 10^−24^), Interferon Gamma Response (NES = 1.83; FDR = 7.66 × 10^−20^), and Interferon Alpha Response (NES = 1.77; FDR = 4.17 × 10^−9^), as well as the Inflammatory Response (NES = 1.77; FDR = 1.82 × 10^−14^) and IL6 JAK STAT3 Signaling (NES = 1.74; FDR = 2.85 × 10^−8^). Conversely, pathways that showed a negative association (negative NES)—indicating significant downregulation or depletion—included those related to energy metabolism, with Oxidative Phosphorylation (NES = −2.90; FDR = 2.97 × 10^−35^), Adipogenesis (NES = −1.97; FDR = 4.17 × 10^−9^) and Fatty Acid Metabolism (NES = −1.92; FDR = 2.01 × 10^−7^) being the most significantly repressed. These results collectively suggest an activation of immune and inflammatory responses concurrently with a repression of energy and lipid metabolism (Figure 2b). Finally, considering the activation of these aging‐associated molecular pathways and aiming to gain a deeper cellular perspective of chronological aging, we applied cell deconvolution methods to bulk RNA‐seq data to explore potential changes in the liver's cellular landscape during aging (Wang et al. 2019). The analyses revealed a progressive relative decline in the hepatocyte population (Slope = −0.440, *p* = 4.33 × 10^−10^), accompanied by a concomitant proportional increase in immune cell infiltration, specifically Kupffer cells (Slope = 0.246, *p* = 5.46 × 10^−11^) and B cells (Slope = 0.086, *p* = 1.19 × 10^−4^) (Figure 2c and Data S3). Together, these observations reinforce the development of a pro‐inflammatory and dysfunctional environment in the liver during aging, in agreement with the GSEA results.

### 2.2 Development of a Murine Transcriptomic Aging Clock

After confirming that our transcriptomic atlas could capture aging‐associated signatures and metabolic pathways—such as a metabolic decline and the emergence of pro‐inflammatory and senescent landscapes—we proceeded to train a predictive model for estimating transcriptomic age, employing this atlas as the training set of samples. Based on previous transcriptomic clock studies, we selected the Elastic Net algorithm for age prediction because it provides a balance between feature selection and regularization (Ren and Kuan 2020; Jung et al. 2023; Meyer and Schumacher 2021). Since the number of variables (genes) far exceeded the number of samples, we first filtered genes based on the differential expression analysis conducted on the training set, selecting only those genes with an adjusted *p*‐value lower than 0.25. After selecting these genes, a repeated cross‐validation scheme was implemented to ensure robust hyperparameter optimization of the predictive model. Through this approach, we generated an aging clock to predict transcriptomic age, with performance metrics obtained upon completion of the training and testing process (Figure S2a). However, due to potential overfitting in the assessment of these metrics, we evaluated the generalization capacity of the predictive model on a second set of 39 independent samples drawn from 8 non‐overlapping studies that were entirely distinct from those used during the cross‐validation. For this purpose, the raw sequencing reads were downloaded and, after ensuring their optimal quality, were subjected to the same processing pipeline employed for the training data. Remarkably, the clock retained high predictive accuracy across these independent datasets (Figure S2b).

**FIGURE 3:**
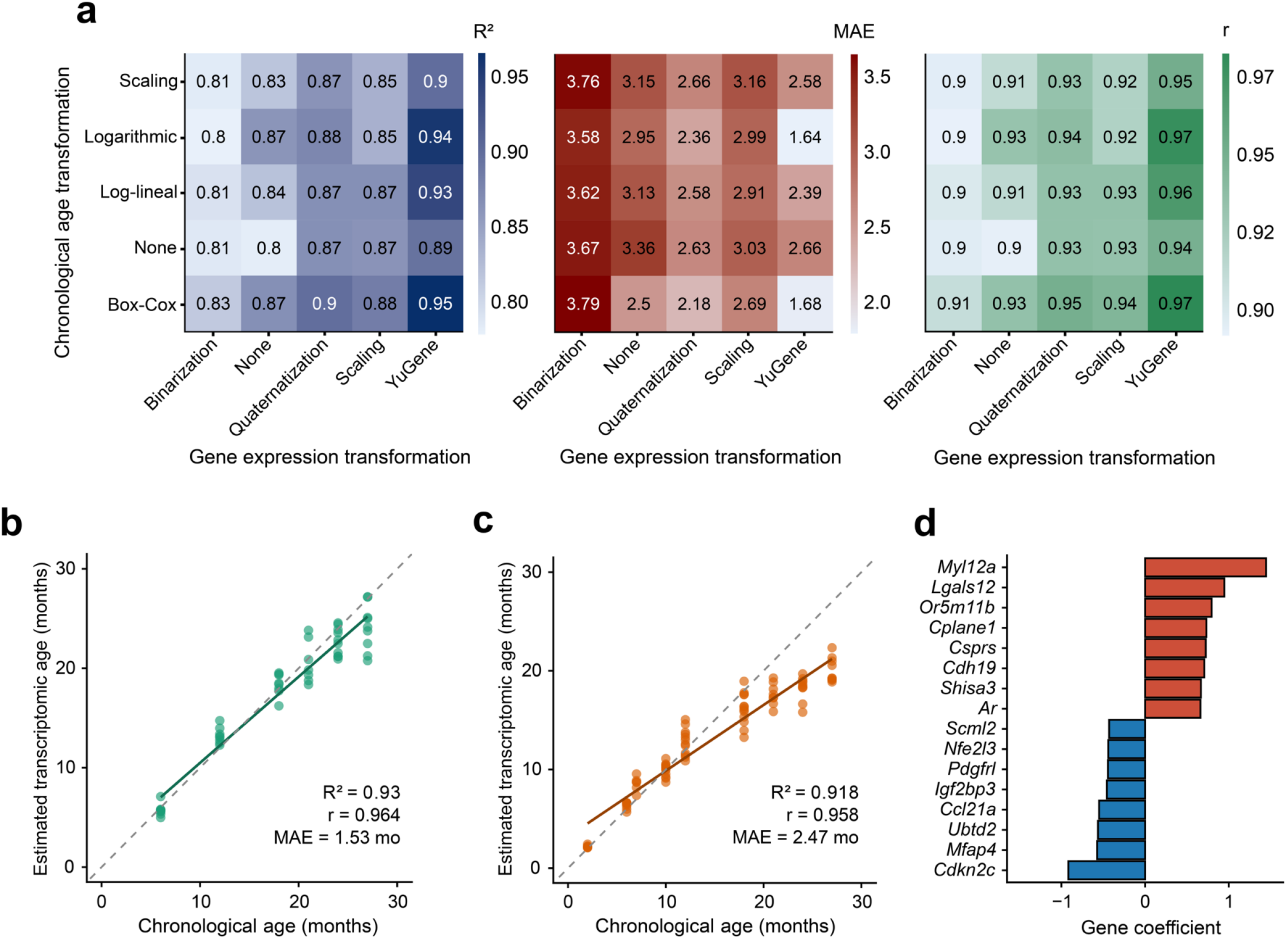
Development of a murine transcriptomic aging clock. (a) Regression model performance matrices after applying different combinations of chronological age and gene expression transformations. These heatmaps illustrate the performance of a predictive regression model, measured by *R* ^2^, MAE and *r*. The *X*‐axis (Transformation of gene expression) represents the transformations applied to the gene expression (Binarization, No transformation, Quaternarization, Scaling, and YuGene), whereas the *Y*‐axis (Transformation of age) shows the transformations applied to the chronological age before model training (Scaling, Logarithmic, Log‐lineal, No transformation, and Box‐Cox). The numerical value and color intensity within each cell denote the resulting metric; from low (lighter colors) to high values (darker colors). (b) External validation of the optimized transcriptomic aging clock in male mice. Scatter plot showing the results from an independent set of 50 external RNA‐seq samples. The *X*‐axis represents chronological age (months), and the *Y*‐axis shows transcriptomic age predicted by the model. Each dot corresponds to a sample. The dashed gray line indicates perfect prediction, and the solid red line is the regression line. Performance metrics include *R* ^2^, Pearson's *r*, and MAE. (c) External validation of the optimized transcriptomic aging clock in female mice. Scatter plot showing the results from an independent set of 84 external RNA‐seq samples. The *X*‐axis represents chronological age (months), and the *Y*‐axis shows transcriptomic age predicted by the model. Each dot corresponds to a sample. The dashed gray line indicates perfect prediction, and the solid red line is the regression line. Performance metrics include *R* ^2^, Pearson's *r*, and MAE. (d) Top gene predictors of the transcriptomic aging clock. Bar plot shows the coefficients assigned to the most influential predictor genes within the aging clock. The *Y*‐axis displays these genes, while the *X*‐axis represents the model coefficients derived from Elastic Net regression. Genes are ordered by their predictive weight: Positive values (red) contribute to an older age prediction, whereas negative values (blue) contribute to a younger age prediction.

Given the good statistical performance of the predictive model, we explored whether numerical transformation of the variables could further improve the predictive power of the transcriptomic clock. In this sense, the chronological age transformations evaluated included logarithmic, scaling, the Box‐Cox transformation and the log‐linear conversion proposed by Steve Horvath in the development of his first epigenetic clock (Horvath 2013). For gene‐expression data, we tested normalization methods such as YuGene transformation and scaling (Lê Lê Cao et al. 2014). In addition, considering the potentially positive impact of categorizing gene expression, we examined the effect of performing binarization and quaternization of the data based on the median gene expression per sample (Meyer and Schumacher 2021). To evaluate the impact of the different transformations, we utilized the second set of samples that had been excluded from the cross‐validation process. This strategy allowed us to observe that the approach based on the Box‐Cox transformation of the chronological age and the YuGene normalization of gene expression further increased the performance of the transcriptomic clock, in comparison with the alternative approaches (Figure 3a). Finally, to assess the final performance of the resulting transcriptomic clock, we estimated chronological age across a third set of 134 samples pooled from 4 distinct studies that had been excluded from both the cross‐validation and the variables transformation evaluation. In this sense, the model yielded statistical parameters comparable to those observed in epigenetic clocks, accurately predicting the ages of both male (*R* ^2^ = 0.930; Pearson's *r* = 0.964; MAE = 1.53; Figure 3b), and female samples (*R* ^2^ = 0.918; Pearson's *r* = 0.958; MAE = 2.47) (Figures 3c, S2c and Data S4). Moreover, we benchmarked the final predictive model against two published transcriptomic clock strategies—“TACO” multi‐tissue and liver specific machine learning models for *M. musculus* and RAPToR, a reference‐based transcriptome‐matching method (Tyshkovskiy et al. 2026; Bulteau and Francesconi 2022)—on this same set of samples, revealing that none of these tools surpassed the statistical performance of our clock (Figure S3 and Data S5).

Therefore, the transcriptomic aging clock relies on the expression of 268 predictor genes, which together capture age‐associated transcriptional changes and enable a robust and accurate estimation of transcriptomic age (Figure 3d and Table S2). Furthermore, given the progressive relative decline in the hepatocyte population and the proportional increase in immune cell fractions with age revealed by the cellular deconvolution analysis (Figure 2c), we next assessed the impact of these compositional shifts on the estimates produced by the developed transcriptomic clock. To account for the compositional (sum‐to‐one) constraint of the cell‐fraction estimates, we modeled predicted transcriptomic age as a function of the isometric log‐ratio (ILR) transformed hepatocyte, Kupffer, B‐cell, NK‐cell, and endothelial cell proportions in the third set of samples. The model explained a significant fraction of the variance in estimated age (*R* ^2^ = 0.352), indicating that shifts in the relative balance of liver cell‐type composition account for roughly 35% of the variance in transcriptomic age estimates (Data S6). Nevertheless, because most of the variance remains unexplained by cellular composition alone, this suggests the aging clock captures complex liver transcriptomic changes during aging, rather than simply tracking changes in cell populations.

### 2.3 Progeria Mouse Models Exhibit Accelerated Transcriptomic Age

**FIGURE 4:**
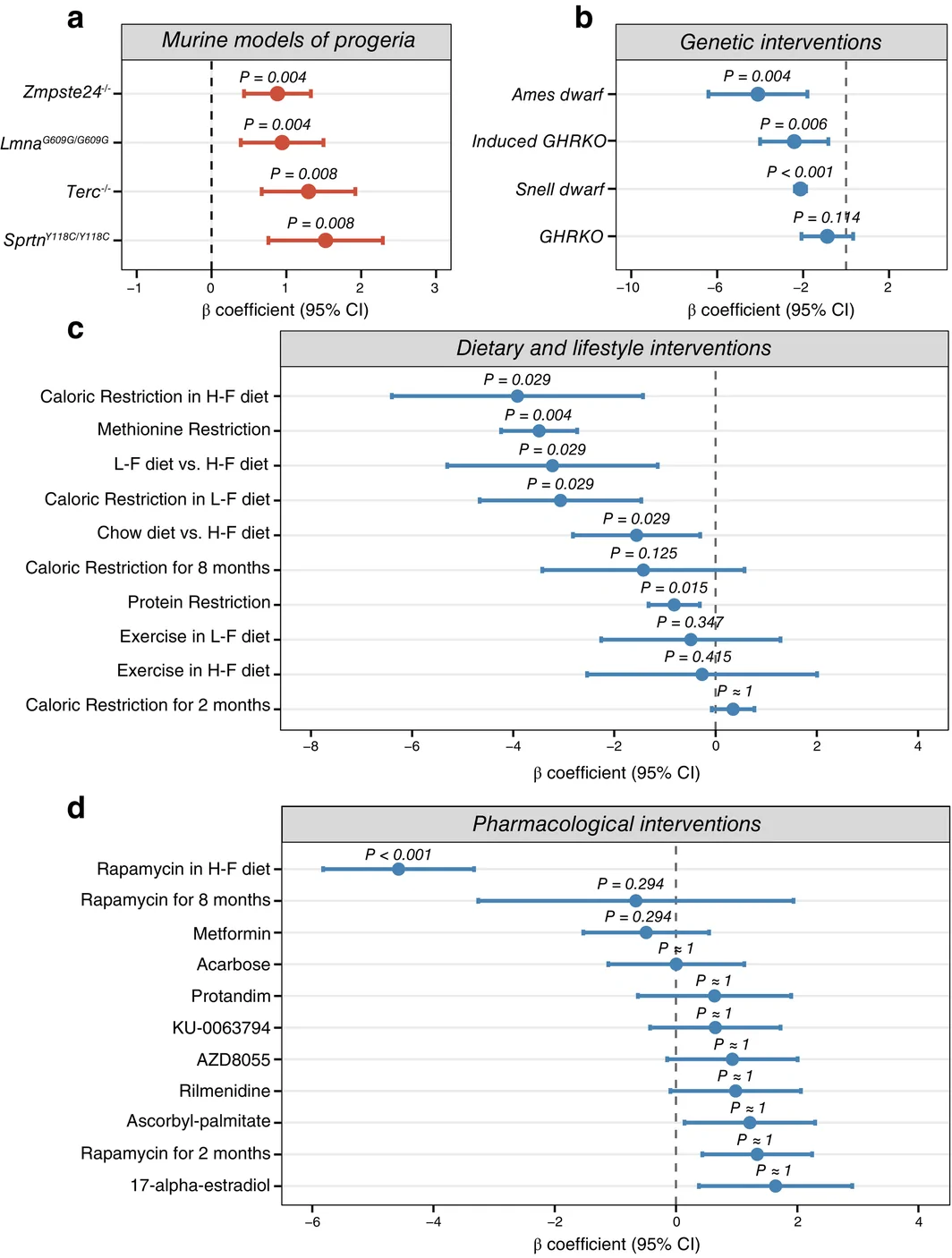
Forest plots showing the effects of multiple genetic and environmental interventions in transcriptomic age. Each plot displays the β coefficients from linear regression models with AgeDev (difference between estimated biological age and chronological age, in months) as the dependent variable and experimental group as the independent variable, adjusted for potential covariates. Horizontal lines represent 95% confidence intervals around each *β* coefficient. Positive *β* values indicate accelerated aging, whereas negative *β* values indicate geroprotective effects. One‐tailed *p*‐values, displayed at the top of each point, were assessed using linear regression and corrected by the Benjamini‐Hochberg false discovery rate (FDR) procedure within each intervention category. Panels correspond to different intervention types: (a) murine models of progeria: *Lmna* ^*G609G/G609G*^ (*n* = 20), *Zmpste24* ^*−/−*^ (*n* = 12), *Terc* ^*−/−*^ (*n* = 6) and *Sprtn* ^*Y118C/Y118C*^ (*n* = 7); (b) genetic interventions: Ames Dwarf (*n* = 16), iGHRKO (*n* = 20), GHRKO (*n* = 6) and Snell Dwarf (*n* = 6); (c) dietary and lifestyle interventions: Protein restriction (*n* = 16), normal vs. H‐F diet (*n* = 15), caloric restriction in L‐F diet (*n* = 6), caloric restriction in H‐F diet (*n* = 6), L‐F diet vs. H‐F diet (*n* = 6), caloric restriction for 8 months (*n* = 12), caloric restriction for 2 months (*n* = 12), exercise in H‐F diet (*n* = 6), exercise in L‐F diet (*n* = 6) and methionine restriction (*n* = 6); (d) pharmacological interventions: Rapamycin in H‐F diet (*n* = 16), metformin (*n* = 6), acarbose (*n* = 24), protandim (*n* = 18), KU‐0063794 (*n* = 12), AZD8055 (*n* = 12), rilmenidine (*n* = 12), ascorbyl‐palmitate (*n* = 12), rapamycin for 2 months (*n* = 12), rapamycin for 8 months (*n* = 12) and 17‐alpha‐estradiol (*n* = 24).

After confirming that the transcriptomic clock predicts age with high precision in healthy mice, we evaluated its performance on mouse models of accelerated aging to determine whether it captures biological aging processes rather than merely reflecting chronological age. Accelerated aging or progeroid syndromes constitute a group of rare diseases that faithfully reproduce many of the systemic alterations typical of aging (Carrero et al. 2016). One of the most representative cases is Hutchinson‐Gilford progeria syndrome (HGPS), characterized by defects in nuclear envelope integrity caused by a mutation in the *LMNA* gene. The murine models *Lmna* ^*G609G/G609G*^ and *Zmpste24* ^*−/−*^, previously generated in our laboratory, constitute highly valuable tools for analyzing pathological aging and its modulation (Pendás et al. 2002; Osorio et al. 2011). To quantify differences in transcriptomic aging, we compared transcriptomic age estimates between murine models of accelerated aging and wild‐type controls using linear regression. Beta coefficients represent the mean difference between estimated transcriptomic age and chronological age, also referred to as age deviation (AgeDev) or age acceleration (Horvath 2013) (Figure 4a). Using this approach, we observed a significant acceleration of transcriptomic age in *Zmpste24* ^*−/−*^ mice (*β* = 0.881, false discovery rate (FDR) = 0.004), as well as in *Lmna* ^*G609G/G609G*^ mice (*β* = 0.943, FDR = 0.004). Besides nuclear envelope alterations, other progeroid syndromes—such as Dyskeratosis congenita and Hoyeraal‐Hreidarsson syndrome—are caused by mutations in different components of the telomerase complex (Carrero et al. 2016). Accordingly, mice genetically deficient in the RNA component of telomerase (*Terc* ^*−/−*^) exhibit accelerated telomere shortening and a reduced lifespan owing to the premature onset of age‐associated diseases (Herrera et al. 1999). We therefore evaluated whether this model exhibits changes in transcriptomic age by analyzing an external RNA‐seq dataset (Ferrara‐Romeo et al. 2020). The accelerated aging phenotype was indeed captured by our transcriptomic aging clock, which detected a higher transcriptomic age deviation in progeroid samples compared to their wild‐type counterparts (*β* = 1.296, FDR = 0.008). Another progeroid disorder related to genome stability is Ruijs–Aalfs syndrome, which is caused by biallelic mutations in the *Sprtn* gene, a key player in DNA repair that resolves DNA–protein crosslinks (Tomaskovic et al. 2026). When we evaluated the transcriptomic age of its murine model (*Sprtn* ^*Y118C/Y118C*^), we confirmed accelerated transcriptomic aging: mutant mice were found to be significantly older than their wild‐type littermates (*β* = 1.524, FDR = 0.008) (Figure 4a). Therefore, the increase in age deviation detected in these progeroid syndromes by the developed aging clock supports the accelerated aging characteristics observed in these experimental models (Data S4 and S7).

### 2.4 Certain Genetic Longevity Interventions Reduce Transcriptomic Age

Beyond the elevated transcriptomic age observed in murine models of accelerated aging and considering the potential utility of this tool for evaluating geroprotective interventions, we investigated the effects of genetic, lifestyle‐related, and pharmacological interventions on transcriptomic age. In this context, *Prop1*‐deficient (also known as Ames dwarf mice), *Pit1*‐deficient mice (Snell dwarf mice), and growth hormone receptor–deficient mice (GHRKO) mice exhibit extended lifespan and a delayed onset of age‐associated decline (Coschigano et al. 2000; Boylston et al. 2006; Brown‐Borg et al. 1996). In this sense, Ames dwarf mice showed negative age acceleration compared to control mice (*β* = −4.101, FDR = 0.004) (Figure 4b). Moreover, consistent with the geroprotective effects observed in Snell dwarf mice, this model also demonstrated a significant deceleration of transcriptomic age (*β* = −2.519, FDR < 0.001) (Figure 4b). Finally, to investigate effects on transcriptomic aging of growth hormone receptor deficiency, we analyzed RNA‐seq data from a study profiling gene expression in GHRKO mice. However, global GHRKO mice displayed only a non‐significant trend toward reduced age acceleration (*β* = −1.337, FDR = 0.114). Therefore, we decided to analyze the impact of other murine models with GHR deficiency, such as the tamoxifen‐inducible global GH receptor knockout model, in which deletion was induced at 12 months and mice were followed until 24 months of age, detecting a negative age deviation in these mice, compared to their wild‐type counterparts (*β* = −2.412, FDR = 0.006) (Figure 4b and Data S4 and S7) (Poudel et al. 2024).

### 2.5 Decelerating Estimated Age Through Diet and Pharmacological Strategies

In addition to genetic interventions with potential geroprotective effects, several environmental interventions have been linked to beneficial effects on health status and to a slower pace of aging in murine models. Among these, dietary strategies have emerged as a major avenue for improving health and extending longevity (Longo and Anderson 2022). Caloric restriction (CR), in particular, is associated with lifespan extension and with delayed onset and progression of age‐related diseases in mice (Green et al. 2022). In this context, we detected a decrease in transcriptomic age deviation following an 8‐month period of 40% CR relative to animals fed a standard diet, which showed a trend toward significance (*β* = −1.428, FDR = 0.125). By contrast, a shorter CR regime (2 months) did not yield a detectable shift in transcriptomic age (*β* = 0.346, FDR ≈1) (Figure 4c). It is also well established that the composition and proportion of specific macronutrients in the diet influence aging biomarkers and the risk of age‐related diseases. For instance, low dietary fat intake has been associated with extended lifespan and improved healthspan in mice, in contrast to the deleterious effects observed with increased dietary fat content (Muller et al. 2013). Consistent with these findings, our clock revealed a pronounced reduction of estimated transcriptomic age in mice fed a low‐fat (LF) diet compared with mice maintained on a high‐fat (HF) diet (*β* = −3.224, FDR = 0.029). Indeed, a similar effect was observed when mice were maintained on a standard chow diet compared with those fed a high‐fat diet (*β* = −1.562, FDR = 0.029) (Havas et al. 2026). Remarkably, the CR‐associated transcriptomic shift previously described was preserved even under HF diet conditions, resulting in a reduction of predicted transcriptomic age compared with HF‐fed mice without CR (*β* = −3.917, FDR = 0.029). A similar pattern was observed under LF diet conditions, where CR induced a further reduction of transcriptomic age in mice relative to LF controls (*β* = −3.066, FDR = 0.029) (Figure 4c) (Green et al. 2017). Beyond calorie intake and lipid proportion, other macronutrients also modulate healthspan and lifespan of mice. For instance, protein restriction has been linked to reduced incidence of age‐related diseases and to increased lifespan (Solon‐Biet et al. 2015). To validate the geroprotective effects of this dietary‐based intervention, we evaluated its impact on transcriptomic age using an additional external study (Pak et al. 2019). A low‐protein diet reduced the estimated transcriptomic age deviation compared with mice maintained on a standard diet (*β* = −0.819, FDR = 0.015). Likewise, restriction of specific amino acids, such as branched‐chain amino acids (BCAAs; leucine, isoleucine, and valine) or methionine, has also been associated with geroprotective effects (Miller et al. 2005). Accordingly, methionine restriction (MR) decreased transcriptomic age deviation relative to mice on a standard diet (β = −3.917, FDR = 0.004) (Figure 4c). In addition to the potential geroprotective effect of these dietary interventions, other strategies based on pharmacological compounds have been reported to produce beneficial effects on healthspan and lifespan in murine models. Using RNA‐seq data from different studies in which several compounds were tested, we found that rapamycin treatment for 2 months in mice with a C57/BL6 genetic background fed with a high‐fat diet led to a significant reduction in transcriptomic age (*β* = −4.574, FDR < 0.001) (Figure 4d) (Havas et al. 2026). We also analyzed data from two transcriptomic studies evaluating multiple pharmacological interventions in UM‐HET3 mice. While non‐significant trends were observed for metformin (*β* = −0.491, FDR = 0.294) and rapamycin treatment over 8 months (*β* = −0.661, FDR = 0.294), no reductions in estimated transcriptomic age were detected in treated mice compared with their respective controls for the other compounds (Data S4 and S7).

Finally, to facilitate the application of the developed transcriptomic clock, and given its relevance for the evaluation of pro‐ and anti‐aging interventions, we developed a web‐based platform for the LivAge predictive model. Users can upload an RNA‐seq matrix with raw counts, and the platform automatically applies the Elastic Net model to predict transcriptomic ages for the samples of interest. The platform provides a rapid, accessible, and reproducible application of the transcriptomic clock, allowing efficient assessment of transcriptomic age across the samples.

## 3 Discussion

The recognition that aging is the main risk factor for numerous diseases has fueled the search for geroprotective interventions that can ultimately slow or prevent these pathologies. Transcriptomic aging clocks are powerful tools for evaluating such strategies because they reflect tissue function and the underlying biological processes that occur during aging. However, their broader use has been limited by modest predictive performance, the many factors that influence gene expression, and the scarcity of mouse‐specific clocks. In the present work, we have developed a liver‐specific transcriptomic aging clock that (i) captures the biological pathways of aging, (ii) detects the impact of lifespan‐shortening interventions on transcriptomic age, (iii) quantifies the effects of genetic, dietary, and pharmacological interventions with geroprotective potential, and (iv) is implemented in a web‐based platform, enabling accurate, accessible, and rapid estimation of transcriptomic ages.

We began by downloading and processing 432 RNA‐seq liver samples from C57BL/6 mice ranging from 1 to 30 months of age. This transcriptomic repository of aging revealed canonical signatures of aging and liver diseases, including loss of major urinary protein expression (Takasugi et al. 2024); downregulation of the carboxylesterases (*Ces2b* and *Ces4a*) (Lian and Nelson 2018; Mitra et al. 2020); reduced expression of *Hsd3b5* (Guillén et al. 2009); and upregulation of *Cidea, Gpnmb*, and *Gzmk* (Yu et al. 2024; Carr et al. 2014; Mogilenko et al. 2021). Gene‐set enrichment analysis and cellular deconvolution confirmed an “inflammaging” phenotype (Rea et al. 2018), characterized by elevated interferon and interleukin signaling, activation of allograft rejection, and an increased proportion of B cells and Kupffer cells. Conversely, we observed a downregulation of oxidative phosphorylation, adipogenesis, and fatty acid metabolism, which have been linked to age‐related decline and increased mortality (Tyshkovskiy et al. 2023).

Using the aggregated data, we constructed and validated a transcriptional aging clock through a strategy that incorporated differential expression analysis to reduce the number of genes input to the machine‐learning model. A relative permissive threshold (FDR < 0.25) was chosen because overly restrictive gene selection can impair the statistical performance of predictive models (Ren and Kuan 2020). Two key preprocessing steps markedly improved performance on optimization set: chronological age transformation with the Box‐Cox method, which accounts for the non‐linear trajectories of gene‐expression changes with age (Schaum et al. 2020), and the YuGene normalization of expression values, which reduce transcriptomic technical noise generated by aggregating data from different studies (Lê Cao et al. 2014). Unlike the “BiT Age” transcriptomic clock, the binarization of gene expression did not improve the predictive capacity of our model; this discrepancy may arise from differences in data preprocessing, as the BiT Age study did not apply prefiltering based on differential expression. Finally, to determine the final performance and generalization capability of the transcriptomic clock, we estimated the age of an external validation sample set that was excluded from both the cross‐validation and the optimization of the clock. Having confirmed the high precision of our clock predicting age in healthy mice, we tested whether it could capture both accelerated and decelerated aging (Justice et al. 2018) by estimating AgeDev. This metric quantifies the difference between predicted and chronological age and is widely used for evaluating biomarkers of aging (Moqri et al. 2023). Accordingly, an effective aging clock should detect a higher estimated age to progeroid models, as they recapitulate many of the characteristic alterations of aging at an accelerated pace (Carrero et al. 2016). When we evaluated the transcriptomic age of two widely used murine models of progeria—*Zmpste24* ^*−/−*^ and *Lmna* ^*G609G/G609G*^ mice (Pendás et al. 2002; Osorio et al. 2011)—we detected an acceleration of the estimated age. Similar results were obtained in other models of accelerated aging, such as *Terc* ^*−/−*^ and *Sprtn* ^*Y118C/Y118C*^ mice, which suffer from genomic‐stability defects (Folgueras et al. 2018). These results demonstrate that our clock captures biological aging processes rather than merely tracking chronological age, a limitation that is often observed with certain epigenetic clocks (Rutledge et al. 2022). Notably, several epigenetic clocks fail to discriminate progeroid *Lmna* ^*G609G/G609G*^ mice from controls (Perez et al. 2023), although differences have been reported in humans depending on the epigenetic clock employed (Horvath et al. 2018), underscoring the added sensitivity of our transcriptomic clock.

The potential of our clock was then tested in mice with lifespan‐extending genetic modifications, such as deficiencies in *Prop1*, *Pit1* or *Ghr*. Ames Dwarf, Snell Dwarf, and inducible GHRKO mice showed a significant reduction in transcriptomic age, consistent with their delayed age‐related phenotypes (Coschigano et al. 2000; Boylston et al. 2006; Brown‐Borg et al. 1996). Constitutive GHRKO mice displayed a marginal reduction trend, possibly reflecting strain‐specific background effects, which has also been reported for epigenetic clocks (Sandoval‐Sierra et al. 2020). In addition to certain genetic interventions, caloric intake and dietary composition are well‐established modulators of aging and longevity (Green et al. 2022). In this sense, we demonstrate that both CR and macronutrient diet proportion significantly influence the estimated transcriptomic age in murine models. Consistent with previous works (Di Francesco et al. 2024; Colman et al. 2014; Flanagan et al. 2020), mice under an 8‐month CR regime exhibited a marked deceleration of transcriptomic aging compared to those fed a standard diet, whereas a 2‐month CR did not yield this effect, highlighting the importance of the duration of the regime (Prosczek et al. 2025). Even on a high‐fat diet, which is associated with increased risk of obesity, metabolic complications, and a higher estimated transcriptomic age according to our clock, CR is sufficient to offset the HFD‐associated increase in estimated age, bringing the transcriptomic profile closer to that of non‐HFD‐fed animals (Muller et al. 2013). Low‐protein or methionine–restricted diets also decelerate transcriptomic aging, in line with previous reports of their geroprotective benefits (Mirzaei et al. 2014; Orgeron et al. 2014).

Furthermore, several drugs targeting aging‐related pathways which have been shown to increase longevity and improve healthspan in murine models were also tested (Harrison et al. 2014; Miller et al. 2014; Strong et al. 2016). Rapamycin, an inhibitor of the mTOR pathway, reduced transcriptomic age in a context of a high fat diet, consistent with its reported geroprotective and lifespan‐extending effects (Havas et al. 2026). In contrast, analysis of transcriptomic data from UM‐HET3 mice did not reveal significant changes in transcriptomic age following pharmacological interventions, aside from non‐significant trends observed for metformin and rapamycin. As observed with previously analyzed interventions, the greater heterogeneity in the effects observed may be explained by the use of mouse strains different from the C57BL/6 background employed to train the transcriptomic clock (Mulvey et al. 2014; Sandoval‐Sierra et al. 2020). In this regard, among the caloric restriction interventions evaluated in the present study, significant reductions in transcriptomic age were observed only in mice with a C57BL/6 genetic background (C. D. Green et al. 2017), whereas such decreases were not as evident in UM‐HET3 mice (Tyshkovskiy et al. 2019). Another possible explanation is that aging clocks integrate changes in gene expression or methylation across multiple molecular pathways, whereas the more specific modes of action of certain pharmacological interventions may limit the detection of a global rejuvenation effect (Ham et al. 2022). Finally, similar to what has been reported for other interventions, the timing and duration of the drug exposure, as well as the pharmacological dose employed, represent critical determinants for achieving maximal geroprotective efficacy (Prosczek et al. 2025; Shindyapina et al. 2022).

The development of murine transcriptomic aging clocks is highly relevant because mice remain a vital experimental model for aging research (Folgueras et al. 2018). Moreover, the ability to identify predictive genes whose expression changes substantially with aging may facilitate the identification and characterization of gerogenes and gerosuppressors, thereby contributing to the design of novel geroprotective interventions (Kroemer et al. 2025). In this regard, our liver‐derived clock exhibits a high predictive performance in estimating transcriptomic age of healthy mice, detects accelerated aging in progeroid and genomic‐instability models, and enables the evaluation of longevity interventions. However, the present study has certain limitations. First, although differential expression analysis allows for the prior selection of genes whose expression varies significantly with age, independently of other factors such as sex, it remains necessary to investigate the impact of the limited homogeneity in age and sex distribution within the aging transcriptomic atlas. A slight sexual dimorphism was observed in the prediction accuracy, where male samples exhibited a lower mean absolute error (MAE = 1.53 months) compared to female samples (MAE = 2.47 months). This is consistent with previously described evidence regarding the existence of sex‐specific transcriptomic trajectories during aging (Schaum et al. 2020; Shavlakadze et al. 2026), although the lower representation of females in the training set may also have contributed to this reduced accuracy. Second, although all RNA‐seq samples used to train the clock were derived from C57BL/6 mice, differences among substrains may affect predictions in a manner analogous to that observed between distinct genetic strains (Mulvey et al. 2014; Daniel et al. 2026). Batch effects and other technical artifacts may be inadvertently captured by the model, reducing its generalizability and compromising the accuracy (Francesconi and Lehner 2015), particularly for estimating absolute transcriptomic age (Moqri et al. 2023). Although unknown biological factors influencing aging cannot be excluded, these too could compromise accuracy and explain certain unexpected deviations. This may ultimately hinder the clock's ability to detect the true impact of geroprotective interventions that act through specific molecular pathways not captured by the model (Shokhirev and Johnson 2021). Furthermore, temporal fluctuations—both normal and stress‐induced—must also be considered, as they have been reported for other aging biomarkers, which could affect the statistical power to determine the true effect of certain interventions on aging in the model (Shokhirev and Johnson 2021; Poganik et al. 2023). In this context, the estimation of regression coefficients (betas) allows the assessment of whether significant differences exist between intervened and control mice, while simultaneously adjusting for confounding factors that may influence the estimates, a common approach in epigenetic clock analyses (Yaskolka Meir et al. 2023). On the other hand, the use of bulk RNA‐seq prevents the detection of cell‐type trajectories and aging rhythms (Zhang et al. 2021). In this context, single‐cell transcriptomic clocks could represent an interesting complementary approach. Moreover, because liver sampling is lethal, this tool does not meet the desirable non‐lethality and repeatability criteria of an aging biomarker, thereby precluding longitudinal studies (Moqri et al. 2023). Nevertheless, the procedure is brief relative to the animal's lifespan and does not appear to violate the *non–age‐accelerating* criterion, as the measurement itself does not accelerate the age estimated by the clock (Butler et al. 2004). Finally, although transcriptomic clocks are characterized by greater biological interpretability, some of the genes that contribute to the clock lack clear functional characterization. In this sense, integrating other methodologies, such as proteomic or metabolomic clocks, may constitute complementary and valuable tools for addressing some of the gaps described in this work (Rutledge et al. 2022).

## 4 Conclusions

In this study, we developed LivAge, an accurate, accessible, and ready‐to‐use transcriptomic clock that provides robust transcriptomic age estimations from hepatic RNA‐seq data. After compiling a curated transcriptomic repository of 432 mouse liver samples ranging in age from 1 to 30 months, we implemented a novel strategy integrating differential expression analysis, Box‐Cox transformation of chronological age, and YuGene normalization of gene expression to train an Elastic Net model. This approach enabled us to reach statistical parameters comparable to those observed in gold‐standard epigenetic clocks when benchmarking external datasets. Moreover, LivAge captured accelerated transcriptomic aging in progeroid murine models and, conversely, detected significant age deceleration following various genetic and environmental geroprotective interventions. Given the potential of this transcriptomic clock for evaluating anti‐aging therapies, and to facilitate community‐wide adoption, we provide a web‐based platform for LivAge, offering a rapid, reproducible, and user‐friendly interface for the standardized assessment of transcriptomic age across diverse experimental datasets.

## 5 Methods

### 5.1 Downloading and Preprocessing of Transcriptomic Data

To train and evaluate a transcriptomic aging clock applicable to murine liver samples, we conducted a comprehensive review of the scientific literature to identify RNA‐seq studies in this tissue that included information on the chronological age of the samples, as well as any genetic or environmental interventions performed on them. Once identified, the raw sequencing reads were downloaded in a Bash programming environment (version 5.2.15) using the SRA Toolkit package (version 3.0.8). Subsequent processing of the raw sequences was carried out through a specialized RNA‐seq pipeline (version 3.22.2) implemented with Nextflow as part of the nf‐core community project, using default parameter settings (Di Tommaso et al. 2017). In this regard, after verifying the optimal quality of the reads with FastQC, adapter sequences were trimmed using Trim Galore, and read alignment and quantification were performed with a hybrid strategy combining the STAR and Salmon programs. The *Mus musculus* genome version 39 (GRCm39) and its corresponding structural annotation (GTF file, version 34) were used as reference datasets, both obtained from the official GENCODE repository (Mudge et al. 2025).

### 5.2 Differential Expression Analysis

The gene counts obtained from the RNA‐seq read processing were transferred to an R programming environment (version 4.5.0), where samples with fewer than 5 million total gene counts were excluded. In addition to the gene expression matrix—structured with genes as rows and biological samples as columns—a second metadata matrix containing the experimental conditions of each biological sample was also loaded. Both data structures were imported into the DESeq2 package (version 1.48.1), specifying the design formula *“*~ age + sex + study” as these variables can influence gene expression (Love and Huber 2014). Within this framework, normalization by sequencing depth was performed through the estimation of size factors, and genes not reaching a minimum of 10 reads in at least six samples were filtered out. Subsequently, differential expression analysis was conducted using the Likelihood Ratio Test (LRT), enabling the identification of genes whose expression levels were most affected by aging. Moreover, a gene set enrichment analysis (GSEA) was performed using the fgsea package (version 3.21) (Korotkevich et al. 2021). In addition, cell‐type deconvolution analysis was carried out using the *MuSiC* package (version 1.0.0) to infer the proportion of each cell type, taking as reference a murine single‐cell liver transcriptomic repository (The Tabula Muris Consortium 2020).

### 5.3 Development and External Validation of the Transcriptomic Clock

The development of the transcriptomic aging clock was conducted in an R programming environment using the glmnet (version 4.1.8) and caret (version 7.0.1) packages (Tay et al. 2023; Kuhn 2008). For this purpose, a matrix with the depth‐normalized gene expression (CPM, counts per million) of the most differentially expressed genes during aging and the age of the training samples was generated to train the transcriptomic clock through the Elastic Net algorithm, which combines the properties of Lasso (L1 penalty) and Ridge (L2 penalty) regression. During model training, a repeated cross‐validation strategy was implemented, combining 5‐fold partitioning (*k* = 5) with 10 repetitions. In each iteration, one subset was used for validation and the remaining four for training (Lumumba et al. 2024). This process was repeated five times per repetition, rotating the subsets, and the entire cycle was executed ten times with different random partitions. Consequently, each predictive model was trained a total of 50 times, validating hyperparameter ranges of [0, 1] for alpha and [0.1, 1] for lambda. After completing training of the predictive model with the best statistical performance, its generalization capacity was assessed using a second set of samples independent from those used during cross‐validation. In this sense, once the studies had been identified, the raw sequencing reads were downloaded and subjected to the same preprocessing pipeline as the training samples. After obtaining transcriptomic age predictions, these were compared with the chronological age annotated for the samples to calculate *R* ^2^, Pearson's *r*, and MAE.

### 5.4 Optimizing Transcriptomic Clock Performance

After developing a baseline transcriptomic aging clock, we evaluated how the numerical transformation of the variables affected its statistical performance. In this sense, we studied several numeric conversions of chronological age with the aim of facilitating the modeling of genes whose change in expression with aging does not follow a linear behavior, in an analogous way to what occurs in epigenetic clocks (Bernabeu et al. 2023). Specifically, we tested the logarithmic transformation and scaling through the “z‐score” method, for which the basic R functions “log” and “scale” were used, respectively. In addition, the effect of the Box‐Cox statistical transformation was also assessed using the MASS package (version 7.3–65), as well as the log‐linear conversion based on a piecewise function, involving a logarithmic transformation before adulthood and a linear transformation thereafter (Venables and Ripley 2002; Horvath 2013). This last approach was implemented in R using basic operations, defining mouse adulthood as 2 months of age. For each of these chronological age transformations evaluated, a new script was generated in which the differential expression analysis, as well as the training and evaluation of the predictive model with the second set of samples, were repeated.

On the other hand, considering the positive effect of the categorization of gene expression in order to reduce the noise of transcriptomic data generated in multiple laboratories, we also evaluated the impact of gene expression binarization on the performance of our transcriptomic clock (Meyer and Schumacher 2021). In addition, to mitigate the potential loss of considerable information in our data with this method, we implemented an alternative approach based on the quaternarization of the transcriptomic data. While for binarization genes with expression levels below the median of the sample were assigned a value of 0 and those above the median were assigned a value of 1, for quaternarization, each gene was labeled with values of 0, 1, 2, or 3, depending on the quartile corresponding to its expression level. Apart from the categorization of gene expression, scaling and YuGene normalization were also assessed for their effectiveness in standardizing transcriptomic data from different sources (Tarkhov et al. 2019; Lê Cao et al. 2014). Therefore, for each chronological age numeric conversion evaluated, the different types of gene expression transformations were performed, generating a predictive model for each combination.

The final performance of the resulting transcriptomic clock and its generalization capacity were assessed employing a third set of samples that had been excluded from both the cross‐validation, and the clock optimization processes, thereby yielding definitive statistical metrics. Furthermore, to compare the statistical performance of our predictive model with that of other transcriptomic clocks, we estimated the age of the same validation sample set using the “TACO” Elastic Net and Bayesian Ridge models, employing the “tAge” package (version 1.0.1) developed by the original authors for its implementation (Tyshkovskiy et al. 2026). Similarly, to apply the “RAPToR” tool, we utilized the “RAPToR” package (version 1.2.0). However, in the absence of a high‐temporal‐resolution reference for murine liver aging, we developed our own using the training sample set of our transcriptomic clock, following the guidelines provided by the authors (Bulteau and Francesconi 2022). Finally, employing the same cellular deconvolution procedure applied previously, we determined the cell proportions in the validation set samples, and regressed them against estimated transcriptomic age both as raw proportions and after isometric log‐ratio (ILR) transformation using the “compositions” package (version 2.0.9), aiming to quantify the effect of cell‐type proportion changes on the transcriptomic aging clock estimates.

### 5.5 Evaluation of Accelerated Aging and Geroprotective Interventions

After developing and optimizing a murine transcriptomic aging clock, to investigate its usefulness in analyzing the relation between predictions and the biological process of aging, we evaluated the transcriptomic age of liver samples from mouse models of accelerated aging, specifically the *Lmna* ^*G609G/G609G*^ and *Zmpste24* ^*−/−*^ models, both generated and characterized by our research group (Pendás et al. 2002; Osorio et al. 2011), as well of the *Terc* ^*−/−*^ y *Sprtn* ^*Y118C/Y118C*^ models, generated by other researchers (Ferrara‐Romeo et al. 2020; Tomaskovic et al. 2026) (Table S3). Moreover, given the potential utility of this tool for evaluating geroprotective interventions, we analyzed the impact of different strategies on the estimated transcriptomic age. In this way, we first analyzed the impact of genetic interventions using murine models, including Snell Dwarf, constitutive and inducible GHRKO, as well as Ames Dwarf mice (Poudel et al. 2024; Cole et al. 2017; Tyshkovskiy et al. 2019). Next, we assessed the effect on transcriptomic age of dietary and lifestyle interventions, such as caloric restriction, high‐ or low‐fat diets, protein restriction, methionine restriction, and physical exercise (Green et al. 2022; Pak et al. 2019). Finally, considering the development of drugs with potential geroprotective effects, we evaluated the impact of compounds such as 17‐α‐estradiol, acarbose, Protandim, KU‐0063794, rilmenidine, rapamycin, AZD8055, and ascorbyl palmitate (Tyshkovskiy et al. 2019). None of the studies utilized in the evaluation of accelerated aging and geroprotective interventions were involved in the training, optimization or external validation of the predictive model (Table S3). For all of them, raw sequencing reads were downloaded and processed using the same bioinformatic pipeline applied to the training and validation samples. In this way, normalized counts were obtained and subsequently used for clock estimation through the loading of the clock and the basic R *predict* function. Estimated values, generated on the Box‐Cox–transformed scale used during predictive model training, were immediately back‐transformed to the original age scale (months) via the inverse Box‐Cox transformation. Finally, to assess differences in transcriptomic aging between groups, we calculated the AgeDev for each sample, defined as the difference between estimated biological age and chronological age (Horvath 2013). Then, linear regression models were fitted with AgeDev as the dependent variable and experimental group (e.g., accelerated aging model or treated with geroprotective interventions) as the independent variable, adjusting for potential covariates such as batch effects or sex. The resulting beta coefficients (*β*) represent the mean difference in AgeDev between groups, where positive values indicate accelerated aging, and negative values indicate a geroprotective effect of the intervention. Statistical significance of these differences was evaluated using the associated *p*‐values, calculated from the one‐tailed *t*‐tests of the *β* coefficients in the linear models. Directional hypotheses were pre‐specified for each comparison: geroprotective interventions were expected to reduce transcriptomic age (*β* < 0), while progeroid and accelerated aging models were expected to accelerate it (*β* > 0). One‐sided *p*‐values were computed for all comparisons based on these pre‐specified directions; consequently, when the observed effect ran counter to the expected direction, the corresponding one‐sided *p*‐value was necessarily large (approaching 1), correctly indicating a lack of support for the hypothesis in that comparison. To account for multiple comparisons, *p*‐values were corrected for false discovery rate (FDR) using the Benjamini‐Hochberg procedure applied independently within each intervention category progeroid and geroprotective (genetic, lifestyle and pharmacological).

### 5.6 Development of the LivAge Web‐Based Platform

To facilitate the application of the LivAge transcriptomic clock, we developed a web‐based platform using Shiny (version 1.12.1) and Shinylive (version 0.3.0). Users can upload raw RNA‐seq count matrices in tab‐delimited format, which are automatically converted to counts per million (CPM). Then, gene expression values are transformed using YuGene normalization, and age estimates are estimated applying the Elastic Net model followed by the inverse Box‐Cox transformation to obtain transcriptomic age in months. Predicted ages can be viewed directly in a tabular format on the web platform or downloaded as CSV files for further analysis.

The platform is publicly accessible at: https://gencanage.uniovi.es/resources/liv_age.

## Author Contributions

V.C.‐C., P.M.Q. and J.M.P.F. designed the study. V.C.‐C. carried out most of the bioinformatic work, with support from P.M.Q., and G.B. C.B. and A.P.U. carried out RNA‐seq analyses of progeroid mice. V.C.‐C. and J.M.P.F. wrote the manuscript with input from all authors. V.C.‐C. and D.R.‐V. implemented the online web platform. All the authors approved the submitted version of the manuscript.

## Funding

This work was supported by Ministerio de Ciencia e Innovación, PDI2020‐118394RB‐100, PID2023‐148089OB‐I00. Longevity Impetus Grant. Consejería de Ciencia, Innovación y Universidad del Gobierno del Principado de Asturias, IDE/2024/000784.

## Conflicts of Interest

The authors are the inventors on a patent application related to this work.

## Supporting information


**Table S1:** Summary of RNA‐seq datasets used in the training and validation of LivAge. For each study, the table reports the accession number (from NCBI or CNCB), the number of samples employed, the genetic background of the mice, the first author, and the year of publication. The role of each dataset is also indicated, distinguishing between datasets employed for repeated cross‐validation (RCV) during model training and testing, those used for optimization of the predictive model through the evaluation of gene expression and chronological age transformations, and those used for the final external validation of the transcriptomic aging clock.

**Table S2:** List of gene predictors employed by LivAge. For each gene included in the transcriptomic aging clock, the table reports the Ensembl gene identifier, the gene symbol and the regression coefficient used in the model.

**Table S3:** Summary of RNA‐seq datasets used in the evaluation of genetic and environment interventions in estimated transcriptomic age. For each study, the table reports the accession number (from NCBI or EMBL‐EBI), the number of samples, the genetic background and sex of the mice, the first author, the year of publication, and the intervention evaluated.

**Data S1:** Differential gene expression analysis in transcriptomic atlas of liver aging. For each gene, the table reports the Ensembl gene identifier, the mean normalized expression across all samples, the log2 fold change per month, the standard error of the log2 fold change, the Wald test statistic, the nominal *p* value, the adjusted *p* value (FDR) and the gene symbol.

**Data S2:** Gene set enrichment analysis results. For each pathway, the table reports the nominal *p* value, the adjusted *p* value (FDR), the standard error of the log2 enrichment score, the enrichment score (ES), the normalized enrichment score (NES) and the number of genes in the pathway.

**Data S3:** Estimated cell‐type proportions in liver RNA‐seq samples by cellular deconvolution. For each sample, the table reports the sample identifier, chronological age in months, study accession number, sex of the animal, cell type, and the estimated proportion of that cell type.

**Data S4:** Estimated liver transcriptomic ages by LivAge for all samples used in this study. For each sample, the table reports the sample identifier, the sample set (Training, Optimization, Validation, or Intervention), sex, chronological age and the estimated transcriptomic age in months.

**Data S5:** Estimated transcriptomic age by the TACO clock and by RAPToR in the validation cohort. For each sample, the table reports the sample identifier, chronological age in months, sex of the animal, and the estimated age from each clock.

**Data S6:** Estimated cell‐type proportions in liver RNA‐seq samples by cellular deconvolution for the external validation set. For each sample, the table reports the sample identifier, chronological age in months, LivAge‐estimated transcriptomic age, sex and the estimated proportion of each hepatic cell type (natural killer cell, hepatocyte, endothelial cell of the hepatic sinusoid, Kupffer cell, B cell) as inferred by reference‐based deconvolution of the bulk RNA‐seq profile.

**Data S7:** FDR‐corrected significance of transcriptomic age effects across interventions. For each intervention, the table reports its category, the intervention label, and the Benjamini‐Hochberg FDR‐corrected *p*‐value from a linear model of transcriptomic AgeDev on treatment, tested both two‐sided and one‐sided in the expected direction—age‐protective for pharmacological/genetic/lifestyle interventions, age‐accelerating for progeroid models. FDR correction was applied separately within each testing direction across all interventions.

**Figure S1:** Bar plots representing the composition of the transcriptomic atlas of liver aging in mice (training set). (a) Distribution of liver RNA seq samples across chronological age groups (0–10, 10–20 and 20–30 months), stratified by males (green) and females (orange). (b) Distribution of samples across the same age groups, stratified by study of origin. Individual SRA accessions are shown, with additional datasets grouped as “Other studies”. Bar heights indicate the number of samples in each category.

**Figure S2:** Development of a murine transcriptomic aging clock. (a) Results of the transcriptomic age prediction computed by cross‐validation. The scatter plot displays the results of the cross‐validation for the first transcriptomic clock trained, illustrating the correlation between the chronological ages and the estimated transcriptomic age. The *X*‐axis represents the chronological age (in months), whereas the *Y*‐axis shows the transcriptomic age predicted by the elastic net model based on the gene expression data. Each dot represents an individual RNA‐seq sample. The dashed gray line represents a perfect prediction, where estimated age equals chronological age, and the solid red line is the regression line of best fit for the predicted data points. The performance metrics shown are *R* ^2^ (Coefficient of Determination), *r* (Pearson Correlation Coefficient) and MAE (Mean Absolute Error). (b) External validation of the transcriptomic aging clock. Scatter plot showing the results from an independent set of 39 external RNA‐seq samples. The *X*‐axis represents chronological age (months), and the *Y*‐axis shows transcriptomic age predicted by the model. Each dot represents a sample. The dashed gray line indicates perfect prediction, and the solid red line is the regression line. Performance metrics include *R* ^2^, Pearson's *r*, and MAE. (c) External validation of the optimized transcriptomic aging clock in male and female samples. Scatter plot showing the results from an independent validation set of 134 external RNA‐seq samples. The *X*‐axis represents chronological age (months), and the *Y*‐axis shows transcriptomic age predicted by the model. Each dot corresponds to a sample. The dashed gray line indicates perfect prediction, and the solid red line is the regression line. Performance metrics include *R* ^2^, Pearson's *r*, and MAE.

**Figure S3:** Evaluation of alternative transcriptomic clocks. Scatter plots showing the benchmarking results on an independent validation set of 89 external RNA‐seq female samples (orange) and 50 external RNA‐seq male samples (blue). The *X*‐axis represents chronological age (months), and the *Y*‐axis shows the transcriptomic age predicted by each respective transcriptomic clock (a–i). Each dot corresponds to a sample, and the dashed gray line indicates perfect prediction. Performance is evaluated using the Mean Absolute Error (MAE).

## Data Availability

To ensure the full reproducibility of the results, all Supporting Information and the source code used in the current study are available in a Zenodo repository at https://zenodo.org/records/19596458.
