# Supplementary material for: LivAge: An Online Aging Clock for Murine Transcriptomic Age Estimation
Source: Aging Cell. 2026 Aug 21;25(9):e70677. doi: 10.1111/acel.70677 (PMC13498547; doi:10.1111/acel.70677)
Supplement: Supplementary file 1 [file ACEL-25-e70677-s001.pdf]

Supplementary Data 3. Estimated cell-type proportions in liver RNA seq samples by cellular deconvolution. For each sample, the table reports the sample identifier, chronological age in months, study accession number, sex of the animal, cell type, and the estimated proportion of that cell type.

Sample	Months	Sex	NK cell	Hepatocyte	Endothelial cell of hepatic sinusoid	Kupffer cell	B cell
CRR117136	2	m	0.016564578	0.841297437	0.106645348	0.01485552	0.020637118
CRR117137	2	m	0.01967469	0.837914526	0.106382909	0.014748979	0.021278896
CRR117138	2	m	0.01306194	0.871179546	0.091898325	0.015378806	0.008481384
CRR117139	2	m	0.013954405	0.830688356	0.111202819	0.020503446	0.023650975
CRR117140	2	m	0.014869488	0.846065006	0.104788561	0.022859588	0.011417357
CRR117141	6	m	0.016408297	0.854585346	0.094845372	0.019520451	0.014640535
CRR117142	6	m	0.014974701	0.850069171	0.100691642	0.020519413	0.013745073
CRR117143	6	m	0.016777366	0.860863138	0.083294863	0.032885288	0.006179345
CRR117144	6	m	0.019188239	0.845850218	0.100916195	0.018539469	0.015505879
CRR117145	6	m	0.014582545	0.862153525	0.08904816	0.027219456	0.006996314
CRR117146	15	m	0.016634727	0.847475139	0.097926153	0.018641928	0.019322052
CRR117147	15	m	0.023651239	0.85908587	0.09079011	0.010302107	0.016170675
CRR117148	15	m	0.020071726	0.855811386	0.087716265	0.025423744	0.010976879
CRR117149	15	m	0.009048085	0.877641252	0.092797018	0.013946541	0.006567104
CRR117150	15	m	0.015184673	0.87118757	0.084574446	0.020516554	0.008536757
CRR117151	19	m	0.018750288	0.819811786	0.116449248	0.02168087	0.023307808
CRR117152	19	m	0.027105496	0.826888576	0.100911156	0.019715201	0.025379571
CRR117153	19	m	0.028433824	0.781574991	0.147343604	0.019102001	0.02354558
CRR117154	19	m	0.024652728	0.778537393	0.130600198	0.037545058	0.028664624
CRR117155	19	m	0.024220681	0.748559132	0.144132633	0.061266504	0.02182105
CRR117156	26	m	0.018874134	0.843188323	0.105363431	0.01579399	0.016780122
CRR117157	26	m	0.025871105	0.849867201	0.099653674	0.011615437	0.012992583
CRR117158	26	m	0.029155453	0.745533176	0.119873155	0.051242643	0.054195573
CRR117159	26	m	0.018765918	0.846895748	0.098475047	0.012108802	0.023754484
CRR117160	26	m	0.025917233	0.844481467	0.096196379	0.01767176	0.015733161
SRR10573512	24	m	0.050903401	0.831174742	0.02523068	0.047447199	0.045243978
SRR10573519	9	m	0.034417949	0.860283316	0.021942759	0.036169579	0.047186397
SRR10573536	9	m	0.020800333	0.808280386	0.076151112	0.02071055	0.07405762
SRR10573553	4	m	0.033521054	0.880921671	0.011885542	0.041227345	0.032444388
SRR10573569	4	m	0.020157361	0.774899332	0.066625578	0.021791261	0.116526467

SRR10573586	4	m	0.027567484	0.901702949	0.007358304	0.036154029	0.027217233
SRR10573637	24	m	0.057712027	0.838872611	0.018058889	0.051724256	0.033632216
SRR10573654	24	m	0.036144903	0.877934565	0.015137052	0.039838306	0.030945174
SRR10573669	18	m	0.045655357	0.870115829	0.017735146	0.03453911	0.031954558
SRR10573684	18	m	0.038513996	0.821433228	0.02368228	0.064261128	0.052109369
SRR10573700	18	m	0.060429024	0.828716848	0.016733716	0.0497198	0.044400612
SRR10573717	12	m	0.03609099	0.808394253	0.043564823	0.045936787	0.066013147
SRR10573733	12	m	0.053089144	0.843349791	0.01585026	0.046216465	0.04149434
SRR10573748	12	m	0.04162112	0.868679051	0.013517506	0.047107182	0.029075141
SRR10573763	9	m	0.040012724	0.869371603	0.01253895	0.035193674	0.042883049
SRR10573779	9	m	0.075793205	0.755930965	0.021590712	0.093972338	0.05271278
SRR10573796	9	m	0.034516305	0.882544066	0.009268471	0.033498268	0.040172891
SRR10573813	4	m	0.035613584	0.871677925	0.017152479	0.042651465	0.032904547
SRR10573830	4	m	0.039380693	0.884433129	0.012273472	0.039939352	0.023973355
SRR10573847	4	m	0.03876076	0.887409471	0.007512984	0.037395774	0.028921011
SRR10573864	24	m	0.038160569	0.875242331	0.015419841	0.040889892	0.030287367
SRR10573881	24	m	0.039974798	0.798749547	0.047131751	0.031463381	0.082680523
SRR10573897	24	m	0.041159205	0.802541757	0.036834445	0.04279691	0.076667683
SRR10573912	18	m	0.025737976	0.870117702	0.020491378	0.037036133	0.046616811
SRR10573929	18	m	0.020117204	0.90880773	0.008489388	0.040394802	0.022190877
SRR10573945	18	m	0.025159843	0.832203422	0.056857292	0.021781731	0.063997712
SRR10573961	12	m	0.019125895	0.887323294	0.011001364	0.038552393	0.043997054
SRR10573977	12	m	0.047667429	0.691623817	0.082044746	0.03947428	0.139189728
SRR10573994	12	m	0.017665957	0.896653165	0.009960527	0.04024001	0.035480341
SRR10574011	9	m	0.024402199	0.769433841	0.067379291	0.032424052	0.106360618
SRR10851479	10	m	0.002190891	0.877260424	0.08308438	0.033046598	0.004417707
SRR10851480	5	m	0.002430532	0.875079156	0.0858856	0.030078838	0.006525874
SRR10851482	10	m	0.003272482	0.893006786	0.075496916	0.019716586	0.008507229
SRR10851484	5	m	0.004519261	0.895185229	0.074375731	0.020423613	0.005496166
SRR10851487	5	m	0.003202761	0.885138253	0.062996647	0.046108867	0.002553472
SRR10851489	10	m	0.000491783	0.909522924	0.065230352	0.021644995	0.003109946

SRR10851491	5	m	0.007061215	0.8359886	0.089519259	0.05164689	0.015784036
SRR10851493	10	m	0.003042123	0.885528169	0.084012403	0.022966872	0.004450433
SRR10851495	5	m	0.004593617	0.906775145	0.066765348	0.01602931	0.00583658
SRR10851497	10	m	0.004182567	0.873356281	0.090900444	0.024884992	0.006675717
SRR10851499	5	m	0.009973964	0.811278478	0.088128096	0.064379211	0.026240251
SRR10851501	10	m	0.000887923	0.892946394	0.077243285	0.025695417	0.003226981
SRR11250383	5	f	0.014922332	0.747420842	0.187818008	0.034722604	0.015116214
SRR11250384	5	f	0.018150461	0.78213004	0.144201887	0.034341139	0.021176473
SRR11250385	5	f	0.019344012	0.73080694	0.172902229	0.060494396	0.016452423
SRR11250386	5	f	0.014725613	0.75610224	0.16377192	0.047721225	0.017679002
SRR11250387	5	f	0.00949201	0.794860132	0.155650382	0.029012904	0.010984572
SRR11250388	5	f	0.023256511	0.675756115	0.167384187	0.115912673	0.017690513
SRR11250389	5	m	0.011589705	0.825645404	0.123948679	0.025749778	0.013066434
SRR11250390	5	m	0.011145331	0.802413211	0.134572008	0.038027378	0.013842071
SRR11250391	5	m	0.013231362	0.840645325	0.112033275	0.023256218	0.01083382
SRR11250392	5	m	0.007314334	0.846675852	0.112026206	0.025026357	0.008957251
SRR11250393	5	m	0.011721482	0.837346983	0.11682211	0.028005177	0.006104247
SRR11250394	5	m	0.028726649	0.662035036	0.220777022	0.046892231	0.041569061
SRR11250697	5	f	0.008222547	0.803339972	0.14254353	0.037463225	0.008430726
SRR11250698	5	f	0.006433732	0.814672586	0.125766484	0.047473672	0.005653526
SRR11250699	5	f	0.003255335	0.860856705	0.100233152	0.031261101	0.004393708
SRR11250700	5	f	0.016250227	0.693969673	0.196420281	0.044930425	0.048429395
SRR11250701	5	m	0.009974538	0.811278408	0.088126913	0.064379377	0.026240765
SRR11250702	5	m	0.007061684	0.83598713	0.089519544	0.05164684	0.015784802
SRR11250703	5	m	0.004518836	0.895186172	0.074375114	0.020423767	0.00549611
SRR11250704	5	m	0.002430603	0.87507881	0.085885879	0.030078844	0.006525863
SRR11250705	5	m	0.004593454	0.906774782	0.06676578	0.016029457	0.005836526
SRR11250706	5	m	0.003202571	0.885138417	0.062996657	0.046108937	0.002553418
SRR11250781	5	m	0.007651604	0.805846286	0.132251112	0.040336126	0.013914871
SRR11250782	5	m	0.045365278	0.613746766	0.104664386	0.18996005	0.04626352
SRR11250783	5	f	0.154601293	0.439380411	0.188769539	0.079180694	0.138068063

SRR11250784	5	f	0.016479944	0.833241305	0.051755093	0.079580005	0.018943653
SRR11250785	5	f	0.010153638	0.892485751	0.046767979	0.03992672	0.010665911
SRR11250786	5	f	0.009161644	0.896351416	0.047916033	0.03604708	0.010523826
SRR11250787	5	m	0.133519009	0.684552901	0.060085323	0.082481477	0.03936129
SRR11250788	5	m	0.018718776	0.861947789	0.06145561	0.040298826	0.017578999
SRR11250789	5	m	0.115081403	0.505338844	0.176465074	0.05085349	0.15226119
SRR11250790	5	m	0.017018992	0.776286242	0.065832784	0.085133332	0.05572865
SRR11250791	5	m	0.011397048	0.886191892	0.043092539	0.037566271	0.02175225
SRR11250792	5	m	0.015887767	0.818684731	0.057175674	0.068498855	0.039752973
SRR11250793	5	m	0.0139075	0.863526531	0.065416102	0.022119843	0.035030024
SRR11251016	5	f	0.002336412	0.843816996	0.08446684	0.049637142	0.01974261
SRR11251017	5	f	0.00373914	0.829521121	0.119559389	0.039578422	0.007601928
SRR1158577	1.5	m	0.008617487	0.895555461	0.072906582	0.009919931	0.013000538
SRR1158578	1.5	m	0.010755057	0.880717564	0.077761614	0.013184617	0.017581147
SRR1158579	1.5	m	0.012556991	0.867065895	0.085157995	0.016402973	0.018816146
SRR1158580	1.5	m	0.009436701	0.890600617	0.073143313	0.011701631	0.015117738
SRR1158581	1.5	m	0.006043146	0.895466501	0.075299493	0.015172375	0.008018485
SRR1158582	1.5	m	0.008732113	0.878502765	0.083595678	0.014638031	0.014531413
SRR1158583	1.5	m	0.010410462	0.880294571	0.081321873	0.012019687	0.015953407
SRR1158584	1.5	m	0.01129648	0.871130304	0.090658006	0.018039035	0.008876175
SRR1166896	27	m	0.034718587	0.543137671	0.165036072	0.167318811	0.089788859
SRR1166897	27	f	0.015136847	0.756249776	0.154229633	0.052880561	0.021503183
SRR1166898	27	f	0.018333676	0.750441364	0.134257958	0.05529704	0.041669963
SRR1166899	27	f	0.016836284	0.733591872	0.153726106	0.071045217	0.024800521
SRR1166900	27	m	0.024578268	0.467959457	0.141481432	0.244478519	0.121502324
SRR1166901	27	m	0.018243626	0.77306996	0.140715138	0.045957644	0.022013632
SRR11902426	6.5	m	0.010703781	0.895616137	0.074189258	0.007655512	0.011835312
SRR11902427	6.5	m	0.009341184	0.90179157	0.068178906	0.01027683	0.010411509
SRR11902428	6.5	m	0.010228752	0.873688197	0.089700733	0.014983723	0.011398595
SRR11902429	6.5	m	0.020712718	0.873218376	0.07556403	0.010986227	0.01951865
SRR11902430	6.5	m	0.012053315	0.893420065	0.075108168	0.007688393	0.011730059

SRR11902431	6.5	m	0.012951846	0.876921191	0.080697914	0.013936523	0.015492526
SRR11902432	6.5	m	0.009939517	0.894157975	0.07315477	0.012873379	0.009874359
SRR11902433	6.5	m	0.013351013	0.862323691	0.088468421	0.018297216	0.017559659
SRR11902434	6.5	m	0.011580357	0.902392397	0.069042781	0.008209703	0.008774763
SRR11902435	6.5	m	0.010447635	0.889378188	0.079930721	0.009034928	0.011208528
SRR11902436	6.5	m	0.01114172	0.904310183	0.067998613	0.008092462	0.008457022
SRR11902437	6.5	m	0.009250113	0.891117907	0.079121238	0.009899198	0.010611544
SRR15881309	2.5	f	0.005691129	0.856285819	0.098834388	0.031248598	0.007940066
SRR15881310	2.5	f	0.009478288	0.843953602	0.100232037	0.032580704	0.01375537
SRR15881311	2.5	f	0.003739739	0.855177386	0.090130799	0.035410088	0.015541987
SRR15881312	2.5	f	0.008792361	0.873873501	0.088351966	0.021028026	0.007954146
SRR15881313	2.5	f	0.010109833	0.858136285	0.094197444	0.031345995	0.006210443
SRR16296667	18	m	0.022415843	0.728608655	0.175943291	0.046585392	0.026446819
SRR16296668	18	f	0.036373565	0.635636322	0.224649251	0.059458003	0.04388286
SRR16296669	18	f	0.023554522	0.670765997	0.230677188	0.039173074	0.035829219
SRR16296685	4	m	0.028463453	0.734451273	0.186844117	0.017297785	0.032943372
SRR16296686	4	f	0.029229939	0.72059781	0.200486933	0.027760326	0.021924991
SRR16296687	4	f	0.03275374	0.727439743	0.190131231	0.023973586	0.0257017
SRR17260234	6	m	0.012520351	0.840233767	0.115567399	0.017205854	0.014472629
SRR17260235	6	m	0.013008638	0.83194183	0.118115694	0.021197649	0.015736189
SRR17260236	6	m	0.013774612	0.852576986	0.111660863	0.009193249	0.012794289
SRR17260237	6	m	0.056156916	0.626150268	0.214597882	0.040939999	0.062154935
SRR17260238	6	m	0.011669213	0.857893109	0.101186402	0.015062001	0.014189276
SRR17260239	6	m	0.011898022	0.821653125	0.128773461	0.026206558	0.011468835
SRR17260240	6	m	0.012360799	0.811339039	0.144430932	0.01794387	0.01392536
SRR17260241	6	m	0.010168174	0.831745797	0.119926888	0.022795804	0.015363336
SRR17260242	6	m	0.013831079	0.856091106	0.102785966	0.014995206	0.012296643
SRR17260243	6	m	0.014355511	0.865842982	0.09075281	0.014266519	0.014782178
SRR17260244	6	m	0.008256891	0.892972652	0.080078385	0.006885345	0.011806726
SRR17260245	6	m	0.010296411	0.84238309	0.11301613	0.017923265	0.016381104
SRR17260246	6	m	0.011636888	0.804570662	0.142938179	0.020645488	0.020208782

SRR17260247	6	m	0.014061553	0.802376628	0.152953039	0.015458766	0.015150015
SRR17260248	6	m	0.015569522	0.801014417	0.146117172	0.021192476	0.016106414
SRR17260249	6	m	0.012490974	0.849211725	0.112228957	0.014207681	0.011860664
SRR17260250	6	m	0.012622089	0.829416014	0.133016951	0.011447583	0.013497364
SRR17260251	6	m	0.014202966	0.807320082	0.14557723	0.016201531	0.01669819
SRR17260252	6	m	0.01307605	0.793440743	0.154499853	0.017454454	0.021528899
SRR17260253	6	m	0.014789827	0.815911658	0.139004469	0.013071791	0.017222255
SRR17260254	6	m	0.020360538	0.834366988	0.107019399	0.020023526	0.018229549
SRR17260255	6	m	0.015647888	0.846327124	0.107613956	0.013953302	0.016457729
SRR17260256	6	m	0.014762781	0.84487221	0.115332536	0.011276123	0.01375635
SRR17260257	6	m	0.009825602	0.8162459	0.14568909	0.014241287	0.01399812
SRR17260378	19	m	0.035958995	0.677521715	0.215299052	0.038445922	0.032774316
SRR17260379	19	m	0.03100805	0.627207851	0.242157995	0.04583262	0.053793483
SRR17260380	19	m	0.038420024	0.648968335	0.211265244	0.043451664	0.057894734
SRR17260381	19	m	0.031304868	0.763132051	0.147779374	0.031775658	0.02600805
SRR17260382	19	m	0.018535877	0.836044759	0.108418987	0.017882386	0.019117992
SRR17260383	19	m	0.02740791	0.702515345	0.194431076	0.047056819	0.028588849
SRR17260384	19	m	0.015896952	0.750511525	0.178915934	0.027515188	0.027160401
SRR17260385	19	m	0.029500922	0.666268421	0.231756565	0.038001316	0.034472776
SRR17260386	19	m	0.031940671	0.695387539	0.194990084	0.045968845	0.03171286
SRR17260387	19	m	0.030978079	0.689276573	0.166911233	0.056959572	0.055874543
SRR17260388	19	m	0.029768855	0.749366925	0.159350217	0.033331076	0.028182927
SRR17260389	19	m	0.031804241	0.694819728	0.194024164	0.043304444	0.036047424
SRR17260390	19	m	0.026432653	0.714863364	0.190353307	0.037121687	0.03122899
SRR17260391	19	m	0.033981588	0.703333302	0.201726044	0.035281712	0.025677354
SRR17260392	19	m	0.027052604	0.721557219	0.175066988	0.046147861	0.030175328
SRR17260393	19	m	0.034608876	0.685963178	0.201610618	0.039994361	0.037822967
SRR17260394	19	m	0.019358874	0.821869907	0.117312818	0.026351396	0.015107005
SRR17260395	19	m	0.019812103	0.695302963	0.184149031	0.062631535	0.038104369
SRR17260396	19	m	0.01828797	0.7063472	0.183526365	0.051999509	0.039838955
SRR17260397	19	m	0.026030911	0.68264628	0.189270828	0.080342628	0.021709353

SRR17260398	19	m	0.017903741	0.830365421	0.114835873	0.017970003	0.018924962
SRR17260399	19	m	0.022003541	0.71531699	0.167370342	0.054465184	0.040843943
SRR17260400	19	m	0.01991016	0.83225815	0.115879648	0.02439358	0.007558464
SRR17260401	19	m	0.030766747	0.694811588	0.1859691	0.051785119	0.036667446
SRR17261649	22	m	0.009762825	0.81184438	0.112896358	0.052801602	0.012694835
SRR17261650	22	m	0.017602022	0.802769914	0.127419623	0.037367357	0.014841084
SRR17261651	22	f	0.016992403	0.687316314	0.13802875	0.147887182	0.009775351
SRR17261652	22	f	0.016566866	0.761661714	0.142570216	0.06093586	0.018265344
SRR17261653	22	f	0.004258096	0.676201561	0.129761426	0.130258934	0.059519983
SRR17261796	25	m	0.020020737	0.827196728	0.093096004	0.04061612	0.019070412
SRR17261797	25	m	0.028104125	0.095637921	0.091084971	0.567470863	0.217702121
SRR21769374	2	m	0.015853365	0.891659263	0.062085186	0.020464582	0.009937603
SRR21769376	2	m	0.023729101	0.826806875	0.105897502	0.02842696	0.015139562
SRR21769522	2	m	0.020947261	0.8789351	0.062551218	0.02611397	0.011452451
SRR21769524	2	m	0.013645369	0.883743869	0.070142642	0.020330091	0.01213803
SRR21769526	2	m	0.019354547	0.817108055	0.114498818	0.027071319	0.021967261
SRR21769528	2	m	0.014364549	0.890947948	0.064273297	0.019424789	0.010989417
SRR21769530	2	m	0.015255224	0.901754427	0.052736881	0.017881992	0.012371476
SRR21769532	2	m	0.017346562	0.822158375	0.120387655	0.020771877	0.019335531
SRR21769534	2	m	0.020116434	0.87348641	0.064498825	0.030393688	0.011504644
SRR21769536	2	m	0.017943198	0.866746823	0.082132251	0.023325369	0.00985236
SRR21769538	2	m	0.032005763	0.757033479	0.130556651	0.055608216	0.024795892
SRR21769540	2	m	0.019441021	0.893854589	0.056509355	0.022617705	0.007577331
SRR21769542	2	m	0.022146493	0.862720088	0.076046631	0.027574726	0.011512062
SRR21769544	2	m	0.014102473	0.829705222	0.108510927	0.032757268	0.01492411
SRR21769546	2	m	0.017994726	0.886559641	0.070653291	0.016935583	0.007856759
SRR21769548	2	m	0.021209365	0.881067445	0.075396022	0.012734734	0.009592434
SRR21769550	2	m	0.022800782	0.81972844	0.109681659	0.032373788	0.01541533
SRR21769552	2	m	0.014908401	0.897027649	0.070221105	0.011116371	0.006726474
SRR21769554	2	m	0.01656888	0.877588122	0.073126525	0.020486172	0.012230301
SRR21769556	2	m	0.023389816	0.810610931	0.118856008	0.029366897	0.017776348

SRR21769558	2	m	0.016046477	0.882963177	0.068988889	0.018908314	0.013093143
SRR21769560	2	m	0.013653896	0.874559727	0.08122747	0.020775055	0.009783852
SRR21769562	2	m	0.021684073	0.832290372	0.107009061	0.026070651	0.012945843
SRR21769564	2	m	0.024096444	0.881839643	0.059096418	0.028577768	0.006389726
SRR23517674	30	m	0.016781967	0.793219994	0.125166392	0.049512309	0.015319339
SRR23517675	30	m	0.03148438	0.572432542	0.144369903	0.176157935	0.075555239
SRR23517676	30	m	0.022259317	0.676214046	0.12557812	0.139066384	0.036882133
SRR23517677	30	m	0.010973238	0.739895076	0.121661687	0.078650872	0.048819126
SRR23517678	24	m	0.019457879	0.767046729	0.127492223	0.062508693	0.023494477
SRR23517679	24	m	0.011843522	0.81394406	0.113609232	0.043013109	0.017590077
SRR23517680	24	m	0.011370843	0.763697919	0.11357131	0.072663397	0.038696532
SRR23517681	24	m	0.013401119	0.71644123	0.117735652	0.090689144	0.061732855
SRR23517682	15	m	0.009288369	0.848089501	0.107501432	0.026906223	0.008214475
SRR23517683	15	m	0.012761024	0.840096978	0.102806807	0.033094316	0.011240876
SRR23517684	15	m	0.01179537	0.821069513	0.113568134	0.036489199	0.017077784
SRR23517685	15	m	0.01142649	0.821762381	0.124198134	0.032908697	0.009704298
SRR23517686	6	m	0.005843069	0.867233568	0.093301934	0.024809355	0.008812074
SRR23517687	6	m	0.005545872	0.866107158	0.097636166	0.019867115	0.010843689
SRR23517688	6	m	0.007287504	0.858433593	0.103525244	0.018645365	0.012108295
SRR23517689	6	m	0.007747475	0.87118521	0.093535236	0.01657035	0.010961729
SRR23730067	2.5	f	0.038929049	0.77175222	0.092958882	0.087426332	0.008933519
SRR23730068	2.5	f	0.018187369	0.8922597	0.043481352	0.041911455	0.004160123
SRR23730069	2.5	f	0.021117789	0.875527476	0.055614186	0.04172656	0.00601399
SRR23730070	2.5	f	0.015885528	0.827733415	0.084237017	0.058450942	0.013693098
SRR23730071	2.5	f	0.047585043	0.816385068	0.090038802	0.03044246	0.015548627
SRR23730072	2.5	f	0.020246215	0.824631357	0.087715855	0.063165121	0.004241452
SRR24886776	24	m	0.043758296	0.393672392	0.069999265	0.407057079	0.085512968
SRR24886777	24	m	0.048096509	0.314328945	0.079930299	0.444092028	0.113552219
SRR24886778	24	m	0.060843933	0.233006734	0.117094791	0.518542452	0.07051209
SRR24886814	24	f	0.016931322	0.608475955	0.103859509	0.233410538	0.037322676
SRR24886815	24	f	0.013229511	0.601959962	0.10059251	0.202031519	0.082186498

SRR24886827	6 m	0.011445543	0.918918369	0.049121672	0.009520062	0.010994353
SRR24886828	6 m	0.006675269	0.922024029	0.052797029	0.013308749	0.005194925
SRR24886829	6 m	0.006918459	0.908644327	0.06191694	0.016741717	0.005778557
SRR28424098	15 m	0.012619238	0.810257376	0.114077507	0.043805304	0.019240575
SRR28424099	15 m	0.009868765	0.812663482	0.117125376	0.046392914	0.013949463
SRR28424100	15 m	0.014038887	0.827211908	0.10998813	0.03844521	0.010315865
SRR28424101	15 m	0.016593287	0.791178252	0.126248572	0.043887715	0.022092174
SRR28424102	15 m	0.009045251	0.858229921	0.094705196	0.033936032	0.0040836
SRR28424103	15 m	0.011160412	0.846469849	0.098669243	0.0372214	0.006479095
SRR28424104	15 m	0.009296759	0.836124679	0.109945697	0.03549687	0.009135996
SRR28424105	15 m	0.010472472	0.845418524	0.101633961	0.032410798	0.010064245
SRR28424106	9 m	0.015888529	0.852879185	0.086659862	0.035812777	0.008759647
SRR28424107	9 m	0.011046391	0.845726305	0.102471182	0.031028199	0.009727923
SRR28424108	9 m	0.013206607	0.838840828	0.107816104	0.027669945	0.012466516
SRR28424109	9 m	0.012504292	0.825705255	0.116282525	0.036511357	0.008996572
SRR28424110	9 m	0.012978762	0.829158897	0.113535338	0.033880183	0.01044682
SRR28424111	9 m	0.007913914	0.835918175	0.080554546	0.052496985	0.023116379
SRR28424112	9 m	0.013183562	0.841466942	0.110539062	0.023443096	0.011367338
SRR28424113	9 m	0.010080788	0.819011938	0.126960958	0.033999256	0.00994706
SRR28424130	9 m	0.010113289	0.854653042	0.092023967	0.036050601	0.007159101
SRR28424131	9 m	0.011822489	0.844671969	0.101046291	0.036558553	0.005900699
SRR28424132	2 m	0.006114663	0.883217064	0.071757436	0.035260183	0.003650655
SRR28424133	2 m	0.024942155	0.806786822	0.046932308	0.053859159	0.067479557
SRR28424134	2 m	0.00808044	0.876035286	0.078481496	0.030287626	0.007115151
SRR28424135	2 m	0.014936261	0.854326865	0.088733029	0.032961382	0.009042464
SRR28424136	2 m	0.011836945	0.858361086	0.084721847	0.03623152	0.008848602
SRR28424137	2 m	0.014263698	0.848626453	0.087992222	0.03776569	0.011351937
SRR28424138	2 m	0.030448792	0.819220927	0.109968639	0.02941643	0.010945211
SRR28424139	2 m	0.022440038	0.825558891	0.11017417	0.033010411	0.00881649
SRR28424140	2 m	0.004682598	0.880570054	0.07283902	0.036786414	0.005121915
SRR28424141	2 m	0.013415282	0.84092573	0.094065886	0.040809931	0.010783171

SRR28424154	30 m	0.010414436	0.809443014	0.100159768	0.065771251	0.014211531
SRR28424155	30 m	0.009655356	0.792851584	0.114580115	0.046674494	0.03623845
SRR28424156	30 m	0.012037891	0.753648461	0.084780036	0.104329715	0.045203897
SRR28424157	30 m	0.00987836	0.797615701	0.111993217	0.054544305	0.025968417
SRR28424158	30 m	0.01895999	0.719059411	0.106001576	0.125330362	0.030648661
SRR28424159	30 m	0.014424412	0.779732981	0.118721804	0.064032408	0.023088395
SRR28424160	30 m	0.019427172	0.672729839	0.123500233	0.118779343	0.065563414
SRR28424161	30 m	0.028730504	0.672469926	0.136759465	0.087342108	0.074697998
SRR28424190	30 m	0.023523866	0.693323016	0.128019341	0.121379992	0.033753786
SRR28424191	30 m	0.012113132	0.806814832	0.094547577	0.06550017	0.021024289
SRR28424192	30 m	0.01401083	0.779367986	0.116359109	0.064419892	0.025842183
SRR28424193	30 m	0.017490717	0.768395482	0.087415763	0.075716553	0.050981485
SRR28424194	24 m	0.13044517	0.429469287	0.085417464	0.177854161	0.176813919
SRR28424195	24 m	0.020215259	0.782649627	0.090810136	0.080479363	0.025845615
SRR28424196	24 m	0.038445809	0.755119267	0.099781249	0.098499979	0.008153696
SRR28424197	24 m	0.034796362	0.722611413	0.112509334	0.087183795	0.042899095
SRR28424198	24 m	0.019269015	0.784865613	0.092801114	0.062154902	0.040909357
SRR28424199	24 m	0.02122956	0.742663978	0.106257824	0.089637911	0.040210727
SRR28424200	24 m	0.017746004	0.751037935	0.088999062	0.080052695	0.062164305
SRR28424201	24 m	0.087209675	0.079022237	0.040999366	0.749265043	0.043503679
SRR28424202	24 m	0.009090897	0.812049745	0.083498555	0.056585612	0.038775191
SRR28424203	24 m	0.012741474	0.792885615	0.081954092	0.074682307	0.037736511
SRR28424204	15 m	0.013092129	0.791739204	0.117831926	0.044449184	0.032887558
SRR28424205	15 m	0.013366529	0.823373475	0.113499394	0.038085096	0.011675506
SRR2925235	2 m	0.006297688	0.874097035	0.078832233	0.039415433	0.001357612
SRR2925236	2 m	0.012091399	0.812977578	0.116425984	0.052123009	0.00638203
SRR2925237	2 m	0.008883859	0.836561458	0.115515401	0.032662079	0.006377202
SRR2925238	2 m	0.009186837	0.821296026	0.122284936	0.034740414	0.012491788
SRR2925239	9 m	0.013913656	0.832126381	0.098214103	0.039135084	0.016610777
SRR2925240	9 m	0.010820851	0.811111662	0.115506778	0.039774519	0.02278619
SRR2925241	9 m	0.007270989	0.82175219	0.116615093	0.039860239	0.014501489

SRR2925242	9	m	0.009092242	0.878570251	0.068660667	0.039137552	0.00453929
SRR2925243	9	m	0.008165961	0.839955521	0.113435403	0.029691459	0.008751655
SRR2925244	15	m	0.013245839	0.711166621	0.140095576	0.082118302	0.053373661
SRR2925245	15	m	0.0076149	0.853739446	0.091259001	0.032654707	0.014731946
SRR2925246	15	m	0.010653186	0.791254098	0.11462602	0.06120708	0.022259615
SRR2925247	15	m	0.00798404	0.700952088	0.139373592	0.094524091	0.05716619
SRR2925248	15	m	0.009754425	0.738587098	0.140561193	0.05756458	0.053532704
SRR2925249	24	m	0.021491502	0.661745342	0.127171576	0.109208242	0.080383338
SRR2925250	24	m	0.015158762	0.74787658	0.092050698	0.096929144	0.047984817
SRR2925251	24	m	0.028606773	0.666267421	0.121689289	0.083308714	0.100127803
SRR2925252	24	m	0.009035738	0.798178895	0.12137951	0.040479096	0.030926761
SRR2925253	24	m	0.018911739	0.764648135	0.139760732	0.056046689	0.020632704
SRR2925254	30	m	0.026894142	0.71200682	0.144426916	0.092182063	0.024490058
SRR2925255	30	m	0.047990046	0.593946987	0.101358218	0.165765949	0.0909388
SRR2925256	30	m	0.015714842	0.767909505	0.136543414	0.065683141	0.014149098
SRR2925257	30	m	0.025733182	0.650402688	0.143852459	0.098984405	0.081027265
SRR2925258	30	m	0.013544475	0.758464459	0.133004137	0.066312764	0.028674165
SRR30855989	3	f	0.010021317	0.82753342	0.123454303	0.030294741	0.008696219
SRR30856001	3	f	0.011089665	0.837431087	0.103461059	0.033976189	0.014042
SRR30856011	2.75	f	0.017582562	0.833816248	0.110600873	0.027069605	0.010930712
SRR30856023	3	f	0.012157448	0.804657743	0.123055864	0.044014744	0.016114201
SRR30856036	2.75	f	0.009741933	0.817836646	0.117153473	0.036889219	0.018378729
SRR30856047	2.75	f	0.011292052	0.823652492	0.117234815	0.035490456	0.012330184
SRR30856072	2.75	f	0.011473267	0.856548402	0.099979043	0.022578576	0.009420713
SRR30856083	3	f	0.010353825	0.831985644	0.10714261	0.034551738	0.015966183
SRR32613941	14	m	0.207191932	0.00057614	0.510041532	0.000532713	0.281657682
SRR32613944	20	m	0.127152553	0.256535939	0.326261161	0	0.290050347
SRR32613945	14	m	0.161844466	0	0.577979863	0.000845849	0.259329822
SRR32613949	20	m	0.033548294	0.761967005	0.14336057	0.038454244	0.022669887
SRR32613950	14	m	0.076188127	0.435386366	0.268724829	0	0.219700678
SRR32613954	26	m	0.091701931	0.420598705	0.214437656	0.083695773	0.189565935

SRR32613955	20 m	0.102156417	0.181914555	0.450105643	0	0.265823386
SRR32613977	14 m	0.149144802	0.123668233	0.264729	0	0.462457965
SRR32613981	26 m	0.039915062	0.780069299	0.125136252	0.017472633	0.037406755
SRR32613982	20 m	0.029060672	0.667234119	0.169696941	0.001398899	0.132609368
SRR32613983	14 m	0.122830054	0.000270161	0.588336356	0.002471181	0.286092248
SRR32613984	14 m	0.13025454	0.000318269	0.55929106	0.00501302	0.305123112
SRR32613989	26 m	0.147330248	0.042509154	0.482823975	0	0.327336623
SRR32613990	20 m	0.112644415	0.13772092	0.363375745	0	0.386258921
SRR32613991	14 m	0.115217745	0.000758464	0.623561101	0.005158951	0.255303739
SRR32613995	26 m	0.102433139	0.08359912	0.50898106	0.000508799	0.304477882
SRR32613996	20 m	0.098625	0.028536462	0.547554842	0	0.325283696
SRR32613997	14 m	0.185340146	0.000201365	0.533555733	0.002247927	0.278654829
SRR32614109	26 m	0.196155879	0.147670548	0.354917067	0.007090561	0.294165945
SRR32614110	20 m	0.21554867	0.150572721	0.373263299	0.000218412	0.260396897
SRR32614111	14 m	0.15876221	0.000242652	0.584797828	0.003933432	0.252263877
SRR5642546	29 m	0.03525541	0.658709714	0.226358723	0.024540902	0.055135252
SRR5642547	29 m	0.022478088	0.502044672	0.242944979	0.05576761	0.176764652
SRR5642548	29 m	0.013874813	0.603104498	0.207013253	0.01642531	0.159582125
SRR5642552	12 m	0.020386223	0.703235875	0.208837854	0.016104884	0.051435164
SRR5642556	12 m	0.01210101	0.601681563	0.241751931	0.008324273	0.136141223
SRR5642557	12 m	0.008660593	0.675231812	0.217186139	0.002374737	0.096546719
SRR5642558	3 m	0.01536143	0.586445385	0.21812617	0.002539855	0.17752716
SRR5642559	3 m	0.010002518	0.644829749	0.18303647	0.004117713	0.15801355
SRR5642560	3 m	0.012658135	0.625948753	0.211088805	0.000819991	0.149484317
SRR5850781	12 m	0.04816297	0.689195581	0.131929275	0.073007654	0.05770452
SRR5850782	12 m	0.040620148	0.681026491	0.128438948	0.079155998	0.070758415
SRR5850783	12 m	0.021019315	0.829372992	0.101404444	0.023211496	0.024991753
SRR5850784	12 m	0.023979779	0.830791305	0.101380258	0.023988461	0.019860197
SRR5850785	12 m	0.030136078	0.718350729	0.179812116	0.020190281	0.051510797
SRR5850786	12 m	0.031495273	0.718849201	0.173698895	0.017902679	0.058053952
SRR5850787	12 m	0.019326395	0.811623876	0.123148403	0.01635395	0.029547375

SRR5850788	12	m	0.019213386	0.811022303	0.11866449	0.015750072	0.035349749
SRR5850789	12	m	0.026887063	0.792578273	0.133867088	0.021457045	0.025210532
SRR5850790	12	m	0.024965228	0.786601834	0.135010369	0.020244796	0.033177773
SRR6081860	2	m	0.007659572	0.843083445	0.058483229	0.070349224	0.02042453
SRR8145413	1.75	f	0.027541203	0.871497976	0.064519912	0.02961476	0.006826151
SRR8145414	1.75	f	0.014945332	0.838143896	0.103353501	0.021817819	0.021739452
SRR8145415	1.75	f	0.022452537	0.859501234	0.088328811	0.020200812	0.009516606
SRR8145416	1.75	f	0.01686799	0.870026294	0.075129431	0.030151061	0.007825224
SRR8145417	1.75	f	0.018253383	0.859409691	0.088637772	0.021272862	0.012426292
SRR8145418	1.75	f	0.022937763	0.848572344	0.089333316	0.024384525	0.014772052
SRR9126652	21	f	0.005784975	0.690866422	0.134966338	0.15169697	0.016685294
SRR9126667	12	f	0.009012781	0.783494427	0.141498348	0.05233979	0.013654654
SRR9126676	3	m	0.000505828	0.83549959	0.108274519	0.046221976	0.009498087
SRR9126677	15	m	0.001090297	0.820485596	0.118632333	0.045469105	0.014322668
SRR9126682	27	m	0.009260133	0.753266243	0.151577414	0.068569216	0.017326994
SRR9126712	21	m	0.004099489	0.801537138	0.111982322	0.063305894	0.019075157
SRR9126727	9	m	0.020404683	0.817545319	0.115769331	0.026348827	0.01993184
SRR9126770	18	m	0.006510608	0.733567923	0.136600168	0.093791818	0.029529483
SRR9126772	1	m	0.002856377	0.843431406	0.103358851	0.046322676	0.00403069
SRR9126806	27	m	0.000920961	0.801146987	0.129280676	0.054783105	0.013868271
SRR9126831	6	m	0.000969363	0.833726097	0.099370408	0.060488718	0.005445413
SRR9126838	12	f	0.002665612	0.78792789	0.14047188	0.056800243	0.012134374
SRR9126867	18	f	0.005908804	0.724892284	0.163701249	0.070454675	0.035042989
SRR9126881	24	m	0.004982266	0.749715349	0.121420097	0.118778622	0.005103666
SRR9126899	1	f	0.001721493	0.786973666	0.134702377	0.073211217	0.003391246
SRR9126914	9	m	0	0.856190644	0.076478111	0.067331245	0
SRR9126918	18	m	0.002419301	0.833046011	0.114736356	0.04292297	0.006875362
SRR9126980	15	f	0.000660611	0.779769515	0.150351226	0.057077221	0.012141428
SRR9127012	6	f	0.002275947	0.777769959	0.142686631	0.066343944	0.010923519
SRR9127017	1	m	0.002657009	0.850380212	0.102191836	0.037975568	0.006795375
SRR9127018	27	m	0.006495749	0.790629061	0.116754903	0.063685043	0.022435244

SRR9127050	3	m	0.000839192	0.83689769	0.107191745	0.046112493	0.00895888
SRR9127056	15	f	0.004345449	0.776309689	0.124916193	0.081545973	0.012882695
SRR9127081	9	f	0.003645884	0.784859765	0.129807453	0.064822385	0.016864513
SRR9127087	24	m	0.005104529	0.76351682	0.127884147	0.081841924	0.021652581
SRR9127099	1	m	0.001197006	0.856339326	0.093071779	0.048461327	0.000930561
SRR9127110	6	m	0.002914235	0.83388971	0.100961488	0.059198734	0.003035832
SRR9127113	21	m	0.003283238	0.76035166	0.111303149	0.111902332	0.013159621
SRR9127116	6	m	0.00037178	0.839320168	0.098810093	0.055267314	0.006230645
SRR9127121	9	m	0	0.823021298	0.125599062	0.038223715	0.013155925
SRR9127212	12	m	0.001581286	0.81361867	0.111282183	0.064485227	0.009032633
SRR9127256	3	f	2.47E-05	0.806914726	0.132977164	0.056870181	0.003213256
SRR9127280	12	m	0.003899745	0.797258744	0.132500004	0.047218569	0.019122938
SRR9127285	21	m	0.003282753	0.817178172	0.12204902	0.046271911	0.011218144
SRR9127297	15	m	0.000780552	0.854549811	0.104510435	0.034380176	0.005779026
SRR9127299	18	f	0.001183456	0.786766864	0.137909105	0.062485474	0.011655101
SRR9127306	21	m	0.001143019	0.808771121	0.124834211	0.049906292	0.015345356
SRR9127313	15	m	0.000764653	0.831636285	0.123215652	0.037081348	0.007302063
SRR9127318	24	m	0.001148189	0.811234532	0.118554732	0.063985147	0.0050774
SRR9127340	12	m	0.002174114	0.838343683	0.105741888	0.038009628	0.015730686
SRR9127346	15	m	0.001237373	0.812894142	0.125486897	0.041998363	0.018383226
SRR9127348	21	f	0.004393033	0.714052647	0.132578199	0.127808466	0.021167654
SRR9127356	18	m	0.004746818	0.801700409	0.125558747	0.055112284	0.012881742
SRR9127436	3	m	0.000909007	0.831711495	0.114924126	0.039728953	0.012726419
SRR9127488	1	f	0.003192344	0.809523574	0.132643589	0.05279809	0.001842403
SRR9127492	27	m	0.000301639	0.722288434	0.123830695	0.114828061	0.038751171
SRR9127503	6	m	0	0.871502437	0.063386846	0.065110718	0
SRR9127505	1	m	0.00267475	0.835318243	0.102958961	0.054208478	0.004839568
SRR9127512	3	m	0.000450684	0.844269456	0.111212338	0.036523724	0.007543797
SRR9127536	12	m	0.006697106	0.824405214	0.109263973	0.045776991	0.013856715
SRR9127585	6	f	0.002828241	0.766552806	0.147984619	0.077438406	0.005195928
