# Supplementary material for: LivAge: An Online Aging Clock for Murine Transcriptomic Age Estimation
Source: Aging Cell. 2026 Aug 21;25(9):e70677. doi: 10.1111/acel.70677 (PMC13498547; doi:10.1111/acel.70677)

Supplemental Figure 3

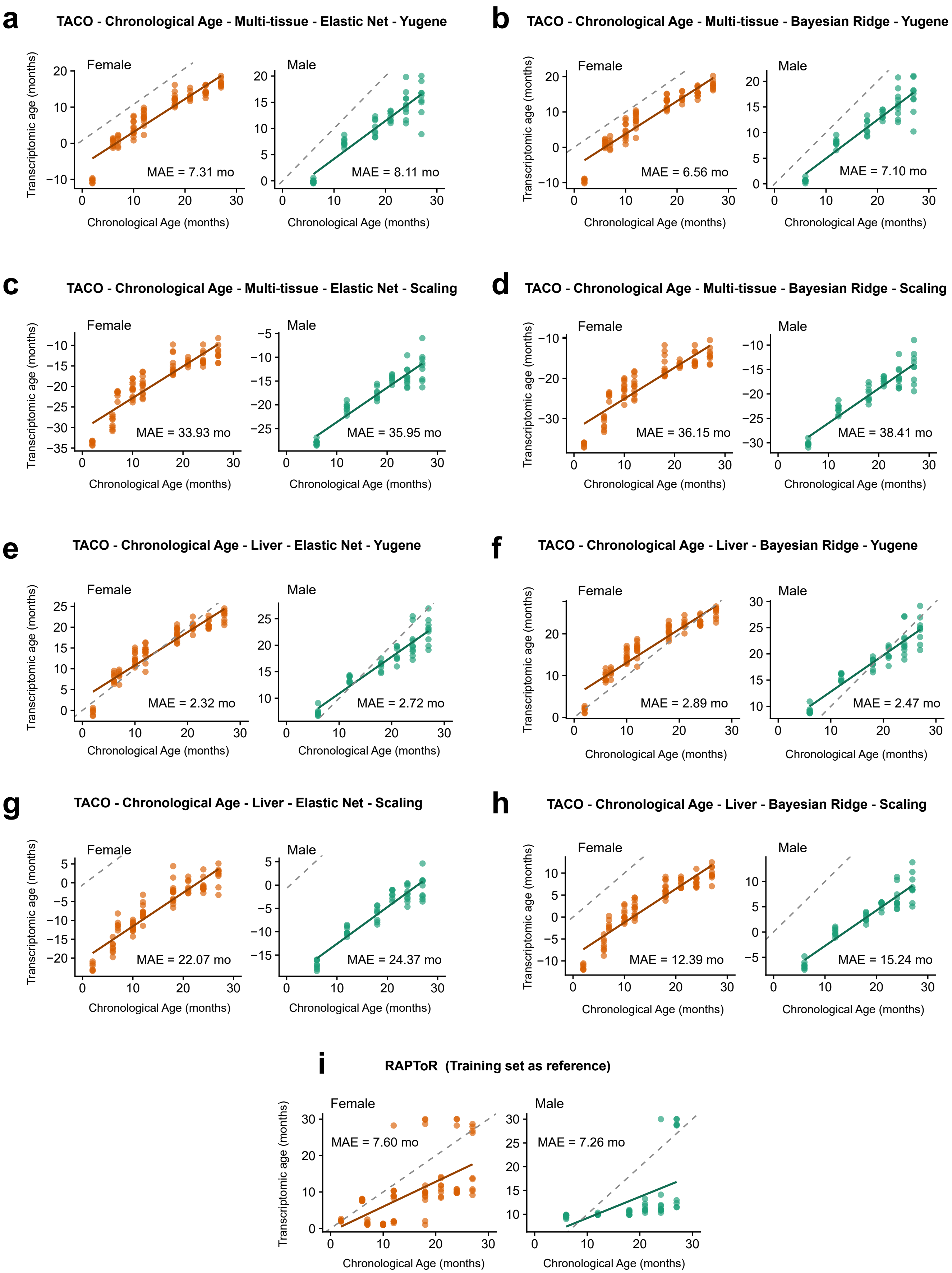

Supplement: Supplementary file 3 [file ACEL-25-e70677-s003.pdf]
