# Supplementary material for: LivAge: An Online Aging Clock for Murine Transcriptomic Age Estimation
Source: Aging Cell. 2026 Aug 21;25(9):e70677. doi: 10.1111/acel.70677 (PMC13498547; doi:10.1111/acel.70677)
Supplement: Supplementary file 4 [file ACEL-25-e70677-s004.pdf]

SUPPLEMENTARY DATA

Supplementary Data 1. Differential gene expression analysis in transcriptomic atlas of liver aging. For each gene, the table reports the Ensembl gene identifier, the mean normalized expression across all samples, the $\log_2$ fold change per month, the standard error of the $\log_2$ fold change, the Wald test statistic, the nominal P value, the adjusted P value (FDR) and the gene symbol.

Gene ID	BaseMean	LFC	lfcSE	Stat	P-val	Adj. P.	Gene symbol
ENSMUSG00000000001.5	1846.07928	-0.0033365	0.00203776	2.63277559	0.10467858	0.21744733	Gnai3
ENSMUSG00000000028.16	25.4289422	0.00148884	0.00605184	0.05776372	0.81006604	0.92193677	Cdc45
ENSMUSG00000000037.18	1.84962316	-0.0356087	0.01531336	5.07931924	0.02421288	0.06665323	Scml2
ENSMUSG00000000049.12	28618.2495	0.00437113	0.00501167	0.75903431	0.3836309	0.5753	Apoh
ENSMUSG00000000056.8	846.986034	-0.0099822	0.00280754	12.8266244	0.00034172	0.00170977	Narf
ENSMUSG00000000058.7	202.533981	-0.0018649	0.00450839	0.16544546	0.68419171	0.83856504	Cav2
ENSMUSG00000000078.8	383.847521	0.04517439	0.00471297	83.6889368	5.79E-20	3.02E-18	Klf6
ENSMUSG00000000085.17	163.123436	-0.0037663	0.0045736	0.68330442	0.40845118	0.60008281	Scmh1
ENSMUSG00000000088.8	2558.61664	-0.0208184	0.00446348	22.1146717	2.57E-06	2.06E-05	Cox5a
ENSMUSG00000000093.7	53.7866404	0.00745174	0.00349439	4.59686078	0.03203056	0.08395074	Tbx2
ENSMUSG00000000120.7	108.611302	0.02213259	0.00435318	24.574717	7.15E-07	6.30E-06	Ngfr
ENSMUSG00000000126.12	13.5598597	-0.0170118	0.00525971	10.5304774	0.00117422	0.00505543	Wnt9a
ENSMUSG00000000127.16	203.681304	-0.0001161	0.00346305	0.00113227	0.97315693	1	Fer
ENSMUSG00000000131.16	670.313018	0.00596055	0.00339344	3.07551525	0.07947941	0.17521275	Xpo6
ENSMUSG00000000134.18	471.988002	0.00211902	0.00279351	0.56592955	0.45188096	0.64364178	Tfe3
ENSMUSG00000000142.16	148.838275	-0.0047397	0.0052592	0.78523925	0.37554357	0.56663066	Axin2
ENSMUSG00000000148.18	178.777649	-0.0011011	0.00255311	0.18726303	0.6652044	0.82467887	Brat1
ENSMUSG00000000149.11	1823.82128	-0.0077906	0.00273551	8.05566956	0.00453615	0.01626723	Gna12
ENSMUSG00000000154.17	1420.98201	0.00498766	0.00405667	1.48951745	0.22229134	0.38756384	Slc22a18
ENSMUSG00000000157.17	6.20732563	0.04918441	0.01479256	9.67586835	0.00186704	0.00760947	Itgb2l
ENSMUSG00000000159.17	649.913021	-0.0021479	0.00371433	0.33523604	0.56259206	0.74355204	Igsf5
ENSMUSG00000000167.15	10.0228987	-0.0247084	0.00518566	22.4310351	2.18E-06	1.77E-05	Pih1d2
ENSMUSG00000000168.11	1142.4436	-2.85E-05	0.0032219	7.64E-05	0.99302593	1	Dlat
ENSMUSG00000000171.6	4632.28472	-0.0176086	0.00322518	30.4593395	3.41E-08	3.79E-07	Sdhd
ENSMUSG00000000183.7	1.86435703	-0.0161241	0.03509299	0.18422735	0.66776521	0.82637653	Fgf6
ENSMUSG00000000184.13	422.110793	0.01401214	0.00604124	4.91587247	0.02661102	0.07210195	Ccnd2
ENSMUSG00000000194.14	398.44128	-0.0004374	0.00299746	0.02076611	0.88541795	0.96705106	Gpr107
ENSMUSG00000000197.9	11.1063572	0.01077837	0.01114198	0.91835674	0.33790681	0.52543974	Nalcn
ENSMUSG00000000202.10	1.61225994	-0.0023458	0.03482757	0.01577877	0.9000378	0.97583394	Btbd17
ENSMUSG00000000204.17	22.0761118	0.06857463	0.01003217	37.8507521	7.64E-10	1.07E-08	Slfn4
ENSMUSG00000000216.9	1.34289148	0.02191156	0.02383696	1.07287018	0.30029804	0.48446166	Scnn1g
ENSMUSG00000000223.14	3.86822274	0.02896668	0.01357891	4.172499	0.04108516	0.10283716	Drp2
ENSMUSG00000000244.18	18.8302771	0.07999172	0.00821885	85.9529508	1.84E-20	1.01E-18	Tspan32
ENSMUSG00000000247.12	60.824524	0.02412222	0.00457463	26.7635478	2.30E-07	2.24E-06	Lhx2
ENSMUSG00000000248.17	2.12069951	0.09686256	0.01424076	40.907501	1.60E-10	2.48E-09	Clec2g
ENSMUSG00000000253.14	34.008285	-0.0017834	0.0059179	0.08938499	0.76496048	0.89336576	Gmpr
ENSMUSG00000000266.12	129.988118	0.00771053	0.00325463	5.54100262	0.01857594	0.05354828	Mid2
ENSMUSG00000000275.17	1764.55075	-0.0003123	0.00299232	0.01053133	0.91826276	0.9854466	Trim25
ENSMUSG00000000276.12	70.6904736	0.01570561	0.00434393	12.5199321	0.00040263	0.00197556	Dgke
ENSMUSG00000000278.11	840.138997	0.00910265	0.00214662	17.9966207	2.21E-05	0.00014645	Scpep1
ENSMUSG00000000282.13	185.785778	0.00798946	0.00381126	4.27609502	0.03865205	0.0977533	Mnt
ENSMUSG00000000290.14	280.639897	0.0728909	0.00552837	158.410735	2.52E-36	1.08E-33	Itgb2
ENSMUSG00000000295.14	39.4701433	0.0212015	0.00594436	12.6607016	0.00037342	0.00184567	Hddc2
ENSMUSG00000000296.9	143.59803	0.00046564	0.00298853	0.02449286	0.87563739	0.96170911	Tpd52l1
ENSMUSG00000000301.17	4938.10557	-0.0051394	0.00467926	1.21250697	0.27083619	0.44774251	Pemt
ENSMUSG00000000303.14	883.855268	-0.0674071	0.00574692	136.233035	1.77E-31	4.45E-29	Cdh1
ENSMUSG00000000305.13	24.7615175	0.00293871	0.00696422	0.16246541	0.68689674	0.84065725	Cdh4
ENSMUSG00000000308.15	9.8900467	0.00876489	0.0122032	0.471836	0.49214373	0.67988649	Ckmt1
ENSMUSG00000000317.12	57.6077042	0.00093712	0.00516886	0.03187952	0.85829234	0.95228541	Bcl6b
ENSMUSG00000000318.17	25.5644503	0.04706734	0.00958476	22.6497168	1.94E-06	1.60E-05	Clec10a
ENSMUSG00000000320.11	17.154092	0.02806538	0.00618438	20.0394576	7.59E-06	5.50E-05	Alox12
ENSMUSG00000000326.14	14717.913	-0.0261672	0.00410258	38.9925124	4.25E-10	6.23E-09	Comt
ENSMUSG00000000339.15	159.804866	0.00090916	0.00224861	0.164087	0.68542125	0.83949976	Rtca
ENSMUSG00000000340.11	1918.55897	-0.0127634	0.00351523	13.0191622	0.00030832	0.00156153	Dbt
ENSMUSG00000000346.10	2333.35328	0.00189602	0.00172451	1.21105596	0.27112309	0.44807417	Dazap2
ENSMUSG00000000355.14	397.93958	-0.0059515	0.00268393	4.89668232	0.02690834	0.07274042	Mcts1
ENSMUSG00000000359.4	13.2789365	0.03679117	0.00611353	34.817595	3.62E-09	4.67E-08	Rem1
ENSMUSG00000000365.10	4.11660494	-0.0196983	0.01412263	1.68193419	0.19466764	0.35059797	Rnf17
ENSMUSG00000000374.9	433.682682	-0.0019338	0.00226111	0.71834402	0.39668764	0.58848719	Trappc10
ENSMUSG00000000378.16	320.483457	-0.0009453	0.00205274	0.21123693	0.64579963	0.81063492	Ccm2

ENSMUSG00000000384.16	453.629105	-0.0134481	0.00193127	49.1166088	2.41E-12	4.83E-11	Tbrg4
ENSMUSG00000000385.9	104.677159	0.01649204	0.00582721	7.91033987	0.0049153	0.01742034	Tmprss2
ENSMUSG00000000386.19	38.7332648	0.05057432	0.00721566	48.177331	3.89E-12	7.59E-11	Mx1
ENSMUSG00000000392.18	5.42094144	0.01518889	0.0088895	2.97316089	0.08465629	0.18441889	Fap
ENSMUSG00000000399.11	1953.12513	-0.0147953	0.00294637	25.5255111	4.37E-07	4.02E-06	Ndufa9
ENSMUSG00000000402.3	4.26493241	0.04360144	0.01165808	13.409372	0.00025037	0.00130014	Egfl6
ENSMUSG00000000409.15	34.9468706	0.07667699	0.00728389	96.6957536	8.08E-23	5.83E-21	Lck
ENSMUSG00000000411.8	1.35868822	-0.005596	0.03847143	-0.08599	1	1	Tssk3
ENSMUSG00000000416.17	15.1699486	0.01830558	0.00874208	4.09422184	0.04303003	0.106821	Cttnbp2
ENSMUSG00000000420.16	1068.64455	0.00224389	0.00190326	1.38421881	0.23938384	0.40896039	Galnt1
ENSMUSG00000000439.10	333.369109	-0.0053057	0.00358071	2.15927806	0.14171126	0.27532217	Mkrn2
ENSMUSG00000000440.13	202.782708	0.02412497	0.00513358	19.9613538	7.90E-06	5.72E-05	Pparg
ENSMUSG00000000441.18	1516.65753	-0.0095966	0.00264861	13.2046315	0.00027926	0.00143374	Raf1
ENSMUSG00000000486.14	35.8100144	0.04915153	0.00580078	68.7778692	1.10E-16	3.80E-15	Septin1
ENSMUSG00000000489.8	43.9481009	0.04294276	0.00590455	49.2762032	2.22E-12	4.47E-11	Pdgfb
ENSMUSG00000000530.17	83.7666894	0.02848921	0.00544305	25.5283712	4.36E-07	4.02E-06	Acvrl1
ENSMUSG00000000531.6	31.2733218	-0.000433	0.00444832	0.00948086	0.92243279	0.98788476	Tamalin
ENSMUSG00000000532.12	216.219764	0.00866908	0.00335558	6.71511358	0.00955992	0.03072776	Acvr1b
ENSMUSG00000000538.19	570.17487	-0.0071931	0.00265086	7.3711209	0.00662796	0.02255803	Tom1l2
ENSMUSG00000000544.15	0.73356613	0.00715558	0.02680546	0.20185554	0.65322726	0.81497055	Gpa33
ENSMUSG00000000552.11	231.366716	0.01244302	0.00390313	10.3520098	0.00129334	0.00550328	Zfp385a
ENSMUSG00000000555.9	435.695467	-0.0025829	0.00358353	0.52893263	0.46705595	0.65810273	Itga5
ENSMUSG00000000560.10	8.22829109	0.02054264	0.01799218	1.52996265	0.21611805	0.37963509	Gabra2
ENSMUSG00000000561.15	505.375534	-0.0070718	0.00219649	10.4891202	0.0012008	0.00516071	Wdr77
ENSMUSG00000000562.6	1.85801241	0.06249568	0.01250747	23.9239007	1.00E-06	8.62E-06	Adora3
ENSMUSG00000000563.18	6747.12223	-0.0124153	0.00320602	15.1084702	0.00010151	0.00057955	Atp5pb
ENSMUSG00000000567.6	254.966148	0.01160268	0.00545724	4.14809607	0.04168137	0.10404241	Sox9
ENSMUSG00000000568.16	799.789405	0.00821364	0.00362076	5.1953939	0.02264682	0.06310822	Hnrnpd
ENSMUSG00000000579.15	26.8361132	0.01521749	0.00657809	5.45530259	0.01950903	0.05574075	Dynlt1c
ENSMUSG00000000581.9	2343.45209	-0.0107926	0.00281411	14.5129639	0.0001392	0.00076852	C1d
ENSMUSG00000000594.8	2706.70766	0.00247974	0.00256601	0.92114098	0.33717557	0.52447556	Gm2a
ENSMUSG00000000600.16	389.60709	-0.0032947	0.00225584	2.10842344	0.14649017	0.28248852	Krit1
ENSMUSG00000000605.10	1461.8838	0.00034588	0.00290808	0.01374584	0.90666776	0.97928404	Clcn4
ENSMUSG00000000627.16	3.73353974	0.00529001	0.01069695	0.20539886	0.65039789	0.81346296	Sema4f
ENSMUSG00000000628.11	257.53808	0.06640223	0.00850351	52.0396067	5.44E-13	1.19E-11	Hk2
ENSMUSG00000000631.21	1805.03418	-0.002719	0.00345836	0.61010261	0.43474918	0.62646592	Myo18a
ENSMUSG00000000632.14	4.578615	0.02524482	0.01909259	1.74799815	0.18612859	0.33902455	Sez6
ENSMUSG00000000673.10	8761.84494	-0.0069782	0.00489866	1.99656985	0.15765564	0.29833647	Haao
ENSMUSG00000000682.8	189.214418	0.0790083	0.0061631	147.630965	5.71E-34	1.90E-31	Cd52
ENSMUSG00000000686.12	548.792392	0.00015034	0.00378381	0.00157607	0.96833255	1	Abhd15
ENSMUSG00000000690.6	3.96311946	0.04217653	0.00955806	18.6427255	1.58E-05	0.00010778	Hoxb6
ENSMUSG00000000693.11	46.4191487	0.02313779	0.0055298	17.2334286	3.31E-05	0.0002105	Loxl3
ENSMUSG00000000706.17	23.7796111	0.00897614	0.03678174	0.11447543	0.73510534	0.87341668	Btn1a1
ENSMUSG00000000708.15	1691.41765	-0.0076299	0.00294181	6.76431825	0.00929984	0.03005565	Kat2b
ENSMUSG00000000711.4	2011.22831	-0.0009931	0.00181184	0.3016214	0.58286773	0.76037159	Rab5b
ENSMUSG00000000732.10	27.5732203	0.03999738	0.00569746	45.3324451	1.66E-11	2.98E-10	Icosl
ENSMUSG00000000738.19	639.668873	-0.0070998	0.00242767	8.52313249	0.0035066	0.01305965	Spg7
ENSMUSG00000000739.15	151.385406	-0.0369048	0.00683227	27.5660184	1.52E-07	1.53E-06	Sult5a1
ENSMUSG00000000740.13	5516.4232	-0.0048486	0.00365158	1.76361368	0.18417442	0.33647853	Rpl13
ENSMUSG00000000743.10	1190.08885	0.0118101	0.00414488	7.99538501	0.00468967	0.01672371	Chmp1a
ENSMUSG00000000751.14	525.767796	0.00329393	0.002848	1.33858166	0.24728425	0.41863703	Rpa1
ENSMUSG00000000753.16	9312.86783	-0.0070648	0.00369758	3.62563893	0.05689572	0.13409493	Serpinf1
ENSMUSG00000000759.15	326.974099	-0.0017257	0.00291916	0.34768714	0.55542531	0.73766887	Tubgcp3
ENSMUSG00000000776.14	151.36155	0.00256721	0.00332016	0.58831211	0.44307256	0.63512273	Polr3d
ENSMUSG00000000782.17	82.1762579	0.02066987	0.00406861	25.2106435	5.14E-07	4.67E-06	Tcf7
ENSMUSG00000000787.13	3910.59729	-0.0026825	0.00257172	1.06788373	0.30142395	0.48584605	Ddx3x
ENSMUSG00000000791.10	22.2417329	0.02948214	0.00600967	23.2258308	1.44E-06	1.21E-05	Il12rb1
ENSMUSG00000000792.3	2.41920878	0.01208168	0.01961915	0.56142806	0.45368543	0.64535459	Slc5a5
ENSMUSG00000000794.10	3.82960945	0.01989058	0.01188154	2.69230284	0.10083399	0.21151463	Kcnn3
ENSMUSG00000000804.15	648.855033	0.00459067	0.00347336	1.74510998	0.18649266	0.33953036	Usp32
ENSMUSG00000000805.19	9.78972586	0.00178098	0.01327057	0.02256368	0.88059727	0.96412269	Car4

ENSMUSG00000000811.15	154.491765	-0.0106958	0.00336452	10.1716213	0.00142619	0.00599502	Txnrd3
ENSMUSG00000000817.11	5.62468653	0.06401212	0.00957305	40.7486439	1.73E-10	2.68E-09	Fasl
ENSMUSG00000000823.17	474.714074	-0.0013128	0.00345254	0.14676452	0.70164668	0.85111286	Zfp512b
ENSMUSG00000000826.17	1197.45758	0.0027756	0.00294982	0.88895616	0.34576034	0.53423539	Dnajc5
ENSMUSG00000000827.19	748.54418	-0.0023289	0.00277421	0.70895084	0.39979233	0.59159934	Tpd52l2
ENSMUSG00000000838.18	450.030302	0.01135499	0.00258411	18.7894047	1.46E-05	0.00010032	Fmr1
ENSMUSG00000000861.16	19.363244	0.0690248	0.00824198	63.214626	1.85E-15	5.53E-14	Bcl11a
ENSMUSG00000000869.13	0.77656041	0.02188614	0.01560883	2.31942209	0.12776749	0.25460662	Il4
ENSMUSG00000000876.12	1849.1496	-0.0290047	0.00395473	51.8232974	6.07E-13	1.31E-11	Pxmp4
ENSMUSG00000000881.13	302.611907	0.00540386	0.00367733	2.15608438	0.14200616	0.27576964	Dlg3
ENSMUSG00000000884.18	28.36625	-0.001596	0.00517824	0.0962737	0.75634802	0.88747945	Gnb1l
ENSMUSG00000000901.18	24.5471308	0.0026774	0.00759242	0.12342312	0.72535109	0.86733236	Mmp11
ENSMUSG00000000902.14	308.105344	-0.0028203	0.00196147	2.07252975	0.14997258	0.28748807	Smarcb1
ENSMUSG00000000903.4	4.39392159	0.01122023	0.00977531	1.26801405	0.26013957	0.43407513	Vpreb3
ENSMUSG00000000915.16	591.0286	-0.0152548	0.0035493	17.9652143	2.25E-05	0.0001486	Hip1r
ENSMUSG00000000916.16	167.036992	-0.0058953	0.0027094	4.79246278	0.02858453	0.07642198	Nsun5
ENSMUSG00000000934.10	309.475959	-0.002609	0.00220271	1.42228127	0.23302828	0.40103649	Top1mt
ENSMUSG00000000938.12	6.29379883	0.01068628	0.02859491	0.41585404	0.51901308	0.70507099	Hoxa10
ENSMUSG00000000942.11	5.75974228	0.01392676	0.00651198	4.6239037	0.0315294	0.08285822	Hoxa4
ENSMUSG00000000948.17	24.1883935	0.02683536	0.0074103	12.7943745	0.00034766	0.00173728	Gm38393
ENSMUSG00000000957.12	622.562439	0.02246745	0.00429122	27.2821656	1.76E-07	1.75E-06	Mmp14
ENSMUSG00000000958.11	83.0507804	0.02266631	0.00453938	23.4889787	1.26E-06	1.06E-05	Slc7a7
ENSMUSG00000000959.8	640.896377	-0.0102083	0.00194863	27.7941823	1.35E-07	1.38E-06	Oxa1l
ENSMUSG00000000976.14	263.660922	-0.0075057	0.00321817	5.31827162	0.02110293	0.05949678	Heatr6
ENSMUSG00000000982.6	8.29388713	0.13266834	0.01100692	126.603884	2.27E-29	4.05E-27	Ccl3
ENSMUSG00000000983.14	2.82684443	0.02254968	0.01390888	2.16219085	0.1414429	0.27495483	Wfdc18
ENSMUSG00000001014.7	6.79505551	-0.0284596	0.00770237	13.7219084	0.00021197	0.00112347	Icam4
ENSMUSG00000001016.13	338.148267	-0.000947	0.00213945	0.19579876	0.65813355	0.81970417	Ilf2
ENSMUSG00000001017.17	999.43263	-0.0026	0.00130145	3.93748748	0.04722126	0.1152484	Chtop
ENSMUSG00000001018.16	341.078417	-0.0003918	0.00243514	0.02602099	0.87184906	0.95974199	Snapin
ENSMUSG00000001020.9	51.6663034	0.11039504	0.01104544	82.735615	9.38E-20	4.76E-18	S100a4
ENSMUSG00000001025.9	80.2814462	0.05450613	0.00772442	44.7835727	2.20E-11	3.88E-10	S100a6
ENSMUSG00000001027.8	5.19103238	0.09738732	0.01462082	36.1406729	1.84E-09	2.45E-08	Scn4a
ENSMUSG00000001029.16	57.8586597	0.00204533	0.00424405	0.23050609	0.63114882	0.7993749	Icam2
ENSMUSG00000001034.18	53.2565505	0.02895327	0.00448676	41.2427099	1.34E-10	2.12E-09	Mapk7
ENSMUSG00000001036.18	186.676913	0.01350871	0.00297747	20.5044889	5.95E-06	4.43E-05	Epn2
ENSMUSG00000001039.13	10.3135092	0.00403894	0.00740504	0.28253786	0.59504261	0.77004703	B9d1
ENSMUSG00000001052.16	428.185544	0.00234621	0.00314598	0.54729141	0.45942654	0.65096926	Sec24b
ENSMUSG00000001053.16	66.845347	-0.0019094	0.00336221	0.32270023	0.56998941	0.74962474	N4bp3
ENSMUSG00000001054.10	212.09708	0.00436554	0.0022772	3.6877113	0.05481478	0.13013201	Rmnd5b
ENSMUSG00000001056.4	398.913835	-0.0126183	0.00404502	9.77534633	0.00176867	0.00724613	Nhp2
ENSMUSG00000001062.19	271.9777	-0.0008817	0.00290531	0.09358548	0.75966751	0.89004609	Vps9d1
ENSMUSG00000001065.16	357.449247	-0.0123908	0.00428724	8.61407379	0.00333575	0.01252022	Zfp276
ENSMUSG00000001076.8	7.47732118	0.02052486	0.00765422	7.08041614	0.00779309	0.02581208	C1ql4
ENSMUSG00000001082.13	71.4517143	0.01546571	0.00535298	8.32899938	0.00390171	0.01430305	Mfsd10
ENSMUSG00000001089.15	450.256878	-0.0004828	0.00397399	0.01447166	0.90424693	0.97788024	Luzp1
ENSMUSG00000001095.6	41.7816676	-0.0466284	0.0074176	38.6950846	4.95E-10	7.17E-09	Slc13a2
ENSMUSG00000001098.16	308.214474	0.00688926	0.00296902	5.3471913	0.02075568	0.05874757	Kctd10
ENSMUSG00000001100.11	1558.18436	-0.0071271	0.00174885	16.7727517	4.21E-05	0.00026374	Poldip2
ENSMUSG00000001103.8	11.2507637	-0.0096988	0.0074577	1.66529877	0.19688941	0.35386982	Sebox
ENSMUSG00000001105.16	1115.45284	-0.0081916	0.00304916	7.22832697	0.00717621	0.02411981	Ift20
ENSMUSG00000001119.8	227.223137	0.01699269	0.0051157	10.9041031	0.00095951	0.00423328	Col6a1
ENSMUSG00000001120.16	22.5390952	0.00742008	0.00554187	1.76504456	0.18399654	0.3362317	Pcbp3
ENSMUSG00000001123.16	7915.99546	0.00185178	0.00353447	0.27778231	0.59815847	0.77262681	Lgals9
ENSMUSG00000001127.13	1134.28004	-0.0017459	0.00159346	1.20486917	0.27235066	0.44968609	Araf
ENSMUSG00000001128.8	673.177945	0.04221454	0.00585635	48.7379967	2.93E-12	5.81E-11	Cfp
ENSMUSG00000001131.12	17.3860151	0.09797409	0.01303288	51.7720608	6.23E-13	1.35E-11	Timp1
ENSMUSG00000001134.10	364.832944	-0.0116109	0.0035668	10.6447056	0.00110386	0.00478699	Uxt
ENSMUSG00000001138.14	232.830078	-0.0104338	0.00366066	8.06176299	0.00452092	0.01622248	Cnnm3
ENSMUSG00000001143.14	275.453423	-0.0155018	0.00259012	34.4955185	4.27E-09	5.46E-08	Lman2l
ENSMUSG00000001150.8	498.033807	-0.010798	0.00491647	4.80976283	0.02829894	0.07580452	Mcm3ap

ENSMUSG00000001151.11	280.638727	0.00187784	0.00358817	0.27271474	0.60151649	0.7752629	Pcnt
ENSMUSG00000001155.14	6219.0525	-0.009704	0.00494737	3.90644115	0.04810136	0.1168643	Ftcd
ENSMUSG00000001156.10	131.593245	0.00980636	0.00322892	9.18965641	0.00243387	0.00955002	Mxd1
ENSMUSG00000001157.14	270.005673	5.99E-05	0.00234811	0.00064536	0.97973271	1	Gmcl1
ENSMUSG00000001158.14	425.917636	-0.0025304	0.0035009	0.52505934	0.46869145	0.65982183	Snrnp27
ENSMUSG00000001166.18	24.0246359	0.02278527	0.00635169	12.8386409	0.00033953	0.00169939	Oas1c
ENSMUSG00000001173.16	132.174901	0.01770989	0.00349419	25.7059737	3.98E-07	3.70E-06	Ocrl
ENSMUSG00000001175.17	4284.09941	0.00041049	0.00264935	0.02362386	0.87784586	0.96288297	Calm1
ENSMUSG00000001211.16	3436.63581	-0.0070207	0.00199496	12.4278568	0.00042298	0.00206208	Agpat3
ENSMUSG00000001225.13	4.68653781	0.01041629	0.01403809	0.57366678	0.4488055	0.64069281	Slc26a3
ENSMUSG00000001227.13	89.4587075	0.01353255	0.00349809	14.3551393	0.00015137	0.00083026	Sema6b
ENSMUSG00000001228.15	34.6767944	0.05163371	0.00971799	26.6384002	2.45E-07	2.37E-06	Uhrf1
ENSMUSG00000001229.10	912.225758	-0.0094168	0.00311594	8.94330294	0.00278489	0.01072573	Dpp9
ENSMUSG00000001240.14	138.092709	-0.0071947	0.00366917	3.93505467	0.0472896	0.11536746	Ramp2
ENSMUSG00000001247.17	3442.72829	-0.0077134	0.00422076	3.27626544	0.0702887	0.15873509	Lsr
ENSMUSG00000001248.16	315.304861	0.00575149	0.00452701	1.58662234	0.20780937	0.36888627	Gramd1a
ENSMUSG00000001249.15	5590.0582	-0.0110373	0.0043368	6.48878767	0.0108557	0.03421199	Hpn
ENSMUSG00000001260.11	4.80429731	0.04953018	0.01950416	46.1214168	1.11E-11	2.04E-10	Gabrg1
ENSMUSG00000001270.10	173.474939	0.03441699	0.00520268	42.7419461	6.25E-11	1.03E-09	Ckb
ENSMUSG00000001280.14	817.223874	0.00428075	0.00358786	1.3538237	0.24461076	0.41530301	Sp1
ENSMUSG00000001281.11	58.0420969	0.06952522	0.00744674	77.7554784	1.17E-18	5.11E-17	Itgb7
ENSMUSG00000001285.13	282.802125	-0.0042481	0.00292052	2.14885396	0.14267635	0.27683156	Myg1
ENSMUSG00000001288.16	117.829455	0.01505954	0.00451739	10.835728	0.0009956	0.00437694	Rarg
ENSMUSG00000001289.13	2433.3921	0.00263413	0.00471955	0.30773754	0.57907185	0.75713669	Pfdn5
ENSMUSG00000001300.17	78.0434816	0.01148603	0.00492554	5.28263839	0.02153909	0.06051469	Efnb2
ENSMUSG00000001305.6	181.537479	-0.0050993	0.00248582	4.21145736	0.04015183	0.1009402	Rrp15
ENSMUSG00000001313.13	299.934016	0.03073676	0.00392261	60.7627517	6.44E-15	1.81E-13	Rnd2
ENSMUSG00000001323.18	1906.43038	0.00204853	0.00276054	0.53287569	0.46540036	0.65639735	Srr
ENSMUSG00000001333.10	10.1738484	0.01938816	0.0151342	1.70397157	0.1917694	0.34675507	Sync
ENSMUSG00000001334.11	93.2922814	0.02593652	0.00780431	10.8700021	0.00097734	0.00430469	Fndc5
ENSMUSG00000001348.16	1836.02978	0.00502697	0.00334004	2.25131312	0.13350107	0.263096	Acp5
ENSMUSG00000001349.6	8.1899411	0.00414192	0.01112011	0.10936664	0.74086636	0.8773331	Cnn1
ENSMUSG00000001366.15	1120.46352	-0.0085161	0.00204675	17.4740528	2.91E-05	0.0001878	Fbxo9
ENSMUSG00000001376.18	257.811586	0.00436808	0.00295332	2.13486327	0.1439833	0.27890852	Vps50
ENSMUSG00000001380.8	577.614004	-0.0010466	0.00165947	0.39939686	0.52740091	0.71226041	Hars1
ENSMUSG00000001383.10	1559.8006	-0.0049393	0.00329512	2.22771762	0.13555396	0.2661873	Zmat2
ENSMUSG00000001403.14	24.2030896	0.02508898	0.01019766	5.3499466	0.02072291	0.05866888	Ube2c
ENSMUSG00000001415.11	571.201706	-0.0095836	0.00236085	16.4950599	4.88E-05	0.00030007	Smg5
ENSMUSG00000001416.14	1741.81763	-0.0096402	0.00308802	9.57946253	0.00196766	0.00796999	Cct3
ENSMUSG00000001418.14	888.117088	-0.0051941	0.0026938	3.75138255	0.05276385	0.12598585	Glmp
ENSMUSG00000001419.18	578.138551	-0.0098241	0.00375702	6.72881235	0.00948677	0.03053843	Mef2d
ENSMUSG00000001420.14	34.2477437	-0.0177793	0.00372458	22.2373665	2.41E-06	1.94E-05	Tmem79
ENSMUSG00000001424.15	2134.55689	-0.003408	0.00284575	1.41043593	0.23498404	0.40343089	Snd1
ENSMUSG00000001435.16	14237.5389	-0.0003415	0.00402392	0.00710012	0.93284795	0.99323546	Col18a1
ENSMUSG00000001436.16	108.295439	0.00205389	0.00407758	0.26249877	0.60840864	0.78110668	Slc19a1
ENSMUSG00000001440.6	2053.62661	0.00303521	0.00250862	1.44625304	0.22913014	0.39614891	Kpnb1
ENSMUSG00000001441.14	575.34415	-0.0021709	0.00247342	0.77061198	0.3800278	0.57147239	Npepps
ENSMUSG00000001444.3	7.58284474	0.05665967	0.0069443	64.8254965	8.18E-16	2.55E-14	Tbx21
ENSMUSG00000001445.11	743.092999	-0.0061833	0.00213932	8.44309333	0.00366432	0.01355076	Mrpl10
ENSMUSG00000001467.6	4019.6485	0.00926695	0.00435842	4.26636146	0.03887409	0.09822477	Cyp51
ENSMUSG00000001472.18	11986.7739	0.00358267	0.00513934	0.47403944	0.49113468	0.67881058	Tcf25
ENSMUSG00000001473.8	158.109614	0.02248487	0.00575056	14.3652683	0.00015055	0.00082652	Tubb6
ENSMUSG00000001482.16	704.666831	-0.0024908	0.00219515	1.28181456	0.25756192	0.4310854	Def8
ENSMUSG00000001493.10	11.6571467	0.00617068	0.00923271	0.43627383	0.50892643	0.69565567	Meox1
ENSMUSG00000001494.7	4.07210085	-0.0187991	0.01615266	1.41900811	0.23356673	0.40171837	Sost
ENSMUSG00000001496.16	3.0539872	-0.0085645	0.04060211	-0.0075037	1	1	Nkx2-1
ENSMUSG00000001497.19	3.21473429	0.00978065	0.01717342	0.61525103	0.43281753	0.62440725	Pax9
ENSMUSG00000001504.11	6.69245167	0.01100932	0.02809863	0.50749846	0.47622376	0.66648156	Irx2
ENSMUSG00000001506.11	301.373393	0.01830894	0.00805467	5.26878219	0.0217112	0.06087497	Col1a1
ENSMUSG00000001507.17	177.609857	-0.0018138	0.00382695	0.22689057	0.63383907	0.80159401	Itga3
ENSMUSG00000001508.16	6.76161659	-0.0069412	0.02768929	0.34614642	0.5563027	0.73845989	Sgca

ENSMUSG00000001517.15	22.6494424	0.01653119	0.01008078	2.49835697	0.11396514	0.23252438	Foxm1
ENSMUSG00000001518.13	181.370604	-0.0032458	0.00386435	0.70114418	0.4023995	0.5940411	Itfg2
ENSMUSG00000001520.13	4.51362104	0.02837472	0.01019881	7.62810732	0.00574656	0.0199321	Nrip2
ENSMUSG00000001521.13	33.3823286	0.02144583	0.00534852	15.941598	6.53E-05	0.00039029	Tulp3
ENSMUSG00000001524.15	183.381571	-0.0063129	0.00363154	3.04753846	0.08085961	0.17766945	Gtf2h4
ENSMUSG00000001525.11	1279.21374	0.01337587	0.00332381	15.8354695	6.91E-05	0.00041039	Tubb5
ENSMUSG00000001542.8	1432.74819	-0.0136165	0.00289538	22.0923018	2.60E-06	2.08E-05	Ell2
ENSMUSG00000001552.15	798.902339	0.00057986	0.0026919	0.04560355	0.83089809	0.93468605	Jup
ENSMUSG00000001555.11	27.4514824	0.01822112	0.00586018	9.55331501	0.00199589	0.00807046	Fkbp10
ENSMUSG00000001558.6	1.69180602	0.01005379	0.01760345	0.45257251	0.50111584	0.68785612	Klhl10
ENSMUSG00000001569.11	426.139469	8.69E-05	0.0036085	0.00057743	0.98082892	1	Nom1
ENSMUSG00000001576.16	1267.71583	-0.0036295	0.00210679	2.93657154	0.08659435	0.18775643	Ergic1
ENSMUSG00000001583.14	3.85930792	-0.0002753	0.01118379	0.00124041	0.9719047	1	Tnk1
ENSMUSG00000001588.13	53.2534497	0.03004696	0.00444065	43.5412495	4.15E-11	7.00E-10	Acap1
ENSMUSG00000001604.15	1108.30488	0.00061467	0.00335046	0.03344391	0.85489472	0.95038526	Tcea3
ENSMUSG00000001627.13	483.905917	0.00178214	0.00410615	0.19625135	0.65776381	0.81941675	Ifrd1
ENSMUSG00000001630.14	500.260893	0.0094721	0.0025431	13.8222429	0.00020094	0.00107179	Stk38l
ENSMUSG00000001632.17	487.228899	0.00239968	0.00381561	0.39521322	0.52957176	0.71410894	Brpf1
ENSMUSG00000001642.19	228.091322	0.01213488	0.00532787	5.0280084	0.02494056	0.06830999	Akr1b1
ENSMUSG00000001661.6	2.34701311	0.01663702	0.01769662	1.46337927	0.22639321	0.39263223	Hoxc6
ENSMUSG00000001663.11	4022.44296	0.00358838	0.00328868	1.17511788	0.27835214	0.45731497	Gstt1
ENSMUSG00000001665.12	1348.19489	0.02563061	0.00421498	36.399107	1.61E-09	2.17E-08	Gstt3
ENSMUSG00000001666.9	5692.94048	-0.0061719	0.0037421	2.71981629	0.09910942	0.20873246	Ddt
ENSMUSG00000001670.14	24189.1199	-0.0069301	0.00634954	1.22530953	0.26832117	0.44451942	Tat
ENSMUSG00000001672.15	22.6465248	0.00471491	0.00656138	0.46694199	0.49439737	0.68186851	Marveld3
ENSMUSG00000001674.12	330.036816	-0.0011594	0.00307164	0.14156848	0.70672669	0.85451121	Ddx18
ENSMUSG00000001687.16	1359.03068	0.00908176	0.00203264	19.674513	9.18E-06	6.56E-05	Ubl3
ENSMUSG00000001700.11	739.882923	-0.0073205	0.00244276	8.91863038	0.00282276	0.01085564	Gramd2b
ENSMUSG00000001707.9	277.63949	-0.0140931	0.00372225	14.079511	0.00017524	0.00095071	Eef1e1
ENSMUSG00000001729.15	929.466868	0.00277285	0.00294805	0.88063428	0.34802801	0.53608449	Akt1
ENSMUSG00000001739.15	10.5684674	0.01095902	0.00748152	2.05290843	0.15191574	0.29026933	Cldn15
ENSMUSG00000001741.13	97.5410468	0.03181118	0.00384882	64.46305	9.84E-16	3.04E-14	Il16
ENSMUSG00000001750.17	743.878825	0.01706977	0.00447327	14.7073116	0.00012556	0.00070159	Tcirg1
ENSMUSG00000001751.10	456.146787	0.00297128	0.00263775	1.27631419	0.25858548	0.43203209	Naglu
ENSMUSG00000001755.13	2192.98827	-0.005023	0.00315552	2.49321461	0.11433797	0.23309974	Coasy
ENSMUSG00000001761.8	126.75331	-0.0037077	0.0035205	1.10261964	0.29369192	0.47588745	Smo
ENSMUSG00000001763.15	188.548452	-0.0399714	0.00367429	115.864893	5.09E-27	6.32E-25	Tspan33
ENSMUSG00000001767.6	309.731683	-0.0051393	0.00259234	3.84256104	0.04996714	0.12052584	Crnkl1
ENSMUSG00000001768.17	333.802722	0.00458585	0.00267599	2.867555	0.09038208	0.1940686	Rin2
ENSMUSG00000001773.15	6.11315426	0.19469141	0.01419582	156.039094	8.30E-36	3.32E-33	Folh1
ENSMUSG00000001774.9	926.071073	-0.0083757	0.00376687	4.673795	0.03062618	0.08090464	Chordc1
ENSMUSG00000001783.4	992.730878	0.00114184	0.00206502	0.30741118	0.57927315	0.75731592	Rtcb
ENSMUSG00000001785.14	277.605473	0.00087381	0.00233909	0.13739975	0.71087982	0.85719895	Pwp1
ENSMUSG00000001786.15	307.630749	0.0006253	0.00227044	0.07623012	0.78247278	0.90508382	Fbxo7
ENSMUSG00000001794.14	1536.45479	0.00261337	0.00227963	1.30389891	0.25350227	0.42580149	Capns1
ENSMUSG00000001802.17	282.620438	-0.0181151	0.00338653	28.3927016	9.90E-08	1.03E-06	Lrp3
ENSMUSG00000001815.16	49.8115475	0.00942275	0.04381476	0.20418165	0.6513665	0.81397256	Evx2
ENSMUSG00000001827.13	7.6903941	0.01746056	0.01402596	1.42449244	0.23266538	0.40058732	Folr1
ENSMUSG00000001829.18	654.459323	-0.0125126	0.00294784	17.8679702	2.37E-05	0.00015573	Clpb
ENSMUSG00000001833.18	1546.57983	0.00649724	0.0024673	6.68696378	0.00971205	0.03113611	Septin7
ENSMUSG00000001844.11	244.269466	-0.0018494	0.00188826	0.97357568	0.32379003	0.51048875	Zdhhc4
ENSMUSG00000001847.15	2404.68704	0.00387692	0.00166237	5.44621542	0.01961077	0.05598396	Rac1
ENSMUSG00000001855.16	288.687428	0.00712476	0.00439775	2.6711683	0.10218099	0.21366108	Nup214
ENSMUSG00000001864.14	19.3645177	-0.0043796	0.01144889	0.12286121	0.72595176	0.86776026	Aif1l
ENSMUSG00000001865.3	2.37891425	0.07106207	0.0188325	7.23507767	0.00714927	0.02404774	Cpa3
ENSMUSG00000001870.17	115.998857	0.03265152	0.00650474	24.8979643	6.04E-07	5.43E-06	Ltbp1
ENSMUSG00000001891.17	7178.29952	-0.0059338	0.00280914	4.5021836	0.0338516	0.08789342	Ugp2
ENSMUSG00000001909.17	447.986477	-0.0102496	0.00393493	6.86040458	0.00881266	0.02868158	Trmt1
ENSMUSG00000001910.6	522.395861	-0.0014961	0.00261947	0.32145681	0.57073347	0.75031044	Nacc1
ENSMUSG00000001911.17	1677.97012	-0.0135071	0.00497715	7.25256621	0.00707998	0.02383845	Nfix
ENSMUSG00000001918.18	81.802018	0.0389907	0.00573677	43.3820468	4.50E-11	7.56E-10	Slc1a5

ENSMUSG00000001924.16	2987.60698	-0.0028595	0.00172672	2.75439023	0.09698779	0.20529765	Uba1
ENSMUSG00000001930.18	155.241151	1.69E-05	0.00569696	6.86E-06	0.99790982	1	Vwf
ENSMUSG00000001942.9	777.531155	0.00240285	0.00328091	0.53886079	0.46290524	0.65416914	Siae
ENSMUSG00000001943.9	8.11241045	0.00550444	0.00632418	0.75679011	0.38433492	0.57595295	Vsig2
ENSMUSG00000001946.15	195.006675	-0.000871	0.00299469	0.08465716	0.77108265	0.8974886	Esam
ENSMUSG00000001948.11	19.2375449	-0.0066366	0.00754277	0.75923542	0.3835679	0.5752421	Spa17
ENSMUSG00000001962.9	325.181103	0.0062593	0.00225235	7.7745669	0.00529869	0.01858916	Fam50a
ENSMUSG00000001964.15	244.767331	0.00665299	0.0027522	5.7060873	0.01690618	0.04955868	Emd
ENSMUSG00000001983.8	261.875168	-0.0108159	0.00290992	13.7495851	0.00020887	0.00110854	Taco1
ENSMUSG00000001985.10	4.37464863	0.03451977	0.01483038	5.86297568	0.01546263	0.04602494	Grik3
ENSMUSG00000001986.17	46.0312341	-0.0001246	0.00723971	0.00027926	0.98666706	1	Gria3
ENSMUSG00000001995.10	119.738655	0.0082666	0.0051365	2.59894616	0.1069348	0.22118055	Sipa1l2
ENSMUSG00000001998.16	112.161634	-0.0013597	0.00331813	0.16438246	0.68515333	0.83935108	Ap4e1
ENSMUSG00000001999.16	247.950585	0.00880302	0.00308303	8.11178915	0.00439783	0.01586528	Blvra
ENSMUSG00000002006.13	11.2337784	0.02625368	0.00785935	11.3655403	0.00074819	0.00340411	Pdzd4
ENSMUSG00000002007.6	8.00556437	0.07694271	0.01117788	39.5420768	3.21E-10	4.76E-09	Srpk3
ENSMUSG00000002010.18	1097.03386	-0.0049369	0.00299314	2.72465304	0.09880959	0.20824976	Idh3g
ENSMUSG00000002012.14	4.61501395	0.02012716	0.00944354	4.56708551	0.03259197	0.08514778	Pnck
ENSMUSG00000002014.13	2086.68429	-0.0130946	0.00341441	14.7335641	0.00012382	0.00069271	Ssr4
ENSMUSG00000002015.6	2097.40888	-0.0042933	0.00212208	4.09124711	0.04310583	0.10698232	Bcap31
ENSMUSG00000002017.11	421.59969	-0.0070064	0.00236423	8.76848491	0.0030648	0.01163095	Fam98a
ENSMUSG00000002020.16	32.2668344	0.04485012	0.0082223	27.4050122	1.65E-07	1.65E-06	Ltbp2
ENSMUSG00000002028.14	590.591937	0.00388751	0.00448427	0.73163761	0.39235289	0.58359307	Kmt2a
ENSMUSG00000002031.14	391.56399	-0.0022191	0.00225392	0.99883998	0.31759136	0.50317761	Ift46
ENSMUSG00000002032.18	581.118607	-0.013384	0.00478254	7.58942927	0.00587115	0.02028967	Tmem25
ENSMUSG00000002033.15	23.9262713	0.10101797	0.00733887	163.108008	2.37E-37	1.21E-34	Cd3g
ENSMUSG00000002043.18	201.057949	-0.0039652	0.00330602	1.46711663	0.22580118	0.39191192	Trappc6a
ENSMUSG00000002052.7	885.520693	-0.0046205	0.00198622	5.38648073	0.02029336	0.05766863	Supt6
ENSMUSG00000002055.10	23.1233506	0.01025068	0.01130595	0.74742143	0.38729385	0.57887914	Spag5
ENSMUSG00000002058.14	130.354808	0.02033612	0.00419883	22.5055705	2.10E-06	1.71E-05	Unc119
ENSMUSG00000002059.19	89.7891077	0.00540426	0.00358517	2.30867267	0.12865387	0.25587572	Rab34
ENSMUSG00000002064.11	580.48209	-0.0079911	0.00298657	7.18614415	0.00734687	0.02460338	Sdf2
ENSMUSG00000002068.17	27.2726746	0.00501218	0.00834623	0.34050011	0.55954069	0.74092335	Ccne1
ENSMUSG00000002076.13	49.8762688	0.11743603	0.00777008	180.954909	3.00E-41	2.36E-38	Hsf2bp
ENSMUSG00000002083.14	145.342619	0.00771942	0.00449691	2.91134286	0.08795861	0.19006796	Bbc3
ENSMUSG00000002100.16	380.838568	0.05942395	0.0166515	7.33636054	0.00675735	0.02292892	Mybpc3
ENSMUSG00000002102.16	1950.1294	-0.0043617	0.00284886	2.30973814	0.12856571	0.2557511	Psmc3
ENSMUSG00000002103.17	1149.71458	0.0015559	0.00324422	0.2239886	0.63601747	0.80335577	Acp2
ENSMUSG00000002104.12	2.55828912	-0.0060874	0.0164102	0.21073064	0.64619534	0.8108727	Rapsn
ENSMUSG00000002105.16	162.618568	0.00986653	0.002856	12.0308204	0.00052328	0.00249234	Slc39a13
ENSMUSG00000002107.20	361.741772	0.01991149	0.00385707	25.0149359	5.69E-07	5.13E-06	Celf2
ENSMUSG00000002108.11	2018.7502	-0.0011013	0.00292378	0.14058911	0.70769606	0.85524001	Nr1h3
ENSMUSG00000002109.15	111.811158	0.01062583	0.00317758	11.4216276	0.00072594	0.00331758	Ddb2
ENSMUSG00000002111.10	208.758481	0.05552943	0.00674079	60.0388537	9.30E-15	2.57E-13	Spi1
ENSMUSG00000002129.12	455.600756	-0.0009245	0.0024138	0.14591526	0.70246985	0.85158583	Sf3a1
ENSMUSG00000002147.19	1078.03455	-0.0044437	0.00184317	5.75923301	0.01640223	0.04830357	Stat6
ENSMUSG00000002190.14	11.3435413	0.0400209	0.02192417	3.93702926	0.04723412	0.11526787	Clgn
ENSMUSG00000002204.10	50.0275256	0.09800153	0.00853593	109.348249	1.36E-25	1.38E-23	Napsa
ENSMUSG00000002205.17	406.003668	0.00209581	0.00195338	1.15880973	0.28171253	0.46145369	Vrk3
ENSMUSG00000002210.12	135.801683	0.00521437	0.00303957	2.90995073	0.08803456	0.19017982	Smg9
ENSMUSG00000002221.12	333.085692	-0.0086724	0.00260248	11.1383195	0.00084562	0.00378893	Paxip1
ENSMUSG00000002222.15	2744.66044	0.00378129	0.00264946	1.98293575	0.15908147	0.30021568	Rmnd5a
ENSMUSG00000002227.16	401.169659	0.00537808	0.00353941	2.36276963	0.1242614	0.24886044	Mov10
ENSMUSG00000002228.8	7.3734177	0.01566722	0.00861149	3.10565871	0.07802066	0.17273782	Ppm1j
ENSMUSG00000002233.14	224.397248	0.01609959	0.00458493	11.9185744	0.00055577	0.00262378	Rhoc
ENSMUSG00000002249.22	159.693377	-0.0006624	0.00369575	0.03140275	0.85934484	0.95283438	Tead3
ENSMUSG00000002250.17	229.546746	-0.0175675	0.00626646	7.77520058	0.00529683	0.01858541	Ppard
ENSMUSG00000002257.9	12.126963	0.05521149	0.00873195	34.9876748	3.32E-09	4.30E-08	Def6
ENSMUSG00000002265.18	187.446514	-0.0359847	0.00527767	46.5317243	9.01E-12	1.68E-10	Peg3
ENSMUSG00000002266.18	4.6692437	-0.0222458	0.01385338	2.36728833	0.12390211	0.24829166	Zim1
ENSMUSG00000002274.15	66.3740182	-0.0067445	0.00505838	1.83704168	0.17529808	0.32382746	Metrn

ENSMUSG00000002279.20	615.650898	-0.0111999	0.00263032	18.013605	2.19E-05	0.00014535	Lmf1
ENSMUSG00000002280.12	431.659602	-0.0140535	0.00330857	18.2953545	1.89E-05	0.00012715	Ciao3
ENSMUSG00000002289.17	1578.67698	-0.041414	0.00528423	56.8013251	4.82E-14	1.20E-12	Angptl4
ENSMUSG00000002297.17	70.9005036	0.00330907	0.00508283	0.40637113	0.52381714	0.70922797	Dbf4
ENSMUSG00000002307.17	229.442169	0.00483554	0.00289524	2.7952098	0.09454639	0.20129727	Daxx
ENSMUSG00000002308.17	87.6503981	-0.0010889	0.00385806	0.08052778	0.77658345	0.90113634	Cd320
ENSMUSG00000002319.17	470.239426	-0.0058755	0.00351244	2.81566003	0.09334849	0.19925046	Ipo4
ENSMUSG00000002320.16	993.986057	-0.0014309	0.00194086	0.54476856	0.46046317	0.65181157	Tm9sf1
ENSMUSG00000002324.9	42.2820149	0.00998677	0.00606463	2.6860548	0.10123017	0.21219471	Rec8
ENSMUSG00000002325.16	685.005814	0.00987286	0.00249371	15.6830406	7.49E-05	0.00044116	Irf9
ENSMUSG00000002326.7	224.703272	-0.0013683	0.00235098	0.33876779	0.56054134	0.74178867	Gmpr2
ENSMUSG00000002329.8	324.715771	-0.0085995	0.00263619	10.7491244	0.00104329	0.00455968	Mdp1
ENSMUSG00000002332.17	2105.10406	-0.0123982	0.00320668	14.8080805	0.00011902	0.0006692	Dhrs1
ENSMUSG00000002341.9	9.90696545	0.0213861	0.01779257	1.6502694	0.19892227	0.35673514	Ncan
ENSMUSG00000002342.18	281.337688	-0.0082952	0.00365038	5.15499968	0.02317954	0.06429644	Tmem161a
ENSMUSG00000002343.12	77.5585021	-0.0061402	0.00379103	2.55890582	0.10967447	0.22578179	Armc6
ENSMUSG00000002345.19	183.428698	-0.0032484	0.00206535	2.49310459	0.11434597	0.23309974	Borcs8
ENSMUSG00000002346.18	1337.28177	-0.0240329	0.00346266	46.4495247	9.40E-12	1.75E-10	Slc25a42
ENSMUSG00000002365.11	850.111779	0.00010545	0.00159305	0.00438421	0.94720797	1	Snx9
ENSMUSG00000002372.10	487.914047	-0.0030205	0.00191052	2.51621805	0.11268054	0.2304611	Ranbp3
ENSMUSG00000002379.8	1026.74303	-0.0143121	0.00408914	12.4900051	0.00040914	0.00200329	Ndufa11
ENSMUSG00000002384.3	4.75458044	0.04171956	0.01379378	8.16972089	0.00425955	0.01543014	Bmp8b
ENSMUSG00000002393.15	1311.02385	-0.0095134	0.00263696	12.9704374	0.00031645	0.00159755	Nr2f6
ENSMUSG00000002395.15	923.692909	-0.0031943	0.00316084	1.02464567	0.31141957	0.4964611	Use1
ENSMUSG00000002396.9	182.33654	-0.0029428	0.0034271	0.72707556	0.39383276	0.58524064	Ocel1
ENSMUSG00000002409.19	165.637802	-0.003345	0.00358268	0.86670184	0.35187	0.54038324	Dyrk1b
ENSMUSG00000002413.16	464.188349	0.0059701	0.00329882	3.20569005	0.07338255	0.16452479	Braf
ENSMUSG00000002416.14	1533.00801	-0.0165963	0.004365	14.6405711	0.00013008	0.00072345	Ndufb2
ENSMUSG00000002428.13	292.994573	0.01274123	0.00281392	20.1570158	7.13E-06	5.23E-05	Hltf
ENSMUSG00000002455.15	965.8631	-0.0188006	0.00300633	38.8256246	4.63E-10	6.74E-09	Prpf6
ENSMUSG00000002458.14	52.79123	0.04478749	0.00601085	51.0279864	9.11E-13	1.92E-11	Rgs19
ENSMUSG00000002475.17	1013.14914	-0.0066216	0.00306092	4.55978427	0.0327312	0.08543581	Abhd3
ENSMUSG00000002477.6	317.730924	0.00210935	0.00370968	0.32415554	0.56912095	0.74886956	Snrpd1
ENSMUSG00000002486.16	47.3679805	-0.0049225	0.0052631	0.86725754	0.35171566	0.54025174	Tchp
ENSMUSG00000002489.17	50.4999719	0.03191879	0.00663857	22.1323417	2.54E-06	2.04E-05	Tiam1
ENSMUSG00000002496.11	1206.67557	-0.0087074	0.00476959	3.26715516	0.07068009	0.15943579	Tsc2
ENSMUSG00000002500.16	25.0379634	-0.0031351	0.01648192	-0.2559171	1	1	Rpl3l
ENSMUSG00000002504.16	371.218412	-0.0071396	0.00271479	6.96628504	0.00830598	0.02723926	Nherf2
ENSMUSG00000002524.18	996.280084	-0.0037333	0.00157327	5.67399525	0.0172182	0.05030452	Puf60
ENSMUSG00000002546.18	816.66446	0.00175859	0.00315601	0.30733757	0.57931857	0.75733332	Golga2
ENSMUSG00000002550.17	768.462415	-0.0096403	0.00311113	9.20605957	0.00241215	0.00947586	Uck1
ENSMUSG00000002565.17	3.41082667	0.05212103	0.01545396	11.4068315	0.00073175	0.00333893	Scin
ENSMUSG00000002578.19	29.8466406	-0.0296422	0.00796521	12.6900689	0.0003676	0.00181996	Ikzf4
ENSMUSG00000002580.8	1047.82257	-0.0092344	0.00370298	6.22410444	0.01260226	0.0386524	Mien1
ENSMUSG00000002588.14	13490.5194	-0.0010857	0.0048792	0.04872016	0.82530545	0.93098166	Pon1
ENSMUSG00000002602.17	621.651251	0.0417466	0.00498246	67.0508253	2.65E-16	8.69E-15	Axl
ENSMUSG00000002603.16	162.016642	0.02906201	0.00519435	29.5072644	5.57E-08	6.00E-07	Tgfb1
ENSMUSG00000002608.9	343.924225	-0.0047495	0.00286121	2.73861706	0.0979495	0.20678812	Ccdc97
ENSMUSG00000002617.15	63.5548093	0.01394812	0.00482704	8.07126119	0.00449728	0.01615241	Zfp40
ENSMUSG00000002625.11	660.66862	-0.0063001	0.00447248	1.97487867	0.15993097	0.30143294	Akap8l
ENSMUSG00000002633.5	5.09686411	-0.0019545	0.01058684	0.03204651	0.85792561	0.95209865	Shh
ENSMUSG00000002635.12	158.204204	-0.0043924	0.00291868	2.31235079	0.12834981	0.25542175	Pdcd2l
ENSMUSG00000002658.10	329.266396	0.00061168	0.00242738	0.0634569	0.8011133	0.91632046	Gtf2f1
ENSMUSG00000002660.9	721.651998	-0.0103107	0.00317902	10.5479327	0.00116318	0.00501635	Clpp
ENSMUSG00000002661.15	238.264166	-0.0185282	0.00418645	19.5847837	9.62E-06	6.85E-05	Alkbh7
ENSMUSG00000002668.9	48.4696835	0.04763509	0.00639331	51.1300683	8.64E-13	1.83E-11	Dennd1c
ENSMUSG00000002679.15	229.395922	-0.0052529	0.00276503	3.58285176	0.05837885	0.13685753	Med6
ENSMUSG00000002688.9	5.01437624	0.02744398	0.00923551	8.48567749	0.00357953	0.01329084	Prkd1
ENSMUSG00000002699.14	298.203569	0.06770039	0.00715052	80.8806986	2.40E-19	1.15E-17	Lcp2
ENSMUSG00000002718.15	657.899135	-3.28E-05	0.0021213	0.00023407	0.98779336	1	Cse1l
ENSMUSG00000002728.15	335.571738	-0.0070399	0.0032303	4.7388666	0.02948849	0.07837678	Naa20

ENSMUSG00000002731.7	308.965224	-0.006045	0.00226159	7.12633047	0.007596	0.02528356	Prkra
ENSMUSG00000002732.15	60.9151704	0.00739968	0.00414257	3.13420237	0.07666588	0.17041197	Fkbp7
ENSMUSG00000002733.9	348.930885	0.00213726	0.00168766	1.58529415	0.20799976	0.36914088	Plekha3
ENSMUSG00000002741.10	983.41168	-0.007665	0.00199438	14.7781568	0.00012093	0.0006791	Ykt6
ENSMUSG00000002748.8	1434.92464	-0.0025441	0.00301295	0.70421492	0.40137103	0.59300534	Baz1b
ENSMUSG00000002763.17	1416.22067	-0.0201484	0.0035537	31.6891631	1.81E-08	2.10E-07	Pex6
ENSMUSG00000002767.14	705.638039	-0.0099064	0.00374371	7.15915793	0.00745822	0.02489521	Mrpl2
ENSMUSG00000002768.9	871.570299	-0.0043313	0.00288298	2.26461787	0.13235886	0.26152434	Mea1
ENSMUSG00000002769.10	31076.3317	-0.0030129	0.00554805	0.28762754	0.59174488	0.76762188	Gnmt
ENSMUSG00000002771.13	5.98500266	-0.0122274	0.00786948	2.45214441	0.11736443	0.23795732	Grin2d
ENSMUSG00000002778.15	1302.48118	0.00013911	0.00237065	0.00346372	0.95306893	1	Kdelr1
ENSMUSG00000002781.20	320.080513	0.0020103	0.00398541	0.24750521	0.61883724	0.78956481	Tmem143
ENSMUSG00000002797.10	170.500296	-0.0018493	0.00347139	0.27999195	0.59670649	0.77158127	Ggct
ENSMUSG00000002799.7	14.4435792	0.01030432	0.01175277	0.7063586	0.40065533	0.59235608	Jag2
ENSMUSG00000002803.15	190.639827	-0.0033528	0.00226519	2.19037663	0.13887535	0.27115183	Btbd6
ENSMUSG00000002804.5	243.875152	-0.0008574	0.00402325	0.04582459	0.83049498	0.93458124	Nudt14
ENSMUSG00000002808.8	21.270229	-0.0054111	0.00965634	0.3006734	0.58346057	0.76080832	Epdr1
ENSMUSG00000002812.5	878.236839	0.00112807	0.00308631	0.13454845	0.71376189	0.85896158	Flii
ENSMUSG00000002814.16	119.366893	-0.0006137	0.00225825	0.07310498	0.78686844	0.90814522	Top3a
ENSMUSG00000002820.7	522.763508	-0.0118434	0.00272192	19.196062	1.18E-05	8.24E-05	Atg4d
ENSMUSG00000002825.9	45.8597122	-0.0119457	0.00409963	8.55196064	0.0034515	0.01287273	Qtrt1
ENSMUSG00000002831.16	229.430477	0.02123005	0.00759227	6.92545183	0.00849775	0.02781404	Plin4
ENSMUSG00000002833.12	470.404874	-0.0007562	0.00298426	0.06608769	0.79712068	0.91457354	Hdgfl2
ENSMUSG00000002835.10	63.5545456	0.0160943	0.00702184	5.18646223	0.02276351	0.06337348	Chaf1a
ENSMUSG00000002844.10	139.578654	-0.0019755	0.00365711	0.28677363	0.59229552	0.76787128	Adprh
ENSMUSG00000002845.15	233.799771	-0.00339	0.00418554	0.66963102	0.41318028	0.60469534	Tmem39a
ENSMUSG00000002846.10	576.46951	-0.0069981	0.00287513	5.96135272	0.01462282	0.04398594	Timmdc1
ENSMUSG00000002847.8	704.874755	-0.009866	0.00296918	11.4550933	0.00071298	0.00326406	Pla1a
ENSMUSG00000002870.9	93.92273	0.03877776	0.00592918	40.7067902	1.77E-10	2.73E-09	Mcm2
ENSMUSG00000002871.15	286.039774	0.00275695	0.00328144	0.70705452	0.40042338	0.59214046	Tpra1
ENSMUSG00000002881.15	1546.81776	0.00394491	0.00266242	2.13421328	0.14404435	0.27898094	Nab1
ENSMUSG00000002885.15	473.052064	0.02986489	0.00386637	57.9885295	2.64E-14	6.86E-13	Adgre5
ENSMUSG00000002897.6	366.063458	0.00602938	0.00332806	3.17017791	0.07499428	0.16739046	Il17ra
ENSMUSG00000002900.18	288.07596	0.01488327	0.00451448	10.5958052	0.00113345	0.00490252	Lamb1
ENSMUSG00000002908.19	61.4407549	-0.0027865	0.0041344	0.44764542	0.50345524	0.69033749	Kcnn1
ENSMUSG00000002910.12	192.238125	0.02072142	0.00449391	21.1138008	4.33E-06	3.32E-05	Arrdc2
ENSMUSG00000002944.16	2049.5895	0.0507156	0.00670272	48.0277291	4.20E-12	8.16E-11	Cd36
ENSMUSG00000002948.20	702.070978	-0.0077372	0.00424809	3.29004889	0.06970095	0.15764926	Map2k7
ENSMUSG00000002949.16	745.998234	-0.014269	0.00211798	45.8567916	1.27E-11	2.32E-10	Timm44
ENSMUSG00000002957.12	1642.33547	-0.002226	0.00212683	1.08242746	0.29815515	0.48189457	Ap2a2
ENSMUSG00000002963.18	373.850483	-0.006124	0.00343479	3.18920756	0.07412594	0.16578175	Pnkp
ENSMUSG00000002968.10	527.813783	-0.0086057	0.00281794	9.44045487	0.00212251	0.00850662	Med25
ENSMUSG00000002980.15	111.101259	-1.89E-05	0.00570264	1.15E-05	0.99729317	1	Bcam
ENSMUSG00000002981.11	1606.80853	-0.0100353	0.00262172	14.4907489	0.00014085	0.00077691	Clptm1
ENSMUSG00000002983.19	54.4369985	0.04111698	0.00559776	50.107854	1.46E-12	2.98E-11	Relb
ENSMUSG00000002984.18	535.276757	-0.0119562	0.00286882	17.6743452	2.62E-05	0.00017056	Tomm40
ENSMUSG00000002985.17	288810.951	-0.0015126	0.0040448	0.13765932	0.71061914	0.85701606	Apoe
ENSMUSG00000002992.18	4317.25703	0.01276787	0.00530592	5.77504751	0.01625528	0.04793062	Apoc2
ENSMUSG00000002996.18	1711.29379	-0.0053386	0.00264106	4.05824155	0.04395625	0.10864006	Hbp1
ENSMUSG00000002997.16	25.8244745	0.04574208	0.00689227	41.0036959	1.52E-10	2.37E-09	Prkar2b
ENSMUSG00000003031.15	580.453536	0.01167044	0.00254082	20.5777078	5.73E-06	4.28E-05	Cdkn1b
ENSMUSG00000003032.9	66.103023	0.01763949	0.00576476	8.87876202	0.00288507	0.01105202	Klf4
ENSMUSG00000003033.16	461.640869	-0.0008136	0.00176034	0.21321885	0.64425611	0.80964539	Ap1m1
ENSMUSG00000003037.17	1452.33861	0.00614759	0.00303118	4.08790711	0.04319109	0.10711936	Rab8a
ENSMUSG00000003038.16	580.463963	0.01755474	0.00376901	21.1270929	4.30E-06	3.30E-05	Hmgn2
ENSMUSG00000003039.10	1116.29239	-0.0031556	0.00292995	1.16022548	0.28141878	0.46116469	Fam32a
ENSMUSG00000003051.14	18.823218	0.04269748	0.00752907	29.8529695	4.66E-08	5.08E-07	Elf3
ENSMUSG00000003053.18	19407.7683	-0.0086992	0.0060957	1.97832081	0.15956742	0.30096418	Cyp2c29
ENSMUSG00000003062.14	338.013829	0.00796775	0.00345461	5.26342928	0.02177807	0.06101895	Stard3nl
ENSMUSG00000003068.18	1267.37506	-0.0062856	0.00329644	3.63225553	0.05666997	0.13375573	Stk11
ENSMUSG00000003070.7	13.8756017	0.00043331	0.00804399	0.00358542	0.95225249	1	Efna2

ENSMUSG00000003072.16	3408.99211	-0.0116156	0.00291978	15.994092	6.35E-05	0.00038105	Atp5f1d
ENSMUSG00000003099.12	671.603643	-0.0049646	0.00193059	6.65055412	0.0099125	0.03167265	Ppp5c
ENSMUSG00000003119.15	389.909758	0.0015996	0.00305119	0.27050177	0.60299538	0.77631809	Cdk12
ENSMUSG00000003123.16	267.236297	-0.0084738	0.00395636	4.45698481	0.0347588	0.08981388	Lipe
ENSMUSG00000003131.8	731.657648	0.00295554	0.00334936	0.76747397	0.38099964	0.57247911	Pafah1b2
ENSMUSG00000003134.11	351.556086	-0.006136	0.00445907	1.9054148	0.16747348	0.31242818	Tbc1d8
ENSMUSG00000003135.16	305.165225	-0.0019981	0.00178374	1.23558239	0.26632414	0.44195467	Cnot11
ENSMUSG00000003153.11	36.3942353	0.06472872	0.00762519	64.7634797	8.44E-16	2.63E-14	Slc2a3
ENSMUSG00000003154.17	211.419008	0.00434294	0.00298365	2.09749496	0.14754069	0.2839108	Foxj2
ENSMUSG00000003161.16	719.254025	0.01520677	0.00223988	44.7525774	2.24E-11	3.94E-10	Sri
ENSMUSG00000003166.21	1200.2429	-0.0053081	0.00214577	6.13089144	0.01328399	0.04044876	Dgcr2
ENSMUSG00000003184.16	850.589038	-0.0052293	0.00276045	3.6273526	0.05683716	0.13400743	Irf3
ENSMUSG00000003190.13	69.5849628	0.00013243	0.00318059	0.00176025	0.96653428	1	Bcl2l12
ENSMUSG00000003198.11	124.010481	-0.0011673	0.00244184	0.22308526	0.63669909	0.80373039	Zfp959
ENSMUSG00000003199.17	1145.12705	-0.0024678	0.00271031	0.82259848	0.36442153	0.55417448	Mpnd
ENSMUSG00000003200.11	329.23417	-0.0073748	0.00211101	12.3639039	0.00043771	0.00212339	Sh3gl1
ENSMUSG00000003206.8	19.5636169	0.05178686	0.00707767	50.1657819	1.41E-12	2.90E-11	Ebi3
ENSMUSG00000003208.10	77.3450628	0.00446127	0.0021393	4.33203483	0.03740151	0.09513124	Yju2
ENSMUSG00000003226.8	1733.70809	-0.0029496	0.00291115	1.00891983	0.31516175	0.5005323	Ranbp2
ENSMUSG00000003227.5	1.45367664	0.03400126	0.01293381	6.77864843	0.00922547	0.02984391	Edar
ENSMUSG00000003228.10	142.480922	-0.0076919	0.00491397	2.31948699	0.12776216	0.25460662	Grk5
ENSMUSG00000003233.13	343.630084	-0.0102869	0.00308779	11.0789318	0.00087314	0.00389372	Dvl3
ENSMUSG00000003234.17	748.211836	-0.0088701	0.00257061	12.0740417	0.00051129	0.00244163	Abcf3
ENSMUSG00000003235.14	682.357025	-0.0063313	0.00272126	5.46012765	0.01945523	0.05562072	Eif2b5
ENSMUSG00000003269.20	303.870753	-0.0087023	0.00277914	9.82688519	0.00171979	0.0070692	Cyth2
ENSMUSG00000003271.18	1.42230832	0.04171091	0.01352708	9.37180611	0.00220348	0.0087938	Sult2b1
ENSMUSG00000003273.15	10.4861292	-0.0049968	0.00929913	0.2623182	0.60853198	0.78110668	Car11
ENSMUSG00000003279.18	79.9421902	0.01283757	0.00606433	4.18818039	0.04070677	0.10212848	Dlgap1
ENSMUSG00000003282.10	7.15217626	-0.0378636	0.00925831	16.0252342	6.25E-05	0.0003756	Plag1
ENSMUSG00000003283.16	145.5788	0.06123355	0.00536844	119.450821	8.34E-28	1.20E-25	Hck
ENSMUSG00000003299.11	504.383307	-0.0089088	0.00224229	15.9010968	6.67E-05	0.00039791	Mrpl4
ENSMUSG00000003308.16	964.786872	0.00406367	0.00176656	5.24454974	0.02201563	0.06160405	Keap1
ENSMUSG00000003309.15	6.06025917	0.02217372	0.00825175	6.89987448	0.00862018	0.02816395	Ap1m2
ENSMUSG00000003316.15	897.982729	0.00667699	0.00340443	3.78153163	0.0518212	0.12413058	Glg1
ENSMUSG00000003341.10	3.74500623	-0.0295379	0.02932382	0.15086119	0.69771373	0.84834911	Atp8b3
ENSMUSG00000003344.16	390.093248	-0.002944	0.00229862	1.64415793	0.19975594	0.35795803	Btbd2
ENSMUSG00000003345.17	1110.53798	-0.0009832	0.00282368	0.12141621	0.72750355	0.86844199	Csnk1g2
ENSMUSG00000003346.15	836.734608	-0.0033294	0.00310087	1.15188538	0.28315482	0.46317287	Abhd17a
ENSMUSG00000003348.11	115.846786	0.03113148	0.0042536	50.6718963	1.09E-12	2.28E-11	Mob3a
ENSMUSG00000003352.15	17.3302246	0.04318185	0.0087141	23.845797	1.04E-06	8.95E-06	Cacnb3
ENSMUSG00000003354.7	2.39333083	-0.0157419	0.01737104	0.97376999	0.32374175	0.51044675	Ccdc65
ENSMUSG00000003355.8	219.74882	-0.0389053	0.00573548	47.1933957	6.43E-12	1.22E-10	Fkbp11
ENSMUSG00000003360.16	728.589761	0.00035931	0.00275565	0.0168378	0.89675588	0.97379762	Ddx23
ENSMUSG00000003363.16	433.469435	0.02151439	0.00388665	30.1867847	3.92E-08	4.32E-07	Pld3
ENSMUSG00000003378.10	124.018408	-0.0019649	0.00561055	0.11709023	0.73221205	0.87157259	Grik5
ENSMUSG00000003379.8	43.7148253	0.08466981	0.0072963	112.472976	2.81E-26	3.18E-24	Cd79a
ENSMUSG00000003380.12	875.958096	-0.0113021	0.00402316	7.88896189	0.00497374	0.017601	Rabac1
ENSMUSG00000003382.19	338.669054	-0.0002408	0.00253993	0.00890075	0.92483609	0.98901915	Etv3
ENSMUSG00000003402.15	1566.11123	-0.0077986	0.00220682	12.5382043	0.00039872	0.0019596	Prkcsh
ENSMUSG00000003410.9	18.438141	0.03366842	0.01636816	5.30958868	0.02120836	0.05973495	Elavl3
ENSMUSG00000003411.11	8.11887696	-0.0202809	0.00866374	5.79647491	0.01605834	0.0474805	Rab3b
ENSMUSG00000003418.12	10.7786035	0.1146024	0.01046194	100.182274	1.39E-23	1.09E-21	St8sia6
ENSMUSG00000003420.9	2525.9501	-0.0098676	0.00255668	14.8882	0.00011407	0.00064367	Fcgrt
ENSMUSG00000003421.14	284.573474	-0.000441	0.00226612	0.0379176	0.8456087	0.94426255	Nosip
ENSMUSG00000003423.16	534.269575	0.00463377	0.00354125	1.69787364	0.19256629	0.34779605	Pih1d1
ENSMUSG00000003429.12	4793.87958	-0.0031285	0.00337458	0.86152752	0.35331158	0.54196195	Rps11
ENSMUSG00000003435.10	1063.25965	-0.0009615	0.00222909	0.18580273	0.66643317	0.82559445	Supt5
ENSMUSG00000003437.15	762.581205	0.0009571	0.00311742	0.09423071	0.75886604	0.88946894	Paf1
ENSMUSG00000003438.18	483.091522	-0.0099665	0.00383121	6.86795487	0.0087755	0.02857642	Timm50
ENSMUSG00000003444.9	247.516128	-0.0058052	0.0028264	4.24867541	0.03928096	0.09906765	Med29
ENSMUSG00000003452.16	32.043879	0.03242071	0.00896174	11.0214202	0.00090065	0.00399976	Bicd1

ENSMUSG00000003458.13	804.875375	0.00216033	0.00162868	1.78871014	0.1810834	0.33204019	Ncstn
ENSMUSG00000003464.14	1978.39694	-0.0047335	0.00204236	5.4062625	0.02006463	0.05710684	Pex19
ENSMUSG00000003469.10	11.2396602	0.02345244	0.01049064	4.59153255	0.03213027	0.08416533	Phyhip
ENSMUSG00000003476.17	7.59941659	0.00715947	0.00955238	0.52954156	0.46679966	0.65787926	Crhr2
ENSMUSG00000003477.6	10499.7136	-0.0413657	0.00533522	63.2837763	1.79E-15	5.34E-14	Inmt
ENSMUSG00000003484.5	24.8631656	0.08230656	0.00759661	105.192559	1.11E-24	1.01E-22	Cyp4f18
ENSMUSG00000003500.14	62.8562416	0.02227031	0.00447677	24.1924828	8.72E-07	7.56E-06	Impdh1
ENSMUSG00000003518.14	984.800621	0.00667552	0.00261666	6.37966733	0.01154351	0.03593311	Dusp3
ENSMUSG00000003526.13	2618.61227	-0.0017965	0.00391188	0.20933549	0.64728874	0.81156057	Prodh
ENSMUSG00000003527.10	120.705319	0.00459431	0.00254175	3.30457755	0.06908711	0.15655767	Ess2
ENSMUSG00000003528.16	3663.14143	0.00155221	0.00361661	0.18087958	0.67061843	0.82877326	Slc25a1
ENSMUSG00000003531.16	847.543481	-0.0071225	0.0048873	2.14803867	0.14275215	0.27693306	Dgcr6
ENSMUSG00000003534.18	52.8937741	0.01664869	0.00675813	5.89779988	0.01515981	0.04525494	Ddr1
ENSMUSG00000003541.7	31.0302167	0.04113418	0.00803047	22.645324	1.95E-06	1.60E-05	Ier3
ENSMUSG00000003545.4	11.6290865	0.00713895	0.0113311	0.31993885	0.57164439	0.75100566	Fosb
ENSMUSG00000003546.11	947.69683	-0.0066709	0.00209828	10.1095256	0.00147505	0.00618058	Klc4
ENSMUSG00000003549.10	63.1834419	0.0031574	0.00422533	0.56434767	0.45251378	0.64434724	Ercc1
ENSMUSG00000003555.9	924.68572	0.0284986	0.00730051	14.6857455	0.000127	0.00070884	Cyp17a1
ENSMUSG00000003559.9	858.782445	0.005052	0.0024214	4.29459258	0.03823376	0.09685118	As3mt
ENSMUSG00000003573.16	41.2232919	0.01694311	0.00513192	10.6591452	0.00109528	0.00475576	Homer3
ENSMUSG00000003575.14	157.905233	0.0061056	0.00392582	2.36820913	0.12382903	0.24822638	Crtc1
ENSMUSG00000003581.15	176.320559	-0.0118826	0.00289675	16.8455503	4.05E-05	0.00025476	Rnf215
ENSMUSG00000003585.14	8777.92733	-0.0047155	0.00484866	0.92297092	0.33669611	0.5240754	Sec14l2
ENSMUSG00000003604.15	233.74177	-0.0147618	0.0029355	25.2218763	5.11E-07	4.64E-06	Aven
ENSMUSG00000003617.17	24239.303	0.01487113	0.0042925	11.9263065	0.00055347	0.002615	Cp
ENSMUSG00000003623.5	11792.6811	-0.0161531	0.00429197	13.6158365	0.00022429	0.00118028	Crot
ENSMUSG00000003644.18	303.952126	0.01567916	0.00385032	16.7281608	4.31E-05	0.00026901	Rps6ka1
ENSMUSG00000003657.10	2.42762705	0.00903259	0.03027667	0.0977711	0.75452098	0.88645993	Calb2
ENSMUSG00000003660.11	1201.21081	-0.0043252	0.00247371	3.05955237	0.08026378	0.17661176	Snrnp200
ENSMUSG00000003662.11	672.29422	-0.0061689	0.00255771	5.93018629	0.01488367	0.04459995	Ciao1
ENSMUSG00000003665.8	1.90903171	0.02249254	0.01490798	2.28390437	0.13072242	0.25894172	Has1
ENSMUSG00000003680.17	64.0895657	0.0090832	0.00401717	4.95503761	0.02601476	0.07078503	Taf6l
ENSMUSG00000003721.15	5031.70371	-0.0174852	0.00495248	11.8569639	0.00057446	0.00270067	Insig2
ENSMUSG00000003731.14	854.077366	-0.0058354	0.00279956	4.27794826	0.03860993	0.09766771	Kpna6
ENSMUSG00000003746.17	4033.69885	1.48E-05	0.00262123	3.15E-05	0.99552434	1	Man1a
ENSMUSG00000003752.10	107.858445	-0.0011371	0.00272036	0.17382269	0.67673657	0.8328736	Itpkc
ENSMUSG00000003762.14	327.395782	0.00617543	0.00433271	2.01038738	0.15622542	0.29636715	Coq8b
ENSMUSG00000003778.15	342.240639	0.00189046	0.00251864	0.55237139	0.45735036	0.64904126	Brd8
ENSMUSG00000003779.17	37.7330204	0.00602608	0.00882928	0.43815627	0.50801369	0.69481072	Kif20a
ENSMUSG00000003808.19	307.701499	-0.0083275	0.00205759	16.577885	4.67E-05	0.0002892	Farsa
ENSMUSG00000003809.15	8221.4439	-0.0092898	0.00268829	11.8820865	0.00056676	0.00266993	Gcdh
ENSMUSG00000003810.15	318.864806	-0.0011159	0.00375727	0.08843052	0.76618198	0.89430238	Mast2
ENSMUSG00000003812.14	446.018657	0.0038247	0.00486434	0.59899411	0.43896208	0.63091771	Dnase2a
ENSMUSG00000003813.16	755.944309	-0.0023777	0.00256461	0.87164815	0.35049944	0.53891002	Rad23a
ENSMUSG00000003814.9	18385.1699	-0.0048115	0.00360116	1.74318644	0.18673559	0.33985181	Calr
ENSMUSG00000003824.13	33.25711	0.02432039	0.00571887	16.6389254	4.52E-05	0.00028049	Syce2
ENSMUSG00000003847.17	721.101423	-0.0055818	0.00458627	1.45683709	0.22743404	0.39373601	Nfat5
ENSMUSG00000003848.15	136.751476	-0.0039968	0.0028189	2.00831943	0.15643853	0.29667598	Nob1
ENSMUSG00000003849.9	188.574045	0.04122437	0.00533158	57.7179296	3.03E-14	7.81E-13	Nqo1
ENSMUSG00000003863.19	2.7857448	0.04114626	0.01570917	7.51699088	0.00611197	0.02098965	Ppfia3
ENSMUSG00000003865.17	61.563652	0.00421257	0.00492668	0.71464755	0.39790524	0.58965777	Gys1
ENSMUSG00000003868.15	182.9081	-0.0081286	0.00383517	4.44306672	0.03504325	0.09036077	Ruvbl2
ENSMUSG00000003873.12	330.998922	0.00643843	0.00388905	2.73147169	0.09838858	0.20758313	Bax
ENSMUSG00000003882.6	20.4159067	0.08424257	0.00720158	119.48107	8.22E-28	1.19E-25	Il7r
ENSMUSG00000003923.15	971.55403	-0.0032448	0.00319945	1.01427024	0.31388199	0.49920571	Tfam
ENSMUSG00000003929.12	98.9001228	0.00429711	0.00388358	1.20417139	0.27248954	0.44982602	Zfp81
ENSMUSG00000003934.6	16.1722131	-0.002106	0.01031708	0.03522123	0.85113285	0.9483632	Efnb3
ENSMUSG00000003948.18	2747.5539	-0.012362	0.00308418	15.3944106	8.72E-05	0.00050498	Mmd
ENSMUSG00000003949.17	1012.93228	0.01001629	0.0049102	4.04843816	0.04421223	0.1090328	Hlf
ENSMUSG00000003955.9	1134.95773	-0.0135263	0.00343989	15.500547	8.25E-05	0.0004807	Fam162a
ENSMUSG00000003970.10	5237.49988	-0.0044134	0.00314136	1.98043256	0.15934485	0.3006165	Rpl8

ENSMUSG00000003974.7	1.93445278	0.00760662	0.02559775	0.08661063	0.76853109	0.89580303	Grm3
ENSMUSG00000003992.16	44.3315733	0.01270991	0.00488979	6.45759761	0.01104789	0.03471564	Ssbp2
ENSMUSG00000004018.10	153.810836	0.00567443	0.00365767	2.33088913	0.12682942	0.25295101	Fancl
ENSMUSG00000004031.14	6.04802415	0.01992052	0.01749263	1.73553761	0.18770524	0.34118493	Brinp2
ENSMUSG00000004032.11	173.000919	0.0080868	0.0033614	5.79203071	0.01609898	0.04757086	Gstm5
ENSMUSG00000004035.13	989.420048	0.00517774	0.00296313	3.0731277	0.0795962	0.17543742	Gstm7
ENSMUSG00000004038.10	1610.01848	0.0386336	0.00836627	20.753899	5.22E-06	3.93E-05	Gstm3
ENSMUSG00000004040.17	2251.40271	0.01234752	0.0036969	10.6716982	0.00108787	0.00472881	Stat3
ENSMUSG00000004043.15	264.071769	-0.0084825	0.00299257	7.72283477	0.00545267	0.01904886	Stat5a
ENSMUSG00000004044.10	274.98489	0.01012101	0.00489128	4.18221864	0.04085019	0.1023884	Cavin1
ENSMUSG00000004054.10	623.578291	-0.0070253	0.00263874	6.97767316	0.00825328	0.02711547	Map3k11
ENSMUSG00000004056.17	1842.33721	-0.0048999	0.00158103	9.56555961	0.00198262	0.00802368	Akt2
ENSMUSG00000004069.18	1730.17141	-0.0131487	0.00178949	54.3140652	1.71E-13	3.96E-12	Dnaja3
ENSMUSG00000004070.16	831.246703	-0.0045344	0.00229952	3.97796109	0.04609933	0.11297785	Hmox2
ENSMUSG00000004071.14	1413.92604	-0.0092985	0.00244142	14.751912	0.00012262	0.00068747	Cdip1
ENSMUSG00000004085.15	322.282848	0.00115061	0.00285469	0.1576167	0.69136031	0.84358118	Map3k20
ENSMUSG00000004096.10	1004.30898	-0.0032859	0.00303392	1.17749309	0.27786694	0.45674052	Cwc15
ENSMUSG00000004098.8	570.826937	-0.0130233	0.00497756	6.45721363	0.01105028	0.03471851	Col5a3
ENSMUSG00000004099.17	258.070707	0.01575867	0.00363021	18.5563591	1.65E-05	0.00011222	Dnmt1
ENSMUSG00000004100.13	243.934497	-0.0086705	0.00399619	4.65061775	0.03104237	0.08178792	Ppan
ENSMUSG00000004105.9	338.462864	0.01671797	0.00355736	21.7780713	3.06E-06	2.42E-05	Angptl2
ENSMUSG00000004110.19	29.098309	0.09310073	0.00950875	75.3403418	3.96E-18	1.61E-16	Cacna1e
ENSMUSG00000004113.20	8.5696922	0.06575695	0.01958776	9.05430707	0.00262077	0.01018343	Cacna1b
ENSMUSG00000004127.13	229.575003	-0.0083284	0.0050491	2.56106347	0.10952488	0.22551322	Trmt10a
ENSMUSG00000004151.18	48.9625447	0.03208549	0.00624317	24.6635598	6.83E-07	6.05E-06	Etv1
ENSMUSG00000004187.12	117.461765	-0.0097788	0.00454056	4.65511997	0.03096107	0.08163754	Kifc2
ENSMUSG00000004207.16	7824.99112	0.01574387	0.00241214	42.4593019	7.22E-11	1.18E-09	Psap
ENSMUSG00000004221.17	1454.15525	-0.0106564	0.00349646	9.07393082	0.00259279	0.01008636	Ikbkg
ENSMUSG00000004231.16	2.18781626	0.00537877	0.0305903	0.11666752	0.73267732	0.87175964	Pax2
ENSMUSG00000004233.15	100.248414	0.00567021	0.00347663	2.62749225	0.10502748	0.21805126	Wars2
ENSMUSG00000004263.16	352.924614	0.00220636	0.00474064	0.21162825	0.64549418	0.81042402	Atn1
ENSMUSG00000004264.18	2983.2328	-0.0067296	0.00244291	7.70502934	0.00550672	0.01921323	Phb2
ENSMUSG00000004266.16	557.262578	0.02593482	0.00476304	28.7135149	8.39E-08	8.82E-07	Ptpn6
ENSMUSG00000004267.17	20.9564089	0.03860322	0.01113289	10.8855856	0.00096916	0.00427421	Eno2
ENSMUSG00000004268.12	920.912161	-0.0111824	0.0031401	12.7403411	0.00035785	0.00178066	Emg1
ENSMUSG00000004270.14	1684.6248	-0.0023835	0.00282915	0.71796697	0.39681159	0.5885918	Lpcat3
ENSMUSG00000004285.13	953.037116	-0.0048252	0.0037902	1.61798835	0.20337251	0.3630047	Atp6v1f
ENSMUSG00000004296.15	2.18130362	0.07916028	0.01356296	29.93475	4.47E-08	4.88E-07	Il12b
ENSMUSG00000004317.15	153.403409	0.01411744	0.00532846	6.84072811	0.00891025	0.02895924	Clcn5
ENSMUSG00000004319.16	1031.62328	-0.0033357	0.00233133	2.01924579	0.15531627	0.2949746	Clcn3
ENSMUSG00000004328.16	4.59361919	0.00523996	0.00968677	0.33444969	0.56305062	0.74379531	Hif3a
ENSMUSG00000004341.9	35.4777008	0.07936549	0.00791804	88.5917741	4.85E-21	2.81E-19	Gpx6
ENSMUSG00000004347.18	42.4877217	0.01292035	0.00878743	1.9225047	0.16558081	0.30968114	Pde1c
ENSMUSG00000004356.9	346.630108	0.01778724	0.00386789	20.8117936	5.07E-06	3.83E-05	Utp20
ENSMUSG00000004359.17	51.8042959	0.05590962	0.00665142	64.342765	1.05E-15	3.22E-14	Spic
ENSMUSG00000004360.10	4.4598618	0.0397387	0.01587458	7.22630177	0.00718431	0.02413804	9330159F19Rik
ENSMUSG00000004364.15	1183.35515	-0.0091701	0.00260432	12.3109235	0.00045032	0.00217646	Cul3
ENSMUSG00000004366.5	5.33828339	0.01527237	0.03029223	0.45811696	0.49850533	0.68572569	Sst
ENSMUSG00000004371.16	1.26499444	0.05337288	0.01822603	17.7961235	2.46E-05	0.00016107	Il11
ENSMUSG00000004383.19	117.307058	0.0174672	0.00313156	30.695099	3.02E-08	3.40E-07	Large1
ENSMUSG00000004393.11	406.83032	-0.0030228	0.00211842	2.07221298	0.15000372	0.28750104	Ddx56
ENSMUSG00000004394.13	1038.2132	-0.0031263	0.00209427	2.22211866	0.13604625	0.26690093	Tmed4
ENSMUSG00000004446.13	257.628638	0.00658997	0.00281913	5.35918866	0.02061336	0.05844996	Bid
ENSMUSG00000004451.15	282.645445	0.01940113	0.00272454	50.0823915	1.47E-12	3.02E-11	Ralb
ENSMUSG00000004455.17	1456.02333	0.00179083	0.00268721	0.44913252	0.5027472	0.68956707	Ppp1cc
ENSMUSG00000004460.18	1620.84316	-0.0090881	0.00297985	9.00886285	0.00268674	0.01040547	Dnajb11
ENSMUSG00000004462.8	410.929271	-0.006406	0.0028811	4.896822	0.02690617	0.07274042	Tbccd1
ENSMUSG00000004473.11	41.3449365	0.01681948	0.00373543	20.0677465	7.47E-06	5.44E-05	Clec11a
ENSMUSG00000004500.10	107.099775	0.00770393	0.00342308	5.12454239	0.02358979	0.06528086	Zfp324
ENSMUSG00000004508.7	90.7383777	0.03833208	0.0048658	59.6312019	1.14E-14	3.12E-13	Gab2
ENSMUSG00000004530.13	514.292664	0.00642725	0.00355349	3.19295061	0.07395641	0.16549677	Coro1c

ENSMUSG00000004535.13	2733.72458	-0.0048335	0.00233946	4.20328481	0.04034576	0.10136291	Tax1bp1
ENSMUSG00000004552.17	7.25661646	0.07244564	0.01000557	45.6458986	1.42E-11	2.55E-10	Ctse
ENSMUSG00000004558.16	20854.3146	-0.0054396	0.00244806	4.89916056	0.02686975	0.07269443	Ndrg2
ENSMUSG00000004561.15	190.007006	-0.0118548	0.00303889	15.3964936	8.71E-05	0.00050455	Mettl17
ENSMUSG00000004562.17	178.675422	-0.0073281	0.00406738	3.3293701	0.06805293	0.15478269	Arhgef40
ENSMUSG00000004565.16	441.621537	-0.006586	0.00402626	2.64863128	0.1036391	0.21583234	Pnpla6
ENSMUSG00000004567.17	577.550015	0.00058032	0.0031685	0.03386531	0.85399362	0.94975475	Mcoln1
ENSMUSG00000004568.14	432.421255	-0.0047569	0.00280795	2.85415836	0.09113793	0.19541629	Arhgef18
ENSMUSG00000004591.17	1068.59766	-0.0035242	0.00382597	0.82499234	0.36372456	0.55332818	Pkn2
ENSMUSG00000004609.12	53.4545453	0.05393005	0.00676752	56.8175107	4.78E-14	1.19E-12	Cd33
ENSMUSG00000004610.5	5137.50097	-0.0158619	0.00363639	19.187208	1.19E-05	8.27E-05	Etfb
ENSMUSG00000004612.10	25.8866177	0.09365247	0.00794933	119.798708	7.00E-28	1.03E-25	Nkg7
ENSMUSG00000004626.15	683.644117	-0.0045068	0.00460975	0.94267754	0.3315902	0.51862642	Stxbp2
ENSMUSG00000004630.19	0.73925939	-0.0051582	0.02716954	-0.0345265	1	1	Pcp2
ENSMUSG00000004631.16	119.019437	0.00975993	0.00332472	8.39591174	0.00376066	0.01385487	Sgce
ENSMUSG00000004633.18	697.072515	-0.0001811	0.00228577	0.00634884	0.93649209	0.99509404	Chn2
ENSMUSG00000004637.16	128.425006	-0.0060924	0.00316827	3.72418452	0.05362981	0.12773081	Wwox
ENSMUSG00000004642.14	277.582768	0.00145694	0.00221598	0.43514261	0.50947628	0.69608454	Slbp
ENSMUSG00000004651.7	1.54090674	0.00944884	0.02356713	0.27550351	0.59966363	0.77385055	Tyr
ENSMUSG00000004655.6	1845.25833	-0.0061459	0.00301607	4.15764912	0.04144689	0.10356673	Aqp1
ENSMUSG00000004661.16	86.6772085	0.00706901	0.00509718	1.84658215	0.17418143	0.32234157	Arid3b
ENSMUSG00000004665.11	163.827689	0.03076319	0.00499222	36.7606003	1.34E-09	1.82E-08	Cnn2
ENSMUSG00000004667.19	560.889918	-0.0074218	0.00346474	4.6035411	0.03190599	0.08370714	Polr2e
ENSMUSG00000004668.15	10.9629056	0.05798946	0.01806444	9.93706233	0.00161984	0.00670861	Abca13
ENSMUSG00000004677.19	512.484775	0.01637789	0.00270034	36.6191537	1.44E-09	1.94E-08	Myo9b
ENSMUSG00000004698.12	49.7333928	0.04427194	0.0064607	43.077304	5.26E-11	8.78E-10	Hdac9
ENSMUSG00000004707.15	62.1151934	0.0791328	0.00599976	159.759936	1.28E-36	5.79E-34	Ly9
ENSMUSG00000004709.15	11.310854	0.07789165	0.00799106	88.6418805	4.73E-21	2.75E-19	Cd244a
ENSMUSG00000004730.16	578.142948	0.04810213	0.00598889	58.4166591	2.12E-14	5.57E-13	Adgre1
ENSMUSG00000004748.6	497.874231	-0.0142038	0.00350375	16.6513149	4.49E-05	0.00027903	Mtfp1
ENSMUSG00000004768.15	166.182447	0.01613448	0.00380162	18.0064314	2.20E-05	0.00014586	Rab23
ENSMUSG00000004771.13	1175.92649	-0.0029951	0.00223265	1.80896014	0.17863302	0.32844079	Rab11a
ENSMUSG00000004788.12	277.502417	-0.0093511	0.0037763	6.137675	0.01323312	0.04030949	Eif2b2
ENSMUSG00000004789.11	3107.05256	-0.0114612	0.0018064	40.3340778	2.14E-10	3.27E-09	Dlst
ENSMUSG00000004791.8	2.62432114	0.0090297	0.01536136	0.3918146	0.53134711	0.71601171	Pgf
ENSMUSG00000004798.15	1394.83775	-0.0228457	0.0027391	68.4021574	1.33E-16	4.53E-15	Ulk2
ENSMUSG00000004814.11	55.3674685	0.02682587	0.00856751	8.90450747	0.00284468	0.01092033	Ccl24
ENSMUSG00000004815.13	418.969059	-0.0080593	0.0036545	4.90451359	0.02678659	0.07251103	Dgkq
ENSMUSG00000004837.3	83.3288219	0.02554804	0.00458529	30.2559768	3.79E-08	4.18E-07	Grap
ENSMUSG00000004843.8	596.862383	0.0100445	0.00268508	13.7509015	0.00020872	0.00110801	Chmp2b
ENSMUSG00000004846.11	409.860405	-0.0032206	0.00286878	1.24599085	0.26431959	0.43937147	Plod3
ENSMUSG00000004849.15	438.009518	0.00120468	0.00366166	0.10905585	0.74122161	0.87741294	Ap1s1
ENSMUSG00000004864.14	5.90388749	0.02174621	0.00915967	5.74600088	0.01652624	0.04860803	Mapk13
ENSMUSG00000004865.17	564.26511	-0.0024994	0.00293459	0.71665648	0.39724284	0.58904625	Srpk1
ENSMUSG00000004872.16	1.70302643	-0.0229996	0.03354112	0.4247567	0.51457304	0.70120248	Pax3
ENSMUSG00000004880.12	309.474969	0.01520401	0.00303941	24.389911	7.87E-07	6.87E-06	Lbr
ENSMUSG00000004891.17	114.073711	0.00440975	0.00610127	0.50952471	0.47534468	0.66580436	Nes
ENSMUSG00000004892.14	5.0103777	0.01724014	0.02308363	1.02563931	0.31118507	0.49626023	Bcan
ENSMUSG00000004895.10	333.443019	-0.0035652	0.00229441	2.40280397	0.12111798	0.24403629	Prcc
ENSMUSG00000004896.17	188.103135	-0.0041011	0.00387006	1.12757767	0.28829262	0.46935092	Mettl25b
ENSMUSG00000004897.18	3134.74394	-0.0041623	0.00285484	2.11012127	0.14632772	0.28222141	Hdgf
ENSMUSG00000004902.8	1.57529086	0.01489089	0.01422269	1.25251283	0.26307308	0.43779311	Slc25a18
ENSMUSG00000004929.13	140.569087	-0.0061666	0.00326143	3.50155396	0.06131127	0.14240155	Thop1
ENSMUSG00000004931.12	511.249434	-0.015644	0.00331544	21.7548135	3.10E-06	2.45E-05	Apba3
ENSMUSG00000004933.18	10.7150925	0.06197198	0.00775678	61.6852129	4.03E-15	1.15E-13	Matk
ENSMUSG00000004934.15	245.080448	-0.0043197	0.00342957	1.59368913	0.20679985	0.36756453	Pias4
ENSMUSG00000004936.9	834.912033	-0.0013671	0.00192585	0.49932806	0.47979551	0.66953361	Map2k1
ENSMUSG00000004937.7	1025.61048	-0.003062	0.00205738	2.22191178	0.13606448	0.26690093	Sgta
ENSMUSG00000004939.8	1.27048755	-0.0171756	0.04925519	0.14694111	0.70147585	0.85099318	Nmrk2
ENSMUSG00000004945.16	848.219437	-0.0122569	0.00314655	15.3392882	8.98E-05	0.00051878	Tmem242
ENSMUSG00000004947.16	84.8111088	-0.0053697	0.00330029	2.64125883	0.10412101	0.21646525	Dtx2

ENSMUSG00000004951.11	444.052448	-0.0278244	0.00592414	18.1288987	2.06E-05	0.00013778	Hspb1
ENSMUSG00000004952.14	61.1615113	0.05814996	0.00579542	93.8244098	3.45E-22	2.36E-20	Rasa4
ENSMUSG00000004961.8	4.17945526	0.00445923	0.0149726	0.09454357	0.75847851	0.88922599	Syt5
ENSMUSG00000004980.17	5695.27652	-0.0022939	0.00235468	0.94904982	0.32996128	0.51676405	Hnrnpa2b1
ENSMUSG00000004988.10	2.82290354	0.03525871	0.03143843	-0.932254	1	1	Fxyd4
ENSMUSG00000004994.13	83.9624253	0.00866899	0.00438367	3.9989504	0.04552861	0.11177659	Yju2b
ENSMUSG00000004996.10	300.266696	-0.0051232	0.00303197	2.88239537	0.08955272	0.19280606	Mri1
ENSMUSG00000005034.16	1014.53528	0.00657599	0.00221833	8.70307938	0.00317673	0.01199205	Prkacb
ENSMUSG00000005043.14	137.462247	0.01131549	0.00339404	11.0333888	0.00089485	0.00397777	Sgsh
ENSMUSG00000005045.17	24.8594036	0.02975548	0.01316124	4.85565996	0.02755557	0.07413319	Chd5
ENSMUSG00000005054.8	763.526246	0.01327355	0.00389623	11.6388689	0.00064588	0.00299699	Cstb
ENSMUSG00000005057.14	49.9721338	0.00446786	0.00569234	0.56697047	0.45146529	0.64324395	Sh2b2
ENSMUSG00000005069.13	2110.96996	-0.0017962	0.00295205	0.36823235	0.54396937	0.72741023	Pex5
ENSMUSG00000005078.17	361.16344	0.00189866	0.0018529	1.04770172	0.30603699	0.49101891	Jkamp
ENSMUSG00000005087.18	314.473645	0.0578695	0.00512639	117.610194	2.11E-27	2.81E-25	Cd44
ENSMUSG00000005089.16	2655.78639	0.0031201	0.0052781	0.33841067	0.56074805	0.74198056	Slc1a2
ENSMUSG00000005102.14	192.736245	0.00757204	0.00347009	4.72886149	0.02966051	0.07873639	Eif2ak4
ENSMUSG00000005103.13	1008.90106	0.01420437	0.00237641	35.1420718	3.07E-09	4.00E-08	Wdr1
ENSMUSG00000005107.14	634.748866	0.00260495	0.00352739	0.54423462	0.46068304	0.65196629	Slc2a9
ENSMUSG00000005124.11	47.5199051	-0.0060489	0.00676124	0.76526768	0.38168503	0.57318369	Ccn4
ENSMUSG00000005125.14	428.51859	-0.0015662	0.00571077	0.07554553	0.78342728	0.90573579	Ndrg1
ENSMUSG00000005131.5	5.78621817	0.02735914	0.0102263	6.5583223	0.01043947	0.03305927	4930550C14Rik
ENSMUSG00000005142.11	1866.78666	0.01023887	0.00282039	13.202034	0.00027965	0.00143479	Man2b1
ENSMUSG00000005148.9	14.4979491	0.01931471	0.009716	3.88713767	0.04865728	0.11798927	Klf5
ENSMUSG00000005150.17	246.993128	-0.0099808	0.00354009	8.03842944	0.00457952	0.01639287	Wdr83
ENSMUSG00000005161.16	2800.61158	-0.0106359	0.00425851	6.17921922	0.01292593	0.03951665	Prdx2
ENSMUSG00000005198.16	952.133396	0.00483395	0.00321558	2.25759546	0.13296037	0.26244901	Polr2a
ENSMUSG00000005204.13	552.363896	0.00187881	0.0035321	0.27411526	0.60058448	0.77449687	Senp3
ENSMUSG00000005220.11	54.7438253	0.06876103	0.0125531	25.440876	4.56E-07	4.19E-06	Corin
ENSMUSG00000005225.16	587.340909	-0.0068102	0.00231072	8.64005318	0.00328852	0.01235867	Plekha8
ENSMUSG00000005233.17	107.093953	-0.0289189	0.00524549	29.6813187	5.09E-08	5.52E-07	Spc25
ENSMUSG00000005237.15	8.66351975	0.05903374	0.01168498	20.5576042	5.79E-06	4.32E-05	Dnah2
ENSMUSG00000005251.16	112.962426	-0.0019718	0.00333056	0.35685643	0.5502573	0.73286852	Ripk4
ENSMUSG00000005262.14	603.087885	-0.0040173	0.0023063	3.06579162	0.07995621	0.17604996	Ufd1
ENSMUSG00000005267.14	12.8032991	0.03118615	0.00610024	25.2674833	4.99E-07	4.54E-06	Zfp287
ENSMUSG00000005268.21	2819.40645	0.03099883	0.00466967	43.6857238	3.86E-11	6.54E-10	Prlr
ENSMUSG00000005299.7	1324.54334	-0.0224584	0.00258233	75.8013464	3.14E-18	1.30E-16	Letm1
ENSMUSG00000005312.16	2550.72224	-0.0048061	0.00215051	4.87856686	0.02719219	0.07333961	Ubqln1
ENSMUSG00000005320.10	639.079681	0.01246466	0.00390135	10.0114247	0.00155572	0.00647375	Fgfr4
ENSMUSG00000005338.15	192.583666	0.00451081	0.00467064	0.92405742	0.33641188	0.52380582	Cadm3
ENSMUSG00000005339.11	0.64495934	0.07435924	0.02918046	5.38229224	0.02034213	0.05779205	Fcer1a
ENSMUSG00000005354.17	2797.82032	-0.0087266	0.00321983	7.46305812	0.00629779	0.02153999	Txn2
ENSMUSG00000005360.15	40.2278585	0.06417888	0.00923518	42.2162156	8.17E-11	1.33E-09	Slc1a3
ENSMUSG00000005362.15	656.513672	0.00358985	0.00362514	0.95602854	0.32818952	0.51522084	Crbn
ENSMUSG00000005364.12	3.32629129	0.10784742	0.01347163	53.3132073	2.84E-13	6.44E-12	Il5ra
ENSMUSG00000005370.5	266.232769	0.00311188	0.00234182	1.71474333	0.1903711	0.3448612	Msh6
ENSMUSG00000005371.16	516.641794	0.00622252	0.00241075	6.5509175	0.01048301	0.03317931	Fbxo11
ENSMUSG00000005373.14	4932.38137	-0.0238742	0.00534361	19.3765784	1.07E-05	7.56E-05	Mlxipl
ENSMUSG00000005374.14	331.591472	-0.0037611	0.00275791	1.82797703	0.17636668	0.32531355	Tbl2
ENSMUSG00000005378.18	360.739176	-0.0123651	0.00277663	19.685478	9.13E-06	6.53E-05	Bud23
ENSMUSG00000005397.9	454.561989	0.02463262	0.00520122	21.8201548	2.99E-06	2.37E-05	Nid1
ENSMUSG00000005410.7	75.2015506	0.03208611	0.00754716	17.4304232	2.98E-05	0.00019163	Mcm5
ENSMUSG00000005413.9	642.774579	0.04737051	0.00616284	53.5280157	2.55E-13	5.81E-12	Hmox1
ENSMUSG00000005417.18	1827.87015	-0.0082762	0.00211324	15.4263135	8.58E-05	0.00049775	Mprip
ENSMUSG00000005442.14	759.646724	0.00802247	0.00447033	3.13115202	0.07680945	0.17068283	Cic
ENSMUSG00000005447.13	54.0842628	0.019481	0.0043229	20.3576693	6.42E-06	4.75E-05	Pafah1b3
ENSMUSG00000005465.10	26.9336451	0.0343396	0.00488665	47.2738355	6.17E-12	1.18E-10	Il27ra
ENSMUSG00000005469.11	1020.83327	-0.0032036	0.00222932	2.06464736	0.1507498	0.28850898	Prkaca
ENSMUSG00000005470.9	18.0535515	0.0234235	0.00843996	7.20127454	0.00728518	0.02443867	Asf1b
ENSMUSG00000005481.19	263.120927	-0.0008374	0.00281769	0.08900287	0.76544865	0.8936894	Ddx39a
ENSMUSG00000005483.11	470.747509	-0.0096425	0.00381873	6.25326804	0.01239644	0.03816505	Dnajb1

ENSMUSG00000005501.15	667.871715	-0.0023128	0.00281062	0.66954085	0.41321173	0.60470385	Usp40
ENSMUSG00000005505.13	259.13906	-0.0102974	0.00218063	22.2770063	2.36E-06	1.90E-05	Kbtbd4
ENSMUSG00000005506.17	1940.27726	-0.0060261	0.00253229	5.63270802	0.01762838	0.05128719	Celf1
ENSMUSG00000005510.10	1807.53054	-0.0141687	0.00310067	21.2175793	4.10E-06	3.16E-05	Ndufs3
ENSMUSG00000005514.15	6125.81822	0.00541104	0.00463437	1.36333519	0.2429603	0.4132013	Por
ENSMUSG00000005533.11	108.240538	0.02461903	0.00486556	24.2431359	8.49E-07	7.38E-06	Igf1r
ENSMUSG00000005534.11	2428.07326	-0.0008965	0.00322971	0.07648122	0.78212384	0.9048498	Insr
ENSMUSG00000005540.11	10.4926018	0.02297431	0.00819475	6.88026292	0.00871527	0.0284156	Fcer2a
ENSMUSG00000005547.15	11663.6052	0.01939546	0.00757995	6.31585359	0.01196631	0.0370439	Cyp2a5
ENSMUSG00000005553.15	22.4888177	-0.0228914	0.00893194	5.72475987	0.01672732	0.04910763	Atp4a
ENSMUSG00000005566.15	914.467498	-0.0015651	0.00268375	0.34270183	0.55827381	0.74003519	Trim28
ENSMUSG00000005575.17	715.159895	0.00150899	0.00299563	0.25674728	0.61236368	0.78440285	Ube2m
ENSMUSG00000005580.12	316.636627	-0.0055713	0.00464017	1.44396907	0.22949814	0.39661072	Adcy9
ENSMUSG00000005583.17	413.525212	0.03471003	0.00559596	34.9160037	3.44E-09	4.46E-08	Mef2c
ENSMUSG00000005609.17	815.544504	-0.0057488	0.00290135	3.91007227	0.04799753	0.1166962	Ctr9
ENSMUSG00000005610.18	10471.5225	0.00106864	0.00229521	0.21221202	0.64503914	0.81019774	Eif4g2
ENSMUSG00000005611.16	44.5893031	0.0153722	0.00529915	7.88741739	0.00497799	0.01761339	Irag1
ENSMUSG00000005615.16	1628.33457	-0.0004	0.00354061	0.01262148	0.91054958	0.9810526	Pcyt1a
ENSMUSG00000005621.12	540.452026	-0.0038034	0.0032187	1.36334181	0.24295915	0.4132013	Zfp592
ENSMUSG00000005625.16	1809.43182	-0.0073705	0.00366332	3.97028778	0.04630986	0.11335246	Psmd4
ENSMUSG00000005628.13	7.93510834	-0.0116575	0.00945885	1.43806659	0.23045246	0.39792189	Tmod4
ENSMUSG00000005640.13	1.66494146	0.08647313	0.01547088	31.0257573	2.55E-08	2.89E-07	Insrr
ENSMUSG00000005656.10	382.751322	0.01550697	0.00308554	24.8943795	6.06E-07	5.43E-06	Snx6
ENSMUSG00000005667.9	43.8440665	0.00183489	0.00639761	0.07836679	0.77952301	0.90327065	Mthfd2
ENSMUSG00000005672.13	106.446497	-0.005262	0.00431333	1.4814127	0.22355362	0.38894957	Kit
ENSMUSG00000005674.10	457.847828	-0.0168297	0.00478795	12.0844839	0.00050843	0.00243046	Tomm40l
ENSMUSG00000005677.15	3168.48631	-0.0007723	0.0056032	0.01923252	0.88970194	0.96970934	Nr1i3
ENSMUSG00000005681.13	99532.8988	-0.0081146	0.00642695	1.57794487	0.20905697	0.37059884	Apoa2
ENSMUSG00000005682.10	470.227357	-0.0051574	0.00290162	3.17277104	0.07487531	0.16714074	Pan2
ENSMUSG00000005683.9	2659.67227	-0.0104145	0.0028256	13.6177366	0.00022406	0.00117935	Cs
ENSMUSG00000005686.18	43.054287	0.03136012	0.00661454	21.409429	3.71E-06	2.89E-05	Ampd3
ENSMUSG00000005687.15	584.085939	0.00117011	0.00260259	0.20354059	0.65187804	0.81405974	Bcas2
ENSMUSG00000005696.12	5.52466932	0.07642056	0.01160232	37.1819322	1.08E-09	1.48E-08	Sh2d1a
ENSMUSG00000005698.16	689.286425	0.00015631	0.00299284	0.00262006	0.9591769	1	Ctcf
ENSMUSG00000005699.17	58.2991481	-0.005445	0.00427241	1.56095371	0.21152563	0.37373913	Pard6a
ENSMUSG00000005716.17	9.46099743	0.02051534	0.02552155	0.96426602	0.32611439	0.51286436	Pvalb
ENSMUSG00000005718.9	67.3770582	0.01155744	0.00379607	9.12087984	0.00252708	0.00986655	Tfap4
ENSMUSG00000005732.15	504.235487	0.00620964	0.00313969	3.86855371	0.0491989	0.11899152	Ranbp1
ENSMUSG00000005763.16	11.9589364	0.08190021	0.00834184	81.5499082	1.71E-19	8.40E-18	Cd247
ENSMUSG00000005774.13	200.14523	0.01710797	0.00317459	28.6392874	8.72E-08	9.15E-07	Rfx5
ENSMUSG00000005779.13	2297.40297	-0.0028264	0.00390381	0.53301778	0.46534088	0.65635271	Psmb4
ENSMUSG00000005800.4	8.30570704	0.08490771	0.01400866	23.5502301	1.22E-06	1.03E-05	Mmp8
ENSMUSG00000005802.13	285.768179	-0.0028212	0.00266421	1.12485739	0.28887493	0.47003203	Slc30a4
ENSMUSG00000005803.15	2762.67268	0.00649032	0.00262926	6.18031223	0.01291795	0.03950249	Sqor
ENSMUSG00000005804.15	282.684729	-5.79E-05	0.00234001	0.00061227	0.98025908	1	Bloc1s6
ENSMUSG00000005813.13	656.412715	-0.0031985	0.00168782	3.58472903	0.05831293	0.13678455	Metap1
ENSMUSG00000005823.10	392.109102	-0.0079954	0.00228018	12.3634146	0.00043783	0.00212351	Gpr108
ENSMUSG00000005824.8	5.54120938	0.03002022	0.00815594	12.4722401	0.00041304	0.0020195	Tnfsf14
ENSMUSG00000005836.11	164.308646	0.00724591	0.00383251	3.46812923	0.06256203	0.14472224	Gata6
ENSMUSG00000005846.13	853.308899	-0.0022201	0.0023211	0.9126262	0.33941857	0.52706079	Rsl1d1
ENSMUSG00000005871.16	821.291344	0.00517402	0.00369546	1.9244393	0.1653681	0.30938145	Apc
ENSMUSG00000005873.7	538.298314	-0.0105507	0.00529589	3.88706319	0.04865944	0.11798927	Reep5
ENSMUSG00000005881.14	1266.74808	-0.0077908	0.00312543	6.19968466	0.01277731	0.03914347	Ergic3
ENSMUSG00000005882.19	814.557511	-0.0201115	0.00444187	20.1136961	7.30E-06	5.33E-05	Uqcc1
ENSMUSG00000005883.16	2.2267644	0.0473308	0.01226623	13.2718944	0.00026941	0.00138803	Spo11
ENSMUSG00000005886.15	873.377198	-0.0021636	0.00378175	0.31965192	0.5718169	0.75110805	Ncoa2
ENSMUSG00000005893.15	502.364971	0.00250779	0.00354219	0.48856816	0.48456671	0.67364084	Nr2c2
ENSMUSG00000005897.15	284.467102	0.00353502	0.00239733	2.0954948	0.14773388	0.28417818	Nr2c1
ENSMUSG00000005899.15	265.994162	-0.0079485	0.00375793	4.47047053	0.03448549	0.08925435	Smpd4
ENSMUSG00000005907.15	561.151834	-0.009816	0.00357324	7.54921379	0.00600363	0.02066874	Pex1
ENSMUSG00000005917.16	1.43890934	0.02627152	0.03778902	0.73151474	0.39239264	0.58361539	Otx1

ENSMUSG00000005936.20	659.398287	0.00175915	0.00169928	1.07577032	0.29964569	0.48366232	Kctd20
ENSMUSG00000005947.12	24.4751856	0.02494132	0.00638057	14.4642378	0.00014285	0.00078737	Itgae
ENSMUSG00000005949.10	282.050095	0.00189756	0.00266877	0.50915095	0.47550664	0.66587303	Ctns
ENSMUSG00000005950.15	5.07230407	0.01469942	0.01069097	1.73981349	0.18716246	0.34040822	P2rx5
ENSMUSG00000005951.14	884.850696	0.00029836	0.00335395	0.00801022	0.92868469	0.99094992	Shpk
ENSMUSG00000005952.16	4.2108339	-0.0414245	0.01296082	9.63752492	0.00190642	0.00774988	Trpv1
ENSMUSG00000005958.16	12.3075496	0.00788679	0.0079905	0.96268271	0.3265119	0.51333886	Ephb3
ENSMUSG00000005968.15	107.821054	-0.0202356	0.00422034	23.1146427	1.53E-06	1.28E-05	Tuft1
ENSMUSG00000005973.7	190.996274	0.00710782	0.00325218	4.71818859	0.02984517	0.07913751	Rcn1
ENSMUSG00000005980.16	28.1178728	0.03264134	0.00836296	13.6752852	0.0002173	0.00114862	Dnase1
ENSMUSG00000005981.12	2733.17719	-0.009746	0.00214146	20.8909575	4.86E-06	3.69E-05	Trap1
ENSMUSG00000005982.15	1480.87438	-0.010943	0.00252121	18.8632654	1.40E-05	9.69E-05	Naa60
ENSMUSG00000005983.16	22.8255221	0.00846762	0.00696025	1.43630065	0.23073891	0.39831721	1700037C18Rik
ENSMUSG00000005986.17	8.91038395	0.04044979	0.00803566	23.9213423	1.00E-06	8.63E-06	Ankrd13d
ENSMUSG00000006005.19	1969.64557	0.0027129	0.0035471	0.58000476	0.44631046	0.63834791	Tpr
ENSMUSG00000006010.15	719.924174	-0.0041184	0.00303539	1.81782508	0.17757238	0.32705155	Odr4
ENSMUSG00000006014.18	818.411569	0.01563086	0.00526094	8.6106359	0.00334206	0.01254188	Prg4
ENSMUSG00000006019.16	126.442197	0.00034914	0.00293087	0.01401904	0.90574913	0.97869704	Dhx34
ENSMUSG00000006021.6	224.159952	-0.0063885	0.00271691	5.57758592	0.01819178	0.05266618	Kptn
ENSMUSG00000006024.14	1185.63251	0.00412355	0.00242337	2.90221903	0.0884577	0.19090158	Napa
ENSMUSG00000006050.13	943.138739	-0.015377	0.00258891	35.5184083	2.53E-09	3.32E-08	Sra1
ENSMUSG00000006057.16	2879.30668	-0.024052	0.00378717	41.3011477	1.30E-10	2.06E-09	Atp5mc1
ENSMUSG00000006058.11	600.187982	-0.0047024	0.00365569	1.64867993	0.1991387	0.35704613	Snf8
ENSMUSG00000006095.13	402.398116	-0.000478	0.00424446	0.01249459	0.91099849	0.98143545	Tbcb
ENSMUSG00000006127.10	260.547838	0.00359147	0.00252729	2.02818135	0.15440528	0.29374116	Inpp5k
ENSMUSG00000006134.5	677.227363	0.00255378	0.00270318	0.89110167	0.34517894	0.53358176	Crkl
ENSMUSG00000006154.14	49.1929528	0.00765827	0.0081288	0.76309282	0.38236236	0.57405466	Eps8l1
ENSMUSG00000006169.21	1276.41779	0.00752657	0.00235466	9.99968231	0.00156567	0.00651057	Clint1
ENSMUSG00000006179.10	3.75514059	0.06741601	0.01799888	12.0587003	0.00051551	0.00245932	Prss16
ENSMUSG00000006191.18	177.782871	0.00029477	0.00303344	0.00922278	0.92349252	0.98831592	Cdkal1
ENSMUSG00000006205.14	74.8759374	0.01987914	0.00679452	8.30778346	0.00394755	0.01445088	Htra1
ENSMUSG00000006215.13	174.869123	-0.0078349	0.00261225	8.91753822	0.00282445	0.01086036	Zbtb17
ENSMUSG00000006216.11	4.0221273	0.00179194	0.03460601	0.02640984	0.87090344	0.95915167	Clcnkb
ENSMUSG00000006218.5	38.1368831	-0.0071928	0.00532664	1.68616119	0.19410778	0.34973588	Fam131c
ENSMUSG00000006219.13	65.450267	0.03926233	0.00751742	24.1184378	9.06E-07	7.84E-06	Fblim1
ENSMUSG00000006221.8	177.194617	-0.0074345	0.01439644	0.01618005	0.89878145	0.97509933	Hspb7
ENSMUSG00000006235.7	19.0861345	-0.0071087	0.00564123	1.54869405	0.21332834	0.3759386	Epor
ENSMUSG00000006241.17	69.4693536	-0.0110369	0.00292722	14.4068483	0.00014727	0.00080984	Ccdc159
ENSMUSG00000006262.16	1009.55996	-0.0104048	0.00246472	17.5229378	2.84E-05	0.00018363	Mob1b
ENSMUSG00000006269.8	1.98812625	0.01062631	0.02782501	0.25035754	0.61682343	0.78814657	Atp6v1b1
ENSMUSG00000006273.15	896.070865	0.01306197	0.00256248	25.462947	4.51E-07	4.15E-06	Atp6v1b2
ENSMUSG00000006276.11	279.906637	0.00151866	0.00231123	0.43691326	0.50861607	0.69539265	Eps15l1
ENSMUSG00000006281.9	400.156673	0.02314662	0.00535104	17.815048	2.43E-05	0.00015959	Tep1
ENSMUSG00000006288.9	172.844893	0.00525978	0.00246267	4.53876044	0.03313558	0.08627175	Ttc5
ENSMUSG00000006289.15	358.393386	-0.0043975	0.00319138	1.89758114	0.16834932	0.31373949	Osgep
ENSMUSG00000006299.14	2228.31926	-0.0066092	0.00260918	6.4830726	0.01089066	0.03429466	Aamp
ENSMUSG00000006301.18	880.987814	-0.0058832	0.00281835	4.32404251	0.03757755	0.09554809	Tmbim1
ENSMUSG00000006304.15	2103.57263	0.00478211	0.00272947	3.04056035	0.08120788	0.17828996	Arpc2
ENSMUSG00000006307.17	473.255019	0.00215296	0.00406846	0.27922861	0.59720726	0.77194774	Kmt2b
ENSMUSG00000006310.11	8.68427694	0.04375989	0.00910344	18.9138451	1.37E-05	9.46E-05	Zbtb32
ENSMUSG00000006315.10	1332.67069	-0.0179317	0.00277597	42.245925	8.05E-11	1.31E-09	Tmem147
ENSMUSG00000006333.15	7720.75484	-0.0012598	0.00487412	0.06652061	0.79647182	0.91400692	Rps9
ENSMUSG00000006335.17	136.840162	-0.0072999	0.00287062	6.44046459	0.01115495	0.03497283	Tfpt
ENSMUSG00000006342.16	56.4621676	0.03346366	0.00474908	48.9347865	2.65E-12	5.28E-11	Susd2
ENSMUSG00000006344.18	65.1630475	0.03547399	0.0050942	45.6563432	1.41E-11	2.54E-10	Ggt5
ENSMUSG00000006345.11	24.2373608	0.02141236	0.01338803	2.41288132	0.12034069	0.24276047	Ggt1
ENSMUSG00000006356.11	913.486879	-0.0040467	0.00403813	0.99158442	0.31935544	0.5051856	Crip2
ENSMUSG00000006360.12	123.832271	0.04271013	0.00685771	36.674075	1.40E-09	1.89E-08	Crip1
ENSMUSG00000006362.17	76.4261787	0.00326168	0.00500956	0.41317744	0.52036115	0.70612958	Cbfa2t3
ENSMUSG00000006369.15	34.7620814	-0.0148908	0.00711437	4.2231996	0.0398749	0.10035099	Fbln1
ENSMUSG00000006373.11	23074.415	-0.0110864	0.00384636	8.13626505	0.00433886	0.01568132	Pgrmc1

ENSMUSG00000006378.16	1410.20929	-0.0139962	0.00392729	12.7433007	0.00035729	0.00177822	Gcat
ENSMUSG00000006386.16	240.5681	-0.0101308	0.00383255	6.95528926	0.00835718	0.02739956	Tek
ENSMUSG00000006389.14	2.07492213	0.08103769	0.01446582	31.0896291	2.46E-08	2.81E-07	Mpl
ENSMUSG00000006390.16	668.009325	-0.0033637	0.0024174	1.93837015	0.16384561	0.30704454	Elovl1
ENSMUSG00000006392.17	596.345086	-0.0071388	0.00210364	11.5270915	0.00068589	0.00315658	Med8
ENSMUSG00000006395.18	811.855716	-0.0036614	0.00447532	0.6682861	0.41364979	0.60504459	Hyi
ENSMUSG00000006398.16	29.4553749	0.01060203	0.01032715	0.93309109	0.33406099	0.52100526	Cdc20
ENSMUSG00000006403.14	11.2291115	0.02821835	0.00832638	11.0565735	0.00088373	0.00393575	Adamts4
ENSMUSG00000006411.13	9.32207734	0.05975761	0.00705893	68.6242043	1.19E-16	4.08E-15	Nectin4
ENSMUSG00000006412.11	1015.97102	-0.0087568	0.00338624	6.68736541	0.00970986	0.03113489	Pfdn2
ENSMUSG00000006418.18	1210.13648	-0.0001065	0.0016962	0.00390502	0.95017249	1	Rnf114
ENSMUSG00000006423.16	728.20091	-0.00306	0.00233546	1.69555733	0.19287001	0.34818462	Steep1
ENSMUSG00000006435.17	11.9948898	0.02670328	0.00878755	8.46324382	0.00362395	0.01342672	Neurl1a
ENSMUSG00000006442.11	361.169224	-0.0030693	0.00411957	0.55879266	0.45474711	0.64647491	Srm
ENSMUSG00000006445.4	369.534011	-0.0041575	0.00448746	0.78878825	0.37446678	0.56541132	Epha2
ENSMUSG00000006456.11	279.684145	-0.008784	0.00301355	8.50391961	0.00354382	0.01317746	Rbm14
ENSMUSG00000006457.5	1.76631789	0.00723508	0.015184	0.28754849	0.59179582	0.76762188	Actn3
ENSMUSG00000006463.14	70.0471185	0.00686031	0.00285982	5.73372276	0.01664217	0.04890023	Zdhhc24
ENSMUSG00000006464.10	26.7869584	0.0195529	0.00556143	12.4045798	0.00042828	0.00208407	Bbs1
ENSMUSG00000006471.19	206.273203	-0.0059383	0.00298267	3.97534763	0.04617092	0.11310629	Ndor1
ENSMUSG00000006476.20	1704.19679	-0.0161993	0.00335977	23.1147546	1.53E-06	1.28E-05	Nsmf
ENSMUSG00000006494.12	2079.99417	0.01026422	0.00406858	6.23840931	0.01250087	0.03841135	Pdk1
ENSMUSG00000006498.17	1600.99651	0.00322584	0.00257046	1.55112247	0.21296981	0.37553125	Ptbp1
ENSMUSG00000006517.6	539.710661	0.01583341	0.00564736	7.44542939	0.00635977	0.02172677	Mvd
ENSMUSG00000006519.12	300.666158	0.05881663	0.00612893	81.4493006	1.80E-19	8.76E-18	Cyba
ENSMUSG00000006522.18	17914.2766	0.00653567	0.00601064	1.17320396	0.27874389	0.45773539	Itih3
ENSMUSG00000006526.14	310.549605	-0.0026237	0.00430009	0.3777851	0.538791	0.72273927	Stimate
ENSMUSG00000006527.17	564.444418	-0.0057263	0.00299764	3.64401729	0.056271	0.13306452	Sfmbt1
ENSMUSG00000006529.17	10944.4758	-0.0005863	0.00491567	0.01417863	0.90521669	0.97843549	Itih1
ENSMUSG00000006538.13	38.8692498	-0.0060308	0.00666572	0.84634167	0.35758933	0.54639167	Ihh
ENSMUSG00000006542.14	3.51293969	-0.0299976	0.0128105	5.56945768	0.01827642	0.05283987	Prkag3
ENSMUSG00000006567.12	533.332317	0.00786986	0.0037308	4.49827842	0.033929	0.08805937	Atp7b
ENSMUSG00000006574.16	19.1947018	0.0497851	0.01328148	8.51370741	0.00352481	0.01311711	Slc4a1
ENSMUSG00000006575.15	46.1022467	-0.0018495	0.00576487	0.10297447	0.74828894	0.88197206	Rundc3a
ENSMUSG00000006576.17	60.0911973	0.0150339	0.00945308	2.37065011	0.12363555	0.24799516	Slc4a3
ENSMUSG00000006585.4	55.1616213	0.0167741	0.00622444	7.12424811	0.00760483	0.0253058	Cdt1
ENSMUSG00000006586.16	20.0241593	0.02552646	0.00773758	10.3868021	0.00126919	0.00542009	Runx1t1
ENSMUSG00000006587.7	4.42346584	0.02961761	0.01178603	6.48582915	0.01087378	0.03425982	Snai3
ENSMUSG00000006589.9	464.697552	0.00834509	0.0038786	4.77169458	0.02893134	0.07712189	Aprt
ENSMUSG00000006599.14	570.858015	-0.0041668	0.00246779	2.79933786	0.09430324	0.20087025	Gtf2h1
ENSMUSG00000006611.16	963.960883	0.00117987	0.00301915	0.15114317	0.69744529	0.84824135	Hfe
ENSMUSG00000006638.17	49.5630712	-0.0096548	0.00378788	6.51233369	0.01071288	0.03384783	Abhd1
ENSMUSG00000006641.13	194.008141	0.01663947	0.00519228	10.2483636	0.00136806	0.00577538	Slc5a6
ENSMUSG00000006642.4	3.18307866	0.03013355	0.00830461	13.1171875	0.0002926	0.00149248	Tcf23
ENSMUSG00000006649.18	17.2990815	-0.0017065	0.0118109	-0.005848	1	1	Nphs1
ENSMUSG00000006651.9	40.4941368	-0.0004097	0.0077255	0.00098868	0.97491601	1	Aplp1
ENSMUSG00000006673.15	824.662607	-0.0003125	0.00232498	0.01791766	0.89351575	0.97242813	Qrich1
ENSMUSG00000006675.11	8.07576035	0.01968981	0.00817423	5.55687287	0.01840827	0.05314289	P4htm
ENSMUSG00000006676.18	1103.66373	-0.0076727	0.00194285	15.6932361	7.45E-05	0.00043923	Usp19
ENSMUSG00000006678.7	92.0393181	0.01956459	0.0043087	19.6415464	9.34E-06	6.67E-05	Pola1
ENSMUSG00000006699.18	3277.44007	0.01076654	0.00221392	23.3472848	1.35E-06	1.14E-05	Cdc42
ENSMUSG00000006705.14	270.704022	-0.0050739	0.00352207	1.98428272	0.15893996	0.30007233	Pknox1
ENSMUSG00000006711.16	142.144588	0.02565199	0.00619994	16.0885099	6.04E-05	0.00036372	D130043K22Rik
ENSMUSG00000006715.12	181.45388	0.00067459	0.00492011	0.01851121	0.89177707	0.97112755	Gmnn
ENSMUSG00000006717.8	1236.30426	-0.0018773	0.00367042	0.2611678	0.609319	0.78164896	Acot13
ENSMUSG00000006720.17	4.62599875	0.02623363	0.00806672	10.3207536	0.00131543	0.00557915	Zfp184
ENSMUSG00000006728.13	728.539066	0.00054809	0.00334628	0.02708117	0.8692875	0.95831986	Cdk4
ENSMUSG00000006731.11	1431.13081	0.00468972	0.00299395	2.46697635	0.11626139	0.23618456	B4galnt1
ENSMUSG00000006732.15	198.588282	0.00250544	0.00445744	0.31462079	0.57485843	0.75375867	Mettl1
ENSMUSG00000006736.10	4515.29133	-0.0062452	0.00318142	3.77786351	0.05193493	0.12430864	Tspan31
ENSMUSG00000006740.15	2494.65016	0.00124224	0.00419871	0.08513945	0.77044975	0.89706412	Kif5b

ENSMUSG00000006763.15	95.5531803	0.00650428	0.00300668	4.74472184	0.0293883	0.07815453	Saal1
ENSMUSG00000006777.8	29.3302431	-0.0394535	0.00819378	21.9411854	2.81E-06	2.24E-05	Krt23
ENSMUSG00000006782.17	305.568472	0.024489	0.00324398	56.527086	5.54E-14	1.37E-12	Cnp
ENSMUSG00000006784.15	10.5260631	0.0005889	0.00802482	0.00507473	0.94320903	0.99876665	Odad4
ENSMUSG00000006800.15	245.58165	0.01145166	0.004761	5.87026101	0.01539877	0.04586418	Sulf2
ENSMUSG00000006818.6	3845.94333	-0.0186137	0.00284176	43.1290747	5.12E-11	8.57E-10	Sod2
ENSMUSG00000006850.9	124.134192	0.00264918	0.00340084	0.59412392	0.44082884	0.63284433	Tmco6
ENSMUSG00000006906.11	234.221454	-0.0022114	0.00329305	0.44720312	0.50366615	0.69058655	Stambp
ENSMUSG00000006920.15	318.016312	0.00732451	0.00243161	8.98833381	0.00271709	0.01050052	Ezh1
ENSMUSG00000006930.16	42.5886017	0.02444956	0.00571022	18.083968	2.11E-05	0.00014071	Hap1
ENSMUSG00000006931.16	22.6988047	0.01634094	0.00703925	4.92900919	0.02640946	0.0716371	P3h4
ENSMUSG00000006932.18	3779.91683	-0.003249	0.00196823	2.72963921	0.09850153	0.20774268	Ctnnb1
ENSMUSG00000006941.6	302.568144	-0.0007206	0.00271853	0.0701628	0.79109989	0.91082276	Eif1b
ENSMUSG00000006958.15	96.5471355	-0.008316	0.00386843	4.63535916	0.03131959	0.08239864	Chrd
ENSMUSG00000006998.17	3784.63275	-0.0095488	0.0039604	5.8050865	0.01597988	0.0472663	Psmd2
ENSMUSG00000007021.6	5.17039887	0.03050281	0.0105826	8.35069442	0.0038554	0.01415527	Syngr3
ENSMUSG00000007029.17	588.448129	0.00601546	0.00371016	2.62526063	0.10517523	0.21827216	Vars
ENSMUSG00000007030.9	7.03810689	-0.0117099	0.00642798	3.34018075	0.06760718	0.15390242	Vwa7
ENSMUSG00000007033.5	14.2756242	-0.0141875	0.01111929	1.32902137	0.24897939	0.4204502	Hspa1l
ENSMUSG00000007034.16	9.51829575	0.00376685	0.00895795	0.18610497	0.66617838	0.82535189	Slc44a4
ENSMUSG00000007035.16	16.4928316	-0.0125676	0.00775235	2.64478325	0.10389033	0.21619346	Msh5
ENSMUSG00000007036.16	618.927058	0.00094823	0.00288124	0.10972174	0.74046115	0.87714381	Abhd16a
ENSMUSG00000007038.6	735.600609	0.00782716	0.00286055	7.61846606	0.00577736	0.0200154	Neu1
ENSMUSG00000007039.13	56.1723523	8.88E-06	0.00526925	2.21E-06	0.99881328	1	Ddah2
ENSMUSG00000007041.10	366.658214	0.03033752	0.00422914	50.2673543	1.34E-12	2.76E-11	Clic1
ENSMUSG00000007050.18	198.682382	-0.0023156	0.00372768	0.38830235	0.53319311	0.71759731	Lsm2
ENSMUSG00000007080.15	42.9977985	0.01809851	0.00633162	7.8410092	0.00510741	0.01801725	Pole
ENSMUSG00000007097.15	164.266701	0.01062064	0.00741272	2.02551678	0.1546763	0.29411441	Atp1a2
ENSMUSG00000007107.7	3.52843636	0.01044605	0.0135406	0.48963024	0.48409229	0.67321939	Atp1a4
ENSMUSG00000007122.12	5.46314501	0.014447	0.00984575	2.00202595	0.15708912	0.29750313	Casq1
ENSMUSG00000007207.11	11.2433498	0.01121125	0.0070398	2.52905468	0.11176716	0.22899063	Stx1a
ENSMUSG00000007216.7	49.8873309	-0.0033584	0.00451789	0.54005876	0.46240838	0.6535453	Zfp775
ENSMUSG00000007279.15	9.39304985	-0.0160512	0.02532815	0.20832834	0.6480808	0.81215377	Scube2
ENSMUSG00000007338.11	781.25976	-0.0122368	0.00337462	12.9687695	0.00031673	0.00159863	Mrpl49
ENSMUSG00000007379.16	19.7195918	0.00402052	0.00428655	0.87189322	0.35043172	0.53884102	Dennd2c
ENSMUSG00000007411.17	666.938883	-0.0066234	0.00245748	7.16561909	0.0074314	0.02481975	Mark3
ENSMUSG00000007415.16	926.040056	-0.000285	0.00184251	0.02379716	0.87740215	0.9625626	Gatad1
ENSMUSG00000007458.15	2159.36799	0.00449823	0.00184411	5.90270446	0.01511765	0.04515767	M6pr
ENSMUSG00000007476.19	462.689868	-0.0016637	0.00240874	0.47266313	0.49176455	0.67950508	Lrrc8a
ENSMUSG00000007564.16	1132.50392	0.001574	0.00314505	0.25267418	0.61519837	0.78670943	Ppp2r1a
ENSMUSG00000007570.17	134.933711	-0.0089694	0.00350683	6.53382082	0.01058424	0.03348173	Fance
ENSMUSG00000007589.10	252.157533	0.00244581	0.00262967	0.87357738	0.34996684	0.53830167	Tinf2
ENSMUSG00000007591.16	1.8195793	0.00549052	0.01066257	0.2733446	0.60109696	0.77489467	Tssk4
ENSMUSG00000007594.11	193.44382	0.02745793	0.00523416	26.1893185	3.10E-07	2.94E-06	Hapln4
ENSMUSG00000007603.10	228.527512	-0.0038075	0.00208397	3.34066638	0.06758723	0.15387186	Dus3l
ENSMUSG00000007610.17	147.429498	-0.010905	0.00329903	11.2342747	0.000803	0.00362482	Gtpbp3
ENSMUSG00000007613.16	593.979847	0.01049092	0.00287034	13.131386	0.00029039	0.00148314	Tgfbr1
ENSMUSG00000007617.19	280.950125	-0.0044697	0.00285041	2.42678033	0.11927766	0.24111098	Homer1
ENSMUSG00000007646.14	20.4082262	-0.0182198	0.00698731	6.39262175	0.01145957	0.03575146	Rad51c
ENSMUSG00000007653.13	9.08045106	0.02427955	0.02452777	1.21557742	0.27023033	0.44702291	Gabrb2
ENSMUSG00000007655.17	148.36706	4.48E-05	0.00722576	1.12E-05	0.997327	1	Cav1
ENSMUSG00000007656.14	1394.89742	-0.0028418	0.00331535	0.7392924	0.38988764	0.58131929	Arpp19
ENSMUSG00000007659.19	758.682326	0.00303616	0.00269036	1.28106004	0.25770203	0.43120326	Bcl2l1
ENSMUSG00000007670.11	683.584667	0.00078295	0.00307844	0.06353813	0.80098873	0.91629257	Khsrp
ENSMUSG00000007682.9	7.26328757	0.03742712	0.01607495	5.62165766	0.01773986	0.05155402	Dio2
ENSMUSG00000007721.7	520.316135	-0.0082553	0.00288204	8.21473437	0.00415516	0.01509838	Ccdc124
ENSMUSG00000007739.11	1823.1477	-0.0046405	0.00218495	4.43530583	0.03520291	0.09068326	Cct4
ENSMUSG00000007777.10	409.737236	-0.0104337	0.00278167	14.2347732	0.00016136	0.00088058	Trappc2b
ENSMUSG00000007783.11	11.9575762	0.02517668	0.00728657	11.5356599	0.00068274	0.00314632	Cpt1c
ENSMUSG00000007805.5	2.32527972	0.02678377	0.01132844	5.39693791	0.02017211	0.05737121	Twist2
ENSMUSG00000007812.17	771.289301	-0.0011747	0.00232123	0.25205565	0.61563133	0.7870924	Zfp655

ENSMUSG00000007815.14	3008.49605	0.00513267	0.00198416	6.599744	0.01019934	0.03242509	Rhoa
ENSMUSG00000007817.16	788.703516	0.00135153	0.00391658	0.11707947	0.73222388	0.87157259	Zmiz1
ENSMUSG00000007827.11	161.023936	0.00182705	0.00406589	0.19939593	0.65520888	0.81696746	Ankrd26
ENSMUSG00000007833.16	1278.9419	-0.0032012	0.00453853	0.48495965	0.48618435	0.67492908	Aldh16a1
ENSMUSG00000007836.7	739.043265	0.00223376	0.00256584	0.73588586	0.39098198	0.58228837	Hnrnpa0
ENSMUSG00000007837.12	395.624625	-0.0128476	0.00370731	11.9768201	0.00053866	0.00255633	Prrg2
ENSMUSG00000007850.17	2348.04985	-0.0127775	0.00466037	7.24286021	0.00711835	0.02395397	Hnrnph1
ENSMUSG00000007867.10	43.7936357	0.00308722	0.00357624	0.75726498	0.3841858	0.57580265	Ift43
ENSMUSG00000007872.4	1229.95906	0.01154005	0.00525463	4.53300627	0.03324716	0.08651452	Id3
ENSMUSG00000007877.3	100.999346	0.0081935	0.02155745	-0.4473359	1	1	Tcap
ENSMUSG00000007880.17	834.379751	0.0027428	0.00322285	0.71143126	0.39896908	0.59064051	Arid1a
ENSMUSG00000007888.16	1.01692342	0.00996076	0.02155046	0.14482554	0.70353013	0.85209474	Crlf1
ENSMUSG00000007891.17	7312.7599	0.01194072	0.00348699	11.3974591	0.00073545	0.00335258	Ctsd
ENSMUSG00000007892.9	6508.12556	-0.0052577	0.00469117	1.26380024	0.26093298	0.43508631	Rplp1
ENSMUSG00000007907.5	3.47741401	-0.1212835	0.09841468	0.21048411	0.64638823	0.81100929	Cabs1
ENSMUSG00000007908.15	2.96710886	0.02239239	0.01163189	3.43291858	0.06390907	0.14720305	Hmgcll1
ENSMUSG00000007944.9	3.96126934	0.01352883	0.02321889	0.32223579	0.5702671	0.74988958	Ttc9b
ENSMUSG00000007950.10	87.3826106	-0.0027189	0.00370574	0.54504708	0.46034855	0.65180579	Abhd8
ENSMUSG00000007987.13	79.7195064	0.00916933	0.0047381	3.72417718	0.05363005	0.12773081	Ift22
ENSMUSG00000007989.8	20.8915154	-0.0055729	0.0080614	0.45145988	0.50164249	0.68837211	Fzd3
ENSMUSG00000008035.13	1179.27289	0.00315466	0.00423903	0.54315076	0.46112988	0.65228555	Mid1ip1
ENSMUSG00000008036.12	811.49246	-0.0038729	0.00375021	1.07544901	0.29971788	0.48372423	Ap2s1
ENSMUSG00000008090.15	524.562159	0.00340113	0.00335548	1.03868263	0.30812806	0.4930467	Fgfrl1
ENSMUSG00000008129.15	2.2774306	0.04272865	0.01306559	9.99081175	0.00157323	0.00653855	Brme1
ENSMUSG00000008136.15	372.965696	0.04578637	0.01161305	11.446864	0.00071615	0.00327664	Fhl2
ENSMUSG00000008140.19	1021.65102	-0.0013559	0.00283533	0.23104707	0.63074853	0.7991424	Emc10
ENSMUSG00000008153.13	370.045118	0.02873574	0.00673695	13.5200165	0.00023603	0.00123465	Clstn3
ENSMUSG00000008167.15	506.941068	0.00482427	0.00325877	2.2103082	0.13709128	0.26849082	Fbxw9
ENSMUSG00000008193.14	68.185231	0.07264876	0.00794092	70.4131896	4.81E-17	1.75E-15	Spib
ENSMUSG00000008200.16	505.138017	0.00536926	0.00364225	2.17963584	0.13984755	0.2726663	Fnbp4
ENSMUSG00000008206.17	120.267677	0.00888108	0.00417688	4.33968779	0.03723375	0.09478632	Cers4
ENSMUSG00000008226.15	1083.09756	-0.0006495	0.00229709	0.07834429	0.77955385	0.90327065	Scrn3
ENSMUSG00000008301.12	331.868434	-0.0017551	0.0022386	0.61001512	0.43478212	0.62647511	Phax
ENSMUSG00000008305.20	839.48378	-0.0130517	0.00304793	18.4478398	1.75E-05	0.0001182	Tle1
ENSMUSG00000008307.12	35.137938	0.00167402	0.00806147	0.04253012	0.83661257	0.93842889	1700109H08Rik
ENSMUSG00000008318.11	15.7659451	0.06287399	0.00896666	41.8918808	9.65E-11	1.55E-09	Relt
ENSMUSG00000008333.13	342.02678	0.00120878	0.00281386	0.18099969	0.67051552	0.82877326	Snrpb2
ENSMUSG00000008348.10	8755.84612	-0.0040281	0.00345542	1.38127311	0.23988441	0.40951894	Ubc
ENSMUSG00000008373.17	244.362216	-0.0054305	0.00285217	3.61816398	0.05715191	0.13459661	Prpf31
ENSMUSG00000008384.9	113.26733	0.01331515	0.00444218	8.78801157	0.00303217	0.01152937	Sertad1
ENSMUSG00000008393.10	1528.26006	-0.0040127	0.00174821	5.2956434	0.02137883	0.06014326	Carhsp1
ENSMUSG00000008398.17	212.13637	0.00513264	0.00335009	2.3049148	0.12896535	0.25619277	Elk3
ENSMUSG00000008429.9	638.088797	0.00262039	0.00206934	1.57004996	0.21019976	0.37203748	Herpud2
ENSMUSG00000008435.16	177.166468	-0.0103713	0.00348778	8.9739192	0.00273861	0.01057164	Rdh13
ENSMUSG00000008450.9	343.079991	-0.0018173	0.00254145	0.50872085	0.47569311	0.66605508	Nutf2
ENSMUSG00000008461.7	4.34769575	0.00913132	0.01003526	0.71376572	0.39819651	0.59001107	Fut1
ENSMUSG00000008475.14	1760.50591	0.00891438	0.00227461	15.001626	0.00010742	0.00060947	Arpc5
ENSMUSG00000008489.19	10.7626939	0.03386853	0.01525882	4.78013791	0.02878981	0.076848	Elavl2
ENSMUSG00000008496.20	106.575046	0.06499567	0.00650373	89.3329576	3.34E-21	1.98E-19	Pou2f2
ENSMUSG00000008540.12	62867.6499	-0.0184814	0.00401675	20.6854075	5.41E-06	4.07E-05	Mgst1
ENSMUSG00000008575.18	2316.18404	-0.0005342	0.00319587	0.02740135	0.86852406	0.95800441	Nfib
ENSMUSG00000008601.13	4.70719749	0.00421977	0.00852056	0.27445526	0.60035869	0.77436618	Rab25
ENSMUSG00000008604.12	613.55688	-0.0076328	0.0022633	11.3862706	0.00073989	0.00337088	Ubqln4
ENSMUSG00000008658.17	25.4045226	0.0201287	0.01331097	2.64177706	0.10408706	0.21645168	Rbfox1
ENSMUSG00000008668.16	8660.55602	-0.0018842	0.00457504	0.17193028	0.67840198	0.83393907	Rps18
ENSMUSG00000008682.14	10713.7637	-0.0041467	0.00274191	2.27502215	0.13147325	0.26016679	Rpl10
ENSMUSG00000008683.17	4625.71349	-0.0066873	0.00461179	2.07156698	0.15006727	0.28757609	Rps15a
ENSMUSG00000008690.16	931.41244	-0.0044752	0.00330366	1.83216534	0.175872	0.3245534	Ncaph2
ENSMUSG00000008730.18	1304.10275	0.0012004	0.00275258	0.18619346	0.66610383	0.82535189	Hipk1
ENSMUSG00000008734.10	68.7330707	0.09839036	0.01010577	69.3671944	8.17E-17	2.88E-15	Gprc5b
ENSMUSG00000008763.17	2788.4481	0.00716746	0.0047747	2.16707977	0.14099375	0.27433415	Man1a2

ENSMUSG00000008822.16	215.633777	-0.0214698	0.00394581	29.4778186	5.66E-08	6.09E-07	Acyp1
ENSMUSG00000008845.10	238.024007	-0.0601918	0.00742692	61.93797	3.54E-15	1.02E-13	Cd163
ENSMUSG00000008855.18	755.619994	-0.0068089	0.00337761	4.02193447	0.04491217	0.11048169	Hdac5
ENSMUSG00000008859.9	610.016904	-0.0006075	0.00262627	0.05322161	0.81754936	0.92611878	Rala
ENSMUSG00000008892.14	1508.24698	-0.0060685	0.00251785	5.78079857	0.01620218	0.04780991	Vdac3
ENSMUSG00000008932.11	1.7904385	0.01896038	0.00893383	4.5472346	0.03297196	0.08591657	Slc1a7
ENSMUSG00000008958.15	305.540481	-0.0047547	0.00303832	2.43234716	0.11885482	0.24044624	Vps72
ENSMUSG00000008976.17	1056.12148	-0.0038295	0.00308137	1.50873348	0.21933245	0.3839091	Gabpa
ENSMUSG00000008999.8	35.1658459	-0.0193162	0.00528153	12.7681486	0.00035257	0.00175772	Bmp7
ENSMUSG00000009013.6	418.31707	-0.0061515	0.00462122	1.72516989	0.18902892	0.3430094	Dynll1
ENSMUSG00000009030.15	507.678858	0.00086663	0.00237949	0.13083368	0.71756921	0.86147753	Pdcl
ENSMUSG00000009035.16	506.138555	0.00634968	0.0035293	3.15032447	0.07591187	0.16899078	Tmem184b
ENSMUSG00000009073.17	509.931317	-0.0040036	0.00313131	1.62155241	0.20287546	0.36219973	Nf2
ENSMUSG00000009076.11	80.6674966	0.00633025	0.00348711	3.31375606	0.0687023	0.15591034	Zmat5
ENSMUSG00000009079.17	1930.46509	0.00037822	0.00308955	0.01492439	0.90276802	0.97702372	Ewsr1
ENSMUSG00000009090.18	1016.07584	-0.0009797	0.00240852	0.16384818	0.68563803	0.83963959	Ap1b1
ENSMUSG00000009092.6	62.4356025	-0.0232887	0.00983032	4.46502667	0.03459555	0.08948543	Derl3
ENSMUSG00000009097.11	4.11126074	0.03459076	0.01407428	6.25804874	0.01236303	0.03807213	Tbx1
ENSMUSG00000009108.16	24.7658699	-0.0228199	0.00614582	13.1484502	0.00028776	0.00147129	Gnat2
ENSMUSG00000009112.5	836.26328	-0.0081459	0.00363137	4.96061791	0.02593094	0.0705894	Bcl2l13
ENSMUSG00000009115.6	1.53267661	-0.0378022	0.01308336	8.97508235	0.00273686	0.01056665	Spatc1l
ENSMUSG00000009145.7	158.14205	0.0191746	0.00497148	14.7484491	0.00012285	0.00068824	Dqx1
ENSMUSG00000009185.3	28.0474276	0.18859781	0.01534746	82.9076268	8.60E-20	4.38E-18	Ccl8
ENSMUSG00000009207.16	92.3454393	-0.0006236	0.0033314	0.03453344	0.85257671	0.94920341	Lnpk
ENSMUSG00000009216.8	9.58800936	0.02844476	0.01589889	3.66781296	0.05547293	0.13146951	Fam163b
ENSMUSG00000009246.15	1.58228835	0.0141224	0.01421094	0.99169747	0.31932785	0.50517585	Trpm5
ENSMUSG00000009281.7	8205.08386	0.00576126	0.00352052	2.70027521	0.10033093	0.21072175	Rarres2
ENSMUSG00000009291.14	3836.84453	-0.0020793	0.00192565	1.16490069	0.28045149	0.45989911	Pttg1ip
ENSMUSG00000009292.19	153.234822	0.04661423	0.00576226	60.7890618	6.35E-15	1.79E-13	Trpm2
ENSMUSG00000009293.18	646.479649	-0.0126628	0.00344682	13.4456423	0.00024558	0.00127806	Ube2g2
ENSMUSG00000009350.14	15.1359744	0.13281377	0.02036141	16.0577278	6.14E-05	0.0003693	Mpo
ENSMUSG00000009376.16	1469.97155	-0.0006677	0.00421341	0.02492931	0.87454333	0.96122741	Met
ENSMUSG00000009378.5	1213.54026	0.02012594	0.00415615	23.3751475	1.33E-06	1.12E-05	Slc16a12
ENSMUSG00000009394.14	30.8094217	0.03759314	0.01034712	12.5755358	0.00039083	0.00192404	Syn2
ENSMUSG00000009406.14	116.292204	-5.01E-05	0.00305668	0.00026322	0.98705571	1	Elk1
ENSMUSG00000009418.18	170.027884	0.01465847	0.00453736	10.1499054	0.00144309	0.00605958	Nav1
ENSMUSG00000009470.18	1466.67962	0.00233095	0.00289291	0.63522617	0.42544477	0.61705451	Tnpo1
ENSMUSG00000009471.5	1.46979829	-0.0038215	0.04714412	-0.0342654	1	1	Myod1
ENSMUSG00000009535.14	261.829769	-0.0051324	0.00253017	4.05406749	0.04406505	0.10880635	Rnmt
ENSMUSG00000009545.15	31.1292299	-0.00891	0.00597859	2.29900699	0.12945674	0.25695251	Kcnq1
ENSMUSG00000009549.15	838.001935	-0.0046453	0.00309261	2.2555143	0.13313922	0.26261536	Srp14
ENSMUSG00000009555.17	569.048188	0.00096476	0.0024457	0.15632005	0.69256741	0.84453906	Cdk9
ENSMUSG00000009563.17	565.110997	0.00061604	0.00406095	0.02287777	0.87977536	0.96369968	Tor2a
ENSMUSG00000009566.13	2219.35496	-0.0119343	0.00408627	8.22440264	0.00413308	0.01502973	Fpgs
ENSMUSG00000009569.15	499.13173	0.00363602	0.00328056	1.18989143	0.27535146	0.45364903	Mrtfb
ENSMUSG00000009575.15	801.362593	-0.0040262	0.00297462	1.79599032	0.180198	0.33082821	Cbx5
ENSMUSG00000009585.19	276.426174	0.0585828	0.00535947	107.748097	3.05E-25	2.94E-23	Apobec3
ENSMUSG00000009614.17	13094.53	-0.0103113	0.00453172	5.03652023	0.0248183	0.0680577	Sardh
ENSMUSG00000009621.19	788.766257	0.0069317	0.00342966	4.10979565	0.04263551	0.10597547	Vav2
ENSMUSG00000009628.15	2.14639279	0.0235688	0.01717692	1.57317728	0.2097462	0.37144095	Tex15
ENSMUSG00000009630.11	394.645575	0.00053457	0.00213141	0.06250495	0.80257969	0.91721448	Ppp2cb
ENSMUSG00000009633.4	2189.63832	-0.0115395	0.00659983	2.55447442	0.10998238	0.22621824	G0s2
ENSMUSG00000009640.12	151.885078	-0.0012864	0.0023403	0.30389403	0.58145145	0.75923788	Dmap1
ENSMUSG00000009646.14	958.390134	-0.0032704	0.00426945	0.58675843	0.44367536	0.63587088	Pla2g12b
ENSMUSG00000009647.14	308.001507	0.00149335	0.00263849	0.31849144	0.57251565	0.75163575	Mcu
ENSMUSG00000009654.5	462.285385	-0.002037	0.00349572	0.33457698	0.56297634	0.74379465	Oit3
ENSMUSG00000009681.11	543.817143	-0.0044865	0.00308929	2.06621342	0.15059502	0.28832974	Bcr
ENSMUSG00000009687.15	130.55862	0.06264595	0.00608969	98.0934314	3.99E-23	2.96E-21	Fxyd5
ENSMUSG00000009731.5	6.32870123	-9.65E-05	0.00862942	0.00087468	0.97640598	1	Kcnd1
ENSMUSG00000009733.10	139.459573	-0.0050555	0.00373159	1.83385298	0.17567313	0.32426599	Tfcp2
ENSMUSG00000009734.19	2.73762675	0.00431044	0.020359	0.14726366	0.70116413	0.85079006	Pou6f2

ENSMUSG00000009739.19	124.024921	0.01151969	0.0043011	7.12188021	0.00761488	0.02533567	Pou6f1
ENSMUSG00000009741.17	1030.09262	-0.0029962	0.00217779	1.87066832	0.17139856	0.3182371	Ubp1
ENSMUSG00000009772.16	209.265705	-0.0042782	0.00367829	1.31113085	0.25219001	0.42422066	Nuak2
ENSMUSG00000009828.16	310.807469	0.00137867	0.0032754	0.17338306	0.67712251	0.83310441	Cilk1
ENSMUSG00000009863.15	3458.76175	-0.0167663	0.00308453	29.9068736	4.53E-08	4.95E-07	Sdhb
ENSMUSG00000009876.14	3.76368088	0.02325164	0.01258234	2.91393572	0.08781733	0.18984964	Cox4i2
ENSMUSG00000009894.16	985.640115	-0.0083539	0.00233869	12.8527434	0.00033698	0.00168786	Snap47
ENSMUSG00000009905.6	537.779522	-0.0076077	0.00295922	6.48129217	0.01090157	0.03432445	Kdsr
ENSMUSG00000009907.18	1110.33568	0.00442568	0.00300149	2.16202637	0.14145803	0.27495483	Vps4b
ENSMUSG00000009927.10	9442.89803	-0.0060318	0.00385346	2.45531164	0.11712792	0.23766166	Rps25
ENSMUSG00000009995.18	595.380342	-0.0042209	0.00227043	3.49652017	0.06149792	0.14273667	Tafazzin
ENSMUSG00000010021.14	6.59463972	0.03087096	0.00848539	12.1907145	0.00048028	0.00230946	Kif19a
ENSMUSG00000010025.20	6298.72515	0.00670023	0.00389579	2.65074328	0.10350149	0.21566163	Aldh3a2
ENSMUSG00000010044.13	4.79137187	-0.0241578	0.01033117	5.48549582	0.01917488	0.05495246	Zmynd10
ENSMUSG00000010045.3	375.569781	-0.0067275	0.00200733	11.2898149	0.00077933	0.00353216	Tmem115
ENSMUSG00000010047.13	763.367848	-0.0061498	0.0027767	4.77358286	0.02889962	0.07706734	Hyal2
ENSMUSG00000010048.11	591.218592	0.00061841	0.00238942	0.06721294	0.79543881	0.91331028	Ifrd2
ENSMUSG00000010051.16	896.67745	-0.0216195	0.00517882	16.7283982	4.31E-05	0.00026901	Hyal1
ENSMUSG00000010054.5	773.236696	-0.0036889	0.00283011	1.68977925	0.19363007	0.34908419	Tusc2
ENSMUSG00000010057.9	236.295761	-0.0069779	0.00223656	9.77795085	0.00176617	0.00723964	Nprl2
ENSMUSG00000010064.16	23060.3691	0.00015587	0.0043008	0.00133393	0.97086538	1	Slc38a3
ENSMUSG00000010066.17	4.37849636	-0.0077716	0.01767057	0.1221588	0.72670479	0.86810052	Cacna2d2
ENSMUSG00000010067.14	146.724943	0.01044756	0.00302569	11.7394898	0.00061188	0.00285283	Rassf1
ENSMUSG00000010080.16	8.68212704	0.022422	0.00988987	5.00465443	0.02527925	0.06911283	Epn3
ENSMUSG00000010086.18	4.36449083	0.00483535	0.01788895	0.09217087	0.76143527	0.89131623	Rnf112
ENSMUSG00000010095.14	669.792582	0.0082169	0.00313414	6.83782112	0.00892476	0.02899042	Slc3a2
ENSMUSG00000010097.16	1016.28458	-0.0073198	0.00494598	2.21031314	0.13709084	0.26849082	Nxf1
ENSMUSG00000010110.18	828.223847	-0.0046322	0.00199783	5.40363483	0.02009486	0.05718441	Stx5a
ENSMUSG00000010122.15	1873.17662	0.00446512	0.00278635	2.52479223	0.11206954	0.22947112	Slc47a1
ENSMUSG00000010142.13	15.9384959	0.09237551	0.00871531	96.2188905	1.03E-22	7.35E-21	Tnfrsf13b
ENSMUSG00000010154.8	4.30870609	0.04510287	0.01477178	8.52099355	0.00351073	0.01307088	Spire2
ENSMUSG00000010175.14	2219.90993	-0.0111469	0.00415021	7.0787516	0.00780033	0.02583244	Prox1
ENSMUSG00000010205.12	422.666931	0.0011664	0.00299942	0.15027086	0.69827665	0.84885852	Raver1
ENSMUSG00000010277.8	2889.36922	-0.0075183	0.00219495	11.5950389	0.00066128	0.00305882	Bltp2
ENSMUSG00000010290.8	267.438889	-0.0118971	0.0019761	35.6278876	2.39E-09	3.15E-08	AI597479
ENSMUSG00000010307.8	229.305494	0.03784345	0.00560175	44.0497993	3.20E-11	5.50E-10	Tmem86a
ENSMUSG00000010311.16	1.70449302	0.03127479	0.01223607	6.62253482	0.01006964	0.03208112	Optc
ENSMUSG00000010342.17	9.12120492	0.03890313	0.01217463	8.3088719	0.00394519	0.01444446	Tex14
ENSMUSG00000010358.14	436.600573	-0.0006621	0.00308762	0.04729117	0.82784572	0.93249935	Ifi35
ENSMUSG00000010362.10	175.973488	-0.0070302	0.00411031	2.87074967	0.09020284	0.19385187	Rdm1
ENSMUSG00000010376.17	927.995883	-0.0136784	0.00360978	14.5006532	0.00014011	0.00077338	Nedd8
ENSMUSG00000010392.9	765.772376	0.00168051	0.00251908	0.43494671	0.5095716	0.69617445	Gosr1
ENSMUSG00000010406.13	661.473931	0.00250418	0.00395621	0.40021091	0.52698036	0.71191699	Mrpl52
ENSMUSG00000010453.13	866.059274	-0.0047312	0.00271607	3.06025516	0.08022907	0.17656833	Kansl3
ENSMUSG00000010461.17	14.4127062	0.0515302	0.00714406	47.1033853	6.73E-12	1.28E-10	Eya4
ENSMUSG00000010476.16	5.86642646	0.04974032	0.0112693	18.0045748	2.20E-05	0.00014596	Ebf3
ENSMUSG00000010517.8	636.750167	-0.0074949	0.0027239	7.5420329	0.0060276	0.02074522	Faf1
ENSMUSG00000010538.15	38.9334425	-0.0168375	0.00486499	11.981678	0.00053726	0.00255172	Tsacc
ENSMUSG00000010554.15	286.622455	0.00020025	0.00292175	0.00463521	0.94572003	1	Mettl16
ENSMUSG00000010592.10	2.45703239	0.00755861	0.01383048	0.35556972	0.55097706	0.73347885	Dazl
ENSMUSG00000010601.15	2938.50963	-0.000567	0.00596782	0.00927591	0.92327317	0.9882157	Apol7a
ENSMUSG00000010607.9	374.220371	-0.0139403	0.00391258	12.7798029	0.00035038	0.00174902	Pigyl
ENSMUSG00000010608.16	5428.09603	0.00276171	0.00395547	0.48214104	0.48745411	0.6758173	Rbm25
ENSMUSG00000010609.16	3153.03085	-0.0071196	0.00464094	2.30164356	0.12923718	0.2565815	Psen2
ENSMUSG00000010651.5	16088.635	-0.0214279	0.00626359	10.5624188	0.0011541	0.0049809	Acaa1b
ENSMUSG00000010660.4	15.586418	0.0176755	0.00711041	5.74156056	0.01656807	0.04871284	Plcd1
ENSMUSG00000010663.6	11497.458	0.00833009	0.0035012	5.55809992	0.01839537	0.05311215	Fads1
ENSMUSG00000010721.16	122.712242	0.00112206	0.00302192	0.13940788	0.70887037	0.85602354	Lmbr1
ENSMUSG00000010751.16	24.3285161	0.01241463	0.00807206	2.23480446	0.13493369	0.26525638	Tnfrsf22
ENSMUSG00000010755.18	774.779949	-0.0007588	0.00417535	0.03216892	0.85765741	0.9520736	Cars
ENSMUSG00000010760.6	5.73726882	0.01242133	0.00902555	1.86245539	0.17234168	0.31960539	Phlda2

ENSMUSG00000010797.7	58.2364076	-0.0285838	0.00441772	41.8670356	9.77E-11	1.57E-09	Wnt2
ENSMUSG00000010803.14	25.6638244	0.02168899	0.01653544	1.85529195	0.17316916	0.3207538	Gabra1
ENSMUSG00000010830.9	9.37964331	0.00184319	0.00881063	0.04179703	0.83800716	0.93917284	Kdelr3
ENSMUSG00000010911.13	319.896525	-0.0063034	0.00363486	3.08192963	0.07916655	0.17468787	Apip
ENSMUSG00000010914.11	571.999449	-0.0205967	0.00336176	37.3973854	9.64E-10	1.34E-08	Pdhx
ENSMUSG00000010936.12	432.144432	-0.0004812	0.002358	0.04168295	0.83822533	0.93921058	Vac14
ENSMUSG00000011008.14	11.6162463	0.06837904	0.00940371	41.4564039	1.21E-10	1.91E-09	Mcoln2
ENSMUSG00000011034.7	39.8121133	0.03301417	0.00520393	39.9440008	2.61E-10	3.94E-09	Slc5a1
ENSMUSG00000011096.18	666.469111	-0.0055595	0.00312817	3.10829681	0.07789438	0.17249242	Akt1s1
ENSMUSG00000011114.19	778.882065	0.00786603	0.0034362	5.11675734	0.02369586	0.06550521	Tbrg1
ENSMUSG00000011148.15	342.654303	0.00790988	0.00402934	3.76801183	0.05224169	0.12490346	Adssl1
ENSMUSG00000011154.18	1.55207968	-0.0029087	0.02259724	-0.0698775	1	1	Cfap161
ENSMUSG00000011158.9	265.306063	-0.0018522	0.00260607	0.51606478	0.47252518	0.66315476	Brf1
ENSMUSG00000011179.9	1489.8205	-0.0052401	0.00350465	2.2055962	0.13751072	0.26911535	Odc1
ENSMUSG00000011254.17	56.3011638	0.0080019	0.00388561	4.15236608	0.04157639	0.10382432	Thg1l
ENSMUSG00000011256.17	52.6239406	0.03230548	0.0066333	21.1264627	4.30E-06	3.30E-05	Adam19
ENSMUSG00000011257.20	754.294454	-0.0136555	0.00325694	17.4966108	2.88E-05	0.00018583	Pabpc4
ENSMUSG00000011263.18	140.724597	-0.0016348	0.00325901	0.24975374	0.61724853	0.78847616	Exoc3l2
ENSMUSG00000011267.9	7.2020223	0.00373183	0.00638643	0.32846199	0.56656613	0.74649621	Zfp296
ENSMUSG00000011305.12	861.415661	-0.0214814	0.00459085	21.3863141	3.75E-06	2.92E-05	Plin5
ENSMUSG00000011306.8	278.899829	0.00242719	0.00171797	1.99701316	0.15760952	0.29830768	Sugp1
ENSMUSG00000011350.17	0.72554547	-0.0112881	0.07082544	0.01725728	0.89548503	0.97320224	Gm5893
ENSMUSG00000011382.9	3293.05331	-0.0029996	0.00337884	0.77143563	0.3797733	0.57129491	Dhdh
ENSMUSG00000011427.17	148.405504	-0.0079853	0.00351181	5.11681422	0.02369508	0.06550521	Zfp790
ENSMUSG00000011463.6	5.13191113	-0.1109932	0.04317865	3.51709921	0.06073867	0.14135834	Cpb1
ENSMUSG00000011658.17	138.252624	-0.0097892	0.0031429	9.61706809	0.00192777	0.0078232	Fuz
ENSMUSG00000011751.18	9.71982363	0.04274211	0.01159156	12.8255657	0.00034192	0.00171037	Sptbn4
ENSMUSG00000011752.8	3125.60771	-0.0058877	0.00230588	6.57399608	0.01034794	0.03281344	Pgam1
ENSMUSG00000011831.17	2606.88952	0.00032203	0.00394987	0.00650895	0.93569799	0.99465354	Evi5
ENSMUSG00000011832.17	94.5814091	-0.0002108	0.00296316	0.00508216	0.94316751	0.99876665	Evi5l
ENSMUSG00000011837.5	156.646962	-0.0008314	0.00264278	0.09867483	0.75342572	0.88590154	Snapc2
ENSMUSG00000011877.14	193.302318	0.00641126	0.00353745	3.29559522	0.06946593	0.15723422	Git1
ENSMUSG00000011884.14	125.537107	0.0442077	0.00456295	88.1429929	6.09E-21	3.49E-19	Gltp
ENSMUSG00000011958.18	951.024068	0.00185295	0.00225746	0.66182727	0.41591559	0.60715387	Bnip2
ENSMUSG00000011960.13	974.395298	-0.0064597	0.00399566	2.58946881	0.10757641	0.22221906	Ccnt1
ENSMUSG00000012017.5	75.8879917	0.01075227	0.00369032	8.20220205	0.00418396	0.01519366	Scarf2
ENSMUSG00000012042.10	1.77169757	-0.0088011	0.01436032	0.31940426	0.57196589	0.75126064	4930579F01Rik
ENSMUSG00000012076.9	119.576581	-0.0029175	0.00499505	0.33133052	0.56487668	0.74531111	Brms1l
ENSMUSG00000012114.18	478.906132	0.00046715	0.00274834	0.02851129	0.86591238	0.956376	Med15
ENSMUSG00000012117.14	437.635868	0.00024951	0.00220272	0.01277819	0.90999831	0.98081708	Dhdds
ENSMUSG00000012123.18	67.9294189	0.05070687	0.00811365	36.0902461	1.88E-09	2.51E-08	Crybg2
ENSMUSG00000012126.17	7.12645222	0.01997517	0.01089317	3.12361808	0.07716527	0.17124766	Ubxn11
ENSMUSG00000012187.14	26.9189137	0.0324927	0.00949071	8.17253808	0.00425294	0.01541567	Mogat1
ENSMUSG00000012282.3	2.57565939	-0.0108324	0.18254549	-0.0010268	1	1	Wnt8a
ENSMUSG00000012296.17	107.935564	-0.0128099	0.00429123	8.79578626	0.00301927	0.01148926	Tjap1
ENSMUSG00000012350.16	27.622355	0.01618689	0.00775335	4.2472031	0.03931503	0.09914298	Ehf
ENSMUSG00000012405.17	4999.86444	-0.0019081	0.00309505	0.38305235	0.53597418	0.72027161	Rpl15
ENSMUSG00000012422.15	1361.31854	-0.0085592	0.00289654	8.65162533	0.0032677	0.01229021	Tmem167
ENSMUSG00000012428.10	4004.86644	0.01512405	0.00642653	4.68738842	0.0303848	0.08034909	Steap4
ENSMUSG00000012429.10	281.049803	-0.0069582	0.00330105	4.3456688	0.03710319	0.09452537	Mplkip
ENSMUSG00000012443.5	37.5316329	0.01737499	0.0071115	5.54228235	0.01856236	0.05352222	Kif11
ENSMUSG00000012483.5	259.418694	-0.0114069	0.00454914	6.36466429	0.01164151	0.03617069	Rpa3
ENSMUSG00000012519.15	114.702423	0.04465273	0.00492509	79.3069032	5.32E-19	2.43E-17	Mlkl
ENSMUSG00000012535.15	922.670049	-0.0040577	0.00247184	2.67574415	0.10188769	0.21328864	Tnpo3
ENSMUSG00000012609.20	204.188341	-0.0141313	0.00383203	13.4077659	0.00025058	0.00130097	Ttll5
ENSMUSG00000012640.17	280.300286	-0.003169	0.00325763	0.95549071	0.32832561	0.51533159	Zfp715
ENSMUSG00000012705.17	2.51971083	0.01326925	0.02207092	0.38843576	0.53312278	0.71757322	Retn
ENSMUSG00000012777.15	9.53373652	-0.0127734	0.03851061	0.07414941	0.78538837	0.90711375	Acp4
ENSMUSG00000012819.17	3.65721889	0.07072255	0.01923869	10.6502993	0.00110053	0.0047768	Cdh23
ENSMUSG00000012848.16	5006.51491	-0.000158	0.00353621	0.0020183	0.96416669	1	Rps5
ENSMUSG00000012889.9	7.9202272	0.07154905	0.00978735	44.902333	2.07E-11	3.66E-10	Podnl1

ENSMUSG00000013033.17	121.411803	0.02092127	0.00515989	15.9247424	6.59E-05	0.00039357	Adgrl1
ENSMUSG00000013076.18	247.257538	-0.0039491	0.00400221	0.95561533	0.32829407	0.51531638	Amotl1
ENSMUSG00000013089.16	113.261209	0.01977586	0.00452371	18.0952359	2.10E-05	0.00013996	Etv5
ENSMUSG00000013150.16	150.87075	-0.0170156	0.00456655	12.9233648	0.00032451	0.00163264	Gfod2
ENSMUSG00000013155.11	9.43860664	-0.0031708	0.00703584	0.20400413	0.65150806	0.81397256	Enkd1
ENSMUSG00000013160.8	1152.73492	-0.0018713	0.00210578	0.78446304	0.37577965	0.56687808	Atp6v0d1
ENSMUSG00000013236.18	145.239237	0.01508472	0.00582733	6.61465848	0.01011427	0.03220346	Ptprs
ENSMUSG00000013275.10	129.151868	0.00785808	0.00458741	2.78883576	0.09492317	0.20183313	Slc41a1
ENSMUSG00000013367.6	10.5136152	0.05256997	0.00878443	33.221409	8.22E-09	1.01E-07	Iglon5
ENSMUSG00000013415.10	6.50453989	0.00234373	0.02189075	-0.5508536	1	1	Igf2bp1
ENSMUSG00000013418.9	1.67982572	0.01083962	0.0223194	0.40956959	0.52218804	0.70807906	B4galnt2
ENSMUSG00000013419.8	36.7565711	0.0100379	0.0065734	2.29822799	0.12952169	0.25703816	Zfp651
ENSMUSG00000013465.20	407.953072	-0.0002882	0.00216298	0.01763352	0.89435847	0.97287826	Nelfb
ENSMUSG00000013483.15	3.14775929	-0.0058031	0.0109293	0.30181852	0.58274461	0.76029508	Card14
ENSMUSG00000013495.16	183.298647	0.0015177	0.00302354	0.24944505	0.61746611	0.78862053	Tmem175
ENSMUSG00000013523.14	13.4785317	0.07660388	0.01354936	29.0903625	6.91E-08	7.34E-07	Bcas1
ENSMUSG00000013539.15	646.619	-0.0089909	0.00282332	10.3530715	0.00129259	0.00550135	Tango2
ENSMUSG00000013584.6	11.1445285	0.00299773	0.00830015	0.12615445	0.72245304	0.86513898	Aldh1a2
ENSMUSG00000013593.13	3579.1942	-0.0127367	0.00323366	15.7072156	7.39E-05	0.00043643	Ndufs2
ENSMUSG00000013611.15	1.05879134	0.08904612	0.01549795	30.8170023	2.84E-08	3.20E-07	Snx31
ENSMUSG00000013622.16	1181.8202	-0.0025594	0.00267646	0.91922488	0.33767858	0.52518872	Atraid
ENSMUSG00000013629.17	239.362298	-0.0061313	0.00553954	1.16793431	0.27982609	0.45912892	Cad
ENSMUSG00000013643.14	9.14353697	-0.0007703	0.02495924	-0.0053482	1	1	Lypd8
ENSMUSG00000013646.18	310.115845	-0.0050885	0.00259418	3.83666328	0.05014322	0.12085407	Sh3bp5l
ENSMUSG00000013662.7	1169.46113	-0.0045595	0.00201333	5.12343778	0.02360481	0.06531476	Atad1
ENSMUSG00000013663.9	2943.05712	0.00367383	0.00247414	2.14553561	0.14298513	0.27724821	Pten
ENSMUSG00000013698.13	468.931289	0.01991183	0.00348207	31.6871856	1.81E-08	2.10E-07	Pea15a
ENSMUSG00000013701.14	2330.7662	-0.0199064	0.00294795	45.8020004	1.31E-11	2.38E-10	Timm23
ENSMUSG00000013707.4	42.0552095	0.05903921	0.00657153	72.3665271	1.79E-17	6.85E-16	Tnfaip8l2
ENSMUSG00000013736.17	370.778783	-0.0037328	0.00249859	2.19787958	0.13820073	0.27017011	Trnt1
ENSMUSG00000013766.12	2.4532715	0.02896226	0.01202895	5.46099485	0.01944557	0.0556066	Ly6g6e
ENSMUSG00000013787.16	918.799613	0.00514215	0.00273701	3.53624409	0.06004128	0.14001716	Ehmt2
ENSMUSG00000013822.8	811.069765	-0.0093696	0.00306624	9.34348585	0.00223779	0.00890963	Elof1
ENSMUSG00000013833.16	300.863019	-0.0010603	0.00256807	0.1678595	0.68202119	0.83718092	Med16
ENSMUSG00000013846.11	1694.0969	-0.004937	0.00346592	1.95432812	0.16212122	0.30453895	St3gal1
ENSMUSG00000013858.15	1324.39436	-0.0116899	0.00260636	20.0980697	7.36E-06	5.37E-05	Tmem259
ENSMUSG00000013878.19	482.679787	0.00172838	0.00201586	0.73353253	0.39174055	0.58309602	Rnf170
ENSMUSG00000013921.16	42.1366958	0.03519598	0.00980736	12.2691961	0.0004605	0.00222248	Clip3
ENSMUSG00000013936.13	869.844806	-0.1645121	0.03948867	5.35721981	0.02063665	0.05850192	Myl2
ENSMUSG00000013973.13	385.93896	-0.003656	0.00353963	1.04365263	0.30697349	0.49210063	Dedd
ENSMUSG00000013974.4	7.39307999	0.10370873	0.01095684	73.1831659	1.18E-17	4.61E-16	Mcemp1
ENSMUSG00000013997.11	1543.92103	-0.0040764	0.00215628	3.5532468	0.05942906	0.13880081	Nit1
ENSMUSG00000014030.16	36.6585761	0.06065443	0.01063553	24.5639778	7.19E-07	6.33E-06	Pax5
ENSMUSG00000014039.19	107.559747	0.0067161	0.00453611	2.18649881	0.13922547	0.27158813	Prdm15
ENSMUSG00000014074.18	395.79723	-0.0024346	0.00451122	0.2831307	0.59465655	0.76995677	Rnf168
ENSMUSG00000014075.16	36.4977086	0.0172524	0.00534536	10.3477757	0.00129631	0.00551194	Dynlt2b
ENSMUSG00000014077.14	5292.29885	-0.0082536	0.00245326	11.2353511	0.00080254	0.00362341	Chp1
ENSMUSG00000014158.13	16.0939391	0.03907752	0.00733165	27.3942853	1.66E-07	1.66E-06	Trpv4
ENSMUSG00000014164.16	194.093653	-0.0003067	0.00337766	0.00827988	0.92749746	0.99043878	Klhl3
ENSMUSG00000014177.11	508.560852	-0.0007451	0.00201039	0.13492577	0.71337852	0.85867547	Tvp23b
ENSMUSG00000014195.17	1042.4122	0.00270248	0.00223443	1.45725741	0.227367	0.39364884	Dnajc7
ENSMUSG00000014226.11	828.815658	-0.0080635	0.00306154	6.65683641	0.00987761	0.03157352	Cacybp
ENSMUSG00000014232.15	130.583879	0.00370016	0.00252555	2.14177795	0.14333569	0.27784412	Cluap1
ENSMUSG00000014243.6	145.795664	-0.0061866	0.0037908	2.64996838	0.10355196	0.21573858	Zswim7
ENSMUSG00000014245.9	66.1542013	-0.006482	0.00233461	7.77756687	0.0052899	0.0185666	Pigl
ENSMUSG00000014294.5	1487.52548	-0.0069908	0.00470339	2.2437136	0.13415844	0.26404046	Ndufa2
ENSMUSG00000014301.14	324.162248	-0.0138814	0.00403071	11.9770708	0.00053859	0.00255633	Pam16
ENSMUSG00000014303.14	36.6186569	0.01908195	0.00651168	8.31581175	0.00393014	0.01439505	Glis2
ENSMUSG00000014313.16	5406.24166	-0.016087	0.00398602	16.5723345	4.68E-05	0.00028997	Cox6c
ENSMUSG00000014329.15	130.937738	0.02692475	0.00462999	32.9809683	9.31E-09	1.13E-07	Bicc1
ENSMUSG00000014349.8	800.626139	0.00168434	0.00188877	0.79405071	0.37287808	0.56364792	Ube2z

ENSMUSG00000014353.16	500.178591	-0.0013349	0.00261865	0.25869093	0.61102093	0.78327843	Tmem87b
ENSMUSG00000014355.11	756.391742	-0.0018961	0.00297938	0.39892461	0.52764515	0.71244783	Anapc1
ENSMUSG00000014361.6	441.389077	0.01683779	0.00392162	18.3309968	1.86E-05	0.00012511	Mertk
ENSMUSG00000014402.16	742.124711	-0.0033055	0.0019864	2.76620288	0.09627429	0.20411016	Tsg101
ENSMUSG00000014418.17	295.887283	0.006595	0.00307442	4.57628256	0.03241747	0.08479521	Hps5
ENSMUSG00000014426.10	420.756166	0.00129663	0.00319892	0.1641073	0.68540283	0.83949976	Map3k4
ENSMUSG00000014444.18	151.995525	0.02788108	0.00541986	26.5309756	2.59E-07	2.50E-06	Piezo1
ENSMUSG00000014453.5	12.647246	0.09602423	0.00963774	74.8376942	5.11E-18	2.06E-16	Blk
ENSMUSG00000014470.5	210.821048	0.00924144	0.00291449	10.0213061	0.0015474	0.00644366	Rnf166
ENSMUSG00000014496.9	655.868579	0.00189926	0.00233088	0.67079353	0.41277508	0.6042148	Ankrd28
ENSMUSG00000014498.10	472.268999	-0.0127089	0.00418566	9.16530896	0.00246646	0.00965477	Ankrd52
ENSMUSG00000014503.16	22.5147756	-0.0289902	0.00858953	10.7980642	0.00101606	0.00445692	Pkd2l2
ENSMUSG00000014504.17	505.457842	-0.0116139	0.00301819	14.7740523	0.00012119	0.00068042	Srp19
ENSMUSG00000014542.4	2198.13334	0.02792353	0.00595812	20.7056371	5.36E-06	4.03E-05	Clec4f
ENSMUSG00000014543.15	2.29799693	0.06767677	0.01371298	21.3511492	3.82E-06	2.97E-05	Klra17
ENSMUSG00000014547.11	402.589749	0.00416041	0.00323035	1.65300546	0.19855036	0.35620361	Wdfy2
ENSMUSG00000014550.15	265.631222	-0.0029116	0.00285556	1.01238852	0.3143313	0.49965077	Rbsn
ENSMUSG00000014551.9	344.805389	-0.0117404	0.00384643	9.31144778	0.00227726	0.00904083	Mrps25
ENSMUSG00000014554.14	202.317071	-0.004156	0.00252364	2.69890666	0.10041709	0.21086516	Dguok
ENSMUSG00000014592.23	195.69406	-0.0092042	0.00492882	3.4451867	0.06343625	0.14633073	Camta1
ENSMUSG00000014599.11	280.61407	0.02664791	0.00595833	19.2909158	1.12E-05	7.87E-05	Csf1
ENSMUSG00000014601.14	287.079608	-0.0016402	0.00229292	0.51168478	0.47441043	0.66518691	Strip1
ENSMUSG00000014602.16	30.3120365	0.02468261	0.01191589	3.72645729	0.05355687	0.12759519	Kif1a
ENSMUSG00000014606.15	1411.33393	-0.0037078	0.00353416	1.11674296	0.29062086	0.47204625	Slc25a11
ENSMUSG00000014609.17	3.45027646	-0.0045336	0.01210739	0.11898715	0.73013562	0.87031782	Chrne
ENSMUSG00000014633.16	162.449442	-0.0071025	0.00418242	2.90494407	0.08830832	0.19063152	Cmc2
ENSMUSG00000014668.16	363.211156	-0.0009305	0.00242285	0.14700755	0.70141161	0.85095902	Chfr
ENSMUSG00000014686.9	1.39300023	0.10400676	0.01709822	38.370189	5.85E-10	8.39E-09	Ceacam16
ENSMUSG00000014704.11	6.06457151	0.01220829	0.00599457	4.14009466	0.04187883	0.10444176	Hoxa2
ENSMUSG00000014725.17	3.09226692	0.03052468	0.0185446	4.10329404	0.04279975	0.10635007	Adam28
ENSMUSG00000014748.14	1299.12725	-0.0019552	0.00215192	0.80975028	0.36819409	0.55857888	Tex261
ENSMUSG00000014763.9	452.421114	0.00242769	0.00179249	1.83142576	0.17595923	0.32468898	Fam120b
ENSMUSG00000014767.18	232.194187	-0.0041762	0.0027935	2.22203976	0.1360532	0.26690093	Tbp
ENSMUSG00000014769.11	2593.59937	-0.0046218	0.00279902	2.74761693	0.0973995	0.20594035	Psmb1
ENSMUSG00000014771.14	293.355054	-4.23E-05	0.00369485	0.00013426	0.99075497	1	Pdcd2
ENSMUSG00000014773.14	74.1527924	0.01140676	0.00453759	6.48412185	0.01088423	0.03428358	Dll1
ENSMUSG00000014776.8	27.1893168	0.01194549	0.00588012	4.24957291	0.0392602	0.09902589	Nol3
ENSMUSG00000014778.11	115.158543	0.01226539	0.00421217	8.33480407	0.00388927	0.01426851	Fhod1
ENSMUSG00000014782.17	3.46367187	0.04196078	0.0144219	7.43048662	0.0064128	0.02188573	Plekhg4
ENSMUSG00000014786.9	10.319382	0.0346355	0.00794813	18.3660437	1.82E-05	0.00012294	Slc9a5
ENSMUSG00000014791.11	398.418984	-0.0007832	0.00266605	0.08685786	0.7682104	0.895738	Elmo3
ENSMUSG00000014813.10	4.96218967	0.03423428	0.01227374	7.75490566	0.00535668	0.01876192	Stc1
ENSMUSG00000014837.16	24.644923	-0.0041489	0.00466756	0.78240016	0.37640809	0.56760829	Matcap1
ENSMUSG00000014846.13	13.1549766	0.03014731	0.00816633	13.1531332	0.00028704	0.00146793	Tppp3
ENSMUSG00000014850.16	243.578003	0.00076319	0.00274121	0.07744357	0.78079217	0.90391115	Msh3
ENSMUSG00000014852.17	90.7039085	0.01124778	0.00364113	9.44386526	0.00211857	0.0084966	Adamts13
ENSMUSG00000014856.13	833.596006	-0.0166518	0.004161	15.974054	6.42E-05	0.00038471	Tmem208
ENSMUSG00000014859.10	308.570596	-0.0050011	0.0025035	4.05178546	0.04412465	0.10890792	E2f4
ENSMUSG00000014867.10	4435.60651	-0.0020271	0.00204124	0.9817653	0.32176337	0.50800662	Surf4
ENSMUSG00000014873.16	224.25981	0.00125457	0.00247483	0.26333695	0.60783684	0.78051174	Surf2
ENSMUSG00000014905.5	1429.13514	-0.0023555	0.00423278	0.30959539	0.57792857	0.7561868	Dnajb9
ENSMUSG00000014907.11	88.1080321	0.00362864	0.00307676	1.35750253	0.24397078	0.41445226	Naf1
ENSMUSG00000014932.16	552.487443	-0.0041284	0.00235181	3.06970562	0.07976391	0.17572498	Yes1
ENSMUSG00000014956.16	2828.22793	-0.0033608	0.00306503	1.16450788	0.2805326	0.46000014	Ppp1cb
ENSMUSG00000014959.13	1569.41964	-0.0002424	0.00150118	0.0262882	0.87119847	0.95936627	Gorasp2
ENSMUSG00000014980.15	157.792394	-0.0059932	0.00305278	3.81490075	0.05079861	0.12214661	Tsen15
ENSMUSG00000015002.19	1109.34719	-0.001414	0.00240904	0.34082342	0.55935431	0.74077512	Efr3a
ENSMUSG00000015013.10	246.221169	-0.0001976	0.00422402	0.00218738	0.96269695	1	Trappc2l
ENSMUSG00000015016.9	428.012189	-0.0106439	0.00251389	17.9807486	2.23E-05	0.00014759	Acsf3
ENSMUSG00000015023.8	418.097443	-0.0039964	0.00207037	3.69081615	0.05471284	0.12995945	Ddx19a
ENSMUSG00000015027.12	168.028385	0.03502113	0.0039888	77.3144771	1.46E-18	6.30E-17	Galns

ENSMUSG00000015053.15	29.5404868	0.00640031	0.00579212	1.19389779	0.27454473	0.45250958	Gata2
ENSMUSG00000015083.12	7316.44928	-0.0085086	0.00484494	3.02921507	0.08177757	0.1792737	C8g
ENSMUSG00000015085.9	53.8778739	0.00335836	0.0044983	0.55692931	0.45550012	0.64719433	Entpd2
ENSMUSG00000015087.15	526.680141	-0.0038133	0.00349122	1.19428439	0.27446704	0.45241315	Rabl6
ENSMUSG00000015090.14	60.0475799	-0.0412157	0.00821982	24.6128974	7.01E-07	6.19E-06	Ptgds
ENSMUSG00000015092.10	2223.7508	-0.0080391	0.00283939	8.06284758	0.00451821	0.01621771	Edf1
ENSMUSG00000015093.16	4.96116108	-0.0373249	0.00913999	16.6391712	4.52E-05	0.00028049	Clic3
ENSMUSG00000015094.17	77.8230518	0.00554699	0.00503902	1.21052333	0.2712285	0.44812826	Npdc1
ENSMUSG00000015095.13	589.838181	-0.0103854	0.00198811	27.4039356	1.65E-07	1.65E-06	Fbxw5
ENSMUSG00000015112.16	3770.21208	-0.0136914	0.00314809	18.6051821	1.61E-05	0.00010973	Slc25a13
ENSMUSG00000015120.17	1604.29614	0.00502113	0.00149741	11.1497111	0.00084045	0.00376931	Ube2i
ENSMUSG00000015126.11	219.193793	-0.0023649	0.00268967	0.78555478	0.37544766	0.5665222	Tsr3
ENSMUSG00000015127.15	391.822802	-0.0010507	0.00300777	0.11906332	0.73005263	0.87027617	Unkl
ENSMUSG00000015133.18	303.246343	0.01361544	0.00363873	14.0233932	0.00018055	0.00097537	Lrrk1
ENSMUSG00000015134.16	15.7410569	0.03956599	0.00638978	37.1888199	1.07E-09	1.48E-08	Aldh1a3
ENSMUSG00000015143.16	494.084328	0.00697416	0.0030426	5.30403631	0.02127607	0.05988988	Actn1
ENSMUSG00000015149.15	795.622238	-0.0017276	0.00159178	1.17418885	0.27854221	0.45753163	Sirt2
ENSMUSG00000015165.17	1492.86995	0.00070847	0.00273219	0.06682237	0.79602087	0.91380065	Hnrnpl
ENSMUSG00000015176.12	543.251005	-0.0160627	0.0038143	17.709602	2.57E-05	0.00016766	Nolc1
ENSMUSG00000015189.13	561.970225	-0.0046395	0.00278558	2.71352494	0.09950091	0.20940737	Casd1
ENSMUSG00000015202.14	159.80739	0.00077033	0.00268884	0.08061708	0.7764629	0.90104078	Cnksr3
ENSMUSG00000015214.15	590.906178	-0.0004249	0.00276811	0.02325588	0.87879355	0.9632329	Mtmr1
ENSMUSG00000015217.12	279.448323	-0.0071879	0.00320896	5.05002851	0.02462553	0.06758301	Hmgb3
ENSMUSG00000015222.19	121.521052	0.02117116	0.0069787	8.53833924	0.00347743	0.01296327	Map2
ENSMUSG00000015224.11	155.790054	0.00281958	0.00467416	0.37274821	0.54151005	0.72514939	Cyp2j9
ENSMUSG00000015243.5	3153.93084	-0.0071327	0.0032882	4.69077124	0.03032503	0.08022487	Abca1
ENSMUSG00000015247.11	1281.81823	-0.0019346	0.00356402	0.28975789	0.59037573	0.76651997	Nipsnap3b
ENSMUSG00000015289.5	330.99617	-0.0089233	0.00322764	7.773373	0.0053022	0.01859869	Lage3
ENSMUSG00000015290.16	684.447413	-0.0069078	0.00299757	5.32352835	0.02103937	0.0593511	Ubl4a
ENSMUSG00000015291.11	2190.16054	-0.0029326	0.0033225	0.78670771	0.3750975	0.56617495	Gdi1
ENSMUSG00000015305.7	726.263207	-0.0055609	0.00351265	2.45563751	0.11710362	0.23763279	Sash1
ENSMUSG00000015312.10	38.574488	0.03279598	0.00733984	18.1208014	2.07E-05	0.00013825	Gadd45b
ENSMUSG00000015314.11	29.2622685	0.08770486	0.00874405	82.037657	1.34E-19	6.69E-18	Slamf6
ENSMUSG00000015316.12	4.83991882	0.08444761	0.01019789	58.8962893	1.66E-14	4.43E-13	Slamf1
ENSMUSG00000015335.17	189.132502	-0.0073435	0.00274713	7.27184712	0.00700437	0.02364465	Zdhhc12
ENSMUSG00000015337.6	618.966465	-0.0138853	0.00372947	14.0138055	0.00018147	0.00097991	Endog
ENSMUSG00000015340.11	642.525734	0.07373404	0.00589164	142.32445	8.26E-33	2.53E-30	Cybb
ENSMUSG00000015341.18	1305.63221	-0.0060754	0.00176459	11.7306271	0.0006148	0.00286404	Golga7
ENSMUSG00000015342.7	105.346808	-0.0088341	0.0034074	6.40445449	0.01138344	0.03556627	Xk
ENSMUSG00000015354.9	58.3343868	0.0210081	0.00691744	8.1136644	0.00439328	0.01585373	Pcolce2
ENSMUSG00000015355.14	73.6039143	0.05917564	0.00596805	88.2591286	5.74E-21	3.30E-19	Cd48
ENSMUSG00000015357.11	3205.51108	-0.0169342	0.00506993	10.5931172	0.00113509	0.00490875	Clpx
ENSMUSG00000015363.14	1529.12985	-0.0057812	0.00250041	5.36993552	0.02048673	0.05813983	Trabd
ENSMUSG00000015365.16	33.4767884	-0.1086049	0.06508455	0.14575824	0.70262234	0.85163938	Mov10l1
ENSMUSG00000015377.11	108.084563	-0.0199956	0.00329288	37.2604488	1.03E-09	1.43E-08	Dennd6b
ENSMUSG00000015396.5	71.8685265	0.06313159	0.00587503	101.26445	8.05E-24	6.55E-22	Cd83
ENSMUSG00000015401.13	108.015689	-0.0036545	0.01527448	0.0636918	0.80075328	0.91618159	Cltrn
ENSMUSG00000015405.16	9.61472777	-0.0209189	0.00654805	10.3238453	0.00131322	0.00557081	Ace2
ENSMUSG00000015437.6	22.5927981	0.10558485	0.0084191	136.063651	1.93E-31	4.80E-29	Gzmb
ENSMUSG00000015452.15	6.29765645	0.01394159	0.0072481	3.57015535	0.05882681	0.13763407	Ager
ENSMUSG00000015461.16	355.144954	-0.002046	0.00342749	0.3551325	0.55122203	0.73361487	Atf6b
ENSMUSG00000015467.15	9.19741157	-0.0028517	0.0068795	0.16784626	0.68203304	0.83718092	Egfl8
ENSMUSG00000015468.15	89.6030446	-0.0021004	0.00706531	0.08386701	0.77212379	0.89794939	Notch4
ENSMUSG00000015474.17	425.185367	-0.0020204	0.00248853	0.66362477	0.41528317	0.60653099	Ppt2
ENSMUSG00000015476.7	17.4940014	0.03480891	0.00885859	14.6151813	0.00013185	0.00073257	Prrt1
ENSMUSG00000015478.14	1053.29544	-0.0109767	0.00318481	11.7439917	0.0006104	0.00284692	Rnf5
ENSMUSG00000015484.4	2.1868806	0.02279946	0.01442432	2.92979993	0.08695827	0.18835501	Fam163a
ENSMUSG00000015488.15	607.938194	-0.0064662	0.00285411	5.165274	0.02304282	0.06401508	Cacfd1
ENSMUSG00000015501.11	216.125244	0.01377326	0.00365052	13.8918812	0.00019363	0.00103631	Hivep2
ENSMUSG00000015519.13	1.46991576	-0.0009584	0.02680467	-0.0183659	1	1	2310057J18Rik
ENSMUSG00000015522.20	731.979422	0.00182767	0.00193952	0.87887496	0.34851	0.53647126	Arnt

ENSMUSG00000015533.10	10.0760648	0.03963366	0.00948451	15.631385	7.70E-05	0.00045146	Itga2
ENSMUSG00000015536.15	2155.30184	-0.0071794	0.00241168	8.98479924	0.00272235	0.01051749	Mocs2
ENSMUSG00000015542.18	399.851248	-0.0018159	0.00327119	0.30889318	0.57836017	0.75654167	Nat9
ENSMUSG00000015568.17	2373.37408	0.03635863	0.00715228	24.8330987	6.25E-07	5.60E-06	Lpl
ENSMUSG00000015575.16	652.88022	0.00578577	0.00338022	2.91967289	0.0875056	0.18931454	Atp6v0e
ENSMUSG00000015579.6	7.07722578	-0.0230251	0.04010095	0.34074876	0.55939734	0.74077512	Nkx2-5
ENSMUSG00000015597.18	826.325206	-0.0042771	0.00466844	0.83368031	0.36121048	0.55078294	Zfp318
ENSMUSG00000015599.10	38.1255933	0.01233968	0.00710656	2.75249646	0.09710271	0.2054786	Ttbk1
ENSMUSG00000015605.7	177.607702	-0.0085245	0.00413453	4.18429572	0.04080016	0.10230838	Srf
ENSMUSG00000015619.11	13.3872502	0.04381949	0.01150908	12.5231671	0.00040194	0.00197296	Gata3
ENSMUSG00000015627.6	4.31860599	0.02810016	0.00760539	13.4022432	0.00025132	0.00130423	Gata5
ENSMUSG00000015647.10	88.6074472	-0.0078119	0.0077187	1.01539986	0.31361267	0.49901291	Lama5
ENSMUSG00000015652.10	5.77710405	0.00369906	0.01089118	0.11275824	0.73702551	0.87459669	Steap1
ENSMUSG00000015653.14	241.009486	0.02987729	0.00465081	41.9608815	9.31E-11	1.50E-09	Steap2
ENSMUSG00000015656.18	25382.9647	-0.0073901	0.00387927	3.5076419	0.06108634	0.14201898	Hspa8
ENSMUSG00000015659.13	60.735131	0.00132464	0.00455398	0.08441838	0.77139671	0.8975756	Serac1
ENSMUSG00000015668.14	308.597037	-0.0032604	0.00240877	1.83753208	0.17524048	0.32379334	Pdzd11
ENSMUSG00000015671.12	1729.13254	-0.005753	0.00252124	5.2237542	0.0222804	0.06226369	Psma2
ENSMUSG00000015672.5	642.025365	-0.013647	0.00260732	27.5892133	1.50E-07	1.51E-06	Mrpl32
ENSMUSG00000015697.15	417.119496	-0.0049051	0.00191067	6.60387168	0.01017573	0.03236675	Setdb1
ENSMUSG00000015702.15	20.801808	0.01835612	0.00473305	14.6730487	0.00012786	0.0007126	Anxa9
ENSMUSG00000015709.10	15.0077629	0.02987735	0.01629383	2.05083743	0.1521225	0.2905467	Arnt2
ENSMUSG00000015711.9	304.31074	0.00484121	0.0021983	4.80474061	0.02838154	0.07598263	Prune1
ENSMUSG00000015714.12	5401.12076	-0.0034025	0.00225897	2.26720134	0.13213833	0.26121991	Cers2
ENSMUSG00000015733.14	1798.24666	0.00925129	0.00298079	9.30817207	0.00228134	0.00904939	Capza2
ENSMUSG00000015745.10	128.196422	0.02406585	0.00493147	21.9064825	2.86E-06	2.27E-05	Plekho1
ENSMUSG00000015747.6	238.933837	0.00026286	0.00456005	0.00325158	0.95452715	1	Vps45
ENSMUSG00000015748.14	218.671408	0.00173755	0.00347791	0.25418242	0.61414538	0.78581865	Prpf3
ENSMUSG00000015749.13	475.551831	0.00635511	0.00324199	3.78916878	0.05158526	0.12369764	Anp32e
ENSMUSG00000015750.16	1248.18559	-0.0019007	0.00221392	0.75491883	0.38492335	0.57657832	Aph1a
ENSMUSG00000015755.17	1426.81478	0.00345785	0.00310241	1.22121767	0.26912182	0.44556417	Tab2
ENSMUSG00000015757.11	394.10961	-0.0020324	0.00238536	0.71626725	0.39737106	0.58916229	Ppil4
ENSMUSG00000015759.12	1409.79025	-0.0140371	0.00303471	21.2071116	4.12E-06	3.17E-05	Cnih1
ENSMUSG00000015766.15	101.133274	0.02981198	0.00547637	27.5000945	1.57E-07	1.58E-06	Eps8
ENSMUSG00000015776.13	461.923117	0.00213619	0.00206327	1.07042799	0.30084879	0.48521743	Med22
ENSMUSG00000015790.17	497.536866	-0.0039065	0.00214341	3.33119714	0.06797738	0.15463056	Surf1
ENSMUSG00000015804.15	596.800667	-0.0003989	0.00425424	0.00880877	0.9252243	0.98916486	Med28
ENSMUSG00000015806.13	7980.1821	-0.0045066	0.00337597	1.76867783	0.18354579	0.33551198	Qdpr
ENSMUSG00000015812.9	4.18565003	-0.0047224	0.0092527	0.30378716	0.5815179	0.75926618	Gnrh1
ENSMUSG00000015829.14	11.4491461	0.0200095	0.01294108	1.85517495	0.17318271	0.3207538	Tnr
ENSMUSG00000015837.16	6809.20998	-0.0015304	0.00221354	0.46683737	0.49444573	0.68186851	Sqstm1
ENSMUSG00000015839.7	1363.19126	0.0114785	0.00238303	22.6101014	1.98E-06	1.63E-05	Nfe2l2
ENSMUSG00000015843.11	100.847951	0.00578918	0.00424487	1.91131905	0.16681681	0.31149879	Rxrg
ENSMUSG00000015846.17	6019.14801	-0.0076448	0.00314207	5.81843573	0.01585904	0.04696778	Rxra
ENSMUSG00000015850.12	73.5513688	0.00569016	0.00646389	0.77579188	0.37843123	0.569603	Adamtsl4
ENSMUSG00000015852.14	2.86245514	0.00392257	0.01489588	0.12283824	0.72597635	0.86776026	Fcrl2
ENSMUSG00000015854.8	2422.45399	0.05753024	0.00637377	74.3895797	6.41E-18	2.57E-16	Cd5l
ENSMUSG00000015869.18	984.291279	-0.0034902	0.00215792	2.62501502	0.10519151	0.21828673	Prpsap1
ENSMUSG00000015879.9	8.06913171	0.02367591	0.00673883	12.0367553	0.00052162	0.00248492	Fam184b
ENSMUSG00000015880.14	23.982021	0.03113144	0.01094154	7.21193116	0.00724205	0.0243182	Ncapg
ENSMUSG00000015882.19	293.987464	0.00319463	0.00337533	0.88023762	0.3481366	0.53618584	Lcorl
ENSMUSG00000015889.9	486.473635	-0.0023509	0.00229248	1.06246556	0.30265352	0.48726184	Lta4h
ENSMUSG00000015890.3	3309.65435	-0.0030768	0.00480388	0.41404553	0.51992327	0.70586025	Amdhd1
ENSMUSG00000015932.9	3493.48512	-0.000424	0.00186081	0.05204254	0.81954647	0.92756274	Dstn
ENSMUSG00000015937.16	1114.8539	-0.0021321	0.00233328	0.82859594	0.36267884	0.55220084	Macroh2a1
ENSMUSG00000015942.10	112.593408	-0.0030027	0.00440315	0.46703283	0.49435538	0.68186351	Gtf2ird2
ENSMUSG00000015943.12	314.769308	-0.0081102	0.00388565	4.37564047	0.03645613	0.0933357	Bola1
ENSMUSG00000015944.10	385.75593	0.00894937	0.00614051	1.96638633	0.16083196	0.30279235	Castor2
ENSMUSG00000015947.11	112.649105	0.05920244	0.00704144	63.8413753	1.35E-15	4.10E-14	Fcgr1
ENSMUSG00000015950.14	138.85319	0.05229964	0.00519604	92.3643749	7.21E-22	4.68E-20	Ncf1
ENSMUSG00000015957.13	1.84639329	0.02069746	0.01383236	2.49962468	0.11387343	0.23237747	Wnt11

ENSMUSG00000015961.9	834.114423	-0.0019668	0.0017495	1.23601562	0.26624033	0.44186809	Adss
ENSMUSG00000015962.6	0.89918785	0.00504836	0.024862	0.08080046	0.77621557	0.90088674	1700016C15Rik
ENSMUSG00000015966.18	165.131419	0.00566843	0.00379424	2.22502886	0.13579012	0.26658447	Il17rb
ENSMUSG00000015968.19	54.1649206	0.01802367	0.00648322	7.33516085	0.00676186	0.02293762	Cacna1d
ENSMUSG00000015970.20	3059.62104	-0.0036709	0.00415278	0.77190267	0.37962909	0.57114148	Chdh
ENSMUSG00000015971.6	451.204614	-0.0045703	0.00147702	9.54492882	0.00200503	0.00810186	Actr8
ENSMUSG00000015980.15	12.9946383	0.02242435	0.00804951	7.22160321	0.00720313	0.02419785	Lrrc27
ENSMUSG00000015981.13	8.2723829	0.05327094	0.00739196	49.4209723	2.07E-12	4.17E-11	Stk32c
ENSMUSG00000015994.11	648.246013	2.56E-05	0.00186043	0.00018949	0.98901698	1	Fnta
ENSMUSG00000016018.5	470.797588	-0.0076017	0.00232873	10.5763134	0.00114546	0.00494904	Mtrex
ENSMUSG00000016024.10	1667.89147	0.01422806	0.00460947	9.2446927	0.00236179	0.00931369	Lbp
ENSMUSG00000016028.12	413.627602	0.0128956	0.00601667	4.57130006	0.03251188	0.08500447	Celsr1
ENSMUSG00000016087.14	102.159418	0.02474436	0.00443882	28.9045116	7.60E-08	8.06E-07	Fli1
ENSMUSG00000016128.15	489.248093	0.00059506	0.00450714	0.0171562	0.89578979	0.97330466	Stard13
ENSMUSG00000016150.16	5.6024908	0.02826428	0.0163219	3.32118523	0.06839251	0.15540517	Tenm1
ENSMUSG00000016181.11	147.393584	-0.0040706	0.0043488	0.85570035	0.35494472	0.54361858	Utp25
ENSMUSG00000016194.15	9694.0198	-0.00327	0.00365536	0.8002126	0.37102981	0.5616526	Hsd11b1
ENSMUSG00000016200.15	7.86923674	0.01773119	0.01566927	1.29534609	0.25506507	0.42766508	Syt14
ENSMUSG00000016206.7	81.7291749	0.0391895	0.00470897	66.1564221	4.17E-16	1.34E-14	H2-M3
ENSMUSG00000016239.12	56.2008124	-0.0161212	0.00687713	5.35190789	0.02069961	0.05864274	Lonrf3
ENSMUSG00000016252.8	6055.47982	-0.0172874	0.00594196	8.526548	0.00350003	0.01304134	Atp5f1e
ENSMUSG00000016253.13	468.334299	-0.0109147	0.00254967	18.5620372	1.64E-05	0.00011192	Nelfcd
ENSMUSG00000016255.6	5.87118896	0.07724022	0.01190806	36.3290067	1.67E-09	2.24E-08	Tubb1
ENSMUSG00000016256.11	3471.81969	0.01312506	0.00273067	22.9724774	1.64E-06	1.37E-05	Ctsz
ENSMUSG00000016257.16	611.114219	-0.0063587	0.00247141	6.674132	0.00978221	0.03129725	Prelid3b
ENSMUSG00000016262.15	19.3429206	-0.0263094	0.00923472	7.11571694	0.0076411	0.02540142	Sertad4
ENSMUSG00000016283.14	15.2329403	0.1594138	0.01124329	158.266256	2.71E-36	1.14E-33	H2-M2
ENSMUSG00000016308.14	506.575653	-0.0073503	0.00296253	6.24847783	0.01243001	0.03824842	Ube2a
ENSMUSG00000016319.4	7225.53764	-0.0036894	0.00217915	2.93518396	0.08666879	0.18788328	Slc25a5
ENSMUSG00000016344.15	916.349053	-0.0129749	0.00358869	13.1760138	0.00028356	0.00145202	Ppdpf
ENSMUSG00000016346.18	8.29929317	0.01520177	0.01683558	0.87045212	0.35083017	0.53927793	Kcnq2
ENSMUSG00000016349.11	92.9433408	-0.0039141	0.01529019	0.03716271	0.84713415	0.94528581	Eef1a2
ENSMUSG00000016356.19	24.8271934	0.00041938	0.00722849	0.00263667	0.95904778	1	Col20a1
ENSMUSG00000016382.16	1459.12118	0.00663403	0.0024943	7.04068028	0.00796788	0.02630236	Pls3
ENSMUSG00000016386.16	9.9500595	0.035535	0.01030111	10.5455706	0.00116467	0.00502008	Mpped2
ENSMUSG00000016409.5	1425.14124	0.00205806	0.00490249	0.17121814	0.67903147	0.83440847	Nkap
ENSMUSG00000016427.8	1364.6338	-0.0185388	0.00377009	24.2560854	8.43E-07	7.34E-06	Ndufa1
ENSMUSG00000016458.16	27.7564759	0.06040005	0.00634307	83.5377889	6.25E-20	3.24E-18	Wt1
ENSMUSG00000016477.19	145.806536	-0.0038529	0.00253524	2.30301759	0.12912293	0.25639782	E2f3
ENSMUSG00000016481.16	1014.38924	-0.0037996	0.00216732	3.07893913	0.07931224	0.17492599	Cr1l
ENSMUSG00000016487.16	850.248771	0.00727513	0.00321382	5.09169815	0.02404064	0.06627205	Ppfibp1
ENSMUSG00000016493.14	34.273776	0.00550317	0.00512712	1.09284999	0.2958406	0.47885562	Cd46
ENSMUSG00000016494.10	79.398116	0.03111315	0.0070997	17.9777621	2.24E-05	0.00014778	Cd34
ENSMUSG00000016495.13	122.430274	0.02767905	0.00395144	48.1434217	3.96E-12	7.70E-11	Plgrkt
ENSMUSG00000016496.8	76.8020532	0.06621977	0.00572874	121.428928	3.08E-28	4.75E-26	Cd274
ENSMUSG00000016498.11	2.08347094	0.12954281	0.01364185	80.664154	2.68E-19	1.27E-17	Pdcd1lg2
ENSMUSG00000016503.19	283.293624	0.0046427	0.00314943	2.17388694	0.14037104	0.27342945	Gtf3a
ENSMUSG00000016510.12	503.652958	-0.0079329	0.00272678	8.44927362	0.00365189	0.01351403	Mtif3
ENSMUSG00000016520.8	369.941164	-0.0091821	0.00339479	7.22652686	0.0071834	0.02413804	Lnx2
ENSMUSG00000016526.9	26.298045	0.00959071	0.0053079	3.20700149	0.07332374	0.16442756	Dyrk3
ENSMUSG00000016528.11	1099.50994	6.91E-06	0.00311958	1.38E-06	0.99906381	1	Mapkapk2
ENSMUSG00000016529.6	2.83614043	0.08081029	0.01329926	32.6458263	1.11E-08	1.33E-07	Il10
ENSMUSG00000016534.16	13026.8166	0.00065199	0.00210412	0.09387987	0.75930146	0.88979402	Lamp2
ENSMUSG00000016541.11	1102.49347	0.00010819	0.00187923	0.00329739	0.95420832	1	Atxn10
ENSMUSG00000016552.14	8.43529074	0.03514446	0.00779105	19.3338566	1.10E-05	7.71E-05	Foxred2
ENSMUSG00000016554.10	1168.72747	-0.0059657	0.00225364	6.97805178	0.00825154	0.02711351	Eif3d
ENSMUSG00000016559.15	3261.40394	0.00650733	0.00224083	8.32708091	0.00390584	0.01431372	H3f3b
ENSMUSG00000016619.11	645.526487	0.00021867	0.0019535	0.01244738	0.9111661	0.9815139	Nup50
ENSMUSG00000016624.16	15.9461657	0.0257006	0.00879311	7.48944336	0.00620617	0.02127908	Phf21b
ENSMUSG00000016626.11	2.03168066	-0.0226748	0.05651651	0.24986996	0.61716666	0.78845698	Nlrp14
ENSMUSG00000016637.8	134.808179	0.0033178	0.00306151	1.18431452	0.27647939	0.45509407	Ift27

ENSMUSG00000016664.18	1066.94389	-0.0003547	0.00280683	0.01590131	0.89965246	0.97555069	Pacsin2
ENSMUSG00000016756.18	3225.88194	-0.02854	0.00551013	26.013161	3.39E-07	3.20E-06	Cmah
ENSMUSG00000016757.12	186.607354	-0.0016758	0.00333255	0.25148238	0.61603322	0.78743548	Ttll12
ENSMUSG00000016758.5	69.3227699	-0.0255348	0.00479771	29.4652842	5.69E-08	6.13E-07	Bik
ENSMUSG00000016763.16	17.348042	0.08444935	0.00978696	63.1988699	1.87E-15	5.56E-14	Scube1
ENSMUSG00000016831.13	694.124785	-0.0030333	0.00175448	2.93200165	0.08683976	0.18815016	Tox4
ENSMUSG00000016833.15	654.384491	0.0017195	0.00409272	0.17236125	0.67802176	0.83381935	Mrps18c
ENSMUSG00000016918.16	52.609502	0.0262963	0.00638389	15.8972562	6.69E-05	0.00039862	Sulf1
ENSMUSG00000016921.15	1899.57657	-0.0054894	0.00184604	8.89109974	0.00286564	0.01098302	Srsf6
ENSMUSG00000016933.18	767.714729	-0.0133849	0.00283572	22.8024281	1.80E-06	1.48E-05	Plcg1
ENSMUSG00000016940.19	354.811988	0.00698426	0.00227733	9.3088651	0.00228048	0.00904749	Kctd2
ENSMUSG00000016942.7	6689.66337	-0.0093739	0.0044832	4.37729409	0.03642077	0.09326027	Tmprss6
ENSMUSG00000016946.7	88.6225265	0.00468262	0.00286108	2.64457187	0.10390415	0.21619346	Kctd5
ENSMUSG00000016984.8	126.590643	-0.000114	0.00364797	0.00096106	0.97526874	1	Etaa1
ENSMUSG00000016995.18	2.90581957	0.01173813	0.0154715	0.71340239	0.39831661	0.59011907	Matn4
ENSMUSG00000017002.15	54.4140228	0.06991713	0.00832146	62.3345688	2.90E-15	8.46E-14	Slpi
ENSMUSG00000017009.4	7300.51852	-0.0160443	0.00368399	18.7905645	1.46E-05	0.00010029	Sdc4
ENSMUSG00000017057.10	1023.70866	0.01169815	0.0033492	11.8014261	0.00059185	0.00277083	Il13ra1
ENSMUSG00000017119.21	3907.89313	-0.0117119	0.00346116	11.4981929	0.00069664	0.00319854	Nbr1
ENSMUSG00000017132.19	714.191937	0.0063702	0.00295376	4.63878397	0.03125714	0.08228024	Cyth1
ENSMUSG00000017144.9	427.995191	0.00394599	0.00298151	1.66385984	0.19708298	0.35410979	Rnd3
ENSMUSG00000017146.13	71.6308741	0.034898	0.00681648	25.4258855	4.60E-07	4.21E-06	Brca1
ENSMUSG00000017167.7	48.1556534	0.03563831	0.00741793	22.1018159	2.59E-06	2.07E-05	Cntnap1
ENSMUSG00000017176.18	52.9346924	0.01674199	0.00565665	8.57629201	0.00340568	0.0127372	Nt5c3b
ENSMUSG00000017188.4	1063.03273	-0.0117843	0.00323082	13.2248668	0.00027626	0.00141927	Coa3
ENSMUSG00000017195.16	2.92231583	-0.032635	0.01046178	8.95510356	0.00276696	0.01066189	Zpbp2
ENSMUSG00000017210.15	257.610523	-0.0078711	0.00310513	6.38621539	0.011501	0.03582916	Med24
ENSMUSG00000017221.15	1544.95126	-0.0080551	0.00182906	19.3864036	1.07E-05	7.53E-05	Psmd3
ENSMUSG00000017264.17	378.531117	-0.0031152	0.00247179	1.57503681	0.20947705	0.37112039	Exosc10
ENSMUSG00000017286.16	721.284082	-0.0081237	0.00341002	5.60663685	0.01789257	0.05193382	Glod4
ENSMUSG00000017288.16	489.771407	-0.0022522	0.00184046	1.49597426	0.22129183	0.38644966	Vps53
ENSMUSG00000017291.14	1047.42396	0.00304286	0.00284393	1.12369467	0.28912428	0.47033213	Taok1
ENSMUSG00000017299.14	196.694531	-0.0005404	0.00229415	0.05533668	0.8140244	0.92390065	Dnttip1
ENSMUSG00000017300.10	2.46586974	0.00569687	0.03253516	-0.0370922	1	1	Tnnc2
ENSMUSG00000017307.17	253.2904	-0.0174621	0.00314951	30.5876722	3.19E-08	3.57E-07	Acot8
ENSMUSG00000017309.12	305.638248	-0.000488	0.00327523	0.02202155	0.88202978	0.9650389	Cd300lg
ENSMUSG00000017314.13	12.9234233	0.02573578	0.00922049	7.51177205	0.0061297	0.02104749	Mpp2
ENSMUSG00000017344.5	34331.1025	-0.0041337	0.00446736	0.85792743	0.35431934	0.54294282	Vtn
ENSMUSG00000017376.16	251.23046	-0.0089598	0.00375183	5.65362167	0.01741936	0.05079157	Nlk
ENSMUSG00000017386.11	656.769224	-0.0042704	0.00301859	1.98426423	0.1589419	0.30007233	Traf4
ENSMUSG00000017390.16	280.121314	0.00382385	0.00477077	0.64145826	0.42318323	0.61490881	Aldoc
ENSMUSG00000017400.11	5.66576593	0.07610767	0.01170958	38.7048839	4.93E-10	7.14E-09	Stac2
ENSMUSG00000017404.13	7250.68016	0.00116865	0.00388071	0.09125047	0.76259342	0.89200122	Rpl19
ENSMUSG00000017405.15	98.1300037	0.00162547	0.00369988	0.19141461	0.66174185	0.82228772	Nek8
ENSMUSG00000017412.17	29.1040801	0.00850382	0.00927747	0.70477929	0.40118243	0.59286488	Cacnb4
ENSMUSG00000017417.15	20.3355159	0.02089355	0.00555765	13.3373418	0.00026017	0.00134483	Plxdc1
ENSMUSG00000017418.15	605.616596	-0.0040205	0.00323401	1.57733229	0.20914537	0.37065507	Arl5b
ENSMUSG00000017421.19	1423.59013	0.00545763	0.00166165	10.7618838	0.00103612	0.00453646	Zfp207
ENSMUSG00000017428.17	2063.27568	-0.0067026	0.00292657	5.17504892	0.02291353	0.06372343	Psmd11
ENSMUSG00000017446.15	209.931519	0.00275712	0.00487008	0.31897929	0.5722217	0.7514711	C1qtnf1
ENSMUSG00000017453.5	4217.57134	-0.0059715	0.00438958	1.85498232	0.17320503	0.32076114	Pipox
ENSMUSG00000017466.11	452.264803	0.0318794	0.00483809	41.6957511	1.07E-10	1.70E-09	Timp2
ENSMUSG00000017478.16	798.645346	-0.0036765	0.00242772	2.27517	0.13146071	0.26016381	Zc3h18
ENSMUSG00000017485.12	1101.19874	0.00095905	0.00334918	0.08013129	0.77711958	0.90149234	Top2b
ENSMUSG00000017491.10	73.3417437	0.02231162	0.00428744	26.4425259	2.71E-07	2.60E-06	Rarb
ENSMUSG00000017493.13	21980.1642	-0.005964	0.00316979	3.51953826	0.06064934	0.14120917	Igfbp4
ENSMUSG00000017499.16	11.0916136	0.0445527	0.01050005	16.5383312	4.77E-05	0.00029429	Cdc6
ENSMUSG00000017548.16	493.305802	0.00137428	0.00218312	0.39111462	0.53171409	0.71630927	Suz12
ENSMUSG00000017550.15	46.0686357	0.01610301	0.00520844	9.04878122	0.0026287	0.01020921	Atad5
ENSMUSG00000017561.17	186.955507	0.00901037	0.00256336	12.1173111	0.00049956	0.00239386	Crlf3
ENSMUSG00000017588.3	0.18643636	0.0052074	0.10535729	0.01230266	0.9116819	0.98182408	Krt27

ENSMUSG00000017607.10	15.4920513	0.03158817	0.00862619	12.5186086	0.00040292	0.00197634	Tns4
ENSMUSG00000017615.13	743.973906	0.00089634	0.00253428	0.12391206	0.72482968	0.86701657	Tnfaip1
ENSMUSG00000017631.20	172.267898	0.05038659	0.00485154	101.871692	5.92E-24	4.90E-22	Abr
ENSMUSG00000017639.14	162.472372	0.00541266	0.00608007	0.77267301	0.3793914	0.57086607	Rab11fip4
ENSMUSG00000017652.17	26.8945215	0.06133728	0.00816942	46.7092531	8.23E-12	1.54E-10	Cd40
ENSMUSG00000017664.17	357.160606	0.00591348	0.00294167	4.05524167	0.04403442	0.1087814	Slc35c2
ENSMUSG00000017667.10	44.3390855	0.00633607	0.00385896	2.67594363	0.10187493	0.21328085	Zfp334
ENSMUSG00000017670.17	210.05288	0.00985791	0.00316817	9.56022155	0.00198839	0.00804291	Elmo2
ENSMUSG00000017677.12	779.885519	-0.005987	0.00431557	1.89987661	0.16809213	0.31339916	Wsb1
ENSMUSG00000017679.17	347.428535	-0.0100122	0.00313657	9.96487049	0.00159555	0.00661733	Ttpal
ENSMUSG00000017686.18	739.604054	-0.0069885	0.00161427	18.7927415	1.46E-05	0.0001002	Rhot1
ENSMUSG00000017688.16	6.39992991	0.01015708	0.00848838	1.44079417	0.23001087	0.39729303	Hnf4g
ENSMUSG00000017692.9	8.68970351	0.02703485	0.00839149	9.71840815	0.00182431	0.00745079	Rhbdl3
ENSMUSG00000017697.4	34.9372754	0.00799257	0.00457828	2.97010363	0.08481642	0.18466538	Ada
ENSMUSG00000017707.10	4368.26812	0.00614556	0.00232843	6.86585818	0.0087858	0.02860207	Serinc3
ENSMUSG00000017713.10	94.6616226	-0.0126251	0.00351672	13.1055876	0.00029442	0.00150013	Tha1
ENSMUSG00000017715.12	204.848753	0.00347545	0.00240497	2.09594315	0.14769055	0.28412963	Pgs1
ENSMUSG00000017716.16	46.0001025	0.01866595	0.00962403	3.34336125	0.06747664	0.15370909	Birc5
ENSMUSG00000017718.16	1938.52564	-0.0067519	0.00372642	3.23283421	0.07217568	0.16231269	Afmid
ENSMUSG00000017721.16	637.265301	-0.0062479	0.00314934	3.904713	0.04815085	0.11696045	Pigt
ENSMUSG00000017723.12	103.983903	0.04122763	0.00626327	38.4945885	5.49E-10	7.90E-09	Wfdc2
ENSMUSG00000017724.15	4.8570856	-0.0014804	0.01134505	0.01407993	0.90554562	0.97866278	Etv4
ENSMUSG00000017734.16	111.70532	0.00353787	0.00524406	0.46264464	0.49639058	0.6835514	Dbndd2
ENSMUSG00000017737.3	46.4298485	0.0540544	0.00997977	21.4368992	3.66E-06	2.85E-05	Mmp9
ENSMUSG00000017740.18	62.2941396	-0.0014789	0.00624481	0.05413588	0.81601675	0.92522695	Slc12a5
ENSMUSG00000017747.14	347.413609	-0.0056092	0.0041477	1.79480438	0.18034189	0.33101509	Ghdc
ENSMUSG00000017754.14	1698.34295	0.03563625	0.00482432	47.5700375	5.31E-12	1.02E-10	Pltp
ENSMUSG00000017756.10	1473.9735	-0.0142934	0.00349087	16.6787244	4.43E-05	0.0002751	Slc12a7
ENSMUSG00000017760.17	2446.89683	0.01202807	0.0021337	31.7032851	1.80E-08	2.09E-07	Ctsa
ENSMUSG00000017764.3	68.083757	-0.0041783	0.00285814	2.11652178	0.14571714	0.28131985	Zswim1
ENSMUSG00000017765.18	324.81918	0.00110954	0.00246574	0.20407925	0.65144815	0.81397256	Slc12a4
ENSMUSG00000017774.20	778.205672	0.01137501	0.00300884	13.9853024	0.00018425	0.00099215	Myo1c
ENSMUSG00000017776.16	1715.24051	-0.0009744	0.0019857	0.23908025	0.6248712	0.79382808	Crk
ENSMUSG00000017778.15	5635.11416	-0.0157537	0.00423382	13.9309496	0.00018965	0.00101846	Cox7c
ENSMUSG00000017781.19	1047.20609	0.00776662	0.0024917	9.54446703	0.00200553	0.00810251	Pitpna
ENSMUSG00000017801.16	759.302297	-0.0035837	0.00249966	2.04899181	0.15230702	0.29083021	Mlx
ENSMUSG00000017802.15	1408.68647	-0.0095436	0.00186751	25.9488463	3.51E-07	3.29E-06	Retreg3
ENSMUSG00000017817.12	43.2112176	-0.0099203	0.01143904	0.69020688	0.40609402	0.59769944	Jph2
ENSMUSG00000017830.16	211.84067	0.01559603	0.00399522	15.4517298	8.46E-05	0.00049206	Dhx58
ENSMUSG00000017831.9	1113.91758	-0.0029247	0.00195997	2.19234803	0.13869774	0.27096205	Rab5a
ENSMUSG00000017832.3	2.17541416	-0.0187351	0.01873078	0.94355292	0.33136579	0.51837856	Hspb9
ENSMUSG00000017837.13	312.490761	0.00505266	0.00262904	3.70527159	0.05424084	0.12904398	Nkiras2
ENSMUSG00000017843.15	1061.30276	0.00076841	0.00191876	0.15840363	0.69063053	0.8431396	Ppp2r5c
ENSMUSG00000017858.13	473.883992	-0.0019032	0.00226884	0.69932136	0.40301182	0.5946243	Ift52
ENSMUSG00000017861.12	12.3505288	0.03608251	0.00928973	13.7853177	0.00020493	0.00108937	Mybl2
ENSMUSG00000017868.17	1033.43659	-0.0003507	0.00464659	0.00544655	0.94116893	0.9975917	Sgk2
ENSMUSG00000017897.19	3.1619662	0.03978873	0.01187102	10.4004964	0.00125981	0.00538529	Eya2
ENSMUSG00000017929.14	651.269288	0.02187443	0.00411842	27.3318381	1.71E-07	1.71E-06	B4galt5
ENSMUSG00000017943.16	1.2476384	0.01758572	0.02421803	0.66577982	0.41452683	0.6059141	Gdap1l1
ENSMUSG00000017950.17	9172.90273	-0.0067922	0.00493155	1.86804755	0.17169886	0.31869752	Hnf4a
ENSMUSG00000017969.14	36.5043536	0.00805013	0.00525824	2.30538533	0.1289263	0.25613681	Ptgis
ENSMUSG00000017978.20	304.822245	0.00012474	0.00340169	0.0013314	0.97089295	1	Cadps2
ENSMUSG00000017999.17	435.537615	-0.0052192	0.00275073	3.57176331	0.05876987	0.13756166	Ddx27
ENSMUSG00000018001.19	169.938788	0.01776483	0.00468172	13.5330329	0.0002344	0.00122694	Cyth3
ENSMUSG00000018008.9	197.552901	0.06194174	0.0057796	102.552006	4.20E-24	3.54E-22	Cyth4
ENSMUSG00000018012.12	6.7700585	0.014584	0.00858398	2.58229059	0.10806518	0.22307241	Rac3
ENSMUSG00000018040.10	423.110358	8.78E-05	0.00323691	0.00074278	0.97825719	1	Rrp7a
ENSMUSG00000018042.19	9848.52993	-0.0106862	0.00351689	9.19278235	0.00242971	0.00953848	Cyb5r3
ENSMUSG00000018068.14	133.022304	-0.0035677	0.00415121	0.73995669	0.38967475	0.58118558	Ints2
ENSMUSG00000018076.13	355.278813	0.00091303	0.00386234	0.05518116	0.81428115	0.9240142	Med13l
ENSMUSG00000018102.6	2859.72683	-0.0155757	0.00295137	26.8909475	2.15E-07	2.11E-06	H2bc4

ENSMUSG00000018126.16	4.10552844	0.02911773	0.01894283	2.74269413	0.09769992	0.20642752	Baiap2l2
ENSMUSG00000018143.11	274.159401	0.00804015	0.0028008	8.09197088	0.00444618	0.01600299	Mafk
ENSMUSG00000018160.16	588.600427	-0.0041596	0.00229362	3.2576643	0.07109032	0.16020794	Med1
ENSMUSG00000018166.9	2172.04301	-0.0048216	0.00419062	1.30566682	0.2531807	0.42541115	Erbb3
ENSMUSG00000018167.11	265.369781	-0.0018477	0.00191477	0.93205143	0.33433043	0.52125314	Stard3
ENSMUSG00000018168.9	29.8771873	0.10050868	0.00759405	137.602518	8.90E-32	2.39E-29	Ikzf3
ENSMUSG00000018169.13	27.9627848	0.02240738	0.00460475	22.6328062	1.96E-06	1.61E-05	Mfng
ENSMUSG00000018171.10	2837.57437	-0.009578	0.0027094	12.31184	0.00045009	0.00217628	Vmp1
ENSMUSG00000018189.13	321.668431	-0.0056396	0.00218597	6.62889385	0.01003376	0.03198488	Uchl5
ENSMUSG00000018196.19	467.540651	-0.0057244	0.0027981	4.21386007	0.040095	0.10081883	Glrx2
ENSMUSG00000018199.10	147.505009	0.02366292	0.00318901	52.7857181	3.72E-13	8.29E-12	Ro60
ENSMUSG00000018209.16	394.047132	0.0043024	0.00278547	2.34357498	0.12580055	0.25132406	Stk4
ENSMUSG00000018211.14	17.1978961	0.11749066	0.01280107	69.8023783	6.56E-17	2.33E-15	Wfdc15b
ENSMUSG00000018217.13	138.261672	0.02514074	0.00514985	22.7372407	1.86E-06	1.53E-05	Pmp22
ENSMUSG00000018238.7	7.28980813	-0.0293086	0.00889652	10.4100567	0.00125331	0.00536197	Gdf9
ENSMUSG00000018239.10	75.5270368	-0.0057822	0.00424594	1.82477627	0.17674579	0.32578355	Zcchc10
ENSMUSG00000018263.12	9.86656221	-0.0124307	0.02554334	0.20333821	0.65203973	0.81413494	Tbx5
ENSMUSG00000018286.7	2271.93189	-0.0096967	0.00302403	10.3515519	0.00129366	0.00550365	Psmb6
ENSMUSG00000018287.13	500.362057	-0.0035565	0.00237407	2.24241217	0.13427137	0.2641966	Spag7
ENSMUSG00000018293.5	3051.18296	0.00731238	0.0031268	5.46791719	0.0193687	0.05542709	Pfn1
ENSMUSG00000018322.11	215.721678	0.00559119	0.00276049	4.0489345	0.04419923	0.1090328	Tomm34
ENSMUSG00000018326.10	2064.48064	0.00610847	0.00209561	8.44710643	0.00365625	0.0135263	Ywhab
ENSMUSG00000018334.19	51.314915	0.02123915	0.00521813	16.01285	6.29E-05	0.00037773	Ksr1
ENSMUSG00000018339.13	1033.28944	0.05358283	0.00633287	70.39372	4.86E-17	1.77E-15	Gpx3
ENSMUSG00000018340.14	1932.25302	0.00424125	0.00226115	3.55535369	0.05935366	0.13867413	Anxa6
ENSMUSG00000018341.13	3.21351497	0.02853597	0.00904437	9.97510426	0.00158671	0.00658296	Il12rb2
ENSMUSG00000018347.17	78.1492754	0.00866851	0.00230941	13.9730237	0.00018545	0.00099842	Zkscan6
ENSMUSG00000018362.15	901.649697	-0.0106023	0.0027262	15.2169705	9.58E-05	0.00055012	Kpna2
ENSMUSG00000018363.18	855.347874	-0.0039837	0.00266748	2.16996048	0.14072985	0.273919	Smurf2
ENSMUSG00000018372.14	104.91477	8.06E-05	0.00410878	0.00037622	0.98452488	1	Cep95
ENSMUSG00000018377.11	351.032176	0.00273303	0.00216499	1.56290575	0.21124027	0.37340276	Vezf1
ENSMUSG00000018378.14	62.2197598	0.00599441	0.0054277	1.21260951	0.27081593	0.4477404	Cuedc1
ENSMUSG00000018379.18	1949.36009	-0.0013524	0.00220085	0.37573526	0.53989453	0.72380791	Srsf1
ENSMUSG00000018381.16	118.521995	0.02983135	0.00495598	34.8398781	3.58E-09	4.62E-08	Abi3
ENSMUSG00000018387.13	629.420096	-0.0086387	0.00585948	2.17079849	0.14065318	0.27380703	Shroom1
ENSMUSG00000018395.20	130.776488	0.0076153	0.00430326	3.08030426	0.0792457	0.17481192	Kif3a
ENSMUSG00000018398.19	160.49489	0.02708605	0.00461645	33.8772733	5.87E-09	7.35E-08	Septin8
ENSMUSG00000018401.18	1001.49883	-0.0040617	0.00296747	1.82577172	0.17662779	0.3256933	Mtmr4
ENSMUSG00000018405.8	72.2094401	-0.0113146	0.00558684	3.88133392	0.04882575	0.11828309	Mrm1
ENSMUSG00000018411.18	164.831612	0.02842827	0.0051008	28.8778651	7.71E-08	8.16E-07	Mapt
ENSMUSG00000018412.17	489.647265	-0.0017711	0.00261597	0.45398317	0.50044945	0.68749495	Kansl1
ENSMUSG00000018415.15	631.579818	-0.0118074	0.00214568	29.692423	5.06E-08	5.49E-07	Gid4
ENSMUSG00000018417.15	3554.43851	-0.0113225	0.00392113	8.36600629	0.00382305	0.01405618	Myo1b
ENSMUSG00000018425.19	896.871353	-0.0019016	0.00329143	0.32282202	0.56991663	0.74962474	Dhx40
ENSMUSG00000018427.9	451.566527	-0.0017666	0.00495677	0.12205189	0.72681961	0.86815156	Ypel2
ENSMUSG00000018428.16	1948.55464	-0.0082229	0.00234725	11.9794048	0.00053792	0.00255432	Akap1
ENSMUSG00000018433.15	264.898185	-0.0008384	0.00238757	0.12284276	0.72597151	0.86776026	Nol11
ENSMUSG00000018442.14	2196.29575	-0.0083033	0.00269955	9.29466631	0.00229822	0.00909799	Derl2
ENSMUSG00000018446.9	1125.35854	-0.007903	0.0035791	4.92382769	0.02648877	0.07180371	C1qbp
ENSMUSG00000018449.13	397.503153	-0.0009162	0.00363716	0.06459212	0.79937983	0.91551776	Rpain
ENSMUSG00000018451.8	14.7283455	0.03649391	0.01350383	7.17200521	0.007405	0.02474206	6330403K07Rik
ENSMUSG00000018459.16	358.441107	0.03171347	0.00543407	32.710211	1.07E-08	1.28E-07	Slc13a3
ENSMUSG00000018470.9	11.9411735	0.0043756	0.00718079	0.38131482	0.5369004	0.72126975	Kcnab3
ENSMUSG00000018474.19	1853.16752	-0.0181124	0.00402606	20.0707486	7.46E-06	5.43E-05	Chd3
ENSMUSG00000018476.8	349.553089	-0.005866	0.00436293	1.74145933	0.18695403	0.34012916	Kdm6b
ENSMUSG00000018481.10	580.146362	0.00040708	0.00185896	0.04726797	0.8278873	0.93249935	Appbp2
ENSMUSG00000018486.3	20.3564238	-0.0017578	0.00449688	0.15628669	0.69259854	0.84453906	Wnt9b
ENSMUSG00000018500.3	1.70209542	0.08871672	0.01461365	36.2420821	1.74E-09	2.34E-08	Adora2b
ENSMUSG00000018501.18	5221.13632	-0.0031833	0.00420774	0.56616399	0.45178728	0.6435874	Ncor1
ENSMUSG00000018507.17	42.571708	0.05555409	0.0051144	110.532399	7.49E-26	7.89E-24	Trpv2
ENSMUSG00000018509.9	219.118555	-0.0022279	0.0032446	0.47775129	0.48944266	0.67737107	Cenpv

ENSMUSG00000018537.17	70.8113396	-0.0182978	0.00568663	10.6664884	0.00109094	0.00473945	Pcgf2
ENSMUSG00000018541.11	247.086327	-0.005716	0.00298149	3.62601528	0.05688286	0.13408345	Cwc25
ENSMUSG00000018547.9	373.806682	0.00758739	0.00289114	6.82527827	0.00898766	0.02917463	Pip4k2b
ENSMUSG00000018548.16	265.676965	-0.0026272	0.00364591	0.51312982	0.47378711	0.66449086	Trim37
ENSMUSG00000018554.14	10.1098024	-0.034028	0.01449162	5.04249823	0.0247328	0.06783113	Ybx2
ENSMUSG00000018559.17	486.72641	0.0015141	0.00348196	0.19173088	0.66147991	0.82217881	Ctdnep1
ENSMUSG00000018565.18	308.275169	-0.0089019	0.00297741	8.99452188	0.0027079	0.01047541	Elp5
ENSMUSG00000018566.15	64.803694	0.0352636	0.01049065	9.74867093	0.00179452	0.00734183	Slc2a4
ENSMUSG00000018567.15	3298.68887	-0.0024888	0.00279404	0.79409057	0.37286608	0.56364792	Gabarap
ENSMUSG00000018569.13	13.4810445	0.01085906	0.00768326	1.88610436	0.16964197	0.31560585	Cldn7
ENSMUSG00000018570.18	3.79727246	-0.0045624	0.01129355	0.17598895	0.67484318	0.83160142	2810408A11Rik
ENSMUSG00000018572.11	320.210712	0.00592826	0.002443	5.83368416	0.01572216	0.0466444	Phf23
ENSMUSG00000018574.15	6546.34717	-0.0198459	0.00324732	37.4368876	9.44E-10	1.31E-08	Acadvl
ENSMUSG00000018581.16	7.00850429	0.01523256	0.01161929	1.46231203	0.22656261	0.39277822	Dnah11
ENSMUSG00000018583.6	1134.57324	0.00114625	0.00161553	0.49948081	0.47972834	0.66947947	G3bp1
ENSMUSG00000018585.10	1463.23096	7.53E-05	0.00407391	0.00034168	0.98525219	1	Atox1
ENSMUSG00000018593.14	1437.50465	-0.0018273	0.00421657	0.19247332	0.66086602	0.82159984	Sparc
ENSMUSG00000018599.6	332.213812	-0.0179727	0.00300645	35.9043173	2.07E-09	2.75E-08	Mief2
ENSMUSG00000018604.19	603.015695	-0.009637	0.0050885	3.40571745	0.06497086	0.14919702	Tbx3
ENSMUSG00000018623.10	13.2774688	0.1162518	0.01352052	62.578909	2.56E-15	7.52E-14	Mmp7
ENSMUSG00000018648.16	20.0345663	0.01185926	0.00811332	2.18678383	0.13919971	0.27156032	Dusp14
ENSMUSG00000018651.15	94.7597317	0.00156542	0.00317063	0.24297177	0.62206793	0.79205864	Tada2a
ENSMUSG00000018654.19	104.265312	0.0693731	0.00607721	113.621389	1.58E-26	1.84E-24	Ikzf1
ENSMUSG00000018659.13	2588.7807	-0.0010183	0.0028201	0.12800257	0.7205121	0.86368547	Pnpo
ENSMUSG00000018661.18	288.970352	-0.0015069	0.00209592	0.51672374	0.4722426	0.66295542	Cog1
ENSMUSG00000018666.14	592.902218	-0.0061497	0.0026459	5.33124643	0.0209464	0.0591596	Cbx1
ENSMUSG00000018669.15	1006.82024	-0.0004294	0.0026058	0.02693781	0.86963085	0.95837696	Cdk5rap3
ENSMUSG00000018672.16	294.834706	0.00576449	0.00299086	3.6884311	0.05479113	0.13011515	Copz2
ENSMUSG00000018677.10	5864.37884	-0.0022712	0.00283719	0.64181714	0.42305355	0.61475824	Slc25a39
ENSMUSG00000018678.14	86.3607501	0.00650659	0.00346164	3.4793413	0.06213947	0.14384351	Sp2
ENSMUSG00000018697.15	235.977982	0.00212506	0.00210781	1.01089459	0.31468862	0.50011763	Aatf
ENSMUSG00000018698.16	1.82935626	0.00255359	0.02089284	0.02522629	0.87380449	0.96081655	Lhx1
ENSMUSG00000018707.14	2323.13074	-0.0044469	0.00335049	1.73452159	0.18783448	0.34131464	Dync1h1
ENSMUSG00000018727.20	68.0751409	0.00127944	0.00592727	0.04507968	0.83185758	0.93527472	Cpsf4l
ENSMUSG00000018733.11	237.56953	-0.0044704	0.00328063	1.82136578	0.17715079	0.32642801	Pex12
ENSMUSG00000018736.15	591.645107	0.00700567	0.00272995	6.61408021	0.01011756	0.03220415	Ndel1
ENSMUSG00000018740.15	26.3989208	-0.0048939	0.00524414	0.85878447	0.35407907	0.54271569	Slc25a35
ENSMUSG00000018750.15	352.314752	0.01617842	0.00343763	21.2360431	4.06E-06	3.13E-05	Zbtb4
ENSMUSG00000018752.20	70.4078012	0.01560444	0.00511592	8.91474736	0.00282877	0.01086989	Tnfsfm13
ENSMUSG00000018761.15	857.373868	-0.0003974	0.0029468	0.01805227	0.8931189	0.97213081	Mpdu1
ENSMUSG00000018765.7	577.921176	-0.0084367	0.00254527	10.8596283	0.00098283	0.00432564	Fxr2
ENSMUSG00000018770.10	6374.16969	-0.0193137	0.00374199	26.9163814	2.12E-07	2.08E-06	Atp5mc3
ENSMUSG00000018774.14	273.581871	0.05746757	0.00533566	108.838993	1.76E-25	1.76E-23	Cd68
ENSMUSG00000018796.14	18249.3759	-0.0265555	0.00378771	46.1842428	1.08E-11	1.98E-10	Acsl1
ENSMUSG00000018800.15	140.183391	0.01908299	0.0040299	22.2872494	2.35E-06	1.89E-05	Abca5
ENSMUSG00000018809.3	59.1687027	0.01315179	0.00348318	14.0360698	0.00017934	0.00097015	Smyd4
ENSMUSG00000018819.11	128.132474	0.07120502	0.00646178	105.085975	1.17E-24	1.05E-22	Lsp1
ENSMUSG00000018820.14	323.316579	-0.001047	0.00206897	0.25782898	0.61161561	0.78377909	Zfyve27
ENSMUSG00000018821.4	822.610459	-0.0071375	0.00408961	3.12546008	0.07707811	0.17111281	Avpi1
ENSMUSG00000018822.8	5.06452487	0.02349477	0.01070212	4.78271565	0.02874675	0.07675129	Sfrp5
ENSMUSG00000018830.11	91.9890495	0.00177992	0.00607268	0.08145966	0.77532898	0.90036438	Myh11
ENSMUSG00000018841.18	210.956227	-0.0046004	0.00278909	2.67354951	0.10202825	0.21348813	Rad51d
ENSMUSG00000018845.15	86.5395109	-0.0201857	0.00679064	8.45867426	0.00363307	0.01344993	Unc45b
ENSMUSG00000018846.9	1372.72436	0.00353073	0.0028933	1.44729986	0.22896171	0.39591575	Pank3
ENSMUSG00000018848.5	563.663191	0.0023195	0.00231043	1.00533125	0.31602393	0.50156385	Rars
ENSMUSG00000018849.7	1194.73039	0.00252787	0.0040891	0.37993318	0.537639	0.72180974	Wwc1
ENSMUSG00000018858.16	641.00008	-0.0095478	0.00304608	9.98535693	0.0015779	0.00655333	Mrpl58
ENSMUSG00000018861.9	313.811296	-0.0075812	0.00403781	3.49293696	0.06163115	0.14297555	Fdxr
ENSMUSG00000018865.10	29.9352451	0.02452481	0.01290872	3.57174839	0.0587704	0.13756166	Sult4a1
ENSMUSG00000018868.5	37.6507802	-0.0081458	0.01172631	0.42021552	0.51682952	0.70319822	Pnpla5
ENSMUSG00000018882.13	449.741303	-0.0070049	0.00197705	12.5347335	0.00039946	0.00196241	Mrpl45

ENSMUSG00000018893.16	837.887778	-0.1123283	0.03064572	0.99528671	0.31845368	0.50423258	Mb
ENSMUSG00000018899.18	654.9529	0.0242785	0.00325776	53.7579067	2.27E-13	5.20E-12	Irf1
ENSMUSG00000018900.8	496.244513	-0.0071149	0.00397582	3.03304007	0.08158502	0.17895139	Slc22a5
ENSMUSG00000018906.15	111.788461	-0.0079498	0.00467654	2.75862213	0.09673152	0.2048364	P4ha2
ENSMUSG00000018907.11	1.02786047	0.04990203	0.01396105	13.965087	0.00018624	0.00100196	Alox12e
ENSMUSG00000018909.16	313.422145	0.01601568	0.00413318	14.5846526	0.000134	0.00074314	Arrb1
ENSMUSG00000018919.11	2.1697918	0.02631346	0.01393696	3.04591265	0.08094061	0.17778592	Tm4sf5
ENSMUSG00000018920.12	276.681107	0.05021464	0.00446836	118.684118	1.23E-27	1.69E-25	Cxcl16
ENSMUSG00000018921.12	330.140773	-0.005299	0.00374277	1.99031255	0.1583082	0.29921126	Pelp1
ENSMUSG00000018923.14	181.702969	0.00557488	0.00335418	2.74237395	0.0977195	0.20643189	Med11
ENSMUSG00000018924.7	7.30698484	0.02408846	0.01076593	4.22599651	0.03980924	0.10021241	Alox15
ENSMUSG00000018925.4	0.98218587	0.09038525	0.01668798	31.1664833	2.37E-08	2.71E-07	Heatr9
ENSMUSG00000018927.4	368.50455	0.05934778	0.0063391	77.386269	1.41E-18	6.08E-17	Ccl6
ENSMUSG00000018930.4	9.95940854	0.12780004	0.01073806	122.527451	1.77E-28	2.76E-26	Ccl4
ENSMUSG00000018931.4	127.569757	-0.0004774	0.00260792	0.03333704	0.85512418	0.95051411	Natd1
ENSMUSG00000018932.10	1139.39993	-0.0082379	0.0032412	6.43098429	0.01121464	0.03513196	Map2k3
ENSMUSG00000018965.12	1028.71341	0.01481339	0.00264477	30.6158401	3.15E-08	3.52E-07	Ywhah
ENSMUSG00000018974.8	547.094842	-0.0049942	0.00428585	1.34516255	0.24612559	0.41727416	Sart3
ENSMUSG00000018983.10	36.872784	0.02365817	0.00635908	12.7525376	0.00035553	0.0017703	E2f2
ENSMUSG00000018986.11	4.63397928	0.02741029	0.01056394	6.23204551	0.01254587	0.0385095	Slfn3
ENSMUSG00000018995.13	417.41203	-0.0178555	0.00310386	33.8638277	5.91E-09	7.39E-08	Nars2
ENSMUSG00000018999.15	457.01997	-0.0018403	0.00320682	0.33217592	0.56438063	0.74482327	Slc35b4
ENSMUSG00000019027.9	14.6446951	0.08532749	0.01162386	47.7199401	4.92E-12	9.45E-11	Dnah1
ENSMUSG00000019039.15	553.753201	-0.013306	0.00323819	16.9956201	3.75E-05	0.00023659	Dalrd3
ENSMUSG00000019054.14	1096.13486	-0.0050002	0.00343245	2.12102679	0.14528911	0.2808383	Fis1
ENSMUSG00000019055.16	852.862491	-0.000583	0.00262467	0.04832467	0.82600455	0.93133299	Plod1
ENSMUSG00000019066.14	223.980967	0.01433071	0.00332652	19.2200236	1.16E-05	8.15E-05	Rab3d
ENSMUSG00000019080.9	183.779211	-0.0141412	0.00573612	5.88206296	0.01529589	0.04560921	Mfsd3
ENSMUSG00000019082.19	3733.54489	-0.0170104	0.00505268	10.7637202	0.00103509	0.00453364	Slc25a22
ENSMUSG00000019087.14	1327.58174	0.00452061	0.00214057	4.41962274	0.03552789	0.09136075	Atp6ap1
ENSMUSG00000019088.15	95.6249855	0.01424389	0.004636	9.21555919	0.00239967	0.00943467	Dnase1l1
ENSMUSG00000019122.9	2405.89662	0.00683884	0.00459152	2.20677383	0.13740576	0.26897271	Ccl9
ENSMUSG00000019124.11	15.2816877	-0.0110022	0.01503419	0.48400439	0.48661407	0.67509437	Scrn1
ENSMUSG00000019132.12	3441.96827	0.00541438	0.00281456	3.64417743	0.05626559	0.13306452	BC005537
ENSMUSG00000019139.11	148.212575	-0.0021171	0.00580983	0.12715813	0.72139697	0.86422537	Isyna1
ENSMUSG00000019143.16	456.783295	-0.0100468	0.00191258	27.68054	1.43E-07	1.45E-06	Hars2
ENSMUSG00000019146.3	6.65499729	0.02150368	0.02145607	1.21815366	0.2697233	0.44630937	Cacng2
ENSMUSG00000019158.10	314.755702	-0.0110832	0.00486175	5.21801549	0.02235405	0.06243251	Tmem160
ENSMUSG00000019173.12	1429.40348	0.0084101	0.00219315	14.6851665	0.00012704	0.00070888	Rab5c
ENSMUSG00000019179.11	2853.79588	-0.0135763	0.00297506	20.9846853	4.63E-06	3.52E-05	Mdh2
ENSMUSG00000019188.17	1827.23939	-0.0080345	0.00233304	11.9026693	0.00056054	0.00264388	H13
ENSMUSG00000019189.14	572.17583	0.02922581	0.00423305	46.9139161	7.42E-12	1.40E-10	Rnf145
ENSMUSG00000019194.16	88.5978522	0.04886831	0.00452625	112.762142	2.43E-26	2.76E-24	Scn1b
ENSMUSG00000019210.11	1550.52202	0.00733967	0.00317202	5.35042356	0.02071724	0.05866692	Atp6v1e1
ENSMUSG00000019214.15	10.3225032	0.01326916	0.01036254	1.60603591	0.20504993	0.36522486	Chtf18
ENSMUSG00000019230.15	4.01150039	0.03608896	0.01520103	5.26255528	0.02178901	0.06104234	Lhx9
ENSMUSG00000019232.15	2318.23238	-0.0294108	0.00657964	20.2069517	6.95E-06	5.11E-05	Etnppl
ENSMUSG00000019235.10	18.1237624	0.00418458	0.00712158	0.33042496	0.56540896	0.74571713	Rps6kl1
ENSMUSG00000019254.17	433.8048	0.00569239	0.00283362	4.03282591	0.04462314	0.10990824	Ppp1r12c
ENSMUSG00000019256.18	760.137594	0.0087184	0.00341869	6.41501134	0.01131596	0.03541173	Ahr
ENSMUSG00000019261.4	110.491791	0.00812333	0.00430758	3.40891611	0.06484503	0.14894163	Map1s
ENSMUSG00000019278.6	31.1966473	0.03006622	0.00685084	18.4778758	1.72E-05	0.00011663	Dpep1
ENSMUSG00000019295.9	359.051593	-0.0085927	0.00146433	34.6563976	3.93E-09	5.05E-08	Tmem129
ENSMUSG00000019297.10	388.632345	-0.0032407	0.00351749	0.86079344	0.35351675	0.54215873	Nop9
ENSMUSG00000019301.6	3.5978394	0.0128051	0.00802797	2.56133481	0.10950609	0.22549421	Hsd17b1
ENSMUSG00000019302.18	745.887263	-0.0017627	0.00247459	0.50325117	0.47807506	0.66812102	Atp6v0a1
ENSMUSG00000019303.15	21.5705352	0.00940333	0.00791803	1.3682507	0.24211266	0.41224872	Psmc3ip
ENSMUSG00000019312.11	1621.70491	-0.0016208	0.00402604	0.16072725	0.68848783	0.84181825	Grb7
ENSMUSG00000019320.15	21.295114	-0.0061415	0.00754918	0.65276157	0.41912707	0.61055717	Noxo1
ENSMUSG00000019326.15	7.83559564	0.01075477	0.01138085	0.85137585	0.35616341	0.54495427	Aoc3
ENSMUSG00000019338.14	219.665588	0.00177019	0.00359421	0.24209352	0.62269813	0.79240346	Zfp687

ENSMUSG00000019359.15	0.9050038	0.01695699	0.03322884	0.55698384	0.45547806	0.64719433	Gdpd2
ENSMUSG00000019362.9	978.72148	-0.0072275	0.00325179	4.97746702	0.02567956	0.07003677	D8Ertd738e
ENSMUSG00000019368.14	3240.68674	-0.0050808	0.00496717	1.04379644	0.30694016	0.49208066	Sec14l4
ENSMUSG00000019370.11	997.725328	0.00788651	0.00272894	8.30319092	0.00395755	0.01448159	Calm3
ENSMUSG00000019373.12	867.104387	-0.0075109	0.00213394	12.390128	0.00043161	0.00209766	Cops3
ENSMUSG00000019428.17	4527.37008	-0.00163	0.00254902	0.40535348	0.52433736	0.70981008	Fkbp8
ENSMUSG00000019432.16	872.687575	-0.0004181	0.00202858	0.04299283	0.83573877	0.93793868	Ddx39b
ENSMUSG00000019433.10	164.179295	0.00050921	0.00322705	0.0251188	0.8740714	0.96088988	Gipc1
ENSMUSG00000019437.18	834.329762	-0.0052821	0.00364569	2.05209223	0.15199719	0.29037791	Tlcd1
ENSMUSG00000019461.13	132.824652	0.00634396	0.00370627	2.97060932	0.08478991	0.18464176	Plscr3
ENSMUSG00000019464.7	13.9789566	0.00886368	0.00892413	0.98029613	0.32212571	0.5083745	Ptger1
ENSMUSG00000019467.16	43.6475124	0.01181246	0.00701864	2.81590435	0.09333428	0.19923816	Arhgef25
ENSMUSG00000019470.13	354.252366	-0.0001787	0.00175218	0.01025954	0.91932076	0.98622302	Xab2
ENSMUSG00000019471.11	1401.75489	-0.0050678	0.00302393	2.7854886	0.09512168	0.20215714	Cdc37
ENSMUSG00000019478.18	358.413392	0.00475324	0.00258946	3.44358246	0.06349787	0.14641497	Rab4a
ENSMUSG00000019487.12	167.442015	-0.0059198	0.0034732	2.86756983	0.09038125	0.1940686	Trip10
ENSMUSG00000019494.15	1414.46782	-0.0126591	0.00267042	22.7391588	1.86E-06	1.53E-05	Cops6
ENSMUSG00000019505.8	7753.71757	0.00074955	0.002585	0.08322976	0.77296733	0.89855725	Ubb
ENSMUSG00000019518.12	232.572796	-0.0024888	0.00285676	0.73646988	0.39079405	0.58215553	Ap4m1
ENSMUSG00000019528.19	125.704202	0.00484226	0.00477745	1.01000836	0.31490084	0.50035375	Gyg
ENSMUSG00000019539.12	118.699041	0.00712676	0.00449174	2.49294261	0.11435773	0.23310358	Rcn3
ENSMUSG00000019558.15	137.466399	0.0129853	0.0052708	6.05654815	0.01385482	0.04198636	Slc6a8
ENSMUSG00000019564.13	34.9759342	0.0144611	0.00533511	6.89735803	0.00863232	0.02819581	Arid3a
ENSMUSG00000019577.7	418.845095	0.00296852	0.00771153	0.12921026	0.71925227	0.86261996	Pdk4
ENSMUSG00000019578.12	929.685958	-0.0081344	0.00296502	7.52461215	0.00608617	0.02091321	Ubxn6
ENSMUSG00000019579.14	1166.9611	-0.0099691	0.00319965	9.76427992	0.00177935	0.00728734	Mydgf
ENSMUSG00000019590.17	157.872029	0.01714927	0.00540523	9.46967677	0.00208896	0.00839357	Cyb561
ENSMUSG00000019647.18	152.276271	-0.0046292	0.00332455	1.93894909	0.16378269	0.30702054	Sema6a
ENSMUSG00000019659.9	278.874335	0.00302712	0.00327462	0.85478987	0.35520083	0.54383038	Ccdc12
ENSMUSG00000019689.5	577.3542	-0.0183046	0.00468136	15.4562847	8.44E-05	0.000491	Fmc1
ENSMUSG00000019699.17	103.375987	0.0279848	0.0049901	30.2168618	3.86E-08	4.26E-07	Akt3
ENSMUSG00000019710.14	1047.60011	-0.0104837	0.00267568	15.4447314	8.50E-05	0.00049365	Mrpl24
ENSMUSG00000019715.15	620.415789	0.00097725	0.00215217	0.20577948	0.65009571	0.81332444	Gle1
ENSMUSG00000019718.9	548.80317	0.00225372	0.00294218	0.58119159	0.44584563	0.63808951	L3hypdh
ENSMUSG00000019726.13	1590.54314	0.00556051	0.00686806	0.64002804	0.42370065	0.61543315	Lyst
ENSMUSG00000019731.14	815.742105	-0.0038462	0.00242645	2.47941265	0.11534532	0.23471096	Slc35e1
ENSMUSG00000019732.15	44.2175196	-0.0183817	0.00440966	16.7003761	4.38E-05	0.00027248	Calr3
ENSMUSG00000019734.17	63.1402571	-0.0057451	0.0055677	1.06720261	0.30157817	0.48595935	Tmc4
ENSMUSG00000019737.15	46.6282653	0.01124155	0.00690317	2.74989382	0.09726089	0.20572104	Syne4
ENSMUSG00000019738.16	418.889475	-0.010902	0.00459653	5.69188921	0.0170435	0.04986651	Polr2i
ENSMUSG00000019761.11	69.6026085	-0.0109533	0.00604712	3.12705879	0.07700255	0.17103137	Krt10
ENSMUSG00000019762.5	1809.7121	0.0044455	0.00456673	0.94881248	0.33002175	0.51678212	Iyd
ENSMUSG00000019763.12	601.184568	-0.0115695	0.00249353	21.4472085	3.64E-06	2.84E-05	Rmnd1
ENSMUSG00000019768.17	555.357525	0.02176102	0.00490836	18.9125945	1.37E-05	9.46E-05	Esr1
ENSMUSG00000019773.8	22.8559058	0.00828216	0.00567368	2.04858015	0.15234821	0.29084283	Fbxo5
ENSMUSG00000019774.9	96.4669281	-0.0103455	0.00369763	7.91375163	0.00490604	0.01739274	Mtrf1l
ENSMUSG00000019775.18	14.9076337	0.05505022	0.0114917	20.4185555	6.22E-06	4.62E-05	Rgs17
ENSMUSG00000019777.17	616.876166	-0.0021245	0.00210921	1.00976985	0.31495798	0.50041085	Hdac2
ENSMUSG00000019779.16	97.5524465	0.01538442	0.00369838	17.5185957	2.85E-05	0.00018395	Frk
ENSMUSG00000019782.11	715.941469	-0.0067607	0.0037785	3.19989838	0.07364285	0.1649934	Rwdd1
ENSMUSG00000019785.15	4.03061367	0.04460979	0.02251415	5.49511937	0.01906963	0.05469313	Clvs2
ENSMUSG00000019787.10	604.525981	-0.0168974	0.02162003	1.02601665	0.31109608	0.49624727	Trdn
ENSMUSG00000019789.10	8.3824808	0.00500494	0.00722673	0.46586264	0.49489673	0.68228452	Hey2
ENSMUSG00000019790.18	303.565011	0.0078389	0.00301013	6.85347971	0.00884688	0.02877707	Stxbp5
ENSMUSG00000019791.13	481.532571	-0.0068159	0.00298544	5.23035908	0.02219595	0.0620571	Hint3
ENSMUSG00000019792.9	128.704867	8.65E-05	0.00217818	0.00159048	0.96818819	1	Trmt11
ENSMUSG00000019794.14	371.716875	-0.0019868	0.00346773	0.32998047	0.56567059	0.74586342	Katna1
ENSMUSG00000019795.18	844.973241	-0.0014032	0.00260057	0.29101696	0.58956958	0.76607871	Pcmt1
ENSMUSG00000019796.13	45.3461729	-0.0001627	0.00552999	0.00077339	0.97781378	1	Lrp11
ENSMUSG00000019797.12	353.723045	-0.0080331	0.0028474	7.98395654	0.00471937	0.01681436	Mtres1
ENSMUSG00000019802.14	3182.19485	-0.0052918	0.00291508	3.30483541	0.06907627	0.15655163	Sec63

ENSMUSG00000019804.13	1237.0938	-0.0009763	0.0022569	0.18489884	0.66719662	0.82619308	Snx3
ENSMUSG00000019806.14	279.769784	0.01139922	0.00310449	13.4360753	0.00024683	0.00128346	Aig1
ENSMUSG00000019808.9	42.411363	-0.0008039	0.00252641	0.10135217	0.75021297	0.8835775	Adat2
ENSMUSG00000019809.17	586.176343	-0.0091794	0.00329801	7.69089096	0.00555002	0.0193243	Pex3
ENSMUSG00000019810.16	277.148484	0.02602255	0.00411312	38.9397224	4.37E-10	6.38E-09	Fuca2
ENSMUSG00000019813.17	82.1842756	0.00821015	0.00367098	4.97447914	0.02572395	0.07013352	Cep57l1
ENSMUSG00000019814.6	278.544218	-0.0026906	0.00228597	1.38135883	0.23986982	0.40951894	Ltv1
ENSMUSG00000019817.19	54.1141676	0.00298998	0.00660299	0.20783394	0.64847047	0.81215377	Plagl1
ENSMUSG00000019818.16	5866.53276	0.00152521	0.00234075	0.42029206	0.51679135	0.70319822	Cd164
ENSMUSG00000019820.12	792.069856	0.00169506	0.00276179	0.36731672	0.54447053	0.7278328	Utrn
ENSMUSG00000019822.13	388.591826	-0.012823	0.00252617	26.5115793	2.62E-07	2.52E-06	Smpd2
ENSMUSG00000019823.18	45.0305024	0.05212384	0.00657607	57.4319373	3.50E-14	8.94E-13	Mical1
ENSMUSG00000019826.12	207.569968	-0.0014395	0.0020044	0.51018938	0.47505689	0.66559889	Zbtb24
ENSMUSG00000019828.14	17.1335061	0.00794987	0.01547743	0.07803366	0.77998003	0.90343314	Grm1
ENSMUSG00000019831.15	37.888686	-0.007083	0.00605626	1.33662566	0.24762992	0.41889114	Wasf1
ENSMUSG00000019832.6	794.032295	0.01620235	0.00353376	20.3270912	6.53E-06	4.82E-05	Rab32
ENSMUSG00000019834.16	1.3281173	-0.0298953	0.07145766	0.21406897	0.64359671	0.80929747	Slc22a16
ENSMUSG00000019837.9	421.691872	-0.007543	0.00309816	5.89482357	0.01518545	0.04532001	Gtf3c6
ENSMUSG00000019838.12	1750.79085	-0.004911	0.00327873	2.21650353	0.13654198	0.26761502	Slc16a10
ENSMUSG00000019841.17	330.119961	0.00098637	0.00302389	0.10883948	0.74146926	0.87757396	Rev3l
ENSMUSG00000019842.7	75.3859741	0.01317371	0.00511629	6.36318829	0.0116512	0.03618715	Traf3ip2
ENSMUSG00000019843.15	225.552781	0.02277526	0.0044742	24.5543894	7.22E-07	6.36E-06	Fyn
ENSMUSG00000019845.9	24.0393751	0.00021561	0.00539169	0.00157401	0.96835319	1	Tube1
ENSMUSG00000019846.12	126.018022	-0.0023198	0.00537462	0.18238293	0.66933333	0.82771622	Lama4
ENSMUSG00000019848.15	5.18675311	0.02093646	0.01335468	2.13438144	0.14402855	0.27897326	Popdc3
ENSMUSG00000019849.12	611.074141	-0.0081786	0.00187937	19.2023825	1.18E-05	8.22E-05	Prep
ENSMUSG00000019850.12	100.181786	0.04588568	0.00631867	46.553422	8.92E-12	1.67E-10	Tnfaip3
ENSMUSG00000019851.9	1921.35897	-0.0083633	0.00252429	10.9405036	0.00094085	0.00416103	Perp
ENSMUSG00000019852.8	13.5196971	0.01782827	0.01109046	2.42895647	0.11911217	0.24090032	Arfgef3
ENSMUSG00000019853.7	15.482788	-0.010415	0.00784183	1.84153004	0.17477172	0.32302843	Hebp2
ENSMUSG00000019854.18	276.80789	-0.001497	0.00242334	0.37900622	0.53813557	0.72218865	Reps1
ENSMUSG00000019856.15	22.9580716	0.01763038	0.00753123	4.87547237	0.02724098	0.07343763	Fam184a
ENSMUSG00000019857.8	173.90362	-0.0048026	0.00265873	3.29979847	0.06928838	0.15691174	Asf1a
ENSMUSG00000019861.16	612.198468	-0.0028319	0.00189967	2.20408216	0.1376458	0.26926381	Gopc
ENSMUSG00000019863.8	91.4455899	-0.0110746	0.00350397	10.1734332	0.00142479	0.0059902	Qrsl1
ENSMUSG00000019864.15	426.232047	-0.0244588	0.0024746	98.4130276	3.40E-23	2.56E-21	Rtn4ip1
ENSMUSG00000019866.16	454.078653	0.01659444	0.00310283	28.7509457	8.23E-08	8.67E-07	Crybg1
ENSMUSG00000019868.17	291.52404	-0.0021255	0.00266644	0.63040591	0.42720646	0.61865276	Vta1
ENSMUSG00000019872.14	888.753887	0.01728905	0.00305926	30.93883	2.66E-08	3.02E-07	Smpdl3a
ENSMUSG00000019873.9	1992.69534	0.00340279	0.0019741	2.95957817	0.08537021	0.18556279	Reep3
ENSMUSG00000019874.12	85.3641528	0.05029048	0.00603471	64.0733454	1.20E-15	3.67E-14	Fabp7
ENSMUSG00000019876.16	51.685953	0.05019822	0.00835641	31.3043294	2.21E-08	2.53E-07	Pkib
ENSMUSG00000019877.11	3579.31736	-0.0008401	0.00219941	0.14424062	0.70410113	0.85264308	Serinc1
ENSMUSG00000019878.9	437.756844	-0.0034719	0.00246286	1.98900261	0.1584452	0.2993716	Hsf2
ENSMUSG00000019880.11	249.436047	0.00664621	0.00525589	1.52921711	0.21622998	0.37974686	Rspo3
ENSMUSG00000019883.12	1643.4211	-0.0059	0.00304244	3.67213751	0.05532918	0.13119475	Echdc1
ENSMUSG00000019888.18	2.36861459	0.00565565	0.01726606	0.18768159	0.66485326	0.82459005	Mgat4c
ENSMUSG00000019889.11	1108.70862	-0.0052827	0.00258509	4.17051434	0.04113331	0.10294676	Ptprk
ENSMUSG00000019890.5	1.17465003	0.049104	0.01778673	8.09950286	0.00442774	0.01595366	Nts
ENSMUSG00000019891.16	85.770001	0.00172138	0.00285331	0.36409553	0.54624044	0.72933071	Dcbld1
ENSMUSG00000019892.14	2.88694363	0.01177136	0.0224949	0.55392732	0.45671742	0.64861134	Lrriq1
ENSMUSG00000019894.15	7.62260922	0.01219815	0.01793956	0.49169928	0.48317027	0.67241291	Slc6a15
ENSMUSG00000019897.9	357.837529	0.0004878	0.00336083	0.02081628	0.88528058	0.96699059	Ccdc59
ENSMUSG00000019899.18	60.5137171	0.00024863	0.00657703	0.00136661	0.97051072	1	Lama2
ENSMUSG00000019906.16	256.191127	-0.0106919	0.00433255	6.04049301	0.01398138	0.04231552	Lin7a
ENSMUSG00000019907.11	1322.43787	0.00558869	0.00384965	2.05245071	0.15196141	0.29033307	Ppp1r12a
ENSMUSG00000019913.5	3.40593456	0.00191249	0.02712749	0.03509135	0.85140437	0.94841998	Sim1
ENSMUSG00000019916.15	281.948142	0.00141712	0.00434793	0.10039742	0.75135323	0.88444961	P4ha1
ENSMUSG00000019917.11	357.840787	-0.0024349	0.00264284	0.84327284	0.35846241	0.54732879	Septin10
ENSMUSG00000019920.19	432.554568	0.01274835	0.00344171	13.1252842	0.00029134	0.00148669	Lims1
ENSMUSG00000019923.15	1162.65402	0.00930787	0.00225541	16.8242288	4.10E-05	0.00025743	Zwint

ENSMUSG00000019927.7	169.058763	0.00791008	0.00294938	7.23087947	0.00716601	0.02409373	Ube2d1
ENSMUSG00000019929.17	3625.00284	0.02787628	0.00379682	51.3821587	7.60E-13	1.62E-11	Dcn
ENSMUSG00000019935.17	364.583402	-0.023249	0.0069412	10.0235312	0.00154553	0.00643816	Slc17a8
ENSMUSG00000019942.14	58.6872655	-0.0036197	0.0091791	0.14490952	0.70344826	0.85207121	Cdk1
ENSMUSG00000019943.11	1791.06892	0.01384604	0.0035867	14.4342458	0.00014514	0.00079908	Atp2b1
ENSMUSG00000019944.15	1101.53846	-0.0029284	0.00358028	0.67939511	0.40979509	0.60157012	Rhobtb1
ENSMUSG00000019945.11	35.0923167	0.02162859	0.00555789	15.2759935	9.29E-05	0.00053489	Cabcoco1
ENSMUSG00000019947.11	1290.39153	-0.0193431	0.00563255	11.2172931	0.00081039	0.00365394	Arid5b
ENSMUSG00000019948.5	258.510108	-0.0154421	0.00319125	23.6877955	1.13E-06	9.66E-06	Actr6
ENSMUSG00000019951.11	1066.75086	0.00314887	0.00252547	1.57728048	0.20915285	0.37065507	Bltp3b
ENSMUSG00000019952.16	127.50616	-0.0033688	0.00344532	0.91466078	0.33888079	0.52653771	Poc1b
ENSMUSG00000019960.9	928.77808	0.00966569	0.00383224	6.12351657	0.01333952	0.04060212	Dusp6
ENSMUSG00000019961.18	977.843334	0.01242623	0.00241622	25.8442281	3.70E-07	3.47E-06	Tmpo
ENSMUSG00000019966.19	853.811275	-0.0068285	0.00583517	1.2576536	0.2620957	0.43659787	Kitl
ENSMUSG00000019969.15	613.779994	0.00530748	0.00237491	4.94917149	0.02610317	0.07096164	Psen1
ENSMUSG00000019970.16	509.085518	0.00801288	0.00485312	2.70825029	0.09983043	0.20993229	Sgk1
ENSMUSG00000019971.11	116.603795	0.00523074	0.00564454	0.7947501	0.37266765	0.56344482	Cep290
ENSMUSG00000019975.13	76.6123306	0.01526917	0.00458122	10.9340501	0.00094413	0.00417242	Ikbip
ENSMUSG00000019977.17	1078.1872	-0.0118468	0.00205206	33.5604514	6.91E-09	8.55E-08	Hbs1l
ENSMUSG00000019978.17	392.659569	0.01445674	0.00373231	14.5312004	0.00013786	0.00076201	Epb41l2
ENSMUSG00000019979.13	89.541906	0.03804003	0.00608357	38.0228925	6.99E-10	9.92E-09	Apaf1
ENSMUSG00000019982.16	4.76710898	0.08024869	0.0122325	33.4446478	7.33E-09	9.05E-08	Myb
ENSMUSG00000019984.17	199.561264	0.00600924	0.0029041	4.1991602	0.040444	0.10154484	Med23
ENSMUSG00000019986.18	47.4331067	0.03979212	0.00510182	55.938662	7.48E-14	1.81E-12	Ahi1
ENSMUSG00000019987.10	23911.4135	-0.0025921	0.00526248	0.24816843	0.6183677	0.78926949	Arg1
ENSMUSG00000019988.8	344.740367	-0.0003566	0.00278493	0.01638903	0.89813342	0.97479981	Nedd1
ENSMUSG00000019989.9	1222.60043	-0.0041312	0.00432066	0.88097212	0.34793556	0.5360443	Enpp3
ENSMUSG00000019990.16	103.757779	0.02063597	0.00434113	21.3926216	3.74E-06	2.91E-05	Pde7b
ENSMUSG00000019992.9	5.47261963	0.02137842	0.0115343	3.6333216	0.05663368	0.13373668	Mtfr2
ENSMUSG00000019996.18	586.931767	-0.0066252	0.00378191	3.00826725	0.0828408	0.181066	Map7
ENSMUSG00000019997.12	233.56819	0.04276582	0.00610653	46.1914421	1.07E-11	1.98E-10	Ccn2
ENSMUSG00000019998.7	487.453242	0.00991602	0.00326103	9.09275014	0.00256625	0.00999628	Stx7
ENSMUSG00000020000.8	183.980118	-0.2647189	0.02087781	61.0754966	5.49E-15	1.56E-13	Moxd1
ENSMUSG00000020003.17	1673.88485	-0.0058584	0.00239678	5.97987041	0.01447007	0.04360431	Pex7
ENSMUSG00000020009.14	432.672954	0.01855773	0.00318623	32.9909194	9.26E-09	1.13E-07	Ifngr1
ENSMUSG00000020010.8	1163.33914	0.00861322	0.00438866	4.00299039	0.04541961	0.11154383	Vnn3
ENSMUSG00000020014.20	15.0715028	0.0012602	0.00967226	0.01497419	0.90260674	0.97702372	Cfap54
ENSMUSG00000020015.11	431.605067	0.01034901	0.00324368	9.79427283	0.00175056	0.00718315	Cdk17
ENSMUSG00000020017.15	7048.09518	-0.0081168	0.00643991	1.58395066	0.20819255	0.36939964	Hal
ENSMUSG00000020018.7	252.395283	-0.0049842	0.00370146	1.8152891	0.17787505	0.32750671	Snrpf
ENSMUSG00000020019.5	240.59527	0.01639845	0.00376306	18.0289561	2.18E-05	0.00014435	Ntn4
ENSMUSG00000020021.5	805.656081	-0.0136655	0.00360935	14.0044873	0.00018237	0.00098398	Fgd6
ENSMUSG00000020022.18	1234.05701	-0.0180318	0.0035949	25.5597106	4.29E-07	3.96E-06	Ndufa12
ENSMUSG00000020023.19	339.979257	0.00883086	0.00327068	7.02864644	0.0080216	0.02645377	Tmcc3
ENSMUSG00000020024.8	220.63007	0.00452975	0.00321001	1.99126843	0.15820832	0.29909488	Cep83
ENSMUSG00000020027.19	508.969624	-0.0277982	0.00645907	17.1994797	3.37E-05	0.000214	Socs2
ENSMUSG00000020029.7	3345.72257	0.00175535	0.00169364	1.06114454	0.30295429	0.4876462	Nudt4
ENSMUSG00000020032.15	86.3007246	0.01444042	0.00445548	10.1045988	0.001479	0.00619383	Nuak1
ENSMUSG00000020034.8	441.230511	-0.0111803	0.00345174	10.371147	0.00128	0.00545538	Tcp11l2
ENSMUSG00000020037.16	71.4591941	-0.0021976	0.00688116	0.09221026	0.76138586	0.89131623	Rfx4
ENSMUSG00000020038.11	369.87842	-0.0282175	0.0070197	15.6599055	7.58E-05	0.00044626	Cry1
ENSMUSG00000020042.16	29.2625904	0.03704895	0.00613089	34.5094849	4.24E-09	5.42E-08	Abtb3
ENSMUSG00000020044.14	1029.95175	0.02479888	0.00419669	33.6445677	6.62E-09	8.22E-08	Timp3
ENSMUSG00000020048.14	21108.512	-0.0006413	0.00365594	0.02938476	0.86389365	0.95513034	Hsp90b1
ENSMUSG00000020051.18	16024.9746	-0.0084023	0.00477361	3.07701187	0.07940629	0.17511704	Pah
ENSMUSG00000020052.10	10.6158918	0.06323237	0.03017379	5.44779671	0.01959302	0.05594008	Ascl1
ENSMUSG00000020053.19	11294.4129	-0.0092628	0.00380459	5.85937893	0.01549426	0.04610745	Igf1
ENSMUSG00000020056.17	409.225541	-0.0076202	0.00323596	5.52924377	0.01870119	0.05385668	Washc3
ENSMUSG00000020057.3	43.1371027	0.06749585	0.00664148	91.0598291	1.39E-21	8.74E-20	Dram1
ENSMUSG00000020059.10	5.60518829	-0.0103291	0.01417539	0.40962946	0.52215764	0.70807854	Sycp3
ENSMUSG00000020061.19	1.6166286	0.03951067	0.01738897	4.49316244	0.03403067	0.08827173	Mybpc1

ENSMUSG00000020062.8	30.3111911	0.00272891	0.01283282	0.04074468	0.84003156	0.94047488	Slc5a8
ENSMUSG00000020063.17	217.565874	-0.0023266	0.00248206	0.85471694	0.35522135	0.54383038	Sirt1
ENSMUSG00000020064.10	1039.8688	-0.0040829	0.00248231	2.64337052	0.10398273	0.21629214	Herc4
ENSMUSG00000020067.9	10.718793	0.00110016	0.01121209	0.00968429	0.92160769	0.98746372	Mypn
ENSMUSG00000020069.17	315.404846	-0.0051462	0.00297424	2.94060698	0.08637827	0.1873568	Hnrnph3
ENSMUSG00000020070.19	107.407833	0.01227156	0.00430593	7.69389594	0.00554079	0.01930355	Rufy2
ENSMUSG00000020072.16	2552.81324	-0.0143119	0.00432632	11.0167941	0.0009029	0.004009	Pbld2
ENSMUSG00000020074.8	4127.35728	-0.0038988	0.00363623	1.12427401	0.28900001	0.47019067	Ccar1
ENSMUSG00000020075.9	730.057467	-0.0028548	0.00296323	0.91862918	0.33783516	0.52536297	Ddx21
ENSMUSG00000020076.8	1132.01657	0.00211654	0.00219359	0.92579759	0.33595731	0.5232362	Ddx50
ENSMUSG00000020077.15	235.947212	0.03745181	0.00496418	52.4095448	4.51E-13	9.95E-12	Srgn
ENSMUSG00000020078.17	930.799855	0.00483301	0.00231017	4.31800964	0.03771101	0.09580481	Vps26a
ENSMUSG00000020079.16	296.267357	-0.013168	0.00284111	21.4775063	3.58E-06	2.80E-05	Supv3l1
ENSMUSG00000020080.9	7.20159485	0.04587524	0.00939862	23.0130185	1.61E-06	1.34E-05	Hkdc1
ENSMUSG00000020083.15	41.1142509	0.02024295	0.00538346	13.1452855	0.00028824	0.00147328	Fam241b
ENSMUSG00000020085.16	412.677901	-0.0017668	0.00271287	0.43002657	0.51197591	0.69861389	Aifm2
ENSMUSG00000020086.7	16.6371815	0.02029542	0.00501192	16.1031745	6.00E-05	0.00036137	Macroh2a2
ENSMUSG00000020087.6	744.579868	-0.0128905	0.00301892	18.464718	1.73E-05	0.00011731	Tysnd1
ENSMUSG00000020088.11	1592.2809	0.00378549	0.00236723	2.53641615	0.11124704	0.22822601	Sar1a
ENSMUSG00000020089.9	2574.64688	0.00015641	0.00309655	0.00253721	0.95982696	1	Ppa1
ENSMUSG00000020091.17	3934.93557	-0.0083364	0.00340552	5.8727704	0.01537683	0.04581582	Eif4ebp2
ENSMUSG00000020092.10	226.765909	-0.0064865	0.0025687	6.40178809	0.01140055	0.03561029	Pald1
ENSMUSG00000020097.15	1367.77413	0.00675924	0.00283795	5.62439489	0.01771218	0.0514926	Sgpl1
ENSMUSG00000020098.8	4104.34676	-0.0004536	0.00419807	0.01146379	0.91473417	0.98331526	Pcbd1
ENSMUSG00000020099.9	106.954205	0.02801784	0.00502661	30.1339002	4.03E-08	4.43E-07	Unc5b
ENSMUSG00000020100.16	146.213462	0.02948623	0.00408785	50.3880024	1.26E-12	2.60E-11	Slc29a3
ENSMUSG00000020101.15	191.669554	0.0326444	0.00492587	41.0806002	1.46E-10	2.29E-09	Vsir
ENSMUSG00000020102.16	1396.50121	0.03338441	0.00323421	105.337051	1.03E-24	9.42E-23	Slc16a7
ENSMUSG00000020105.10	160.918534	0.0057222	0.00455158	1.56617373	0.21076355	0.37281155	Lrig3
ENSMUSG00000020107.11	614.048647	-0.0097757	0.0034304	8.04608229	0.00456022	0.01633368	Anapc16
ENSMUSG00000020108.5	164.007766	-0.0007821	0.00560888	0.01953686	0.8888383	0.96943077	Ddit4
ENSMUSG00000020109.14	873.695017	-0.0008373	0.00158333	0.27797844	0.59802929	0.77254457	Dnajb12
ENSMUSG00000020111.17	456.203433	-0.0009211	0.00299705	0.0934372	0.75985212	0.89012973	Micu1
ENSMUSG00000020114.13	1152.80025	-0.0007771	0.00304724	0.06485278	0.7989841	0.91547812	Cand1
ENSMUSG00000020115.4	522.147411	0.00528304	0.00223011	5.46513711	0.01939953	0.05549513	Tbk1
ENSMUSG00000020116.8	644.117377	-0.0112163	0.00216477	26.6112671	2.49E-07	2.40E-06	Pno1
ENSMUSG00000020120.16	166.447325	0.08314057	0.00620622	151.476746	8.24E-35	3.09E-32	Plek
ENSMUSG00000020121.16	23.3201038	0.0046729	0.00494257	0.88500813	0.34683365	0.53520801	Srgap1
ENSMUSG00000020122.17	9921.97929	-0.0456072	0.00577865	58.681948	1.85E-14	4.91E-13	Egfr
ENSMUSG00000020123.7	567.840312	-0.0127164	0.00534343	5.4278223	0.01981838	0.05646047	Avpr1a
ENSMUSG00000020124.11	841.836602	-0.0057606	0.00314045	3.31705616	0.0685645	0.155676	Usp15
ENSMUSG00000020125.8	7.89462905	0.06145844	0.01483568	12.8718863	0.00033356	0.00167241	Elane
ENSMUSG00000020128.16	858.963989	4.17E-06	0.00218337	3.72E-06	0.99846106	1	Vps54
ENSMUSG00000020130.10	996.792437	-0.005849	0.00292184	3.9225213	0.04764338	0.1159786	Tbc1d15
ENSMUSG00000020131.15	192.787208	-0.0052606	0.00643267	0.66126017	0.41611541	0.60734561	Pcsk4
ENSMUSG00000020132.11	908.175975	-0.0016675	0.00221663	0.55914294	0.45460577	0.6463519	Rab21
ENSMUSG00000020133.9	298.485218	-0.0025395	0.00429443	0.35473648	0.55144409	0.7337862	2310011J03Rik
ENSMUSG00000020134.14	446.145457	0.00833701	0.0027653	8.99635463	0.00270519	0.01046662	Peli1
ENSMUSG00000020135.14	9.19019565	0.00233509	0.00803107	0.07906456	0.77856912	0.90259677	Apc2
ENSMUSG00000020137.6	281.450797	-0.0092391	0.00424022	4.61437316	0.03170508	0.08326423	Thap2
ENSMUSG00000020140.16	125.282532	-0.0013519	0.00528982	0.06499164	0.79877363	0.91540133	Lgr5
ENSMUSG00000020142.13	113.502859	0.02200802	0.00586558	13.99509	0.00018329	0.00098812	Slc1a4
ENSMUSG00000020143.16	132.986311	0.06125694	0.00587458	99.0061412	2.52E-23	1.93E-21	Dock2
ENSMUSG00000020149.18	3030.03992	0.00069954	0.00195631	0.12532623	0.72332806	0.86573362	Rab1a
ENSMUSG00000020150.14	2612.03252	-0.003332	0.00408824	0.66563981	0.41457591	0.60594828	Gamt
ENSMUSG00000020151.17	3.4772711	0.02604949	0.01044782	6.15984799	0.01306824	0.03988452	Ptprr
ENSMUSG00000020152.8	2029.12816	0.00504527	0.0018172	7.53579598	0.0060485	0.02080804	Actr2
ENSMUSG00000020153.15	1155.33972	-0.0206053	0.00323787	41.011498	1.51E-10	2.37E-09	Ndufs7
ENSMUSG00000020154.11	1754.36372	0.00039012	0.004282	0.00814872	0.92807247	0.99048799	Ptprb
ENSMUSG00000020155.3	4.54624973	0.01591826	0.01181818	1.84492712	0.17437455	0.32257241	Kcnmb1
ENSMUSG00000020156.17	303.581782	0.00218564	0.00420311	0.27099988	0.60266183	0.77597893	Pwwp3a

ENSMUSG00000020159.9	2.36914728	-0.003277	0.01324273	0.04588294	0.83038873	0.9345142	Gabrp
ENSMUSG00000020160.19	81.4139184	-0.0149274	0.00362879	17.4425731	2.96E-05	0.00019057	Meis1
ENSMUSG00000020163.13	3373.8102	-0.0152129	0.00434509	12.3240056	0.00044717	0.00216303	Uqcr11
ENSMUSG00000020166.16	567.859709	-0.0005284	0.00239307	0.04788603	0.82678343	0.93197878	Cnot2
ENSMUSG00000020167.15	304.883751	0.01176394	0.00337663	11.931207	0.00055201	0.00260918	Tcf3
ENSMUSG00000020168.6	0.87910344	-0.0007342	0.03376157	-0.0025615	1	1	Or14c43
ENSMUSG00000020170.5	372.597722	-0.0005151	0.00267215	0.03693733	0.84759269	0.94566326	Frs2
ENSMUSG00000020171.9	415.239856	-0.001689	0.0022196	0.57801988	0.44708951	0.63890991	Yeats4
ENSMUSG00000020173.18	372.719269	-0.0137394	0.00348534	15.7637183	7.18E-05	0.00042447	Cobl
ENSMUSG00000020175.16	32.1435332	0.00726002	0.00551922	1.69885888	0.19243728	0.34764289	Rab36
ENSMUSG00000020176.18	132.467922	-0.0166611	0.00501955	11.406282	0.00073196	0.00333927	Grb10
ENSMUSG00000020178.7	36.9249435	0.02664829	0.00458243	32.8999957	9.70E-09	1.17E-07	Adora2a
ENSMUSG00000020180.11	872.908009	-0.0178522	0.00457828	14.9779191	0.00010878	0.00061644	Snrpd3
ENSMUSG00000020181.19	12.9984985	0.06332666	0.01314338	21.2814241	3.97E-06	3.07E-05	Nav3
ENSMUSG00000020182.17	1072.84805	-0.0240696	0.00483012	24.5166641	7.37E-07	6.47E-06	Ddc
ENSMUSG00000020183.12	69.8939345	0.01847492	0.00439251	17.7580487	2.51E-05	0.00016385	Cpm
ENSMUSG00000020184.16	605.10978	0.00268201	0.00225596	1.39826786	0.2370138	0.40601337	Mdm2
ENSMUSG00000020185.17	13.1210521	0.00961827	0.00843451	1.16821643	0.27976802	0.45909751	E2f7
ENSMUSG00000020186.8	525.031723	-0.0047564	0.00341144	1.93369969	0.16435425	0.30787541	Csrp2
ENSMUSG00000020189.16	912.654306	-0.00015	0.00301825	0.00242736	0.96070556	1	Osbpl8
ENSMUSG00000020190.14	1989.68432	0.00122718	0.00375205	0.10705376	0.74352365	0.87885881	Mknk2
ENSMUSG00000020191.12	2.92914273	0.06144828	0.02087491	8.74483314	0.00310481	0.01175629	Spata48
ENSMUSG00000020193.4	6.6099917	0.0686459	0.0101052	41.5822634	1.13E-10	1.80E-09	Zpbp
ENSMUSG00000020196.11	332.253174	0.00245255	0.00379957	0.43156591	0.51122158	0.69803973	Cabin1
ENSMUSG00000020198.9	1182.92944	-0.0005189	0.00306989	0.02846788	0.86601354	0.95637879	Ap3d1
ENSMUSG00000020205.9	1289.47497	-0.0185886	0.00727938	5.95093676	0.01470947	0.0441846	Phlda1
ENSMUSG00000020211.16	225.629547	-0.0004211	0.00366168	0.01309541	0.90889284	0.9803872	Sf3a2
ENSMUSG00000020212.15	22.8345638	0.0191899	0.00661674	8.35894638	0.00383793	0.01409771	Mdm1
ENSMUSG00000020218.12	6.47831673	-0.0121843	0.01323295	0.72442282	0.39469697	0.58619247	Wif1
ENSMUSG00000020219.8	1323.264	-0.0123641	0.00516965	5.78715152	0.01614373	0.0476601	Timm13
ENSMUSG00000020220.17	1679.3887	-0.0024424	0.0030735	0.64106569	0.42332516	0.61503925	Vps13d
ENSMUSG00000020224.16	555.891849	-0.0012664	0.00374975	0.11372521	0.73594225	0.87388279	Llph
ENSMUSG00000020225.11	840.383052	0.00232504	0.00236663	0.96501347	0.32592696	0.51269263	Tmbim4
ENSMUSG00000020227.11	47.6492144	0.06008619	0.00633737	77.9719441	1.05E-18	4.62E-17	Irak3
ENSMUSG00000020228.12	240.776532	-0.0020477	0.00242398	0.7064337	0.40063028	0.59235608	Helb
ENSMUSG00000020230.16	23.0580004	0.02222677	0.00557037	15.7016383	7.42E-05	0.00043761	Prmt2
ENSMUSG00000020231.16	79.3909116	0.00072147	0.00406683	0.03212421	0.85775531	0.95209266	Dip2a
ENSMUSG00000020232.19	421.244928	-0.0089706	0.0034674	6.72068049	0.00953013	0.0306487	Hmg20b
ENSMUSG00000020234.11	3.16128775	0.0043053	0.01261199	0.11663536	0.73271276	0.87175964	Tektip1
ENSMUSG00000020235.17	209.602748	-0.0017546	0.00210979	0.69776585	0.40353542	0.59515923	Fzr1
ENSMUSG00000020238.15	953.60962	-0.0059736	0.00211759	7.96444207	0.00477052	0.01698377	Ncln
ENSMUSG00000020241.14	139.16602	0.02049388	0.00544165	13.9866138	0.00018412	0.00099168	Col6a2
ENSMUSG00000020246.14	234.862705	-0.0052459	0.00264254	3.93260329	0.04735858	0.11549427	Hcfc2
ENSMUSG00000020248.19	636.738648	0.00069853	0.00230798	0.09020977	0.76391065	0.89280137	Nfyb
ENSMUSG00000020250.12	2434.41138	0.00219382	0.00215766	1.01060568	0.31475778	0.50019384	Txnrd1
ENSMUSG00000020251.15	5.04508969	-0.0077391	0.01092383	0.48940194	0.48419421	0.67328172	Glt8d2
ENSMUSG00000020253.16	217.686858	-0.002084	0.00228873	0.82491271	0.36374771	0.55332818	Ppm1m
ENSMUSG00000020255.9	353.806861	0.00570423	0.00280386	3.98192125	0.04599108	0.11278286	Nopchap1
ENSMUSG00000020256.15	19.5312893	0.0132725	0.00969864	1.75314089	0.18548237	0.33816086	Aldh1l2
ENSMUSG00000020257.14	704.012887	-0.0035926	0.00205187	3.01994604	0.0822462	0.18001661	Wdr82
ENSMUSG00000020258.17	4273.91989	-0.0082699	0.00436974	3.45858295	0.06292422	0.14540322	Glyctk
ENSMUSG00000020260.10	309.814869	-0.0050795	0.00250818	4.08839826	0.04317854	0.1070995	Pofut2
ENSMUSG00000020261.16	490.294774	0.00047807	0.00364814	0.01723303	0.89555805	0.97320224	Slc36a1
ENSMUSG00000020262.16	239.350157	0.00084577	0.00351545	0.05857799	0.80875777	0.92098111	Adarb1
ENSMUSG00000020263.15	416.402841	0.0074707	0.00331229	4.97137525	0.02577015	0.07023512	Appl2
ENSMUSG00000020264.6	4.94507292	0.05572805	0.02290095	6.04871823	0.0139164	0.04215129	Slc36a2
ENSMUSG00000020265.17	1524.53778	0.00295446	0.00237147	1.54127739	0.21442777	0.37753757	Sumo3
ENSMUSG00000020267.7	4516.5458	-0.0065949	0.00360542	3.37483259	0.06619931	0.15147233	Hint1
ENSMUSG00000020268.15	142.541347	-0.0151596	0.00684374	4.64008251	0.0312335	0.08222718	Lyrm7
ENSMUSG00000020271.18	634.933059	0.00217685	0.00253636	0.74085171	0.38938818	0.58086836	Fbxw11
ENSMUSG00000020272.9	104.665649	0.04507868	0.00477388	84.9833698	3.01E-20	1.61E-18	Stk10

ENSMUSG00000020273.14	139.324395	0.0004388	0.00258503	0.02844061	0.86607711	0.95637879	Papolg
ENSMUSG00000020275.10	152.402577	0.01946178	0.0035815	28.4247832	9.74E-08	1.01E-06	Rel
ENSMUSG00000020277.11	657.503201	0.00096325	0.00278176	0.11905308	0.73006378	0.87027617	Pfkl
ENSMUSG00000020279.11	4.81391261	0.12728943	0.0136351	55.9993977	7.25E-14	1.76E-12	Il9r
ENSMUSG00000020280.13	337.554904	-0.0044028	0.00250529	3.01711866	0.08238973	0.18031401	Pus10
ENSMUSG00000020282.19	104.730714	0.01488527	0.00437094	11.4100732	0.00073047	0.00333376	Rhbdf1
ENSMUSG00000020283.6	1438.38662	-0.0098226	0.00217302	19.9470834	7.96E-06	5.75E-05	Pex13
ENSMUSG00000020284.17	97.8964915	0.00163126	0.00357492	0.21028923	0.64654081	0.81100929	Cfap410
ENSMUSG00000020286.14	1.75215431	0.00065924	0.02400335	0.02116779	0.88432279	0.96643364	1700093K21Rik
ENSMUSG00000020287.16	176.485053	0.00707477	0.00229803	9.51250298	0.00204077	0.00822514	Mpg
ENSMUSG00000020288.14	660.190739	-0.0210475	0.00283567	52.7347756	3.82E-13	8.49E-12	Ahsa2
ENSMUSG00000020289.16	205.794272	-0.0069448	0.0030294	5.31141589	0.02118613	0.05967946	Nprl3
ENSMUSG00000020290.16	675.795293	0.00313251	0.00271638	1.27914933	0.25805726	0.43154698	Xpo1
ENSMUSG00000020297.11	69.0947259	0.05948789	0.01185401	22.7802519	1.82E-06	1.50E-05	Nsg2
ENSMUSG00000020300.15	1133.83223	-0.0077588	0.00388683	3.81820169	0.05069862	0.12196835	Cpeb4
ENSMUSG00000020303.3	11.8474684	0.0187598	0.01102575	2.71615861	0.09933682	0.20911802	Stc2
ENSMUSG00000020305.14	129.867847	0.00301625	0.00255945	1.36508828	0.24265758	0.41293807	Asb3
ENSMUSG00000020307.17	1068.42547	-0.0138472	0.00362273	14.5586941	0.00013586	0.00075168	Cdc34
ENSMUSG00000020308.8	246.888057	-0.0068635	0.00317301	4.67753568	0.03055956	0.08074131	Tpgs1
ENSMUSG00000020309.7	649.180259	-0.0103974	0.00296501	12.4386999	0.00042053	0.00205141	Chac2
ENSMUSG00000020311.18	508.457857	-0.0018476	0.00258513	0.5059276	0.4769071	0.66700254	Erlec1
ENSMUSG00000020312.13	27.2542757	0.06541457	0.01216628	26.144267	3.17E-07	3.00E-06	Shc2
ENSMUSG00000020315.19	2014.49593	0.00856803	0.00256134	10.8781072	0.00097308	0.0042891	Sptbn1
ENSMUSG00000020319.10	45.0783099	0.00760952	0.00409321	3.3282707	0.06809844	0.15485632	Wdpcp
ENSMUSG00000020321.17	9584.56326	-0.0141716	0.00322288	19.3524211	1.09E-05	7.65E-05	Mdh1
ENSMUSG00000020325.11	7.89663424	0.03056936	0.00736475	17.1458996	3.46E-05	0.00021971	Fstl3
ENSMUSG00000020326.8	1295.22633	0.01053851	0.00395179	6.70119632	0.00963482	0.03093058	Ccng1
ENSMUSG00000020328.11	571.261672	-0.0041955	0.00276591	2.30567766	0.12890205	0.25613174	Nudcd2
ENSMUSG00000020329.13	353.711178	-0.0108121	0.00391388	7.7093873	0.00549344	0.01917541	Polrmt
ENSMUSG00000020330.17	23.2002862	0.03197253	0.01170607	6.28618685	0.01216826	0.03756551	Hmmr
ENSMUSG00000020331.11	10.191138	-0.0236161	0.00693091	11.3327467	0.00076152	0.00345675	Hcn2
ENSMUSG00000020333.18	19.1952076	-0.0142681	0.01047143	1.67450082	0.19565677	0.35216427	Acsl6
ENSMUSG00000020334.7	44.1224431	0.00094748	0.00429055	0.04856176	0.82558509	0.93110744	Slc22a4
ENSMUSG00000020335.14	42.4170868	0.00106726	0.00378853	0.07827346	0.77965094	0.90327065	Zfp354b
ENSMUSG00000020340.17	148.273646	0.03625004	0.00507759	46.3246785	1.00E-11	1.86E-10	Cyfip2
ENSMUSG00000020346.17	1046.23792	-0.0034333	0.00206305	2.7460482	0.09749512	0.20608711	Mgat1
ENSMUSG00000020349.5	2239.85842	-0.0024599	0.00132633	3.41482081	0.06461343	0.14849189	Ppp2ca
ENSMUSG00000020354.16	5.77265693	0.01551044	0.01136079	1.85871483	0.1727732	0.32028835	Sgcd
ENSMUSG00000020357.4	491.362998	0.00983979	0.00377041	6.75520544	0.00934746	0.03018471	Flt4
ENSMUSG00000020358.18	2385.33962	0.00017115	0.00206555	0.00680065	0.93427609	0.99421747	Hnrnpab
ENSMUSG00000020359.14	284.948478	0.0005026	0.0020498	0.06114299	0.80469843	0.91835376	Phykpl
ENSMUSG00000020361.14	2436.00896	-0.0057689	0.00229757	6.23179144	0.01254767	0.03851001	Hspa4
ENSMUSG00000020362.14	734.074221	0.00381549	0.00240692	2.49721004	0.11404818	0.23267367	Cnot6
ENSMUSG00000020363.7	17.152088	0.04134527	0.00688387	34.1048971	5.22E-09	6.58E-08	Gfpt2
ENSMUSG00000020364.15	103.257639	0.00494138	0.00386623	1.62595184	0.20226388	0.36136663	Zfp354a
ENSMUSG00000020366.19	961.536046	0.0067079	0.00218415	9.42993618	0.00213472	0.0085512	Mapk9
ENSMUSG00000020368.16	11708.9904	-0.0040755	0.00322812	1.54621675	0.21369482	0.3764438	Canx
ENSMUSG00000020372.16	9425.31024	-0.0016684	0.00487511	0.11863158	0.73052342	0.87054925	Rack1
ENSMUSG00000020374.17	2.73653357	0.0224861	0.02442173	1.31895219	0.25078019	0.42246279	Rasgef1c
ENSMUSG00000020375.10	349.747077	0.00264652	0.00206351	1.64772695	0.19926859	0.35723414	Rufy1
ENSMUSG00000020376.18	1457.01522	0.00593993	0.00239322	6.15065807	0.01313632	0.04008192	Rnf130
ENSMUSG00000020377.16	7.07297418	-0.0103299	0.00748453	1.78638996	0.18136663	0.33243702	Ltc4s
ENSMUSG00000020380.17	275.308887	0.00766989	0.00408825	3.48217032	0.06203333	0.14365421	Rad50
ENSMUSG00000020381.11	116.160208	-0.0045141	0.00470463	0.9106102	0.33995257	0.52764683	Mrnip
ENSMUSG00000020385.17	730.905887	0.00089998	0.00274365	0.10739538	0.74312917	0.87865674	Clk4
ENSMUSG00000020386.6	5372.85998	-0.0109654	0.00300252	13.0673155	0.00030049	0.00152681	Sar1b
ENSMUSG00000020387.16	204.820817	0.02042277	0.00365264	31.0636102	2.50E-08	2.84E-07	Jade2
ENSMUSG00000020388.13	20.6551385	0.03669123	0.0063818	32.195309	1.39E-08	1.65E-07	Pdlim4
ENSMUSG00000020389.20	18.2359313	0.01886638	0.0079341	5.00848243	0.02522341	0.06898415	Cdkl3
ENSMUSG00000020390.13	2401.83752	-0.0094595	0.00253295	13.6065157	0.0002254	0.00118562	Ube2b
ENSMUSG00000020392.9	274.455485	0.00842114	0.00226084	13.9209988	0.00019066	0.0010227	Cdkn2aipnl

ENSMUSG00000020393.17	265.022064	-0.0029321	0.00351309	0.70201749	0.40210662	0.59372583	Kremen1
ENSMUSG00000020395.14	15.6427153	0.05810994	0.00683453	67.7273256	1.88E-16	6.27E-15	Itk
ENSMUSG00000020396.9	11.3069051	0.03294488	0.00823951	15.5660024	7.97E-05	0.00046584	Nefh
ENSMUSG00000020397.16	290.494529	-0.0042633	0.00231222	3.36152139	0.06673639	0.15234654	Med7
ENSMUSG00000020399.15	23.5213752	0.10689859	0.0069698	212.762863	3.43E-48	4.49E-45	Havcr2
ENSMUSG00000020400.18	216.13488	0.01610851	0.00342708	21.2367397	4.06E-06	3.13E-05	Tnip1
ENSMUSG00000020401.7	1.35639157	0.04619022	0.02632441	2.75028998	0.09723679	0.20568852	Garin3
ENSMUSG00000020402.12	3576.81417	-0.0073122	0.00261427	7.80414983	0.00521264	0.01833636	Vdac1
ENSMUSG00000020407.14	38.8520501	0.01151495	0.0043469	6.97334936	0.00827325	0.02714703	Upp1
ENSMUSG00000020409.11	531.750971	-0.000403	0.0026296	0.02301833	0.87940941	0.96360378	Slu7
ENSMUSG00000020412.17	509.173709	-0.0044902	0.00203491	4.88417354	0.02710401	0.07315198	Ascc2
ENSMUSG00000020413.12	128.055958	0.00244823	0.00277457	0.77184437	0.37964709	0.57114148	Hus1
ENSMUSG00000020415.17	350.623195	-0.0003203	0.00403245	0.00640005	0.93623701	0.99500231	Pttg1
ENSMUSG00000020419.12	7.00495237	0.02737707	0.01081722	5.71983659	0.01677429	0.04923326	Hormad2
ENSMUSG00000020420.9	34.6084545	0.00769111	0.0032204	5.71136013	0.01685548	0.04942848	Zfp607a
ENSMUSG00000020422.15	759.44886	0.01101032	0.00272335	16.1483356	5.86E-05	0.00035358	Tns3
ENSMUSG00000020423.7	399.794876	0.02495759	0.00638513	13.558982	0.00023118	0.00121161	Btg2
ENSMUSG00000020424.4	28.2004276	0.01075563	0.00394193	7.57795388	0.00590865	0.02040131	Castor1
ENSMUSG00000020427.12	463.66277	0.02364813	0.00345474	47.3612325	5.90E-12	1.13E-10	Igfbp3
ENSMUSG00000020429.8	1524.70044	-0.0361241	0.00941914	13.769194	0.0002067	0.00109801	Igfbp1
ENSMUSG00000020430.13	521.488253	-0.0010855	0.00267214	0.16354799	0.68591075	0.83988636	Pes1
ENSMUSG00000020431.6	54.0628469	0.02804253	0.01056204	6.38411674	0.0115146	0.03586207	Adcy1
ENSMUSG00000020432.13	1833.86922	0.01659878	0.00251016	43.8523445	3.54E-11	6.04E-10	Tcn2
ENSMUSG00000020434.5	1.66157759	0.00794374	0.02422	0.27753775	0.59831962	0.77275033	4921536K21Rik
ENSMUSG00000020435.18	28.3238241	0.03425867	0.00738392	19.6749161	9.18E-06	6.56E-05	Osbp2
ENSMUSG00000020436.18	10.7910062	0.0254354	0.01677938	2.6721373	0.1021188	0.21358414	Gabrg2
ENSMUSG00000020437.13	58.0362401	0.06249157	0.00608565	91.8889804	9.17E-22	5.84E-20	Myo1g
ENSMUSG00000020439.18	139.25387	-0.0032561	0.00496509	0.43148479	0.51126128	0.69803973	Smtn
ENSMUSG00000020440.15	1802.33711	-0.0022773	0.00538253	0.17931788	0.6719601	0.82965246	Arf5
ENSMUSG00000020441.7	175.365417	0.00215781	0.00403882	0.28479671	0.59357438	0.76889528	2310033P09Rik
ENSMUSG00000020444.21	863.782326	-0.0105843	0.00413125	6.48026343	0.01090788	0.03433918	Guk1
ENSMUSG00000020448.17	554.659254	-0.0020581	0.00155017	1.7686821	0.18354526	0.33551198	Rnf185
ENSMUSG00000020451.18	136.831566	0.00297489	0.00391107	0.57911706	0.44665861	0.63847819	Limk2
ENSMUSG00000020453.18	86.2121207	0.01596551	0.00389841	16.5224929	4.81E-05	0.00029641	Patz1
ENSMUSG00000020454.18	472.617874	0.00411738	0.00265655	2.42523667	0.11939521	0.24122455	Eif4enif1
ENSMUSG00000020455.17	301.139922	-0.0036645	0.00251496	2.13035497	0.14440731	0.27950032	Trim11
ENSMUSG00000020456.18	3633.5927	-0.0088574	0.0029051	9.28919842	0.00230509	0.00911812	Ogdh
ENSMUSG00000020457.14	868.352667	-0.0046318	0.00260272	3.16527396	0.07521981	0.16776443	Drg1
ENSMUSG00000020458.17	1572.22148	0.02170642	0.00268455	66.2739933	3.92E-16	1.27E-14	Rtn4
ENSMUSG00000020459.15	628.049743	-0.0076931	0.00250374	9.32432873	0.00226131	0.00898808	Mtif2
ENSMUSG00000020460.16	6264.91221	-0.0046317	0.00403413	1.31605063	0.25130207	0.42307362	Rps27a
ENSMUSG00000020462.15	358.515319	3.12E-05	0.00269367	0.00012983	0.99090882	1	Cfap36
ENSMUSG00000020463.16	919.600476	-0.0030756	0.00307537	0.97157357	0.32428805	0.51096653	Ppp4r3b
ENSMUSG00000020464.16	498.552871	-0.0053992	0.00219458	6.08220692	0.01365501	0.04142341	Pnpt1
ENSMUSG00000020467.16	311.559522	0.03745325	0.00312927	135.839868	2.16E-31	5.31E-29	Efemp1
ENSMUSG00000020469.9	21.3590499	0.02007195	0.01131849	-1.6180015	1	1	Myl7
ENSMUSG00000020471.12	199.802485	-0.0036997	0.00412773	0.79153748	0.37363563	0.56447412	Pold2
ENSMUSG00000020472.15	322.119658	0.00127838	0.00302206	0.1812416	0.6703084	0.82861079	Zkscan17
ENSMUSG00000020473.14	60.3230536	0.01585953	0.00573285	7.4050495	0.00650411	0.02217168	Aebp1
ENSMUSG00000020474.12	70.5701938	0.00886776	0.0044544	3.83622837	0.05015623	0.12087307	Polm
ENSMUSG00000020475.4	57.1817621	-0.0197305	0.01760941	0.99952857	0.31742461	0.50298207	Pgam2
ENSMUSG00000020476.15	509.349454	0.01313987	0.00251758	27.3098222	1.73E-07	1.72E-06	Dbnl
ENSMUSG00000020477.11	944.481557	-0.0153328	0.00326072	22.4238159	2.19E-06	1.78E-05	Mrps24
ENSMUSG00000020481.16	1.78360951	-0.0428525	0.02990972	2.22360306	0.13591554	0.26678628	Ankrd36
ENSMUSG00000020482.5	516.959881	-0.0124056	0.00282308	19.0723846	1.26E-05	8.75E-05	Ccdc117
ENSMUSG00000020483.15	2472.55068	-0.0089924	0.00301567	8.84673002	0.00293614	0.01122033	Dynll2
ENSMUSG00000020484.20	3924.86971	-0.0111427	0.00308306	12.6807011	0.00036945	0.00182833	Xbp1
ENSMUSG00000020485.15	623.861748	-0.0073948	0.00326568	5.13948103	0.02338764	0.06477464	Supt4a
ENSMUSG00000020486.20	227.469353	0.0164449	0.00277656	34.7070123	3.83E-09	4.93E-08	Septin4
ENSMUSG00000020490.18	1.12089756	0.08528137	0.02523457	10.415981	0.00124929	0.00534609	Btnl10
ENSMUSG00000020491.16	52.9330821	0.00785436	0.00334647	5.43718023	0.01971247	0.05622501	2810021J22Rik

ENSMUSG00000020492.12	53.8861977	0.01012117	0.0055672	3.16937249	0.07503127	0.16745718	Ska2
ENSMUSG00000020493.9	10.7281363	0.03436319	0.01109106	8.96509807	0.00275186	0.01061239	Prr11
ENSMUSG00000020495.14	170.51684	-0.0081021	0.00177896	20.662406	5.48E-06	4.11E-05	Smg8
ENSMUSG00000020496.11	1469.98827	-0.0036913	0.00170064	4.70795875	0.03002328	0.07949361	Rnf187
ENSMUSG00000020513.16	60.6968046	-0.011882	0.00342006	12.360037	0.00043862	0.00212692	Tubd1
ENSMUSG00000020514.9	441.979861	-0.0139692	0.00297673	22.2673517	2.37E-06	1.91E-05	Mrpl22
ENSMUSG00000020515.14	810.797236	-0.0102389	0.00300102	11.5755677	0.00066824	0.00308739	Cnot8
ENSMUSG00000020516.16	666.399374	-0.0050997	0.00305383	2.76627649	0.09626987	0.20411016	Rps6kb1
ENSMUSG00000020519.6	220.880687	-0.0011419	0.00239154	0.22651048	0.63412342	0.80186587	Sap30l
ENSMUSG00000020520.15	136.463417	0.01324598	0.00323645	16.465185	4.96E-05	0.00030408	Galnt10
ENSMUSG00000020521.8	915.511693	-0.0085388	0.00271517	9.7588776	0.00178459	0.00730498	Rnft1
ENSMUSG00000020522.14	296.489301	0.00511829	0.00337713	2.2470606	0.13386847	0.26361267	Mfap3
ENSMUSG00000020523.15	680.751471	-0.005681	0.00230169	6.11251239	0.01342282	0.0408188	Fam114a2
ENSMUSG00000020524.17	25.4731732	-0.0003566	0.01393547	0.00112598	0.97323155	1	Gria1
ENSMUSG00000020525.18	106.552336	-0.0055712	0.00241502	5.21374337	0.02240904	0.06256386	Ppm1d
ENSMUSG00000020526.13	205.369002	-0.0144472	0.0035377	16.7895562	4.18E-05	0.00026162	Znhit3
ENSMUSG00000020527.15	199.088357	-0.0029643	0.00360538	0.69402537	0.40479857	0.59631372	Myo19
ENSMUSG00000020528.15	196.408211	-0.002466	0.00246193	0.98307555	0.32144068	0.50766873	Prpsap2
ENSMUSG00000020530.15	1909.68648	0.00397677	0.00316561	1.55191785	0.21285254	0.37535252	Ggnbp2
ENSMUSG00000020532.19	3210.01727	0.00677949	0.00498949	1.84618736	0.17422748	0.32240148	Acaca
ENSMUSG00000020534.15	3371.97568	-0.004877	0.00334686	2.10941777	0.14639501	0.2823281	Shmt1
ENSMUSG00000020536.13	146.749104	0.00261445	0.00259051	1.01219991	0.31437639	0.49968875	Llgl1
ENSMUSG00000020537.10	493.201639	-0.0055006	0.00216687	6.51065005	0.01072303	0.03387082	Drg2
ENSMUSG00000020538.16	4620.31378	0.01788474	0.00494894	13.4061646	0.0002508	0.00130179	Srebf1
ENSMUSG00000020541.13	383.665976	-0.0020909	0.00253832	0.67492565	0.41133955	0.60289925	Tom1l1
ENSMUSG00000020542.19	4.27582307	-0.0146063	0.01770982	-0.5656947	1	1	Myocd
ENSMUSG00000020544.15	165.951568	-0.0199564	0.00236492	71.6892942	2.52E-17	9.48E-16	Cox11
ENSMUSG00000020546.15	64.2561615	-0.0015969	0.00358908	0.19540328	0.65845706	0.8199772	Stxbp4
ENSMUSG00000020547.6	230.78251	0.00042983	0.00379869	0.01265538	0.91043005	0.98098286	Bzw2
ENSMUSG00000020549.15	420.300314	-0.0071512	0.00199914	12.7696627	0.00035229	0.00175704	Elac2
ENSMUSG00000020553.9	2056.13887	-0.0008582	0.00353418	0.05799145	0.80969917	0.92160818	Pctp
ENSMUSG00000020561.9	535.261502	-0.0070077	0.00386225	3.20234055	0.07353297	0.16479937	Polr1f
ENSMUSG00000020564.18	196.642774	-0.0079182	0.00311379	6.38845945	0.01148647	0.0358028	Atxn7l1
ENSMUSG00000020566.20	1.56242201	-0.0155838	0.02338098	0.71235552	0.39866294	0.59040862	Atp6v1c2
ENSMUSG00000020570.16	1145.27893	0.0024196	0.0023757	1.03418598	0.30917752	0.49425655	Sypl
ENSMUSG00000020571.14	4379.83823	-0.0123045	0.00313119	15.0595351	0.00010417	0.00059331	Pdia6
ENSMUSG00000020572.9	1775.38606	0.01676113	0.00396731	17.7209202	2.56E-05	0.0001668	Nampt
ENSMUSG00000020573.18	45.7685829	0.04792033	0.00606865	56.601029	5.34E-14	1.32E-12	Pik3cg
ENSMUSG00000020576.11	393.699365	-0.0009396	0.00297714	0.09912814	0.75287842	0.88543458	Nbas
ENSMUSG00000020577.18	65.2099754	0.04494292	0.0056269	59.1159281	1.49E-14	3.99E-13	Tspan13
ENSMUSG00000020580.11	799.903022	0.00575434	0.00307729	3.39653457	0.06533354	0.14986938	Rock2
ENSMUSG00000020581.12	1.11784267	-0.0076247	0.03120383	0.0351608	0.85125911	0.9483632	Agr2
ENSMUSG00000020585.6	3711.09784	0.00393997	0.00188755	4.3311627	0.03742068	0.09516973	Laptm4a
ENSMUSG00000020589.18	169.327574	0.04983932	0.00533995	81.1154422	2.13E-19	1.03E-17	Cyria
ENSMUSG00000020590.17	768.964089	0.00074814	0.00350214	0.044908	0.83217329	0.93545131	Snx13
ENSMUSG00000020591.12	3.59956414	0.00052195	0.0161749	-0.004389	1	1	Ntsr2
ENSMUSG00000020592.16	4355.58518	0.0086475	0.00393214	4.73580619	0.029541	0.07848923	Sdc1
ENSMUSG00000020593.17	3073.07424	-0.0279245	0.00737676	15.4095303	8.66E-05	0.00050149	Lpin1
ENSMUSG00000020594.15	1289.8872	0.00188803	0.0026816	0.48715356	0.48519979	0.6741773	Pum2
ENSMUSG00000020598.17	14.0985982	0.02996846	0.01433686	4.35351326	0.03693268	0.0942029	Nrcam
ENSMUSG00000020599.14	8.90869732	0.04248994	0.0070001	34.6420773	3.96E-09	5.08E-08	Rgs9
ENSMUSG00000020600.14	2.87511628	0.03330287	0.01096737	8.83021957	0.00296283	0.01130033	Slc7a15
ENSMUSG00000020601.9	77.1170952	0.02596204	0.00415687	37.947731	7.27E-10	1.03E-08	Trib2
ENSMUSG00000020604.14	487.352537	-0.0090136	0.0042971	4.36352336	0.03671629	0.09382053	Arsg
ENSMUSG00000020605.10	225.58164	0.00753442	0.00263297	8.2070588	0.00417277	0.01515549	Hs1bp3
ENSMUSG00000020607.8	54.0200906	0.01774865	0.00508088	11.4017336	0.00073376	0.00334552	Lratd1
ENSMUSG00000020608.8	637.749477	-0.0052239	0.00315104	2.78525593	0.09513549	0.20216828	Smc6
ENSMUSG00000020609.15	84674.3381	-0.0014636	0.00568223	0.0644754	0.79955732	0.91563211	Apob
ENSMUSG00000020610.18	290.574809	-0.0011636	0.00209811	0.30834572	0.5786971	0.75681451	Amz2
ENSMUSG00000020611.15	723.54688	0.01297373	0.00229875	31.6071881	1.89E-08	2.19E-07	Gna13
ENSMUSG00000020612.17	2720.12569	-0.0021426	0.00192766	1.23128236	0.26715779	0.44293433	Prkar1a

ENSMUSG00000020614.14	533.446571	-0.0005266	0.0032155	0.02682336	0.86990561	0.95849733	Fam20a
ENSMUSG00000020617.14	1.46129169	-0.0162653	0.06850143	0.12194799	0.72693126	0.86815156	1700012B07Rik
ENSMUSG00000020620.15	1367.08298	0.00115178	0.00370629	0.09514881	0.7577308	0.88865987	Abca8b
ENSMUSG00000020621.7	407.52697	-0.0082379	0.00215638	14.6472213	0.00012963	0.00072124	Rdh14
ENSMUSG00000020622.18	3.54773136	-0.0102537	0.03863131	-0.0460503	1	1	Nt5c1b
ENSMUSG00000020623.12	101.888635	0.01001903	0.00526627	3.87011815	0.04915306	0.11891723	Map2k6
ENSMUSG00000020627.11	7.4259973	0.03864312	0.00797072	22.8319124	1.77E-06	1.46E-05	Klhl29
ENSMUSG00000020628.17	405.719194	0.0041251	0.00177601	5.37666189	0.02040789	0.05794093	Trappc12
ENSMUSG00000020629.13	3066.01356	0.0025671	0.00249869	1.04070647	0.30765722	0.49269441	Adi1
ENSMUSG00000020630.10	89.0188684	0.00462724	0.00263833	3.08407903	0.07906201	0.17448831	Rnaseh1
ENSMUSG00000020634.13	574.928648	-0.0022677	0.00296038	0.57395751	0.44869058	0.64056752	Ubxn2a
ENSMUSG00000020635.9	6.01859251	0.03611689	0.009445	12.7816811	0.00035003	0.00174763	Fkbp1b
ENSMUSG00000020636.15	1.10151773	-0.0296178	0.07857549	0.27670483	0.59886916	0.77320605	Allc
ENSMUSG00000020638.9	164.935146	0.02032144	0.00502139	16.5210584	4.81E-05	0.00029652	Cmpk2
ENSMUSG00000020639.15	18.945432	-0.0219898	0.00662215	10.2998631	0.0013304	0.00563759	Pfn4
ENSMUSG00000020640.11	1102.04826	-0.0003235	0.00369287	0.00758241	0.93061027	0.99201726	Itsn2
ENSMUSG00000020641.17	291.602704	0.01854138	0.00541273	11.7670874	0.00060287	0.00281683	Rsad2
ENSMUSG00000020642.14	156.052176	0.01751115	0.00574164	8.74312496	0.00310771	0.01176542	Rnf144a
ENSMUSG00000020644.10	2757.70154	0.00146145	0.00436295	0.10414787	0.74690769	0.88117874	Id2
ENSMUSG00000020646.18	8.71398814	0.02400328	0.01207251	3.1885134	0.07415742	0.16582072	Mboat2
ENSMUSG00000020647.11	490.109612	0.00520446	0.00400996	1.65146806	0.19875924	0.3565241	Ncoa1
ENSMUSG00000020648.4	72.5222392	-0.0084639	0.00386278	4.79651208	0.02851741	0.07627713	Dus4l
ENSMUSG00000020649.12	134.28774	0.00311028	0.00514294	0.35624368	0.55059983	0.73311774	Rrm2
ENSMUSG00000020650.16	595.542221	0.00221875	0.00468277	0.21456769	0.64321061	0.80897806	Bcap29
ENSMUSG00000020651.9	12.6270057	0.05435638	0.00998407	27.3130863	1.73E-07	1.72E-06	Slc26a4
ENSMUSG00000020652.18	68.7176878	0.00827322	0.00316598	6.7355116	0.00945121	0.03045105	Cenpo
ENSMUSG00000020653.13	224.366738	0.00554033	0.00385719	1.97349266	0.16007762	0.30163704	Klf11
ENSMUSG00000020654.16	33.0982924	0.03280592	0.0057794	30.0908208	4.12E-08	4.52E-07	Adcy3
ENSMUSG00000020656.17	31.5640468	0.00151626	0.00668911	0.04834526	0.82596808	0.93133299	Grhl1
ENSMUSG00000020657.17	28.3783974	0.012563	0.0051468	5.718268	0.01678928	0.04927113	Dnajc27
ENSMUSG00000020658.11	32.9027086	-0.0031178	0.00670676	0.19447436	0.65921844	0.82049215	Efr3b
ENSMUSG00000020659.18	183.977635	-0.0011764	0.00265091	0.19377154	0.65979596	0.82099433	Cbll1
ENSMUSG00000020660.7	2.88737608	0.00677229	0.00979001	0.44799591	0.5032882	0.69022884	Pomc
ENSMUSG00000020661.17	535.364712	0.00753238	0.00447363	2.72200274	0.09897376	0.20848398	Dnmt3a
ENSMUSG00000020664.11	3585.44149	-0.0103225	0.00308828	11.0533611	0.00088527	0.0039396	Dld
ENSMUSG00000020668.11	53.6056137	0.02980997	0.00585241	25.3789929	4.71E-07	4.31E-06	Kif3c
ENSMUSG00000020669.16	132.553345	0.01403366	0.00280003	25.0015445	5.73E-07	5.16E-06	Sh3yl1
ENSMUSG00000020671.10	1714.92753	0.00258276	0.00187512	1.86603876	0.17192946	0.31897492	Rab10
ENSMUSG00000020672.15	125.614985	-0.0330175	0.00403547	67.8548126	1.76E-16	5.89E-15	Sntg2
ENSMUSG00000020674.18	225.606407	-0.0086025	0.00403679	4.55006944	0.03291741	0.08581741	Pxdn
ENSMUSG00000020676.3	3.16416638	0.04704633	0.01640629	8.85098757	0.0029293	0.01119782	Ccl11
ENSMUSG00000020677.8	320.14961	-0.0022408	0.00218623	1.0429305	0.30714089	0.49216027	Ddx52
ENSMUSG00000020679.12	251.546714	-0.0041747	0.00409958	1.02260601	0.31190165	0.49712883	Hnf1b
ENSMUSG00000020680.16	746.199199	-0.0062443	0.00347577	3.19046188	0.07406908	0.1656703	Taf15
ENSMUSG00000020681.15	41.1938997	0.04217804	0.00677018	35.9405825	2.03E-09	2.70E-08	Ace
ENSMUSG00000020682.18	6.96364377	-0.0012963	0.00913056	0.0206688	0.88568489	0.96720825	Mmp28
ENSMUSG00000020684.15	15.5032605	0.04130297	0.00781841	27.5735688	1.51E-07	1.53E-06	Rasl10b
ENSMUSG00000020687.14	529.148088	0.00347529	0.00245734	1.94937704	0.162654	0.30532963	Cdc27
ENSMUSG00000020689.5	100.282596	0.00992319	0.00426261	5.29443803	0.02139363	0.06017055	Itgb3
ENSMUSG00000020690.13	1.10795694	-0.0090601	0.02128876	0.18669883	0.66567847	0.82501066	Efcab3
ENSMUSG00000020691.14	154.400626	-0.005306	0.0024318	4.75454033	0.0292211	0.0777537	Mettl2
ENSMUSG00000020692.15	56.4466382	0.00330085	0.00436039	0.57947447	0.44651839	0.63843251	Nle1
ENSMUSG00000020694.18	431.018331	5.10E-05	0.00230924	0.00047917	0.9825358	1	Tlk2
ENSMUSG00000020695.15	48.514731	0.04705376	0.0059942	56.7744035	4.89E-14	1.21E-12	Mrc2
ENSMUSG00000020696.19	442.133139	-0.0007111	0.0019037	0.13896283	0.70931429	0.85624353	Rffl
ENSMUSG00000020697.18	538.790905	-0.0103184	0.00320988	10.2522625	0.00136517	0.00576525	Lig3
ENSMUSG00000020698.12	11.554036	0.00128719	0.00582859	0.04749706	0.82747726	0.93249935	Cct6b
ENSMUSG00000020700.12	271.720714	0.00549952	0.00342091	2.55210017	0.11014775	0.22645963	Map3k3
ENSMUSG00000020701.13	87.6493233	0.0013762	0.00419124	0.10821545	0.74218504	0.87800736	Tmem132e
ENSMUSG00000020704.14	4.24115484	0.01548856	0.02332794	0.67559026	0.41110935	0.6026742	Asic2
ENSMUSG00000020705.7	771.039558	0.00278945	0.0016365	2.89077353	0.08908814	0.19196354	Ddx42

ENSMUSG00000020706.14	738.21606	-0.0051147	0.00259542	3.91636233	0.04781824	0.11630825	Ftsj3
ENSMUSG00000020707.7	200.926564	0.00351619	0.00263311	1.76112215	0.18448461	0.33686259	Rnf135
ENSMUSG00000020708.13	1131.3316	-0.0067273	0.00213422	9.90173303	0.00165123	0.0068135	Psmc5
ENSMUSG00000020709.16	436.790669	-0.0008122	0.00471934	0.02871115	0.86544769	0.95604183	Adap2
ENSMUSG00000020712.12	2.34703529	0.03233981	0.01908391	2.70984774	0.09973051	0.20974086	Tcam1
ENSMUSG00000020715.10	1114.16436	-0.0193717	0.00349858	30.0291056	4.26E-08	4.66E-07	Ern1
ENSMUSG00000020716.17	472.750264	0.00230932	0.00305101	0.56297051	0.45306585	0.64486218	Nf1
ENSMUSG00000020717.20	428.298143	0.0088849	0.00338137	6.67668489	0.00976821	0.03126841	Pecam1
ENSMUSG00000020718.13	126.467022	-0.007362	0.00335552	4.87686133	0.02721907	0.07339534	Polg2
ENSMUSG00000020719.15	9102.04094	-0.0018254	0.00212312	0.7328955	0.39194625	0.5832089	Ddx5
ENSMUSG00000020720.14	1353.53422	-0.0047087	0.00197707	5.6459206	0.01749603	0.05098361	Psmd12
ENSMUSG00000020721.17	368.298268	-0.0028956	0.00378448	0.57670749	0.44760578	0.63948331	Helz
ENSMUSG00000020722.6	0.89862072	0.04078429	0.01614534	6.99965754	0.00815253	0.02683679	Cacng1
ENSMUSG00000020723.4	4.46446907	0.01393224	0.01880336	0.8172456	0.36598672	0.55583791	Cacng4
ENSMUSG00000020728.18	17.1583823	-7.72E-05	0.00907788	6.76E-05	0.99343858	1	Cep112
ENSMUSG00000020732.14	8.5441093	0.05092658	0.00764709	42.214314	8.18E-11	1.33E-09	Rab37
ENSMUSG00000020733.4	3153.51398	-0.0007897	0.00332005	0.05644728	0.81220193	0.92285359	Nherf1
ENSMUSG00000020734.14	1.93569285	0.02349106	0.01244548	3.53558201	0.06006525	0.14005922	Grin2c
ENSMUSG00000020736.13	337.981692	0.00185364	0.00308964	0.36547599	0.54548063	0.72856369	Nt5c
ENSMUSG00000020737.13	234.57356	0.00817738	0.00407643	4.05414042	0.04406315	0.10880635	Jpt1
ENSMUSG00000020738.17	1065.30974	0.00285811	0.00262949	1.17919384	0.27752017	0.45632953	Sumo2
ENSMUSG00000020739.12	292.990272	-0.0025021	0.0032581	0.60100072	0.43819646	0.63004765	Nup85
ENSMUSG00000020740.14	245.325144	-0.0046442	0.00579116	0.63799194	0.42443888	0.61606209	Gga3
ENSMUSG00000020741.18	4512.87371	-0.0117015	0.00225016	26.9589476	2.08E-07	2.04E-06	Cluh
ENSMUSG00000020743.16	793.380463	-0.0019128	0.00252848	0.56283325	0.45312093	0.64490166	Mif4gd
ENSMUSG00000020744.14	216.132674	-0.0036857	0.00292435	1.56576103	0.21082368	0.37288994	Slc25a19
ENSMUSG00000020745.16	1666.98088	-0.0017629	0.00326689	0.2812553	0.59587962	0.77087481	Pafah1b1
ENSMUSG00000020747.19	628.594686	-0.0031185	0.00305197	1.03172919	0.30975288	0.49497502	Tmem94
ENSMUSG00000020752.12	234.851378	-0.0112478	0.00318009	12.6024431	0.00038524	0.00189909	Recql5
ENSMUSG00000020755.10	392.581116	-0.000132	0.00351424	0.00140019	0.97015082	1	Sap30bp
ENSMUSG00000020758.16	14.7273351	-0.0036548	0.00715767	0.25366125	0.6145088	0.78612597	Itgb4
ENSMUSG00000020766.5	888.54657	-0.0011673	0.0042886	0.0746279	0.78471403	0.90664569	Galk1
ENSMUSG00000020770.14	156.237135	0.00560448	0.00327112	2.87276429	0.09009001	0.19364683	Unk
ENSMUSG00000020773.12	91.7042482	0.0033778	0.00422315	0.63397685	0.42590032	0.61748739	Trim47
ENSMUSG00000020774.10	211.039082	-0.004854	0.00341738	1.9960408	0.15771069	0.29841672	Aspa
ENSMUSG00000020775.14	912.740311	-0.0157798	0.00279359	31.8541477	1.66E-08	1.94E-07	Mrpl38
ENSMUSG00000020776.19	211.877048	-0.0102768	0.00593903	2.66777635	0.102399	0.21392231	Fbf1
ENSMUSG00000020777.17	39881.5645	-0.0068245	0.0035239	3.57901299	0.05851391	0.13711963	Acox1
ENSMUSG00000020778.13	57.7538279	-0.0060102	0.00556292	1.11553759	0.29088136	0.47237178	Ten1
ENSMUSG00000020780.16	772.461177	-0.0064634	0.0022047	8.64761893	0.00327489	0.0123153	Srp68
ENSMUSG00000020781.15	102.818521	-0.0024534	0.00322474	0.57252062	0.44925902	0.640836	Tsen54
ENSMUSG00000020782.19	407.629061	-0.0142573	0.00440909	10.5260168	0.00117706	0.00506514	Llgl2
ENSMUSG00000020783.15	323.190977	-0.0025754	0.00214565	1.42977677	0.23180087	0.39947796	Ncbp3
ENSMUSG00000020785.18	12.0449872	0.05619987	0.00699939	60.8992877	6.01E-15	1.70E-13	Camkk1
ENSMUSG00000020787.15	2.47791963	0.03327161	0.01120021	8.45480902	0.00364079	0.01347643	P2rx1
ENSMUSG00000020788.16	66.4917911	0.06320387	0.00672995	74.3381199	6.58E-18	2.64E-16	Atp2a3
ENSMUSG00000020790.15	660.90429	0.00881229	0.00242447	13.0523894	0.0003029	0.00153803	Ankfy1
ENSMUSG00000020792.16	515.441183	0.00127517	0.00250909	0.26185632	0.60884769	0.78134184	Exoc7
ENSMUSG00000020793.6	1.27513298	-0.0359942	0.01325878	7.7534357	0.00536105	0.01876384	Galr2
ENSMUSG00000020794.14	1035.96276	-0.008199	0.00189908	18.6344368	1.58E-05	0.00010818	Ube2g1
ENSMUSG00000020798.15	3.8581887	0.04015848	0.0105509	13.4254545	0.00024823	0.00128961	Spns3
ENSMUSG00000020799.17	1.60823901	-0.0197255	0.02663057	0.70412207	0.40140207	0.59301405	Tekt1
ENSMUSG00000020801.9	111.369727	-0.0098042	0.00416673	5.49133021	0.019111	0.05479601	Med31
ENSMUSG00000020802.9	208.376251	-0.0099831	0.00333081	8.82491704	0.00297145	0.01132955	Ube2o
ENSMUSG00000020803.4	979.536479	-0.0018692	0.00290909	0.41322698	0.52033614	0.70612958	Txndc17
ENSMUSG00000020805.15	38.9220804	0.04448513	0.0079931	29.1652399	6.65E-08	7.07E-07	Slc13a5
ENSMUSG00000020806.16	197.407447	0.02267258	0.00361099	37.3040761	1.01E-09	1.40E-08	Rhbdf2
ENSMUSG00000020807.16	47.0356388	0.00898563	0.01017186	0.75026893	0.3863911	0.57782275	4933427D14Rik
ENSMUSG00000020808.4	5.5139366	-0.0028556	0.0112256	0.06148944	0.80415712	0.91805563	Pimreg
ENSMUSG00000020810.6	236.91343	0.04797496	0.0042945	116.684344	3.37E-27	4.31E-25	Cygb
ENSMUSG00000020811.17	10.9092128	0.02159106	0.00599592	12.4815537	0.00041099	0.00201112	Wscd1

ENSMUSG00000020814.14	74.3883441	0.01871659	0.00473619	15.1797074	9.77E-05	0.00055945	Mxra7
ENSMUSG00000020817.17	1604.67976	-0.0035083	0.0039164	0.7864832	0.37516565	0.56624157	Rabep1
ENSMUSG00000020818.17	241.019208	0.00317568	0.0030021	1.10823336	0.2924663	0.47439035	Mfsd11
ENSMUSG00000020821.18	3225.68353	-0.0073656	0.00255445	8.3156593	0.00393047	0.01439505	Kif1c
ENSMUSG00000020823.17	268.737798	0.00991136	0.00298911	10.8478687	0.0009891	0.00435076	Sec14l1
ENSMUSG00000020826.10	7.57677506	0.10733648	0.01546727	45.0633401	1.91E-11	3.40E-10	Nos2
ENSMUSG00000020827.19	931.131959	-0.0040568	0.00322316	1.58485511	0.20806274	0.36919709	Mink1
ENSMUSG00000020828.14	127.143526	0.02094413	0.0036341	32.8992738	9.71E-09	1.17E-07	Pld2
ENSMUSG00000020829.10	685.084917	-0.0113671	0.00282366	16.0061104	6.31E-05	0.00037883	Slc46a1
ENSMUSG00000020830.4	59.4106665	-0.0112369	0.00580903	3.69226203	0.05466543	0.12986894	Vmo1
ENSMUSG00000020831.19	152.073029	0.00205952	0.00397447	0.27126328	0.60248561	0.77587909	0610010K14Rik
ENSMUSG00000020832.15	248.995007	-0.0050818	0.00179101	8.06977016	0.00450098	0.01616324	Eral1
ENSMUSG00000020834.18	79.8533386	8.73E-05	0.00327852	0.00070698	0.97878742	1	Dhrs13
ENSMUSG00000020836.16	26.3216914	0.00065618	0.0139236	-0.3802026	1	1	Coro6
ENSMUSG00000020838.13	7.08145713	0.00914183	0.00751319	1.41876704	0.23360645	0.40173837	Slc6a4
ENSMUSG00000020839.17	18.8978985	0.00847715	0.01608371	0.36970049	0.54316757	0.72659474	Tmigd1
ENSMUSG00000020840.11	762.151045	-0.0005251	0.00267028	0.03796301	0.84551744	0.94426255	Blmh
ENSMUSG00000020841.6	689.116626	0.008638	0.00291659	8.55968981	0.00343688	0.01283443	Cpd
ENSMUSG00000020843.16	237.779407	-0.0109548	0.00356386	9.56423447	0.00198405	0.0080281	Timm22
ENSMUSG00000020844.7	42.6079881	0.01457729	0.00465323	9.77565575	0.00176838	0.00724613	Nxn
ENSMUSG00000020846.7	61.7471219	0.01661431	0.00603286	7.26852774	0.00701733	0.02366562	Rflnb
ENSMUSG00000020847.16	39.3730657	0.00045705	0.00372365	0.01512967	0.90210496	0.9768179	Rph3al
ENSMUSG00000020848.8	3.16428174	0.02081023	0.00949791	4.78558221	0.02869894	0.07664968	Doc2b
ENSMUSG00000020849.14	4897.36535	-0.0024836	0.00270528	0.83250463	0.36154929	0.55119265	Ywhae
ENSMUSG00000020850.15	3143.81946	-0.003823	0.00269597	1.99926972	0.15737501	0.29797279	Prpf8
ENSMUSG00000020857.12	6030.78656	-0.0209875	0.0034316	37.9260464	7.35E-10	1.04E-08	Nme2
ENSMUSG00000020859.18	1815.3127	0.00626208	0.00263702	5.57629477	0.0182052	0.05268562	Spag9
ENSMUSG00000020863.16	5392.06572	0.00084597	0.00422015	0.0393464	0.84276398	0.94229303	Luc7l3
ENSMUSG00000020864.14	931.292794	-0.005147	0.00254352	4.08954758	0.04314919	0.10706047	Ankrd40
ENSMUSG00000020865.17	3902.2555	0.00603847	0.00386306	2.42005384	0.11979081	0.24187879	Abcc3
ENSMUSG00000020866.18	13.6592131	-0.0065113	0.01450245	0.15854907	0.69049588	0.8431396	Cacna1g
ENSMUSG00000020867.14	2.08218923	-0.0201077	0.03514721	0.46906993	0.49341536	0.68088548	Spata20
ENSMUSG00000020868.16	300.0372	0.00091348	0.0022871	0.15934847	0.68975707	0.84262756	Xylt2
ENSMUSG00000020869.9	1406.71393	0.00326694	0.00307511	1.11252532	0.29153366	0.47323557	Lrrc59
ENSMUSG00000020871.9	4.63540296	0.04578499	0.00716882	40.7983312	1.69E-10	2.62E-09	Dlx4
ENSMUSG00000020872.5	1.42759829	0.01445807	0.03909859	0.42574538	0.51408404	0.7007968	Tac4
ENSMUSG00000020873.16	1031.63837	-0.0109837	0.00387212	7.93923453	0.00483743	0.01718825	Slc35b1
ENSMUSG00000020875.10	2.78904434	0.00593638	0.03003026	0.01885021	0.89079679	0.97040936	Hoxb9
ENSMUSG00000020876.15	133.201025	-0.002873	0.00266996	1.15310159	0.28290082	0.46288579	Snx11
ENSMUSG00000020877.12	557.453581	-0.0110856	0.00319814	12.1330904	0.00049535	0.00237562	Scrn2
ENSMUSG00000020878.7	17.4096592	-0.0008172	0.00528951	0.02386776	0.87722187	0.96246709	Lrrc46
ENSMUSG00000020882.19	11.790225	0.02681518	0.00737228	12.9750451	0.00031567	0.00159465	Cacnb1
ENSMUSG00000020883.19	667.131837	-0.0109006	0.00324346	11.2543113	0.00079438	0.00359208	Fbxl20
ENSMUSG00000020884.16	8851.67461	-0.0039034	0.00543113	0.5198478	0.47090661	0.66178628	Asgr1
ENSMUSG00000020886.18	67.6244487	0.03278154	0.00745398	18.041668	2.16E-05	0.00014351	Dlg4
ENSMUSG00000020888.17	100.755336	0.01600801	0.00374365	18.4759707	1.72E-05	0.00011672	Dvl2
ENSMUSG00000020889.12	1059.85809	0.00354603	0.00778029	0.19535557	0.65849611	0.81998253	Nr1d1
ENSMUSG00000020890.12	2.26317531	-0.0001166	0.02463255	0.00057285	0.980905	1	Gucy2e
ENSMUSG00000020891.12	1.10504618	0.06364355	0.01844603	11.2123337	0.00081255	0.00366302	Alox8
ENSMUSG00000020892.11	3.15684378	0.04949983	0.01289824	11.4825446	0.00070253	0.0032212	Aloxe3
ENSMUSG00000020893.18	396.524641	0.00966479	0.00699861	1.99685952	0.1576255	0.29830768	Per1
ENSMUSG00000020894.18	468.422733	0.00591677	0.00386107	2.38131275	0.12279432	0.24665533	Vamp2
ENSMUSG00000020895.15	23.6464934	0.0050119	0.00579499	0.74915037	0.38674536	0.57824254	Tmem107
ENSMUSG00000020897.13	15.3823619	0.02948528	0.01010958	8.10993628	0.00440233	0.01587665	Aurkb
ENSMUSG00000020898.19	125.627199	0.00665353	0.00507687	1.70225506	0.19199333	0.34701104	Ctc1
ENSMUSG00000020899.17	172.664407	0.0011162	0.00317521	0.12272559	0.72609697	0.86776026	Pfas
ENSMUSG00000020900.16	295.802916	0.00351919	0.00427294	0.65885445	0.41696466	0.60819594	Myh10
ENSMUSG00000020901.14	38.4526833	0.06862508	0.0066245	98.3711691	3.47E-23	2.61E-21	Pik3r5
ENSMUSG00000020902.13	215.086367	0.01812164	0.00357894	25.0986835	5.45E-07	4.92E-06	Ntn1
ENSMUSG00000020903.14	295.348992	-0.0022598	0.00329217	0.47157454	0.4922637	0.67997256	Stx8
ENSMUSG00000020905.12	7.74407845	0.04628143	0.01269234	11.5772359	0.00066764	0.00308522	Usp43

ENSMUSG00000020908.16	2.80328039	0.0410334	0.02498119	1.62428012	0.20249602	0.36165918	Myh3
ENSMUSG00000020910.17	343.29549	0.00175813	0.00258677	0.46372747	0.49588707	0.68308691	Adprm
ENSMUSG00000020911.15	31.7035014	0.00702067	0.00651715	1.20695504	0.27193601	0.44921436	Krt19
ENSMUSG00000020914.18	127.689278	0.03600979	0.00923663	14.0940808	0.00017389	0.00094393	Top2a
ENSMUSG00000020917.18	8773.51814	0.00472506	0.00488269	0.91070358	0.33992781	0.52764312	Acly
ENSMUSG00000020918.15	307.868607	-0.0069199	0.00216149	10.2915455	0.00133641	0.00566103	Kat2a
ENSMUSG00000020919.12	1070.03319	-0.0046151	0.00274554	2.79877783	0.09433619	0.20092229	Stat5b
ENSMUSG00000020921.7	88.2395524	-0.0060609	0.00308123	3.8168697	0.05073895	0.12205291	Tmem101
ENSMUSG00000020922.12	596.893006	0.00266038	0.00332251	0.62029197	0.43093881	0.62245837	Lsm12
ENSMUSG00000020923.18	948.635728	-0.0021106	0.00185597	1.28704939	0.25659242	0.42976771	Ubtf
ENSMUSG00000020925.18	246.384472	-0.004211	0.00239276	3.05080377	0.0806972	0.17736689	Ccdc43
ENSMUSG00000020926.17	227.190751	0.03820973	0.00611535	38.874357	4.52E-10	6.58E-09	Adam11
ENSMUSG00000020928.15	1.08698935	-0.0145652	0.02166482	0.55253394	0.45728417	0.64899696	Higd1b
ENSMUSG00000020929.15	467.723188	0.00209138	0.00371361	0.31977548	0.5717426	0.75109285	Eftud2
ENSMUSG00000020930.10	1.91901184	0.00703199	0.01087007	0.4460534	0.50421511	0.69112138	Ccdc103
ENSMUSG00000020932.15	12.4603932	0.02555698	0.00723956	11.6609648	0.00063825	0.0029651	Gfap
ENSMUSG00000020935.9	317.953834	-0.0148078	0.00263994	31.4857389	2.01E-08	2.32E-07	Dcakd
ENSMUSG00000020936.9	1325.18232	-0.0037326	0.00381062	0.95283226	0.32899942	0.51591659	Nmt1
ENSMUSG00000020937.15	15.0457213	0.01069066	0.00731516	2.10801953	0.14652885	0.28254001	Plcd3
ENSMUSG00000020941.8	76.6990334	0.04051241	0.00412859	90.4898321	1.86E-21	1.13E-19	Map3k14
ENSMUSG00000020946.14	1140.61968	-0.0014852	0.0020258	0.54394574	0.46080207	0.65205649	Gosr2
ENSMUSG00000020948.10	150.660954	0.00403525	0.00304268	1.73189004	0.1881697	0.3418711	Klhl28
ENSMUSG00000020949.10	547.846318	-0.0096344	0.00368176	6.73472011	0.00945541	0.03045824	Fkbp3
ENSMUSG00000020950.11	11.1628515	0.02344174	0.02864721	1.21081995	0.27116979	0.44807417	Foxg1
ENSMUSG00000020952.11	736.323028	-0.0032637	0.00230282	1.99095219	0.15824136	0.29913285	Scfd1
ENSMUSG00000020953.18	11.6946969	0.08990484	0.01434555	31.0710734	2.49E-08	2.83E-07	Coch
ENSMUSG00000020954.17	572.882249	-0.0032965	0.00254759	1.65751769	0.19793881	0.35534974	Strn3
ENSMUSG00000020955.10	180.490275	-0.0038748	0.00325944	1.40660203	0.2356213	0.40423072	Ap4s1
ENSMUSG00000020956.15	249.575195	0.00355423	0.00303775	1.35468429	0.24446087	0.41509446	Dtd2
ENSMUSG00000020961.16	227.862096	-0.0145309	0.00393168	13.4724384	0.00024209	0.00126272	Ston2
ENSMUSG00000020962.15	669.983102	0.00661619	0.00325819	4.01575897	0.04507693	0.11084075	Gtf2a1
ENSMUSG00000020964.16	3573.65829	-0.0002097	0.00408272	0.00253362	0.9598554	1	Sel1l
ENSMUSG00000020973.8	55.6404863	-0.0013193	0.00362774	0.13037097	0.71804771	0.86190861	Dnaaf2
ENSMUSG00000020974.7	13.0839118	0.03296567	0.00786609	16.3599738	5.24E-05	0.00031948	Pole2
ENSMUSG00000020978.11	787.695147	-0.0056049	0.00220723	6.45437772	0.01106793	0.03476471	Klhdc2
ENSMUSG00000020982.10	789.584098	0.00133841	0.0033207	0.16271226	0.68667159	0.84051254	Nemf
ENSMUSG00000020986.14	1611.74368	-0.0033322	0.00251101	1.73510518	0.18776023	0.3412323	Sec23a
ENSMUSG00000020988.10	1145.93158	-0.0023072	0.00258295	0.79575101	0.37236679	0.56309829	L2hgdh
ENSMUSG00000020990.6	11.0323243	0.01762962	0.00823522	4.34621628	0.03709127	0.09450519	Cdkl1
ENSMUSG00000020993.10	541.290069	-0.0013624	0.00202018	0.45088078	0.50191698	0.68862858	Trappc6b
ENSMUSG00000020994.6	1151.41711	-0.0034402	0.00343207	1.00315642	0.31654795	0.50199018	Pnn
ENSMUSG00000021000.18	5865.04068	-0.0062404	0.00303899	4.14106712	0.04185478	0.10439792	Mia2
ENSMUSG00000021003.11	168.479273	0.0227625	0.00346505	40.9726537	1.54E-10	2.41E-09	Galc
ENSMUSG00000021007.15	44.2162848	0.00775142	0.00408408	3.51093827	0.06096492	0.14180657	Spata7
ENSMUSG00000021009.16	329.192045	0.00583324	0.0023972	5.96576516	0.01458627	0.043904	Ptpn21
ENSMUSG00000021010.10	11.747019	0.04684221	0.01112193	18.3196529	1.87E-05	0.00012575	Npas3
ENSMUSG00000021012.17	1053.35382	-0.0042939	0.00183446	5.50330236	0.0189806	0.05448836	Zc3h14
ENSMUSG00000021013.17	91.8524086	0.0133051	0.00351957	14.0622478	0.00017686	0.00095828	Ttc8
ENSMUSG00000021018.9	156.249423	-0.0051701	0.00396147	1.71375203	0.19049929	0.34498742	Polr2h
ENSMUSG00000021022.10	274.627678	0.00243373	0.00318053	0.57128073	0.44975044	0.64121165	Ppp2r3c
ENSMUSG00000021023.15	210.554237	-0.0069992	0.00267607	6.7514736	0.00936703	0.03023549	Prorp
ENSMUSG00000021024.15	2426.70724	-0.0068341	0.00284172	5.72638232	0.01671188	0.04906838	Psma6
ENSMUSG00000021025.9	817.477851	0.00672373	0.00422161	2.53211317	0.11155074	0.22864654	Nfkbia
ENSMUSG00000021027.19	226.692516	0.00533736	0.00412934	1.66416617	0.19704175	0.35406268	Ralgapa1
ENSMUSG00000021028.9	76.0363553	0.0090527	0.00372298	5.79021133	0.01611565	0.04760222	Mbip
ENSMUSG00000021032.14	3.16033013	0.0014821	0.00802172	0.03482556	0.85196165	0.94872408	Ngb
ENSMUSG00000021033.12	14011.89	-0.0050778	0.0032069	2.49062571	0.11452618	0.23332596	Gstz1
ENSMUSG00000021036.11	447.539654	0.01591111	0.00315558	25.055722	5.57E-07	5.03E-06	Sptlc2
ENSMUSG00000021037.8	1429.18699	-0.0127816	0.00275893	20.707378	5.35E-06	4.03E-05	Ahsa1
ENSMUSG00000021038.18	225.824982	-0.0016924	0.0021789	0.60709471	0.4358838	0.62737264	Vipas39
ENSMUSG00000021039.10	1033.68643	-0.0019419	0.00308815	0.39616068	0.52907873	0.71364812	Snw1

ENSMUSG00000021040.16	727.910917	-0.0107213	0.00390166	7.5994844	0.0058385	0.02019162	Slirp
ENSMUSG00000021044.16	223.330185	-0.0073309	0.00203581	13.0173054	0.00030863	0.00156274	Adck1
ENSMUSG00000021047.8	17.3870451	0.04657314	0.01284082	11.538024	0.00068187	0.00314298	Nova1
ENSMUSG00000021048.8	3574.94576	-0.0001727	0.00285056	0.00369089	0.95155615	1	Mthfd1
ENSMUSG00000021051.11	1120.45738	-0.0055258	0.00405054	1.84697922	0.17413514	0.32228118	Ppp2r5e
ENSMUSG00000021054.7	1568.32532	0.00545031	0.00224198	5.73328265	0.01664634	0.04890639	Sgpp1
ENSMUSG00000021057.16	35.807535	0.01262397	0.00605652	4.18206971	0.04085378	0.1023884	Akap5
ENSMUSG00000021061.16	35.6342866	-0.0193337	0.0065535	8.76323819	0.00307363	0.01165695	Sptb
ENSMUSG00000021062.16	28.3463132	0.05689078	0.01419413	-31.991528	1	1	Rab15
ENSMUSG00000021065.17	114.834394	0.00947762	0.00344018	7.57589524	0.0059154	0.02040969	Fut8
ENSMUSG00000021066.10	50.3388197	-0.0013303	0.00566911	0.05648427	0.81214156	0.92282945	Atl1
ENSMUSG00000021067.9	282.146335	0.00675323	0.00197136	11.5305263	0.00068463	0.0031526	Sav1
ENSMUSG00000021068.18	123.929112	0.02480211	0.00562487	18.8297147	1.43E-05	9.85E-05	Nin
ENSMUSG00000021069.18	8203.40809	-0.0136388	0.0039453	11.8160827	0.00058721	0.00275238	Pygl
ENSMUSG00000021070.7	5.11297484	0.01726703	0.00966503	3.42232287	0.06432045	0.14799162	Bdkrb2
ENSMUSG00000021071.18	31.3198928	0.03682576	0.0138758	6.73973708	0.00942885	0.03040176	Trim9
ENSMUSG00000021072.13	896.226666	0.00295553	0.00224017	1.7211793	0.18954131	0.34374507	Tmx1
ENSMUSG00000021076.7	863.883663	-0.0015627	0.00292871	0.28540509	0.59318022	0.76874458	Actr10
ENSMUSG00000021079.17	506.781751	-0.0240318	0.00320146	56.3915874	5.94E-14	1.46E-12	Timm9
ENSMUSG00000021081.13	4.14234122	0.01873699	0.03453321	0.66794444	0.41376919	0.60514417	Serpina1f
ENSMUSG00000021086.6	2.33802185	-0.0384408	0.04311999	-1.1941188	1	1	Ccdc175
ENSMUSG00000021087.19	90.9156691	0.02728567	0.00540388	25.0555158	5.57E-07	5.03E-06	Rtn1
ENSMUSG00000021090.17	2.37014038	0.00584532	0.01444466	0.14742605	0.70100735	0.85064359	Lrrc9
ENSMUSG00000021091.9	7221.90961	-0.0024607	0.0060173	0.1626047	0.68676967	0.84055304	Serpina3n
ENSMUSG00000021094.11	619.101656	0.0157269	0.00304665	26.5607086	2.55E-07	2.46E-06	Dhrs7
ENSMUSG00000021096.12	2227.35378	0.00183581	0.0021117	0.74610435	0.38771243	0.57921113	Ppm1a
ENSMUSG00000021097.16	2277.06182	-0.013167	0.0043666	8.90892891	0.0028378	0.01089925	Clmn
ENSMUSG00000021098.16	2.01140335	0.01404667	0.02398832	0.81942896	0.36534718	0.55508107	4930447C04Rik
ENSMUSG00000021102.5	1555.21558	-0.017493	0.00378488	21.246147	4.04E-06	3.12E-05	Glrx5
ENSMUSG00000021103.13	156.020567	0.00398391	0.00312527	1.61660064	0.20356643	0.36326836	Mnat1
ENSMUSG00000021108.19	98.3001448	0.0225414	0.00433989	25.8045135	3.78E-07	3.53E-06	Prkch
ENSMUSG00000021109.14	917.665899	0.01039329	0.00236864	18.6861541	1.54E-05	0.00010555	Hif1a
ENSMUSG00000021111.16	1955.0884	-0.0034964	0.00218898	2.50878987	0.11321284	0.23128956	Papola
ENSMUSG00000021112.10	442.015273	0.00156815	0.00319557	0.23337091	0.62903553	0.79765764	Pals1
ENSMUSG00000021113.6	150.013486	0.00354032	0.00351108	0.99950906	0.31742933	0.50298207	Snapc1
ENSMUSG00000021114.10	752.698616	-0.000863	0.00275254	0.09819961	0.75400097	0.88618033	Atp6v1d
ENSMUSG00000021115.16	108.396722	0.00702761	0.00248031	8.09771548	0.00443211	0.01596453	Vrk1
ENSMUSG00000021116.11	1164.51265	-0.0102955	0.00293174	11.9974084	0.00053275	0.00253253	Eif2s1
ENSMUSG00000021118.8	10.119621	0.00387569	0.00703034	0.30127829	0.58308215	0.76048306	Plek2
ENSMUSG00000021120.12	98.1437506	0.00608437	0.00292586	4.29572967	0.0382082	0.09680723	Pigh
ENSMUSG00000021123.13	3.76364999	0.08085197	0.01123018	39.6493767	3.04E-10	4.53E-09	Rdh12
ENSMUSG00000021124.14	1043.34297	-0.0078522	0.00286425	7.47234618	0.00626538	0.02144469	Vti1b
ENSMUSG00000021125.7	7.93223992	0.06505035	0.00723617	77.6737654	1.22E-18	5.30E-17	Arg2
ENSMUSG00000021127.9	2567.30148	0.00579408	0.00399087	2.02434517	0.15479564	0.29422273	Zfp36l1
ENSMUSG00000021130.9	5.95896081	0.01639591	0.01039526	2.42879455	0.11912447	0.24090455	Galnt16
ENSMUSG00000021131.15	565.80483	-0.0004132	0.00342324	0.01440713	0.90445961	0.97793089	Erh
ENSMUSG00000021133.10	893.942714	0.004144	0.00297717	1.91237774	0.16669937	0.31137811	Susd6
ENSMUSG00000021134.18	4638.8705	0.00227649	0.00241431	0.89396073	0.34440624	0.53266659	Srsf5
ENSMUSG00000021135.9	9513.24009	-0.0069932	0.00562376	1.55346249	0.21262501	0.37503541	Slc10a1
ENSMUSG00000021136.14	1661.93438	0.00630335	0.0031187	4.16342272	0.04130586	0.10330187	Smoc1
ENSMUSG00000021139.18	149.432542	-0.0157573	0.00392532	15.4488329	8.48E-05	0.0004927	Gm20498
ENSMUSG00000021140.10	644.411698	-0.0025668	0.00368527	0.48481417	0.48624975	0.67494625	Pcnx
ENSMUSG00000021143.11	317.507557	0.00402157	0.00257214	2.4389161	0.11835798	0.23962158	Pacs2
ENSMUSG00000021144.15	478.967874	-0.001576	0.00280061	0.31839793	0.57257203	0.75163822	Mta1
ENSMUSG00000021147.18	318.605054	-0.0030031	0.00359233	0.66632885	0.41433447	0.60578309	Wdr37
ENSMUSG00000021149.9	768.241622	-0.0137183	0.00287081	22.8229019	1.78E-06	1.47E-05	Gtpbp4
ENSMUSG00000021156.18	1109.25545	0.00198983	0.00370015	0.28527804	0.59326248	0.76874458	Zmynd11
ENSMUSG00000021171.9	659.244433	0.0114912	0.00239423	22.605475	1.99E-06	1.63E-05	Esyt2
ENSMUSG00000021175.16	12.1124748	0.05099963	0.00883654	30.8827025	2.74E-08	3.10E-07	Cdca7l
ENSMUSG00000021176.7	3.43115446	-0.0049693	0.01127835	0.16596011	0.68372743	0.83821379	Efcab11
ENSMUSG00000021177.17	59.195419	0.00811009	0.00388079	4.43429068	0.03522385	0.09071738	Tdp1

ENSMUSG00000021178.10	2328.36066	-0.0040493	0.00273203	2.16350159	0.14132232	0.27481476	Psmc1
ENSMUSG00000021179.9	181.341757	0.00459206	0.00314127	2.08570394	0.14868367	0.28559761	Nrde2
ENSMUSG00000021180.10	55.8180595	0.0121622	0.00452622	7.15049635	0.00749432	0.0249945	Rps6ka5
ENSMUSG00000021182.17	117.796685	0.04165767	0.00556468	52.3758218	4.58E-13	1.01E-11	Ccdc88c
ENSMUSG00000021185.17	681.930085	0.00189084	0.00263833	0.50189413	0.47866904	0.66856204	Dglucy
ENSMUSG00000021186.10	123.477447	0.01442399	0.00417083	12.148872	0.00049118	0.002358	Fbln5
ENSMUSG00000021187.15	69.3110897	0.00984231	0.00449029	4.80143467	0.02843605	0.07609401	Tc2n
ENSMUSG00000021188.15	1102.6155	-0.001758	0.00455008	0.14552586	0.70284821	0.85165054	Trip11
ENSMUSG00000021189.12	360.747108	-0.0003415	0.00271888	0.01538363	0.90129092	0.97646956	Atxn3
ENSMUSG00000021190.15	1418.19461	0.04340238	0.00400821	112.004435	3.57E-26	3.91E-24	Lgmn
ENSMUSG00000021192.17	477.95659	-0.0047264	0.00211397	4.9632674	0.02589124	0.07050812	Golga5
ENSMUSG00000021193.11	587.776887	0.00058137	0.00238461	0.05906415	0.80798125	0.92067471	Pitrm1
ENSMUSG00000021194.7	4.51594773	0.00148704	0.0193338	0.0047699	0.94493831	0.9995208	Chga
ENSMUSG00000021196.15	114.499335	0.0413703	0.00617268	42.6402918	6.58E-11	1.09E-09	Pfkp
ENSMUSG00000021198.17	10.6622141	0.03930982	0.01058323	11.7789298	0.00059905	0.00280063	Unc79
ENSMUSG00000021200.15	68.2759338	0.04095452	0.00765727	25.7998208	3.79E-07	3.54E-06	Asb2
ENSMUSG00000021203.16	50.0142987	0.00690711	0.00418753	2.71851387	0.09919033	0.20885031	Otub2
ENSMUSG00000021207.10	26.4523757	-0.0612054	0.01457177	18.1609621	2.03E-05	0.00013563	Akr1c21
ENSMUSG00000021208.10	222.613114	0.10287811	0.00746233	167.108415	3.17E-38	1.82E-35	Ifi27l2b
ENSMUSG00000021209.13	122.694409	0.01054234	0.00442484	5.67401576	0.017218	0.05030452	Ppp4r4
ENSMUSG00000021210.17	24978.6335	-0.0175006	0.00569617	9.39791391	0.00217232	0.0086812	Akr1c6
ENSMUSG00000021211.5	1275.73123	-0.0084218	0.00406822	4.23299481	0.03964543	0.09984806	Akr1c12
ENSMUSG00000021213.13	2167.308	-0.00972	0.00406065	5.66966597	0.01726074	0.05040385	Akr1c13
ENSMUSG00000021214.15	4.88423047	0.00229142	0.01156565	0.03952917	0.84240399	0.94223647	Akr1c18
ENSMUSG00000021215.16	1658.13872	0.00116621	0.00382161	0.09017903	0.76394969	0.89280137	Net1
ENSMUSG00000021217.8	25.5127063	0.02051137	0.00474125	18.0833002	2.11E-05	0.00014072	Tshz3
ENSMUSG00000021218.11	4954.14814	0.00194643	0.00165284	1.37593917	0.24079407	0.41062605	Gdi2
ENSMUSG00000021219.17	7.31934822	0.0032505	0.0123154	0.05903145	0.80803337	0.92068962	Rgs6
ENSMUSG00000021221.17	19.8892423	-0.0016364	0.00557329	0.0869436	0.7680993	0.89569811	Dpf3
ENSMUSG00000021222.10	60.1519339	0.01127726	0.00460654	5.94929031	0.01472321	0.04421463	Dcaf4
ENSMUSG00000021223.14	47.0658471	0.03133575	0.00482866	40.6559686	1.82E-10	2.80E-09	Papln
ENSMUSG00000021224.16	671.512707	-0.0080612	0.00248499	10.3957093	0.00126308	0.00539596	Numb
ENSMUSG00000021226.8	160.841616	0.01230817	0.00594362	3.8875014	0.04864675	0.11798927	Acot2
ENSMUSG00000021228.16	772.314346	0.05308834	0.00903193	33.2525498	8.09E-09	9.92E-08	Acot3
ENSMUSG00000021234.10	14.9264395	-0.0155109	0.0063454	5.85715602	0.01551384	0.04614825	Fam161b
ENSMUSG00000021235.14	217.791992	-0.014995	0.00315586	22.4677794	2.14E-06	1.74E-05	Coq6
ENSMUSG00000021236.17	4565.40675	0.00466841	0.00289137	2.48116964	0.11521654	0.23455009	Entpd5
ENSMUSG00000021238.12	7919.96135	-0.0046906	0.00258869	3.21731113	0.07286323	0.16354702	Aldh6a1
ENSMUSG00000021240.7	198.955526	7.68E-05	0.00394078	0.00038977	0.98424863	1	Abcd4
ENSMUSG00000021241.8	649.142265	-0.0062742	0.00278914	5.09680991	0.02396989	0.0661315	Isca2
ENSMUSG00000021242.10	1725.66808	0.0235891	0.00337076	48.2277484	3.79E-12	7.43E-11	Npc2
ENSMUSG00000021243.15	487.155506	-0.0089971	0.00334568	7.15731532	0.00746589	0.02491375	Fcf1
ENSMUSG00000021244.16	607.113846	-0.0004921	0.00308458	0.02518393	0.8739096	0.9608443	Ylpm1
ENSMUSG00000021245.16	253.093078	0.00050716	0.00329538	0.02333387	0.87859206	0.96311918	Mlh3
ENSMUSG00000021248.10	2352.25196	0.00085543	0.00199044	0.1837907	0.66813561	0.82668086	Tmed10
ENSMUSG00000021250.14	74.5833872	0.03125145	0.00820852	12.7781534	0.00035069	0.00175019	Fos
ENSMUSG00000021252.12	1401.0175	0.00371938	0.00417206	0.78503428	0.37560589	0.56668845	Erg28
ENSMUSG00000021253.8	37.4456441	0.02378335	0.00585694	15.3729488	8.82E-05	0.00051	Tgfb3
ENSMUSG00000021254.10	233.968855	0.00463917	0.00286147	2.56173637	0.10947828	0.22545663	Gpatch2l
ENSMUSG00000021255.18	24.4464891	0.01934039	0.00575067	10.7229558	0.00105815	0.00461491	Esrrb
ENSMUSG00000021256.6	13.6819361	0.00820587	0.00651334	1.53957465	0.21468113	0.37767354	Vash1
ENSMUSG00000021257.15	74.7733133	-0.0067866	0.00374618	3.28665201	0.06984531	0.15788493	Angel1
ENSMUSG00000021258.11	297.076538	-0.0023723	0.00344767	0.46541654	0.49510336	0.68233656	Ccnk
ENSMUSG00000021259.6	70.1408735	0.06370361	0.00696499	75.4612791	3.73E-18	1.53E-16	Cyp46a1
ENSMUSG00000021260.9	44.3793253	0.04415053	0.00644162	42.1956841	8.26E-11	1.34E-09	Hhipl1
ENSMUSG00000021262.16	106.124694	0.06175568	0.00563445	110.961318	6.03E-26	6.44E-24	Evl
ENSMUSG00000021263.12	4.79274597	0.02124097	0.01005443	4.58247812	0.03230047	0.08453605	Degs2
ENSMUSG00000021264.13	1172.84781	-0.0019927	0.00214727	0.83833013	0.35987479	0.54917228	Yy1
ENSMUSG00000021265.4	41.713452	-0.0193968	0.00497185	15.0591701	0.00010419	0.00059331	Slc25a29
ENSMUSG00000021266.17	325.443032	0.00194938	0.0025314	0.59139683	0.44187948	0.633802	Wars1
ENSMUSG00000021270.14	10459.9153	-0.0217053	0.00529582	14.9490504	0.00011045	0.00062444	Hsp90aa1

ENSMUSG00000021271.6	206.988714	-0.0064401	0.00357001	3.22995031	0.07230288	0.16253677	Zfp839
ENSMUSG00000021273.12	2836.87191	0.01481206	0.00358849	16.4498709	5.00E-05	0.0003063	Fdft1
ENSMUSG00000021275.17	219.468381	-0.0041259	0.00345858	1.42266515	0.23296523	0.40095723	Tecpr2
ENSMUSG00000021276.11	305.092523	-0.0054912	0.00290276	3.64387836	0.0562757	0.13306452	Cinp
ENSMUSG00000021277.17	227.353453	0.00646071	0.00371698	2.90557991	0.0882735	0.19058978	Traf3
ENSMUSG00000021278.8	5.56617616	-0.0085887	0.01229997	-0.0408526	1	1	Amn
ENSMUSG00000021279.6	1185.31651	-0.0072197	0.00290326	6.14241131	0.01319772	0.04022717	Cdc42bpb
ENSMUSG00000021280.10	34.0593162	0.01719784	0.00681278	5.97324734	0.01452451	0.04374043	Exoc3l4
ENSMUSG00000021281.16	588.951747	0.00913422	0.00503878	3.09547641	0.07851017	0.17361155	Tnfaip2
ENSMUSG00000021282.18	6123.83352	-0.009181	0.00318625	8.17046116	0.00425781	0.01542621	Eif5
ENSMUSG00000021285.16	275.811426	-0.0030839	0.00234167	1.69071845	0.19350629	0.34893571	Ppp1r13b
ENSMUSG00000021286.9	691.832322	-0.0061071	0.00308934	3.94670772	0.04696316	0.11472534	Zfyve21
ENSMUSG00000021287.13	57.1963392	-0.007204	0.00454675	2.39558157	0.12167849	0.24491457	Xrcc3
ENSMUSG00000021288.20	659.902261	0.00877867	0.00418266	4.37075934	0.0365607	0.09351789	Klc1
ENSMUSG00000021290.7	2815.93888	-0.0162674	0.00448075	13.3583715	0.00025727	0.00133187	Atp5mj
ENSMUSG00000021294.8	24.1451458	0.00370238	0.00543925	0.4458725	0.50430159	0.69117655	Kif26a
ENSMUSG00000021298.9	12.959721	0.08499776	0.00790516	104.354397	1.69E-24	1.51E-22	Gpr132
ENSMUSG00000021301.10	7.22399599	0.00170989	0.01724775	-0.0014017	1	1	Hecw1
ENSMUSG00000021302.11	286.53891	-0.0074525	0.00279893	7.10072048	0.0077053	0.02557517	Ggps1
ENSMUSG00000021303.15	5.39604341	0.00452523	0.01836041	0.05629008	0.81245873	0.92301198	Gng4
ENSMUSG00000021306.12	63.9852336	0.0542322	0.00681657	57.3332818	3.68E-14	9.37E-13	Gpr137b
ENSMUSG00000021311.10	259.045525	-0.0008924	0.0040608	0.0465579	0.82916485	0.93358231	Mtr
ENSMUSG00000021313.18	157.88437	0.01147169	0.01504655	0.77678486	0.37812625	0.56932561	Ryr2
ENSMUSG00000021314.14	4.10379898	0.03555093	0.01345714	7.18096885	0.00736809	0.0246397	Amph
ENSMUSG00000021318.16	15.405727	0.02852622	0.00688909	16.6905901	4.40E-05	0.0002737	Gli3
ENSMUSG00000021319.8	2.61514197	0.00875378	0.0152029	0.36149285	0.54767831	0.73050617	Sfrp4
ENSMUSG00000021322.9	77.2569989	0.07945211	0.006718	123.712503	9.74E-29	1.55E-26	Aoah
ENSMUSG00000021326.14	327.307722	-0.0046146	0.0020534	5.08569308	0.02412403	0.06643968	Trim27
ENSMUSG00000021327.20	1032.69145	-0.0124028	0.002649	21.57346	3.41E-06	2.67E-05	Zkscan3
ENSMUSG00000021335.14	204.092507	0.00939242	0.00490762	3.5222544	0.06055003	0.1410084	Slc17a1
ENSMUSG00000021336.18	829.068754	0.00136129	0.00466925	0.08495057	0.77069738	0.89722023	Slc17a4
ENSMUSG00000021338.18	389.485918	-0.039686	0.00557611	49.903104	1.62E-12	3.30E-11	Carmil1
ENSMUSG00000021339.5	547.77919	-0.003172	0.00473716	0.45463214	0.50014339	0.68724477	Mrs2
ENSMUSG00000021340.14	4240.19205	0.00249017	0.0039086	0.39993834	0.5271211	0.71206634	Gpld1
ENSMUSG00000021346.15	2.57262313	-0.0239567	0.13626715	0.08613592	0.76914824	0.89621235	Prl8a8
ENSMUSG00000021356.11	18.7757476	0.0885067	0.00765135	112.417239	2.90E-26	3.24E-24	Irf4
ENSMUSG00000021357.15	230.941863	0.00729858	0.00211066	12.0071361	0.00052997	0.00252269	Exoc2
ENSMUSG00000021359.17	3.32345104	-0.006003	0.0214845	0.04355246	0.83468847	0.93707279	Tfap2a
ENSMUSG00000021360.17	535.763804	0.0061377	0.0030897	3.98226535	0.04598168	0.11277155	Gcnt2
ENSMUSG00000021361.8	2507.34829	-0.0082963	0.00293298	7.99994726	0.00467787	0.0166892	Tmem14c
ENSMUSG00000021363.15	4.92835638	0.02006262	0.01259215	2.26687397	0.13216625	0.26123484	Mak
ENSMUSG00000021364.17	12196.6472	-0.0146983	0.00538717	7.24791702	0.00709833	0.02389342	Elovl2
ENSMUSG00000021365.16	177.833826	0.02899544	0.00486912	32.948882	9.46E-09	1.15E-07	Nedd9
ENSMUSG00000021366.9	380.042956	-0.0004179	0.00432946	0.00917611	0.92368575	0.98838817	Hivep1
ENSMUSG00000021367.9	16.2013432	0.02210467	0.00629641	11.9852408	0.00053624	0.00254788	Edn1
ENSMUSG00000021368.16	118.719992	-0.008905	0.00264211	11.4862281	0.00070114	0.0032167	Tbc1d7
ENSMUSG00000021371.6	832.60636	-0.0010409	0.00208162	0.24631246	0.61968364	0.79011701	Mcur1
ENSMUSG00000021373.17	28.6900477	0.01319586	0.01127598	1.23284002	0.26685543	0.44258858	Cap2
ENSMUSG00000021374.11	528.095165	0.00477258	0.00313114	2.21905919	0.13631611	0.26726115	Nup153
ENSMUSG00000021375.12	387.025823	-0.0096292	0.00422219	5.0928861	0.02402418	0.06624977	Kif13a
ENSMUSG00000021376.12	863.348807	-0.0066979	0.00365368	3.28925808	0.06973453	0.15769497	Tpmt
ENSMUSG00000021377.16	1465.26393	0.00501868	0.00388492	1.63354801	0.20121301	0.35991279	Dek
ENSMUSG00000021379.3	88.1932912	0.03806474	0.00591603	37.8682664	7.57E-10	1.07E-08	Id4
ENSMUSG00000021381.5	2.08673095	0.00676053	0.01260341	0.31256199	0.57611228	0.75464108	Barx1
ENSMUSG00000021384.15	10.0281984	0.09389144	0.00997216	73.347695	1.09E-17	4.27E-16	Susd3
ENSMUSG00000021385.11	186.205375	-0.0090993	0.00217995	17.4566438	2.94E-05	0.00018932	Ippk
ENSMUSG00000021388.15	20.8615755	-0.0269958	0.01069181	5.91168396	0.01504079	0.04496793	Aspn
ENSMUSG00000021390.7	75.1589452	0.00315734	0.00562486	0.30163699	0.58285799	0.76037159	Ogn
ENSMUSG00000021391.11	8.0551775	0.00679699	0.00880535	0.5625197	0.45324679	0.64496403	Cenpp
ENSMUSG00000021392.9	334.183453	-0.0057951	0.00364628	2.44925905	0.11758036	0.23831317	Nol8
ENSMUSG00000021395.12	1450.10593	-0.0047417	0.0024712	3.57135877	0.05878419	0.13756166	Spin1

ENSMUSG00000021400.9	838.002331	-0.0116344	0.00337113	11.5017206	0.00069532	0.0031931	Wrnip1
ENSMUSG00000021403.4	3.50936676	0.03349426	0.01448277	5.33091143	0.02095043	0.05916388	Serpinb9b
ENSMUSG00000021408.16	660.802633	0.00372781	0.00286172	1.65327522	0.19851374	0.356165	Ripk1
ENSMUSG00000021411.17	1141.82984	0.00799858	0.00361584	4.88250411	0.02713023	0.07319763	Pxdc1
ENSMUSG00000021413.10	1509.80193	-0.0076805	0.00393959	3.81433414	0.0508158	0.12217548	Prpf4b
ENSMUSG00000021414.9	3.69265597	-0.0061988	0.01210187	0.25607342	0.6128307	0.78483058	Fam217a
ENSMUSG00000021415.14	0.96777138	-0.0212319	0.20950068	0.02874093	0.86537861	0.95604183	Tex56
ENSMUSG00000021416.12	31.9745179	0.03285953	0.01316826	5.53699122	0.01861857	0.05364495	Eci3
ENSMUSG00000021417.16	3120.86808	-0.0196895	0.00284682	48.4965774	3.31E-12	6.54E-11	Eci2
ENSMUSG00000021418.16	240.209163	0.00969391	0.00544008	3.03548991	0.08146196	0.17873132	Rpp40
ENSMUSG00000021420.14	751.472857	-0.0105935	0.0033539	10.0563546	0.00151823	0.00634462	Fars2
ENSMUSG00000021423.7	149.901791	0.06028887	0.00614206	86.958989	1.11E-20	6.15E-19	Ly86
ENSMUSG00000021427.11	2531.26791	-0.0062669	0.00289536	4.58527144	0.03224786	0.0844171	Ssr1
ENSMUSG00000021428.10	217.384937	0.00690531	0.00339033	4.12251084	0.04231623	0.10534838	Riok1
ENSMUSG00000021431.17	668.309984	-0.0057042	0.00418576	1.81547488	0.17785286	0.32749142	Snrnp48
ENSMUSG00000021432.16	304.687138	-0.001491	0.00291268	0.25783659	0.61161035	0.78377909	Slc35b3
ENSMUSG00000021448.9	3.57069139	0.01042835	0.01907263	0.40078285	0.52668524	0.71159982	Shc3
ENSMUSG00000021451.17	55.0879788	0.07157959	0.00658077	108.622416	1.96E-25	1.95E-23	Sema4d
ENSMUSG00000021453.3	345.738096	-0.0253824	0.0087881	7.59007385	0.00586905	0.02028539	Gadd45g
ENSMUSG00000021456.9	10.2138815	-0.0031578	0.01005221	0.07526283	0.7838228	0.90595857	Fbp2
ENSMUSG00000021457.15	180.561478	0.06114851	0.00514143	129.559227	5.12E-30	1.04E-27	Syk
ENSMUSG00000021458.19	403.622677	-0.0122721	0.00316765	15.6420226	7.65E-05	0.00044971	Aopep
ENSMUSG00000021460.18	740.807955	-0.005822	0.00258586	5.10688088	0.02383113	0.06580137	Auh
ENSMUSG00000021461.18	88.0628426	0.00270781	0.00397134	0.47056335	0.49272812	0.68033517	Fancc
ENSMUSG00000021464.16	3.07211968	0.00030643	0.0156067	0.00226604	0.96203271	1	Ror2
ENSMUSG00000021466.13	472.555027	-0.0010443	0.00343261	0.09204773	0.76158986	0.89131623	Ptch1
ENSMUSG00000021468.9	485.872751	0.00316852	0.00160559	3.86938219	0.04917462	0.11895719	Sptlc1
ENSMUSG00000021470.20	280.067312	0.00340655	0.00264116	1.63705935	0.20072941	0.35926574	Ercc6l2
ENSMUSG00000021474.10	2129.47354	0.00065642	0.00353922	0.03436051	0.85294207	0.9493413	Sfxn1
ENSMUSG00000021476.10	1673.6088	-0.0054454	0.00253994	4.53747712	0.03316043	0.0863174	Habp4
ENSMUSG00000021477.10	10146.3538	0.00970979	0.00303999	10.367786	0.00128233	0.00546335	Ctsl
ENSMUSG00000021478.7	2.601952	0.04121281	0.02390593	4.50071838	0.03388062	0.08795908	Drd1
ENSMUSG00000021481.12	97.0299812	-0.0055229	0.00404216	1.79346236	0.18050488	0.33123691	Zfp346
ENSMUSG00000021482.11	1924.26461	-0.0090886	0.00210514	18.4124955	1.78E-05	0.00012022	Prxl2c
ENSMUSG00000021483.9	128.331447	-0.0048883	0.00353775	1.86500723	0.172048	0.31914465	Cdk20
ENSMUSG00000021484.9	4083.44567	-0.0044167	0.00256706	2.93051638	0.08691969	0.18830585	Lman2
ENSMUSG00000021485.15	6.22349203	0.01538312	0.01078605	1.88560797	0.16969813	0.31567948	Mxd3
ENSMUSG00000021486.8	2250.2603	0.00183417	0.0023087	0.63553726	0.42533145	0.61692809	Prelid1
ENSMUSG00000021488.9	1386.54893	-0.0049852	0.00294416	2.85774668	0.0909348	0.19501621	Nsd1
ENSMUSG00000021490.12	81.2599006	0.01260388	0.02125257	0.43595978	0.50907897	0.69575394	Slc34a1
ENSMUSG00000021492.16	6950.51458	-0.003596	0.00466551	0.59394239	0.44089866	0.63289123	F12
ENSMUSG00000021493.16	104.503377	-0.011493	0.00436077	6.96794281	0.00829828	0.0272216	Pdlim7
ENSMUSG00000021494.10	425.082951	-0.0049472	0.00175562	7.92320897	0.00488046	0.01731768	Ddx41
ENSMUSG00000021495.11	791.02041	-0.0100955	0.00496172	4.13894536	0.04190727	0.10448563	Fam193b
ENSMUSG00000021496.13	343.368312	-0.0085805	0.00332906	6.61427199	0.01011647	0.03220415	Pcbd2
ENSMUSG00000021497.11	1378.47694	-0.0101807	0.00231267	19.2188227	1.17E-05	8.15E-05	Txndc15
ENSMUSG00000021499.13	1.44955308	-0.0302705	0.01076749	7.81609625	0.00517829	0.01823458	Catsper3
ENSMUSG00000021500.19	1420.87875	-0.0041061	0.00363611	1.29327162	0.25544591	0.42824274	Ddx46
ENSMUSG00000021501.5	332.095384	-0.0010186	0.00255912	0.15806302	0.69094615	0.84325033	Caml
ENSMUSG00000021504.15	201.908566	-0.0034981	0.00279115	1.57707185	0.20918297	0.37065507	B4galt7
ENSMUSG00000021508.12	57.6838093	0.04191211	0.00713762	30.1624438	3.97E-08	4.37E-07	Cxcl14
ENSMUSG00000021509.6	185.211121	0.00438662	0.00449107	0.94604339	0.33072847	0.51758753	Slc25a48
ENSMUSG00000021510.12	204.598256	0.00330858	0.0039113	0.67816796	0.41021829	0.60188609	Zfp729a
ENSMUSG00000021514.19	171.913391	-0.0099305	0.00287779	11.8812528	0.00056702	0.00266993	Zfp369
ENSMUSG00000021518.5	893.006823	0.00130911	0.00200384	0.42323458	0.51532745	0.70196438	Ptdss1
ENSMUSG00000021519.12	394.409717	-0.0119828	0.00265787	20.0698627	7.47E-06	5.44E-05	Mterf3
ENSMUSG00000021520.5	3018.34173	-0.0116435	0.00319204	13.3719244	0.00025542	0.00132257	Uqcrb
ENSMUSG00000021532.12	121.103204	0.00061325	0.00346502	0.03153363	0.8590551	0.95283438	Fastkd3
ENSMUSG00000021534.9	1.70105598	-0.0309697	0.01796965	3.59398617	0.05798899	0.13624149	Cfap90
ENSMUSG00000021536.8	14.5047728	0.03174686	0.0098008	10.026783	0.0015428	0.00643021	Adcy2
ENSMUSG00000021537.10	699.030763	-0.0020426	0.00325365	0.39147689	0.5315241	0.71620929	Cetn3

ENSMUSG00000021539.10	1667.63832	-0.0007348	0.00444628	0.02662661	0.87037938	0.95888495	Lect2
ENSMUSG00000021540.17	621.360894	0.00640627	0.00247594	6.60657866	0.01016027	0.03233137	Smad5
ENSMUSG00000021541.15	3.05548616	0.01236129	0.07530555	0.11854919	0.73061337	0.87054925	Trpc7
ENSMUSG00000021546.19	4762.75615	-0.0014398	0.00177037	0.65656594	0.41777492	0.60930245	Hnrnpk
ENSMUSG00000021548.11	410.751922	-0.0025648	0.00248309	1.04956483	0.30560733	0.49051069	Ccnh
ENSMUSG00000021549.15	628.514708	0.00456633	0.00322892	1.95977848	0.16153701	0.30373673	Rasa1
ENSMUSG00000021550.7	491.859808	0.00067094	0.00209674	0.10327332	0.74793633	0.88190903	Qng1
ENSMUSG00000021552.8	297.055753	-0.002108	0.00284677	0.55052292	0.45810411	0.64984827	Gkap1
ENSMUSG00000021553.15	2.47518838	0.02562188	0.01211273	4.44322191	0.03504006	0.09036077	Slc28a3
ENSMUSG00000021555.16	557.413169	-0.0105267	0.00367261	8.07344291	0.00449187	0.0161428	Naa35
ENSMUSG00000021556.13	76.5287198	0.01876618	0.005909	9.48246944	0.00207445	0.00834093	Golm1
ENSMUSG00000021557.16	330.115053	-0.0110676	0.00490523	4.8613951	0.02746412	0.07392932	Agtpbp1
ENSMUSG00000021559.15	1457.4411	-0.0031159	0.00331119	0.87521322	0.3495161	0.53769208	Dapk1
ENSMUSG00000021565.17	7.97926095	0.06006145	0.01472763	14.9507246	0.00011036	0.00062411	Slc6a19
ENSMUSG00000021567.16	8.53032341	0.02418618	0.00669777	12.3253292	0.00044685	0.00216194	Nkd2
ENSMUSG00000021569.11	19.7613094	-0.0048183	0.00786908	0.36101585	0.5479426	0.73066268	Trip13
ENSMUSG00000021572.10	21.3419613	0.01333966	0.0066275	3.95989413	0.04659663	0.11393616	Cep72
ENSMUSG00000021573.16	278.444623	0.0001669	0.0047887	0.0012185	0.97215392	1	Tppp
ENSMUSG00000021575.17	7.48174469	0.02794544	0.01028174	6.58860096	0.01026338	0.03258913	Ahrr
ENSMUSG00000021576.7	1523.45006	-0.0032214	0.00222237	2.10277017	0.14703254	0.28327966	Pdcd6
ENSMUSG00000021577.15	8784.59041	-0.0142141	0.00257826	30.0791676	4.15E-08	4.55E-07	Sdha
ENSMUSG00000021578.18	547.568249	-0.0023462	0.00343825	0.46305121	0.49620143	0.68339023	Ccdc127
ENSMUSG00000021579.5	38.0921325	0.01672925	0.0085512	3.31613329	0.06860301	0.15573344	Lrrc14b
ENSMUSG00000021583.8	2001.90769	0.01314273	0.00325024	16.1624833	5.81E-05	0.00035131	Erap1
ENSMUSG00000021585.11	1047.56753	0.0033885	0.00296145	1.28060748	0.25778612	0.43130761	Cast
ENSMUSG00000021587.6	26.130352	0.05319939	0.01500616	11.5400643	0.00068112	0.00314076	Pcsk1
ENSMUSG00000021589.14	119.632943	-0.0101857	0.00394916	6.5680078	0.01038281	0.03290634	Rhobtb3
ENSMUSG00000021590.8	2.02263478	-0.0201657	0.01278697	2.35168606	0.12514758	0.25029517	Spata9
ENSMUSG00000021591.9	701.289476	0.0020572	0.00263135	0.63586772	0.42521113	0.61679149	Glrx
ENSMUSG00000021592.19	172.414041	0.00374884	0.00272765	1.8645172	0.17210435	0.31922407	Arsk
ENSMUSG00000021594.12	1444.845	-0.0419462	0.00459009	78.8632526	6.66E-19	3.03E-17	Srd5a1
ENSMUSG00000021595.19	1447.20668	-0.0077677	0.00189272	16.7460768	4.27E-05	0.00026684	Nsun2
ENSMUSG00000021596.18	11.2827583	0.06500976	0.00977475	39.5845077	3.14E-10	4.66E-09	Mctp1
ENSMUSG00000021597.17	94.5830912	0.0028077	0.00438832	0.3845716	0.53516669	0.71953975	Slf1
ENSMUSG00000021598.10	270.785001	0.00367326	0.00295003	1.55204051	0.21283446	0.3753487	Med10
ENSMUSG00000021604.16	2.85776367	0.02002115	0.06297308	-0.0991363	1	1	Irx4
ENSMUSG00000021606.9	2142.01476	-0.0188008	0.00485338	14.876738	0.00011477	0.00064729	Ndufs6
ENSMUSG00000021607.10	375.389592	-0.0084881	0.00274753	9.53974293	0.0020107	0.00811783	Mrpl36
ENSMUSG00000021608.16	75.1583335	0.02825961	0.00416037	43.7166647	3.80E-11	6.45E-10	Lpcat1
ENSMUSG00000021610.9	1493.03392	-0.0030652	0.0028345	1.16429692	0.28057618	0.46000763	Clptm1l
ENSMUSG00000021611.10	111.22752	-0.0029259	0.00602127	0.23783816	0.62577189	0.7946757	Tert
ENSMUSG00000021612.16	14.5812258	0.01189568	0.03352448	0.56339068	0.4528973	0.64466118	Slc6a18
ENSMUSG00000021613.10	12.0466489	-0.0168686	0.01392224	1.30034158	0.25415084	0.42646569	Hapln1
ENSMUSG00000021614.17	33.437245	0.08739779	0.01283153	34.7395876	3.77E-09	4.85E-08	Vcan
ENSMUSG00000021615.14	113.840058	0.00911575	0.00273676	10.9833237	0.00091935	0.00407668	Xrcc4
ENSMUSG00000021619.7	105.657811	-0.0063066	0.00330418	3.57186573	0.05876625	0.13756166	Atg10
ENSMUSG00000021620.5	2080.69668	-0.0115119	0.00393953	8.43606687	0.00367851	0.01359657	Acot12
ENSMUSG00000021621.16	192.32346	-0.0022255	0.00297737	0.55625218	0.45577425	0.64750581	Zcchc9
ENSMUSG00000021622.4	607.95504	-0.1265317	0.03688202	5.09492566	0.02399594	0.06618738	Ckmt2
ENSMUSG00000021624.10	58.3231574	0.06379364	0.00619239	98.6294684	3.04E-23	2.31E-21	Cd180
ENSMUSG00000021629.11	802.020827	0.00171663	0.00259274	0.4212437	0.51631712	0.70282266	Slc30a5
ENSMUSG00000021635.15	269.709087	-0.0055025	0.00190692	8.26380309	0.00404433	0.01476391	Rad17
ENSMUSG00000021636.17	217.481607	0.00502505	0.00342183	2.12208239	0.14518902	0.28069084	Marveld2
ENSMUSG00000021638.13	393.494259	0.00482202	0.00389055	1.51252995	0.21875344	0.3831231	Ocln
ENSMUSG00000021639.18	201.940102	0.00159103	0.00227994	0.48417975	0.48653513	0.67509437	Gtf2h2
ENSMUSG00000021640.7	18.5485013	0.07038662	0.00670076	98.4345348	3.36E-23	2.54E-21	Naip1
ENSMUSG00000021643.16	127.036827	-0.0093011	0.00424748	4.65115698	0.03103262	0.08177136	Serf1
ENSMUSG00000021645.17	111.151333	0.00404512	0.00367569	1.21270383	0.2707973	0.4477404	Smn1
ENSMUSG00000021646.10	1030.83571	-0.011032	0.00225801	24.0596451	9.34E-07	8.07E-06	Mccc2
ENSMUSG00000021650.8	421.418271	-0.0083483	0.00213028	15.4373505	8.53E-05	0.00049546	Ptcd2
ENSMUSG00000021660.15	2975.09598	0.00048548	0.00280863	0.02986713	0.86279206	0.9546284	Btf3

ENSMUSG00000021661.17	174.927007	-0.0051099	0.00199109	6.57205986	0.0103592	0.03284475	Ankra2
ENSMUSG00000021662.12	14.5116009	0.02752186	0.01066541	5.78551798	0.01615874	0.04768768	Arhgef28
ENSMUSG00000021665.10	360.868742	0.04708021	0.00405251	128.493728	8.75E-30	1.72E-27	Hexb
ENSMUSG00000021666.17	782.034554	-0.0086688	0.00222468	14.974475	0.00010898	0.00061727	Gfm2
ENSMUSG00000021668.16	114.698545	0.01254023	0.00342702	12.9574674	0.00031865	0.00160735	Polk
ENSMUSG00000021669.17	810.511568	0.00797388	0.00249463	10.257197	0.00136152	0.00575191	Cert1
ENSMUSG00000021670.15	3013.43942	0.02102982	0.00486259	18.4136417	1.78E-05	0.00012018	Hmgcr
ENSMUSG00000021671.10	114.625084	-0.0039735	0.00419495	0.88930697	0.34566518	0.53412336	Poc5
ENSMUSG00000021675.5	6.79524589	0.05087503	0.00979495	24.9465615	5.89E-07	5.30E-06	F2rl2
ENSMUSG00000021676.11	6748.42975	0.0018519	0.00481416	0.14342654	0.70489806	0.85334525	Iqgap2
ENSMUSG00000021678.10	8.43622484	0.04273452	0.00812301	26.1348784	3.18E-07	3.01E-06	F2rl1
ENSMUSG00000021681.9	552.692003	-0.0046117	0.00184619	6.15463952	0.01310678	0.03999697	Aggf1
ENSMUSG00000021684.18	30.37659	0.02230113	0.00636572	11.1072458	0.00085991	0.00383763	Pde8b
ENSMUSG00000021686.7	900.729294	-0.0066935	0.00301128	4.84696768	0.02769478	0.07446523	Ap3b1
ENSMUSG00000021687.15	1107.34558	0.00184258	0.00263665	0.48258442	0.48725401	0.67566424	Scamp1
ENSMUSG00000021690.11	422.64609	-0.0053568	0.00313755	2.87588368	0.08991561	0.1934108	Jmy
ENSMUSG00000021692.10	100.323315	-0.0038792	0.00347013	1.25341308	0.2629016	0.43760036	Dimt1
ENSMUSG00000021693.21	228.513187	0.0046252	0.00351737	1.67180212	0.19601733	0.35262487	Kif2a
ENSMUSG00000021694.19	62.8317219	-0.0028162	0.0030648	0.84650279	0.35754357	0.54638088	Ercc8
ENSMUSG00000021696.10	21.9290283	0.03843815	0.00855177	19.8408538	8.42E-06	6.05E-05	Elovl7
ENSMUSG00000021697.13	5.13578851	0.02418363	0.01315812	2.96747214	0.08495451	0.18491483	Depdc1b
ENSMUSG00000021699.18	87.6723818	0.02373847	0.00502327	21.7238173	3.15E-06	2.49E-05	Pde4d
ENSMUSG00000021700.11	41.7899158	0.02037805	0.0158528	1.96667141	0.16080162	0.30278357	Rab3c
ENSMUSG00000021701.9	282.579891	0.01426771	0.00354569	15.3782952	8.80E-05	0.00050868	Plk2
ENSMUSG00000021702.8	5.65493859	0.02489091	0.02160479	1.96799222	0.16066114	0.30256736	Thbs4
ENSMUSG00000021703.10	806.506751	0.00488108	0.00286748	2.86773236	0.09037212	0.1940686	Serinc5
ENSMUSG00000021704.9	320.843001	0.00566173	0.00311396	3.27668582	0.0702707	0.15870963	Mtx3
ENSMUSG00000021706.15	161.227938	0.00655937	0.00305229	4.44895663	0.03492258	0.09013831	Zfyve16
ENSMUSG00000021707.6	773.125964	-6.98E-06	0.00319903	4.62E-06	0.99828584	1	Dhfr
ENSMUSG00000021708.19	23.129963	0.02541089	0.00623709	15.4662777	8.40E-05	0.00048865	Rasgrf2
ENSMUSG00000021709.15	1545.46126	0.00231817	0.00267789	0.72656261	0.39399965	0.58530427	Erbin
ENSMUSG00000021710.12	665.820262	0.00225177	0.00320945	0.48061136	0.48814553	0.67642326	Nln
ENSMUSG00000021711.18	479.305233	-0.0071209	0.00215068	10.8660597	0.00097943	0.00431226	Trappc13
ENSMUSG00000021712.16	314.703396	0.0009735	0.00263241	0.13432845	0.71398571	0.85914325	Trim23
ENSMUSG00000021713.10	151.57383	-0.0002153	0.00225827	0.00883041	0.92513277	0.98912881	Ppwd1
ENSMUSG00000021714.16	23.9565778	0.00167583	0.00642606	0.06171224	0.80380986	0.91805563	Cenpk
ENSMUSG00000021715.13	202.135832	0.00379188	0.00299688	1.59488513	0.20662957	0.36731723	Cwc27
ENSMUSG00000021716.15	671.811271	-0.0023267	0.00376999	0.37707416	0.53917327	0.72316979	Srek1ip1
ENSMUSG00000021719.10	57.5455688	-0.0089926	0.00515293	3.01308292	0.08259506	0.18072986	Rgs7bp
ENSMUSG00000021720.12	19.3210359	0.05312316	0.00661583	59.8765226	1.01E-14	2.77E-13	Rnf180
ENSMUSG00000021721.6	2.83037375	-0.0060451	0.0308923	0.05518267	0.81427865	0.9240142	Htr1a
ENSMUSG00000021725.10	42.8844005	0.04755209	0.00561538	67.1787701	2.48E-16	8.18E-15	Parp8
ENSMUSG00000021728.9	27.6968141	0.06241348	0.00666463	77.0587959	1.66E-18	7.13E-17	Emb
ENSMUSG00000021730.9	10.3069633	0.03233872	0.01671679	4.0460668	0.04427439	0.10916325	Hcn1
ENSMUSG00000021731.11	412.35325	-0.0053527	0.00194797	7.48550259	0.00621977	0.02131331	Mrps30
ENSMUSG00000021732.15	2.22180032	0.01102419	0.02416608	0.25220124	0.61552937	0.7870047	Fgf10
ENSMUSG00000021733.12	269.047798	-6.62E-05	0.00387429	0.00028653	0.98649463	1	Slc4a7
ENSMUSG00000021737.5	1257.50367	-0.0095157	0.00200887	22.3921188	2.22E-06	1.80E-05	Psmd6
ENSMUSG00000021738.6	931.103181	-0.0020221	0.00452295	0.19796919	0.65636508	0.81810636	Atxn7
ENSMUSG00000021743.7	1.25406619	0.0141748	0.03483946	0.35872922	0.54921284	0.73180804	Fezf2
ENSMUSG00000021747.13	16.7183567	-0.1278427	0.00835593	281.640252	3.30E-63	2.59E-59	Cfap20dc
ENSMUSG00000021748.10	3448.5784	-0.0240541	0.00383433	38.9620121	4.32E-10	6.31E-09	Pdhb
ENSMUSG00000021749.7	1.46034235	0.02082989	0.01357104	2.93428919	0.08671683	0.18793559	Fam3d
ENSMUSG00000021750.17	15.0909583	-0.0130608	0.01138278	1.32511293	0.2496765	0.42114495	Fam107a
ENSMUSG00000021751.14	3033.61666	-0.0008925	0.00421755	0.0443468	0.83320971	0.9360364	Acox2
ENSMUSG00000021752.15	154.399561	-0.0041752	0.00235365	3.1395088	0.07641682	0.16998652	Kctd6
ENSMUSG00000021754.18	378.251131	0.0179417	0.00368642	22.4819138	2.12E-06	1.73E-05	Map3k1
ENSMUSG00000021756.13	3378.36032	-0.0052085	0.00369765	1.89690409	0.16842526	0.31385622	Il6st
ENSMUSG00000021758.15	3.63228811	-0.0045387	0.01519502	0.06003797	0.80643594	0.91944843	Ddx4
ENSMUSG00000021759.15	267.366176	-0.0018969	0.00299219	0.40289102	0.52559998	0.71094805	Plpp1
ENSMUSG00000021760.6	28.6532327	0.02918974	0.00626008	20.7028813	5.36E-06	4.03E-05	Gpx8

ENSMUSG00000021763.19	5.76842024	0.0101794	0.00951393	1.07711218	0.29934448	0.48328735	Cspg4b
ENSMUSG00000021764.9	2184.58511	-0.0169148	0.00421355	16.3023476	5.40E-05	0.00032892	Ndufs4
ENSMUSG00000021765.10	121.861842	0.00260549	0.0064132	0.16409726	0.68541194	0.83949976	Fst
ENSMUSG00000021767.21	273.135814	0.00149555	0.00310204	0.23012102	0.63143411	0.79949565	Kat6b
ENSMUSG00000021770.12	643.037147	0.00187284	0.00265961	0.4886819	0.48451587	0.67364084	Samd8
ENSMUSG00000021771.15	1485.89059	-0.0046194	0.0023442	3.90705324	0.04808384	0.1168643	Vdac2
ENSMUSG00000021772.16	354.655008	-0.0076907	0.00231258	11.1082231	0.00085946	0.00383633	Nkiras1
ENSMUSG00000021773.12	69.3410099	-0.0016189	0.00502735	0.10307074	0.74817529	0.88197206	Comtd1
ENSMUSG00000021774.14	577.269018	-0.005824	0.00213882	7.39064081	0.00655641	0.02233062	Ube2e1
ENSMUSG00000021775.12	1507.71708	0.00906144	0.00501973	3.12627865	0.07703941	0.17108119	Nr1d2
ENSMUSG00000021779.20	782.027833	-0.0052417	0.0035357	2.1547255	0.14213184	0.27595667	Thrb
ENSMUSG00000021782.15	101.573788	0.00962846	0.00457703	4.37462416	0.03647787	0.09335804	Dlg5
ENSMUSG00000021785.9	743.703712	-0.0013639	0.00250973	0.28980867	0.59034317	0.7665199	Ngly1
ENSMUSG00000021786.14	603.921102	-0.0037912	0.00238264	2.47664011	0.11554885	0.23502374	Oxsm
ENSMUSG00000021789.15	21.3325842	-0.0269737	0.00704891	14.77231	0.0001213	0.00068088	Sftpa1
ENSMUSG00000021792.16	1256.64652	-0.0061159	0.00265602	5.22988931	0.02220195	0.0620665	Prxl2a
ENSMUSG00000021794.17	18776.5716	-0.0054468	0.00294618	3.38341007	0.06585567	0.15078865	Glud1
ENSMUSG00000021795.11	28.2256833	0.06046401	0.00799336	56.6109944	5.31E-14	1.31E-12	Sftpd
ENSMUSG00000021796.16	705.894169	-0.0045749	0.00274872	2.72417017	0.09883948	0.20827672	Bmpr1a
ENSMUSG00000021798.15	160.129073	-0.0028977	0.01243214	0.04701308	0.82834472	0.93288156	Ldb3
ENSMUSG00000021804.7	8.81355572	0.00876969	0.01200799	0.80403966	0.36988832	0.5603922	Rgr
ENSMUSG00000021806.5	178.968336	-0.0010669	0.00528099	0.03950396	0.84245359	0.94224726	Nid2
ENSMUSG00000021807.7	1610.47047	-0.0083439	0.00279668	8.89152417	0.00286497	0.01098302	Rtraf
ENSMUSG00000021809.11	221.810726	-0.0063523	0.00347439	3.26934183	0.07058594	0.1592691	Nudt13
ENSMUSG00000021810.4	541.928042	-0.0024208	0.00154504	2.43015491	0.11902114	0.24075751	Ecd
ENSMUSG00000021811.8	146.340884	0.00892919	0.0036996	5.62167613	0.01773968	0.05155402	Dnajc9
ENSMUSG00000021814.18	1772.00982	0.00429213	0.00199073	4.5953314	0.03205914	0.08399766	Anxa7
ENSMUSG00000021815.5	8.09781549	-0.000931	0.00665325	0.01939138	0.88925028	0.96954458	Mss51
ENSMUSG00000021816.12	571.521659	0.00488622	0.00230701	4.41384299	0.03564845	0.09160086	Ppp3cb
ENSMUSG00000021819.12	611.373653	-0.0033483	0.00387203	0.73890254	0.39001266	0.58143219	Zswim8
ENSMUSG00000021820.19	212.160472	0.00220216	0.00345825	0.40399303	0.52503426	0.71030504	Camk2g
ENSMUSG00000021822.4	71.1305318	0.05899732	0.00686441	68.3526473	1.37E-16	4.63E-15	Plau
ENSMUSG00000021823.11	665.342087	-0.0084645	0.00368376	5.14478748	0.02331626	0.06463768	Vcl
ENSMUSG00000021824.14	2021.96865	-0.002375	0.00390841	0.36487792	0.54580957	0.72892047	Ap3m1
ENSMUSG00000021830.15	109.640426	0.02207529	0.00413057	28.0606298	1.18E-07	1.21E-06	Txndc16
ENSMUSG00000021831.10	482.097637	0.00491763	0.00330864	2.18973652	0.13893308	0.27124209	Ero1a
ENSMUSG00000021832.9	2022.14858	-0.0072974	0.00273487	6.97383809	0.00827099	0.02714698	Psmc6
ENSMUSG00000021835.16	55.9024625	-0.0094378	0.00693899	1.77727533	0.18248426	0.33385616	Bmp4
ENSMUSG00000021838.18	137.183407	0.0377932	0.00543838	44.544036	2.49E-11	4.33E-10	Samd4
ENSMUSG00000021840.18	1245.8471	-0.0044706	0.0021099	4.44075755	0.03509067	0.09043771	Mapk1ip1l
ENSMUSG00000021843.18	816.67983	-0.0013328	0.00358012	0.13785273	0.71042509	0.85686965	Ktn1
ENSMUSG00000021846.11	1026.4953	-0.0088001	0.00386131	5.19065795	0.02270862	0.06324306	Peli2
ENSMUSG00000021850.15	20.0129981	0.021077	0.00826658	6.24217514	0.01247432	0.03835788	ccdc198
ENSMUSG00000021852.15	1.66594869	0.00411123	0.02529771	0.06378816	0.80060578	0.91618159	Slc35f4
ENSMUSG00000021866.16	634.4783	0.00068748	0.00219773	0.09830533	0.75387286	0.88616719	Anxa11
ENSMUSG00000021868.8	738.883441	0.01049821	0.00286154	13.3905973	0.00025289	0.00131091	Ppif
ENSMUSG00000021870.18	1678.87329	0.00202159	0.00256838	0.60511085	0.43663462	0.62814664	Slmap
ENSMUSG00000021871.20	544.254365	-0.0051769	0.00566302	0.82120863	0.36482703	0.55463399	Gm49342
ENSMUSG00000021872.9	2.90309841	-0.0008339	0.00767531	0.00886263	0.92499674	0.98907054	Rnase10
ENSMUSG00000021876.16	21871.0733	0.01162583	0.0040248	8.36188251	0.00383173	0.01408153	Rnase4
ENSMUSG00000021877.13	2403.53263	0.00214301	0.00265549	0.64041883	0.42355918	0.61534134	Arf4
ENSMUSG00000021879.14	4.52065606	0.00729617	0.01202933	0.18889096	0.66384127	0.82372475	Dnah12
ENSMUSG00000021880.8	29.798419	0.05989498	0.00707188	66.6402858	3.26E-16	1.06E-14	Rnase6
ENSMUSG00000021884.19	6549.51958	-0.0189156	0.00470925	15.7824423	7.11E-05	0.00042079	Hacl1
ENSMUSG00000021886.8	62.0468891	0.06799194	0.00634535	103.995003	2.03E-24	1.80E-22	Gpr65
ENSMUSG00000021890.7	736.342359	-0.0033057	0.00219665	2.2609262	0.13267469	0.26200194	Eaf1
ENSMUSG00000021891.9	295.441432	-0.0064705	0.00344895	3.5394955	0.05992369	0.13977057	Mettl6
ENSMUSG00000021892.15	1024.2938	0.00544737	0.0025489	4.52596871	0.03338417	0.08682314	Sh3bp5
ENSMUSG00000021893.15	677.380967	-0.0029345	0.00159209	3.36030134	0.06678585	0.15242994	Capn7
ENSMUSG00000021895.11	514.457098	0.00039888	0.00259097	0.02354287	0.87805379	0.96289089	Arhgef3
ENSMUSG00000021898.18	21.9104846	0.02589843	0.02921265	-0.4919331	1	1	Asb14

ENSMUSG00000021900.11	1154.7249	-0.0026218	0.00231753	1.26906863	0.25994148	0.43386657	Btd
ENSMUSG00000021901.11	620.36502	-0.0075831	0.0022933	10.9244038	0.00094906	0.00419185	Bap1
ENSMUSG00000021902.8	148.102667	-0.0080346	0.00322937	6.09662079	0.01354406	0.04113447	Phf7
ENSMUSG00000021903.12	86.0834735	0.04173636	0.00514173	59.7980952	1.05E-14	2.88E-13	Galnt15
ENSMUSG00000021904.7	10.4125999	0.00672641	0.01189832	0.35291278	0.55246889	0.73469761	Sema3g
ENSMUSG00000021905.15	787.542564	-0.0050614	0.00372149	1.84252709	0.17465505	0.32288869	Dph3
ENSMUSG00000021906.15	504.645899	-0.006779	0.00313479	4.68800929	0.03037382	0.08033156	Oxnad1
ENSMUSG00000021910.16	2115.42524	-0.003582	0.00250741	2.02262918	0.15497061	0.29450785	Nisch
ENSMUSG00000021911.17	361.841169	0.00642636	0.00308694	4.2867344	0.03841087	0.09724763	Parg
ENSMUSG00000021913.9	20.9876801	-0.005503	0.01226672	0.15189843	0.69672772	0.84758716	Ogdhl
ENSMUSG00000021916.17	167.465941	0.0006826	0.0033932	0.03987631	0.84172262	0.94198449	Glt8d1
ENSMUSG00000021917.17	1241.77393	-0.0066576	0.00310348	4.6374081	0.03128221	0.08232786	Spcs1
ENSMUSG00000021918.11	262.941667	0.00361015	0.00285839	1.55723024	0.21207123	0.37439461	Nek4
ENSMUSG00000021922.18	34970.9543	0.00765982	0.0058038	1.72486426	0.18906811	0.34305412	Itih4
ENSMUSG00000021928.10	1961.87583	0.00033674	0.00467041	0.00523452	0.9423234	0.99823219	Ebpl
ENSMUSG00000021929.10	813.78135	-0.0021397	0.00230148	0.84671265	0.35748398	0.54633689	Kpna3
ENSMUSG00000021930.15	828.666837	-0.0104415	0.00253714	16.3842607	5.17E-05	0.00031574	Spryd7
ENSMUSG00000021932.14	137.408632	0.01079832	0.00431904	6.20634733	0.0127293	0.03900654	Rnaseh2b
ENSMUSG00000021936.15	406.093354	0.00253066	0.00266166	0.89718034	0.34353889	0.53167378	Mapk8
ENSMUSG00000021938.13	187.889569	0.01169781	0.00463167	6.19098142	0.0128403	0.0393007	Pspc1
ENSMUSG00000021939.9	12188.5505	0.02055432	0.00251242	65.9015142	4.74E-16	1.52E-14	Ctsb
ENSMUSG00000021943.8	137.134986	0.02109323	0.00399222	27.6407708	1.46E-07	1.48E-06	Gdf10
ENSMUSG00000021944.16	331.480937	-0.010197	0.00419701	5.69553924	0.01700809	0.04978934	Gata4
ENSMUSG00000021945.10	532.499851	-0.0006434	0.00267925	0.05734068	0.81074958	0.92218936	Zmym2
ENSMUSG00000021947.12	356.602366	0.0170895	0.00407373	17.2855443	3.22E-05	0.00020497	Cryl1
ENSMUSG00000021948.18	268.369405	0.02392777	0.00382599	37.981496	7.14E-10	1.01E-08	Prkcd
ENSMUSG00000021950.16	7.79825497	0.05096361	0.0107981	21.6094828	3.34E-06	2.63E-05	Anxa8
ENSMUSG00000021951.8	127.589033	-0.0103936	0.0032903	9.95950367	0.00160021	0.00663081	Eef1akmt1
ENSMUSG00000021952.18	292.953393	-0.0002666	0.00404103	0.00425495	0.94799092	1	Xpo4
ENSMUSG00000021957.8	2384.4081	0.00511393	0.00300219	2.8851847	0.08939775	0.19254273	Tkt
ENSMUSG00000021958.6	73.3208872	-0.0081935	0.00449344	3.34725225	0.06731729	0.15340562	Pinx1
ENSMUSG00000021959.17	385.887494	-0.0027488	0.00441629	0.37026493	0.54285989	0.72637961	Lats2
ENSMUSG00000021962.10	168.346013	0.00018252	0.00350051	0.00272316	0.95838217	1	Dcp1a
ENSMUSG00000021963.18	1405.70684	-0.0060744	0.00336448	3.29134633	0.06964589	0.15754246	Sap18
ENSMUSG00000021965.4	5.84733862	0.01962971	0.01254188	2.28691216	0.13046926	0.25850537	Ska3
ENSMUSG00000021967.5	669.267729	-0.00899	0.00390528	5.43850568	0.01969751	0.05619225	Mrpl57
ENSMUSG00000021969.9	417.510391	0.00806032	0.00356666	5.02697814	0.0249554	0.06833834	Zdhhc20
ENSMUSG00000021972.15	289.8231	-0.0039027	0.00249635	2.42362553	0.11951803	0.24141066	Hmbox1
ENSMUSG00000021973.10	807.458864	-0.0004875	0.00243823	0.03938119	0.84269539	0.94229303	Micu2
ENSMUSG00000021974.16	10.7688141	0.04539472	0.01078407	15.1359216	0.00010004	0.0005716	Fgf9
ENSMUSG00000021975.10	163.043101	-0.0044589	0.00225681	3.91638287	0.04781766	0.11630825	Ints9
ENSMUSG00000021977.12	2.26480068	-0.308656	0.09714319	-0.6345751	1	1	1700129C05Rik
ENSMUSG00000021978.10	540.840083	-0.0049228	0.00229574	4.57320405	0.03247577	0.08491946	Extl3
ENSMUSG00000021981.11	1458.05087	-0.0015438	0.00209361	0.54216105	0.4615385	0.65274612	Cab39l
ENSMUSG00000021982.15	988.287591	-0.0164685	0.00267876	37.176759	1.08E-09	1.49E-08	Cdadc1
ENSMUSG00000021983.17	19.8292504	0.05090365	0.00956077	25.8952304	3.60E-07	3.38E-06	Atp8a2
ENSMUSG00000021986.10	15.801698	-0.0009533	0.00870188	0.01201786	0.91270599	0.98234708	Amer2
ENSMUSG00000021987.10	547.06282	0.00671377	0.00254681	6.76244648	0.0093096	0.03008307	Mtmr6
ENSMUSG00000021990.17	606.030014	0.00898867	0.00369498	5.74219185	0.01656211	0.04870141	Spata13
ENSMUSG00000021991.10	7.42002994	0.0244566	0.01705525	2.56607312	0.10917845	0.22497666	Cacna2d3
ENSMUSG00000021993.11	611.139314	-0.0109924	0.00276645	15.5290358	8.12E-05	0.00047433	Mipep
ENSMUSG00000021994.16	37.6753791	0.01813258	0.00585429	9.30570216	0.00228442	0.00906006	Wnt5a
ENSMUSG00000021996.17	4656.17457	-0.0013022	0.00263136	0.24229131	0.62255609	0.79232399	Esd
ENSMUSG00000021998.17	1855.25588	0.01141456	0.00287591	15.2029066	9.66E-05	0.00055329	Lcp1
ENSMUSG00000021999.10	5811.70549	-0.0043548	0.00488535	0.80033286	0.37099387	0.56163424	Cpb2
ENSMUSG00000022000.12	498.532686	-0.0038112	0.00341138	1.21273986	0.27079018	0.4477404	Zc3h13
ENSMUSG00000022003.8	823.064981	-0.0493455	0.00653847	49.1882647	2.33E-12	4.67E-11	Slc25a30
ENSMUSG00000022008.5	386.650486	0.00454865	0.00185372	5.92178057	0.01495484	0.044745	Gpalpp1
ENSMUSG00000022009.2	161.882134	0.00322382	0.00221882	2.09053383	0.14821427	0.28481194	Nufip1
ENSMUSG00000022010.20	1211.19849	-0.0272939	0.00428579	39.8381785	2.76E-10	4.15E-09	Tsc22d1
ENSMUSG00000022012.10	1.53019478	0.03120352	0.01848005	2.89820612	0.08867819	0.19123743	Enox1

ENSMUSG00000022013.5	1468.53646	-0.0056146	0.0046879	1.41639296	0.23399802	0.40214786	Dnajc15
ENSMUSG00000022014.17	82.204628	0.04699287	0.00551205	68.8486797	1.06E-16	3.67E-15	Epsti1
ENSMUSG00000022016.18	793.022175	0.00599389	0.00294193	4.10274814	0.04281357	0.106362	Akap11
ENSMUSG00000022018.8	7.17568532	0.05213174	0.01368633	13.1600517	0.00028598	0.00146316	Rgcc
ENSMUSG00000022019.17	262.167381	-0.0008987	0.00298915	0.08954776	0.76475288	0.89331432	Tdrd3
ENSMUSG00000022020.16	272.706045	-0.0054742	0.00420971	1.663315	0.19715633	0.35416065	Naa16
ENSMUSG00000022021.16	15.1622464	0.01160485	0.01149004	0.89865909	0.34314151	0.5311982	Diaph3
ENSMUSG00000022022.4	185.394553	-0.0103607	0.00251963	17.0333596	3.67E-05	0.00023231	Mtrf1
ENSMUSG00000022023.7	426.323904	-0.0027181	0.00253776	1.14906934	0.28374405	0.46381504	Wbp4
ENSMUSG00000022024.11	620.874571	-0.0028265	0.00288434	0.9609352	0.32695137	0.51375582	Sugt1
ENSMUSG00000022025.14	85.8184358	-0.0294166	0.00691537	16.7710898	4.22E-05	0.00026383	Cnmd
ENSMUSG00000022026.8	5.29076006	0.06507202	0.01667881	12.5253882	0.00040146	0.00197103	Olfm4
ENSMUSG00000022031.7	395.254299	-0.0035594	0.00163492	4.72710521	0.02969082	0.0787991	Elp3
ENSMUSG00000022032.15	266.93583	-0.0256525	0.00944098	6.39857175	0.01142122	0.03565598	Scara5
ENSMUSG00000022033.10	15.8322973	0.0078049	0.01123187	0.44143013	0.506433	0.69325181	Pbk
ENSMUSG00000022034.11	24.644321	-0.0112232	0.00875569	1.42998177	0.23176741	0.39944949	Esco2
ENSMUSG00000022035.7	1464.73633	-0.0114153	0.00379123	9.00950963	0.00268578	0.0104035	Ccdc25
ENSMUSG00000022037.16	24809.5145	-0.0031818	0.00325957	0.9409914	0.33202301	0.51899362	Clu
ENSMUSG00000022039.7	2.92446231	-0.0390009	0.01466262	7.9290606	0.0048647	0.01727476	Adam2
ENSMUSG00000022040.9	11626.32	-0.0216705	0.00293617	52.7001138	3.89E-13	8.64E-12	Ephx2
ENSMUSG00000022041.11	90.5473256	-0.0536399	0.00538972	94.2599394	2.77E-22	1.91E-20	Chrna2
ENSMUSG00000022043.20	150.392445	0.01749488	0.00429775	16.2121022	5.66E-05	0.00034337	Trim35
ENSMUSG00000022044.15	4.09718452	0.0211364	0.02084223	1.61830554	0.20332822	0.3629531	Stmn4
ENSMUSG00000022048.9	201.378877	0.03584549	0.00384339	85.3045249	2.56E-20	1.38E-18	Dpysl2
ENSMUSG00000022051.16	2000.70894	-0.0078761	0.00214945	13.3078714	0.00026429	0.00136433	Bnip3l
ENSMUSG00000022052.10	409.881858	0.00024838	0.00270849	0.00827157	0.92753379	0.99043878	Ppp2r2a
ENSMUSG00000022053.14	3.14895559	0.00433604	0.02070816	0.14747311	0.70096193	0.85063224	Ebf2
ENSMUSG00000022054.12	17.7615214	0.03676421	0.01534966	6.27004645	0.01227959	0.03786957	Nefm
ENSMUSG00000022055.8	23.2813917	0.04352275	0.01793189	6.83788328	0.00892445	0.02899042	Nefl
ENSMUSG00000022057.9	65.4382575	0.07751972	0.00512404	219.771141	1.01E-49	1.60E-46	Adamdec1
ENSMUSG00000022064.11	87.0038326	0.00321478	0.00327205	0.95013015	0.32968617	0.5164969	Pibf1
ENSMUSG00000022066.17	337.687674	-0.0009958	0.00333019	0.09045532	0.76359911	0.89268418	Entpd4b
ENSMUSG00000022070.8	22.9502796	0.01199337	0.00537231	4.83715415	0.02785282	0.07485605	Bora
ENSMUSG00000022074.7	11.3347697	0.03745019	0.00901254	16.7383812	4.29E-05	0.00026778	Tnfrsf10b
ENSMUSG00000022075.8	153.559998	-0.0090279	0.00274411	10.943704	0.00093922	0.00415463	Rhobtb2
ENSMUSG00000022076.11	1.67664055	0.01406027	0.02573304	0.53737125	0.46352421	0.65452597	Klhl1
ENSMUSG00000022089.10	274.559075	0.01275949	0.00499988	6.40267265	0.01139487	0.03559727	Bin3
ENSMUSG00000022090.11	40.5961432	0.03829869	0.00640448	34.4528361	4.37E-09	5.56E-08	Pdlim2
ENSMUSG00000022091.7	552.288558	0.00377342	0.00406963	0.85365688	0.35551988	0.54404022	Sorbs3
ENSMUSG00000022092.12	67.562641	0.0305253	0.00395347	57.8215202	2.87E-14	7.43E-13	Ppp3cc
ENSMUSG00000022094.16	3364.54872	-0.0036866	0.00388384	0.87025967	0.35088343	0.53932464	Slc39a14
ENSMUSG00000022095.10	235.237461	-0.0067679	0.00302005	4.99057113	0.0254858	0.06960491	Fhip2b
ENSMUSG00000022096.15	32.9850808	0.03502002	0.00863904	13.8118733	0.00020206	0.00107723	Hr
ENSMUSG00000022097.5	8.32155384	-0.0485728	0.03656686	2.07654229	0.14957867	0.28694287	Sftpc
ENSMUSG00000022098.11	1861.41484	-0.0134585	0.00313363	18.3934239	1.80E-05	0.00012132	Bmp1
ENSMUSG00000022099.19	28.8006652	0.01974154	0.00749969	6.57655268	0.01033308	0.03277516	Dmtn
ENSMUSG00000022100.15	1103.68741	-0.0020862	0.00250662	0.68708216	0.40715862	0.59874327	Xpo7
ENSMUSG00000022102.14	88.8036223	0.04799862	0.0074248	36.1021462	1.87E-09	2.50E-08	Dok2
ENSMUSG00000022103.11	30.0694682	0.02920638	0.00697192	16.7082671	4.36E-05	0.00027156	Gfra2
ENSMUSG00000022105.7	227.346378	0.01544092	0.00415935	13.1882855	0.0002817	0.00144441	Rb1
ENSMUSG00000022106.15	916.429316	0.00770884	0.00262589	8.43157022	0.00368762	0.01361768	Rcbtb2
ENSMUSG00000022108.9	16222.1572	0.00364547	0.00183739	3.8918676	0.04852044	0.11774906	Itm2b
ENSMUSG00000022109.7	198.494941	-0.0023862	0.00240356	0.98130974	0.32187567	0.5081499	Med4
ENSMUSG00000022110.14	2487.68769	-0.0162179	0.00303943	28.0924756	1.16E-07	1.19E-06	Sucla2
ENSMUSG00000022111.10	288.636762	-0.0047673	0.00286328	2.73193084	0.0983603	0.20754394	Uchl3
ENSMUSG00000022112.15	5.69317057	0.01218691	0.02275904	0.32072673	0.57117123	0.75071857	Gpc5
ENSMUSG00000022114.6	32.8252313	0.01300814	0.00444302	8.4491702	0.0036521	0.01351403	Spry2
ENSMUSG00000022119.16	430.504422	0.00108159	0.0022008	0.23809306	0.62558681	0.79456599	Rbm26
ENSMUSG00000022120.5	60.1834424	0.01822663	0.00502925	12.242917	0.00046703	0.00225101	Obi1
ENSMUSG00000022122.16	230.616578	0.0016762	0.00312982	0.2853442	0.59321964	0.76874458	Ednrb
ENSMUSG00000022123.10	1.6445545	0.05030335	0.01726834	9.57243072	0.00197521	0.0079992	Scel

ENSMUSG00000022124.16	847.41906	0.00762646	0.0024216	9.67942242	0.00186343	0.00759739	Fbxl3
ENSMUSG00000022125.8	739.934265	0.00981425	0.00245607	15.681877	7.49E-05	0.00044132	Cln5
ENSMUSG00000022126.7	24.5155364	0.15816404	0.01085015	180.544332	3.69E-41	2.80E-38	Acod1
ENSMUSG00000022129.5	93.4539137	-0.0477455	0.00797619	31.3313657	2.18E-08	2.50E-07	Dct
ENSMUSG00000022130.11	643.247947	-0.0108969	0.00271723	16.1397572	5.88E-05	0.000355	Tgds
ENSMUSG00000022131.4	427.888664	-0.0060353	0.00203593	8.72812348	0.00313339	0.0118493	Gpr180
ENSMUSG00000022132.16	24.8988668	0.06845005	0.0132172	22.2716446	2.37E-06	1.91E-05	Cldn10
ENSMUSG00000022136.9	4696.49036	-0.0054043	0.00304308	3.03893092	0.08128944	0.17841919	Dnajc3
ENSMUSG00000022139.18	1015.80848	0.01372036	0.00295615	20.5929047	5.68E-06	4.25E-05	Mbnl2
ENSMUSG00000022141.8	952.140604	-8.17E-05	0.00317124	0.00065702	0.97955049	1	Nipbl
ENSMUSG00000022142.9	399.369513	-0.0080359	0.00257128	9.68974393	0.00185299	0.00756006	Nup155
ENSMUSG00000022146.13	109.204295	0.02681175	0.00484081	27.7392818	1.39E-07	1.41E-06	Osmr
ENSMUSG00000022148.17	443.173104	0.04854778	0.00645209	52.6378051	4.01E-13	8.90E-12	Fyb
ENSMUSG00000022149.19	9222.46832	-0.028178	0.00593556	20.8270992	5.03E-06	3.80E-05	C9
ENSMUSG00000022150.17	356.58342	0.0074198	0.00442238	2.77490398	0.0957524	0.20314774	Dab2
ENSMUSG00000022151.17	332.162287	-0.0072186	0.0024578	8.50684975	0.00353812	0.0131604	Ttc33
ENSMUSG00000022155.10	0.80688641	-0.0212768	0.06289607	0.18242223	0.66929982	0.82771622	Mroh2b
ENSMUSG00000022157.9	0.61131969	0.09269383	0.02690612	14.7147466	0.00012506	0.00069933	Mcpt8
ENSMUSG00000022159.17	60.5366371	0.01432286	0.00493582	8.36014247	0.0038354	0.01409283	Rab2b
ENSMUSG00000022160.19	182.643107	-0.0044837	0.00314466	2.02533975	0.15469432	0.29412497	Mettl3
ENSMUSG00000022174.9	1025.20619	-0.0062115	0.0030169	4.33602086	0.03731403	0.09492922	Dad1
ENSMUSG00000022175.10	874.008883	-0.0045136	0.00295097	2.34341024	0.12581386	0.25132935	Lrp10
ENSMUSG00000022176.12	2.97788413	0.01600073	0.01307726	1.48681586	0.22271115	0.38803762	Rem2
ENSMUSG00000022177.10	167.131167	-0.0092558	0.00297569	9.70628776	0.00183639	0.0074949	Haus4
ENSMUSG00000022178.12	121.521942	-0.0102411	0.00416765	6.05560145	0.01386225	0.04200348	Ajuba
ENSMUSG00000022179.6	61.3539403	-0.0032238	0.00389978	0.68005508	0.40956776	0.60127382	4931414P19Rik
ENSMUSG00000022180.8	149.587655	0.05312142	0.0051493	102.901781	3.52E-24	3.01E-22	Slc7a8
ENSMUSG00000022181.17	3490.62439	-0.0371989	0.00523046	46.2042715	1.07E-11	1.97E-10	C6
ENSMUSG00000022184.9	234.86666	0.00348548	0.00249597	1.93935296	0.16373881	0.30696634	Fbxo4
ENSMUSG00000022185.20	1662.1251	0.00298158	0.0036943	0.6379618	0.42444983	0.61606209	Acin1
ENSMUSG00000022186.15	604.615326	0.03538679	0.00613643	30.5548644	3.25E-08	3.62E-07	Oxct1
ENSMUSG00000022191.17	230.98108	-3.06E-05	0.0030008	0.00010434	0.99184994	1	Drosha
ENSMUSG00000022193.8	1603.83167	-0.0137853	0.0032973	17.5745164	2.76E-05	0.00017921	Psmb5
ENSMUSG00000022194.16	1262.09027	-0.007632	0.00420473	3.26717721	0.07067914	0.15943579	Pabpn1
ENSMUSG00000022195.8	329.887712	-0.0017173	0.00196016	0.76164237	0.38281503	0.5744821	6030458C11Rik
ENSMUSG00000022197.17	43.8712836	0.01270484	0.00672522	3.37343136	0.06625562	0.15152938	Pdzd2
ENSMUSG00000022199.13	30.648747	0.01829018	0.00669802	7.1078617	0.00767466	0.02548783	Slc22a17
ENSMUSG00000022200.9	1382.99403	-0.0045213	0.00197317	5.16093616	0.02310044	0.06410719	Golph3
ENSMUSG00000022201.17	1189.30311	-0.0037867	0.00276676	1.80811556	0.17873445	0.32857584	Zfr
ENSMUSG00000022203.8	5.02156195	0.02515529	0.00867553	8.16125522	0.00427947	0.01549519	Efs
ENSMUSG00000022204.10	298.445479	-0.0070986	0.0031078	5.14981343	0.02324887	0.06445843	Ngdn
ENSMUSG00000022205.16	1334.02779	-0.0025272	0.00270602	0.87146688	0.35054953	0.53895191	Sub1
ENSMUSG00000022206.8	55.3625509	-0.0204035	0.00544271	13.4823171	0.00024082	0.00125664	Npr3
ENSMUSG00000022208.13	16.4013093	0.0139088	0.01530643	1.11009034	0.29206231	0.47399585	Jph4
ENSMUSG00000022210.8	2167.24345	-0.0118067	0.00275758	18.0150337	2.19E-05	0.00014529	Dhrs4
ENSMUSG00000022211.10	4.50008231	0.01118578	0.01051911	1.07375797	0.30009815	0.48420543	Carmil3
ENSMUSG00000022212.16	5.02404329	0.02046338	0.02561665	57.0789522	4.19E-14	1.06E-12	Cpne6
ENSMUSG00000022214.16	5363.21121	-0.0091866	0.00233599	15.2666306	9.34E-05	0.00053741	Dcaf11
ENSMUSG00000022215.7	615.272732	0.01755228	0.00595659	7.06403514	0.00786467	0.02601795	Fitm1
ENSMUSG00000022216.18	2066.34907	0.01141753	0.00352811	10.5362877	0.00117053	0.00504167	Psme1
ENSMUSG00000022217.15	297.722857	-0.0142	0.00357385	15.5919608	7.86E-05	0.00046017	Emc9
ENSMUSG00000022218.17	264.652251	0.01353737	0.00525068	6.44161685	0.01114771	0.0349548	Tgm1
ENSMUSG00000022219.11	3444.09616	-0.0042015	0.00392214	1.14027945	0.28559329	0.46609488	Cideb
ENSMUSG00000022220.15	88.5854903	0.00321349	0.00464427	0.46581031	0.49492096	0.68228452	Adcy4
ENSMUSG00000022221.15	35.0678948	0.04078173	0.00688295	32.4239926	1.24E-08	1.48E-07	Ripk3
ENSMUSG00000022223.10	178.923444	0.00277807	0.00360238	0.59309772	0.44122375	0.63308268	Sdr39u1
ENSMUSG00000022225.8	1.49233521	0.04305418	0.01893668	3.42325392	0.06428419	0.14795149	Cma1
ENSMUSG00000022228.15	897.792787	0.00117884	0.00238115	0.24379604	0.62147776	0.79165236	Zscan26
ENSMUSG00000022231.11	40.4444566	0.01389335	0.00672918	4.07872104	0.04342652	0.10758499	Sema5a
ENSMUSG00000022234.16	2382.30634	-0.0066573	0.00236193	7.86842347	0.00503055	0.01778335	Cct5
ENSMUSG00000022235.16	2694.50566	-0.0123333	0.00291492	17.6969724	2.59E-05	0.00016867	Cmbl

ENSMUSG00000022236.11	188.264731	-0.0016564	0.00390055	0.18008575	0.67129956	0.82914073	Ropn1l
ENSMUSG00000022237.18	582.774567	-0.0141161	0.00304275	21.2445045	4.04E-06	3.12E-05	Ankrd33b
ENSMUSG00000022240.11	14.9301757	0.02990044	0.01047816	7.48660078	0.00621598	0.02130341	Ctnnd2
ENSMUSG00000022241.7	2606.06993	-0.0069789	0.0033604	4.18331436	0.04082379	0.10235675	Tars
ENSMUSG00000022244.8	2295.66274	-0.0149554	0.00267829	31.4165246	2.08E-08	2.40E-07	Amacr
ENSMUSG00000022246.15	561.115099	-0.0056742	0.00357822	2.4527937	0.11731591	0.23789983	Rai14
ENSMUSG00000022247.13	474.782944	-0.0020809	0.00311002	0.44193482	0.50619008	0.6930619	Brix1
ENSMUSG00000022248.15	92.1346666	-0.0040489	0.00295431	1.83721015	0.17527829	0.32382746	Rad1
ENSMUSG00000022249.15	1.21279255	-0.0135707	0.10775037	0.02151337	0.88338906	0.96595338	Ttc23l
ENSMUSG00000022253.18	3384.50498	-0.0069022	0.00265626	6.61459876	0.01011461	0.03220346	Nadk2
ENSMUSG00000022255.17	2260.41194	0.00427636	0.00373482	1.27632024	0.25858435	0.43203209	Mtdh
ENSMUSG00000022257.5	387.814558	0.00430631	0.00226956	3.54211269	0.05982921	0.13959162	Laptm4b
ENSMUSG00000022261.7	3253.18832	-0.0015098	0.00210108	0.51809819	0.47165407	0.6624456	Sdc2
ENSMUSG00000022262.9	9.38542514	0.00776494	0.00912859	0.73713674	0.39057963	0.5819219	Dnah5
ENSMUSG00000022263.11	426.216324	0.00059827	0.00362426	0.02650262	0.87067886	0.9590357	Trio
ENSMUSG00000022265.8	524.312605	-0.0089227	0.00334884	7.06323356	0.00786819	0.02602426	Ank
ENSMUSG00000022269.14	1.77243139	-0.019003	0.0305138	0.49175803	0.48314413	0.67241291	Marchf11
ENSMUSG00000022270.17	2050.9169	0.00525154	0.0039203	1.77826389	0.18236266	0.33365958	Retreg1
ENSMUSG00000022272.18	1180.25735	0.00436997	0.00362037	1.43909251	0.23028625	0.39768132	Myo10
ENSMUSG00000022280.10	606.036335	-0.0052029	0.00186551	7.53966892	0.00603551	0.02076942	Rnf19a
ENSMUSG00000022283.16	4825.69221	-0.001397	0.00301889	0.20961651	0.64706815	0.81149302	Pabpc1
ENSMUSG00000022285.18	2608.11999	0.00630996	0.00197913	9.83826326	0.00170919	0.00702935	Ywhaz
ENSMUSG00000022286.17	7.8406859	-0.0040558	0.00831036	0.22913124	0.63216875	0.79999636	Grhl2
ENSMUSG00000022292.17	118.865718	0.01235054	0.00435714	7.67223924	0.00560768	0.01950491	Rrm2b
ENSMUSG00000022295.9	723.020992	-0.0013556	0.00289079	0.21683963	0.64145858	0.80772207	Atp6v1c1
ENSMUSG00000022296.11	7.64119687	0.0494883	0.01659985	10.6365129	0.00110877	0.00480458	Baalc
ENSMUSG00000022297.16	106.136489	-0.0071599	0.00454705	2.41495455	0.12018146	0.24252223	Fzd6
ENSMUSG00000022299.10	618.269914	-0.0042575	0.00316266	1.78577926	0.18144126	0.3324896	Slc25a32
ENSMUSG00000022300.11	443.622755	-0.0025368	0.00287688	0.77820702	0.37769006	0.56885664	Dcaf13
ENSMUSG00000022303.10	1.09973824	0.034341	0.01779193	5.30898106	0.02121576	0.05974865	Dcstamp
ENSMUSG00000022304.14	3510.16643	-0.001389	0.00443696	0.09778984	0.75449821	0.88645993	Dpys
ENSMUSG00000022305.14	88.6001257	0.02033101	0.00404997	24.0821235	9.23E-07	7.98E-06	Lrp12
ENSMUSG00000022306.10	28.3940323	0.01876465	0.00542108	11.3284076	0.0007633	0.0034635	Zfpm2
ENSMUSG00000022307.17	1509.30058	-0.0024333	0.00304063	0.63633236	0.42504203	0.61665998	Oxr1
ENSMUSG00000022309.10	47.307154	0.01713942	0.00585469	8.33073528	0.00389799	0.01429383	Angpt1
ENSMUSG00000022311.16	5.44436954	0.00721877	0.01449115	0.27107269	0.60261311	0.77597893	Csmd3
ENSMUSG00000022312.12	2462.39538	-0.0004771	0.00269739	0.03154579	0.85902821	0.95283438	Eif3h
ENSMUSG00000022313.11	125.506051	-0.0086803	0.00460759	3.48677832	0.06186086	0.1434097	Utp23
ENSMUSG00000022314.11	972.66948	0.00230902	0.00194716	1.38089454	0.23994883	0.40956963	Rad21
ENSMUSG00000022321.16	4.38943697	0.01127297	0.01933837	0.44451683	0.50495039	0.69178438	Cdh10
ENSMUSG00000022322.9	15.0851769	0.00028329	0.00918866	0.00084035	0.97687356	1	Shcbp1
ENSMUSG00000022323.12	10391.2638	-0.0050991	0.00417077	1.49360541	0.2216579	0.38677394	Rida
ENSMUSG00000022324.17	53.444236	0.03478096	0.00564582	37.5400161	8.96E-10	1.25E-08	Matn2
ENSMUSG00000022325.15	98.4580642	-0.0021359	0.00449301	0.22188876	0.63760453	0.80437208	Pop1
ENSMUSG00000022329.16	220.758363	0.00388779	0.00265341	2.10299987	0.14701046	0.28326025	Stk3
ENSMUSG00000022330.6	1.20407339	0.0345195	0.03058394	1.83702413	0.17530014	0.32382746	Osr2
ENSMUSG00000022332.9	335.599441	-0.0078413	0.00331877	5.51431827	0.01886142	0.05422423	Khdrbs3
ENSMUSG00000022335.18	51.0216071	-0.0018624	0.00339347	0.29206219	0.58890205	0.76566032	Zfat
ENSMUSG00000022336.4	2488.04533	-0.0023247	0.00267717	0.74403039	0.38837285	0.57986544	Eif3e
ENSMUSG00000022337.8	606.206418	-0.0089799	0.00310759	8.27327825	0.00402328	0.01469616	Emc2
ENSMUSG00000022338.9	1347.81597	-0.0054011	0.00286189	3.59502212	0.05795286	0.13618373	Eny2
ENSMUSG00000022339.10	435.143419	0.00329823	0.00239422	1.88607602	0.16964517	0.31560585	Ebag9
ENSMUSG00000022340.16	27.6830703	0.0843598	0.00960258	69.1628403	9.07E-17	3.18E-15	Sybu
ENSMUSG00000022342.7	4.18309027	0.02300914	0.02697319	0.94213075	0.33173047	0.51874261	Kcnv1
ENSMUSG00000022346.18	141.45113	0.00719627	0.0083953	0.69974443	0.40286958	0.59454899	Myc
ENSMUSG00000022347.9	3779.09974	0.13807396	0.01674478	36.6372113	1.42E-09	1.93E-08	A1bg
ENSMUSG00000022350.8	522.207848	0.00412837	0.00229982	3.19598494	0.07381929	0.16526832	Washc5
ENSMUSG00000022351.15	1898.5847	0.01838564	0.00562305	9.90612558	0.0016473	0.00680082	Sqle
ENSMUSG00000022353.11	3070.4724	-0.008124	0.00501401	2.56540491	0.10922459	0.22505208	Mtss1
ENSMUSG00000022354.5	5429.32259	-0.0201048	0.00367962	30.3474428	3.61E-08	4.00E-07	Ndufb9
ENSMUSG00000022357.3	2.78369091	-0.0082983	0.01651542	0.22203125	0.63749655	0.80427888	Klhl38

ENSMUSG00000022358.8	132.502978	0.00544724	0.0046759	1.30182901	0.2538794	0.42616181	Fbxo32
ENSMUSG00000022359.11	115.625926	0.00057146	0.00309128	0.03428849	0.85309452	0.94937658	Ntaq1
ENSMUSG00000022360.9	124.154862	0.01827352	0.00534537	11.1721099	0.00083036	0.00372833	Atad2
ENSMUSG00000022361.15	898.039594	-0.0035933	0.00253468	1.975465	0.15986898	0.30138835	Zhx1
ENSMUSG00000022362.14	18.3310952	-0.0114053	0.0097654	1.16430286	0.28057495	0.46000763	Gm29394
ENSMUSG00000022364.15	200.321863	0.02105672	0.00432046	22.8399688	1.76E-06	1.46E-05	Tbc1d31
ENSMUSG00000022365.7	2322.23994	-0.0038983	0.00184342	4.49440166	0.03400601	0.08823606	Derl1
ENSMUSG00000022366.13	9.43771157	0.03397566	0.02220138	3.86516212	0.04929843	0.11918335	Slc22a22
ENSMUSG00000022369.14	50.0354024	0.01049673	0.007123	2.07339075	0.14988796	0.28737257	Mtbp
ENSMUSG00000022370.14	937.386138	-0.0117312	0.00333587	12.7055082	0.00036458	0.00180798	Mrpl13
ENSMUSG00000022371.18	1039.75944	0.01972601	0.00287075	46.087076	1.13E-11	2.07E-10	Col14a1
ENSMUSG00000022372.15	110.959725	0.06414352	0.00551166	123.152755	1.29E-28	2.04E-26	Sla
ENSMUSG00000022375.7	2.37811604	-0.0037992	0.02641282	-0.0041772	1	1	Dnaaf11
ENSMUSG00000022376.9	2.50496893	0.02371787	0.01541909	2.47619918	0.11558126	0.23504911	Adcy8
ENSMUSG00000022377.17	451.711241	0.00680091	0.00490381	1.84534754	0.17432547	0.32251413	Asap1
ENSMUSG00000022378.15	240.89318	0.03972806	0.00384884	100.856381	9.89E-24	7.85E-22	Cyrib
ENSMUSG00000022382.16	6.08318938	0.03260165	0.01162961	7.88952687	0.00497219	0.01759814	Wnt7b
ENSMUSG00000022383.14	1697.21185	-0.0145438	0.00421287	11.7635741	0.00060401	0.00282047	Ppara
ENSMUSG00000022385.12	15.6262121	0.01331408	0.00793022	2.70036128	0.10032551	0.21072175	Gtse1
ENSMUSG00000022386.13	137.159425	-0.0069026	0.00302139	5.27506861	0.02163294	0.06070606	Trmu
ENSMUSG00000022387.18	455.31228	-0.000975	0.00267018	0.13319219	0.71514495	0.85983631	Brd1
ENSMUSG00000022388.15	8.69806531	-0.0772752	0.01225489	35.1742344	3.01E-09	3.94E-08	Ttll8
ENSMUSG00000022389.16	2420.75549	0.01533693	0.00567585	7.18466913	0.00735291	0.02460987	Tef
ENSMUSG00000022390.12	610.213355	-0.0010309	0.00300327	0.11888158	0.73025068	0.870367	Zc3h7b
ENSMUSG00000022391.17	860.730323	-0.0248222	0.00258898	90.6540695	1.71E-21	1.05E-19	Rangap1
ENSMUSG00000022394.15	287.716048	-0.009486	0.00223755	18.033423	2.17E-05	0.00014405	L3mbtl2
ENSMUSG00000022400.10	1174.18133	-0.003453	0.00372781	0.86491019	0.35236825	0.54093709	Rbx1
ENSMUSG00000022401.13	407.30494	-0.0104329	0.00282831	13.3036299	0.00026489	0.00136712	Xpnpep3
ENSMUSG00000022403.16	5263.80035	-0.0008325	0.00337953	0.05875031	0.80848215	0.92088955	St13
ENSMUSG00000022404.9	979.941878	-0.0168408	0.00230754	53.9292554	2.08E-13	4.78E-12	Slc25a17
ENSMUSG00000022407.11	328.125073	-0.0120033	0.00338012	12.4249871	0.00042363	0.0020644	Adsl
ENSMUSG00000022408.8	48.7295884	0.04524048	0.00648193	45.7366268	1.35E-11	2.45E-10	Fam83f
ENSMUSG00000022412.15	591.525682	-0.0148088	0.00231671	40.122956	2.38E-10	3.62E-09	Mief1
ENSMUSG00000022414.9	81.5442987	0.00643112	0.00369346	2.9649051	0.08508945	0.18510605	Tab1
ENSMUSG00000022415.13	25.5382887	0.03905542	0.00956112	14.6576617	0.00012891	0.00071777	Syngr1
ENSMUSG00000022416.16	8.91781264	0.09216042	0.01123401	60.1879834	8.62E-15	2.39E-13	Cacna1i
ENSMUSG00000022419.17	4240.44846	-0.0130875	0.00439472	8.76639909	0.00306831	0.01163979	Deptor
ENSMUSG00000022420.17	104.982821	0.00199125	0.0027148	0.53450298	0.46471984	0.65575125	Dnal4
ENSMUSG00000022421.22	30.2382973	0.02309339	0.00883911	6.76643137	0.00928884	0.0300242	Nptxr
ENSMUSG00000022422.14	3.35724157	0.04258003	0.01149357	12.9486058	0.00032016	0.00161318	Dscc1
ENSMUSG00000022425.17	1229.18649	-0.0023658	0.00345472	0.47636674	0.49007266	0.67778322	Enpp2
ENSMUSG00000022426.7	696.442296	0.00324431	0.00177464	3.33311907	0.067898	0.15448989	Josd1
ENSMUSG00000022427.5	917.429392	-0.0060592	0.00250496	5.9071698	0.01507938	0.04506617	Tomm22
ENSMUSG00000022428.9	108.143788	-0.0027349	0.00324632	0.71742062	0.3969913	0.58882133	Cby1
ENSMUSG00000022432.8	28.6694909	0.06242898	0.01239291	18.5330928	1.67E-05	0.0001135	Smc1b
ENSMUSG00000022433.22	168.552004	0.01969331	0.00468158	17.4920987	2.89E-05	0.00018617	Csnk1e
ENSMUSG00000022434.9	52.2342824	0.01745284	0.00522023	11.0733324	0.00087578	0.00390402	Fam118a
ENSMUSG00000022436.17	44.3355864	0.03947303	0.00638697	36.3087125	1.68E-09	2.26E-08	Sh3bp1
ENSMUSG00000022437.8	1591.72461	-0.005409	0.00268479	4.09552211	0.04299695	0.10675011	Samm50
ENSMUSG00000022438.7	91.0598697	0.02787864	0.00632678	18.5340822	1.67E-05	0.00011347	Parvb
ENSMUSG00000022439.10	48.2611303	0.07735143	0.00732126	97.9536521	4.28E-23	3.16E-21	Parvg
ENSMUSG00000022440.10	27.1452364	-0.0033305	0.00809575	0.17130446	0.67895509	0.83435808	C1qtnf6
ENSMUSG00000022441.18	9.11304544	-0.0049626	0.03375061	-0.0658466	1	1	Efcab6
ENSMUSG00000022442.17	63.0704594	-0.0101408	0.00488572	4.26967534	0.03879834	0.09806019	Ttll1
ENSMUSG00000022443.18	3192.72964	0.01803075	0.00357879	24.4444121	7.65E-07	6.70E-06	Myh9
ENSMUSG00000022445.8	11802.5462	0.00230952	0.0046432	0.24633738	0.61966593	0.79011701	Cyp2d26
ENSMUSG00000022449.15	2.03699038	0.01613988	0.0203151	1.05091408	0.30529666	0.49014546	Adamts20
ENSMUSG00000022450.7	2706.17615	-0.012829	0.00306654	17.6712748	2.63E-05	0.00017079	Ndufa6
ENSMUSG00000022451.13	1220.6677	0.01354788	0.00307586	18.8025106	1.45E-05	9.98E-05	Twf1
ENSMUSG00000022452.17	1034.25836	-0.0095726	0.00321362	8.99151065	0.00271237	0.01048924	Smdt1
ENSMUSG00000022453.9	468.569425	0.00666457	0.00263361	6.44450333	0.01112962	0.03493055	Naga

ENSMUSG00000022454.18	9.12270962	0.01870725	0.01960736	1.09326646	0.2957486	0.47875954	Nell2
ENSMUSG00000022456.19	24.5408242	0.02418936	0.01044738	4.96992935	0.0257917	0.07028574	Septin3
ENSMUSG00000022462.8	2303.4007	-0.0120538	0.00331482	13.4832725	0.0002407	0.00125628	Slc38a2
ENSMUSG00000022463.9	960.906816	0.00938722	0.00270109	11.8078951	0.0005898	0.00276177	Srebf2
ENSMUSG00000022464.15	19248.3408	0.000245	0.00447866	0.00296522	0.95657359	1	Slc38a4
ENSMUSG00000022466.7	156.842441	-0.0013906	0.00332106	0.17388425	0.67668257	0.8328736	Rpap3
ENSMUSG00000022468.13	13.5681804	0.03396253	0.00627934	27.9261438	1.26E-07	1.29E-06	Endou
ENSMUSG00000022469.18	70.3136591	0.00838571	0.00528651	2.53601167	0.11127555	0.22824115	Rapgef3
ENSMUSG00000022471.15	298.254949	-0.0020305	0.0024302	0.69929271	0.40302146	0.5946243	Xrcc6
ENSMUSG00000022472.17	729.728029	-0.0025379	0.00234954	1.15844346	0.28178859	0.46148213	Desi1
ENSMUSG00000022474.16	64.7988184	0.0159705	0.00497876	9.2079394	0.00240968	0.00946771	Pmm1
ENSMUSG00000022475.20	145.05754	0.01858712	0.00402517	21.0737466	4.42E-06	3.38E-05	Hdac7
ENSMUSG00000022476.9	284.163807	-0.0045655	0.00384049	1.41019903	0.23502336	0.40346902	Polr3h
ENSMUSG00000022477.14	3197.6049	-0.0100604	0.00326175	9.54084843	0.00200949	0.00811433	Aco2
ENSMUSG00000022479.16	23.0563033	0.07821775	0.01690477	15.5812094	7.90E-05	0.00046245	Vdr
ENSMUSG00000022483.18	8.34564413	0.03874725	0.02823979	-1.2894042	1	1	Col2a1
ENSMUSG00000022488.10	225.21208	0.05916003	0.0054132	112.341233	3.01E-26	3.35E-24	Nckap1l
ENSMUSG00000022489.7	38.7892416	0.05697999	0.00688067	62.8588574	2.22E-15	6.58E-14	Pde1b
ENSMUSG00000022490.8	6.93463659	0.00804355	0.02000516	0.16185038	0.6874586	0.84108303	Ppp1r1a
ENSMUSG00000022494.16	17.1106661	0.03406138	0.00831753	15.8524013	6.85E-05	0.00040705	Shisa9
ENSMUSG00000022498.17	568.718744	-0.0047715	0.00342766	1.95076671	0.16250425	0.30513696	Txndc11
ENSMUSG00000022500.16	1641.57097	0.01753248	0.00397424	18.1657322	2.02E-05	0.00013537	Litaf
ENSMUSG00000022501.7	5.7167383	-0.285027	0.05491585	3.22866467	0.07235967	0.16260243	Prm1
ENSMUSG00000022503.11	502.766538	-0.0025518	0.0028047	0.83463597	0.36093541	0.55039908	Nubp1
ENSMUSG00000022504.11	68.7559124	0.08200577	0.00654254	136.304967	1.71E-31	4.34E-29	Ciita
ENSMUSG00000022505.10	343.928756	0.03567428	0.00501571	48.3402882	3.58E-12	7.05E-11	Emp2
ENSMUSG00000022507.7	627.265291	-0.0018315	0.00213152	0.73337316	0.391792	0.58312652	Hapstr1
ENSMUSG00000022508.6	607.620548	-0.0272952	0.00763262	12.735393	0.0003588	0.00178462	Bcl6
ENSMUSG00000022510.15	3.25374586	0.01638031	0.01495772	1.12583876	0.28866468	0.46982239	Trp63
ENSMUSG00000022512.4	1374.07961	-0.0094356	0.00539018	3.00058515	0.08323445	0.18174113	Cldn1
ENSMUSG00000022514.15	2680.65967	-0.0127812	0.00384329	10.8743539	0.00097505	0.004297	Il1rap
ENSMUSG00000022515.7	199.189712	-0.0056626	0.00287674	3.88914815	0.04859907	0.11790348	Anks3
ENSMUSG00000022516.11	289.279969	0.00695547	0.00307475	5.21028002	0.02245372	0.06265894	Nudt16l1
ENSMUSG00000022517.18	1034.58724	-0.0044071	0.00274273	2.57353416	0.10866472	0.22405508	Mgrn1
ENSMUSG00000022518.17	4.49615605	-0.0232111	0.02617034	1.27862349	0.25815513	0.43160889	4930562C15Rik
ENSMUSG00000022519.15	144.675941	0.00368827	0.00974545	-1.7236681	1	1	Srl
ENSMUSG00000022521.11	1184.79854	0.00545232	0.00434686	1.53839862	0.21485632	0.37785274	Crebbp
ENSMUSG00000022523.11	34.1465206	0.02147529	0.00715845	8.25049992	0.00407408	0.01485828	Fgf12
ENSMUSG00000022525.14	10.0284111	0.02687127	0.02321376	2.10162821	0.14714238	0.28337553	Plaat1
ENSMUSG00000022526.9	50.2170691	0.00538772	0.00434996	1.51299805	0.21868218	0.38302824	Zfp251
ENSMUSG00000022528.9	257.357672	0.01074055	0.00449478	5.43495472	0.0197376	0.05627113	Hes1
ENSMUSG00000022529.12	269.420715	0.00632281	0.00218406	8.3127029	0.00393688	0.01441626	Zfp263
ENSMUSG00000022533.15	2732.1837	-0.0071352	0.00310044	5.20516467	0.02251988	0.06282128	Atp13a3
ENSMUSG00000022534.15	21.8117727	0.08492422	0.00719081	125.33462	4.30E-29	7.24E-27	Mefv
ENSMUSG00000022536.15	1552.90626	0.00083017	0.00185628	0.2008681	0.65402105	0.81574514	Glyr1
ENSMUSG00000022537.19	50.3475976	0.00321436	0.00380551	0.73359723	0.39171967	0.58309602	Tmem44
ENSMUSG00000022538.21	356.276924	-0.0045833	0.00230626	3.92818805	0.04748308	0.11570781	Lsg1
ENSMUSG00000022540.18	252.563385	-0.0133415	0.00350059	14.9538238	0.00011017	0.00062331	Rogdi
ENSMUSG00000022544.15	192.413049	0.00397541	0.00391797	1.02474608	0.31139586	0.49645686	Eef2kmt
ENSMUSG00000022545.14	146.745566	0.00663738	0.0021854	9.16298788	0.00246959	0.00966287	Ercc4
ENSMUSG00000022546.6	4030.68459	-0.0041299	0.00333028	1.55843492	0.21189453	0.37419469	Gpt
ENSMUSG00000022548.15	11.2029659	0.01825418	0.01546628	1.27944889	0.25800153	0.43148437	Apod
ENSMUSG00000022550.19	499.413991	-0.0347113	0.00441516	61.5042436	4.42E-15	1.26E-13	Adck5
ENSMUSG00000022551.9	3184.93527	-0.0238298	0.00328544	53.0702131	3.22E-13	7.24E-12	Cyc1
ENSMUSG00000022552.17	491.481985	-0.0012203	0.00311011	0.15702945	0.69190628	0.84402905	Sharpin
ENSMUSG00000022553.17	379.079673	-0.0055507	0.00331589	2.77732876	0.09560751	0.20293365	Maf1
ENSMUSG00000022554.8	68.286687	-0.0052269	0.00315113	2.76055285	0.09661484	0.20467796	Hgh1
ENSMUSG00000022555.13	328.263441	-0.0105987	0.00270251	15.4319829	8.55E-05	0.0004965	Dgat1
ENSMUSG00000022556.12	254.11013	-0.0132918	0.00280461	22.4840174	2.12E-06	1.73E-05	Hsf1
ENSMUSG00000022557.12	422.295933	-0.0070502	0.00216638	10.5683506	0.0011504	0.00496767	Bop1
ENSMUSG00000022558.17	563.444603	-0.0003417	0.00463725	0.00548916	0.94093968	0.99743835	Mroh1

ENSMUSG00000022559.9	210.948064	-0.0094866	0.00307457	9.59318443	0.00195301	0.0079188	Fbxl6
ENSMUSG00000022560.10	144.269569	-0.0034598	0.00306541	1.29698576	0.25476454	0.42724798	Slc52a2
ENSMUSG00000022561.14	338.95177	-0.0072419	0.00328043	4.88421504	0.02710336	0.07315198	Gpaa1
ENSMUSG00000022562.16	2011.993	-0.0139241	0.00271745	25.8088837	3.77E-07	3.53E-06	Oplah
ENSMUSG00000022564.8	2443.86622	-0.0002859	0.00239764	0.01405774	0.90561973	0.97866278	Grina
ENSMUSG00000022565.16	1548.00966	-0.0079937	0.00420802	3.50156494	0.06131087	0.14240155	Plec
ENSMUSG00000022568.18	346.996943	-0.0102056	0.00351067	8.44694962	0.00365656	0.0135263	Scrib
ENSMUSG00000022570.8	384.507833	-0.0057282	0.00266175	4.65285971	0.03100186	0.08170855	Gfus
ENSMUSG00000022571.10	405.107014	-0.0169266	0.00253956	44.715611	2.28E-11	4.01E-10	Pycrl
ENSMUSG00000022574.8	1554.49713	-0.0027856	0.00420201	0.43312155	0.51046121	0.69718792	Naprt
ENSMUSG00000022575.6	530.572944	0.01033503	0.00330833	9.68324521	0.00185956	0.00758291	Gsdmd
ENSMUSG00000022577.19	2.18088565	0.01340839	0.02671273	0.3175436	0.5730876	0.75218944	Ly6h
ENSMUSG00000022579.11	485.95105	0.00337123	0.00445472	0.56847111	0.4508671	0.64254691	Gpihbp1
ENSMUSG00000022580.14	1.75363595	0.02670551	0.01762215	2.70658066	0.09993499	0.21009597	Rhpn1
ENSMUSG00000022582.5	1.10785174	0.03375275	0.02386965	2.04491744	0.15271527	0.29133757	Ly6g
ENSMUSG00000022583.13	1.64472817	0.00618408	0.01382291	0.23987289	0.62429794	0.79339895	Ly6f
ENSMUSG00000022584.15	25.5526631	0.07519888	0.00784146	81.6435797	1.63E-19	8.03E-18	Ly6c2
ENSMUSG00000022586.12	2.84920418	0.13502486	0.01438975	77.8840379	1.09E-18	4.81E-17	Ly6i
ENSMUSG00000022587.16	5845.7083	0.05137584	0.00441303	132.474508	1.18E-30	2.70E-28	Ly6e
ENSMUSG00000022594.15	44.6749891	0.02945113	0.00698657	16.7086052	4.36E-05	0.00027156	Lynx1
ENSMUSG00000022598.7	0.79825843	0.00151864	0.03876589	0.02820582	0.86662586	0.95676079	Psca
ENSMUSG00000022601.12	312.434172	-0.0068485	0.0024606	7.57768167	0.00590954	0.02040141	Zbtb11
ENSMUSG00000022602.15	7.70607107	0.02680309	0.01047918	6.30482302	0.01204099	0.03724575	Arc
ENSMUSG00000022604.19	107.329839	-0.0217946	0.00452691	22.6350959	1.96E-06	1.61E-05	Cep97
ENSMUSG00000022607.19	449.329794	-0.0015285	0.00362668	0.1751715	0.67555604	0.83213207	Ptk2
ENSMUSG00000022610.11	66.1168459	0.00467248	0.00432224	1.16573757	0.28027879	0.45974685	Mapk12
ENSMUSG00000022613.9	14.9526216	0.00803537	0.01308888	0.39794511	0.5281524	0.71284707	Miox
ENSMUSG00000022614.9	399.877858	-0.0022943	0.00281428	0.66592379	0.41447638	0.60588859	Lmf2
ENSMUSG00000022615.8	690.876799	-0.0203694	0.00541458	13.9895545	0.00018383	0.00099059	Tymp
ENSMUSG00000022617.16	800.815661	-0.0050434	0.00414746	1.49182476	0.22193355	0.38711173	Chkb
ENSMUSG00000022619.6	10.8932139	0.04776931	0.01514829	9.37610014	0.00219833	0.00877663	Mapk8ip2
ENSMUSG00000022620.15	753.767983	0.01103739	0.00372612	8.44212648	0.00366627	0.01355584	Arsa
ENSMUSG00000022621.17	48.7955169	-0.0047298	0.00425754	1.23367322	0.26669387	0.44240361	Rabl2
ENSMUSG00000022622.6	1.4245049	-0.0039988	0.01667426	0.05523407	0.81419376	0.92400394	Acr
ENSMUSG00000022623.18	153.026748	-0.0023865	0.00452446	0.2798202	0.59681909	0.77161514	Shank3
ENSMUSG00000022629.19	1376.75284	-0.0043434	0.00379546	1.28017405	0.25786668	0.43132857	Kif21a
ENSMUSG00000022634.10	382.922524	-0.0069919	0.00211678	10.8691428	0.0009778	0.00430589	Yaf2
ENSMUSG00000022635.10	784.736255	-0.0071933	0.00344578	4.35428366	0.03691598	0.09418067	Zcrb1
ENSMUSG00000022636.14	1145.90063	0.01333793	0.00447801	8.75178751	0.00309299	0.01172472	Alcam
ENSMUSG00000022637.12	179.66873	0.01683891	0.00330021	25.5692013	4.27E-07	3.94E-06	Cblb
ENSMUSG00000022641.16	407.922326	0.00204824	0.00381494	0.28513959	0.59335216	0.76876789	Bbx
ENSMUSG00000022651.7	13.2879201	0.05644681	0.01280064	12.4703237	0.00041347	0.00202115	Retnlg
ENSMUSG00000022656.16	957.7565	-0.0051457	0.00227862	5.05256754	0.02458948	0.06751526	Nectin3
ENSMUSG00000022657.10	4.89848027	0.08835289	0.00981621	72.144364	2.00E-17	7.66E-16	Cd96
ENSMUSG00000022658.11	9.61761741	-0.0042084	0.00955974	0.20928995	0.6473245	0.81156057	Tagln3
ENSMUSG00000022659.14	5.21849248	0.07337609	0.00904236	59.1540022	1.46E-14	3.92E-13	Gcsam
ENSMUSG00000022661.15	70.2693119	0.0147954	0.0084213	2.86761116	0.09037893	0.1940686	Cd200
ENSMUSG00000022663.4	1498.78367	-0.0005846	0.00221638	0.06951641	0.79204219	0.91118387	Atg3
ENSMUSG00000022664.12	371.560513	0.00750098	0.00309457	5.7525421	0.01646481	0.04845759	Slc35a5
ENSMUSG00000022665.16	436.367995	0.01770052	0.00532174	10.9754071	0.00092329	0.00409183	Ccdc80
ENSMUSG00000022667.19	25.6507337	0.05082282	0.00601219	66.9856025	2.73E-16	8.97E-15	Cd200r1
ENSMUSG00000022668.11	349.644404	-0.0040599	0.00272723	2.18932015	0.13897064	0.27125886	Gtpbp8
ENSMUSG00000022671.14	202.887459	-0.0061602	0.00327171	3.58836943	0.05818531	0.13658028	Mzt2
ENSMUSG00000022672.9	186.128845	-0.0023141	0.00326073	0.50340474	0.47800792	0.66806677	Prkdc
ENSMUSG00000022673.6	110.888615	0.02208252	0.00467604	21.1433225	4.26E-06	3.27E-05	Mcm4
ENSMUSG00000022674.16	904.347457	0.00320087	0.00291162	1.17811546	0.27773998	0.45659547	Ube2v2
ENSMUSG00000022676.8	92.0517362	0.01842403	0.00442798	17.5229697	2.84E-05	0.00018363	Snai2
ENSMUSG00000022677.16	634.175469	-0.0054767	0.00405041	1.81330809	0.1781119	0.32784043	Cep20
ENSMUSG00000022678.17	287.989835	-0.0028038	0.0024115	1.35246734	0.24484724	0.41564036	Nde1
ENSMUSG00000022679.14	588.168168	-0.0100965	0.00444155	5.06812534	0.02436972	0.06701461	Mpv17l
ENSMUSG00000022680.15	1489.3038	-0.0071307	0.00252964	7.8385498	0.00511436	0.01803369	Pdxdc1

ENSMUSG00000022681.16	299.70063	0.00396074	0.00279658	2.00471775	0.15681048	0.297165	Ntan1
ENSMUSG00000022682.9	396.292161	-0.0109623	0.0024472	20.1164778	7.29E-06	5.32E-05	Rrn3
ENSMUSG00000022684.16	1040.07919	-0.0089523	0.00273596	10.7232418	0.00105798	0.00461491	Bfar
ENSMUSG00000022685.10	382.519838	0.01207694	0.00220549	29.5767236	5.37E-08	5.80E-07	Parn
ENSMUSG00000022686.15	10.1074984	0.06716102	0.00784108	63.5537836	1.56E-15	4.69E-14	B3gnt5
ENSMUSG00000022687.13	10.9266648	0.02613064	0.00803986	10.4623686	0.00121831	0.00522836	Boc
ENSMUSG00000022696.18	5.2335987	0.05479653	0.01046981	25.3567675	4.76E-07	4.35E-06	Sidt1
ENSMUSG00000022698.18	1534.62255	-0.0026793	0.0022336	1.40937247	0.23516059	0.40364586	Naa50
ENSMUSG00000022701.19	155.414215	-0.0024871	0.00561114	0.19180474	0.66141877	0.82214615	Ccdc191
ENSMUSG00000022702.17	221.588725	-0.0075809	0.00304145	6.18215424	0.01290451	0.03947163	Hira
ENSMUSG00000022704.17	122.398103	-0.0097633	0.00344658	8.07240778	0.00449444	0.01614957	Qtrt2
ENSMUSG00000022706.17	358.52455	-0.0056868	0.0025634	5.01184113	0.02517452	0.06886641	Mrpl40
ENSMUSG00000022707.18	2541.91818	0.00767654	0.00314196	5.79165514	0.01610242	0.04757506	Gbe1
ENSMUSG00000022708.17	5083.72055	-0.0112942	0.00471344	5.55893298	0.01838662	0.05309338	Zbtb20
ENSMUSG00000022710.18	1396.72459	-0.0044669	0.00382806	1.34385879	0.24635461	0.41754238	Usp7
ENSMUSG00000022711.17	720.539559	-0.0042229	0.00213876	3.92394663	0.047603	0.11589228	Pmm2
ENSMUSG00000022718.13	243.266482	0.00475703	0.00305719	2.39088086	0.12204484	0.2455053	Dgcr8
ENSMUSG00000022721.20	295.438715	-0.0021423	0.0034411	0.38603915	0.53438878	0.71875534	Trmt2a
ENSMUSG00000022722.19	59.4411357	0.0081675	0.00356877	5.17163133	0.02295865	0.06383386	Arl6
ENSMUSG00000022723.17	345.336022	-0.0217539	0.00463726	21.2078554	4.12E-06	3.17E-05	Crybg3
ENSMUSG00000022724.16	270.269805	-0.0009165	0.00191444	0.22645001	0.63416868	0.80186587	Riox2
ENSMUSG00000022742.7	2313.30501	-0.0065423	0.00293393	4.89755534	0.02689474	0.07273057	Cpox
ENSMUSG00000022744.13	417.539571	-0.0009861	0.00214708	0.20820306	0.64817949	0.81215377	Cldnd1
ENSMUSG00000022747.18	121.532256	0.02339212	0.00537865	18.2422352	1.95E-05	0.00013041	St3gal6
ENSMUSG00000022748.9	69.2360913	-0.0140893	0.00459192	9.33622353	0.00224668	0.00893897	Cmss1
ENSMUSG00000022749.9	242.899691	0.00886246	0.00313604	7.81819882	0.00517227	0.0182161	Tbc1d23
ENSMUSG00000022750.19	280.247622	-0.0091541	0.00477329	3.50046921	0.06135144	0.14248082	Klhl22
ENSMUSG00000022751.10	4563.69709	-0.0112399	0.00329821	11.6517899	0.00064141	0.00297859	Nit2
ENSMUSG00000022752.10	2332.7322	-0.0055008	0.00156117	12.3711627	0.00043602	0.00211678	Tomm70a
ENSMUSG00000022754.7	14.1727681	0.01227996	0.00954648	1.53128882	0.21591912	0.37934215	Tmem45a
ENSMUSG00000022755.5	1.53564886	0.02100994	0.02358668	1.23877791	0.26570671	0.44121082	Adgrg7
ENSMUSG00000022756.18	24.7315365	0.00602671	0.00597796	1.02935906	0.31030925	0.49558328	Slc7a4
ENSMUSG00000022757.20	939.703919	0.00163887	0.00218133	0.56384093	0.4527168	0.64448254	Tfg
ENSMUSG00000022758.15	2.82908078	-0.0044955	0.0108413	0.16007597	0.68908658	0.84220107	P2rx6
ENSMUSG00000022760.18	209.130207	-0.0009424	0.00266614	0.12620871	0.72239582	0.86511439	Thap7
ENSMUSG00000022761.13	574.790985	0.0006027	0.0021026	0.08201695	0.77458249	0.89969972	Lztr1
ENSMUSG00000022762.19	82.5695795	0.02482371	0.00604323	15.1001741	0.00010195	0.00058183	Ncam2
ENSMUSG00000022763.19	86.4477891	-0.0066341	0.00530903	1.5029812	0.22021322	0.38498036	Aifm3
ENSMUSG00000022765.11	595.047655	0.00343948	0.00194142	3.09158228	0.07869825	0.17394592	Snap29
ENSMUSG00000022766.15	9123.34885	0.00390446	0.00453283	0.74216874	0.38896704	0.58049712	Serpind1
ENSMUSG00000022768.18	2.68498824	0.00876117	0.01250604	0.43049353	0.51174688	0.69845853	Ccdc116
ENSMUSG00000022769.10	705.493217	-0.0054107	0.00527938	0.9753843	0.32334101	0.50995126	Sdf2l1
ENSMUSG00000022770.18	985.83598	0.00096329	0.00279426	0.11838205	0.73079595	0.87068908	Dlg1
ENSMUSG00000022771.18	725.993371	0.00162781	0.00184913	0.7697917	0.3802815	0.57173122	Ppil2
ENSMUSG00000022772.13	496.248124	-0.0107061	0.0027593	15.205196	9.64E-05	0.00055289	Senp5
ENSMUSG00000022773.20	11.1722591	-0.0102426	0.00887878	1.20443401	0.27243726	0.44979037	Ypel1
ENSMUSG00000022774.14	428.721989	-0.0136421	0.00324807	17.1435019	3.47E-05	0.00021993	Ncbp2
ENSMUSG00000022779.17	405.54089	-0.0035393	0.00255243	1.93983853	0.16368607	0.30691234	Top3b
ENSMUSG00000022781.9	636.954162	0.00558476	0.00209669	6.97602734	0.00826088	0.02713072	Pak2
ENSMUSG00000022783.12	1.63934072	-0.0131158	0.01930997	0.45532847	0.49981535	0.68698749	Spag6l
ENSMUSG00000022787.11	138.485168	-0.0106804	0.00227303	22.0410434	2.67E-06	2.13E-05	Wdr53
ENSMUSG00000022788.17	443.413354	0.00026701	0.00392084	0.00440495	0.94708344	1	Fgd4
ENSMUSG00000022789.16	791.578556	-0.0101937	0.00202878	25.1809035	5.22E-07	4.73E-06	Dnm1l
ENSMUSG00000022790.15	456.420338	0.00715324	0.00439142	2.58704148	0.10774142	0.22252093	Igsf11
ENSMUSG00000022791.20	397.354369	-0.0132805	0.0049459	6.86770347	0.00877674	0.02857649	Tnk2
ENSMUSG00000022792.17	289.929849	-0.0151159	0.00266257	31.9336906	1.60E-08	1.87E-07	Yars2
ENSMUSG00000022793.18	36.6372266	0.01037652	0.00442099	5.34075962	0.02083239	0.05891708	B4galt4
ENSMUSG00000022797.17	563.245602	0.00983854	0.005064	3.65418078	0.05592865	0.13236336	Tfrc
ENSMUSG00000022799.5	180.675253	0.01220073	0.0035499	11.4719986	0.00070653	0.00323638	Arhgap31
ENSMUSG00000022800.15	827.229557	-0.0074192	0.00326327	5.18122319	0.02283224	0.06353402	Fyttd1
ENSMUSG00000022801.14	207.175836	0.00364206	0.0030883	1.35789916	0.24390191	0.41443737	Lrch3

ENSMUSG00000022802.3	43.6425293	-0.011901	0.00519421	5.06427544	0.02442391	0.06714014	Lmln
ENSMUSG00000022803.13	46.3157222	0.01171637	0.01269892	1.23219714	0.26698017	0.4427332	Popdc2
ENSMUSG00000022805.18	1.8495519	-0.0153836	0.01510801	0.95523406	0.32839058	0.51539927	Cfap91
ENSMUSG00000022807.10	362.140195	-0.0023855	0.00244482	0.93838926	0.33269243	0.51969244	Osbpl11
ENSMUSG00000022808.6	599.371254	0.00335807	0.00180203	3.4546457	0.06307425	0.14567854	Snx4
ENSMUSG00000022809.5	1016.91409	-0.0072289	0.0038042	3.63795248	0.05647635	0.13348544	Nr1i2
ENSMUSG00000022811.18	842.669744	0.0001166	0.00284663	0.00168295	0.96727697	1	Zfp148
ENSMUSG00000022812.15	1036.70079	-0.0005426	0.00364196	0.02170151	0.88288393	0.96553533	Gsk3b
ENSMUSG00000022814.7	557.168035	0.00957253	0.00228651	17.5465281	2.80E-05	0.00018167	Umps
ENSMUSG00000022816.12	231.343068	0.01815082	0.00543939	11.0443414	0.00088958	0.00395732	Fstl1
ENSMUSG00000022817.16	1244.59187	0.02018411	0.00287171	48.3136229	3.63E-12	7.14E-11	Itgb5
ENSMUSG00000022820.5	1377.72661	-0.0151898	0.00472422	10.4272146	0.00124172	0.00531914	Ndufb4
ENSMUSG00000022821.14	8846.21095	-0.0088393	0.00449194	3.88366868	0.0487579	0.11815514	Hgd
ENSMUSG00000022822.18	186.150281	0.02313672	0.00566752	16.2783474	5.47E-05	0.00033268	Abcc5
ENSMUSG00000022824.13	4.07977237	0.05244036	0.01578859	10.7570585	0.00103882	0.00454661	Muc13
ENSMUSG00000022827.15	229.745135	0.00152471	0.00187277	0.66229827	0.41574974	0.60702447	Rabl3
ENSMUSG00000022828.11	295.138434	-0.0079148	0.00226978	12.2030763	0.00047711	0.00229607	Gtf2e1
ENSMUSG00000022829.15	8.9940485	0.00794907	0.01018297	0.52272051	0.46968349	0.66080859	Stxbp5l
ENSMUSG00000022831.15	189.408574	0.05385755	0.00453837	131.071107	2.39E-30	5.32E-28	Hcls1
ENSMUSG00000022833.8	25.1506707	0.01775431	0.00445702	15.6846336	7.48E-05	0.00044101	Ccdc14
ENSMUSG00000022836.12	1489.04435	-0.0026517	0.00377454	0.49655829	0.4810163	0.67063605	Mylk
ENSMUSG00000022837.15	129.124171	-0.0043328	0.00423451	1.0286438	0.31047741	0.49566267	Iqcb1
ENSMUSG00000022838.15	12.3640431	0.0256374	0.00717712	12.0455518	0.00051916	0.00247623	Eaf2
ENSMUSG00000022840.10	46.0631263	0.00338582	0.00529852	0.40790808	0.5230332	0.70877684	Adcy5
ENSMUSG00000022841.16	3004.39573	-0.0023036	0.0020031	1.33829401	0.24733505	0.41865906	Ap2m1
ENSMUSG00000022842.19	2.04235243	0.00876923	0.02658303	0.06865928	0.79329896	0.91207587	Ece2
ENSMUSG00000022843.18	666.28739	-0.0045319	0.00352081	1.60611691	0.20503851	0.36522486	Clcn2
ENSMUSG00000022844.9	978.091993	-0.0090501	0.00367454	5.97181455	0.01453632	0.0437704	Pdia5
ENSMUSG00000022847.16	240.500673	-0.0053958	0.00445855	1.43344919	0.23120235	0.39887258	Thpo
ENSMUSG00000022848.9	700.010663	-0.0180004	0.00304506	34.0742892	5.30E-09	6.68E-08	Slc49a4
ENSMUSG00000022849.6	58.6064026	0.00373578	0.00342872	1.18752903	0.27582855	0.45433984	Hspbap1
ENSMUSG00000022853.8	5997.64662	-0.0031962	0.004449	0.51123109	0.47460641	0.66524436	Ehhadh
ENSMUSG00000022855.10	531.413537	-0.0022147	0.00203494	1.17606608	0.27815832	0.4570602	Senp2
ENSMUSG00000022856.10	153.308736	0.00217905	0.00314988	0.48183669	0.48759155	0.6759336	Tmem41a
ENSMUSG00000022858.17	1464.39306	-0.0026346	0.00391779	0.44321888	0.50557292	0.69247636	Tra2b
ENSMUSG00000022861.18	5.0140348	0.055665	0.01109882	23.5665147	1.21E-06	1.03E-05	Dgkg
ENSMUSG00000022863.16	132.792922	-0.0080832	0.00519122	2.28781538	0.13039334	0.25846196	Btg3
ENSMUSG00000022864.15	223.955051	0.00386802	0.00329151	1.34886695	0.2454763	0.41647273	D16Ertd472e
ENSMUSG00000022865.15	2003.35202	0.00314252	0.00329881	0.88415424	0.34706639	0.53537657	Cxadr
ENSMUSG00000022867.12	688.694133	0.00406875	0.0026167	2.38350308	0.1226223	0.24639373	Usp25
ENSMUSG00000022868.7	112552.672	-0.0101602	0.00509203	4.0317592	0.04465136	0.10996626	Ahsg
ENSMUSG00000022871.14	7704.51877	0.00393974	0.00515603	0.57958392	0.44647546	0.63840983	Fetub
ENSMUSG00000022875.19	42284.3744	0.01312138	0.00534028	6.03755754	0.01400465	0.04236616	Kng1
ENSMUSG00000022876.19	24.5817759	0.072217	0.00740422	82.6928311	9.58E-20	4.85E-18	Samsn1
ENSMUSG00000022877.10	14914.823	0.00684221	0.00522443	1.7252872	0.18901389	0.3430085	Hrg
ENSMUSG00000022878.6	3.74533794	-0.0096105	0.02235741	0.18994515	0.66296228	0.82289889	Adipoq
ENSMUSG00000022881.16	58.7250222	-0.0015257	0.00435025	0.12164778	0.72725416	0.86825163	Rfc4
ENSMUSG00000022883.12	216.144967	0.04831938	0.00552375	71.9064214	2.26E-17	8.57E-16	Robo1
ENSMUSG00000022884.17	6214.96298	-0.0081374	0.00207407	15.2465249	9.44E-05	0.00054237	Eif4a2
ENSMUSG00000022885.17	1883.17925	0.01883155	0.00361823	26.360414	2.83E-07	2.70E-06	St6gal1
ENSMUSG00000022887.10	3056.54638	0.00174837	0.00337689	0.27220079	0.60185927	0.77533901	Masp1
ENSMUSG00000022889.8	602.591464	-0.0141109	0.00256996	29.7497222	4.92E-08	5.35E-07	Mrpl39
ENSMUSG00000022890.14	3626.1655	-0.0141064	0.00308779	21.0402079	4.50E-06	3.43E-05	Atp5pf
ENSMUSG00000022892.12	1494.23764	0.02460644	0.0032608	56.0349165	7.12E-14	1.73E-12	App
ENSMUSG00000022893.15	233.707197	0.01003459	0.00356326	7.56050695	0.00596612	0.02055762	Adamts1
ENSMUSG00000022894.7	95.6371384	0.02375356	0.00459821	26.1940849	3.09E-07	2.93E-06	Adamts5
ENSMUSG00000022895.17	546.510372	0.00462881	0.00339189	1.8137006	0.17806494	0.32780517	Ets2
ENSMUSG00000022897.16	438.906003	-0.0003353	0.00259167	0.01622578	0.8986393	0.97504154	Dyrk1a
ENSMUSG00000022898.14	422.734915	0.00410478	0.00304496	1.78044613	0.18209455	0.33332419	Vps26c
ENSMUSG00000022899.11	78.5047213	0.0082936	0.00954168	0.72665311	0.3939702	0.58530427	Slc15a2
ENSMUSG00000022900.15	8.05607508	0.03183087	0.00813912	14.6857099	0.00012701	0.00070884	Ildr1

ENSMUSG00000022901.14	114.094505	0.04488044	0.0058428	55.9146633	7.57E-14	1.83E-12	Cd86
ENSMUSG00000022905.13	1256.8291	-0.011894	0.0036028	10.6181957	0.0011198	0.00484885	Kpna1
ENSMUSG00000022906.16	1059.40014	0.00625806	0.00288332	4.71534473	0.02989457	0.07922591	Parp9
ENSMUSG00000022911.12	156.580897	0.01617288	0.00450236	12.7108037	0.00036355	0.00180405	Arl13b
ENSMUSG00000022912.9	1328.86298	0.01349992	0.00259842	26.7625397	2.30E-07	2.24E-06	Pros1
ENSMUSG00000022913.17	316.631235	-0.0074872	0.00212748	12.6232381	0.00038098	0.00187948	Psmg1
ENSMUSG00000022914.18	786.031944	0.00115184	0.00306596	0.14204405	0.70625735	0.85422129	Brwd1
ENSMUSG00000022935.17	1.3823391	0.01583501	0.02696407	0.65423538	0.41860248	0.61001903	Grik1
ENSMUSG00000022938.9	1.50299663	0.04529731	0.0168256	6.83793562	0.00892419	0.02899042	Fam3b
ENSMUSG00000022940.18	355.388271	0.00128274	0.00432955	0.08682929	0.76824744	0.895738	Pigp
ENSMUSG00000022941.9	35.6630495	0.00471346	0.00401754	1.38753724	0.23882144	0.40820657	Ripply3
ENSMUSG00000022945.13	32.3758535	0.03417455	0.00636887	27.9474615	1.25E-07	1.27E-06	Chaf1b
ENSMUSG00000022946.11	636.840362	0.02260304	0.0038978	32.3509082	1.29E-08	1.53E-07	Dop1b
ENSMUSG00000022947.9	34.7240827	0.05263957	0.00934108	27.3915392	1.66E-07	1.66E-06	Cbr3
ENSMUSG00000022948.17	142.839387	0.01885468	0.00654046	8.30308175	0.00395779	0.01448159	Setd4
ENSMUSG00000022949.10	4.81906073	-0.0022202	0.00964073	0.03764746	0.84615275	0.9445929	Clic6
ENSMUSG00000022951.19	455.03879	-0.0023627	0.00415523	0.31471975	0.5747983	0.75372173	Rcan1
ENSMUSG00000022952.18	34.4842964	0.05317954	0.00760172	44.0730069	3.16E-11	5.44E-10	Runx1
ENSMUSG00000022956.12	3199.56797	-0.0191038	0.00353885	29.7050804	5.03E-08	5.46E-07	Atp5po
ENSMUSG00000022957.21	1074.0275	0.00897722	0.00375416	5.56580177	0.01831462	0.05292436	Itsn1
ENSMUSG00000022960.14	61.8564072	0.02485577	0.00498518	24.2592659	8.42E-07	7.33E-06	Donson
ENSMUSG00000022961.19	3526.63592	-0.0015579	0.00217665	0.50828151	0.47588372	0.66616378	Son
ENSMUSG00000022962.16	588.93874	-0.0055781	0.0031566	3.12276321	0.07720576	0.17127414	Gart
ENSMUSG00000022964.15	184.537418	0.00768558	0.00332405	5.30098901	0.02131332	0.05998758	Tmem50b
ENSMUSG00000022965.9	434.293199	-0.0002344	0.00450414	0.00262445	0.95914274	1	Ifngr2
ENSMUSG00000022967.14	665.354151	0.00417983	0.00164285	6.40119478	0.01140436	0.03561744	Ifnar1
ENSMUSG00000022969.14	234.481678	0.03199408	0.00509202	35.9646888	2.01E-09	2.67E-08	Il10rb
ENSMUSG00000022971.19	1146.13291	0.00020558	0.0022041	0.00869931	0.92568901	0.98927508	Ifnar2
ENSMUSG00000022972.11	263.922009	-0.0054603	0.00281531	3.84257465	0.04996674	0.12052584	Cfap298
ENSMUSG00000022973.19	257.944781	0.00603224	0.00352694	2.90117294	0.08851512	0.19100802	Synj1
ENSMUSG00000022974.13	381.339648	-0.0056527	0.00355985	2.50975854	0.11314327	0.23118748	Paxbp1
ENSMUSG00000022978.12	52.5366434	0.01010479	0.00409013	5.93927199	0.01480713	0.04441575	Mis18a
ENSMUSG00000022982.11	14320.6647	-0.0064704	0.00317194	4.16584004	0.04124695	0.10318723	Sod1
ENSMUSG00000022983.17	397.566034	-0.0012321	0.00312499	0.15440228	0.69436339	0.84580153	Scaf4
ENSMUSG00000022987.13	12.4176247	0.01343938	0.00699563	3.63488434	0.05658054	0.1336781	Zfp641
ENSMUSG00000022992.18	540.424942	-0.0042175	0.00178238	5.60472512	0.0179121	0.05197133	Kansl2
ENSMUSG00000022994.10	557.933912	-0.003968	0.00425895	0.86246479	0.35304986	0.5417014	Adcy6
ENSMUSG00000022995.18	122.142266	0.00508689	0.00939239	0.26576023	0.60619013	0.7789061	Enah
ENSMUSG00000022996.16	0.86796503	0.05456928	0.02311644	8.30729066	0.00394862	0.01445256	Wnt10b
ENSMUSG00000022999.16	173.240967	-0.0115073	0.00372369	9.51210124	0.00204121	0.00822553	Lmbr1l
ENSMUSG00000023000.6	4.87552485	0.00718092	0.00693386	1.02610908	0.31107429	0.49624607	Dhh
ENSMUSG00000023004.9	1487.0468	0.00868025	0.00436762	3.90673531	0.04809294	0.1168643	Tuba1b
ENSMUSG00000023007.16	213.800619	-0.0022548	0.00422417	0.28746496	0.59184964	0.76762188	Prpf40b
ENSMUSG00000023008.19	85.4225494	0.0170534	0.00482286	12.0979129	0.00050478	0.00241595	Fmnl3
ENSMUSG00000023009.15	25.3518659	0.02742623	0.00782204	11.3361422	0.00076013	0.00345176	Nckap5l
ENSMUSG00000023010.16	24582.1744	0.00773155	0.00277871	7.61770479	0.0057798	0.02002091	Tmbim6
ENSMUSG00000023011.9	40.441722	0.02045544	0.01706684	1.49789303	0.22099584	0.38600397	Faim2
ENSMUSG00000023013.6	4.11575284	0.00213364	0.06005546	0.01159813	0.91423793	0.98312004	Aqp2
ENSMUSG00000023015.15	37.3253507	0.02632623	0.00973425	6.88427333	0.00869574	0.0283676	Racgap1
ENSMUSG00000023017.11	9.95740508	0.01448274	0.01255408	1.52882895	0.21628828	0.37982097	Asic1
ENSMUSG00000023018.6	128.677455	-0.0009176	0.0039637	0.05222808	0.81923063	0.92744426	Smarcd1
ENSMUSG00000023019.13	5934.56071	-0.0119896	0.0034361	11.8370557	0.00058064	0.00272643	Gpd1
ENSMUSG00000023020.4	773.312967	-0.0137086	0.00376175	12.984743	0.00031404	0.00158709	Cox14
ENSMUSG00000023021.16	63.9141377	0.0286825	0.00478985	34.5118957	4.24E-09	5.42E-08	Cers5
ENSMUSG00000023022.15	1297.51222	0.00986376	0.00282437	11.7943494	0.00059411	0.00277863	Lima1
ENSMUSG00000023025.17	1333.42049	-0.0034669	0.0028852	1.40693532	0.23556581	0.40416493	Larp4
ENSMUSG00000023026.17	175.970143	0.0120403	0.00362842	10.8093253	0.0010099	0.00443319	Dip2b
ENSMUSG00000023027.14	524.836199	-0.0050441	0.00228228	4.77980361	0.0287954	0.076848	Atf1
ENSMUSG00000023030.17	584.889901	-0.0046328	0.00304374	2.25369772	0.13329555	0.26286863	Slc11a2
ENSMUSG00000023031.9	417.100937	-0.0255748	0.0051383	24.0246848	9.51E-07	8.20E-06	Cela1
ENSMUSG00000023032.13	27.6438579	0.0435715	0.00908059	19.569426	9.70E-06	6.89E-05	Slc4a8

ENSMUSG00000023033.15	270.540077	0.05713446	0.00983669	29.6726247	5.12E-08	5.54E-07	Scn8a
ENSMUSG00000023034.8	71.9969298	0.02906978	0.00874097	9.58639794	0.00196024	0.00794266	Nr4a1
ENSMUSG00000023036.15	92.2039968	-0.0024856	0.01143316	0.04411732	0.83363548	0.93638092	Pcdhgc4
ENSMUSG00000023039.18	29.2850828	0.02336492	0.00778251	9.19924551	0.00242115	0.00950961	Krt7
ENSMUSG00000023043.8	2875.44801	0.01739721	0.00471968	13.3869465	0.00025338	0.00131317	Krt18
ENSMUSG00000023044.3	4574.28036	-0.0274071	0.00597938	18.4475004	1.75E-05	0.0001182	Csad
ENSMUSG00000023045.14	611.245862	0.00458973	0.00459671	1.00200948	0.31682476	0.5022603	Soat2
ENSMUSG00000023046.7	29.1782647	-0.0131961	0.00751613	2.96161327	0.08526283	0.18541071	Igfbp6
ENSMUSG00000023047.12	6.22520659	-0.0187718	0.0090448	3.92067018	0.04769586	0.11609439	Amhr2
ENSMUSG00000023048.14	691.590807	0.00340553	0.00284846	1.42056821	0.2333099	0.40144899	Prr13
ENSMUSG00000023050.10	99.1876041	0.0117462	0.0053737	4.71912546	0.02982891	0.07912108	Map3k12
ENSMUSG00000023051.13	212.887046	-0.0163205	0.00278964	34.5965153	4.06E-09	5.20E-08	Tarbp2
ENSMUSG00000023052.9	13.9967701	-0.0047121	0.00703786	0.45559903	0.49968799	0.68698749	Npff
ENSMUSG00000023055.11	597.222266	-0.0060709	0.00332748	3.2968797	0.06941162	0.15714556	Calcoco1
ENSMUSG00000023057.6	2166.41993	-0.0162627	0.00574141	7.46939194	0.00627567	0.02147679	Fabp2
ENSMUSG00000023064.5	10.0018287	0.02979289	0.00926208	10.2706903	0.0013516	0.00571819	Sncg
ENSMUSG00000023066.11	33.9146941	0.01953299	0.00560814	11.6885434	0.00062886	0.00292551	Rttn
ENSMUSG00000023067.15	281.952379	0.01069128	0.00870751	1.34147804	0.24677348	0.41816218	Cdkn1a
ENSMUSG00000023068.17	931.684846	0.00224431	0.00213588	1.08734623	0.29705995	0.48045367	Nus1
ENSMUSG00000023070.7	18150.5363	-0.0040914	0.00526012	0.58470529	0.44447388	0.63674448	Rgn
ENSMUSG00000023072.15	106.746096	-0.0025579	0.00365682	0.48541271	0.48598076	0.67481115	Cep89
ENSMUSG00000023073.3	778.723598	0.02505853	0.00659131	12.0605646	0.000515	0.00245736	Slc10a2
ENSMUSG00000023074.12	217.436027	7.15E-06	0.00280286	4.78E-06	0.99825531	1	Mospd1
ENSMUSG00000023075.10	651.829285	-0.0052103	0.00233185	4.94898985	0.02610592	0.07096164	Akirin1
ENSMUSG00000023078.7	402.693298	0.12435679	0.01067916	119.373067	8.68E-28	1.24E-25	Cxcl13
ENSMUSG00000023079.15	321.986911	-0.0050459	0.00359884	1.92517882	0.16528687	0.30932763	Gtf2ird1
ENSMUSG00000023084.12	7.84819902	-0.0299496	0.01029587	8.4102183	0.00373118	0.01375873	Lrrc71
ENSMUSG00000023087.17	1543.59675	0.0839728	0.00796113	90.770719	1.61E-21	1.00E-19	Noct
ENSMUSG00000023088.18	87.9149628	0.03168487	0.00732412	17.1428505	3.47E-05	0.00021994	Abcc1
ENSMUSG00000023089.13	2273.83009	-0.0125691	0.00443227	8.1877162	0.0042175	0.01529806	Ndufa5
ENSMUSG00000023092.17	170.804745	0.01547509	0.00385326	15.7828728	7.10E-05	0.00042079	Fhl1
ENSMUSG00000023094.15	216.996227	-0.0140471	0.00344592	16.6896988	4.40E-05	0.0002737	Msrb2
ENSMUSG00000023104.10	345.606254	0.00748369	0.00252716	8.77888304	0.00304738	0.01157601	Rfc2
ENSMUSG00000023106.16	1105.88082	-0.0023728	0.00280344	0.7064815	0.40061435	0.59235608	Denr
ENSMUSG00000023110.13	275.100093	-0.0045219	0.00265277	2.87122175	0.09017639	0.19381268	Prmt5
ENSMUSG00000023118.16	931.157977	-0.0069503	0.00301696	5.32208052	0.02105686	0.05939333	Sympk
ENSMUSG00000023122.7	94.3380406	0.02819925	0.00500745	32.7897581	1.03E-08	1.24E-07	Sult1c2
ENSMUSG00000023132.9	22.5609337	0.06655111	0.01032578	36.5872788	1.46E-09	1.97E-08	Gzma
ENSMUSG00000023143.11	250.13043	-0.001157	0.00231873	0.24899502	0.6177836	0.78881789	Nagpa
ENSMUSG00000023147.18	105.000241	0.00313194	0.00366518	0.74387988	0.38842084	0.57986544	Get1
ENSMUSG00000023150.15	2776.56004	-0.0036353	0.00335685	1.14935609	0.28368398	0.46374899	Ivns1abp
ENSMUSG00000023156.5	349.19234	-0.0067861	0.0032316	4.40595638	0.03581364	0.09193518	Rpp14
ENSMUSG00000023169.16	100.21985	0.04926862	0.00705955	42.7281984	6.29E-11	1.04E-09	Slc38a1
ENSMUSG00000023170.15	416.14469	0.00284968	0.00329398	0.74661109	0.38755131	0.57919054	Gps2
ENSMUSG00000023175.16	5724.56662	-0.0046127	0.00248142	3.48229767	0.06202856	0.14365421	Bsg
ENSMUSG00000023176.10	5327.25017	-0.0030833	0.00473917	0.4163593	0.5187593	0.70480742	Cpn2
ENSMUSG00000023186.15	609.829957	0.01468754	0.00256238	32.0219965	1.52E-08	1.80E-07	Vwa5a
ENSMUSG00000023191.10	60.1675769	0.01451432	0.00403227	12.7170203	0.00036234	0.00179958	P3h3
ENSMUSG00000023206.17	562.066958	-0.0166431	0.00364809	20.8090358	5.07E-06	3.83E-05	Il15ra
ENSMUSG00000023216.14	2.57881286	0.08310531	0.02000432	11.0071854	0.00090759	0.00402832	Epb42
ENSMUSG00000023224.13	12691.2643	0.0052147	0.00339389	2.34032552	0.12606321	0.25172086	Serping1
ENSMUSG00000023232.18	182.972083	-0.0042964	0.00453159	0.86906437	0.35121446	0.53965761	Serinc2
ENSMUSG00000023235.15	125.046606	-0.013626	0.00316747	18.2284292	1.96E-05	0.00013129	Ccl25
ENSMUSG00000023236.8	20.3798701	0.02216014	0.01577621	2.49542414	0.11417761	0.23287731	Scg5
ENSMUSG00000023243.10	599.797997	-0.0035739	0.00513875	0.49762882	0.48054385	0.67033992	Kcnk5
ENSMUSG00000023247.3	0.85478366	-0.0032978	0.02291782	-0.0131266	1	1	Guca2a
ENSMUSG00000023249.16	593.887092	0.02080059	0.003195	40.0230911	2.51E-10	3.80E-09	Parp3
ENSMUSG00000023259.18	51.3778998	-0.020477	0.00389531	28.0408281	1.19E-07	1.22E-06	Slc26a6
ENSMUSG00000023262.14	495.984645	-0.0103048	0.00317798	10.7137362	0.00106343	0.00463453	Acy1
ENSMUSG00000023266.12	36.5135683	0.00285787	0.00432075	0.43470705	0.50968826	0.69625318	Frs3
ENSMUSG00000023272.5	772.204484	-0.0106494	0.00601076	2.81520374	0.09337504	0.19928311	Creld2

ENSMUSG00000023274.15	41.3822161	0.00343459	0.00730702	0.20529707	0.65047875	0.81351585	Cd4
ENSMUSG00000023277.14	85.9430693	0.02858571	0.0059217	21.3962362	3.74E-06	2.90E-05	Twf2
ENSMUSG00000023284.16	53.8190873	0.00073127	0.00387318	0.03401604	0.8536727	0.94965195	Zfp605
ENSMUSG00000023286.17	499.96918	-0.0033253	0.00220012	2.2936385	0.12990508	0.25766892	Ube2j2
ENSMUSG00000023307.15	1494.43006	0.00092119	0.00184142	0.24781693	0.61861645	0.78945394	Marchf5
ENSMUSG00000023328.15	13.7369145	0.01447738	0.00748426	3.37872991	0.06604293	0.15116205	Ache
ENSMUSG00000023330.13	127.097827	-0.0022696	0.00276347	0.68203297	0.40888756	0.60057429	Dtwd1
ENSMUSG00000023336.7	78.4966481	0.00946435	0.00340127	7.63493494	0.00572485	0.0198714	Wfdc1
ENSMUSG00000023341.17	107.535013	0.01983421	0.00527818	14.1592	0.00016797	0.00091349	Mx2
ENSMUSG00000023345.18	38.5552832	-0.0032696	0.00450933	0.52148841	0.47020746	0.66117729	Poc1a
ENSMUSG00000023348.12	232.437341	0.00444735	0.00269648	2.73891752	0.09793109	0.20678812	Trip6
ENSMUSG00000023349.15	169.709664	0.10006851	0.00697751	170.394569	6.07E-39	3.67E-36	Clec4n
ENSMUSG00000023353.15	419.158368	-0.0013309	0.00224799	0.35056582	0.55379301	0.73620572	Agap3
ENSMUSG00000023367.15	3211.06605	0.02961078	0.00377589	60.3567283	7.91E-15	2.21E-13	Tmem176a
ENSMUSG00000023391.9	1.60434068	0.02638088	0.05656101	0.21800995	0.64056044	0.8068065	Dlx2
ENSMUSG00000023393.16	85.6703813	-0.0023421	0.00477328	0.23826291	0.62546356	0.79449502	Slc17a9
ENSMUSG00000023411.13	13.1730855	0.01930806	0.00761948	6.23712922	0.01250991	0.03843412	Nfatc4
ENSMUSG00000023439.12	2.36255981	0.18424314	0.03054465	-18.428348	1	1	Gnb3
ENSMUSG00000023452.20	543.594891	-0.0048414	0.0017125	7.96319292	0.00477381	0.0169914	Pisd
ENSMUSG00000023456.17	5854.36331	-0.0024739	0.00235141	1.10919982	0.29225596	0.47417961	Tpi1
ENSMUSG00000023460.15	459.07253	-0.0008855	0.00282894	0.09634358	0.7562624	0.88746728	Rab12
ENSMUSG00000023467.19	11.0837167	0.03932255	0.00862406	18.2157202	1.97E-05	0.00013209	Tulp2
ENSMUSG00000023473.14	8.06875421	0.06789197	0.01496661	17.8556725	2.38E-05	0.00015657	Celsr3
ENSMUSG00000023484.15	5.6019646	0.02902883	0.00880313	10.0457241	0.00152702	0.00637344	Prph
ENSMUSG00000023495.14	212.570117	-0.0086748	0.00482246	3.2454039	0.07162404	0.16127203	Pcbp4
ENSMUSG00000023505.14	43.9979756	0.00682834	0.00803421	0.68725531	0.40709952	0.59869369	Cdca3
ENSMUSG00000023571.5	20.7595423	0.00506418	0.00664016	0.56706205	0.45142875	0.64323074	C1qtnf12
ENSMUSG00000023572.17	308.98018	-0.0024815	0.0020006	1.54079144	0.21450004	0.37756894	Ccndbp1
ENSMUSG00000023707.14	390.243591	-0.0104206	0.00392875	7.06521619	0.00785948	0.02600642	Ogfod2
ENSMUSG00000023723.18	537.883942	-0.0156565	0.00376524	16.8230518	4.10E-05	0.00025752	Mrps23
ENSMUSG00000023755.11	10.0440189	0.02049698	0.00721405	8.04425255	0.00456482	0.0163477	Rhebl1
ENSMUSG00000023764.19	258.63514	0.00939914	0.00320787	8.65949859	0.00325361	0.01223917	Sfi1
ENSMUSG00000023781.3	1.77941756	0.00932657	0.0120689	0.59054754	0.44220747	0.63415245	Hes7
ENSMUSG00000023791.19	321.831144	-0.0138538	0.0031932	19.156279	1.20E-05	8.39E-05	Pigx
ENSMUSG00000023800.16	271.334893	0.02199315	0.00465168	20.6490003	5.52E-06	4.14E-05	Tiam2
ENSMUSG00000023805.18	107.29855	0.00743803	0.00485863	2.24707179	0.13386751	0.26361267	Synj2
ENSMUSG00000023806.11	411.293052	-0.0029408	0.0020507	2.04837747	0.1523685	0.29084283	Rsph3b
ENSMUSG00000023809.11	46.1796686	0.00503416	0.00576236	0.729837	0.39293603	0.58427621	Rps6ka2
ENSMUSG00000023826.18	46.9740305	0.00862674	0.00452585	3.57814304	0.05854456	0.13717783	Prkn
ENSMUSG00000023827.9	25.6425052	0.01798124	0.0053633	10.9599494	0.00093102	0.00412146	Agpat4
ENSMUSG00000023828.4	132.261625	-0.0111431	0.0054264	4.21104997	0.04016147	0.10095369	Slc22a3
ENSMUSG00000023829.10	6077.47818	-0.0122787	0.00490373	6.1989619	0.01278253	0.03914972	Slc22a1
ENSMUSG00000023830.15	849.249151	-0.0012136	0.00300889	0.16212443	0.68720809	0.84090739	Igf2r
ENSMUSG00000023832.15	2133.80822	0.00919013	0.00414732	4.74415366	0.02939801	0.07816272	Acat2
ENSMUSG00000023845.8	883.253516	0.01368782	0.00322947	17.4726295	2.91E-05	0.00018788	Lnpep
ENSMUSG00000023852.15	690.977122	0.00099186	0.00368793	0.0705992	0.79046634	0.91057306	Chd1
ENSMUSG00000023861.19	4329.50639	0.00177768	0.00293788	0.36676319	0.54477392	0.7280733	Mpc1
ENSMUSG00000023868.18	38.2636958	0.00782555	0.00917014	0.66017235	0.4164991	0.60770478	Pde10a
ENSMUSG00000023873.14	16.3580448	0.00023791	0.00703402	0.00107161	0.97388551	1	1700010I14Rik
ENSMUSG00000023882.16	88.6251313	0.01906878	0.00312654	36.5737985	1.47E-09	1.98E-08	Zfp54
ENSMUSG00000023883.14	401.776624	-0.0032979	0.00280701	1.38778987	0.23877869	0.40817076	Phf10
ENSMUSG00000023885.10	81.9661226	0.03175618	0.00509387	36.2748003	1.71E-09	2.30E-08	Thbs2
ENSMUSG00000023886.11	190.64568	0.033438	0.00535494	37.5188592	9.05E-10	1.26E-08	Smoc2
ENSMUSG00000023892.9	114.149808	0.00569913	0.0027978	4.04779028	0.0442292	0.10906325	Zfp51
ENSMUSG00000023903.9	4.3166855	0.06518571	0.01126255	31.3883939	2.11E-08	2.43E-07	Mmp25
ENSMUSG00000023904.11	477.779322	-0.0033254	0.00355006	0.88330824	0.34729718	0.53552527	Hcfc1r1
ENSMUSG00000023905.16	153.33452	-0.0014221	0.00594499	0.05311079	0.81773607	0.92624132	Tnfrsf12a
ENSMUSG00000023906.4	5.06639702	-0.0069943	0.0079509	0.76712926	0.38110661	0.57257025	Cldn6
ENSMUSG00000023908.9	30.8100896	0.01370794	0.0072914	3.61327546	0.05732012	0.134885	Pkmyt1
ENSMUSG00000023909.5	43.9009698	0.00574646	0.00451154	1.66160568	0.19738667	0.35449341	Paqr4
ENSMUSG00000023911.13	12.3155821	-0.0131978	0.00562307	5.74649663	0.01652157	0.04860038	Flywch2

ENSMUSG00000023912.11	25.5356251	0.00122092	0.00499939	0.06155532	0.80405436	0.91805563	Slc25a27
ENSMUSG00000023913.18	134.023766	0.07396008	0.00545777	175.983263	3.65E-40	2.61E-37	Pla2g7
ENSMUSG00000023914.17	93.1424665	0.02783152	0.02052296	2.37232235	0.1235032	0.24778455	Mep1a
ENSMUSG00000023915.6	183.673046	0.05214715	0.00407514	149.410811	2.33E-34	8.21E-32	Tnfrsf21
ENSMUSG00000023919.10	63.2674914	0.005116	0.00416915	1.48041674	0.22370933	0.38911565	Cenpq
ENSMUSG00000023921.10	3108.57302	-0.0068897	0.00261675	6.89468746	0.00864523	0.02823015	Mmut
ENSMUSG00000023923.11	305.232926	0.0085861	0.00259762	10.7361498	0.00105063	0.00458636	Tbc1d5
ENSMUSG00000023926.9	1.08672386	0.06952938	0.03209359	3.91176843	0.04794912	0.1165905	Rhag
ENSMUSG00000023927.16	34.2181808	0.03938069	0.0060106	41.1582487	1.40E-10	2.21E-09	Satb1
ENSMUSG00000023930.15	8.88665379	0.07016261	0.01673864	14.1354197	0.00017011	0.0009249	Crisp2
ENSMUSG00000023932.10	478.224819	-0.0004069	0.00244358	0.02734757	0.86865198	0.95805591	Cdc5l
ENSMUSG00000023935.11	1.96441373	-6.37E-05	0.01638077	-0.0015541	1	1	Spats1
ENSMUSG00000023938.9	289.015639	-0.0127907	0.00259228	24.1657455	8.84E-07	7.66E-06	Aars2
ENSMUSG00000023939.8	1122.69668	-0.0075862	0.00428392	3.14001122	0.07639328	0.16995019	Mrpl14
ENSMUSG00000023940.15	12.0649185	0.02530917	0.0117181	3.78061003	0.05184975	0.12418037	Sgo1
ENSMUSG00000023942.16	3153.64135	-0.0032972	0.0026365	1.58738125	0.20770068	0.36880435	Slc29a1
ENSMUSG00000023944.15	25281.43	-0.0077905	0.00382887	3.89534252	0.04842016	0.11755409	Hsp90ab1
ENSMUSG00000023947.9	70.3753357	0.04182148	0.00381224	114.679494	9.25E-27	1.10E-24	Nfkbie
ENSMUSG00000023949.8	1.21943034	-0.0150049	0.04692228	0.15094432	0.69763456	0.84829658	Tcte1
ENSMUSG00000023951.19	2171.12972	-0.0114006	0.00329861	11.8477034	0.00057733	0.00271305	Vegfa
ENSMUSG00000023952.14	475.880288	-0.0036286	0.0031635	1.32049715	0.25050285	0.42220667	Gtpbp2
ENSMUSG00000023953.10	95.2115942	0.00080305	0.00266364	0.08922569	0.76516384	0.89352695	Polh
ENSMUSG00000023959.11	100.601434	0.00774678	0.00608307	1.50779862	0.21947531	0.38408409	Clic5
ENSMUSG00000023960.14	522.036924	-0.0031853	0.00262759	1.48204375	0.22345503	0.38894957	Enpp5
ENSMUSG00000023961.17	288.364475	0.00570475	0.00267907	4.47330342	0.03442837	0.08913584	Enpp4
ENSMUSG00000023963.11	490.030709	-0.0102121	0.00416174	6.36467083	0.01164147	0.03617069	Cyp39a1
ENSMUSG00000023965.14	422.294156	-0.0030773	0.00227961	1.79456629	0.1803708	0.33104238	Fbxl17
ENSMUSG00000023966.9	6.29388837	-0.0257184	0.0074765	12.1841662	0.00048197	0.00231616	Rsph9
ENSMUSG00000023967.10	557.416347	-0.0125332	0.00339573	13.6008497	0.00022608	0.00118838	Mrps18a
ENSMUSG00000023968.16	4.75267	-0.0298944	0.00837032	12.6525788	0.00037505	0.00185254	Crip3
ENSMUSG00000023971.10	262.003295	-0.0021689	0.00235016	0.84464793	0.35807084	0.54693066	Rrp36
ENSMUSG00000023972.11	17.8763061	0.01254732	0.00610692	4.4982053	0.03393045	0.08805937	Ptk7
ENSMUSG00000023973.14	569.05527	0.00254397	0.0020906	1.47390126	0.22473117	0.39048986	Cnpy3
ENSMUSG00000023977.16	1371.43448	-0.0065804	0.00291065	5.03517435	0.02483759	0.06808335	Ubr2
ENSMUSG00000023979.15	5.12355751	-0.0069235	0.0071575	0.94613418	0.33070526	0.51758556	Guca1b
ENSMUSG00000023980.8	182.942004	0.00085103	0.00220723	0.14812781	0.70033095	0.85047914	Taf8
ENSMUSG00000023982.9	25.3787512	0.01619221	0.00847749	3.62657335	0.05686378	0.13405189	Guca1a
ENSMUSG00000023984.18	7.04360397	0.00139939	0.01066957	0.01655001	0.89763711	0.97444051	Gm20517
ENSMUSG00000023988.9	279.487317	-0.0123848	0.0025854	22.9992084	1.62E-06	1.35E-05	Bysl
ENSMUSG00000023990.19	141.43075	-0.0031285	0.00365024	0.73167208	0.39234174	0.58359307	Tfeb
ENSMUSG00000023991.17	329.643961	-0.0020652	0.00351862	0.34337527	0.5578874	0.73968926	Foxp4
ENSMUSG00000023992.15	56.7141758	0.11800752	0.00983303	115.655855	5.65E-27	6.87E-25	Trem2
ENSMUSG00000023993.8	3.04821293	0.04243594	0.01459062	6.89376817	0.00864967	0.02824076	Treml1
ENSMUSG00000023994.14	176.455984	0.01299963	0.00345499	13.6284364	0.00022279	0.00117343	Nfya
ENSMUSG00000023999.15	1.58452595	0.00454532	0.02200768	0.19454758	0.65915835	0.82046066	Kif6
ENSMUSG00000024002.19	1258.28892	-0.0011252	0.00446291	0.06264286	0.80236652	0.91720751	Brd4
ENSMUSG00000024006.18	1099.29671	-0.0025442	0.00258738	0.9713251	0.32434993	0.51102988	Stk38
ENSMUSG00000024007.16	327.49584	0.00541079	0.00337931	2.55264134	0.11011003	0.2264413	Ppil1
ENSMUSG00000024008.17	6.05401887	0.03717365	0.01639402	4.83428551	0.0278992	0.07496158	Cpne5
ENSMUSG00000024011.18	14.0755162	0.03321323	0.01096204	8.40999088	0.00373165	0.01375873	Pi16
ENSMUSG00000024012.19	1112.37015	0.00301632	0.00248942	1.45999542	0.22693085	0.39323933	Mtch1
ENSMUSG00000024013.16	91.0447542	0.04859912	0.00559844	68.7761659	1.10E-16	3.80E-15	Fgd2
ENSMUSG00000024014.9	428.976839	-0.0086899	0.00435328	3.71928863	0.05378728	0.12807944	Pim1
ENSMUSG00000024018.19	271.289356	-0.0177555	0.00493944	12.9568801	0.00031875	0.00160744	Ccdc167
ENSMUSG00000024019.19	815.182649	-0.0046779	0.0042621	1.18654573	0.27602743	0.45454046	Cmtr1
ENSMUSG00000024026.14	8057.98674	-0.0142646	0.00294674	22.9761179	1.64E-06	1.37E-05	Glo1
ENSMUSG00000024027.9	4.62089438	0.0393088	0.00959077	15.9646686	6.45E-05	0.00038604	Glp1r
ENSMUSG00000024028.8	2.40295226	-0.0372017	0.01348817	9.17041492	0.00245959	0.00963492	Tff2
ENSMUSG00000024029.5	181.032403	-0.0173496	0.01249488	1.41894099	0.23357779	0.40171837	Tff3
ENSMUSG00000024030.8	207.589191	0.03949845	0.00448433	75.3615079	3.92E-18	1.60E-16	Abcg1
ENSMUSG00000024033.11	7.42577643	-0.0143645	0.01181239	1.46162741	0.22667136	0.39293479	Rsph1

ENSMUSG00000024036.17	81.4633237	0.02039718	0.00707953	7.20628731	0.00726486	0.02438438	Slc37a1
ENSMUSG00000024037.14	225.482503	-0.0112998	0.003285	12.0410532	0.00052042	0.00248121	Wdr4
ENSMUSG00000024038.18	1597.9417	-0.0181608	0.00314032	33.3180863	7.83E-09	9.60E-08	Ndufv3
ENSMUSG00000024039.16	8678.5508	0.00045158	0.00505618	0.00777774	0.92972448	0.99165566	Cbs
ENSMUSG00000024042.8	522.801246	0.01466305	0.00575501	6.12160143	0.01335398	0.04063041	Sik1
ENSMUSG00000024043.14	16.8756175	0.01492806	0.00603114	5.98128105	0.0144585	0.04358606	Arhgap28
ENSMUSG00000024044.20	93.405559	0.05250681	0.00618927	66.8960255	2.86E-16	9.38E-15	Epb41l3
ENSMUSG00000024045.7	823.554356	0.00176241	0.00267186	0.43567398	0.50921787	0.69585241	Akap8
ENSMUSG00000024048.16	982.622861	0.01203602	0.00405018	8.58916683	0.00338169	0.01266885	Myl12a
ENSMUSG00000024049.16	244.417346	0.00882287	0.00874623	-4.7572318	1	1	Myom1
ENSMUSG00000024050.19	273.846279	0.00142729	0.00326331	0.18787908	0.66468773	0.82450954	Wiz
ENSMUSG00000024052.18	4702.30894	-0.001936	0.00481696	0.16031982	0.68886224	0.84205778	Lpin2
ENSMUSG00000024053.12	78.9160817	0.05169535	0.00720061	46.1705499	1.08E-11	1.99E-10	Emilin2
ENSMUSG00000024054.15	309.619709	0.00675256	0.00346156	3.74353711	0.05301211	0.12646341	Smchd1
ENSMUSG00000024055.16	1004.15883	-0.0167518	0.00422413	15.4782627	8.35E-05	0.00048604	Cyp4f13
ENSMUSG00000024056.11	11.4860785	0.03267972	0.00984925	9.91281211	0.00164132	0.00677853	Ndc80
ENSMUSG00000024059.11	66.8323458	0.01688599	0.00758993	4.72284156	0.02976452	0.07896805	Clip4
ENSMUSG00000024063.14	355.521201	0.03911993	0.00541286	48.2646686	3.72E-12	7.30E-11	Lbh
ENSMUSG00000024064.15	5.50261257	-0.0103642	0.01130967	0.82096726	0.36489751	0.55464798	Galnt14
ENSMUSG00000024065.9	1189.84074	-0.0046264	0.00367667	1.57928363	0.20886391	0.3703958	Ehd3
ENSMUSG00000024066.10	2854.37039	0.00754571	0.00304603	6.1401858	0.01321434	0.04025749	Xdh
ENSMUSG00000024067.15	494.57025	-0.0046253	0.00329635	1.98106555	0.1592782	0.30053885	Dpy30
ENSMUSG00000024068.9	600.167079	-0.0046472	0.00193124	5.69173268	0.01704502	0.04986651	Spast
ENSMUSG00000024069.15	211.379734	-0.0071559	0.00201546	12.7032459	0.00036502	0.00180946	Slc30a6
ENSMUSG00000024070.16	3636.20459	-0.0121757	0.00397582	9.3425925	0.00223889	0.00891247	Prkd3
ENSMUSG00000024072.10	764.254716	-0.0078425	0.00369381	4.53510954	0.03320633	0.0864178	Yipf4
ENSMUSG00000024073.17	1803.83295	0.00531822	0.00332667	2.51617669	0.11268349	0.2304611	Birc6
ENSMUSG00000024074.9	347.284056	0.0064712	0.00335918	3.54078523	0.05987711	0.13968956	Crim1
ENSMUSG00000024076.11	4.42008688	0.00954825	0.01731178	0.29089679	0.58964642	0.76612133	Vit
ENSMUSG00000024077.16	382.444536	-0.0020525	0.0028937	0.48991037	0.48396729	0.67316462	Strn
ENSMUSG00000024078.8	183.285962	-0.00624	0.00372615	2.77596991	0.09568868	0.20305111	Ttc27
ENSMUSG00000024079.5	375.632719	0.01263053	0.00311683	16.3807388	5.18E-05	0.00031616	Eif2ak2
ENSMUSG00000024081.10	1462.29466	-0.0074297	0.00418813	3.07121547	0.07968987	0.17559466	Cebpz
ENSMUSG00000024082.6	258.499869	-0.0054449	0.0027345	4.01309399	0.04514823	0.11100449	Ndufaf7
ENSMUSG00000024083.17	886.280489	0.00476648	0.00254052	3.4974381	0.06146384	0.1426716	Pja2
ENSMUSG00000024084.9	215.878451	-0.0143882	0.00337444	18.7687953	1.48E-05	0.00010135	Qpct
ENSMUSG00000024085.14	4289.76578	0.01620323	0.00338806	22.2663875	2.37E-06	1.91E-05	Man2a1
ENSMUSG00000024087.5	40.9159905	0.06632615	0.00780186	64.7522381	8.49E-16	2.64E-14	Cyp1b1
ENSMUSG00000024091.10	1290.73572	-0.0035793	0.00172986	4.23970841	0.03948895	0.09953899	Vapa
ENSMUSG00000024095.17	546.356437	0.00121545	0.00186925	0.42199617	0.51594267	0.70251841	Hnrnpll
ENSMUSG00000024096.18	806.922034	-0.0004263	0.00329973	0.01667971	0.89723901	0.97418769	Ralbp1
ENSMUSG00000024097.12	771.167934	-0.0017262	0.00263556	0.4312589	0.51137187	0.69810829	Srsf7
ENSMUSG00000024098.7	971.84608	-0.0047799	0.00224388	4.4606622	0.03468405	0.08965019	Twsg1
ENSMUSG00000024099.16	2667.07194	-0.0167258	0.00384721	19.1249478	1.22E-05	8.52E-05	Ndufv2
ENSMUSG00000024101.16	274.002259	0.00468472	0.00211748	4.92595184	0.02645622	0.07173198	Washc1
ENSMUSG00000024104.12	1017.59074	0.01350636	0.0022484	35.4855321	2.57E-09	3.37E-08	Washc2
ENSMUSG00000024109.19	300.107867	0.01903807	0.00559515	11.1663322	0.00083295	0.00373924	Nrxn1
ENSMUSG00000024112.18	15.4683344	0.10800863	0.0132534	58.2451	2.31E-14	6.06E-13	Cacna1h
ENSMUSG00000024118.15	1326.00842	-0.0322777	0.00469465	45.7172463	1.37E-11	2.47E-10	Tedc2
ENSMUSG00000024120.13	2024.59381	-0.0137766	0.00220003	39.5912673	3.13E-10	4.65E-09	Lrpprc
ENSMUSG00000024121.14	2595.44232	0.00509803	0.00258772	3.90581062	0.04811941	0.11689611	Atp6v0c
ENSMUSG00000024122.17	728.556231	-0.0010558	0.00317819	0.10853055	0.74182333	0.87777279	Pdpk1
ENSMUSG00000024124.11	4.1712482	0.03760572	0.01011782	12.454302	0.00041703	0.00203603	Prss30
ENSMUSG00000024127.16	469.48188	-0.0064777	0.00268813	5.81976794	0.01584704	0.0469499	Prepl
ENSMUSG00000024130.17	1400.44862	0.00032514	0.00349317	0.00865038	0.92589766	0.98945323	Abca3
ENSMUSG00000024131.9	1302.27677	0.03081331	0.00564524	27.1632463	1.87E-07	1.85E-06	Slc3a1
ENSMUSG00000024132.7	1870.6331	-0.0245469	0.00339713	51.8443043	6.01E-13	1.30E-11	Eci1
ENSMUSG00000024135.11	263.192016	-0.0034389	0.00232432	2.18769323	0.13911753	0.2714449	Srbd1
ENSMUSG00000024136.15	14.8067605	-0.00858	0.00612196	1.91171654	0.16677271	0.31146576	Dnase1l2
ENSMUSG00000024137.10	144.153537	-0.0026813	0.00407074	0.4279758	0.51298387	0.69970145	E4f1
ENSMUSG00000024140.11	3390.10161	-0.0032083	0.00342061	0.87864787	0.34857228	0.53650652	Epas1

ENSMUSG00000024142.16	151.10268	-0.0017102	0.00303584	0.31430931	0.57504778	0.75386306	Mlst8
ENSMUSG00000024143.16	266.453049	0.01987524	0.00508196	14.6511863	0.00012935	0.0007199	Rhoq
ENSMUSG00000024145.7	86.2134986	-0.0061762	0.00352718	3.10049722	0.07826839	0.17320128	Pigf
ENSMUSG00000024146.10	777.719978	-0.0074735	0.00257664	8.38732958	0.00377846	0.01390741	Cript
ENSMUSG00000024150.13	4869.7504	0.00195481	0.00232695	0.69203146	0.40547427	0.59708535	Mcfd2
ENSMUSG00000024151.14	167.845421	0.00456044	0.00280974	2.66529311	0.10255893	0.21416157	Msh2
ENSMUSG00000024155.9	123.586745	0.01991363	0.00451034	19.1527963	1.21E-05	8.40E-05	Meiob
ENSMUSG00000024158.18	4338.69443	-0.0167361	0.00287318	33.9910751	5.54E-09	6.95E-08	Hagh
ENSMUSG00000024160.18	261.82262	-0.0056225	0.00319859	3.11510315	0.07756957	0.17195073	Spsb3
ENSMUSG00000024163.19	427.058858	-0.0022037	0.00407979	0.29170997	0.58912682	0.76574976	Mapk8ip3
ENSMUSG00000024164.16	129826.76	0.0140357	0.00456684	9.33672105	0.00224607	0.00893805	C3
ENSMUSG00000024165.10	546.224552	0.00683994	0.0032727	4.27182411	0.03874931	0.09796777	Jpt2
ENSMUSG00000024168.9	221.596299	-0.0011269	0.0029145	0.14819465	0.70026662	0.8504448	Tmem204
ENSMUSG00000024169.17	151.523831	0.01055895	0.00364702	8.33180669	0.00389569	0.01428763	Ift140
ENSMUSG00000024170.16	102.446364	0.00344654	0.00437577	0.62534833	0.42906673	0.62047862	Telo2
ENSMUSG00000024172.11	2.86510645	0.02032289	0.01906829	1.86136847	0.17246694	0.31979601	St6gal2
ENSMUSG00000024174.10	91.9680786	0.02711555	0.00462952	32.3436911	1.29E-08	1.54E-07	Pot1b
ENSMUSG00000024176.12	6.63856772	0.03803136	0.01598255	6.80831762	0.00907342	0.02942064	Sox8
ENSMUSG00000024177.10	29.0433625	-0.0045455	0.00554503	0.6779572	0.41029104	0.60188609	Nme4
ENSMUSG00000024180.15	391.53291	0.00406079	0.00227853	3.13624558	0.07656988	0.17024671	Pgap6
ENSMUSG00000024181.10	916.055523	-0.0164429	0.00383318	18.7025602	1.53E-05	0.00010472	Mrpl28
ENSMUSG00000024182.19	398.26548	0.00164382	0.00245837	0.45029007	0.50219724	0.68889287	Axin1
ENSMUSG00000024186.16	5.50170438	0.02081596	0.01004663	4.08906103	0.04316161	0.10708003	Rgs11
ENSMUSG00000024187.16	871.430529	-0.0066711	0.00287407	5.43505258	0.0197365	0.05627113	Fam234a
ENSMUSG00000024188.17	713.105074	-0.007318	0.00325529	5.08196701	0.02417593	0.06657483	Luc7l
ENSMUSG00000024190.8	632.133354	-0.0152049	0.00495654	9.30342397	0.00228726	0.00906715	Dusp1
ENSMUSG00000024191.16	316.540768	-0.0032053	0.00294123	1.21514549	0.27031546	0.4471299	Bnip1
ENSMUSG00000024193.9	302.495505	0.00027336	0.0037737	0.00526977	0.94212984	0.9981168	Phf1
ENSMUSG00000024194.17	550.318182	-0.002861	0.00309561	0.86789187	0.35153959	0.54003661	Cuta
ENSMUSG00000024197.11	911.342488	0.00655015	0.0027162	5.81912034	0.01585287	0.0469554	Plin3
ENSMUSG00000024201.13	260.787585	-0.0101838	0.00391868	6.70797947	0.00959824	0.03083274	Kdm4b
ENSMUSG00000024206.16	26.0602617	0.01845841	0.00502459	13.233631	0.00027497	0.00141357	Rfx2
ENSMUSG00000024208.17	564.569327	-0.0115692	0.00443741	6.82727356	0.00897762	0.02914606	Uqcc2
ENSMUSG00000024209.10	1.77323943	0.04412799	0.02078165	4.71408316	0.02991651	0.07926431	Acsbg3
ENSMUSG00000024210.4	2.25321139	0.02898389	0.02457862	1.9241845	0.1653961	0.30940929	Ip6k3
ENSMUSG00000024211.16	40.3403793	0.02653699	0.0080085	8.52340737	0.00350607	0.01305965	Grm8
ENSMUSG00000024212.10	304.29089	-0.0061269	0.00306363	3.9849678	0.04590798	0.11260249	Mllt1
ENSMUSG00000024213.15	699.092252	-0.0033357	0.00344977	0.93122081	0.3345459	0.5214512	Nudt3
ENSMUSG00000024215.16	1.95120298	0.03396253	0.01261691	6.67650037	0.00976922	0.03126841	Spdef
ENSMUSG00000024217.11	393.790365	-0.0037003	0.00339788	1.19708426	0.27390522	0.45164489	Snrpc
ENSMUSG00000024218.14	235.569469	-0.0062624	0.00329641	3.5978121	0.05785567	0.13599599	Taf11
ENSMUSG00000024219.17	333.730499	-0.0087825	0.00333733	6.89024365	0.00866675	0.02828866	Anks1
ENSMUSG00000024220.15	372.9476	-0.0021465	0.00347598	0.37970642	0.5377604	0.7218556	Zfp523
ENSMUSG00000024222.19	897.371914	0.00135097	0.00574411	0.05743806	0.810592	0.92218936	Fkbp5
ENSMUSG00000024225.6	3.06018433	-0.3125586	0.07281129	4.92750246	0.02643249	0.07167588	Clps
ENSMUSG00000024228.13	1270.85048	-0.0017293	0.0032024	0.28191903	0.59544616	0.7703891	Nudt12
ENSMUSG00000024231.16	830.244452	-0.0198279	0.0023945	68.0757418	1.57E-16	5.29E-15	Cul2
ENSMUSG00000024232.3	77.4035418	-0.0017269	0.00352997	0.23811243	0.62557275	0.79456599	Bambi
ENSMUSG00000024234.8	289.885224	-0.0023039	0.00154537	2.20782816	0.13731186	0.26883354	Mtpap
ENSMUSG00000024235.12	68.6596476	0.03640101	0.00447573	61.6153707	4.18E-15	1.19E-13	Map3k8
ENSMUSG00000024236.19	685.709899	-0.0005519	0.00386327	0.02038304	0.88647246	0.96775467	Svil
ENSMUSG00000024238.16	419.268657	0.00079145	0.0043273	0.0323286	0.85730836	0.95173092	Zeb1
ENSMUSG00000024240.14	314.147246	0.00317197	0.00261489	1.45764862	0.22730462	0.39359614	Epc1
ENSMUSG00000024241.8	779.166202	-0.0048063	0.00287253	2.70764584	0.09986827	0.20997442	Sos1
ENSMUSG00000024242.16	537.985248	0.00290285	0.0024218	1.41768801	0.23378432	0.40186845	Map4k3
ENSMUSG00000024245.5	24.0030285	0.03340412	0.00699583	22.9025439	1.70E-06	1.42E-05	Tmem178
ENSMUSG00000024246.10	43.3905748	0.00115539	0.00319712	0.12949441	0.71895683	0.86242843	Thumpd2
ENSMUSG00000024247.15	313.482019	-0.0036358	0.00355999	1.03792499	0.30830456	0.49326219	Pkdcc
ENSMUSG00000024248.15	2299.17071	-0.006264	0.00332767	3.56611004	0.0589703	0.13791507	Cox7a2l
ENSMUSG00000024251.11	144.4927	0.00249233	0.00371217	0.44631164	0.50409172	0.69102886	Thada
ENSMUSG00000024253.10	20.4539562	0.02979238	0.00630744	21.9111389	2.86E-06	2.27E-05	Dync2li1

ENSMUSG00000024254.16	1402.72457	0.00152015	0.0055447	0.07717991	0.78115612	0.90419025	Abcg8
ENSMUSG00000024256.8	2.96824796	-0.0046417	0.02764765	0.10180357	0.74967592	0.88320519	Adcyap1
ENSMUSG00000024258.5	95.4616719	-0.0044302	0.00343325	1.66974961	0.19629208	0.35301143	Polr2d
ENSMUSG00000024259.10	889.297848	-0.0063255	0.00194043	10.5890757	0.00113758	0.00491734	Slc25a46
ENSMUSG00000024260.16	366.794803	0.00127698	0.00280966	0.20429069	0.65127959	0.81396145	Sap130
ENSMUSG00000024261.7	19.6679767	0.02885144	0.01320998	4.76024669	0.02912437	0.07753589	Syt4
ENSMUSG00000024268.17	43.204297	0.01773945	0.00813309	4.20747871	0.04024612	0.1011449	Celf4
ENSMUSG00000024269.12	112.360438	-0.0030074	0.00227338	1.75415803	0.18535486	0.33800679	Tpgs2
ENSMUSG00000024270.12	72.3404368	0.01123631	0.00492246	4.98301047	0.0255974	0.06983694	Slc39a6
ENSMUSG00000024271.9	502.642808	0.0019844	0.00141857	1.96586732	0.16088721	0.30280763	Elp2
ENSMUSG00000024273.9	110.47552	-0.0009729	0.00313894	0.09653802	0.75602437	0.88732041	2700062C07Rik
ENSMUSG00000024276.8	383.67902	-0.0083696	0.00289186	8.20139371	0.00418582	0.01519745	Zfp397
ENSMUSG00000024277.15	360.381764	0.01490973	0.00345718	18.6679706	1.56E-05	0.00010645	Mapre2
ENSMUSG00000024283.16	1145.26947	0.00044738	0.0027019	0.02690644	0.86970608	0.95837696	Wac
ENSMUSG00000024286.9	1271.00407	-0.0053473	0.0023502	5.20024268	0.02258373	0.06296961	Ccny
ENSMUSG00000024287.9	488.866403	-0.0022832	0.00306994	0.54920684	0.45864196	0.65026119	Thoc1
ENSMUSG00000024290.10	1815.15038	0.00202801	0.003734	0.28747558	0.5918428	0.76762188	Rock1
ENSMUSG00000024292.16	3719.06882	-0.0153407	0.00464342	10.7047603	0.0010686	0.00465363	Cyp4f14
ENSMUSG00000024293.17	316.101357	0.00230453	0.00246332	0.86564452	0.35216392	0.54065861	Esco1
ENSMUSG00000024294.16	1398.85834	0.00116085	0.00252401	0.20619797	0.64976386	0.81314428	Mib1
ENSMUSG00000024298.16	1307.36819	-0.0041289	0.00341809	1.41699078	0.23389934	0.40200757	Zfp871
ENSMUSG00000024299.18	146.358514	0.01120886	0.00403976	7.62434346	0.00575856	0.019962	Adamts10
ENSMUSG00000024300.18	103.133532	0.06653833	0.00579302	121.197331	3.46E-28	5.26E-26	Myo1f
ENSMUSG00000024301.11	9.45356798	0.01503397	0.00853529	2.83151966	0.09243086	0.1976316	Kifc5b
ENSMUSG00000024302.17	51.697041	0.03061296	0.0095248	10.0490709	0.00152425	0.00636412	Dtna
ENSMUSG00000024304.15	2172.79103	-0.0013756	0.00249469	0.30091477	0.58330951	0.76069546	Cdh2
ENSMUSG00000024306.14	2.09239093	-0.0116206	0.06823934	-0.0292112	1	1	Ccdc178
ENSMUSG00000024308.16	1718.56441	0.01527743	0.00268581	32.3150067	1.31E-08	1.56E-07	Tapbp
ENSMUSG00000024309.16	422.948136	-0.0100448	0.00384514	6.94871761	0.00838793	0.02748129	Pfdn6
ENSMUSG00000024312.11	294.089326	-0.0025463	0.00219713	1.35462997	0.24447033	0.41509446	Wdr46
ENSMUSG00000024313.9	9.07662278	0.00723608	0.01177381	0.34038172	0.55960897	0.74097216	Mep1b
ENSMUSG00000024317.16	407.557781	-0.001836	0.00253192	0.52053596	0.47061314	0.66149473	Rnf138
ENSMUSG00000024319.19	398.353216	-0.0095623	0.0041603	5.35222021	0.0206959	0.05864274	Vps52
ENSMUSG00000024325.9	259.133752	-0.0033331	0.00280017	1.39753857	0.23713613	0.40606162	Ring1
ENSMUSG00000024327.17	1369.51294	-0.009163	0.00322997	7.99299257	0.00469587	0.01674329	Slc39a7
ENSMUSG00000024330.18	23.0544014	0.00772761	0.00858488	0.73748091	0.39046903	0.58189186	Col11a2
ENSMUSG00000024331.12	899.022612	0.00408708	0.00309972	1.73767782	0.18743333	0.34079576	Dsc2
ENSMUSG00000024334.10	8.74273461	0.06924299	0.00829475	60.640576	6.85E-15	1.93E-13	H2-Oa
ENSMUSG00000024335.21	2467.15786	-0.0004832	0.00393789	0.01503762	0.90240171	0.97691499	Brd2
ENSMUSG00000024338.16	652.748247	0.02904453	0.00369672	60.4496662	7.55E-15	2.11E-13	Psmb8
ENSMUSG00000024339.13	485.839437	0.01754127	0.00300759	33.5958803	6.78E-09	8.41E-08	Tap2
ENSMUSG00000024340.17	2.1465635	0.06863971	0.01205178	28.1655272	1.11E-07	1.15E-06	Btnl2
ENSMUSG00000024346.7	642.556977	-0.0083046	0.00327516	6.49675438	0.01080716	0.03407723	Pfdn1
ENSMUSG00000024347.17	4.42725131	0.04846383	0.01408428	11.8087622	0.00058953	0.00276122	Psd2
ENSMUSG00000024349.11	70.6982189	0.04253468	0.0058422	47.7757459	4.78E-12	9.20E-11	Sting1
ENSMUSG00000024350.7	214.892844	-0.0051306	0.00332452	2.34077413	0.12602691	0.25166969	Dnajc18
ENSMUSG00000024352.13	43.6050558	0.01586838	0.00482151	10.5302382	0.00117437	0.00505543	Spata24
ENSMUSG00000024353.6	25.2924438	0.10365755	0.01095237	65.1173433	7.06E-16	2.21E-14	Mzb1
ENSMUSG00000024354.14	1250.82751	-0.0013596	0.00530451	0.06645076	0.79657636	0.91408241	Slc23a1
ENSMUSG00000024357.11	885.589542	-0.002667	0.00251954	1.10831693	0.2924481	0.47439035	Sil1
ENSMUSG00000024359.11	8861.16	-0.0090573	0.00215765	17.731887	2.54E-05	0.00016598	Hspa9
ENSMUSG00000024360.9	1738.16288	-0.0010167	0.00184655	0.2976851	0.58533731	0.76232831	Etf1
ENSMUSG00000024365.10	16.9731248	-0.0276011	0.01056086	5.22199067	0.02230301	0.06230888	Cyp21a1
ENSMUSG00000024369.17	371.441029	-0.0032446	0.00270159	1.44066769	0.23003132	0.39729927	Nelfe
ENSMUSG00000024370.18	232.034275	-0.0013743	0.00260476	0.2753049	0.5997952	0.77397798	Cdc23
ENSMUSG00000024371.15	1765.64448	-0.0039549	0.00341142	1.36962463	0.24187639	0.4119536	C2
ENSMUSG00000024376.8	15.2591938	0.02613466	0.00899554	7.92164437	0.00488469	0.01733005	Epb41l4a
ENSMUSG00000024378.10	2200.43738	-0.0072156	0.00412987	2.95690752	0.08551135	0.18581819	Stard4
ENSMUSG00000024379.5	4.61401265	0.03932264	0.0102457	13.3504554	0.00025836	0.00133633	Tslp
ENSMUSG00000024381.17	243.896661	0.00878123	0.00270355	10.4310141	0.00123917	0.00531014	Bin1
ENSMUSG00000024382.14	337.294859	-0.0002576	0.00194851	0.01773684	0.89405123	0.97274159	Ercc3

ENSMUSG00000024383.10	503.759405	0.00054808	0.00296054	0.03364559	0.85446273	0.95018189	Map3k2
ENSMUSG00000024384.5	859.023577	-0.0031956	0.0032069	0.97914927	0.32240893	0.50878743	Iws1
ENSMUSG00000024386.10	6893.6025	-0.0083373	0.00458769	3.30626206	0.06901631	0.15643078	Proc
ENSMUSG00000024387.14	1018.01621	-0.0074212	0.00240201	9.66249116	0.00188068	0.00765979	Csnk2b
ENSMUSG00000024388.12	6.17135487	0.06104359	0.0200145	-0.2099859	1	1	Myo7b
ENSMUSG00000024391.8	3648.62138	-0.0226534	0.00530708	18.6483495	1.57E-05	0.00010749	Apom
ENSMUSG00000024392.18	1173.99654	-0.0073325	0.00242357	9.20882244	0.00240852	0.00946472	Bag6
ENSMUSG00000024393.15	1976.24882	-0.0025514	0.00514294	0.24705138	0.61915899	0.78984714	Prrc2a
ENSMUSG00000024395.10	1310.06448	-0.0200198	0.00498848	15.7695062	7.15E-05	0.00042357	Lims2
ENSMUSG00000024397.15	148.113818	0.06292424	0.00633728	90.272468	2.08E-21	1.25E-19	Aif1
ENSMUSG00000024399.6	27.9147862	0.07276784	0.00721395	89.0312851	3.89E-21	2.29E-19	Ltb
ENSMUSG00000024400.17	695.909268	-0.0097021	0.00221643	18.9284949	1.36E-05	9.39E-05	Wdr33
ENSMUSG00000024401.15	9.76400828	0.10195985	0.00911816	108.458412	2.13E-25	2.10E-23	Tnf
ENSMUSG00000024402.6	1.40210599	0.08151437	0.01582027	24.5528514	7.23E-07	6.36E-06	Lta
ENSMUSG00000024403.17	42.1401509	0.01119981	0.00521674	4.55119561	0.03289577	0.08577994	Atp6v1g2
ENSMUSG00000024404.8	1483.50905	-0.0019388	0.00200818	0.9241267	0.33639376	0.52380582	Riok3
ENSMUSG00000024410.16	354.938326	-0.0120848	0.00257879	22.0816236	2.61E-06	2.09E-05	Rmc1
ENSMUSG00000024411.12	81.9422702	-0.0149528	0.00712984	4.11568446	0.04248733	0.1056851	Aqp4
ENSMUSG00000024413.15	1984.49388	-0.0009144	0.0030837	0.08790125	0.76686244	0.8948969	Npc1
ENSMUSG00000024414.14	682.167706	-0.0007182	0.00284257	0.06466504	0.79926904	0.91547978	Mrpl27
ENSMUSG00000024420.10	20.888655	0.00992701	0.0073007	1.74136658	0.18696576	0.34012916	Zfp521
ENSMUSG00000024421.17	100.749943	-0.041152	0.00581965	45.323066	1.67E-11	2.99E-10	Lama3
ENSMUSG00000024422.15	652.08243	-0.0112492	0.0034415	10.7286248	0.00105491	0.00460249	Dhx16
ENSMUSG00000024423.7	314.020245	0.02387028	0.00384002	36.4764809	1.55E-09	2.08E-08	Impact
ENSMUSG00000024424.16	4886.95401	-0.0401984	0.00499944	61.4271889	4.59E-15	1.31E-13	Ttc39c
ENSMUSG00000024425.5	4079.73804	-0.0041381	0.0025581	2.60198672	0.10672985	0.22081844	Ndfip1
ENSMUSG00000024426.18	202.021828	-0.0176369	0.00350273	24.8895363	6.07E-07	5.44E-06	Atat1
ENSMUSG00000024427.8	147.913238	-0.0133956	0.00464067	8.36379237	0.00382771	0.01406893	Spry4
ENSMUSG00000024429.10	404.885001	0.00163253	0.00300352	0.29810682	0.58507171	0.76202449	Gnl1
ENSMUSG00000024430.15	49.9548408	-0.0580536	0.00785388	50.4596262	1.22E-12	2.51E-11	Cabyr
ENSMUSG00000024431.16	1696.07473	-0.0014239	0.00247681	0.32556495	0.56828235	0.74796359	Nr3c1
ENSMUSG00000024436.17	708.320022	-0.0114612	0.00256143	20.1899408	7.01E-06	5.15E-05	Mrps18b
ENSMUSG00000024440.14	123.101692	-0.0070374	0.00343925	4.21963117	0.03995885	0.10053006	Pcdh12
ENSMUSG00000024442.7	552.914288	-0.0003907	0.00209028	0.03477672	0.85206431	0.9487673	Dele1
ENSMUSG00000024446.14	136.180078	-0.0114556	0.00314406	13.372586	0.00025533	0.0013224	Rpp21
ENSMUSG00000024451.10	235.152289	-0.0003747	0.00374337	0.00989186	0.92077478	0.98692997	Arap3
ENSMUSG00000024454.17	620.768959	0.00035908	0.00192758	0.03527271	0.85102536	0.9483632	Hdac3
ENSMUSG00000024456.18	1148.88382	0.00407244	0.00283261	2.06285947	0.15092672	0.28875386	Diaph1
ENSMUSG00000024457.18	634.024288	-0.0011099	0.00276078	0.16146559	0.68781076	0.8413393	Trim26
ENSMUSG00000024462.18	105.647657	0.02438254	0.00606389	15.8032821	7.03E-05	0.0004167	Gabbr1
ENSMUSG00000024471.13	23.1534179	0.08587319	0.03726559	-3.1585008	1	1	Myot
ENSMUSG00000024472.10	320.988882	0.00595948	0.00459692	1.68826568	0.19382975	0.34938253	Dcp2
ENSMUSG00000024474.9	1200.83298	0.00169445	0.00205848	0.67310075	0.41197263	0.60345215	Ik
ENSMUSG00000024477.5	613.674169	0.00019989	0.00299174	0.00435228	0.94740025	1	Pggt1b
ENSMUSG00000024479.4	1292.82233	-0.0118017	0.00365798	10.2547005	0.00136337	0.00575867	Mal2
ENSMUSG00000024480.10	914.168162	-0.0044668	0.00284605	2.38522933	0.12248692	0.24616363	Ap3s1
ENSMUSG00000024481.6	3.13734638	-0.0100304	0.01319631	0.53273271	0.46546023	0.65640329	Lvrn
ENSMUSG00000024483.20	6273.71795	-0.0003911	0.00524276	0.00554887	0.94061992	0.99727868	Ankhd1
ENSMUSG00000024485.17	13.1924178	0.11472208	0.01014724	116.739751	3.27E-27	4.22E-25	Slc4a9
ENSMUSG00000024486.7	41.7524693	0.02681313	0.00750311	12.1009425	0.00050396	0.00241301	Hbegf
ENSMUSG00000024487.6	871.640193	-0.0012659	0.00228637	0.30078803	0.58338882	0.76075682	Yipf5
ENSMUSG00000024491.17	437.324237	0.00619423	0.00353535	2.99137641	0.08370899	0.18262512	Rbm27
ENSMUSG00000024493.10	543.074326	-0.0008199	0.0029362	0.07810157	0.77988678	0.90341834	Lars
ENSMUSG00000024498.17	733.415934	0.00077505	0.00386768	0.03985928	0.84175598	0.94198449	Tcerg1
ENSMUSG00000024500.21	10.6279265	0.0227363	0.00933196	5.96341398	0.01460573	0.04395137	Ppp2r2b
ENSMUSG00000024501.21	73.6860946	0.01056938	0.00705188	2.23204234	0.13517506	0.26557593	Dpysl3
ENSMUSG00000024502.11	4.36806512	0.01972416	0.01910319	1.02483958	0.31137379	0.49645523	Jakmip2
ENSMUSG00000024503.4	15.5799858	0.00628696	0.01634724	0.1217592	0.72713427	0.86818816	Spink1
ENSMUSG00000024505.17	49.2700502	0.00563926	0.00332176	2.74538673	0.09753548	0.20613546	Dtwd2
ENSMUSG00000024507.7	7037.87074	-0.0036187	0.00287763	1.49784083	0.22100388	0.38600397	Hsd17b4
ENSMUSG00000024511.16	32.5124985	0.02454944	0.00544832	20.0115993	7.70E-06	5.58E-05	Rab27b

ENSMUSG00000024513.17	722.780739	-4.43E-06	0.00204101	4.45E-06	0.99831752	1	Mbd2
ENSMUSG00000024515.14	563.41222	-0.0031845	0.00255336	1.51789717	0.21793799	0.38220577	Smad4
ENSMUSG00000024516.14	813.477624	0.00359592	0.00382023	0.88694967	0.34630526	0.53462206	Sec11c
ENSMUSG00000024521.9	29.0186986	0.06408049	0.00636101	93.2503354	4.61E-22	3.09E-20	Pmaip1
ENSMUSG00000024524.18	28.3053525	0.01316414	0.00634645	4.18577256	0.04076463	0.10226277	Gnal
ENSMUSG00000024525.8	109.26796	-0.0072145	0.00733157	0.93861464	0.33263438	0.51969244	Impa2
ENSMUSG00000024526.10	88.8723613	0.22328284	0.01704649	56.0044225	7.23E-14	1.75E-12	Cidea
ENSMUSG00000024527.11	1247.15085	-0.0108801	0.00205783	28.3299709	1.02E-07	1.06E-06	Afg3l2
ENSMUSG00000024528.9	121.951986	0.00622374	0.00285406	4.73122741	0.02961974	0.07865648	Srfbp1
ENSMUSG00000024529.15	13.0661091	0.02651301	0.00940102	7.58265449	0.00589326	0.02035414	Lox
ENSMUSG00000024530.9	1.8012074	0.03936534	0.02660863	2.78055422	0.09541515	0.20267126	Prelid3a
ENSMUSG00000024532.14	0.74001481	-0.0233709	0.16537576	0.04893071	0.82493448	0.93068519	1700034E13Rik
ENSMUSG00000024533.18	35.3871311	0.04425136	0.00695604	37.0543033	1.15E-09	1.58E-08	Spire1
ENSMUSG00000024534.16	17.4601691	0.01284196	0.00625921	4.01240379	0.04516671	0.11103836	Sncaip
ENSMUSG00000024535.17	200.604202	0.00251662	0.00258282	0.9444207	0.33114354	0.5180996	Snx24
ENSMUSG00000024537.6	207.53409	-0.0053745	0.00259006	4.29667806	0.03818689	0.09677405	Psmg2
ENSMUSG00000024538.9	92.9545841	0.01316506	0.0052461	6.26756412	0.01229681	0.0379177	Ppic
ENSMUSG00000024539.18	238.475119	0.00224062	0.00221206	1.01328609	0.31411688	0.49950128	Ptpn2
ENSMUSG00000024542.10	88.6691848	0.02536161	0.00546982	20.9172796	4.80E-06	3.64E-05	Cep192
ENSMUSG00000024544.10	132.861786	-0.0082245	0.00328118	6.26281342	0.01232982	0.03799468	Ldlrad4
ENSMUSG00000024548.12	32.7449652	0.0759325	0.00645488	127.919727	1.17E-29	2.24E-27	Setbp1
ENSMUSG00000024552.17	2.74833454	0.00675996	0.03386069	0.21117402	0.64584877	0.81065346	Slc14a2
ENSMUSG00000024556.5	76.2371682	0.03010657	0.00516042	31.8272169	1.69E-08	1.96E-07	Me2
ENSMUSG00000024558.13	25.7393795	0.03058969	0.00573184	27.4803381	1.59E-07	1.59E-06	Mapk4
ENSMUSG00000024560.8	513.284266	-0.0095401	0.00235328	16.5599293	4.71E-05	0.00029157	Cxxc1
ENSMUSG00000024561.12	861.626585	-0.0214644	0.00370554	32.0818019	1.48E-08	1.75E-07	Mbd1
ENSMUSG00000024563.17	446.981844	0.00102926	0.00308644	0.10920827	0.74104732	0.87738276	Smad2
ENSMUSG00000024565.11	2.26436597	0.00202924	0.02725877	0.02673792	0.87011113	0.95863421	Sall3
ENSMUSG00000024566.11	603.647531	0.00134399	0.00194226	0.47666593	0.48993641	0.67777325	Atp9b
ENSMUSG00000024570.8	488.074608	-0.0055954	0.00288342	3.77408265	0.05205244	0.12453933	Rbfa
ENSMUSG00000024571.16	1025.86596	0.00015039	0.00172585	0.0076656	0.93023163	0.99177898	Naa12
ENSMUSG00000024576.15	2074.22381	0.00096993	0.00201557	0.22913329	0.63216723	0.79999636	Csnk1a1
ENSMUSG00000024579.8	48.5362117	0.01122388	0.0055348	4.08861126	0.0431731	0.10709726	Pcyox1l
ENSMUSG00000024580.9	1125.90724	0.00528803	0.00340494	2.30571804	0.1288987	0.25613174	Grpel2
ENSMUSG00000024581.14	378.314978	0.00368376	0.00258277	2.00465741	0.15681672	0.297165	Napg
ENSMUSG00000024583.4	2022.04853	-0.0063671	0.0020741	9.33078465	0.00225336	0.008961	Txnl1
ENSMUSG00000024587.11	2234.4183	-0.0043407	0.00274234	2.45514028	0.1171407	0.23766715	Nars
ENSMUSG00000024588.11	3193.21305	-0.003041	0.00203593	2.23153144	0.13521976	0.26563995	Fech
ENSMUSG00000024589.19	1103.55215	-0.0030991	0.00395786	0.57923924	0.44661067	0.63846022	Nedd4l
ENSMUSG00000024590.9	100.254969	0.01227717	0.00410449	8.63041064	0.00330597	0.0124203	Lmnb1
ENSMUSG00000024592.16	373.520513	-0.0035538	0.00299545	1.35560823	0.24430007	0.41489497	C330018D20Rik
ENSMUSG00000024593.16	6.81629016	0.01682844	0.02140605	0.91434962	0.33896296	0.52659753	Megf10
ENSMUSG00000024594.10	668.183727	-0.0015132	0.00281652	0.28865035	0.59108672	0.76703516	Prrc1
ENSMUSG00000024597.12	225.035809	-0.0036617	0.00298473	1.48193112	0.22347262	0.38894957	Slc12a2
ENSMUSG00000024598.10	12.2293696	0.03757226	0.01892952	4.8833542	0.02711688	0.07317833	Fbn2
ENSMUSG00000024600.9	4.00013687	0.01687181	0.00731801	5.50409299	0.01897202	0.05447999	Slc27a6
ENSMUSG00000024601.10	1893.82433	-0.0064601	0.00173633	13.8105919	0.00020219	0.00107741	Isoc1
ENSMUSG00000024603.9	608.756661	0.00691165	0.00199558	11.7394003	0.00061191	0.00285283	Dctn4
ENSMUSG00000024604.8	316.163665	0.00770465	0.00208674	13.5695334	0.00022989	0.00120598	Rbm22
ENSMUSG00000024608.12	7697.60543	-0.0045239	0.00524849	0.74443279	0.38824459	0.57976144	Rps14
ENSMUSG00000024610.17	2806.04145	0.07623964	0.00555466	172.305684	2.32E-39	1.48E-36	Cd74
ENSMUSG00000024613.17	219.589755	0.00960095	0.00383355	6.14168968	0.01320311	0.04023366	Tcof1
ENSMUSG00000024614.8	587.019753	0.01099573	0.0029041	13.7958293	0.00020379	0.00108451	Tmx3
ENSMUSG00000024617.17	79.0231015	0.03158391	0.01164323	6.66676962	0.00982271	0.03141402	Camk2a
ENSMUSG00000024620.12	218.172531	0.0217595	0.00421788	26.3298757	2.88E-07	2.74E-06	Pdgfrb
ENSMUSG00000024621.17	1376.57756	0.04745344	0.00509309	82.9579473	8.38E-20	4.29E-18	Csf1r
ENSMUSG00000024622.9	261.503149	-0.0065669	0.00208269	9.98374216	0.00157928	0.00655403	Hmgxb3
ENSMUSG00000024639.6	457.088084	0.00624926	0.00252513	6.00382334	0.01427491	0.04309331	Gnaq
ENSMUSG00000024640.10	46.0087756	0.02179806	0.00995824	4.18267321	0.04083924	0.10238459	Psat1
ENSMUSG00000024642.19	239.24684	-0.0005478	0.00306345	0.03134084	0.85948213	0.95293308	Tle4
ENSMUSG00000024644.18	791.942844	0.01231613	0.00352368	12.1443684	0.00049236	0.00236274	Cndp2

ENSMUSG00000024645.6	506.242733	-0.0109102	0.00265931	16.8246497	4.10E-05	0.00025743	Timm21
ENSMUSG00000024646.15	16264.1156	-0.0033017	0.00366139	0.81963329	0.3652874	0.55506176	Cyb5a
ENSMUSG00000024650.6	21.5189372	-0.0273518	0.02783572	1.40901681	0.23521968	0.40368852	Slc22a6
ENSMUSG00000024653.3	67.9509554	-0.0334753	0.04123154	0.40673048	0.52363366	0.70910166	Scgb1a1
ENSMUSG00000024654.9	316.188805	0.01221229	0.00369995	10.7751063	0.00102874	0.00450751	Asrgl1
ENSMUSG00000024659.16	146.929772	0.03605467	0.00584571	34.6530233	3.94E-09	5.06E-08	Anxa1
ENSMUSG00000024660.10	66.9227866	0.02653344	0.00763567	11.5447845	0.0006794	0.00313402	Incenp
ENSMUSG00000024661.8	23685.5442	-0.0011457	0.00281258	0.16583362	0.68384146	0.83826645	Fth1
ENSMUSG00000024663.21	104.043997	0.02178221	0.0043805	23.9826946	9.72E-07	8.37E-06	Rab3il1
ENSMUSG00000024664.6	108.910085	0.01015136	0.00642098	2.43651102	0.11853962	0.23994812	Fads3
ENSMUSG00000024665.9	13911.4444	-0.0035629	0.00437283	0.65733022	0.41750406	0.60894507	Fads2
ENSMUSG00000024666.7	32.1997532	0.00330176	0.00369328	0.79936722	0.37128263	0.56181896	Tmem138
ENSMUSG00000024667.13	57.5593644	-0.007235	0.00288218	6.26387119	0.01232247	0.03798316	Tmem216
ENSMUSG00000024668.9	612.724527	-0.008579	0.00287837	8.79128286	0.00302673	0.01151057	Sdhaf2
ENSMUSG00000024669.9	7.33643471	0.10481011	0.0092757	108.740492	1.85E-25	1.84E-23	Cd5
ENSMUSG00000024670.18	11.1131715	0.09958108	0.00824045	129.39651	5.55E-30	1.12E-27	Cd6
ENSMUSG00000024672.12	61.8114645	0.0993917	0.00823003	125.37173	4.22E-29	7.16E-27	Ms4a7
ENSMUSG00000024673.10	46.8443717	0.10242053	0.00777565	133.010326	8.99E-31	2.12E-28	Ms4a1
ENSMUSG00000024675.20	30.1682199	0.07048108	0.00905278	53.9963858	2.01E-13	4.63E-12	Ms4a4c
ENSMUSG00000024677.14	101.735513	0.073524	0.00742578	87.4643487	8.58E-21	4.82E-19	Ms4a6b
ENSMUSG00000024678.7	131.726919	0.02488118	0.003444	49.8870015	1.63E-12	3.32E-11	Ms4a4d
ENSMUSG00000024679.11	89.1141177	0.0733899	0.00719711	92.1987774	7.84E-22	5.07E-20	Ms4a6d
ENSMUSG00000024680.13	1.59480463	0.07370057	0.01686285	18.8891115	1.39E-05	9.58E-05	Ms4a2
ENSMUSG00000024681.12	2.47986044	0.14435514	0.03520392	5.07639521	0.02425375	0.06675016	Ms4a3
ENSMUSG00000024683.7	557.958388	-0.0068187	0.00243324	7.86236041	0.00504745	0.01783506	Mrpl16
ENSMUSG00000024687.12	1046.08554	-0.0012578	0.00221013	0.32306396	0.56977211	0.74954794	Osbp
ENSMUSG00000024691.14	108.669306	0.03838152	0.00539541	46.994939	7.12E-12	1.35E-10	Fam111a
ENSMUSG00000024694.10	2997.75404	-0.0516088	0.00564537	77.4719382	1.35E-18	5.84E-17	Keg1
ENSMUSG00000024695.17	1876.34719	-0.0066177	0.00217199	9.17836617	0.00244893	0.00960112	Zfp91
ENSMUSG00000024696.10	47.2173479	0.06290655	0.00573263	110.693498	6.91E-26	7.30E-24	Lpxn
ENSMUSG00000024697.5	52.0570938	-0.0149358	0.00511084	8.50483634	0.00354204	0.0131729	Gna14
ENSMUSG00000024712.10	1083.13696	0.00485134	0.00196036	6.06836716	0.01376241	0.0417224	Rfk
ENSMUSG00000024713.17	231.637949	0.00732291	0.00310164	5.54131687	0.0185726	0.05354521	Pcsk5
ENSMUSG00000024725.14	874.727709	0.01051955	0.00263396	15.6431267	7.65E-05	0.00044958	Ostf1
ENSMUSG00000024726.11	467.642707	-0.010808	0.00357141	8.93807245	0.00279287	0.01075122	Carnmt1
ENSMUSG00000024727.10	6.84697932	0.0227567	0.00842936	7.48354307	0.00622654	0.02132722	Trpm6
ENSMUSG00000024730.8	33.987948	0.06500026	0.00818518	51.8129381	6.10E-13	1.32E-11	Ms4a8a
ENSMUSG00000024732.8	187.675319	-0.0013364	0.00341827	0.1516992	0.6969168	0.84777346	Ccdc86
ENSMUSG00000024735.14	1460.05942	0.00299124	0.00298796	0.99174182	0.31931703	0.50517585	Prpf19
ENSMUSG00000024736.16	46.2416027	0.0186465	0.00648027	8.26767436	0.00403572	0.01473702	Tmem132a
ENSMUSG00000024737.3	164.658909	0.05483178	0.00497332	114.555026	9.85E-27	1.16E-24	Slc15a3
ENSMUSG00000024740.11	3545.86415	-0.0060177	0.00161146	13.9696971	0.00018578	0.00099996	Ddb1
ENSMUSG00000024742.16	77.4923403	0.01671506	0.00406958	16.7306679	4.31E-05	0.0002688	Fen1
ENSMUSG00000024743.17	49.7945912	-0.0042127	0.00735408	0.32539325	0.56838439	0.74805614	Syt7
ENSMUSG00000024747.4	3804.25144	0.01142055	0.0047763	5.43846071	0.01969802	0.05619225	Aldh1a7
ENSMUSG00000024750.12	1088.86555	0.0039873	0.00208287	3.58685785	0.05823826	0.13667693	Zfand5
ENSMUSG00000024754.14	322.312552	-0.002295	0.00302732	0.57280361	0.44914698	0.64075614	Cemip2
ENSMUSG00000024757.8	5.07646621	-0.0085125	0.02368787	0.114295	0.73530634	0.87356749	Slc22a19
ENSMUSG00000024758.16	1884.78876	0.00375545	0.00240351	2.42370103	0.11951227	0.24141066	Rtn3
ENSMUSG00000024759.15	2370.75586	0.00036255	0.00288933	0.0156078	0.90057802	0.97601489	Atl3
ENSMUSG00000024764.11	224.249316	0.00064661	0.00208835	0.0968927	0.75559087	0.88698817	Naa40
ENSMUSG00000024766.16	76.2648871	0.01186934	0.00409372	8.26874832	0.00403333	0.01473059	Lipo3
ENSMUSG00000024767.12	688.622899	0.00093953	0.00223868	0.17636478	0.67451608	0.83155036	Otub1
ENSMUSG00000024769.8	250.173871	0.0020161	0.00363031	0.31343666	0.57557893	0.75420025	Cdc42bpg
ENSMUSG00000024772.10	757.757902	0.01209006	0.00294099	16.4596263	4.97E-05	0.00030485	Ehd1
ENSMUSG00000024773.16	822.561772	-0.0129384	0.0040346	10.0559549	0.00151856	0.00634488	Atg2a
ENSMUSG00000024776.19	28.593984	0.05076425	0.00738531	41.8820339	9.69E-11	1.56E-09	Stambpl1
ENSMUSG00000024777.11	165.169968	-0.0064343	0.00266553	5.82689978	0.01578291	0.04678344	Ppp2r5b
ENSMUSG00000024778.14	370.271816	0.00040732	0.00352787	0.01293457	0.90945164	0.98051784	Fas
ENSMUSG00000024780.8	1097.39554	-0.0173773	0.00220898	61.968854	3.49E-15	1.01E-13	Cdc37l1
ENSMUSG00000024781.17	4701.16987	0.01022465	0.00322775	9.79790756	0.00174711	0.00717022	Lipa

ENSMUSG00000024782.8	6481.58619	-0.0058335	0.00215813	7.29593532	0.00691108	0.02338661	Ak3
ENSMUSG00000024785.8	2331.28219	-0.0177953	0.00354846	24.8357873	6.24E-07	5.59E-06	Rcl1
ENSMUSG00000024787.15	185.895648	-0.0008217	0.00244522	0.11293093	0.73683168	0.87449974	Snx15
ENSMUSG00000024789.14	370.145027	0.01120823	0.00272792	16.5570964	4.72E-05	0.00029193	Jak2
ENSMUSG00000024790.9	92.5638363	-0.005281	0.00276894	3.68339004	0.054957	0.13040401	Sac3d1
ENSMUSG00000024791.11	7.62610916	0.0296052	0.00965391	8.87656911	0.00288854	0.01106351	Cdca5
ENSMUSG00000024792.10	193.025847	-0.0031183	0.00221356	1.96587824	0.16088604	0.30280763	Zfpl1
ENSMUSG00000024793.15	4.23607133	0.01026196	0.00897897	1.28014652	0.25787179	0.43132857	Tnfrsf25
ENSMUSG00000024795.12	42.7311249	0.03407068	0.00901772	13.1696824	0.00028451	0.0014566	Kif20b
ENSMUSG00000024797.15	316.129996	0.00346851	0.00273312	1.61303232	0.20406608	0.36388466	Vps51
ENSMUSG00000024798.16	2.48965156	0.07951448	0.01378903	33.7880771	6.15E-09	7.67E-08	Htr7
ENSMUSG00000024799.17	2216.66902	-0.0157917	0.00425175	13.3528652	0.00025803	0.0013352	Tm7sf2
ENSMUSG00000024800.9	97.0255933	-0.0011573	0.00315753	0.13123127	0.71715882	0.86116016	Rpp30
ENSMUSG00000024803.10	1416.24623	0.02440063	0.01161548	0.34127776	0.5590926	0.74061224	Ankrd1
ENSMUSG00000024805.17	419.795437	-0.0001185	0.00278581	0.00181459	0.96602202	1	Pcgf5
ENSMUSG00000024806.5	8.61365789	-0.0046691	0.06022927	-0.0207956	1	1	Mlana
ENSMUSG00000024807.19	1399.52113	-0.0097296	0.00518879	3.23611262	0.07203136	0.16201904	Syvn1
ENSMUSG00000024810.17	55.8455924	0.02983985	0.00563801	27.0038209	2.03E-07	2.00E-06	Il33
ENSMUSG00000024811.13	1157.02177	0.00541113	0.00223662	5.83263351	0.01573156	0.04666639	Tnks2
ENSMUSG00000024812.12	433.385936	0.00046361	0.00306854	0.02242218	0.88096944	0.9644254	Tjp2
ENSMUSG00000024816.13	321.801735	-0.0018125	0.00260711	0.48707955	0.48523295	0.6741773	Frmd8
ENSMUSG00000024817.14	589.87798	-0.0027469	0.00235296	1.34056099	0.24693506	0.41832814	Uhrf2
ENSMUSG00000024818.16	412.956095	-0.0099947	0.003569	7.64235147	0.00570135	0.01980152	Slc25a45
ENSMUSG00000024824.8	77.1818025	0.00833089	0.00440522	3.55385906	0.05940714	0.13878035	Rad9a
ENSMUSG00000024826.16	462.273137	-0.0032031	0.00265209	1.46931976	0.22545305	0.39148462	Dpf2
ENSMUSG00000024827.11	2710.81684	-0.0088657	0.00514648	2.94558822	0.08611234	0.18688312	Gldc
ENSMUSG00000024829.13	343.868615	-0.0139638	0.00274711	25.9089425	3.58E-07	3.36E-06	Mrpl21
ENSMUSG00000024830.18	282.260691	-0.0089237	0.0034629	6.7211763	0.00952748	0.03064436	Rps6kb2
ENSMUSG00000024831.15	90.1241516	-0.0057257	0.00299163	3.65233315	0.05599072	0.13248367	Ighmbp2
ENSMUSG00000024833.16	150.718451	0.00780492	0.00317595	6.0015502	0.01429331	0.04313231	Pola2
ENSMUSG00000024835.16	1135.0596	0.0129589	0.00277999	21.3218382	3.88E-06	3.01E-05	Coro1b
ENSMUSG00000024841.9	409.552748	-0.0074417	0.00271319	7.57148622	0.00592989	0.02045369	Eif1ad
ENSMUSG00000024842.9	8.83649866	0.04747348	0.00987164	20.0675175	7.48E-06	5.44E-05	Cabp4
ENSMUSG00000024843.16	1461.7725	-0.0060707	0.00704078	0.69930885	0.40301603	0.5946243	Chka
ENSMUSG00000024844.16	518.032318	-0.0084257	0.00318792	7.0446909	0.00795005	0.02624895	Banf1
ENSMUSG00000024845.20	767.406538	-0.0013359	0.00438895	0.0901849	0.76394222	0.89280137	Tmem134
ENSMUSG00000024847.16	389.946797	0.00063398	0.00296005	0.04476704	0.83243297	0.93560262	Aip
ENSMUSG00000024851.15	130.133055	0.04142156	0.00523559	60.2376695	8.41E-15	2.34E-13	Pitpnm1
ENSMUSG00000024853.11	1837.87854	-1.28E-05	0.00216125	3.55E-05	0.9952448	1	Sf3b2
ENSMUSG00000024854.5	235.774747	0.01149255	0.00338508	11.5391175	0.00068147	0.00314174	Pold4
ENSMUSG00000024855.11	82.6863285	0.02341202	0.00472057	24.623625	6.97E-07	6.16E-06	Pacs1
ENSMUSG00000024856.11	672.930541	-0.0001598	0.0033886	0.00218103	0.96275115	1	Cdk2ap2
ENSMUSG00000024857.17	4.862288	-0.0120926	0.01270008	0.94163159	0.33185859	0.51887416	Cabp2
ENSMUSG00000024858.15	595.382259	0.02366367	0.0039554	34.1194103	5.18E-09	6.54E-08	Grk2
ENSMUSG00000024862.17	126.597367	-0.0093302	0.00367188	6.45979805	0.01103422	0.03467976	Klc2
ENSMUSG00000024863.7	6514.01514	0.00895443	0.0055184	2.59379379	0.10728309	0.22174909	Mbl2
ENSMUSG00000024866.11	971.536204	-0.0071918	0.00385889	3.42035016	0.06439735	0.14810272	Acy3
ENSMUSG00000024867.15	6.46462637	0.0319244	0.01369983	4.65311399	0.03099727	0.08170855	Pip5k1b
ENSMUSG00000024869.11	37.4888214	-0.0148008	0.00571608	6.81259919	0.00905169	0.02936633	Gm49405
ENSMUSG00000024870.7	1763.71938	-0.0026023	0.00226395	1.32891434	0.24899845	0.42045228	Rab1b
ENSMUSG00000024871.12	47.8526263	-0.0292574	0.00707846	16.8379254	4.07E-05	0.00025572	Doc2g
ENSMUSG00000024873.8	3.89289058	0.016733	0.01101237	2.14654642	0.14289099	0.27711124	Cnih2
ENSMUSG00000024875.6	895.005656	-0.0067738	0.00298625	5.15198989	0.02321975	0.0644004	Yif1a
ENSMUSG00000024878.10	111.341554	-0.0066602	0.00284863	5.38644252	0.0202938	0.05766863	Zng1
ENSMUSG00000024883.9	16.0940751	0.00547626	0.01111578	0.22334186	0.6365053	0.803689	Rin1
ENSMUSG00000024885.10	62.875326	0.03689945	0.00485816	55.0768284	1.16E-13	2.73E-12	Aldh3b1
ENSMUSG00000024887.11	471.693351	0.01271613	0.00369828	11.617546	0.00065333	0.00302792	Asah2
ENSMUSG00000024889.6	147.815921	-0.0033834	0.00288206	1.38645093	0.23900537	0.40843216	Rce1
ENSMUSG00000024891.7	15.2665007	0.00052396	0.00570553	0.01027703	0.91925224	0.98619438	Slc29a2
ENSMUSG00000024892.18	8028.8354	-0.0030086	0.00317855	0.89336467	0.34456714	0.53284557	Pcx
ENSMUSG00000024896.10	722.255371	0.0078222	0.00196764	15.8733835	6.77E-05	0.00040296	Minpp1

ENSMUSG00000024897.10	18.6645391	0.04569748	0.00607554	55.5346634	9.18E-14	2.19E-12	Apba1
ENSMUSG00000024899.8	3916.95684	0.00862303	0.00376309	5.2641421	0.02176915	0.06100121	Papss2
ENSMUSG00000024900.6	6687.4411	-0.0027319	0.00370258	0.52488878	0.46876369	0.65983888	Cpt1a
ENSMUSG00000024901.16	63.6598077	-0.0019043	0.00499009	0.14286576	0.70544851	0.85378092	Peli3
ENSMUSG00000024902.6	645.679888	-0.0053619	0.00340888	2.49895013	0.11392222	0.23245691	Mrpl11
ENSMUSG00000024905.16	16.156647	0.00920396	0.00546071	2.86516248	0.09051657	0.19426058	Tesmin
ENSMUSG00000024906.11	141.008473	-0.0063504	0.00359304	3.15885933	0.07551593	0.1682364	Mus81
ENSMUSG00000024908.16	1304.89962	0.00235301	0.00216811	1.16091682	0.28127548	0.46099392	Ppp6r3
ENSMUSG00000024909.16	71.87372	0.01711857	0.00500319	11.7500891	0.0006084	0.0028393	Efemp2
ENSMUSG00000024910.6	16.1655243	0.06653452	0.00728525	73.974897	7.91E-18	3.14E-16	Ctsw
ENSMUSG00000024911.8	399.239397	-0.0028899	0.00416706	0.49650354	0.48104048	0.67063605	Fibp
ENSMUSG00000024912.7	1.01690339	0.04504112	0.02232043	5.20774472	0.02248649	0.06273554	Fosl1
ENSMUSG00000024913.17	1117.13594	-0.0047001	0.00345686	1.81691248	0.17768123	0.32722648	Lrp5
ENSMUSG00000024914.17	958.023967	0.00104333	0.00365367	0.08107923	0.77584017	0.90071698	Drap1
ENSMUSG00000024921.18	4001.45323	-0.0074719	0.00379138	3.87107211	0.04912513	0.11887405	Smarca2
ENSMUSG00000024922.3	1.53854522	-0.0117138	0.01978088	0.28853524	0.59116072	0.76703516	Ovol1
ENSMUSG00000024924.15	168.284729	0.02957921	0.00822796	13.1267977	0.0002911	0.00148613	Vldlr
ENSMUSG00000024925.12	506.263052	-0.0145898	0.0042556	11.5794495	0.00066685	0.00308216	Rnaseh2c
ENSMUSG00000024926.11	217.602575	0.00933765	0.00267098	11.9546355	0.00054512	0.00258225	Kat5
ENSMUSG00000024927.9	780.665018	0.00216415	0.00277709	0.59557661	0.44027075	0.63222075	Rela
ENSMUSG00000024935.12	23.581733	0.07287283	0.01211806	34.4407236	4.39E-09	5.59E-08	Slc1a1
ENSMUSG00000024937.16	283.567569	0.03769858	0.00421248	74.1067527	7.40E-18	2.95E-16	Ehbp1l1
ENSMUSG00000024939.7	142.807441	0.00903992	0.00347181	6.88681117	0.0086834	0.02833519	Fam89b
ENSMUSG00000024940.12	77.363573	0.0098017	0.00693259	1.87587332	0.17080391	0.31728606	Ltbp3
ENSMUSG00000024941.10	698.520979	-0.0046588	0.00191741	5.89307247	0.01520056	0.04534788	Scyl1
ENSMUSG00000024942.20	334.988058	0.01400333	0.00219141	39.7650161	2.86E-10	4.28E-09	Capn1
ENSMUSG00000024943.10	446.901528	-0.0094462	0.00340265	7.73993814	0.00540127	0.01889001	Smc5
ENSMUSG00000024944.7	97.5003944	-0.0011229	0.00418436	0.07140234	0.78930581	0.90976723	Arl2
ENSMUSG00000024947.18	379.661628	-0.006096	0.00297626	4.14346404	0.04179556	0.10427227	Men1
ENSMUSG00000024948.16	261.056754	0.00357176	0.00352911	1.02875528	0.3104512	0.49566267	Map4k2
ENSMUSG00000024949.18	1520.98116	-0.0008215	0.00257498	0.09987201	0.75198328	0.88492047	Sf1
ENSMUSG00000024953.18	4165.33776	-0.0143816	0.00304937	22.5344328	2.06E-06	1.69E-05	Prdx5
ENSMUSG00000024955.16	518.066711	-0.0069596	0.00327772	4.57845886	0.03237632	0.08469697	Esrra
ENSMUSG00000024957.10	2.07886793	0.00924519	0.01466456	0.43632558	0.50890129	0.69565567	Kcnk4
ENSMUSG00000024958.15	257.922105	-0.0149047	0.00287069	27.010629	2.02E-07	1.99E-06	Gpr137
ENSMUSG00000024959.15	301.560776	-0.002582	0.00314464	0.68111528	0.40920295	0.60092526	Bad
ENSMUSG00000024960.10	379.18963	-0.000192	0.00227269	0.00726998	0.93205139	0.99282915	Plcb3
ENSMUSG00000024962.14	262.953998	0.00038079	0.00430884	0.00745813	0.93117987	0.99234583	Vegfb
ENSMUSG00000024963.13	157.139821	0.00191895	0.00319413	0.36305749	0.54681307	0.72969854	Dnajc4
ENSMUSG00000024965.10	142.240765	0.05319879	0.0052077	97.7935826	4.64E-23	3.40E-21	Fermt3
ENSMUSG00000024966.10	1445.15874	-0.0117831	0.0032053	12.8715374	0.00033362	0.00167241	Stip1
ENSMUSG00000024968.15	3.56469383	0.01239743	0.01238483	0.96748141	0.32530911	0.51206225	Rcor2
ENSMUSG00000024969.17	356.81125	0.00477542	0.00272349	3.03382517	0.08154556	0.17889811	Mark2
ENSMUSG00000024970.6	60.5921956	-0.0018205	0.00471902	0.15401896	0.69472391	0.84615327	Spindoc
ENSMUSG00000024972.17	0.60497554	0.03740979	0.02847528	2.45328748	0.11727901	0.23784547	Lgals12
ENSMUSG00000024973.18	65.707941	-0.0346288	0.0468187	-0.0483642	1	1	Plaat5
ENSMUSG00000024974.11	766.235372	0.00485625	0.00313842	2.36516684	0.12407065	0.24856284	Smc3
ENSMUSG00000024975.14	1770.20962	-0.0104276	0.00347103	8.87308627	0.00289405	0.01108283	Pdcd4
ENSMUSG00000024976.15	730.380101	-0.003386	0.00250343	1.81077431	0.17841537	0.32827092	Shoc2
ENSMUSG00000024978.12	5920.01114	0.00781298	0.00452958	2.99249901	0.08365098	0.18254923	Gpam
ENSMUSG00000024979.14	3.03183927	0.01196802	0.01026119	1.32775662	0.24920471	0.42074032	Tectb
ENSMUSG00000024981.7	8749.78716	0.00509632	0.00423966	1.41781264	0.23376377	0.40186241	Acsl5
ENSMUSG00000024982.11	1255.2197	-0.0068317	0.00205506	11.0062658	0.00090804	0.00402956	Zdhhc6
ENSMUSG00000024983.11	247.782362	0.00524744	0.00217425	5.82746157	0.01577787	0.04678302	Vti1a
ENSMUSG00000024985.22	406.499018	0.00439256	0.00414416	1.12971925	0.28783523	0.46884543	Tcf7l2
ENSMUSG00000024986.13	1552.61854	-0.0125443	0.00558231	5.05881407	0.024501	0.06732066	Hhex
ENSMUSG00000024987.7	496.681479	0.04677039	0.00729176	37.0670518	1.14E-09	1.57E-08	Cyp26a1
ENSMUSG00000024989.16	9.80754743	0.03574307	0.01062612	10.276464	0.00134738	0.00570237	Cep55
ENSMUSG00000024990.14	43424.8419	-0.0061554	0.005239	1.37029983	0.24176038	0.41182726	Rbp4
ENSMUSG00000024991.9	4604.69839	-0.0035256	0.00283494	1.51803358	0.21791731	0.38219793	Eif3a
ENSMUSG00000024992.14	9.35165588	0.01965248	0.0087459	5.11024487	0.02378496	0.06571303	Pde6c

ENSMUSG00000024993.16	539.734863	0.00326129	0.002512	1.63296082	0.20129401	0.36003038	Dennd10
ENSMUSG00000024997.8	2068.83549	-0.0056121	0.00211505	7.03653819	0.00798633	0.02635219	Prdx3
ENSMUSG00000024998.18	255.614723	0.00171193	0.00395218	0.18670137	0.66567634	0.82501066	Plce1
ENSMUSG00000024999.8	147.983512	-0.0032528	0.0025729	1.57400824	0.20962587	0.3712725	Noc3l
ENSMUSG00000025001.12	84.4988185	0.0198478	0.00925747	4.3237616	0.03758375	0.09555356	Hells
ENSMUSG00000025002.6	205.861106	0.00496476	0.00706056	0.51150854	0.47448655	0.66518691	Cyp2c55
ENSMUSG00000025003.7	275.602895	0.06056735	0.00569911	113.077127	2.08E-26	2.40E-24	Cyp2c39
ENSMUSG00000025004.17	2186.55878	-0.0365509	0.01011296	11.4926449	0.00069872	0.00320623	Cyp2c40
ENSMUSG00000025006.19	591.361733	-0.0043985	0.00533827	0.64932929	0.42035258	0.61196444	Sorbs1
ENSMUSG00000025007.14	50.7371409	0.02778451	0.00760274	12.8960894	0.00032927	0.00165343	Aldh18a1
ENSMUSG00000025008.16	19.5570339	-0.0102134	0.00575138	3.18067776	0.07451382	0.1665387	Tctn3
ENSMUSG00000025010.15	63.1023959	-0.0067912	0.00249946	7.34611891	0.00672077	0.02282777	Ccnj
ENSMUSG00000025014.14	69.8404591	0.04253558	0.0058226	46.1271793	1.11E-11	2.03E-10	Dntt
ENSMUSG00000025016.12	3514.1669	-0.0018969	0.00175771	1.13993114	0.28566688	0.46616126	Tm9sf3
ENSMUSG00000025017.11	1335.70555	0.01680043	0.00429638	14.9421039	0.00011086	0.00062644	Pik3ap1
ENSMUSG00000025019.18	288.266754	-0.0069804	0.00450597	2.34234435	0.12589995	0.25145875	Lcor
ENSMUSG00000025020.12	7.52933264	0.03293973	0.01076378	9.05858046	0.00261465	0.01016151	Slit1
ENSMUSG00000025024.8	419.487956	-0.0023035	0.00326084	0.49414939	0.48208217	0.67141365	Smndc1
ENSMUSG00000025025.15	468.499478	0.00111218	0.00213027	0.27143253	0.60237244	0.77583568	Mxi1
ENSMUSG00000025026.16	307.107458	0.00084985	0.00285239	0.08754719	0.76731887	0.89505355	Add3
ENSMUSG00000025027.19	460.853947	-0.0071535	0.00201165	12.7092564	0.00036385	0.00180479	Xpnpep1
ENSMUSG00000025034.10	717.694411	0.00067834	0.0034954	0.03658884	0.84830458	0.94623371	Trim8
ENSMUSG00000025035.10	201.08168	-0.0053191	0.00318492	2.79304597	0.09467412	0.20149645	Arl3
ENSMUSG00000025036.8	863.475619	-0.0012126	0.00403125	0.09236562	0.76119103	0.89125555	Sfxn2
ENSMUSG00000025037.7	225.941791	0.0100865	0.00220552	20.6909448	5.40E-06	4.06E-05	Maoa
ENSMUSG00000025040.14	236.267581	0.01184099	0.00321206	13.3100731	0.00026398	0.00136303	Fundc1
ENSMUSG00000025041.18	387.325573	0.01762094	0.00295776	35.2827036	2.85E-09	3.73E-08	Nt5c2
ENSMUSG00000025044.17	488.944832	0.04738433	0.00636891	49.8431719	1.67E-12	3.39E-11	Msr1
ENSMUSG00000025047.10	414.033298	-0.006948	0.0023212	8.97330709	0.00273952	0.01057346	Pdcd11
ENSMUSG00000025049.7	76.9412443	-0.0026931	0.00252194	1.12662537	0.2884963	0.46963032	Taf5
ENSMUSG00000025050.9	176.547483	-0.0106958	0.00269917	15.6850312	7.48E-05	0.00044101	Pcgf6
ENSMUSG00000025058.5	15.1611652	0.0733812	0.0076223	83.5499353	6.21E-20	3.23E-18	Tasl
ENSMUSG00000025059.17	2129.16572	0.0011785	0.00308792	0.14166896	0.70662745	0.85451121	Gk
ENSMUSG00000025060.16	806.782233	8.40E-05	0.00339194	0.00058774	0.98065852	1	Slk
ENSMUSG00000025064.16	3.1329841	0.00912339	0.01032998	0.92543596	0.3360517	0.52334866	Col17a1
ENSMUSG00000025066.11	820.623046	-0.0033934	0.00255726	1.76319079	0.18422702	0.3365225	Sfr1
ENSMUSG00000025068.9	1301.01436	-0.015541	0.00354599	19.638917	9.35E-06	6.67E-05	Gsto1
ENSMUSG00000025069.16	3.8103459	-0.0189549	0.00915532	3.92729729	0.04750824	0.11574012	Gsto2
ENSMUSG00000025075.15	2657.88648	-0.0017335	0.00372256	0.21672735	0.64154491	0.80777197	Habp2
ENSMUSG00000025076.13	402.66178	0.00536052	0.00256094	4.39272906	0.0360925	0.0925503	Casp7
ENSMUSG00000025077.15	326.067497	0.02041358	0.00400388	25.6262579	4.14E-07	3.84E-06	Dclre1a
ENSMUSG00000025078.10	614.695514	0.01063712	0.0025987	16.4689407	4.95E-05	0.0003036	Nhlrc2
ENSMUSG00000025081.14	0.93751221	-0.0162555	0.03754952	0.05755738	0.81039911	0.92209336	Tdrd1
ENSMUSG00000025083.19	29.8457296	0.01172376	0.00608807	3.70186345	0.05435173	0.12927995	Afap1l2
ENSMUSG00000025085.17	677.861958	0.00166373	0.00328512	0.24830721	0.61826956	0.78926747	Ablim1
ENSMUSG00000025086.14	242.838983	-0.0108586	0.00247075	19.5770164	9.66E-06	6.87E-05	Trub1
ENSMUSG00000025089.16	3229.28266	-0.0029732	0.00412594	0.50632111	0.47673577	0.66692111	Gfra1
ENSMUSG00000025092.8	50.1196472	0.01466575	0.00486647	8.98892798	0.0027162	0.01049891	Hspa12a
ENSMUSG00000025094.9	23.2059339	-0.0153416	0.00622193	6.25827947	0.01236142	0.03807213	Slc18a2
ENSMUSG00000025102.15	141.174724	-0.0090653	0.00336935	7.27132444	0.00700641	0.02364476	3110040N11Rik
ENSMUSG00000025103.9	914.67071	-0.0013218	0.00246486	0.27931858	0.59714819	0.7719137	Btbd1
ENSMUSG00000025104.14	68.7163652	0.02787319	0.0044935	37.5354653	8.98E-10	1.25E-08	Hdgfl3
ENSMUSG00000025105.11	5.65097997	0.00034947	0.00851327	0.00159401	0.96815285	1	Bnc1
ENSMUSG00000025127.16	3641.29267	-0.0191073	0.00415734	21.2803093	3.97E-06	3.07E-05	Gcgr
ENSMUSG00000025128.8	4.36490469	0.06851898	0.01182148	30.3812948	3.55E-08	3.94E-07	Bhlhe22
ENSMUSG00000025130.12	26319.2228	-0.0005888	0.00297442	0.03876086	0.84392319	0.94322003	P4hb
ENSMUSG00000025132.14	1422.36479	0.00908805	0.00348707	6.83031184	0.00896236	0.02910454	Arhgdia
ENSMUSG00000025133.10	373.814414	-0.0036348	0.00253817	2.08338468	0.14890967	0.28593856	Ints4
ENSMUSG00000025134.3	281.995328	0.00679575	0.00430955	2.46946827	0.11607719	0.23589493	Alyref
ENSMUSG00000025135.13	339.321588	0.00276021	0.00330051	0.69445842	0.40465203	0.59624682	Anapc11
ENSMUSG00000025137.16	7891.52621	-0.0071658	0.00378693	3.53328899	0.06014836	0.14022529	Pcyt2

ENSMUSG00000025138.15	752.422156	-0.007015	0.00268897	6.84736016	0.00887723	0.02885988	Sirt7
ENSMUSG00000025139.15	1191.25001	0.00705345	0.00183203	14.7497668	0.00012276	0.00068793	Tollip
ENSMUSG00000025140.16	10.3087318	0.00198161	0.01057798	0.03423687	0.85320387	0.94945348	Pycr1
ENSMUSG00000025141.4	12.8662796	-0.0137662	0.0291491	0.10346923	0.74770549	0.88175152	Myadml2
ENSMUSG00000025142.19	1135.88376	-0.0046971	0.00337429	1.94574484	0.16304612	0.30593523	Aspscr1
ENSMUSG00000025144.18	418.453017	-0.0027207	0.00359378	0.57880114	0.44678262	0.63861674	Cenpx
ENSMUSG00000025145.14	147.795515	0.00149712	0.00344319	0.19266232	0.66070997	0.82159984	Lrrc45
ENSMUSG00000025147.8	339.870898	-0.0095153	0.00225296	17.8757836	2.36E-05	0.00015514	Mob2
ENSMUSG00000025150.11	8.97467634	0.00202197	0.01792104	0.12736857	0.72117615	0.86401529	Cbr2
ENSMUSG00000025151.17	1674.33744	-0.0039384	0.00206926	3.57143212	0.0587816	0.13756166	Maged1
ENSMUSG00000025153.10	18231.3859	0.01260209	0.00625222	3.95193811	0.04681742	0.1144167	Fasn
ENSMUSG00000025154.16	113.913373	0.04621639	0.00506348	78.7891366	6.91E-19	3.13E-17	Arhgap19
ENSMUSG00000025155.16	1067.31393	-0.0107587	0.00272564	15.8924801	6.70E-05	0.00039942	Dus1l
ENSMUSG00000025156.18	906.886226	-0.0069661	0.00169267	16.8972883	3.95E-05	0.00024804	Gps1
ENSMUSG00000025157.9	262.356371	-0.0057896	0.0037049	2.47806818	0.11544397	0.23487117	Zdhhc16
ENSMUSG00000025158.8	226.674854	-0.0065033	0.00258813	6.34687773	0.01175881	0.03647813	Rfng
ENSMUSG00000025159.9	459.35146	-0.002425	0.00393563	0.38112923	0.53699951	0.72132072	Mms19
ENSMUSG00000025161.17	16.2714812	0.07413997	0.00853531	63.1093902	1.96E-15	5.82E-14	Slc16a3
ENSMUSG00000025162.19	1157.97706	0.00102045	0.00314788	0.10548923	0.74533922	0.88007864	Csnk1d
ENSMUSG00000025163.7	14.2069895	0.0802934	0.00673222	129.586611	5.05E-30	1.04E-27	Cd7
ENSMUSG00000025165.13	16.6332279	0.04330709	0.00675616	40.350878	2.12E-10	3.25E-09	Sectm1a
ENSMUSG00000025169.7	104.84945	-0.0073669	0.00235098	10.0415879	0.00153045	0.0063855	Ogfod3
ENSMUSG00000025170.14	11.8258282	0.00047131	0.00659504	0.00480168	0.94475545	0.9994266	Rab40b
ENSMUSG00000025171.3	137.320952	-0.005209	0.00293919	3.09959402	0.07831182	0.17325416	Ubtd1
ENSMUSG00000025172.4	8.72274193	0.01847097	0.00713514	6.25572646	0.01237925	0.03811709	Ankrd2
ENSMUSG00000025173.14	738.75078	-0.0108488	0.00307371	12.4139023	0.00042615	0.00207497	Wdr45b
ENSMUSG00000025175.13	234.350765	-0.0179637	0.00401607	20.2349607	6.85E-06	5.04E-05	Fn3k
ENSMUSG00000025176.15	2221.5367	0.00151612	0.00374777	0.16708341	0.68271699	0.83767169	Hoga1
ENSMUSG00000025178.10	1137.29784	-0.0040779	0.00255653	2.48601015	0.11486258	0.23386989	Pi4k2a
ENSMUSG00000025184.11	271.005449	0.00091057	0.0028452	0.10007703	0.75173722	0.88473573	R3hcc1l
ENSMUSG00000025185.15	76.2959901	-0.0055387	0.008791	0.37998118	0.53761331	0.72180974	Loxl4
ENSMUSG00000025188.16	210.177533	0.00545694	0.00376888	2.07678336	0.14955505	0.28692088	Hps1
ENSMUSG00000025189.10	14.6429264	0.05244411	0.00996033	21.717025	3.16E-06	2.49E-05	Cnnm1
ENSMUSG00000025190.14	4404.28319	-0.0055404	0.00454797	1.49462766	0.22149984	0.386634	Got1
ENSMUSG00000025192.14	71.5620529	0.01392773	0.00464564	8.91646825	0.00282611	0.01086496	Entpd7
ENSMUSG00000025193.15	246.782096	-0.0142365	0.00301448	22.3431676	2.28E-06	1.84E-05	Cutc
ENSMUSG00000025194.6	8845.46018	0.00282793	0.00509334	0.31136105	0.57684619	0.75527335	Abcc2
ENSMUSG00000025195.10	628.643973	-0.009185	0.00364994	6.09147622	0.01358355	0.04122256	Dnmbp
ENSMUSG00000025196.5	4214.26378	-0.0024149	0.00401122	0.35970096	0.54867236	0.7313771	Cpn1
ENSMUSG00000025197.10	4842.00937	-0.0098758	0.00535451	3.31313573	0.06872823	0.15591345	Cyp2c23
ENSMUSG00000025198.17	1102.75304	0.00358913	0.00309285	1.28940995	0.25615672	0.42919034	Erlin1
ENSMUSG00000025199.17	2517.0995	-0.0099196	0.00354376	7.73311262	0.00542172	0.01895031	Chuk
ENSMUSG00000025200.8	278.367761	0.01317826	0.00232874	31.4278932	2.07E-08	2.39E-07	Cwf19l1
ENSMUSG00000025202.9	6.09497826	-0.0047447	0.01123498	0.19023844	0.66271824	0.82289764	Scd3
ENSMUSG00000025203.7	563.973625	0.07782785	0.007406	103.905267	2.12E-24	1.87E-22	Scd2
ENSMUSG00000025204.11	3220.6259	-0.0199766	0.00444774	20.5309325	5.87E-06	4.38E-05	Ndufb8
ENSMUSG00000025207.17	4898.27995	-0.0120487	0.00536819	4.96047649	0.02593306	0.0705894	Sema4g
ENSMUSG00000025208.10	554.214466	-0.008907	0.00243135	13.5307902	0.00023468	0.00122813	Mrpl43
ENSMUSG00000025209.6	460.532777	-0.0071214	0.00219788	10.4094848	0.0012537	0.00536266	Twnk
ENSMUSG00000025212.18	59.4931048	0.03981997	0.00569335	47.5579266	5.34E-12	1.02E-10	Sfxn3
ENSMUSG00000025213.12	17.7400978	0.00067868	0.00478401	0.01952532	0.88887093	0.96943077	Kazald1
ENSMUSG00000025215.11	4.58426207	-0.0346379	0.02821469	2.76058906	0.09661266	0.20467796	Tlx1
ENSMUSG00000025217.16	248.215497	-0.0029655	0.00207447	2.06878721	0.15034103	0.28792701	Btrc
ENSMUSG00000025218.7	126.166989	-0.0069121	0.00269982	6.55950975	0.01043251	0.03304252	Poll
ENSMUSG00000025220.9	1251.22948	-0.0027222	0.00219541	1.48113291	0.22359735	0.38894957	Oga
ENSMUSG00000025221.17	15.3207838	0.01219253	0.01424007	0.87289268	0.35015573	0.53851762	Kcnip2
ENSMUSG00000025223.16	502.06966	0.00299455	0.00304339	0.9832653	0.32139398	0.5076613	Ldb1
ENSMUSG00000025224.17	1063.10272	-0.0142441	0.00335557	17.7264433	2.55E-05	0.00016641	Gbf1
ENSMUSG00000025225.16	162.716355	0.02011412	0.00451818	18.2692476	1.92E-05	0.00012872	Nfkb2
ENSMUSG00000025226.12	117.362229	-0.0096478	0.00430704	4.98530009	0.02556355	0.06978494	Fbxl15
ENSMUSG00000025227.15	79.3241354	-0.0024084	0.00383689	0.38311864	0.5359389	0.72027161	Mfsd13a

ENSMUSG00000025228.6	963.867833	-0.0054514	0.00169683	10.2608208	0.00135885	0.00574165	Actr1a
ENSMUSG00000025229.16	11.5291572	-0.0805249	0.01355062	33.1538292	8.51E-09	1.04E-07	Pitx3
ENSMUSG00000025231.18	93.2176657	0.00182093	0.00372656	0.23716268	0.62626293	0.79505422	Sufu
ENSMUSG00000025232.9	986.749333	0.02196054	0.00310511	48.1690995	3.91E-12	7.61E-11	Hexa
ENSMUSG00000025234.12	1215.16728	-0.0067633	0.00371033	3.22628723	0.0724648	0.16280766	Arih1
ENSMUSG00000025235.9	63.3487662	-0.0018316	0.00337602	0.29172377	0.58911801	0.76574976	Bbs4
ENSMUSG00000025236.12	366.039076	0.00574063	0.00249277	5.2740792	0.02164523	0.06073334	Adpgk
ENSMUSG00000025237.17	233.105327	-0.0003357	0.00280217	0.01444814	0.90432439	0.97791917	Parp6
ENSMUSG00000025239.4	827.239219	-0.0046827	0.00229523	4.16058169	0.04137519	0.10344113	Limd1
ENSMUSG00000025240.11	1322.86578	-0.0045801	0.00242627	3.54655307	0.05966928	0.13923226	Sacm1l
ENSMUSG00000025241.17	935.853206	-0.0058236	0.00369109	2.49337742	0.11432615	0.23309963	Fyco1
ENSMUSG00000025243.18	98.2932411	-0.0053409	0.0104556	0.23093456	0.63083173	0.79920488	Slc6a20b
ENSMUSG00000025245.15	285.756002	-0.0140182	0.00358375	14.7835989	0.00012058	0.0006773	Lztfl1
ENSMUSG00000025246.14	1013.57832	-0.0010298	0.0034056	0.09148607	0.76229636	0.89179713	Tbl1x
ENSMUSG00000025255.19	432.795375	0.00221773	0.00540748	0.16678629	0.68298387	0.83791494	Zfhx4
ENSMUSG00000025257.6	26.7602334	-0.0033343	0.00503588	0.42266586	0.51560982	0.7021868	Ribc1
ENSMUSG00000025260.11	2584.7811	-0.0128511	0.00299068	18.0731675	2.13E-05	0.00014131	Hsd17b10
ENSMUSG00000025261.19	3762.63574	-0.0061211	0.00500804	1.44179394	0.22984926	0.39710664	Huwe1
ENSMUSG00000025262.9	76.9625311	-0.0027746	0.0043382	0.40246541	0.52581875	0.71120319	Fam120c
ENSMUSG00000025264.13	210.399365	0.00055986	0.00260821	0.04659683	0.82909454	0.93358231	Tsr2
ENSMUSG00000025265.15	45.0553649	0.00740194	0.00640814	1.38238124	0.23969595	0.40928609	Fgd1
ENSMUSG00000025266.12	363.52683	0.00896578	0.00291182	9.27196783	0.00232688	0.00919445	Gnl3l
ENSMUSG00000025268.16	112.044991	0.00884543	0.00395979	5.10565446	0.02384798	0.06583315	Maged2
ENSMUSG00000025269.17	133.291456	-0.0101497	0.00419413	5.89970184	0.01514345	0.04521182	Apex2
ENSMUSG00000025270.14	1614.19354	-0.0344063	0.00424922	61.2170844	5.11E-15	1.45E-13	Alas2
ENSMUSG00000025271.14	612.11757	-0.0200211	0.00379299	27.4844928	1.58E-07	1.59E-06	Pfkfb1
ENSMUSG00000025272.17	18.5562191	0.03055839	0.01138918	6.650468	0.00991298	0.03167265	Tro
ENSMUSG00000025277.15	561.17621	-0.0241514	0.00360943	44.5493982	2.48E-11	4.32E-10	Abhd6
ENSMUSG00000025278.10	1403.1944	-0.002624	0.00286508	0.83772937	0.36004698	0.54935386	Flnb
ENSMUSG00000025279.8	1092.5479	-0.0051108	0.00417489	1.50188281	0.22038189	0.38512656	Dnase1l3
ENSMUSG00000025280.9	263.6415	-0.0033485	0.0028249	1.42770039	0.2321401	0.39994569	Polr3a
ENSMUSG00000025283.16	1905.37633	0.0270134	0.00375724	49.3797323	2.11E-12	4.25E-11	Sat1
ENSMUSG00000025287.16	111.659523	0.03626802	0.00457833	60.5195556	7.29E-15	2.05E-13	Acot9
ENSMUSG00000025289.16	1453.43934	-0.0132652	0.00303067	19.562813	9.73E-06	6.91E-05	Prdx4
ENSMUSG00000025290.18	13448.68	-0.0042317	0.00444536	0.89952186	0.34290995	0.53090942	Rps24
ENSMUSG00000025314.18	1354.75892	0.01720968	0.00320885	27.9593361	1.24E-07	1.27E-06	Ptprj
ENSMUSG00000025316.17	156.817756	-0.0057167	0.00414203	1.88219144	0.17008529	0.31629989	Banp
ENSMUSG00000025317.8	973.989005	-0.0155632	0.00510471	9.25444396	0.00234925	0.00927198	Car5a
ENSMUSG00000025318.14	6.35170648	0.02031134	0.0189225	1.25783676	0.26206096	0.43657083	Jph3
ENSMUSG00000025321.15	37.3701053	0.03680328	0.0065348	30.3599094	3.59E-08	3.98E-07	Itgb8
ENSMUSG00000025323.11	117.358459	0.01146206	0.00404124	7.77295968	0.00530341	0.01860018	Sp4
ENSMUSG00000025324.9	12.0174006	0.02382946	0.00714965	10.7943793	0.00101809	0.00446248	Atp10a
ENSMUSG00000025326.13	1693.37255	-0.0025198	0.00359518	0.48292474	0.48710051	0.67565	Ube3a
ENSMUSG00000025330.7	4.07347192	0.0506537	0.01108972	16.6844454	4.41E-05	0.00027434	Padi4
ENSMUSG00000025332.15	926.230944	-0.0117431	0.00312186	13.9196985	0.00019079	0.00102318	Kdm5c
ENSMUSG00000025337.11	855.366195	0.0038013	0.002189	3.00248159	0.08313708	0.1816126	Sbds
ENSMUSG00000025340.15	262.397362	-0.0081298	0.0023933	11.5494808	0.00067768	0.00312733	Rabgef1
ENSMUSG00000025347.5	9434.92432	-0.003441	0.00471695	0.5250431	0.46869833	0.65982183	Tmt1b
ENSMUSG00000025348.11	147.049987	-0.0037262	0.00474307	0.62911013	0.42768191	0.61896702	Itga7
ENSMUSG00000025350.16	352.05252	0.00227967	0.00307698	0.55795267	0.45508632	0.64689304	Rdh5
ENSMUSG00000025351.15	324.448351	0.06204032	0.0071854	69.6482121	7.09E-17	2.51E-15	Cd63
ENSMUSG00000025352.7	11.6009913	0.0446715	0.00955271	20.6229218	5.59E-06	4.19E-05	Gdf11
ENSMUSG00000025353.6	693.418491	-0.0111204	0.00261233	17.8650579	2.37E-05	0.00015592	Ormdl2
ENSMUSG00000025354.6	769.096242	0.00063114	0.0015098	0.17377281	0.67678033	0.8328736	Dnajc14
ENSMUSG00000025355.8	1039.45929	-0.000527	0.00319664	0.02694326	0.86961776	0.95837696	Mmp19
ENSMUSG00000025357.9	120.889466	-0.0148224	0.00324537	21.5917643	3.37E-06	2.65E-05	Dgka
ENSMUSG00000025358.17	237.048313	-0.0004188	0.00303248	0.0191331	0.88998557	0.96992883	Cdk2
ENSMUSG00000025359.11	6.5438979	-0.0144218	0.00651654	4.65460291	0.0309704	0.081653	Pmel
ENSMUSG00000025362.7	2359.79846	-0.0015987	0.00326102	0.24104613	0.62345156	0.79279322	Rps26
ENSMUSG00000025364.14	1291.51718	-0.0057296	0.00195573	8.62425094	0.00331717	0.01245443	Pa2g4
ENSMUSG00000025366.9	323.125875	0.01281602	0.002784	20.9377826	4.74E-06	3.61E-05	Esyt1

ENSMUSG00000025369.16	1740.7881	-0.0040699	0.00322475	1.56367518	0.21112791	0.37323212	Smarcc2
ENSMUSG00000025371.13	269.281143	0.00052896	0.00292807	0.03235963	0.85724063	0.95170052	Chmp6
ENSMUSG00000025372.17	596.810689	0.0003428	0.004285	0.00646488	0.93591557	0.99479683	Baiap2
ENSMUSG00000025373.15	163.036868	0.00639222	0.00228144	7.81553547	0.0051799	0.01823752	Rnf41
ENSMUSG00000025374.14	548.570881	0.00059901	0.0029105	0.04300637	0.83571328	0.93793868	Nabp2
ENSMUSG00000025375.16	162.010949	0.00862176	0.00612295	1.596796	0.20635786	0.36702786	Aatk
ENSMUSG00000025377.15	157.110867	-0.0103268	0.00270429	14.6734129	0.00012784	0.0007126	Tepsin
ENSMUSG00000025381.4	1495.30151	-0.0066122	0.00261978	6.42064033	0.01128015	0.03531841	Cnpy2
ENSMUSG00000025383.3	2.79035544	0.00146925	0.01039375	0.02885411	0.86511632	0.95595799	Il23a
ENSMUSG00000025384.16	113.043075	-0.0105247	0.00407501	6.73987053	0.00942815	0.03040176	Faap100
ENSMUSG00000025386.3	7.49479047	-0.0351618	0.00738895	22.7796304	1.82E-06	1.50E-05	Pde6g
ENSMUSG00000025393.13	21828.6673	-0.0183042	0.00387056	22.3778972	2.24E-06	1.81E-05	Atp5f1b
ENSMUSG00000025395.16	78.7235556	0.00617421	0.00481862	1.61576019	0.20368398	0.36342313	Prim1
ENSMUSG00000025396.8	2311.39368	-0.0155968	0.00647736	6.09229524	0.01357726	0.04121406	Hsd17b6
ENSMUSG00000025401.10	1.8181679	0.00569243	0.0142296	0.14584043	0.70254251	0.85163014	Myo1a
ENSMUSG00000025402.13	291.855647	0.01402495	0.00418867	10.6745884	0.00108617	0.00472404	Nab2
ENSMUSG00000025403.6	3103.74331	-0.0075446	0.00280507	7.2048679	0.00727061	0.02439673	Shmt2
ENSMUSG00000025404.16	1690.04986	-0.0073818	0.00398783	3.36787429	0.06647948	0.1519071	R3hdm2
ENSMUSG00000025405.10	896.267709	-0.0131098	0.00452797	8.39301131	0.00376666	0.01387483	Inhbc
ENSMUSG00000025407.8	3.05205791	6.03E-05	0.01271934	0.00027998	0.98664993	1	Gli1
ENSMUSG00000025408.16	136.059497	0.01750168	0.00552544	9.81316241	0.00173267	0.00711594	Ddit3
ENSMUSG00000025409.15	442.326966	-0.0011292	0.00503597	0.0493056	0.82427602	0.93023354	Mbd6
ENSMUSG00000025410.11	1674.6209	-0.0060089	0.00199032	9.14668973	0.00249168	0.00973801	Dctn2
ENSMUSG00000025413.14	369.509589	-0.009639	0.0021912	19.4026944	1.06E-05	7.48E-05	Ttc4
ENSMUSG00000025417.9	421.928716	0.00247486	0.00242782	1.0473242	0.30612415	0.49107286	Pip4k2c
ENSMUSG00000025421.16	574.317718	-0.0133919	0.00196301	46.9032433	7.46E-12	1.40E-10	Hdhd2
ENSMUSG00000025422.11	56.9520833	0.01889491	0.00640878	8.24853257	0.0040785	0.01486332	Agap2
ENSMUSG00000025423.17	467.23259	-0.0019801	0.00180939	1.20186885	0.27294848	0.45047672	Pias2
ENSMUSG00000025427.16	9.98885395	0.0242548	0.00901359	6.84008089	0.00891348	0.02896574	Ark2c
ENSMUSG00000025428.16	15912.0253	-0.0164629	0.00300301	30.2148128	3.87E-08	4.27E-07	Atp5f1a
ENSMUSG00000025429.10	514.89693	0.01200865	0.00458315	6.62220568	0.0100715	0.03208112	Pstpip2
ENSMUSG00000025432.12	17.3305865	0.00801971	0.01574969	0.40916518	0.52239353	0.70813776	Avil
ENSMUSG00000025436.13	79.6827907	-0.0161643	0.00353938	21.1520108	4.24E-06	3.26E-05	Atp23
ENSMUSG00000025437.16	725.800685	-0.00176	0.00227925	0.59321777	0.44117752	0.63308268	Usp33
ENSMUSG00000025439.6	572.860793	-0.0016071	0.00232105	0.48070122	0.48810486	0.67640666	Clns1a
ENSMUSG00000025451.16	1029.00807	-0.0082573	0.00238825	11.8932744	0.00056337	0.0026557	Paip1
ENSMUSG00000025453.19	558.40549	-0.0126965	0.00432063	8.90516802	0.00284365	0.01091816	Nnt
ENSMUSG00000025461.12	1.42831439	0.06738843	0.01753504	15.9821732	6.39E-05	0.00038336	Scart1
ENSMUSG00000025464.16	1137.60149	-0.0012238	0.0040764	0.0886089	0.76595315	0.89416804	Paox
ENSMUSG00000025465.14	4972.95115	-0.0043167	0.00257829	2.83358297	0.0923122	0.19741368	Echs1
ENSMUSG00000025466.20	378.279609	-0.0042933	0.00373412	1.37187066	0.24149074	0.41149919	Fuom
ENSMUSG00000025470.12	113.520642	-0.0087633	0.00292572	9.03129335	0.00265397	0.01029039	Zfp511
ENSMUSG00000025473.17	60.3620086	0.05654215	0.00926146	33.3523332	7.69E-09	9.44E-08	Adam8
ENSMUSG00000025474.10	204.067485	0.00721668	0.00280455	6.6315083	0.01001904	0.03194661	Tubgcp2
ENSMUSG00000025477.15	352.67915	-0.0062973	0.00366776	2.92257605	0.08734831	0.18904362	Inpp5a
ENSMUSG00000025478.16	5.0594042	-0.0088606	0.00891072	1.04464551	0.30674351	0.49179881	Dpysl4
ENSMUSG00000025479.10	88293.0466	-0.0022661	0.00555491	0.16286814	0.6865295	0.84046947	Cyp2e1
ENSMUSG00000025480.6	2.73724971	-0.0093345	0.01805976	0.17343754	0.67707465	0.83308898	Syce1
ENSMUSG00000025481.13	3267.97217	-0.0111573	0.00437173	6.53312241	0.01058839	0.03349039	Urah
ENSMUSG00000025484.10	454.297511	-0.0108793	0.00361926	9.08942002	0.00257092	0.01001285	Bet1l
ENSMUSG00000025485.14	420.901057	0.00171616	0.00229146	0.56779752	0.45113545	0.64285167	Ric8a
ENSMUSG00000025486.18	1450.53836	-0.0093153	0.00273862	11.5936911	0.00066176	0.00306044	Sirt3
ENSMUSG00000025487.16	1282.68177	-0.0024515	0.00441963	0.31084136	0.57716435	0.75560095	Psmd13
ENSMUSG00000025488.10	58.9929084	0.04012717	0.01796547	1.33628254	0.24769062	0.41893373	Cox8b
ENSMUSG00000025489.6	2.46587619	0.00448506	0.01009308	0.20599082	0.64992807	0.81326349	Ifitm5
ENSMUSG00000025491.15	134.179536	0.03021483	0.00375422	63.3099611	1.77E-15	5.28E-14	Ifitm1
ENSMUSG00000025492.7	5531.23745	0.00766857	0.00350434	4.96538506	0.02585956	0.07044624	Ifitm3
ENSMUSG00000025494.9	260.06104	-0.0005502	0.00269368	0.04284603	0.83601546	0.9380193	Sigirr
ENSMUSG00000025495.16	209.687037	-0.013679	0.00352121	15.2449496	9.44E-05	0.00054269	Ptdss2
ENSMUSG00000025497.16	1286.95791	0.01268939	0.00379106	10.9087787	0.0009571	0.00422419	Cdhr5
ENSMUSG00000025498.16	936.499541	0.04527592	0.00501714	80.5272717	2.87E-19	1.35E-17	Irf7

ENSMUSG00000025499.19	819.181897	-0.0100066	0.00363694	7.58656116	0.0058805	0.02031603	Hras
ENSMUSG00000025500.13	20.7101393	-0.0194228	0.00521481	14.2497768	0.00016008	0.00087419	Lmntd2
ENSMUSG00000025503.6	1707.18622	-0.0017422	0.00265782	0.43005954	0.51195973	0.69861389	Taldo1
ENSMUSG00000025504.15	1163.51506	-0.0129896	0.00454966	7.90333874	0.00493436	0.01748264	Eps8l2
ENSMUSG00000025505.17	136.875966	-0.0025138	0.00288408	0.75135037	0.38604903	0.57764083	Tmem80
ENSMUSG00000025507.14	41.3823241	-0.0025214	0.00428228	0.34592981	0.55642626	0.73858235	Pidd1
ENSMUSG00000025508.14	3067.31502	0.00296495	0.00533466	0.30932706	0.57809342	0.75627664	Rplp2
ENSMUSG00000025509.16	1192.32222	-0.0078185	0.00372496	4.41624608	0.03559827	0.09149945	Pnpla2
ENSMUSG00000025510.15	638.592157	-0.0021957	0.00339669	0.40853561	0.52271371	0.7083846	Cd151
ENSMUSG00000025511.16	836.456783	0.00948551	0.00422389	4.91088914	0.0266879	0.07228535	Tspan4
ENSMUSG00000025512.16	630.417602	-0.0246579	0.00286552	73.7206224	9.00E-18	3.56E-16	Chid1
ENSMUSG00000025515.17	8.97598923	-0.0091265	0.00956949	0.83750784	0.3601105	0.54935427	Muc2
ENSMUSG00000025519.6	1.33041832	-0.0043115	0.02785801	-0.0616405	1	1	Tktl2
ENSMUSG00000025521.16	205.060567	0.00653329	0.0030806	4.49982522	0.03389832	0.08799535	Tmem192
ENSMUSG00000025525.13	405.393121	-0.0080236	0.0019857	16.4007871	5.13E-05	0.00031335	Apool
ENSMUSG00000025528.11	1.8468344	-0.0626544	0.01831907	12.4165255	0.00042555	0.00207249	2010106E10Rik
ENSMUSG00000025529.15	1.67549876	0.00523456	0.0248767	0.02998012	0.86253535	0.95447869	Zfp711
ENSMUSG00000025531.15	371.185141	0.00715804	0.00308299	5.31260435	0.02117168	0.05966727	Chm
ENSMUSG00000025532.14	421.592291	-0.0027546	0.00259851	1.14654036	0.28427455	0.4643924	Crcp
ENSMUSG00000025533.16	8336.68825	0.00010664	0.00460562	0.00055744	0.98116349	1	Asl
ENSMUSG00000025534.18	534.322283	0.00579152	0.00254751	5.17304227	0.02294001	0.06378956	Gusb
ENSMUSG00000025537.13	9.6479204	0.03480184	0.01179869	7.26650132	0.00702525	0.02368462	Phkg1
ENSMUSG00000025538.16	136.900189	-0.004023	0.00388688	1.09560139	0.29523344	0.47805685	Sumf2
ENSMUSG00000025544.14	3208.49234	-0.0018898	0.00222001	0.71228202	0.39868728	0.59040862	Tm9sf2
ENSMUSG00000025545.11	682.806727	-0.0075104	0.00296316	6.4101214	0.01134717	0.03548114	Clybl
ENSMUSG00000025551.14	3.9660202	0.01248931	0.02033993	0.39602636	0.52914858	0.71370151	Fgf14
ENSMUSG00000025555.15	359.789095	-0.0058689	0.00356484	2.66273218	0.10272415	0.21444961	Farp1
ENSMUSG00000025557.11	1.43602682	0.03273604	0.0171883	3.82546776	0.05047926	0.12149018	Slc15a1
ENSMUSG00000025558.20	292.778076	0.00473222	0.00251796	3.5493987	0.05956703	0.13903494	Dock9
ENSMUSG00000025571.14	509.924354	-0.0002806	0.00359369	0.006011	0.93820144	0.99602975	Tnrc6c
ENSMUSG00000025572.19	147.562118	0.01870169	0.00457681	16.2108432	5.67E-05	0.00034351	Tmc6
ENSMUSG00000025574.14	408.975993	-0.0075173	0.00449959	2.7675541	0.09619304	0.20399291	Tk1
ENSMUSG00000025575.15	545.957178	3.15E-05	0.00161456	0.00038232	0.98439992	1	Cant1
ENSMUSG00000025576.18	45.3033795	0.01705949	0.00719919	5.3264067	0.02100465	0.05926024	Rbfox3
ENSMUSG00000025577.8	137.727836	-0.0045022	0.00366596	1.49964929	0.22072533	0.38563168	Cbx2
ENSMUSG00000025578.10	117.134939	-0.0041277	0.0040547	1.0280305	0.3106217	0.49579233	Cbx8
ENSMUSG00000025579.15	1567.83269	-0.0028601	0.00329208	0.73565873	0.3910551	0.5823605	Gaa
ENSMUSG00000025580.16	614.719924	-0.0041151	0.00295318	1.95726614	0.161806	0.30411874	Eif4a3
ENSMUSG00000025582.5	21.850088	0.06777217	0.01114292	31.9012937	1.62E-08	1.90E-07	Nptx1
ENSMUSG00000025583.17	589.55812	-0.0050721	0.00255645	3.88464728	0.0487295	0.11809843	Rptor
ENSMUSG00000025584.18	700.914019	0.00078405	0.00238265	0.10729988	0.74323938	0.87866779	Pde8a
ENSMUSG00000025586.18	86.7397238	0.02348824	0.00526672	18.8374224	1.42E-05	9.81E-05	Cpeb1
ENSMUSG00000025588.5	46.1111191	0.014243	0.00425828	10.6907759	0.00107671	0.00468634	Nat1
ENSMUSG00000025591.7	116.67209	0.00444552	0.00260162	2.87996717	0.08968785	0.19304414	Tma16
ENSMUSG00000025592.18	1.13909544	0.00953389	0.02484276	0.37676412	0.53934013	0.72318797	Dach2
ENSMUSG00000025597.14	27.134477	0.00743853	0.00518866	2.00345911	0.1569407	0.29727824	Klhl4
ENSMUSG00000025602.14	20.7725312	0.04030745	0.00461963	73.2791698	1.13E-17	4.41E-16	Zfp202
ENSMUSG00000025607.16	560.483055	-0.0028989	0.00486162	0.34343341	0.55785407	0.73968664	Copg2
ENSMUSG00000025608.10	189.6023	0.00032006	0.0041559	0.00595362	0.93849651	0.99608629	Podxl
ENSMUSG00000025609.16	1290.69542	-0.0018826	0.00166361	1.27871068	0.2581389	0.43160889	Mkln1
ENSMUSG00000025610.8	7.29271189	-0.0178082	0.01605731	1.49498872	0.22144404	0.38660157	Map3k7cl
ENSMUSG00000025612.6	703.287946	-0.0068979	0.00324186	4.41317794	0.03566235	0.09162659	Bach1
ENSMUSG00000025613.14	2397.4927	-0.0050738	0.00157005	10.3782233	0.0012751	0.00543943	Cct8
ENSMUSG00000025616.15	852.904638	-0.0101888	0.00346448	8.52574145	0.00350158	0.01304506	Usp16
ENSMUSG00000025626.17	198.209811	0.00766088	0.00313389	5.76995569	0.01630245	0.04805768	Phf6
ENSMUSG00000025630.9	1556.55562	-0.0006395	0.00265008	0.05719421	0.81098686	0.92218936	Hprt
ENSMUSG00000025645.9	126.740526	-0.0091219	0.00279568	10.7401599	0.00104835	0.00457812	Ccdc51
ENSMUSG00000025646.10	164.559398	-0.0019171	0.00271121	0.4959571	0.48128195	0.67089338	Atrip
ENSMUSG00000025647.17	698.597397	0.03132043	0.00348535	78.85113	6.70E-19	3.04E-17	Shisa5
ENSMUSG00000025648.19	31.8002223	0.04587248	0.00582483	57.1805567	3.98E-14	1.01E-12	Pfkfb4
ENSMUSG00000025650.14	31.7563092	-0.0526808	0.01198615	17.8492581	2.39E-05	0.00015701	Col7a1

ENSMUSG00000025651.15	4059.54032	-0.0161191	0.00282767	33.1303434	8.62E-09	1.05E-07	Uqcrc1
ENSMUSG00000025656.18	116.381376	0.00515408	0.00517577	0.95627068	0.32812827	0.51515898	Arhgef9
ENSMUSG00000025658.17	11.0996688	0.00802656	0.01723611	0.27410492	0.60059136	0.77449687	Cnksr2
ENSMUSG00000025665.18	18.4111794	-0.0033568	0.01174101	0.0906948	0.76329571	0.89239083	Rps6ka6
ENSMUSG00000025666.17	142.982072	0.02732608	0.00442015	35.7667582	2.22E-09	2.95E-08	Tmem47
ENSMUSG00000025701.13	9.70035377	0.04684961	0.01224041	11.7454675	0.00060992	0.00284579	Alox5
ENSMUSG00000025702.16	2171.36147	-0.0026802	0.0027883	0.89366616	0.34448574	0.53275462	Marchf8
ENSMUSG00000025722.17	261.864135	0.00144062	0.0029725	0.23087115	0.63087863	0.79922137	Wdr73
ENSMUSG00000025723.13	5.69444425	-0.0024492	0.00789302	0.08921564	0.76517669	0.89352695	Nmb
ENSMUSG00000025724.13	837.944438	-0.0081769	0.00241157	11.5291607	0.00068513	0.0031543	Sec11a
ENSMUSG00000025726.12	7.53461379	0.06627956	0.00918777	40.9224056	1.58E-10	2.47E-09	Slc28a1
ENSMUSG00000025728.17	659.577532	-0.0014772	0.00263715	0.31138773	0.57682986	0.75527335	Pigq
ENSMUSG00000025730.16	188.067419	-0.0011299	0.00232302	0.23793678	0.62570027	0.7946673	Rab40c
ENSMUSG00000025731.17	1742.15499	-0.0203823	0.00380594	28.4781036	9.48E-08	9.90E-07	Mettl26
ENSMUSG00000025732.7	510.938662	-0.0206637	0.00490033	18.1045131	2.09E-05	0.00013936	Mcrip2
ENSMUSG00000025733.18	663.463063	-0.0125728	0.00309257	16.5640391	4.70E-05	0.00029102	Rhot2
ENSMUSG00000025735.14	29.8494181	-0.0118399	0.00619314	3.4381595	0.06370662	0.14682279	Rhbdl1
ENSMUSG00000025736.16	647.735852	-0.0040774	0.00209595	3.76554896	0.05231868	0.12507485	Jmjd8
ENSMUSG00000025737.6	299.052462	0.00066932	0.00209808	0.10165587	0.7498515	0.8832841	Wdr24
ENSMUSG00000025738.9	15.2161325	0.03315553	0.00838578	13.9627395	0.00018647	0.00100298	Fbxl16
ENSMUSG00000025742.6	294.570245	0.02325396	0.0022305	104.65831	1.45E-24	1.30E-22	Prps2
ENSMUSG00000025743.15	1317.09632	0.03897445	0.00425483	80.1685148	3.44E-19	1.61E-17	Sdc3
ENSMUSG00000025745.13	5576.57274	-0.0193079	0.00367395	27.0505632	1.98E-07	1.95E-06	Hadha
ENSMUSG00000025746.12	2.43014442	0.01818974	0.01510354	1.28028216	0.25784658	0.43132857	Il6
ENSMUSG00000025747.13	148.335118	-7.50E-05	0.00406894	0.00034209	0.98524341	1	Tyms
ENSMUSG00000025757.14	624.408799	-0.0144368	0.00425236	10.6365519	0.00110874	0.00480458	Hspa4l
ENSMUSG00000025758.11	32.4610019	0.01427387	0.00700267	3.89080781	0.04855107	0.11781126	Plk4
ENSMUSG00000025759.12	298.060101	-0.0108185	0.00302402	12.311097	0.00045027	0.00217646	Mfsd8
ENSMUSG00000025762.17	1635.89653	-0.0107239	0.00336209	10.1162021	0.00146972	0.00616261	Larp1b
ENSMUSG00000025764.15	864.877671	0.00626588	0.00309525	4.06967089	0.04365978	0.10805452	Jade1
ENSMUSG00000025766.15	235.684096	-4.87E-05	0.00270606	0.00032044	0.98571794	1	D3Ertd751e
ENSMUSG00000025776.14	2.42532259	-0.0206194	0.02112175	1.10070219	0.29411205	0.47653547	Crispld1
ENSMUSG00000025777.9	8.79416476	0.03949803	0.0179593	5.44406559	0.01963492	0.05604612	Gdap1
ENSMUSG00000025779.11	58.2539235	0.00921794	0.00335545	7.44362965	0.00636614	0.02174536	Ly96
ENSMUSG00000025780.8	305.693832	-0.0231896	0.00403157	33.3895657	7.54E-09	9.29E-08	Itih5
ENSMUSG00000025781.15	5625.3375	-0.0155456	0.00322841	23.4298291	1.30E-06	1.09E-05	Atp5f1c
ENSMUSG00000025782.13	310.653192	-0.002673	0.00430075	0.37903067	0.53812246	0.72218865	Taf3
ENSMUSG00000025784.6	9.23361097	0.00494125	0.01060984	0.20867515	0.6478078	0.81190258	Clec3b
ENSMUSG00000025785.6	331.646464	-0.0035463	0.00235465	2.31305071	0.12829204	0.25532833	Exosc7
ENSMUSG00000025786.18	543.424168	-0.0002176	0.00207569	0.01099176	0.91650149	0.98434129	Zdhhc3
ENSMUSG00000025787.7	1.77736091	-0.0055929	0.01052841	0.21257663	0.64475532	0.81004476	Tgm4
ENSMUSG00000025789.10	4.20031762	0.01575596	0.01040824	2.24627695	0.1339363	0.2637133	St8sia2
ENSMUSG00000025790.15	123.71211	0.01605573	0.0035229	19.8532139	8.36E-06	6.02E-05	Slco3a1
ENSMUSG00000025791.19	3639.19058	-0.0021942	0.00242718	0.81976494	0.3652489	0.55503902	Pgm1
ENSMUSG00000025792.10	2955.24877	0.00393893	0.00338412	1.30752027	0.25284412	0.42499457	Slc25a10
ENSMUSG00000025793.18	811.351355	-0.0021754	0.00228552	0.90316834	0.34193358	0.52984991	Hgs
ENSMUSG00000025794.10	4878.07224	-0.0016162	0.00381503	0.17961704	0.67170256	0.82942132	Rpl14
ENSMUSG00000025795.9	1564.3691	-0.0085966	0.00245938	12.1946579	0.00047927	0.00230552	Rassf3
ENSMUSG00000025804.6	23.8105479	0.09083393	0.00967058	68.2516774	1.44E-16	4.86E-15	Ccr1
ENSMUSG00000025808.18	0.76227116	-0.010065	0.04704529	0.00681163	0.93422318	0.99420601	Ccdc7a
ENSMUSG00000025809.16	3540.05567	-0.0011247	0.00244367	0.20794491	0.64838295	0.81215377	Itgb1
ENSMUSG00000025810.10	1142.40857	-0.0210989	0.00324631	41.750276	1.04E-10	1.66E-09	Nrp1
ENSMUSG00000025812.19	1263.89825	-0.0116312	0.00340192	11.5542371	0.00067595	0.00311995	Pard3
ENSMUSG00000025813.15	676.281634	-0.0138001	0.00402709	12.0051908	0.00053053	0.00252329	Homer2
ENSMUSG00000025815.15	960.983446	0.00886409	0.00459353	3.7509477	0.05277758	0.12600587	Dhtkd1
ENSMUSG00000025816.16	106.007667	0.00092224	0.0037325	0.06073322	0.80534078	0.91890899	Sec61a2
ENSMUSG00000025817.14	405.477562	-0.0038215	0.00288057	1.74102053	0.18700957	0.34018261	Nudt5
ENSMUSG00000025821.10	92.281651	0.00171443	0.00270413	0.3969875	0.52864915	0.71323183	Zfp282
ENSMUSG00000025823.10	4338.88425	0.00604892	0.00352858	2.7921741	0.09472563	0.20155153	Pdia4
ENSMUSG00000025825.13	1276.03134	-0.0035998	0.00236885	2.3037345	0.12906336	0.2563227	Iscu
ENSMUSG00000025854.16	912.304894	0.00783219	0.00321133	5.94465935	0.01476194	0.0442971	Fam20c

ENSMUSG00000025855.14	25.5502522	0.0417271	0.00841005	21.0805985	4.40E-06	3.37E-05	Prkar1b
ENSMUSG00000025856.16	197.751859	0.01539477	0.00414608	13.5150167	0.00023666	0.00123713	Pdgfa
ENSMUSG00000025857.11	139.352723	-0.0085324	0.00244366	12.2845164	0.00045673	0.00220521	Dnaaf5
ENSMUSG00000025858.13	510.49451	-0.00148	0.00256407	0.33694288	0.56159918	0.74269012	Get4
ENSMUSG00000025860.16	2099.05668	-0.01055	0.00293232	12.625283	0.00038056	0.00187782	Xiap
ENSMUSG00000025862.15	1261.37502	-0.0060781	0.00353476	2.83397453	0.0922897	0.19740136	Stag2
ENSMUSG00000025867.9	56.0191067	0.02354217	0.00608009	14.7624757	0.00012194	0.00068412	Cplx2
ENSMUSG00000025868.7	820.134494	-0.0113555	0.00243728	21.8981166	2.88E-06	2.28E-05	Higd2a
ENSMUSG00000025869.12	450.868862	-0.0168739	0.00281104	35.8509821	2.13E-09	2.82E-08	Nop16
ENSMUSG00000025870.11	32.9911738	-0.0007521	0.00411173	0.03289663	0.8560738	0.95096663	Arl10
ENSMUSG00000025871.19	1248.57682	-0.0131488	0.0024251	29.4219917	5.82E-08	6.25E-07	4833439L19Rik
ENSMUSG00000025872.6	398.386071	-0.0038101	0.0021523	3.18039686	0.07452663	0.16655155	Thoc3
ENSMUSG00000025873.17	979.4826	-0.0024948	0.00246609	1.01703668	0.31322295	0.4986925	Faf2
ENSMUSG00000025875.15	41.245691	0.01536421	0.00436916	11.8798554	0.00056744	0.0026714	Tspan17
ENSMUSG00000025876.16	45.4144864	0.02113041	0.00492772	17.6028728	2.72E-05	0.0001767	Unc5a
ENSMUSG00000025877.15	97.9547003	0.05078675	0.00526359	90.8004779	1.59E-21	9.89E-20	Hk3
ENSMUSG00000025878.17	466.169	-0.0037917	0.0028866	1.74410012	0.18662015	0.33971261	Uimc1
ENSMUSG00000025880.13	248.850254	0.01167016	0.00435665	6.93905709	0.00843336	0.02761476	Smad7
ENSMUSG00000025885.19	483.645817	0.00321981	0.00338823	0.88461031	0.34694205	0.53521978	Myo5b
ENSMUSG00000025887.11	61.6995611	0.05246228	0.0059162	71.7108742	2.49E-17	9.40E-16	Casp12
ENSMUSG00000025888.7	82.5242328	0.04092231	0.00549217	53.0620671	3.23E-13	7.26E-12	Casp1
ENSMUSG00000025889.14	56.2234087	0.02179012	0.0077278	6.89580453	0.00863983	0.02821642	Snca
ENSMUSG00000025892.17	11.6861905	0.05837086	0.00998535	31.468375	2.03E-08	2.34E-07	Gria4
ENSMUSG00000025893.9	93.9916449	-0.0061808	0.00510693	1.43470775	0.23099767	0.39861801	Kbtbd3
ENSMUSG00000025894.12	639.121362	-0.015008	0.0024927	35.9589078	2.02E-09	2.68E-08	Aasdhppt
ENSMUSG00000025898.7	601.932133	0.00382318	0.00363003	1.07714	0.29933824	0.48328735	Cwf19l2
ENSMUSG00000025899.15	205.970206	-0.0031955	0.00279101	1.2828764	0.2573649	0.43087795	Alkbh8
ENSMUSG00000025900.14	2.47505918	-0.0003374	0.02221922	-0.0079584	1	1	Rp1
ENSMUSG00000025902.14	37.4130095	-0.000988	0.00451184	0.04780472	0.82692823	0.93204288	Sox17
ENSMUSG00000025903.15	3716.60527	-0.0093327	0.00249715	13.8113886	0.00020211	0.00107727	Lypla1
ENSMUSG00000025905.15	1.50409247	0.01514658	0.02339864	0.5942622	0.44077567	0.63283023	Oprk1
ENSMUSG00000025907.15	1065.95119	-0.0108176	0.0027123	15.566149	7.97E-05	0.00046584	Rb1cc1
ENSMUSG00000025909.17	36.3713886	0.05425317	0.0075047	48.6308897	3.09E-12	6.12E-11	Sntg1
ENSMUSG00000025911.15	1932.83359	-0.0033834	0.00237111	2.03203518	0.15401425	0.29323378	Adhfe1
ENSMUSG00000025912.17	27.5049561	0.04739257	0.00863472	26.7716008	2.29E-07	2.24E-06	Mybl1
ENSMUSG00000025915.15	281.713897	0.01234187	0.00306762	15.9030468	6.67E-05	0.0003976	Sgk3
ENSMUSG00000025916.11	6.14031342	-0.0449356	0.01542968	8.92369941	0.00281494	0.01082732	Ppp1r42
ENSMUSG00000025917.10	1106.08331	-0.0101986	0.00283218	12.9213864	0.00032485	0.00163313	Cops5
ENSMUSG00000025920.20	137.587259	0.00246521	0.00401998	0.36873584	0.54369415	0.72712465	Stau2
ENSMUSG00000025921.8	505.407511	0.00434338	0.00260371	2.77513236	0.09573874	0.20313908	Rdh10
ENSMUSG00000025925.15	180.080771	0.00102037	0.00244703	0.17446141	0.67617687	0.83267936	Terf1
ENSMUSG00000025927.14	6.59357685	0.02312292	0.02433805	1.29894945	0.25440521	0.42671037	Tfap2b
ENSMUSG00000025930.7	9.6853963	0.04586657	0.00811508	28.525696	9.25E-08	9.68E-07	Msc
ENSMUSG00000025931.16	28.1290961	0.0181868	0.00619961	8.3006836	0.00396302	0.01449623	Paqr8
ENSMUSG00000025932.15	4.7585957	0.08359849	0.02208433	4.79035393	0.02861955	0.07647603	Eya1
ENSMUSG00000025933.16	72.2503395	-0.0129157	0.00360284	12.6685077	0.00037187	0.00183875	Tmem14a
ENSMUSG00000025934.16	30379.1746	0.00042497	0.0047892	0.00767848	0.93017319	0.99177898	Gsta3
ENSMUSG00000025935.11	3203.12273	0.00331006	0.00248054	1.74611791	0.18636551	0.33935126	Tram1
ENSMUSG00000025937.7	7111.54368	-0.0096484	0.00339016	7.83612044	0.00512124	0.01804985	Lactb2
ENSMUSG00000025938.17	9.78326342	0.02368267	0.01353928	3.02883567	0.08179669	0.1792823	Slco5a1
ENSMUSG00000025939.20	441.151394	-0.0009054	0.00193122	0.21530068	0.64264413	0.80862066	Ube2w
ENSMUSG00000025940.7	926.177537	-0.0097114	0.00258411	14.0064462	0.00018218	0.0009833	Tmem70
ENSMUSG00000025949.17	519.692633	0.00388136	0.00290809	1.76077633	0.18452771	0.33688913	Pikfyve
ENSMUSG00000025950.17	8783.80444	-0.002237	0.00260092	0.72145896	0.39566576	0.58748332	Idh1
ENSMUSG00000025956.11	146.119765	0.00164103	0.00266848	0.38102968	0.53705269	0.72135106	Mettl21a
ENSMUSG00000025958.15	424.752183	0.00629321	0.00341234	3.29334693	0.06956109	0.15740842	Creb1
ENSMUSG00000025959.14	181.302211	0.00826408	0.00322817	6.351096	0.01173088	0.03640715	Klf7
ENSMUSG00000025962.16	427.932213	-0.0087781	0.00377939	5.39185693	0.02023093	0.05752462	Fastkd2
ENSMUSG00000025964.16	295.911317	0.01428571	0.00418789	11.7583137	0.00060572	0.00282734	Adam23
ENSMUSG00000025967.17	5521.15728	-0.0057568	0.00390931	2.19266287	0.1386694	0.27093881	Eef1b2
ENSMUSG00000025968.17	2215.06108	-0.0165632	0.00332209	25.0038864	5.72E-07	5.16E-06	Ndufs1

ENSMUSG00000025969.16	463.683817	0.01824022	0.00343539	26.7244452	2.35E-07	2.28E-06	Nrp2
ENSMUSG00000025971.7	370.712898	-0.0129732	0.0021128	37.8541013	7.62E-10	1.07E-08	Maip1
ENSMUSG00000025978.15	23.9683817	0.02513392	0.0052302	21.9349662	2.82E-06	2.24E-05	Rftn2
ENSMUSG00000025979.14	828.976522	-0.0061653	0.00278886	4.77286327	0.0289117	0.07708698	Mob4
ENSMUSG00000025980.15	7400.7127	-0.0159325	0.00213735	54.6861868	1.41E-13	3.31E-12	Hspd1
ENSMUSG00000025981.14	554.246228	-0.011047	0.00500558	4.95048183	0.0260834	0.07093313	Coq10b
ENSMUSG00000025982.14	2736.91071	0.00466305	0.00266645	3.08787952	0.07887754	0.1741834	Sf3b1
ENSMUSG00000025983.12	3.73577508	0.00920006	0.01611608	0.35936122	0.54886121	0.73158749	Ccdc150
ENSMUSG00000025986.7	142.362217	0.01315478	0.00451703	7.77259012	0.00530449	0.01860122	Slc39a10
ENSMUSG00000025991.9	64203.9856	-0.0066625	0.0060851	1.18796879	0.27573966	0.45422514	Cps1
ENSMUSG00000025993.11	3527.96872	0.01969608	0.00341638	32.9762407	9.33E-09	1.13E-07	Slc40a1
ENSMUSG00000025995.17	314.655178	-0.0086212	0.00440369	3.86158211	0.04940372	0.11940119	Wdr75
ENSMUSG00000025997.14	26.2520783	0.06685025	0.00835554	51.692248	6.49E-13	1.40E-11	Ikzf2
ENSMUSG00000026000.17	373.059873	-0.014154	0.00227692	38.9191996	4.42E-10	6.44E-09	Lancl1
ENSMUSG00000026003.6	4017.00231	-0.005418	0.00285262	3.57763984	0.0585623	0.13720576	Acadl
ENSMUSG00000026004.16	234.92812	0.00333107	0.00269124	1.51370757	0.21857421	0.38302824	Kansl1l
ENSMUSG00000026005.16	613.470371	-0.0117801	0.00209122	31.3685796	2.13E-08	2.45E-07	Rpe
ENSMUSG00000026009.15	23.9403249	0.03166879	0.00655916	21.8594994	2.93E-06	2.33E-05	Icos
ENSMUSG00000026011.14	4.0286825	0.13589579	0.01537926	56.6485718	5.21E-14	1.29E-12	Ctla4
ENSMUSG00000026012.3	13.2895856	0.06528021	0.00790742	58.7993681	1.75E-14	4.64E-13	Cd28
ENSMUSG00000026014.16	1510.66207	-0.0106457	0.00342445	9.36836148	0.00220763	0.00880736	Raph1
ENSMUSG00000026017.14	103.113304	0.01024792	0.00401621	6.26288965	0.01232929	0.03799468	Carf
ENSMUSG00000026018.13	7.8828083	0.0388108	0.01744503	4.37997935	0.03636344	0.09315433	Ica1l
ENSMUSG00000026019.16	318.594874	-0.0168872	0.00373169	20.5085629	5.94E-06	4.43E-05	Wdr12
ENSMUSG00000026020.10	550.684229	-0.0053214	0.0043043	1.51110893	0.21896995	0.38344534	Nop58
ENSMUSG00000026021.16	942.178744	0.00271151	0.00301974	0.80285593	0.37024086	0.56081825	Sumo1
ENSMUSG00000026023.17	1.7171277	-0.0138054	0.01478316	0.84829426	0.35703533	0.54579269	Cdk15
ENSMUSG00000026024.15	1462.42083	-0.004809	0.00365983	1.69018359	0.19357677	0.34903308	Als2
ENSMUSG00000026027.14	614.030718	-0.0106966	0.00191306	30.8958276	2.72E-08	3.08E-07	Stradb
ENSMUSG00000026028.13	479.494638	-0.0035748	0.0037974	0.87065092	0.35077517	0.53922852	Trak2
ENSMUSG00000026029.15	495.393251	-0.0031804	0.00272388	1.32979049	0.24884249	0.4202792	Casp8
ENSMUSG00000026031.16	2216.17071	-0.0002974	0.00274712	0.01159468	0.91425064	0.98312004	Cflar
ENSMUSG00000026032.9	1183.56203	-0.0283933	0.00402605	49.8604353	1.65E-12	3.36E-11	Ndufb3
ENSMUSG00000026034.18	1413.69987	-0.0004086	0.00337712	0.01465694	0.90363888	0.9776261	Clk1
ENSMUSG00000026035.16	185.518355	0.00294116	0.00299657	0.95304167	0.32894627	0.51591659	Ppil3
ENSMUSG00000026036.18	251.92463	-0.006166	0.00291894	4.42867232	0.03533999	0.09096682	Nif3l1
ENSMUSG00000026037.15	121.275719	0.00464316	0.00395972	1.33578701	0.24777831	0.419052	Orc2
ENSMUSG00000026039.10	17.8362734	0.01943106	0.01025808	3.43965594	0.06364895	0.14673287	Sgo2a
ENSMUSG00000026042.17	279.876191	0.08461557	0.00913224	71.704228	2.50E-17	9.42E-16	Col5a2
ENSMUSG00000026043.19	925.578247	-5.96E-05	0.00652587	9.35E-05	0.99228622	1	Col3a1
ENSMUSG00000026047.14	134.214309	-0.0074558	0.00258591	8.48533644	0.0035802	0.01329084	Poglut2
ENSMUSG00000026048.17	315.540844	-0.0066453	0.0027369	5.92643066	0.01491542	0.04466673	Ercc5
ENSMUSG00000026049.12	149.052486	-0.0167882	0.00344328	23.8434231	1.04E-06	8.96E-06	Tex30
ENSMUSG00000026058.12	3.36453302	0.02078267	0.01929529	1.564137	0.21106051	0.37318557	Khdrbs2
ENSMUSG00000026062.13	5.6334012	0.01939374	0.01477366	1.59457012	0.2066744	0.36736925	Slc9a2
ENSMUSG00000026064.17	166.814835	0.00546253	0.00640095	0.6756507	0.41108842	0.6026742	Ptp4a1
ENSMUSG00000026065.9	0.85817117	0.04127903	0.02982546	4.43857673	0.03513553	0.09053934	Slc9a4
ENSMUSG00000026068.12	18.7995198	0.06071105	0.00896841	38.2528272	6.21E-10	8.88E-09	Il18rap
ENSMUSG00000026069.16	4.10481594	0.04803525	0.01504511	7.77033166	0.00531113	0.01861895	Il1rl1
ENSMUSG00000026070.17	16.2664286	0.06441823	0.00709414	76.3072419	2.43E-18	1.02E-16	Il18r1
ENSMUSG00000026072.13	767.712842	-0.0184758	0.00536576	11.4172024	0.00072767	0.00332356	Il1r1
ENSMUSG00000026073.14	3.51346869	0.07219695	0.01399787	22.5270903	2.07E-06	1.69E-05	Il1r2
ENSMUSG00000026074.16	357.062001	0.01659787	0.00473924	11.777117	0.00059963	0.0028028	Map4k4
ENSMUSG00000026077.16	77.0783227	-0.0038222	0.01199328	0.088908	0.76557003	0.89376502	Npas2
ENSMUSG00000026078.11	333.884219	9.95E-05	0.0030336	0.00109998	0.9735422	1	Pdcl3
ENSMUSG00000026080.15	6.02543444	0.0725262	0.01070366	42.0998802	8.67E-11	1.40E-09	Chst10
ENSMUSG00000026082.12	249.853261	0.00175053	0.00334355	0.27988036	0.59677964	0.77160644	Rev1
ENSMUSG00000026083.13	3209.84535	-0.0039651	0.00376703	1.10284197	0.29364326	0.47584128	Eif5b
ENSMUSG00000026087.12	879.09691	-0.0107767	0.00325751	11.0325438	0.00089526	0.00397883	Mrpl30
ENSMUSG00000026088.16	212.227174	0.00095666	0.00409475	0.05493277	0.81469199	0.9242742	Mitd1
ENSMUSG00000026090.17	9.90970008	0.03874179	0.00876597	19.5866624	9.61E-06	6.84E-05	Cracdl

ENSMUSG00000026094.15	176.810989	0.03073831	0.00491389	35.5593904	2.47E-09	3.26E-08	Stk17b
ENSMUSG00000026095.16	795.057457	-0.0057256	0.00261664	4.77755002	0.02883311	0.07692962	Asnsd1
ENSMUSG00000026096.15	345.395645	-0.0097478	0.00302181	10.440538	0.00123279	0.00528571	Osgepl1
ENSMUSG00000026097.10	828.433772	-0.010858	0.00260628	17.3700598	3.08E-05	0.00019706	Ormdl1
ENSMUSG00000026098.14	132.978649	-0.0096964	0.00377875	6.58410549	0.01028933	0.03266714	Pms1
ENSMUSG00000026102.10	285.004556	-0.0088891	0.00373701	5.60009802	0.01795947	0.05208313	Inpp1
ENSMUSG00000026103.15	219.17409	0.02760936	0.00493518	29.4208328	5.82E-08	6.25E-07	Gls
ENSMUSG00000026104.15	1514.47502	0.03729377	0.00440436	70.774956	4.00E-17	1.47E-15	Stat1
ENSMUSG00000026107.12	424.35541	0.00670326	0.00377142	3.07981604	0.07926949	0.17484805	Nabp1
ENSMUSG00000026109.15	5.60226655	-0.0162153	0.01056807	2.22177134	0.13607686	0.26690301	Tmeff2
ENSMUSG00000026110.16	59.3306112	0.04775288	0.0066767	47.4054751	5.77E-12	1.10E-10	Mgat4a
ENSMUSG00000026111.16	554.617651	-0.003631	0.00256498	2.00416583	0.15686756	0.29722673	Unc50
ENSMUSG00000026112.8	1808.13473	-0.0076181	0.00202291	14.1241716	0.00017113	0.00093002	Coa5
ENSMUSG00000026113.18	391.704432	-0.0002493	0.0035289	0.00502771	0.94347232	0.99884896	Inpp4a
ENSMUSG00000026114.15	4.2493638	-0.0117663	0.03505887	0.10637135	0.74431374	0.87943531	Cnga3
ENSMUSG00000026116.12	602.254335	-0.0017607	0.00284753	0.37812537	0.53860822	0.72257627	Tmem131
ENSMUSG00000026117.11	239.237665	0.02834666	0.00527232	26.5869299	2.52E-07	2.43E-06	Zap70
ENSMUSG00000026121.14	120.386728	0.01190623	0.00421876	7.70152716	0.00551741	0.01924485	Sema4c
ENSMUSG00000026123.12	498.874886	0.0160967	0.00379784	17.4410133	2.96E-05	0.00019067	Plekhb2
ENSMUSG00000026126.16	87.4510529	0.05031084	0.00813492	32.9958436	9.24E-09	1.12E-07	Ptpn18
ENSMUSG00000026127.14	632.504862	-0.0064807	0.00350257	3.3687678	0.06644343	0.15185415	Imp4
ENSMUSG00000026131.22	1970.80346	-0.0029488	0.00357625	0.67269756	0.41211269	0.60355329	Dst
ENSMUSG00000026134.12	48.8706325	0.0109568	0.00375673	8.4915295	0.00356804	0.01325488	Prim2
ENSMUSG00000026135.18	124.007945	-0.00485	0.00368731	1.69800578	0.19254898	0.34779142	Zfp142
ENSMUSG00000026141.14	2.07302579	0.03094764	0.02102591	3.8358316	0.0501681	0.12088933	Col19a1
ENSMUSG00000026142.16	375.045189	0.00462931	0.00205535	4.99653047	0.02539818	0.06938973	Rhbdd1
ENSMUSG00000026149.3	1.43384141	-0.0030438	0.01762439	-0.0315037	1	1	Tm4sf20
ENSMUSG00000026150.15	858.391721	-0.0054712	0.00279291	3.85404673	0.04962612	0.11982818	Mff
ENSMUSG00000026153.16	531.102622	-0.0123733	0.0027381	20.6791081	5.43E-06	4.08E-05	Fam135a
ENSMUSG00000026154.5	321.64096	-0.0126996	0.00389048	10.8273799	0.0010001	0.00439508	Sdhaf4
ENSMUSG00000026155.14	597.248723	-0.0030705	0.00315542	0.93648456	0.33318357	0.52004947	Smap1
ENSMUSG00000026156.9	16.9607462	0.01478344	0.01021586	2.31480682	0.12814722	0.25522192	B3gat2
ENSMUSG00000026158.12	391.467246	0.01258303	0.00375934	10.619991	0.00111872	0.00484503	Ogfrl1
ENSMUSG00000026159.14	445.890673	0.00733169	0.00268785	7.27516108	0.00699146	0.02360782	Agfg1
ENSMUSG00000026162.9	60.2214453	-0.0155923	0.0028121	30.0424251	4.23E-08	4.63E-07	Nhej1
ENSMUSG00000026163.18	10.6227027	0.02674752	0.01846179	2.17797241	0.13999879	0.27289352	Sphkap
ENSMUSG00000026166.15	0.87075074	0.08194788	0.02687842	11.7143215	0.00062021	0.00288811	Ccl20
ENSMUSG00000026167.15	2.36546058	0.11949128	0.02502052	10.2785872	0.00134583	0.00569683	Wnt10a
ENSMUSG00000026170.7	7213.9213	-0.0165553	0.00365874	20.1528937	7.15E-06	5.24E-05	Cyp27a1
ENSMUSG00000026171.13	226.991202	-0.0056968	0.00273936	4.37757168	0.03641484	0.09325521	Rnf25
ENSMUSG00000026172.13	202.049417	-0.0112335	0.0021081	28.6235617	8.79E-08	9.21E-07	Bcs1l
ENSMUSG00000026173.17	4.2167295	0.00757862	0.01492854	0.33562497	0.56236552	0.74345346	Plcd4
ENSMUSG00000026174.10	341.878418	0.00646617	0.0018842	11.795364	0.00059378	0.00277822	Cnot9
ENSMUSG00000026175.13	12.3834565	0.02796354	0.00958328	8.38969868	0.00377353	0.01389363	Vil1
ENSMUSG00000026176.14	2442.57032	-0.0006303	0.00221994	0.0800061	0.77728914	0.90161792	Ctdsp1
ENSMUSG00000026177.12	212.507969	0.04363758	0.00537277	61.8075695	3.79E-15	1.09E-13	Slc11a1
ENSMUSG00000026179.15	1637.58148	-0.0144313	0.00326138	19.5789919	9.65E-06	6.87E-05	Pnkd
ENSMUSG00000026180.10	8.9155076	0.05245777	0.01021966	20.6675797	5.46E-06	4.10E-05	Cxcr2
ENSMUSG00000026181.14	523.879343	-0.0035075	0.00287438	1.48455061	0.22306389	0.3885374	Ppm1f
ENSMUSG00000026182.7	8.65920985	-0.1584968	0.06895536	1.49318664	0.22172269	0.38685837	Tnp1
ENSMUSG00000026185.9	374.438719	0.01866418	0.00525005	11.9981232	0.00053254	0.00253236	Igfbp5
ENSMUSG00000026187.10	361.575969	0.00186751	0.00186797	1.01296224	0.31419422	0.49956754	Xrcc5
ENSMUSG00000026188.12	31.5532501	0.01759652	0.01016565	2.84405043	0.09171276	0.19641671	Tmem169
ENSMUSG00000026189.14	2784.13477	-0.0094425	0.0037524	6.36764656	0.01162196	0.03612875	Pecr
ENSMUSG00000026192.14	413.121833	-0.0001803	0.00251917	0.00506058	0.94328811	0.99877588	Atic
ENSMUSG00000026193.16	23503.4924	0.00058255	0.00397872	0.02178449	0.88266185	0.96542675	Fn1
ENSMUSG00000026196.8	7.54662274	0.02486504	0.00905604	6.8882079	0.00867662	0.02831698	Bard1
ENSMUSG00000026197.13	461.578142	-0.0002904	0.00278661	0.01090473	0.91683153	0.98449298	Zfand2b
ENSMUSG00000026198.16	1238.44531	-0.0181584	0.00420546	18.2854178	1.90E-05	0.00012778	Abcb6
ENSMUSG00000026199.19	366.083795	-0.0065457	0.00305098	4.67842635	0.03054372	0.0807085	Ankzf1
ENSMUSG00000026200.14	83.3177914	0.02487002	0.0062231	15.8892663	6.72E-05	0.0004	Glb1l

ENSMUSG00000026201.13	1459.38639	-0.0143876	0.00242625	35.1340483	3.08E-09	4.01E-08	Stk16
ENSMUSG00000026202.14	2681.63598	-0.0139507	0.00320822	18.9695408	1.33E-05	9.21E-05	Tuba4a
ENSMUSG00000026203.17	944.891911	-0.0033178	0.00288527	1.2772765	0.25840604	0.43187606	Dnajb2
ENSMUSG00000026204.16	19.694451	0.02609746	0.01412384	3.42265141	0.06430765	0.14798191	Ptprn
ENSMUSG00000026205.9	2.15521664	0.01032776	0.01002239	1.05630453	0.30405955	0.48866427	Slc23a3
ENSMUSG00000026207.17	32.8859514	0.00772629	0.00854921	0.78190416	0.37655941	0.56769129	Speg
ENSMUSG00000026208.10	402.091377	0.01082612	0.00557123	3.59237464	0.05804524	0.13634101	Des
ENSMUSG00000026209.16	826.517756	-0.010366	0.0033686	9.37282487	0.00220226	0.0087904	Dnpep
ENSMUSG00000026211.19	33.0370458	0.00779909	0.0074309	1.06041029	0.30312163	0.48782582	Obsl1
ENSMUSG00000026213.16	125.351516	0.01135963	0.00342035	10.9150285	0.00095387	0.00421154	Stk11ip
ENSMUSG00000026219.15	2319.52813	0.00129957	0.00204482	0.40204298	0.52603606	0.71121161	Trip12
ENSMUSG00000026220.7	2.21736497	0.02995471	0.02337659	2.09889101	0.14740602	0.2837211	Slc16a14
ENSMUSG00000026222.17	275.619712	0.02812423	0.00386304	51.5956664	6.82E-13	1.47E-11	Sp100
ENSMUSG00000026223.16	702.768139	0.01850353	0.00354901	26.7744381	2.29E-07	2.23E-06	Itm2c
ENSMUSG00000026226.17	7.32920793	-0.0562143	0.02381984	-2.0036348	1	1	Spata3
ENSMUSG00000026227.12	1255.20871	-0.0132803	0.00474938	7.64203061	0.00570237	0.01980213	2810459M11Rik
ENSMUSG00000026228.7	2.45303548	0.00153212	0.01509609	0.04534742	0.83136648	0.93502633	Htr2b
ENSMUSG00000026229.18	2431.77618	-0.003456	0.00261286	1.71970678	0.18973079	0.34396545	Psmd1
ENSMUSG00000026234.13	3748.9685	0.00148975	0.00341042	0.19251105	0.66083486	0.82159984	Ncl
ENSMUSG00000026235.15	55.290028	0.03543078	0.00769432	20.3008455	6.62E-06	4.88E-05	Epha4
ENSMUSG00000026238.15	5257.06907	0.00777732	0.00268004	8.24353002	0.00408975	0.01489973	Ptma
ENSMUSG00000026239.15	109.443463	-0.0022355	0.00339921	0.43572976	0.50919075	0.69585241	Pde6d
ENSMUSG00000026240.13	177.135064	-0.019469	0.0040154	23.583315	1.20E-06	1.02E-05	Cops7b
ENSMUSG00000026245.17	578.51742	-0.0047847	0.00231828	4.25964593	0.03902806	0.09854574	Farsb
ENSMUSG00000026248.10	373.151278	-0.0100909	0.00215095	22.0030469	2.72E-06	2.17E-05	Mrpl44
ENSMUSG00000026249.11	309.236599	0.00208137	0.00584247	0.09766306	0.75465229	0.88645993	Serpine2
ENSMUSG00000026254.18	956.52023	-0.0110954	0.00226855	24.0413732	9.43E-07	8.14E-06	Eif4e2
ENSMUSG00000026255.16	12.3497595	0.0105261	0.00734696	2.05470148	0.15173699	0.28999827	Efhd1
ENSMUSG00000026259.15	473.508587	0.00342693	0.00451886	0.57560878	0.44803871	0.63990801	Ngef
ENSMUSG00000026260.13	2623.50589	-0.0148952	0.00272291	29.9553321	4.42E-08	4.83E-07	Ndufa10
ENSMUSG00000026269.15	1139.97207	-0.005954	0.00276846	4.60572176	0.03186544	0.08362949	Rnpepl1
ENSMUSG00000026270.12	353.548237	0.00262529	0.00314384	0.71303462	0.39843823	0.59026217	Capn10
ENSMUSG00000026271.16	28.5367284	0.06825311	0.0081305	64.4504949	9.90E-16	3.06E-14	Gpr35
ENSMUSG00000026272.7	4585.82096	-0.0044242	0.00552678	0.63961552	0.42385006	0.61550157	Agxt
ENSMUSG00000026273.14	244.071948	-0.0095774	0.00203754	22.0963504	2.59E-06	2.08E-05	Mterf4
ENSMUSG00000026274.12	16.8199634	0.00583033	0.00738165	0.58092832	0.44594868	0.63815596	Pask
ENSMUSG00000026275.14	490.504821	-0.006702	0.00394326	2.87452739	0.08999139	0.19352087	Ppp1r7
ENSMUSG00000026276.21	381.410037	0.00465087	0.00382671	1.42412999	0.23272482	0.40063117	Septin2
ENSMUSG00000026277.15	844.986221	-0.0059572	0.00196438	9.27104832	0.00232804	0.00919753	Stk25
ENSMUSG00000026278.15	223.623208	-0.013189	0.00471919	7.70733578	0.00549969	0.01919274	Bok
ENSMUSG00000026279.15	210.564217	-0.0047605	0.00321284	2.18925887	0.13897617	0.27125886	Thap4
ENSMUSG00000026280.18	474.717115	-0.0021225	0.00295655	0.51954734	0.47103484	0.66181139	Atg4b
ENSMUSG00000026281.16	317.178145	-0.0059153	0.00310319	3.66993026	0.0554025	0.13131579	Dtymk
ENSMUSG00000026283.14	229.246865	-0.003003	0.00305202	0.945468	0.33087557	0.51774903	Ing5
ENSMUSG00000026285.9	5.96430486	0.18668002	0.01503295	109.153795	1.50E-25	1.52E-23	Pdcd1
ENSMUSG00000026288.15	132.811062	0.0468859	0.00537185	70.6941629	4.17E-17	1.53E-15	Inpp5d
ENSMUSG00000026289.16	583.529821	-0.0028734	0.00222736	1.68439591	0.19434136	0.3500638	Atg16l1
ENSMUSG00000026295.13	1610.46873	-0.0087679	0.00454059	3.84929833	0.04976681	0.12013099	Spp2
ENSMUSG00000026301.17	1.16609687	-0.0288158	0.02020882	1.97783592	0.15961857	0.3010366	Iqca
ENSMUSG00000026303.7	32.4908412	0.0455045	0.00408549	117.686301	2.03E-27	2.72E-25	Mlph
ENSMUSG00000026304.14	493.248158	-0.0019032	0.00424959	0.20245793	0.65274415	0.81453403	Rab17
ENSMUSG00000026305.16	800.896658	0.0005979	0.00329755	0.0324095	0.85713185	0.95166934	Lrrfip1
ENSMUSG00000026307.13	1426.07349	0.00054377	0.0029502	0.03420876	0.85326347	0.94947501	Scly
ENSMUSG00000026308.9	3.79560704	0.03867544	0.02645523	0.17865054	0.67253552	0.83015618	Klhl30
ENSMUSG00000026309.15	483.74888	-0.0070538	0.00191275	13.579478	0.00022867	0.00120015	Ilkap
ENSMUSG00000026311.17	126.045048	-0.005952	0.00389858	2.32366473	0.12741952	0.25399905	Asb1
ENSMUSG00000026312.18	3.43904921	0.00813276	0.02108106	0.35817641	0.54952076	0.7321356	Cdh7
ENSMUSG00000026313.17	250.386436	0.00247236	0.00406523	0.36290806	0.54689559	0.72970976	Hdac4
ENSMUSG00000026315.14	88.7395289	0.04059204	0.00505572	59.6670468	1.12E-14	3.06E-13	Serpinb8
ENSMUSG00000026317.8	616.095893	-0.0003163	0.00365579	0.00752891	0.9308549	0.99214351	Cln8
ENSMUSG00000026319.14	398.516991	0.00123831	0.00286042	0.18878856	0.66392681	0.82378756	Relch

ENSMUSG00000026321.9	32.1717196	0.05567371	0.00678	63.6706446	1.47E-15	4.46E-14	Tnfrsf11a
ENSMUSG00000026333.15	202.342776	-0.0158343	0.00331029	22.9623261	1.65E-06	1.37E-05	Gin1
ENSMUSG00000026335.17	727.852631	0.01021689	0.00371657	7.30395264	0.00688031	0.02329586	Pam
ENSMUSG00000026339.19	266.422894	0.00264842	0.00267073	0.97826796	0.32262679	0.50906312	Ccdc93
ENSMUSG00000026341.17	1817.54206	0.01607793	0.00243582	42.5438659	6.91E-11	1.14E-09	Actr3
ENSMUSG00000026342.11	945.606413	0.00359041	0.00253064	1.9829582	0.15907911	0.30021568	Slc35f5
ENSMUSG00000026343.7	102.362962	0.0153916	0.00307992	24.6205645	6.98E-07	6.17E-06	Gpr39
ENSMUSG00000026344.10	8.5638412	0.03094962	0.01551625	3.84242277	0.04997126	0.12052584	Lypd1
ENSMUSG00000026347.14	7.8607617	0.04063263	0.00997014	15.3860027	8.76E-05	0.00050686	Tmem163
ENSMUSG00000026348.8	519.381833	-0.0040482	0.00667371	0.33783858	0.5610795	0.74231026	Acmsd
ENSMUSG00000026349.15	645.419836	-0.0035793	0.00269352	1.75820344	0.18484876	0.3372925	Ccnt2
ENSMUSG00000026353.10	5519.08924	-0.0056083	0.00419509	1.74047567	0.18707857	0.34028188	Ubxn4
ENSMUSG00000026355.12	124.369075	0.02418237	0.00622689	14.917002	0.00011235	0.00063468	Mcm6
ENSMUSG00000026356.16	1435.53354	-0.0066548	0.00198413	11.2028569	0.00081671	0.00367752	Dars
ENSMUSG00000026357.4	8.53714613	0.07097121	0.01126979	30.3867155	3.54E-08	3.93E-07	Rgs18
ENSMUSG00000026358.14	59.8018675	0.08715015	0.00881856	83.020152	8.12E-20	4.16E-18	Rgs1
ENSMUSG00000026360.10	156.447225	0.04673567	0.00645522	47.2253503	6.33E-12	1.20E-10	Rgs2
ENSMUSG00000026361.10	433.961385	-0.0005369	0.00330426	0.02585168	0.87226307	0.95996741	Cdc73
ENSMUSG00000026365.16	19403.2528	-0.0009748	0.00399937	0.05877919	0.80843599	0.92088955	Cfh
ENSMUSG00000026368.7	3626.82883	9.11E-05	0.0047499	0.00038433	0.98435896	1	F13b
ENSMUSG00000026374.15	826.88203	-0.0064287	0.00250111	6.49744441	0.01080297	0.03407455	Tsn
ENSMUSG00000026377.13	433.405055	-0.0041391	0.00215653	3.6869819	0.05483876	0.13017583	Nifk
ENSMUSG00000026380.11	140.357118	0.0165321	0.00463437	12.5086518	0.00040507	0.00198546	Tfcp2l1
ENSMUSG00000026383.15	1015.35533	-0.0052701	0.00324636	2.64872197	0.10363318	0.21583234	Epb41l5
ENSMUSG00000026384.14	246.932868	-0.0104763	0.0046628	4.85942177	0.02749555	0.07399704	Ptpn4
ENSMUSG00000026385.17	19791.7994	-0.0061496	0.0037081	2.73273334	0.0983109	0.20746166	Dbi
ENSMUSG00000026388.16	7.94331319	0.00239046	0.0069022	0.11984582	0.72920178	0.8694723	3110009E18Rik
ENSMUSG00000026389.17	358.32488	-0.0070807	0.00313053	5.19775901	0.02261603	0.0630373	Steap3
ENSMUSG00000026390.8	321.357716	0.06017611	0.0097322	33.1623332	8.48E-09	1.04E-07	Marco
ENSMUSG00000026393.11	1442.07139	-0.0017582	0.00215433	0.64677378	0.4212685	0.61280615	Nek7
ENSMUSG00000026394.6	10.408189	0.00119843	0.04587904	0.00646456	0.93591714	0.99479683	Atp6v1g3
ENSMUSG00000026395.17	399.706714	0.06692184	0.00500335	159.292457	1.62E-36	7.19E-34	Ptprc
ENSMUSG00000026398.15	1066.05585	0.00164058	0.00400569	0.16622568	0.68348818	0.83805116	Nr5a2
ENSMUSG00000026399.13	362.649802	0.00460775	0.00313081	2.1171229	0.14565995	0.28123244	Cd55
ENSMUSG00000026404.13	77.0231705	0.00664679	0.00278913	5.67714417	0.01718732	0.05025166	Ddx59
ENSMUSG00000026405.15	10860.7035	-0.0027417	0.00522384	0.26697222	0.6053701	0.77814918	C4bp
ENSMUSG00000026407.18	5.12912717	0.09339542	0.0123799	44.635997	2.37E-11	4.15E-10	Cacna1s
ENSMUSG00000026409.15	275.205049	-0.0052809	0.00445093	1.42691242	0.23226899	0.40008008	Pfkfb2
ENSMUSG00000026411.18	350.59368	-0.0055255	0.00342453	2.7024407	0.10019476	0.21051073	Tmem9
ENSMUSG00000026413.13	8.3902869	0.03307024	0.01222051	6.99920301	0.0081546	0.02683986	Pkp1
ENSMUSG00000026414.15	4019.10981	-0.0064125	0.01129319	-2.6875498	1	1	Tnnt2
ENSMUSG00000026415.12	2.09095018	0.00453834	0.0111523	0.15096815	0.69761188	0.84829658	Fcamr
ENSMUSG00000026417.14	25190.5885	0.01226794	0.00565665	4.48773412	0.0341389	0.0884738	Pigr
ENSMUSG00000026421.15	308.304155	0.03267831	0.00492224	41.8671566	9.77E-11	1.57E-09	Csrp1
ENSMUSG00000026424.9	3.04178537	0.01860006	0.01603697	1.49426325	0.22155617	0.38665363	Gpr37l1
ENSMUSG00000026425.16	364.288086	0.01581822	0.00342829	21.3418987	3.84E-06	2.98E-05	Srgap2
ENSMUSG00000026426.11	294.850714	0.01631276	0.00361327	19.9368265	8.00E-06	5.78E-05	Arl8a
ENSMUSG00000026427.17	519.067611	-0.008243	0.00196789	17.7742289	2.49E-05	0.00016264	Eif2d
ENSMUSG00000026429.10	14.8870356	-0.0135073	0.00835656	2.52068909	0.11236147	0.22996857	Ube2t
ENSMUSG00000026430.17	193.180139	0.02086867	0.0033006	39.3940866	3.46E-10	5.12E-09	Rassf5
ENSMUSG00000026433.14	198.246955	-0.0044098	0.00288712	2.3528316	0.12505567	0.25013255	Rab29
ENSMUSG00000026434.13	2547.99651	-0.0044792	0.00427832	1.07910159	0.29889862	0.48279845	Nucks1
ENSMUSG00000026435.16	554.367217	-0.01602	0.00437397	13.2967941	0.00026586	0.00137182	Slc45a3
ENSMUSG00000026436.16	760.359278	-0.0017974	0.00274632	0.42514858	0.51437912	0.70103697	Elk4
ENSMUSG00000026437.13	586.102349	-0.0078382	0.00391459	3.9883982	0.0458146	0.11238514	Cdk18
ENSMUSG00000026439.15	380.594876	-0.0020257	0.00206644	0.95580605	0.32824581	0.51527492	Rbbp5
ENSMUSG00000026442.15	59.232142	0.03907746	0.01034956	12.0063826	0.00053019	0.0025232	Nfasc
ENSMUSG00000026443.4	9.74491271	0.02149305	0.0172241	1.99311935	0.15801511	0.29882491	Lrrn2
ENSMUSG00000026447.18	158.417627	0.00834596	0.00272563	9.36304862	0.00221404	0.00882695	Pik3c2b
ENSMUSG00000026450.15	0.32309609	0.06486341	0.03794909	3.72739712	0.05352674	0.12754918	Chit1
ENSMUSG00000026452.16	5.1928586	0.00197961	0.01775173	0.03196837	0.85809709	0.95220315	Syt2

ENSMUSG00000026455.15	290.856148	-0.0073759	0.00161667	20.7759508	5.16E-06	3.89E-05	Klhl12
ENSMUSG00000026456.19	157.107459	-0.0007817	0.00375681	0.04296399	0.83579309	0.93793868	Cyb5r1
ENSMUSG00000026457.15	2135.85442	-0.0024224	0.0018193	1.7653941	0.18395312	0.33619599	Adipor1
ENSMUSG00000026458.14	36.5356114	0.04494693	0.00636186	47.7719579	4.79E-12	9.21E-11	Ppfia4
ENSMUSG00000026463.18	755.890473	0.02085621	0.00800092	6.38766838	0.01149159	0.03581108	Atp2b4
ENSMUSG00000026466.17	1169.59815	0.00054946	0.00290338	0.0355819	0.85038153	0.94784992	Tor1aip1
ENSMUSG00000026468.15	2.75136048	-0.0109724	0.03114187	0.14856274	0.69991265	0.85014625	Lhx4
ENSMUSG00000026469.15	658.851054	0.00527294	0.00300222	2.97331499	0.08464823	0.18441836	Xpr1
ENSMUSG00000026470.15	200.423459	0.00328504	0.00324192	1.0165729	0.31333331	0.49876355	Stx6
ENSMUSG00000026471.15	173.612544	0.02379636	0.00382913	38.0175465	7.01E-10	9.94E-09	Mr1
ENSMUSG00000026473.17	14838.8959	0.00086429	0.00326581	0.06977941	0.79165823	0.91114288	Glul
ENSMUSG00000026475.8	1252.61232	0.01138412	0.01008763	1.25968112	0.26171146	0.43617344	Rgs16
ENSMUSG00000026478.15	378.522544	0.00021394	0.00414897	0.00257145	0.95955703	1	Lamc1
ENSMUSG00000026479.14	30.7051684	0.0293345	0.00793407	12.6278193	0.00038005	0.00187567	Lamc2
ENSMUSG00000026480.13	112.748207	0.05487569	0.00553875	91.0861668	1.38E-21	8.65E-20	Ncf2
ENSMUSG00000026482.14	366.386777	0.02517798	0.00335914	54.0397463	1.96E-13	4.54E-12	Rgl1
ENSMUSG00000026483.14	157.48166	0.04327398	0.0051334	65.9756405	4.57E-16	1.47E-14	Niban1
ENSMUSG00000026484.14	358.077264	-0.0043826	0.00240483	3.29381102	0.06954144	0.15737904	Rnf2
ENSMUSG00000026489.14	6190.08056	-0.0147514	0.00549032	7.11380745	0.00764925	0.02542133	Coq8a
ENSMUSG00000026490.19	614.445563	-0.0058531	0.00324551	3.25123438	0.07136969	0.16076072	Cdc42bpa
ENSMUSG00000026491.14	2692.63674	-0.0098693	0.00370259	7.11650861	0.00763773	0.02540095	Ahctf1
ENSMUSG00000026492.12	588.568984	-0.0107503	0.00255886	17.3477744	3.11E-05	0.00019885	Tfb2m
ENSMUSG00000026494.13	21.1867502	-0.004068	0.00687084	0.330323	0.56546896	0.74571713	Kif26b
ENSMUSG00000026495.9	153.653081	0.0079021	0.0046171	2.82963957	0.09253913	0.19777648	Efcab2
ENSMUSG00000026496.12	672.315576	-0.0057301	0.00280773	4.1129351	0.04255645	0.10581239	Parp1
ENSMUSG00000026499.6	478.331606	0.00268746	0.00301318	0.78156113	0.37666412	0.5677603	Acbd3
ENSMUSG00000026500.7	351.146965	-0.0031011	0.00328184	0.89608288	0.34383421	0.5319214	Cox20
ENSMUSG00000026502.14	465.43765	-0.0073215	0.0037174	3.75939561	0.05251156	0.12549778	Desi2
ENSMUSG00000026504.18	143.244776	0.00686375	0.00281055	5.86818491	0.01541694	0.04591211	Sdccag8
ENSMUSG00000026509.16	899.582427	-0.0033924	0.00226499	2.21512861	0.13666367	0.26780902	Capn2
ENSMUSG00000026510.11	333.744118	0.00189006	0.0027608	0.47253768	0.49182203	0.67952166	Trp53bp2
ENSMUSG00000026511.8	1655.49723	-0.0042904	0.00312306	1.90202989	0.16785129	0.31305871	Srp9
ENSMUSG00000026514.15	4.76070065	0.02042932	0.03030648	0.46461763	0.49547379	0.68272739	Cnih3
ENSMUSG00000026516.9	391.321411	-0.0005588	0.00258827	0.04648018	0.82930531	0.93365132	Nvl
ENSMUSG00000026519.17	284.133326	0.000883	0.0037571	0.05375077	0.81666065	0.92555658	Tmem63a
ENSMUSG00000026520.9	99.1243575	0.00888904	0.00391573	5.19401686	0.02266477	0.06314332	Pycr2
ENSMUSG00000026525.10	11.1891552	-0.013845	0.00485227	7.95911066	0.00478459	0.01702102	Opn3
ENSMUSG00000026526.15	3669.22793	-0.0138567	0.00354865	15.0094621	0.00010697	0.00060709	Fh1
ENSMUSG00000026527.14	19.0821448	-0.0177549	0.00946912	3.22600674	0.07247722	0.16282005	Rgs7
ENSMUSG00000026531.5	2.78867393	-0.0064507	0.03035122	-0.0052583	1	1	Mptx1
ENSMUSG00000026532.8	13.6960104	0.04388534	0.01485487	6.35411023	0.01171097	0.0363632	Spta1
ENSMUSG00000026535.10	1.46108524	-0.0163906	0.1520324	0.02959506	0.86341225	0.95486666	Ifi202b
ENSMUSG00000026536.10	112.08713	0.05309275	0.00703158	53.5249274	2.55E-13	5.81E-12	Ifi211
ENSMUSG00000026542.7	2779.46222	0.01824758	0.00725161	5.86347141	0.01545828	0.04601779	Apcs
ENSMUSG00000026544.7	203.625431	0.00275844	0.00349776	0.61811623	0.43174817	0.6232839	Dusp23
ENSMUSG00000026546.17	9.20167027	0.00334824	0.01062313	0.08982973	0.76439373	0.89301781	Cfap45
ENSMUSG00000026547.16	410.176838	0.01877997	0.00503777	13.2446616	0.00027336	0.0014062	Tagln2
ENSMUSG00000026548.5	27.751334	0.07790795	0.00755928	96.9245524	7.20E-23	5.21E-21	Slamf9
ENSMUSG00000026553.18	3536.47008	0.00038549	0.00160162	0.05766249	0.81022936	0.92198917	Copa
ENSMUSG00000026554.16	2532.31331	-0.006721	0.00157371	18.2662554	1.92E-05	0.00012885	Dcaf8
ENSMUSG00000026556.17	20.5260876	0.00352923	0.00719046	0.23275007	0.62949215	0.79802214	Vangl2
ENSMUSG00000026558.14	205.720961	-0.00684	0.00424195	2.59229902	0.10738436	0.22193101	Uck2
ENSMUSG00000026563.14	274.503702	0.00246474	0.00232855	1.08515643	0.29754688	0.48104331	Tada1
ENSMUSG00000026564.10	15.7847103	0.03263336	0.03428271	-0.9223348	1	1	Styxl2
ENSMUSG00000026565.19	251.897599	-0.0032426	0.00600773	0.27213698	0.60190186	0.77533901	Pou2f1
ENSMUSG00000026566.16	104.570717	0.00267204	0.00504581	0.27627284	0.5991546	0.77340523	Mpzl1
ENSMUSG00000026567.17	70.5831496	-0.0118441	0.01558909	0.49334631	0.48243838	0.67163213	Adcy10
ENSMUSG00000026568.7	4177.16894	-0.0127201	0.00396801	10.424465	0.00124357	0.00532513	Mpc2
ENSMUSG00000026571.13	736.993851	0.00160879	0.00281373	0.32964824	0.56586629	0.74594916	Dcaf6
ENSMUSG00000026573.8	5.79962804	0.06919757	0.00918558	51.4486777	7.35E-13	1.57E-11	Xcl1
ENSMUSG00000026574.6	164.14304	0.00171248	0.00643984	0.06882105	0.79306113	0.91191205	Dpt

ENSMUSG00000026575.16	107.903407	-0.0037034	0.00307275	1.51124575	0.21894909	0.38343728	Nme7
ENSMUSG00000026576.13	1389.70155	0.0027622	0.00394471	0.50526824	0.47719441	0.66728444	Atp1b1
ENSMUSG00000026577.14	199.388116	-0.0048614	0.00259822	3.49924822	0.06139669	0.1425438	Blzf1
ENSMUSG00000026578.7	110.077331	0.01053841	0.00325625	10.445978	0.00122917	0.00527112	Ccdc181
ENSMUSG00000026579.9	9585.18354	0.00232637	0.00500637	0.21406237	0.64360182	0.80929747	F5
ENSMUSG00000026580.17	35.2964291	0.02200526	0.00572522	14.1789571	0.00016622	0.00090437	Selp
ENSMUSG00000026581.15	55.9434053	0.05463317	0.00695231	52.8902492	3.53E-13	7.87E-12	Sell
ENSMUSG00000026582.8	4.98854188	0.02994075	0.01119807	6.43412589	0.01119482	0.03508386	Sele
ENSMUSG00000026584.15	425.488617	-0.0099213	0.00249881	15.5470427	8.05E-05	0.00047019	Scyl3
ENSMUSG00000026585.14	166.981618	0.00659822	0.00284485	5.29809911	0.02134871	0.0600671	Kifap3
ENSMUSG00000026586.17	56.0082538	0.03245953	0.0081917	13.1614285	0.00028577	0.0014624	Prrx1
ENSMUSG00000026587.16	12.4174308	0.06797239	0.01169354	26.9206998	2.12E-07	2.08E-06	Astn1
ENSMUSG00000026589.15	1543.55865	-0.0025578	0.0037279	0.45662103	0.49920739	0.68642835	Sec16b
ENSMUSG00000026592.14	6.30021967	-0.0452341	0.02623889	0.59333408	0.44113274	0.63308268	Tex35
ENSMUSG00000026594.15	459.849877	0.00449384	0.00343101	1.67615541	0.19543609	0.35182077	Ralgps2
ENSMUSG00000026596.14	211.175057	0.00188665	0.00382502	0.23471819	0.62804721	0.79666143	Abl2
ENSMUSG00000026600.13	193.891691	0.04278441	0.00558939	53.8106411	2.21E-13	5.07E-12	Soat1
ENSMUSG00000026601.16	3.80170863	-0.0016979	0.0161169	0.00210802	0.96337942	1	Axdnd1
ENSMUSG00000026602.10	2.84340231	0.03388495	0.03908928	1.60720069	0.20488575	0.3650427	Nphs2
ENSMUSG00000026603.14	291.058417	-0.0166957	0.00300736	30.9608499	2.63E-08	2.98E-07	Smyd2
ENSMUSG00000026604.18	77.5161759	0.00832773	0.00547738	2.26265735	0.13252648	0.26178975	Ptpn14
ENSMUSG00000026605.15	52.9887854	0.03364115	0.01231974	5.84466813	0.01562432	0.04640081	Cenpf
ENSMUSG00000026608.14	247.258615	-0.0010293	0.00277998	0.13523088	0.71306896	0.85846738	Kctd3
ENSMUSG00000026609.16	11.812563	0.05426788	0.01221858	17.2295427	3.31E-05	0.00021087	Ush2a
ENSMUSG00000026610.14	84.7734069	0.01297002	0.00803724	2.54700241	0.11050373	0.22704993	Esrrg
ENSMUSG00000026611.18	5.9271632	-0.0297193	0.00829866	11.9862206	0.00053595	0.00254705	Spata17
ENSMUSG00000026614.8	887.086105	-0.0168286	0.00555541	8.85291472	0.00292621	0.01118782	Slc30a10
ENSMUSG00000026615.15	2535.95003	-0.0040696	0.00253512	2.56913383	0.10896738	0.2246006	Eprs
ENSMUSG00000026616.17	30.1908241	0.02474087	0.01134912	4.30924027	0.03790589	0.09621699	Cr2
ENSMUSG00000026617.17	1104.8283	-0.009471	0.00259403	13.0212511	0.00030798	0.00156046	Bpnt1
ENSMUSG00000026618.12	1147.28238	0.00185369	0.00192092	0.93126467	0.33453452	0.5214512	Iars2
ENSMUSG00000026620.12	24.8743268	0.02462989	0.00462036	27.9460416	1.25E-07	1.28E-06	Mark1
ENSMUSG00000026621.15	7538.66905	-0.0109371	0.00522861	4.36944076	0.036589	0.09356913	Mtarc1
ENSMUSG00000026622.16	61.7860904	-0.0150468	0.00606702	5.98393135	0.01443679	0.04353741	Nek2
ENSMUSG00000026623.17	4247.93404	0.00071748	0.00304532	0.05392364	0.81637131	0.92536206	Lpgat1
ENSMUSG00000026626.12	3515.42556	-0.0034968	0.00276747	1.56532929	0.21088661	0.37297328	Ppp2r5a
ENSMUSG00000026627.8	45.7849019	0.01932183	0.00427439	19.8388492	8.43E-06	6.06E-05	Pacc1
ENSMUSG00000026628.14	70.6532996	0.0499429	0.00878349	27.264797	1.77E-07	1.76E-06	Atf3
ENSMUSG00000026630.10	17.7739064	0.05768659	0.00752214	53.4362748	2.67E-13	6.07E-12	Batf3
ENSMUSG00000026632.18	86.0533211	-0.0010633	0.00350918	0.09422493	0.75887321	0.88946894	Tatdn3
ENSMUSG00000026634.17	407.301634	0.00513102	0.00205008	6.17618162	0.01294814	0.03956197	Angel2
ENSMUSG00000026637.15	89.4580524	0.00775986	0.00360516	4.52800352	0.0333445	0.08672952	Traf5
ENSMUSG00000026638.16	902.869817	-0.0200647	0.00442718	19.8474543	8.39E-06	6.04E-05	Irf6
ENSMUSG00000026639.19	80.5662937	-0.0121021	0.00671718	3.00224111	0.08314942	0.18162274	Lamb3
ENSMUSG00000026640.13	468.331321	0.01028361	0.00413054	6.19894227	0.01278267	0.03914972	Plxna2
ENSMUSG00000026641.14	454.752457	0.00375542	0.0036382	1.05602684	0.30412312	0.48869383	Usf1
ENSMUSG00000026643.17	634.208569	-0.006259	0.00373714	2.75157559	0.09715864	0.20557852	Nmt2
ENSMUSG00000026646.17	19.0462934	0.00286491	0.00493414	0.32408803	0.56916118	0.74886956	Suv39h2
ENSMUSG00000026648.19	135.644342	-0.0094692	0.00366919	6.4071969	0.01136587	0.03552079	Dclre1c
ENSMUSG00000026649.15	28.9795049	0.00807793	0.0044398	3.3172331	0.06855712	0.15567423	Cfap126
ENSMUSG00000026650.16	23.1174554	-0.0748086	0.01066968	47.4062592	5.77E-12	1.10E-10	Meig1
ENSMUSG00000026655.16	1231.38077	5.20E-05	0.00407644	0.00015477	0.99007409	1	Fam107b
ENSMUSG00000026656.16	910.374721	0.01366434	0.0027601	24.4259514	7.72E-07	6.76E-06	Fcgr2b
ENSMUSG00000026657.18	189.997705	0.01179579	0.00407985	8.08878508	0.004454	0.0160287	Frmd4a
ENSMUSG00000026659.14	451.188308	0.0037888	0.00401253	0.88953523	0.34560329	0.53406271	Dusp12
ENSMUSG00000026662.14	604.84326	-0.0027028	0.00196959	1.88873328	0.16934486	0.3151466	Sephs1
ENSMUSG00000026663.7	1470.10936	0.00167765	0.00241802	0.47658149	0.48997486	0.67777325	Atf6
ENSMUSG00000026664.8	27639.1851	-0.0197474	0.00281267	48.8156888	2.81E-12	5.60E-11	Phyh
ENSMUSG00000026667.15	1147.42504	0.00350358	0.00316233	1.20256606	0.27280941	0.45031022	Uhmk1
ENSMUSG00000026669.15	1304.40411	-0.0196595	0.00576682	11.039549	0.00089189	0.00396607	Mcm10
ENSMUSG00000026670.16	690.34708	0.0019652	0.00236699	0.69406679	0.40478455	0.59631372	Uap1

ENSMUSG00000026672.12	1452.85414	-0.0065318	0.00252603	6.60029643	0.01019618	0.0324194	Optn
ENSMUSG00000026674.10	144.858913	0.00284643	0.00501066	0.3185875	0.57245775	0.75163575	Ddr2
ENSMUSG00000026675.13	1425.78828	0.012217	0.00372143	10.231898	0.00138033	0.00581675	Hsd17b7
ENSMUSG00000026676.8	66.9151393	0.05825317	0.00513822	120.551143	4.79E-28	7.20E-26	Ccdc3
ENSMUSG00000026678.11	1021.95856	-0.0022769	0.00416717	0.27952743	0.59701112	0.77182111	Rgs5
ENSMUSG00000026679.4	6.19576033	0.01839588	0.01398197	1.50361839	0.22011545	0.38485123	Enkur
ENSMUSG00000026683.15	18.5546571	0.03515107	0.01060654	10.2321487	0.00138014	0.00581675	Nuf2
ENSMUSG00000026687.15	4952.01844	-0.0045859	0.00347277	1.72819934	0.18864102	0.34259545	Aldh9a1
ENSMUSG00000026688.6	414.337107	0.02634203	0.00557207	21.6134825	3.33E-06	2.62E-05	Mgst3
ENSMUSG00000026691.11	974.375007	0.13839794	0.01732352	31.6299156	1.87E-08	2.16E-07	Fmo3
ENSMUSG00000026692.13	199.855855	0.02278915	0.004143	31.8760724	1.64E-08	1.92E-07	Fmo4
ENSMUSG00000026694.20	166.00452	-0.0033592	0.00303851	1.22210614	0.26894772	0.44533845	Mettl13
ENSMUSG00000026696.16	289.51075	0.00655515	0.0028585	5.14408952	0.02332564	0.06464088	Vamp4
ENSMUSG00000026697.11	2.82779074	0.0167918	0.01394656	1.38648064	0.23900033	0.40843216	Myoc
ENSMUSG00000026698.9	268.251051	-0.0064399	0.00299089	4.68994435	0.03033963	0.08025014	Pigc
ENSMUSG00000026700.5	0.94572745	0.11137517	0.02390333	22.5985456	2.00E-06	1.63E-05	Tnfsf4
ENSMUSG00000026701.16	8283.2099	-0.0054566	0.00247095	4.89411052	0.02694845	0.07283215	Prdx6
ENSMUSG00000026705.17	191.611018	-0.0004723	0.00203363	0.05416541	0.81596748	0.92521556	Klhl20
ENSMUSG00000026707.16	62.5436223	-0.0039276	0.00444706	0.75158029	0.38597636	0.57760539	Nsun6
ENSMUSG00000026708.17	123.396316	-0.0008334	0.0031415	0.07091118	0.7900147	0.91023416	Cenpl
ENSMUSG00000026709.11	284.834562	-0.0110079	0.00260698	18.01645	2.19E-05	0.00014522	Dars2
ENSMUSG00000026712.4	1080.72428	0.01151529	0.00385521	8.57477894	0.00340851	0.01274464	Mrc1
ENSMUSG00000026715.13	37314.36	-0.0027133	0.00521093	0.26812211	0.60459426	0.77761808	Serpinc1
ENSMUSG00000026718.18	331.252298	0.00441709	0.00285578	2.33984137	0.12610239	0.25175648	Stam
ENSMUSG00000026721.17	752.638679	0.00575537	0.00311332	3.32295132	0.06831908	0.15526825	Rabgap1l
ENSMUSG00000026723.11	53.7604083	0.02336252	0.00553336	17.8470554	2.39E-05	0.0001571	Trdmt1
ENSMUSG00000026726.11	16.3717163	0.06170686	0.0174237	7.99582008	0.00468855	0.01672222	Cubn
ENSMUSG00000026727.11	419.362412	0.01057889	0.00268019	15.4755177	8.36E-05	0.00048651	Rsu1
ENSMUSG00000026728.10	683.14028	0.03561491	0.0055892	37.91126	7.40E-10	1.04E-08	Vim
ENSMUSG00000026730.13	1361.73196	0.00526252	0.00347834	2.24912507	0.13368997	0.2633602	Pter
ENSMUSG00000026737.13	144.517854	0.04032524	0.00423524	84.5995449	3.65E-20	1.93E-18	Pip4k2a
ENSMUSG00000026739.14	510.815191	0.00221433	0.00283726	0.59623388	0.44001859	0.63201257	Bmi1
ENSMUSG00000026740.13	634.627602	-0.0039446	0.00339099	1.32691184	0.24935535	0.42084402	Dnajc1
ENSMUSG00000026743.17	645.565468	-0.0020933	0.00390028	0.28260641	0.59499795	0.77004703	Mllt10
ENSMUSG00000026748.14	37.1883203	0.04280787	0.00697297	35.7108514	2.29E-09	3.02E-08	Plxdc2
ENSMUSG00000026749.12	1066.79107	-0.0017122	0.00332699	0.26235061	0.60850984	0.78110668	Nek6
ENSMUSG00000026750.7	2184.7353	-0.012118	0.00250354	23.5195977	1.24E-06	1.05E-05	Psmb7
ENSMUSG00000026753.6	922.215637	-0.0072146	0.00199441	13.0806414	0.00029836	0.00151729	Ppp6c
ENSMUSG00000026754.17	336.434889	-0.0063945	0.00305391	4.42423859	0.03543192	0.09115371	Golga1
ENSMUSG00000026755.15	1052.44144	-0.0130884	0.0032939	15.5524885	8.02E-05	0.00046907	Arpc5l
ENSMUSG00000026761.13	464.369762	-0.0047505	0.00320352	2.15373141	0.14222387	0.27606717	Orc4
ENSMUSG00000026764.16	52.7427813	0.05912535	0.0116797	22.8465625	1.75E-06	1.45E-05	Kif5c
ENSMUSG00000026765.13	1.64376491	0.03521512	0.02096405	3.63280893	0.05665113	0.13375511	Lypd6b
ENSMUSG00000026766.17	989.547998	-0.0064385	0.00242892	6.94886584	0.00838724	0.02748129	Mmadhc
ENSMUSG00000026767.13	659.638793	-0.0127965	0.00241315	27.7354475	1.39E-07	1.41E-06	Mindy3
ENSMUSG00000026768.11	157.792315	0.00328663	0.00540196	0.35917673	0.54896381	0.73164155	Itga8
ENSMUSG00000026770.6	6.16955929	0.0448729	0.00941397	20.073151	7.45E-06	5.43E-05	Il2ra
ENSMUSG00000026771.15	268.079306	0.00406053	0.00367488	1.18460238	0.27642103	0.45502976	Spopl
ENSMUSG00000026773.20	322.755635	-0.0274673	0.00644917	18.4724356	1.72E-05	0.0001169	Pfkfb3
ENSMUSG00000026775.10	2079.85762	-0.0060336	0.00260305	5.24897179	0.02195974	0.06148415	Yme1l1
ENSMUSG00000026778.14	32.3123101	0.01606545	0.00499098	10.0405001	0.00153136	0.00638814	Prkcq
ENSMUSG00000026779.7	21.2458216	0.01718691	0.0073779	5.20975088	0.02246055	0.0626706	Mastl
ENSMUSG00000026781.18	4529.85724	-0.0064522	0.00289324	4.95386065	0.02603247	0.07081082	Acbd5
ENSMUSG00000026782.16	101.953759	0.02545678	0.00478331	27.5556995	1.53E-07	1.54E-06	Abi2
ENSMUSG00000026784.15	310.773709	-0.0060669	0.00398471	2.37968699	0.12292217	0.24678758	Pdss1
ENSMUSG00000026785.15	22.0920155	-0.0114552	0.00604886	3.58590179	0.05827178	0.13671523	Pkn3
ENSMUSG00000026786.15	70.1866434	0.057819	0.00587734	88.9899527	3.97E-21	2.33E-19	Apbb1ip
ENSMUSG00000026787.4	9.9307197	0.03075713	0.01651252	4.06084793	0.04388846	0.10849524	Gad2
ENSMUSG00000026788.14	460.721254	-0.0070716	0.00301026	5.47527241	0.01928735	0.05522028	Zbtb43
ENSMUSG00000026790.20	243.973894	-0.0022484	0.00419829	0.28235143	0.59516413	0.77012418	Odf2
ENSMUSG00000026791.15	251.098293	-0.013083	0.00357944	13.522037	0.00023578	0.0012336	Slc2a8

ENSMUSG00000026792.17	198.62695	0.00184969	0.00304215	0.37350276	0.54110113	0.72469238	Lrsam1
ENSMUSG00000026796.17	190.439224	0.02578606	0.00578319	19.9560041	7.92E-06	5.73E-05	Niban2
ENSMUSG00000026797.17	111.156345	0.01967795	0.00494474	15.7119145	7.38E-05	0.00043546	Stxbp1
ENSMUSG00000026798.11	122.181448	-0.0053677	0.00437658	1.4792458	0.22389256	0.38936226	Coq4
ENSMUSG00000026799.16	83.2779621	-0.0089068	0.00285346	9.82976848	0.0017171	0.00705935	Med27
ENSMUSG00000026803.18	223.379678	-0.0064056	0.00398756	2.4764665	0.11556161	0.23502942	Ttf1
ENSMUSG00000026805.15	3.96699745	0.02323935	0.00941586	5.69933866	0.01697131	0.04969401	Barhl1
ENSMUSG00000026806.16	105.123659	0.00208775	0.00226215	0.8591172	0.35398585	0.54267861	Ddx31
ENSMUSG00000026807.9	1.41114833	0.01433624	0.01410786	1.09219782	0.29598476	0.47897746	Ak8
ENSMUSG00000026809.16	3.74183508	0.00720923	0.0141901	0.25410127	0.61420194	0.78581865	Spaca9
ENSMUSG00000026810.13	217.906401	-0.0021606	0.00272104	0.63362261	0.42602962	0.61763689	Dpm2
ENSMUSG00000026811.19	817.061562	-0.0044917	0.00290078	2.36165999	0.12434981	0.24895293	St6galnac6
ENSMUSG00000026812.17	479.15567	-0.0007896	0.00308522	0.06552114	0.79797325	0.91515563	Tsc1
ENSMUSG00000026814.17	1222.47459	-0.0013524	0.00322455	0.17422561	0.67638336	0.83279147	Eng
ENSMUSG00000026816.15	123.688646	-0.0011684	0.00237337	0.24109238	0.62341825	0.79279322	Gtf3c5
ENSMUSG00000026817.15	129.966918	0.03087335	0.01093325	7.09235912	0.00774133	0.02567308	Ak1
ENSMUSG00000026818.6	4.50200797	-0.2642656	0.07443897	2.73589236	0.09811668	0.207104	Cel
ENSMUSG00000026819.16	3035.931	-0.0218692	0.00539804	16.1097488	5.98E-05	0.0003603	Slc25a25
ENSMUSG00000026820.6	331.341108	-0.0080028	0.00309135	6.83498279	0.00893896	0.02903253	Ptges2
ENSMUSG00000026821.17	73.5230594	0.02763407	0.00618367	18.9248331	1.36E-05	9.41E-05	Ralgds
ENSMUSG00000026822.15	1281.30513	0.07315291	0.01348586	20.0407677	7.58E-06	5.50E-05	Lcn2
ENSMUSG00000026824.12	46.8952155	0.01379283	0.01737125	0.73175735	0.39231416	0.58359307	Kcnj3
ENSMUSG00000026825.18	24.339236	0.04166553	0.01076701	13.5818116	0.00022839	0.00119919	Dnm1
ENSMUSG00000026826.14	21.4165223	-0.0015015	0.00781278	0.03616225	0.84918082	0.94703196	Nr4a2
ENSMUSG00000026827.13	1139.75113	-0.0097199	0.00463187	4.34144255	0.0371954	0.09470229	Gpd2
ENSMUSG00000026829.10	7.03671833	0.05254224	0.00774635	44.6515514	2.35E-11	4.13E-10	Gbgt1
ENSMUSG00000026830.10	5.17253474	0.03457706	0.02502238	2.55855953	0.10969849	0.22581155	Ermn
ENSMUSG00000026832.13	57.2951843	0.08423902	0.00749242	106.269198	6.44E-25	5.95E-23	Cytip
ENSMUSG00000026833.19	122.364286	0.0162468	0.0044317	13.006745	0.00031037	0.00157124	Olfm1
ENSMUSG00000026834.14	11.3960675	0.0499932	0.00876297	31.3460923	2.16E-08	2.48E-07	Acvr1c
ENSMUSG00000026835.16	1.11159763	0.06218305	0.03539673	2.9186158	0.08756295	0.18937786	Fcnb
ENSMUSG00000026836.16	209.253663	0.00100993	0.00234302	0.18608893	0.6661919	0.82535189	Acvr1
ENSMUSG00000026837.17	184.339913	0.01008838	0.0054886	3.38878279	0.06564139	0.15040002	Col5a1
ENSMUSG00000026839.18	6165.4788	0.00484544	0.00732635	0.45509582	0.49992491	0.68706265	Upp2
ENSMUSG00000026840.15	53.5947063	0.03210875	0.00569379	30.9644467	2.63E-08	2.98E-07	Lamc3
ENSMUSG00000026841.8	2.51533351	0.05079283	0.02808888	3.36339701	0.06666043	0.1522026	Fibcd1
ENSMUSG00000026842.17	1583.75383	0.00647711	0.00471141	1.8653699	0.17200631	0.31909241	Abl1
ENSMUSG00000026843.16	416.315648	-0.0015744	0.00327508	0.22559096	0.63481252	0.80248087	Fubp3
ENSMUSG00000026848.15	744.308271	-0.0009574	0.00229701	0.1718001	0.67851693	0.83394999	Tor1b
ENSMUSG00000026849.19	828.944963	-0.0005535	0.00290447	0.03601192	0.84949088	0.94724338	Tor1a
ENSMUSG00000026851.10	444.889602	-0.0015334	0.00206764	0.54682318	0.45961866	0.65112413	BC005624
ENSMUSG00000026853.16	1134.37104	-0.0063577	0.0050784	1.42916211	0.23190123	0.39962172	Crat
ENSMUSG00000026854.17	165.520181	0.01731991	0.0036286	22.8757634	1.73E-06	1.43E-05	Usp20
ENSMUSG00000026856.15	271.044974	-0.0041775	0.00301141	1.89200918	0.16897548	0.31468264	Dolpp1
ENSMUSG00000026857.10	113.944983	3.91E-05	0.00296519	0.00017551	0.98942981	1	Ntmt1
ENSMUSG00000026858.19	935.660877	-0.0062823	0.00342086	3.31765295	0.06853961	0.15564946	Miga2
ENSMUSG00000026860.17	316.076331	-0.0048169	0.00270132	3.19095014	0.07404696	0.16565225	Sh3glb2
ENSMUSG00000026864.14	14686.812	-0.0047159	0.00404013	1.3031919	0.25363101	0.42596322	Hspa5
ENSMUSG00000026866.18	2814.64882	-0.0091105	0.00460004	3.8601616	0.04944557	0.11949007	Kynu
ENSMUSG00000026867.20	881.546856	-0.0006166	0.00243073	0.06425087	0.7998992	0.91584576	Gapvd1
ENSMUSG00000026869.13	645.933331	-0.0041138	0.00217694	3.56683106	0.0589447	0.13788254	Psmd5
ENSMUSG00000026870.21	1005.6427	-0.000515	0.00399726	0.01640155	0.89809472	0.97479981	Cutal
ENSMUSG00000026872.19	646.582281	0.03414464	0.00494033	44.7065875	2.29E-11	4.03E-10	Zeb2
ENSMUSG00000026873.10	21.3644537	0.01928633	0.00616889	9.43697493	0.00212654	0.00851989	Phf19
ENSMUSG00000026874.11	15348.0193	-0.0023185	0.00536274	0.18233505	0.66937417	0.82771622	Hc
ENSMUSG00000026875.15	23.680476	0.06943947	0.00728613	79.7413936	4.27E-19	1.95E-17	Traf1
ENSMUSG00000026878.18	2755.37848	-0.00038	0.00179406	0.04431766	0.83326372	0.93605249	Rab14
ENSMUSG00000026879.15	796.102361	0.02090176	0.00492998	17.4651622	2.93E-05	0.00018857	Gsn
ENSMUSG00000026880.12	1145.49587	0.00088726	0.00305482	0.0821902	0.77435097	0.89960802	Stom
ENSMUSG00000026883.18	252.997947	0.00775117	0.00316495	6.0205186	0.01414049	0.04273675	Dab2ip
ENSMUSG00000026885.14	6.45164872	-0.017451	0.00936246	3.27993959	0.07013151	0.15844028	Ttll11

ENSMUSG00000026887.10	476.108225	-0.0145164	0.00274395	27.5013614	1.57E-07	1.58E-06	Mrrf
ENSMUSG00000026888.15	1015.33607	-0.0108765	0.00304938	12.7257779	0.00036065	0.00179231	Grb14
ENSMUSG00000026889.13	303.002052	-0.0088612	0.00213372	17.1563168	3.44E-05	0.00021857	Rbm18
ENSMUSG00000026890.20	112.053525	-0.0022031	0.00503955	0.18377219	0.66815133	0.82668086	Lhx6
ENSMUSG00000026893.5	140.710417	0.0041178	0.00396425	1.0594282	0.30334563	0.4880097	Gca
ENSMUSG00000026895.8	1773.71551	-0.0095763	0.00319695	9.0962919	0.00256128	0.00998189	Ndufa8
ENSMUSG00000026896.15	751.313348	0.01711384	0.00350086	23.8419321	1.05E-06	8.97E-06	Ifih1
ENSMUSG00000026904.18	33.3976978	0.03055966	0.0148762	4.24365032	0.03939737	0.09931877	Slc4a10
ENSMUSG00000026914.16	1255.01309	-0.007489	0.00245717	9.09280208	0.00256617	0.00999628	Psmd14
ENSMUSG00000026915.17	895.822209	-0.0034815	0.00285761	1.44456796	0.22940157	0.39650194	Strbp
ENSMUSG00000026917.16	495.942016	-0.0061685	0.00115127	28.6516852	8.66E-08	9.09E-07	Wdr5
ENSMUSG00000026918.17	1033.24785	-0.0102786	0.00383152	7.20998797	0.0072499	0.02434105	Brd3
ENSMUSG00000026921.21	439.334153	-0.0056847	0.00407027	1.96100178	0.16140622	0.30362175	Egfl7
ENSMUSG00000026922.14	3418.27072	-0.0108675	0.00311557	12.096731	0.0005051	0.00241699	Agpat2
ENSMUSG00000026923.16	450.156322	0.01315104	0.00454572	8.05777634	0.00453088	0.0162508	Notch1
ENSMUSG00000026924.18	1845.37233	-0.0019354	0.00248146	0.60832951	0.43541747	0.62696926	Sec16a
ENSMUSG00000026925.17	188.182312	-0.0007837	0.00496727	0.02537208	0.87344342	0.96072285	Inpp5e
ENSMUSG00000026926.15	1164.78267	-0.0054171	0.00169284	10.2609179	0.00135878	0.00574165	Pmpca
ENSMUSG00000026927.18	608.311205	0.0018002	0.00185693	0.93551837	0.33343307	0.52030906	Entr1
ENSMUSG00000026928.15	15.3648156	0.06483761	0.00854067	52.2975334	4.77E-13	1.05E-11	Card9
ENSMUSG00000026930.16	46.3881857	0.0127909	0.00576381	4.64466886	0.03115015	0.0820169	Gpsm1
ENSMUSG00000026932.15	550.248865	0.00013394	0.00311431	0.00183373	0.9658434	1	Nacc2
ENSMUSG00000026933.19	262.057419	-0.0003861	0.00458495	0.00710955	0.93280348	0.99323294	Camsap1
ENSMUSG00000026938.11	848.846296	0.01221228	0.00471561	6.31008603	0.0120053	0.03714997	Fcna
ENSMUSG00000026939.13	256.000767	0.00227007	0.00448063	0.25246792	0.61534268	0.78685131	Tmem141
ENSMUSG00000026940.4	1.99245798	-0.0123557	0.01466951	0.76905784	0.38050867	0.57199985	Ccdc183
ENSMUSG00000026941.17	32.3545657	-0.0116388	0.00897933	1.6544373	0.19835607	0.35593626	Mamdc4
ENSMUSG00000026942.14	206.615424	0.00186405	0.00264954	0.49199132	0.48304037	0.67235114	Traf2
ENSMUSG00000026944.19	872.625837	-0.0196751	0.00386057	25.5134909	4.39E-07	4.05E-06	Abca2
ENSMUSG00000026946.10	349.394942	0.01045005	0.00310705	11.3575519	0.00075142	0.00341579	Nmi
ENSMUSG00000026950.19	386.008726	-0.002483	0.00639316	0.14209653	0.70620561	0.85422129	Neb
ENSMUSG00000026955.14	2.5024224	0.02634633	0.01675137	2.25338809	0.13332221	0.26289398	Sapcd2
ENSMUSG00000026956.16	153.378718	0.0404209	0.00700288	31.6603386	1.84E-08	2.13E-07	Uap1l1
ENSMUSG00000026958.14	361.228775	-0.0081142	0.0035891	5.0320508	0.02488242	0.06817806	Dpp7
ENSMUSG00000026959.14	12.3605787	0.02599056	0.01720623	3.43485191	0.06383431	0.14705958	Grin1
ENSMUSG00000026960.7	188.626396	-0.0069386	0.00248666	7.79994489	0.00522478	0.01836296	Arl6ip6
ENSMUSG00000026961.7	0.43866353	0.00147937	0.02977496	-0.0014244	1	1	Lrrc26
ENSMUSG00000026965.13	643.326428	0.00169187	0.00236525	0.51157259	0.47445889	0.66518691	Anapc2
ENSMUSG00000026966.7	312.13179	-0.0052887	0.0034054	2.43447297	0.11869378	0.24017395	Ssna1
ENSMUSG00000026969.4	23.1969239	0.0080792	0.01453188	0.55602883	0.45586473	0.64759534	Fam166a
ENSMUSG00000026970.17	908.065243	0.00918692	0.00313341	8.46827698	0.00361394	0.01339382	Rbms1
ENSMUSG00000026971.16	22.9020931	0.02256355	0.0123846	2.76477711	0.09636011	0.20425537	Itgb6
ENSMUSG00000026972.16	64.7699159	0.02546774	0.00543219	21.1535786	4.24E-06	3.26E-05	Arrdc1
ENSMUSG00000026974.12	162.48022	-0.0134521	0.00283109	22.4612588	2.14E-06	1.75E-05	Zmynd19
ENSMUSG00000026975.11	65.8627321	0.00280705	0.00364797	0.59722263	0.43963968	0.63154526	Dph7
ENSMUSG00000026976.16	7.76059585	0.10370036	0.01201323	60.2154847	8.50E-15	2.36E-13	Pax8
ENSMUSG00000026977.18	635.362166	0.00395757	0.00375543	1.09490274	0.29538747	0.47827341	Marchf7
ENSMUSG00000026979.17	104.821956	0.05636871	0.00613287	78.2135402	9.25E-19	4.12E-17	Psd4
ENSMUSG00000026980.16	71.5295737	0.0165505	0.00415776	15.5931301	7.85E-05	0.00046	Ly75
ENSMUSG00000026981.16	96.2560017	0.06629405	0.00792006	56.8500315	4.70E-14	1.18E-12	Il1rn
ENSMUSG00000026986.15	603.214886	0.00871814	0.00281024	9.49792809	0.00205704	0.00827941	Hnmt
ENSMUSG00000026987.17	870.603121	-0.0030196	0.00411121	0.52696635	0.46788507	0.65896645	Baz2b
ENSMUSG00000026988.16	84.2413895	-0.0029725	0.00368365	0.64024302	0.42362281	0.61539588	Wdsub1
ENSMUSG00000026991.21	2269.57989	0.00068342	0.00340093	0.03911053	0.84322985	0.94257911	Pkp4
ENSMUSG00000026994.10	16.5913105	0.03824069	0.00918179	15.1033496	0.00010178	0.00058099	Galnt3
ENSMUSG00000026999.15	113.179055	-0.0072384	0.00341752	4.42172154	0.03548422	0.0912584	Nup35
ENSMUSG00000027001.11	155.928661	-0.0064359	0.00361922	3.10205569	0.0781935	0.17305732	Dusp19
ENSMUSG00000027002.14	2370.85795	-0.0047565	0.00282874	2.76003035	0.0966464	0.20468635	Nckap1
ENSMUSG00000027004.4	50.1947091	0.03966466	0.00564702	46.1013286	1.12E-11	2.06E-10	Frzb
ENSMUSG00000027006.14	649.915102	0.01223154	0.00258692	22.3724264	2.25E-06	1.82E-05	Dnajc10
ENSMUSG00000027007.17	1003.91387	0.00398768	0.00227677	2.99527855	0.08350755	0.18228682	Itprid2

ENSMUSG00000027009.19	134.413676	0.07139402	0.00556012	144.971852	2.18E-33	7.04E-31	Itga4
ENSMUSG00000027010.17	362.927643	-0.0061108	0.00493871	1.53799066	0.21491714	0.3779196	Slc25a12
ENSMUSG00000027011.15	510.940037	-0.0026228	0.00195353	1.80922443	0.17860129	0.32844079	Ube2e3
ENSMUSG00000027012.16	2120.87489	-0.0015658	0.00349057	0.19662527	0.65745873	0.81922019	Dync1i2
ENSMUSG00000027014.15	218.122255	0.00047385	0.00259176	0.03262823	0.85665576	0.95140947	Cwc22
ENSMUSG00000027015.5	4.88759062	-0.0005939	0.00980568	0.00349888	0.95283156	1	Cybrd1
ENSMUSG00000027016.18	272.139916	0.00140999	0.00375631	0.1437242	0.70460637	0.8531235	Zfp385b
ENSMUSG00000027018.12	203.089019	0.00792588	0.00320367	6.01768811	0.01416319	0.04279987	Hat1
ENSMUSG00000027022.15	410.38292	0.02467963	0.02230375	-2.1460735	1	1	Xirp2
ENSMUSG00000027030.16	96.0934462	0.03275575	0.0058996	28.5597739	9.09E-08	9.51E-07	Stk39
ENSMUSG00000027035.11	348.064393	0.03511413	0.00534749	41.1762695	1.39E-10	2.19E-09	Cers6
ENSMUSG00000027048.17	9871.35022	-0.0175262	0.00568052	9.24573809	0.00236044	0.00930993	Abcb11
ENSMUSG00000027067.17	1300.64623	0.00226562	0.00325984	0.47906471	0.48884627	0.67697551	Ssrp1
ENSMUSG00000027068.7	107.644826	0.03545716	0.00760586	18.813919	1.44E-05	9.92E-05	Dhrs9
ENSMUSG00000027070.15	166.241858	-0.0307617	0.00888702	9.40065951	0.00216907	0.00867115	Lrp2
ENSMUSG00000027071.15	11.7794714	-0.0271895	0.00822643	10.8857403	0.00096907	0.00427421	P2rx3
ENSMUSG00000027073.6	2.93991411	0.14855211	0.01781802	52.0811064	5.33E-13	1.16E-11	Prg2
ENSMUSG00000027074.15	600.915991	0.00573216	0.00310109	3.31779814	0.06853356	0.15564946	Slc43a3
ENSMUSG00000027075.17	155.597685	-0.0146729	0.00500293	8.05102514	0.00454779	0.01629648	Slc43a1
ENSMUSG00000027076.11	365.833241	-0.0208161	0.00413813	25.5787171	4.25E-07	3.93E-06	Timm10
ENSMUSG00000027078.15	1258.68904	0.01052519	0.00381133	7.57023837	0.00593399	0.02046187	Ube2l6
ENSMUSG00000027079.16	287.408954	-0.0141519	0.00296943	22.3762241	2.24E-06	1.82E-05	Clp1
ENSMUSG00000027080.7	309.915998	-0.0022453	0.00316931	0.4977704	0.48048143	0.67029248	Med19
ENSMUSG00000027082.16	187.42522	-0.0042324	0.0042265	1.00465801	0.31618602	0.50172012	Tfpi
ENSMUSG00000027086.17	372.762443	-0.0126699	0.00235937	29.3929599	5.91E-08	6.34E-07	Fastkd1
ENSMUSG00000027087.12	731.899592	0.01074088	0.00500029	4.5590161	0.03274588	0.08544577	Itgav
ENSMUSG00000027088.11	548.984453	-0.0159977	0.0028591	30.7881492	2.88E-08	3.24E-07	Phospho2
ENSMUSG00000027091.15	1068.90119	-0.0113691	0.00250831	20.3978803	6.29E-06	4.66E-05	Zc3h15
ENSMUSG00000027099.10	846.682779	-0.0115702	0.00275526	17.8853827	2.35E-05	0.0001544	Mtx2
ENSMUSG00000027102.5	12.2985167	0.04548142	0.01730672	6.5306732	0.01060298	0.03352305	Hoxd8
ENSMUSG00000027104.19	645.483177	0.00545759	0.00254053	4.56731411	0.03258762	0.08514585	Atf2
ENSMUSG00000027108.16	693.655977	-0.0102757	0.00198428	26.9323253	2.11E-07	2.07E-06	Ola1
ENSMUSG00000027109.17	882.558126	0.00409664	0.00231281	3.01220365	0.08263987	0.18079436	Sp3
ENSMUSG00000027111.17	103.192813	0.02806252	0.00554512	25.9506398	3.50E-07	3.29E-06	Itga6
ENSMUSG00000027115.15	15.8203511	0.02079707	0.00658454	9.50385908	0.0020504	0.00825833	Kif18a
ENSMUSG00000027122.16	230.675675	0.00800279	0.00215104	13.6408639	0.00022132	0.00116725	Arl14ep
ENSMUSG00000027130.16	321.843961	0.01193492	0.00308536	14.6598061	0.00012876	0.00071729	Slc12a6
ENSMUSG00000027131.4	746.706896	-0.0120241	0.00218689	30.3332367	3.64E-08	4.03E-07	Emc4
ENSMUSG00000027132.4	168.15731	-2.65E-05	0.0023946	0.00012107	0.99122082	1	Katnbl1
ENSMUSG00000027133.4	987.70356	-0.0152474	0.0032417	22.0034445	2.72E-06	2.17E-05	Nop10
ENSMUSG00000027134.5	26.2718576	0.03806101	0.00768486	23.4883084	1.26E-06	1.06E-05	Lpcat4
ENSMUSG00000027157.12	3.9386966	0.01410078	0.04900016	-0.0790461	1	1	Potefam1
ENSMUSG00000027160.17	139.987875	0.02255241	0.00651867	11.5241385	0.00068698	0.00316036	Ccdc34
ENSMUSG00000027162.8	1019.52296	0.00234353	0.00227849	1.03510716	0.30896215	0.49397921	Lin7c
ENSMUSG00000027163.14	259.918786	-0.0080711	0.00338588	5.58835612	0.01808026	0.05239476	Commd9
ENSMUSG00000027164.9	374.160485	0.00196113	0.00235145	0.6911527	0.40577258	0.59733817	Traf6
ENSMUSG00000027165.17	219.426238	-0.0034662	0.00342556	1.02562534	0.31118837	0.49626023	Iftap
ENSMUSG00000027166.15	121.046978	-0.0061741	0.00362868	2.86719287	0.09040242	0.19408625	Dnajc24
ENSMUSG00000027167.12	177.379689	-0.0054752	0.00313069	3.03852135	0.08130995	0.17844761	Elp4
ENSMUSG00000027168.22	2.32889609	0.01689461	0.01864237	1.23595315	0.26625241	0.44186809	Pax6
ENSMUSG00000027170.13	1679.43755	-0.0071183	0.00312907	5.16314542	0.02307108	0.06406391	Eif3m
ENSMUSG00000027171.11	10.5000966	-0.008761	0.00955316	0.81452189	0.36678672	0.55676609	Prrg4
ENSMUSG00000027173.8	580.065408	-0.003985	0.00327304	1.48133889	0.22356516	0.38894957	Depdc7
ENSMUSG00000027175.11	91.1209567	0.01101993	0.0044382	6.2343824	0.01252933	0.0384786	Tcp11l1
ENSMUSG00000027176.9	277.510166	0.00036862	0.00325531	0.01284279	0.90977207	0.98075251	Cstf3
ENSMUSG00000027177.15	1661.19155	0.0037075	0.00286514	1.64072403	0.20022616	0.35867174	Hipk3
ENSMUSG00000027180.13	2565.08553	-0.0011277	0.00210241	0.29017121	0.59011084	0.76629596	Fbxo3
ENSMUSG00000027184.15	4360.3747	-0.0025167	0.00266888	0.86261498	0.35300794	0.5417014	Caprin1
ENSMUSG00000027185.16	508.091018	-0.010158	0.00307296	10.9287196	0.00094685	0.00418288	Nat10
ENSMUSG00000027186.17	2.94229981	-0.0087177	0.01732186	0.24338364	0.62177289	0.7918112	Elf5
ENSMUSG00000027187.11	49222.9377	-0.0125867	0.00385757	10.415868	0.00124937	0.00534609	Cat

ENSMUSG00000027188.9	96.6831269	0.03551246	0.00388726	80.0505042	3.65E-19	1.70E-17	Pamr1
ENSMUSG00000027189.17	1037.62306	0.00561389	0.0031797	3.06819998	0.07983783	0.17585497	Trim44
ENSMUSG00000027193.13	1277.36116	0.00171081	0.00163577	1.07988894	0.2987224	0.48267908	Api5
ENSMUSG00000027194.17	609.374034	0.00548602	0.00283449	3.71047184	0.0540721	0.12867971	Ttc17
ENSMUSG00000027195.11	2910.90375	-0.0091791	0.00268595	11.1983401	0.00081871	0.003683	Hsd17b12
ENSMUSG00000027198.17	544.750807	-0.0009801	0.00185356	0.28099799	0.59604784	0.7709561	Ext2
ENSMUSG00000027199.15	265.095678	0.01055	0.00461724	5.09679647	0.02397007	0.0661315	Gatm
ENSMUSG00000027200.18	336.335696	0.02356246	0.00361725	41.1409771	1.42E-10	2.22E-09	Sema6d
ENSMUSG00000027201.17	224.661961	0.01027077	0.00280501	12.9963449	0.0003121	0.00157864	Myef2
ENSMUSG00000027202.13	78.2482001	0.01249428	0.03269173	0.40404701	0.52500658	0.71030504	Slc12a1
ENSMUSG00000027203.16	178.786616	-0.0037618	0.00374326	1.00897157	0.31514935	0.5005323	Dut
ENSMUSG00000027204.14	105.981439	0.0150681	0.00620091	5.7296501	0.01668081	0.04900156	Fbn1
ENSMUSG00000027206.14	1261.79052	-0.0072967	0.00251628	8.24173383	0.0040938	0.01490705	Cops2
ENSMUSG00000027207.16	260.114499	0.00659963	0.0023175	8.07150686	0.00449667	0.01615241	Galk2
ENSMUSG00000027208.15	2.22260178	0.03729614	0.01559968	5.28906611	0.02145973	0.06032047	Fgf7
ENSMUSG00000027210.21	151.03078	0.01291277	0.00440566	8.35397958	0.00384844	0.0141319	Meis2
ENSMUSG00000027215.14	1170.45295	0.00513624	0.00281041	3.35718887	0.0669122	0.152674	Cd82
ENSMUSG00000027217.14	47.0990277	-0.0066209	0.00462	2.06929605	0.15029088	0.28786427	Tspan18
ENSMUSG00000027219.14	68.2113375	0.05934809	0.00746083	55.590628	8.92E-14	2.13E-12	Slc28a2
ENSMUSG00000027220.3	6.7887192	0.04437212	0.01772164	6.72974471	0.00948182	0.03052664	Syt13
ENSMUSG00000027221.6	16.2234207	0.0570245	0.01004252	30.1908274	3.92E-08	4.32E-07	Chst1
ENSMUSG00000027222.15	1207.71316	-0.0046776	0.00353565	1.7451286	0.18649031	0.33953036	Pex16
ENSMUSG00000027223.16	36.872979	0.01531885	0.00473586	10.5637461	0.00115327	0.00497824	Mapk8ip1
ENSMUSG00000027225.8	0.55734982	0.04203974	0.03375397	1.92921434	0.16484443	0.30862205	Duoxa2
ENSMUSG00000027227.8	23862.7385	-0.008207	0.0038203	4.59536375	0.03205854	0.08399766	Sord
ENSMUSG00000027230.10	33.9727605	0.02669308	0.0059288	19.5491051	9.80E-06	6.96E-05	Creb3l1
ENSMUSG00000027236.9	1357.49637	-0.0070836	0.00335542	4.38390235	0.03627985	0.09298016	Eif3j1
ENSMUSG00000027238.18	27.0588237	0.00044271	0.01809774	0.00070674	0.97879113	1	Frmd5
ENSMUSG00000027239.15	12.3065613	0.01316784	0.00880341	2.15549911	0.14206027	0.27584042	Mdk
ENSMUSG00000027242.15	53.9005755	0.01380565	0.00314239	18.8536006	1.41E-05	9.74E-05	Wdr76
ENSMUSG00000027243.9	270.010688	-0.0083354	0.0027104	9.42810039	0.00213686	0.0085554	Harbi1
ENSMUSG00000027244.15	786.692467	-0.0106424	0.00249874	18.0735662	2.13E-05	0.00014131	Atg13
ENSMUSG00000027245.12	366.92685	-0.0095768	0.0064669	2.26517136	0.13231158	0.26147472	Hypk
ENSMUSG00000027246.13	35.164411	0.0196038	0.00589495	11.3309928	0.00076224	0.00345935	Ell3
ENSMUSG00000027247.17	263.681823	0.00752539	0.00276292	7.27664054	0.00698571	0.02359514	Arhgap1
ENSMUSG00000027248.14	8201.51732	-0.0038198	0.00257783	2.12068466	0.14532157	0.28085501	Pdia3
ENSMUSG00000027249.16	29107.3537	-0.0007448	0.00540327	-3.063231	1	1	F2
ENSMUSG00000027253.16	405.509479	0.00455587	0.00348033	1.70054306	0.19221697	0.34735133	Lrp4
ENSMUSG00000027254.14	60.7604671	0.02082901	0.00991068	3.85954332	0.04946379	0.11952187	Map1a
ENSMUSG00000027255.15	1917.11244	-0.0051156	0.00207281	6.12511161	0.01332749	0.04057074	Arfgap2
ENSMUSG00000027257.14	765.646268	-0.0008558	0.00281086	0.09369152	0.75953558	0.88997994	Pacsin3
ENSMUSG00000027259.16	231.86044	-0.0060153	0.00262994	5.24741058	0.02197946	0.06152474	Adal
ENSMUSG00000027261.3	4852.53904	-0.0359483	0.00508635	48.3164434	3.63E-12	7.13E-11	Hao1
ENSMUSG00000027263.13	449.165272	-0.0070719	0.00232424	9.15528706	0.00248	0.00970002	Tubgcp4
ENSMUSG00000027270.15	3.13356133	0.03356778	0.02608356	2.04102703	0.15310624	0.29190557	Lamp5
ENSMUSG00000027272.6	489.327409	0.00399377	0.00269862	2.15844987	0.14178766	0.27544715	Ubr1
ENSMUSG00000027273.14	200.01827	0.02677687	0.01769021	2.89892249	0.08863879	0.1911764	Snap25
ENSMUSG00000027274.17	835.924474	-0.0166837	0.00432579	14.7003195	0.00012603	0.00070403	Mkks
ENSMUSG00000027276.8	118.883887	0.00417913	0.00349837	1.39805396	0.23704967	0.40601337	Jag1
ENSMUSG00000027281.17	68.140867	0.00464903	0.00307715	2.22052129	0.13618707	0.26708059	Slx4ip
ENSMUSG00000027282.18	3231.43637	-0.0163407	0.00272254	36.7777634	1.32E-09	1.80E-08	Mtch2
ENSMUSG00000027284.17	157.848475	-0.0060231	0.00409646	2.10622588	0.14670073	0.2828252	Cdan1
ENSMUSG00000027285.14	303.019818	-0.0014363	0.00233393	0.37814302	0.53859874	0.72257627	Haus2
ENSMUSG00000027286.17	202.744057	-0.0018555	0.00221794	0.70469404	0.40121091	0.59286488	Lrrc57
ENSMUSG00000027287.15	803.275551	0.00112346	0.0025638	0.18608329	0.66619665	0.82535189	Snap23
ENSMUSG00000027288.17	1911.96904	-0.0121317	0.00359489	11.3480486	0.00075527	0.00343036	Zfp106
ENSMUSG00000027291.16	507.165617	0.01089733	0.00519899	4.37307812	0.03651098	0.09342027	Vps39
ENSMUSG00000027293.14	260.973788	0.02058587	0.00473619	17.9341515	2.29E-05	0.00015091	Ehd4
ENSMUSG00000027296.8	22.6275292	0.00266101	0.00570551	0.22689106	0.63383871	0.80159401	Itpka
ENSMUSG00000027297.16	2.73358656	0.01062481	0.00935318	1.32022883	0.25055099	0.42225763	Ltk
ENSMUSG00000027298.18	10.8515625	-0.0037912	0.00881111	0.19109867	0.66200377	0.82248888	Tyro3

ENSMUSG00000027300.11	105.912529	-0.0055281	0.00240838	5.24078749	0.02206328	0.06173009	Ubox5
ENSMUSG00000027303.19	684.869114	0.00625048	0.00219864	7.93344002	0.00485294	0.01723559	Ptpra
ENSMUSG00000027304.10	742.546489	-0.0004032	0.00365493	0.01206304	0.91254272	0.98233956	Rtf1
ENSMUSG00000027305.10	426.864475	-0.0175757	0.00249724	49.4858892	2.00E-12	4.03E-11	Ndufaf1
ENSMUSG00000027306.16	25.6939695	0.03243667	0.00927219	10.9239142	0.00094931	0.00419217	Nusap1
ENSMUSG00000027309.19	787.96361	-0.0097252	0.00289377	11.1295771	0.00084962	0.00380393	Dnaaf9
ENSMUSG00000027312.15	1439.41484	-0.0160541	0.0034607	21.3215825	3.88E-06	3.01E-05	Atrn
ENSMUSG00000027313.4	54.304492	-0.0189024	0.00643465	8.69910259	0.00318367	0.01201055	Chac1
ENSMUSG00000027314.7	92.842827	0.01298656	0.00389983	10.7092252	0.00106603	0.00464327	Dll4
ENSMUSG00000027315.14	17.8097285	0.03073131	0.00752166	16.4447453	5.01E-05	0.00030694	Spint1
ENSMUSG00000027316.16	4.80744176	0.03850064	0.01086131	11.5237041	0.00068714	0.00316048	Gfra4
ENSMUSG00000027318.18	22.6019196	0.00864215	0.00671186	1.59172715	0.20707955	0.36789531	Adam33
ENSMUSG00000027322.12	194.545361	0.01742881	0.00632826	7.48969752	0.0062053	0.02127908	Siglec1
ENSMUSG00000027323.11	19.3292805	0.01328198	0.00904634	2.08017024	0.14922353	0.28637882	Rad51
ENSMUSG00000027324.2	40.6836642	-0.0079902	0.00323861	5.95680742	0.01466056	0.04406832	Rpusd2
ENSMUSG00000027326.14	17.7749063	0.03493792	0.0104839	9.84942242	0.00169885	0.00699164	Knl1
ENSMUSG00000027327.17	71.8268642	0.0087656	0.00390143	4.8979654	0.02688836	0.07272807	Adissp
ENSMUSG00000027329.10	23.0620264	0.00704783	0.00509152	1.89000465	0.1692014	0.31497902	Spef1
ENSMUSG00000027330.17	32.2863807	0.03644469	0.00687257	26.5090361	2.62E-07	2.52E-06	Cdc25b
ENSMUSG00000027331.16	39.7828749	0.01715911	0.01070843	2.24525745	0.1340246	0.26382108	Knstrn
ENSMUSG00000027332.12	3175.65714	-0.0070394	0.00288434	5.97550096	0.01450596	0.04369775	Ivd
ENSMUSG00000027333.19	70.6807249	0.00931542	0.00563624	2.67775237	0.10175926	0.21309054	Smox
ENSMUSG00000027335.10	1.07661176	0.01194402	0.02194964	0.52106367	0.4703883	0.66129692	Adra1d
ENSMUSG00000027338.17	2.60265155	0.04055441	0.02681976	2.11905204	0.14547656	0.2810164	Prnd
ENSMUSG00000027339.16	77.3902836	0.06556984	0.00500858	158.553491	2.34E-36	1.02E-33	Rassf2
ENSMUSG00000027340.16	412.290501	0.0049909	0.00352621	1.97503709	0.15991422	0.30142546	Slc23a2
ENSMUSG00000027341.11	338.830612	-0.0030061	0.00224333	1.78385365	0.18167683	0.33271448	Tmem230
ENSMUSG00000027342.15	580.691181	0.01004111	0.00331386	8.96890961	0.00274612	0.01059373	Pcna
ENSMUSG00000027344.15	2.12913152	0.0101692	0.01246278	0.67704629	0.41060568	0.60219784	Fsip1
ENSMUSG00000027346.16	1985.75965	-0.0241764	0.00588286	16.3500605	5.27E-05	0.00032107	Gpcpd1
ENSMUSG00000027347.20	84.1580913	0.06333837	0.00618991	95.6179892	1.39E-22	9.81E-21	Rasgrp1
ENSMUSG00000027349.6	184.156293	0.00083604	0.0036638	0.05103876	0.82126551	0.9286336	Fam98b
ENSMUSG00000027350.9	17.7772807	0.04177827	0.01483545	8.14731047	0.00431251	0.01559804	Chgb
ENSMUSG00000027351.15	410.269681	0.01959368	0.00319619	36.1668629	1.81E-09	2.42E-08	Spred1
ENSMUSG00000027353.15	31.0431197	0.00467549	0.00378684	1.4919832	0.22190901	0.38709754	Mcm8
ENSMUSG00000027355.16	4.20833322	-0.2497523	0.12889521	-0.0502018	1	1	Tmco5
ENSMUSG00000027356.9	19.8450857	0.06687338	0.00760339	70.8701559	3.82E-17	1.41E-15	Fermt1
ENSMUSG00000027357.17	1233.9223	0.00959874	0.00331977	8.09985031	0.00442689	0.01595304	Crls1
ENSMUSG00000027358.7	360.136591	-0.0027695	0.00321281	0.7346369	0.3913843	0.58274037	Bmp2
ENSMUSG00000027359.17	19983.2575	-0.0145325	0.00500555	8.24151829	0.00409429	0.01490705	Slc27a2
ENSMUSG00000027360.6	60.3001286	-0.0016882	0.00459153	0.13399162	0.71432877	0.85920732	Hdc
ENSMUSG00000027361.16	188.961597	0.00087664	0.00234893	0.13602337	0.71226677	0.8580379	Gabpb1
ENSMUSG00000027363.16	1055.8513	-0.0005	0.00249596	0.03977963	0.84191207	0.94201137	Usp8
ENSMUSG00000027364.15	44.5260479	-0.0027982	0.00986866	0.07686416	0.78159287	0.90441296	Usp50
ENSMUSG00000027365.15	1028.48395	0.00318699	0.00290497	1.19299039	0.27472719	0.4527154	Trpm7
ENSMUSG00000027366.13	3609.19353	-0.0002618	0.00292335	0.00793243	0.92903089	0.99119297	Sppl2a
ENSMUSG00000027367.17	2381.40136	-0.0056664	0.00194289	8.5891068	0.0033818	0.01266885	Stard7
ENSMUSG00000027368.7	11.4028675	0.06822227	0.00814709	64.6740187	8.84E-16	2.74E-14	Dusp2
ENSMUSG00000027371.11	288.621312	-0.0107913	0.00424221	6.54376098	0.01052526	0.0333041	Fahd2a
ENSMUSG00000027374.13	855.467377	-0.0185636	0.00303295	37.8013088	7.83E-10	1.10E-08	Mrps5
ENSMUSG00000027375.15	57.4392293	0.02267181	0.00668168	11.3682947	0.00074708	0.00339972	Mal
ENSMUSG00000027376.16	4.57151866	0.02859438	0.01575489	3.67128797	0.05535739	0.13123524	Prom2
ENSMUSG00000027377.3	2.86240213	-0.0122009	0.0141291	0.64881201	0.42053774	0.61215843	Mall
ENSMUSG00000027378.17	66.8823389	0.00032382	0.0046277	0.00514096	0.94284029	0.99855551	Nphp1
ENSMUSG00000027379.15	10.2728765	0.02624203	0.01203706	4.18895547	0.04068816	0.10209265	Bub1
ENSMUSG00000027381.18	136.796024	0.01402611	0.00376003	13.4415158	0.00024612	0.00128031	Bcl2l11
ENSMUSG00000027384.7	139.909534	-0.0134672	0.0034236	15.7273498	7.32E-05	0.00043224	Ndufaf5
ENSMUSG00000027386.10	18.073856	0.03965738	0.00461309	68.7362032	1.13E-16	3.87E-15	Fbln7
ENSMUSG00000027387.12	70.5511001	-0.0078697	0.00375598	4.44872142	0.03492739	0.09014087	Zc3h8
ENSMUSG00000027394.14	112.411863	-0.0020572	0.00322937	0.40175602	0.52618376	0.71124822	Ttl
ENSMUSG00000027395.16	261.001227	-0.007418	0.00247726	9.00278705	0.00269568	0.01043477	Polr1b

ENSMUSG00000027397.15	602.597426	0.02166569	0.00415917	27.0584138	1.97E-07	1.95E-06	Slc20a1
ENSMUSG00000027398.14	79.0327416	0.07345607	0.00697724	99.0383141	2.48E-23	1.92E-21	Il1b
ENSMUSG00000027399.2	79.2394424	0.02421075	0.00557328	18.0740488	2.12E-05	0.00014131	Il1a
ENSMUSG00000027401.10	1.25983072	0.00600365	0.01451482	0.33871553	0.56057158	0.74178867	Tgm3
ENSMUSG00000027404.17	809.143424	-0.0062009	0.00280194	4.93520916	0.02631488	0.07143086	Snrpb
ENSMUSG00000027405.17	614.687523	-0.0038325	0.00415996	0.86041923	0.3536214	0.54226086	Nop56
ENSMUSG00000027406.14	3062.39133	-0.0211056	0.00338925	38.6722215	5.01E-10	7.24E-09	Idh3b
ENSMUSG00000027408.8	120.988169	0.06192865	0.00907964	37.9267296	7.35E-10	1.04E-08	Cpxm1
ENSMUSG00000027411.18	428.701338	-0.0018892	0.00354359	0.28284115	0.59484504	0.77001397	Vps16
ENSMUSG00000027412.13	81.6812825	-6.22E-05	0.00394179	0.0002472	0.98745566	1	Lpin3
ENSMUSG00000027419.10	14.9730984	0.02885613	0.01632183	3.66158491	0.05568064	0.13188227	Pcsk2
ENSMUSG00000027420.14	1.88480418	0.01456293	0.009797	2.17113708	0.14062222	0.27379195	Bfsp1
ENSMUSG00000027422.16	7042.04346	-0.0005364	0.00363178	0.0214697	0.88350662	0.96603714	Rrbp1
ENSMUSG00000027423.16	1290.25102	0.02096093	0.00304958	45.7558885	1.34E-11	2.43E-10	Snx5
ENSMUSG00000027424.12	153.235154	-0.0132332	0.00236481	30.5303254	3.29E-08	3.66E-07	Mgme1
ENSMUSG00000027425.19	358.528717	0.00086193	0.00266835	0.10523405	0.74563675	0.88017187	Kat14
ENSMUSG00000027427.14	242.665855	-0.0041237	0.00227123	3.25983319	0.07099635	0.16004204	Polr3f
ENSMUSG00000027428.10	439.279812	-0.0030114	0.00191827	2.44760985	0.11770397	0.23850222	Rbbp9
ENSMUSG00000027429.17	837.953711	-0.0012333	0.00330463	0.13942974	0.70884859	0.85602354	Sec23b
ENSMUSG00000027430.13	182.139441	0.00430761	0.00270773	2.53363757	0.11144304	0.22850522	Dtd1
ENSMUSG00000027433.6	1240.95304	0.00472425	0.00381776	1.52241793	0.21725396	0.38126129	Xrn2
ENSMUSG00000027434.12	2.70294694	-0.0087881	0.04267427	0.09962679	0.75227796	0.88503052	Nkx2-2
ENSMUSG00000027435.9	166.378361	0.01399678	0.00502026	7.52890978	0.00607166	0.02087555	Cd93
ENSMUSG00000027438.15	85.8585005	0.00823281	0.00464454	3.11798987	0.07743225	0.17171089	Napb
ENSMUSG00000027439.10	922.586298	-0.0069311	0.00240507	8.25735069	0.00405873	0.01481418	Gzf1
ENSMUSG00000027442.11	2.70411674	0.18280737	0.07869539	-0.5335779	1	1	Cst8
ENSMUSG00000027447.7	2869.25092	0.00811764	0.00351295	5.22601643	0.02225144	0.06219012	Cst3
ENSMUSG00000027452.12	95.2497979	0.05583115	0.00795086	44.1777734	3.00E-11	5.17E-10	Acss1
ENSMUSG00000027454.11	52.2758471	0.00519145	0.00381102	1.85298222	0.17343694	0.32111498	Gins1
ENSMUSG00000027455.17	907.242151	-0.0030203	0.00171679	3.09941039	0.07832066	0.17325745	Nsfl1c
ENSMUSG00000027456.9	5.32491447	0.10975273	0.01252883	65.938259	4.65E-16	1.49E-14	Sdcbp2
ENSMUSG00000027457.16	4.94014681	0.03964371	0.01465959	6.34416465	0.01177681	0.03652436	Snph
ENSMUSG00000027459.17	47.446578	0.00112858	0.00393552	0.08283246	0.77349502	0.89897976	Fam110a
ENSMUSG00000027463.15	11.462103	0.02330698	0.0063311	13.4988206	0.00023871	0.00124674	Slc52a3
ENSMUSG00000027465.14	695.7968	0.00306549	0.00204508	2.26682547	0.13217039	0.26123484	Tbc1d20
ENSMUSG00000027466.16	763.673572	-0.0049252	0.00226369	4.76860006	0.02898338	0.07720828	Rbck1
ENSMUSG00000027469.17	34.1618387	0.01846539	0.01071139	2.75050845	0.09722351	0.20567886	Tpx2
ENSMUSG00000027472.15	353.237606	-0.0087425	0.00341057	6.45991628	0.01103348	0.03467976	Pdrg1
ENSMUSG00000027474.14	64.1190133	0.00444434	0.00393251	1.30746778	0.25285364	0.42499457	Ccm2l
ENSMUSG00000027475.10	285.153693	0.00698361	0.00260573	7.15416086	0.00747903	0.02495054	Kif3b
ENSMUSG00000027478.16	52.9437186	-0.0052639	0.00605432	0.74871014	0.38688492	0.57841453	Dnmt3b
ENSMUSG00000027479.15	702.244974	0.01034591	0.00327788	9.71971354	0.00182302	0.00744678	Mapre1
ENSMUSG00000027483.8	0.89411442	-0.0101359	0.06690464	0.02002686	0.8874621	0.96843162	Bpifa1
ENSMUSG00000027487.12	153.383417	0.00351487	0.0042949	0.65148204	0.41958331	0.61096137	Cdk5rap1
ENSMUSG00000027488.13	272.121809	0.0040877	0.00258816	2.54764354	0.11045889	0.22702017	Snta1
ENSMUSG00000027489.16	2.34949119	0.05126778	0.0127439	15.8161249	6.98E-05	0.0004144	Necab3
ENSMUSG00000027490.18	68.6091628	0.00714365	0.00462366	2.29440885	0.12984064	0.25758443	E2f1
ENSMUSG00000027495.5	1924.8343	-0.009449	0.00253984	13.6822696	0.00021649	0.00114487	Fam210b
ENSMUSG00000027496.16	30.6125522	0.00629514	0.00754051	0.64495497	0.4219222	0.61356786	Aurka
ENSMUSG00000027498.15	345.190416	-0.0034863	0.00196499	3.09894015	0.07834328	0.17329125	Cstf1
ENSMUSG00000027499.13	127.587248	0.025742	0.00684128	12.9212792	0.00032487	0.00163313	Pkia
ENSMUSG00000027500.11	25.6968697	0.03827833	0.01245105	8.80250239	0.00300818	0.01145477	Stmn2
ENSMUSG00000027502.12	1301.43773	-0.0043221	0.00223866	3.65758199	0.05581458	0.13213316	Rtf2
ENSMUSG00000027505.3	2.17926262	-0.0366681	0.01202747	9.11756302	0.00253167	0.00988063	Fam209
ENSMUSG00000027506.16	389.719055	0.01790772	0.00339445	27.0576414	1.97E-07	1.95E-06	Tpd52
ENSMUSG00000027508.16	185.83321	0.0189574	0.00480625	15.0215851	0.00010629	0.00060379	Pag1
ENSMUSG00000027509.12	298.984096	-0.0051889	0.00217748	5.74529913	0.01653284	0.04862139	Rae1
ENSMUSG00000027510.18	61.078468	-0.0014345	0.00640262	0.04731249	0.82780753	0.93249935	Rbm38
ENSMUSG00000027513.12	35489.5294	-0.0087023	0.00627561	1.95398541	0.16215803	0.30455218	Pck1
ENSMUSG00000027514.15	203.87381	0.0598523	0.00576939	103.437062	2.69E-24	2.31E-22	Zbp1
ENSMUSG00000027519.11	619.664379	-0.0044922	0.00276326	2.60720205	0.1063793	0.22022841	Rab22a

ENSMUSG00000027520.17	7.49432176	0.01697394	0.01549412	1.39004252	0.23839792	0.40771904	Zdbf2
ENSMUSG00000027522.17	513.504818	0.00410836	0.00346339	1.37548695	0.24087138	0.41069851	Stx16
ENSMUSG00000027523.21	6362.5734	-0.0031949	0.00234459	1.8553664	0.17316054	0.3207538	Gnas
ENSMUSG00000027524.10	1.07388365	0.00406386	0.02459339	0.05318663	0.81760827	0.92614103	Edn3
ENSMUSG00000027525.19	6.43496355	0.04827226	0.01314688	12.0402589	0.00052064	0.0024813	Phactr3
ENSMUSG00000027528.13	5.70886579	-0.0939744	0.07966774	0.90138453	0.34241074	0.5303454	Fabp9
ENSMUSG00000027530.16	5.31419487	-0.0255349	0.01502105	2.78912448	0.09490607	0.20183313	Fabp12
ENSMUSG00000027531.17	1134.75281	-0.0035041	0.0023776	2.16542737	0.14114538	0.27453858	Impa1
ENSMUSG00000027533.11	1930.44086	-0.0266477	0.00646006	16.9099855	3.92E-05	0.00024652	Fabp5
ENSMUSG00000027534.17	89.0604875	-0.0013757	0.00295388	0.21686471	0.64143931	0.80772207	Snx16
ENSMUSG00000027536.7	73.9433495	0.01695191	0.00476743	12.4340903	0.00042157	0.00205606	Chmp4c
ENSMUSG00000027540.14	511.114673	0.02059454	0.0038038	27.3366983	1.71E-07	1.70E-06	Ptpn1
ENSMUSG00000027544.17	19.7237971	0.0557034	0.00751858	49.0505367	2.49E-12	4.99E-11	Nfatc2
ENSMUSG00000027546.16	1013.75614	0.00043506	0.00240598	0.03204562	0.85792757	0.95209865	Atp9a
ENSMUSG00000027547.18	4.60938335	0.00802795	0.01879862	0.16050617	0.68869093	0.84197928	Sall4
ENSMUSG00000027550.15	107.630967	0.02556105	0.00477516	28.1381102	1.13E-07	1.16E-06	Lrrcc1
ENSMUSG00000027551.16	143.767899	-0.0028211	0.00244108	1.33122515	0.24858738	0.4199987	Zfp64
ENSMUSG00000027552.16	115.304961	-0.0062793	0.00243921	6.71341217	0.00956905	0.0307529	E2f5
ENSMUSG00000027555.9	14.6175788	0.04464975	0.00698759	37.5907226	8.73E-10	1.22E-08	Car13
ENSMUSG00000027556.16	70.2428336	-0.0075725	0.00578959	1.689744	0.19363472	0.34908419	Car1
ENSMUSG00000027559.6	39646.2662	-0.0272688	0.00640571	16.2325896	5.60E-05	0.00034011	Car3
ENSMUSG00000027560.5	2.18841475	0.03772597	0.01829917	5.10010055	0.02392446	0.06602881	Dok5
ENSMUSG00000027562.13	151.490007	0.00069821	0.00565128	0.01543599	0.90112393	0.97642803	Car2
ENSMUSG00000027564.6	2.96035206	-0.4560061	0.18338253	-0.0235497	1	1	Cypt12
ENSMUSG00000027566.16	2565.33611	-0.0063961	0.00297425	4.63544591	0.031318	0.08239864	Psma7
ENSMUSG00000027569.16	168.097639	-0.0002974	0.00290125	0.01056294	0.91814062	0.9854052	Mrgbp
ENSMUSG00000027570.16	5.68405334	0.01074144	0.00908092	1.42530021	0.23253298	0.40041782	Col9a3
ENSMUSG00000027573.12	584.986132	0.00326627	0.00183595	3.17515922	0.07476592	0.16694396	Gid8
ENSMUSG00000027574.16	11.1484797	-0.0077438	0.00835077	0.8411013	0.35908199	0.54813978	Nkain4
ENSMUSG00000027575.20	471.81692	-0.0040176	0.00329722	1.47176347	0.22506766	0.39098814	Arfgap1
ENSMUSG00000027577.15	358.203205	0.02799657	0.00973804	7.63035353	0.00573941	0.01991022	Chrna4
ENSMUSG00000027579.7	17.7210956	-0.0279481	0.00686559	16.3973116	5.14E-05	0.00031382	Srms
ENSMUSG00000027580.18	1819.55263	0.00030781	0.00365808	0.00700548	0.93329596	0.99353306	Helz2
ENSMUSG00000027581.13	41.0003226	0.00576512	0.01672503	0.03250098	0.85693253	0.95153376	Stmn3
ENSMUSG00000027582.17	336.077725	-0.0025057	0.00270625	0.85450801	0.35528016	0.54384981	Zgpat
ENSMUSG00000027583.14	132.767456	-0.0022201	0.00250321	0.7790913	0.3774192	0.56860733	Zbtb46
ENSMUSG00000027589.15	709.666867	-0.0092062	0.0025681	12.8384097	0.00033958	0.00169939	Pcmtd2
ENSMUSG00000027593.16	1586.9037	-0.0021579	0.00346544	0.39503743	0.52966333	0.71415752	Raly
ENSMUSG00000027597.16	32091.1559	-0.0098749	0.00396709	6.07100049	0.01374191	0.0416656	Ahcy
ENSMUSG00000027598.17	1209.41234	-0.0020736	0.00322151	0.40643531	0.52378437	0.70922797	Itch
ENSMUSG00000027599.10	841.733745	-0.0056541	0.00197209	8.23701107	0.00410447	0.0149395	Armc1
ENSMUSG00000027601.14	1683.03629	-0.0134697	0.00204142	43.9459525	3.38E-11	5.78E-10	Mtfr1
ENSMUSG00000027602.10	666.872433	-0.0208226	0.00436224	22.0147017	2.71E-06	2.16E-05	Map1lc3a
ENSMUSG00000027603.16	8.55208845	0.02378911	0.00782407	8.72017744	0.00314707	0.01189724	Ggt7
ENSMUSG00000027605.19	5980.27597	0.00533793	0.00526898	0.98656046	0.32058448	0.50665409	Acss2
ENSMUSG00000027606.13	1.3009507	-0.0357598	0.06207109	0.73425524	0.39150737	0.58288546	Dnajc5b
ENSMUSG00000027610.18	1137.62468	0.0073269	0.00343485	4.55957061	0.03273528	0.08543702	Gss
ENSMUSG00000027611.13	21.3316214	0.05665795	0.00676656	68.241872	1.45E-16	4.88E-15	Procr
ENSMUSG00000027612.8	2.95907193	0.04488451	0.01433333	10.2985307	0.00133136	0.00564065	Mmp24
ENSMUSG00000027613.16	857.802508	0.00665662	0.00332195	3.99296163	0.04569069	0.11213949	Eif6
ENSMUSG00000027615.15	176.467153	0.01587392	0.00292888	28.7606325	8.19E-08	8.64E-07	Hps3
ENSMUSG00000027618.18	995.745905	-0.0095409	0.00234633	16.6955682	4.39E-05	0.0002731	Nfs1
ENSMUSG00000027620.17	4056.00788	0.00166983	0.00343988	0.23399092	0.62858028	0.79720893	Rbm39
ENSMUSG00000027624.21	575.263062	0.05572478	0.00654356	67.3861647	2.23E-16	7.40E-15	Epb41l1
ENSMUSG00000027628.11	356.237058	-0.0058704	0.00226028	6.69186502	0.00968538	0.03107597	Aar2
ENSMUSG00000027630.14	779.037881	-0.0003754	0.00406953	0.00825127	0.92762249	0.99044382	Tbl1xr1
ENSMUSG00000027634.15	397.217934	-0.010847	0.00381009	8.07630521	0.00448478	0.01611978	Ndrg3
ENSMUSG00000027635.16	17.6460461	0.01264109	0.00749026	2.7274988	0.09863365	0.20796895	Dsn1
ENSMUSG00000027636.12	15.7614295	0.04581763	0.00859904	25.1446777	5.32E-07	4.82E-06	Sla2
ENSMUSG00000027637.4	1966.42628	-0.0081424	0.00272462	8.93634127	0.00279552	0.01075965	Rab5if
ENSMUSG00000027639.17	513.310882	0.03411794	0.00372496	81.5080206	1.75E-19	8.52E-18	Samhd1

ENSMUSG00000027641.14	60.4726628	0.02964277	0.00511304	32.4598126	1.22E-08	1.46E-07	Rbl1
ENSMUSG00000027642.16	3093.64544	0.00689845	0.00311736	4.85129936	0.02762531	0.07430387	Rpn2
ENSMUSG00000027646.16	53.736941	0.02629567	0.0057872	20.3078079	6.59E-06	4.87E-05	Src
ENSMUSG00000027649.16	244.164889	-0.0022501	0.00194539	1.32673581	0.24938675	0.42086691	Ctnnbl1
ENSMUSG00000027650.13	237.645337	-0.0068655	0.00281481	5.85838611	0.015503	0.04612182	Tti1
ENSMUSG00000027651.17	499.208404	-0.0118793	0.00268818	19.2333228	1.16E-05	8.09E-05	Rprd1b
ENSMUSG00000027652.16	411.322061	0.00117483	0.00333901	0.1213459	0.72757931	0.86844199	Ralgapb
ENSMUSG00000027654.3	7.30381568	-0.0062331	0.0096591	0.41024098	0.52184722	0.70786108	Fam83d
ENSMUSG00000027655.15	99.3279633	0.00110688	0.00295912	0.13885858	0.70941838	0.85624353	Dhx35
ENSMUSG00000027656.8	7.92586938	0.02027932	0.01151362	3.02886356	0.08179529	0.1792823	Ccn5
ENSMUSG00000027660.17	393.049979	0.00855903	0.00392318	4.56307447	0.03266838	0.08532544	Skil
ENSMUSG00000027661.5	4.99130965	0.02288292	0.00864545	6.65423402	0.00989205	0.03161434	Slc2a10
ENSMUSG00000027663.14	159.510867	0.00971983	0.00335483	8.33612678	0.00388644	0.01426034	Zmat3
ENSMUSG00000027665.14	739.36539	-0.0057644	0.00331399	2.961352	0.08527661	0.18541071	Pik3ca
ENSMUSG00000027667.14	311.145285	-0.0030866	0.002573	1.46833653	0.22560833	0.39169456	Zfp639
ENSMUSG00000027668.14	1323.56393	-0.0158232	0.00472054	11.1369086	0.00084627	0.00379109	Mfn1
ENSMUSG00000027669.15	79.3384381	0.01189796	0.00477737	6.03949011	0.01398932	0.04233413	Gnb4
ENSMUSG00000027670.5	24.0691309	0.01745283	0.00561681	8.83091537	0.0029617	0.01129968	Ocstamp
ENSMUSG00000027671.15	350.657246	-0.0070331	0.00222227	10.0718422	0.00150552	0.00629597	Actl6a
ENSMUSG00000027673.13	1726.75958	-0.0153127	0.00318725	23.3614529	1.34E-06	1.13E-05	Ndufb5
ENSMUSG00000027674.17	19.9746785	0.06062981	0.01037772	31.1451332	2.39E-08	2.73E-07	Pex5l
ENSMUSG00000027676.12	7.97116746	0.02033585	0.01082284	3.56637669	0.05896083	0.1379066	Ccdc39
ENSMUSG00000027677.18	629.424354	-0.000885	0.00298708	0.08663828	0.7684952	0.89580303	Ttc14
ENSMUSG00000027678.18	540.597445	0.00115658	0.00299238	0.14624078	0.70215401	0.85133421	Ncoa3
ENSMUSG00000027679.14	990.277119	-0.0058094	0.00336577	3.0506475	0.08070496	0.17736743	Dnajc19
ENSMUSG00000027680.16	984.238815	-0.0053799	0.00272382	3.81589004	0.05076863	0.1220994	Fxr1
ENSMUSG00000027684.17	46.6465875	0.01028187	0.00524961	3.75525022	0.05264192	0.12573288	Mecom
ENSMUSG00000027690.14	5472.18606	-0.0009224	0.00564128	0.02693967	0.86962638	0.95837696	Slc2a2
ENSMUSG00000027692.17	79.5770833	0.01995936	0.00507494	14.3138439	0.00015472	0.00084729	Tnik
ENSMUSG00000027695.17	196.537245	0.01338784	0.00409169	10.2731857	0.00134978	0.00571148	Pld1
ENSMUSG00000027698.15	758.252538	-0.0060775	0.00359473	2.72657056	0.098691	0.20803699	Nceh1
ENSMUSG00000027699.20	49.7590254	0.00642066	0.00695835	0.76848604	0.38068582	0.57221191	Ect2
ENSMUSG00000027702.8	1.64068367	-0.0109942	0.02601026	0.19754476	0.65670999	0.81849301	Lrrc34
ENSMUSG00000027706.9	4040.76247	-0.0152818	0.0039566	14.473199	0.00014217	0.00078382	Sec62
ENSMUSG00000027708.15	1191.51939	-0.0038309	0.00288668	1.72576091	0.18895317	0.34296268	Dcun1d1
ENSMUSG00000027709.10	956.401014	-0.0092641	0.00281524	10.9387024	0.00094176	0.00416274	Mccc1
ENSMUSG00000027710.15	665.669182	0.01388686	0.00307277	19.9765621	7.84E-06	5.68E-05	Acad9
ENSMUSG00000027712.14	1710.81728	0.02440082	0.00355878	45.7718056	1.33E-11	2.41E-10	Anxa5
ENSMUSG00000027713.7	13.5597055	0.01269538	0.00496135	6.54730983	0.01050429	0.0332422	1810062G17Rik
ENSMUSG00000027714.12	261.774713	0.0032495	0.00269494	1.45526804	0.22768453	0.39411179	Exosc9
ENSMUSG00000027715.10	49.2268112	0.01643316	0.00868582	3.44647426	0.06338685	0.14627162	Ccna2
ENSMUSG00000027716.14	20.9767407	-0.0166841	0.00987519	2.64579325	0.10382432	0.2161342	Trpc3
ENSMUSG00000027718.9	1.46077438	0.12087749	0.02130404	26.4570413	2.69E-07	2.59E-06	Il21
ENSMUSG00000027722.15	116.69423	-0.0020884	0.00548104	0.1426467	0.70566388	0.85389728	Afg2a
ENSMUSG00000027737.11	18.7506601	0.0787125	0.0159771	18.5425349	1.66E-05	0.000113	Slc7a11
ENSMUSG00000027739.10	190.15693	0.00412961	0.00263317	2.42574845	0.11935622	0.24119382	Rab33b
ENSMUSG00000027742.14	461.190709	-0.0040341	0.00245862	2.65981001	0.10291303	0.21474888	Cog6
ENSMUSG00000027746.14	1557.09817	-0.0082334	0.00315645	6.71889995	0.00953965	0.03067095	Ufm1
ENSMUSG00000027748.12	1.70278477	0.04637034	0.02118548	4.86316782	0.02743592	0.07387169	Trpc4
ENSMUSG00000027750.17	205.702768	0.00085306	0.00515674	0.02703378	0.86940089	0.95837696	Postn
ENSMUSG00000027751.15	322.821819	0.00568474	0.00282016	4.0521405	0.04411537	0.10890208	Supt20
ENSMUSG00000027752.16	176.293302	-0.0018167	0.00354597	0.26050957	0.60977029	0.78218535	Exosc8
ENSMUSG00000027761.5	9331.78037	-0.0233328	0.00483554	23.2027492	1.46E-06	1.22E-05	Aadac
ENSMUSG00000027762.7	856.18952	0.01624215	0.00543817	8.2493928	0.00407656	0.01486087	Sucnr1
ENSMUSG00000027763.14	2152.17992	0.01013964	0.00269316	13.5805019	0.00022855	0.00119976	Mbnl1
ENSMUSG00000027765.7	175.776018	-0.0111723	0.00359355	9.50334905	0.00205097	0.00825922	P2ry1
ENSMUSG00000027770.6	761.285841	-0.0056402	0.00210792	7.08882712	0.0077566	0.0257165	Dhx36
ENSMUSG00000027774.17	1568.65982	-0.0080447	0.00274751	8.48209918	0.00358658	0.01330704	Gfm1
ENSMUSG00000027775.10	1407.36562	0.00102178	0.00267975	0.14411767	0.70422132	0.85274485	Mfsd1
ENSMUSG00000027777.16	74.9291602	0.0006637	0.00550355	0.01432876	0.90471859	0.97807639	Schip1
ENSMUSG00000027778.17	108.776135	-0.0102937	0.00516977	3.83991198	0.05004615	0.12066944	Ift80

ENSMUSG00000027782.11	1050.1308	-0.0004586	0.00265562	0.02871296	0.8654435	0.95604183	Kpna4
ENSMUSG00000027784.11	343.332979	0.00789804	0.00308798	6.41092163	0.01134205	0.03546986	Ppm1l
ENSMUSG00000027787.15	551.065247	-0.0109167	0.00259756	17.366261	3.08E-05	0.0001974	Nmd3
ENSMUSG00000027792.12	2430.74784	0.01999807	0.00323552	37.9875531	7.12E-10	1.01E-08	Bche
ENSMUSG00000027793.7	35.749759	-0.0208044	0.049375	0.60852516	0.43534365	0.62690124	Ccna1
ENSMUSG00000027796.3	225.534702	-0.017875	0.00460988	14.5733066	0.00013481	0.00074692	Smad9
ENSMUSG00000027797.16	96.1877304	0.07948172	0.01169269	40.0126967	2.52E-10	3.81E-09	Dclk1
ENSMUSG00000027799.13	61.295285	0.01804324	0.00556925	10.0680476	0.00150863	0.00630784	Nbea
ENSMUSG00000027800.15	475.385038	0.00895446	0.00503162	3.12275286	0.07720625	0.17127414	Tm4sf1
ENSMUSG00000027801.8	1702.15585	0.02379651	0.00496399	22.8269078	1.77E-06	1.47E-05	Tm4sf4
ENSMUSG00000027803.15	402.865635	0.01413428	0.0035054	15.5071793	8.22E-05	0.00047937	Wwtr1
ENSMUSG00000027804.14	704.120944	-0.0081193	0.00232907	11.8455997	0.00057798	0.00271557	Ppid
ENSMUSG00000027805.17	108.249343	-0.0117903	0.00539838	4.77149255	0.02893473	0.07712222	Pfn2
ENSMUSG00000027806.10	518.385617	0.00370078	0.00387326	0.88310907	0.34735154	0.53552527	Tsc22d2
ENSMUSG00000027808.9	6466.44952	-0.0090212	0.00299776	8.84569787	0.00293781	0.01122486	Serp1
ENSMUSG00000027809.15	4416.07958	-0.0181712	0.00380296	22.4456386	2.16E-06	1.76E-05	Etfdh
ENSMUSG00000027810.15	802.615027	-0.0006108	0.00193392	0.09948664	0.75244656	0.88510322	Eif2a
ENSMUSG00000027811.10	23.5312217	-0.0019821	0.00554277	0.12377178	0.72497915	0.86706345	4930579G24Rik
ENSMUSG00000027820.13	982.629534	0.01736137	0.00457202	12.972863	0.00031604	0.00159616	Mme
ENSMUSG00000027822.17	1398.0602	-0.0095243	0.00273351	11.8815606	0.00056692	0.00266993	Slc33a1
ENSMUSG00000027823.10	890.297619	-0.0023826	0.00194971	1.51021978	0.21910555	0.38359735	Gmps
ENSMUSG00000027824.6	2.2504962	0.03963061	0.01302089	8.90907319	0.00283757	0.01089925	Vmn2r1
ENSMUSG00000027827.18	24.4197458	0.02584407	0.00628042	16.4749289	4.93E-05	0.00030279	Kcnab1
ENSMUSG00000027828.13	6143.90548	0.00244895	0.00232851	1.08372461	0.29786583	0.48149293	Ssr3
ENSMUSG00000027829.16	700.15417	-0.0020317	0.00336915	0.35531986	0.55111702	0.73353683	Ccnl1
ENSMUSG00000027831.10	15.3164282	0.0198673	0.00893381	4.60982014	0.03178937	0.08344842	Veph1
ENSMUSG00000027832.6	5.11219932	-0.0384088	0.0229271	2.30272692	0.12914709	0.25642421	Ptx3
ENSMUSG00000027833.17	3.63265316	0.01927526	0.02389313	1.14821437	0.28392326	0.46404365	Shox2
ENSMUSG00000027834.16	45.22513	0.03524323	0.00789125	18.6264938	1.59E-05	0.0001086	Serpini1
ENSMUSG00000027835.12	1044.77956	-0.0022444	0.00319301	0.48719431	0.48518154	0.6741773	Pdcd10
ENSMUSG00000027840.6	6.04852068	-0.0093475	0.0079493	1.34756159	0.24570486	0.41668067	Wnt2b
ENSMUSG00000027843.14	38.3355922	0.08829812	0.00705297	137.111141	1.14E-31	2.99E-29	Ptpn22
ENSMUSG00000027845.16	104.852616	0.00277194	0.00352927	0.60605936	0.4362754	0.62778303	Dclre1b
ENSMUSG00000027848.14	102.421877	0.04076276	0.00518536	60.1272826	8.89E-15	2.46E-13	Olfml3
ENSMUSG00000027849.20	4.78125328	0.02049418	0.0220489	0.79831934	0.37159634	0.56214939	Syt6
ENSMUSG00000027852.15	569.049383	0.00566028	0.00331196	2.85947748	0.090837	0.19482418	Nras
ENSMUSG00000027854.13	416.637297	0.00760083	0.00416609	3.30095625	0.06923956	0.15681625	Sike1
ENSMUSG00000027855.14	5.92392483	0.01608958	0.01323372	1.45185015	0.22823131	0.39488437	Sycp1
ENSMUSG00000027857.11	1.91211006	-0.0082174	0.02180291	0.12332287	0.72545815	0.8674164	Tshb
ENSMUSG00000027858.14	65.2381145	0.01093283	0.00486181	5.05093769	0.02461262	0.06755652	Tspan2
ENSMUSG00000027859.11	54.6415477	0.01502862	0.00393782	14.3057449	0.00015539	0.00085035	Ngf
ENSMUSG00000027860.17	145.806713	-0.0071911	0.00444917	2.60308703	0.10665578	0.22068456	Vangl1
ENSMUSG00000027861.14	670.929407	-0.0362419	0.01656873	1.85128756	0.17363372	0.32145407	Casq2
ENSMUSG00000027863.9	19.6350857	0.1025768	0.00731006	164.722364	1.05E-37	5.64E-35	Cd2
ENSMUSG00000027864.10	80.2493434	0.00163452	0.00569033	0.08399718	0.77195191	0.89788232	Ptgfrn
ENSMUSG00000027865.11	515.076566	-0.0020145	0.00246344	0.66402142	0.41514381	0.60645296	Gdap2
ENSMUSG00000027867.11	6.30856844	0.00709381	0.0127777	0.28039723	0.59644098	0.77129541	Spag17
ENSMUSG00000027868.12	10.122187	0.00217574	0.02372249	-1.2891857	1	1	Tbx15
ENSMUSG00000027870.9	146.786271	0.15959397	0.01225124	134.94475	3.39E-31	8.17E-29	Hao2
ENSMUSG00000027871.17	4.24544169	-0.0216425	0.01981246	0.93238421	0.33424415	0.52118753	Hsd3b1
ENSMUSG00000027875.13	37892.1013	-0.0144424	0.00428587	11.0523717	0.00088574	0.00394096	Hmgcs2
ENSMUSG00000027876.5	1.6775765	-0.0056392	0.01226486	0.22103424	0.638253	0.80503178	Reg4
ENSMUSG00000027878.12	829.640619	0.01172737	0.00297718	15.4083674	8.66E-05	0.00050163	Notch2
ENSMUSG00000027879.10	983.404088	-0.0038803	0.00207074	3.48576851	0.06189861	0.14346902	Sec22b
ENSMUSG00000027881.15	1660.40115	0.00529345	0.00357651	2.16978904	0.14074554	0.273919	Prpf38b
ENSMUSG00000027882.19	471.186125	0.00747163	0.00209094	12.5386434	0.00039862	0.00195954	Stxbp3
ENSMUSG00000027883.16	273.486876	0.01055054	0.00446815	5.61291529	0.01782858	0.05177597	Gpsm2
ENSMUSG00000027884.17	788.605299	-0.0021962	0.00240142	0.8387949	0.35974166	0.54900464	Clcc1
ENSMUSG00000027887.12	2.59160034	-0.0033886	0.01613142	0.03497946	0.85163872	0.94847251	Sypl2
ENSMUSG00000027889.18	479.526872	-0.0063937	0.00323036	3.93165378	0.04738532	0.11551013	Ampd2
ENSMUSG00000027890.18	988.980567	-0.0005688	0.0038927	0.02122645	0.88416374	0.96635246	Gstm4

ENSMUSG00000027893.15	1794.55999	-0.0117372	0.00184516	40.1825093	2.31E-10	3.52E-09	Ahcyl1
ENSMUSG00000027894.15	15.9294106	0.04767337	0.01523144	10.7546873	0.00104016	0.00454991	Slc6a17
ENSMUSG00000027895.11	2.51214639	0.0090871	0.01356016	0.48650717	0.48548953	0.67428783	Kcnc4
ENSMUSG00000027896.16	12.5163201	-0.0019068	0.01080302	0.02798431	0.86714572	0.95715551	Slc16a4
ENSMUSG00000027900.16	1395.509	0.00019515	0.0024892	0.00599968	0.93825952	0.99602975	Dram2
ENSMUSG00000027901.13	219.307985	0.02089249	0.00284954	53.6887549	2.35E-13	5.37E-12	Dennd2d
ENSMUSG00000027905.16	201.008437	-0.0064946	0.00208617	9.61902612	0.00192572	0.00781621	Ddx20
ENSMUSG00000027907.5	207.136597	0.05152683	0.00638952	61.6903058	4.02E-15	1.15E-13	S100a11
ENSMUSG00000027908.3	0.69581018	0.057084	0.02769028	5.16329823	0.02306905	0.06406391	Tchhl1
ENSMUSG00000027931.13	204.369555	-0.016301	0.00336416	24.0081612	9.59E-07	8.27E-06	Npr1
ENSMUSG00000027932.15	16.285355	0.00061059	0.00595763	0.01053845	0.91823524	0.9854466	Slc27a3
ENSMUSG00000027933.12	449.202236	-0.0054291	0.00195639	7.62022145	0.00577174	0.01999886	Ints3
ENSMUSG00000027935.15	86.8288727	0.00617783	0.0027639	5.03455997	0.0248464	0.06808821	Rab13
ENSMUSG00000027936.19	472.213912	0.00179618	0.00342818	0.27461825	0.6002505	0.77426898	Crtc2
ENSMUSG00000027937.16	1044.0274	-0.0105593	0.00274369	14.8370363	0.00011721	0.00065964	Jtb
ENSMUSG00000027938.12	3.23380039	-0.002309	0.00811384	0.08089358	0.77609009	0.90085184	Creb3l4
ENSMUSG00000027939.12	6.24383136	0.02828793	0.01001402	7.81170338	0.0051909	0.01826532	Nup210l
ENSMUSG00000027940.19	1969.48782	0.00989552	0.00259325	14.3482203	0.00015192	0.00083274	Tpm3
ENSMUSG00000027942.18	652.272	-0.003204	0.00333618	0.92839752	0.33527967	0.5224223	4933434E20Rik
ENSMUSG00000027944.14	1151.47011	-0.0166776	0.00321336	27.3863073	1.67E-07	1.66E-06	Hax1
ENSMUSG00000027947.12	995.906106	-0.0107483	0.00592149	3.34162274	0.06754796	0.153827	Il6ra
ENSMUSG00000027950.8	43.4951495	0.01026154	0.00439665	5.28843556	0.0214675	0.06033513	Chrnb2
ENSMUSG00000027951.17	438.373522	0.01106858	0.00293364	14.381281	0.00014928	0.00082015	Adar
ENSMUSG00000027952.17	1175.83366	0.00630631	0.00443818	1.9313803	0.16460751	0.30825191	Pmvk
ENSMUSG00000027953.14	131.512878	0.0071112	0.00324022	4.86815152	0.02735679	0.07368245	Slc50a1
ENSMUSG00000027954.10	863.127936	0.00302301	0.00431821	0.47129303	0.49239291	0.68008346	Efna1
ENSMUSG00000027955.17	31.5936658	0.00501505	0.00810354	0.36303117	0.5468276	0.72969854	Gask1b
ENSMUSG00000027956.12	125.46963	-0.0048913	0.00542881	0.79384798	0.37293911	0.56367458	Tmem144
ENSMUSG00000027957.14	1393.86779	-0.0006672	0.00249075	0.07095949	0.78994486	0.91023416	Slc35a3
ENSMUSG00000027959.15	35.3700649	-0.0009066	0.00541552	0.02715308	0.86911563	0.95820882	Sass6
ENSMUSG00000027961.8	120.916542	0.02536737	0.0086674	5.56529871	0.01831988	0.05293309	Lrrc39
ENSMUSG00000027962.15	540.088533	0.06213283	0.00602059	93.4425558	4.18E-22	2.84E-20	Vcam1
ENSMUSG00000027963.15	297.954676	-0.0046475	0.00509458	0.81620965	0.36629071	0.55615635	Extl2
ENSMUSG00000027965.16	115.062232	0.0439553	0.00515268	67.1239691	2.55E-16	8.40E-15	Olfm3
ENSMUSG00000027966.21	2.89842684	-0.0184493	0.02206763	-0.9125789	1	1	Col11a1
ENSMUSG00000027967.9	1.47279152	0.00239018	0.08436717	0.00673649	0.93458619	0.99423346	Neurog2
ENSMUSG00000027968.12	296.146815	0.00319606	0.0031209	1.02615872	0.31106258	0.49624607	Larp7
ENSMUSG00000027971.17	2.80408167	0.01509014	0.02012483	1.18055387	0.27724327	0.45603318	Ndst4
ENSMUSG00000027977.16	3.14148775	0.02337831	0.01673286	1.69070567	0.19350797	0.34893571	Ndst3
ENSMUSG00000027978.10	5.44521465	0.02303084	0.00904306	6.46101365	0.01102667	0.03467206	Prss12
ENSMUSG00000027981.15	274.870472	0.00376361	0.00274145	1.83680648	0.17532571	0.32384934	Rnpc3
ENSMUSG00000027983.14	613.67768	-0.0339023	0.00543989	37.5253353	9.02E-10	1.26E-08	Cyp2u1
ENSMUSG00000027984.9	5732.53524	-0.0031112	0.00251494	1.51751075	0.21799658	0.3822801	Hadh
ENSMUSG00000027985.15	7.84307216	0.05288183	0.00948208	29.2575643	6.34E-08	6.76E-07	Lef1
ENSMUSG00000027993.17	758.127171	0.01076945	0.00428522	6.25227368	0.0124034	0.03818149	Trim2
ENSMUSG00000027994.15	6.54655289	0.07750361	0.01089464	41.7239046	1.05E-10	1.68E-09	Mcub
ENSMUSG00000027995.11	94.9407712	0.04652002	0.00680027	39.6678959	3.01E-10	4.50E-09	Tlr2
ENSMUSG00000027996.14	2.48448036	0.0165304	0.01660205	0.87961417	0.34830738	0.53630877	Sfrp2
ENSMUSG00000027997.10	367.629702	0.00501614	0.00305662	2.74545891	0.09753107	0.20613546	Casp6
ENSMUSG00000027998.12	404.176521	-0.0061063	0.00194323	9.77892803	0.00176523	0.00723705	Plrg1
ENSMUSG00000027999.16	244.650946	0.00311836	0.00400782	0.60982324	0.43485437	0.62650267	Pla2g12a
ENSMUSG00000028001.17	88685.1006	-0.0030291	0.0065213	0.2118856	0.64529349	0.81026089	Fga
ENSMUSG00000028003.7	469.912541	0.01942174	0.00412157	20.780067	5.15E-06	3.89E-05	Lrat
ENSMUSG00000028005.14	136.316079	0.00645688	0.00336509	3.63114513	0.05670779	0.13377774	Gucy1b1
ENSMUSG00000028007.14	484.192969	0.00662492	0.00279075	5.57091015	0.01826127	0.052809	Snx7
ENSMUSG00000028008.11	81.2670759	-0.0378561	0.00437821	74.9495568	4.83E-18	1.96E-16	Asic5
ENSMUSG00000028010.17	88.4294774	-0.0056793	0.00508195	1.26108793	0.26144526	0.43582217	Gar1
ENSMUSG00000028011.17	23958.8616	-0.020535	0.0056739	13.293306	0.00026636	0.00137407	Tdo2
ENSMUSG00000028013.17	806.579436	-0.0028044	0.00308271	0.82232325	0.36450178	0.55426078	Ppa2
ENSMUSG00000028015.4	1129.67863	0.00956161	0.00189147	25.3971208	4.67E-07	4.27E-06	Ctso
ENSMUSG00000028016.10	143.93301	0.00838369	0.00263217	10.0194975	0.00154892	0.00644772	Ints12

ENSMUSG00000028017.8	66.8362053	0.02454615	0.0071398	11.4583831	0.00071172	0.00325892	Egf
ENSMUSG00000028018.16	73.2424246	0.00446861	0.00269115	2.7492762	0.09729847	0.20578207	Gstcd
ENSMUSG00000028019.10	110.861364	0.02329536	0.00473983	23.13349	1.51E-06	1.27E-05	Pdgfc
ENSMUSG00000028020.17	11.3375503	0.01324863	0.01722217	0.78924889	0.37432734	0.5653112	Glrb
ENSMUSG00000028024.15	1799.11037	0.01573463	0.0034677	20.3901433	6.32E-06	4.68E-05	Enpep
ENSMUSG00000028028.12	77.4436536	0.0513648	0.0071616	43.9096064	3.44E-11	5.88E-10	Alpk1
ENSMUSG00000028029.12	530.967396	-0.0056911	0.00307435	3.42035216	0.06439727	0.14810272	Aimp1
ENSMUSG00000028030.13	417.335156	0.01153285	0.00294775	14.6828864	0.0001272	0.00070957	Tbck
ENSMUSG00000028031.7	4.46297143	0.02512675	0.01502708	3.4573208	0.06297227	0.1455	Dkk2
ENSMUSG00000028032.14	262.139095	0.0058502	0.00293737	3.82789173	0.0504063	0.12135173	Papss1
ENSMUSG00000028033.17	13.5107054	0.04146737	0.00975398	16.9911237	3.76E-05	0.00023703	Kcnq5
ENSMUSG00000028034.16	819.622599	-0.0027542	0.00311802	0.76220324	0.3826399	0.57432511	Fubp1
ENSMUSG00000028035.14	654.331878	-0.0065622	0.00322793	4.07862401	0.04342901	0.10758499	Dnajb4
ENSMUSG00000028036.7	11.7847611	0.07317944	0.01028858	45.9081837	1.24E-11	2.26E-10	Ptgfr
ENSMUSG00000028037.14	139.914513	0.05675281	0.00662712	71.8783054	2.29E-17	8.67E-16	Ifi44
ENSMUSG00000028039.12	8.42498819	0.00089306	0.0147427	0.0066579	0.93496802	0.99441539	Efna3
ENSMUSG00000028040.8	2.20751099	0.02139982	0.01128331	3.84863089	0.04978662	0.12015421	Efna4
ENSMUSG00000028041.18	85.0756654	0.01399998	0.00603492	5.2459831	0.0219975	0.06156062	Adam15
ENSMUSG00000028042.16	1135.05134	-0.0052357	0.00348984	2.18726883	0.13915587	0.27149726	Zbtb7b
ENSMUSG00000028044.7	91.5855859	0.00588701	0.00518991	1.30859599	0.25264902	0.42482211	Cks1b
ENSMUSG00000028047.12	14.8716309	0.02004166	0.00982465	3.9357308	0.0472706	0.11534495	Thbs3
ENSMUSG00000028048.12	406.757061	0.00794498	0.00208261	14.58341	0.00013409	0.00074345	Gba
ENSMUSG00000028049.16	653.274669	-0.00806	0.0023132	12.2321518	0.00046973	0.00226242	Scamp3
ENSMUSG00000028051.11	576.63783	0.03360619	0.00943064	11.2081729	0.00081438	0.00366844	Hcn3
ENSMUSG00000028053.14	891.542674	0.0012346	0.0035075	0.12238617	0.72646077	0.86791124	Ash1l
ENSMUSG00000028057.15	262.07398	0.00628212	0.00347368	3.24071415	0.07182932	0.1616571	Rit1
ENSMUSG00000028059.17	163.693916	0.05247742	0.00624951	64.934714	7.74E-16	2.42E-14	Arhgef2
ENSMUSG00000028060.15	836.375167	-0.0047093	0.0029647	2.522424	0.11223794	0.22973566	Khdc4
ENSMUSG00000028062.16	791.400973	-0.0076095	0.00326154	5.51414416	0.0188633	0.05422423	Lamtor2
ENSMUSG00000028063.16	721.912151	0.00887822	0.00251799	12.3701782	0.00043625	0.00211714	Lmna
ENSMUSG00000028064.18	1356.79742	0.01017424	0.00369268	7.48776379	0.00621197	0.02129275	Sema4a
ENSMUSG00000028066.16	295.114507	0.00055732	0.0031822	0.0309655	0.86031737	0.95360048	Pmf1
ENSMUSG00000028068.15	13.0360331	0.01918366	0.01045276	3.28248654	0.07002277	0.15821027	Iqgap3
ENSMUSG00000028069.17	310.693577	-0.0040157	0.0040194	1.00367522	0.31642284	0.50196053	Gpatch4
ENSMUSG00000028070.8	892.44551	-0.0029862	0.00230105	1.70035147	0.19224202	0.34736997	Naxe
ENSMUSG00000028071.13	14.9396447	0.0726391	0.00776193	76.6339026	2.06E-18	8.73E-17	Sh2d2a
ENSMUSG00000028072.7	13.8354997	0.03643193	0.0084828	15.3323589	9.02E-05	0.00052043	Ntrk1
ENSMUSG00000028073.17	149.857392	0.0060869	0.00348041	3.05415884	0.08053068	0.17706691	Pear1
ENSMUSG00000028076.13	3742.32038	0.00040857	0.00383926	0.01142052	0.91489463	0.98336958	Cd1d1
ENSMUSG00000028078.15	26.3322414	0.01408416	0.00735574	3.48326407	0.06199235	0.14359481	Dclk2
ENSMUSG00000028080.17	497.712415	0.00036532	0.00206552	0.03101662	0.86020332	0.95355339	Lrba
ENSMUSG00000028081.7	8972.39152	0.0004245	0.0031879	0.01767444	0.89423667	0.97287826	Rps3a1
ENSMUSG00000028082.15	1421.6131	-0.0061915	0.00314117	3.85615398	0.04956382	0.11969141	Sh3d19
ENSMUSG00000028085.13	625.963633	-0.0130586	0.00266293	23.6452978	1.16E-06	9.86E-06	Gatb
ENSMUSG00000028086.16	243.343022	0.00486435	0.00349657	1.88418309	0.16985947	0.31590487	Fbxw7
ENSMUSG00000028088.15	12369.7263	0.00391642	0.00423147	0.84320727	0.3584811	0.54732879	Fmo5
ENSMUSG00000028089.6	188.843953	0.00660365	0.00261346	6.34807346	0.01175088	0.03645835	Chd1l
ENSMUSG00000028093.16	537.505969	-0.0065684	0.00203091	10.6017152	0.00112983	0.00488777	Acp6
ENSMUSG00000028096.14	522.318718	-0.0110616	0.00267589	16.9369491	3.86E-05	0.0002433	Gpr89
ENSMUSG00000028098.14	588.808203	0.00215103	0.00151816	2.00341596	0.15694516	0.29727824	Rnf115
ENSMUSG00000028099.9	358.376804	-0.0031748	0.00148526	4.60336753	0.03190922	0.08370714	Polr3c
ENSMUSG00000028100.12	2.85206273	-0.0094646	0.00924195	1.04035726	0.3077384	0.49279095	Nudt17
ENSMUSG00000028101.19	138.151112	0.00190923	0.00310475	0.37617804	0.53965582	0.72357013	Pias3
ENSMUSG00000028102.16	457.768223	-0.0061486	0.00214873	8.3467925	0.00386369	0.01418349	Pex11b
ENSMUSG00000028104.15	197.216601	0.00759278	0.00307609	6.07189646	0.01373494	0.04164982	Polr3gl
ENSMUSG00000028106.14	444.380031	0.00398523	0.00240826	2.72666261	0.09868531	0.20803699	Rprd2
ENSMUSG00000028107.15	414.695402	-0.0135734	0.00205588	43.8745477	3.50E-11	5.98E-10	Tars2
ENSMUSG00000028108.17	1912.95778	0.00266717	0.0040205	0.43878609	0.50770894	0.69455502	Ecm1
ENSMUSG00000028111.5	19.4470898	0.03759564	0.00635083	34.1445559	5.12E-09	6.46E-08	Ctsk
ENSMUSG00000028114.17	143.497753	0.00265509	0.0029769	0.77588225	0.37840346	0.569603	Mettl14
ENSMUSG00000028116.14	161.816522	-0.0171642	0.02736957	-0.9756106	1	1	Myoz2

ENSMUSG00000028121.9	773.156709	-0.0087138	0.00366022	5.61105789	0.01784749	0.05182208	Bcar3
ENSMUSG00000028124.17	3867.87781	-0.0023164	0.0031529	0.53827755	0.46314744	0.6542189	Gclm
ENSMUSG00000028125.15	7.50761514	0.00510327	0.01011948	0.24879216	0.61792684	0.78895807	Abca4
ENSMUSG00000028126.17	228.816678	-0.004068	0.0022384	3.29953993	0.06929929	0.15692138	Pip5k1a
ENSMUSG00000028127.11	10926.8154	-0.0238575	0.00342026	47.1085974	6.72E-12	1.27E-10	Abcd3
ENSMUSG00000028128.14	86.6623035	-0.0066503	0.00463499	2.03252846	0.15396428	0.29319547	F3
ENSMUSG00000028132.16	3920.38373	-0.0041278	0.00393972	1.03998349	0.30782531	0.492857	Tlcd4
ENSMUSG00000028133.18	106.473379	0.00729509	0.00407647	3.16860461	0.07506656	0.16752008	Rwdd3
ENSMUSG00000028134.12	211.592784	0.00545877	0.00332653	2.6745379	0.10196492	0.21339349	Ptbp2
ENSMUSG00000028136.16	554.663519	0.00210963	0.00260394	0.64745919	0.42102255	0.6125995	Snx27
ENSMUSG00000028137.10	3.77271551	0.02446745	0.01679631	2.36751886	0.12388381	0.24829166	Celf3
ENSMUSG00000028138.9	6008.6489	-0.0035616	0.00252011	2.01038961	0.15622519	0.29636715	Adh5
ENSMUSG00000028140.14	740.424359	-0.0123184	0.00251863	23.9254446	1.00E-06	8.62E-06	Mrpl9
ENSMUSG00000028141.14	4.93241621	-0.0255681	0.01147745	4.46806675	0.03453404	0.08936039	Oaz3
ENSMUSG00000028145.10	479.443701	-0.0164295	0.00349129	22.4992603	2.10E-06	1.71E-05	Them4
ENSMUSG00000028148.12	2.05668125	-0.0554334	0.01026584	30.3742194	3.56E-08	3.95E-07	Them5
ENSMUSG00000028149.13	527.676413	0.00749699	0.00239183	9.79107234	0.00175361	0.00719316	Rap1gds1
ENSMUSG00000028150.15	1086.93178	-0.0083881	0.0041182	4.18538506	0.04077395	0.10227527	Rorc
ENSMUSG00000028152.11	33.6509377	0.01767222	0.00769585	5.21080074	0.02244699	0.06265501	Tspan5
ENSMUSG00000028156.13	1137.59472	-0.0077734	0.00319997	5.85156742	0.01556318	0.04625641	Eif4e
ENSMUSG00000028158.15	5845.37588	-0.0086215	0.00358835	5.65434374	0.01741219	0.05077694	Mttp
ENSMUSG00000028159.15	35.7598256	0.03834704	0.00625198	34.8852105	3.50E-09	4.52E-08	Dapp1
ENSMUSG00000028161.18	1033.44299	0.00361818	0.00294595	1.50621251	0.21971793	0.38441267	Ppp3ca
ENSMUSG00000028163.18	496.297526	0.00494948	0.00253583	3.68099484	0.055036	0.13057833	Nfkb1
ENSMUSG00000028164.16	376.726943	-0.0016244	0.00272138	0.35996681	0.54852467	0.73126288	Manba
ENSMUSG00000028165.10	1017.52489	-0.0081725	0.00191488	18.31869	1.87E-05	0.00012575	Cisd2
ENSMUSG00000028167.16	295.073669	-0.0012188	0.00475129	0.0628836	0.80199502	0.91704341	Bdh2
ENSMUSG00000028173.11	174.040868	0.03040487	0.00408961	52.9812567	3.37E-13	7.53E-12	Wls
ENSMUSG00000028175.16	7.56183397	0.03357028	0.01295194	6.04984221	0.01390754	0.04212988	Depdc1a
ENSMUSG00000028176.14	4.26569096	0.02046963	0.020557	1.59194635	0.20704827	0.36786747	Lrrc7
ENSMUSG00000028179.13	6389.02505	0.00299047	0.00534985	0.30517735	0.58065474	0.75861756	Cth
ENSMUSG00000028180.19	1506.89414	-0.0016664	0.00333212	0.24123523	0.62331539	0.79279322	Zranb2
ENSMUSG00000028182.15	39.7017555	0.01181368	0.00395605	8.84435926	0.00293996	0.01123128	Lrriq3
ENSMUSG00000028184.14	767.509676	-0.0023592	0.00262983	0.79820685	0.37163003	0.56216431	Adgrl2
ENSMUSG00000028185.13	475.117005	0.00179096	0.00357302	0.24927684	0.61758474	0.78864936	Dnase2b
ENSMUSG00000028186.15	37911.7894	0.00128331	0.00575712	0.04969649	0.82359224	0.92983805	Uox
ENSMUSG00000028187.11	374.598344	-0.0077718	0.00233415	11.1279938	0.00085035	0.00380574	Rpf1
ENSMUSG00000028188.14	41.7634622	-0.0086331	0.00576784	2.18314341	0.13952923	0.27209064	Spata1
ENSMUSG00000028189.13	264.370673	0.01441874	0.00279611	26.4545817	2.70E-07	2.59E-06	Ctbs
ENSMUSG00000028191.12	262.638269	0.01037227	0.00275446	13.8977611	0.00019303	0.00103331	Bcl10
ENSMUSG00000028194.16	2462.96017	0.01132728	0.00411517	7.400772	0.00651959	0.02222125	Ddah1
ENSMUSG00000028195.5	286.461515	0.00276389	0.0057171	0.22288111	0.63685337	0.80373039	Ccn1
ENSMUSG00000028197.5	22.4901321	0.03227872	0.00934637	10.5609143	0.00115504	0.00498313	Col24a1
ENSMUSG00000028199.19	1557.11019	0.0073519	0.00325397	5.06669309	0.02438987	0.06706219	Cryz
ENSMUSG00000028207.19	1250.2274	-0.0012446	0.00359917	0.11754627	0.73171115	0.87130397	Asph
ENSMUSG00000028211.12	1498.61603	0.0109682	0.00407165	7.07795456	0.0078038	0.02584031	Trp53inp1
ENSMUSG00000028212.17	77.9217357	0.01644525	0.00870335	3.39591366	0.06535814	0.14991123	Ccne2
ENSMUSG00000028214.14	61.915741	0.0262785	0.00375874	46.2903031	1.02E-11	1.89E-10	Gem
ENSMUSG00000028217.12	2.08167154	0.07909508	0.01382013	27.6038514	1.49E-07	1.50E-06	Cdh17
ENSMUSG00000028218.20	250.913784	0.00239184	0.00292373	0.67844521	0.41012262	0.60183522	Cibar1
ENSMUSG00000028221.4	266.827087	0.00131458	0.00213948	0.37695172	0.53923916	0.72317591	Pip4p2
ENSMUSG00000028222.3	20.9512225	0.01742856	0.01493966	1.15434424	0.28264159	0.46265421	Calb1
ENSMUSG00000028223.9	3360.75123	-0.0205622	0.00318114	41.1306341	1.42E-10	2.23E-09	Decr1
ENSMUSG00000028224.15	330.599065	-0.0062044	0.00228216	7.20169341	0.00728348	0.02443644	Nbn
ENSMUSG00000028226.16	5.26753981	0.01965703	0.01814485	1.33701529	0.24756101	0.41885366	Mmp16
ENSMUSG00000028228.6	1040.81578	0.00534118	0.00262993	4.07017447	0.04364677	0.10803365	Cpne3
ENSMUSG00000028229.12	886.492934	-0.0144135	0.00321075	20.1434684	7.18E-06	5.26E-05	Rmdn1
ENSMUSG00000028232.14	238.627918	0.00381578	0.00271838	1.95471248	0.16207994	0.30450991	Tmem68
ENSMUSG00000028233.7	306.955955	-0.0028678	0.00277725	1.04246717	0.30724837	0.49224018	Tgs1
ENSMUSG00000028234.7	3966.85703	-0.0018193	0.00401714	0.20592275	0.64998205	0.81327403	Rps20
ENSMUSG00000028236.4	2.3190944	-0.136721	0.01494595	89.9253713	2.47E-21	1.48E-19	Sdr16c5

ENSMUSG00000028238.7	132.748935	0.10452667	0.00675435	207.716358	4.33E-47	5.37E-44	Atp6v0d2
ENSMUSG00000028240.3	3092.91263	0.00769951	0.00799517	0.91665708	0.33835425	0.52599679	Cyp7a1
ENSMUSG00000028243.6	101.288847	0.01072813	0.00288483	13.674529	0.00021738	0.00114882	Ubxn2b
ENSMUSG00000028245.16	387.662343	0.00643441	0.00285884	4.96640386	0.02584433	0.07041288	Nsmaf
ENSMUSG00000028246.14	4.39271457	0.03569399	0.0133739	7.23801039	0.0071376	0.02401191	Faxc
ENSMUSG00000028247.3	374.924543	-0.0211734	0.00274485	59.6049426	1.16E-14	3.15E-13	Coq3
ENSMUSG00000028248.16	1758.99838	0.00018857	0.00375649	0.00250585	0.96007578	1	Pnisr
ENSMUSG00000028249.16	1295.50103	0.0126324	0.00270196	21.1411691	4.27E-06	3.28E-05	Sdcbp
ENSMUSG00000028251.6	436.279809	-0.0029223	0.00280362	1.10558973	0.29304266	0.47512918	Tstd3
ENSMUSG00000028252.21	413.323477	-0.0011838	0.00199917	0.34648734	0.55610833	0.73832654	Ccnc
ENSMUSG00000028255.7	4.37362534	0.01300418	0.01471071	0.89432117	0.34430899	0.53255111	Clca1
ENSMUSG00000028256.17	193.30628	0.00898498	0.0035888	6.16167759	0.01305473	0.03985361	Odf2l
ENSMUSG00000028261.14	764.008179	-0.0212232	0.00298799	51.2803445	8.01E-13	1.70E-11	Ndufaf4
ENSMUSG00000028262.14	22.921959	6.91E-05	0.01573143	0.00109636	0.97358581	1	Clca3a2
ENSMUSG00000028264.17	2.24547496	0.00023038	0.01320845	0.00041722	0.98370352	1	Spaca1
ENSMUSG00000028266.18	307.555935	0.01232123	0.00426722	8.21909982	0.00414518	0.01506675	Lmo4
ENSMUSG00000028268.15	263.006674	0.05907852	0.0051484	130.606699	3.02E-30	6.53E-28	Gbp3
ENSMUSG00000028270.13	225.287122	0.0746485	0.00562209	162.130025	3.88E-37	1.90E-34	Gbp2
ENSMUSG00000028271.10	382.113866	-0.0089134	0.0023458	14.4546163	0.00014358	0.00079103	Gtf2b
ENSMUSG00000028273.17	1422.09152	-0.0068736	0.00400693	2.83629401	0.09215654	0.19717019	Pdlim5
ENSMUSG00000028274.18	226.493244	-0.0030171	0.00234652	1.64069416	0.20023025	0.35867174	Rngtt
ENSMUSG00000028277.14	775.637752	-0.0013998	0.00215915	0.42311618	0.51538621	0.70200388	Ube2j1
ENSMUSG00000028278.15	105.016696	0.03541821	0.00620534	31.0715009	2.49E-08	2.83E-07	Rragd
ENSMUSG00000028282.13	179.146211	0.0123552	0.00502995	5.69208489	0.0170416	0.04986651	Casp8ap2
ENSMUSG00000028284.14	821.437115	-0.0026518	0.00247635	1.13880911	0.28590411	0.46644078	Map3k7
ENSMUSG00000028287.5	1.27425183	-0.0182178	0.06941895	0.00582472	0.93916464	0.99636278	1700009N14Rik
ENSMUSG00000028289.13	53.2019541	0.0131831	0.00453676	8.25522051	0.0040635	0.01482928	Epha7
ENSMUSG00000028291.8	290.70467	-0.004535	0.00207687	4.76014514	0.02912609	0.07753589	Akirin2
ENSMUSG00000028292.15	219.444907	-0.0037592	0.00264761	1.99850884	0.15745404	0.29805064	Rars2
ENSMUSG00000028293.15	473.976086	-0.0046838	0.00224854	4.30624538	0.03797268	0.0963347	Slc35a1
ENSMUSG00000028294.16	1.72002816	-0.0425635	0.0361522	2.20648377	0.1374316	0.26900098	Cfap206
ENSMUSG00000028295.15	193.659774	-0.006429	0.00447181	2.06079257	0.15113154	0.28901146	Smim8
ENSMUSG00000028298.11	1.88566836	-0.007616	0.02256051	0.02493173	0.87453729	0.96122741	Cga
ENSMUSG00000028300.15	248.390711	-0.0051314	0.00333863	2.29123875	0.13010605	0.25802322	C9orf72
ENSMUSG00000028307.10	72012.2768	-0.0029895	0.00438018	0.46548134	0.49507334	0.68233505	Aldob
ENSMUSG00000028309.15	690.333561	-0.0008207	0.00277891	0.08609009	0.76920792	0.89623761	Rnf20
ENSMUSG00000028310.3	1.49833579	-0.0137422	0.03769608	-0.0092208	1	1	Ppp3r2
ENSMUSG00000028312.20	129.655134	0.01756399	0.0061078	7.75337997	0.00536121	0.01876384	Smc2
ENSMUSG00000028318.15	85.652366	-0.0058532	0.00441209	1.75829112	0.18483781	0.3372925	Polr1e
ENSMUSG00000028322.12	207.800807	-0.0014216	0.00264742	0.28638256	0.59254805	0.76807201	Exosc3
ENSMUSG00000028327.15	2894.52768	-0.0041785	0.00483125	0.73725705	0.39054096	0.5819219	Stra6l
ENSMUSG00000028328.14	77.5900023	-0.0062657	0.01042128	0.30485206	0.58085648	0.75875504	Tmod1
ENSMUSG00000028329.14	303.717081	0.0058735	0.00381899	2.36913022	0.12375599	0.2481262	Xpa
ENSMUSG00000028330.9	427.018461	-0.0080317	0.00218095	13.5608854	0.00023095	0.00121101	Ncbp1
ENSMUSG00000028331.13	47.4226547	-0.0011712	0.00314129	0.13983502	0.70844508	0.85578915	Trmo
ENSMUSG00000028332.14	3.32151038	0.04784736	0.03177958	2.43919259	0.11833712	0.23962048	Hemgn
ENSMUSG00000028333.11	1687.00449	0.00147275	0.00428319	0.11642486	0.73294484	0.8719478	Anp32b
ENSMUSG00000028334.11	265.829099	-2.30E-05	0.00336345	4.54E-05	0.99462568	1	Nans
ENSMUSG00000028337.15	34.6967505	0.05339138	0.00659664	61.5620156	4.29E-15	1.22E-13	Coro2a
ENSMUSG00000028339.18	186.98268	0.02018604	0.00597536	11.2995484	0.00077526	0.00351437	Col15a1
ENSMUSG00000028341.10	8.28688588	0.0409779	0.01020089	14.0574695	0.00017731	0.00096027	Nr4a3
ENSMUSG00000028343.11	1524.77255	-0.0096182	0.00321005	9.04653	0.00263194	0.01021842	Erp44
ENSMUSG00000028344.13	230.139343	0.00207369	0.00312082	0.42864123	0.51265643	0.69932274	Invs
ENSMUSG00000028345.16	142.957645	0.00984738	0.00371494	6.8291451	0.00896822	0.02911955	Tex10
ENSMUSG00000028347.15	13.2467797	0.02478403	0.01088671	4.80558016	0.02836771	0.07595424	Tmeff1
ENSMUSG00000028348.8	58.0854535	0.03908222	0.03275845	-1.3195844	1	1	Cavin4
ENSMUSG00000028351.6	8.86671333	0.02932431	0.01609011	3.84092423	0.05001594	0.12060894	Brinp1
ENSMUSG00000028354.14	22.4495268	-0.0111561	0.00963311	1.20629896	0.27206634	0.44939819	Fmn2
ENSMUSG00000028356.5	37119.5437	0.00263781	0.00543164	0.23681957	0.62651269	0.79522808	Ambp
ENSMUSG00000028357.14	12.3178346	0.01729841	0.00746775	5.49504114	0.01907048	0.05469313	Kif12
ENSMUSG00000028358.16	29.4410278	-0.0080061	0.007629	1.13293752	0.28714963	0.46808426	Zfp618

ENSMUSG00000028359.5	75.8720171	0.07445347	0.01069636	42.7908809	6.09E-11	1.01E-09	Orm3
ENSMUSG00000028360.11	2.97385371	0.02040597	0.02317389	1.43430028	0.23106391	0.39870315	Slc44a5
ENSMUSG00000028362.3	5.98602576	0.14293156	0.01238363	90.5730515	1.78E-21	1.09E-19	Tnfsf8
ENSMUSG00000028364.16	41.6023354	0.05989969	0.00700374	68.6398469	1.18E-16	4.05E-15	Tnc
ENSMUSG00000028367.6	3845.32692	-0.0030473	0.00306664	0.99208846	0.31923247	0.50517585	Txn1
ENSMUSG00000028369.16	36.6908018	0.05094455	0.00744632	43.940924	3.38E-11	5.79E-10	Svep1
ENSMUSG00000028370.8	10.85137	0.03146551	0.01023069	9.15505536	0.00248032	0.00970002	Pappa
ENSMUSG00000028373.17	27.9790814	0.01898045	0.00436701	18.4201141	1.77E-05	0.00011981	Astn2
ENSMUSG00000028378.7	72.5196224	-0.0096134	0.00447671	4.77117213	0.02894012	0.07712786	Ptgr1
ENSMUSG00000028381.9	444.813209	0.01244012	0.0025877	22.906836	1.70E-06	1.41E-05	Ugcg
ENSMUSG00000028382.16	1527.01841	0.00552001	0.00265528	4.19108288	0.04063713	0.10198632	Ptbp3
ENSMUSG00000028383.18	1601.68347	-0.0153769	0.00368492	16.700728	4.38E-05	0.00027248	Hsdl2
ENSMUSG00000028385.15	188.356884	0.01324873	0.00371521	12.7003587	0.00036559	0.00181187	Snx30
ENSMUSG00000028389.13	19.9898178	0.00179192	0.00587082	0.09088348	0.76305698	0.8923196	Zfp37
ENSMUSG00000028391.17	4.97599107	0.01311402	0.00970552	1.73495058	0.1877799	0.34124174	Wdr31
ENSMUSG00000028392.16	8.61896979	0.0336528	0.00878048	13.6590353	0.00021918	0.0011573	Bspry
ENSMUSG00000028393.11	2048.04628	-0.0054177	0.0032506	2.80176281	0.09416073	0.20061879	Alad
ENSMUSG00000028394.10	512.5211	-0.0033565	0.00249012	1.80396145	0.17923431	0.32936661	Pole3
ENSMUSG00000028396.6	8.77979469	0.04353032	0.04342613	-0.3541582	1	1	2310002L09Rik
ENSMUSG00000028397.14	186.343646	0.00372531	0.00226219	2.69070304	0.10093527	0.21168943	Kdm4c
ENSMUSG00000028398.9	451.574922	-0.008223	0.00349506	5.3525862	0.02069156	0.05864274	Dmac1
ENSMUSG00000028399.19	2794.18729	-0.0022181	0.00387827	0.32056897	0.57126592	0.75080118	Ptprd
ENSMUSG00000028402.20	441.982658	-0.0029692	0.00452031	0.43281824	0.51060931	0.69730943	Mpdz
ENSMUSG00000028403.16	293.332307	0.00142548	0.00337766	0.17231712	0.67806066	0.83382371	Zdhhc21
ENSMUSG00000028405.10	3736.79401	-0.0085862	0.00258986	10.9746512	0.00092367	0.00409273	Aco1
ENSMUSG00000028407.5	157.709676	-0.0123827	0.00456372	7.39541705	0.00653903	0.02228106	Smim27
ENSMUSG00000028409.12	1031.69005	-0.0061236	0.00186426	10.7566754	0.00103904	0.00454671	Smu1
ENSMUSG00000028410.14	4587.82814	-0.0121584	0.00353205	11.2527206	0.00079506	0.00359448	Dnaja1
ENSMUSG00000028411.16	199.600381	-0.001895	0.00249402	0.57463339	0.44842358	0.64026386	Aptx
ENSMUSG00000028412.18	1091.11318	0.0134624	0.00268653	24.6929532	6.72E-07	5.97E-06	Slc44a1
ENSMUSG00000028413.14	991.581899	-0.0029076	0.00272244	1.1048135	0.29321216	0.47530599	B4galt1
ENSMUSG00000028414.18	281.943072	-0.0061654	0.0031693	3.77748683	0.05194663	0.12432402	Fktn
ENSMUSG00000028415.5	1.03176767	0.00777188	0.02091649	0.14722196	0.70120442	0.85079517	Spink4
ENSMUSG00000028416.14	1547.16157	-0.0057435	0.00242628	5.60612884	0.01789776	0.05194249	Bag1
ENSMUSG00000028419.6	992.088555	-0.0009904	0.0019294	0.26244299	0.60844674	0.78110668	Chmp5
ENSMUSG00000028420.14	1096.28112	0.00152485	0.0023272	0.4342213	0.50992486	0.69653603	Tmem38b
ENSMUSG00000028423.16	922.826099	0.00237727	0.00212754	1.25814704	0.26200212	0.43654289	Nfx1
ENSMUSG00000028426.11	3423.47533	0.00017922	0.00226356	0.00614335	0.93752616	0.99576187	Rad23b
ENSMUSG00000028427.14	8.36614416	0.00564232	0.01624191	0.09154041	0.7622279	0.89176128	Aqp7
ENSMUSG00000028430.15	370.981564	-0.0059059	0.00228166	6.68168498	0.00974085	0.03119705	Nol6
ENSMUSG00000028431.12	243.992968	-0.0032477	0.00303913	1.14843371	0.28387727	0.46400064	Elp1
ENSMUSG00000028433.15	449.971157	-0.0031921	0.00436354	0.52703726	0.46785513	0.65896645	Ubap2
ENSMUSG00000028434.13	1422.61023	-0.018385	0.00343357	28.4716002	9.51E-08	9.92E-07	Epb41l4b
ENSMUSG00000028435.9	6.29921889	0.04197042	0.01550236	7.4429471	0.00636855	0.02174731	Aqp3
ENSMUSG00000028436.9	585.377826	-0.0066633	0.00148653	20.1644217	7.11E-06	5.21E-05	Dcaf12
ENSMUSG00000028437.15	788.581475	-0.0041295	0.00254333	2.64442349	0.10391385	0.21619346	Ubap1
ENSMUSG00000028438.17	17.1459128	0.01008272	0.00656189	2.32534252	0.1272822	0.25374677	Kif24
ENSMUSG00000028439.15	123.575205	0.01703975	0.00349688	23.4506038	1.28E-06	1.08E-05	Fam219a
ENSMUSG00000028443.7	229.421055	-0.0028331	0.00287685	0.98095222	0.32196383	0.50825506	Nudt2
ENSMUSG00000028444.18	38.6575479	0.00374246	0.00513403	0.5179653	0.47171092	0.6624456	Cntfr
ENSMUSG00000028445.8	384.592299	-0.0325116	0.00734713	18.9582726	1.34E-05	9.26E-05	Enho
ENSMUSG00000028447.12	528.193243	-0.0073244	0.00321118	5.19963268	0.02259166	0.06298427	Dctn3
ENSMUSG00000028451.13	1.9090259	-0.0100956	0.01319005	0.41766825	0.51810284	0.7042216	1700022I11Rik
ENSMUSG00000028452.8	6436.80731	-0.006754	0.00163252	16.9350999	3.87E-05	0.00024347	Vcp
ENSMUSG00000028453.11	56.0385465	0.00942804	0.00446476	4.35886867	0.03681675	0.09402922	Fancg
ENSMUSG00000028454.17	214.734783	-0.0110895	0.00315139	12.315134	0.0004493	0.00217289	Pigo
ENSMUSG00000028455.16	892.778933	-0.0097019	0.00248319	15.3223932	9.06E-05	0.00052306	Stoml2
ENSMUSG00000028456.19	237.01255	0.01878005	0.00443107	15.2134111	9.60E-05	0.00055089	Unc13b
ENSMUSG00000028457.19	17.2635233	0.02389002	0.00671503	11.9152546	0.00055676	0.00262793	Atp8b5
ENSMUSG00000028458.13	273.229807	-0.0032826	0.00318053	1.06788609	0.30142342	0.48584605	Tesk1
ENSMUSG00000028459.12	91.1792109	0.06364472	0.0061104	93.71552	3.64E-22	2.48E-20	Cd72

ENSMUSG00000028460.7	2.23447639	0.06939903	0.01108909	38.4367392	5.66E-10	8.12E-09	Sit1
ENSMUSG00000028461.13	925.578421	-0.0072467	0.00401659	3.28636184	0.06985765	0.15789771	Ccdc107
ENSMUSG00000028463.15	5.14216684	0.03336258	0.01015569	9.91671895	0.00163784	0.0067689	Car9
ENSMUSG00000028464.17	140.798112	0.00636286	0.00603925	0.98753436	0.32034575	0.50641252	Tpm2
ENSMUSG00000028465.17	2547.68004	0.00585494	0.00419463	1.92943564	0.1648202	0.3086012	Tln1
ENSMUSG00000028466.17	568.866733	-0.000674	0.0025305	0.07053703	0.79055646	0.91057306	Creb3
ENSMUSG00000028467.16	201.949223	-0.0109004	0.00327816	11.3189633	0.0007672	0.00348049	Gba2
ENSMUSG00000028468.16	677.528002	-0.0131658	0.00412264	10.0530266	0.00152098	0.00635272	Rgp1
ENSMUSG00000028469.16	779.51926	-0.003369	0.00416329	0.60797227	0.43555231	0.62704857	Npr2
ENSMUSG00000028470.11	1112.22166	-0.0036774	0.00388528	0.90736425	0.34081473	0.52849811	Hint2
ENSMUSG00000028475.10	8.95090758	0.02475276	0.00677009	12.4029527	0.00042866	0.00208545	Spaar
ENSMUSG00000028476.14	105.703109	-0.0031939	0.00416201	0.56125664	0.45375437	0.64537481	Reck
ENSMUSG00000028478.19	1580.25604	0.00644478	0.00247505	6.73353004	0.00946172	0.0304744	Clta
ENSMUSG00000028479.19	2434.03793	-0.008922	0.00364889	5.96345165	0.01460542	0.04395137	Gne
ENSMUSG00000028480.15	44.3982052	0.04534402	0.00642609	45.3541118	1.64E-11	2.95E-10	Glipr2
ENSMUSG00000028483.14	110.166695	-0.003981	0.0025182	2.49163066	0.11445309	0.23321732	Snapc3
ENSMUSG00000028484.17	1065.05956	0.00218263	0.00376327	0.32633209	0.56782691	0.74753101	Psip1
ENSMUSG00000028487.19	5.44247983	0.07993482	0.01409957	28.2539942	1.06E-07	1.10E-06	Bnc2
ENSMUSG00000028488.16	10.0789645	0.0318334	0.01172415	7.28732759	0.00694427	0.02348209	Sh3gl2
ENSMUSG00000028494.13	8516.83239	-0.0106484	0.00413029	6.00002481	0.01430568	0.04316409	Plin2
ENSMUSG00000028495.15	9246.47797	-0.0003599	0.00301156	0.01433301	0.90470453	0.97807639	Rps6
ENSMUSG00000028496.18	109.925408	0.00373921	0.00567511	0.42141336	0.51623265	0.70279153	Mllt3
ENSMUSG00000028497.13	86.2709938	0.04315321	0.00575412	52.5945615	4.10E-13	9.08E-12	Hacd4
ENSMUSG00000028514.17	1501.53179	-0.006552	0.00241745	7.27628811	0.00698708	0.02359639	Usp24
ENSMUSG00000028517.9	2617.35283	-0.0052054	0.00354126	2.1158695	0.14577924	0.28139372	Plpp3
ENSMUSG00000028518.9	1374.2887	-0.0159339	0.00329134	23.2878979	1.39E-06	1.17E-05	Prkaa2
ENSMUSG00000028519.17	23.4490281	-0.0106953	0.00827504	1.56219477	0.21134415	0.37352355	Dab1
ENSMUSG00000028520.13	1.92622003	-0.0260339	0.01981255	1.707453	0.19131616	0.34609473	4921539E11Rik
ENSMUSG00000028521.18	2191.45398	0.00059499	0.00427032	0.01901611	0.89032032	0.9701118	Slc35d1
ENSMUSG00000028522.17	887.030269	0.00543561	0.00324564	2.71549553	0.09937811	0.20918626	Mier1
ENSMUSG00000028524.22	25.7375632	0.03447003	0.00891421	13.4464956	0.00024546	0.00127776	Sgip1
ENSMUSG00000028525.17	405.152845	0.01593826	0.00415558	14.5874593	0.0001338	0.00074238	Pde4b
ENSMUSG00000028527.19	1422.8107	-0.0054051	0.00358894	2.27854781	0.13117465	0.25961947	Ak4
ENSMUSG00000028528.18	18.4890015	0.03507499	0.01156053	8.24154815	0.00409422	0.01490705	Dnajc6
ENSMUSG00000028530.15	2097.45981	0.00179007	0.0022693	0.61096842	0.43442342	0.62611126	Jak1
ENSMUSG00000028532.15	54.5026319	0.01207692	0.007741	2.15390465	0.14220783	0.27605875	Cachd1
ENSMUSG00000028536.13	152.750799	-0.0006292	0.00413145	0.02292184	0.8796605	0.9636572	2610528J11Rik
ENSMUSG00000028538.13	1055.23794	-0.0046137	0.00233154	3.81169111	0.05089604	0.12230606	St3gal3
ENSMUSG00000028540.7	65.8513946	-0.0041651	0.0030755	1.83471068	0.17557216	0.32417762	Dph2
ENSMUSG00000028541.15	9.42410743	0.01809898	0.00857342	4.4032399	0.03587072	0.0920717	B4galt2
ENSMUSG00000028542.18	529.321014	-0.0146665	0.00439349	11.0018837	0.00091019	0.00403834	Slc6a9
ENSMUSG00000028544.15	2.00465069	0.09145094	0.01766749	27.180514	1.85E-07	1.84E-06	Slc5a9
ENSMUSG00000028545.14	13.1589225	0.04329683	0.01069634	14.0247312	0.00018042	0.0009749	Bend5
ENSMUSG00000028546.18	12.1862214	0.01560479	0.01890322	0.88056101	0.34804807	0.53608449	Elavl4
ENSMUSG00000028549.18	91.1210984	0.0067653	0.00571202	1.38305481	0.23958149	0.40914988	Itgb3bp
ENSMUSG00000028550.16	271.895974	5.85E-05	0.00230202	0.00064525	0.97973453	1	Atg4c
ENSMUSG00000028551.15	99.6083054	-0.0181238	0.00587762	9.98438587	0.00157873	0.00655403	Cdkn2c
ENSMUSG00000028552.14	1021.99129	0.00674138	0.00266714	6.23415845	0.01253091	0.0384786	Eps15
ENSMUSG00000028553.13	11044.9413	0.00860937	0.00438059	3.78958657	0.05157239	0.12367934	Angptl3
ENSMUSG00000028555.16	27.4571966	0.03239175	0.00974176	10.3820202	0.00127248	0.0054312	Ttc39a
ENSMUSG00000028556.16	314.868367	0.02080498	0.0032042	41.3522112	1.27E-10	2.01E-09	Dock7
ENSMUSG00000028557.11	2531.66069	-0.0029081	0.0022625	1.59943287	0.2059836	0.36652804	Rnf11
ENSMUSG00000028559.18	2545.24842	-0.0044922	0.00210998	4.52251024	0.03345172	0.08698921	Osbpl9
ENSMUSG00000028560.12	430.28191	0.0030286	0.00259189	1.34585466	0.24600412	0.41711809	Usp1
ENSMUSG00000028563.17	507.167763	-0.0097071	0.0034064	8.1177427	0.00438341	0.01582537	Tm2d1
ENSMUSG00000028565.19	1397.50529	0.00642512	0.00372779	2.97857307	0.08437363	0.18387105	Nfia
ENSMUSG00000028567.9	561.022914	-0.0028308	0.00175657	2.61837513	0.10563256	0.21899003	Txndc12
ENSMUSG00000028568.17	911.797206	-0.0034596	0.00236724	2.13201497	0.14425102	0.27928949	Btf3l4
ENSMUSG00000028571.16	9.29458488	-0.0026024	0.02624	-0.0090444	1	1	Cyp2j13
ENSMUSG00000028572.14	1239.44642	-0.0018707	0.00336822	0.30205108	0.58259942	0.76018975	Hook1
ENSMUSG00000028573.19	1578.16584	-0.0040504	0.00352748	1.30804236	0.2527494	0.42496061	Fggy

ENSMUSG00000028576.13	110.637893	0.00409422	0.00364672	1.25825219	0.26198219	0.43654289	Ift74
ENSMUSG00000028577.14	957.839881	-0.002296	0.00168918	1.8113621	0.17834492	0.32816689	Plaa
ENSMUSG00000028578.9	94.837072	-0.004119	0.00241757	2.84575016	0.09161582	0.19625687	Caap1
ENSMUSG00000028580.16	836.275268	-0.0022116	0.00213813	1.05413623	0.3045564	0.48929004	Pum1
ENSMUSG00000028581.18	585.652889	0.05549858	0.00470788	131.585729	1.84E-30	4.18E-28	Laptm5
ENSMUSG00000028582.15	207.933915	0.00970895	0.00329963	8.55818334	0.00343972	0.01284113	Cc2d1b
ENSMUSG00000028583.15	12.6930414	0.02149619	0.00712502	8.80055595	0.00301139	0.0114633	Pdpn
ENSMUSG00000028587.19	3.99460823	0.08052001	0.01526611	24.0811536	9.24E-07	7.98E-06	Orc1
ENSMUSG00000028597.12	31.6077633	-0.0075702	0.00697312	1.15630121	0.28223396	0.46208317	Gpx7
ENSMUSG00000028599.11	626.73547	0.00889865	0.00302128	8.67824668	0.00322031	0.01213131	Tnfrsf1b
ENSMUSG00000028600.17	83.9027669	0.01926671	0.00456024	17.8952725	2.33E-05	0.00015373	Podn
ENSMUSG00000028601.19	2629.88303	-0.017778	0.00307491	33.7293263	6.33E-09	7.89E-08	Echdc2
ENSMUSG00000028602.13	5.74517602	0.12302092	0.01478317	55.2362495	1.07E-13	2.53E-12	Tnfrsf8
ENSMUSG00000028603.16	57468.5368	-0.0149888	0.00303498	23.620619	1.17E-06	9.98E-06	Scp2
ENSMUSG00000028607.17	2262.19676	-0.0209764	0.00317543	42.457437	7.22E-11	1.18E-09	Cpt2
ENSMUSG00000028608.16	348.180508	-0.0060386	0.00309261	3.809137	0.05097371	0.12246775	Czib
ENSMUSG00000028609.7	359.894712	-0.0022526	0.00320459	0.49719742	0.48073415	0.6705035	Magoh
ENSMUSG00000028610.17	2.55414406	-0.0606312	0.0560811	0.38888957	0.53288367	0.71742646	Dmrtb1
ENSMUSG00000028613.16	22.9242235	0.04382262	0.01270261	9.59282256	0.00195339	0.007919	Lrp8
ENSMUSG00000028614.15	195.344943	-0.0037577	0.00332717	1.27805522	0.25826095	0.43170395	Ndc1
ENSMUSG00000028617.11	318.687993	0.00017866	0.00311393	0.00332918	0.95398833	1	Lrrc42
ENSMUSG00000028618.12	4017.8023	-0.0013699	0.00230222	0.35277561	0.55254611	0.73475518	Tmem59
ENSMUSG00000028619.16	262.993853	-0.0011199	0.00393308	0.08321317	0.77298934	0.89855725	Tceanc2
ENSMUSG00000028621.18	166.854916	-0.0123896	0.00314868	15.159473	9.88E-05	0.00056507	Cyb5rl
ENSMUSG00000028622.12	622.823396	-0.0155633	0.00248167	39.8080509	2.80E-10	4.21E-09	Mrpl37
ENSMUSG00000028629.10	64.0253348	0.01137474	0.00391383	8.184753	0.00422439	0.01531927	Exo5
ENSMUSG00000028630.10	977.920538	-0.0045863	0.00306357	2.16985017	0.14073995	0.273919	Dyrk2
ENSMUSG00000028631.8	8.25975425	0.01266227	0.00533516	5.70574856	0.01690944	0.04956209	Kcnq4
ENSMUSG00000028633.8	113.912035	-0.0087598	0.00489405	3.17555365	0.07474788	0.16691946	Ctps
ENSMUSG00000028634.18	52.9179866	0.01947243	0.00642397	8.73152279	0.00312755	0.01183102	Hivep3
ENSMUSG00000028636.15	452.225222	-0.0065605	0.00332036	3.95817091	0.04664436	0.1140174	Ppcs
ENSMUSG00000028637.17	85.4016877	-0.0285058	0.00404201	51.2063087	8.32E-13	1.76E-11	Ccdc30
ENSMUSG00000028639.15	4517.84461	-0.0052987	0.00257086	4.19539253	0.04053397	0.10174906	Ybx1
ENSMUSG00000028641.17	238.006229	-0.0128558	0.00302399	18.4611784	1.73E-05	0.00011749	P3h1
ENSMUSG00000028642.8	1.1810778	0.02983291	0.01454988	4.1203961	0.04236916	0.10544676	Tmem269
ENSMUSG00000028643.12	106.642337	0.00742776	0.00420273	3.15535726	0.07567812	0.16853406	Svbp
ENSMUSG00000028644.18	40.5722142	0.08875929	0.01651914	20.2464728	6.81E-06	5.01E-05	Ermap
ENSMUSG00000028645.12	206.962295	0.00755725	0.00346406	4.75039835	0.02929151	0.0779147	Slc2a1
ENSMUSG00000028646.17	843.940226	0.01201879	0.00208553	32.7821561	1.03E-08	1.24E-07	Rragc
ENSMUSG00000028647.14	739.033952	-0.0169199	0.00427259	15.9426858	6.53E-05	0.00039025	Mycbp
ENSMUSG00000028648.14	1386.64852	-0.0143822	0.00405225	12.6066499	0.00038438	0.00189544	Ndufs5
ENSMUSG00000028649.23	1738.52387	0.00665391	0.00231831	8.03074271	0.004599	0.01644761	Macf1
ENSMUSG00000028651.13	198.674428	-0.001334	0.00394896	0.11458832	0.73497968	0.8733788	Ppie
ENSMUSG00000028653.17	205.723998	-0.0141945	0.00235045	36.4712276	1.55E-09	2.09E-08	Trit1
ENSMUSG00000028654.14	174.321372	-0.0022046	0.00462067	0.22395848	0.63604017	0.80335577	Mycl
ENSMUSG00000028655.12	1167.79803	0.0245471	0.0072132	11.3704713	0.00074621	0.00339639	Mfsd2a
ENSMUSG00000028656.15	1248.66685	0.01829757	0.00218437	68.9572341	1.01E-16	3.50E-15	Cap1
ENSMUSG00000028657.15	606.263048	0.0172491	0.002362	52.2934978	4.78E-13	1.05E-11	Ppt1
ENSMUSG00000028664.15	20.8558022	0.08339323	0.0101618	57.5367531	3.32E-14	8.50E-13	Ephb2
ENSMUSG00000028668.6	784.456798	-0.0038099	0.00187673	4.09113747	0.04310862	0.10698232	Eloa
ENSMUSG00000028669.16	382.286295	-0.0042034	0.00266637	2.46878758	0.11612747	0.23593613	Pithd1
ENSMUSG00000028670.15	756.547773	-0.0050959	0.00295009	2.99197075	0.08367827	0.18259189	Lypla2
ENSMUSG00000028671.17	400.856569	-0.0034761	0.00529731	0.41738656	0.51824399	0.70426957	Gale
ENSMUSG00000028672.14	4167.65626	-0.0067476	0.00321572	4.35566732	0.03688601	0.09413475	Hmgcl
ENSMUSG00000028673.11	1556.14629	0.00591281	0.00323651	3.32200547	0.06835839	0.15534262	Fuca1
ENSMUSG00000028675.13	1169.85724	0.00556102	0.00303624	3.24436194	0.07166959	0.1613438	Pnrc2
ENSMUSG00000028676.18	950.925629	-0.0009028	0.00257997	0.11836349	0.73081624	0.87068908	Srsf10
ENSMUSG00000028677.20	331.284529	0.00656905	0.0023765	7.60236857	0.00582917	0.0201623	Rnf220
ENSMUSG00000028678.14	13.0401672	0.01112073	0.01090016	0.91470211	0.33886988	0.52653771	Kif2c
ENSMUSG00000028680.15	753.241502	-0.0113118	0.00632611	3.16498881	0.07523295	0.16776443	Plk3
ENSMUSG00000028681.12	16.3776841	-0.0107452	0.00597901	3.13811086	0.07648235	0.1701002	Ptch2

ENSMUSG00000028683.15	169.250981	-0.0076955	0.00303223	6.50564499	0.01075326	0.03394045	Eif2b3
ENSMUSG00000028684.15	1230.35439	-0.010002	0.00329519	9.24046026	0.00236725	0.00933212	Urod
ENSMUSG00000028687.18	9.676948	0.01434817	0.0071333	4.13629282	0.04197299	0.1046043	Mutyh
ENSMUSG00000028688.14	139.31455	0.00253084	0.00276653	0.84433352	0.35816032	0.54701607	Toe1
ENSMUSG00000028689.15	63.5135565	-0.0140374	0.00375543	14.0268834	0.00018022	0.00097401	Ccdc163
ENSMUSG00000028690.5	262.889145	-0.0098529	0.00292918	11.3573841	0.00075149	0.00341579	Mmachc
ENSMUSG00000028691.13	21311.1122	-0.0086872	0.00269834	10.3417616	0.00130054	0.00552893	Prdx1
ENSMUSG00000028692.15	6566.15358	-0.001133	0.0029157	0.15042105	0.69813331	0.84872802	Akr1a1
ENSMUSG00000028693.16	401.491857	-0.0044864	0.0028685	2.45641022	0.11704601	0.23755676	Nasp
ENSMUSG00000028696.13	195.981187	-0.0111216	0.0021671	26.1246997	3.20E-07	3.03E-06	Ipp
ENSMUSG00000028698.14	60.233602	-0.0020344	0.00526983	0.14324745	0.70507371	0.85347029	Pik3r3
ENSMUSG00000028699.10	3.00268325	0.00571428	0.01840691	0.122678	0.72614793	0.86776026	Tspan1
ENSMUSG00000028700.15	367.492079	-0.0065053	0.00234317	7.7540794	0.00535914	0.01876214	Pomgnt1
ENSMUSG00000028701.5	13.4459629	0.01591874	0.00599497	6.78448408	0.00919536	0.02975875	Lurap1
ENSMUSG00000028702.16	19.5644019	0.03807779	0.00963468	15.2150542	9.59E-05	0.00055054	Rad54l
ENSMUSG00000028703.15	498.818258	-0.0016811	0.00237766	0.50251235	0.47839829	0.66833513	Lrrc41
ENSMUSG00000028706.15	355.212456	-0.0136449	0.00219066	38.8695609	4.53E-10	6.60E-09	Nsun4
ENSMUSG00000028707.16	6.68032116	0.00352917	0.03428382	0.06257981	0.80246396	0.91721424	Dmbx1
ENSMUSG00000028708.17	276.26339	0.00394419	0.00277773	2.02191878	0.15504312	0.2945507	Mknk1
ENSMUSG00000028709.4	250.890872	0.00216633	0.00347727	0.38584106	0.53449366	0.71885541	Mob3c
ENSMUSG00000028710.19	329.809707	-0.0019206	0.00296231	0.42025593	0.51680937	0.70319822	Atpaf1
ENSMUSG00000028712.13	144.145091	-0.0501699	0.00798537	39.7350858	2.91E-10	4.35E-09	Cyp4a31
ENSMUSG00000028713.18	436.275814	0.00830274	0.00409115	4.00537584	0.04535538	0.1114093	Cyp4b1
ENSMUSG00000028715.3	6780.28409	0.00895935	0.00956091	0.81890635	0.36550012	0.5552419	Cyp4a14
ENSMUSG00000028716.16	122.553465	0.0056265	0.00631989	0.80421225	0.36983695	0.56035038	Pdzk1ip1
ENSMUSG00000028717.13	23.065913	0.01415801	0.00481818	8.34163363	0.00387467	0.01422161	Tal1
ENSMUSG00000028718.17	9.80141381	0.02661564	0.01074488	5.71232476	0.01684622	0.04940747	Stil
ENSMUSG00000028719.9	2010.6122	0.00233703	0.00236836	0.96028601	0.32711483	0.5139442	Cmpk1
ENSMUSG00000028729.16	376.026695	-0.0044224	0.00247208	3.20696818	0.07332524	0.16442756	Ebna1bp2
ENSMUSG00000028730.15	2.58308334	-0.0174079	0.02667368	0.29027532	0.59004415	0.76627959	Cfap57
ENSMUSG00000028737.16	7346.28491	-0.0014666	0.00275813	0.27913521	0.59726859	0.7719817	Aldh4a1
ENSMUSG00000028741.14	381.511179	-0.0066835	0.00325981	4.25681616	0.03909313	0.09866779	Mrto4
ENSMUSG00000028743.8	2213.70285	-0.006229	0.00347831	3.17960944	0.07456255	0.16660026	Akr7a5
ENSMUSG00000028744.16	213.432438	0.00308035	0.00255129	1.4748502	0.224582	0.39034569	Slc66a1
ENSMUSG00000028745.19	1618.91394	0.00588811	0.00226156	6.73733243	0.00944157	0.03042613	Capzb
ENSMUSG00000028753.13	7.45403195	0.00856246	0.0316154	0.05329876	0.8174195	0.92610512	Vwa5b1
ENSMUSG00000028755.4	144.577107	0.00599315	0.00452537	1.69293977	0.1932139	0.34872535	Cda
ENSMUSG00000028756.13	3038.33102	-0.0026814	0.00247884	1.15358362	0.28280023	0.4628317	Pink1
ENSMUSG00000028757.5	1875.70087	-0.0004403	0.00239348	0.03377297	0.85419056	0.94992402	Ddost
ENSMUSG00000028758.13	2.10823909	0.02462948	0.01363861	3.55185348	0.05947898	0.13885723	Kif17
ENSMUSG00000028759.14	1779.03671	-0.0032781	0.00213713	2.34712402	0.12551438	0.25085858	Hp1bp3
ENSMUSG00000028760.18	1059.18946	0.00139395	0.00288572	0.23024085	0.6313453	0.79942611	Eif4g3
ENSMUSG00000028763.19	582.603034	0.0023416	0.00503082	0.21660489	0.64163909	0.80777687	Hspg2
ENSMUSG00000028766.11	293.97548	0.00551397	0.00429755	1.6418736	0.2000686	0.35846383	Alpl
ENSMUSG00000028771.14	446.061347	-0.0006232	0.00288066	0.04484944	0.83228111	0.93549374	Ptpn12
ENSMUSG00000028772.20	361.93485	-0.0037486	0.00332369	1.26720536	0.26029161	0.43426737	Zcchc17
ENSMUSG00000028773.9	983.772145	-0.0174819	0.01587781	1.08562711	0.29744214	0.48093989	Fabp3
ENSMUSG00000028776.15	382.442557	-0.0027174	0.00216335	1.59086344	0.20720282	0.36805886	Tinagl1
ENSMUSG00000028779.17	573.20395	0.00172414	0.00218804	0.62302959	0.42992369	0.62141073	Pef1
ENSMUSG00000028780.14	59.8068821	0.00265657	0.00678142	0.15292331	0.69575724	0.84675595	Sema3c
ENSMUSG00000028782.15	7.19144284	0.01720998	0.01352512	1.54885823	0.21330408	0.37592393	Adgrb2
ENSMUSG00000028785.14	28.4888557	0.0809944	0.01203506	39.092921	4.04E-10	5.93E-09	Hpca
ENSMUSG00000028786.16	1.69173522	0.02347711	0.01226796	3.631752	0.05668711	0.13375573	Tmem54
ENSMUSG00000028788.15	3736.30776	0.00705041	0.00247362	7.79732084	0.00523238	0.01838385	Ptp4a2
ENSMUSG00000028789.17	24.3296509	0.01936114	0.00585752	10.9618311	0.00093008	0.00411805	Azin2
ENSMUSG00000028790.14	562.29379	0.00537177	0.00208874	6.55360332	0.0104672	0.03313371	Khdrbs1
ENSMUSG00000028792.15	4326.0545	-0.0121104	0.0035304	11.7368376	0.00061275	0.00285619	Ak2
ENSMUSG00000028793.16	523.984733	0.00940035	0.00238053	15.2565832	9.38E-05	0.00054015	Rnf19b
ENSMUSG00000028795.16	67.090505	-0.0023409	0.00454787	0.26705445	0.60531455	0.77814918	Ccdc28b
ENSMUSG00000028796.18	493.401513	0.00668407	0.0029278	5.13545332	0.02344196	0.06490986	Phc2
ENSMUSG00000028797.21	988.018004	-0.0050519	0.00224034	5.05575511	0.02454428	0.06742389	Tmem234

ENSMUSG00000028798.17	1624.81305	-0.0118884	0.00295307	16.5134016	4.83E-05	0.00029749	Eif3i
ENSMUSG00000028799.16	111.744663	0.00586467	0.00399309	2.16252028	0.14141258	0.27494494	Zfp362
ENSMUSG00000028800.16	559.205934	-0.0005097	0.00345586	0.02173825	0.88278555	0.9654725	Hdac1
ENSMUSG00000028803.19	238.870683	-0.0032382	0.00431056	0.53868514	0.46297816	0.6542189	Nipal3
ENSMUSG00000028804.21	2.7368712	0.01022304	0.01775068	0.49470545	0.48183579	0.67126775	Csmd2
ENSMUSG00000028807.3	38.5946831	0.00710093	0.00354592	4.03624762	0.04453274	0.10969704	Zbtb8a
ENSMUSG00000028809.17	1254.88134	0.00047258	0.00342443	0.01900257	0.89035912	0.9701118	Srrm1
ENSMUSG00000028811.13	527.230141	-0.0084411	0.00242833	11.9542718	0.00054522	0.00258225	Yars
ENSMUSG00000028820.15	1301.3286	0.00402362	0.00293993	1.85727543	0.17293958	0.32044568	Sfpq
ENSMUSG00000028821.9	687.937506	-0.0003456	0.00198558	0.03050899	0.86134034	0.95391409	Syf2
ENSMUSG00000028822.12	1726.16659	0.00204081	0.00338692	0.35883656	0.54915309	0.73180416	Tmem50a
ENSMUSG00000028825.8	1.7520847	0.01860033	0.02973337	0.37447115	0.54057714	0.72427019	Rhd
ENSMUSG00000028826.15	788.958355	-0.0046845	0.00241196	3.7587832	0.05253079	0.12551831	Maco1
ENSMUSG00000028830.15	469.72155	-0.0044778	0.00260356	2.92924485	0.08698817	0.18838518	AU040320
ENSMUSG00000028832.12	203.786662	0.01025804	0.00645016	2.42796445	0.11918758	0.24099084	Stmn1
ENSMUSG00000028833.14	214.059514	0.00270891	0.00392509	0.46684372	0.4944428	0.68186851	Ncdn
ENSMUSG00000028834.14	35.6392527	0.00294399	0.02168985	0.08914399	0.76526823	0.89358961	Trim63
ENSMUSG00000028836.15	2.0156078	0.02150892	0.02077283	1.31703139	0.25112552	0.42286332	Slc30a2
ENSMUSG00000028837.9	1157.64027	-0.0068526	0.00226532	9.19486262	0.00242695	0.00952923	Psmb2
ENSMUSG00000028838.12	115.263087	-0.0015664	0.0064721	0.05722544	0.81093624	0.92218936	Extl1
ENSMUSG00000028840.11	101.990706	-0.0035387	0.00400126	0.77931476	0.37735079	0.56855729	Zfp593
ENSMUSG00000028841.15	4.62205966	0.01484506	0.00911867	2.5156506	0.1127211	0.23050459	Cnksr1
ENSMUSG00000028842.16	294.842216	-0.0115809	0.00433652	6.98014482	0.00824189	0.02708937	ago-03
ENSMUSG00000028843.9	172.152549	0.05551538	0.00534949	101.685578	6.51E-24	5.35E-22	Sh3bgrl3
ENSMUSG00000028845.16	4.55057856	-0.007023	0.01021361	0.51102291	0.47469638	0.66529139	Tekt2
ENSMUSG00000028847.9	523.823542	-0.0048342	0.00210387	5.32751646	0.02099128	0.05923939	Trappc3
ENSMUSG00000028848.14	132.104455	-0.0059106	0.00277686	4.5695105	0.03254586	0.08507445	Gpn2
ENSMUSG00000028849.18	872.156378	-0.0027604	0.00327311	0.69182856	0.40554312	0.59714945	Map7d1
ENSMUSG00000028850.3	79.4470292	-0.0059026	0.00410983	2.02660913	0.15456513	0.29399783	Gpatch3
ENSMUSG00000028851.7	915.748142	-0.0072753	0.00285481	6.37078173	0.01160145	0.03608014	Nudc
ENSMUSG00000028854.10	177.850771	0.0053435	0.00356912	2.2571599	0.13299778	0.26248334	Slc9a1
ENSMUSG00000028857.17	363.128225	0.00334854	0.00275467	1.49453007	0.22151492	0.386634	Tmem222
ENSMUSG00000028859.15	63.6672288	0.06112988	0.00606871	94.2445725	2.79E-22	1.92E-20	Csf3r
ENSMUSG00000028860.14	108.890002	0.01930128	0.00416339	20.057144	7.52E-06	5.46E-05	Sytl1
ENSMUSG00000028861.14	410.998402	-0.0075695	0.00293393	6.70390289	0.00962021	0.03089207	Mrps15
ENSMUSG00000028862.7	32.1247437	0.00758224	0.00736675	1.022433	0.31194259	0.49716048	Map3k6
ENSMUSG00000028863.14	171.572502	-0.0050961	0.00260627	3.76227484	0.05242121	0.12529456	Meaf6
ENSMUSG00000028864.8	217.484025	0.01456017	0.00477475	8.8411944	0.00294506	0.01124895	Hgf
ENSMUSG00000028868.14	1010.25717	0.00031274	0.00520301	0.00337187	0.95369459	1	Wasf2
ENSMUSG00000028869.14	822.58768	-0.0003733	0.00331887	0.01272524	0.91018421	0.9809278	Gnl2
ENSMUSG00000028871.8	10.4732598	0.00040631	0.00851313	0.00229026	0.96183046	1	Rspo1
ENSMUSG00000028873.17	21.0096367	0.00325576	0.00752066	0.18057189	0.67088223	0.82880174	Cdca8
ENSMUSG00000028874.15	80.2689219	0.06300245	0.00621243	94.2750458	2.75E-22	1.90E-20	Fgr
ENSMUSG00000028876.8	1.9561629	0.00741686	0.0242784	0.17366298	0.67687671	0.83293233	Epha10
ENSMUSG00000028878.12	343.529179	0.00931028	0.00238988	15.0297145	0.00010583	0.00060177	Fam76a
ENSMUSG00000028879.5	501.671292	0.00076843	0.00280895	0.074167	0.78536353	0.90711375	Stx12
ENSMUSG00000028882.14	208.233673	0.0017052	0.00252615	0.45301164	0.50090823	0.68782008	Ppp1r8
ENSMUSG00000028883.20	10.7127015	4.47E-05	0.00846016	9.26E-05	0.99232157	1	Sema3a
ENSMUSG00000028884.15	110.908313	0.01356763	0.00379145	12.621313	0.00038137	0.00188103	Rpa2
ENSMUSG00000028885.9	12.0707113	0.05430562	0.00628348	73.0397612	1.27E-17	4.94E-16	Smpdl3b
ENSMUSG00000028886.16	329.604998	-0.0039293	0.00329959	1.39950145	0.23680706	0.40585172	Eya3
ENSMUSG00000028889.18	290.993708	-0.014648	0.00334837	19.6959082	9.08E-06	6.50E-05	Yrdc
ENSMUSG00000028890.14	400.669458	-0.0052922	0.00312857	2.84334605	0.09175297	0.19648497	Mtf1
ENSMUSG00000028893.9	148.200221	0.00559216	0.00349128	2.58167912	0.10810693	0.22310001	Sesn2
ENSMUSG00000028894.19	700.662838	0.00784574	0.00325744	5.79121754	0.01610643	0.04758095	Inpp5b
ENSMUSG00000028896.14	127.011264	0.00791958	0.00377545	4.29899068	0.038135	0.09666331	Rcc1
ENSMUSG00000028898.16	364.922696	0.00185691	0.00377839	0.24505525	0.62057855	0.79080262	Trnau1ap
ENSMUSG00000028899.12	319.16725	-0.0130473	0.00288467	19.9077424	8.13E-06	5.86E-05	Taf12
ENSMUSG00000028901.14	125.92332	-0.0021564	0.00280286	0.57117033	0.44979423	0.64121165	Gmeb1
ENSMUSG00000028902.5	312.801756	0.00276966	0.00183878	2.27355117	0.13159805	0.26034824	Sf3a3
ENSMUSG00000028906.18	2213.50063	-0.0009178	0.00287799	0.10066755	0.75103002	0.884319	Epb41

ENSMUSG00000028907.8	389.714165	0.00112536	0.00223361	0.25296004	0.61499849	0.78656557	Utp11
ENSMUSG00000028909.18	52.7883235	0.017063	0.00726826	5.35844034	0.02062221	0.05846802	Ptpru
ENSMUSG00000028910.13	365.345343	-0.018099	0.0026945	45.8378135	1.28E-11	2.34E-10	Mecr
ENSMUSG00000028911.17	640.637718	-0.004101	0.00239065	2.9712995	0.08475375	0.18461414	Srsf4
ENSMUSG00000028914.14	384.386214	0.0032967	0.0028187	1.34778607	0.24566553	0.4166739	Casp9
ENSMUSG00000028917.15	245.702181	0.01692869	0.00366508	20.4531183	6.11E-06	4.54E-05	Plekhm2
ENSMUSG00000028919.12	875.802559	0.00137214	0.0034627	0.15267037	0.69599641	0.84691592	Arhgef19
ENSMUSG00000028920.10	215.424336	-0.0033439	0.00289065	1.34270701	0.24655714	0.41785563	Fbxo42
ENSMUSG00000028923.15	158.91846	0.01803924	0.0036005	24.5155832	7.37E-07	6.47E-06	Necap2
ENSMUSG00000028926.16	72.0178367	0.04835631	0.00585995	62.1838103	3.13E-15	9.12E-14	Cdk14
ENSMUSG00000028927.7	13.7435263	0.0728032	0.00751204	90.9028266	1.51E-21	9.44E-20	Padi2
ENSMUSG00000028931.13	50.4962368	0.07458391	0.00810292	77.514981	1.32E-18	5.72E-17	Kcnab2
ENSMUSG00000028932.9	1529.23803	-0.0076553	0.00182596	17.5571955	2.79E-05	0.00018075	Psmc2
ENSMUSG00000028933.12	33.7166107	0.00181367	0.00423798	0.17853746	0.67263315	0.83015618	Xrcc2
ENSMUSG00000028936.16	3819.44962	-0.0015562	0.0041289	0.1414446	0.70684909	0.85456674	Rpl22
ENSMUSG00000028937.15	322.763946	-0.0015494	0.0027115	0.32655839	0.56769269	0.74745955	Acot7
ENSMUSG00000028938.10	2.18842893	-0.150292	0.1293542	0.04728657	0.82785397	0.93249935	Galntl5
ENSMUSG00000028943.19	18.3809371	0.01273718	0.00738537	3.08462411	0.07903552	0.17444618	Espn
ENSMUSG00000028944.15	508.692949	0.00823602	0.00302219	7.46692569	0.00628428	0.02150311	Prkag2
ENSMUSG00000028945.10	827.348845	-0.0068772	0.00243098	7.89582263	0.00495491	0.01755016	Rheb
ENSMUSG00000028948.17	314.568172	-0.0097234	0.0022785	18.1811137	2.01E-05	0.00013432	Nol9
ENSMUSG00000028949.14	65.079255	0.00238364	0.00553793	0.184256	0.66774093	0.82637653	Smarcd3
ENSMUSG00000028952.13	203.138032	-0.009074	0.00406703	5.02568929	0.02497398	0.06837335	Zbtb48
ENSMUSG00000028953.11	635.890755	-0.0079123	0.00193889	16.8051679	4.14E-05	0.00025969	Abcf2
ENSMUSG00000028954.12	661.656944	0.00255972	0.00145401	3.07143199	0.07967926	0.17558768	Nub1
ENSMUSG00000028955.4	329.480518	0.01023242	0.00323485	9.75183198	0.00179144	0.00733049	Vamp3
ENSMUSG00000028957.13	361.611904	0.02991003	0.00881224	10.2517813	0.00136553	0.00576572	Per3
ENSMUSG00000028958.16	120.047389	-0.0015419	0.00334098	0.21056253	0.64632686	0.81099459	Tmub1
ENSMUSG00000028959.15	543.692175	-0.0103396	0.00306463	11.7198495	0.00061837	0.00288011	Fastk
ENSMUSG00000028960.21	957.019652	-0.0060084	0.00325034	3.35172075	0.0671348	0.15309305	Ube4b
ENSMUSG00000028961.16	917.764613	0.01764799	0.00311683	31.4954077	2.00E-08	2.31E-07	Pgd
ENSMUSG00000028962.15	502.449114	-0.0016108	0.00243155	0.44193993	0.50618762	0.6930619	Slc4a2
ENSMUSG00000028964.15	2252.5626	-0.005456	0.00264988	4.25142627	0.03921738	0.09894963	Park7
ENSMUSG00000028965.14	4.81514687	0.1225735	0.0146155	53.7791326	2.24E-13	5.15E-12	Tnfrsf9
ENSMUSG00000028967.11	16926.7627	-0.0104125	0.00440426	5.53249147	0.01866651	0.0537765	Errfi1
ENSMUSG00000028969.11	301.658148	-0.0051243	0.00256584	4.02602387	0.04480342	0.11026016	Cdk5
ENSMUSG00000028970.10	67.7006784	-0.0110457	0.00694542	2.33915246	0.12615818	0.25182522	Abcb1b
ENSMUSG00000028973.19	499.936686	-0.0100313	0.00353229	8.2235962	0.00413492	0.01503374	Abcb8
ENSMUSG00000028974.14	280.82066	-0.0081007	0.00199709	16.525813	4.80E-05	0.00029601	Dffa
ENSMUSG00000028975.17	905.868106	-0.0094768	0.00232944	16.5095149	4.84E-05	0.00029802	Pex14
ENSMUSG00000028976.11	124.212466	0.01025511	0.00631382	2.61404118	0.10592153	0.21939626	Slc2a5
ENSMUSG00000028977.18	31.9005033	0.01841496	0.00630389	8.02865128	0.00460431	0.01646411	Casz1
ENSMUSG00000028978.13	62.8542643	-0.0041838	0.00350423	1.43306812	0.23126437	0.39893231	Nos3
ENSMUSG00000028979.18	3916.63223	-0.0130884	0.00401113	10.6516127	0.00109975	0.00477429	Masp2
ENSMUSG00000028980.15	7088.00337	-0.0117953	0.0038308	9.02504065	0.00266306	0.01032226	H6pd
ENSMUSG00000028982.10	413.844411	-0.0245065	0.00364016	44.7299113	2.26E-11	3.98E-10	Slc25a33
ENSMUSG00000028986.13	322.148018	-0.0020013	0.00277299	0.49793611	0.48040839	0.67023023	Klhl7
ENSMUSG00000028988.14	228.132394	-0.0009811	0.00306763	0.10366928	0.74747001	0.88157954	Ctnnbip1
ENSMUSG00000028989.4	11.6435885	0.0256918	0.00751117	11.1267287	0.00085093	0.00380761	Angptl7
ENSMUSG00000028990.14	103.267334	-0.0006757	0.00416381	0.02617322	0.871478	0.95951266	Lzic
ENSMUSG00000028991.16	812.3157	0.00220151	0.00319433	0.46853347	0.49366262	0.68114698	Mtor
ENSMUSG00000028992.14	357.008802	-0.0195704	0.00305181	41.0233094	1.50E-10	2.35E-09	Nmnat1
ENSMUSG00000028995.15	252.03676	0.02839982	0.00371402	54.7346896	1.38E-13	3.23E-12	Hycc1
ENSMUSG00000028996.10	2.61136286	0.01488681	0.0188984	0.75454751	0.38504027	0.5766802	Rbp7
ENSMUSG00000028998.7	1022.5785	-0.0115485	0.00383472	9.12721474	0.00251835	0.0098357	Tomm7
ENSMUSG00000028999.16	338.286769	-0.0198292	0.00375692	27.2485735	1.79E-07	1.78E-06	Rint1
ENSMUSG00000029001.16	51.0262577	-0.0005783	0.00360668	0.02573523	0.87254863	0.96001922	Fbxo44
ENSMUSG00000029003.12	257.802832	-0.0002649	0.00337977	0.00624535	0.93701074	0.99546575	Mad2l2
ENSMUSG00000029004.16	1688.43478	0.00427916	0.00460084	0.85835801	0.3541986	0.54279307	Kmt2e
ENSMUSG00000029007.9	145.713491	0.02436682	0.00458655	27.9798882	1.23E-07	1.26E-06	Agtrap
ENSMUSG00000029009.18	266.357931	-0.0068461	0.00613187	1.15922905	0.28162549	0.46140725	Mthfr

ENSMUSG00000029012.12	266.910853	0.00217585	0.00186121	1.36972871	0.2418585	0.4119536	Orc5
ENSMUSG00000029014.15	1386.80862	0.00269142	0.00320701	0.68877942	0.40657986	0.59822785	Dnajc2
ENSMUSG00000029016.14	110.637517	0.01493241	0.00424423	12.3079714	0.00045103	0.00217946	Clcn6
ENSMUSG00000029017.14	2265.23291	-0.010195	0.00265343	14.7529282	0.00012256	0.00068726	Pmpcb
ENSMUSG00000029019.9	25.918222	0.02387928	0.0573413	-0.1323207	1	1	Nppb
ENSMUSG00000029020.14	1926.36589	-0.0084491	0.00318025	6.98173884	0.00823456	0.02707657	Mfn2
ENSMUSG00000029022.19	253.740807	-0.0040592	0.00478213	0.73226118	0.39215123	0.58341945	Miip
ENSMUSG00000029027.10	51.1213277	-0.0060644	0.00329809	3.41903712	0.06444859	0.14818527	Dffb
ENSMUSG00000029028.15	426.798247	-0.0066335	0.00272757	5.85148307	0.01556393	0.04625641	Lrrc47
ENSMUSG00000029029.15	140.764874	-0.0018509	0.00342379	0.29582348	0.58651265	0.76333822	Wrap73
ENSMUSG00000029030.15	814.122599	0.00080117	0.0020918	0.14556057	0.70281445	0.85165054	Tprgl
ENSMUSG00000029032.13	95.7094923	0.00814495	0.00468759	3.14786206	0.07602651	0.16921406	Arhgef16
ENSMUSG00000029033.17	75.5827686	-0.0020193	0.00424641	0.22369845	0.63623622	0.8034194	Acap3
ENSMUSG00000029034.10	320.030178	-0.0054737	0.00240393	5.17114597	0.02296506	0.06384417	Ints11
ENSMUSG00000029036.19	930.941631	-0.01165	0.00273716	18.3852242	1.80E-05	0.00012181	Atad3a
ENSMUSG00000029038.10	928.681192	-0.0051703	0.00231298	4.99803955	0.02537605	0.06934532	Ssu72
ENSMUSG00000029047.14	97.9420604	-0.0054849	0.00307215	3.15195914	0.07583586	0.16883751	Pex10
ENSMUSG00000029048.4	2018.09478	-0.0041979	0.00210738	3.99231092	0.04570833	0.11215688	Rer1
ENSMUSG00000029049.15	31.925178	-0.0198961	0.00459086	19.0498972	1.27E-05	8.85E-05	Morn1
ENSMUSG00000029050.16	566.589642	0.01184531	0.00276328	18.2591911	1.93E-05	0.00012929	Ski
ENSMUSG00000029053.17	348.163628	-0.0136142	0.00364223	13.8575672	0.0001972	0.00105336	Prkcz
ENSMUSG00000029054.9	6.12955696	-0.0017699	0.01157276	0.01938688	0.88926303	0.96954458	Gabrd
ENSMUSG00000029055.18	4.19982918	0.0121448	0.01230718	0.92123697	0.33715039	0.52447556	Plch2
ENSMUSG00000029056.14	199.613234	-0.0072429	0.00235258	9.45215024	0.00210902	0.00846838	Pank4
ENSMUSG00000029059.10	257.82583	-0.0137955	0.00517788	7.11495541	0.00764435	0.02540863	Prxl2b
ENSMUSG00000029060.18	340.315183	-0.0050978	0.00254571	4.00400423	0.0453923	0.11148837	Mib2
ENSMUSG00000029061.3	26.803828	0.0373211	0.00575179	40.0148317	2.52E-10	3.81E-09	Mmp23
ENSMUSG00000029062.15	772.966089	0.00261993	0.00313653	0.6990339	0.40310851	0.59467834	Cdk11b
ENSMUSG00000029063.17	3819.33182	0.00338773	0.00197925	2.8674857	0.09038597	0.1940686	Nadk
ENSMUSG00000029064.16	1602.41633	0.01175516	0.00223012	27.374536	1.68E-07	1.67E-06	Gnb1
ENSMUSG00000029066.13	739.865727	-0.0154942	0.00342197	20.9214901	4.78E-06	3.63E-05	Mrpl20
ENSMUSG00000029068.17	1840.34889	-0.010567	0.00346975	9.31928001	0.00226755	0.00900985	Ccnl2
ENSMUSG00000029070.10	156.877091	0.00797269	0.00493561	2.63667988	0.10442156	0.21703269	Mxra8
ENSMUSG00000029071.17	1031.94685	-0.0071654	0.00264338	7.49369926	0.00619152	0.0212412	Dvl1
ENSMUSG00000029072.4	4.58948601	0.00762103	0.00728914	1.12579503	0.28867405	0.46982239	Tas1r3
ENSMUSG00000029073.10	299.010047	-0.0004246	0.00243219	0.03089875	0.86046646	0.953621	Cptp
ENSMUSG00000029074.15	4.34920389	0.01304019	0.01069304	1.45268483	0.22809764	0.39468204	Ttll10
ENSMUSG00000029075.7	6.50806974	0.04817818	0.00948717	22.3593419	2.26E-06	1.83E-05	Tnfrsf4
ENSMUSG00000029076.15	2343.17658	0.00230035	0.00249104	0.84813498	0.35708048	0.54582633	Sdf4
ENSMUSG00000029082.18	17.3169514	0.04457976	0.00897292	20.8480626	4.97E-06	3.76E-05	Bst1
ENSMUSG00000029084.6	373.502373	0.02821422	0.00405498	45.4497856	1.57E-11	2.82E-10	Cd38
ENSMUSG00000029086.16	66.3070547	0.04580285	0.00793327	29.3843491	5.94E-08	6.37E-07	Prom1
ENSMUSG00000029088.17	4.10017522	0.03599855	0.01305062	7.78249205	0.0052755	0.01852708	Kcnip4
ENSMUSG00000029089.9	114.837441	0.00136131	0.00313012	0.18808818	0.6645126	0.82434093	5730480H06Rik
ENSMUSG00000029090.13	883.688915	0.00823386	0.00455233	3.13375843	0.07668676	0.17044231	Adgra3
ENSMUSG00000029093.15	8.57093466	0.04274465	0.01036689	16.4649064	4.96E-05	0.00030408	Sorcs2
ENSMUSG00000029094.13	57.5779912	0.02475573	0.00479702	25.463809	4.51E-07	4.15E-06	Afap1
ENSMUSG00000029095.18	59.1906711	0.01215512	0.00522817	5.29118209	0.02143367	0.06026159	Ablim2
ENSMUSG00000029096.16	54.4376351	0.03551372	0.00679672	25.2833754	4.95E-07	4.51E-06	Htra3
ENSMUSG00000029097.12	58.6896949	0.0022509	0.00280219	0.64386573	0.42231442	0.61394895	Trmt44
ENSMUSG00000029098.18	527.971303	-0.013255	0.00275573	23.357042	1.35E-06	1.13E-05	Acox3
ENSMUSG00000029101.17	112.90814	0.02926963	0.00508422	32.1402078	1.43E-08	1.70E-07	Rgs12
ENSMUSG00000029102.10	3731.75007	-0.0030242	0.00462006	0.42783331	0.51305403	0.69975668	Hgfac
ENSMUSG00000029103.17	818.429732	-0.0025103	0.00246347	1.04100804	0.30758714	0.4926472	Lrpap1
ENSMUSG00000029104.16	447.355639	0.00253593	0.0031235	0.66186871	0.41590099	0.60715387	Htt
ENSMUSG00000029106.15	752.788973	0.01263492	0.00255268	24.6195014	6.98E-07	6.17E-06	Add1
ENSMUSG00000029108.15	67.896614	0.02117549	0.0062362	10.9458506	0.00093813	0.0041506	Pcdh7
ENSMUSG00000029110.14	1614.54826	-0.0036863	0.00195554	3.55184934	0.05947912	0.13885723	Rnf4
ENSMUSG00000029111.8	199.410234	-0.0086868	0.00216894	16.0947528	6.03E-05	0.0003628	Nelfa
ENSMUSG00000029119.10	869.384276	0.0137376	0.00285458	22.8263629	1.77E-06	1.47E-05	Man2b2
ENSMUSG00000029120.11	32.5606846	0.03653669	0.01527327	6.20993854	0.0127035	0.03894266	Ppp2r2c

ENSMUSG00000029121.17	7.00280935	0.08975106	0.01265838	44.8649189	2.11E-11	3.73E-10	Crmp1
ENSMUSG00000029122.12	59.1382243	0.02574725	0.00615054	16.4242489	5.06E-05	0.00030979	Evc
ENSMUSG00000029123.9	1.62148473	0.04955797	0.02539486	3.71794859	0.05383047	0.1281564	Stk32b
ENSMUSG00000029125.15	298.274057	-0.0008856	0.00257396	0.11796165	0.73125584	0.87096647	Stx18
ENSMUSG00000029126.8	41.3839458	0.02036813	0.00645239	9.29950254	0.00229216	0.00907857	Nsg1
ENSMUSG00000029127.16	43.6845505	-0.0009528	0.00378339	0.06373333	0.8006897	0.91618159	Zbtb49
ENSMUSG00000029128.13	302.248237	-0.0032015	0.00301697	1.10997739	0.29208686	0.47400308	Rab28
ENSMUSG00000029130.13	13.9534333	-0.0023528	0.00901667	0.0652915	0.79831994	0.91521751	Rnf32
ENSMUSG00000029131.15	1359.39335	-0.0052747	0.00218137	5.81063332	0.01592956	0.04714109	Dnajb6
ENSMUSG00000029134.15	1.86171221	-0.0240627	0.01492491	2.3136271	0.12824449	0.25531987	Plb1
ENSMUSG00000029135.11	200.159653	0.00850336	0.00426263	3.73712509	0.05321593	0.12684699	Fosl2
ENSMUSG00000029136.10	186.200835	7.95E-06	0.00267901	9.98E-06	0.9974794	1	Rbks
ENSMUSG00000029141.17	296.66979	-0.0063483	0.00274624	5.3143904	0.02114999	0.05961326	Slc4a1ap
ENSMUSG00000029145.13	550.575952	-0.014001	0.00405306	11.7339001	0.00061372	0.00286014	Eif2b4
ENSMUSG00000029146.15	271.806162	0.00478834	0.00296153	2.60343199	0.10663258	0.22068456	Snx17
ENSMUSG00000029147.12	793.719736	-0.0062694	0.00202725	9.64058666	0.00190324	0.00774365	Ppm1g
ENSMUSG00000029148.16	803.072166	-0.006077	0.00235301	6.68154066	0.00974164	0.03119705	Nrbp1
ENSMUSG00000029149.15	15.3484161	-0.0058881	0.00595143	0.99623207	0.31822396	0.50400418	Krtcap3
ENSMUSG00000029151.15	12.7330018	0.00310838	0.00793804	0.15394252	0.69479586	0.84615352	Slc30a3
ENSMUSG00000029152.14	1767.22666	-0.0065311	0.0021749	8.98994882	0.00271469	0.01049477	Ociad1
ENSMUSG00000029153.12	850.170422	-0.0125102	0.00406924	9.24326597	0.00236363	0.00931939	Ociad2
ENSMUSG00000029154.14	1.51192428	0.01314026	0.03606899	0.3475843	0.5554838	0.737705	Cwh43
ENSMUSG00000029155.18	1.33763931	-0.0225164	0.04704781	0.29140303	0.58932283	0.76586965	Spata18
ENSMUSG00000029156.12	92.8680558	0.01087855	0.00462187	5.23266779	0.02216651	0.06198915	Sgcb
ENSMUSG00000029158.10	24.7402527	0.11511662	0.04216152	-2.4399694	1	1	Yipf7
ENSMUSG00000029161.9	32.6949658	0.0244217	0.0078106	9.13055473	0.00251375	0.00982101	Cgref1
ENSMUSG00000029162.16	7890.89298	-0.010095	0.00483252	4.35699297	0.03685731	0.09408187	Khk
ENSMUSG00000029163.10	397.99122	0.01139445	0.0037635	9.22714017	0.00238454	0.00938927	Emilin1
ENSMUSG00000029165.18	199.250255	-0.0040272	0.00422095	0.93557009	0.33341971	0.52030906	Agbl5
ENSMUSG00000029166.15	544.344745	-0.0031251	0.00314235	0.9743541	0.32359667	0.51031248	Mapre3
ENSMUSG00000029167.14	289.073365	0.00947419	0.00497341	3.76257284	0.05241187	0.12528493	Ppargc1a
ENSMUSG00000029168.15	3.89495062	0.02659736	0.01283301	4.37997168	0.0363636	0.09315433	Dpysl5
ENSMUSG00000029169.12	1211.18524	-0.0010897	0.00217173	0.24623086	0.61974164	0.79011701	Dhx15
ENSMUSG00000029171.13	109.996629	0.01104494	0.00475303	5.34905041	0.02073356	0.058692	Pgm2
ENSMUSG00000029173.13	1211.3519	-0.0042132	0.00261402	2.5922171	0.10738992	0.22193101	Sepsecs
ENSMUSG00000029174.19	185.301304	0.0232483	0.00335522	46.2021972	1.07E-11	1.97E-10	Tbc1d1
ENSMUSG00000029175.18	377.667886	-0.0010084	0.00239749	0.17603421	0.67480377	0.8315963	Slc35f6
ENSMUSG00000029176.14	329.744224	0.01033544	0.00288106	12.877763	0.00033251	0.00166828	Anapc4
ENSMUSG00000029177.10	139.594793	-0.0165483	0.00509001	10.4477975	0.00122796	0.00526689	Cenpa
ENSMUSG00000029178.16	1235.29534	0.00168785	0.00307697	0.29605096	0.58636877	0.76323869	Klf3
ENSMUSG00000029179.15	126.451252	0.00646278	0.00322637	3.96560867	0.04643873	0.11358539	Zcchc4
ENSMUSG00000029185.15	572.049113	0.00248004	0.00316937	0.6033363	0.4373079	0.62896177	Fam114a1
ENSMUSG00000029186.13	547.540878	-0.0111854	0.00237905	21.5832562	3.39E-06	2.66E-05	Pi4k2b
ENSMUSG00000029188.15	40.8796189	-0.0158581	0.00931208	2.57683554	0.10843825	0.22366634	Slc34a2
ENSMUSG00000029189.11	401.514429	-0.0262709	0.00523152	24.9016871	6.03E-07	5.42E-06	Sel1l3
ENSMUSG00000029190.20	1005.39866	0.00448502	0.00270991	2.68429635	0.10134198	0.21239133	D5Ertd579e
ENSMUSG00000029191.17	553.905968	0.00436904	0.00342474	1.59552047	0.20653918	0.36722628	Rfc1
ENSMUSG00000029192.18	347.062912	0.00489063	0.00249508	3.77053083	0.05216308	0.12476608	Tbc1d14
ENSMUSG00000029193.8	11.1275766	-0.0236514	0.02358032	1.53159503	0.21587322	0.37928976	Cckar
ENSMUSG00000029195.11	536.639668	0.00423711	0.00449196	0.89826989	0.34324604	0.53132357	Klb
ENSMUSG00000029196.10	435.882609	-0.0049827	0.00263713	3.58382322	0.05834473	0.13682246	Tada2b
ENSMUSG00000029198.4	2540.50782	-0.0158839	0.00291561	30.1880475	3.92E-08	4.32E-07	Grpel1
ENSMUSG00000029199.12	647.667778	-0.005227	0.00348321	2.23077242	0.1352862	0.26572788	Lias
ENSMUSG00000029201.15	3538.69035	0.01415734	0.00400304	11.6005285	0.00065933	0.00305041	Ugdh
ENSMUSG00000029202.13	989.645101	-0.0010371	0.00195043	0.27786892	0.59810141	0.77259543	Pds5a
ENSMUSG00000029203.17	907.872703	-0.0077002	0.00230376	11.0658087	0.00087934	0.00391841	Ube2k
ENSMUSG00000029204.8	28.7231085	0.07812733	0.00671572	119.317543	8.92E-28	1.27E-25	Rhoh
ENSMUSG00000029206.14	9.22718085	-0.0052427	0.00894995	0.3430881	0.55805211	0.73978289	Nsun7
ENSMUSG00000029207.18	298.464806	0.00189968	0.00395914	0.22774412	0.6332016	0.8010885	Apbb2
ENSMUSG00000029208.17	344.149386	-0.0044371	0.00228774	3.75913858	0.05251963	0.12550435	Guf1
ENSMUSG00000029209.16	84.6967634	0.02341051	0.00378518	37.7476022	8.05E-10	1.13E-08	Gnpda2

ENSMUSG00000029211.12	6.11165726	0.02097041	0.01730624	1.46954019	0.22541826	0.39145302	Gabra4
ENSMUSG00000029212.12	13.529966	0.00958383	0.01453111	0.54100397	0.46201695	0.65322693	Gabrb1
ENSMUSG00000029213.12	449.893419	0.00332103	0.00278476	1.41121091	0.23485548	0.40328997	Commd8
ENSMUSG00000029217.17	355.494772	-0.0087416	0.0028069	9.80134118	0.00174385	0.00715809	Tec
ENSMUSG00000029221.16	1187.47009	-0.0066236	0.00237953	7.74661994	0.00538132	0.01882304	Slc30a9
ENSMUSG00000029223.13	17.298772	0.03830638	0.01537737	5.77955425	0.01621365	0.04783778	Uchl1
ENSMUSG00000029227.16	745.254841	-0.0009595	0.0022867	0.17407529	0.67651508	0.83279147	Fip1l1
ENSMUSG00000029228.16	15.3159295	0.03206903	0.00801396	15.7637766	7.18E-05	0.00042447	Lnx1
ENSMUSG00000029229.9	355.743585	-0.0004508	0.00383772	0.01388239	0.90620747	0.97905006	Chic2
ENSMUSG00000029231.16	175.81367	0.02869106	0.00463625	36.124394	1.85E-09	2.47E-08	Pdgfra
ENSMUSG00000029233.16	176.306587	0.00815631	0.00274934	8.78521187	0.00303682	0.01154149	Srd5a3
ENSMUSG00000029234.14	177.466025	0.00849145	0.00289479	8.47313369	0.0036043	0.01336651	Tmem165
ENSMUSG00000029235.15	5.36701748	0.13623059	0.12266627	-0.5304773	1	1	Pdcl2
ENSMUSG00000029238.12	1012.46225	-0.0157513	0.00416174	13.7914648	0.00020426	0.00108654	Clock
ENSMUSG00000029245.17	12.5873163	0.0270975	0.0162863	3.23216774	0.07220505	0.16236327	Epha5
ENSMUSG00000029246.15	448.767893	-0.0011324	0.00300259	0.14505495	0.70330654	0.85194642	Ppat
ENSMUSG00000029247.18	4119.05775	-0.0105215	0.0023314	20.5966986	5.67E-06	4.24E-05	Paics
ENSMUSG00000029249.16	493.732759	-0.0015327	0.00350441	0.18565913	0.66655431	0.82570116	Rest
ENSMUSG00000029250.6	834.95254	-0.0105272	0.00350547	9.0025247	0.00269607	0.01043477	Polr2b
ENSMUSG00000029253.13	90.5332683	0.00351272	0.00278309	1.55637452	0.21219685	0.37447624	Cenpc1
ENSMUSG00000029254.17	57.0293376	0.05768614	0.00636773	76.7578653	1.93E-18	8.21E-17	Stap1
ENSMUSG00000029260.16	8940.95603	-0.0095063	0.00461721	4.1605951	0.04137486	0.10344113	Ugt2b34
ENSMUSG00000029263.16	253.24697	-0.0059849	0.0024029	6.18624157	0.01287473	0.03939077	Pigg
ENSMUSG00000029265.5	452.947483	0.00575264	0.00234808	5.91195899	0.01503844	0.04496793	Dr1
ENSMUSG00000029267.18	208.104336	0.00087048	0.00322388	0.07293141	0.7871155	0.90826352	Mtf2
ENSMUSG00000029268.15	0.51582497	-0.0107943	0.02717791	0.14106292	0.7072266	0.85476027	Ugt2a2
ENSMUSG00000029269.12	656.098196	0.00467389	0.00476378	0.96820902	0.32512725	0.51188126	Sult1b1
ENSMUSG00000029270.11	225.259703	0.00314733	0.00329982	0.91757708	0.33811196	0.52568943	Dipk1a
ENSMUSG00000029272.9	22.4071561	0.17402025	0.01332905	123.879937	8.95E-29	1.44E-26	Sult1e1
ENSMUSG00000029273.11	3319.04023	0.00156395	0.00535248	0.08668012	0.7684409	0.89579922	Sult1d1
ENSMUSG00000029275.19	3.41250484	0.10384861	0.01134074	72.4888148	1.68E-17	6.45E-16	Gfi1
ENSMUSG00000029276.14	84.8985705	0.00202027	0.00355111	0.32212487	0.57033347	0.74990998	Glmn
ENSMUSG00000029279.16	49.4700856	-0.0128609	0.00516787	6.16015644	0.01306597	0.03988273	Brdt
ENSMUSG00000029283.18	12.3057798	0.04459587	0.00864751	25.7109867	3.97E-07	3.69E-06	Cdc7
ENSMUSG00000029287.15	258.769029	0.00148157	0.00339556	0.18957439	0.66327108	0.82318321	Tgfbr3
ENSMUSG00000029290.13	358.433591	-0.0009035	0.00401681	0.04893779	0.82492202	0.93068519	Zfp326
ENSMUSG00000029291.12	907.255054	-0.0017812	0.00234589	0.57355972	0.44884784	0.64071446	Rufy3
ENSMUSG00000029298.16	225.135927	0.03892882	0.00532056	52.1802466	5.06E-13	1.11E-11	Gbp9
ENSMUSG00000029299.15	99.4307773	0.04622164	0.00515854	76.1009562	2.70E-18	1.13E-16	Abcg3
ENSMUSG00000029304.15	1115.58787	0.02652424	0.00613029	18.8443215	1.42E-05	9.78E-05	Spp1
ENSMUSG00000029307.8	19.7571002	0.02668696	0.02079094	2.04244524	0.15296359	0.29168075	Dmp1
ENSMUSG00000029309.8	330.124108	0.02967761	0.00509137	32.0651491	1.49E-08	1.76E-07	Sparcl1
ENSMUSG00000029310.14	576.517711	0.00163268	0.00201412	0.65592717	0.4180015	0.60946431	Nudt9
ENSMUSG00000029311.17	2215.84714	-0.0088141	0.00342828	6.59860886	0.01020585	0.03243702	Hsd17b11
ENSMUSG00000029312.13	61.0319421	-0.0022006	0.00276404	0.62534391	0.42906836	0.62047862	Klhl8
ENSMUSG00000029313.19	604.533939	-0.0046185	0.00303226	2.29767593	0.12956774	0.25708629	Aff1
ENSMUSG00000029314.15	154.023403	0.03255539	0.00608759	27.1879606	1.85E-07	1.83E-06	Gpat3
ENSMUSG00000029319.9	748.853728	-0.0047818	0.00207066	5.33419268	0.02091102	0.0590809	Coq2
ENSMUSG00000029321.2	5.91754964	0.01836666	0.00857255	4.52093065	0.03348261	0.08705037	Slc10a6
ENSMUSG00000029322.13	252.255025	0.0495743	0.00603699	62.6292639	2.50E-15	7.35E-14	Plac8
ENSMUSG00000029326.14	107.061268	0.01739337	0.00348041	25.3405253	4.81E-07	4.39E-06	Enoph1
ENSMUSG00000029328.16	1078.11055	0.00251921	0.00218873	1.33087201	0.24865014	0.42007466	Hnrnpdl
ENSMUSG00000029330.9	22.6854239	0.04587928	0.00712381	39.6498633	3.04E-10	4.53E-09	Cds1
ENSMUSG00000029334.15	29.5524005	0.05503714	0.01418929	13.9105689	0.00019172	0.00102723	Prkg2
ENSMUSG00000029335.6	5.37411903	0.04244961	0.01138598	13.463596	0.00024324	0.0012684	Bmp3
ENSMUSG00000029338.14	572.197101	-0.0004136	0.00456382	0.00804511	0.92852994	0.99089102	Antxr2
ENSMUSG00000029344.16	487.318198	-0.0004989	0.00256164	0.03776641	0.84591294	0.94441458	Tpst2
ENSMUSG00000029345.14	371.792646	-0.0058426	0.00203471	8.21774128	0.00414828	0.01507571	Tfip11
ENSMUSG00000029346.13	96.8146229	-0.0055638	0.00265752	4.41610445	0.03560123	0.09149945	Srrd
ENSMUSG00000029348.12	3.25454895	0.03366577	0.02497389	2.59810085	0.10699186	0.22124407	Asphd2
ENSMUSG00000029352.15	15.647349	-0.0270733	0.00991618	7.1325352	0.00756976	0.02521401	Crybb3

ENSMUSG00000029359.14	14.2881524	0.02568849	0.00988449	6.19441925	0.01281538	0.03922952	Tesc
ENSMUSG00000029361.19	6.45433445	0.04618759	0.00962658	21.4657726	3.60E-06	2.81E-05	Nos1
ENSMUSG00000029363.15	211.793566	0.00711725	0.00402903	3.13470068	0.07664245	0.17037596	Rfc5
ENSMUSG00000029364.16	914.763029	-0.0029738	0.00180109	2.71083727	0.09966867	0.20964819	Wsb2
ENSMUSG00000029366.11	130.816874	0.01981462	0.00402658	23.700468	1.13E-06	9.60E-06	Dck
ENSMUSG00000029368.11	1110350.38	0.00627942	0.00583911	1.15258772	0.2830081	0.46296498	Alb
ENSMUSG00000029369.18	3907.37331	-0.0023004	0.00465296	0.24498703	0.6206272	0.79082188	Afm
ENSMUSG00000029370.11	216.977587	0.00515799	0.00434334	1.43338479	0.23121283	0.39887258	Rassf6
ENSMUSG00000029371.8	4.48929869	0.07491442	0.01376421	27.7543276	1.38E-07	1.40E-06	Cxcl5
ENSMUSG00000029372.8	27.2272882	0.08313167	0.01011136	51.2716618	8.04E-13	1.71E-11	Ppbp
ENSMUSG00000029373.8	66.3402776	0.08592439	0.01061885	49.9185188	1.60E-12	3.27E-11	Pf4
ENSMUSG00000029375.7	5.72759794	-0.0069088	0.03306681	-0.0132906	1	1	Cxcl15
ENSMUSG00000029376.9	117.240714	0.01466956	0.00492683	8.48010944	0.00359051	0.01331745	Mthfd2l
ENSMUSG00000029377.6	12.3315692	0.05011331	0.00919956	28.5615864	9.08E-08	9.51E-07	Ereg
ENSMUSG00000029378.6	5.35336578	0.05189338	0.02515014	-0.4290765	1	1	Areg
ENSMUSG00000029380.12	490.976617	0.0283063	0.00993091	5.56691881	0.01830294	0.05289709	Cxcl1
ENSMUSG00000029381.17	182.389794	0.00502061	0.00528337	0.88089117	0.34795771	0.5360443	Shroom3
ENSMUSG00000029384.6	3.58546772	-0.0237611	0.00985223	5.70209532	0.01694467	0.04965301	2010109A12Rik
ENSMUSG00000029385.15	374.176212	-0.0024634	0.00386095	0.38877726	0.53294282	0.71746515	Ccng2
ENSMUSG00000029387.11	228.588543	-0.0072712	0.00276347	6.94667054	0.00839754	0.02750511	Gtf2h3
ENSMUSG00000029388.15	379.548484	-0.0057281	0.00214685	7.13607266	0.00755484	0.02516787	Eif2b1
ENSMUSG00000029389.18	140.516779	-0.0047373	0.00330224	2.06765179	0.15045302	0.28810464	Ddx55
ENSMUSG00000029390.14	5161.04324	1.37E-05	0.00199124	4.61E-05	0.99458394	1	Tmed2
ENSMUSG00000029392.13	135.775366	0.00188531	0.00490434	0.14523872	0.70312758	0.85185777	Rilpl1
ENSMUSG00000029394.12	487.745135	-0.0069971	0.00230941	9.23102138	0.00237949	0.00937565	Cdk2ap1
ENSMUSG00000029397.16	575.450941	-0.0030571	0.00230319	1.74408893	0.18662156	0.33971261	Rchy1
ENSMUSG00000029401.9	167.287018	0.01023529	0.003299	9.42424535	0.00214135	0.00857186	Rilpl2
ENSMUSG00000029402.9	75.0564107	-0.0037878	0.00303615	1.54133166	0.2144197	0.37753757	Snrnp35
ENSMUSG00000029403.15	137.950065	-0.0059368	0.00325576	3.30424208	0.06910122	0.15656304	Cdkl2
ENSMUSG00000029404.16	639.143293	-0.0054054	0.00423651	1.64680297	0.19939462	0.35741918	Arl6ip4
ENSMUSG00000029405.17	1586.00375	0.00226077	0.00226312	0.98616114	0.32068244	0.50674099	G3bp2
ENSMUSG00000029406.16	588.722982	-0.0042971	0.00342435	1.57100146	0.21006164	0.37190465	Pitpnm2
ENSMUSG00000029407.11	1705.8551	-0.0031427	0.00222645	1.95992801	0.16152102	0.30373673	Uso1
ENSMUSG00000029408.14	41.474001	0.00279225	0.00343738	0.66234176	0.41573443	0.60702447	Abcb9
ENSMUSG00000029413.15	102.493711	0.03953325	0.00469878	67.2256941	2.42E-16	8.00E-15	Naaa
ENSMUSG00000029414.12	19.249955	0.0387071	0.01357042	7.18188235	0.00736434	0.02463402	Kntc1
ENSMUSG00000029415.5	260.723175	-4.21E-05	0.00330159	0.00016334	0.989803	1	Sdad1
ENSMUSG00000029416.18	435.415972	0.00477405	0.00440304	1.1278288	0.28823893	0.46934094	Slc15a4
ENSMUSG00000029417.10	694.046695	0.07569351	0.00773011	87.307957	9.29E-21	5.19E-19	Cxcl9
ENSMUSG00000029419.9	11.0468229	0.06380679	0.01186462	25.2786884	4.96E-07	4.52E-06	Ajm1
ENSMUSG00000029420.15	6.51480136	-0.0004599	0.01878378	-0.0019327	1	1	Rimbp2
ENSMUSG00000029422.16	929.20616	0.00060782	0.00286057	0.04455097	0.83283186	0.93587937	Rsrc2
ENSMUSG00000029423.11	3.44759683	-0.0229978	0.03653177	0.40230676	0.52590035	0.71120972	Piwil1
ENSMUSG00000029426.9	3551.23251	-0.0007382	0.00219759	0.11108227	0.73891533	0.87604589	Scarb2
ENSMUSG00000029427.13	357.214406	0.00157627	0.00246229	0.40907082	0.5224415	0.70813776	Zcchc8
ENSMUSG00000029428.14	249.218244	0.00800568	0.00328923	5.7621873	0.01637468	0.04824046	Stx2
ENSMUSG00000029430.14	2725.00706	-0.0158942	0.00375611	17.6966279	2.59E-05	0.00016867	Ran
ENSMUSG00000029432.13	435.206591	0.00949826	0.00488109	3.63275234	0.05665305	0.13375511	Nipsnap2
ENSMUSG00000029433.17	398.381064	-0.0052284	0.00227229	5.28061901	0.02156408	0.06054165	Diablo
ENSMUSG00000029434.13	474.496431	-0.0013302	0.00183584	0.52653181	0.46806862	0.65917521	Vps33a
ENSMUSG00000029436.10	19.0761605	0.01614248	0.00655778	5.8432115	0.01563726	0.04642754	Mmp17
ENSMUSG00000029438.10	227.352867	-0.0057555	0.00302395	3.60857357	0.0574824	0.13519944	Bcl7a
ENSMUSG00000029439.15	575.460198	0.00045711	0.00387318	0.01404092	0.90567593	0.97866278	Sfswap
ENSMUSG00000029440.16	843.695677	-0.0107625	0.00457417	5.57152647	0.01825484	0.05280337	Psmd9
ENSMUSG00000029442.20	28.7325961	0.02638896	0.00589973	19.2860865	1.13E-05	7.89E-05	Cfap251
ENSMUSG00000029445.14	27363.2422	-0.0091272	0.00533345	2.92701684	0.08710831	0.18861073	Hpd
ENSMUSG00000029446.15	124.882782	-0.009512	0.00437071	4.56267687	0.03267596	0.08532941	Psph
ENSMUSG00000029447.14	2640.35769	-0.007165	0.00213168	11.1177357	0.00085506	0.00382308	Cct6a
ENSMUSG00000029449.12	13.3595059	0.05183136	0.00757428	42.4044466	7.42E-11	1.21E-09	Rhof
ENSMUSG00000029452.19	4.61634386	-0.0297016	0.0109517	6.59032136	0.01025347	0.03256642	Tmem116
ENSMUSG00000029454.16	450.864949	-0.0102277	0.00245176	17.4111978	3.01E-05	0.00019332	Mapkapk5

ENSMUSG00000029455.15	29333.3736	-0.0005346	0.00317308	0.02761144	0.86802559	0.95781294	Aldh2
ENSMUSG00000029456.13	499.954314	0.00825607	0.0028923	8.19404659	0.00420281	0.01525272	Acad10
ENSMUSG00000029458.15	2443.17587	-0.0013823	0.00254604	0.28922456	0.59071788	0.76678294	Brap
ENSMUSG00000029461.18	621.879103	0.00028948	0.00342501	0.00705045	0.93308268	0.99335128	Fam168a
ENSMUSG00000029462.19	1096.6854	-0.0048299	0.00287769	2.81116412	0.09361042	0.19970111	Vps29
ENSMUSG00000029463.6	55.3596752	-0.0023562	0.00335777	0.48982143	0.48400697	0.67318012	Fam216a
ENSMUSG00000029464.11	227.952939	-0.005006	0.00213373	5.48932912	0.01913288	0.05484305	Gpn3
ENSMUSG00000029465.15	1005.77986	0.00781062	0.00242687	10.2875971	0.00133927	0.00567112	Arpc3
ENSMUSG00000029466.12	332.938255	-0.0013905	0.00265491	0.27642934	0.59905116	0.77331403	Anapc7
ENSMUSG00000029467.16	5847.94754	-0.011215	0.00499755	4.81137036	0.02827255	0.07574244	Atp2a2
ENSMUSG00000029468.18	119.16728	0.05433242	0.00636954	63.5557689	1.56E-15	4.69E-14	P2rx7
ENSMUSG00000029469.15	119.733167	0.0063761	0.00551525	1.24581394	0.2643535	0.43938977	Ift81
ENSMUSG00000029470.16	1255.79806	0.0098432	0.00356502	7.57597227	0.00591515	0.02040969	P2rx4
ENSMUSG00000029471.14	226.002371	-0.0013891	0.0036341	0.1445668	0.70378255	0.85234482	Camkk2
ENSMUSG00000029472.14	1276.6159	-0.0031514	0.00295772	1.13077115	0.28761091	0.46861558	Anapc5
ENSMUSG00000029474.8	186.881411	0.00108359	0.00381408	0.07799589	0.78003191	0.90344682	Rnf34
ENSMUSG00000029475.18	174.836675	0.00615211	0.00290321	4.4154205	0.0356155	0.09152617	Kdm2b
ENSMUSG00000029477.15	1.25968223	-0.0183867	0.03784562	0.31302811	0.57582793	0.7544008	Morn3
ENSMUSG00000029478.17	778.657612	-0.0098325	0.00401764	5.87499698	0.0153574	0.04576947	Ncor2
ENSMUSG00000029480.14	203.63299	-0.0150812	0.00408684	13.2769118	0.00026869	0.00138493	Dhx37
ENSMUSG00000029482.5	3408.36355	0.01399615	0.00664898	4.22789255	0.03976479	0.10012728	Aacs
ENSMUSG00000029484.13	136.398663	0.03636243	0.00582039	36.7234657	1.36E-09	1.85E-08	Anxa3
ENSMUSG00000029486.15	832.976744	-0.0135341	0.00261223	26.4554081	2.70E-07	2.59E-06	Mrpl1
ENSMUSG00000029490.5	68.2343278	0.01284011	0.0043908	8.77209506	0.00305874	0.01161543	Slc49a3
ENSMUSG00000029499.12	1806.36979	-0.0086328	0.00309353	7.95711926	0.00478986	0.01703462	Pxmp2
ENSMUSG00000029500.17	322.788672	-0.0038442	0.00201751	3.60701258	0.05753639	0.13528594	Pgam5
ENSMUSG00000029501.16	564.506196	0.00118501	0.00235984	0.24591893	0.61996346	0.79023228	Ankle2
ENSMUSG00000029502.15	576.356691	-0.0056795	0.00315468	3.22328296	0.07259789	0.16299799	Golga3
ENSMUSG00000029503.17	0.86085174	-0.0046573	0.01737867	0.00765922	0.9302606	0.99177898	P2rx2
ENSMUSG00000029504.6	192.462324	-0.0159706	0.0044868	12.7726718	0.00035172	0.00175495	Ddx51
ENSMUSG00000029505.19	807.932977	-0.0015481	0.00365745	0.17644107	0.67444973	0.83155036	Ep400
ENSMUSG00000029507.17	241.232659	-0.0045779	0.00238772	3.69767832	0.05448824	0.12955237	Pus1
ENSMUSG00000029510.16	3.70141306	0.01857922	0.01076722	2.94636802	0.08607079	0.18682733	Gpc2
ENSMUSG00000029512.12	1468.80867	-0.0132287	0.00339494	15.0805868	0.00010302	0.00058718	Ulk1
ENSMUSG00000029513.15	434.972801	0.01201365	0.00236952	25.5678762	4.27E-07	3.94E-06	Prkab1
ENSMUSG00000029516.20	41.836833	0.01977937	0.00689708	7.48435032	0.00622375	0.02132076	Cit
ENSMUSG00000029518.16	324.517044	0.00523485	0.00246703	4.4406806	0.03509226	0.09043771	Rab35
ENSMUSG00000029521.8	44.6064915	0.00841369	0.00530507	2.50434819	0.11353245	0.23182204	Chek2
ENSMUSG00000029524.17	129.206665	-0.0133367	0.00356181	14.22433	0.00016226	0.00088446	Sirt4
ENSMUSG00000029528.20	681.142561	0.00248821	0.00241063	1.04537536	0.30657459	0.49166168	Pxn
ENSMUSG00000029530.17	10.430294	0.01347398	0.00831274	2.69616703	0.10058981	0.21113387	Ccr9
ENSMUSG00000029534.18	150.765255	-0.0017811	0.00306458	0.34518102	0.55685379	0.73894192	St7
ENSMUSG00000029535.5	329.041044	-0.0118062	0.00294814	16.0117953	6.29E-05	0.00037779	Triap1
ENSMUSG00000029536.9	359.636056	-0.0040289	0.00240519	2.79252046	0.09470516	0.20154434	Gatc
ENSMUSG00000029538.15	334.707843	-0.0002039	0.00220948	0.00851637	0.92647228	0.98974711	Srsf9
ENSMUSG00000029544.17	10.6105584	-0.0034473	0.01054132	0.09695571	0.75551395	0.88694202	Cabp1
ENSMUSG00000029545.14	2551.89116	-0.0164778	0.00306294	28.8953091	7.64E-08	8.09E-07	Acads
ENSMUSG00000029547.11	363.654403	-0.0040936	0.00346913	1.39524474	0.23752138	0.40660323	Ints1
ENSMUSG00000029550.12	330.430988	0.00522884	0.00316854	2.70793645	0.09985008	0.20995488	Sppl3
ENSMUSG00000029551.14	232.368902	-0.011317	0.00447411	6.47112516	0.01096411	0.03448547	Psmg3
ENSMUSG00000029552.20	49.0036225	0.01726345	0.00661613	6.1097789	0.01344359	0.04086891	Tes
ENSMUSG00000029553.11	61.5934842	0.03822862	0.00589655	39.6508246	3.04E-10	4.53E-09	Tfec
ENSMUSG00000029554.16	147.289955	-0.0081161	0.00362082	5.11909308	0.02366398	0.06545545	Mad1l1
ENSMUSG00000029556.13	458.95776	-0.0068863	0.0037224	3.22919783	0.07233611	0.16256499	Hnf1a
ENSMUSG00000029557.8	137.185512	-0.0093805	0.00331732	8.02302781	0.00461863	0.0165003	Mrm2
ENSMUSG00000029559.16	199.307559	0.00517978	0.00351903	2.13869995	0.14362356	0.27825737	2210016L21Rik
ENSMUSG00000029560.13	250.293104	0.00645513	0.00331314	3.80586479	0.0510734	0.12266977	Snx8
ENSMUSG00000029561.18	175.74885	0.04233245	0.00562015	57.031011	4.29E-14	1.08E-12	Oasl2
ENSMUSG00000029563.17	116.377481	0.01022648	0.00447588	5.18495422	0.02278327	0.06342102	Foxp2
ENSMUSG00000029569.10	448.064993	0.00889424	0.0021856	16.1361712	5.89E-05	0.00035558	Tmem168
ENSMUSG00000029570.6	85.7808778	0.03536075	0.00474247	52.1296339	5.20E-13	1.14E-11	Lfng

ENSMUSG00000029571.15	4439.95798	0.00844015	0.00336516	6.0993789	0.01352294	0.0410809	Tmem106b
ENSMUSG00000029575.18	550.439676	-0.0027242	0.00318542	0.71520377	0.39772168	0.58942283	Mmab
ENSMUSG00000029576.18	1.75269346	0.02616159	0.01834805	2.97890969	0.08435608	0.18386679	Radil
ENSMUSG00000029577.14	1005.53973	-0.0073492	0.00266915	7.6152607	0.00578764	0.02003923	Ube3b
ENSMUSG00000029578.16	1481.01093	-0.0113129	0.00269181	17.5035496	2.87E-05	0.00018531	Wipi2
ENSMUSG00000029580.15	20028.8339	0.02199481	0.00371062	34.1835569	5.02E-09	6.34E-08	Actb
ENSMUSG00000029581.15	101.393486	0.02795443	0.00651032	17.6676746	2.63E-05	0.00017101	Fscn1
ENSMUSG00000029587.17	185.9626	-0.0011028	0.00273131	0.16122475	0.68803141	0.84149952	Zfp12
ENSMUSG00000029591.15	128.666254	0.0045271	0.00509408	0.77639928	0.37824464	0.56946751	Ung
ENSMUSG00000029592.12	267.488408	-0.0018388	0.00262189	0.48816149	0.48474857	0.67385394	Usp30
ENSMUSG00000029594.13	195.531971	0.00360112	0.00405953	0.78361626	0.37603744	0.56715814	Rbm19
ENSMUSG00000029596.14	202.202379	-0.0022725	0.00491684	0.21302646	0.64440557	0.80979006	Sdsl
ENSMUSG00000029597.15	3058.84041	0.00100447	0.0060685	0.02777507	0.86763872	0.95755496	Sds
ENSMUSG00000029598.13	1241.10666	0.00024875	0.00240093	0.01071737	0.91754653	0.98492936	Plbd2
ENSMUSG00000029599.14	667.558361	-0.0061659	0.00241733	6.49588669	0.01081244	0.03408931	Ddx54
ENSMUSG00000029600.11	67.1648422	-0.0142736	0.003164	20.7103013	5.34E-06	4.02E-05	Rita1
ENSMUSG00000029601.14	2.96075174	-0.0179119	0.01047869	2.89419452	0.0888992	0.19159142	Iqcd
ENSMUSG00000029602.12	9.5561831	0.0567807	0.01295288	15.4093632	8.66E-05	0.00050149	Rasal1
ENSMUSG00000029603.16	16.2235824	0.04344623	0.00748746	31.9794502	1.56E-08	1.83E-07	Dtx1
ENSMUSG00000029605.18	28.382759	0.01373757	0.00564541	5.81705656	0.01587148	0.04699872	Oas1b
ENSMUSG00000029607.15	2.21662375	0.00964925	0.01444066	0.69241035	0.40534574	0.59696135	Ankrd61
ENSMUSG00000029608.11	9.76703428	0.03654611	0.01889356	4.83183347	0.0279389	0.07501901	Rph3a
ENSMUSG00000029610.14	160.067001	0.0016652	0.00331112	0.25621902	0.61272972	0.78478649	Aimp2
ENSMUSG00000029613.16	856.277393	-0.0042244	0.00189739	4.94909363	0.02610435	0.07096164	Eif2ak1
ENSMUSG00000029614.14	6345.60252	-0.0024231	0.00314366	0.59725304	0.43962804	0.63154526	Rpl6
ENSMUSG00000029616.10	916.548064	-0.0024584	0.00303578	0.6465738	0.42134031	0.6128728	Erp29
ENSMUSG00000029617.11	534.114972	-0.0037459	0.00227581	2.69345816	0.10076092	0.21142554	Ccz1
ENSMUSG00000029621.15	1101.49038	0.00520814	0.00237115	4.80846313	0.02832029	0.0758531	Arpc1a
ENSMUSG00000029622.17	1063.61181	0.02767966	0.00423572	40.2499101	2.23E-10	3.41E-09	Arpc1b
ENSMUSG00000029623.15	1863.42012	-0.0034108	0.00345881	0.96510988	0.3259028	0.51268881	Pdap1
ENSMUSG00000029624.11	215.065978	-0.007161	0.00187905	14.621139	0.00013143	0.0007306	Ptcd1
ENSMUSG00000029625.17	265.396164	0.00653536	0.00243263	7.19693153	0.00730283	0.02448744	Cpsf4
ENSMUSG00000029627.13	102.463435	-0.0059409	0.00255175	5.43702044	0.01971427	0.05622501	Zkscan14
ENSMUSG00000029629.18	596.279649	0.00179197	0.00356141	0.25197598	0.61568715	0.7871211	Phf14
ENSMUSG00000029630.16	9495.66692	0.00495734	0.00515436	0.94012645	0.33224532	0.51920348	Cyp3a25
ENSMUSG00000029632.8	9243.57795	-0.0158512	0.0040753	15.1628141	9.86E-05	0.0005642	Ndufa4
ENSMUSG00000029634.16	727.162771	-0.0010711	0.00201335	0.28055183	0.59633976	0.77129137	Rnf6
ENSMUSG00000029635.16	562.671157	-0.0057284	0.00219002	6.77245957	0.00925752	0.02994347	Cdk8
ENSMUSG00000029636.12	20.6673669	0.02124334	0.0123605	2.54336453	0.11075855	0.22743288	Wasf3
ENSMUSG00000029638.18	98.0965575	0.01743622	0.00670492	6.67498173	0.00977755	0.03128657	Glcci1
ENSMUSG00000029640.18	584.33069	0.01518751	0.00297611	25.4329601	4.58E-07	4.20E-06	Usp12
ENSMUSG00000029641.10	11.4937991	0.01901606	0.00702002	7.05967931	0.00788381	0.02606498	Rasl11a
ENSMUSG00000029642.12	1080.22475	-0.0042113	0.00306404	1.90772655	0.16721602	0.31212068	Polr1d
ENSMUSG00000029647.16	622.574293	0.00611558	0.00447021	1.8672045	0.1717956	0.31882689	Pan3
ENSMUSG00000029648.14	346.18254	0.00185895	0.00354136	0.27499099	0.60000326	0.77411156	Flt1
ENSMUSG00000029649.11	2506.58603	-0.0095478	0.00319113	8.8644409	0.00290779	0.0111228	Pomp
ENSMUSG00000029650.11	682.227983	-0.0068964	0.00382192	3.26170031	0.07091556	0.15990577	Slc46a3
ENSMUSG00000029651.17	66.9978426	-0.003465	0.00921376	0.1320794	0.71628571	0.86050603	Mtus2
ENSMUSG00000029655.18	924.301576	-0.0035352	0.00249508	1.99369778	0.15795478	0.29873478	N4bp2l2
ENSMUSG00000029656.14	5561.21928	-0.041974	0.00604445	47.3702942	5.88E-12	1.12E-10	C8b
ENSMUSG00000029657.16	1021.46828	-0.0220103	0.00552741	14.2341937	0.00016141	0.00088065	Hsph1
ENSMUSG00000029658.18	0.935354	0.07874891	0.02449028	11.4387733	0.00071927	0.00328802	Wdr95
ENSMUSG00000029659.17	13.0664149	0.02460134	0.00864917	7.35551172	0.00668575	0.02272518	Medag
ENSMUSG00000029661.17	376.61039	0.01063219	0.00592547	3.32603645	0.06819102	0.15503696	Col1a2
ENSMUSG00000029663.11	19.2935683	0.01499073	0.00811807	3.19800283	0.07372825	0.1651177	Gngt1
ENSMUSG00000029664.11	1800.06395	-0.009314	0.00415263	4.80750099	0.02833611	0.07588685	Tfpi2
ENSMUSG00000029669.15	3060.66145	-0.0049777	0.00259915	3.69060747	0.05471968	0.12995945	Tspan12
ENSMUSG00000029670.13	164.109032	-0.0010574	0.00286194	0.13741485	0.71086465	0.85719895	Ing3
ENSMUSG00000029672.17	515.599857	-0.0007535	0.00250429	0.0895698	0.76472478	0.89331432	Fam3c
ENSMUSG00000029673.20	34.132075	0.01694861	0.006966	5.82241098	0.01582324	0.04689119	Auts2
ENSMUSG00000029674.14	52.6488818	0.0332472	0.00460644	51.446116	7.36E-13	1.57E-11	Limk1

ENSMUSG00000029675.13	68.7842646	-0.0123493	0.0069613	3.15327922	0.07577454	0.16871692	Eln
ENSMUSG00000029676.16	108.216336	0.0112327	0.00335261	10.7345698	0.00105152	0.00458943	Pot1a
ENSMUSG00000029679.8	1.80972365	0.00074305	0.02080606	0.00750789	0.93095127	0.99220138	Hyal6
ENSMUSG00000029681.13	288.563598	0.00410071	0.0028778	2.03280571	0.1539362	0.29317985	Bcl7b
ENSMUSG00000029683.8	368.507506	-0.0387688	0.01507998	7.08800906	0.00776014	0.02572101	Lmod2
ENSMUSG00000029684.15	1320.80064	0.00059658	0.00291952	0.04084095	0.83984523	0.94036567	Wasl
ENSMUSG00000029685.16	13.2675503	0.03109693	0.02274692	-0.0735047	1	1	Asb15
ENSMUSG00000029686.16	1197.20432	-0.0041971	0.00229485	3.30445632	0.06909221	0.15655767	Cul1
ENSMUSG00000029687.17	116.761891	-0.0008605	0.00558206	0.02319267	0.87895711	0.96325074	Ezh2
ENSMUSG00000029695.14	5585.90701	0.00225302	0.004312	0.26786541	0.60476728	0.77775579	Aass
ENSMUSG00000029699.14	37.8584266	-0.0001117	0.00398027	0.00076174	0.97798144	1	Ssc4d
ENSMUSG00000029700.12	21.5038654	0.02170915	0.01859256	1.75875748	0.18477957	0.33721843	Slc13a1
ENSMUSG00000029701.16	465.700171	-0.0082552	0.00260903	9.82524889	0.00172132	0.00707425	Rbm28
ENSMUSG00000029703.11	176.098608	0.00548117	0.0057118	0.9075708	0.34075978	0.52848239	Lrwd1
ENSMUSG00000029705.18	494.963262	-0.0154326	0.00223412	47.4076147	5.77E-12	1.10E-10	Cux1
ENSMUSG00000029708.10	293.445862	0.00173397	0.002669	0.42100839	0.51643431	0.70282266	Gcc1
ENSMUSG00000029710.16	427.364795	-0.0091913	0.00235089	15.7423569	7.26E-05	0.00042904	Ephb4
ENSMUSG00000029713.16	1414.04529	0.00286312	0.00349212	0.67441695	0.41151588	0.60308274	Gnb2
ENSMUSG00000029714.12	400.048962	-0.0049382	0.00343528	2.05060141	0.15214608	0.29056821	Gigyf1
ENSMUSG00000029715.6	81.7774924	-0.0047444	0.0035088	1.83383698	0.17567501	0.32426599	Pop7
ENSMUSG00000029716.14	5977.12083	-0.0038862	0.00438152	0.77811487	0.3777183	0.56885664	Tfr2
ENSMUSG00000029718.15	165.509297	0.01214019	0.00472216	6.58304916	0.01029544	0.03267708	Pcolce
ENSMUSG00000029720.10	140.457122	0.00857387	0.00562443	2.33775822	0.12627115	0.25200808	Gm20605
ENSMUSG00000029722.16	676.451572	-0.0008127	0.00339923	0.05664546	0.81187871	0.92264605	Agfg2
ENSMUSG00000029723.17	269.713117	0.01333866	0.00432536	9.19029514	0.00243302	0.00954827	Spacdr
ENSMUSG00000029725.11	280.274484	0.00149594	0.00669044	0.05015812	0.82278837	0.92950888	Ppp1r35
ENSMUSG00000029726.17	369.483762	0.00160482	0.00323148	0.24821755	0.61833296	0.78926949	Mepce
ENSMUSG00000029727.8	3372.31252	0.01060666	0.00490401	4.63030539	0.03141197	0.08256801	Cyp3a13
ENSMUSG00000029729.13	798.595886	-0.0039664	0.00329314	1.43069794	0.23165057	0.39930367	Zkscan1
ENSMUSG00000029730.17	241.760893	0.00449525	0.00292691	2.3142873	0.12819004	0.25525025	Mcm7
ENSMUSG00000029735.18	334.665698	-0.0003561	0.00352233	0.0100363	0.92020035	0.98667298	Tpk1
ENSMUSG00000029752.13	90.5926155	-0.0149217	0.01009085	1.80988978	0.17852145	0.32838925	Asns
ENSMUSG00000029754.14	2.29975186	0.00330201	0.03448572	0.03977398	0.84192316	0.94201137	Dlx6
ENSMUSG00000029755.11	1.74448182	0.00388444	0.05190097	0.04319806	0.83535279	0.93764007	Dlx5
ENSMUSG00000029757.17	5.45917821	-0.0016728	0.01978457	-0.0150326	1	1	Dync1i1
ENSMUSG00000029759.10	1228.35605	0.00020033	0.00249189	0.00637359	0.93636869	0.99509404	Pon3
ENSMUSG00000029761.17	10109.9715	0.00346246	0.00388268	0.77070069	0.38000037	0.57147239	Cald1
ENSMUSG00000029762.7	50.0043156	0.00927357	0.00425721	4.78972192	0.02863005	0.07649165	Akr1b8
ENSMUSG00000029763.18	406.619946	0.01360237	0.00282401	22.269969	2.37E-06	1.91E-05	Exoc4
ENSMUSG00000029765.13	82.1554639	0.01531731	0.00489824	9.71484731	0.00182785	0.00746265	Plxna4
ENSMUSG00000029767.17	2532.18274	0.00029998	0.00365556	0.00654305	0.93553013	0.99465354	Calu
ENSMUSG00000029769.18	18.9774328	-0.0056486	0.01295645	0.18999851	0.66291786	0.82289889	Ccdc136
ENSMUSG00000029771.13	283.838007	0.00015967	0.00453728	0.0012172	0.97216875	1	Irf5
ENSMUSG00000029772.18	388.570375	-0.0102391	0.00423086	5.50049283	0.01901112	0.05454941	Ahcyl2
ENSMUSG00000029775.15	934.070102	-0.0079	0.00270987	8.48998816	0.00357106	0.01326201	Klhdc10
ENSMUSG00000029776.12	5181.78321	-0.0087894	0.00246129	12.7612419	0.00035388	0.0017631	Hibadh
ENSMUSG00000029777.11	759.196774	0.00023492	0.00178556	0.01745021	0.89490578	0.97287826	Gars
ENSMUSG00000029778.13	68.9410779	0.02462131	0.00506457	22.7830612	1.81E-06	1.50E-05	Adcyap1r1
ENSMUSG00000029780.15	532.622703	-0.0030615	0.00253762	1.45909795	0.2270737	0.39339161	Nt5c3
ENSMUSG00000029781.8	629.118141	-0.000533	0.00252463	0.04440297	0.83310567	0.93602325	Fkbp9
ENSMUSG00000029782.21	207.035028	0.01064416	0.00235799	20.1061228	7.33E-06	5.34E-05	Tmem209
ENSMUSG00000029787.11	420.926916	0.00770085	0.00340846	4.91013724	0.02669952	0.07230852	Avl9
ENSMUSG00000029790.17	50.9273388	-0.0004849	0.00426514	0.01273518	0.91014926	0.9809278	Cep41
ENSMUSG00000029797.15	18.999659	-0.0557946	0.00979284	27.4731257	1.59E-07	1.60E-06	Sspo
ENSMUSG00000029798.12	337.781893	0.02287894	0.00399752	31.5648921	1.93E-08	2.23E-07	Herc6
ENSMUSG00000029802.14	1435.4613	-0.0208822	0.00330708	39.0493249	4.13E-10	6.06E-09	Abcg2
ENSMUSG00000029804.18	196.009107	0.01959928	0.00624291	9.76246215	0.00178111	0.00729328	Herc3
ENSMUSG00000029810.16	3718.57366	0.03132683	0.00364372	72.5124772	1.66E-17	6.39E-16	Tmem176b
ENSMUSG00000029811.15	2.7047369	0.04774089	0.01393846	11.3916394	0.00073775	0.0033618	Aoc1
ENSMUSG00000029814.11	7.50237801	-0.0547185	0.016497	10.7552118	0.00103986	0.00454946	Igf2bp3
ENSMUSG00000029815.13	522.494769	-0.016319	0.00384213	18.1059801	2.09E-05	0.00013929	Malsu1

ENSMUSG00000029816.11	69.5476108	0.17697741	0.01214511	166.9516	3.43E-38	1.92E-35	Gpnmb
ENSMUSG00000029817.12	503.028884	-0.0049259	0.00321723	2.29506777	0.12978555	0.25749679	Tra2a
ENSMUSG00000029819.7	0.98055759	0.01742895	0.03465153	0.28358187	0.59436309	0.76962107	Npy
ENSMUSG00000029821.16	99.0383738	0.01643435	0.0043887	14.0009631	0.00018272	0.00098527	Gsdme
ENSMUSG00000029822.16	176.472602	0.08899933	0.00726753	114.902604	8.27E-27	9.85E-25	Osbpl3
ENSMUSG00000029823.17	2447.07688	-0.0016886	0.00343974	0.23918359	0.62479639	0.79381856	Luc7l2
ENSMUSG00000029826.15	965.495777	0.00785186	0.00232623	11.2180774	0.00081004	0.0036531	Zc3hav1
ENSMUSG00000029828.5	6.11888352	-0.0176811	0.00765028	5.18891602	0.02273139	0.06329153	4921507P07Rik
ENSMUSG00000029829.5	0.68642882	0.00195024	0.07390665	0.00482542	0.94461927	0.9994266	Tmem213
ENSMUSG00000029830.16	2.67080524	0.04451161	0.01556553	9.63864542	0.00190526	0.00774649	Svopl
ENSMUSG00000029832.17	7.38698495	-0.0225376	0.00852328	6.87295958	0.00875096	0.02851619	Nfe2l3
ENSMUSG00000029833.18	524.564817	0.02838282	0.00388596	50.9681655	9.39E-13	1.98E-11	Trim24
ENSMUSG00000029836.16	1042.16155	0.00329435	0.00306402	1.11917518	0.29009613	0.47155116	Cbx3
ENSMUSG00000029838.12	40.1890946	0.00893547	0.01312339	0.43712511	0.50851332	0.69530685	Ptn
ENSMUSG00000029840.9	2383.78219	0.00360536	0.00198033	3.25628328	0.07115022	0.1603123	Mtpn
ENSMUSG00000029843.5	7.59544595	0.01328933	0.00874935	2.09380874	0.14789695	0.28441072	Slc13a4
ENSMUSG00000029844.10	4.19589688	0.04472378	0.00832143	27.7707369	1.37E-07	1.39E-06	Hoxa1
ENSMUSG00000029851.6	21.1476208	0.01598902	0.00405404	15.1902449	9.72E-05	0.00055674	Tcaf2
ENSMUSG00000029859.7	574.11693	0.00467168	0.00346119	1.81648112	0.17773271	0.32729573	Epha1
ENSMUSG00000029860.17	626.937237	0.01405962	0.00358839	15.1372604	1.00E-04	0.00057134	Zyx
ENSMUSG00000029861.18	6.33400938	0.01575643	0.02142332	0.6725737	0.41215574	0.60355329	Fam131b
ENSMUSG00000029862.17	5.74119602	0.04572995	0.01917823	5.57715467	0.01819626	0.05266622	Clcn1
ENSMUSG00000029863.14	106.856693	0.01260381	0.00320145	15.0466745	0.00010488	0.00059696	Casp2
ENSMUSG00000029864.8	1563.27464	-0.0052323	0.00290404	3.22126543	0.07268742	0.16316793	Gstk1
ENSMUSG00000029865.5	0.55712749	-0.0113793	0.0415451	0.02200953	0.88206176	0.9650389	Sval1
ENSMUSG00000029867.6	1.16241557	-0.0209369	0.10685499	0.06064745	0.80547554	0.91892938	Llcfc1
ENSMUSG00000029869.8	27.9563475	0.02528209	0.00784809	9.42395318	0.00214169	0.00857186	Ephb6
ENSMUSG00000029882.6	6.22909284	-0.2938789	0.05566006	5.92452913	0.01493153	0.04469793	Prss3b
ENSMUSG00000029883.13	1.3516441	-0.0240745	0.08327954	0.069464	0.7921188	0.91118387	Prss59
ENSMUSG00000029910.15	140.031306	0.01720378	0.00707417	5.64054674	0.01754973	0.05110402	Mad2l1
ENSMUSG00000029911.15	581.564637	-0.0067949	0.00257906	6.93846317	0.00843616	0.0276201	Ssbp1
ENSMUSG00000029913.15	6.84789703	0.01380093	0.00819654	2.81768752	0.09323063	0.19907095	Prdm5
ENSMUSG00000029915.15	17.9571743	0.11065188	0.01099355	81.0797952	2.17E-19	1.05E-17	Clec5a
ENSMUSG00000029916.12	137.013496	0.00531951	0.00282869	3.49145242	0.06168644	0.14306159	Agk
ENSMUSG00000029918.10	805.970313	-0.0131015	0.00398527	10.8437531	0.0009913	0.00435963	Mrps33
ENSMUSG00000029919.6	64.013793	0.07796678	0.00708909	102.604346	4.09E-24	3.46E-22	Hpgds
ENSMUSG00000029920.10	280.930835	0.00391282	0.0028783	1.80963458	0.17855207	0.32841996	Smarcad1
ENSMUSG00000029922.16	446.902797	0.00703737	0.00291765	5.78708931	0.0161443	0.0476601	Mkrn1
ENSMUSG00000029923.5	8.66554446	0.06629808	0.00728612	74.9022996	4.95E-18	2.00E-16	Rab19
ENSMUSG00000029924.13	436.548761	-0.0037621	0.00269989	1.92949405	0.16481381	0.3086012	Slc37a3
ENSMUSG00000029925.14	84.5281451	0.05439525	0.00563537	86.8772483	1.15E-20	6.39E-19	Tbxas1
ENSMUSG00000029992.15	531.629327	0.00782348	0.00335374	5.39426327	0.02020305	0.05745228	Gfpt1
ENSMUSG00000029993.17	553.51362	-0.0046103	0.00246553	3.47369678	0.06235182	0.14427842	Nfu1
ENSMUSG00000029994.18	915.199149	0.01024148	0.00193133	28.0016871	1.21E-07	1.24E-06	Anxa4
ENSMUSG00000029998.15	3428.31696	0.00105588	0.00278971	0.14261015	0.70569983	0.85389728	Pcyox1
ENSMUSG00000029999.15	370.287483	-0.004647	0.00329306	1.97521054	0.15989588	0.30141497	Tgfa
ENSMUSG00000030000.11	6.750641	0.04450495	0.01932639	5.32722848	0.02099475	0.05923939	Add2
ENSMUSG00000030002.16	1195.9212	-0.0058849	0.00258067	5.09139703	0.02404481	0.0662743	Dusp11
ENSMUSG00000030004.7	318.380259	0.02309034	0.00590736	13.1339675	0.00028999	0.00148142	Nat8
ENSMUSG00000030007.9	1734.03695	-0.011909	0.00292083	16.4399294	5.02E-05	0.0003074	Cct7
ENSMUSG00000030008.10	108.950373	-0.0096607	0.00306734	9.98547626	0.0015778	0.00655333	Pradc1
ENSMUSG00000030016.16	904.888276	-0.0003397	0.00294262	0.01299552	0.90923947	0.98050013	Zfp638
ENSMUSG00000030017.3	0.87109501	-0.0175862	0.02974263	0.12089316	0.7280678	0.86877105	Reg3g
ENSMUSG00000030019.7	191.121344	0.01428208	0.00250071	31.9344303	1.59E-08	1.87E-07	Fbxl14
ENSMUSG00000030020.14	55.136108	0.01920417	0.00440912	18.4201875	1.77E-05	0.00011981	Prickle2
ENSMUSG00000030022.15	51.4834682	0.01637806	0.00511538	10.0722054	0.00150523	0.00629585	Adamts9
ENSMUSG00000030029.15	474.903254	-0.0170719	0.00338736	25.158064	5.28E-07	4.79E-06	Lrig1
ENSMUSG00000030030.6	13.2414063	-0.0141171	0.00610396	5.27254338	0.02166434	0.06077249	1700003E16Rik
ENSMUSG00000030031.15	44.7266359	-0.0215781	0.00591753	13.0039253	0.00031084	0.00157327	Kbtbd8
ENSMUSG00000030032.15	13.0315949	-0.0153307	0.00697499	4.79438887	0.02855258	0.07635388	Wdr54
ENSMUSG00000030034.13	203.053709	-0.0062468	0.00331981	3.5388164	0.05994822	0.13981399	Ino80b

ENSMUSG00000030035.15	478.105417	-0.0154623	0.00279616	30.3590949	3.59E-08	3.98E-07	Wbp1
ENSMUSG00000030036.9	698.796283	-0.0014533	0.00248283	0.34262778	0.55831633	0.74004996	Mogs
ENSMUSG00000030037.10	917.110755	-0.0184789	0.00342665	29.3677303	5.99E-08	6.42E-07	Mrpl53
ENSMUSG00000030041.10	1.06640164	0.05162489	0.02087192	5.92514391	0.01492632	0.04469325	M1ap
ENSMUSG00000030042.16	638.584094	-0.0058778	0.00291967	3.99217442	0.04571204	0.11215688	Pole4
ENSMUSG00000030045.11	337.529601	-0.0119108	0.00400179	8.777446	0.00304978	0.01158327	Mrpl19
ENSMUSG00000030046.7	83.6876047	-0.0029739	0.00595521	0.24526047	0.62043228	0.79074712	Bmp10
ENSMUSG00000030047.15	69.4531476	0.05738617	0.00523002	112.793903	2.39E-26	2.73E-24	Arhgap25
ENSMUSG00000030051.11	61.3483734	0.0008952	0.00281728	0.10057393	0.75114198	0.88439495	Aplf
ENSMUSG00000030054.5	3.65168067	0.08848744	0.01417649	34.8831402	3.50E-09	4.52E-08	Gp9
ENSMUSG00000030055.17	2388.60189	-0.0087791	0.00354971	6.06417911	0.01379509	0.04181608	Rab43
ENSMUSG00000030056.9	400.144597	0.00255712	0.00508227	0.24636732	0.61964465	0.79011701	Isy1
ENSMUSG00000030057.16	5928.73711	-0.0057597	0.00206531	7.86526979	0.00503933	0.01781173	Cnbp
ENSMUSG00000030058.18	1662.00102	0.0042988	0.00215316	3.98162008	0.0459993	0.1127913	Copg1
ENSMUSG00000030059.16	1358.60774	-0.0024133	0.00366045	0.42301252	0.51543767	0.70203343	Tmf1
ENSMUSG00000030060.15	120.158946	0.00074741	0.00310791	0.05730832	0.81080198	0.92218936	Hmces
ENSMUSG00000030061.17	699.142478	-0.0102459	0.00180741	32.0044177	1.54E-08	1.81E-07	Uba3
ENSMUSG00000030062.8	2544.67947	-0.0015552	0.0024409	0.40146778	0.5263322	0.7113476	Rpn1
ENSMUSG00000030064.17	1230.27048	0.01799517	0.00376118	22.599501	2.00E-06	1.63E-05	Frmd4b
ENSMUSG00000030067.18	490.662617	-0.008255	0.00390283	4.35831961	0.03682862	0.09403916	Foxp1
ENSMUSG00000030068.5	18.4996446	0.01942871	0.01158045	2.56309014	0.10938459	0.22530302	Gm20696
ENSMUSG00000030074.10	9.19826762	0.03503112	0.01544858	4.98423652	0.02557927	0.06980362	Gxylt2
ENSMUSG00000030075.11	2.29361113	0.02150177	0.02380557	1.07432221	0.2999712	0.48403371	Cntn3
ENSMUSG00000030077.12	28.9945617	0.03166988	0.01433765	4.25763713	0.03907424	0.09864122	Chl1
ENSMUSG00000030079.16	273.925377	-0.0041231	0.00203721	4.08348234	0.04330432	0.1073889	Ruvbl1
ENSMUSG00000030082.15	3066.82061	-0.0041954	0.00265133	2.49512543	0.11419928	0.23290135	Sec61a1
ENSMUSG00000030083.12	410.811522	-0.0131193	0.00275112	22.4082787	2.20E-06	1.79E-05	Abtb1
ENSMUSG00000030084.13	225.277359	0.01577127	0.00484672	10.4510782	0.00122578	0.0052585	Plxna1
ENSMUSG00000030086.17	71.0131672	0.00354106	0.00455207	0.58488393	0.44440432	0.63674448	Chchd6
ENSMUSG00000030087.12	2275.34313	-0.0102044	0.00313836	10.9517641	0.00093515	0.00413815	Klf15
ENSMUSG00000030088.16	19200.1829	0.00080071	0.0047026	0.02897723	0.86483163	0.95576417	Aldh1l1
ENSMUSG00000030089.16	55.3418349	0.00979374	0.00731202	1.6966979	0.19272039	0.34794114	Slc41a3
ENSMUSG00000030091.18	317.849871	0.01164842	0.00496905	5.57405495	0.0182285	0.05274011	Nup210
ENSMUSG00000030093.8	16.9170147	0.01663967	0.02195252	0.93722806	0.33299173	0.51995724	Wnt7a
ENSMUSG00000030094.9	366.845151	0.00016556	0.00182497	0.00815721	0.92803508	0.99048799	Xpc
ENSMUSG00000030095.11	212.643579	0.01924513	0.0030675	38.9782507	4.29E-10	6.27E-09	Tmem43
ENSMUSG00000030096.9	2198.31459	0.00819191	0.00308028	7.2698811	0.00701204	0.02365362	Slc6a6
ENSMUSG00000030098.16	7.85755404	0.05187271	0.01061882	21.4322258	3.67E-06	2.86E-05	Grip2
ENSMUSG00000030101.16	502.883425	0.00104036	0.00203087	0.26002859	0.61010052	0.78236972	Sumf1
ENSMUSG00000030102.12	1341.93381	0.00324368	0.00420533	0.58045956	0.44613224	0.63824721	Itpr1
ENSMUSG00000030103.12	2046.09113	0.01423663	0.00445838	9.88465958	0.00166662	0.0068686	Bhlhe40
ENSMUSG00000030104.10	3460.33354	0.00363088	0.00297648	1.48150392	0.22353937	0.38894957	Edem1
ENSMUSG00000030105.9	1192.80367	-0.0078888	0.00221378	12.488364	0.00040949	0.00200464	Arl8b
ENSMUSG00000030107.11	197.81553	0.02406299	0.00561988	19.6140315	9.48E-06	6.75E-05	Usp18
ENSMUSG00000030108.15	2996.50233	-0.0065353	0.00381751	2.91227936	0.08790755	0.18997504	Slc6a13
ENSMUSG00000030109.16	1139.97074	-0.0042721	0.0047998	0.78343038	0.37609406	0.56720727	Slc6a12
ENSMUSG00000030110.14	2.63080744	0.0374094	0.01508285	6.79353655	0.00914885	0.02962449	Ret
ENSMUSG00000030111.10	7.56827296	-0.0217631	0.01458318	1.6620309	0.19732934	0.35441744	A2m
ENSMUSG00000030114.9	1.21738413	0.10032878	0.01511289	42.5027861	7.06E-11	1.16E-09	Klrg1
ENSMUSG00000030116.15	4.41897052	0.03429448	0.0173	3.79958654	0.05126524	0.12306213	Mfap5
ENSMUSG00000030117.6	6.6221529	0.17820228	0.01226447	172.69272	1.91E-39	1.25E-36	Gdf3
ENSMUSG00000030120.15	2014.32715	-0.0093829	0.00199202	22.0693077	2.63E-06	2.10E-05	Mlf2
ENSMUSG00000030122.14	9208.86365	-0.0126434	0.00465872	7.28067992	0.00697001	0.02355226	Ptms
ENSMUSG00000030123.16	519.917504	0.00754491	0.00420815	3.1237087	0.07716098	0.17124766	Plxnd1
ENSMUSG00000030124.3	32.5522163	0.05164898	0.00743641	43.5991779	4.03E-11	6.82E-10	Lag3
ENSMUSG00000030125.12	4.5175046	-0.0188689	0.01907192	1.13465793	0.28678397	0.4676498	Lrrc23
ENSMUSG00000030126.18	664.118565	-0.0076213	0.00278537	7.42133694	0.00644549	0.02199094	Tmcc1
ENSMUSG00000030127.16	889.005524	-0.0007842	0.00182284	0.18442697	0.66759605	0.82629726	Cops7a
ENSMUSG00000030131.9	14690.4059	0.01513673	0.00588259	5.8894227	0.01523209	0.04543047	Mug2
ENSMUSG00000030134.12	13.4670295	0.04987958	0.01246124	15.2266832	9.53E-05	0.0005477	Rasgef1a
ENSMUSG00000030137.9	91.1166259	0.01885799	0.00871409	4.77203622	0.02892559	0.0771153	Tuba8

ENSMUSG00000030138.9	514.561775	-0.001393	0.00190179	0.53354125	0.46512184	0.65612223	Bms1
ENSMUSG00000030142.11	18.4993747	0.12513461	0.01217584	86.0982266	1.71E-20	9.41E-19	Clec4e
ENSMUSG00000030144.5	10.3035959	0.08804079	0.01034448	62.907934	2.17E-15	6.43E-14	Clec4d
ENSMUSG00000030145.15	12.0780112	0.0004451	0.00694889	0.00416996	0.94851222	1	Zfp248
ENSMUSG00000030147.13	10.4513596	0.07354371	0.00956197	57.5782603	3.25E-14	8.34E-13	Clec4b1
ENSMUSG00000030148.16	75.1430825	0.0703048	0.00844311	59.4094916	1.28E-14	3.48E-13	Clec4a2
ENSMUSG00000030149.16	52.835769	0.0435083	0.00656044	40.6392952	1.83E-10	2.82E-09	Klrk1
ENSMUSG00000030154.11	2.41235989	0.04703895	0.01097089	17.1130867	3.52E-05	0.0002233	Klrb1f
ENSMUSG00000030156.6	11.7308915	0.07057961	0.00809855	68.2170171	1.46E-16	4.93E-15	Cd69
ENSMUSG00000030157.6	3269.44561	-0.0082357	0.00517493	2.52574021	0.11200221	0.22939251	Clec2d
ENSMUSG00000030159.16	221.347807	0.04495575	0.00500804	74.9298917	4.88E-18	1.98E-16	Clec1b
ENSMUSG00000030160.8	16.8282103	0.00170454	0.02945566	-0.0345802	1	1	Tmem52b
ENSMUSG00000030161.9	5018.20519	-0.0081993	0.0029498	7.62627531	0.0057524	0.0199482	Gabarapl1
ENSMUSG00000030162.15	3.81987718	0.09100816	0.01313657	40.1375498	2.37E-10	3.60E-09	Olr1
ENSMUSG00000030165.18	12.4782945	0.07045623	0.00822082	68.8491656	1.06E-16	3.67E-15	Klrd1
ENSMUSG00000030166.15	93.1007443	-0.0081367	0.00333794	5.88985291	0.01522837	0.04542512	Rad52
ENSMUSG00000030167.16	13.257885	0.04560252	0.00813931	29.3224484	6.13E-08	6.56E-07	Klrc1
ENSMUSG00000030168.14	8680.49798	-0.0145914	0.00344883	17.5745348	2.76E-05	0.00017921	Adipor2
ENSMUSG00000030170.15	101.395946	0.00827314	0.00417856	3.89234325	0.0485067	0.11772783	Wnt5b
ENSMUSG00000030172.16	938.518644	-0.0074461	0.00371566	3.96883825	0.04634974	0.11342654	Erc1
ENSMUSG00000030177.15	74.2685891	-0.0073048	0.00380264	3.60946226	0.05745169	0.13514777	Ccdc77
ENSMUSG00000030180.16	3095.88402	0.00307535	0.00462168	0.43789103	0.50814213	0.69494609	Kdm5a
ENSMUSG00000030187.16	79.9512839	0.05171739	0.00588247	70.4879265	4.63E-17	1.69E-15	Klra2
ENSMUSG00000030188.13	43.0059486	0.00706873	0.00473579	2.17552209	0.14022192	0.27321555	Magohb
ENSMUSG00000030189.16	432.495798	-0.0090056	0.00412921	4.64672545	0.03111284	0.08194613	Ybx3
ENSMUSG00000030199.17	366.020601	0.00457875	0.00297413	2.34012105	0.12607976	0.25173259	Etv6
ENSMUSG00000030200.14	13.2041642	0.03891202	0.00871093	17.4867193	2.89E-05	0.0001866	Bcl2l14
ENSMUSG00000030201.16	1692.15659	0.00254478	0.00276404	0.82468012	0.36381535	0.55339539	Lrp6
ENSMUSG00000030203.18	682.843845	0.00051652	0.00241698	0.04532977	0.8313988	0.93502633	Dusp16
ENSMUSG00000030204.11	551.472033	-0.0048872	0.00186746	6.86965659	0.00876715	0.02856499	Ddx47
ENSMUSG00000030206.14	4.19168061	0.00420253	0.01894806	-0.1489113	1	1	Gsg1
ENSMUSG00000030207.16	545.879478	-0.0003544	0.00303868	0.01383518	0.90636636	0.97905006	Fam234b
ENSMUSG00000030208.16	106.07623	0.04633313	0.00806389	29.8745279	4.61E-08	5.02E-07	Emp1
ENSMUSG00000030209.15	63.7272934	0.03716344	0.01191735	7.89099357	0.00496816	0.01758651	Grin2b
ENSMUSG00000030213.13	886.884949	0.01018	0.00472979	4.51579108	0.03358335	0.08727912	Atf7ip
ENSMUSG00000030214.8	907.944073	0.00658153	0.00344074	3.58334862	0.0583614	0.13683025	Plbd1
ENSMUSG00000030216.13	587.542672	-0.0020183	0.00326045	0.3736498	0.54102151	0.72467241	Wbp11
ENSMUSG00000030217.3	64.7550609	0.02130275	0.00458424	20.6237514	5.59E-06	4.19E-05	Art4
ENSMUSG00000030218.3	70.5128526	-0.0009365	0.00709461	0.01728961	0.89538773	0.97320224	Mgp
ENSMUSG00000030219.14	2.62363516	-0.0165263	0.01291946	1.62274737	0.20270913	0.36195757	Erp27
ENSMUSG00000030220.14	297.180316	0.04093237	0.00546884	51.2893279	7.97E-13	1.70E-11	Arhgdib
ENSMUSG00000030222.14	66.8237212	0.00867747	0.00313365	7.52163616	0.00609623	0.02094474	Rerg
ENSMUSG00000030223.15	23.9979048	0.06946464	0.00775503	71.4910962	2.79E-17	1.04E-15	Ptpro
ENSMUSG00000030224.11	1573.98105	-0.0023649	0.00203473	1.33935046	0.24714855	0.41852712	Strap
ENSMUSG00000030225.12	1088.20523	-0.0065903	0.00280149	5.54551144	0.01852814	0.0534301	Dera
ENSMUSG00000030226.13	7.09985345	0.00754487	0.02047842	0.13643048	0.71185571	0.85779625	Lmo3
ENSMUSG00000030228.21	150.176102	0.01623727	0.00468069	11.9616385	0.00054307	0.00257413	Pik3c2g
ENSMUSG00000030230.16	1.38984646	-0.0206737	0.08852182	0.09262392	0.76086752	0.89102056	Plcz1
ENSMUSG00000030231.12	511.9118	0.00533895	0.00281705	3.61244425	0.05734877	0.13493691	Plekha5
ENSMUSG00000030232.20	456.389162	0.00916086	0.00238267	14.3058484	0.00015538	0.00085035	Aebp2
ENSMUSG00000030235.18	3.44087152	-0.0065124	0.01119467	0.30883502	0.57839594	0.75654651	Slco1c1
ENSMUSG00000030236.11	20316.0117	-0.0103263	0.00582411	3.08892272	0.07882698	0.17412184	Slco1b2
ENSMUSG00000030237.15	1270.97849	0.04484524	0.00606403	56.7968263	4.83E-14	1.20E-12	Slco1a4
ENSMUSG00000030243.17	110.829826	0.01230094	0.004352	7.79991461	0.00522487	0.01836296	Recql
ENSMUSG00000030244.8	2880.63127	-0.0134775	0.00564349	5.76029175	0.01639235	0.04828649	Gys2
ENSMUSG00000030245.11	142.72258	0.00849688	0.00459734	3.34878666	0.06725457	0.1533217	Golt1b
ENSMUSG00000030246.13	379.720961	0.00255781	0.00585864	0.18752175	0.6649873	0.82466956	Ldhb
ENSMUSG00000030247.10	98.8695577	0.0206398	0.00474946	17.2102834	3.35E-05	0.00021285	Kcnj8
ENSMUSG00000030249.16	688.875151	0.02522511	0.00439978	30.2286543	3.84E-08	4.24E-07	Abcc9
ENSMUSG00000030254.17	39.2588056	0.01328329	0.00485449	7.36447042	0.00665252	0.02262202	Rad18
ENSMUSG00000030255.14	71.7090067	0.02086877	0.00568158	12.5703279	0.00039192	0.00192901	Sspn

ENSMUSG00000030256.12	74.8702916	0.02425666	0.01005336	4.99556989	0.02541228	0.06942021	Bhlhe41
ENSMUSG00000030257.17	132.020122	0.01585667	0.00755686	3.64886343	0.05610749	0.13274663	Srgap3
ENSMUSG00000030259.13	637.562747	-0.0060943	0.0027916	4.75283611	0.02925005	0.07782196	Rassf8
ENSMUSG00000030263.14	62.5530344	0.04361293	0.00537402	61.739063	3.92E-15	1.13E-13	Irag2
ENSMUSG00000030264.15	372.455734	-0.0045286	0.00247122	3.30782524	0.06895068	0.15631208	Thumpd3
ENSMUSG00000030265.15	686.993071	0.0020895	0.00286997	0.51733808	0.4719794	0.66271964	Kras
ENSMUSG00000030268.18	47.2141133	0.10544632	0.02181381	9.26099768	0.00234085	0.00924504	Bcat1
ENSMUSG00000030269.15	324.618436	-0.0068335	0.002585	7.04663257	0.00794144	0.02623345	Mtmr14
ENSMUSG00000030270.12	11.8296806	0.00768783	0.01812206	0.37521331	0.54017619	0.72406204	Cpne9
ENSMUSG00000030271.15	71.4305868	0.00341154	0.0032317	1.10610722	0.29292972	0.47497872	Ogg1
ENSMUSG00000030272.16	314.637693	0.01303048	0.00325583	15.7635698	7.18E-05	0.00042447	Camk1
ENSMUSG00000030275.7	1720.43329	0.00746326	0.00276559	7.10614988	0.00768199	0.02550773	Etnk1
ENSMUSG00000030276.20	17.8713149	0.01375578	0.00741386	3.2721257	0.07046626	0.15904471	Ttll3
ENSMUSG00000030278.12	231.248006	0.08591046	0.01059131	37.0400255	1.16E-09	1.59E-08	Cidec
ENSMUSG00000030279.16	132.962159	0.0120914	0.00332371	12.9293024	0.00032348	0.00162816	C2cd5
ENSMUSG00000030281.17	457.416248	-0.0202102	0.00564918	12.0848179	0.00050834	0.00243046	Il17rc
ENSMUSG00000030282.15	821.074198	-0.0031047	0.00204901	2.25913654	0.1328281	0.26227573	Cmas
ENSMUSG00000030283.8	11.1138038	0.00189987	0.01183327	0.02658936	0.87046927	0.95890467	St8sia1
ENSMUSG00000030284.14	684.60615	-0.0203988	0.00285426	48.5375725	3.24E-12	6.41E-11	Creld1
ENSMUSG00000030286.7	938.17183	-0.0062565	0.0015274	16.7501678	4.26E-05	0.00026633	Emc3
ENSMUSG00000030287.16	887.059806	-0.0093073	0.00413294	5.05189746	0.02459899	0.06752697	Itpr2
ENSMUSG00000030291.13	585.638062	-0.0047344	0.00336524	1.95356465	0.16220324	0.30459599	Med21
ENSMUSG00000030298.11	1015.13072	-0.0051485	0.00232211	4.93354265	0.02634026	0.07145865	Sec13
ENSMUSG00000030301.18	519.254474	-0.0114502	0.00418864	7.15437763	0.00747812	0.02495054	Ccdc91
ENSMUSG00000030302.18	107.01325	0.0139277	0.00737291	3.59736014	0.0578714	0.13601941	Atp2b2
ENSMUSG00000030303.16	11.8107016	0.01535208	0.00946135	2.65787605	0.10303824	0.21493411	Far2
ENSMUSG00000030304.12	1406.20046	-0.0184437	0.00298204	38.1347084	6.60E-10	9.42E-09	Ergic2
ENSMUSG00000030306.15	50.7548312	0.02575604	0.00635401	14.9941542	0.00010784	0.0006116	Tmtc1
ENSMUSG00000030307.9	9.96813651	0.01292443	0.01971674	0.708145	0.40006031	0.59181025	Slc6a11
ENSMUSG00000030309.17	40.6593463	-0.0045125	0.00558753	0.66706751	0.41407588	0.60545897	Caprin2
ENSMUSG00000030310.12	21.2986625	0.02960814	0.01340165	4.63247941	0.0313722	0.08250943	Slc6a1
ENSMUSG00000030313.16	1916.73503	0.00372978	0.00355162	1.09145553	0.29614893	0.47921026	Dennd5b
ENSMUSG00000030314.17	670.243499	-0.0047519	0.00304177	2.39988841	0.12134391	0.24436624	Atg7
ENSMUSG00000030315.16	330.754311	-0.0116749	0.00320526	12.9315787	0.00032309	0.00162653	Vgll4
ENSMUSG00000030316.14	47.3646479	0.01101067	0.00325842	11.6265443	0.00065017	0.00301454	Tamm41
ENSMUSG00000030317.9	5.45994119	0.0868018	0.02035543	15.5697252	7.95E-05	0.00046515	Timp4
ENSMUSG00000030319.9	18.6847174	0.00740934	0.00897061	0.69562058	0.40425917	0.59585406	Cand2
ENSMUSG00000030321.17	2.13790444	-0.0294446	0.01052635	8.05200396	0.00454533	0.01629275	Efcab12
ENSMUSG00000030322.13	35.078507	0.01834822	0.0044641	16.2530489	5.54E-05	0.00033672	Mbd4
ENSMUSG00000030323.14	73.6458816	0.00040739	0.00415514	0.00936887	0.92289084	0.98807551	Ift122
ENSMUSG00000030325.17	10.7038297	0.02398341	0.00711323	11.0156948	0.00090344	0.00401063	Klrb1c
ENSMUSG00000030327.9	746.335004	-0.0055662	0.00178752	9.61928712	0.00192544	0.00781621	Necap1
ENSMUSG00000030329.12	19.9591593	0.06671465	0.0086058	54.5106379	1.55E-13	3.61E-12	Pianp
ENSMUSG00000030330.16	416.824024	-0.0084569	0.00247227	11.6150373	0.00065421	0.00303086	Ing4
ENSMUSG00000030335.7	1039.52903	-0.0144952	0.00320061	20.6675648	5.46E-06	4.10E-05	Mrpl51
ENSMUSG00000030336.15	9.17002943	0.05172841	0.00818407	37.2916911	1.02E-09	1.41E-08	Cd27
ENSMUSG00000030337.17	113.811861	0.0039319	0.00408247	0.92819812	0.33533158	0.52246867	Vamp1
ENSMUSG00000030339.8	1375.86406	-0.0004607	0.00315703	0.02139063	0.8837198	0.96613585	Ltbr
ENSMUSG00000030340.17	445.66613	-0.011959	0.00561445	4.3423877	0.03717476	0.09467701	Scnn1a
ENSMUSG00000030341.18	1326.80059	0.0143206	0.00269599	27.4899074	1.58E-07	1.59E-06	Tnfrsf1a
ENSMUSG00000030342.9	580.436323	0.01920886	0.00354817	29.3018241	6.19E-08	6.62E-07	Cd9
ENSMUSG00000030344.12	2.17045865	0.00334339	0.02018831	-27.008734	1	1	Akap3
ENSMUSG00000030345.17	1.3700685	0.04573616	0.01918382	6.34325123	0.01178287	0.03653836	Dyrk4
ENSMUSG00000030346.17	13.2160047	0.03250562	0.01132139	7.19080081	0.00732783	0.02455728	Rad51ap1
ENSMUSG00000030347.7	162.599543	0.00369291	0.00337824	1.18669862	0.2759965	0.45452125	D6Wsu163e
ENSMUSG00000030350.9	11.0745264	0.00774972	0.03409127	0.13838058	0.70989627	0.85651862	Prmt8
ENSMUSG00000030352.16	632.743058	0.00422765	0.00434979	0.94643238	0.33062907	0.51756934	Tspan9
ENSMUSG00000030353.16	7.95291177	0.01006647	0.00767523	1.72135843	0.18951828	0.34374507	Tead4
ENSMUSG00000030357.11	2963.11194	-0.0132802	0.00309553	17.7795731	2.48E-05	0.00016223	Fkbp4
ENSMUSG00000030359.15	99964.5234	0.00348939	0.0057966	0.36321701	0.546725	0.72968854	Pzp
ENSMUSG00000030364.7	174.011599	-0.0261029	0.0096954	7.06762801	0.00784891	0.02597572	Clec2h

ENSMUSG00000030365.12	16.0298947	0.0715573	0.00709894	90.5826325	1.77E-21	1.09E-19	Clec2i
ENSMUSG00000030374.16	545.793077	-0.0020134	0.00224343	0.80631745	0.36921124	0.5598339	Strn4
ENSMUSG00000030376.9	8.93523088	0.03543435	0.01409641	6.62291292	0.0100675	0.03207946	Slc8a2
ENSMUSG00000030378.16	25965.2956	-0.0434901	0.00762021	30.6729419	3.05E-08	3.43E-07	Sult2a8
ENSMUSG00000030380.18	10.1145226	0.003604	0.00582008	0.38530876	0.53477568	0.7191117	Mzf1
ENSMUSG00000030382.16	14321.3364	-0.0030817	0.00522754	0.34193421	0.55871488	0.74032861	Slc27a5
ENSMUSG00000030386.20	169.422794	0.00213268	0.00250637	0.71946284	0.39632016	0.58817771	Zfp606
ENSMUSG00000030393.9	16.2297707	0.026986	0.00563795	21.8082781	3.01E-06	2.39E-05	Zik1
ENSMUSG00000030397.12	205.795959	-0.0018083	0.00267357	0.45867987	0.49824159	0.68549997	Mark4
ENSMUSG00000030399.3	219.180558	-0.0167448	0.02633081	0.71211959	0.39874106	0.59045119	Ckm
ENSMUSG00000030400.18	212.165609	-0.004906	0.00386833	1.60046655	0.20583711	0.36642422	Ercc2
ENSMUSG00000030401.17	16.8145799	0.01806763	0.00767506	5.22877828	0.02221613	0.0620988	Rtn2
ENSMUSG00000030402.6	1.72541589	0.04497838	0.01410076	9.97133247	0.00158996	0.0065953	Ppm1n
ENSMUSG00000030403.10	307.870511	0.02565505	0.00349053	53.0175551	3.31E-13	7.40E-12	Vasp
ENSMUSG00000030407.3	216.075422	-0.0068002	0.0022162	9.4430603	0.0021195	0.00849888	Qpctl
ENSMUSG00000030409.17	583.641984	0.0297413	0.00585122	24.6434278	6.90E-07	6.10E-06	Dmpk
ENSMUSG00000030410.17	274.630219	-0.0027377	0.002731	1.00904249	0.31513234	0.5005323	Dmwd
ENSMUSG00000030411.17	32.1678857	0.00689691	0.00474041	2.11992919	0.14539327	0.28092452	Nova2
ENSMUSG00000030413.8	8.00791075	0.07982161	0.01001233	52.032797	5.46E-13	1.19E-11	Pglyrp1
ENSMUSG00000030417.16	629.550938	-0.0051836	0.00341147	2.3117325	0.12840087	0.25548266	Pdcd5
ENSMUSG00000030421.10	625.764791	-0.002532	0.00206478	1.502818	0.22023827	0.38498036	Uri1
ENSMUSG00000030423.8	229.522005	0.00531452	0.00284024	3.49961322	0.06138316	0.14252641	Pop4
ENSMUSG00000030427.18	7.0677899	0.08822936	0.01094613	56.6919127	5.10E-14	1.26E-12	Lilra6
ENSMUSG00000030428.17	37.7199785	0.00074456	0.00876391	0.00658355	0.93533133	0.99456572	Ttyh1
ENSMUSG00000030431.9	215.371337	0.00057785	0.0059821	0.00885324	0.92503633	0.98907054	Tmem238
ENSMUSG00000030432.13	3710.3868	-0.0011045	0.00402562	0.07610204	0.782651	0.90510451	Rpl28
ENSMUSG00000030433.16	5.56526154	0.02442808	0.02148463	-0.1424708	1	1	Sbk2
ENSMUSG00000030435.18	934.053586	0.00087667	0.00170556	0.26392328	0.60743753	0.78025387	U2af2
ENSMUSG00000030443.17	5.93446025	0.03048669	0.00785057	14.5919678	0.00013348	0.00074078	Zfp583
ENSMUSG00000030446.18	59.0082265	0.01048876	0.00380632	7.49313462	0.00619347	0.02124215	Zfp273
ENSMUSG00000030447.16	904.500099	0.00749069	0.00214012	12.1123213	0.0005009	0.00239979	Cyfip1
ENSMUSG00000030451.17	1004.09467	-0.0016651	0.00423059	0.1538332	0.69489881	0.84623519	Herc2
ENSMUSG00000030452.17	701.794984	-0.0030854	0.00258366	1.38555441	0.23915729	0.40860296	Nipa2
ENSMUSG00000030465.21	1010.4164	-0.0032013	0.00357231	0.79992185	0.37111674	0.5616584	Psd3
ENSMUSG00000030468.13	18.5498513	0.07267156	0.00870073	59.4427187	1.26E-14	3.42E-13	Siglecg
ENSMUSG00000030469.11	113.442007	0.00448158	0.00330597	1.82492268	0.17672843	0.32577701	Zfp719
ENSMUSG00000030470.16	531.643666	-0.0335702	0.00650459	25.671026	4.05E-07	3.76E-06	Csrp3
ENSMUSG00000030471.18	110.357214	-0.0060922	0.00317579	3.58657267	0.05824826	0.13667693	Zdhhc13
ENSMUSG00000030474.10	92.954059	0.04889472	0.00568328	68.2199793	1.46E-16	4.93E-15	Siglece
ENSMUSG00000030483.15	222.43661	0.02230451	0.0106648	3.88586136	0.04869428	0.11803733	Cyp2b10
ENSMUSG00000030486.10	33.1870119	0.00095084	0.00318452	0.08848848	0.76610761	0.89425983	Zfp108
ENSMUSG00000030492.17	5.04052786	0.00705893	0.02206403	0.18208138	0.6695906	0.82788045	Slc7a9
ENSMUSG00000030493.14	39.9982264	0.01033697	0.00355007	8.41035175	0.0037309	0.01375873	Faap24
ENSMUSG00000030494.15	291.070062	-0.002305	0.00305354	0.57160364	0.44962237	0.64112166	Rhpn2
ENSMUSG00000030495.13	1.29905562	-0.0082961	0.0203511	0.10653572	0.74412318	0.87930307	Slc7a10
ENSMUSG00000030498.16	1417.45488	0.00885687	0.00393972	4.93596231	0.02630341	0.07141081	Gas2
ENSMUSG00000030499.10	118.069176	-0.0304706	0.00521349	33.2176526	8.24E-09	1.01E-07	Kctd15
ENSMUSG00000030500.7	4.76433177	0.02798443	0.02127012	2.22305571	0.13596372	0.26684677	Slc17a6
ENSMUSG00000030505.18	177.291675	-0.0016118	0.00283989	0.31775252	0.57296144	0.7520657	Prmt3
ENSMUSG00000030509.18	210.075964	0.00087175	0.00257871	0.11256144	0.7372466	0.87469707	Asb7
ENSMUSG00000030512.14	271.269216	-0.0056568	0.00247115	5.25241661	0.02191631	0.06138441	Snrpa1
ENSMUSG00000030513.15	1722.80252	-0.0032743	0.00369659	0.74371855	0.38847229	0.57990554	Pcsk6
ENSMUSG00000030515.10	237.224299	-0.0084298	0.00296793	8.02425162	0.00461551	0.01649415	Tarsl2
ENSMUSG00000030516.15	875.197197	0.00412402	0.00303055	1.82056246	0.17724634	0.32652755	Tjp1
ENSMUSG00000030518.18	15.213025	0.03142701	0.0103061	8.68599158	0.00320665	0.01208759	Entrep2
ENSMUSG00000030519.15	12.7977306	0.07670807	0.00879044	65.5495864	5.67E-16	1.80E-14	Apba2
ENSMUSG00000030521.12	282.762991	-0.0040272	0.00341517	1.36555597	0.2425769	0.412898	Mphosph10
ENSMUSG00000030522.15	637.833259	0.00378901	0.00281308	1.78937485	0.18100235	0.33194319	Mtmr10
ENSMUSG00000030523.19	3.06547511	0.04195939	0.01429344	7.34198834	0.00673623	0.02286711	Trpm1
ENSMUSG00000030525.9	1.65821993	-0.0041542	0.01308717	0.07996116	0.77735006	0.90162667	Chrna7
ENSMUSG00000030527.16	257.794078	-0.0076137	0.00430049	3.0263204	0.08192361	0.17949376	Crtc3

ENSMUSG00000030528.13	76.8756045	0.00874951	0.00606407	2.0532929	0.15187739	0.29021957	Blm
ENSMUSG00000030530.16	3498.49524	-0.0031331	0.00317222	0.96811506	0.32515073	0.51188126	Furin
ENSMUSG00000030532.7	38.4617525	-0.0059117	0.00505782	1.35616782	0.24420274	0.41475954	Hddc3
ENSMUSG00000030533.17	242.948252	-0.0066522	0.00298677	4.90129455	0.02683657	0.07263151	Unc45a
ENSMUSG00000030534.14	106.242829	0.00015564	0.00300112	0.0026507	0.95893906	1	Vps33b
ENSMUSG00000030536.11	451.370027	0.05227288	0.00477922	111.610575	4.35E-26	4.73E-24	Iqgap1
ENSMUSG00000030538.16	828.610001	-0.0100746	0.00360294	7.83127576	0.00513498	0.01809288	Cib1
ENSMUSG00000030539.14	117.792785	0.01586587	0.00348314	19.8419654	8.41E-06	6.05E-05	Sema4b
ENSMUSG00000030541.17	2433.80612	0.00832446	0.0037755	4.92022853	0.02654401	0.07194518	Idh2
ENSMUSG00000030543.11	3.16965218	0.01451194	0.00894811	2.58012819	0.1082129	0.22327963	Mesp2
ENSMUSG00000030544.6	9.84754269	0.01600497	0.01354427	1.37156642	0.24154293	0.41154602	Mesp1
ENSMUSG00000030545.10	1716.52027	-0.0083301	0.0040584	3.90192448	0.04823083	0.1171306	Pex11a
ENSMUSG00000030546.15	2.72415348	0.01203645	0.02107717	0.26001035	0.61011304	0.78236972	Plin1
ENSMUSG00000030549.7	1.53675078	-0.003442	0.04569298	-0.029972	1	1	Rhcg
ENSMUSG00000030551.15	663.518736	0.00079152	0.00332629	0.05604487	0.81286007	0.92326471	Nr2f2
ENSMUSG00000030553.4	3.21639724	-0.0092317	0.00889292	1.03096663	0.30993174	0.49512667	Pgpep1l
ENSMUSG00000030554.17	32.3794979	0.03431336	0.00561299	35.0310428	3.24E-09	4.22E-08	Synm
ENSMUSG00000030555.17	742.666675	-0.0017519	0.00349657	0.25104835	0.61633786	0.78773951	Ttc23
ENSMUSG00000030556.14	527.946481	-0.0053128	0.00386442	1.86032543	0.17258725	0.31999393	Lrrc28
ENSMUSG00000030557.18	731.927456	0.00756078	0.00311024	5.66351136	0.01732141	0.05055597	Mef2a
ENSMUSG00000030559.9	18.1214172	0.04340354	0.00630318	44.3388729	2.76E-11	4.77E-10	Rab38
ENSMUSG00000030560.18	2536.75432	0.03220264	0.00411184	57.3773342	3.60E-14	9.17E-13	Ctsc
ENSMUSG00000030562.18	776.27808	-0.006283	0.00450627	1.82583197	0.17662065	0.3256933	Nox4
ENSMUSG00000030577.15	57.7087057	0.0696185	0.00709072	84.0995248	4.70E-20	2.47E-18	Cd22
ENSMUSG00000030579.11	470.652922	0.05507146	0.00627293	68.3308231	1.38E-16	4.68E-15	Tyrobp
ENSMUSG00000030583.17	161.566871	-0.0075956	0.00486679	2.42570482	0.11935955	0.24119382	Sipa1l3
ENSMUSG00000030584.15	6.34984367	0.02774834	0.01225804	4.44333324	0.03503778	0.09036077	Dpf1
ENSMUSG00000030587.6	247.482701	-0.0022945	0.00634032	0.1313506	0.71703578	0.86110009	2200002D01Rik
ENSMUSG00000030588.13	428.812573	-0.0140206	0.00240827	33.9865193	5.55E-09	6.97E-08	Yif1b
ENSMUSG00000030589.17	26.0279289	0.05357447	0.00906729	31.6393433	1.86E-08	2.15E-07	Rasgrp4
ENSMUSG00000030590.16	178.247563	-0.0027327	0.00373447	0.54325446	0.4610871	0.65228555	Fam98c
ENSMUSG00000030591.18	1980.64915	-0.000766	0.00208111	0.13521924	0.71308077	0.85846738	Psmd8
ENSMUSG00000030592.20	10.3014589	0.09291162	0.01264274	41.072937	1.47E-10	2.30E-09	Ryr1
ENSMUSG00000030595.16	289.641309	0.00220213	0.00245562	0.8015393	0.37063354	0.56123284	Nfkbib
ENSMUSG00000030598.16	16.2932199	0.00719121	0.00453164	2.51501017	0.11276691	0.23056288	Fbxo17
ENSMUSG00000030600.16	6.64431527	0.00100154	0.00947955	0.01107432	0.91618965	0.98411736	Lrfn1
ENSMUSG00000030602.14	210.102197	0.00136636	0.00275842	0.24198128	0.62277878	0.79240346	Pak4
ENSMUSG00000030603.17	1407.07452	-0.0037109	0.00198219	3.514678	0.06082748	0.1415427	Psmc4
ENSMUSG00000030604.10	114.629333	0.00203451	0.00310497	0.42114524	0.51636614	0.70282266	Zfp626
ENSMUSG00000030605.16	297.695892	0.02740807	0.004401	38.1925983	6.41E-10	9.15E-09	Mfge8
ENSMUSG00000030609.19	329.221592	0.00215286	0.00289724	0.54549446	0.46016452	0.65164383	Aen
ENSMUSG00000030610.14	103.045785	0.00480462	0.00234378	4.16024281	0.04138347	0.10344113	Det1
ENSMUSG00000030611.11	232.988882	-0.0136618	0.00378617	13.0994316	0.00029539	0.00150409	Mrps11
ENSMUSG00000030612.7	425.735461	-0.0145167	0.00375854	15.095858	0.00010219	0.00058288	Mrpl46
ENSMUSG00000030613.14	301.891141	-0.0049451	0.0036233	1.83787978	0.17519965	0.32374326	Ccdc90b
ENSMUSG00000030614.9	529.585684	-0.0065026	0.00220727	8.64009212	0.00328845	0.01235867	Tmem126b
ENSMUSG00000030615.13	784.379037	-0.0143685	0.00300591	23.1779822	1.48E-06	1.24E-05	Tmem126a
ENSMUSG00000030616.17	30.8159231	0.03719326	0.00496902	55.0276861	1.19E-13	2.80E-12	Sytl2
ENSMUSG00000030617.14	1.17826944	-0.0148215	0.04534204	0.05303063	0.81787124	0.92634994	Ccdc83
ENSMUSG00000030619.11	346.705515	-0.0015176	0.00209948	0.51958453	0.47101896	0.66181139	Eed
ENSMUSG00000030621.18	16.3003984	0.02596485	0.00939884	7.84791119	0.00508795	0.01795934	Me3
ENSMUSG00000030629.16	2235.73156	-0.0063234	0.00330469	3.60236602	0.0576974	0.135651	Zfand6
ENSMUSG00000030630.17	8702.62957	-0.0058203	0.0035183	2.73872281	0.09794302	0.20678812	Fah
ENSMUSG00000030638.14	3.42914349	0.03622989	0.02055285	3.12278098	0.07720492	0.17127414	Sh3gl3
ENSMUSG00000030641.11	22.1773502	0.0194257	0.00643351	8.3844357	0.00378448	0.01392522	Ddias
ENSMUSG00000030643.15	259.501762	-0.0107688	0.00536116	3.83472962	0.05020109	0.12094411	Rab30
ENSMUSG00000030647.9	1284.75689	-0.0103004	0.00335358	9.59846897	0.0019474	0.00789804	Ndufc2
ENSMUSG00000030649.19	211.563197	0.00537806	0.00423935	1.54072705	0.21450962	0.37756894	Anapc15
ENSMUSG00000030650.19	11.5905534	0.02186423	0.01823616	0.54696949	0.45955861	0.65107817	Tmc5
ENSMUSG00000030651.6	20.5240055	0.05307541	0.00801956	39.2039139	3.82E-10	5.62E-09	Art2b
ENSMUSG00000030652.12	407.634081	-0.0027461	0.00303232	0.81663809	0.36616495	0.55600119	Coq7

ENSMUSG00000030653.18	59.6151958	0.01223855	0.04229269	-0.5595061	1	1	Gm45837
ENSMUSG00000030654.10	3384.36612	-0.0062592	0.00295787	4.35942765	0.03680467	0.09400855	Arl6ip1
ENSMUSG00000030655.16	1087.96025	0.00647271	0.00352013	3.3356784	0.06779245	0.15427949	Smg1
ENSMUSG00000030657.12	18.6686725	0.05304528	0.00817805	36.8310322	1.29E-09	1.76E-08	Xylt1
ENSMUSG00000030659.15	86.1567036	-0.0068487	0.00571856	1.37333497	0.24123971	0.4111482	Nucb2
ENSMUSG00000030660.10	830.751179	0.00130351	0.00300077	0.18450272	0.66753189	0.82629235	Pik3c2a
ENSMUSG00000030662.11	996.155946	0.0012033	0.00239965	0.24662788	0.61945957	0.79001694	Ipo5
ENSMUSG00000030663.13	833.756338	0.00181834	0.00189933	0.91590439	0.33855265	0.52623585	1110004F10Rik
ENSMUSG00000030670.17	415.761117	-0.0014912	0.0036131	0.16528301	0.68433843	0.83865771	Cyp2r1
ENSMUSG00000030671.10	1530.27338	-0.0020323	0.00364961	0.30778597	0.57904199	0.75713669	Pde3b
ENSMUSG00000030672.13	14.9192699	0.00598923	0.00708917	0.68504321	0.4078555	0.5994982	Mylpf
ENSMUSG00000030674.6	2610.88787	-0.0065498	0.00410008	2.55065764	0.11024836	0.22664671	Qprt
ENSMUSG00000030677.9	30.309302	0.00453877	0.00935611	0.22437589	0.63572575	0.80324785	Kif22
ENSMUSG00000030678.8	792.966513	-0.0015379	0.0025231	0.36759342	0.54431899	0.72775396	Maz
ENSMUSG00000030680.7	43.8782082	-0.0159298	0.01115485	1.75564722	0.18516837	0.33776257	Pagr1a
ENSMUSG00000030681.19	860.933236	0.01973313	0.00292761	44.6108647	2.40E-11	4.20E-10	Mvp
ENSMUSG00000030682.5	674.568079	-0.004923	0.00210823	5.4707238	0.01933762	0.05534486	Cdipt
ENSMUSG00000030683.12	12.4487586	0.0225742	0.00852147	6.72483953	0.00950793	0.03059431	Sez6l2
ENSMUSG00000030685.6	53.6489307	0.01000719	0.00334486	8.89108671	0.00286566	0.01098302	Kctd13
ENSMUSG00000030688.16	13263.3684	-0.0020799	0.00366842	0.32026061	0.57145107	0.75087716	Stard10
ENSMUSG00000030689.16	178.990064	0.00350735	0.00343981	1.02715566	0.31082766	0.49598682	Ino80e
ENSMUSG00000030691.16	456.422053	0.00029817	0.00239094	0.01544593	0.90109228	0.97642803	Fchsd2
ENSMUSG00000030695.17	1997.12906	-0.0013443	0.00410223	0.1059536	0.74479879	0.87970475	Aldoa
ENSMUSG00000030697.8	553.460839	0.00119246	0.00297513	0.16120581	0.68804877	0.84149952	Ppp4c
ENSMUSG00000030699.17	5.57663928	0.0026747	0.0071249	0.14086605	0.70742156	0.85495208	Tbx6
ENSMUSG00000030701.18	713.123639	0.02826169	0.00415055	41.9750825	9.24E-11	1.49E-09	Plekhb1
ENSMUSG00000030703.9	8.73978934	0.02394726	0.00773802	9.07501887	0.00259125	0.01008202	Gdpd3
ENSMUSG00000030704.15	1344.34505	0.00089528	0.00225573	0.15574966	0.69310024	0.84495099	Rab6a
ENSMUSG00000030706.18	686.851117	-0.0036621	0.00273686	1.78618009	0.18139228	0.33243702	Mrpl48
ENSMUSG00000030707.16	193.589617	0.069596	0.00574865	126.902095	1.95E-29	3.54E-27	Coro1a
ENSMUSG00000030708.15	7.38050699	0.0377026	0.00978618	13.0844064	0.00029776	0.0015149	Dnajb13
ENSMUSG00000030711.17	5493.49229	0.02065354	0.00355876	32.8680911	9.86E-09	1.19E-07	Sult1a1
ENSMUSG00000030714.15	234.263526	-0.0054173	0.00431735	1.57728287	0.20915251	0.37065507	Sgf29
ENSMUSG00000030717.10	51.3122812	0.0449536	0.00822584	28.1091951	1.15E-07	1.18E-06	Nupr1
ENSMUSG00000030718.10	496.419934	-0.0084491	0.00276138	9.32846468	0.00225621	0.00897084	Ppme1
ENSMUSG00000030720.17	198.776915	0.01207466	0.00302412	15.8553589	6.84E-05	0.00040672	Cln3
ENSMUSG00000030722.8	68.6553635	-0.0065336	0.00304436	4.5471462	0.03297366	0.08591657	Nfatc2ip
ENSMUSG00000030724.8	36.5176194	0.0745184	0.00869847	59.2169464	1.41E-14	3.81E-13	Cd19
ENSMUSG00000030725.5	75.2272071	0.00392735	0.00431102	0.81463139	0.36675451	0.55675304	Lipt2
ENSMUSG00000030726.17	201.35485	0.01545484	0.00265174	33.4057609	7.48E-09	9.22E-08	Pold3
ENSMUSG00000030727.13	171.940645	-0.0010739	0.00284639	0.14139344	0.70689965	0.85458407	Rabep2
ENSMUSG00000030729.18	156.339486	0.01108184	0.00447685	5.9558251	0.01466874	0.04408471	Pgm2l1
ENSMUSG00000030730.13	5.26222716	0.01388593	0.01938001	0.74127799	0.3892518	0.58081185	Atp2a1
ENSMUSG00000030731.14	74.4891388	-0.00689	0.00460462	2.13279277	0.14417786	0.2792166	Syt3
ENSMUSG00000030732.14	2.066079	0.02753945	0.00981392	8.46232867	0.00362577	0.01343136	Chrdl2
ENSMUSG00000030733.11	593.587949	-0.0059092	0.00220727	7.17907681	0.00737587	0.02465521	Sh2b1
ENSMUSG00000030737.18	3079.11413	0.00095135	0.00347191	0.07376043	0.78593827	0.90757112	Slco2b1
ENSMUSG00000030738.12	2575.12911	-0.0032463	0.00286727	1.26068332	0.26152179	0.43591893	Eif3c
ENSMUSG00000030739.20	366.300359	-0.0054747	0.00442955	1.48509197	0.22297953	0.38844784	Myh14
ENSMUSG00000030741.16	425.564559	-0.0168054	0.00266929	39.3719607	3.50E-10	5.17E-09	Spns1
ENSMUSG00000030742.8	18.0426551	0.06462502	0.00766025	61.7168219	3.97E-15	1.14E-13	Lat
ENSMUSG00000030744.14	11692.7786	-0.0058036	0.00401537	2.06095757	0.15111518	0.28901146	Rps3
ENSMUSG00000030745.10	19.8981583	0.09730995	0.00813507	116.564901	3.57E-27	4.56E-25	Il21r
ENSMUSG00000030747.6	7939.76222	-0.0071897	0.0027739	6.81271396	0.00905111	0.02936633	Dgat2
ENSMUSG00000030748.10	594.515911	0.01435435	0.00251497	31.9016352	1.62E-08	1.90E-07	Il4ra
ENSMUSG00000030750.14	287.05686	-0.0033514	0.00357169	0.88082243	0.34797652	0.5360443	Nsmce1
ENSMUSG00000030751.19	1685.45692	-0.0064502	0.00190811	11.417409	0.00072759	0.00332356	Psma1
ENSMUSG00000030752.9	44.454914	-0.0055327	0.00302899	3.28945084	0.06972634	0.15769158	Kdm8
ENSMUSG00000030753.16	789.094721	0.00157602	0.00308678	0.2549425	0.61361621	0.78537859	Thap12
ENSMUSG00000030754.10	1863.87299	-0.0025854	0.00246913	1.08898712	0.29669574	0.47996335	Copb1
ENSMUSG00000030757.14	6.1879955	0.03074251	0.00953705	9.84963007	0.00169866	0.00699164	Zkscan2

ENSMUSG00000030759.17	165.934719	0.04077411	0.0051666	57.1806078	3.98E-14	1.01E-12	Far1
ENSMUSG00000030760.15	416.958766	0.00943332	0.00314768	8.83551163	0.00295425	0.01127855	Acer3
ENSMUSG00000030761.17	293.389405	0.00736498	0.00327716	4.9860188	0.02555293	0.06976404	Myo7a
ENSMUSG00000030762.12	2771.0707	0.00831216	0.0063235	1.59074469	0.20721977	0.36806126	Aqp8
ENSMUSG00000030763.8	176.143855	-0.0021005	0.002314	0.83105653	0.3619672	0.5515802	Lcmt1
ENSMUSG00000030766.16	246.810039	0.01147544	0.00308088	13.9142199	0.00019135	0.00102547	Arhgap17
ENSMUSG00000030768.13	46.8577461	-0.0034014	0.00492924	0.46203338	0.4966752	0.68382355	Disp1
ENSMUSG00000030769.16	6.53932121	0.07010271	0.01343156	21.9822945	2.75E-06	2.20E-05	Slc5a11
ENSMUSG00000030770.16	1553.11604	0.00049156	0.00191443	0.06500261	0.79875701	0.91540133	Parva
ENSMUSG00000030772.7	89.4936362	0.05230953	0.00525353	88.8577005	4.24E-21	2.48E-19	Dkk3
ENSMUSG00000030774.14	83.5389832	0.0427706	0.00640376	44.3572076	2.74E-11	4.74E-10	Pak1
ENSMUSG00000030775.12	5.90907645	0.04141477	0.01399905	7.19194116	0.00732317	0.02454517	Trat1
ENSMUSG00000030779.17	1312.86376	0.00027581	0.00347143	0.00619669	0.9372561	0.99559189	Rbbp6
ENSMUSG00000030780.16	313.750617	-0.0024328	0.00229579	1.13332257	0.28706774	0.46801546	Rusf1
ENSMUSG00000030781.14	27.0285981	-0.0013448	0.02069939	0.00502304	0.94349852	0.99884896	Slc5a2
ENSMUSG00000030782.18	46.0604653	0.00254713	0.00523966	0.23710034	0.62630829	0.79505422	Tgfb1i1
ENSMUSG00000030785.9	180.006815	-0.0324296	0.01825392	-0.3393595	1	1	Cox6a2
ENSMUSG00000030786.19	93.9900658	0.07576238	0.00694859	105.358344	1.02E-24	9.35E-23	Itgam
ENSMUSG00000030787.5	227.575905	0.00612302	0.00651562	0.82602494	0.36342448	0.55301483	Lyve1
ENSMUSG00000030788.17	1344.58336	0.00348113	0.00286046	1.44738456	0.22894809	0.39591575	Rnf141
ENSMUSG00000030789.10	53.6872439	0.10755816	0.00794106	160.990408	6.87E-37	3.31E-34	Itgax
ENSMUSG00000030790.16	37.3440151	0.03730551	0.00503472	52.142418	5.16E-13	1.13E-11	Adm
ENSMUSG00000030792.9	2.96373046	0.03959398	0.0154381	5.59324965	0.01802982	0.05226143	Dkkl1
ENSMUSG00000030793.5	88.0471651	0.05407742	0.00764116	43.7017568	3.82E-11	6.49E-10	Pycard
ENSMUSG00000030795.19	1549.08277	0.01197846	0.00469484	6.50960034	0.01072936	0.03388628	Fus
ENSMUSG00000030796.18	21.8241496	0.0158975	0.00757381	4.2677946	0.03884131	0.09815827	Tead2
ENSMUSG00000030798.16	68.0079832	0.05817734	0.0060974	80.3301349	3.17E-19	1.48E-17	Cd37
ENSMUSG00000030800.11	151.239758	0.0335345	0.00539429	37.8027857	7.83E-10	1.10E-08	Prss8
ENSMUSG00000030801.11	284.086879	-0.0066325	0.00188254	12.3525846	0.00044038	0.00213367	Kat8
ENSMUSG00000030802.15	268.643001	-0.0006535	0.00349537	0.03503704	0.85151806	0.94843175	Bckdk
ENSMUSG00000030804.9	5.18177817	-0.0150243	0.01484119	0.93345902	0.33396571	0.5208911	Gm21974
ENSMUSG00000030805.15	448.88101	0.00357936	0.00347101	1.04742688	0.30610045	0.49106823	Stx4a
ENSMUSG00000030806.7	107.250539	-0.0034137	0.00658347	0.24788746	0.61856653	0.78943294	Stx1b
ENSMUSG00000030811.15	74.858895	0.0006432	0.00431226	0.02183173	0.88253562	0.96537822	Fbxl19
ENSMUSG00000030814.18	288.43132	-0.0062459	0.00442015	1.98548281	0.15881399	0.29988888	Bcl7c
ENSMUSG00000030815.12	378.981083	-0.0029722	0.00312798	0.91598535	0.3385313	0.52623585	Phkg2
ENSMUSG00000030816.9	567.224351	-0.0015254	0.00263649	0.33437	0.56309713	0.74379531	Rnf40
ENSMUSG00000030822.16	493.033113	-0.0075337	0.00279462	7.33579495	0.00675948	0.02293283	Prr14
ENSMUSG00000030823.7	49.0837085	-0.0005759	0.00304141	0.03610493	0.84929896	0.94711893	9130019O22Rik
ENSMUSG00000030824.18	2601.65545	9.11E-05	0.00314763	0.00082757	0.97705006	1	Nucb1
ENSMUSG00000030826.20	364.137833	0.006599	0.00313962	4.46372313	0.03462196	0.08953876	Bcat2
ENSMUSG00000030827.6	82.4732375	-0.0053919	0.00963781	0.25499116	0.61358237	0.78537859	Fgf21
ENSMUSG00000030830.20	302.525963	0.05414966	0.00506769	107.521629	3.42E-25	3.25E-23	Itgal
ENSMUSG00000030834.8	2365.98684	-0.0073866	0.00500424	2.12339302	0.14506486	0.28047379	Abcc6
ENSMUSG00000030835.7	737.439273	-0.0073463	0.00238039	9.52429066	0.0020277	0.00817667	Nomo1
ENSMUSG00000030838.21	8.12796741	-0.004828	0.01402719	0.1352737	0.71302555	0.85846738	Ush1c
ENSMUSG00000030839.13	64.2211559	0.01036904	0.00391969	7.10970015	0.00766679	0.02546528	Sergef
ENSMUSG00000030842.13	888.000979	-0.0026526	0.00265013	1.00681614	0.3156668	0.50113194	Lamtor1
ENSMUSG00000030844.12	62.3284247	0.04985846	0.00642073	55.1210133	1.13E-13	2.68E-12	Rgs10
ENSMUSG00000030846.17	938.884792	-0.0014317	0.00221343	0.41421024	0.51984025	0.70585052	Tial1
ENSMUSG00000030847.9	762.322316	0.00400801	0.00397845	0.96629482	0.32560598	0.51234611	Bag3
ENSMUSG00000030849.19	656.921781	0.00633911	0.00344585	3.36883003	0.06644092	0.15185415	Fgfr2
ENSMUSG00000030850.18	564.395106	-0.0016011	0.00296375	0.28684957	0.59224651	0.7678525	Ate1
ENSMUSG00000030851.16	7.05408635	-0.0763263	0.05048624	2.10204968	0.14710183	0.28334371	Ldhc
ENSMUSG00000030852.19	465.649575	-0.0077167	0.00471638	2.49235667	0.11440031	0.23315006	Tacc2
ENSMUSG00000030854.18	4.46420652	0.04745066	0.01770827	7.94718409	0.00481623	0.01711807	Ptpn5
ENSMUSG00000030861.16	2880.22527	-0.0226779	0.00310993	51.9758916	5.62E-13	1.22E-11	Acadsb
ENSMUSG00000030862.14	63.7384371	0.01540088	0.00973322	2.15927374	0.14171166	0.27532217	Cpxm2
ENSMUSG00000030865.5	57.3499426	0.02457975	0.00666094	12.509109	0.00040497	0.00198538	Chp2
ENSMUSG00000030867.8	15.631667	0.01574804	0.01026991	2.30697033	0.12879487	0.2560482	Plk1
ENSMUSG00000030868.7	444.449878	1.86E-05	0.00178535	0.00010569	0.99179741	1	Dctn5

ENSMUSG00000030869.12	1587.29091	-0.0173564	0.00409186	17.8619535	2.38E-05	0.00015609	Ndufab1
ENSMUSG00000030870.13	755.550937	-0.0039867	0.00301364	1.69752249	0.1926123	0.34779924	Ubfd1
ENSMUSG00000030871.12	195.45973	-0.0109198	0.00235545	21.3682132	3.79E-06	2.94E-05	Ears2
ENSMUSG00000030872.15	297.415539	0.01059465	0.00280656	13.8417041	0.00019887	0.00106171	Gga2
ENSMUSG00000030873.10	1.04180948	-0.0167275	0.01943899	1.15285023	0.28295329	0.46293953	Scnn1b
ENSMUSG00000030876.8	736.597068	-0.0018902	0.00200687	0.8822209	0.34759411	0.53577015	Mettl9
ENSMUSG00000030878.12	28.1555215	0.01209641	0.00641401	3.3450164	0.06740881	0.15358425	Cdr2
ENSMUSG00000030879.11	1119.59163	-0.006115	0.00363238	2.88742231	0.08927365	0.19232813	Mrpl17
ENSMUSG00000030880.15	229.220934	-0.0052991	0.00199741	7.05978505	0.00788335	0.02606498	Polr3e
ENSMUSG00000030881.16	229.651376	-0.0051438	0.00309108	2.78066219	0.09540871	0.20267126	Arfip2
ENSMUSG00000030882.20	41.0076847	-0.0028044	0.00900263	0.09301428	0.76037954	0.89065466	Dnhd1
ENSMUSG00000030884.13	4488.67935	-0.0159811	0.00279739	32.8519109	9.95E-09	1.20E-07	Uqcrc2
ENSMUSG00000030887.5	8.28486596	0.01129952	0.01151402	1.03769089	0.30835912	0.49327445	Pdzd9
ENSMUSG00000030888.15	387.875337	-0.0044047	0.00483044	0.81782406	0.36581713	0.55565191	Rrp8
ENSMUSG00000030889.15	4.2460914	0.00520678	0.01435879	0.13313079	0.71520776	0.85984354	Vwa3a
ENSMUSG00000030890.17	1028.48789	-0.0055335	0.00190257	8.43581594	0.00367902	0.01359657	Ilk
ENSMUSG00000030894.6	1516.96004	0.00228219	0.00241129	0.89571498	0.34393329	0.53203978	Tpp1
ENSMUSG00000030895.10	86767.1025	0.01872929	0.00679646	7.48298167	0.00622848	0.02133077	Hpx
ENSMUSG00000030905.6	17.6199443	0.01795313	0.00574151	9.98584177	0.00157748	0.00655333	Crym
ENSMUSG00000030909.7	1273.86387	0.00523973	0.00451353	1.34765296	0.24568885	0.41668067	Anks4b
ENSMUSG00000030917.14	52.3284467	0.01489659	0.00453741	10.6661786	0.00109112	0.00473945	Ldaf1
ENSMUSG00000030921.18	229.599381	0.03873346	0.00443581	74.0334641	7.68E-18	3.05E-16	Trim30a
ENSMUSG00000030922.13	52.4959956	-0.0053207	0.00363883	2.11295379	0.14605716	0.2818148	Lyrm1
ENSMUSG00000030924.17	59.7738332	-0.0028301	0.00615959	0.21431994	0.64340235	0.80913291	Rexo5
ENSMUSG00000030929.18	128.245817	-0.0011957	0.00271999	0.19008109	0.66284914	0.82289764	Eri2
ENSMUSG00000030930.15	277.18596	0.00972679	0.00255907	14.1618899	0.00016773	0.0009124	Chst15
ENSMUSG00000030934.10	11358.4095	0.00602239	0.00396992	2.21772	0.13643441	0.26747087	Oat
ENSMUSG00000030935.16	2351.4523	-0.0147755	0.00506383	8.67372432	0.00322831	0.01215757	Acsm3
ENSMUSG00000030942.9	366.610709	-0.0003024	0.00172476	0.03055523	0.86123637	0.95391409	Thumpd1
ENSMUSG00000030945.18	170.003672	-0.0941611	0.01058947	73.798492	8.65E-18	3.42E-16	Acsm2
ENSMUSG00000030946.14	913.996916	0.00495933	0.00371528	1.78627246	0.18138099	0.33243702	Lhpp
ENSMUSG00000030956.16	243.425063	0.00873512	0.0035668	5.83897452	0.01567496	0.04651605	Fam53b
ENSMUSG00000030960.17	79.540303	0.00279345	0.0030731	0.83021401	0.36221065	0.55170168	Eef1akmt2
ENSMUSG00000030963.7	37.6713535	-0.0061759	0.02382723	0.08451396	0.77127094	0.8974886	Umod
ENSMUSG00000030965.20	556.669898	-0.0014129	0.00285351	0.24193909	0.6228091	0.79240346	Abraxas2
ENSMUSG00000030966.14	142.587088	0.02213995	0.00350337	39.2742077	3.68E-10	5.43E-09	Trim21
ENSMUSG00000030967.16	985.800305	-0.0094043	0.00246853	14.6617945	0.00012863	0.0007167	Zranb1
ENSMUSG00000030968.4	143.699443	-0.0436867	0.00480668	80.8627036	2.42E-19	1.16E-17	Pdilt
ENSMUSG00000030970.17	51.9230933	0.0179054	0.00481072	13.383592	0.00025384	0.00131524	Ctbp2
ENSMUSG00000030972.7	1911.44333	-0.0069478	0.00317403	4.8160536	0.02819583	0.07557982	Acsm5
ENSMUSG00000030978.11	227.60013	0.01018781	0.00453766	4.99431269	0.02543075	0.06946262	Rrm1
ENSMUSG00000030979.14	453.068813	-0.0137251	0.00243934	31.7082733	1.79E-08	2.08E-07	Uros
ENSMUSG00000030980.19	816.887957	-0.0040313	0.00365927	1.19957533	0.27340657	0.4511065	Knop1
ENSMUSG00000030982.20	477.59888	0.0033598	0.00223656	2.27931775	0.13110954	0.25953416	Vps35l
ENSMUSG00000030983.5	1349.56531	-0.0052504	0.00225167	5.52476608	0.01874911	0.05396174	Bccip
ENSMUSG00000030986.14	545.282848	-0.0021674	0.00282626	0.58544576	0.44418564	0.63652485	Dhx32
ENSMUSG00000030987.6	677.051501	-0.0001255	0.00257254	0.00236571	0.96120735	1	Stim1
ENSMUSG00000030990.19	419.730889	-0.0154727	0.00280785	30.4218991	3.48E-08	3.86E-07	Pgap2
ENSMUSG00000030994.17	4.18988466	0.01218132	0.01759878	0.80137185	0.37068353	0.56127249	D7Ertd443e
ENSMUSG00000030996.9	14.0250169	0.00360338	0.04458935	-0.0969419	1	1	Art1
ENSMUSG00000031004.9	123.077673	0.04750905	0.00849518	29.0271779	7.14E-08	7.57E-07	Mki67
ENSMUSG00000031007.3	819.683184	0.01121958	0.00255056	19.0708063	1.26E-05	8.76E-05	Atp6ap2
ENSMUSG00000031010.18	2857.03856	0.00014485	0.00291222	0.00242914	0.9606911	1	Usp9x
ENSMUSG00000031012.18	404.042172	-0.0023738	0.0034761	0.45270342	0.50105393	0.6878446	Cask
ENSMUSG00000031015.9	199.099588	0.02426835	0.00326508	53.0712136	3.22E-13	7.24E-12	Swap70
ENSMUSG00000031016.10	177.838623	0.02142936	0.00676188	9.93550649	0.00162121	0.00671074	Wee1
ENSMUSG00000031021.14	392.967366	0.00429797	0.0021592	3.96380438	0.04648853	0.11369539	Tmem9b
ENSMUSG00000031023.6	242.836332	-0.013014	0.00296143	19.2679019	1.14E-05	7.96E-05	Akip1
ENSMUSG00000031024.14	765.729929	-0.003406	0.00522779	0.39433123	0.53003146	0.71456542	Dennd2b
ENSMUSG00000031026.16	6.48261996	-0.0041482	0.0135631	0.11675629	0.73257953	0.87173305	Trim66
ENSMUSG00000031027.16	1.60109921	-0.0042452	0.04812292	-0.040044	1	1	Stk33

ENSMUSG00000031028.15	11.0675164	0.05287767	0.0141352	13.2040023	0.00027935	0.00143391	Tub
ENSMUSG00000031029.7	1154.35985	0.00098097	0.00253193	0.14937574	0.69913264	0.84946128	Eif3f
ENSMUSG00000031059.10	2566.30909	-0.0170499	0.00362024	22.5020462	2.10E-06	1.71E-05	Ndufb11
ENSMUSG00000031060.17	440.302385	-0.0090302	0.00245613	13.4808004	0.00024102	0.00125738	Rbm10
ENSMUSG00000031065.15	502.222762	-0.0015417	0.00245634	0.39709627	0.52859269	0.71319645	Cdk16
ENSMUSG00000031066.11	36.7442563	0.03757727	0.0052351	48.6337367	3.09E-12	6.11E-11	Usp11
ENSMUSG00000031068.9	1512.67194	-0.0084121	0.00275092	9.28584558	0.00230931	0.00913117	Glrx3
ENSMUSG00000031070.16	5.30114222	0.00796414	0.00710441	1.24799037	0.26393665	0.43895148	Mrgprf
ENSMUSG00000031072.15	286.433076	0.00277649	0.0036912	0.54982696	0.45838841	0.6501077	LTO1
ENSMUSG00000031075.20	66.6172343	0.01158621	0.00503098	5.10922033	0.02379901	0.06574415	Ano1
ENSMUSG00000031077.8	184.831044	0.00212583	0.00218644	0.93828259	0.33271991	0.51969244	Fadd
ENSMUSG00000031078.16	948.973435	0.01550129	0.00290977	28.0671006	1.17E-07	1.20E-06	Cttn
ENSMUSG00000031079.13	7.09749254	0.03817816	0.00848335	17.7869006	2.47E-05	0.0001617	Zfp300
ENSMUSG00000031089.8	1.1901115	0.00665139	0.01625781	0.25028606	0.61687373	0.78816813	Slc6a14
ENSMUSG00000031090.15	347.612886	-0.0062855	0.00276045	5.30860157	0.02122038	0.05975063	Nadsyn1
ENSMUSG00000031093.15	54.6561171	0.05384499	0.00689243	56.8500207	4.70E-14	1.18E-12	Dock11
ENSMUSG00000031095.16	504.44933	0.00445464	0.00198825	4.89318031	0.02696297	0.07286305	Cul4b
ENSMUSG00000031097.16	5.9468486	0.00901091	0.01331806	0.40083297	0.52665939	0.71159982	Tnni2
ENSMUSG00000031099.17	7.40840684	-0.0057891	0.01011606	0.28918884	0.59074081	0.76678294	Smarca1
ENSMUSG00000031101.7	52.2283739	0.07196083	0.00612166	125.919101	3.20E-29	5.68E-27	Sash3
ENSMUSG00000031103.13	73.8290489	0.04772975	0.00550312	70.0892548	5.67E-17	2.04E-15	Elf4
ENSMUSG00000031104.3	2.09145493	0.0628404	0.01659432	16.2554355	5.54E-05	0.00033638	Rab33a
ENSMUSG00000031105.17	60.4850694	-0.0076976	0.00450973	2.86666645	0.090432	0.19411442	Slc25a14
ENSMUSG00000031107.7	27.67989	0.01440079	0.00527288	7.11904335	0.00762694	0.02536863	Rbmx2
ENSMUSG00000031109.17	275.442445	0.00088868	0.0036112	0.05906462	0.8079805	0.92067471	Enox2
ENSMUSG00000031111.17	12.1852115	-0.0099413	0.00833365	1.36548197	0.24258966	0.412898	Igsf1
ENSMUSG00000031112.11	15.0187177	0.04821403	0.00672801	45.8326355	1.29E-11	2.34E-10	Stk26
ENSMUSG00000031119.5	816.561035	-0.005123	0.00272474	3.55767229	0.0592708	0.13852171	Gpc4
ENSMUSG00000031125.3	2.70450167	0.02405868	0.01658952	1.59602208	0.20646785	0.36719581	3830403N18Rik
ENSMUSG00000031129.10	104.052176	0.02298722	0.00377981	34.9986645	3.30E-09	4.28E-08	Slc9a9
ENSMUSG00000031132.2	1.3700536	0.02840932	0.01148549	6.26588013	0.0123085	0.0379488	Cd40lg
ENSMUSG00000031133.13	127.368841	0.0274439	0.00493333	28.7974106	8.04E-08	8.48E-07	Arhgef6
ENSMUSG00000031134.17	308.607659	0.00254966	0.00217553	1.39189784	0.23808486	0.40733132	Rbmx
ENSMUSG00000031137.18	11.4743025	0.05646384	0.01417228	13.8891669	0.00019391	0.00103734	Fgf13
ENSMUSG00000031138.5	3613.39471	-0.0011549	0.00452993	0.06367749	0.80077518	0.91618159	F9
ENSMUSG00000031139.17	1.51980308	0.0475302	0.01593413	8.09418232	0.00444076	0.01599079	Mcf2
ENSMUSG00000031142.18	9.01856319	0.05877896	0.01108141	24.7702495	6.46E-07	5.75E-06	Cacna1f
ENSMUSG00000031143.5	231.835472	0.0019145	0.00231986	0.68972206	0.40625893	0.59790485	Ccdc22
ENSMUSG00000031144.16	118.600856	0.03197222	0.00588242	27.3341261	1.71E-07	1.71E-06	Syp
ENSMUSG00000031145.16	75.035603	-0.0008749	0.00337781	0.06659241	0.79636442	0.91397262	Prickle3
ENSMUSG00000031146.7	36.5320722	0.03827832	0.00608687	36.7947767	1.31E-09	1.79E-08	Plp2
ENSMUSG00000031147.9	260.023862	-0.0105457	0.00339057	9.81440876	0.0017315	0.00711236	Magix
ENSMUSG00000031148.15	437.014541	-0.0018828	0.00216832	0.74621988	0.38767569	0.57921113	Gpkow
ENSMUSG00000031149.8	69.0176237	0.00025705	0.00410299	0.00385418	0.95049751	1	Praf2
ENSMUSG00000031150.14	352.986155	0.02923485	0.00995215	7.58237292	0.00589418	0.02035434	Ccdc120
ENSMUSG00000031153.17	426.199814	-0.0001577	0.00285832	0.00305531	0.95591952	1	Gripap1
ENSMUSG00000031154.16	677.59722	-1.31E-05	0.00214928	3.69E-05	0.99515628	1	Otud5
ENSMUSG00000031155.18	69.7606486	0.00784466	0.00396652	3.75853105	0.05253872	0.12552453	Pim2
ENSMUSG00000031156.19	219.779242	-0.0036726	0.00272436	1.81063253	0.17843237	0.32827659	Slc35a2
ENSMUSG00000031157.11	451.258098	0.00038201	0.00240795	0.02506783	0.87419815	0.96098237	Pqbp1
ENSMUSG00000031158.13	743.576219	-0.0096819	0.00328014	8.73250681	0.00312586	0.01182654	Timm17b
ENSMUSG00000031161.16	304.201142	-0.0016537	0.00275732	0.36152393	0.5476611	0.73050617	Hdac6
ENSMUSG00000031163.3	3.00561545	0.07700698	0.01837023	17.1928363	3.38E-05	0.00021469	Glod5
ENSMUSG00000031165.7	41.3270679	0.05423053	0.0057898	81.046433	2.20E-19	1.07E-17	Was
ENSMUSG00000031166.14	533.004265	-0.0026728	0.00236874	1.27492848	0.25884413	0.43243361	Wdr13
ENSMUSG00000031167.17	1324.1418	0.03871261	0.00423981	80.9291643	2.34E-19	1.13E-17	Rbm3
ENSMUSG00000031168.15	3801.76297	-0.0085905	0.00371625	5.29950246	0.02133152	0.06003164	Ebp
ENSMUSG00000031169.14	57.8926531	-0.0065594	0.00338936	3.84189334	0.04998704	0.12055158	Porcn
ENSMUSG00000031170.15	2.51041421	-0.0603524	0.01675339	14.2820641	0.00015736	0.00086012	Slc38a5
ENSMUSG00000031171.12	308.433058	-0.0087744	0.00215608	16.552278	4.73E-05	0.00029252	Ftsj1
ENSMUSG00000031173.14	8535.74181	-0.0121736	0.00497365	5.97541956	0.01450663	0.04369775	Otc

ENSMUSG00000031174.18	48.1945334	0.00094115	0.00346891	0.07197812	0.78847811	0.90928207	Rpgr
ENSMUSG00000031176.9	1624.53425	0.00178419	0.00256605	0.47046047	0.49277541	0.68036064	Dynlt3
ENSMUSG00000031189.13	2.96738157	0.00776373	0.01312204	0.40478058	0.52463064	0.71012558	Aff2
ENSMUSG00000031196.14	395.948946	0.00364315	0.00447726	0.64533487	0.42178554	0.61344476	F8
ENSMUSG00000031197.12	361.017648	0.00391572	0.00319556	1.46237256	0.226553	0.39277822	Vbp1
ENSMUSG00000031198.5	467.457635	0.01095401	0.00586706	3.29902847	0.06932087	0.15695518	Fundc2
ENSMUSG00000031200.17	62.1546294	0.00024647	0.00337646	0.0053194	0.94185846	0.99805345	Mtcp1
ENSMUSG00000031201.18	534.185891	-0.005557	0.00263369	4.39232684	0.03610101	0.09256207	Brcc3
ENSMUSG00000031202.6	3.4480682	0.06453792	0.01519239	15.2858141	9.24E-05	0.00053263	Rab39b
ENSMUSG00000031204.4	0.67813519	-0.0084283	0.07845842	0.01252092	0.91090513	0.98139084	Asb12
ENSMUSG00000031207.17	986.637043	0.03978705	0.00456974	72.107782	2.04E-17	7.79E-16	Msn
ENSMUSG00000031209.15	31.9346688	0.00925716	0.00419846	4.88658345	0.0270662	0.07309175	Heph
ENSMUSG00000031212.4	0.78084842	0.00226684	0.03932821	0.01452095	0.90408478	0.97774971	Pgr15l
ENSMUSG00000031214.14	61.2119742	0.00711265	0.00646199	1.19110301	0.27510718	0.45327824	Ophn1
ENSMUSG00000031216.14	212.171603	-0.0004875	0.00321477	0.02240411	0.88101706	0.96443277	Stard8
ENSMUSG00000031217.9	252.232891	-0.0029176	0.0024758	1.36327944	0.24296993	0.4132013	Efnb1
ENSMUSG00000031220.15	21.0347554	-0.013647	0.00435107	10.0838684	0.00149573	0.00625723	Awat2
ENSMUSG00000031221.8	854.844671	-0.002771	0.00189112	2.1478373	0.14277088	0.27694661	Igbp1
ENSMUSG00000031226.14	180.280357	-0.0047151	0.00282717	2.76562707	0.09630894	0.20416526	Pbdc1
ENSMUSG00000031227.9	54.95693	0.00172756	0.00382877	0.20525881	0.65050916	0.81351585	Magee1
ENSMUSG00000031229.17	2079.05262	0.00650826	0.00393958	2.67099011	0.10219243	0.21366108	Atrx
ENSMUSG00000031230.4	11.756467	0.02667541	0.04048763	-0.3577047	1	1	Fgf16
ENSMUSG00000031231.5	9843.16367	-0.0166239	0.00436822	14.6583612	0.00012886	0.00071767	Cox7b
ENSMUSG00000031232.18	2314.75072	-0.0079842	0.00410518	3.69393479	0.05461064	0.12976492	Magt1
ENSMUSG00000031233.7	2.17496299	-0.0628742	0.04054175	0.22927493	0.63206198	0.79998571	Pgk2
ENSMUSG00000031239.6	26.0572585	0.00370304	0.00571	0.41793438	0.51796955	0.70418064	Itm2a
ENSMUSG00000031241.18	2.25125897	-0.0019274	0.03665491	-0.0055162	1	1	Tbx22
ENSMUSG00000031242.8	416.532823	0.00642304	0.00238121	7.14017067	0.0075376	0.02511752	Chmp1b2
ENSMUSG00000031245.9	2452.43805	-0.007837	0.00429665	3.33253858	0.06792196	0.15452951	Hmgn5
ENSMUSG00000031246.15	2469.51354	0.00787391	0.00272789	8.0328476	0.00459366	0.01643099	Sh3bgrl
ENSMUSG00000031253.16	42.6982102	0.01902803	0.00377382	25.1047733	5.43E-07	4.91E-06	Srpx2
ENSMUSG00000031255.15	45.6967545	-0.0040284	0.00415062	0.9307741	0.33466186	0.52159747	Sytl4
ENSMUSG00000031256.12	320.807363	0.00596414	0.00292665	4.06579641	0.04376005	0.10824588	Cstf2
ENSMUSG00000031257.15	1.48598977	-0.0023917	0.01748624	0.02643377	0.87084547	0.95915167	Nox1
ENSMUSG00000031258.5	4.18114948	0.03748149	0.00892485	16.9191298	3.90E-05	0.0002454	Xkrx
ENSMUSG00000031262.13	9.61926223	0.02994667	0.01143384	6.05424804	0.01387288	0.04203029	Cenpi
ENSMUSG00000031264.14	38.953562	0.05598108	0.00624802	74.9064735	4.94E-18	2.00E-16	Btk
ENSMUSG00000031266.8	107.160279	0.01647267	0.00319284	25.9363017	3.53E-07	3.31E-06	Gla
ENSMUSG00000031271.15	2543.97308	0.05842948	0.00602643	82.8381666	8.91E-20	4.53E-18	Serpina7
ENSMUSG00000031273.17	7.47896922	0.04505518	0.01035117	17.8933145	2.34E-05	0.00015384	Col4a6
ENSMUSG00000031274.17	49.2855868	-0.0118356	0.00711753	2.94386078	0.08620446	0.18703141	Col4a5
ENSMUSG00000031278.13	1490.93492	0.00283547	0.00338634	0.6661548	0.41439544	0.60583468	Acsl4
ENSMUSG00000031283.18	13.7313242	0.02354805	0.00701344	10.5451799	0.00116491	0.00502022	Chrdl1
ENSMUSG00000031284.17	41.7181482	0.03340353	0.01402475	5.10704422	0.02382888	0.06580137	Pak3
ENSMUSG00000031285.15	6.54495473	-0.0001433	0.01193177	-0.0003033	1	1	Dcx
ENSMUSG00000031286.7	101.424683	-0.0127573	0.00320184	16.0232702	6.26E-05	0.00037589	Glt28d2
ENSMUSG00000031289.11	4.50331586	-0.0672191	0.01051605	39.5832115	3.14E-10	4.66E-09	Il13ra2
ENSMUSG00000031290.15	6.9662627	-0.0338779	0.01448865	5.28109415	0.0215582	0.06053437	Lrch2
ENSMUSG00000031292.15	131.643457	-0.0040804	0.00419319	0.94908591	0.32995208	0.51676405	Cdkl5
ENSMUSG00000031295.14	453.681242	0.01281651	0.00375146	11.5600899	0.00067383	0.00311137	Phka2
ENSMUSG00000031297.15	1.56000744	0.05408598	0.01468961	14.2094969	0.00016354	0.00089104	Slc7a3
ENSMUSG00000031298.16	39.5248233	0.04455824	0.00610835	47.260319	6.22E-12	1.18E-10	Adgrg2
ENSMUSG00000031299.11	2656.24724	-0.0120031	0.00310073	14.6461987	0.0001297	0.00072146	Pdha1
ENSMUSG00000031302.17	11.1085872	0.03990006	0.01281114	9.11770406	0.00253147	0.00988063	Nlgn3
ENSMUSG00000031303.9	51.9168732	0.03252394	0.00528301	35.0700482	3.18E-09	4.14E-08	Map3k15
ENSMUSG00000031304.19	112.615225	0.06614561	0.00585503	116.336187	4.01E-27	5.03E-25	Il2rg
ENSMUSG00000031309.16	278.042532	0.01018803	0.0041615	5.66656879	0.01729125	0.05047417	Rps6ka3
ENSMUSG00000031310.17	256.497359	0.01125445	0.00355396	10.04346	0.0015289	0.00638014	Zmym3
ENSMUSG00000031311.18	1784.7184	0.00386297	0.00181253	4.52101848	0.03348089	0.08705037	Nono
ENSMUSG00000031312.6	39.4323216	-0.0130011	0.01409609	0.7887719	0.37447173	0.56541132	Itgb1bp2
ENSMUSG00000031314.19	457.57977	0.00446476	0.00277679	2.57631282	0.10847407	0.2237011	Taf1

ENSMUSG00000031320.10	5957.97694	0.00070457	0.00323303	0.04750971	0.82745465	0.93249935	Rps4x
ENSMUSG00000031326.3	1.78661068	-0.0181131	0.0190548	1.10639215	0.29286756	0.47494323	Cdx4
ENSMUSG00000031327.11	91.646766	-0.0418696	0.00619144	48.3814284	3.51E-12	6.91E-11	Chic1
ENSMUSG00000031328.17	827.8418	0.02836672	0.00542223	25.9799724	3.45E-07	3.25E-06	Flna
ENSMUSG00000031333.8	952.340398	0.00056909	0.00239072	0.05603459	0.81287692	0.92326471	Abcb7
ENSMUSG00000031337.17	277.085409	0.00191191	0.00286638	0.43835627	0.50791688	0.6947186	Mtm1
ENSMUSG00000031340.9	7.59947216	0.03430145	0.01217727	7.51836623	0.0061073	0.02097727	Gabre
ENSMUSG00000031342.18	188.721666	0.02647781	0.0064135	16.2347076	5.60E-05	0.00033982	Gpm6b
ENSMUSG00000031343.14	67.5752563	0.05191956	0.00424446	140.824873	1.76E-32	5.18E-30	Gabra3
ENSMUSG00000031344.12	3.4313085	0.03190152	0.00878932	13.0870232	0.00029735	0.00151311	Gabrq
ENSMUSG00000031347.13	371.454927	0.0035711	0.00311194	1.30596566	0.2531264	0.42535189	Cetn2
ENSMUSG00000031349.5	1468.23302	0.00125035	0.00433807	0.08133962	0.77549014	0.90048791	Nsdhl
ENSMUSG00000031351.16	7.66274907	0.0094446	0.00691461	1.84578119	0.17427487	0.32246387	Zfp185
ENSMUSG00000031352.11	252.315709	-0.0086736	0.00284206	9.22016803	0.00239364	0.00941748	Hccs
ENSMUSG00000031353.14	1129.57741	-0.0014308	0.00197218	0.52239506	0.4698218	0.66085502	Rbbp7
ENSMUSG00000031355.17	117.310066	-4.32E-05	0.00374547	0.00013161	0.99084687	1	Arhgap6
ENSMUSG00000031357.4	894.148716	0.00255718	0.00212521	1.43802443	0.2304593	0.39792189	Syap1
ENSMUSG00000031358.18	551.201638	-0.002012	0.0025014	0.63246434	0.42645281	0.61802249	Msl3
ENSMUSG00000031360.15	230.734585	0.01955153	0.00443121	19.4731106	1.02E-05	7.23E-05	Ctps2
ENSMUSG00000031362.18	3.64814488	0.07485506	0.01375432	22.3244555	2.30E-06	1.86E-05	Xlr4c
ENSMUSG00000031365.14	224.108241	0.01073651	0.0027729	14.6532363	0.00012921	0.00071928	Zfp275
ENSMUSG00000031367.16	94.6116429	0.03889684	0.00491055	58.6403246	1.89E-14	5.01E-13	Ap1s2
ENSMUSG00000031370.13	448.966364	0.00432296	0.00471511	0.81099134	0.36782732	0.55816607	Zrsr2
ENSMUSG00000031371.15	220.45101	0.00939746	0.00361321	6.85231831	0.00885263	0.02879181	Haus7
ENSMUSG00000031373.5	71.124812	0.00971195	0.00421916	5.20120262	0.02257127	0.06294955	Car5b
ENSMUSG00000031375.19	1929.32632	0.02070106	0.00443377	21.2649482	4.00E-06	3.09E-05	Bgn
ENSMUSG00000031376.16	2.518606	0.00182456	0.02281091	0.00608244	0.93783601	0.99588386	Atp2b3
ENSMUSG00000031377.12	7.55537681	0.00465649	0.00919728	0.2369152	0.62644305	0.79518249	Bmx
ENSMUSG00000031378.15	450.238913	0.01003909	0.00280646	12.8944819	0.00032955	0.0016545	Abcd1
ENSMUSG00000031379.14	313.245225	0.00025525	0.00338192	0.00564387	0.94011471	0.99699614	Pir
ENSMUSG00000031380.11	18.1395323	-0.0046441	0.00595593	0.61384839	0.43334249	0.62498948	Vegfd
ENSMUSG00000031381.17	131.106756	0.00583146	0.00377458	2.30956094	0.12858037	0.2557511	Piga
ENSMUSG00000031382.15	27.1950209	0.06028985	0.02411165	1.82726754	0.17645063	0.32544296	Asb11
ENSMUSG00000031383.9	1.82767792	0.06573143	0.0134009	24.6668137	6.81E-07	6.04E-06	Dusp9
ENSMUSG00000031385.15	6.24669158	0.04218475	0.00926105	20.0264922	7.64E-06	5.54E-05	Plxnb3
ENSMUSG00000031386.15	738.701391	0.00311974	0.00252398	1.5327113	0.21570598	0.37908064	Hcfc1
ENSMUSG00000031387.15	95.3035267	0.0483667	0.00573146	69.1598241	9.08E-17	3.18E-15	Renbp
ENSMUSG00000031388.15	493.505325	-0.0049028	0.00316234	2.435659	0.11860404	0.24005792	Naa10
ENSMUSG00000031389.18	139.38897	0.0375671	0.00457321	64.8141479	8.23E-16	2.56E-14	Arhgap4
ENSMUSG00000031390.9	3.45690378	0.0138172	0.01858701	0.88505073	0.34682205	0.53520801	Avpr2
ENSMUSG00000031391.19	10.056107	0.03986285	0.01025867	13.1033026	0.00029478	0.00150164	L1cam
ENSMUSG00000031392.19	1232.69123	3.67E-05	0.00243477	0.00022153	0.98812495	1	Irak1
ENSMUSG00000031393.18	353.046241	0.00468596	0.00194175	5.87024599	0.0153989	0.04586418	Mecp2
ENSMUSG00000031398.14	18.8958567	0.04003298	0.00863319	20.055058	7.52E-06	5.47E-05	Plxna3
ENSMUSG00000031399.16	246.404456	0.00110945	0.00211551	0.27868889	0.59756186	0.7721945	Fam3a
ENSMUSG00000031400.11	202.058001	0.00726779	0.00587391	1.53701279	0.21506299	0.37809152	G6pdx
ENSMUSG00000031402.13	611.49502	0.01065775	0.00297155	12.8023231	0.00034619	0.00173065	Mpp1
ENSMUSG00000031403.15	387.956446	-0.001889	0.00375743	0.25041243	0.61678482	0.78813993	Dkc1
ENSMUSG00000031409.2	3.98588559	0.01089583	0.03697273	0.10005883	0.75175904	0.88473573	Tceal6
ENSMUSG00000031410.15	1.55249498	-0.0126356	0.01547813	0.84909522	0.35680842	0.54558726	Nxf7
ENSMUSG00000031422.17	1777.06958	-0.0005187	0.00261357	0.03793749	0.84556872	0.94426255	Morf4l2
ENSMUSG00000031425.16	91.1343258	0.01136901	0.00678487	2.74288961	0.09768798	0.20642077	Plp1
ENSMUSG00000031428.12	13.9242643	0.09040235	0.00926781	86.2340224	1.60E-20	8.81E-19	Zcchc18
ENSMUSG00000031429.15	285.159421	-0.0005493	0.00283772	0.03736184	0.8467302	0.94501388	Psmd10
ENSMUSG00000031431.14	1567.0742	-0.0227603	0.00454908	26.742809	2.32E-07	2.26E-06	Tsc22d3
ENSMUSG00000031432.4	525.941606	0.00626132	0.002101	8.8372225	0.00295148	0.01127163	Prps1
ENSMUSG00000031433.16	62.0904922	0.00864079	0.00530614	2.55805609	0.10973344	0.22584405	Rbm41
ENSMUSG00000031434.16	61.5287421	0.00927494	0.00506353	3.48536113	0.06191385	0.14347854	Morc4
ENSMUSG00000031438.12	1917.13	-0.002389	0.00263281	0.81480284	0.36670409	0.55671232	Rnf128
ENSMUSG00000031441.16	730.818499	0.04756199	0.00653961	45.899851	1.24E-11	2.27E-10	Atp11a
ENSMUSG00000031442.22	35.9877822	0.03458221	0.00814292	17.4329338	2.98E-05	0.00019143	Mcf2l

ENSMUSG00000031443.8	1548.07277	-0.0067713	0.00439303	2.38592007	0.12243279	0.24607645	F7
ENSMUSG00000031444.17	7858.38209	-0.0017594	0.00496114	0.12697343	0.72159096	0.86435479	F10
ENSMUSG00000031445.6	7340.58064	-0.0028301	0.00494787	0.32683934	0.56752615	0.74735536	Proz
ENSMUSG00000031446.15	1979.05425	-0.0038255	0.00168469	5.17041464	0.02297473	0.06386353	Cul4a
ENSMUSG00000031447.8	11286.576	0.00155971	0.00189982	0.66544412	0.41464452	0.6059759	Lamp1
ENSMUSG00000031448.13	113.245632	0.00433335	0.02052796	0.04829521	0.82605674	0.93133299	Adprhl1
ENSMUSG00000031450.9	47.5968873	0.01527223	0.01992951	0.19087335	0.66219073	0.82258548	Grk1
ENSMUSG00000031451.7	1085.73599	0.03849896	0.00400671	87.0694175	1.05E-20	5.83E-19	Gas6
ENSMUSG00000031452.16	1.16579931	-0.0243001	0.06390635	0.28869291	0.59105937	0.76703516	1700029H14Rik
ENSMUSG00000031453.17	253.798656	0.03666961	0.00378993	89.351425	3.31E-21	1.96E-19	Rasa3
ENSMUSG00000031458.9	53.4462291	-0.0042518	0.00496881	0.7462647	0.38766143	0.57921113	Coprs
ENSMUSG00000031461.5	248.363884	0.03382767	0.02263424	-2.9411343	1	1	Myom2
ENSMUSG00000031465.7	34.1582176	0.03886105	0.006585	32.6992701	1.08E-08	1.29E-07	Angpt2
ENSMUSG00000031467.11	230.149417	-0.0005998	0.0026663	0.05010218	0.82288557	0.92952866	Agpat5
ENSMUSG00000031478.17	27.8729685	0.01332229	0.00393486	11.0604278	0.0008819	0.00392832	Nek3
ENSMUSG00000031479.10	548.910479	-0.0034992	0.00250196	1.94724886	0.16288362	0.30567895	Vps36
ENSMUSG00000031480.16	67.8617544	0.00258218	0.00328795	0.63509016	0.42549433	0.61708845	Thsd1
ENSMUSG00000031481.9	1.22429681	-0.0109111	0.01798152	0.30410666	0.58131929	0.75911786	Tpte
ENSMUSG00000031482.10	5937.71791	-0.0083561	0.00353715	5.5787476	0.01817972	0.05263772	Slc25a15
ENSMUSG00000031483.9	721.063565	0.00346399	0.00271509	1.58702626	0.20775151	0.36883908	Erlin2
ENSMUSG00000031485.16	1385.35428	-0.0002841	0.00331877	0.00712962	0.93270892	0.99322193	Plpbp
ENSMUSG00000031486.16	99.3173835	0.00579363	0.00349014	2.74132129	0.09778388	0.20654941	Adgra2
ENSMUSG00000031487.6	103.142252	-0.0013099	0.00322187	0.16442192	0.68511756	0.83935108	Brf2
ENSMUSG00000031488.15	192.118842	0.00676299	0.00311253	4.63427965	0.0313393	0.0824413	Rab11fip1
ENSMUSG00000031489.16	107.100169	-0.0175758	0.0065417	7.13954225	0.00754024	0.02512277	Adrb3
ENSMUSG00000031490.7	598.267916	-0.0108045	0.00362808	8.98804958	0.00271751	0.01050052	Eif4ebp1
ENSMUSG00000031492.14	1.04389314	0.01999616	0.02843309	1.13071446	0.28762299	0.46861558	Chrnb3
ENSMUSG00000031493.11	3.50892314	-0.000448	0.00996917	-0.0005444	1	1	Ggn
ENSMUSG00000031494.8	7.01565777	0.01177423	0.00710788	2.65481988	0.10323645	0.21521433	Cd209a
ENSMUSG00000031497.11	15.7172622	0.05803084	0.0071966	56.4393144	5.80E-14	1.43E-12	Tnfsf13b
ENSMUSG00000031502.12	818.772185	-0.0040948	0.00516709	0.62200984	0.4303014	0.62177714	Col4a1
ENSMUSG00000031503.14	485.224378	-0.0122	0.00489381	6.39241479	0.0114609	0.03575146	Col4a2
ENSMUSG00000031504.7	161.894132	0.00832932	0.00291347	8.06255707	0.00451894	0.01621784	Rab20
ENSMUSG00000031505.12	904.925332	-0.0103213	0.00228296	20.6244729	5.59E-06	4.19E-05	Naxd
ENSMUSG00000031506.12	21.5193003	0.08334583	0.00734842	113.436597	1.73E-26	2.01E-24	Ptpn7
ENSMUSG00000031508.15	183.417506	0.00226852	0.00298612	0.57964636	0.44645097	0.63840983	Ankrd10
ENSMUSG00000031511.17	462.702731	0.00122899	0.00369893	0.10872677	0.74159838	0.87768274	Arhgef7
ENSMUSG00000031513.9	338.013318	0.00340629	0.00238028	2.04396779	0.1528106	0.29145972	Leprotl1
ENSMUSG00000031516.12	251.017245	0.00304348	0.00308233	0.9704773	0.32456117	0.5112944	Dctn6
ENSMUSG00000031517.9	265.460632	0.02335381	0.00552436	16.9481043	3.84E-05	0.00024207	Gpm6a
ENSMUSG00000031518.7	2.69932115	-0.0940266	0.08223775	0.12518543	0.72347713	0.86579388	Spata4
ENSMUSG00000031519.5	9.73895981	0.03300115	0.01317711	5.85314044	0.01554928	0.04622451	Asb5
ENSMUSG00000031520.7	52.5798503	0.00473202	0.00316776	2.22238981	0.13602236	0.26690093	Vegfc
ENSMUSG00000031521.6	286.221452	0.00306186	0.00232558	1.726943	0.18880178	0.34278183	Aga
ENSMUSG00000031523.17	759.327625	-0.0072914	0.00307638	5.48197898	0.01921349	0.05504306	Dlc1
ENSMUSG00000031527.8	269.09411	0.0039964	0.00284208	1.91031755	0.16692799	0.31168173	Eri1
ENSMUSG00000031529.6	548.02998	-0.0045635	0.00294479	2.37967723	0.12292294	0.24678758	Tnks
ENSMUSG00000031530.7	30.7435965	0.00450076	0.0055931	0.63954856	0.42387432	0.61550157	Dusp4
ENSMUSG00000031532.8	2052.62474	-0.0044779	0.00211002	4.45151069	0.03487039	0.09001346	Saraf
ENSMUSG00000031533.5	506.487189	-0.0142407	0.00233808	37.085603	1.13E-09	1.55E-08	Mrps31
ENSMUSG00000031534.16	301.54262	0.00436862	0.00291688	2.27135786	0.13178439	0.26060263	Smim19
ENSMUSG00000031535.8	3.80963704	-0.0238157	0.01325635	2.42660566	0.11929095	0.24111719	Dkk4
ENSMUSG00000031536.7	335.264794	-0.0067251	0.00262654	6.39531077	0.01144222	0.03570736	Polb
ENSMUSG00000031537.16	1033.74403	0.00433841	0.00195671	4.86103353	0.02746988	0.07393638	Ikbkb
ENSMUSG00000031538.7	29.6728774	0.05863408	0.0083766	46.3813904	9.73E-12	1.81E-10	Plat
ENSMUSG00000031539.7	60.8280123	0.03084685	0.00440821	48.1723597	3.90E-12	7.60E-11	Ap3m2
ENSMUSG00000031540.15	530.485911	0.00249306	0.00301643	0.67639608	0.41083049	0.60246684	Kat6a
ENSMUSG00000031543.19	317.558299	0.02266946	0.01272236	2.69803011	0.10047232	0.21096235	Ank1
ENSMUSG00000031545.7	3009.49232	-0.0081524	0.00271519	8.89454173	0.00286024	0.0109676	Gpat4
ENSMUSG00000031546.7	287.437003	-0.0034544	0.00249959	1.90804331	0.16718077	0.3120796	Gins4
ENSMUSG00000031548.8	39.7818727	0.01225408	0.00614337	3.89921897	0.04830856	0.11730729	Sfrp1

ENSMUSG00000031549.17	1363.86514	-0.0155158	0.00495432	9.63936586	0.00190451	0.00774649	Ido2
ENSMUSG00000031553.17	1.88144167	0.05705038	0.03697184	-1.0248504	1	1	Adam3
ENSMUSG00000031554.19	5.17309291	-0.0489114	0.02470348	3.97425238	0.04620096	0.11315637	Adam5
ENSMUSG00000031555.17	1452.64644	-3.92E-05	0.002162	0.0003192	0.98574561	1	Adam9
ENSMUSG00000031556.7	889.488346	-0.0089042	0.00225779	15.5517587	8.03E-05	0.00046913	Tm2d2
ENSMUSG00000031557.17	146.992568	0.04274513	0.00473455	75.4795484	3.69E-18	1.52E-16	Plekha2
ENSMUSG00000031558.16	17.3420581	-0.0164241	0.01255129	1.50565725	0.21980295	0.38447585	Slit2
ENSMUSG00000031561.17	372.120723	0.01142984	0.00495516	5.16262426	0.023078	0.06407507	Tenm3
ENSMUSG00000031562.17	4.24169852	0.02508543	0.01238145	3.19794164	0.07373101	0.1651177	Dctd
ENSMUSG00000031563.9	387.426427	0.01018101	0.0026391	14.8837972	0.00011434	0.00064502	Wwc2
ENSMUSG00000031565.20	175.36401	0.01889396	0.00595107	9.30539718	0.0022848	0.00906006	Fgfr1
ENSMUSG00000031568.17	380.950158	-0.0023979	0.00240749	0.99200526	0.31925277	0.50517585	Rwdd4a
ENSMUSG00000031570.18	288.720072	0.01441034	0.00315099	20.5445991	5.83E-06	4.35E-05	Plpp5
ENSMUSG00000031574.9	27.7788776	-0.0036521	0.00542753	0.45131816	0.50170964	0.6884242	Star
ENSMUSG00000031575.19	350.378263	0.00025547	0.00157751	0.02652938	0.87061416	0.95900923	Ash2l
ENSMUSG00000031577.11	182.030251	-0.0037099	0.00192719	3.66729769	0.05549008	0.13149695	Tti2
ENSMUSG00000031578.6	324.580397	-0.0049061	0.00290032	2.84291106	0.09177781	0.19650248	Mak16
ENSMUSG00000031583.14	232.968768	0.0085013	0.00305372	7.64450503	0.00569455	0.01978372	Wrn
ENSMUSG00000031584.17	1338.93569	0.00453329	0.00242	3.46465286	0.06269367	0.1449841	Gsr
ENSMUSG00000031585.14	229.749196	-0.0048629	0.0030913	2.51771005	0.11257395	0.23028355	Gtf2e2
ENSMUSG00000031586.18	583.117662	-0.0097472	0.00328596	8.69254523	0.00319514	0.01204806	Rbpms
ENSMUSG00000031590.9	340.024121	-0.0003238	0.00334163	0.00936948	0.92288833	0.98807551	Frg1
ENSMUSG00000031591.15	913.163878	0.02206383	0.00301882	52.0203584	5.49E-13	1.20E-11	Asah1
ENSMUSG00000031592.11	437.420594	0.00257807	0.00335791	0.5728834	0.4491154	0.64075614	Pcm1
ENSMUSG00000031594.10	7610.99296	0.01882277	0.00733408	6.28409446	0.01218263	0.03760496	Fgl1
ENSMUSG00000031595.10	15.6262281	-0.015349	0.00823358	3.36438912	0.06662029	0.15215514	Pdgfrl
ENSMUSG00000031596.16	8832.23272	0.00964877	0.00539035	3.19843715	0.07370868	0.16509903	Slc7a2
ENSMUSG00000031600.12	1096.92968	-0.0134929	0.00269608	24.8938265	6.06E-07	5.43E-06	Vps37a
ENSMUSG00000031601.17	583.428102	-0.000948	0.00239067	0.15659119	0.69231452	0.84442196	Cnot7
ENSMUSG00000031604.11	3585.37544	0.01193895	0.00486853	5.67627339	0.01719585	0.05027038	Msmo1
ENSMUSG00000031605.9	625.473626	-0.0084677	0.00289323	8.47131831	0.0036079	0.01337609	Klhl2
ENSMUSG00000031608.14	74.0014595	0.00945017	0.0039485	5.44055047	0.01967447	0.05614543	Galnt7
ENSMUSG00000031609.4	76.8673171	0.008536	0.00393806	4.62225918	0.03155965	0.08291921	Sap30
ENSMUSG00000031613.10	2056.69186	0.00273764	0.00384886	0.51631699	0.47241699	0.66308183	Hpgd
ENSMUSG00000031616.8	109.095598	0.02940579	0.00399741	49.6988536	1.79E-12	3.63E-11	Ednra
ENSMUSG00000031617.17	323.758453	0.00439213	0.00276065	2.48738109	0.11476255	0.23368967	Tmem184c
ENSMUSG00000031618.14	452.111344	0.00132533	0.00481539	0.07777065	0.7803416	0.90363005	Nr3c2
ENSMUSG00000031622.17	620.06173	-0.0024912	0.00247777	1.02265712	0.31188956	0.49712883	Sin3b
ENSMUSG00000031626.19	1488.62999	-0.0037421	0.00323471	1.31603868	0.25130422	0.42307362	Sorbs2
ENSMUSG00000031627.10	1318.61652	-0.0017168	0.00458286	0.13719722	0.71108341	0.85726913	Irf2
ENSMUSG00000031628.10	548.084016	0.00601194	0.002698	4.91957716	0.02655402	0.07196404	Casp3
ENSMUSG00000031629.15	11.771311	0.01611224	0.01099175	1.84438172	0.17443825	0.32266493	Cenpu
ENSMUSG00000031631.16	282.569886	-0.0008411	0.00274431	0.09202745	0.76161533	0.89131623	Cfap97
ENSMUSG00000031633.5	2247.04167	0.01919335	0.00651065	8.04749042	0.00455667	0.01632347	Slc25a4
ENSMUSG00000031634.14	659.013363	-0.0077295	0.00176935	19.1051206	1.24E-05	8.61E-05	Ufsp2
ENSMUSG00000031636.8	8.77514214	-0.0009595	0.00807387	0.01340524	0.90782612	0.97995363	Pdlim3
ENSMUSG00000031637.14	37.3137335	-0.0180645	0.00560076	9.8162382	0.00172978	0.00710652	Lrp2bp
ENSMUSG00000031639.13	147.334554	0.01069404	0.00368465	8.17744968	0.00424144	0.01537871	Tlr3
ENSMUSG00000031640.12	666.451242	-0.0228531	0.0133882	2.79219032	0.09472467	0.20155153	Gm45753
ENSMUSG00000031641.15	561.697683	-0.0071062	0.00317452	5.05239964	0.02459186	0.06751526	Cbr4
ENSMUSG00000031642.10	95.2017508	0.00696182	0.00316988	4.79130975	0.02860367	0.07645582	Sh3rf1
ENSMUSG00000031644.20	416.183159	-0.0110668	0.00393372	7.82809213	0.00514404	0.01812207	Nek1
ENSMUSG00000031645.5	1079.59444	-0.0278287	0.00515932	29.7388358	4.94E-08	5.37E-07	F11
ENSMUSG00000031647.11	28.7746318	0.01871743	0.00786802	5.31176342	0.02118191	0.05967709	Mfap3l
ENSMUSG00000031652.12	341.648469	0.00356973	0.00261077	1.84281273	0.17462164	0.32287754	N4bp1
ENSMUSG00000031654.17	2.37838327	-0.0184231	0.04116137	0.35130264	0.55337666	0.73573517	Cbln1
ENSMUSG00000031657.17	474.711515	-0.0034433	0.00336958	1.04562964	0.30651577	0.49160078	Heatr3
ENSMUSG00000031659.14	117.600326	0.05761883	0.00559147	101.425149	7.42E-24	6.08E-22	Adcy7
ENSMUSG00000031660.15	908.755763	-0.0049931	0.00337174	2.17380991	0.14037807	0.27342945	Brd7
ENSMUSG00000031661.13	104.474952	0.00708095	0.00328926	4.61399569	0.03171206	0.08327329	Nkd1
ENSMUSG00000031662.9	27.6359853	0.06240788	0.00657864	84.8563321	3.21E-20	1.71E-18	Snx20

ENSMUSG00000031665.8	468.476428	0.01064738	0.0041356	6.68800095	0.0097064	0.03113069	Sall1
ENSMUSG00000031666.16	777.995215	-0.0019587	0.00255235	0.58238141	0.44538039	0.63769086	Rbl2
ENSMUSG00000031667.17	296.711284	0.00161583	0.00343962	0.21598374	0.64211729	0.8081677	Aktip
ENSMUSG00000031668.15	353.745549	0.00863428	0.00371589	5.43137496	0.0197781	0.05635934	Eif2ak3
ENSMUSG00000031669.11	17.0946827	0.00779646	0.00540198	2.0246558	0.15476399	0.29418628	Gins3
ENSMUSG00000031671.12	257.524386	-0.0075583	0.00249368	9.39706526	0.00217333	0.00868375	Setd6
ENSMUSG00000031672.9	8634.51209	-0.0067848	0.00207672	10.6473158	0.00110231	0.00478275	Got2
ENSMUSG00000031673.7	20.0560284	0.02269109	0.00971446	5.29457854	0.02139191	0.06017055	Cdh11
ENSMUSG00000031681.17	371.99523	0.00709936	0.00298963	5.47582433	0.01928127	0.05521038	Smad1
ENSMUSG00000031683.17	680.236575	-0.008374	0.00353524	5.59826182	0.01797831	0.05213134	Lsm6
ENSMUSG00000031684.12	177.752566	-0.0116395	0.00528008	4.86780749	0.02736224	0.07368873	Slc10a7
ENSMUSG00000031691.15	639.747221	0.00186385	0.00293721	0.40454048	0.52475363	0.71016979	Tnpo2
ENSMUSG00000031696.10	2020.3797	-0.0007395	0.00219931	0.11164543	0.73827856	0.87564113	Vps35
ENSMUSG00000031697.13	97.4720027	0.00523166	0.00395297	1.75367787	0.18541504	0.33809039	Orc6
ENSMUSG00000031698.15	190.873385	-0.0004884	0.00876686	0.00318237	0.95501317	1	Mylk3
ENSMUSG00000031700.12	7590.7079	0.00713593	0.00319437	4.93714865	0.02628536	0.0713836	Gpt2
ENSMUSG00000031701.7	2140.10876	-0.0082346	0.00211564	14.9505106	0.00011037	0.00062411	Dnaja2
ENSMUSG00000031703.9	1254.98176	-0.0032614	0.00163851	3.9135342	0.04789877	0.11648009	Itfg1
ENSMUSG00000031706.8	144.022122	-0.0034724	0.00308121	1.22453549	0.26847239	0.44470748	Rfx1
ENSMUSG00000031708.18	3098.15846	-0.0071276	0.00295054	5.92125619	0.01495929	0.04475264	Tecr
ENSMUSG00000031709.16	63.209991	0.0654736	0.00580111	116.20778	4.28E-27	5.34E-25	Tbc1d9
ENSMUSG00000031710.5	0.81697439	0.01697729	0.03466499	0.52471447	0.46883752	0.65990343	Ucp1
ENSMUSG00000031711.8	293.651858	0.00523642	0.00221986	5.60542049	0.017905	0.0519571	Zfp330
ENSMUSG00000031712.11	53.3399095	0.02793404	0.00641224	18.0712281	2.13E-05	0.00014142	Il15
ENSMUSG00000031714.10	279.794356	-0.0007031	0.0027877	0.0640133	0.80026165	0.9160384	Gab1
ENSMUSG00000031715.15	1021.72079	0.00011508	0.00311288	0.00132329	0.97098171	1	Smarca5
ENSMUSG00000031722.11	55359.2501	0.01100149	0.00653457	2.78809869	0.09496684	0.20188265	Hp
ENSMUSG00000031723.9	215.213649	-0.0075363	0.00269693	7.80099379	0.00522175	0.01835766	Txnl4b
ENSMUSG00000031725.9	7417.76273	-0.0162025	0.00607903	6.88591348	0.00868777	0.0283455	Ces1f
ENSMUSG00000031727.9	6.17856601	-0.0059408	0.03438349	-0.0151114	1	1	Pmfbp1
ENSMUSG00000031728.11	52.8291617	0.00502653	0.00336882	2.19201616	0.13872762	0.27098034	Zfp821
ENSMUSG00000031729.7	974.321129	0.00115803	0.00139059	0.69632471	0.40402141	0.59561529	Ist1
ENSMUSG00000031730.19	143.838928	-0.0114726	0.0035653	10.4363926	0.00123556	0.00529566	Dhodh
ENSMUSG00000031731.17	1412.12402	-0.003501	0.00245313	2.00492539	0.15678901	0.297165	Ap1g1
ENSMUSG00000031732.13	90.0753543	-0.0010776	0.00415491	0.0652313	0.79841094	0.91521751	Phlpp2
ENSMUSG00000031734.14	25.7522494	-0.0068348	0.00866705	0.59952612	0.43875889	0.63074097	Irx3
ENSMUSG00000031737.12	3.27097476	-0.0020719	0.03319967	-0.0448165	1	1	Irx5
ENSMUSG00000031740.9	69.7197692	0.02038345	0.00705108	8.48236648	0.00358605	0.01330704	Mmp2
ENSMUSG00000031748.17	50.6416185	0.03752169	0.00912058	15.5247969	8.14E-05	0.00047528	Gnao1
ENSMUSG00000031749.13	60.2483902	0.01039453	0.00494128	4.35533195	0.03689327	0.0941431	St3gal2
ENSMUSG00000031750.16	41.2817449	0.0160545	0.0058438	7.44673552	0.00635516	0.0217173	Il34
ENSMUSG00000031751.15	5479.2657	-0.0012807	0.0023384	0.29521656	0.58689686	0.76367077	Amfr
ENSMUSG00000031753.17	949.15237	-0.0125198	0.00248115	25.2872047	4.94E-07	4.51E-06	Cog4
ENSMUSG00000031754.11	576.75022	0.00050742	0.00260568	0.037807	0.84583121	0.94441275	Nudt21
ENSMUSG00000031755.7	52.0655777	0.0002049	0.00495294	0.00165585	0.96754135	1	Bbs2
ENSMUSG00000031756.6	12.9114555	0.00403221	0.00678138	0.35239298	0.55276164	0.7349589	Cenpn
ENSMUSG00000031758.11	33.190088	0.0054616	0.00439956	1.58937176	0.20741591	0.36835417	Cdyl2
ENSMUSG00000031760.10	7.56222705	0.02958891	0.01732332	3.85804905	0.04950786	0.11959161	Mt3
ENSMUSG00000031762.8	1173.13597	0.01160791	0.01378154	0.17625067	0.67461535	0.83155036	Mt2
ENSMUSG00000031765.9	2562.85401	0.01443697	0.01186249	1.02619958	0.31105295	0.49624607	Mt1
ENSMUSG00000031766.5	32.1048179	0.00576844	0.01279478	0.19633138	0.65769849	0.81937865	Slc12a3
ENSMUSG00000031767.14	14637.3656	-0.0539829	0.00492155	117.06646	2.78E-27	3.62E-25	Nudt7
ENSMUSG00000031770.17	5784.47974	-0.0107573	0.00402675	7.13115133	0.00757561	0.02522928	Herpud1
ENSMUSG00000031772.18	1.45055115	0.01127187	0.03263314	0.47931436	0.48873305	0.67691923	Cntnap4
ENSMUSG00000031774.9	413.101688	-0.0030401	0.002709	1.25542578	0.26251871	0.437179	Psme3ip1
ENSMUSG00000031775.6	241.112004	-0.0115882	0.00467045	5.989905	0.01438798	0.04339576	Pllp
ENSMUSG00000031776.18	174.321353	0.00575248	0.00504432	1.25343594	0.26289725	0.43760036	Arl2bp
ENSMUSG00000031778.14	52.8841714	0.06200481	0.00859454	48.4066864	3.46E-12	6.83E-11	Cx3cl1
ENSMUSG00000031779.4	6.7337631	0.05812481	0.01099677	24.4731744	7.54E-07	6.61E-06	Ccl22
ENSMUSG00000031781.15	459.361431	-0.0083101	0.00283767	8.79554589	0.00301967	0.01148926	Ciapin1
ENSMUSG00000031782.16	1899.7003	-0.0138851	0.0034363	16.1783064	5.77E-05	0.00034901	Coq9

ENSMUSG00000031783.11	434.592143	-0.0042097	0.00267063	2.53134355	0.11160515	0.22873819	Polr2c
ENSMUSG00000031785.17	83.6388158	0.02063313	0.00613511	11.2595849	0.00079213	0.00358258	Adgrg1
ENSMUSG00000031787.9	53.4619814	0.00519343	0.00504191	1.033536	0.30932961	0.49439914	Katnb1
ENSMUSG00000031788.15	351.583878	0.00063034	0.00342586	0.03321569	0.85538519	0.95053546	Kifc3
ENSMUSG00000031789.18	5.12020712	0.01355375	0.01474278	0.72661063	0.39398403	0.58530427	Cngb1
ENSMUSG00000031790.9	687.030545	-0.0231682	0.00402829	32.7130211	1.07E-08	1.28E-07	Mmp15
ENSMUSG00000031791.9	94.5299705	-0.0071604	0.00561492	1.56873915	0.21039021	0.37229076	Tmem38a
ENSMUSG00000031792.16	159.235988	0.00206953	0.00264085	0.6192209	0.43133695	0.62284274	Usb1
ENSMUSG00000031796.8	476.003222	-0.0055757	0.00265787	4.43607938	0.03518696	0.09066198	Cfap20
ENSMUSG00000031799.11	647.445919	0.01307261	0.0038182	11.2680255	0.00078854	0.00356838	Tpm4
ENSMUSG00000031803.9	49.3458064	0.00293166	0.00361943	0.65204259	0.41938335	0.61074189	B3gnt3
ENSMUSG00000031805.18	221.0955	0.02509163	0.0031925	59.9513164	9.72E-15	2.68E-13	Jak3
ENSMUSG00000031807.11	688.930692	-0.0102778	0.0035314	8.43230821	0.00368612	0.01361768	Pgls
ENSMUSG00000031808.8	150.017725	-0.0143629	0.00529348	7.34037192	0.00674229	0.02288439	Slc27a1
ENSMUSG00000031812.17	2850.45994	-0.0138093	0.00247352	30.8385285	2.80E-08	3.17E-07	Map1lc3b
ENSMUSG00000031813.9	762.588747	-0.0015159	0.00322908	0.22169737	0.63774964	0.80451212	Mvb12a
ENSMUSG00000031816.16	204.438836	-0.0106523	0.00273516	15.3874575	8.76E-05	0.00050666	Mthfsd
ENSMUSG00000031818.13	8914.66963	-0.0156821	0.00420045	13.9940886	0.00018339	0.00098843	Cox4i1
ENSMUSG00000031819.14	654.085565	-0.0030136	0.00360434	0.70927551	0.39968443	0.59147678	Emc8
ENSMUSG00000031820.10	834.852979	-0.0072243	0.00238171	9.36662066	0.00220973	0.00881424	Babam1
ENSMUSG00000031821.12	48.645936	0.02452772	0.00952042	5.80639313	0.01596801	0.04724304	Gins2
ENSMUSG00000031822.21	324.759951	0.00032755	0.00459385	0.0049907	0.94368037	0.99894211	Gse1
ENSMUSG00000031823.15	307.78125	-0.006382	0.00288053	4.8851297	0.027089	0.07313717	Zdhhc7
ENSMUSG00000031824.16	163.358415	0.01718865	0.00514953	10.5064732	0.00118957	0.0051162	6430548M08Rik
ENSMUSG00000031825.17	44.8649755	0.05054896	0.00620642	59.4005959	1.29E-14	3.49E-13	Crispld2
ENSMUSG00000031826.21	1368.95682	-0.0147849	0.00329517	19.8832281	8.23E-06	5.93E-05	Usp10
ENSMUSG00000031827.15	348.310813	0.05904918	0.00539544	111.39523	4.85E-26	5.22E-24	Cotl1
ENSMUSG00000031828.10	80.1439345	0.00771425	0.00254095	9.12727017	0.00251827	0.0098357	Klhl36
ENSMUSG00000031831.7	4.26862033	-0.0261067	0.01904486	2.65386537	0.10329844	0.21532453	Dnaaf1
ENSMUSG00000031832.16	38.5912514	0.00892797	0.00409361	4.68038682	0.03050888	0.08062548	Taf1c
ENSMUSG00000031833.11	492.579088	0.00328936	0.00436358	0.56525241	0.45215167	0.64395292	Mast3
ENSMUSG00000031834.16	371.672356	-0.001717	0.00358269	0.23225854	0.62985419	0.79830947	Pik3r2
ENSMUSG00000031835.17	1104.98286	-0.0054454	0.00216427	6.28767591	0.01215804	0.03754379	Mbtps1
ENSMUSG00000031837.15	3.29953425	0.00574294	0.01101139	0.27252115	0.60164555	0.7752629	Necab2
ENSMUSG00000031838.11	350.622235	0.0503731	0.00440119	124.96725	5.17E-29	8.53E-27	Ifi30
ENSMUSG00000031839.8	1492.54775	0.00180578	0.00270284	0.43842173	0.50788521	0.69471556	Hsbp1
ENSMUSG00000031840.15	87.1239267	0.00291087	0.00372679	0.62752109	0.42826606	0.61960157	Rab3a
ENSMUSG00000031841.20	73.1770985	-0.0114147	0.00561882	3.93400088	0.04731924	0.11541589	Cdh13
ENSMUSG00000031842.16	243.865255	-0.008727	0.00471931	3.40252421	0.06509673	0.14942788	Pde4c
ENSMUSG00000031843.3	68.772966	0.00999992	0.00373598	7.08534867	0.00777167	0.0257556	Mphosph6
ENSMUSG00000031844.9	2299.21251	-0.0349592	0.00475972	54.2656354	1.75E-13	4.06E-12	Hsd17b2
ENSMUSG00000031845.17	164.551569	-0.0081651	0.00504943	2.62548977	0.10516005	0.21825985	Bco1
ENSMUSG00000031847.9	10.7838919	-0.0138171	0.00706677	3.58878142	0.05817088	0.13656002	1700030J22Rik
ENSMUSG00000031848.16	397.603619	-0.0015392	0.00351782	0.19163966	0.66155544	0.82222936	Lsm4
ENSMUSG00000031849.10	5.04609962	-0.0040222	0.01546018	0.05972454	0.80693184	0.91974554	Comp
ENSMUSG00000031851.15	128.973172	0.02143876	0.00421808	24.7948989	6.38E-07	5.69E-06	Ntpcr
ENSMUSG00000031853.6	116.246672	-0.0033815	0.00390823	0.75028238	0.38638684	0.57782275	Map3k21
ENSMUSG00000031858.18	546.598325	-0.0043058	0.00246438	3.05792375	0.08034427	0.1767559	Mau2
ENSMUSG00000031860.18	11.5936181	0.00923474	0.00704361	1.71042968	0.19092963	0.34568713	Pbx4
ENSMUSG00000031861.17	30.1004141	0.02080354	0.00611712	11.1124416	0.00085751	0.00383124	Lpar2
ENSMUSG00000031862.12	784.616361	-0.0026748	0.00212077	1.60486109	0.20521568	0.36544898	Atp13a1
ENSMUSG00000031864.17	132.96314	0.00205248	0.00327021	0.39110378	0.53171978	0.71630927	Ints10
ENSMUSG00000031865.17	557.398234	0.0021782	0.00285674	0.57921807	0.44661898	0.63846022	Dctn1
ENSMUSG00000031870.17	3.89568168	0.03407388	0.01449158	5.93689681	0.0148271	0.04446999	Pgr
ENSMUSG00000031871.10	532.6962	0.01581548	0.00426405	13.183726	0.00028239	0.00144703	Cdh5
ENSMUSG00000031872.15	1.20058432	0.03178466	0.01924427	3.52266859	0.06053491	0.1409872	Bean1
ENSMUSG00000031875.8	159.723702	0.02859218	0.00390532	51.5399643	7.02E-13	1.51E-11	Cmtm3
ENSMUSG00000031877.10	810.734025	0.00153925	0.00305977	0.25809625	0.61143108	0.78367642	Ces2g
ENSMUSG00000031878.19	483.137737	-0.0025373	0.0025051	1.0256831	0.31117474	0.49626023	Nae1
ENSMUSG00000031879.15	263.571591	-0.0134773	0.00284898	22.2979427	2.33E-06	1.88E-05	Ciao2b
ENSMUSG00000031880.8	36.9930499	0.03147949	0.01103188	6.92151798	0.00851647	0.02785983	Rrad

ENSMUSG00000031881.7	13.469479	0.03220046	0.01791608	2.8118375	0.09357114	0.19963537	Cdh16
ENSMUSG00000031883.14	21.9598791	-0.0049731	0.00413822	1.4184193	0.23366375	0.40177919	Car7
ENSMUSG00000031885.15	483.720798	-0.0034334	0.00396993	0.72778372	0.39360252	0.58504594	Cbfb
ENSMUSG00000031886.15	3397.86099	-0.0032544	0.00467156	0.46740647	0.49418274	0.68170512	Ces2e
ENSMUSG00000031887.14	200.177416	0.00164124	0.00270964	0.36666304	0.54482884	0.72810545	Tradd
ENSMUSG00000031889.10	1295.17188	-0.026073	0.0030992	71.9034436	2.26E-17	8.57E-16	Phaf1
ENSMUSG00000031891.7	1.4000759	0.00259873	0.0177205	0.07509028	0.7840646	0.90616168	Hsd11b2
ENSMUSG00000031893.8	1.5004516	-0.0218337	0.02449858	0.96072821	0.32700347	0.51380347	Tsnaxip1
ENSMUSG00000031896.8	5.05370824	-0.0755047	0.01348505	25.9113525	3.57E-07	3.35E-06	Ctrl
ENSMUSG00000031897.10	668.957851	0.01620375	0.00539326	9.1173619	0.00253195	0.00988063	Psmb10
ENSMUSG00000031898.10	1.1839845	-0.0108636	0.03690177	0.14149489	0.70679939	0.85455047	Dpep3
ENSMUSG00000031901.13	131.07206	-0.0055825	0.00267785	4.34138165	0.03719673	0.09470229	Dus2
ENSMUSG00000031902.11	675.90336	-0.0057845	0.00276947	4.30326712	0.03803923	0.0964724	Nfatc3
ENSMUSG00000031903.8	322.172255	0.03074325	0.00329678	85.0360315	2.93E-20	1.58E-18	Pla2g15
ENSMUSG00000031904.6	22.0621874	0.04647309	0.00921805	22.3238322	2.30E-06	1.86E-05	Slc7a6
ENSMUSG00000031906.10	64.0420283	0.05101141	0.00703303	51.3033758	7.91E-13	1.68E-11	Smpd3
ENSMUSG00000031907.10	49.2920221	0.03501624	0.00477472	50.6360018	1.11E-12	2.31E-11	Zfp90
ENSMUSG00000031910.15	14.8785934	-0.0131356	0.00922341	1.95752225	0.16177856	0.30411874	Has3
ENSMUSG00000031913.10	405.020689	-0.0050354	0.0019094	6.9211118	0.0085184	0.02786229	Vps4a
ENSMUSG00000031916.18	587.336689	-0.0036247	0.0020568	3.13694959	0.07653683	0.17020532	Cog8
ENSMUSG00000031917.13	334.327475	-0.0055896	0.00339067	2.66870086	0.10233953	0.21385489	Nip7
ENSMUSG00000031918.17	547.318538	0.00455625	0.00191839	5.57297553	0.01823974	0.05276616	Mtmr2
ENSMUSG00000031919.7	1.84305251	-0.0252662	0.01283432	4.09344395	0.04304984	0.10685893	Tmed6
ENSMUSG00000031921.18	172.150605	0.00052069	0.00273426	0.03679597	0.84788104	0.94594022	Terf2
ENSMUSG00000031922.13	243.09033	0.00581625	0.00242955	5.64048527	0.01755035	0.05110402	Cep57
ENSMUSG00000031924.5	9734.18265	-0.004113	0.00288635	1.95438867	0.16211471	0.30453895	Cyb5b
ENSMUSG00000031925.18	81.6116649	0.02183401	0.0051082	17.4048404	3.02E-05	0.00019386	Maml2
ENSMUSG00000031928.16	132.964557	0.00836427	0.00408611	4.0551619	0.0440365	0.1087814	Mre11a
ENSMUSG00000031930.12	399.656791	0.01114601	0.00289962	14.7085162	0.00012548	0.00070131	Wwp2
ENSMUSG00000031931.5	265.047765	0.00377317	0.00366333	1.04515767	0.30662496	0.49170036	Ankrd49
ENSMUSG00000031933.18	2.30828181	0.06484705	0.01251345	22.2600702	2.38E-06	1.92E-05	Izumo1r
ENSMUSG00000031934.15	23.2823452	0.03256738	0.00784892	15.4324054	8.55E-05	0.0004965	Panx1
ENSMUSG00000031935.10	253.208021	-0.0050194	0.00271743	3.39715156	0.06530911	0.14983825	Med17
ENSMUSG00000031937.8	4.19528596	-0.0049521	0.00786441	0.40228012	0.52591405	0.71120972	Vstm5
ENSMUSG00000031938.16	3459.50054	-0.0030384	0.00381575	0.61015861	0.4347281	0.62646592	4931406C07Rik
ENSMUSG00000031939.17	634.166499	0.00033931	0.00425344	0.00614008	0.93754277	0.99576187	Taf1d
ENSMUSG00000031948.15	940.459119	-0.0016576	0.00219159	0.57335124	0.44893029	0.64073467	Kars
ENSMUSG00000031949.18	87.7908272	-0.0128786	0.00269753	22.6176264	1.98E-06	1.62E-05	Adat1
ENSMUSG00000031950.8	1785.05863	-0.0188591	0.00275741	47.0153484	7.04E-12	1.33E-10	Gabarapl2
ENSMUSG00000031951.9	11.0754339	0.03118766	0.00704072	18.3730381	1.82E-05	0.00012256	Tmem231
ENSMUSG00000031953.3	229.31258	0.00215842	0.00252178	0.72090659	0.39584669	0.58767798	Tmem170
ENSMUSG00000031954.7	819.721295	8.08E-05	0.00258675	0.00096644	0.97519967	1	Cfdp1
ENSMUSG00000031955.11	575.755297	-0.0119044	0.00285094	17.3273456	3.15E-05	0.00020089	Bcar1
ENSMUSG00000031957.7	8.74165358	-0.0162465	0.04294081	0.18470137	0.66736371	0.82620849	Ctrb1
ENSMUSG00000031958.17	2767.41975	-0.0095395	0.0031961	8.66845583	0.00323765	0.01218888	Ldhd
ENSMUSG00000031959.16	205.438151	-0.008712	0.00223325	14.9759225	0.00010889	0.00061694	Wdr59
ENSMUSG00000031960.15	3576.22304	-0.0032387	0.00279814	1.31180993	0.25206722	0.42411595	Aars
ENSMUSG00000031962.7	2.79525996	-0.0308691	0.00848023	12.7687619	0.00035246	0.00175751	Cdh15
ENSMUSG00000031963.9	33.3679093	0.04688632	0.00970555	20.4807912	6.02E-06	4.49E-05	Bmper
ENSMUSG00000031965.16	124.157105	0.03942341	0.00600267	39.5354762	3.22E-10	4.77E-09	Tbx20
ENSMUSG00000031967.16	1059.33839	-0.0104359	0.00217074	23.3446648	1.35E-06	1.14E-05	Afg3l1
ENSMUSG00000031969.17	1052.48977	-0.0125569	0.00246008	26.3679487	2.82E-07	2.69E-06	Acad8
ENSMUSG00000031970.18	1.33365998	0.0115321	0.01795376	0.70702212	0.40043417	0.59214046	Dbndd1
ENSMUSG00000031971.16	11.4691003	0.00890083	0.00877643	0.97683113	0.3229824	0.50952197	Ccsap
ENSMUSG00000031972.6	44.321784	-0.036891	0.02392888	0.77584487	0.37841495	0.569603	Acta1
ENSMUSG00000031974.9	719.602482	-0.0153587	0.00236739	41.6882485	1.07E-10	1.71E-09	Abcb10
ENSMUSG00000031976.15	146.439788	-0.0033844	0.00254525	1.7799486	0.18215564	0.33335839	Urb2
ENSMUSG00000031979.18	347.434295	-0.0033954	0.00201881	2.79378416	0.09463052	0.20142872	Cog2
ENSMUSG00000031980.11	9069.31527	0.00213051	0.00442795	0.23259419	0.62960691	0.79812472	Agt
ENSMUSG00000031982.6	26.7707856	-0.0092882	0.0039014	5.72148703	0.01675853	0.04919312	Arv1
ENSMUSG00000031983.8	279.932501	-0.0016537	0.00273698	0.36386116	0.54636963	0.72942062	2310022B05Rik

ENSMUSG00000031984.9	622.074068	-0.007662	0.00247306	9.53805738	0.00201255	0.00812251	2810004N23Rik
ENSMUSG00000031985.10	873.196246	-0.0094179	0.00299578	9.83824349	0.00170921	0.00702935	Gnpat
ENSMUSG00000031986.7	201.371383	-0.0007068	0.00212656	0.1099666	0.74018216	0.87697475	Sprtn
ENSMUSG00000031987.7	523.731839	-0.0013731	0.00332413	0.16653745	0.6832076	0.83793412	Egln1
ENSMUSG00000031988.11	458.658269	-0.0023709	0.00160749	2.19171956	0.13875433	0.27098284	Vps26b
ENSMUSG00000031990.16	31.82989	0.00961241	0.00466874	4.15444944	0.04152527	0.10374061	Jam3
ENSMUSG00000031993.8	549.137473	-0.0059124	0.001814	10.5100383	0.00118728	0.00510727	Snx19
ENSMUSG00000031994.14	2.50625181	0.01516077	0.01382803	1.44914082	0.22866586	0.39557819	Adamts8
ENSMUSG00000031995.10	21.5995811	0.02903572	0.00686622	17.0872857	3.57E-05	0.00022611	St14
ENSMUSG00000031996.18	6825.62085	0.00730332	0.00253394	8.16045301	0.00428137	0.01549967	Aplp2
ENSMUSG00000031997.10	1.5111982	0.07456778	0.01751078	17.9511562	2.27E-05	0.00014966	Trpc6
ENSMUSG00000032000.18	318.865917	0.01702921	0.00340585	24.3652305	7.97E-07	6.95E-06	Birc3
ENSMUSG00000032002.11	560.677753	-0.0065864	0.00255931	6.64362379	0.00995113	0.03177303	Dcun1d5
ENSMUSG00000032006.16	54.265148	0.01713837	0.00435711	15.143651	9.96E-05	0.00056954	Pdgfd
ENSMUSG00000032009.9	612.827203	0.00299403	0.0033948	0.77050496	0.38006088	0.57147239	Sesn3
ENSMUSG00000032010.16	469.342459	0.00859653	0.00986157	0.75477765	0.3849678	0.57660828	Usp2
ENSMUSG00000032011.6	51.4485128	0.06958618	0.00704111	90.3152323	2.03E-21	1.23E-19	Thy1
ENSMUSG00000032012.10	929.245609	-0.0008834	0.00468057	0.03513788	0.85130703	0.9483718	Nectin1
ENSMUSG00000032013.7	1.25392059	0.04976448	0.02345217	5.99470202	0.0143489	0.04328345	Trim29
ENSMUSG00000032014.7	4594.2229	-0.0073471	0.00380064	3.69621892	0.05453592	0.12963961	Oaf
ENSMUSG00000032017.15	3.52583003	0.01481953	0.00878551	2.87796576	0.0897994	0.19321371	Grik4
ENSMUSG00000032018.15	13118.253	0.00741828	0.0041965	3.05991716	0.08024576	0.17658859	Sc5d
ENSMUSG00000032020.16	77.8527746	0.04340568	0.00609872	46.4407609	9.44E-12	1.76E-10	Ubash3b
ENSMUSG00000032021.15	2.25013459	0.09557989	0.01377565	40.5188352	1.95E-10	2.99E-09	Crtam
ENSMUSG00000032024.11	43.8086301	0.03246851	0.00700613	20.6081713	5.64E-06	4.22E-05	Clmp
ENSMUSG00000032026.8	1124.76463	-0.0078103	0.00231447	11.1488484	0.00084084	0.00377035	Rexo2
ENSMUSG00000032028.16	465.040222	0.00269878	0.0047067	0.31293985	0.57588175	0.75442941	Nxpe2
ENSMUSG00000032030.18	544.266071	0.00191159	0.00255383	0.5470532	0.45952426	0.65106862	Cul5
ENSMUSG00000032033.12	2.0496045	0.01120172	0.0195492	0.26043376	0.60982231	0.78220954	Barx2
ENSMUSG00000032034.7	28.8604085	-0.0404064	0.01269962	9.25649652	0.00234662	0.00926314	Kcnj5
ENSMUSG00000032035.17	547.448583	0.01302012	0.00292814	19.3976552	1.06E-05	7.49E-05	Ets1
ENSMUSG00000032036.16	4.67789569	0.00602207	0.01255113	0.24066945	0.62372302	0.79292466	Kirrel3
ENSMUSG00000032038.8	1441.49675	0.00240786	0.00297346	0.66118828	0.41614075	0.60734561	St3gal4
ENSMUSG00000032040.16	237.522873	-0.0069058	0.00235091	8.66631295	0.00324146	0.01219932	Dcps
ENSMUSG00000032041.16	508.117062	-0.008425	0.00308662	7.30111663	0.00689118	0.02332931	Tirap
ENSMUSG00000032042.12	4135.02564	-0.0074793	0.00396808	3.51772613	0.06071569	0.14133787	Srpr
ENSMUSG00000032044.5	117.340497	-0.0039056	0.00229445	2.88265712	0.08953816	0.19279232	Rpusd4
ENSMUSG00000032046.16	541.264234	0.01090295	0.0027852	15.1929406	9.71E-05	0.00055608	Abhd12
ENSMUSG00000032047.6	7779.25745	-0.0156918	0.00293848	28.3154067	1.03E-07	1.07E-06	Acat1
ENSMUSG00000032050.19	6953.99205	-0.0041249	0.00410444	0.98203772	0.32169624	0.50793464	Rdx
ENSMUSG00000032051.10	1498.64834	-0.0174683	0.00328089	28.4288507	9.72E-08	1.01E-06	Fdx1
ENSMUSG00000032053.9	19.4769259	0.10220472	0.00802834	128.181972	1.02E-29	2.00E-27	Pou2af1
ENSMUSG00000032058.15	358.671766	0.00549107	0.0027704	3.82796474	0.0504041	0.12135173	Ppp2r1b
ENSMUSG00000032059.14	282.490898	-0.005972	0.00320532	3.4918741	0.06167073	0.14304465	Alg9
ENSMUSG00000032060.11	274.00826	0.00069773	0.00648136	0.0111927	0.91574457	0.9838637	Cryab
ENSMUSG00000032062.4	242.92997	-0.0019219	0.00226287	0.72131574	0.39571266	0.58751598	2310030G06Rik
ENSMUSG00000032064.18	413.510362	0.01436013	0.0042601	11.3121663	0.00077001	0.00349191	Dixdc1
ENSMUSG00000032065.7	21.626359	0.01393792	0.00569994	5.8093818	0.0159409	0.04716874	Tex12
ENSMUSG00000032066.10	398.112791	-0.0023941	0.00392091	0.36278515	0.54696349	0.72975907	Bco2
ENSMUSG00000032067.7	1099.55621	0.00101105	0.00262699	0.14779967	0.700647	0.85056083	Pts
ENSMUSG00000032068.15	48.8802817	0.00505527	0.00449099	1.25786836	0.26205497	0.43657083	Plet1
ENSMUSG00000032076.21	700.769813	0.01649279	0.00329572	24.6338763	6.93E-07	6.13E-06	Cadm1
ENSMUSG00000032077.6	113.159553	-0.0129812	0.00394856	10.7628193	0.0010356	0.00453501	Bud13
ENSMUSG00000032078.18	832.706108	-0.0081082	0.00298335	7.41099902	0.00648263	0.02210486	Zpr1
ENSMUSG00000032079.13	23771.8019	-0.0059889	0.00506948	1.35880938	0.24374393	0.41422943	Apoa5
ENSMUSG00000032080.7	9362.60986	0.03740454	0.00696987	25.2753523	4.97E-07	4.53E-06	Apoa4
ENSMUSG00000032081.11	30719.5244	-0.0085903	0.00592189	2.08283323	0.14896346	0.28597534	Apoc3
ENSMUSG00000032083.9	147235.137	-0.0064075	0.00600296	1.14599354	0.28438941	0.46442431	Apoa1
ENSMUSG00000032085.6	248.116653	0.01066445	0.00553357	3.43446089	0.06384942	0.14708003	Tagln
ENSMUSG00000032086.13	527.119776	-0.0122521	0.00291113	17.7185851	2.56E-05	0.00016696	Bace1
ENSMUSG00000032087.12	2.88303473	0.00976227	0.01484357	0.43148239	0.51126246	0.69803973	Dscaml1

ENSMUSG00000032089.17	111.069969	0.05458276	0.0046094	130.087949	3.92E-30	8.29E-28	Il10ra
ENSMUSG00000032091.16	20.9389231	0.09936236	0.00955954	94.5778857	2.36E-22	1.64E-20	Tmprss4
ENSMUSG00000032092.6	639.111524	-0.0013059	0.00369824	0.12776823	0.72075733	0.86381008	Mpzl2
ENSMUSG00000032093.8	19.3522304	0.09188837	0.00771973	121.326316	3.24E-28	4.96E-26	Cd3e
ENSMUSG00000032094.9	20.7129403	0.08280981	0.00954905	61.7366343	3.93E-15	1.13E-13	Cd3d
ENSMUSG00000032096.17	3220.59991	0.00147843	0.00202275	0.53063397	0.46634045	0.65744804	Arcn1
ENSMUSG00000032097.11	1973.5063	0.00147327	0.00264456	0.30632978	0.57994116	0.75789105	Ddx6
ENSMUSG00000032098.14	25.136297	-0.0003499	0.00693368	0.00226918	0.96200642	1	Treh
ENSMUSG00000032101.7	7.31972098	0.02238773	0.00849998	6.68204634	0.00973888	0.03119668	Ddx25
ENSMUSG00000032103.11	97.1467918	-0.0030298	0.00259632	1.33324741	0.24822832	0.41960246	Pus3
ENSMUSG00000032105.6	3.35844422	0.02505084	0.01571731	2.64362753	0.10396591	0.21627624	Nherf4
ENSMUSG00000032109.16	194.745767	0.0023132	0.00306312	0.56236684	0.45330818	0.64497356	Nlrx1
ENSMUSG00000032112.11	396.287492	-0.002862	0.00355824	0.64431817	0.42215144	0.61378768	Trappc4
ENSMUSG00000032113.17	19.1838809	0.01155047	0.0093629	1.38391458	0.23943548	0.40898936	Chek1
ENSMUSG00000032114.10	2496.30623	-0.0125435	0.00386393	10.9121398	0.00095536	0.00421732	Slc37a4
ENSMUSG00000032115.15	2850.13989	-0.0115507	0.00473922	5.62083361	0.01774821	0.05156555	Hyou1
ENSMUSG00000032116.19	2135.36344	0.0010887	0.00333815	0.10412838	0.74693057	0.88117874	Stt3a
ENSMUSG00000032118.17	11.5792934	0.01715965	0.01093738	2.36185136	0.12433455	0.24895293	Fez1
ENSMUSG00000032119.6	206.044603	0.00212917	0.00346667	0.37599787	0.53975293	0.7236592	Hinfp
ENSMUSG00000032120.12	732.719982	-0.0040777	0.003419	1.43121457	0.23156632	0.39924063	C2cd2l
ENSMUSG00000032121.10	81.3732379	-0.0098971	0.00379366	6.84591365	0.00888443	0.02887928	Tmem218
ENSMUSG00000032122.16	66.7680948	0.04537257	0.00541918	65.6363267	5.42E-16	1.72E-14	Slc37a2
ENSMUSG00000032123.6	482.634672	-0.0058814	0.00205562	8.23050956	0.0041192	0.01498386	Dpagt1
ENSMUSG00000032125.22	209.776049	0.00218985	0.00321385	0.46474601	0.49541423	0.68268521	Robo4
ENSMUSG00000032126.17	979.24052	-0.008222	0.00247366	11.03114	0.00089594	0.00398109	Hmbs
ENSMUSG00000032127.10	506.116959	0.00504514	0.00170398	8.83264187	0.0029589	0.01129288	Vps11
ENSMUSG00000032128.16	5.14178814	0.02020203	0.00902231	4.55054317	0.03290831	0.08580315	Robo3
ENSMUSG00000032131.17	2.64552628	0.02940083	0.01475042	4.1660958	0.04124073	0.10318275	Abcg4
ENSMUSG00000032135.16	249.600864	0.0143531	0.00397617	13.1759971	0.00028356	0.00145202	Mcam
ENSMUSG00000032171.8	313.040458	-0.0057484	0.00278637	4.27288391	0.03872515	0.09791718	Pin1
ENSMUSG00000032172.9	17.3008534	0.03790153	0.00684458	30.9453301	2.65E-08	3.01E-07	Olfm2
ENSMUSG00000032174.6	3.75625587	0.04103733	0.01337027	8.92390759	0.00281462	0.01082732	Icam5
ENSMUSG00000032175.12	408.887658	-0.0006094	0.00219615	0.07642026	0.78220849	0.90490334	Tyk2
ENSMUSG00000032177.18	85.908909	-0.0039697	0.00400343	0.99882332	0.3175954	0.50317761	Pde4a
ENSMUSG00000032178.15	344.3871	0.0118255	0.0024157	23.8275337	1.05E-06	9.02E-06	Ilf3
ENSMUSG00000032179.8	214.547917	0.02049706	0.00303251	43.7358924	3.76E-11	6.39E-10	Bmp5
ENSMUSG00000032180.12	268.362319	-0.008387	0.00386565	4.81686269	0.0281826	0.07555294	Tmed1
ENSMUSG00000032181.8	7.18688538	0.02624409	0.01906155	2.64083453	0.10414882	0.21650398	Scg3
ENSMUSG00000032182.16	352.340791	-0.0067501	0.00278095	5.91807804	0.0149863	0.04482207	Yipf2
ENSMUSG00000032184.6	13.9897286	-0.0074087	0.00973996	0.5380379	0.46324702	0.65429932	Lysmd2
ENSMUSG00000032185.17	422.698938	-0.007225	0.00295842	5.81086769	0.01592743	0.04714073	Carm1
ENSMUSG00000032186.16	33.8905876	0.04597947	0.01153028	14.6109238	0.00013215	0.00073406	Tmod2
ENSMUSG00000032187.17	1053.58098	0.00141551	0.00339139	0.16915055	0.68086785	0.83617568	Smarca4
ENSMUSG00000032192.10	22.2141297	0.01505393	0.00552453	7.1836995	0.00735689	0.02461968	Gnb5
ENSMUSG00000032193.10	3332.5093	0.00641883	0.00434451	2.12731494	0.14469402	0.27987151	Ldlr
ENSMUSG00000032194.10	772.800999	0.00616606	0.00306084	4.11121646	0.04259971	0.1059088	Kank2
ENSMUSG00000032198.10	393.326911	0.00733781	0.00409778	3.1964556	0.07379805	0.16523645	Dock6
ENSMUSG00000032199.14	1391.59586	-0.0051535	0.00260448	3.88740631	0.0486495	0.11798927	Polr2m
ENSMUSG00000032202.12	54.0405236	0.01407154	0.0039043	12.9838221	0.00031419	0.00158753	Rab27a
ENSMUSG00000032204.14	5919.67676	-0.0003062	0.00392319	0.00607731	0.93786219	0.99588386	Aqp9
ENSMUSG00000032207.11	2379.9212	-0.0147619	0.00459116	10.1562497	0.00143813	0.00604091	Lipc
ENSMUSG00000032212.11	1287.73752	-0.0016143	0.00328618	0.2411931	0.62334572	0.79279322	Sltm
ENSMUSG00000032215.16	441.550935	-0.0032938	0.00244848	1.79348941	0.18050159	0.33123691	Rsl24d1
ENSMUSG00000032216.16	4702.09482	-0.0001481	0.00303802	0.00236902	0.96118022	1	Nedd4
ENSMUSG00000032217.14	470.065175	-0.0015347	0.00197993	0.59307481	0.44123257	0.63308268	Rnf111
ENSMUSG00000032218.8	23.3788375	0.01098888	0.00983998	1.15325662	0.28286846	0.46286496	Ccnb2
ENSMUSG00000032220.11	949.398886	-0.0123952	0.00303254	16.4404695	5.02E-05	0.0003074	Myo1e
ENSMUSG00000032221.15	29.8289496	0.01833777	0.01049398	2.61883896	0.10560168	0.21896451	Mns1
ENSMUSG00000032224.16	74.206825	-0.0016237	0.0059976	0.0723134	0.78799778	0.90891329	Fam81a
ENSMUSG00000032226.6	6.80473223	0.01543442	0.02412986	0.54035431	0.46228593	0.65348971	Gcnt3
ENSMUSG00000032228.17	428.314664	0.005075	0.00307166	2.67545823	0.10190599	0.21330801	Tcf12

ENSMUSG00000032231.15	388.177178	0.03793973	0.00618006	35.3173466	2.80E-09	3.67E-08	Anxa2
ENSMUSG00000032232.15	1552.94654	0.0010658	0.00339777	0.09766878	0.75464533	0.88645993	Cgnl1
ENSMUSG00000032235.16	238.860592	0.00081278	0.00313588	0.06592449	0.79736587	0.91476587	Ice2
ENSMUSG00000032238.18	1720.08503	-0.0003388	0.00392086	0.0072968	0.93192645	0.99279211	Rora
ENSMUSG00000032239.11	1108.92734	0.00123822	0.00394807	0.09722594	0.75518436	0.88681641	Rp9
ENSMUSG00000032243.9	4.47167975	0.00829021	0.00833375	1.00089209	0.31709474	0.50258696	Itga11
ENSMUSG00000032244.10	371.445197	0.00145642	0.00237013	0.37241599	0.54169028	0.7252672	Fem1b
ENSMUSG00000032245.13	97.4660672	0.03403403	0.0054185	38.5794632	5.26E-10	7.58E-09	Cln6
ENSMUSG00000032246.15	37.2344847	0.02553135	0.00769488	9.90765934	0.00164592	0.00679635	Calml4
ENSMUSG00000032249.15	2555.84859	-0.0053902	0.00394573	1.83084557	0.1760277	0.3247645	Anp32a
ENSMUSG00000032251.13	34.8411843	0.01177792	0.00488677	5.56991618	0.01827164	0.05283251	Irak1bp1
ENSMUSG00000032252.15	546.860078	0.00539684	0.00292974	3.32728017	0.06813947	0.15493469	Glce
ENSMUSG00000032253.14	636.960799	0.00965446	0.0038359	6.11184107	0.01342792	0.04082904	Phip
ENSMUSG00000032254.11	42.1871979	0.0377768	0.00931342	15.3083028	9.13E-05	0.00052684	Kif23
ENSMUSG00000032258.16	12.4443167	0.02045547	0.00836232	5.35395683	0.0206753	0.05860445	Lca5
ENSMUSG00000032261.17	808.283909	0.00020354	0.00292383	0.00480011	0.9447645	0.9994266	Sh3bgrl2
ENSMUSG00000032262.14	9.31830467	0.02769357	0.01845686	2.58951154	0.10757351	0.22221906	Elovl4
ENSMUSG00000032263.15	1218.05164	0.00702572	0.0030628	5.35135151	0.02070621	0.05864274	Bckdhb
ENSMUSG00000032264.10	239.413561	-0.00985	0.00170251	33.480347	7.20E-09	8.89E-08	Zw10
ENSMUSG00000032265.15	298.986216	-0.0098068	0.00405063	5.95449318	0.01467982	0.04411241	Tent5a
ENSMUSG00000032267.9	186.229308	-0.0055598	0.00465144	1.37783431	0.24047039	0.41019271	Usp28
ENSMUSG00000032271.14	2450.44683	-0.0024136	0.00646384	0.13208847	0.71627639	0.86050603	Nnmt
ENSMUSG00000032278.12	6.64345274	0.03273541	0.01395951	5.32801	0.02098533	0.05923409	Paqr5
ENSMUSG00000032279.12	791.325757	-0.0150224	0.00346888	19.1547952	1.21E-05	8.40E-05	Idh3a
ENSMUSG00000032280.18	333.529691	0.01414076	0.0038906	12.5531061	0.00039555	0.00194565	Tle3
ENSMUSG00000032281.12	6.88358642	0.05267561	0.01089763	21.5841939	3.39E-06	2.66E-05	Acsbg1
ENSMUSG00000032285.16	171.209322	-0.016496	0.0044563	13.0231882	0.00030766	0.00155951	Dnaja4
ENSMUSG00000032288.10	482.357124	-0.0052948	0.00298213	3.15691098	0.07560612	0.16838961	Imp3
ENSMUSG00000032289.16	32.9272157	0.0182018	0.00630664	7.96795943	0.00476126	0.01695592	Thsd4
ENSMUSG00000032290.8	404.999692	0.00772459	0.00284981	7.16041704	0.00745299	0.02488127	Ptpn9
ENSMUSG00000032293.9	1642.26996	-0.0068289	0.00284326	5.58802345	0.01808369	0.05239827	Ireb2
ENSMUSG00000032294.18	927.067887	0.04977274	0.00710951	44.3470836	2.75E-11	4.76E-10	Pkm
ENSMUSG00000032295.16	442.067131	-0.0022877	0.00282396	0.67045997	0.41289129	0.60434739	Man2c1
ENSMUSG00000032297.12	3.03432866	0.00149476	0.02789806	0.03415447	0.85337862	0.94949395	Celf6
ENSMUSG00000032298.14	80.1954464	-0.0041902	0.00239833	3.08191532	0.07916725	0.17468787	Neil1
ENSMUSG00000032299.11	308.545754	-0.0070612	0.00288751	6.00726977	0.01424705	0.04302656	Commd4
ENSMUSG00000032300.8	249.649924	-0.016088	0.00381158	17.0206754	3.70E-05	0.0002338	1700017B05Rik
ENSMUSG00000032301.14	3127.05953	-0.0065584	0.00307209	4.54088885	0.0330944	0.08619243	Psma4
ENSMUSG00000032305.16	179.422736	0.00675367	0.00256524	6.78496335	0.00919289	0.02975484	Fam219b
ENSMUSG00000032306.15	188.029656	-0.0158092	0.00330631	22.6666481	1.93E-06	1.58E-05	Mpi
ENSMUSG00000032307.17	637.706802	-0.0127127	0.0025837	23.7834739	1.08E-06	9.22E-06	Ube2q2
ENSMUSG00000032308.10	165.870401	-0.0045418	0.00346085	1.7000194	0.19228544	0.34742182	Ulk3
ENSMUSG00000032309.16	961.702914	-0.0067494	0.00173842	15.0862324	0.00010271	0.00058557	Fbxo22
ENSMUSG00000032310.5	8029.75135	-0.0316293	0.00604517	27.6332182	1.47E-07	1.48E-06	Cyp1a2
ENSMUSG00000032311.18	169.752831	0.01516768	0.00659325	4.95469359	0.02601994	0.07078503	Nrg4
ENSMUSG00000032312.8	951.3622	0.00317347	0.00217857	2.10132718	0.14717135	0.28340818	Csk
ENSMUSG00000032313.12	17.7611807	0.00931929	0.0068519	1.76292109	0.18426058	0.33653168	Tmem266
ENSMUSG00000032314.15	6178.25916	-0.0133279	0.00285273	21.7831012	3.05E-06	2.41E-05	Etfa
ENSMUSG00000032315.7	19.2643536	-0.0233942	0.00534767	20.5797074	5.72E-06	4.27E-05	Cyp1a1
ENSMUSG00000032316.9	894.397107	-0.0088778	0.00295709	9.22100118	0.00239255	0.00941609	Clk3
ENSMUSG00000032320.8	183.886067	0.01302296	0.00337734	14.8625728	0.00011563	0.0006517	Rcn2
ENSMUSG00000032322.15	21.6407856	0.08509442	0.00826258	96.0129637	1.14E-22	8.13E-21	Pstpip1
ENSMUSG00000032323.14	2.29085651	0.10792647	0.01818402	30.7337315	2.96E-08	3.33E-07	Cyp11a1
ENSMUSG00000032324.12	192.299438	0.01972558	0.00509741	14.4968828	0.00014039	0.00077475	Tspan3
ENSMUSG00000032327.15	1.15825806	0.00185508	0.02690026	-0.0047858	1	1	Stra6
ENSMUSG00000032328.13	4277.80667	-0.0148269	0.00225422	42.2846272	7.89E-11	1.29E-09	Tmem30a
ENSMUSG00000032329.5	283.125575	-0.0012645	0.00234362	0.29046735	0.5899212	0.76622509	Hmg20a
ENSMUSG00000032330.8	3153.4577	-0.0192803	0.00360578	28.8195975	7.94E-08	8.38E-07	Cox7a2
ENSMUSG00000032332.18	57.2219761	0.00126997	0.00726819	0.02981396	0.86291303	0.95467267	Col12a1
ENSMUSG00000032333.7	76.0729183	0.01884499	0.00390139	22.9598428	1.65E-06	1.38E-05	Stoml1
ENSMUSG00000032334.11	47.5891277	0.01766056	0.00627904	7.6109957	0.00580134	0.0200749	Loxl1

ENSMUSG00000032336.19	904.698166	0.00638193	0.0030366	4.37973566	0.03636864	0.09315711	Nptn
ENSMUSG00000032338.10	1.71296456	-0.0007456	0.02452458	-0.0145501	1	1	Hcn4
ENSMUSG00000032340.9	331.270584	-0.0083363	0.00313581	7.12042741	0.00762105	0.02535263	Neo1
ENSMUSG00000032342.14	279.626438	-0.0140794	0.00220584	40.3329338	2.14E-10	3.27E-09	Mto1
ENSMUSG00000032344.18	16.6112884	0.03214792	0.00596501	27.4498752	1.61E-07	1.62E-06	Cgas
ENSMUSG00000032348.7	1280.69619	0.01486436	0.00418014	12.5343515	0.00039954	0.00196241	Gsta4
ENSMUSG00000032349.14	11653.8863	0.00873832	0.0047846	3.05380524	0.08054822	0.17708895	Elovl5
ENSMUSG00000032350.10	5247.85721	-0.0079124	0.00428823	3.31161714	0.06879177	0.1560418	Gclc
ENSMUSG00000032352.17	22.8577948	0.0135598	0.0059912	4.85201013	0.02761393	0.07428173	Lrrc1
ENSMUSG00000032353.10	371.947291	0.00618414	0.00368001	2.81084399	0.09362911	0.1997229	Tmed3
ENSMUSG00000032355.17	62.2007022	0.01626484	0.02031789	0.52950826	0.46681367	0.65787926	Mlip
ENSMUSG00000032356.13	7.68455983	0.05630634	0.01382302	15.8485278	6.86E-05	0.00040767	Rasgrf1
ENSMUSG00000032357.13	13.7613467	0.10849552	0.01791753	31.8927081	1.63E-08	1.91E-07	Tinag
ENSMUSG00000032358.13	1.96775532	0.0159475	0.01469779	1.29109809	0.25584569	0.42876058	Fam83b
ENSMUSG00000032359.15	5129.90745	0.00920555	0.0025948	12.5265293	0.00040121	0.00197024	Ctsh
ENSMUSG00000032360.17	14.5678949	-0.040039	0.00782658	23.7618275	1.09E-06	9.31E-06	Hcrtr2
ENSMUSG00000032363.16	120.015914	0.01565983	0.00521778	9.42267299	0.00214319	0.00857639	Adamts7
ENSMUSG00000032366.16	8626.85721	0.00632209	0.00607284	1.05470943	0.30442495	0.48913094	Tpm1
ENSMUSG00000032368.15	4.70609353	0.02285691	0.01569177	1.86894331	0.17159615	0.31855701	Zic1
ENSMUSG00000032369.14	363.962671	0.01233902	0.00369106	10.591703	0.00113596	0.00491161	Plscr1
ENSMUSG00000032370.9	879.220245	-0.012786	0.00362917	12.1357837	0.00049463	0.00237268	Lactb
ENSMUSG00000032372.15	638.52539	0.01465099	0.00325659	19.932247	8.02E-06	5.79E-05	Plscr2
ENSMUSG00000032373.16	7.38422814	0.06008033	0.01712122	11.5018056	0.00069529	0.0031931	Car12
ENSMUSG00000032374.15	41.2205828	0.02117003	0.00725139	7.9199253	0.00488933	0.01734131	Plod2
ENSMUSG00000032375.16	81.2565743	0.00441145	0.00504174	0.73224732	0.39215571	0.58341945	Aph1b
ENSMUSG00000032376.13	590.544223	-0.0129833	0.00271008	22.8733693	1.73E-06	1.44E-05	Usp3
ENSMUSG00000032377.9	86.0396826	0.02325362	0.00328958	48.8794102	2.72E-12	5.42E-11	Plscr4
ENSMUSG00000032380.10	211.125165	0.01681129	0.0027454	37.0478948	1.15E-09	1.58E-08	Dapk2
ENSMUSG00000032381.6	1500.64764	-0.0054623	0.0028572	3.66238576	0.05565389	0.13183214	Ciao2a
ENSMUSG00000032382.15	370.626418	0.01433735	0.00232494	37.5890507	8.73E-10	1.22E-08	Snx1
ENSMUSG00000032383.8	3941.7025	-0.0149175	0.00303484	24.2399363	8.51E-07	7.39E-06	Ppib
ENSMUSG00000032384.17	215.293348	0.01573091	0.00447093	12.0420294	0.00052014	0.00248041	Csnk1g1
ENSMUSG00000032386.16	1025.05849	-0.0116193	0.0022262	27.3210622	1.72E-07	1.72E-06	Trip4
ENSMUSG00000032387.16	947.34122	-0.0060805	0.00309354	3.77958707	0.05188146	0.12420586	Rbpms2
ENSMUSG00000032388.8	490.48187	0.01156635	0.00248248	21.7027968	3.18E-06	2.51E-05	Spg21
ENSMUSG00000032392.12	365.263855	0.01018656	0.00310235	10.8010178	0.00101444	0.00445064	Parp16
ENSMUSG00000032393.16	970.735702	-0.0004759	0.00263997	0.03205989	0.85789628	0.95209865	Dpp8
ENSMUSG00000032396.18	388.142409	-0.0095073	0.00185142	26.0983697	3.24E-07	3.06E-06	Dis3l
ENSMUSG00000032397.8	111.027934	0.00812682	0.00419684	3.68038261	0.05505621	0.13060001	Tipin
ENSMUSG00000032398.8	225.610749	6.67E-05	0.00237657	0.00078341	0.97767053	1	Snapc5
ENSMUSG00000032399.9	8573.211	-0.000639	0.00197476	0.1055201	0.74530325	0.88007864	Rpl4
ENSMUSG00000032400.19	20.1189892	0.01916056	0.00912672	4.13678365	0.04196083	0.10459607	Zwilch
ENSMUSG00000032401.16	63.7044071	0.02014568	0.03943056	0.85333363	0.35561098	0.54414432	Lctl
ENSMUSG00000032402.13	298.095812	0.00617989	0.00366219	2.79108617	0.09478996	0.20165202	Smad3
ENSMUSG00000032403.9	156.023795	-0.0093554	0.00289638	10.4045219	0.00125707	0.00537612	2300009A05Rik
ENSMUSG00000032405.11	409.015236	0.01110872	0.00227083	23.5531174	1.22E-06	1.03E-05	Pias1
ENSMUSG00000032407.15	1293.58248	0.01002513	0.00441081	4.95892191	0.02595638	0.07064474	U2surp
ENSMUSG00000032409.16	264.964864	0.00027459	0.00358448	0.00579762	0.93930606	0.99642317	Atr
ENSMUSG00000032410.14	452.642721	0.00617809	0.00303049	4.13038576	0.04211975	0.1049146	Xrn1
ENSMUSG00000032411.16	385.415909	0.00038788	0.00354151	0.01168182	0.91393028	0.98312004	Tfdp2
ENSMUSG00000032412.9	886.856182	0.02407864	0.00430702	28.4688648	9.52E-08	9.93E-07	Atp1b3
ENSMUSG00000032413.13	84.6905122	-0.0005111	0.00388506	0.01683847	0.89675384	0.97379762	Rasa2
ENSMUSG00000032415.15	29.4792687	-0.0048442	0.00295496	2.68985243	0.10098917	0.21174598	Ube2cbp
ENSMUSG00000032417.11	5.86954804	0.02454874	0.0088461	7.44102204	0.00637537	0.02176743	Rwdd2a
ENSMUSG00000032418.16	6066.74259	-0.0306513	0.00484718	36.7428213	1.35E-09	1.83E-08	Me1
ENSMUSG00000032419.9	5.43850019	-0.0127551	0.02952226	0.0618438	0.80360512	0.91794994	Tbx18
ENSMUSG00000032420.9	394.111855	0.02782797	0.005296	26.153619	3.15E-07	2.99E-06	Nt5e
ENSMUSG00000032422.19	395.129078	0.00015872	0.00158516	0.01015431	0.91973415	0.98644397	Snx14
ENSMUSG00000032423.14	1053.58417	0.00185493	0.00271339	0.45417126	0.50036072	0.68741309	Syncrip
ENSMUSG00000032425.16	138.118312	-0.007832	0.00290784	7.18163662	0.00736535	0.02463402	Zfp949
ENSMUSG00000032431.7	110.717083	0.01174445	0.00402741	8.55109536	0.00345314	0.01287681	Crtap

ENSMUSG00000032434.10	2214.77235	-0.0174474	0.00260963	44.1118867	3.10E-11	5.34E-10	Cmtm6
ENSMUSG00000032435.10	356.543237	0.00083591	0.00229499	0.13176819	0.71660572	0.86080277	Dync1li1
ENSMUSG00000032436.18	70.94134	0.04131456	0.0055768	50.8020615	1.02E-12	2.14E-11	Cmtm7
ENSMUSG00000032437.11	1757.11341	0.00410222	0.00200534	4.12469642	0.0422616	0.10524571	Stt3b
ENSMUSG00000032440.14	683.620101	0.02006349	0.00370374	28.3069923	1.04E-07	1.07E-06	Tgfbr2
ENSMUSG00000032443.18	3.93855021	3.33E-05	0.01010872	-1.42E-05	1	1	Zcwpw2
ENSMUSG00000032446.15	5.90069263	0.15260523	0.0126246	116.444794	3.80E-27	4.79E-25	Eomes
ENSMUSG00000032449.14	142.839641	0.02804985	0.00522378	26.860992	2.19E-07	2.14E-06	Slc25a36
ENSMUSG00000032452.13	8.40864049	0.02563526	0.00802553	9.63896776	0.00190492	0.00774649	Clstn2
ENSMUSG00000032454.10	2.26866689	-0.0246204	0.03698818	0.88867362	0.345837	0.53431884	Rbp2
ENSMUSG00000032456.14	136.036874	0.01091277	0.00317432	11.8933028	0.00056336	0.0026557	Nmnat3
ENSMUSG00000032458.7	2215.77462	-0.0008959	0.00193853	0.21188297	0.64529554	0.81026089	Copb2
ENSMUSG00000032459.10	414.631646	-0.0111718	0.00228011	24.2021816	8.67E-07	7.52E-06	Mrps22
ENSMUSG00000032462.15	282.714329	0.00879079	0.00247711	12.4966021	0.00040769	0.00199706	Pik3cb
ENSMUSG00000032463.11	124.035321	0.00972082	0.00258277	13.9182109	0.00019094	0.00102376	Faim
ENSMUSG00000032468.12	334.582628	0.00452599	0.00188452	5.73372373	0.01664216	0.04890023	Armc8
ENSMUSG00000032469.13	171.322886	-0.0022688	0.00281379	0.65364534	0.41881238	0.61017739	Dbr1
ENSMUSG00000032470.18	78.9100526	-0.0028885	0.00510539	0.31448781	0.57493925	0.75382274	Mras
ENSMUSG00000032473.14	2.87595219	0.03165652	0.01119271	6.43516853	0.01118825	0.03506793	Cldn18
ENSMUSG00000032475.16	518.053867	-0.0040723	0.00298038	1.82265222	0.1769979	0.32617177	Nck1
ENSMUSG00000032477.15	82.6894522	0.00251083	0.00353182	0.49442931	0.48195811	0.67131804	Cdc25a
ENSMUSG00000032478.15	216.732658	-0.0072997	0.00393802	3.48533342	0.06191489	0.14347854	Nme6
ENSMUSG00000032479.16	899.681128	0.00151406	0.00301087	0.25532033	0.61335353	0.78524434	Map4
ENSMUSG00000032480.18	477.637816	-0.0073121	0.00402603	3.27165121	0.07048664	0.15907549	Dhx30
ENSMUSG00000032481.18	725.485495	-0.0040674	0.00340676	1.41250756	0.23464057	0.40304657	Smarcc1
ENSMUSG00000032482.10	63.0053412	-0.0889703	0.00861996	93.0349287	5.14E-22	3.43E-20	Cspg5
ENSMUSG00000032484.9	36.9887303	0.16522857	0.01894796	31.0806169	2.48E-08	2.82E-07	Ngp
ENSMUSG00000032485.15	2216.67676	-0.0197988	0.0038254	26.1564455	3.15E-07	2.98E-06	Scap
ENSMUSG00000032487.9	36.4103757	0.13731669	0.02009784	24.7862056	6.41E-07	5.71E-06	Ptgs2
ENSMUSG00000032489.18	12.610017	-0.0082586	0.00575881	2.08316891	0.14893071	0.28595569	Kif9
ENSMUSG00000032491.15	12.8044308	0.02267691	0.00616795	13.4481767	0.00024524	0.00127719	Nradd
ENSMUSG00000032492.15	218.607508	0.00872297	0.00429006	4.19461362	0.04055259	0.10178498	Pth1r
ENSMUSG00000032495.9	193.09609	0.00331811	0.01617927	0.24607066	0.61985554	0.79013742	Lrrc2
ENSMUSG00000032496.8	31.9402902	0.12970586	0.01989332	5.84777956	0.01559672	0.04634218	Ltf
ENSMUSG00000032497.20	624.433816	-0.0049203	0.00235658	4.37074373	0.03656103	0.09351789	Lrrfip2
ENSMUSG00000032498.10	257.415842	0.00194233	0.0028635	0.46101934	0.49714797	0.68428908	Mlh1
ENSMUSG00000032500.11	195.59245	0.00355276	0.00530625	0.47002176	0.49297716	0.68051968	Dclk3
ENSMUSG00000032501.10	859.074116	-0.0027703	0.00396059	0.47612693	0.49018192	0.67785124	Trib1
ENSMUSG00000032502.9	1.71407628	0.0239011	0.01344706	2.93279257	0.08679724	0.1880753	Stac
ENSMUSG00000032503.19	39.0987257	0.02773296	0.01558965	3.44464919	0.06345689	0.14636165	Arpp21
ENSMUSG00000032504.16	2004.61332	0.00149278	0.00221514	0.45089443	0.50191051	0.68862858	Pdcd6ip
ENSMUSG00000032507.16	7.6386516	0.04855495	0.01130597	17.5147665	2.85E-05	0.00018427	Fbxl2
ENSMUSG00000032508.9	313.333314	0.00231959	0.00275627	0.6738983	0.41169578	0.60327141	Myd88
ENSMUSG00000032511.18	57.7705222	0.00069323	0.02003718	0.0099369	0.9205952	0.98678232	Scn5a
ENSMUSG00000032512.8	502.96419	-0.0047274	0.00318446	2.15070105	0.14250481	0.27654422	Wdr48
ENSMUSG00000032513.9	492.083334	-0.0088277	0.00291216	9.07584842	0.00259007	0.01007911	Gorasp1
ENSMUSG00000032514.12	2.04393099	-0.0210634	0.01550455	2.13235352	0.14421917	0.27925075	Ttc21a
ENSMUSG00000032515.9	176.407195	-0.0056222	0.00475106	1.35157156	0.24500356	0.41585018	Csrnp1
ENSMUSG00000032517.17	27.3835605	0.02221598	0.01694451	2.08024134	0.14921658	0.28637882	Mobp
ENSMUSG00000032518.7	10477.0988	-0.0070644	0.00431487	2.70302772	0.10015788	0.21047075	Rpsa
ENSMUSG00000032519.12	342.850784	-0.0077473	0.00262241	8.72649744	0.00313618	0.01185797	Slc25a38
ENSMUSG00000032523.12	6.52913538	-0.0351818	0.02896068	-1.0445444	1	1	Hhatl
ENSMUSG00000032525.16	1109.64305	-0.0015281	0.00392513	0.14988399	0.69864625	0.84908901	Nktr
ENSMUSG00000032526.12	693.572306	-0.0134396	0.00376748	12.6958354	0.00036647	0.00181473	Ss18l2
ENSMUSG00000032527.14	2754.13938	-0.007821	0.00253575	9.42889496	0.00213593	0.00855315	Pccb
ENSMUSG00000032528.6	584.761574	-0.0034445	0.00340524	1.0184674	0.31288282	0.49827749	Vipr1
ENSMUSG00000032531.16	576.500799	-0.0036071	0.00300014	1.39862306	0.23695425	0.40600994	Amotl2
ENSMUSG00000032532.8	4.39799628	0.03429725	0.02738162	2.40897314	0.12064148	0.24320085	Cck
ENSMUSG00000032534.19	222.079173	-0.0044755	0.00300264	2.19672601	0.13830421	0.2703276	Cep63
ENSMUSG00000032536.13	1258.92794	-0.007106	0.00305305	5.34044769	0.02083612	0.0589186	Trak1
ENSMUSG00000032537.16	8.34479164	0.02056858	0.00889193	5.30850746	0.02122153	0.05975063	Ephb1

ENSMUSG00000032540.17	711.769318	-0.0300845	0.00308656	94.8377343	2.07E-22	1.44E-20	Abhd5
ENSMUSG00000032547.13	751.660836	-0.0055644	0.00262948	4.44198524	0.03506545	0.09039826	Ryk
ENSMUSG00000032548.15	1434.6007	-0.0124141	0.0044365	7.76579755	0.00532448	0.01866297	Slco2a1
ENSMUSG00000032549.8	59.5061743	0.02644223	0.00563444	20.6539689	5.50E-06	4.13E-05	Rab6b
ENSMUSG00000032551.8	275.742321	0.0023151	0.00347472	0.43001989	0.51197919	0.69861389	1110059G10Rik
ENSMUSG00000032553.15	569.336107	0.00017971	0.00434099	0.00165627	0.96753722	1	Srprb
ENSMUSG00000032554.16	281743.673	0.01021934	0.00489059	4.33787256	0.03727347	0.09486389	Trf
ENSMUSG00000032555.11	255.293189	0.00950188	0.00400349	5.36578114	0.02053559	0.05826445	Topbp1
ENSMUSG00000032556.12	1.1224323	0.07761386	0.01690626	22.6196316	1.97E-06	1.62E-05	Bfsp2
ENSMUSG00000032557.14	586.46538	-0.0040502	0.00240288	2.79843645	0.09435628	0.20094693	Uba5
ENSMUSG00000032558.20	158.048038	0.00397253	0.00553743	0.48702078	0.48525929	0.6741773	Nphp3
ENSMUSG00000032560.15	763.952622	-0.0008144	0.00338192	0.05721111	0.81095946	0.92218936	Dnajc13
ENSMUSG00000032561.16	243.00012	0.0361931	0.00691551	25.3721703	4.73E-07	4.32E-06	Acp3
ENSMUSG00000032562.14	1703.87776	0.01963672	0.00282287	47.2827633	6.15E-12	1.17E-10	Gnai2
ENSMUSG00000032563.17	459.3843	-0.0028385	0.00276905	1.04137041	0.30750297	0.4925477	Mrpl3
ENSMUSG00000032564.17	5.99578228	0.05317544	0.02056542	6.70781643	0.00959912	0.03083274	Cpne4
ENSMUSG00000032565.10	166.360327	0.00886255	0.00367728	5.70036783	0.01696136	0.04968436	Nudt16
ENSMUSG00000032567.17	79.4047718	-0.0081055	0.00311778	6.80717241	0.00907924	0.02943142	Aste1
ENSMUSG00000032570.18	831.822771	-0.0021382	0.00243661	0.75392117	0.3852376	0.57686585	Atp2c1
ENSMUSG00000032571.15	350.202284	-0.0016343	0.00211954	0.59795069	0.439361	0.63133718	Pik3r4
ENSMUSG00000032572.10	2.20316766	0.01421142	0.02088931	0.95054625	0.32958029	0.51644172	Col6a4
ENSMUSG00000032575.17	2049.45709	-0.00256	0.00452847	0.30476691	0.58090931	0.75878203	Manf
ENSMUSG00000032577.17	101.249253	0.01258926	0.00438925	7.89396989	0.00495999	0.01756287	Mapkapk3
ENSMUSG00000032578.8	386.225255	-0.0195257	0.00888484	4.60277675	0.03192022	0.08371736	Cish
ENSMUSG00000032579.15	238.29879	-0.0033412	0.00585846	0.3278046	0.56695469	0.746883	Hemk1
ENSMUSG00000032580.13	883.645426	-0.000802	0.00264584	0.0912056	0.76265005	0.89200122	Rbm5
ENSMUSG00000032582.15	607.881779	-0.0014929	0.00263202	0.32049933	0.57130772	0.75081428	Rbm6
ENSMUSG00000032583.8	247.339537	-0.016794	0.00230241	53.0746241	3.21E-13	7.23E-12	Mon1a
ENSMUSG00000032584.13	9.05766843	0.02892274	0.00982928	8.15453353	0.00429536	0.01554557	Mst1r
ENSMUSG00000032586.10	9.25538776	0.02908364	0.00918711	9.08865201	0.002572	0.0100154	Traip
ENSMUSG00000032589.15	17.3759226	0.03555815	0.01298871	7.12600361	0.00759739	0.0252846	Bsn
ENSMUSG00000032590.15	1009.25265	-0.0059532	0.00350201	2.88612484	0.08934559	0.19244795	Apeh
ENSMUSG00000032591.16	2342.32904	-0.0017071	0.00486247	0.12458218	0.72411693	0.86633975	Mst1
ENSMUSG00000032593.6	16.5468097	-0.0029592	0.00533002	0.310508	0.57736862	0.75577556	Amigo3
ENSMUSG00000032594.12	1294.57862	0.00165685	0.00476861	0.11631573	0.73306524	0.87200307	Ip6k1
ENSMUSG00000032596.15	257.940252	0.0249756	0.00446678	31.2930672	2.22E-08	2.54E-07	Uba7
ENSMUSG00000032598.9	125.583225	0.00045756	0.00625327	0.00528897	0.94202472	0.9980951	Nckipsd
ENSMUSG00000032599.13	539.912372	-0.0092881	0.00397668	5.54625908	0.01852023	0.05342034	Ip6k2
ENSMUSG00000032601.14	1958.37997	-0.0009109	0.00310101	0.08479119	0.77090657	0.8974188	Prkar2a
ENSMUSG00000032602.7	2098.47681	-0.0177909	0.00280477	39.0615704	4.11E-10	6.02E-09	Slc25a20
ENSMUSG00000032604.17	779.484778	-0.0072271	0.00236205	9.37679168	0.0021975	0.00877585	Qars
ENSMUSG00000032606.8	324.037746	-0.0005173	0.00366596	0.01958283	0.88870847	0.96943077	Nicn1
ENSMUSG00000032607.13	772.808591	0.00052814	0.00347825	0.02231188	0.88126041	0.96460962	Amt
ENSMUSG00000032609.13	147.304097	-0.0070589	0.0050947	1.96092797	0.16141411	0.30362175	Klhdc8b
ENSMUSG00000032611.10	3.96045144	-0.0299772	0.00830933	12.6980271	0.00036604	0.00181299	1700102P08Rik
ENSMUSG00000032612.15	1501.37019	-0.0049708	0.00306587	2.62396308	0.10526125	0.21837381	Usp4
ENSMUSG00000032615.15	143.480021	-0.0098422	0.00263094	14.3458459	0.00015211	0.00083359	Nt5m
ENSMUSG00000032621.9	1638.59157	0.00334816	0.00425786	0.61358017	0.43344299	0.62500375	Srek1
ENSMUSG00000032624.17	364.679545	0.00154458	0.00265072	0.33170991	0.56465396	0.74510061	Eml4
ENSMUSG00000032625.15	29.3467048	0.04021689	0.00851857	20.1327194	7.23E-06	5.28E-05	Thsd7a
ENSMUSG00000032633.13	536.136202	0.00249589	0.00300797	0.69235429	0.40536475	0.59696135	Flcn
ENSMUSG00000032637.16	978.538485	-0.0054554	0.00368609	2.19189541	0.1387385	0.27098034	Atxn2l
ENSMUSG00000032640.11	97.3726795	0.0206827	0.00329965	38.2373568	6.26E-10	8.95E-09	Chsy1
ENSMUSG00000032641.19	53.9027991	-0.0023972	0.00325017	0.53731828	0.46354625	0.65452597	Gpr19
ENSMUSG00000032643.13	11.9903269	0.04573208	0.00801896	31.3173649	2.19E-08	2.51E-07	Fhl3
ENSMUSG00000032648.15	140.91636	0.0027982	0.00786037	0.12110327	0.72784097	0.86859889	Pygm
ENSMUSG00000032649.15	17.9028228	-0.0388087	0.00655437	34.7842463	3.68E-09	4.75E-08	Colgalt2
ENSMUSG00000032652.14	888.80128	-0.0022252	0.0022925	0.94053288	0.33214084	0.51907459	Crebl2
ENSMUSG00000032656.15	18.8907429	0.00069208	0.0054871	0.01583299	0.89986714	0.97569375	Marchf3
ENSMUSG00000032657.16	21.4240693	0.03571721	0.00675215	25.6926601	4.00E-07	3.72E-06	Entrep3
ENSMUSG00000032661.10	54.3360255	0.02399175	0.00663221	12.76485	0.00035319	0.00176007	Oas3

ENSMUSG00000032666.17	131.908966	0.00033995	0.00638851	0.00266466	0.95883124	1	1700025G04Rik
ENSMUSG00000032667.18	1311.53914	0.00311233	0.00264662	1.35746906	0.2439766	0.41445226	Pon2
ENSMUSG00000032671.6	2.89950698	-0.0104849	0.00873969	1.41342085	0.23448934	0.40284548	A930018P22Rik
ENSMUSG00000032673.6	468.798718	-0.0075296	0.00314723	5.63703085	0.01758496	0.05119217	Prorsd1
ENSMUSG00000032679.13	2486.91132	-0.0019928	0.00475979	0.17147582	0.67880352	0.83425875	Cd59a
ENSMUSG00000032680.12	1.25899331	0.03179087	0.02418878	3.16424483	0.07526724	0.16782502	6820408C15Rik
ENSMUSG00000032688.10	192.839886	0.01445258	0.00319448	19.9879707	7.79E-06	5.64E-05	Malt1
ENSMUSG00000032690.17	76.6242057	0.02052307	0.00599225	11.5834438	0.00066542	0.00307615	Oas2
ENSMUSG00000032691.15	22.3839912	0.05390673	0.00651828	64.1604464	1.15E-15	3.52E-14	Nlrp3
ENSMUSG00000032698.16	98.7965112	0.03321623	0.00478703	44.8516784	2.13E-11	3.75E-10	Lmo2
ENSMUSG00000032702.17	906.44328	-0.0030012	0.0033335	0.80185146	0.37054039	0.56112781	Kank1
ENSMUSG00000032705.10	212.577956	-0.0014254	0.00244301	0.33822554	0.56085527	0.7420808	Exd2
ENSMUSG00000032712.17	629.155242	0.02214872	0.00437782	24.6101585	7.02E-07	6.19E-06	Resf1
ENSMUSG00000032714.7	70.3832083	0.0051949	0.0036167	2.06075894	0.15113488	0.28901146	Syde1
ENSMUSG00000032715.10	115.555807	0.00608811	0.00639464	0.93416179	0.33378382	0.52067626	Trib3
ENSMUSG00000032717.15	9.13649251	0.0091107	0.00592813	2.28109329	0.13095953	0.25930251	Mdfi
ENSMUSG00000032718.5	19.8188155	0.00552166	0.00418491	1.79612628	0.18018152	0.3308237	Mansc1
ENSMUSG00000032719.5	2.09073978	-0.0188685	0.0106017	2.8052948	0.09395357	0.20027013	Sbspon
ENSMUSG00000032724.6	321.214141	-0.0082718	0.00454135	3.25950615	0.07101051	0.16005866	Abtb2
ENSMUSG00000032725.11	248.605049	0.0317171	0.00575188	28.3503194	1.01E-07	1.05E-06	Folr2
ENSMUSG00000032727.15	320.497319	-0.0052084	0.00342959	2.2229959	0.13596899	0.26684677	Mier3
ENSMUSG00000032733.7	313.830847	0.00231781	0.00214406	1.15975367	0.28151663	0.46126094	Snx33
ENSMUSG00000032735.16	449.798791	-0.0244908	0.00340084	50.053916	1.50E-12	3.06E-11	Ablim3
ENSMUSG00000032737.14	545.456954	-0.0084897	0.00275639	9.54333593	0.00200677	0.00810611	Inppl1
ENSMUSG00000032739.17	15.5545746	0.03445541	0.00957048	12.4679251	0.000414	0.00202333	Pram1
ENSMUSG00000032740.18	127.4917	0.03622578	0.00679524	26.402965	2.77E-07	2.65E-06	Ccdc88a
ENSMUSG00000032741.10	751.830017	-0.0094139	0.0029774	9.95996557	0.00159981	0.00663032	Tpcn1
ENSMUSG00000032743.16	313.452366	0.00189102	0.00408345	0.21132978	0.64572713	0.81058705	Katnip
ENSMUSG00000032744.5	53.1670966	0.00055912	0.00551246	0.00995545	0.92052138	0.98678232	Heyl
ENSMUSG00000032745.19	947.578802	-0.0039454	0.00214909	3.3157418	0.06861935	0.15575555	Gpbp1
ENSMUSG00000032750.14	11.8914529	0.04212982	0.00689576	34.8872243	3.49E-09	4.52E-08	Gab3
ENSMUSG00000032754.15	791.180754	0.01318345	0.00439438	8.75459414	0.00308823	0.01170857	Slc8b1
ENSMUSG00000032757.7	1671.58723	-0.0068151	0.00326906	4.184926	0.04078499	0.10229107	Bet1
ENSMUSG00000032758.5	498.596777	-0.0491456	0.03037944	3.2873359	0.06981622	0.15784943	Kap
ENSMUSG00000032763.12	452.241882	-0.0032726	0.00314978	1.08606749	0.29734417	0.48084742	Ilvbl
ENSMUSG00000032766.10	235.73993	0.00384544	0.00469612	0.66399636	0.41515261	0.60645296	Gng11
ENSMUSG00000032773.10	6.15969638	0.03661191	0.01005449	12.814782	0.00034389	0.00171958	Chrm1
ENSMUSG00000032776.10	309.612046	0.00029351	0.00424441	0.00466217	0.94556266	0.99995699	Mctp2
ENSMUSG00000032777.10	889.96464	-0.0116368	0.00249869	21.6277173	3.31E-06	2.60E-05	Gtf3c1
ENSMUSG00000032782.20	162.895559	0.00184576	0.00506743	0.13229209	0.71606726	0.8604027	Cntrob
ENSMUSG00000032783.10	6.82377167	0.01764333	0.01475896	1.38345916	0.23951281	0.4090622	Troap
ENSMUSG00000032786.17	7031.36009	-0.0151613	0.00675145	5.14348672	0.02333374	0.06465573	Alas1
ENSMUSG00000032788.16	1035.38153	-0.0116162	0.00352738	10.9803361	0.00092084	0.00408249	Pdxk
ENSMUSG00000032796.9	95.5250484	0.01812631	0.00516302	11.9440373	0.00054823	0.00259491	Lama1
ENSMUSG00000032802.9	827.434445	0.01626331	0.00358618	19.8414643	8.41E-06	6.05E-05	Srxn1
ENSMUSG00000032803.16	2798.38862	-0.0020973	0.00313086	0.44024494	0.50700426	0.69383246	Cdv3
ENSMUSG00000032806.14	88.5791485	0.00781586	0.00289733	7.18557058	0.00734922	0.02460338	Slc10a3
ENSMUSG00000032808.3	1014.30301	0.04513772	0.00536033	65.15095	6.94E-16	2.18E-14	Cyp2c38
ENSMUSG00000032812.19	1159.37958	0.0013729	0.00400462	0.1170131	0.73229687	0.87157259	Arap1
ENSMUSG00000032815.17	16.3463076	0.01284061	0.00969289	1.75655756	0.18505447	0.33758952	Fanca
ENSMUSG00000032816.17	14.6997794	0.0217979	0.01151591	3.36345265	0.06665818	0.1522026	Igdcc4
ENSMUSG00000032826.20	125.640673	0.02056034	0.00631146	9.840722	0.0017069	0.00702356	Ank2
ENSMUSG00000032827.16	285.184821	-0.0044757	0.00504557	0.73735361	0.39050993	0.58191605	Ppp1r9a
ENSMUSG00000032834.12	247.569367	-0.0069908	0.00231167	8.88082513	0.00288181	0.01104133	Pwp2
ENSMUSG00000032839.14	17.5126314	0.02582028	0.0063273	15.9038081	6.66E-05	0.00039754	Trpc1
ENSMUSG00000032840.8	147.471797	0.00968322	0.00284743	11.6642593	0.00063712	0.00296044	Spring1
ENSMUSG00000032841.16	38.2001432	0.02237495	0.0048971	19.4446057	1.04E-05	7.32E-05	Prr5l
ENSMUSG00000032842.14	141.606528	-0.0135911	0.00403554	11.525635	0.00068643	0.00315844	Abcc10
ENSMUSG00000032845.17	30.6718381	0.00952917	0.01233471	2.06700394	0.15051696	0.28820368	Alpk2
ENSMUSG00000032846.13	85.1362969	0.0137395	0.00366455	13.6004866	0.00022613	0.00118838	Zswim6
ENSMUSG00000032849.15	99.98677	0.04608385	0.006264	51.3065762	7.90E-13	1.68E-11	Abcc4

ENSMUSG00000032850.17	4.36263772	0.02179982	0.01148285	3.46081455	0.06283935	0.1452498	Rnft2
ENSMUSG00000032854.13	18.4918017	0.03018087	0.00933749	10.0326288	0.00153791	0.0064121	Ugt8a
ENSMUSG00000032855.7	539.920419	0.00377824	0.00556005	0.44791861	0.50332503	0.69023921	Pkd1
ENSMUSG00000032860.6	233.526289	-0.0117	0.0034675	11.2479432	0.00079711	0.00360236	P2ry2
ENSMUSG00000032867.9	687.763978	-0.0059739	0.00256755	5.37880547	0.02038283	0.05788674	Fbxw8
ENSMUSG00000032869.16	457.810173	-0.0080793	0.00220508	13.6372818	0.00022174	0.00116896	Psmf1
ENSMUSG00000032870.9	715.475606	0.0023696	0.00211356	1.24871718	0.26379763	0.43878215	Smap2
ENSMUSG00000032872.17	646.115149	-0.0016338	0.00267445	0.37086803	0.5425315	0.72602258	Cyb5r4
ENSMUSG00000032875.9	113.221987	0.00991457	0.00503119	3.89013201	0.04857061	0.11784655	Arhgef17
ENSMUSG00000032878.18	13.4571028	0.02133108	0.01376473	2.09240254	0.14803311	0.28455655	Ccdc85a
ENSMUSG00000032883.16	823.568061	0.02273797	0.00370711	37.8303256	7.72E-10	1.09E-08	Acsl3
ENSMUSG00000032890.18	44.7283602	0.0413404	0.00651779	38.1131359	6.68E-10	9.51E-09	Rims3
ENSMUSG00000032892.15	38.437076	-0.0292054	0.00613073	22.4058365	2.21E-06	1.79E-05	Rangrf
ENSMUSG00000032897.18	378.473063	-0.0031375	0.00229279	1.8889704	0.1693181	0.31514651	Nfyc
ENSMUSG00000032898.10	1567.73581	-0.0025941	0.00461393	0.31440465	0.57498981	0.75384712	Fbxo21
ENSMUSG00000032899.15	3.83939288	0.0634497	0.01405108	18.5836984	1.63E-05	0.00011081	Styk1
ENSMUSG00000032902.2	1238.70916	-0.0073252	0.00334795	4.58123146	0.03232398	0.0845788	Slc16a1
ENSMUSG00000032905.6	748.87433	-0.0074594	0.00160394	21.436267	3.66E-06	2.85E-05	Atg12
ENSMUSG00000032908.10	9.03599229	0.01087082	0.00776433	1.91964474	0.16589583	0.31022112	Sgpp2
ENSMUSG00000032911.7	9.96064026	-0.006274	0.01109906	0.32973152	0.56581722	0.7459422	Cspg4
ENSMUSG00000032913.14	161.15045	0.00605195	0.00310543	3.68652099	0.05485392	0.13019871	Lrig2
ENSMUSG00000032915.7	201.218551	0.04263315	0.00547752	56.7774546	4.88E-14	1.21E-12	Adgre4
ENSMUSG00000032925.17	21.1226002	0.00055628	0.00825993	0.00421234	0.94825159	1	Itgbl1
ENSMUSG00000032932.16	799.426566	-0.0022633	0.00314108	0.51336745	0.47368474	0.6644263	Hspa13
ENSMUSG00000032936.14	10.0461373	0.00605701	0.0185831	0.05172966	0.82008041	0.92778283	Camkv
ENSMUSG00000032939.15	114.649437	0.0067851	0.0040883	2.7309108	0.09842314	0.2076208	Nup93
ENSMUSG00000032940.17	1.45920416	0.06170024	0.02055878	11.6998402	0.00062505	0.00290895	Rbm11
ENSMUSG00000032942.15	5.14387432	-0.001847	0.02198051	0.11794815	0.73127063	0.87096647	Ucp3
ENSMUSG00000032946.17	330.824931	0.02923454	0.00374091	58.9864101	1.59E-14	4.24E-13	Rasgrp2
ENSMUSG00000032952.12	306.11714	-0.009802	0.00194147	25.6854824	4.02E-07	3.73E-06	Ap4b1
ENSMUSG00000032959.13	2989.75747	-0.0099776	0.00260254	14.6038771	0.00013264	0.00073629	Pebp1
ENSMUSG00000032965.12	27.6370849	0.04060292	0.00631181	40.7292208	1.75E-10	2.70E-09	Ift57
ENSMUSG00000032966.15	989.292172	0.00606122	0.00342654	3.11386184	0.0776287	0.17204947	Fkbp1a
ENSMUSG00000032968.5	2.47634924	0.00816804	0.01070561	0.58226527	0.44542577	0.63769086	Inha
ENSMUSG00000032977.10	432.10375	-0.0069068	0.00231737	8.90229481	0.00284812	0.01092969	Slx9
ENSMUSG00000032978.15	4.98623284	0.0306952	0.02941762	1.70971974	0.19102174	0.34582268	Guca2b
ENSMUSG00000032985.16	6.64955512	-2.37E-05	0.00986974	1.04E-05	0.99742127	1	5730522E02Rik
ENSMUSG00000032997.17	64.3853168	0.02532587	0.00481586	26.2324153	3.03E-07	2.88E-06	Chpf
ENSMUSG00000032998.17	284.033064	0.00468617	0.00315393	2.15823908	0.14180712	0.27546226	Foxj3
ENSMUSG00000033004.17	706.661227	0.00754876	0.00322994	5.3991441	0.02014663	0.05731256	Mycbp2
ENSMUSG00000033006.11	4.11886031	-0.0090551	0.01801922	0.22075991	0.63846151	0.80519481	Sox10
ENSMUSG00000033009.16	274.170908	-0.0007965	0.00330054	0.05806413	0.80958225	0.92156404	Ogfod1
ENSMUSG00000033014.13	353.967793	0.00040989	0.00264641	0.02381619	0.87735353	0.9625626	Trim33
ENSMUSG00000033016.17	202.853209	0.01105787	0.00319841	11.7645797	0.00060369	0.00282007	Nfatc1
ENSMUSG00000033020.8	387.880569	-0.0087062	0.00356806	5.94742265	0.01473882	0.04424343	Polr2f
ENSMUSG00000033021.17	404.748203	-0.0018298	0.00345982	0.28230253	0.59519601	0.77012418	Gmppa
ENSMUSG00000033022.9	23822.9885	0.0093998	0.00416458	5.14012877	0.02337891	0.06475808	Cdo1
ENSMUSG00000033024.17	5.43027409	0.05804322	0.01299824	5.50374737	0.01897577	0.05448113	Klra9
ENSMUSG00000033029.13	9.80012608	-0.0029442	0.00892211	0.10522948	0.74564208	0.88017187	1700088E04Rik
ENSMUSG00000033031.14	39.6397129	0.01873476	0.00729578	6.38757991	0.01149216	0.03581108	Cip2a
ENSMUSG00000033032.16	100.106046	-0.009266	0.00412576	5.06329881	0.02443768	0.06717016	Afap1l1
ENSMUSG00000033033.11	42.618205	0.03350941	0.00462277	50.624642	1.12E-12	2.33E-11	Calhm2
ENSMUSG00000033036.10	4.57358495	0.00117684	0.01162604	0.01476605	0.90328262	0.97733033	Ppihl
ENSMUSG00000033039.11	309.379251	-0.0141713	0.00352312	15.9415214	6.53E-05	0.00039029	Micall1
ENSMUSG00000033044.13	25.6264106	-0.0157024	0.04039117	0.03503406	0.85152431	0.94843175	Dhrs7c
ENSMUSG00000033047.6	1376.52211	-0.0050346	0.00168105	8.93555526	0.00279672	0.01076253	Eif3l
ENSMUSG00000033054.8	143.453892	0.00385267	0.00276842	1.88616155	0.1696355	0.31560585	Npat
ENSMUSG00000033055.13	150.426814	-0.001326	0.0028668	0.21344114	0.64408354	0.8095203	Ankrd54
ENSMUSG00000033059.8	232.147848	0.01437038	0.00646291	4.84076107	0.02779463	0.07471667	Pygb
ENSMUSG00000033060.17	541.038545	0.00809355	0.00426382	3.53071048	0.06024197	0.14040189	Lmo7
ENSMUSG00000033063.9	11.3399771	0.01429903	0.02278029	0.63061556	0.42712961	0.61865276	Cntnap3

ENSMUSG00000033065.15	467.848841	-0.0130824	0.00429267	9.36160007	0.00221579	0.00883211	Pfkm
ENSMUSG00000033066.18	76.0018019	0.0294675	0.00534447	28.461334	9.56E-08	9.97E-07	Gas7
ENSMUSG00000033068.17	273.572861	0.00032195	0.00332238	0.00951007	0.9223138	0.98786128	Entpd6
ENSMUSG00000033075.18	183.567412	0.00433952	0.00308285	1.93860772	0.16381979	0.30702054	Senp1
ENSMUSG00000033082.11	70.2292039	0.00644499	0.00454622	1.9968377	0.15762777	0.29830768	Clec1a
ENSMUSG00000033083.17	136.166326	0.03695351	0.00465438	55.5836104	8.96E-14	2.14E-12	Tbc1d4
ENSMUSG00000033088.20	318.178645	0.00108825	0.00233444	0.2177789	0.64073752	0.80698645	Triobp
ENSMUSG00000033096.8	599.98898	-0.0040476	0.00176224	5.33135309	0.02094512	0.0591596	Apmap
ENSMUSG00000033099.11	183.00768	-0.0029719	0.00321873	0.85574915	0.354931	0.54361858	Nol12
ENSMUSG00000033102.16	627.050472	-0.0065371	0.0034637	3.53117593	0.06022506	0.14037635	Cdc14b
ENSMUSG00000033105.13	1585.89298	0.01350598	0.00500404	6.86928043	0.008769	0.02856706	Lss
ENSMUSG00000033106.7	344.989098	-0.0066158	0.00256686	6.64473078	0.00994495	0.0317576	Slc7a6os
ENSMUSG00000033107.7	1604.71092	-0.0104665	0.00737117	1.89155373	0.16902678	0.31472848	Rnf125
ENSMUSG00000033111.17	359.004945	0.00434491	0.0029695	2.06473752	0.15074088	0.28850898	Cep15
ENSMUSG00000033114.12	935.08338	0.00074497	0.00348071	0.04492952	0.83213368	0.93545131	Slc35d2
ENSMUSG00000033124.17	1057.96228	-0.0165511	0.00296066	30.8044647	2.85E-08	3.22E-07	Atg9a
ENSMUSG00000033126.6	79.1031991	-0.0070646	0.00240686	8.55704606	0.00344187	0.01284698	Ybey
ENSMUSG00000033128.10	498.654404	-0.0034141	0.00262227	1.70249255	0.19196233	0.34699751	Gga1
ENSMUSG00000033147.17	276.792042	-0.0010767	0.00501336	0.04439597	0.83311863	0.93602325	Slc22a15
ENSMUSG00000033149.18	1039.0245	-0.0034301	0.00312707	1.18313246	0.27671921	0.45542525	Phldb2
ENSMUSG00000033152.14	6.38254762	0.01879657	0.01065594	2.99368672	0.08358966	0.18243229	Podxl2
ENSMUSG00000033157.18	124.267835	-0.0034868	0.00310577	1.25901832	0.26183699	0.43635182	Abhd10
ENSMUSG00000033159.15	838.092713	-0.0028496	0.00284959	1.00049364	0.31719109	0.50270589	Cnppd1
ENSMUSG00000033161.11	5388.29847	0.00224097	0.00258029	0.76569086	0.38155343	0.57302255	Atp1a1
ENSMUSG00000033166.12	199.070973	0.00202897	0.00330205	0.36742308	0.54441227	0.72779617	Dis3
ENSMUSG00000033170.15	205.756133	0.00030606	0.00369896	0.00691671	0.93371895	0.99380393	Card10
ENSMUSG00000033174.18	3188.18113	-0.0230496	0.00431956	27.0572136	1.98E-07	1.95E-06	Mgll
ENSMUSG00000033177.14	2.3362627	0.0192368	0.01499285	1.47100334	0.22518746	0.39113862	Tmprss7
ENSMUSG00000033182.13	45.3323339	-0.0315092	0.00896648	10.3013907	0.0013293	0.00563394	Kbtbd12
ENSMUSG00000033184.15	2298.27786	-0.0006834	0.00272996	0.06202646	0.80332124	0.91779337	Tmed7
ENSMUSG00000033186.10	567.713548	0.00038708	0.00268194	0.02052229	0.88608798	0.96751406	Mzt1
ENSMUSG00000033187.9	2.23021343	0.04052261	0.01472365	7.01508149	0.0080826	0.02663631	BC016579
ENSMUSG00000033191.15	224.902029	-0.0120782	0.00339586	12.5968528	0.0003864	0.00190421	Tie1
ENSMUSG00000033192.6	77.1726682	0.06724056	0.00507051	163.064878	2.42E-37	1.22E-34	Lpcat2
ENSMUSG00000033196.18	1.95006544	0.00454354	0.03233878	0.11067296	0.73937927	0.87628937	Myh2
ENSMUSG00000033207.8	74.7073051	0.02169209	0.00319026	44.6420466	2.37E-11	4.15E-10	Mamdc2
ENSMUSG00000033208.8	9.30646488	-0.0127098	0.0142499	0.93705531	0.33303629	0.51995724	S100b
ENSMUSG00000033209.18	61.6097751	0.00680975	0.00567317	1.32566975	0.24957704	0.4210711	Ttc28
ENSMUSG00000033210.17	0.94132568	-0.0211353	0.13908934	0.03481432	0.85198528	0.94872408	Slc9c1
ENSMUSG00000033213.17	2.31381831	0.07720827	0.01609047	21.4168839	3.70E-06	2.88E-05	AA467197
ENSMUSG00000033214.11	2.59906978	0.01006311	0.02221887	0.06056812	0.80560025	0.91898276	Slitrk5
ENSMUSG00000033216.10	497.894549	0.00191152	0.00256173	0.55003449	0.4583036	0.65008111	Eefsec
ENSMUSG00000033220.8	276.590123	0.06885502	0.00760799	70.832765	3.89E-17	1.44E-15	Rac2
ENSMUSG00000033222.17	92.1655879	0.00039805	0.00373063	0.01149984	0.91460072	0.98330625	Ttf2
ENSMUSG00000033227.8	2.01770384	0.03263863	0.01336633	5.95188602	0.01470155	0.04417207	Wnt6
ENSMUSG00000033228.9	1229.23824	0.00263373	0.00237077	1.22214842	0.26893944	0.44533845	Scaf11
ENSMUSG00000033233.18	19.1070318	-0.0065034	0.00482	1.7925358	0.18061751	0.33137282	Trim45
ENSMUSG00000033237.20	721.223272	0.00187767	0.00391911	0.22472375	0.63546399	0.80304617	Arid2
ENSMUSG00000033249.11	95.3875081	-0.0002675	0.00346617	0.00601847	0.9381631	0.99602975	Hsf4
ENSMUSG00000033253.19	263.699889	-0.0080539	0.00407663	3.85070304	0.04972514	0.12005499	Szt2
ENSMUSG00000033256.15	123.213161	-0.0033313	0.00404115	0.68933449	0.40639084	0.59806166	Shf
ENSMUSG00000033257.15	411.970202	-0.0110328	0.00331637	10.8785287	0.00097285	0.00428893	Ttll4
ENSMUSG00000033268.9	0.83180825	-0.0307406	0.03640418	0.80721633	0.36894453	0.55950142	Duox1
ENSMUSG00000033272.13	290.462691	-0.004131	0.00374276	1.212027	0.27093105	0.44786794	Slc35a4
ENSMUSG00000033276.19	15.9676573	-0.0011214	0.00700109	0.02428032	0.87617376	0.96183824	Stk36
ENSMUSG00000033278.11	102.353876	0.01441069	0.00498161	8.05076246	0.00454845	0.01629648	Ptprm
ENSMUSG00000033282.15	106.902913	0.01267739	0.00657979	3.69256775	0.05465541	0.12985823	Rpgrip1l
ENSMUSG00000033285.16	334.723234	-0.0040148	0.00344758	1.33258282	0.24834625	0.41974165	Wdr3
ENSMUSG00000033287.18	79.4223477	0.0145297	0.00494998	8.33950591	0.00387921	0.01423606	Kctd17
ENSMUSG00000033294.12	175.691572	-0.0131232	0.00270146	23.7231607	1.11E-06	9.50E-06	Noc4l
ENSMUSG00000033295.15	3160.80598	-0.0113885	0.00524787	4.63703958	0.03128893	0.08233636	Ptprf

ENSMUSG00000033306.16	1515.25779	0.00610756	0.00393516	2.39654442	0.1216036	0.24478472	Lpp
ENSMUSG00000033307.8	1556.66902	-0.0012278	0.00320239	0.15063994	0.69792456	0.84851798	Mif
ENSMUSG00000033308.17	8823.97875	0.00666456	0.00402189	2.65499814	0.10322488	0.21520923	Dpyd
ENSMUSG00000033313.12	53.777946	0.0063459	0.00279091	5.2168079	0.02236958	0.06246849	Fbxl8
ENSMUSG00000033316.15	1.59136796	0.04676184	0.01418892	10.1646835	0.00143156	0.00601655	Galnt9
ENSMUSG00000033318.8	1159.29825	0.00311827	0.00456391	0.46703691	0.49435349	0.68186351	Gstt2
ENSMUSG00000033319.6	329.255001	0.00332163	0.00234281	1.96570713	0.16090426	0.30280763	Fem1c
ENSMUSG00000033323.15	178.162591	-0.0021439	0.0026638	0.64003641	0.42369761	0.61543315	Ctdp1
ENSMUSG00000033326.16	487.572524	0.00423578	0.00248992	2.78120667	0.09537629	0.20262521	Kdm4a
ENSMUSG00000033327.19	295.303119	0.01522412	0.00429275	12.5499295	0.00039622	0.00194856	Tnxb
ENSMUSG00000033335.19	902.060454	-0.0059872	0.00202582	8.74169363	0.00311016	0.01177088	Dnm2
ENSMUSG00000033342.16	11.9156329	0.03003018	0.00657815	19.9581883	7.92E-06	5.72E-05	Plppr5
ENSMUSG00000033350.8	66.0554101	0.01913286	0.00420901	20.2279886	6.87E-06	5.05E-05	Chst2
ENSMUSG00000033352.12	723.334425	0.0010577	0.00210433	0.25123293	0.61620827	0.78761655	Map2k4
ENSMUSG00000033355.7	346.885278	0.03395332	0.00417027	66.7613715	3.06E-16	9.98E-15	Rtp4
ENSMUSG00000033356.4	31.3981117	0.00310494	0.00422171	0.52366779	0.46928129	0.66037041	Pus7l
ENSMUSG00000033361.14	28.2395017	0.01009214	0.00658702	2.25295172	0.13335981	0.26290742	Prrg3
ENSMUSG00000033364.14	194.076882	0.00626811	0.00433317	2.00482701	0.15679918	0.297165	Usp37
ENSMUSG00000033365.15	243.336697	-0.0158922	0.00429625	13.8098696	0.00020227	0.00107741	Ipo13
ENSMUSG00000033373.17	61.1596967	0.00337316	0.00361584	0.85931848	0.35392948	0.54262745	Fntb
ENSMUSG00000033377.15	1403.10088	-0.0013506	0.00378144	0.1243864	0.72432493	0.86654465	Palmd
ENSMUSG00000033379.14	1748.749	-0.0029412	0.00423411	0.48130656	0.48783109	0.67614645	Atp6v0b
ENSMUSG00000033382.16	830.922618	-0.0086995	0.00259568	11.0828694	0.00087129	0.0038862	Trappc8
ENSMUSG00000033386.11	618.060809	-0.0014484	0.00347251	0.17582956	0.67498202	0.83168561	Frrs1
ENSMUSG00000033389.17	21.5278824	-0.030174	0.00685466	20.4661817	6.07E-06	4.52E-05	Arhgap44
ENSMUSG00000033392.17	247.972587	0.01442163	0.00298477	23.0613423	1.57E-06	1.31E-05	Clasp2
ENSMUSG00000033396.14	325.550667	-0.0006971	0.00284539	0.05980094	0.80681084	0.91974554	Spg11
ENSMUSG00000033400.15	1163.90534	0.00943955	0.00297096	9.90219675	0.00165082	0.00681297	Agl
ENSMUSG00000033405.5	45.0248467	-0.0023815	0.00372529	0.39735465	0.5284586	0.71310236	Nudt15
ENSMUSG00000033411.17	206.250129	0.01010022	0.00330876	9.18729975	0.002437	0.00956073	Ctdspl2
ENSMUSG00000033416.16	3197.93169	-0.0146057	0.00281399	26.5858042	2.52E-07	2.43E-06	Gucd1
ENSMUSG00000033417.17	1234.44801	-0.0029002	0.00245493	1.37986546	0.24012407	0.40972048	Cacul1
ENSMUSG00000033419.17	18.7792658	0.02695902	0.01505645	3.4121475	0.06471817	0.14868915	Snap91
ENSMUSG00000033420.13	41.8467266	0.01618233	0.00503243	10.0918005	0.00148931	0.00623478	Antxr1
ENSMUSG00000033423.17	365.838254	-0.001616	0.00271507	0.35553691	0.55099543	0.73347885	Eri3
ENSMUSG00000033427.15	5288.589	-0.0054188	0.00455897	1.40478453	0.23592412	0.40466194	Upb1
ENSMUSG00000033429.11	915.060108	-0.0106541	0.00327086	10.6496469	0.00110092	0.0047776	Mcee
ENSMUSG00000033430.11	285.835589	-0.0057858	0.00173653	11.0259477	0.00089845	0.00399151	Terf2ip
ENSMUSG00000033434.16	155.312942	-0.006252	0.00310822	4.0303951	0.04468747	0.11002075	Gtpbp6
ENSMUSG00000033436.14	59.5712171	0.01820568	0.00395889	20.7807685	5.15E-06	3.89E-05	Armcx2
ENSMUSG00000033439.13	90.3126324	0.00153923	0.00339738	0.20196962	0.6531357	0.81489942	Trmt13
ENSMUSG00000033444.15	413.766779	0.00642626	0.00258419	6.14797955	0.01315623	0.04013229	Specc1l
ENSMUSG00000033446.8	372.521147	-0.0017473	0.00227053	0.58272556	0.44524597	0.63754076	Lpar6
ENSMUSG00000033450.9	18.1130396	0.07050908	0.00744784	79.8751325	3.99E-19	1.84E-17	Tagap
ENSMUSG00000033453.9	29.2268316	0.04368698	0.00612737	48.1961546	3.86E-12	7.52E-11	Adamts15
ENSMUSG00000033454.7	267.779838	0.00629546	0.00209726	8.82992318	0.00296331	0.01130034	Zbtb1
ENSMUSG00000033458.18	71.9571454	-0.0003613	0.00377291	0.00901335	0.92436354	0.98879942	Fan1
ENSMUSG00000033460.15	46.0625796	0.00409545	0.00368028	1.2198207	0.26939584	0.44592395	Armcx1
ENSMUSG00000033467.13	95.0765684	0.01117445	0.00324852	11.4802215	0.00070341	0.0032246	Crlf2
ENSMUSG00000033470.13	13.7785748	0.02632997	0.00825199	9.30304072	0.00228774	0.00906715	Cysltr2
ENSMUSG00000033475.16	1043.87094	-0.0015787	0.00366016	0.18704861	0.66538447	0.82485852	Tomm6
ENSMUSG00000033478.7	897.461493	-0.0002059	0.00233351	0.00769333	0.93010587	0.99177898	Fhip2a
ENSMUSG00000033486.15	50.328505	-0.010555	0.00412904	6.37181021	0.01159473	0.03606399	Catsper2
ENSMUSG00000033487.15	1470.92883	-0.0031057	0.00339173	0.83613611	0.36050419	0.54984815	Fndc3a
ENSMUSG00000033488.12	337.511408	0.00299726	0.0032017	0.86348655	0.35276484	0.54140497	Cryzl2
ENSMUSG00000033491.15	1.49314206	0.02875741	0.01522345	3.72427371	0.05362695	0.12773081	Prss35
ENSMUSG00000033498.15	1.61823801	0.09127447	0.01589574	30.0720118	4.16E-08	4.56E-07	Strc
ENSMUSG00000033499.15	1684.76221	-0.0069839	0.00249489	7.80308019	0.00521572	0.01834448	Larp4b
ENSMUSG00000033502.16	45.9088139	0.03358212	0.00415213	63.4797285	1.62E-15	4.87E-14	Cdc14a
ENSMUSG00000033508.9	2.60755246	0.08342833	0.01321658	33.3511028	7.69E-09	9.44E-08	Asprv1
ENSMUSG00000033510.15	5.60881033	-0.0012776	0.03126133	-0.0233846	1	1	Otud7a

ENSMUSG00000033526.17	86.4725056	-0.007304	0.00380163	3.62729168	0.05683924	0.13400743	Ppip5k1
ENSMUSG00000033530.9	341.498204	-0.0092722	0.00286734	10.3365734	0.0013042	0.0055405	Ttc7b
ENSMUSG00000033533.15	5505.96603	-0.0186028	0.00481536	14.7512479	0.00012267	0.00068755	Acsm1
ENSMUSG00000033538.14	47.7168795	0.04864128	0.00609168	58.4192696	2.12E-14	5.57E-13	Casp4
ENSMUSG00000033540.19	122.406873	0.00217147	0.0037724	0.32987066	0.56573526	0.74590149	Idua
ENSMUSG00000033542.14	513.148892	0.00567644	0.00221763	6.4797749	0.01091088	0.03433918	Arhgef5
ENSMUSG00000033543.18	939.371606	-0.0052236	0.00338145	2.39249948	0.12191856	0.24529311	Gtf2a2
ENSMUSG00000033544.7	4.89105413	0.01066888	0.01003818	1.10525605	0.29311551	0.47521462	Angptl1
ENSMUSG00000033545.15	585.402881	-0.0106394	0.00349265	9.28995519	0.00230414	0.00911683	Znrf1
ENSMUSG00000033554.18	152.023344	-0.0037902	0.00235088	2.62557383	0.10515448	0.21825985	Dph5
ENSMUSG00000033557.18	470.118789	0.00139157	0.00239719	0.33407916	0.56326695	0.74389463	Fam20b
ENSMUSG00000033565.20	568.268882	0.01214536	0.0038403	9.92149465	0.0016336	0.00675538	Rbfox2
ENSMUSG00000033569.18	7.99091627	0.02241213	0.01762707	1.83453711	0.17559258	0.32418995	Adgrb3
ENSMUSG00000033576.13	1.40931424	0.0362583	0.01945023	2.66928745	0.10230182	0.21379502	Apol6
ENSMUSG00000033577.19	3704.29004	-0.0166009	0.00428326	14.9593612	0.00010985	0.00062163	Myo6
ENSMUSG00000033578.8	5.63128292	-0.0092975	0.01325883	0.57483362	0.44834453	0.64021892	Tmem35a
ENSMUSG00000033579.17	2.6404041	0.05889352	0.0141844	18.2758544	1.91E-05	0.00012835	Fa2h
ENSMUSG00000033581.18	47.4579571	0.01050411	0.00685454	2.14009538	0.14349297	0.27805052	Igf2bp2
ENSMUSG00000033585.6	21.9160031	0.01854719	0.0051049	13.0314038	0.00030631	0.00155369	Ndn
ENSMUSG00000033589.5	124.626809	0.00164273	0.00363603	0.2002095	0.6545518	0.81627763	Reep4
ENSMUSG00000033590.9	8.47438848	0.00136098	0.00864684	0.02758558	0.86808684	0.95783572	Myo5c
ENSMUSG00000033594.12	428.612008	-0.0166086	0.00423065	15.9587393	6.47E-05	0.00038715	Spata2l
ENSMUSG00000033595.8	4.82519245	0.0035989	0.01030646	0.1039256	0.74716867	0.88133457	Lgi3
ENSMUSG00000033596.6	429.123585	5.43E-05	0.00212216	0.00064222	0.97978209	1	Rfwd3
ENSMUSG00000033597.10	9.11046672	0.02185501	0.01016993	4.46492822	0.03459754	0.08948543	Caskin1
ENSMUSG00000033610.17	4422.47193	-0.00737	0.00327082	4.89282474	0.02696853	0.07286971	Pank1
ENSMUSG00000033615.10	11.7910321	0.00824768	0.021455	0.17693716	0.67401872	0.8313673	Cplx1
ENSMUSG00000033618.8	340.254139	-0.0036066	0.00508847	0.48279159	0.48716056	0.67565382	Map3k13
ENSMUSG00000033623.14	543.440072	-0.0060692	0.00252015	5.7001154	0.0169638	0.04968436	Pcgf3
ENSMUSG00000033624.11	322.515615	-0.0108833	0.00525462	4.2023009	0.04036917	0.10140013	Pdpr
ENSMUSG00000033628.16	330.726675	0.00309976	0.00187488	2.70000173	0.10034814	0.21073913	Pik3c3
ENSMUSG00000033629.7	3130.50274	-0.0030746	0.00242454	1.60003357	0.20589845	0.36644686	Hacd3
ENSMUSG00000033632.18	57.9316773	0.01463192	0.00380002	14.8496767	0.00011643	0.0006557	AW554918
ENSMUSG00000033634.8	19200.2917	0.00586998	0.00515076	1.26771449	0.26019588	0.43413837	Nat8f2
ENSMUSG00000033644.6	5.55979107	0.05246418	0.01018333	24.8833163	6.09E-07	5.46E-06	Piwil2
ENSMUSG00000033653.20	163.80641	0.00962546	0.00374852	6.38237177	0.01152593	0.03588787	Vps8
ENSMUSG00000033658.17	404.114885	-0.0111225	0.00249969	19.4852138	1.01E-05	7.19E-05	Ddx19b
ENSMUSG00000033669.9	45.919154	0.00917889	0.00419169	4.76934451	0.02897085	0.07718362	Zfp7
ENSMUSG00000033671.19	764.123779	-0.0005361	0.00388516	0.01872712	0.89115167	0.97066146	Cep350
ENSMUSG00000033676.14	44.9386085	0.02041046	0.00710902	7.18599691	0.00734747	0.02460338	Gabrb3
ENSMUSG00000033684.15	6194.81434	-0.0098757	0.00361734	7.37788443	0.00660308	0.02248307	Qsox1
ENSMUSG00000033685.14	699.99813	0.0351994	0.00482776	51.8052025	6.13E-13	1.32E-11	Ucp2
ENSMUSG00000033688.11	8487.02661	-0.009793	0.00435057	5.03506237	0.02483919	0.06808335	Inhca
ENSMUSG00000033697.17	93.8899929	0.01006839	0.00637579	2.43461569	0.11868298	0.24017395	Arhgap39
ENSMUSG00000033701.14	392.417657	-0.0038976	0.00296356	1.72571112	0.18895955	0.34296268	Acbd6
ENSMUSG00000033703.10	159.066934	-0.0040936	0.00239402	2.96318425	0.08518004	0.18526894	Fcsk
ENSMUSG00000033705.18	145.439494	0.02297874	0.00419198	30.1327013	4.03E-08	4.43E-07	Stard9
ENSMUSG00000033706.14	209.97341	0.00594598	0.00251093	5.56424138	0.01833095	0.05295858	Smyd5
ENSMUSG00000033707.10	5.20600737	-0.0157733	0.0107979	2.09543268	0.14773988	0.28417818	Lrrc24
ENSMUSG00000033712.7	322.266632	0.00402075	0.00208376	3.74453745	0.05298039	0.12640051	Ccar2
ENSMUSG00000033713.13	1295.96973	-0.0057628	0.00438417	1.7022098	0.19199924	0.34701104	Foxn3
ENSMUSG00000033715.16	7779.14488	-0.0191726	0.00407342	21.7187142	3.16E-06	2.49E-05	Akr1c14
ENSMUSG00000033717.7	5.48547155	0.01224423	0.01902295	0.63465058	0.42565456	0.61728286	Adra2a
ENSMUSG00000033720.13	470.82842	-0.0023262	0.00283261	0.66909207	0.41336833	0.60485491	Sfxn5
ENSMUSG00000033721.17	34.217191	0.01977113	0.00571591	10.9674089	0.00092728	0.00410644	Vav3
ENSMUSG00000033722.10	13.0147457	0.00749994	0.00817454	0.80220169	0.37043591	0.56104164	BC034090
ENSMUSG00000033726.9	2.89609125	0.0121543	0.05723575	0.18678824	0.66560329	0.82501066	Emx1
ENSMUSG00000033728.15	188.730276	-0.0087991	0.0028546	9.48420014	0.00207249	0.00833448	Lrrc14
ENSMUSG00000033730.5	16.9731834	0.0820455	0.01260663	37.3767386	9.74E-10	1.35E-08	Egr3
ENSMUSG00000033732.11	663.170483	-0.0003136	0.0023863	0.01701498	0.89621716	0.97354151	Sf3b3
ENSMUSG00000033735.10	1468.4755	-0.0023879	0.00278335	0.74918656	0.38673389	0.57824254	Spr

ENSMUSG00000033739.9	82.37351	0.00466529	0.0040139	1.35920706	0.24367495	0.41419122	Fkbpl
ENSMUSG00000033740.18	2.10273313	0.08910588	0.02215991	16.6390876	4.52E-05	0.00028049	St18
ENSMUSG00000033751.6	717.341694	-0.0113724	0.00331722	11.9217124	0.00055483	0.00262041	Gadd45gip1
ENSMUSG00000033752.8	88.7061924	-0.0019423	0.00365588	0.28256255	0.59502653	0.77004703	Mnd1
ENSMUSG00000033760.18	313.427353	-0.0006006	0.00254891	0.0549136	0.81472375	0.9242742	Rbm4b
ENSMUSG00000033762.9	33.5521882	0.00674433	0.00548626	1.51041819	0.21907529	0.38358739	Recql4
ENSMUSG00000033763.15	73.7299182	0.00279424	0.00471065	0.34564568	0.55658842	0.73867288	Mtss2
ENSMUSG00000033767.15	495.149095	-0.0025712	0.00419702	0.37500343	0.54028952	0.72408108	Tmem131l
ENSMUSG00000033768.18	51.2712634	0.02630895	0.00612884	17.3620095	3.09E-05	0.00019768	Nrxn2
ENSMUSG00000033769.17	170.724108	0.00465002	0.00315318	2.11586914	0.14577927	0.28139372	Exoc6b
ENSMUSG00000033773.15	206.528544	-0.0021523	0.00200906	1.14605806	0.28437586	0.46442431	Rpap2
ENSMUSG00000033777.5	77.6467371	0.07837589	0.00677067	119.070667	1.01E-27	1.40E-25	Tlr13
ENSMUSG00000033781.8	831.491323	-0.0084216	0.00280453	8.86481875	0.00290719	0.0111223	Asb13
ENSMUSG00000033788.16	121.131559	-0.0168784	0.00617721	7.5291577	0.00607083	0.02087555	Dysf
ENSMUSG00000033790.12	127.652971	0.00454694	0.00364152	1.525334	0.21681408	0.38063096	Tubgcp5
ENSMUSG00000033792.13	87.2679672	0.01363134	0.00534483	6.27883414	0.01221885	0.03769702	Atp7a
ENSMUSG00000033793.13	1144.76113	-0.0020001	0.00402148	0.24330916	0.62182621	0.79183636	Atp6v1h
ENSMUSG00000033799.11	372.258685	0.001959	0.00515499	0.137927	0.71035062	0.85686747	Tasor2
ENSMUSG00000033805.13	2.25547169	0.0197675	0.01281482	2.34470802	0.12570911	0.25118393	Ephx4
ENSMUSG00000033808.17	199.434919	0.01046923	0.00253086	16.5782269	4.67E-05	0.0002892	Tmem87a
ENSMUSG00000033809.16	190.802249	-0.0127041	0.00239144	28.6388816	8.72E-08	9.15E-07	Alg3
ENSMUSG00000033813.16	1748.08305	-0.003722	0.00201715	3.35660956	0.06693575	0.15269818	Tcea1
ENSMUSG00000033819.17	416.870412	-0.0033064	0.00258357	1.67202701	0.19598725	0.35260715	Ppp1r16a
ENSMUSG00000033825.12	2.23450495	0.04078207	0.02256307	2.39356562	0.12183546	0.24516774	Tpsb2
ENSMUSG00000033826.11	19.9711857	0.00264744	0.01009622	0.06067156	0.80543765	0.91892938	Dnah8
ENSMUSG00000033831.6	90420.925	-0.0034062	0.00631972	0.28516609	0.593335	0.76876789	Fgb
ENSMUSG00000033845.14	1390.50305	-0.0092482	0.00327372	8.01601743	0.00463654	0.01655928	Mrpl15
ENSMUSG00000033847.16	2.91376016	0.03890291	0.0115186	10.5318671	0.00117334	0.00505282	Pla2g4c
ENSMUSG00000033849.4	29.2665546	0.04989738	0.01168654	17.9975099	2.21E-05	0.00014642	B3galt2
ENSMUSG00000033852.17	20.0006068	-0.0206035	0.00955966	4.49079974	0.03407773	0.08835412	Gm28042
ENSMUSG00000033854.11	28.5082162	-0.0143262	0.00679389	4.21907107	0.03997204	0.10055253	Kcnk10
ENSMUSG00000033855.16	106.075615	-0.004511	0.0056141	0.62282613	0.42999901	0.62144343	Ston1
ENSMUSG00000033857.13	115.686718	0.00995918	0.0042841	5.34210062	0.02081637	0.05888403	Engase
ENSMUSG00000033860.14	70342.6293	-0.0055183	0.00639467	0.72754177	0.39368116	0.58510347	Fgg
ENSMUSG00000033862.8	346.086595	-0.0067083	0.00292042	5.40208856	0.02011267	0.05722285	Cdk10
ENSMUSG00000033863.3	2280.54994	-0.0122416	0.00350909	11.9617851	0.00054303	0.00257413	Klf9
ENSMUSG00000033871.15	107.560214	-0.0090853	0.00537275	2.87892975	0.08974565	0.19313535	Ppargc1b
ENSMUSG00000033880.12	1436.53822	0.01715538	0.00341488	25.9783404	3.45E-07	3.25E-06	Lgals3bp
ENSMUSG00000033882.16	5.54547191	0.02201465	0.00739212	8.5566938	0.00344254	0.01284743	Rbm46
ENSMUSG00000033883.12	117.633081	0.0128209	0.00266441	22.6749168	1.92E-06	1.58E-05	Zfp267
ENSMUSG00000033885.16	459.976605	-0.0028658	0.00190391	2.25570269	0.13312302	0.26261536	Pxk
ENSMUSG00000033898.18	13818.8555	-0.0126444	0.00495226	6.44319816	0.0111378	0.03493337	Cfhr2
ENSMUSG00000033900.14	46.1826869	0.01113524	0.00757892	1.92663853	0.16512668	0.30909586	Map9
ENSMUSG00000033902.13	100.434539	-0.0042351	0.00377042	1.24500684	0.26450829	0.43954588	Mapkbp1
ENSMUSG00000033904.17	141.377018	-0.0057863	0.00351962	2.57808317	0.1083528	0.22350964	Ccp110
ENSMUSG00000033906.6	5.4103825	0.04139963	0.00920802	19.9120805	8.11E-06	5.85E-05	Zdhhc15
ENSMUSG00000033909.18	357.259773	-0.0061406	0.00380025	2.6332682	0.10464611	0.21742276	Usp36
ENSMUSG00000033910.14	186.212519	0.01556344	0.0033229	21.549787	3.45E-06	2.70E-05	Gucy1a1
ENSMUSG00000033916.6	1672.5608	-0.0013399	0.00291057	0.21258342	0.64475004	0.81004476	Chmp2a
ENSMUSG00000033917.16	841.610532	-0.0062451	0.00293807	4.49301016	0.0340337	0.08827173	Gde1
ENSMUSG00000033918.17	515.074212	-0.0060554	0.00294877	4.19955556	0.04043458	0.10153198	Parl
ENSMUSG00000033931.10	213.656993	-0.0010986	0.00285578	0.14633981	0.702058	0.85129144	Rbm34
ENSMUSG00000033933.14	293.959263	-0.0076706	0.00429753	3.16613039	0.07518037	0.16772648	Vhl
ENSMUSG00000033938.6	1300.39791	-0.0133691	0.00409907	10.8222667	0.00100287	0.00440641	Ndufb7
ENSMUSG00000033940.9	1024.16289	0.00265555	0.00319943	0.68811977	0.40680465	0.59840929	Brk1
ENSMUSG00000033943.16	712.710126	-0.0035637	0.00464945	0.57997048	0.44632389	0.63834791	Mga
ENSMUSG00000033948.4	58.7817791	-0.007908	0.00378156	4.35010078	0.03700676	0.09436123	Zswim5
ENSMUSG00000033949.13	12.4226866	0.03249605	0.00853568	13.5657493	0.00023035	0.00120815	Trim36
ENSMUSG00000033952.15	88.4837714	0.01111399	0.0088423	1.49124734	0.22202303	0.38723916	Aspm
ENSMUSG00000033953.11	983.066698	0.01133407	0.00301527	13.961729	0.00018657	0.00100329	Ppp3r1
ENSMUSG00000033955.14	890.098408	-0.0036333	0.00357496	1.03915436	0.30801823	0.49296549	Tnks1bp1

ENSMUSG00000033960.7	189.1165	-0.0039267	0.00366037	1.14692057	0.28419471	0.46432644	Jcad
ENSMUSG00000033961.17	73.324715	0.0023117	0.0027601	0.69659136	0.40393142	0.59555708	Zfp446
ENSMUSG00000033964.13	436.180121	-0.0044839	0.0033284	1.78502538	0.18153345	0.33262642	Zbtb41
ENSMUSG00000033965.11	2585.5027	-0.0088517	0.00404495	4.76993852	0.02896086	0.07716571	Slc16a2
ENSMUSG00000033966.9	5.63948993	0.00795186	0.00667387	1.39793622	0.23706942	0.40601337	Cdkl4
ENSMUSG00000033967.5	10.338653	0.01473736	0.008666	2.65945488	0.10293601	0.21477784	Rnf225
ENSMUSG00000033970.16	114.120495	0.00447605	0.00371292	1.45812318	0.22722898	0.39355432	Rfc3
ENSMUSG00000033972.10	207.827622	-0.0012219	0.00219263	0.30628293	0.57997014	0.75789105	Zfp944
ENSMUSG00000033981.15	67.1825784	0.02115268	0.01415762	2.31080004	0.12847791	0.25561197	Gria2
ENSMUSG00000033983.15	80.497756	-0.0018879	0.00263885	0.5089561	0.4755911	0.66595178	Coil
ENSMUSG00000033985.18	282.670881	-0.0087211	0.00218051	16.0827036	6.06E-05	0.00036465	Tesk2
ENSMUSG00000033987.18	7.56636011	-0.0386926	0.01185387	10.6106628	0.00112437	0.00486775	Dnah17
ENSMUSG00000033991.10	339.045053	-0.0053071	0.00263439	4.02208193	0.04490824	0.11048169	Skic3
ENSMUSG00000033998.10	11.6898281	-0.0044044	0.00774633	0.29601379	0.58639228	0.76323869	Kcnk1
ENSMUSG00000034006.18	1454.47758	-0.010314	0.00368258	7.81365082	0.0051853	0.0182511	Slc66a2
ENSMUSG00000034007.11	203.192827	0.00540359	0.00393226	1.85786873	0.17287098	0.3203689	Scaper
ENSMUSG00000034009.15	4.70513555	0.01460651	0.01815917	0.96816907	0.32513723	0.51188126	Rxfp1
ENSMUSG00000034021.16	438.450732	-0.0025966	0.00232659	1.22546241	0.26829131	0.44451942	Pds5b
ENSMUSG00000034022.9	761.931291	-0.0130314	0.00363437	12.9418246	0.00032132	0.00161834	Cpsf1
ENSMUSG00000034023.17	13.4802901	0.02586141	0.00979103	6.37543126	0.01157109	0.03599997	Fancd2
ENSMUSG00000034024.9	1699.60965	-0.0076033	0.00152218	24.7343925	6.58E-07	5.85E-06	Cct2
ENSMUSG00000034028.17	20.8691655	0.02789379	0.00895665	8.13804733	0.00433459	0.01567311	Cd226
ENSMUSG00000034032.16	170.896285	-0.001548	0.00224061	0.47803029	0.48931587	0.67729613	Rpap1
ENSMUSG00000034035.19	42.869794	-0.0034526	0.00350566	0.98259897	0.321558	0.50776007	Ccdc17
ENSMUSG00000034037.15	143.752205	-0.0022051	0.00303336	0.52730586	0.46774174	0.65887223	Fgd5
ENSMUSG00000034040.18	5.16396709	0.02199993	0.01318753	2.53236865	0.11153268	0.22864654	Galnt17
ENSMUSG00000034041.6	18.6691476	0.04663977	0.00630307	50.2553795	1.35E-12	2.78E-11	Lyl1
ENSMUSG00000034042.17	1035.09908	-0.0013062	0.00187907	0.48208592	0.487479	0.6758173	Gpbp1l1
ENSMUSG00000034055.17	165.079057	-0.0083532	0.00268636	9.81668684	0.00172936	0.00710603	Phka1
ENSMUSG00000034059.15	6.91623837	0.06651996	0.01385244	19.4313721	1.04E-05	7.37E-05	Ypel4
ENSMUSG00000034063.10	9.5870323	0.00470712	0.01026167	0.20379469	0.65167516	0.81398137	4930590J08Rik
ENSMUSG00000034064.15	243.772889	0.00432412	0.00204179	4.39950157	0.03594944	0.09223359	Poglut1
ENSMUSG00000034066.14	375.562471	-0.0028407	0.00243434	1.33222812	0.24840922	0.41978791	Farp2
ENSMUSG00000034071.16	22.4644222	0.01159176	0.00482064	5.57423458	0.01822663	0.05274011	Zfp551
ENSMUSG00000034075.11	1160.98505	-0.0026054	0.00257347	1.02091268	0.31230262	0.49753256	Zdhhc5
ENSMUSG00000034083.17	264.667536	-0.005214	0.00338557	2.31651272	0.12800672	0.25500311	Ccdc174
ENSMUSG00000034088.16	8038.32305	-0.0043016	0.00289411	2.17607448	0.14017159	0.27316261	Hdlbp
ENSMUSG00000034098.15	3.37682995	0.02843467	0.01868269	2.95609022	0.08555459	0.1858779	Fstl5
ENSMUSG00000034101.15	2366.07473	-0.0023971	0.00329069	0.5308318	0.46625736	0.6573702	Ctnnd1
ENSMUSG00000034105.10	157.773867	-0.0029555	0.00248761	1.4090709	0.23521069	0.40368852	Meak7
ENSMUSG00000034108.7	2412.51648	-0.0176659	0.00372244	22.5109528	2.09E-06	1.71E-05	Ccs
ENSMUSG00000034109.17	1477.9614	0.00314859	0.00289084	1.16619591	0.28018426	0.45965263	Golim4
ENSMUSG00000034110.9	80.6955754	0.00719964	0.00368488	3.80711418	0.05103531	0.12260326	Kctd7
ENSMUSG00000034111.7	83.4865373	0.00482218	0.00365375	1.69129373	0.19343051	0.34890273	Tmed8
ENSMUSG00000034116.18	107.100459	0.06587035	0.00606315	107.829038	2.93E-25	2.84E-23	Vav1
ENSMUSG00000034118.16	366.277135	-0.0142757	0.00251715	32.6341696	1.11E-08	1.33E-07	Tpst1
ENSMUSG00000034120.19	2260.39436	0.00100987	0.00340051	0.08898824	0.76546736	0.8936894	Srsf2
ENSMUSG00000034121.13	32.9297483	0.00056463	0.00416053	0.01760178	0.89445302	0.97287826	Mks1
ENSMUSG00000034126.6	369.659238	-0.0114747	0.00210441	29.7442342	4.93E-08	5.36E-07	Pomt2
ENSMUSG00000034127.16	36.842736	0.03890791	0.00722363	27.7036732	1.41E-07	1.44E-06	Tspan8
ENSMUSG00000034135.16	1100.67086	-0.006522	0.00490142	1.74968407	0.18591645	0.33876897	Sik3
ENSMUSG00000034145.15	5.1781756	0.06036599	0.01489726	15.5882585	7.87E-05	0.00046085	Tmem63c
ENSMUSG00000034151.14	6.69933405	-0.0169957	0.05966104	0.14995958	0.69857398	0.84908146	Zbbx
ENSMUSG00000034152.7	538.502854	-0.0020668	0.00286417	0.51145652	0.47450902	0.66518691	Exoc3
ENSMUSG00000034154.16	322.529754	-0.0008288	0.00346463	0.05683046	0.81157753	0.92249964	Ino80
ENSMUSG00000034156.17	22.1405981	0.0293359	0.01050047	6.25819042	0.01236204	0.03807213	Tspoap1
ENSMUSG00000034157.13	686.173083	-0.0150914	0.00262274	32.1730464	1.41E-08	1.67E-07	Cipc
ENSMUSG00000034158.10	1815.71764	-0.0031035	0.00286435	1.13438781	0.28684134	0.46771102	Lrrc58
ENSMUSG00000034159.13	2.43448826	0.02920539	0.01012335	8.41749117	0.00371628	0.01371495	Mab21l4
ENSMUSG00000034160.14	1498.076	0.00727477	0.00303562	5.7689478	0.0163118	0.04807323	Ogt
ENSMUSG00000034161.9	4.17939909	-0.0120125	0.01266602	0.96671156	0.32550168	0.51227484	Scx

ENSMUSG00000034163.11	791.097004	-0.0063247	0.00315409	3.94207136	0.04709276	0.11499428	Zfc3h1
ENSMUSG00000034164.18	4.21162715	-0.0008037	0.00770465	0.00960025	0.9219475	0.98764835	Emid1
ENSMUSG00000034165.17	592.57306	-0.0014411	0.00262857	0.29940644	0.58425477	0.76154922	Ccnd3
ENSMUSG00000034168.8	442.711086	-0.0023924	0.0026636	0.78193541	0.37654988	0.56769129	Irf2bpl
ENSMUSG00000034171.14	2631.27494	-0.0074898	0.0039206	3.62751075	0.05683176	0.13400743	Faah
ENSMUSG00000034173.14	128.297176	-0.0199742	0.00567509	12.5910263	0.0003876	0.00190936	Zbed5
ENSMUSG00000034175.14	209.32446	-0.009652	0.00269844	12.9221921	0.00032471	0.00163313	Rhbdd3
ENSMUSG00000034177.16	380.13411	0.00750251	0.00492799	2.28750293	0.1304196	0.25846196	Rnf43
ENSMUSG00000034187.19	330.120056	0.01159362	0.00354776	10.5829714	0.00114134	0.00493215	Nsf
ENSMUSG00000034189.6	199.921083	0.01232422	0.00316453	14.7684481	0.00012155	0.00068212	Hsdl1
ENSMUSG00000034190.10	720.936903	-0.0150438	0.0017306	76.169674	2.60E-18	1.10E-16	Chmp7
ENSMUSG00000034192.6	122.594302	0.00083461	0.00471345	0.03037261	0.86164748	0.95394374	Lsm3
ENSMUSG00000034194.19	164.535627	-0.0044006	0.00227093	3.82166321	0.05059399	0.12174147	R3hcc1
ENSMUSG00000034201.14	346.793746	0.00225018	0.00256925	0.75892118	0.38366635	0.57531658	Gas2l1
ENSMUSG00000034203.9	322.706883	-0.0102877	0.00292119	12.4551444	0.00041684	0.00203554	Chchd4
ENSMUSG00000034205.17	87.6739182	0.00135102	0.00541919	0.06256011	0.80249441	0.91721424	Loxl2
ENSMUSG00000034206.16	32.7684834	0.02497649	0.01173376	3.9445127	0.04702447	0.11485132	Polq
ENSMUSG00000034209.5	1.39212003	-0.0147884	0.01303146	1.26235445	0.2612059	0.43548471	Rasl10a
ENSMUSG00000034210.15	403.978628	0.00534202	0.0021147	6.3167742	0.0119601	0.03702953	Efcab14
ENSMUSG00000034211.15	929.521082	-0.0105019	0.00253358	17.4183294	3.00E-05	0.0001928	Mrps17
ENSMUSG00000034212.15	3.2056099	0.00539244	0.01109693	0.23133159	0.63053823	0.79900471	Ankmy1
ENSMUSG00000034216.13	278.621511	7.42E-05	0.00189349	0.0015166	0.96893543	1	Vps18
ENSMUSG00000034218.17	194.97726	0.00012265	0.00613862	0.00040501	0.98394386	1	Atm
ENSMUSG00000034220.8	257.83892	0.04477734	0.00601584	46.4282836	9.50E-12	1.77E-10	Gpc1
ENSMUSG00000034226.8	2.35471974	0.01944138	0.01741102	1.40740096	0.23548833	0.40407122	Rhov
ENSMUSG00000034227.8	8.31198467	0.01259192	0.00868188	2.13929808	0.14356757	0.27817174	Foxj1
ENSMUSG00000034235.18	96.7177757	0.00069998	0.00342855	0.04115358	0.83924176	0.9400815	Usp54
ENSMUSG00000034239.15	3.55607781	-0.0148967	0.02365904	0.4141785	0.51985625	0.70585052	Lrrc37
ENSMUSG00000034243.18	1919.63875	-0.0011502	0.00445894	0.06666457	0.79625655	0.91389328	Golgb1
ENSMUSG00000034245.11	812.423332	-0.0014537	0.00293482	0.2452273	0.62045591	0.79074712	Hdac11
ENSMUSG00000034247.10	385.648932	0.00349615	0.00240861	2.08016616	0.14922393	0.28637882	Plekhm1
ENSMUSG00000034248.8	802.480765	-0.0017571	0.0033153	0.28103768	0.59602189	0.7709561	Slc25a37
ENSMUSG00000034252.15	902.964264	-0.0043386	0.00198881	4.73428842	0.02956708	0.07853858	Senp6
ENSMUSG00000034254.18	172.545015	0.00517219	0.00371489	1.96200729	0.1612988	0.30345874	Agpat1
ENSMUSG00000034255.19	69.6235955	0.04612873	0.00588451	58.0418831	2.57E-14	6.69E-13	Arhgap27
ENSMUSG00000034258.5	74.5155636	0.04148716	0.00571408	51.0614081	8.95E-13	1.89E-11	Flvcr2
ENSMUSG00000034259.9	301.720302	-0.0082473	0.00287228	8.36890868	0.00381695	0.01403594	Exosc4
ENSMUSG00000034263.14	186.544339	-5.90E-05	0.00158922	0.00138041	0.97036229	1	Ints14
ENSMUSG00000034265.9	72.7426437	0.0079219	0.00355666	4.87956875	0.02717641	0.07330544	Zdhhc14
ENSMUSG00000034266.7	16.1116959	0.07900586	0.0077683	90.5209644	1.83E-21	1.11E-19	Batf
ENSMUSG00000034269.13	758.27446	0.00125964	0.00215421	0.33781765	0.56109163	0.74231026	Setd5
ENSMUSG00000034271.17	36.336659	0.03213872	0.00634529	24.2467815	8.47E-07	7.37E-06	Jdp2
ENSMUSG00000034274.12	269.59407	-0.0014273	0.00220337	0.420067	0.51690362	0.70325846	Thoc5
ENSMUSG00000034275.20	14.1122905	0.0595298	0.01073158	28.2246594	1.08E-07	1.12E-06	Igsf9b
ENSMUSG00000034278.4	47.5844976	0.00461803	0.00362064	1.61493956	0.20379884	0.36357306	Dnajc17
ENSMUSG00000034282.4	11.574399	0.03497952	0.00713197	23.8906781	1.02E-06	8.76E-06	Evpl
ENSMUSG00000034285.16	4472.89794	-0.0071764	0.00419149	2.91978105	0.08749974	0.18931454	Nipsnap1
ENSMUSG00000034290.10	1003.18794	-0.0032183	0.0021781	2.16333266	0.14133785	0.27482231	Nek9
ENSMUSG00000034292.14	128.106138	-0.0051011	0.00256239	4.00927256	0.04525067	0.11120999	Traf3ip1
ENSMUSG00000034295.11	54.2007552	-0.0136342	0.00737321	3.32873044	0.06807941	0.15482797	Fhod3
ENSMUSG00000034297.15	788.197072	-0.0012433	0.0031399	0.15326843	0.69543129	0.84666507	Med13
ENSMUSG00000034300.18	321.408554	-0.0068467	0.00217141	9.93782283	0.00161917	0.00670701	Fam53c
ENSMUSG00000034303.9	38.6348716	0.0053496	0.00556344	0.90445273	0.34159057	0.52942273	Ccdc15
ENSMUSG00000034308.14	2168.80081	-0.0037044	0.00380728	0.93123503	0.33454221	0.5214512	Sdr42e1
ENSMUSG00000034311.4	16.0722498	0.02604087	0.01020536	5.95916261	0.01464099	0.04402939	Kif4
ENSMUSG00000034312.16	700.135439	0.0106539	0.00451655	5.45536892	0.01950828	0.05574075	Iqsec1
ENSMUSG00000034317.15	19.529227	0.04242038	0.0087606	19.9931387	7.77E-06	5.63E-05	Trim59
ENSMUSG00000034320.15	153.452882	0.02289661	0.00307745	54.4681215	1.58E-13	3.67E-12	Slc26a2
ENSMUSG00000034321.15	162.478288	-0.0046643	0.00212456	4.7797675	0.02879601	0.076848	Exosc1
ENSMUSG00000034327.19	214.171352	-0.0058717	0.00398258	2.14676296	0.14287083	0.27709494	Kctd9
ENSMUSG00000034329.17	61.250805	0.00563318	0.00552047	1.03114565	0.30988974	0.49510987	Brip1

ENSMUSG00000034330.11	74.5534465	0.05776062	0.00527606	112.978027	2.18E-26	2.50E-24	Plcg2
ENSMUSG00000034333.16	144.124664	-6.21E-05	0.00332736	0.00035858	0.98489204	1	Zbed4
ENSMUSG00000034334.9	72.8402117	-0.0058027	0.00332263	3.04264872	0.08110348	0.17809392	Fam151b
ENSMUSG00000034336.4	9.02831217	0.03453745	0.01759008	4.20000622	0.04042383	0.10151581	Ina
ENSMUSG00000034341.18	1823.02855	-0.0016154	0.00235447	0.47003048	0.49297315	0.68051968	Wbp2
ENSMUSG00000034342.10	316.945145	0.01683302	0.00488347	11.246872	0.00079757	0.0036036	Cbl
ENSMUSG00000034343.15	712.952888	-0.0125561	0.00226537	30.6194426	3.14E-08	3.52E-07	Ube2f
ENSMUSG00000034345.15	668.058115	-0.0044385	0.00344943	1.63816869	0.20057692	0.3591014	Gtf2h5
ENSMUSG00000034349.15	432.192949	0.01053206	0.00439015	5.45506334	0.0195117	0.05574163	Smc4
ENSMUSG00000034353.15	84.6787262	0.01415362	0.00399122	12.1809825	0.00048279	0.00231964	Ramp1
ENSMUSG00000034354.18	683.313784	-9.74E-06	0.00200254	2.35E-05	0.99612902	1	Mtmr3
ENSMUSG00000034361.11	164.655982	-0.0074381	0.00376241	3.90649967	0.04809968	0.1168643	Cpne2
ENSMUSG00000034371.10	5370.24771	-0.0064381	0.00403195	2.52556378	0.11201474	0.22939825	Tkfc
ENSMUSG00000034377.11	785.849949	-0.0015026	0.00225347	0.45280287	0.50100691	0.68782008	Tulp4
ENSMUSG00000034379.10	47.612731	0.01238751	0.00320942	14.6742301	0.00012778	0.00071249	Wdr5b
ENSMUSG00000034382.15	428.667128	-0.0119295	0.00421183	7.84197517	0.00510468	0.01801032	AI661453
ENSMUSG00000034390.17	373.716641	0.00588538	0.00425336	1.87704673	0.17067019	0.31711257	Cmip
ENSMUSG00000034394.15	7.75637483	0.07702918	0.01380614	29.0883186	6.92E-08	7.34E-07	Lif
ENSMUSG00000034401.17	41.1487606	0.0231091	0.00598986	14.1194466	0.00017156	0.00093193	Spata6
ENSMUSG00000034403.17	993.662573	-0.0109987	0.00216089	25.7499085	3.89E-07	3.62E-06	Pja1
ENSMUSG00000034412.14	196.96797	0.00647016	0.00352771	3.35318065	0.06707529	0.15297214	Tbc1d10a
ENSMUSG00000034413.15	37.5034123	0.01733896	0.00776907	4.88511574	0.02708922	0.07313717	Neurl1b
ENSMUSG00000034416.19	1.25782587	0.03757674	0.01924962	4.65292234	0.03100073	0.08170855	Pkd1l2
ENSMUSG00000034422.15	759.109322	0.01921914	0.00382799	24.948587	5.89E-07	5.30E-06	Parp14
ENSMUSG00000034424.6	1844.97771	-0.0078825	0.00233635	11.4947397	0.00069793	0.00320325	Gcsh
ENSMUSG00000034427.18	17.7750553	0.01305095	0.01076122	1.37636455	0.24072137	0.41053176	Myo15b
ENSMUSG00000034429.16	440.406533	-0.0088363	0.00370315	5.65338084	0.01742175	0.05079227	Zfp707
ENSMUSG00000034430.17	238.355541	-0.0023728	0.00208383	1.30534538	0.25323913	0.4254201	Zxdc
ENSMUSG00000034432.12	451.486102	-0.0023501	0.00254466	0.83692231	0.36027848	0.54957497	Cops8
ENSMUSG00000034435.7	768.687608	0.00321719	0.00351607	0.82202332	0.36458926	0.55435806	Tmem30b
ENSMUSG00000034438.17	115.540607	0.06187659	0.0061719	92.0888167	8.29E-22	5.33E-20	Gbp8
ENSMUSG00000034442.12	144.573707	-0.0064438	0.00484856	1.73591459	0.18765731	0.34112411	Trmt5
ENSMUSG00000034445.9	277.428847	-0.0065371	0.0026144	6.29866013	0.01208293	0.03736076	Cyb561a3
ENSMUSG00000034449.8	318.148428	-0.0080191	0.00249695	10.3388625	0.00130258	0.00553562	Dhrs11
ENSMUSG00000034450.9	6323.29636	-0.012387	0.00458376	7.19265976	0.00732024	0.02453882	Gulo
ENSMUSG00000034452.8	2.0820791	0.0440985	0.01525491	9.51311023	0.00204009	0.00822514	Slc24a1
ENSMUSG00000034453.9	278.661855	-0.0033112	0.00283347	1.34024309	0.2469911	0.41838065	Polr3b
ENSMUSG00000034456.16	6816.96717	0.00411197	0.00500792	0.67353496	0.41182187	0.60336639	Uroc1
ENSMUSG00000034457.11	28.3788783	0.05435136	0.00742362	45.3384095	1.66E-11	2.98E-10	Eda2r
ENSMUSG00000034459.9	274.995733	0.03188409	0.00588158	30.1331665	4.03E-08	4.43E-07	Ifit1
ENSMUSG00000034460.10	24.5661759	0.00479669	0.01750298	0.10963411	0.74056108	0.87720337	Six4
ENSMUSG00000034462.10	403.043877	0.00965602	0.00251175	14.394307	0.00014825	0.00081468	Pkd2
ENSMUSG00000034463.5	61.3939183	0.01425474	0.00500474	8.26601438	0.00403941	0.01474822	Scara3
ENSMUSG00000034471.13	231.657316	0.00262662	0.00329749	0.63085346	0.42704243	0.61861091	Caskin2
ENSMUSG00000034472.14	2.89256625	0.03307233	0.01124756	9.04102074	0.00263988	0.0102442	Rasd2
ENSMUSG00000034473.15	423.68622	-0.0003327	0.00291991	0.01293113	0.90946363	0.98051784	Sec22a
ENSMUSG00000034480.20	386.755825	-0.0019236	0.00430235	0.19661501	0.6574671	0.81922019	Diaph2
ENSMUSG00000034484.9	1078.02446	0.01145668	0.00253008	20.302688	6.61E-06	4.88E-05	Snx2
ENSMUSG00000034485.11	194.121743	0.00726042	0.00379092	3.62200762	0.05702003	0.13432624	Uaca
ENSMUSG00000034487.5	206.079778	0.01226159	0.00223249	29.2758101	6.28E-08	6.70E-07	Poglut3
ENSMUSG00000034488.15	16.4954823	0.0232427	0.01555783	2.37867477	0.12300185	0.24692499	Edil3
ENSMUSG00000034501.8	149.281039	0.00795786	0.00223722	12.6797947	0.00036963	0.00182883	Pcnx4
ENSMUSG00000034509.10	116.090602	-0.0144868	0.00297739	23.7406848	1.10E-06	9.41E-06	Mad2l1bp
ENSMUSG00000034518.15	157.811226	0.01158053	0.00377096	9.22139766	0.00239203	0.00941562	Hmgxb4
ENSMUSG00000034520.15	44.0073065	0.00861259	0.0089005	1.02551542	0.3112143	0.49626803	Gjc1
ENSMUSG00000034522.11	587.806853	0.00562144	0.00426693	1.74314153	0.18674127	0.33985181	Zfp395
ENSMUSG00000034525.8	384.420141	-0.005164	0.00287547	3.15967888	0.07547803	0.16819962	Ice1
ENSMUSG00000034528.16	6373.87841	0.01244681	0.005234	5.64116656	0.01754353	0.05109678	Hsd17b13
ENSMUSG00000034533.11	2.40599955	0.0761241	0.01295246	30.5977793	3.17E-08	3.55E-07	Scn10a
ENSMUSG00000034538.9	30.2878426	0.01136348	0.00305046	13.6045657	0.00022564	0.0011865	Zfp418
ENSMUSG00000034543.16	374.252699	0.00377296	0.00346018	1.171173	0.27916035	0.45835545	Morc2a

ENSMUSG00000034544.18	649.540431	0.00280028	0.00356652	0.61122707	0.43432617	0.62600935	Rsrc1
ENSMUSG00000034551.13	5.58850302	0.0301487	0.00806399	13.6367294	0.0002218	0.00116904	Hdx
ENSMUSG00000034557.15	418.744968	-0.0061927	0.00285012	4.72350497	0.02975304	0.07894647	Zfyve9
ENSMUSG00000034560.7	580.170355	-0.0023856	0.00363606	0.4093301	0.52230971	0.70812191	Washc4
ENSMUSG00000034563.17	1128.12076	-0.0041937	0.00197466	4.45610795	0.03477665	0.08984031	Ccpg1
ENSMUSG00000034566.11	4514.24775	-0.0078483	0.00336232	5.50722861	0.01893803	0.05440573	Atp5pd
ENSMUSG00000034570.13	4.92254235	0.03683587	0.01338738	7.2834784	0.00695917	0.02352234	Inpp5j
ENSMUSG00000034573.15	38.423414	0.02160419	0.00526915	16.3147464	5.36E-05	0.00032686	Ptpn13
ENSMUSG00000034574.11	1088.34815	-0.0024175	0.00301636	0.63969511	0.42382122	0.61550157	Daam1
ENSMUSG00000034575.15	332.118359	-0.0029528	0.00290254	1.05113792	0.30524516	0.49009614	Tent4a
ENSMUSG00000034584.4	349.653969	-0.0094251	0.00638975	2.1172395	0.14564886	0.28123244	Exph5
ENSMUSG00000034586.15	42.3239855	0.01601345	0.00462228	11.7603815	0.00060505	0.00282476	Hid1
ENSMUSG00000034591.6	784.558135	0.02612289	0.00617976	16.2774321	5.47E-05	0.00033276	Slc41a2
ENSMUSG00000034593.17	70.9940029	0.07299177	0.00908678	56.8315006	4.75E-14	1.19E-12	Myo5a
ENSMUSG00000034595.18	129.685816	0.03910099	0.00523616	53.2776392	2.90E-13	6.55E-12	Ppp1r18
ENSMUSG00000034601.18	72.609539	-0.0016871	0.00336137	0.24958714	0.61736594	0.78855272	2700049A03Rik
ENSMUSG00000034602.16	1071.13818	-0.0043476	0.00293298	2.17723912	0.14006553	0.27300103	Mon2
ENSMUSG00000034607.7	3.06653314	0.0421945	0.00978111	18.3125403	1.87E-05	0.00012611	Pof1b
ENSMUSG00000034610.15	392.026416	0.01253514	0.00430033	8.31713184	0.00392729	0.01438786	Tut4
ENSMUSG00000034612.8	23.4384588	0.10349629	0.00967228	100.757368	1.04E-23	8.23E-22	Chst11
ENSMUSG00000034613.13	92.9341078	0.03085844	0.00531449	31.9355759	1.59E-08	1.87E-07	Ppm1h
ENSMUSG00000034614.15	52.6799578	0.0287585	0.00852583	10.4658343	0.00121603	0.0052202	Pik3ip1
ENSMUSG00000034616.18	218.886849	0.00942789	0.00321192	8.60445108	0.00335342	0.01257654	Ssh3
ENSMUSG00000034617.8	140.907237	0.00083698	0.00342457	0.05802074	0.80965203	0.921599	Mtrr
ENSMUSG00000034620.18	396.315147	-0.0086406	0.00295816	8.60798832	0.00334692	0.01255613	Rxylt1
ENSMUSG00000034621.15	449.137209	-0.0047856	0.00328539	2.09926603	0.14736987	0.28367466	Gpatch8
ENSMUSG00000034634.8	113.070165	0.09908066	0.01071293	73.2562637	1.14E-17	4.45E-16	Ly6d
ENSMUSG00000034636.10	983.129429	-0.001767	0.00272917	0.4164942	0.51869157	0.704756	Zyg11b
ENSMUSG00000034639.8	62.2480536	-0.002298	0.0027538	0.69447289	0.40464714	0.59624682	Setmar
ENSMUSG00000034640.10	190.265881	0.01093833	0.00382231	7.99845839	0.00468172	0.0167004	Tiparp
ENSMUSG00000034641.3	291.503628	0.04381288	0.00591266	50.5506715	1.16E-12	2.41E-11	Cd300ld
ENSMUSG00000034645.15	58.563652	-0.0414189	0.00472004	76.0280789	2.80E-18	1.17E-16	Zyg11a
ENSMUSG00000034647.16	404.455619	0.02166912	0.00454546	22.3559239	2.27E-06	1.83E-05	Ankrd12
ENSMUSG00000034648.10	8.66012624	0.01838674	0.00981057	3.36115251	0.06675134	0.15236592	Lrrn1
ENSMUSG00000034652.13	177.077477	0.05272809	0.00624195	66.4982754	3.50E-16	1.13E-14	Cd300a
ENSMUSG00000034653.12	194.141198	-0.0005113	0.00322412	0.02481673	0.87482458	0.96137166	Ythdc2
ENSMUSG00000034656.19	23.8346377	0.03907633	0.00916061	17.7636617	2.50E-05	0.00016346	Cacna1a
ENSMUSG00000034659.15	415.03868	-0.0045581	0.00272786	2.78657377	0.09505727	0.20203846	Tmem109
ENSMUSG00000034663.14	234.814359	0.02113146	0.00268706	59.233097	1.40E-14	3.78E-13	Bmp2k
ENSMUSG00000034664.14	62.2067039	-0.0010884	0.00584642	0.03348353	0.85480976	0.95034376	Itga2b
ENSMUSG00000034667.9	1466.21354	-0.0011932	0.00279156	0.18177767	0.66984997	0.8280875	Xpot
ENSMUSG00000034673.15	301.216992	0.00398905	0.0031936	1.53989505	0.21463343	0.37766487	Pbx2
ENSMUSG00000034674.19	362.158238	-0.0053064	0.00204475	6.72374294	0.00951378	0.03060864	Tdg
ENSMUSG00000034675.18	57.0187608	0.03204626	0.00572877	30.690152	3.03E-08	3.40E-07	Dbn1
ENSMUSG00000034681.18	482.28278	-0.0027126	0.00195397	1.93353803	0.16437189	0.307884	Rnps1
ENSMUSG00000034684.13	113.20569	0.01258962	0.00459046	7.52845864	0.00607318	0.02087773	Sema3f
ENSMUSG00000034685.5	43.8053231	-0.0077061	0.00749732	1.02930215	0.31032263	0.49558328	Fam171a2
ENSMUSG00000034686.9	2.08281243	0.02741268	0.01143577	5.63253901	0.01763008	0.05128719	Prr7
ENSMUSG00000034687.9	95.0723867	-0.0003826	0.00593146	0.00397494	0.94972896	1	Fras1
ENSMUSG00000034689.3	0.86778912	-0.0139742	0.08898694	-0.0053291	1	1	Ccdc202
ENSMUSG00000034706.17	1.97823561	0.02116585	0.0165412	1.43130368	0.23155179	0.39924063	Dnai2
ENSMUSG00000034707.8	2707.76919	0.00684249	0.00234506	8.55492553	0.00344589	0.01285585	Gns
ENSMUSG00000034708.13	3473.72038	0.01867219	0.00344663	29.1039223	6.86E-08	7.29E-07	Grn
ENSMUSG00000034709.9	365.804963	0.01412831	0.00296694	22.4797389	2.12E-06	1.73E-05	Ppp1r21
ENSMUSG00000034714.10	1098.95904	0.00392786	0.00301207	1.66120727	0.1974404	0.35456291	Ttyh2
ENSMUSG00000034723.12	510.29375	-0.0187822	0.00281972	43.6023222	4.02E-11	6.82E-10	Tmx4
ENSMUSG00000034724.19	909.411404	0.0021767	0.00366623	0.34007973	0.5597832	0.74116126	Cnot6l
ENSMUSG00000034729.17	668.965961	-0.0107996	0.00244553	19.6739908	9.18E-06	6.56E-05	Mrps10
ENSMUSG00000034730.19	9.61891347	0.02302679	0.01634756	2.65747096	0.10306449	0.21496985	Adgrb1
ENSMUSG00000034731.12	82.9421929	0.00058752	0.00500134	0.01374171	0.90668172	0.97928404	Dgkh
ENSMUSG00000034738.9	310.133869	0.00157546	0.00290075	0.29682939	0.58587697	0.76286266	Nostrin

ENSMUSG00000034744.14	249.473454	0.01172994	0.00329347	12.4746262	0.00041252	0.00201734	Nagk
ENSMUSG00000034748.17	44.8743247	0.00732546	0.0033814	4.66481252	0.03078678	0.08125065	Sirt6
ENSMUSG00000034751.18	165.737279	0.01772434	0.00442943	15.6361244	7.68E-05	0.00045067	Mast4
ENSMUSG00000034755.20	33.9991187	0.10011809	0.00962837	92.8704126	5.58E-22	3.71E-20	Pcdh11x
ENSMUSG00000034757.16	452.518854	0.0029914	0.0020618	2.1011099	0.14719226	0.28342531	Tmub2
ENSMUSG00000034758.13	33.0939031	-0.0125733	0.0060943	4.31133906	0.03785915	0.09611174	Tle6
ENSMUSG00000034761.17	419.377259	-0.0095288	0.00241346	15.4603596	8.43E-05	0.00049006	Map4k5
ENSMUSG00000034762.10	1.75378266	0.03032706	0.01461869	4.48250423	0.03424351	0.08871567	Glis1
ENSMUSG00000034765.7	25.5568507	0.02008275	0.00678846	8.27434199	0.00402092	0.0146921	Dusp5
ENSMUSG00000034768.5	2.88802658	-0.0002114	0.0102215	0.00174356	0.96669324	1	Asb16
ENSMUSG00000034771.16	182.963595	-0.0100533	0.00520669	3.61745209	0.05717638	0.13464077	Tle2
ENSMUSG00000034773.17	3.75946931	0.00855584	0.01388707	0.30283427	0.58211102	0.75972059	Hrob
ENSMUSG00000034774.7	186.190365	0.05633506	0.00616303	80.5512224	2.83E-19	1.34E-17	Dsg1c
ENSMUSG00000034780.7	583.141181	-0.0179533	0.00571379	9.07942475	0.00258501	0.01006108	B3galt1
ENSMUSG00000034781.6	1049.23288	-0.0012146	0.00225316	0.28878335	0.59100125	0.76703516	Gna11
ENSMUSG00000034783.8	244.551967	0.07332953	0.00746786	78.1548288	9.53E-19	4.23E-17	Cd207
ENSMUSG00000034785.16	2002.34742	-0.058393	0.0062896	79.7804797	4.18E-19	1.92E-17	Dio1
ENSMUSG00000034786.18	59.6149571	0.04224429	0.00579922	50.5072427	1.19E-12	2.46E-11	Gpsm3
ENSMUSG00000034789.9	491.913897	-0.0060776	0.00385743	2.52304244	0.11219393	0.22966552	Rab24
ENSMUSG00000034792.9	16.9133909	0.03167125	0.00750118	16.7513786	4.26E-05	0.00026623	Gna15
ENSMUSG00000034793.16	83.3650749	0.00328886	0.00344993	0.9062997	0.34109813	0.5289028	G6pc3
ENSMUSG00000034795.15	176.748405	0.02121979	0.00460338	20.1513566	7.15E-06	5.24E-05	Ccdc122
ENSMUSG00000034796.15	3.73195382	0.05571882	0.0101239	28.3372346	1.02E-07	1.06E-06	Cpne7
ENSMUSG00000034799.17	31.0415459	0.01953752	0.00881828	4.47144385	0.03446586	0.08921582	Unc13a
ENSMUSG00000034800.16	69.6320636	-0.008177	0.00272001	9.08277583	0.00258028	0.01004597	Zfp661
ENSMUSG00000034801.14	326.791344	0.00610191	0.00306926	3.90872356	0.04803607	0.11677785	Sos2
ENSMUSG00000034807.10	1234.05526	0.00739201	0.00256203	8.18754494	0.0042179	0.01529806	Colgalt1
ENSMUSG00000034810.8	20.0960591	0.02610074	0.00762075	11.4828959	0.0007024	0.0032212	Scn7a
ENSMUSG00000034813.20	4.84727758	0.03236744	0.00957719	11.6342142	0.0006475	0.00300332	Grip1
ENSMUSG00000034818.18	6.40945992	0.02696089	0.0166807	2.90423836	0.08834698	0.19069752	Celf5
ENSMUSG00000034820.5	526.357763	-0.0028149	0.0026108	1.15747302	0.28199023	0.46171618	Cpsf7
ENSMUSG00000034825.15	15.3346451	0.03574949	0.01390499	5.90102281	0.01513209	0.04518364	Nrip3
ENSMUSG00000034826.15	352.372402	0.00620166	0.00220987	7.63220424	0.00573352	0.01989566	Nup54
ENSMUSG00000034829.10	3.420557	0.00470363	0.01733463	0.14843298	0.70003738	0.85025396	Nxnl1
ENSMUSG00000034832.16	410.942944	-0.0017349	0.00352165	0.24514738	0.62051288	0.79076165	Tet3
ENSMUSG00000034833.11	6.42737976	0.06371942	0.00907642	42.5738697	6.81E-11	1.12E-09	Tespa1
ENSMUSG00000034837.13	125.770398	0.03471063	0.00726452	21.1675741	4.21E-06	3.24E-05	Gnat1
ENSMUSG00000034839.6	5.35940438	0.06698647	0.01106189	34.5625837	4.13E-09	5.28E-08	Larp6
ENSMUSG00000034842.17	89.6416766	0.00884272	0.00659131	0.0252927	0.87363989	0.96079021	Art3
ENSMUSG00000034845.7	805.309304	0.01065008	0.00309119	11.90249	0.00056059	0.00264388	Plvap
ENSMUSG00000034848.18	76.1246888	0.01161528	0.00332757	11.9412807	0.00054904	0.00259823	Ttc21b
ENSMUSG00000034850.16	920.41768	0.00028968	0.00170456	0.02880493	0.86523023	0.95599514	Tmem127
ENSMUSG00000034853.17	167.959024	0.01878564	0.00545049	11.6082194	0.00065661	0.0030402	Acot11
ENSMUSG00000034854.9	190.076569	0.01506127	0.00249588	36.5808159	1.46E-09	1.98E-08	Mfsd12
ENSMUSG00000034855.14	92.8874485	0.04411161	0.00664846	41.7431876	1.04E-10	1.66E-09	Cxcl10
ENSMUSG00000034858.17	1171.64062	-0.0184548	0.0036156	26.7452791	2.32E-07	2.26E-06	Atosa
ENSMUSG00000034863.10	67.1804771	0.00955071	0.00356266	7.21210853	0.00724134	0.0243182	Ano8
ENSMUSG00000034867.17	568.760007	0.00872589	0.00316427	7.60888133	0.00580815	0.0200955	Ankrd27
ENSMUSG00000034868.10	1035.30522	0.01187536	0.00269171	19.3623473	1.08E-05	7.61E-05	Myl12b
ENSMUSG00000034871.3	2.4574171	-0.0028843	0.02624989	-0.0664742	1	1	Fam151a
ENSMUSG00000034872.7	12.4967384	0.0015981	0.00511846	0.09482703	0.758128	0.88890471	Gipc3
ENSMUSG00000034875.6	897.903758	0.01168565	0.00274807	18.1555205	2.04E-05	0.00013598	Nudt19
ENSMUSG00000034880.8	510.718546	-0.0158965	0.00315718	25.6914559	4.01E-07	3.72E-06	Mrpl34
ENSMUSG00000034881.9	22.4358244	0.00156807	0.00432346	0.13640211	0.71188433	0.85779625	Tbxa2r
ENSMUSG00000034883.10	72.7112891	0.01449816	0.01823398	0.79014991	0.3740548	0.56499881	Lrr1
ENSMUSG00000034889.9	217.825342	-0.0055507	0.00233323	5.6436689	0.01751851	0.05103021	Cactin
ENSMUSG00000034891.14	9.3816547	0.02549176	0.02169882	2.06990253	0.15023112	0.28779656	Sncb
ENSMUSG00000034892.9	2528.5598	-0.0020212	0.00417909	0.23559197	0.62740811	0.79606475	Rps29
ENSMUSG00000034893.10	352.607127	0.00089918	0.0019985	0.20492015	0.65077841	0.81359374	Cog3
ENSMUSG00000034898.18	43.3374283	-0.0015779	0.00729048	0.04434793	0.83320763	0.9360364	Filip1
ENSMUSG00000034902.18	523.029679	0.0133925	0.00270364	24.7906748	6.39E-07	5.70E-06	Pip5k1c

ENSMUSG00000034903.19	825.576166	-0.0025577	0.00378176	0.45243901	0.50117898	0.68785612	Cobll1
ENSMUSG00000034906.16	21.7334993	-0.0065409	0.00816823	0.59003739	0.44240466	0.63431953	Ncaph
ENSMUSG00000034908.17	2242.55441	0.00053934	0.00452692	0.01406626	0.90559126	0.97866278	Sidt2
ENSMUSG00000034910.6	31.1364445	0.01262046	0.00703998	3.11754515	0.07745339	0.17174162	Pygo1
ENSMUSG00000034911.9	254.850419	-0.0071146	0.00376445	3.58653268	0.05824966	0.13667693	Ushbp1
ENSMUSG00000034912.19	14.0388726	0.01082003	0.01497642	0.49005805	0.48390141	0.67311269	Mdga2
ENSMUSG00000034913.11	1.47170725	-0.0147896	0.07180475	-0.013867	1	1	Cby2
ENSMUSG00000034917.9	416.029799	-0.0034822	0.00459216	0.56257801	0.45322338	0.64496403	Tjp3
ENSMUSG00000034918.9	4.75972467	0.01635892	0.01083357	2.29124044	0.13010591	0.25802322	Cdhr2
ENSMUSG00000034919.3	1.43081958	0.0324584	0.01312206	6.44363986	0.01113503	0.03493337	Ttc22
ENSMUSG00000034926.4	11952.5016	0.01941575	0.00443232	18.6253355	1.59E-05	0.00010864	Dhcr24
ENSMUSG00000034928.17	800.599062	-0.000275	0.00346597	0.00609987	0.93774718	0.99588386	Rnf44
ENSMUSG00000034930.17	173.575632	-0.0042181	0.00363595	1.33172825	0.24849799	0.41988379	Rtkn
ENSMUSG00000034931.16	317.370453	-0.0060996	0.00249244	5.95693553	0.0146595	0.04406832	Dhx8
ENSMUSG00000034932.5	388.998188	-0.0092252	0.00386479	5.77731994	0.01623428	0.04789264	Mrpl54
ENSMUSG00000034936.3	368.570305	-0.026329	0.00523196	27.1074969	1.92E-07	1.90E-06	Arl4d
ENSMUSG00000034940.16	334.620327	-0.0031887	0.00335451	0.88325163	0.34731263	0.53552527	Synrg
ENSMUSG00000034947.14	219.941031	0.02992937	0.0035985	67.4290461	2.18E-16	7.26E-15	Tmem106a
ENSMUSG00000034949.19	10.2349716	0.00617746	0.00931588	0.38705748	0.53385019	0.71816986	Zfr2
ENSMUSG00000034951.11	420.24487	-0.0026488	0.00274462	0.9328282	0.3341291	0.52104257	Cog7
ENSMUSG00000034957.11	5551.73051	-0.0048713	0.0038758	1.53201351	0.2158105	0.37922418	Cebpa
ENSMUSG00000034958.12	5.02031429	0.0669184	0.01609833	18.3276874	1.86E-05	0.0001253	Atcay
ENSMUSG00000034959.9	16.9813049	0.06584869	0.00611637	109.139348	1.51E-25	1.52E-23	Rubcnl
ENSMUSG00000034968.4	2.4796523	0.02315184	0.01012065	4.80620748	0.02835739	0.07593521	Lbx2
ENSMUSG00000034973.17	286.28449	0.00924081	0.00275958	11.2111876	0.00081306	0.00366388	Dop1a
ENSMUSG00000034974.14	301.208318	-0.0044658	0.00286195	2.40933684	0.12061345	0.24318099	Dapk3
ENSMUSG00000034981.10	28.1688458	0.04004271	0.00865153	18.9493541	1.34E-05	9.30E-05	Parm1
ENSMUSG00000034987.5	6.62067165	0.09082558	0.00958335	80.3964607	3.06E-19	1.44E-17	Hrh2
ENSMUSG00000034993.8	510.399374	0.02421337	0.00280432	72.9905883	1.30E-17	5.05E-16	Vat1
ENSMUSG00000034994.11	21295.8011	-0.0038136	0.00263958	2.06388819	0.15082489	0.28858245	Eef2
ENSMUSG00000034997.6	2.9450238	0.0864635	0.01227894	48.9842317	2.58E-12	5.15E-11	Htr2a
ENSMUSG00000034998.19	314.290697	0.01043157	0.00263235	15.2496061	9.42E-05	0.00054162	Foxn2
ENSMUSG00000035000.9	2320.51404	0.00227829	0.00339275	0.45064518	0.50202873	0.68874183	Dpp4
ENSMUSG00000035004.4	114.885123	0.06077924	0.00593786	95.1809378	1.74E-22	1.22E-20	Igsf6
ENSMUSG00000035007.6	210.043191	-0.0055783	0.00148446	14.0434193	0.00017864	0.00096703	Rundc1
ENSMUSG00000035011.16	916.925458	-0.0017733	0.00320633	0.30355336	0.58166331	0.75926618	Zbtb7a
ENSMUSG00000035021.15	537.164614	0.0085737	0.00520101	2.62742671	0.10503182	0.21805126	Baz1a
ENSMUSG00000035024.18	184.555971	-0.0025249	0.00311596	0.66844622	0.41359385	0.60500028	Ncapd3
ENSMUSG00000035027.19	1585.50732	-0.0135906	0.00264538	26.5167073	2.61E-07	2.52E-06	Map2k2
ENSMUSG00000035031.16	11030.8869	-0.025349	0.00620317	16.1700537	5.79E-05	0.00035009	C8a
ENSMUSG00000035033.16	10.3344289	0.0357841	0.02055653	3.01043121	0.08273028	0.18089148	Tbr1
ENSMUSG00000035041.9	4269.56165	-0.0262954	0.00504176	26.6645561	2.42E-07	2.35E-06	Creb3l3
ENSMUSG00000035042.3	71.6900223	0.13806028	0.00845406	223.467186	1.59E-50	2.67E-47	Ccl5
ENSMUSG00000035045.18	7.45316687	0.03315398	0.00908278	12.1387074	0.00049386	0.00236944	Zc3h12b
ENSMUSG00000035047.10	187.386943	0.00277057	0.00324439	0.71718316	0.39706944	0.5889002	Kri1
ENSMUSG00000035048.14	665.030777	-0.0134927	0.00333753	16.4403664	5.02E-05	0.0003074	Anapc13
ENSMUSG00000035049.5	132.558587	-0.0114423	0.00372018	9.54805338	0.00200162	0.00808946	Rrp12
ENSMUSG00000035051.16	210.817179	0.00175924	0.00345636	0.25586419	0.61297586	0.78493124	Dhx57
ENSMUSG00000035062.14	30.5982595	0.0109372	0.00461204	5.52196036	0.0187792	0.05403517	Zc4h2
ENSMUSG00000035064.18	192.62768	-0.0005807	0.00483719	0.01473443	0.90338575	0.97739707	Eef2k
ENSMUSG00000035067.10	46.1878789	0.00402113	0.00412436	0.90045948	0.34265854	0.53062466	Xkr6
ENSMUSG00000035069.4	271.8182	-0.0108464	0.00226872	22.6236493	1.97E-06	1.62E-05	Oma1
ENSMUSG00000035078.7	338.248264	-0.0034079	0.00197369	2.96257506	0.08521213	0.18532166	Mtmr9
ENSMUSG00000035085.6	8.04063808	-0.0022958	0.01772217	0.02536001	0.87347328	0.96072285	1700020L24Rik
ENSMUSG00000035086.14	1089.60016	-0.0071357	0.00192073	13.8171351	0.00020149	0.00107446	Becn1
ENSMUSG00000035093.17	1497.9096	0.01012862	0.00270905	13.573321	0.00022942	0.00120382	Secisbp2l
ENSMUSG00000035095.12	49.6196615	0.01905761	0.00596389	9.89145672	0.00166048	0.00684567	Fam167a
ENSMUSG00000035104.15	1594.81009	-0.0048719	0.00407613	1.42418255	0.2327162	0.40063117	Eva1a
ENSMUSG00000035105.6	288.939014	-0.0085185	0.00342286	6.12821081	0.01330414	0.04050491	Egln3
ENSMUSG00000035107.14	75.2687083	0.01078925	0.0051243	4.34517868	0.03711387	0.09454237	Dcbld2
ENSMUSG00000035109.15	4.81089363	-0.0012433	0.00918532	0.01827909	0.8924536	0.97160892	Shc4

ENSMUSG00000035112.18	51.2908075	-0.0473479	0.00839885	31.1506162	2.39E-08	2.73E-07	Wnk4
ENSMUSG00000035121.7	14.8949035	0.01590287	0.00579189	7.20973516	0.00725092	0.02434105	Neil2
ENSMUSG00000035125.11	81.8786709	-8.99E-05	0.00437958	0.0004072	0.98390036	1	Gcfc2
ENSMUSG00000035126.21	15.8414855	-0.0065841	0.007024	0.84066548	0.35920651	0.5482589	Dnai4
ENSMUSG00000035131.15	1.98294998	0.05839142	0.01694867	12.2427222	0.00046708	0.00225101	Brinp3
ENSMUSG00000035133.10	1831.31782	-0.0078252	0.00346822	4.93915285	0.0262549	0.07130909	Arhgap5
ENSMUSG00000035139.15	293.956258	-0.0056407	0.00226008	6.18509991	0.01288304	0.03941108	Secisbp2
ENSMUSG00000035142.19	199.418124	0.00018569	0.0031971	0.0033894	0.95357453	1	Nubpl
ENSMUSG00000035150.16	1943.17456	6.70E-05	0.0014793	0.00201459	0.9641996	1	Eif2s3x
ENSMUSG00000035151.13	330.737663	0.00513621	0.00273088	3.48380215	0.0619722	0.14356902	Elmod2
ENSMUSG00000035152.15	962.257585	0.0031192	0.00288252	1.15474046	0.282559	0.46255112	Ap2b1
ENSMUSG00000035158.16	98.8705387	0.01216873	0.00430129	7.7001355	0.00552167	0.01925685	Mitf
ENSMUSG00000035161.8	614.919428	-0.0058788	0.00427578	1.90633005	0.16737149	0.31232514	Ints6
ENSMUSG00000035164.9	151.589801	0.00298108	0.00339371	0.74844225	0.38696987	0.57846821	Zc3h12c
ENSMUSG00000035165.15	29.9321729	0.03449238	0.00689267	23.4995686	1.25E-06	1.06E-05	Kcne3
ENSMUSG00000035168.17	269.318546	0.01293586	0.00321543	15.9776423	6.41E-05	0.00038408	Tanc1
ENSMUSG00000035171.10	550.72379	-0.0117475	0.00315456	13.5485262	0.00023247	0.00121793	1110059E24Rik
ENSMUSG00000035172.16	141.072877	-0.0014418	0.0033022	0.19046416	0.66253058	0.82289764	Plekhh3
ENSMUSG00000035173.15	385.481525	-0.0007381	0.0037174	0.03761263	0.84622304	0.94462666	Ccdc186
ENSMUSG00000035181.7	349.775022	0.00045646	0.00310711	0.02108025	0.88456054	0.96656219	Heatr5a
ENSMUSG00000035183.15	19.5994363	0.01255597	0.00487085	6.50789304	0.01073967	0.03391429	Slc24a5
ENSMUSG00000035184.16	24330.1045	0.00407502	0.0057892	0.4515893	0.50158119	0.68832803	Fam124a
ENSMUSG00000035186.7	57.2879491	0.13876001	0.01133877	118.195273	1.57E-27	2.13E-25	Ubd
ENSMUSG00000035189.18	4.40321596	0.01550534	0.01337385	1.25557132	0.26249105	0.43716381	Ano4
ENSMUSG00000035198.10	456.861784	-0.0092201	0.00232991	15.7834644	7.10E-05	0.00042077	Tubg1
ENSMUSG00000035199.7	678.018658	0.00742576	0.00268737	7.59843253	0.00584191	0.02020044	Arl6ip5
ENSMUSG00000035202.9	183900.88	-0.0018481	0.00815535	0.05191779	0.81975915	0.92763887	Lars2
ENSMUSG00000035203.17	1977.91715	-0.003845	0.00248438	2.40225787	0.12116026	0.24407977	Epn1
ENSMUSG00000035206.11	227.325555	-0.0159757	0.00307722	26.9421915	2.10E-07	2.06E-06	Sppl2b
ENSMUSG00000035208.17	81.7050158	0.06471983	0.00604018	108.912525	1.70E-25	1.70E-23	Slfn8
ENSMUSG00000035211.11	1.77690735	0.01301734	0.01331378	0.99592062	0.31829962	0.50405631	Xrra1
ENSMUSG00000035212.15	364.633821	0.01491586	0.0029557	24.6634641	6.83E-07	6.05E-06	Leprot
ENSMUSG00000035215.10	435.362349	-0.0085862	0.00494057	3.06710665	0.07989155	0.17594043	Lsm7
ENSMUSG00000035226.6	12.2469616	0.02717225	0.00966208	7.77132463	0.00530821	0.01861149	Rims4
ENSMUSG00000035227.8	1869.16892	0.00051616	0.00284441	0.03298446	0.8558839	0.95083263	Spcs2
ENSMUSG00000035228.16	6.16843519	0.03805034	0.01140174	8.73749287	0.00311733	0.01179613	Ccdc106
ENSMUSG00000035232.9	18.9650908	0.05252185	0.00800899	39.8019444	2.81E-10	4.21E-09	Pdk3
ENSMUSG00000035234.19	134.379558	0.00323161	0.00256763	1.59560156	0.20652765	0.36722628	Abraxas1
ENSMUSG00000035235.14	36.7646976	0.01276567	0.00397588	10.1210509	0.00146585	0.00614752	Trim13
ENSMUSG00000035236.18	120.69766	0.00390765	0.00541258	0.52060456	0.47058391	0.66149306	Scai
ENSMUSG00000035237.8	5790.43617	0.00367819	0.00394723	0.86572635	0.35214116	0.54065861	Lcat
ENSMUSG00000035239.7	58.8289851	0.02157384	0.00519891	16.6191376	4.57E-05	0.00028335	Neu3
ENSMUSG00000035242.17	8001.13409	-0.0090979	0.00355072	6.66788615	0.00981656	0.0313986	Oaz1
ENSMUSG00000035245.13	127.243642	0.00459906	0.0039548	1.334756	0.24796088	0.41924052	Eogt
ENSMUSG00000035246.17	12.6597713	0.05081837	0.01092358	19.9538341	7.93E-06	5.73E-05	Pcyt1b
ENSMUSG00000035247.18	3069.85867	-0.0106389	0.00360156	8.49125406	0.00356858	0.01325488	Hectd1
ENSMUSG00000035248.10	2235.91202	-0.00416	0.00365577	1.28109861	0.25769487	0.43120326	Tut7
ENSMUSG00000035258.16	110.331456	-0.0040064	0.00392129	1.02826124	0.3105674	0.4957631	Abi3bp
ENSMUSG00000035266.17	103.044534	0.00143469	0.00461748	0.09896058	0.75308057	0.88562815	Helq
ENSMUSG00000035268.15	313.904057	0.00259036	0.00387508	0.44060437	0.5068309	0.69371077	Pkig
ENSMUSG00000035270.16	112.78381	0.03014649	0.00537748	29.6634877	5.14E-08	5.56E-07	Impg2
ENSMUSG00000035273.15	122.104264	0.03669247	0.00335626	115.185164	7.17E-27	8.67E-25	Hpse
ENSMUSG00000035274.14	10.4627429	0.02771356	0.01514989	3.20629048	0.07335562	0.16448005	Tpbg
ENSMUSG00000035275.15	10.0741745	0.0176369	0.00701389	6.44317872	0.01113792	0.03493337	Raver2
ENSMUSG00000035277.16	3.858245	0.01418825	0.03027127	0.29547887	0.58673075	0.76355282	Arx
ENSMUSG00000035278.10	380.533433	0.0009693	0.00450698	0.04589129	0.83037353	0.9345142	Plekhj1
ENSMUSG00000035279.9	12.6940337	0.02330788	0.00972364	5.38904052	0.02026361	0.0576106	Ssc5d
ENSMUSG00000035283.6	24.0068345	0.02670434	0.0062879	17.3483615	3.11E-05	0.00019884	Adrb1
ENSMUSG00000035284.11	598.101066	-0.0071556	0.0033311	4.54010311	0.0331096	0.08621362	Vps13c
ENSMUSG00000035285.7	10.0844394	-0.0144297	0.00689179	4.29608927	0.03820012	0.09679716	Nat14
ENSMUSG00000035293.14	113.575999	0.01182264	0.00351089	11.2710521	0.00078725	0.00356421	G2e3

ENSMUSG00000035295.11	22.7558317	-0.011251	0.00589558	3.58008022	0.05847633	0.13704518	Wdr38
ENSMUSG00000035296.15	46.8859323	0.0040493	0.02702784	2.85641843	0.09100993	0.19515959	Sgcg
ENSMUSG00000035297.15	732.697486	-0.0013487	0.00232313	0.33226791	0.5643267	0.74482327	Cops4
ENSMUSG00000035298.9	2.61349293	0.02306862	0.01227693	3.63176308	0.05668674	0.13375573	Klhl35
ENSMUSG00000035299.18	314.064106	0.00426773	0.0066135	0.39721949	0.52852873	0.71315096	Mid1
ENSMUSG00000035305.6	63.9104558	0.00669871	0.00370158	3.27113712	0.07050873	0.15911012	Ror1
ENSMUSG00000035311.17	247.390679	0.01721506	0.0034426	24.2746688	8.35E-07	7.28E-06	Gnptab
ENSMUSG00000035314.11	26.6961918	0.02186891	0.00736126	8.25026988	0.00407459	0.01485828	Gdpd5
ENSMUSG00000035325.17	2424.59423	0.00015847	0.00288207	0.00306906	0.95582055	1	Sec31a
ENSMUSG00000035329.8	307.186898	-0.004128	0.00352734	1.37474837	0.24099772	0.41085452	Fbxo33
ENSMUSG00000035337.4	3.85864868	0.00128662	0.00840434	0.02290832	0.87969571	0.9636572	Uchl4
ENSMUSG00000035342.15	168.118921	-0.0028128	0.00377683	0.54893712	0.45875231	0.65036557	Lzts2
ENSMUSG00000035351.17	96.9146523	-0.0083375	0.00321199	6.80745274	0.00907782	0.02943084	Nup37
ENSMUSG00000035352.4	5.00118876	0.10879576	0.01708305	29.6084481	5.29E-08	5.71E-07	Ccl12
ENSMUSG00000035354.10	538.83707	-0.0066696	0.0029815	4.93475091	0.02632186	0.07143553	Uvrag
ENSMUSG00000035356.18	271.323534	0.02598094	0.00580855	18.2245578	1.96E-05	0.00013152	Nfkbiz
ENSMUSG00000035357.18	100.904538	0.01006947	0.00568297	3.18404656	0.07436037	0.16621148	Pdzrn3
ENSMUSG00000035365.16	7.39035397	0.0167873	0.01148792	1.82275858	0.17698527	0.32617177	Parpbp
ENSMUSG00000035367.10	347.731174	0.00266123	0.00174529	2.29847066	0.12950145	0.25701963	Rmi1
ENSMUSG00000035370.10	21.4561095	-8.11E-05	0.00587717	0.00019456	0.98887096	1	Gm49322
ENSMUSG00000035372.3	636.056028	-0.0137872	0.00476074	8.09813107	0.00443109	0.0159633	1810055G02Rik
ENSMUSG00000035373.3	15.4042087	0.10214398	0.01349873	43.4794776	4.28E-11	7.21E-10	Ccl7
ENSMUSG00000035376.11	1256.0569	-0.010299	0.00335594	9.31194477	0.00227665	0.0090399	Hacd2
ENSMUSG00000035378.18	83.6054995	-0.0064475	0.00274598	5.54585324	0.01852452	0.05342619	Shq1
ENSMUSG00000035382.10	502.595514	0.0010334	0.00356586	0.08360332	0.77247242	0.89831054	Pcsk7
ENSMUSG00000035383.8	0.94712324	0.00112321	0.05145123	-0.0010945	1	1	Pmch
ENSMUSG00000035385.6	47.2643619	0.0790477	0.00961136	55.7308871	8.31E-14	1.99E-12	Ccl2
ENSMUSG00000035390.17	49.1581167	0.0204816	0.00926073	4.36283944	0.03673103	0.09384079	Brsk1
ENSMUSG00000035392.18	371.387383	0.00654855	0.00252293	6.67893384	0.0097559	0.03123847	Dennd1a
ENSMUSG00000035394.12	2.95106946	-0.0099389	0.01670831	0.27825071	0.59785006	0.77239765	Cfap53
ENSMUSG00000035397.9	78.981092	0.01409837	0.00411615	11.5543598	0.00067591	0.00311995	Klf16
ENSMUSG00000035399.13	430.84925	-0.0026991	0.00196282	1.86629313	0.17190024	0.3189458	Oser1
ENSMUSG00000035401.10	363.700174	-0.0056987	0.00345772	2.68242307	0.10146124	0.2125846	Emsy
ENSMUSG00000035403.11	5.82495149	-4.84E-05	0.00944849	0.00010395	0.99186508	1	Crb2
ENSMUSG00000035407.9	2.51253896	-0.0034206	0.01177611	0.03776965	0.84590642	0.94441458	Kank4
ENSMUSG00000035413.9	172.167997	0.01647856	0.00311435	27.2983312	1.74E-07	1.73E-06	Tmem98
ENSMUSG00000035437.16	478.401476	0.00078667	0.00227506	0.11832269	0.73086084	0.87069824	Rabgap1
ENSMUSG00000035439.14	99.7261162	0.02738075	0.00641519	17.1747253	3.41E-05	0.00021658	Haus8
ENSMUSG00000035441.15	291.103225	0.00983459	0.00285266	11.7444297	0.00061026	0.00284682	Myo1d
ENSMUSG00000035443.11	266.3162	0.00276983	0.00301843	0.85417167	0.35537486	0.54388888	Thyn1
ENSMUSG00000035448.10	163.407031	0.05228562	0.01526175	8.51336994	0.00352547	0.01311747	Ccr3
ENSMUSG00000035451.8	307.265014	-0.0240234	0.00512707	20.7608744	5.20E-06	3.92E-05	Foxa1
ENSMUSG00000035455.13	15.4309897	0.03913746	0.00992878	14.8202649	0.00011826	0.00066521	Fignl1
ENSMUSG00000035456.10	1.96535017	0.03711506	0.02243723	4.26142042	0.03898731	0.09845339	Prdm8
ENSMUSG00000035458.16	1386.81748	0.0043555	0.01372037	-0.0186333	1	1	Tnni3
ENSMUSG00000035459.19	841.872484	0.00394254	0.00430907	0.82115982	0.36484128	0.55463399	Stab2
ENSMUSG00000035469.18	1276.51567	0.00056603	0.00320426	0.03148699	0.85915828	0.95283438	Rcbtb1
ENSMUSG00000035472.15	188.521003	0.00829659	0.00372867	4.91167898	0.0266757	0.0722606	Slc25a21
ENSMUSG00000035473.11	2254.87916	-0.0041042	0.00232095	3.11257993	0.07768981	0.17215256	Galm
ENSMUSG00000035476.10	176.046295	-0.0030672	0.00369639	0.68662375	0.40731515	0.59889353	Tab3
ENSMUSG00000035478.15	696.86681	0.00184609	0.00232354	0.62969646	0.42746668	0.61876943	Mbd3
ENSMUSG00000035486.15	24.3676465	-0.0160847	0.00475971	11.561792	0.00067321	0.00310913	Plk5
ENSMUSG00000035491.6	0.40183402	-0.0165273	0.0581888	0.29169317	0.58913754	0.76574976	Fthl17a
ENSMUSG00000035493.11	890.602516	0.03260947	0.00323161	98.7897006	2.81E-23	2.14E-21	Tgfbi
ENSMUSG00000035495.16	338.003949	0.00273524	0.00231412	1.38284646	0.23961689	0.4091807	Tstd2
ENSMUSG00000035498.11	75.1461287	0.01645105	0.00542788	9.37600917	0.00219844	0.00877663	Cdcp1
ENSMUSG00000035504.17	10074.7204	-0.004564	0.00409738	1.19697966	0.27392618	0.45164788	Reep6
ENSMUSG00000035505.15	135.07915	-0.0162216	0.00346244	21.6299997	3.31E-06	2.60E-05	Cox18
ENSMUSG00000035506.17	8.80419098	0.02301367	0.00637023	12.7134263	0.00036304	0.00180191	Slc12a8
ENSMUSG00000035509.18	42.5443765	0.00933961	0.00408176	5.13234705	0.02348395	0.06501849	Fbxl21
ENSMUSG00000035513.21	26.090136	0.00454632	0.00501964	0.7934198	0.37306805	0.56383334	Ntng2

ENSMUSG00000035517.18	584.54645	-0.000335	0.00334578	0.01015557	0.9197292	0.98644397	Tdrd7
ENSMUSG00000035521.14	402.517938	0.00629589	0.00196585	10.3934762	0.00126461	0.00540152	Gnptg
ENSMUSG00000035523.9	1.89376289	-0.0018313	0.02935355	-0.0110418	1	1	Vmn1r172
ENSMUSG00000035529.11	155.557391	-0.0021857	0.00233619	0.86445647	0.35249458	0.5410606	Prdm4
ENSMUSG00000035530.14	6118.50963	-0.0104022	0.00409007	6.41208976	0.01133459	0.03545593	Eif1
ENSMUSG00000035539.15	3.68100492	0.01663653	0.0144842	1.3623397	0.2431324	0.41344779	Ccdc180
ENSMUSG00000035540.13	100802.756	-0.0014004	0.00584298	-8.708379	1	1	Gc
ENSMUSG00000035545.14	1462.46307	-0.0014742	0.00410833	0.12587154	0.72275157	0.86536467	Leng8
ENSMUSG00000035547.15	70.1488097	0.02656297	0.00385875	46.5880577	8.76E-12	1.64E-10	Capn5
ENSMUSG00000035557.10	1.11691002	0.05486109	0.02780426	5.18997335	0.02271756	0.06326051	Krt17
ENSMUSG00000035559.10	226.397905	0.00231771	0.00345372	0.45315743	0.50083934	0.68782008	Mpv17l2
ENSMUSG00000035561.6	1307.95011	0.02091378	0.00531386	15.2063269	9.64E-05	0.00055269	Aldh1b1
ENSMUSG00000035566.8	80.7126157	0.02893379	0.00526963	28.3730375	1.00E-07	1.04E-06	Pcdh17
ENSMUSG00000035569.18	1534.29575	0.0013597	0.00295485	0.21028402	0.64654488	0.81100929	Ankrd11
ENSMUSG00000035572.17	499.532986	-0.0081462	0.00237111	11.7094947	0.00062182	0.00289457	Dcaf10
ENSMUSG00000035575.12	613.053321	-0.0059911	0.00241046	6.03684098	0.01401033	0.04237596	Utp6
ENSMUSG00000035578.17	57.6489456	0.00898282	0.00524931	2.80457844	0.09399555	0.20032339	Iqcg
ENSMUSG00000035585.17	481.037555	-0.0029866	0.00307417	0.9413133	0.33194032	0.51889876	Tsen34
ENSMUSG00000035594.11	1.3749077	0.01187296	0.03503326	0.33300937	0.56389241	0.74451222	Chrna5
ENSMUSG00000035595.7	71.718955	-0.0113929	0.00493076	5.32736299	0.02099312	0.05923939	Fam174c
ENSMUSG00000035596.15	578.552618	-0.0002937	0.00245032	0.01422484	0.90506309	0.97833964	Mboat7
ENSMUSG00000035597.20	470.752057	0.00333816	0.00321592	1.06161146	0.30284794	0.48751274	Prpf39
ENSMUSG00000035601.10	84.3317896	-0.0078718	0.00378737	4.31547292	0.03776727	0.09591675	Trmt10b
ENSMUSG00000035606.9	3.00543788	0.01406154	0.01781404	0.93473053	0.33363671	0.52051564	Ky
ENSMUSG00000035614.12	336.043941	0.00028522	0.0033118	0.00739016	0.93149342	0.99255044	Togaram1
ENSMUSG00000035620.16	205.561472	-0.0083362	0.00230445	13.0214542	0.00030794	0.00156046	Ric8b
ENSMUSG00000035621.14	618.535986	0.0010899	0.00340448	0.09934243	0.75262018	0.885175	Midn
ENSMUSG00000035623.15	532.089074	0.00509479	0.00365397	1.92323275	0.16550072	0.30958046	Rsf1
ENSMUSG00000035629.20	323.95704	-0.0015394	0.00212682	0.52359153	0.46931365	0.66037655	Rubcn
ENSMUSG00000035632.17	474.611391	-0.0039267	0.00474584	0.6888244	0.40656453	0.59822785	Cnot3
ENSMUSG00000035637.15	2906.05332	-0.0187979	0.00323039	34.3149815	4.69E-09	5.95E-08	Grhpr
ENSMUSG00000035638.15	1.43071939	0.04289856	0.02599091	3.42542995	0.06419952	0.14779024	Muc20
ENSMUSG00000035640.20	29.7565591	0.00785522	0.00706731	1.17321481	0.27874167	0.45773539	Cbarp
ENSMUSG00000035642.17	289.251373	-0.0189409	0.00354532	28.8847441	7.68E-08	8.14E-07	Aamdc
ENSMUSG00000035649.18	646.827476	0.0054646	0.00401291	1.80799198	0.1787493	0.32857584	Zcchc7
ENSMUSG00000035653.17	5.49633725	0.02505075	0.01798099	2.17417566	0.1403447	0.2734096	Lrfn5
ENSMUSG00000035666.15	447.461256	-0.0031215	0.00255292	1.49463937	0.22149803	0.386634	Gtf3c4
ENSMUSG00000035671.7	150.025166	-0.0090494	0.00343739	6.89260613	0.0086553	0.02825521	Zswim4
ENSMUSG00000035673.11	459.348922	0.00093256	0.00359413	0.06481606	0.79903979	0.91547812	Sbno2
ENSMUSG00000035674.14	1815.50886	-0.0167794	0.00562811	8.83561248	0.00295408	0.01127855	Ndufa3
ENSMUSG00000035678.9	3.03943193	0.06304115	0.01295653	20.8369792	5.00E-06	3.78E-05	Tnfsf9
ENSMUSG00000035681.8	13.1935962	0.03184131	0.01476215	4.89751017	0.02689545	0.07273057	Kcnc2
ENSMUSG00000035683.14	6.82120514	0.03321114	0.01166324	7.63391823	0.00572807	0.01987968	Melk
ENSMUSG00000035686.9	8977.10666	-0.0162556	0.00739465	4.89705405	0.02690255	0.07274042	Thrsp
ENSMUSG00000035692.8	179.629727	0.0232286	0.00591161	16.2431674	5.57E-05	0.00033839	Isg15
ENSMUSG00000035696.16	259.19369	0.0152957	0.00384977	15.492074	8.29E-05	0.00048262	Rnf38
ENSMUSG00000035697.15	205.32621	0.02694862	0.00359554	54.2041471	1.81E-13	4.18E-12	Arhgap45
ENSMUSG00000035699.9	10.8890038	-0.0085335	0.01464407	0.14955468	0.69896129	0.84929684	Slc51a
ENSMUSG00000035704.18	126.127004	-0.0050411	0.00374951	1.79878797	0.17985909	0.33038602	Alg8
ENSMUSG00000035711.6	55.1399638	0.03202209	0.00539917	32.3576849	1.28E-08	1.53E-07	Dok3
ENSMUSG00000035713.16	40.0030247	0.01787695	0.00541895	10.5473427	0.00116355	0.00501635	Usp35
ENSMUSG00000035722.16	138.686615	0.01112051	0.00294291	14.2474533	0.00016028	0.00087507	Abca7
ENSMUSG00000035725.14	127.745423	0.02063018	0.00351217	32.9402583	9.50E-09	1.15E-07	Prkx
ENSMUSG00000035726.9	672.657239	-9.91E-05	0.00217146	0.00206655	0.96374117	1	Supt16
ENSMUSG00000035735.11	27.3887864	0.02973889	0.00508981	33.5162328	7.07E-09	8.75E-08	Dagla
ENSMUSG00000035745.10	2.58918625	-0.0392873	0.0100659	15.0003396	0.00010749	0.00060974	Grin3b
ENSMUSG00000035754.9	834.509223	-0.0188109	0.00364521	26.6276507	2.47E-07	2.39E-06	Wdr18
ENSMUSG00000035757.18	1447.18779	-0.0032488	0.00276344	1.36793001	0.24216785	0.41231293	Selenoo
ENSMUSG00000035759.5	112.715228	-0.0095708	0.0029459	10.3375746	0.00130349	0.00553849	Bbs10
ENSMUSG00000035762.12	202.200812	-0.0025814	0.00500472	0.255051	0.61354076	0.78537859	Tmem161b
ENSMUSG00000035764.6	202.445195	-0.0028767	0.00197613	2.09933326	0.14736339	0.28367466	Fbxo45

ENSMUSG00000035765.11	478.506982	-0.0090606	0.0020558	19.2920847	1.12E-05	7.87E-05	Dym
ENSMUSG00000035769.10	2767.23916	-0.0007197	0.00481086	0.02184178	0.88250877	0.96537822	Xylb
ENSMUSG00000035770.9	879.399694	0.01107457	0.00548827	3.97250908	0.04624881	0.11323829	Dync1li2
ENSMUSG00000035772.12	601.245916	-0.0085055	0.00167368	25.9805416	3.45E-07	3.25E-06	Mrps2
ENSMUSG00000035773.7	7.96246665	0.01209323	0.00589706	4.21633504	0.04003655	0.10069333	Kiss1r
ENSMUSG00000035775.3	1.32214944	0.04898803	0.01920636	7.58338477	0.00589087	0.02034887	Krt20
ENSMUSG00000035776.15	565.653848	-0.0051725	0.0024389	4.44310672	0.03504243	0.09036077	Cd99l2
ENSMUSG00000035778.18	91.3381813	0.02752732	0.00509582	27.2200778	1.82E-07	1.80E-06	Ggta1
ENSMUSG00000035780.3	4841.97892	-0.013167	0.00481867	7.36926276	0.00663481	0.02257483	Ugt2a3
ENSMUSG00000035781.16	702.497861	-0.0006243	0.00260791	0.05706089	0.81120312	0.92225184	R3hdm4
ENSMUSG00000035783.10	133.970396	0.01761512	0.00806906	4.35509067	0.0368985	0.09414625	Acta2
ENSMUSG00000035785.7	0.98930787	-0.0159387	0.05683066	0.03820937	0.84502333	0.94405052	Cmtm2b
ENSMUSG00000035790.16	109.974324	-0.0003519	0.00281473	0.01559503	0.90061851	0.97601489	Cep19
ENSMUSG00000035798.15	268.093782	0.00441306	0.00265936	2.6936598	0.10074817	0.21142554	Zdhhc17
ENSMUSG00000035799.7	11.3691212	-0.0046068	0.00642705	0.5075016	0.4762224	0.66648156	Twist1
ENSMUSG00000035805.14	9.36204445	0.01883158	0.02188221	1.13681951	0.28632539	0.46699886	Mlc1
ENSMUSG00000035811.11	3955.30036	-0.0158458	0.00502308	9.88635989	0.00166508	0.00686345	Ugt2b35
ENSMUSG00000035818.15	4.62950709	0.03621311	0.0110874	9.88104471	0.0016699	0.0068797	Plekhs1
ENSMUSG00000035824.8	380.66504	0.00592082	0.00265957	4.91666223	0.02659886	0.07207728	Tk2
ENSMUSG00000035828.12	923.939662	-0.0306658	0.00509297	36.3023134	1.69E-09	2.27E-08	Pim3
ENSMUSG00000035829.14	7.05581731	0.02349112	0.00869354	6.97448571	0.008268	0.02714491	Ppp1r26
ENSMUSG00000035831.3	0.3389939	0.01119017	0.05351884	0.17717074	0.67381602	0.83120422	Krt25
ENSMUSG00000035834.12	326.821787	-0.0039516	0.00423146	0.84096874	0.35911986	0.54816211	Polr3g
ENSMUSG00000035835.15	13.4067819	0.04527298	0.00839027	25.2323333	5.08E-07	4.62E-06	Plppr3
ENSMUSG00000035836.3	7895.86441	-0.0407505	0.00567439	49.7007732	1.79E-12	3.63E-11	Ugt2b1
ENSMUSG00000035840.8	682.38173	-0.0042098	0.00368878	1.27874217	0.25813304	0.43160889	Lysmd3
ENSMUSG00000035842.11	95.575691	0.00087409	0.0035801	0.0586088	0.80870846	0.92098111	Ddx11
ENSMUSG00000035845.18	141.047755	-0.0024891	0.00366534	0.45801952	0.49855101	0.68572569	Alg12
ENSMUSG00000035847.16	348.387309	0.02762259	0.00322251	71.0062627	3.56E-17	1.32E-15	Ids
ENSMUSG00000035849.15	13.0312152	0.0382267	0.01261766	7.97505774	0.00474262	0.01689211	Krt222
ENSMUSG00000035851.15	851.091524	-0.0014847	0.00288532	0.25888349	0.61088825	0.78319347	Ythdc1
ENSMUSG00000035852.12	8.70367027	0.00981348	0.00790431	1.50775957	0.21948127	0.38408409	Misp
ENSMUSG00000035863.15	83.9977185	0.02297419	0.00614679	13.9063812	0.00019215	0.00102905	Palm
ENSMUSG00000035864.15	250.196248	0.00116838	0.00606345	0.0383698	0.84470244	0.94387021	Syt1
ENSMUSG00000035868.10	140.67266	0.00615832	0.00395704	2.36410979	0.12415472	0.24871014	Zfp983
ENSMUSG00000035873.9	154.552422	0.00544269	0.00335426	2.54326151	0.11076577	0.22743288	Pawr
ENSMUSG00000035875.16	7177.84117	-0.0124567	0.00518858	5.72857302	0.01669104	0.04902551	AI182371
ENSMUSG00000035877.18	1581.11782	-0.0079422	0.00316023	6.23382501	0.01253327	0.03848083	Zhx3
ENSMUSG00000035878.14	1621.13326	0.00043867	0.00454296	0.0087532	0.92545987	0.98916486	Hykk
ENSMUSG00000035885.6	3017.0263	-0.0102221	0.00282055	13.4628169	0.00024334	0.00126865	Cox8a
ENSMUSG00000035890.10	334.930656	-0.0064411	0.00270628	5.74066325	0.01657653	0.04873166	Rnf126
ENSMUSG00000035891.17	130.394732	0.0387542	0.00458989	66.1907371	4.09E-16	1.32E-14	Cerk
ENSMUSG00000035896.6	1.16788643	-0.0535862	0.04776412	-0.1730255	1	1	Rnase1
ENSMUSG00000035898.14	210.632205	0.00834231	0.00335393	5.98298505	0.01444453	0.0435552	Uba6
ENSMUSG00000035900.19	164.406678	0.01548319	0.00355912	18.8690252	1.40E-05	9.67E-05	Gramd4
ENSMUSG00000035901.15	621.563179	0.00832794	0.00246377	11.4050444	0.00073245	0.00334083	Dennd5a
ENSMUSG00000035910.16	74.2088708	0.00308383	0.00706247	0.18776884	0.66478012	0.82454269	Dcdc2a
ENSMUSG00000035914.12	82.9124721	-0.028704	0.00632248	21.9375572	2.82E-06	2.24E-05	Cd276
ENSMUSG00000035919.17	78.3577711	0.00873723	0.00394445	4.92853745	0.02641667	0.0716412	Bbs9
ENSMUSG00000035929.12	710.551445	0.03847918	0.00318471	140.737261	1.84E-32	5.35E-30	H2-Q4
ENSMUSG00000035930.10	3.6379868	-0.0164465	0.0102435	2.53639099	0.11124881	0.22822601	Chst4
ENSMUSG00000035933.6	489.10209	0.00388226	0.00289725	1.75434214	0.1853318	0.33799085	Cog5
ENSMUSG00000035934.17	22.0426862	0.00994928	0.00656844	2.1623219	0.14143083	0.27495483	Pknox2
ENSMUSG00000035936.7	1081.00557	-0.0063279	0.00252554	6.24452417	0.01245778	0.03832214	Aldh5a1
ENSMUSG00000035941.16	1168.36893	0.00205915	0.00240852	0.72073901	0.39590161	0.58772252	Ibtk
ENSMUSG00000035944.18	1456.32279	-0.002325	0.00325041	0.50835803	0.47585052	0.66615831	Ttc38
ENSMUSG00000035946.8	1.95330098	-0.0072019	0.1363959	-0.0025753	1	1	Gsx2
ENSMUSG00000035948.14	603.341913	0.00256154	0.00538429	0.2139259	0.64370757	0.80930101	Acss3
ENSMUSG00000035949.16	718.603641	-0.0021877	0.00173778	1.59286198	0.20691771	0.36769089	Fbxw2
ENSMUSG00000035953.15	409.658406	-0.0001582	0.00279654	0.00316819	0.95511341	1	Pip4p1
ENSMUSG00000035954.11	584.976132	-0.0024246	0.00334505	0.52546943	0.46851786	0.65965033	Dock4

ENSMUSG00000035958.4	429.21413	0.00598021	0.00237175	6.35103326	0.0117313	0.03640715	Tdp2
ENSMUSG00000035960.15	437.408235	-0.0038202	0.00287801	1.74884011	0.18602261	0.33891004	Apex1
ENSMUSG00000035964.9	6.17729519	0.01301917	0.0168406	0.79272118	0.37327857	0.56404307	Tmem59l
ENSMUSG00000035967.16	132.629281	0.02062244	0.00554348	13.0264814	0.00030712	0.00155711	Ints6l
ENSMUSG00000035969.16	358.408963	-0.0021548	0.00530251	0.15972814	0.68940694	0.84228708	Rusc2
ENSMUSG00000035984.15	2.46000207	0.01912424	0.01262242	2.27473778	0.13149736	0.26019269	Nme5
ENSMUSG00000035992.16	843.824587	0.00196229	0.00308549	0.39651046	0.52889693	0.71344369	Fnip1
ENSMUSG00000036002.13	232.683917	-0.0015019	0.00312327	0.23341144	0.62900575	0.79765764	Atosb
ENSMUSG00000036006.21	82.8629483	0.04208343	0.00563078	52.9899595	3.35E-13	7.50E-12	Ripor2
ENSMUSG00000036009.17	53.2981089	0.00593546	0.00292867	4.05205264	0.04411767	0.10890208	Mettl25
ENSMUSG00000036019.9	163.876538	-0.0044026	0.00380676	1.32577968	0.24955741	0.4210711	Tmtc2
ENSMUSG00000036022.16	50.8935302	0.00889867	0.00332312	7.18193977	0.0073641	0.02463402	Pabir2
ENSMUSG00000036023.7	176.479872	0.02154387	0.00340812	38.6418319	5.09E-10	7.35E-09	Parp2
ENSMUSG00000036026.16	1123.90915	-0.0062507	0.00251582	6.31032345	0.01200369	0.03714987	Tmem63b
ENSMUSG00000036027.14	27.8247099	0.04457533	0.00707984	40.0784908	2.44E-10	3.70E-09	Pou2af2
ENSMUSG00000036030.10	5.04102227	0.03360258	0.01084299	9.53493325	0.00201598	0.00813217	Prtg
ENSMUSG00000036036.16	15.1523957	0.00402613	0.00839414	0.22374076	0.63620431	0.8034194	Zfp57
ENSMUSG00000036040.15	199.360548	0.0272746	0.00559086	23.1000334	1.54E-06	1.29E-05	Adamtsl2
ENSMUSG00000036046.15	791.836545	0.00851273	0.0028265	8.96776517	0.00274785	0.01059864	5031439G07Rik
ENSMUSG00000036052.15	80.4980654	-0.0049005	0.00408771	1.41053171	0.23496815	0.40343089	Dnajb5
ENSMUSG00000036053.19	193.425362	0.01404606	0.0034125	16.0765145	6.08E-05	0.00036575	Fmnl2
ENSMUSG00000036054.16	216.439555	-0.0064565	0.00372527	2.98101339	0.08424651	0.18366191	Sugp2
ENSMUSG00000036057.12	297.701215	0.00505622	0.00390493	1.63045028	0.20164078	0.3605139	Ptpn23
ENSMUSG00000036061.11	196.242223	-0.010518	0.00250344	17.9003705	2.33E-05	0.00015336	Smug1
ENSMUSG00000036062.14	33.44019	0.06111147	0.0095015	39.5920637	3.13E-10	4.65E-09	Phf24
ENSMUSG00000036067.13	14.6751739	0.08828373	0.00859395	90.2478229	2.10E-21	1.26E-19	Slc2a6
ENSMUSG00000036073.19	730.553111	-0.0029553	0.00277465	1.14124967	0.28538842	0.46579277	Galt
ENSMUSG00000036078.17	2501.43516	-0.0019926	0.00379864	0.27045908	0.60302398	0.77631809	Sigmar1
ENSMUSG00000036083.16	1154.35056	-0.0167536	0.00471568	12.3666355	0.00043707	0.00212072	Slc17a3
ENSMUSG00000036086.17	58.1908149	0.00255651	0.00326169	0.61374802	0.4333801	0.62498948	Zranb3
ENSMUSG00000036087.19	832.222985	-0.0025925	0.0028829	0.78997445	0.37410785	0.56504276	Slain2
ENSMUSG00000036091.9	5.99744916	-0.0160156	0.00670709	5.4117239	0.02000195	0.05694219	Hyal3
ENSMUSG00000036093.8	2810.06922	-0.0038945	0.00251713	2.361422	0.12436878	0.24896978	Arl5a
ENSMUSG00000036095.12	11.8441192	0.03273946	0.01234973	6.62421015	0.01006017	0.03206477	Dgkb
ENSMUSG00000036097.10	398.010474	0.01177319	0.00246538	22.418518	2.19E-06	1.78E-05	Slf2
ENSMUSG00000036098.16	520.355922	0.00621749	0.00377648	2.64437598	0.10391695	0.21619346	Myrf
ENSMUSG00000036099.17	363.328312	-0.0081988	0.00313923	6.8138397	0.00904541	0.02935719	Vezt
ENSMUSG00000036103.10	1006.78773	0.00263035	0.00301657	0.73798783	0.39030621	0.58172274	Colec12
ENSMUSG00000036104.13	684.004163	-0.0085324	0.00185849	20.8563301	4.95E-06	3.75E-05	Rab3gap1
ENSMUSG00000036106.16	86.9268216	2.06E-06	0.00377552	2.93E-07	0.99956819	1	Prr5
ENSMUSG00000036109.18	11.2491103	0.03466908	0.00946088	12.6022803	0.00038528	0.00189909	Mbnl3
ENSMUSG00000036110.15	3493.78133	-0.0257442	0.00506301	25.6908144	4.01E-07	3.72E-06	Slc17a2
ENSMUSG00000036111.9	1.04668229	0.05084685	0.01643461	11.2465897	0.00079769	0.0036036	Lmo1
ENSMUSG00000036112.16	2175.89059	-0.003529	0.00252358	1.93862594	0.1638178	0.30702054	Metap2
ENSMUSG00000036114.4	209.217932	-0.0081197	0.00350235	5.4613888	0.01944119	0.0556066	Rpp25l
ENSMUSG00000036120.11	300.818524	-0.0059229	0.00365887	2.55431189	0.1099937	0.22622177	Rfxank
ENSMUSG00000036123.9	7.59058874	0.0196449	0.02166401	1.30550541	0.25321004	0.42541115	Slc9a3
ENSMUSG00000036136.10	24.5700001	0.01735928	0.0052623	10.9064197	0.00095832	0.00422878	Fam110c
ENSMUSG00000036138.17	3105.04867	-0.0086522	0.00292269	8.69951191	0.00318295	0.01200978	Acaa1a
ENSMUSG00000036139.7	2.51417559	-0.0063674	0.03765175	-0.0590183	1	1	Hoxc9
ENSMUSG00000036144.7	8.1993458	0.01289501	0.0167744	-0.5018911	1	1	Meox2
ENSMUSG00000036151.16	545.886769	0.0182064	0.00334852	29.3492048	6.04E-08	6.47E-07	Tm6sf2
ENSMUSG00000036155.14	269.056535	0.0222157	0.00334987	42.5468635	6.90E-11	1.14E-09	Mgat5
ENSMUSG00000036158.13	79.6326429	0.00023235	0.00310904	0.00561479	0.94026889	0.99699614	Prickle1
ENSMUSG00000036160.14	238.492834	-0.0050654	0.0030037	2.84554449	0.09162755	0.19625687	Surf6
ENSMUSG00000036167.17	298.959943	-0.0013015	0.00267469	0.23544965	0.62751211	0.79615388	Pphln1
ENSMUSG00000036168.16	12.7209475	-0.0233562	0.01058024	4.21257068	0.04012548	0.10088473	Ccdc38
ENSMUSG00000036169.7	7.30445487	0.1400287	0.02509208	53.3272018	2.82E-13	6.41E-12	Sostdc1
ENSMUSG00000036172.18	2.24893852	0.0265673	0.01060309	6.14220366	0.01319927	0.04022717	Cd200r3
ENSMUSG00000036180.16	853.688038	-0.001773	0.00238334	0.55367093	0.45682162	0.64872026	Gatad2a
ENSMUSG00000036181.3	975.860577	5.03E-05	0.00385473	0.00015942	0.98992611	1	H1f2

ENSMUSG00000036185.10	1.15149724	0.02802766	0.01285746	4.74143092	0.02944457	0.07827769	Sapcd1
ENSMUSG00000036186.6	5.5798838	0.02689604	0.00988645	7.27072133	0.00700877	0.02364932	Dipk1b
ENSMUSG00000036188.5	164.965596	0.00753657	0.00198138	14.377633	0.00014957	0.00082136	Ankmy2
ENSMUSG00000036192.16	6.48314513	0.00898649	0.01834856	0.38122337	0.53694923	0.72129426	Rorb
ENSMUSG00000036196.17	1.8189402	0.03391038	0.01489686	4.47139578	0.03446682	0.08921582	Slc26a8
ENSMUSG00000036197.17	349.704591	-0.0006255	0.00320906	0.03722949	0.84699855	0.94522394	Gxylt1
ENSMUSG00000036198.13	7.46605906	-0.0235048	0.01131577	4.07913637	0.04341584	0.10757497	Arhgap36
ENSMUSG00000036199.10	2027.72995	-0.0146809	0.00333177	19.7264877	8.94E-06	6.41E-05	Ndufa13
ENSMUSG00000036202.17	377.751367	-0.0012445	0.00361594	0.11549302	0.73397503	0.87260119	Rif1
ENSMUSG00000036206.13	44.620338	0.01189779	0.00492405	5.62687763	0.01768711	0.05143874	Sh3bp4
ENSMUSG00000036208.14	111.186678	-0.0026481	0.00310296	0.72355852	0.39497913	0.58657459	Nepro
ENSMUSG00000036214.15	180.958271	-0.0051304	0.00270031	3.60173732	0.05771922	0.13568879	Polr1has
ENSMUSG00000036216.6	3194.78682	-0.0221988	0.00710572	9.49252956	0.0020631	0.00830098	Leap2
ENSMUSG00000036218.17	3.98340577	0.01172955	0.02662073	0.33920724	0.56028717	0.74174524	Pdzrn4
ENSMUSG00000036223.17	4.74625925	0.03816998	0.01420593	6.68211377	0.00973851	0.03119668	Ska1
ENSMUSG00000036225.16	31.5341475	0.00449327	0.00598063	0.54317039	0.46112178	0.65228555	Kctd1
ENSMUSG00000036241.4	2238.76782	-0.0044543	0.00213924	4.336516	0.03730318	0.09492209	Ube2r2
ENSMUSG00000036242.16	22.424029	-0.00286	0.0090063	0.09124895	0.76259534	0.89200122	Armh4
ENSMUSG00000036246.15	138.121725	0.02623856	0.00497002	27.2422469	1.79E-07	1.78E-06	Gmip
ENSMUSG00000036249.17	102.505059	0.02981672	0.00306472	92.3752473	7.17E-22	4.67E-20	Rbm43
ENSMUSG00000036251.17	1.77185489	0.01128915	0.01309101	0.82750096	0.36299615	0.55254128	Trpm8
ENSMUSG00000036256.14	1469.11386	0.01554586	0.00293386	28.0041267	1.21E-07	1.24E-06	Igfbp7
ENSMUSG00000036257.16	2977.71598	-0.0001768	0.00248567	0.00487784	0.9443198	0.99935957	Pnpla8
ENSMUSG00000036264.10	2.67612859	0.0372701	0.01485755	7.46350534	0.00629623	0.02153776	Fstl4
ENSMUSG00000036270.17	323.381455	-0.0060979	0.00258706	5.56872949	0.01828402	0.05285537	Edc4
ENSMUSG00000036273.16	93.4535556	0.01105381	0.00594905	3.38137793	0.06593691	0.1509598	Lrrk2
ENSMUSG00000036275.14	2508.30229	-0.0061504	0.00202839	9.21560247	0.00239961	0.00943467	9530068E07Rik
ENSMUSG00000036278.8	544.040714	-0.0112142	0.00441727	6.30551299	0.01203631	0.03723614	Macrod1
ENSMUSG00000036281.14	152.535359	0.00560404	0.00426759	1.7265016	0.18885829	0.34285804	Snapc4
ENSMUSG00000036282.16	469.161776	-0.0037378	0.00228108	2.62060911	0.10548394	0.21877807	Naa30
ENSMUSG00000036285.11	196.482358	-0.0193279	0.00265963	52.5245049	4.25E-13	9.39E-12	Noa1
ENSMUSG00000036291.7	363.765635	-0.002383	0.00211177	1.2772078	0.25841884	0.43187606	Ap5m1
ENSMUSG00000036292.15	679.652173	-0.0020287	0.00411368	0.24207665	0.62271025	0.79240346	Gramd1c
ENSMUSG00000036295.6	25.6715896	0.00360821	0.00493627	0.52174261	0.47009928	0.66116647	Lrrn3
ENSMUSG00000036298.11	18.0463593	0.0288028	0.00778702	12.9709387	0.00031636	0.00159746	Slc2a13
ENSMUSG00000036299.6	1102.60327	-0.0022882	0.00256701	0.79519736	0.37253317	0.56327761	BC031181
ENSMUSG00000036304.15	17.4344847	0.02158525	0.00650535	10.8432434	0.00099157	0.00436002	Zdhhc23
ENSMUSG00000036306.14	8.90782699	0.02783943	0.00844768	9.90228913	0.00165073	0.00681297	Lzts1
ENSMUSG00000036309.15	4575.55916	-0.0097923	0.00263612	13.6042951	0.00022567	0.0011865	Skp1
ENSMUSG00000036315.14	153.194018	-0.007402	0.00368182	4.05536822	0.04403112	0.1087814	Polr1h
ENSMUSG00000036323.15	2055.03332	-0.0048933	0.00329777	2.17571784	0.14020408	0.27320336	Srp72
ENSMUSG00000036327.19	126.699542	-0.000375	0.00226653	0.02747029	0.86836028	0.95799033	Qsox2
ENSMUSG00000036330.13	74.6035701	-0.0009708	0.00545186	0.03037529	0.86164143	0.95394374	Slc18a1
ENSMUSG00000036333.12	1212.4187	0.00450149	0.00311738	2.02469819	0.15475967	0.29418628	Kidins220
ENSMUSG00000036334.13	42.1317978	0.02152412	0.00513394	16.9686725	3.80E-05	0.00023959	Igsf10
ENSMUSG00000036339.19	473.124602	-0.0023676	0.00227078	1.0595497	0.30331791	0.4880097	Tmem260
ENSMUSG00000036352.17	279.880297	-0.0142445	0.00357899	16.09964	6.01E-05	0.00036195	Ubac1
ENSMUSG00000036353.14	49.311303	0.05222309	0.00573485	77.7399348	1.18E-18	5.14E-17	P2ry12
ENSMUSG00000036356.16	24.3610752	0.0058129	0.00527805	1.15764548	0.28195438	0.46171464	Csgalnact1
ENSMUSG00000036362.3	86.8690535	0.0504057	0.00507286	92.8142623	5.74E-22	3.81E-20	P2ry13
ENSMUSG00000036368.9	1119.97373	-0.0047	0.00369925	1.61479056	0.2038197	0.36358278	Rmdn2
ENSMUSG00000036371.7	3996.65744	0.00039094	0.00245773	0.02511804	0.87407328	0.96088988	Serbp1
ENSMUSG00000036372.15	524.222893	-0.0119699	0.00366022	10.7420574	0.00104728	0.00457428	Tmem258
ENSMUSG00000036376.4	179.047696	-0.0080284	0.00262977	9.39863737	0.00217147	0.00867925	Abt1
ENSMUSG00000036377.21	11.9907175	-0.004394	0.01462928	0.01155897	0.9143823	0.98312004	Cracd
ENSMUSG00000036381.14	36.9669307	0.03335146	0.00543678	36.2286709	1.75E-09	2.35E-08	P2ry14
ENSMUSG00000036390.9	147.371893	0.02140668	0.00588577	12.0235317	0.00052533	0.0025016	Gadd45a
ENSMUSG00000036391.17	2140.91346	-0.0033992	0.00363901	0.85759418	0.35441282	0.54303825	Sec24a
ENSMUSG00000036395.16	6.58817154	0.03018304	0.01371961	4.69992626	0.03016391	0.07983908	Glb1l2
ENSMUSG00000036398.14	476.057411	-0.004705	0.00304344	2.35119784	0.12518678	0.25031006	Ppp1r11
ENSMUSG00000036402.14	1039.84317	0.00209355	0.00225923	0.8502657	0.35647718	0.54520164	Gng12

ENSMUSG00000036403.16	104.698436	0.00159165	0.00282271	0.31412482	0.57516	0.75386306	Cep135
ENSMUSG00000036411.11	6.49928251	0.00441623	0.01081164	0.15987821	0.68926867	0.84224904	Matcap2
ENSMUSG00000036412.6	1.33187723	0.02226142	0.01330282	3.00957506	0.08277399	0.18093673	Arsi
ENSMUSG00000036422.11	2.29446007	0.02133629	0.02567745	1.17518112	0.27833921	0.45731497	Pcdh8
ENSMUSG00000036427.6	3257.37649	-0.0023422	0.00280247	0.7040281	0.4014335	0.59302333	Gpi1
ENSMUSG00000036430.9	123.724702	-0.0010219	0.00315384	0.10584999	0.74491925	0.87971637	Tbcc
ENSMUSG00000036432.9	288.183742	0.00038394	0.00280964	0.01890236	0.89064677	0.97031506	Siah2
ENSMUSG00000036435.14	212.442597	0.00775472	0.00322348	5.69988817	0.01696599	0.04968462	Exoc1
ENSMUSG00000036437.7	4.79237543	0.0297653	0.00931426	9.79052297	0.00175414	0.00719406	Npy1r
ENSMUSG00000036438.15	2631.30784	0.00351685	0.00260845	1.80897255	0.17863153	0.32844079	Calm2
ENSMUSG00000036442.9	253.921425	-0.0052119	0.00194815	7.18812795	0.00733875	0.02458341	Thap11
ENSMUSG00000036446.6	224.536682	0.01427984	0.00599938	5.42067597	0.01989965	0.05667122	Lum
ENSMUSG00000036450.5	467.552778	-0.0117001	0.00254626	21.0286719	4.52E-06	3.45E-05	Hif1an
ENSMUSG00000036452.19	368.717638	0.01256627	0.00451314	7.24995485	0.00709028	0.02386973	Arhgap26
ENSMUSG00000036459.16	28.0128348	0.01110846	0.00476344	5.36603498	0.0205326	0.05826298	Wtip
ENSMUSG00000036461.17	492.603628	0.01078645	0.00262858	16.5000688	4.86E-05	0.00029943	Elf1
ENSMUSG00000036466.18	7.52882599	-0.002262	0.01039108	0.04375669	0.83430694	0.93682555	Megf11
ENSMUSG00000036469.17	94.8095278	0.06590535	0.00631223	96.4714418	9.05E-23	6.51E-21	Marchf1
ENSMUSG00000036473.17	438.146791	0.01751902	0.00355731	24.1414351	8.95E-07	7.75E-06	Tbc1d24
ENSMUSG00000036478.9	1000.90877	0.01197236	0.0033344	12.7372626	0.00035844	0.00178321	Btg1
ENSMUSG00000036492.13	81.789232	0.00329136	0.00422685	0.5975371	0.43951928	0.6314586	Rnf39
ENSMUSG00000036499.10	968.715927	-0.004559	0.00338288	1.78837368	0.18112444	0.33208963	Eea1
ENSMUSG00000036501.8	415.174353	0.00266884	0.00359199	0.54585186	0.46001759	0.65153276	Fam13b
ENSMUSG00000036502.15	14.5813329	0.04070476	0.00895156	19.2542567	1.14E-05	8.01E-05	Tmem255a
ENSMUSG00000036503.14	3569.41166	0.00356878	0.00211065	2.84572365	0.09161734	0.19625687	Rnf13
ENSMUSG00000036504.7	378.054688	-0.0020468	0.00450637	0.20516617	0.65058278	0.81356479	Phpt1
ENSMUSG00000036510.18	2.48465689	0.01243003	0.02175611	0.42045735	0.51670893	0.70315582	Cdh8
ENSMUSG00000036513.16	502.345405	-0.0074201	0.00348841	4.63102556	0.03139879	0.08255176	Commd2
ENSMUSG00000036526.9	20.4424589	0.08554108	0.00770859	110.356834	8.18E-26	8.58E-24	Card11
ENSMUSG00000036528.17	670.024667	-0.0040734	0.00423322	0.9029198	0.34200001	0.52991803	Ppfibp2
ENSMUSG00000036529.18	649.588567	-0.0084995	0.00375969	5.07184199	0.02431753	0.06687888	Sbf1
ENSMUSG00000036533.10	60.0649199	0.02195306	0.00584028	13.3454994	0.00025904	0.00133958	Cdc42ep3
ENSMUSG00000036534.7	361.306076	0.00763762	0.00215024	12.6779324	0.00037	0.00183027	Slc38a7
ENSMUSG00000036537.7	21.4420683	-0.0032851	0.00351447	0.8618986	0.35320793	0.54183819	Rnf113a1
ENSMUSG00000036545.10	124.605693	0.00865206	0.00428552	4.03082969	0.04467596	0.11000389	Adamts2
ENSMUSG00000036550.17	1869.65331	-0.0005079	0.0026843	0.03501611	0.85156191	0.94843175	Cnot1
ENSMUSG00000036552.20	258.374468	0.00677741	0.00291167	5.34552074	0.02077558	0.05879364	Ermard
ENSMUSG00000036553.17	128.384213	0.01230494	0.00344124	12.7146738	0.0003628	0.00180108	Sh3tc1
ENSMUSG00000036555.15	122.653183	0.00228986	0.00384702	0.35501033	0.55129051	0.73362323	Iqce
ENSMUSG00000036560.15	7.96380616	0.00491084	0.00960391	0.2299398	0.63156847	0.79957992	Lgi4
ENSMUSG00000036561.10	507.390269	-0.0014719	0.00206637	0.49374848	0.48225994	0.67146298	Ppp6r2
ENSMUSG00000036564.18	54.6501172	0.01680825	0.01031483	2.44503187	0.11789749	0.23883279	Ndrg4
ENSMUSG00000036565.8	194.127482	0.0246269	0.00459034	28.7339969	8.30E-08	8.74E-07	Ttyh3
ENSMUSG00000036568.8	271.009143	0.00495194	0.0035395	1.95040669	0.16254303	0.30518548	Bicral
ENSMUSG00000036570.15	2871.11347	-0.0098211	0.00432584	5.17858346	0.02286696	0.06361211	Fxyd1
ENSMUSG00000036572.17	1132.38963	-0.0025485	0.00458344	0.30605855	0.58010895	0.75798846	Upf3b
ENSMUSG00000036578.8	3.37960591	-0.0001591	0.01013355	7.89E-05	0.99291071	1	Fxyd7
ENSMUSG00000036580.16	637.979638	-0.003513	0.00233124	2.22025456	0.1362106	0.26708424	Spart
ENSMUSG00000036585.17	1826.23246	-0.0185676	0.00349447	28.5828651	8.98E-08	9.41E-07	Fgf1
ENSMUSG00000036587.14	4.67172456	0.0613892	0.01118603	28.0032345	1.21E-07	1.24E-06	Fut7
ENSMUSG00000036591.17	852.459709	0.00050842	0.00409247	0.01499371	0.90254358	0.97702372	Arhgap21
ENSMUSG00000036594.16	1218.1497	0.07745933	0.00572869	165.659812	6.56E-38	3.60E-35	H2-Aa
ENSMUSG00000036596.7	15.3517686	0.02625663	0.00567353	21.1180679	4.32E-06	3.31E-05	Cpz
ENSMUSG00000036598.5	3.32546581	-0.0071709	0.02688285	0.02891127	0.86498407	0.95588784	Ccdc113
ENSMUSG00000036599.11	26.5654774	0.02234069	0.00584144	14.2286757	0.00016188	0.00088282	Chst12
ENSMUSG00000036602.15	1.8365998	0.0031351	0.02635608	0.04511365	0.83179519	0.93526223	Alx1
ENSMUSG00000036606.18	3242.86847	-0.0081248	0.00391526	4.24557655	0.03935271	0.09922738	Plxnb2
ENSMUSG00000036611.6	413.690624	0.01678217	0.00336834	24.694924	6.72E-07	5.96E-06	Eepd1
ENSMUSG00000036613.9	161.08073	-0.0010472	0.00307756	0.11555906	0.73390187	0.8725582	Eipr1
ENSMUSG00000036615.8	169.587211	-0.004696	0.00378336	1.56211296	0.21135611	0.37352355	Rfxap
ENSMUSG00000036617.18	267.528655	0.01287677	0.00366629	12.2532732	0.00046444	0.00223969	Etl4

ENSMUSG00000036620.15	508.051862	0.0070488	0.00295535	5.75923186	0.01640224	0.04830357	Mgat4b
ENSMUSG00000036622.16	350.946408	0.01369792	0.00332491	16.9058606	3.93E-05	0.00024699	Atp13a2
ENSMUSG00000036632.10	618.24835	-2.99E-05	0.00225884	0.00017629	0.9894064	1	Alg5
ENSMUSG00000036634.16	11.1678777	0.04645534	0.00852325	26.4480341	2.71E-07	2.60E-06	Mag
ENSMUSG00000036636.11	711.244633	-0.0025508	0.00398129	0.4047141	0.52466469	0.71013091	Clcn7
ENSMUSG00000036639.13	345.177478	-0.0102404	0.00457593	4.97248719	0.02575359	0.0701981	Nudt1
ENSMUSG00000036641.10	55.7641574	0.04294338	0.00534701	58.2135347	2.35E-14	6.14E-13	Ccdc148
ENSMUSG00000036644.16	996.280648	0.00104769	0.00234941	0.19608366	0.65790075	0.81946531	Tbc1d9b
ENSMUSG00000036646.14	618.787618	0.00195821	0.00188828	1.0700012	0.30094518	0.48532202	Man1b1
ENSMUSG00000036647.6	1.68485703	-0.013747	0.02919271	0.27258671	0.60160184	0.7752629	Or6b9
ENSMUSG00000036655.9	574.529971	0.01214594	0.00308806	15.2407641	9.46E-05	0.00054376	Colec11
ENSMUSG00000036661.15	123.607354	0.01838607	0.00447263	16.7924229	4.17E-05	0.0002613	Dennd3
ENSMUSG00000036667.15	92.316796	0.01201978	0.0040725	8.69986715	0.00318233	0.01200936	Tcaf1
ENSMUSG00000036672.6	35.6837699	0.01335134	0.00518299	6.58349412	0.01029287	0.03267396	Cenpt
ENSMUSG00000036676.15	212.345417	0.00937637	0.00388102	5.66124767	0.01734378	0.05059679	Tmtc3
ENSMUSG00000036678.9	151.852577	-0.0013443	0.00418533	0.10359677	0.74755533	0.88163609	Aaas
ENSMUSG00000036686.17	210.355377	0.00173196	0.00384462	0.20507119	0.65065829	0.81357294	Cc2d1a
ENSMUSG00000036687.14	247.417812	0.00101957	0.00420176	0.05766637	0.8102231	0.92198917	Tmem184a
ENSMUSG00000036693.13	350.053856	-0.0043829	0.00278892	2.4581334	0.11691765	0.23733709	Nop14
ENSMUSG00000036698.12	685.455118	0.0061211	0.00425765	1.99255582	0.1580739	0.29891213	ago-02
ENSMUSG00000036699.13	3.93733663	0.03476395	0.01724125	4.14891767	0.04166115	0.10400294	Zcchc12
ENSMUSG00000036707.17	1285.9655	0.00169121	0.00162941	1.05890324	0.30346546	0.48813588	Cab39
ENSMUSG00000036712.17	504.443408	0.00572006	0.00241485	5.49511158	0.01906971	0.05469313	Cyld
ENSMUSG00000036718.18	41.346329	0.01979524	0.0047316	17.2229823	3.32E-05	0.00021149	Micall2
ENSMUSG00000036721.15	123.334105	-0.0058017	0.00204917	7.92540348	0.00487455	0.01730319	Zscan12
ENSMUSG00000036733.17	562.285578	-0.0051049	0.00216566	5.60249484	0.01793492	0.05202472	Rbm42
ENSMUSG00000036737.13	517.809761	-0.0054226	0.00180687	8.86760438	0.00290276	0.01110895	Oxsr1
ENSMUSG00000036743.5	20.4921929	0.00541164	0.00593068	0.81063165	0.36793357	0.55829138	Psma8
ENSMUSG00000036744.14	10.3902234	0.16048071	0.01077289	179.951795	4.97E-41	3.66E-38	Or2ag2b
ENSMUSG00000036745.16	60.03356	0.00681721	0.00604356	1.24495785	0.26451769	0.43954588	Ttll7
ENSMUSG00000036748.17	281.634924	0.00170417	0.00351997	0.23649563	0.62674869	0.79548483	Cuedc2
ENSMUSG00000036751.8	3952.57352	-0.0168751	0.0036982	21.0353519	4.51E-06	3.44E-05	Cox6b1
ENSMUSG00000036752.5	2292.62508	-0.009065	0.00298653	9.05854885	0.00261469	0.01016151	Tubb4b
ENSMUSG00000036760.8	11.7835352	0.02042715	0.0190733	1.41002348	0.2350525	0.40348968	Kcnk9
ENSMUSG00000036764.13	313.013699	0.00664545	0.00656144	0.95477022	0.32850804	0.51554932	Dnajc12
ENSMUSG00000036766.13	15.1186233	-0.0005295	0.01601474	-0.0059592	1	1	Dner
ENSMUSG00000036768.7	15.9021312	0.02974542	0.01074599	6.92188538	0.00851472	0.02785882	Kif15
ENSMUSG00000036769.15	93.421651	0.01135558	0.00309215	12.9573788	0.00031866	0.00160735	Wdr44
ENSMUSG00000036775.14	3573.70139	-0.0081562	0.00314293	6.64740411	0.00993004	0.03171427	Decr2
ENSMUSG00000036777.9	34.7476086	0.00884472	0.00849725	1.07923904	0.29886785	0.4827818	Anln
ENSMUSG00000036779.13	766.420845	-0.0006495	0.00223967	0.08305077	0.7732049	0.89876353	Tent4b
ENSMUSG00000036781.14	6696.58963	-0.0094982	0.00384033	6.12106471	0.01335803	0.04063226	Rps27l
ENSMUSG00000036782.14	145.660765	0.00825311	0.00360044	5.2925861	0.02141639	0.0602202	Klhl13
ENSMUSG00000036790.6	2.86403109	0.00644136	0.01959322	0.15630997	0.69257681	0.84453906	Slitrk2
ENSMUSG00000036792.13	189.653684	-0.0008186	0.00410188	0.0389965	0.8434556	0.94278678	Mbd5
ENSMUSG00000036800.9	8.19642451	0.01678807	0.01601284	1.34790356	0.24564495	0.4166739	Fam135b
ENSMUSG00000036805.14	1.65091391	0.0297141	0.01105721	7.58668661	0.00588009	0.02031603	Noxa1
ENSMUSG00000036810.17	307.816042	0.00112616	0.00207029	0.29364185	0.58789614	0.76468971	Cnep1r1
ENSMUSG00000036813.12	1226.35648	-0.0126055	0.00487014	6.68576913	0.00971856	0.03115275	Entpd8
ENSMUSG00000036814.14	1.73784329	0.03833116	0.02059108	3.5054578	0.06116694	0.1421503	Slc6a20a
ENSMUSG00000036815.18	16.6714258	0.04834579	0.0154022	10.1444205	0.00144739	0.00607547	Dpp10
ENSMUSG00000036816.5	1.99963694	-8.55E-05	0.0316979	0.00198081	0.96450086	1	Atoh7
ENSMUSG00000036817.15	280.57054	0.00748595	0.00290234	6.60782446	0.01015316	0.03231312	Sun1
ENSMUSG00000036819.15	152.98065	-0.0080415	0.00218086	13.6226474	0.00022347	0.00117679	Jmjd4
ENSMUSG00000036820.13	288.02604	0.00050901	0.00451707	0.012814	0.90987284	0.98081631	Amdhd2
ENSMUSG00000036822.7	635.689062	0.00110626	0.00285867	0.14655639	0.70184816	0.85126971	Topors
ENSMUSG00000036825.13	182.619213	0.00329062	0.00298726	1.2108554	0.27116278	0.44807417	Ssx2ip
ENSMUSG00000036826.15	5.88216049	-0.0128098	0.022149	0.36040793	0.54827977	0.73101903	Igflr1
ENSMUSG00000036832.6	2.50224119	0.03786775	0.01325535	7.55212054	0.00599395	0.02064447	Lpar3
ENSMUSG00000036833.17	3373.9075	-0.0169405	0.00398102	17.7842851	2.47E-05	0.00016188	Pnpla7
ENSMUSG00000036834.18	3.23969234	0.04277621	0.01347806	9.50824271	0.00204551	0.00824039	Plch1

ENSMUSG00000036835.8	647.331735	-0.0020756	0.00294357	0.50405089	0.47772558	0.66783046	Psenen
ENSMUSG00000036840.6	127.757876	0.00938008	0.00228274	16.6403039	4.52E-05	0.00028049	Siah1a
ENSMUSG00000036845.13	114.850754	0.00120481	0.0038869	0.09518018	0.75769212	0.88865869	Lin37
ENSMUSG00000036850.5	623.346078	-0.0105753	0.00380618	7.75610444	0.00535313	0.01875225	Mrpl41
ENSMUSG00000036853.6	1.62825501	0.05513421	0.01854414	8.06629922	0.00450961	0.01619177	Mcoln3
ENSMUSG00000036854.15	294.612217	-0.0179501	0.0056228	9.91725109	0.00163737	0.00676813	Hspb6
ENSMUSG00000036856.5	50.3380401	0.0259425	0.0049146	27.7210793	1.40E-07	1.42E-06	Wnt4
ENSMUSG00000036860.15	293.480333	-0.0144826	0.00285947	26.0343386	3.35E-07	3.16E-06	Mrpl55
ENSMUSG00000036862.16	69.220131	-0.0029044	0.00426418	0.46976255	0.49309643	0.68064448	Dchs1
ENSMUSG00000036863.13	124.168207	-0.008347	0.00462931	3.2586744	0.07104654	0.16012457	Syde2
ENSMUSG00000036864.17	47.1319805	-0.0074578	0.00571013	1.63808636	0.20058823	0.3591014	Proser3
ENSMUSG00000036867.8	127.507851	0.02109356	0.00329558	39.8660063	2.72E-10	4.10E-09	Smad6
ENSMUSG00000036872.17	7.95256979	0.00971188	0.00997517	0.96340773	0.32632979	0.51308676	Abcc12
ENSMUSG00000036873.14	145.544749	-0.0016561	0.00330174	0.25267843	0.6151954	0.78670943	2410004B18Rik
ENSMUSG00000036875.16	207.519328	-0.0046832	0.00330192	1.98207754	0.15917171	0.30036195	Dna2
ENSMUSG00000036879.16	428.542508	-0.0135064	0.00253629	27.6716531	1.44E-07	1.46E-06	Phkb
ENSMUSG00000036880.11	13484.3996	-0.0190311	0.00334797	32.169586	1.41E-08	1.67E-07	Acaa2
ENSMUSG00000036882.8	16.2698608	0.00983458	0.00887333	1.15156663	0.28322144	0.46324971	Arhgap33
ENSMUSG00000036885.15	763.603425	-0.0139672	0.00587765	5.61945318	0.01776219	0.05159983	Arhgef26
ENSMUSG00000036887.6	1214.391	0.04601764	0.00543798	67.3752607	2.24E-16	7.44E-15	C1qa
ENSMUSG00000036890.14	136.918066	0.01047219	0.0038513	7.09182013	0.00774366	0.02567719	Gtdc1
ENSMUSG00000036892.13	2808.80813	-0.0123989	0.00490583	6.41750083	0.01130011	0.03536682	Prodh2
ENSMUSG00000036893.19	537.393143	-0.008446	0.00297889	8.05358737	0.00454136	0.01628219	Ehmt1
ENSMUSG00000036894.4	91.5637192	0.04821595	0.00470509	98.0853568	4.01E-23	2.96E-21	Rap2b
ENSMUSG00000036896.6	1115.01808	0.04557841	0.00482902	83.0943272	7.82E-20	4.02E-18	C1qc
ENSMUSG00000036898.17	183.042338	0.0066588	0.00440186	2.26672323	0.13217911	0.26123484	Zfp157
ENSMUSG00000036902.12	23.6085654	-0.0205364	0.00892336	5.04540679	0.02469131	0.0677331	Neto2
ENSMUSG00000036904.7	255.329374	-0.0017157	0.00442313	0.14961516	0.6989034	0.84927025	Fzd8
ENSMUSG00000036905.9	1280.11861	0.0515704	0.00530266	87.5405175	8.26E-21	4.67E-19	C1qb
ENSMUSG00000036908.18	761.452629	0.02213312	0.00301859	52.6924373	3.90E-13	8.66E-12	Unc93b1
ENSMUSG00000036913.16	1.73874713	-0.004403	0.01859431	-0.0465877	1	1	Trim67
ENSMUSG00000036916.14	120.947778	0.01340533	0.0035314	14.3295132	0.00015344	0.00084047	Zfp280c
ENSMUSG00000036918.17	888.34991	0.00225198	0.00418918	0.29817149	0.58503101	0.76201646	Ttc7
ENSMUSG00000036923.13	5.57481792	0.00909231	0.00829892	1.12958138	0.28786465	0.46886096	Stox1
ENSMUSG00000036928.15	13.3350083	-0.0158473	0.01293379	1.38814833	0.23871805	0.40811858	Stag3
ENSMUSG00000036931.16	10.2039343	0.0579201	0.00730478	59.8330797	1.03E-14	2.83E-13	Nfkbid
ENSMUSG00000036932.15	1742.01705	-0.0109998	0.00241184	20.8928812	4.86E-06	3.68E-05	Aifm1
ENSMUSG00000036934.10	204.951079	-0.0071007	0.00303308	5.43904749	0.0196914	0.05618697	4921524J17Rik
ENSMUSG00000036938.18	2.93297295	-0.0381462	0.10664877	0.0881292	NA	NA	Try5
ENSMUSG00000036940.16	258.979521	0.0005892	0.00230212	0.06451401	0.79949858	0.9156093	Kdm1a
ENSMUSG00000036941.3	179.130178	0.00383581	0.00244108	2.42543895	0.1193798	0.24121408	Elac1
ENSMUSG00000036943.10	164.815832	0.04226416	0.00446623	84.4178037	4.01E-20	2.11E-18	Rab8b
ENSMUSG00000036944.7	53.3010456	0.05035332	0.00474629	103.631637	2.44E-24	2.13E-22	Tmem71
ENSMUSG00000036948.18	198.601213	0.00546254	0.00401598	1.82513063	0.17670378	0.32575701	Trappc14
ENSMUSG00000036949.17	3.87085856	0.03287372	0.01236101	6.74336901	0.00940968	0.03035238	Slc39a12
ENSMUSG00000036955.17	261.266995	-0.000107	0.00234204	0.00208476	0.96358186	1	Kifbp
ENSMUSG00000036957.9	161.558914	-0.0090211	0.0052239	2.81439074	0.09342236	0.199354	Lrfn3
ENSMUSG00000036959.16	192.721822	-0.0034669	0.00411734	0.69139025	0.40569191	0.59733125	Bcorl1
ENSMUSG00000036961.6	0.73821256	0.01687224	0.02962164	0.61327965	0.43355563	0.62512795	Wnt8b
ENSMUSG00000036964.15	3.27849418	-0.0107879	0.01465394	0.39128153	0.53162655	0.71630641	Trim17
ENSMUSG00000036966.16	308.469831	-0.0001804	0.0029961	0.0035731	0.95233448	1	Spryd3
ENSMUSG00000036968.16	94.4898474	0.00152772	0.00264894	0.32869793	0.5664268	0.74642373	Cnpy4
ENSMUSG00000036972.15	1.59439789	0.01265998	0.02296208	0.33698088	0.56157712	0.74269012	Zic4
ENSMUSG00000036975.6	165.814843	-0.0048052	0.00229025	4.31370343	0.03780657	0.09600622	Tmem177
ENSMUSG00000036977.9	103.9289	0.00055869	0.00554407	0.0100747	0.92004836	0.98659971	Anapc10
ENSMUSG00000036980.14	277.310587	-0.0109063	0.00219075	24.8223369	6.29E-07	5.62E-06	Taf6
ENSMUSG00000036983.8	148.534799	-0.0075194	0.00237146	10.0226608	0.00154626	0.00644006	Tfb1m
ENSMUSG00000036985.13	1627.21154	-0.0028035	0.00340806	0.67255036	0.41216385	0.60355329	Zdhhc9
ENSMUSG00000036986.17	377.437781	0.01721462	0.00347198	24.6833129	6.76E-07	5.99E-06	Pml
ENSMUSG00000036989.17	199.348741	0.00313734	0.00366483	0.74104582	0.38932607	0.58084918	Trim3
ENSMUSG00000036990.14	765.554591	-0.0048751	0.00283907	2.90285351	0.0884229	0.19084393	Otud4

ENSMUSG00000036992.11	164.269895	-0.0038269	0.00332297	1.33671829	0.24761354	0.41889114	Nxt1
ENSMUSG00000036995.8	170.06701	-0.0022171	0.00342717	0.43357719	0.51023888	0.69692461	Asap3
ENSMUSG00000037001.11	122.318947	0.00877942	0.00298171	8.58378616	0.00339169	0.01269786	Zfp39
ENSMUSG00000037003.17	1075.26248	0.00662863	0.00442221	2.18373832	0.13947532	0.27200801	Tns2
ENSMUSG00000037005.17	1.86753097	-0.0052305	0.02281492	-0.1042452	1	1	Xpnpep2
ENSMUSG00000037007.18	156.826028	0.0055907	0.00428475	1.66059321	0.19752325	0.35468469	Zfp113
ENSMUSG00000037010.8	10.8619048	0.0887579	0.01507504	31.9485593	1.58E-08	1.86E-07	Apln
ENSMUSG00000037012.19	138.164752	0.02546336	0.00541795	20.8270392	5.03E-06	3.80E-05	Hk1
ENSMUSG00000037013.18	639.777638	0.00018228	0.00139754	0.01695348	0.89640382	0.97360161	Ss18
ENSMUSG00000037016.12	5.93452024	-0.0185203	0.01152613	1.72533591	0.18900764	0.3430085	Frem2
ENSMUSG00000037017.16	269.909597	-0.0011982	0.00164083	0.53847127	0.46306697	0.6542189	Zscan21
ENSMUSG00000037020.17	45.9498062	-0.0111716	0.0061584	3.2468212	0.07156212	0.161148	Wdr62
ENSMUSG00000037022.4	384.325201	-0.0179726	0.00249049	51.5751035	6.89E-13	1.48E-11	Mmaa
ENSMUSG00000037025.12	477.915845	-0.0060406	0.00492289	1.4576565	0.22730336	0.39359614	Foxa2
ENSMUSG00000037029.9	239.95903	-0.0020989	0.0033211	0.40182068	0.52615048	0.71124822	Zfp146
ENSMUSG00000037031.11	105.804054	0.02117345	0.00455643	21.3958597	3.74E-06	2.90E-05	Tspan15
ENSMUSG00000037032.18	38.7400665	0.00065644	0.00662393	0.00941742	0.92269195	0.98804196	Apbb1
ENSMUSG00000037033.10	0.44355127	0.00102697	0.03556271	0.01609345	0.89905122	0.97523429	Clca3b
ENSMUSG00000037035.6	53.3715486	0.00752449	0.00578636	1.57104339	0.21005555	0.37190465	Inhbb
ENSMUSG00000037049.10	828.069259	-0.0094673	0.00231449	16.7606724	4.24E-05	0.00026507	Smpd1
ENSMUSG00000037053.7	21656.6249	-0.001844	0.00504902	0.13416281	0.71415435	0.85920732	Azgp1
ENSMUSG00000037058.17	1537.09371	-0.009011	0.00209302	18.4674251	1.73E-05	0.00011717	Paip2
ENSMUSG00000037060.4	19.4487449	0.00239291	0.00636903	0.14010256	0.70817908	0.85556071	Cavin3
ENSMUSG00000037062.14	1424.2379	0.0108215	0.00345685	9.44050041	0.00212246	0.00850662	Sh3glb1
ENSMUSG00000037070.11	292.481252	0.00129951	0.00217769	0.35266916	0.55260606	0.73479347	Rbmxl1
ENSMUSG00000037071.4	260706.379	0.01185202	0.00627842	3.34724969	0.0673174	0.15340562	Scd1
ENSMUSG00000037072.17	5598.03739	-0.0053582	0.00272547	3.85814658	0.04950499	0.11959161	Selenof
ENSMUSG00000037075.8	814.480268	-0.0055659	0.00195983	8.07749619	0.00448183	0.01611165	Rnf139
ENSMUSG00000037085.9	78.6046565	-0.0048284	0.00282183	2.9064495	0.08822591	0.19052337	Trmt12
ENSMUSG00000037089.12	733.990426	-0.0012106	0.00298543	0.16884042	0.68114442	0.83635131	Slc35b2
ENSMUSG00000037095.9	7119.84538	0.00423732	0.00607939	0.47936346	0.48871078	0.67691923	Lrg1
ENSMUSG00000037098.19	321.737971	0.00334954	0.00304428	1.2053783	0.27224937	0.44960606	Rab11fip3
ENSMUSG00000037103.9	77.1484558	0.00936643	0.00386605	5.84538745	0.01561793	0.04638769	Dcaf15
ENSMUSG00000037104.10	144.006819	0.01259884	0.00338734	13.7905631	0.00020436	0.00108682	Socs5
ENSMUSG00000037106.9	48.6751968	0.0396067	0.01599018	6.43351817	0.01119865	0.0350912	Fer1l6
ENSMUSG00000037108.14	61.279221	0.00119855	0.00453967	0.06833322	0.79377925	0.91233292	Zcwpw1
ENSMUSG00000037110.21	880.279802	-0.0118671	0.0038005	9.60182144	0.00194384	0.00788571	Ralgapa2
ENSMUSG00000037111.10	206.247752	0.00526145	0.00454982	1.28945907	0.25614766	0.42919034	Setd7
ENSMUSG00000037112.17	182.067394	0.00253143	0.00365503	0.47985277	0.48848901	0.67677995	Sik2
ENSMUSG00000037119.5	1484.41354	0.00239824	0.00243923	0.94625387	0.33067468	0.51758556	Fam91a1
ENSMUSG00000037126.17	131.488293	0.00236077	0.00949348	0.0566517	0.81186854	0.92264605	Psd
ENSMUSG00000037134.18	222.534071	0.00483295	0.00175235	7.57086686	0.00593192	0.02045772	Prmt9
ENSMUSG00000037138.19	16.068464	0.06111778	0.00744046	61.9600181	3.51E-15	1.01E-13	Aff3
ENSMUSG00000037139.16	10.1285218	0.03837236	0.00935078	15.973183	6.42E-05	0.00038479	Myom3
ENSMUSG00000037143.18	2.9059348	0.00848684	0.01375754	0.45663121	0.4992026	0.68642835	Cfap61
ENSMUSG00000037145.15	1.45676006	0.01270578	0.02454442	0.43127357	0.51136469	0.69810829	Lypd8l
ENSMUSG00000037148.9	79.8484614	0.03162645	0.00443883	48.2861019	3.68E-12	7.23E-11	Arhgap10
ENSMUSG00000037149.11	1312.40239	0.00244541	0.00304939	0.61868063	0.431538	0.62301862	Ddx1
ENSMUSG00000037151.9	120.701042	0.00361082	0.00315845	1.30946828	0.25249095	0.42461686	Lrrc20
ENSMUSG00000037152.12	1109.58843	-0.0174313	0.00480173	13.0511644	0.0003031	0.0015387	Ndufc1
ENSMUSG00000037157.9	58.0714883	0.01541254	0.00555923	7.76428354	0.00532894	0.01867307	Il22ra1
ENSMUSG00000037166.6	50.1695344	0.01468898	0.00534656	7.48156224	0.0062334	0.0213445	Ppp1r14a
ENSMUSG00000037169.16	22.5206431	-0.0151792	0.00691462	4.81371593	0.0282341	0.07566521	Mycn
ENSMUSG00000037171.7	8.15916549	0.03329643	0.00664309	24.1385422	8.96E-07	7.76E-06	Nodal
ENSMUSG00000037172.15	209.133389	-0.0021908	0.00374119	0.34586279	0.5564645	0.73859154	Dennd11
ENSMUSG00000037174.19	481.301595	-0.0007294	0.00248367	0.08460274	0.77115419	0.8974886	Elf2
ENSMUSG00000037185.10	12.5589106	0.05494502	0.00875875	37.5200379	9.05E-10	1.26E-08	Krt80
ENSMUSG00000037188.8	4.06788932	0.02605508	0.00764478	11.3620998	0.00074958	0.00340976	Grhl3
ENSMUSG00000037190.13	143.871996	-0.0040337	0.00343403	1.39294267	0.23790878	0.40708913	Cyb561d2
ENSMUSG00000037196.7	6.44511841	0.04254745	0.01260526	10.7946393	0.00101794	0.00446248	Pacrg
ENSMUSG00000037197.12	786.072303	0.00262357	0.00241878	1.17813866	0.27773525	0.45659547	Rbm17

ENSMUSG00000037202.7	8.55148828	0.08715109	0.00829807	100.527692	1.17E-23	9.18E-22	Prf1
ENSMUSG00000037204.8	535.16288	-0.0068524	0.00337121	4.13076379	0.04211034	0.10490225	Atg101
ENSMUSG00000037206.16	81.3700451	0.01004741	0.00493634	4.02516734	0.04482617	0.11030465	Islr
ENSMUSG00000037210.17	338.62851	0.00114813	0.00340499	0.1139745	0.73566382	0.87372812	Fam193a
ENSMUSG00000037211.13	88.6250509	0.00995997	0.00324715	9.40958695	0.00215854	0.00863196	Spry1
ENSMUSG00000037214.13	139.21593	-0.0065737	0.00330745	3.83079347	0.05031911	0.1211913	Thap1
ENSMUSG00000037216.6	109.308408	-0.0104011	0.00251355	16.9855022	3.77E-05	0.00023767	Lipt1
ENSMUSG00000037217.16	24.3913418	0.04353166	0.00809904	27.3860817	1.67E-07	1.66E-06	Syn1
ENSMUSG00000037221.14	405.966865	-0.0042777	0.0044493	0.91341942	0.33920877	0.52687376	Mospd3
ENSMUSG00000037224.16	4.72998846	0.05779073	0.01217199	20.5999844	5.66E-06	4.23E-05	Zfyve28
ENSMUSG00000037225.14	40.0461156	0.02187639	0.00740817	7.9151845	0.00490216	0.01738158	Fgf2
ENSMUSG00000037234.18	1875.21497	-0.0078394	0.00396807	3.82261804	0.05056517	0.12168454	Hook3
ENSMUSG00000037235.14	1351.91561	-0.0082739	0.00258027	10.1229192	0.00146437	0.00614238	Mxd4
ENSMUSG00000037236.17	1545.73203	-0.0005034	0.00238332	0.04388301	0.83407143	0.93669216	Matr3
ENSMUSG00000037239.9	15.1688256	0.01876185	0.00765628	5.72737918	0.01670239	0.04905275	Spred3
ENSMUSG00000037242.9	1537.87292	-0.0045383	0.00257772	3.05441368	0.08051805	0.17705564	Clic4
ENSMUSG00000037243.18	76.0794318	-0.0073503	0.00369354	3.96740691	0.04638916	0.11347591	Zfp692
ENSMUSG00000037251.6	138.815157	0.01545343	0.00297692	26.4944046	2.64E-07	2.54E-06	Pomk
ENSMUSG00000037253.9	333.492994	-0.0032149	0.00223034	2.03989192	0.15322053	0.29200545	Mex3c
ENSMUSG00000037254.19	18020.9359	0.01024766	0.00483343	4.43579543	0.03519282	0.09066715	Itih2
ENSMUSG00000037257.9	552.905697	-0.0082957	0.00226441	13.3767392	0.00025476	0.00131976	Aagab
ENSMUSG00000037259.16	40.0133721	0.01095507	0.00868534	1.57713266	0.20917419	0.37065507	Dzank1
ENSMUSG00000037260.9	870.125567	0.0125261	0.00260834	23.2219897	1.44E-06	1.21E-05	Hgsnat
ENSMUSG00000037262.8	190.380405	-0.0072381	0.00372773	3.74837864	0.05285876	0.12617415	Kin
ENSMUSG00000037263.14	23.3043589	0.08532721	0.01252361	37.0747301	1.14E-09	1.56E-08	Aldh3b3
ENSMUSG00000037266.19	5528.53678	-0.0022468	0.00367176	0.38082252	0.53716337	0.72141756	Rsrp1
ENSMUSG00000037270.19	1178.30516	-0.0065272	0.00341013	3.61973739	0.05709789	0.1344828	Bltp1
ENSMUSG00000037275.15	220.32689	-0.0065203	0.00234838	7.77558728	0.0052957	0.01858419	Gemin5
ENSMUSG00000037278.10	1512.03785	-0.0005971	0.00337656	0.03141628	0.85931486	0.95283438	Tmem97
ENSMUSG00000037279.14	1.87134949	0.01562563	0.0136935	1.5243668	0.21695986	0.38083019	Ovol2
ENSMUSG00000037280.13	41.2832743	0.06501787	0.00608313	107.438091	3.57E-25	3.38E-23	Galnt6
ENSMUSG00000037286.16	626.984834	0.01133977	0.00338509	10.721321	0.00105908	0.00461813	Stag1
ENSMUSG00000037287.16	1397.03136	-0.0056775	0.00383781	2.18853399	0.1390416	0.2713641	Tbcel
ENSMUSG00000037295.8	589.831168	-0.007844	0.00313534	6.15011946	0.01314032	0.04008895	Ldlrap1
ENSMUSG00000037296.8	441.92002	-0.0043509	0.00341997	1.64520938	0.19961222	0.35778203	Lsm1
ENSMUSG00000037300.20	548.708268	0.00281058	0.00343494	0.65488882	0.4183702	0.6098528	Ttc13
ENSMUSG00000037306.15	81.7745994	0.02714827	0.00455632	34.4771717	4.31E-09	5.50E-08	Man1c1
ENSMUSG00000037313.17	49.3339507	0.01350363	0.00718293	3.45038771	0.06323693	0.14598281	Tacc3
ENSMUSG00000037315.16	76.0292489	0.00827165	0.00342037	5.63015417	0.01765408	0.05134901	Jade3
ENSMUSG00000037316.10	779.707534	-0.0050985	0.00342233	2.16529807	0.14115725	0.27453904	Bag4
ENSMUSG00000037318.11	23.3076086	0.07423604	0.00777102	80.4726185	2.95E-19	1.38E-17	Traf3ip3
ENSMUSG00000037321.18	506.776695	0.03316887	0.00395086	67.5646983	2.04E-16	6.78E-15	Tap1
ENSMUSG00000037325.11	109.843486	-0.0036366	0.0041997	0.75256709	0.38566469	0.57724711	Bbs7
ENSMUSG00000037326.11	307.460138	-0.0108177	0.00409604	6.79156605	0.00915895	0.02965313	Capn15
ENSMUSG00000037331.16	2859.34336	-0.0057942	0.00340902	2.84550488	0.09162981	0.19625687	Larp1
ENSMUSG00000037336.15	13.1520923	0.01699268	0.00788484	4.56294632	0.03267082	0.08532544	Mfsd2b
ENSMUSG00000037337.12	23.7260255	0.0720531	0.0075397	80.7158245	2.61E-19	1.24E-17	Map4k1
ENSMUSG00000037339.18	405.920906	-0.0064613	0.00211017	9.44076904	0.00212214	0.00850662	Fam53a
ENSMUSG00000037341.15	11.250922	0.0148639	0.00684062	4.42633772	0.03538836	0.09107151	Slc9a7
ENSMUSG00000037343.9	391.918581	-0.0091445	0.00363632	6.19465431	0.01281368	0.03922941	Taf2
ENSMUSG00000037344.15	174.896597	-0.0076868	0.00487656	2.49213582	0.11441636	0.23316263	Slc12a9
ENSMUSG00000037347.8	24.628337	-0.0056937	0.00374553	2.2817436	0.13090464	0.25923734	Chst7
ENSMUSG00000037348.16	901.606988	-0.0061435	0.00410318	2.08136134	0.14910714	0.28622455	Paqr7
ENSMUSG00000037349.10	128.903351	-0.0007244	0.00368567	0.03946871	0.84252297	0.94228018	Nudt22
ENSMUSG00000037351.10	1164.24265	-0.006041	0.00173348	12.1901608	0.00048042	0.00230967	Actr1b
ENSMUSG00000037353.10	450.120126	-0.0109642	0.0022737	23.2069878	1.45E-06	1.22E-05	Letmd1
ENSMUSG00000037355.15	330.185669	0.00814654	0.00365013	4.9835163	0.02558992	0.0698246	Uvssa
ENSMUSG00000037361.9	836.72022	-0.0012183	0.00434633	0.07792139	0.7801343	0.90347872	Sf3b6
ENSMUSG00000037362.9	7.22001896	0.01817332	0.00896927	3.98049482	0.04603004	0.11284321	Ccn3
ENSMUSG00000037363.10	55.7572421	-0.0070027	0.00438901	2.45454021	0.11718548	0.23770919	Letm2
ENSMUSG00000037364.14	1082.28061	-0.0027559	0.00306321	0.80765545	0.36881433	0.55938517	Srrt

ENSMUSG00000037366.15	1681.43857	-0.0122578	0.00302001	16.2137801	5.66E-05	0.00034315	Pafah2
ENSMUSG00000037369.18	326.55451	-0.0011685	0.00326948	0.12537496	0.72327648	0.86573362	Kdm6a
ENSMUSG00000037370.14	615.799755	0.03605002	0.00307629	130.078766	3.94E-30	8.29E-28	Enpp1
ENSMUSG00000037373.13	810.365931	0.00279668	0.00214354	1.70917479	0.19109247	0.34582268	Ctbp1
ENSMUSG00000037375.17	27.7920752	-0.0074723	0.00653119	1.25345769	0.2628931	0.43760036	Hhat
ENSMUSG00000037376.15	260.881634	0.00468092	0.00285858	2.6812241	0.10153765	0.2127258	Trmt6
ENSMUSG00000037379.8	216.040017	0.0031159	0.00640897	0.22487338	0.63535147	0.802947	Spon2
ENSMUSG00000037386.17	20.6459109	-0.0077857	0.01202158	0.36574133	0.54533481	0.72854331	Rims2
ENSMUSG00000037390.14	11.922142	0.00659086	0.0209569	0.08764209	0.76719642	0.89497776	Muc17
ENSMUSG00000037395.17	184.176501	-0.0004902	0.00274544	0.0316253	0.85885253	0.95277248	Rcor3
ENSMUSG00000037400.18	828.29699	-0.0023231	0.00234737	0.97278452	0.32398671	0.51072685	Atp11b
ENSMUSG00000037405.9	301.322797	0.03610217	0.00472716	53.2243029	2.98E-13	6.72E-12	Icam1
ENSMUSG00000037406.8	13.3842954	-0.0374427	0.00785035	22.7673612	1.83E-06	1.51E-05	Htra4
ENSMUSG00000037408.11	91.423854	0.01091894	0.00349351	9.65949542	0.00188375	0.00766964	Cnnm4
ENSMUSG00000037410.14	734.261623	0.00316375	0.00402274	0.61771589	0.43189734	0.62345538	Tbc1d2b
ENSMUSG00000037411.12	73.9904967	0.0799512	0.01088661	42.4612612	7.21E-11	1.18E-09	Serpine1
ENSMUSG00000037415.8	1128.47759	-0.00921	0.00282341	10.3593995	0.00128817	0.00548624	Ranbp10
ENSMUSG00000037416.14	592.245254	-0.0059737	0.00432922	1.84202286	0.17471404	0.32294713	Dmxl1
ENSMUSG00000037419.10	97.2453289	0.02998627	0.00437825	45.0103655	1.96E-11	3.48E-10	Endod1
ENSMUSG00000037426.19	260.268365	-0.0105284	0.0047579	4.94629444	0.02614665	0.07105599	Depdc5
ENSMUSG00000037428.15	3.42963928	0.01438811	0.02150383	0.52146105	0.47021911	0.66117729	Vgf
ENSMUSG00000037432.16	7.77540534	-0.021849	0.01252832	2.84030425	0.09192681	0.19678577	Fer1l5
ENSMUSG00000037434.8	763.553985	0.00262931	0.00288659	0.81151276	0.36767338	0.55796837	Slc30a1
ENSMUSG00000037437.19	4.52646343	-0.0371621	0.01516671	5.08659681	0.02411146	0.06641282	Adam32
ENSMUSG00000037440.9	952.496686	0.00730008	0.00658996	1.04152293	0.30746754	0.49252441	Vnn1
ENSMUSG00000037443.14	1029.25028	-0.0003284	0.00427006	0.00581138	0.93923418	0.99639173	Cep85
ENSMUSG00000037446.15	2.23848671	0.00156764	0.01862579	0.03288648	0.85609576	0.95096663	Tulp1
ENSMUSG00000037447.17	51.3738867	0.01833849	0.00680494	6.37921491	0.01154645	0.03593752	Arid5a
ENSMUSG00000037455.18	74.7251888	0.00016326	0.00539819	0.0008761	0.97638685	1	Slc18b1
ENSMUSG00000037458.16	1965.45618	-0.0046965	0.00295457	2.51882329	0.1124945	0.23016094	Azin1
ENSMUSG00000037461.11	356.335692	-0.0083792	0.00226413	13.4928278	0.00023948	0.00125018	Ints7
ENSMUSG00000037465.11	694.698777	0.00759455	0.00589512	1.57807826	0.20903772	0.37059258	Klf10
ENSMUSG00000037466.14	9.59047014	0.01389985	0.01021908	1.80673524	0.17890037	0.32877856	Tedc1
ENSMUSG00000037470.15	1497.20573	-0.0021297	0.00275249	0.59260143	0.44141492	0.63328542	Uggt1
ENSMUSG00000037474.14	24.5414646	0.02953294	0.00854469	11.0546954	0.00088463	0.0039376	Dtl
ENSMUSG00000037475.16	2470.58804	0.00073513	0.00411629	0.03091597	0.86042799	0.953621	Thoc2
ENSMUSG00000037486.19	637.202356	0.00302802	0.00351205	0.7426238	0.38882167	0.58035363	Asxl2
ENSMUSG00000037487.8	1867.80843	-0.0063561	0.00219677	8.27336347	0.00402309	0.01469616	Ubr5
ENSMUSG00000037490.6	5.07081276	-0.0012606	0.01048861	-0.0028528	1	1	Slc2a12
ENSMUSG00000037492.17	4.81440995	0.02137743	0.01709296	1.8127363	0.17818033	0.3279408	Zmat4
ENSMUSG00000037493.7	132.380003	-0.0084667	0.00447312	3.43622045	0.06378144	0.14695215	Cib2
ENSMUSG00000037499.10	160.679526	0.00027827	0.00470758	0.00352002	0.95268945	1	Nenf
ENSMUSG00000037503.13	1078.09996	0.004405	0.0028167	2.42487434	0.11942282	0.24125966	Fam168b
ENSMUSG00000037509.22	12.9923999	0.05355912	0.00905897	34.1382002	5.13E-09	6.48E-08	Arhgef4
ENSMUSG00000037514.19	480.154949	0.00466618	0.00195739	5.6758679	0.01719983	0.05027577	Pank2
ENSMUSG00000037519.18	592.594214	-0.0091196	0.00277545	11.0372217	0.00089301	0.0039703	Ppfia1
ENSMUSG00000037523.14	1597.73518	-0.0079077	0.00245126	10.332266	0.00130725	0.00555008	Mavs
ENSMUSG00000037525.6	57.9905764	-0.0111913	0.00363703	9.58906094	0.0019574	0.00793388	Bcdin3d
ENSMUSG00000037526.8	332.966887	-0.0035521	0.0027796	1.60389584	0.20535199	0.36565242	Atg14
ENSMUSG00000037531.9	272.313899	-0.0193038	0.00339486	32.7540329	1.05E-08	1.26E-07	Mrpl47
ENSMUSG00000037533.21	391.638116	0.00124681	0.00316732	0.15243578	0.69621842	0.8470899	Rapgef6
ENSMUSG00000037536.15	375.367638	0.00073989	0.00278342	0.0714749	0.78920131	0.9097357	Fbxo34
ENSMUSG00000037541.23	287.404553	0.00869712	0.00505822	2.94273882	0.08626435	0.18714413	Shank2
ENSMUSG00000037542.14	6344.77103	-0.0019706	0.00465463	0.17847759	0.67268485	0.83015618	Aldh8a1
ENSMUSG00000037544.15	13.9496518	0.03104104	0.01127032	6.90208079	0.00860955	0.02814481	Dlgap5
ENSMUSG00000037548.16	30.6667934	0.091789	0.00691175	148.10178	4.51E-34	1.52E-31	H2-DMb2
ENSMUSG00000037552.18	123.610988	0.01038615	0.00393071	6.97808125	0.0082514	0.02711351	Plekhg2
ENSMUSG00000037553.15	428.837194	0.0039828	0.00366413	1.14451229	0.28470087	0.46471023	Zdhhc18
ENSMUSG00000037563.16	6183.80408	-0.002011	0.00440756	0.20920777	0.64738906	0.81156057	Rps16
ENSMUSG00000037568.13	3.90484606	0.03914651	0.01672693	4.97786957	0.02567358	0.07002857	Vash2
ENSMUSG00000037570.17	142.810846	0.00367287	0.00375033	0.94997759	0.329725	0.5164969	Mcrs1

ENSMUSG00000037572.18	46.1827168	0.01109962	0.00664424	2.61845679	0.10562712	0.21899003	Wdhd1
ENSMUSG00000037573.6	2175.93425	-0.0014407	0.00317108	0.20670294	0.64936397	0.81285949	Tob1
ENSMUSG00000037577.14	3.7859695	0.0022012	0.00911602	0.06059124	0.80556388	0.91898276	Ephx3
ENSMUSG00000037579.8	4.07079902	0.02185895	0.01327169	2.30740435	0.1287589	0.25601193	Kcnh3
ENSMUSG00000037580.11	2935.44188	-0.0105647	0.00358129	8.57981807	0.00339909	0.01271748	Gch1
ENSMUSG00000037583.3	466.483234	-0.0136509	0.0049656	7.67412008	0.00560184	0.01948746	Nr0b2
ENSMUSG00000037593.13	6.78911832	-0.0260176	0.00897136	8.19508968	0.00420039	0.0152463	Rskr
ENSMUSG00000037594.12	13.9385558	-0.0117413	0.00627213	3.50396915	0.06122194	0.14225008	Clba1
ENSMUSG00000037600.17	72.0602412	0.0002744	0.00396292	0.00482086	0.94464543	0.9994266	Kdf1
ENSMUSG00000037601.12	808.697903	-0.0143891	0.00395882	13.5881194	0.00022762	0.00119596	Nme1
ENSMUSG00000037605.18	42.5632851	0.05260726	0.01156081	16.1884865	5.73E-05	0.0003475	Adgrl3
ENSMUSG00000037606.19	64.6196699	0.01970691	0.00508784	15.3338249	9.01E-05	0.00052016	Osbpl5
ENSMUSG00000037608.17	1253.8985	0.00033776	0.00278203	0.01491589	0.90279558	0.97702372	Bclaf1
ENSMUSG00000037613.17	18.5406253	0.03565635	0.00752702	21.0409012	4.50E-06	3.43E-05	Tnfrsf23
ENSMUSG00000037617.13	31.0921964	-0.0078447	0.00541182	2.10183307	0.14712267	0.28336071	Spag1
ENSMUSG00000037621.9	308.154401	0.00058661	0.00450743	0.0171532	0.89579886	0.97330466	Atoh8
ENSMUSG00000037622.15	1611.38958	-0.0171701	0.00334666	26.1753432	3.12E-07	2.96E-06	Wdtc1
ENSMUSG00000037624.17	36.1593471	-0.0395555	0.01168061	11.8582018	0.00057408	0.00269995	Kcnk2
ENSMUSG00000037625.8	7.22072687	0.02800019	0.01665809	3.15400699	0.07574076	0.16865763	Cldn11
ENSMUSG00000037627.17	1.94025917	-0.0150243	0.04062509	0.04529375	0.8314648	0.93505596	Rgs22
ENSMUSG00000037628.11	10.1988971	0.01564427	0.01315172	1.14449053	0.28470545	0.46471023	Cdkn3
ENSMUSG00000037636.7	2.00543873	0.06354155	0.01172863	26.4396595	2.72E-07	2.60E-06	Slc25a43
ENSMUSG00000037638.6	96.7351298	-0.0024729	0.00385552	0.41420885	0.51984095	0.70585052	Zbtb42
ENSMUSG00000037640.16	187.026786	-0.0005392	0.00289521	0.03393148	0.85385264	0.9497266	Zfp60
ENSMUSG00000037643.15	351.42602	0.0030341	0.00296706	1.03899034	0.30805641	0.49296549	Prkci
ENSMUSG00000037646.11	963.558268	0.00155414	0.00319868	0.23310249	0.62923286	0.79786497	Vps13b
ENSMUSG00000037649.11	120.197778	0.06780567	0.00556363	138.281905	6.32E-32	1.75E-29	H2-DMa
ENSMUSG00000037652.16	515.553915	0.00018975	0.00292944	0.00414748	0.94865098	1	Phc3
ENSMUSG00000037653.8	1.53439731	0.00799304	0.03613952	0.10053337	0.75119051	0.88439495	Kctd8
ENSMUSG00000037656.10	1234.12067	-0.0043388	0.00225129	3.73952844	0.05313944	0.12671589	Slc20a2
ENSMUSG00000037661.15	13.1327318	0.02332093	0.00769766	8.20884963	0.00416866	0.01514509	Gpr160
ENSMUSG00000037664.14	57.028998	0.01418892	0.00586367	5.90557682	0.01509302	0.04510123	Cdkn1c
ENSMUSG00000037669.16	857.466744	-0.0106263	0.00254692	17.0110233	3.72E-05	0.00023493	Ldah
ENSMUSG00000037674.17	366.980078	-0.0038893	0.00298862	1.68495402	0.19426748	0.34995744	Rfx7
ENSMUSG00000037679.10	666.80224	0.01292229	0.00332064	14.7589969	0.00012216	0.00068522	Inf2
ENSMUSG00000037681.14	3.69527538	0.00726088	0.01104524	0.42860029	0.51267656	0.69932274	Esyt3
ENSMUSG00000037683.15	2.23390852	0.02406258	0.01342136	3.0863175	0.0789533	0.17431363	Armc3
ENSMUSG00000037685.16	176.527087	0.04884165	0.00465515	101.05901	8.93E-24	7.17E-22	Atp8a1
ENSMUSG00000037686.7	2503.54574	-0.0190305	0.0052931	12.9266043	0.00032394	0.00163016	Aspg
ENSMUSG00000037692.15	217.891998	-0.0067248	0.00409899	2.55557229	0.10990601	0.22612251	Ahdc1
ENSMUSG00000037697.20	430.467847	0.00294446	0.00339204	0.72478495	0.39457883	0.58605392	Ddhd1
ENSMUSG00000037703.15	423.585583	0.0016715	0.00387445	0.1848234	0.66726044	0.82620849	Lzts3
ENSMUSG00000037706.18	3998.34274	0.00512686	0.00202818	6.28796973	0.01215602	0.03754248	Cd81
ENSMUSG00000037709.14	450.564202	0.01603247	0.00461795	12.0996843	0.0005043	0.00241415	Fam13a
ENSMUSG00000037710.9	2321.03139	-0.0120694	0.00321322	14.1070131	0.0001727	0.00093789	Cisd1
ENSMUSG00000037712.18	2333.80824	-0.0150857	0.00390114	14.3523991	0.00015159	0.00083128	Fermt2
ENSMUSG00000037720.17	1675.13093	-0.0145499	0.00227703	39.4926214	3.29E-10	4.87E-09	Tmem33
ENSMUSG00000037722.9	2421.62832	0.00132103	0.00321147	0.16639986	0.68333139	0.83793412	Gnpnat1
ENSMUSG00000037725.9	17.2972379	0.01699518	0.01060572	2.33358818	0.12660973	0.25255558	Ckap2
ENSMUSG00000037730.14	206.475706	-0.0002738	0.00285015	0.00920125	0.92358159	0.98832156	Mynn
ENSMUSG00000037731.6	127.518895	0.04882841	0.00471894	102.141962	5.17E-24	4.31E-22	Themis2
ENSMUSG00000037736.21	212.456279	0.02401306	0.00666395	12.0862643	0.00050795	0.00242912	Limch1
ENSMUSG00000037738.12	5.01298296	0.0343724	0.00792029	18.5934922	1.62E-05	0.00011028	Nek5
ENSMUSG00000037740.9	346.21429	-0.0095533	0.00306284	9.83998298	0.00170759	0.00702516	Mrps26
ENSMUSG00000037742.15	121695.702	0.00419237	0.0024268	2.96587609	0.08503838	0.18501202	Eef1a1
ENSMUSG00000037747.10	9.71643172	0.02671849	0.0135654	3.78645428	0.05166899	0.12386829	Phyhipl
ENSMUSG00000037750.17	286.316409	-0.0195777	0.00350607	30.5776125	3.21E-08	3.58E-07	Fam222b
ENSMUSG00000037752.5	127.667273	0.00389145	0.00315626	1.48855623	0.2224406	0.3877081	Xkr8
ENSMUSG00000037754.14	81.5254805	0.01670149	0.00374467	19.5974556	9.56E-06	6.81E-05	Ppp1r16b
ENSMUSG00000037759.7	30.7936682	0.02549976	0.00518366	23.2178136	1.45E-06	1.22E-05	Ptger2
ENSMUSG00000037761.17	129.68276	-0.0092603	0.003022	9.30952136	0.00227966	0.00904613	Actr5

ENSMUSG00000037762.7	85.0191471	0.04469913	0.00426784	103.697466	2.36E-24	2.07E-22	Slc16a9
ENSMUSG00000037772.14	382.781443	-0.00244	0.00360397	0.461909	0.49673315	0.68386341	Mrpl23
ENSMUSG00000037773.15	265.061246	-0.0082993	0.00241664	11.5191666	0.00068882	0.00316759	Pced1a
ENSMUSG00000037780.4	3663.7501	-0.0201889	0.00474803	17.9715377	2.24E-05	0.00014823	Mbl1
ENSMUSG00000037784.15	20.7145308	0.03346341	0.00656102	25.7540393	3.88E-07	3.62E-06	Dzip1l
ENSMUSG00000037787.15	394.660193	-0.0043348	0.0029842	2.10533947	0.14678576	0.282966	Coa8
ENSMUSG00000037788.15	103.156561	0.02173445	0.00419012	25.9646974	3.48E-07	3.27E-06	Vopp1
ENSMUSG00000037791.16	430.536862	0.00113195	0.00362986	0.09627636	0.75634476	0.88747945	Phf12
ENSMUSG00000037795.15	577.008194	-0.0158021	0.00386826	16.1712827	5.79E-05	0.00035004	N4bp2
ENSMUSG00000037797.15	2908.50036	-0.0024192	0.00560943	0.17882578	0.6723843	0.83004585	Adh4
ENSMUSG00000037798.8	53825.8752	0.0148413	0.00617253	5.83182313	0.0157388	0.04668202	Mat1a
ENSMUSG00000037801.14	2.79833315	-0.0108007	0.01066097	0.95115514	0.32942544	0.51633596	Iqch
ENSMUSG00000037805.16	6040.80217	-3.83E-05	0.00347572	0.00012254	0.9911677	1	Rpl10a
ENSMUSG00000037808.14	220.016502	0.0109947	0.00295009	13.7694085	0.00020667	0.00109801	Fam76b
ENSMUSG00000037813.14	14.6471765	-0.003883	0.00532961	0.53557451	0.4642726	0.65523777	D630003M21Rik
ENSMUSG00000037815.8	2136.39873	0.00122315	0.00214255	0.32294903	0.56984075	0.74959643	Ctnna1
ENSMUSG00000037816.11	29.8803759	0.02518755	0.00553163	20.3244215	6.54E-06	4.83E-05	Fbxw17
ENSMUSG00000037818.18	507.623669	-0.0250497	0.00274848	82.0745575	1.31E-19	6.58E-18	Abhd18
ENSMUSG00000037820.16	2666.3133	0.02712507	0.00371528	51.3445467	7.75E-13	1.65E-11	Tgm2
ENSMUSG00000037822.13	1487.14256	-0.0010981	0.00243993	0.19966022	0.65499525	0.81674427	Smim14
ENSMUSG00000037824.7	427.209697	0.00523869	0.00232383	5.00518836	0.02527145	0.06909952	Tspan14
ENSMUSG00000037826.6	1487.27583	-0.0050923	0.00417932	1.43184258	0.23146396	0.39918435	Ppm1k
ENSMUSG00000037833.15	2.56289823	0.01297445	0.01132773	1.31434349	0.25160975	0.42349729	Sh2d4b
ENSMUSG00000037843.7	1.08132573	0.01739997	0.03124725	0.42667221	0.51362638	0.70028533	Vstm2l
ENSMUSG00000037845.15	133.631307	-0.0007234	0.00315163	0.0517808	0.81999303	0.92776557	Fdxacb1
ENSMUSG00000037846.19	2.74013329	0.00527385	0.01366461	0.18541621	0.66675937	0.82586842	Rtkn2
ENSMUSG00000037847.15	576.925815	-0.0070298	0.00486552	2.08902088	0.14836113	0.2850477	Nmrk1
ENSMUSG00000037849.9	32.7814364	0.04315794	0.00664268	42.3318196	7.70E-11	1.26E-09	Ifi206
ENSMUSG00000037851.15	782.976712	-0.0052738	0.00252556	4.30540584	0.03799143	0.09637189	Iars
ENSMUSG00000037852.9	334.438548	0.02499697	0.00909112	7.33678498	0.00675576	0.0229268	Cpe
ENSMUSG00000037855.16	25.2006782	0.04260683	0.01573062	7.35400965	0.00669134	0.0227409	Zfp365
ENSMUSG00000037857.17	615.449302	-0.0015789	0.00287951	0.29488119	0.5871094	0.76383834	Nufip2
ENSMUSG00000037860.16	44.3703169	0.05450329	0.00793563	42.1876088	8.29E-11	1.35E-09	Aim2
ENSMUSG00000037868.16	21.7233759	0.10421687	0.0129923	55.5044314	9.32E-14	2.22E-12	Egr2
ENSMUSG00000037872.6	26.2778253	0.01861907	0.00762731	5.60955965	0.01786275	0.05186003	Ackr1
ENSMUSG00000037876.18	816.086589	-0.0010281	0.00311796	0.10762464	0.74286482	0.87846324	Jmjd1c
ENSMUSG00000037885.19	286.676605	-0.0062263	0.00312273	3.97548833	0.04616706	0.11310629	Stk35
ENSMUSG00000037887.12	46.9409642	0.02346276	0.00848283	6.65756309	0.00987358	0.03156817	Dusp8
ENSMUSG00000037890.14	62.4828125	-0.0005893	0.00409226	0.02041194	0.88639256	0.96775248	Wdr19
ENSMUSG00000037892.14	27.9107347	0.00101219	0.0063711	0.02469962	0.87511787	0.96150045	Pcdh18
ENSMUSG00000037894.14	1601.75423	-0.0029646	0.00299321	0.95634158	0.32811034	0.51515898	H2az1
ENSMUSG00000037896.18	367.080538	-0.0104428	0.00284753	13.263769	0.00027059	0.00139315	Rcor1
ENSMUSG00000037902.19	634.136487	0.0677194	0.00486563	181.499729	2.28E-41	1.92E-38	Sirpa
ENSMUSG00000037904.15	54.0191885	-0.0115057	0.00532775	4.5862566	0.03222933	0.08438722	Ankrd9
ENSMUSG00000037905.14	550.980513	-0.0189813	0.00248999	59.024503	1.56E-14	4.17E-13	Bri3bp
ENSMUSG00000037907.17	46.8242358	0.00113737	0.00552066	0.04295095	0.83581766	0.93793868	Ankrd13b
ENSMUSG00000037913.13	5.55577351	0.05598161	0.00842941	39.9774434	2.57E-10	3.88E-09	Tmem156
ENSMUSG00000037916.15	2489.03945	-0.0192001	0.00341041	32.0482016	1.50E-08	1.77E-07	Ndufv1
ENSMUSG00000037921.16	159.26462	0.03168615	0.00549087	33.1908481	8.35E-09	1.02E-07	Ddx60
ENSMUSG00000037922.14	79.5995339	0.04834339	0.0057856	63.3126529	1.76E-15	5.28E-14	Bank1
ENSMUSG00000037924.7	2.90101275	0.00096598	0.01165927	0.00763679	0.93036255	0.99184284	Or10j5
ENSMUSG00000037926.16	595.472158	-0.0003211	0.00308216	0.01071073	0.91757199	0.98492936	Ssh2
ENSMUSG00000037933.17	353.736854	-0.0118833	0.00305709	14.889855	0.00011397	0.00064326	Bicd2
ENSMUSG00000037935.17	727.287557	0.00310185	0.0024668	1.56042219	0.21160341	0.37381712	Smarce1
ENSMUSG00000037936.16	2553.78486	-0.0067376	0.00322281	4.40642017	0.0358039	0.09192019	Scarb1
ENSMUSG00000037938.5	184.110271	-0.001938	0.00299967	0.41494676	0.51946935	0.70548765	Chchd5
ENSMUSG00000037940.18	53.8389856	0.02872281	0.00786312	12.0057593	0.00053036	0.00252329	Inpp4b
ENSMUSG00000037942.6	6789.15139	-0.0078773	0.0049441	2.47710386	0.11551478	0.2349747	Crp
ENSMUSG00000037944.9	12.2562934	0.05783524	0.00694278	64.7376918	8.56E-16	2.66E-14	Ccr7
ENSMUSG00000037946.12	36.6915854	0.04534854	0.00652079	44.5475588	2.48E-11	4.32E-10	Fgd3
ENSMUSG00000037949.9	206.42528	-0.0088944	0.00466824	3.63506838	0.05657428	0.1336781	Ano10

ENSMUSG00000037953.8	10.7911916	0.13220729	0.01451218	66.8741822	2.89E-16	9.47E-15	A4gnt
ENSMUSG00000037957.15	345.347581	-0.0076783	0.0021245	12.9550787	0.00031905	0.00160841	Wdr20
ENSMUSG00000037958.14	623.070519	-0.0085265	0.00480842	2.93462471	0.08669881	0.18792359	Nsrp1
ENSMUSG00000037960.12	122.398975	0.00585066	0.0041656	1.97587558	0.15982558	0.30133063	Card19
ENSMUSG00000037962.8	1.93123426	0.01330807	0.01241918	1.10873463	0.29235718	0.47427859	Rflna
ENSMUSG00000037965.16	796.644962	0.00653847	0.00315023	4.33431391	0.03735147	0.0950142	Zc3h7a
ENSMUSG00000037966.16	2095.18767	0.00058446	0.00303722	0.03702742	0.84740924	0.9455033	Ninj1
ENSMUSG00000037971.11	539.07567	-0.0032417	0.0024833	1.71424747	0.19043521	0.34492435	Cfap68
ENSMUSG00000037972.8	66.4936492	0.06151518	0.00550816	114.983636	7.93E-27	9.50E-25	Snn
ENSMUSG00000037977.7	16.4884227	-0.0057582	0.00530541	1.17033543	0.27933233	0.4586059	6430571L13Rik
ENSMUSG00000037979.14	10.2724735	0.03059121	0.00978036	9.32219009	0.00226395	0.00899706	Ccdc92
ENSMUSG00000037989.17	20.1638959	-0.0100193	0.00836967	1.41308386	0.23454513	0.40291197	Wnk2
ENSMUSG00000037990.19	6.22523018	0.02271113	0.01485147	2.78997459	0.09485573	0.20177374	Sh3rf3
ENSMUSG00000037991.10	3.42560295	0.05042822	0.01141229	18.200131	1.99E-05	0.0001331	Rmi2
ENSMUSG00000037992.17	321.599134	0.00732839	0.00287284	6.42429027	0.01125699	0.03525526	Rara
ENSMUSG00000037993.9	395.623526	-0.0007538	0.00198492	0.14186483	0.70643411	0.8543278	Dhx38
ENSMUSG00000037994.15	5.90153913	-0.0009847	0.01078484	0.00529337	0.94200067	0.9980951	Slc9b2
ENSMUSG00000037995.16	13.5390088	0.02433576	0.00893893	7.5349516	0.00605134	0.02081476	Igsf9
ENSMUSG00000037996.18	32.4755605	0.02963068	0.01535909	4.08984479	0.0431416	0.1070529	Slc24a2
ENSMUSG00000037997.13	119.005221	0.02684366	0.0040801	41.9659197	9.29E-11	1.50E-09	Parp11
ENSMUSG00000037999.14	499.030326	0.021171	0.00328653	39.7752616	2.85E-10	4.26E-09	Arap2
ENSMUSG00000038000.11	176.935876	-0.0001092	0.00251394	0.00191524	0.96509292	1	Acd
ENSMUSG00000038002.10	254.334585	-0.0075702	0.00353318	4.59717973	0.0320246	0.08394445	Cramp1
ENSMUSG00000038005.16	149.064031	0.00543458	0.00414252	1.72762843	0.18871405	0.34264893	Hpf1
ENSMUSG00000038007.15	284.396468	0.01457523	0.00326867	19.7043808	9.04E-06	6.47E-05	Acer2
ENSMUSG00000038009.8	1531.63929	-0.0079062	0.00349614	5.04922033	0.02463702	0.06759991	Dnajc22
ENSMUSG00000038010.10	74.9308943	-0.0080849	0.00387317	4.2995648	0.03812213	0.09664107	Ccdc138
ENSMUSG00000038011.16	5.11812188	-0.0055233	0.01774132	-0.026996	1	1	Dnah10
ENSMUSG00000038013.15	243.685024	-1.99E-05	0.00348818	3.06E-05	0.99558491	1	Wipf2
ENSMUSG00000038014.8	2654.5107	-0.0020034	0.00164137	1.48129175	0.22357253	0.38894957	Fam120a
ENSMUSG00000038015.7	18.5869714	-0.1253186	0.06168935	2.17225716	0.14051985	0.27364227	Prm2
ENSMUSG00000038020.6	15.0643462	0.00142572	0.00899198	0.02574319	0.87252909	0.96001922	Rapgefl1
ENSMUSG00000038022.12	53.8574746	-0.0067086	0.00518403	1.69857491	0.19247445	0.34768342	Mindy4
ENSMUSG00000038023.13	674.134367	-0.0025786	0.00286916	0.80003839	0.37108189	0.5616584	Atp6v0a2
ENSMUSG00000038024.18	625.956309	-0.0059621	0.00308681	3.60706002	0.05753475	0.13528594	Dennd4c
ENSMUSG00000038025.9	372.334098	-0.0014446	0.00277329	0.26977988	0.60347947	0.77665012	Phf2
ENSMUSG00000038026.13	6.27314729	0.03434668	0.0089958	13.9314501	0.0001896	0.00101842	Kcnj9
ENSMUSG00000038028.10	201.340885	-0.0144689	0.0034613	17.0953632	3.55E-05	0.00022521	Tigar
ENSMUSG00000038034.16	176.949801	0.02455434	0.00387258	39.7939674	2.82E-10	4.23E-09	Igsf8
ENSMUSG00000038037.6	14.0598887	0.03661503	0.00776625	20.1132238	7.30E-06	5.33E-05	Socs1
ENSMUSG00000038039.14	639.976116	-0.004779	0.00390016	1.45995401	0.22693744	0.39323933	Gcc2
ENSMUSG00000038042.12	35.4855862	0.02101949	0.00527875	15.9688191	6.44E-05	0.00038548	Ptpdc1
ENSMUSG00000038046.5	119.449582	-0.0199906	0.00304733	43.5670819	4.10E-11	6.92E-10	Mrm3
ENSMUSG00000038047.19	96.4671784	0.00425481	0.00326178	1.65175646	0.19872004	0.35648089	Haus6
ENSMUSG00000038055.13	286.851804	-0.0147134	0.00302193	23.8030792	1.07E-06	9.13E-06	Dexi
ENSMUSG00000038056.16	763.09179	-0.0009846	0.00305413	0.10299473	0.74826502	0.88197206	Kmt2c
ENSMUSG00000038057.5	7.47044806	-0.0298855	0.01090091	7.5111751	0.00613174	0.0210514	Dbil5
ENSMUSG00000038058.15	260.030871	0.01956014	0.00346963	31.1497951	2.39E-08	2.73E-07	Nod1
ENSMUSG00000038059.8	24.3045538	0.01934427	0.00633338	9.03870516	0.00264323	0.01025381	Smim3
ENSMUSG00000038060.16	23.8018545	0.01665842	0.00904716	3.08720373	0.0789103	0.17423502	Dlec1
ENSMUSG00000038065.14	93.859131	0.01238522	0.00461261	7.11112265	0.00766071	0.02544867	Mturn
ENSMUSG00000038068.16	745.651062	-0.0163909	0.00320851	25.6634908	4.06E-07	3.77E-06	Rnf144b
ENSMUSG00000038069.7	291.52144	-0.0005349	0.00211392	0.06308623	0.8016829	0.91677541	Cdkn2aip
ENSMUSG00000038070.16	44.0951966	0.01968906	0.00467094	17.53648	2.82E-05	0.00018258	Cntln
ENSMUSG00000038072.15	277.978379	0.00785169	0.00331394	5.49632951	0.01905643	0.05467279	Galnt11
ENSMUSG00000038074.17	143.638847	0.00187304	0.00350667	0.29216933	0.58883372	0.76566032	Fkbp14
ENSMUSG00000038077.8	2.85147527	0.03909392	0.0143243	7.28190059	0.00696528	0.02353963	Kcna6
ENSMUSG00000038079.15	56.1103825	0.02524475	0.00430559	34.088829	5.27E-09	6.63E-08	Tmem237
ENSMUSG00000038080.18	532.631918	-0.0004908	0.00235181	0.0430404	0.83564922	0.93792816	Kdm1b
ENSMUSG00000038084.17	1672.65362	-0.0078532	0.0027703	8.02312886	0.00461837	0.0165003	Opa1
ENSMUSG00000038085.14	19.835046	0.01427439	0.00665371	4.25712674	0.03908598	0.0986603	Cnbd2

ENSMUSG00000038086.5	8.41516024	0.00860431	0.00949936	0.77338262	0.37917264	0.57060013	Hspb2
ENSMUSG00000038092.7	2357.52015	-0.1317551	0.01286726	82.5877904	1.01E-19	5.11E-18	Hsd3b5
ENSMUSG00000038094.16	1.91482926	0.02613526	0.01536623	3.19746224	0.07375263	0.16515044	Atp13a4
ENSMUSG00000038095.16	1584.97093	0.00187673	0.00382785	0.2342557	0.62838609	0.7970055	Sbno1
ENSMUSG00000038102.16	686.305981	-0.0062999	0.00214156	8.58042975	0.00339795	0.01271523	Trappc11
ENSMUSG00000038112.16	4.42859384	0.06205864	0.01283038	20.775745	5.16E-06	3.89E-05	AW551984
ENSMUSG00000038115.17	3.87703209	0.03023005	0.01084065	7.65542754	0.00566018	0.019673	Ano2
ENSMUSG00000038116.17	258.078777	0.02443716	0.00395871	38.0340861	6.95E-10	9.87E-09	Phf20
ENSMUSG00000038119.16	39.5510931	0.0118572	0.00682012	2.89482429	0.08886446	0.19153406	Cdon
ENSMUSG00000038121.7	1736.28738	-0.0076709	0.00302037	6.44836602	0.01110544	0.03487325	Fam210a
ENSMUSG00000038122.10	140.865708	0.00531494	0.00290335	3.30727865	0.06897362	0.15634905	Tbc1d32
ENSMUSG00000038126.18	83.0823459	0.01073642	0.00487654	4.83320542	0.02791668	0.07498855	Mphosph9
ENSMUSG00000038127.16	1290.63619	0.00778962	0.00303043	6.52331957	0.01064691	0.03365742	Ccdc50
ENSMUSG00000038128.8	3.37677897	0.06250737	0.01135595	29.659776	5.15E-08	5.57E-07	Camk4
ENSMUSG00000038132.7	45.3943262	0.01535686	0.01993434	0.85082399	0.35631934	0.54509208	Rbm24
ENSMUSG00000038135.5	65.5473923	0.03624894	0.00760069	19.7105911	9.01E-06	6.46E-05	Crygn
ENSMUSG00000038141.12	129.48368	0.00659342	0.00335674	3.87523691	0.04900339	0.11864032	Tmem181a
ENSMUSG00000038143.17	48.4390468	-0.0035096	0.00475061	0.55170727	0.45762094	0.64930804	Stox2
ENSMUSG00000038145.18	1441.70755	-0.0013717	0.00343463	0.15885314	0.6902146	0.84302373	Snrk
ENSMUSG00000038146.9	37.2764276	-0.0137839	0.00667157	4.14688067	0.0417113	0.10409508	Notch3
ENSMUSG00000038147.15	197.074571	0.05714422	0.00537906	101.827937	6.06E-24	4.99E-22	Cd84
ENSMUSG00000038150.8	1479.33725	-0.0121156	0.00286536	17.5341127	2.82E-05	0.00018276	Ormdl3
ENSMUSG00000038151.14	18.7084427	0.07739652	0.00642175	129.940779	4.22E-30	8.74E-28	Prdm1
ENSMUSG00000038155.12	1207.71027	-0.0242486	0.00874824	6.41933397	0.01128845	0.03533502	Gstp2
ENSMUSG00000038156.17	39.9086954	0.02940696	0.00546169	27.740218	1.39E-07	1.41E-06	Spon1
ENSMUSG00000038160.8	1101.52354	-0.0046456	0.00289134	2.56660045	0.10914205	0.22492131	Atg5
ENSMUSG00000038167.7	218.666224	0.00161719	0.00383967	0.17413655	0.67646139	0.83279147	Plekhg6
ENSMUSG00000038168.6	38.1035812	0.02706307	0.00575809	20.50536	5.95E-06	4.43E-05	P3h2
ENSMUSG00000038170.16	1619.82104	-0.0128198	0.00368698	12.0114558	0.00052875	0.00251736	Pde4dip
ENSMUSG00000038172.15	697.818383	0.00397827	0.00310548	1.59958677	0.20596178	0.36651687	Ttc39b
ENSMUSG00000038173.16	81.7387674	0.02574889	0.00439327	32.0658904	1.49E-08	1.76E-07	Enpp6
ENSMUSG00000038174.15	688.315796	-0.0160925	0.00488152	10.375287	0.00127713	0.00544705	Hycc2
ENSMUSG00000038175.11	226.915047	0.01364174	0.00290383	21.6006437	3.36E-06	2.64E-05	Mylip
ENSMUSG00000038178.17	285.16239	0.02932729	0.00432759	44.3073096	2.81E-11	4.85E-10	Slc43a2
ENSMUSG00000038179.14	28.5151404	0.09986576	0.00808829	127.744546	1.28E-29	2.43E-27	Slamf7
ENSMUSG00000038180.12	23.4099222	-0.0118565	0.00597036	3.78447373	0.05173018	0.12398211	Spag4
ENSMUSG00000038181.17	405.269513	0.00613626	0.00368967	2.81718298	0.09325995	0.19911552	Chpf2
ENSMUSG00000038187.15	65.2276087	0.00622572	0.0036695	2.86273658	0.09065315	0.19448292	Btbd10
ENSMUSG00000038188.18	221.66541	-0.0056933	0.00376651	2.34899484	0.12536382	0.25064262	Scarf1
ENSMUSG00000038193.5	105.669507	0.00828931	0.00449567	3.36441909	0.06661908	0.15215514	Hand2
ENSMUSG00000038195.7	346.106747	-0.0111276	0.00262373	18.2677652	1.92E-05	0.00012879	Rilp
ENSMUSG00000038199.13	1.15933421	-0.0186941	0.07826336	0.11419132	0.73542192	0.8736168	Iqca1l
ENSMUSG00000038204.14	10.0153696	0.00517111	0.01712345	0.30615435	0.58004968	0.757953	Asb10
ENSMUSG00000038205.13	87.506645	0.00777175	0.00406691	3.64574702	0.05621258	0.1329686	Prkab2
ENSMUSG00000038206.14	1160.64071	-0.0082863	0.00229175	12.9544243	0.00031917	0.00160852	Fbxo8
ENSMUSG00000038208.14	32.1353984	0.01561729	0.0035941	19.0835457	1.25E-05	8.70E-05	Pgap3
ENSMUSG00000038212.17	738.009796	0.00346371	0.00208917	2.70558885	0.09999715	0.21018919	Mfsd14b
ENSMUSG00000038213.8	425.680111	0.01454478	0.00389543	13.7951325	0.00020386	0.00108467	Tapbpl
ENSMUSG00000038214.17	115.884887	-0.0081542	0.00291843	7.7542691	0.00535857	0.01876214	Bend3
ENSMUSG00000038215.16	175.522515	-0.007778	0.0033739	5.29283253	0.02141336	0.06021886	Cep44
ENSMUSG00000038217.14	1720.05465	-0.0251467	0.00577341	18.1920695	2.00E-05	0.00013363	Tlcd2
ENSMUSG00000038224.13	13070.9534	0.00028464	0.00505095	0.00317633	0.95505585	1	Serpinf2
ENSMUSG00000038225.16	124.218461	0.00895323	0.00371781	5.65222955	0.01743319	0.05081934	Primpol
ENSMUSG00000038227.16	6.67601014	0.01222755	0.02199192	0.42921332	0.51237521	0.69907339	Hoxa9
ENSMUSG00000038233.11	197.288854	-2.83E-05	0.00587373	1.60E-05	0.99680634	1	Gask1a
ENSMUSG00000038235.5	1370.93617	0.00295253	0.00282423	1.08885629	0.29672476	0.47997738	F11r
ENSMUSG00000038236.9	12.3961143	0.04173526	0.01703923	6.05691413	0.01385195	0.04198306	Hoxa7
ENSMUSG00000038239.12	281.548514	-0.0152987	0.02045971	0.91326013	0.33925089	0.52690448	Hrc
ENSMUSG00000038240.14	181.439237	-0.0106018	0.00244111	18.8138111	1.44E-05	9.92E-05	Pdss2
ENSMUSG00000038241.17	228.439726	0.01603298	0.00423983	14.0897659	0.00017429	0.00094588	Cep250
ENSMUSG00000038244.15	418.440017	-0.0141345	0.00391301	13.1275071	0.00029099	0.00148589	Mical2

ENSMUSG00000038246.8	3.99068257	-0.0013284	0.00981533	0.01571335	0.90024417	0.97587825	Fam50b
ENSMUSG00000038248.9	16.3111718	0.00199219	0.00975207	0.03522847	0.85111771	0.9483632	Sobp
ENSMUSG00000038250.10	459.77812	-0.006809	0.00187145	12.9964667	0.00031208	0.00157864	Usp38
ENSMUSG00000038252.14	90.5843684	0.00910431	0.00625084	2.0550329	0.15170398	0.28995867	Ncapd2
ENSMUSG00000038253.7	11.7749299	0.01619142	0.00607178	7.00705717	0.0081189	0.02674102	Hoxa5
ENSMUSG00000038255.7	11.9887308	-0.0033295	0.03261818	0.01608704	0.89907124	0.97523429	Neurod2
ENSMUSG00000038256.16	124.496788	0.0144698	0.00450616	10.3862269	0.00126959	0.0054208	Bcl9
ENSMUSG00000038257.11	16.3705911	0.0849181	0.01156106	43.9561087	3.36E-11	5.75E-10	Glra3
ENSMUSG00000038260.11	29.3338918	0.02449155	0.00655213	13.6206063	0.00022372	0.00117781	Trpm4
ENSMUSG00000038264.9	34.224472	0.07943638	0.01130513	46.8120677	7.81E-12	1.47E-10	Sema7a
ENSMUSG00000038267.16	1393.65516	-0.002119	0.00373506	0.32075643	0.57115341	0.75071857	Slc22a23
ENSMUSG00000038268.5	193.023261	-0.0037618	0.00309002	1.48117029	0.22359151	0.38894957	Ovca2
ENSMUSG00000038271.18	126.356045	0.00770919	0.00383091	4.0603023	0.04390264	0.10851893	Iffo1
ENSMUSG00000038274.14	5875.00102	0.00429657	0.00483469	0.80194058	0.3705138	0.56112357	Fau
ENSMUSG00000038276.13	2.59527598	-0.0233714	0.00943852	6.28711761	0.01216187	0.0375507	Asic3
ENSMUSG00000038279.11	307.16875	-0.006158	0.00287878	4.58799604	0.03219664	0.08431108	Nop2
ENSMUSG00000038280.13	337.580447	0.00643427	0.00214154	8.99802574	0.00270271	0.01045877	Ostm1
ENSMUSG00000038286.12	2235.84983	-0.0075177	0.00324269	5.33960954	0.02084614	0.05893289	Bphl
ENSMUSG00000038290.16	547.081598	0.00326995	0.00355206	0.83094145	0.36200044	0.55159522	Smg6
ENSMUSG00000038291.17	233.601638	0.0085612	0.00315501	7.17021856	0.00741237	0.02476321	Snx25
ENSMUSG00000038295.15	5.31971371	-0.0074251	0.00899321	0.64839804	0.42068601	0.61228711	Atg9b
ENSMUSG00000038296.15	29.4338417	-0.0025909	0.0042406	0.37032733	0.5428259	0.72637534	Galnt18
ENSMUSG00000038298.15	1152.28745	0.00334238	0.00396723	0.69510456	0.40443354	0.59599934	Pdzk1
ENSMUSG00000038299.17	427.351982	-0.0004446	0.00178721	0.06156838	0.804034	0.91805563	Wdr36
ENSMUSG00000038301.16	708.611674	0.01299058	0.00388999	10.5738184	0.00114701	0.0049539	Snx10
ENSMUSG00000038302.15	188.509562	-0.0162885	0.00280456	33.3640522	7.64E-09	9.40E-08	Afg1l
ENSMUSG00000038304.15	9.25469358	0.06014863	0.00823776	47.5656871	5.32E-12	1.02E-10	Cd160
ENSMUSG00000038305.16	50.4152243	0.01542276	0.006993	4.69713575	0.03021293	0.07995984	Spats2l
ENSMUSG00000038312.11	913.414911	-0.0084228	0.00243109	12.037043	0.00052154	0.00248492	Edem2
ENSMUSG00000038319.15	4.75181823	0.01297892	0.01636248	0.66590291	0.41448369	0.60588859	Kcnh2
ENSMUSG00000038323.4	709.130247	-0.0116561	0.00318627	13.0946246	0.00029614	0.00150763	1700066M21Rik
ENSMUSG00000038324.14	1577.71421	-0.0039204	0.0016424	5.68597475	0.01710104	0.05001799	Trpc4ap
ENSMUSG00000038331.16	23.5578758	0.01615244	0.00695527	5.03453609	0.02484674	0.06808821	Satb2
ENSMUSG00000038332.14	325.85972	0.00674879	0.00339035	3.91905227	0.04774179	0.11616415	Sesn1
ENSMUSG00000038335.14	352.7094	-0.007023	0.00300488	5.33840417	0.02086056	0.05895246	Tsr1
ENSMUSG00000038342.16	203.631494	0.00566148	0.00421411	1.79114474	0.18078675	0.33162516	Mlxip
ENSMUSG00000038344.15	618.179524	-0.0118737	0.00284637	16.9689666	3.80E-05	0.00023959	Txlng
ENSMUSG00000038346.20	406.423381	-0.0001462	0.00264841	0.00303527	0.95606412	1	Zfp384
ENSMUSG00000038349.11	39.4905818	0.01952075	0.00706836	7.27232616	0.00700251	0.02364173	Plcl1
ENSMUSG00000038351.15	150.264114	0.01357347	0.00338872	15.9658983	6.45E-05	0.00038588	Sgsm2
ENSMUSG00000038352.16	37.0505465	0.06873374	0.00846526	55.4197519	9.74E-14	2.31E-12	Arl5c
ENSMUSG00000038354.14	3.65618764	0.02961592	0.01030097	8.46061963	0.00362918	0.01343766	Ankrd35
ENSMUSG00000038357.11	23.4024912	0.17761503	0.02465306	11.8766257	0.00056843	0.0026755	Camp
ENSMUSG00000038365.9	115.794414	0.0051886	0.00223535	5.41002931	0.02002138	0.05699062	Fbxo25
ENSMUSG00000038366.16	2351.04275	0.00473527	0.00299117	2.47091124	0.11597067	0.23570837	Lasp1
ENSMUSG00000038368.17	201.955775	0.00319846	0.00331122	0.92371098	0.33650247	0.52387771	Focad
ENSMUSG00000038369.15	520.743976	0.00308532	0.00339208	0.80559911	0.36942458	0.55997739	Ncoa6
ENSMUSG00000038370.7	153.570974	-0.0218356	0.00655343	11.5034054	0.00069469	0.00319145	Pcp4l1
ENSMUSG00000038371.16	590.57512	0.01043775	0.00342009	9.25673473	0.00234631	0.00926314	Sbf2
ENSMUSG00000038372.15	42.9839577	0.01098064	0.00506193	4.45745956	0.03474914	0.08980859	Gmds
ENSMUSG00000038374.11	643.340997	-0.0067481	0.00366523	3.37143543	0.06633593	0.15167911	Rbm8a
ENSMUSG00000038375.16	3419.6716	-0.0357309	0.00339006	110.738974	6.75E-26	7.17E-24	Trp53inp2
ENSMUSG00000038379.16	11.4683483	0.03256144	0.01226795	6.57161135	0.01036181	0.03284861	Ttk
ENSMUSG00000038383.17	248.195764	-0.0025146	0.00234964	1.15991592	0.28148298	0.46123785	Pigu
ENSMUSG00000038384.17	484.020384	-0.0068283	0.0041224	2.74252293	0.09771039	0.20643114	Setd1b
ENSMUSG00000038387.10	352.852244	0.01676592	0.00297148	32.0700065	1.49E-08	1.76E-07	Rras
ENSMUSG00000038388.15	1332.33326	-0.0065231	0.00249795	6.79674744	0.00913241	0.02958621	Pals2
ENSMUSG00000038390.12	11.9098706	-0.0012573	0.00758809	0.02532237	0.87356642	0.96078049	Gpr162
ENSMUSG00000038393.15	2074.77995	0.00113224	0.00498579	0.04942332	0.8240698	0.93011008	Txnip
ENSMUSG00000038398.9	243.239566	-0.003344	0.00223059	2.2554488	0.13314485	0.26261536	Upf3a
ENSMUSG00000038400.16	109.24765	0.03968837	0.00647791	34.447421	4.38E-09	5.58E-08	Pmepa1

ENSMUSG00000038402.3	5.49114513	0.01462602	0.03404452	0.55293174	0.45712226	0.64899088	Foxf2
ENSMUSG00000038403.11	1135.72384	-0.0002483	0.00322514	0.00591805	0.93868014	0.99608629	Hjv
ENSMUSG00000038406.18	588.777607	-0.0048431	0.00386575	1.55658944	0.21216529	0.37444856	Scaf1
ENSMUSG00000038412.9	1220.8495	-0.0297255	0.00448644	44.0585143	3.19E-11	5.47E-10	Higd1a
ENSMUSG00000038415.11	487.438563	-0.0141239	0.00763873	3.136589	0.07655375	0.17022691	Foxq1
ENSMUSG00000038416.16	421.783367	-0.0032984	0.00234576	1.9988471	0.1574189	0.29800805	Cdc16
ENSMUSG00000038417.5	252.062179	-0.0052154	0.00253042	4.28542672	0.03844043	0.09730784	Fig4
ENSMUSG00000038418.9	571.032999	0.00817023	0.00971598	0.61374836	0.43337997	0.62498948	Egr1
ENSMUSG00000038421.14	14.3993949	0.0719973	0.00756963	77.2324007	1.52E-18	6.55E-17	Fcrla
ENSMUSG00000038422.3	386.648191	-0.0164377	0.004476	13.7091125	0.00021342	0.00113014	Hdhd3
ENSMUSG00000038425.19	97.3389033	-0.0094842	0.00355294	7.10229372	0.00769854	0.02555633	Poli
ENSMUSG00000038429.11	1067.41305	-0.0035127	0.00200007	3.0887179	0.0788369	0.17412184	Usp5
ENSMUSG00000038437.12	418.228109	0.01067782	0.00308439	11.8571473	0.0005744	0.00270067	Mllt6
ENSMUSG00000038446.9	530.76518	-0.0048535	0.00304686	2.51393656	0.11284373	0.23065537	Cdc40
ENSMUSG00000038451.14	198.011227	-6.33E-05	0.00274571	0.00052017	0.98180395	1	Spsb2
ENSMUSG00000038453.18	61.6705657	0.01555372	0.00829366	3.31810345	0.06852083	0.15563678	Srcin1
ENSMUSG00000038456.10	78.1403599	0.01408913	0.00319429	18.8837145	1.39E-05	9.60E-05	Dennd2a
ENSMUSG00000038459.11	1249.35768	-0.0059222	0.00313446	3.47702418	0.06222655	0.14401681	Abhd17c
ENSMUSG00000038462.4	3596.32433	-0.0172127	0.00273601	40.0056398	2.53E-10	3.83E-09	Uqcrfs1
ENSMUSG00000038463.9	13.2592528	-0.0043273	0.00885655	0.23217565	0.62991529	0.79834401	Olfml2b
ENSMUSG00000038467.16	896.90037	0.00918251	0.00270827	11.3527141	0.00075338	0.00342307	Chmp4b
ENSMUSG00000038473.15	196.772653	0.01730388	0.00763602	4.6813299	0.03049213	0.08059026	Nos1ap
ENSMUSG00000038481.14	171.236859	0.01211874	0.00287763	17.3996415	3.03E-05	0.00019426	Cdk19
ENSMUSG00000038482.12	401.457135	0.00269261	0.00235695	1.29275283	0.25554126	0.42836765	Tfdp1
ENSMUSG00000038485.7	284.883153	-0.0087818	0.00337385	6.75645887	0.00934089	0.03016765	Socs7
ENSMUSG00000038486.10	27.5632341	0.02323896	0.01161976	3.48869908	0.06178912	0.14327156	Sv2a
ENSMUSG00000038489.9	662.376983	-0.0106011	0.00447972	5.60707412	0.01788811	0.05192725	Polr2l
ENSMUSG00000038495.15	568.077104	0.00709996	0.00298015	5.5382005	0.01860571	0.05361506	Otud7b
ENSMUSG00000038496.17	2.6852871	0.11220545	0.01288576	70.7276223	4.10E-17	1.51E-15	Slc19a3
ENSMUSG00000038497.8	129.871503	0.00945383	0.00361718	6.70348374	0.00962247	0.03089513	Tmco3
ENSMUSG00000038500.16	109.498548	-0.0046156	0.00398079	1.33833674	0.2473275	0.41865906	Prr3
ENSMUSG00000038502.18	691.110825	-0.0098961	0.00479164	4.17981263	0.04090822	0.10250307	Ptov1
ENSMUSG00000038503.16	655.764638	-0.0011184	0.00375913	0.08811548	0.76658674	0.89468627	Mesd
ENSMUSG00000038506.14	244.933365	-0.0136617	0.00269225	25.8420897	3.71E-07	3.47E-06	Dcun1d2
ENSMUSG00000038507.7	690.400986	0.01452422	0.00240169	36.8265895	1.29E-09	1.76E-08	Parp12
ENSMUSG00000038508.8	122.101269	0.0329544	0.00674235	21.9476153	2.80E-06	2.23E-05	Gdf15
ENSMUSG00000038510.13	189.255632	-0.0008568	0.00365277	0.05506364	0.81447541	0.92414572	Rpf2
ENSMUSG00000038515.11	349.350968	-0.0202406	0.0037842	28.4725165	9.50E-08	9.92E-07	Grtp1
ENSMUSG00000038517.16	157.556653	0.01868665	0.00281313	42.9820752	5.52E-11	9.20E-10	Tbkbp1
ENSMUSG00000038518.16	251.33961	0.00031818	0.00318808	0.00994016	0.92058222	0.98678232	Jarid2
ENSMUSG00000038520.16	520.28695	0.00403394	0.00415375	0.93549885	0.33343812	0.52030906	Tbc1d17
ENSMUSG00000038521.18	8821.20316	0.00221729	0.00309477	0.50578297	0.4769701	0.66705109	C1s1
ENSMUSG00000038522.8	971.130931	-0.0165969	0.00440578	14.3641741	0.00015064	0.00082667	Mfsd4b1
ENSMUSG00000038523.11	3.19304634	0.03156061	0.01320824	5.22316895	0.0222879	0.06227727	1700003F12Rik
ENSMUSG00000038524.14	33.8998915	0.03583443	0.00759559	21.0364137	4.51E-06	3.44E-05	Fchsd1
ENSMUSG00000038525.17	158.342771	0.01129848	0.0024229	21.461805	3.61E-06	2.82E-05	Armc10
ENSMUSG00000038526.15	586.101228	-0.0376334	0.00335691	130.737056	2.83E-30	6.17E-28	Car14
ENSMUSG00000038527.10	2902.98107	-0.0050843	0.00433391	1.36371785	0.24289418	0.41316185	C1rl
ENSMUSG00000038528.16	50.0759654	0.00768952	0.00879667	0.75029802	0.38638189	0.57782275	Mfsd4b5
ENSMUSG00000038530.12	60.5439811	0.03057033	0.00573102	26.8316786	2.22E-07	2.17E-06	Rgs4
ENSMUSG00000038533.16	264.225747	-0.0006883	0.00294946	0.0541685	0.81596233	0.92521556	Cbfa2t2
ENSMUSG00000038534.21	37.8180977	0.02224169	0.00475903	21.7197141	3.16E-06	2.49E-05	Osbpl7
ENSMUSG00000038535.18	276.494084	-0.0002158	0.00342501	0.00391152	0.9501311	1	Zfp280d
ENSMUSG00000038538.18	1251.60424	-0.0044549	0.0050104	0.77881331	0.37750432	0.5686433	Ubn2
ENSMUSG00000038539.16	4097.32677	0.00143861	0.00389455	0.13260676	0.71574442	0.86007479	Atf5
ENSMUSG00000038540.15	20.6078189	0.09237627	0.01017446	70.0343499	5.83E-17	2.09E-15	Tmc3
ENSMUSG00000038541.9	38.1839669	0.01232307	0.00469321	6.72482141	0.00950803	0.03059431	Srd5a2
ENSMUSG00000038542.15	220.378917	-0.0054909	0.0031941	2.92364031	0.08729073	0.18893633	Pcid2
ENSMUSG00000038543.14	47.9661734	0.02823181	0.00609186	20.0402717	7.58E-06	5.50E-05	BC028528
ENSMUSG00000038544.15	118.565127	0.00820446	0.0028348	8.16869914	0.00426195	0.01543647	Inip
ENSMUSG00000038545.14	143.8314	0.01060146	0.00381672	7.61175673	0.0057989	0.02007232	Cul7

ENSMUSG00000038546.10	648.936554	-0.007358	0.00272711	7.02177086	0.00805246	0.0265444	Ranbp9
ENSMUSG00000038550.11	106.98784	0.01329248	0.01207942	1.12107982	0.28968606	0.47098197	Ciart
ENSMUSG00000038552.15	641.898059	-0.006109	0.00382523	2.54674839	0.11052151	0.22704993	Fndc4
ENSMUSG00000038555.8	4.30717001	0.02709392	0.01039849	6.68727612	0.00971035	0.03113489	Reep2
ENSMUSG00000038560.8	2.41849944	0.02517837	0.01092061	5.24662253	0.02198941	0.06154531	Sp6
ENSMUSG00000038563.15	209.731753	0.00367063	0.00212881	2.94654026	0.08606162	0.18682733	Efl1
ENSMUSG00000038564.12	249.112396	-0.0060156	0.00380909	2.47305869	0.11581236	0.2354924	Ift172
ENSMUSG00000038567.6	3.98697206	0.00605474	0.03495029	0.06386081	0.80049466	0.91618159	Cyp24a1
ENSMUSG00000038569.14	38.8060259	0.01100479	0.00432266	6.36818838	0.01161841	0.03612338	Rad9b
ENSMUSG00000038570.16	12.6933332	-0.0127891	0.0087155	2.04817743	0.15238852	0.29084283	Saxo2
ENSMUSG00000038576.16	443.638902	-0.0282663	0.00698304	14.2844426	0.00015716	0.00085923	Susd4
ENSMUSG00000038578.16	133.368384	0.00510168	0.00393169	1.62560016	0.20231269	0.36140324	Susd1
ENSMUSG00000038582.16	413.406408	-0.0089077	0.00327658	7.44319987	0.00636766	0.02174731	Pptc7
ENSMUSG00000038583.14	850.002106	-0.0132556	0.01533042	0.1100283	0.74011191	0.87693558	Pln
ENSMUSG00000038587.11	132.427523	0.00049051	0.0052698	0.0081634	0.92800789	0.99048799	Akap12
ENSMUSG00000038591.4	587.755488	0.0063367	0.00465734	1.78681758	0.18131439	0.33238623	Colec10
ENSMUSG00000038593.19	67.1202828	0.01246851	0.00386192	10.646142	0.00110301	0.00478491	Tctn1
ENSMUSG00000038594.10	702.931459	-0.011735	0.00418017	7.91160745	0.00491186	0.01741075	Cep85l
ENSMUSG00000038599.15	57.0500811	-0.0321657	0.00958649	9.21254836	0.00240362	0.00944716	Capn8
ENSMUSG00000038600.13	17.0316315	-0.0151829	0.01110397	1.64767079	0.19927625	0.35723414	Atp6v0a4
ENSMUSG00000038602.8	8.75031384	-0.0696503	0.012546	30.7656256	2.91E-08	3.28E-07	Slc35f1
ENSMUSG00000038604.10	293.221163	-0.0081268	0.00372206	4.82604461	0.02803287	0.07522603	Ripor1
ENSMUSG00000038605.12	27.1800126	-0.015812	0.0049391	10.3682333	0.00128202	0.00546301	Samd10
ENSMUSG00000038607.13	239.138037	0.00424376	0.00421164	1.00207297	0.31680943	0.5022603	Gng10
ENSMUSG00000038608.16	176.234227	0.05545747	0.00495367	116.96428	2.92E-27	3.79E-25	Dock10
ENSMUSG00000038611.18	411.383802	-0.0048714	0.00362846	1.81954207	0.17736779	0.32670026	Phrf1
ENSMUSG00000038612.17	2150.84737	0.00987299	0.00194841	24.8313886	6.26E-07	5.60E-06	Mcl1
ENSMUSG00000038615.18	3052.5534	-0.0036654	0.00303155	1.43692548	0.23063751	0.39820045	Nfe2l1
ENSMUSG00000038618.13	354.663519	0.0020877	0.00355123	0.34894091	0.55471327	0.73693721	Rassf7
ENSMUSG00000038619.13	819.133256	0.00296976	0.00200078	2.1973393	0.13824918	0.27024244	Ensa
ENSMUSG00000038622.9	124.206466	0.00347417	0.00388666	0.79782577	0.37174422	0.56230099	Med30
ENSMUSG00000038623.10	83.1664006	0.03764829	0.00561869	41.9269938	9.47E-11	1.52E-09	Tm6sf1
ENSMUSG00000038624.14	1.71687553	0.00185016	0.01974173	0.01318987	0.90856628	0.98015409	Nepn
ENSMUSG00000038628.9	411.129807	-0.0045235	0.00253518	3.09250667	0.07865356	0.17389246	Polr3k
ENSMUSG00000038633.6	1522.95499	0.00838004	0.00182281	21.1129123	4.33E-06	3.32E-05	Degs1
ENSMUSG00000038637.16	63.6200893	-0.0048035	0.00274632	3.03088711	0.08169334	0.17917232	Lrrc56
ENSMUSG00000038641.13	3637.64038	0.00442152	0.00472684	0.86220213	0.35312317	0.54174341	Akr1d1
ENSMUSG00000038642.11	1109.21439	0.06169571	0.0048808	149.800668	1.92E-34	6.95E-32	Ctss
ENSMUSG00000038644.15	81.9396951	0.0183998	0.00577495	10.0674244	0.00150914	0.00630794	Pold1
ENSMUSG00000038646.14	717.499114	0.00289981	0.0020375	2.02587716	0.15463961	0.29406836	Ramac
ENSMUSG00000038648.6	502.002677	0.00134032	0.00394644	0.11412268	0.73549847	0.87362698	Creb3l2
ENSMUSG00000038650.16	1565.69559	-0.0047113	0.00218573	4.66996886	0.03069448	0.08103628	Rnh1
ENSMUSG00000038656.6	387.444256	0.02105119	0.01275482	2.7788555	0.0955164	0.2027913	Cyp3a16
ENSMUSG00000038658.16	570.463153	-0.0009159	0.00273436	0.11059947	0.73946268	0.87634241	Ric1
ENSMUSG00000038663.8	84.7669152	0.0594744	0.01324386	8.28373089	0.00400019	0.01462539	Fsd2
ENSMUSG00000038664.17	988.625144	0.00672044	0.00396989	2.80774134	0.09381037	0.20000103	Herc1
ENSMUSG00000038665.16	2.04900539	0.06010106	0.02192819	8.6804733	0.00321637	0.01212037	Dgki
ENSMUSG00000038668.15	44.2802019	0.02945277	0.00614513	21.6045169	3.35E-06	2.63E-05	Lpar1
ENSMUSG00000038670.12	7.82716646	0.00965659	0.01821503	2.61581976	0.10580284	0.21922742	Mybpc2
ENSMUSG00000038671.16	283.258824	-0.003162	0.00228904	1.92807539	0.16496916	0.30883107	Arfrp1
ENSMUSG00000038677.15	8.36017257	0.04376034	0.01108091	13.6936329	0.00021518	0.00113898	Scube3
ENSMUSG00000038679.18	54.8723457	0.05845123	0.0062991	78.0329588	1.01E-18	4.49E-17	Trps1
ENSMUSG00000038683.17	684.536382	-0.0030457	0.00211703	2.07359569	0.14986782	0.28735733	Pak1ip1
ENSMUSG00000038685.19	187.15163	0.00492545	0.00374543	1.73533599	0.18773087	0.34120524	Rtel1
ENSMUSG00000038690.16	3749.46019	-0.0169258	0.00399424	18.161245	2.03E-05	0.00013563	Atp5mf
ENSMUSG00000038692.9	65.3329112	0.01728658	0.00692544	6.13353036	0.01326418	0.04039887	Hoxb4
ENSMUSG00000038695.14	539.38025	-0.0198634	0.00422165	22.0198871	2.70E-06	2.16E-05	Josd2
ENSMUSG00000038696.15	661.842587	-0.0050271	0.00253093	3.93961042	0.0471617	0.11515071	Mapkap1
ENSMUSG00000038697.16	264.126825	-0.00514	0.00192467	7.16718636	0.00742491	0.02480159	Taf5l
ENSMUSG00000038700.5	12.8712124	0.01133069	0.00528901	4.56008661	0.03272542	0.08543018	Hoxb5
ENSMUSG00000038702.7	31.0560933	0.02023555	0.00503836	15.2225431	9.56E-05	0.00054863	Dsel

ENSMUSG00000038704.13	1324.88051	-0.0145263	0.00450439	10.280723	0.00134427	0.00569126	Aspdh
ENSMUSG00000038705.14	240.021539	-0.0021021	0.00214684	0.94229609	0.33168804	0.51871066	Gmeb2
ENSMUSG00000038708.11	1684.70051	-0.0089466	0.00303606	8.57278281	0.00341225	0.01275054	Golga4
ENSMUSG00000038712.17	1433.51044	-0.0071425	0.00296968	5.76943907	0.01630724	0.0480658	Mindy1
ENSMUSG00000038717.10	5141.25033	-0.0165485	0.00477029	11.9839842	0.0005366	0.00254908	Atp5mg
ENSMUSG00000038718.16	60.0145803	0.00618831	0.00409396	2.23141247	0.13523017	0.26563995	Pbx3
ENSMUSG00000038721.9	7.77477996	0.06823671	0.00845382	62.4260521	2.77E-15	8.09E-14	Hoxb7
ENSMUSG00000038722.18	1031.50238	-0.0113724	0.0036579	9.66588538	0.00187721	0.00764697	Bud31
ENSMUSG00000038725.14	24.7809468	0.04211967	0.0098173	15.7245216	7.33E-05	0.00043278	Pkhd1l1
ENSMUSG00000038729.25	341.872729	0.01316817	0.00414537	9.73444265	0.00180847	0.00739247	Pakap
ENSMUSG00000038732.16	23.6734995	0.02906087	0.0076055	14.0029054	0.00018253	0.00098448	Mboat1
ENSMUSG00000038733.15	2022.3279	-0.001763	0.00281205	0.38355907	0.53570461	0.7200491	Wdr26
ENSMUSG00000038736.11	337.214417	-0.0041321	0.00251956	2.67025392	0.10223971	0.21373306	Nudcd1
ENSMUSG00000038738.16	24.6910526	0.04112883	0.01133018	12.4179869	0.00042522	0.00207129	Shank1
ENSMUSG00000038740.10	54.8393729	0.03043263	0.0058591	25.7769044	3.83E-07	3.58E-06	Mvb12b
ENSMUSG00000038742.8	204.325219	-0.0104347	0.00309401	11.4965263	0.00069726	0.00320079	Angptl6
ENSMUSG00000038745.16	2188.66732	-0.0139686	0.00515159	7.2586459	0.00705605	0.02377163	Nlrp6
ENSMUSG00000038751.6	36.4724356	-0.0008306	0.0077666	0.01114599	0.91591991	0.98396206	Ptk6
ENSMUSG00000038754.6	4478.07172	-0.0835161	0.01020464	54.9335732	1.25E-13	2.93E-12	Elovl3
ENSMUSG00000038756.14	1.30064176	-0.0166435	0.02102331	0.51484861	0.47304743	0.66373835	Ttll6
ENSMUSG00000038759.17	227.845163	0.00284824	0.00237644	1.4357052	0.2308356	0.39839664	Nup205
ENSMUSG00000038760.15	2.28486564	0.01085007	0.03144212	0.04195717	0.83770144	0.93906968	Trhr
ENSMUSG00000038762.16	2820.05282	-0.0021724	0.00288972	0.55733297	0.45533683	0.64708502	Abcf1
ENSMUSG00000038763.13	18.2280275	-0.0078914	0.01514307	-3.33521	1	1	Alpk3
ENSMUSG00000038764.15	662.065634	-0.0035298	0.00361652	0.94132733	0.33193672	0.51889876	Ptpn3
ENSMUSG00000038766.17	608.884182	0.00137326	0.00265854	0.26372227	0.60757436	0.78034464	Gabpb2
ENSMUSG00000038768.9	290.089687	-0.0075784	0.0061722	1.34578783	0.24601585	0.41711809	Degs1l
ENSMUSG00000038773.12	474.540044	-1.86E-05	0.0024953	5.47E-05	0.99409763	1	Kdm3b
ENSMUSG00000038774.10	651.344493	-0.0018415	0.00440722	0.17316211	0.6773167	0.83321294	Ascc3
ENSMUSG00000038775.15	32.0721835	0.04193921	0.00797716	25.4015159	4.66E-07	4.26E-06	Vill
ENSMUSG00000038776.14	5349.33393	-0.0047119	0.00371888	1.53925562	0.21472864	0.37772894	Ephx1
ENSMUSG00000038777.20	28.0211564	0.01386248	0.0061182	4.93423857	0.02632966	0.0714381	Sema6c
ENSMUSG00000038780.15	348.175008	0.00429897	0.00309561	1.9255863	0.16524214	0.30926845	Smurf1
ENSMUSG00000038781.8	604.829824	-0.0069661	0.00301017	5.43418632	0.01974629	0.05628909	Stap2
ENSMUSG00000038782.8	4.42955278	-0.0084231	0.00725569	1.33554141	0.24782178	0.41909547	1700028J19Rik
ENSMUSG00000038784.14	283.549679	0.00196421	0.00264447	0.54457461	0.46054302	0.65188548	Cnot4
ENSMUSG00000038791.15	3.8943934	-0.0132051	0.07022037	0.06412824	0.8000862	0.91588202	Scgb3a2
ENSMUSG00000038793.9	11.6115367	0.01869043	0.00683626	7.34729047	0.00671639	0.02281619	Lefty1
ENSMUSG00000038797.14	42.7815862	0.0047578	0.00348663	1.87725146	0.17064687	0.31709422	Zscan2
ENSMUSG00000038801.7	1.83326579	0.00392891	0.01381244	0.09449783	0.75853513	0.88922599	Scgb1c1
ENSMUSG00000038803.8	1198.93331	-0.0027652	0.00319991	0.74010212	0.38962817	0.58115285	Ost4
ENSMUSG00000038805.11	2.25895116	0.01616524	0.02830989	0.58358506	0.44491051	0.63724988	Six3
ENSMUSG00000038806.8	237.240224	-0.0072663	0.00268128	7.25490802	0.00707075	0.02381078	Sde2
ENSMUSG00000038807.20	48.4560612	0.04977612	0.00732426	43.450625	4.35E-11	7.31E-10	Rap1gap2
ENSMUSG00000038811.14	187.147896	0.04368108	0.00580708	52.0064133	5.53E-13	1.20E-11	Gngt2
ENSMUSG00000038812.17	459.499066	-0.0012824	0.00243893	0.27879244	0.59749379	0.7721672	Trmt112
ENSMUSG00000038816.15	105.650663	-0.000792	0.00361266	0.04730067	0.82782871	0.93249935	Ctnnal1
ENSMUSG00000038822.16	98.0023962	0.00939298	0.00411869	5.15766656	0.02314397	0.06422044	Hace1
ENSMUSG00000038827.12	73.3228503	0.00508427	0.00337342	2.24601009	0.13395941	0.26373678	Abitram
ENSMUSG00000038828.14	986.172547	0.00068994	0.00229019	0.09027019	0.76383395	0.89280137	Tmem214
ENSMUSG00000038831.17	126.030784	-0.0091178	0.00452022	4.08016928	0.04338931	0.10754312	Ralgps1
ENSMUSG00000038836.16	139.484592	-0.0056975	0.0035682	2.52629613	0.11196275	0.2293316	Agbl3
ENSMUSG00000038838.16	335.811647	-0.0152931	0.00324129	21.937053	2.82E-06	2.24E-05	Vars2
ENSMUSG00000038843.19	84.2898237	0.07351659	0.00727874	92.0121789	8.61E-22	5.51E-20	Gcnt1
ENSMUSG00000038844.11	312.057202	0.0064095	0.00279419	5.26424972	0.0217678	0.06100121	Kif16b
ENSMUSG00000038845.12	2604.66413	-0.0163601	0.00343376	22.4154175	2.20E-06	1.78E-05	Phb
ENSMUSG00000038848.15	669.110546	-0.0055476	0.00175967	9.91966809	0.00163522	0.00676043	Ythdf1
ENSMUSG00000038855.11	226.080737	0.01121564	0.0035364	9.72738793	0.00181542	0.00741704	Itpkb
ENSMUSG00000038859.8	703.879338	0.0025574	0.00315104	0.65620892	0.41790154	0.60941341	Baiap2l1
ENSMUSG00000038860.16	9.15892172	0.00854783	0.0094522	0.76155629	0.38284193	0.5744821	Garnl3
ENSMUSG00000038861.15	597.743698	0.00120575	0.00267543	0.20562799	0.65021594	0.81336479	Pi4kb

ENSMUSG00000038866.16	578.703943	-0.0057855	0.00322066	3.16597412	0.07518757	0.16772666	Zcchc2
ENSMUSG00000038871.6	305.027552	-0.0091574	0.0030437	9.15453587	0.00248102	0.00970116	Bpgm
ENSMUSG00000038872.11	408.649501	-0.0038392	0.00403422	0.90433441	0.34162215	0.52943689	Zfhx3
ENSMUSG00000038876.13	530.909518	-0.0047118	0.00165969	8.06066835	0.00452365	0.01622981	Rnf146
ENSMUSG00000038879.9	11.0115079	0.00707513	0.00857723	0.63892017	0.42410209	0.61571291	Nipal2
ENSMUSG00000038880.14	646.792537	-0.010224	0.00283749	13.2292707	0.00027561	0.00141624	Mrps34
ENSMUSG00000038884.15	1169.378	0.00530987	0.00466692	1.27454839	0.25891514	0.43252159	Shfl
ENSMUSG00000038886.11	665.000685	-0.0037666	0.00302203	1.53340843	0.21560162	0.37895371	Man2a2
ENSMUSG00000038888.9	90.5743871	9.43E-05	0.00272538	0.00119851	0.97238318	1	Ctu1
ENSMUSG00000038893.13	38.8350329	0.00953397	0.00491731	3.770059	0.05217779	0.12478862	Fam117a
ENSMUSG00000038894.8	504.320708	-0.0106367	0.00568831	3.38091205	0.06595555	0.15098782	Irs2
ENSMUSG00000038895.17	88.2080713	-0.0049969	0.00321902	2.42613599	0.11932671	0.24116879	Zfp653
ENSMUSG00000038900.18	6363.01708	-0.0012158	0.00416348	0.08528694	0.7702566	0.89697206	Rpl12
ENSMUSG00000038902.16	321.734637	-0.000569	0.00350294	0.02627809	0.87122302	0.95936627	Pogz
ENSMUSG00000038903.14	42.805817	0.01913095	0.00394578	23.5071403	1.24E-06	1.05E-05	Ccdc68
ENSMUSG00000038909.18	457.685541	0.00199379	0.00281944	0.4933946	0.48241695	0.67163213	Kat7
ENSMUSG00000038910.8	306.819814	0.01885891	0.0031748	35.0115399	3.28E-09	4.26E-08	Plcl2
ENSMUSG00000038914.16	764.648989	0.00047822	0.00249301	0.03720774	0.8470427	0.94522847	Dido1
ENSMUSG00000038916.8	13.5342904	0.01517517	0.02531556	0.39816313	0.52803942	0.71284285	Soga3
ENSMUSG00000038930.12	53.4316186	0.00124421	0.0055031	0.05100979	0.82131538	0.92863849	Rccd1
ENSMUSG00000038932.14	5.2659511	0.00172861	0.03143048	0.02752589	0.86822835	0.95794704	Tcfl5
ENSMUSG00000038936.14	85.0002827	-0.0065554	0.0047213	1.97613602	0.15979806	0.30131871	Sccpdh
ENSMUSG00000038943.17	35.5050604	0.02844269	0.00960107	8.29533219	0.00397471	0.01453675	Prc1
ENSMUSG00000038949.9	236.392666	0.00038987	0.0033579	0.01362898	0.90706354	0.97942966	Cnst
ENSMUSG00000038954.15	104.915638	0.0100077	0.00357296	7.69274699	0.00554432	0.01931014	Supt3
ENSMUSG00000038957.14	280.390195	-0.0045827	0.00213079	4.60539052	0.03187159	0.08363634	Edc3
ENSMUSG00000038963.16	7.09808657	0.0443664	0.00973313	19.2285399	1.16E-05	8.11E-05	Slco4a1
ENSMUSG00000038965.18	1972.24073	0.0003601	0.0023782	0.02264812	0.88037573	0.96408053	Ube2l3
ENSMUSG00000038967.14	1757.34731	-0.0105733	0.00334498	9.98395298	0.0015791	0.00655403	Pdk2
ENSMUSG00000038970.8	316.465976	-0.003589	0.00336036	1.13703391	0.28627995	0.46695705	Lmtk2
ENSMUSG00000038973.3	2.13001283	0.02605441	0.02133208	2.30634726	0.12884652	0.2561077	Cldnd2
ENSMUSG00000038975.15	751.005595	-0.0105478	0.00213208	24.6357215	6.93E-07	6.13E-06	Rabggtb
ENSMUSG00000038976.13	241.242725	0.03449724	0.00442198	58.7507286	1.79E-14	4.75E-13	Ppp1r9b
ENSMUSG00000038982.11	598.21842	0.00692007	0.00338069	4.15399946	0.04153631	0.10375718	Bloc1s5
ENSMUSG00000038984.5	16.2648764	0.0204909	0.00606407	10.5282566	0.00117563	0.00505993	Tspyl5
ENSMUSG00000038990.15	509.2539	-0.0058561	0.0026107	4.94133321	0.0262218	0.07123716	Cables2
ENSMUSG00000038991.18	3095.80366	0.00101646	0.0021474	0.22224186	0.63733702	0.80417763	Txndc5
ENSMUSG00000039000.9	844.504663	-0.0023981	0.00184487	1.67950069	0.19499081	0.35107277	Ube3c
ENSMUSG00000039001.13	3531.79286	-0.0081212	0.00544506	2.26164636	0.13261301	0.26191681	Rps21
ENSMUSG00000039004.7	127.931898	0.01117813	0.00398006	7.66654315	0.00562541	0.01956081	Bmp6
ENSMUSG00000039005.14	74.1311318	0.03149266	0.00626228	22.8344138	1.77E-06	1.46E-05	Tlr4
ENSMUSG00000039007.11	1259.71761	0.00374279	0.00181815	4.2500215	0.03924983	0.09902092	Cpq
ENSMUSG00000039013.17	3.04824427	0.0748691	0.0105908	48.7540941	2.90E-12	5.77E-11	Siglecf
ENSMUSG00000039016.6	1214.24285	-0.0166485	0.00370707	20.2519734	6.79E-06	5.00E-05	Timm8b
ENSMUSG00000039018.16	249.589362	-0.0105953	0.00364156	8.49432486	0.00356256	0.01324086	Mtg1
ENSMUSG00000039021.16	4.35474356	0.01571744	0.0089297	2.99450215	0.08354759	0.18235734	Ttc16
ENSMUSG00000039031.17	300.645703	0.01654569	0.00327441	24.5984629	7.06E-07	6.23E-06	Arhgap18
ENSMUSG00000039033.12	157.044371	0.00535845	0.00216288	6.14413197	0.01318489	0.04019891	Tasp1
ENSMUSG00000039037.6	7.19669454	0.0210327	0.01672359	1.6320626	0.201418	0.36022482	St6galnac5
ENSMUSG00000039041.16	1247.53489	-0.0079512	0.00234997	11.4772849	0.00070452	0.00322844	Adrm1
ENSMUSG00000039043.6	137.59073	0.00802494	0.00251371	10.2173918	0.00139122	0.00585849	Arpin
ENSMUSG00000039046.16	557.61458	-0.0042438	0.00346889	1.47870928	0.22397658	0.3894369	Usp6nl
ENSMUSG00000039047.18	698.158579	-0.0004373	0.00188138	0.05393665	0.81634956	0.92536206	Pigk
ENSMUSG00000039048.16	613.03609	-0.0132232	0.00301025	19.3669421	1.08E-05	7.60E-05	Foxred1
ENSMUSG00000039050.9	515.593914	-0.0003433	0.00238025	0.02064267	0.88575665	0.96724183	Osbpl2
ENSMUSG00000039055.5	4.01385132	0.0340668	0.01165548	8.04046519	0.00457438	0.01637694	Eme1
ENSMUSG00000039058.12	9.15791191	0.03467802	0.01387963	5.62534229	0.01770261	0.05147142	Ak5
ENSMUSG00000039059.12	2.06541056	-0.0067594	0.02807958	0.14320826	0.70511217	0.85347304	Hrh3
ENSMUSG00000039062.16	2016.91197	0.0026175	0.00394467	0.44564767	0.50440908	0.69128269	Anpep
ENSMUSG00000039063.6	1442.90779	0.00843201	0.00274828	9.39289633	0.00217828	0.00870204	Echdc3
ENSMUSG00000039065.12	260.658613	-0.0060944	0.00217272	7.9630711	0.00477413	0.0169914	Atpsckmt

ENSMUSG00000039067.6	1520.71792	-0.0053858	0.00288162	3.46480776	0.06268779	0.1449841	Psmd7
ENSMUSG00000039068.17	539.355538	0.00542915	0.00241264	5.03177071	0.02488644	0.06818116	Zzz3
ENSMUSG00000039069.20	260.560566	-0.0138875	0.00238813	34.3073647	4.71E-09	5.97E-08	Mtg2
ENSMUSG00000039081.11	56.6247245	0.01882643	0.00442368	17.5307026	2.83E-05	0.00018303	Zfp503
ENSMUSG00000039084.9	4.22382503	-0.0010932	0.01505071	-0.0176465	1	1	Chad
ENSMUSG00000039086.10	104.337246	-0.0006765	0.00284519	0.05654886	0.81203619	0.92275417	Ss18l1
ENSMUSG00000039087.18	764.752285	-0.005544	0.00389363	1.98903785	0.15844152	0.2993716	Rreb1
ENSMUSG00000039089.16	113.169789	0.00045406	0.00306099	0.02247341	0.88083456	0.9643225	L3mbtl3
ENSMUSG00000039092.10	0.70259158	0.07480473	0.03128896	5.94802788	0.01473376	0.04424067	Sptlc3
ENSMUSG00000039095.9	2.48011365	0.04476559	0.01594388	6.51920501	0.01067157	0.03372634	En2
ENSMUSG00000039096.11	126.333973	-0.0131496	0.00406906	10.2462588	0.00136962	0.00577887	Rsad1
ENSMUSG00000039099.8	14.2813209	-0.0045791	0.00491668	0.85481182	0.35519465	0.54383038	Wdr93
ENSMUSG00000039100.11	2301.64266	-0.0066497	0.00246031	7.23446866	0.0071517	0.02405246	Marchf6
ENSMUSG00000039103.13	1716.63188	0.01006169	0.00778545	1.54824568	0.21339461	0.3760273	Nexn
ENSMUSG00000039105.7	1619.63466	0.00308779	0.00317351	0.9524945	0.32908515	0.51600867	Atp6v1g1
ENSMUSG00000039108.15	356.678755	-0.002807	0.0024014	1.35741105	0.24398667	0.41445226	Lsm14b
ENSMUSG00000039109.18	39.9015914	0.08795309	0.01022221	55.4989519	9.35E-14	2.22E-12	F13a1
ENSMUSG00000039110.16	5.83111098	-0.0189408	0.01266745	2.16618778	0.14107558	0.27444808	Mycbpap
ENSMUSG00000039114.17	4793.959	0.00766115	0.00375129	4.17885899	0.04093125	0.10254987	Nrn1
ENSMUSG00000039115.14	449.227261	0.02045125	0.00435108	20.8622099	4.94E-06	3.74E-05	Itga9
ENSMUSG00000039116.8	41.7623145	0.01611358	0.00563129	8.09708309	0.00443366	0.01596766	Adgrg6
ENSMUSG00000039117.17	261.366026	-0.0017807	0.00463768	0.14226568	0.70603893	0.85410796	Taf4
ENSMUSG00000039126.11	56.0468434	0.07307598	0.01022444	42.2002814	8.24E-11	1.34E-09	Prune2
ENSMUSG00000039128.14	728.759803	-0.0080419	0.00194198	17.1810083	3.40E-05	0.00021592	Cdc123
ENSMUSG00000039130.19	149.162702	-0.004773	0.00282475	2.87274637	0.09009101	0.19364683	Zc3hc1
ENSMUSG00000039131.16	26.2789002	0.0596328	0.01130031	26.3359405	2.87E-07	2.74E-06	Gipc2
ENSMUSG00000039137.19	20.6693736	-0.0037821	0.00736109	0.24240311	0.62247583	0.79232136	Whrn
ENSMUSG00000039145.17	1874.28342	0.01107773	0.00534024	4.11979891	0.04238412	0.10547286	Camk1d
ENSMUSG00000039148.6	526.827636	-0.0051979	0.0038879	1.7818446	0.18192298	0.33303597	Sart1
ENSMUSG00000039153.18	26.2029598	0.05918368	0.01079796	27.3202585	1.72E-07	1.72E-06	Runx2
ENSMUSG00000039154.9	1.33671514	0.00896716	0.01294851	0.49189342	0.48308391	0.67237207	Shd
ENSMUSG00000039155.16	4.92318763	0.03617175	0.01988646	4.27495151	0.03867807	0.09780861	Cdh26
ENSMUSG00000039156.20	448.189256	-0.0043878	0.0029102	2.29784813	0.12955337	0.25707941	Stim2
ENSMUSG00000039157.13	522.468436	0.00323458	0.00408766	0.61511256	0.43286931	0.62444376	Eeig1
ENSMUSG00000039158.12	88.8684388	0.05348485	0.0050056	105.105255	1.16E-24	1.05E-22	Akna
ENSMUSG00000039159.17	1776.32688	-0.0085705	0.00236077	12.7524379	0.00035555	0.0017703	Ube2h
ENSMUSG00000039163.11	316.910952	0.00396262	0.00380729	1.08938612	0.29660726	0.47985314	Cmc1
ENSMUSG00000039164.3	87.5043929	0.00198943	0.00266031	0.54958383	0.45848779	0.65016709	Naif1
ENSMUSG00000039166.17	168.442904	0.01490485	0.00382972	14.561705	0.00013564	0.00075071	Akap7
ENSMUSG00000039167.13	407.943469	0.00314925	0.00421999	0.52446727	0.46894227	0.66001148	Adgrl4
ENSMUSG00000039168.16	3580.14035	0.00706504	0.00335902	4.41016564	0.03572537	0.09173855	Dap
ENSMUSG00000039176.18	934.637028	-0.0091677	0.00461086	3.91929838	0.0477348	0.11616415	Polg
ENSMUSG00000039178.10	58.355899	0.01591786	0.00489788	10.1963744	0.00140717	0.00592141	Tbc1d19
ENSMUSG00000039179.14	1.16183986	0.07514067	0.01845259	17.8428932	2.40E-05	0.0001574	Tekt5
ENSMUSG00000039182.7	333.771825	-0.0103953	0.00277907	14.1005346	0.00017329	0.00094091	AW209491
ENSMUSG00000039183.7	456.940948	-0.0089971	0.00217325	17.3638889	3.09E-05	0.00019754	Nubp2
ENSMUSG00000039187.17	9.7429161	0.02183286	0.01153967	3.04867483	0.08080305	0.17756645	Fanci
ENSMUSG00000039191.13	417.569202	0.00475081	0.00378434	1.55598827	0.21225358	0.37449912	Rbpj
ENSMUSG00000039193.10	23.2513757	0.06589659	0.00691712	81.5236401	1.73E-19	8.49E-18	Nlrc4
ENSMUSG00000039195.5	242.461579	-0.0097869	0.00392618	6.37210086	0.01159283	0.03606284	Bbln
ENSMUSG00000039196.3	15877.7323	-0.0011368	0.00660523	0.02975496	0.86304739	0.95473814	Orm1
ENSMUSG00000039197.11	11675.0687	0.0046549	0.00250503	3.40886564	0.06484701	0.14894163	Adk
ENSMUSG00000039199.7	43.3663456	0.01016622	0.00405553	6.36820946	0.01161828	0.03612338	Zdhhc1
ENSMUSG00000039201.11	72.6891936	-0.0026025	0.00305037	0.74132445	0.38923694	0.58081185	Tbc1d25
ENSMUSG00000039202.13	2397.16905	0.03890228	0.0045145	70.3601141	4.94E-17	1.79E-15	Abhd2
ENSMUSG00000039205.17	416.976171	0.00337668	0.00347212	0.95083574	0.32950666	0.51639495	Ciz1
ENSMUSG00000039206.14	498.303039	-0.0027598	0.00228025	1.44663768	0.22906823	0.39607091	Daglb
ENSMUSG00000039208.16	70.4782218	0.03020012	0.00507823	33.0963632	8.77E-09	1.07E-07	Metrnl
ENSMUSG00000039209.13	1.6645317	-0.048328	0.01884516	7.92526766	0.00487491	0.01730319	Rpl39l
ENSMUSG00000039210.17	276.411266	-0.011886	0.00309144	14.5748553	0.0001347	0.00074648	Gpatch2
ENSMUSG00000039217.14	211.976147	0.0125778	0.00386161	10.4003685	0.0012599	0.00538529	Il18

ENSMUSG00000039218.18	3890.0612	-0.0051377	0.00348434	2.18814092	0.13907709	0.27138846	Srrm2
ENSMUSG00000039219.19	951.290704	-0.0062278	0.00363213	2.8908591	0.08908341	0.19196354	Arid4b
ENSMUSG00000039220.17	705.485009	-0.0068701	0.0041888	2.69175707	0.10086853	0.21156826	Ppp1r10
ENSMUSG00000039221.11	1604.58422	-0.0073599	0.00504594	2.19871153	0.13812615	0.27005977	Rpl22l1
ENSMUSG00000039224.2	2.38097419	-0.0149301	0.02708553	0.24044005	0.62388847	0.7930495	D1Pas1
ENSMUSG00000039230.15	424.365856	0.00627523	0.00253208	6.18036489	0.01291756	0.03950249	Tbcd
ENSMUSG00000039231.19	113.660037	0.01141339	0.00266926	18.121379	2.07E-05	0.00013825	Suv39h1
ENSMUSG00000039232.13	32.8169698	0.06506172	0.00718834	69.3007638	8.45E-17	2.97E-15	Stx11
ENSMUSG00000039233.13	331.45506	-0.0070245	0.00240086	8.57304211	0.00341177	0.01275054	Tbce
ENSMUSG00000039234.12	1366.40422	0.00336171	0.00368942	0.82280837	0.36436034	0.55411716	Sec24d
ENSMUSG00000039236.19	55.7486766	0.01329855	0.00476301	7.32937796	0.00678365	0.02299831	Isg20
ENSMUSG00000039238.7	209.044947	-0.0008385	0.0040945	0.040957	0.83962095	0.94036567	Zfp750
ENSMUSG00000039239.15	27.2806834	0.00337858	0.00736724	0.20643828	0.64957348	0.81303546	Tgfb2
ENSMUSG00000039242.11	409.280002	-0.0158112	0.00231131	46.7717201	7.98E-12	1.50E-10	B3galnt2
ENSMUSG00000039244.11	259.826654	-0.0070256	0.0021942	10.2617132	0.00135819	0.00574093	Ints15
ENSMUSG00000039246.9	337.881955	-0.001741	0.00303828	0.3257396	0.5681786	0.74786877	Lyplal1
ENSMUSG00000039252.12	16.1308444	0.03743407	0.00726274	26.2658351	2.98E-07	2.83E-06	Lgi2
ENSMUSG00000039253.8	154.666706	-0.004732	0.00227019	4.32581077	0.03753853	0.09545916	Fn3krp
ENSMUSG00000039254.17	255.049643	-0.0068115	0.00259463	6.87854659	0.00872365	0.02843897	Pomt1
ENSMUSG00000039257.12	2.57776324	0.03047228	0.02865191	2.02207602	0.15502707	0.29454393	Vstm2b
ENSMUSG00000039262.17	1144.3841	-0.0039962	0.00414121	0.92250717	0.33681753	0.5241952	Prrc2b
ENSMUSG00000039263.17	908.389473	0.00189236	0.00190756	0.97847423	0.32257578	0.50901669	Npepl1
ENSMUSG00000039264.10	71.4889672	0.07233016	0.00575465	139.991271	2.67E-32	7.51E-30	Gimap3
ENSMUSG00000039270.10	591.408253	0.00549169	0.00331245	2.7645231	0.0963754	0.20426944	Megf9
ENSMUSG00000039275.10	383.010736	-0.001574	0.00250355	0.39767672	0.52829154	0.71299407	Foxk2
ENSMUSG00000039278.11	12.6054988	0.02388827	0.024524	47.9843436	4.30E-12	8.33E-11	Pcsk1n
ENSMUSG00000039285.13	887.928562	0.00201029	0.00171229	1.36885643	0.24200846	0.41210104	Azi2
ENSMUSG00000039286.13	583.876892	0.01329599	0.00433651	8.98031076	0.00272904	0.01053953	Fndc3b
ENSMUSG00000039294.15	203.207736	0.01380254	0.00288946	22.2151237	2.44E-06	1.96E-05	Cybc1
ENSMUSG00000039298.17	126.088261	0.00836528	0.00444842	3.44909491	0.06328642	0.14608274	Cdk5rap2
ENSMUSG00000039304.12	165.590376	0.03493301	0.0036713	88.5546687	4.94E-21	2.86E-19	Tnfsf10
ENSMUSG00000039307.17	127.849809	0.00679491	0.00341801	4.04194223	0.04438271	0.10937318	Hexdc
ENSMUSG00000039308.7	392.826341	-0.0006935	0.00321812	0.04626675	0.82969164	0.93395251	Ndst2
ENSMUSG00000039316.15	61.2026944	0.03633521	0.00541312	42.1636201	8.39E-11	1.36E-09	Rftn1
ENSMUSG00000039318.13	372.420551	0.0020468	0.00224143	0.83650955	0.36039695	0.54972014	Rab3gap2
ENSMUSG00000039323.19	7050.28832	-0.0051909	0.00601574	0.73958423	0.3897941	0.58127044	Igfbp2
ENSMUSG00000039328.12	20.1041584	0.02724745	0.00537948	25.1713113	5.25E-07	4.75E-06	Rnf122
ENSMUSG00000039330.6	1.33239121	-0.0156224	0.06055174	0.14557358	0.70280181	0.85165054	Tsga10ip
ENSMUSG00000039335.12	1.79124238	-0.0264105	0.06839172	0.08239594	0.77407639	0.89942193	Spata16
ENSMUSG00000039345.17	139.522286	-0.0157113	0.00350167	20.3314883	6.51E-06	4.81E-05	Mettl22
ENSMUSG00000039347.8	19.8118719	0.00316034	0.00627154	0.24859996	0.61806262	0.78908871	Atp6v0e2
ENSMUSG00000039349.6	2529.87105	-0.005318	0.00336746	2.50478865	0.11350071	0.2317773	C130074G19Rik
ENSMUSG00000039354.17	202.793766	-0.000211	0.00234089	0.00820372	0.92783078	0.99048799	Smarcal1
ENSMUSG00000039356.16	193.760975	-0.0010513	0.00246087	0.18181969	0.66981407	0.82808652	Exosc2
ENSMUSG00000039357.3	141.75215	0.00069279	0.00293831	0.05528356	0.81411205	0.92395567	Fut11
ENSMUSG00000039361.12	2150.19608	0.0100389	0.00213442	21.6472275	3.28E-06	2.58E-05	Picalm
ENSMUSG00000039364.15	1.84799517	0.02691899	0.01463218	3.75456816	0.0526634	0.12576098	Sectm1b
ENSMUSG00000039367.12	1832.44044	-0.0049168	0.00233697	4.43377138	0.03523457	0.09072517	Sec24c
ENSMUSG00000039376.14	36.3446974	-0.0356533	0.01159436	9.03986971	0.00264154	0.01024897	Synpo2l
ENSMUSG00000039377.8	78.1696842	0.01797481	0.00455623	15.6148205	7.76E-05	0.00045498	Hlx
ENSMUSG00000039382.13	556.721198	-0.0111121	0.00291487	14.6066715	0.00013244	0.00073537	Wdr45
ENSMUSG00000039384.9	88.8147458	0.02226388	0.00336053	41.5898618	1.13E-10	1.79E-09	Dusp10
ENSMUSG00000039385.6	20.2943908	0.02881748	0.00891836	10.5666119	0.00115149	0.00497143	Cdh6
ENSMUSG00000039391.12	2.01466649	-0.0205405	0.03846766	0.19355228	0.65997639	0.82108888	Ccdc81
ENSMUSG00000039395.9	704.365889	-0.0140973	0.00387453	13.0198743	0.0003082	0.00156127	Mreg
ENSMUSG00000039396.12	8.05251382	0.03437192	0.01152016	7.9628091	0.00477483	0.0169914	Neil3
ENSMUSG00000039405.8	139.462954	0.01910178	0.004186	19.8323542	8.45E-06	6.08E-05	Prss23
ENSMUSG00000039410.17	22.7868333	0.00338381	0.00869722	0.13273915	0.71560873	0.86003362	Prdm16
ENSMUSG00000039414.11	242.453581	-0.0061968	0.00321156	3.7707956	0.05215482	0.12475898	Heatr5b
ENSMUSG00000039419.18	9.64376351	0.06230994	0.01675007	14.028113	0.0001801	0.00097359	Cntnap2
ENSMUSG00000039427.15	315.820454	-0.0035528	0.00203986	3.09096514	0.0787281	0.17395113	Alg1

ENSMUSG00000039428.12	1887.54596	0.00080033	0.00248748	0.10188123	0.74958366	0.88314502	Tmem135
ENSMUSG00000039431.17	41.4267119	-0.0017018	0.00406534	0.17325729	0.67723303	0.83315347	Mtmr7
ENSMUSG00000039438.8	7177.62566	-0.0006404	0.00518901	0.01526659	0.9016652	0.97646956	Ttc36
ENSMUSG00000039449.15	758.751378	-0.0059499	0.00304566	3.80230256	0.05118215	0.12289344	Prpf18
ENSMUSG00000039450.12	1274.50988	-0.0025637	0.00361587	0.50049208	0.47928399	0.66925535	Dcxr
ENSMUSG00000039452.7	26.8192605	0.01783351	0.00594282	8.8230916	0.00297442	0.01133906	Snx22
ENSMUSG00000039456.12	523.631286	0.02235588	0.00569261	14.8950325	0.00011366	0.00064196	Morc3
ENSMUSG00000039457.5	317.726027	0.04252707	0.00544986	56.4105418	5.88E-14	1.45E-12	Ppl
ENSMUSG00000039458.16	195.117584	0.01162003	0.00273514	18.0842613	2.11E-05	0.00014071	Mtmr12
ENSMUSG00000039461.13	328.343379	-0.0097092	0.00261928	13.8573766	0.00019722	0.00105336	Tcta
ENSMUSG00000039463.16	604.37055	0.00275134	0.00255982	1.16264415	0.28091784	0.46051346	Slc9a8
ENSMUSG00000039470.17	40.0789533	-0.0057526	0.00736532	0.59134583	0.44189916	0.633802	Zdhhc2
ENSMUSG00000039473.9	717.756431	-0.0024754	0.00253124	0.94618566	0.33069211	0.51758556	Ubn1
ENSMUSG00000039474.14	201.725838	-0.0080511	0.00374272	4.71571867	0.02988807	0.07922591	Wfs1
ENSMUSG00000039477.17	539.275252	-0.0062656	0.00444841	1.98366255	0.1590051	0.30011957	Tnrc18
ENSMUSG00000039478.16	87.7361718	0.00913845	0.003617	6.32691261	0.01189192	0.0368426	Micu3
ENSMUSG00000039480.15	394.016457	-0.0055452	0.00243513	5.12976358	0.02351893	0.06510769	Nt5dc1
ENSMUSG00000039481.6	14.6644142	-0.0172379	0.00745499	5.24818863	0.02196963	0.06150453	Nrtn
ENSMUSG00000039483.11	147.070468	-0.001815	0.00271888	0.43529652	0.5094014	0.69602256	Asb6
ENSMUSG00000039485.9	104.457672	0.00874927	0.0066576	1.56743571	0.2105798	0.37255571	Tspyl4
ENSMUSG00000039488.16	1.78493294	-0.0099322	0.04223413	-0.0137053	1	1	Cntn5
ENSMUSG00000039496.9	19.3552534	0.00262262	0.00885436	0.07901062	0.77864269	0.9026377	Cdnf
ENSMUSG00000039497.9	252.545051	0.02633973	0.00463556	30.4030001	3.51E-08	3.90E-07	Dse
ENSMUSG00000039501.15	861.613492	0.01143804	0.00307887	13.8450405	0.00019852	0.00106006	Znfx1
ENSMUSG00000039509.9	196.702399	0.00128933	0.00291112	0.1952045	0.6586198	0.82008182	Nup133
ENSMUSG00000039512.13	395.770577	-0.0005072	0.00316048	0.02591852	0.87209948	0.95983846	Bltp3a
ENSMUSG00000039515.12	948.366109	-0.0100797	0.00550597	3.34089507	0.06757784	0.15386533	Ptpa
ENSMUSG00000039518.8	6.34250142	0.04439846	0.03445148	-0.1697292	1	1	Cdsn
ENSMUSG00000039519.7	3542.27204	-0.0447767	0.0063726	42.3922257	7.47E-11	1.22E-09	Cyp7b1
ENSMUSG00000039521.14	20.7619301	0.0678678	0.01246811	24.2414521	8.50E-07	7.38E-06	Foxp3
ENSMUSG00000039523.17	191.8636	-0.0092186	0.00254506	13.0688162	0.00030025	0.00152591	Cep104
ENSMUSG00000039529.10	1005.91909	0.00320081	0.00436097	0.53466566	0.4646519	0.65569461	Atp8b1
ENSMUSG00000039531.9	139.734507	0.01244217	0.00272616	20.3522781	6.44E-06	4.76E-05	Zup1
ENSMUSG00000039533.9	180.877437	0.05398999	0.00715413	55.7644999	8.17E-14	1.96E-12	Mmd2
ENSMUSG00000039536.17	1291.47977	-0.0027051	0.00186202	2.09119958	0.1481497	0.28472268	Stau1
ENSMUSG00000039542.17	42.0870356	0.05243316	0.01042875	21.9171321	2.85E-06	2.26E-05	Ncam1
ENSMUSG00000039546.10	4.31733371	0.07763215	0.01202613	36.0753399	1.90E-09	2.53E-08	Ajap1
ENSMUSG00000039552.15	10.516876	0.0494222	0.0094962	26.6555781	2.43E-07	2.36E-06	Rsph4a
ENSMUSG00000039555.17	16.3730461	-0.099678	0.04782796	0.55101752	0.45790223	0.64966808	Cylc2
ENSMUSG00000039556.17	9.97536707	0.02257602	0.00973918	5.01555311	0.0251206	0.06873487	Ppp1r3f
ENSMUSG00000039568.7	262.826726	-0.0037899	0.00330283	1.31393297	0.2516838	0.42355118	Ubald1
ENSMUSG00000039577.18	2.01420049	0.01413593	0.01329547	1.20547577	0.27222999	0.44960553	Nphp4
ENSMUSG00000039578.18	6.29908119	0.06695294	0.01240822	27.3472993	1.70E-07	1.70E-06	Ccser1
ENSMUSG00000039579.16	8.69063039	0.01291123	0.0150354	0.86784459	0.35155271	0.54003661	Grin3a
ENSMUSG00000039585.16	273.09104	0.04555165	0.00608502	50.5923928	1.14E-12	2.36E-11	Myo9a
ENSMUSG00000039599.17	440.895578	-0.0097729	0.00199106	23.6397801	1.16E-06	9.89E-06	Fam149b
ENSMUSG00000039601.17	293.629485	0.01367901	0.00605047	4.67201622	0.03065792	0.08097397	Rcan2
ENSMUSG00000039607.18	209.747912	0.0142898	0.00427383	10.8304168	0.00099846	0.00438869	Rbms3
ENSMUSG00000039611.3	22.4090826	-0.0098133	0.00735553	1.7508462	0.18577039	0.33855512	Pgap4
ENSMUSG00000039615.10	914.616627	-0.004356	0.00280602	2.42846861	0.11914925	0.240934	Stub1
ENSMUSG00000039616.11	951.973035	-0.0060767	0.00308727	3.84707967	0.04983269	0.12024079	Mocos
ENSMUSG00000039620.18	92.3160599	-0.0082283	0.00502477	2.52314524	0.11218662	0.22966552	Trmt9b
ENSMUSG00000039621.14	231.136466	0.06109248	0.00379054	242.14175	1.34E-54	3.52E-51	Prex1
ENSMUSG00000039623.14	198.103305	-0.0030445	0.00324056	0.88341029	0.34726933	0.53552527	Ap5z1
ENSMUSG00000039628.10	17.2626261	-0.0205925	0.00515036	16.1630879	5.81E-05	0.00035129	Hs3st6
ENSMUSG00000039629.15	20.2370171	0.02447114	0.01029907	5.15629398	0.02316227	0.06426365	Strip2
ENSMUSG00000039630.11	4196.1703	-0.0026423	0.00211516	1.54000786	0.21461663	0.37766487	Hnrnpu
ENSMUSG00000039632.14	26.1231632	-0.0060048	0.00468755	1.66256893	0.19725683	0.35431419	Odad3
ENSMUSG00000039633.8	122.20558	0.00089531	0.00440048	0.04088058	0.83976861	0.94036567	Lonrf1
ENSMUSG00000039634.13	125.148602	-0.0019648	0.00248208	0.62833075	0.42796827	0.61934345	Zfp189
ENSMUSG00000039637.16	311.996093	0.027319	0.00365673	56.0715771	6.99E-14	1.70E-12	Coro7

ENSMUSG00000039639.8	1.38123674	0.00077238	0.0358545	0.01703885	0.89614479	0.97353743	Kcne1
ENSMUSG00000039640.8	2273.5359	-0.0143943	0.003998	13.3117456	0.00026375	0.00136211	Mrpl12
ENSMUSG00000039646.6	142.676044	0.00428518	0.00401917	1.16572843	0.28028068	0.45974685	Vasn
ENSMUSG00000039648.15	1807.67051	-0.0310213	0.00396569	60.2559863	8.33E-15	2.32E-13	Kyat1
ENSMUSG00000039652.17	235.058461	0.00025467	0.00373108	0.00449362	0.94655424	1	Cpeb3
ENSMUSG00000039653.10	4943.96419	-0.0092245	0.00449928	4.13636549	0.04197119	0.1046043	Baat
ENSMUSG00000039656.18	484.747962	-0.0113497	0.00206522	29.9526312	4.43E-08	4.83E-07	Rxrb
ENSMUSG00000039660.17	267.256128	-0.0017605	0.00324714	0.2902496	0.59006063	0.76627959	Spout1
ENSMUSG00000039661.15	1.88333425	0.03316155	0.01729826	4.06105651	0.04388304	0.10849322	Dusp26
ENSMUSG00000039662.17	683.499667	-0.0028901	0.00291489	0.99170991	0.31932482	0.50517585	Icmt
ENSMUSG00000039670.3	165.148006	-0.0140981	0.00360087	15.6188258	7.75E-05	0.00045436	Oxld1
ENSMUSG00000039671.19	569.034989	-0.0025072	0.00386798	0.41344207	0.52022759	0.70610747	Zmynd8
ENSMUSG00000039676.5	3.25859078	0.00458833	0.01017027	0.20250182	0.65270898	0.81453403	Capsl
ENSMUSG00000039678.13	481.96292	-0.001688	0.00299141	0.31746795	0.5731333	0.75220756	Tbc1d13
ENSMUSG00000039680.11	196.412696	-0.0103895	0.00324501	10.2633116	0.00135702	0.00573904	Mrps6
ENSMUSG00000039682.13	5545.10733	-0.0074363	0.00304281	5.85596145	0.01552437	0.04616413	Lap3
ENSMUSG00000039683.17	5.25311402	-0.0118862	0.01726191	0.51640481	0.47237933	0.66306843	Sdk1
ENSMUSG00000039686.15	313.291024	0.00048249	0.00314524	0.02335369	0.8785409	0.96310786	Zer1
ENSMUSG00000039691.11	0.87829856	0.03540253	0.01561266	4.71530262	0.0298953	0.07922591	Tspan10
ENSMUSG00000039693.12	15.5396209	0.03333936	0.00966595	11.1899626	0.00082241	0.00369544	Msantd3
ENSMUSG00000039697.17	91.6265257	0.03684163	0.00538137	43.8250665	3.59E-11	6.11E-10	Ncoa7
ENSMUSG00000039699.15	12.7578635	0.05164079	0.00663191	59.0279658	1.55E-14	4.17E-13	Batf2
ENSMUSG00000039701.12	190.067114	-0.0019263	0.00332423	0.3282636	0.56668334	0.74657172	Usp53
ENSMUSG00000039703.17	1483.54742	-0.0056749	0.00213587	6.97591961	0.00826138	0.02713072	Nploc4
ENSMUSG00000039704.9	1960.12543	-0.0107278	0.00490756	4.59904069	0.03198985	0.08387217	Lmbrd2
ENSMUSG00000039706.12	75.4299246	0.01431591	0.00402638	12.2402411	0.0004677	0.00225355	Ldb2
ENSMUSG00000039713.18	107.22255	0.01315826	0.00481225	7.45752093	0.00631719	0.02159764	Plekhg5
ENSMUSG00000039715.9	89.6474159	-0.0063885	0.00338589	3.57707415	0.05858225	0.13722523	Dync2i2
ENSMUSG00000039716.13	15.2127221	0.00748317	0.01605251	0.21529026	0.64265218	0.80862066	Dock3
ENSMUSG00000039725.7	144.088415	0.00103278	0.00229647	0.20279096	0.65247744	0.81445081	Trp53rka
ENSMUSG00000039735.17	423.243337	0.00241443	0.00304968	0.62072975	0.43077623	0.62229976	Fnbp1l
ENSMUSG00000039737.12	180.401456	-0.0068795	0.00488112	2.00587697	0.15669066	0.29707722	Prkrip1
ENSMUSG00000039738.17	78.7012652	0.01673162	0.00432049	14.7473025	0.00012292	0.0006885	Slx4
ENSMUSG00000039740.7	309.995207	0.00558564	0.00329649	2.93036965	0.08692759	0.18830585	Alg2
ENSMUSG00000039741.17	171.051378	-0.0068493	0.00513661	1.70809306	0.19123297	0.34597077	Bahcc1
ENSMUSG00000039742.16	2.88704512	0.01818607	0.1155731	-0.0470262	1	1	Garin1b
ENSMUSG00000039745.9	674.563077	0.0100003	0.00332231	8.94199291	0.00278688	0.01073167	Htatip2
ENSMUSG00000039747.12	34.9507322	0.05897169	0.00650833	76.861802	1.83E-18	7.83E-17	Orai2
ENSMUSG00000039748.12	11.0583483	0.03995197	0.01439935	6.86844019	0.00877312	0.0285759	Exo1
ENSMUSG00000039753.17	725.427345	0.01223288	0.00269275	20.4069998	6.26E-06	4.64E-05	Fbxl5
ENSMUSG00000039754.11	63.3214217	-0.001701	0.00261938	0.42287753	0.51550469	0.70208417	Alkbh4
ENSMUSG00000039756.13	474.908903	-0.0042332	0.00317211	1.7576291	0.18492052	0.33737123	Dnttip2
ENSMUSG00000039759.11	111.4012	0.00130393	0.003587	0.13087327	0.71752832	0.86147228	Thap3
ENSMUSG00000039763.11	242.138495	-0.0143522	0.00335758	17.9437692	2.28E-05	0.0001502	Dnajc28
ENSMUSG00000039765.16	79.6571439	0.01694398	0.00429283	15.9133614	6.63E-05	0.00039564	Cc2d2a
ENSMUSG00000039768.17	905.613167	-0.0121957	0.00264713	21.4120081	3.70E-06	2.88E-05	Dnajc11
ENSMUSG00000039770.7	908.53762	-0.0027788	0.00227656	1.49667981	0.22118293	0.38628809	Ypel5
ENSMUSG00000039771.14	351.685389	-0.0077355	0.0029027	7.11330805	0.00765138	0.02542483	Polr2j
ENSMUSG00000039774.13	5.082652	0.05384181	0.00825934	40.080683	2.44E-10	3.70E-09	Galnt12
ENSMUSG00000039781.16	82.876027	-0.001617	0.00549654	0.08720608	0.76775956	0.89539047	Cep131
ENSMUSG00000039782.16	795.131038	-0.0117041	0.00450912	6.55984064	0.01043057	0.03304252	Cpeb2
ENSMUSG00000039783.16	4899.68196	-0.014791	0.00415154	12.2487565	0.00046557	0.00224466	Kmo
ENSMUSG00000039787.14	11.0745702	0.01880452	0.00682571	7.34467388	0.00672617	0.02284284	Cercam
ENSMUSG00000039789.8	171.68098	-0.0027218	0.00292714	0.84883629	0.35688175	0.54566401	Zfp597
ENSMUSG00000039795.15	370.902483	0.00343474	0.00238767	2.09342326	0.14793426	0.28443609	Zfand1
ENSMUSG00000039801.8	160.576153	0.04351892	0.00536585	57.6651627	3.11E-14	8.00E-13	Cplane1
ENSMUSG00000039804.16	253.620274	-0.0054834	0.00283838	3.61353984	0.05731101	0.134885	Ncoa5
ENSMUSG00000039809.11	568.11673	0.00238296	0.00580412	0.16857022	0.68138563	0.83651684	Gabbr2
ENSMUSG00000039810.12	127.544431	-0.004806	0.00267871	3.20445352	0.07343804	0.1646179	Zc3h10
ENSMUSG00000039813.15	21.3409687	0.0099284	0.00434338	5.16154828	0.0230923	0.06410349	Tbc1d2
ENSMUSG00000039814.16	1.19954614	0.02977285	0.01442837	4.45623655	0.03477403	0.08984031	Xkr5

ENSMUSG00000039824.5	14.430149	-0.0029685	0.00541399	0.29487697	0.58711207	0.76383834	Myl6b
ENSMUSG00000039826.14	256.366882	-0.0027825	0.00351489	0.61900798	0.43141617	0.62288086	Trub2
ENSMUSG00000039828.8	213.846879	-0.0087434	0.00277232	9.85108763	0.00169731	0.00698775	Wdr70
ENSMUSG00000039830.10	8.51367471	0.00917343	0.0358586	0.10301587	0.74824005	0.88197206	Olig2
ENSMUSG00000039831.17	1389.74855	-0.00899	0.00276954	10.3557325	0.00129073	0.00549616	Arhgap29
ENSMUSG00000039834.18	260.024565	-0.0036294	0.00246952	2.13760778	0.14372586	0.2784327	Zfp335
ENSMUSG00000039835.17	203.191334	-0.0130643	0.00339267	14.5672814	0.00013524	0.00074879	Nhsl1
ENSMUSG00000039838.12	2.0997673	0.00044666	0.02117405	-0.0031339	1	1	Slc45a1
ENSMUSG00000039840.9	490.774054	0.00443324	0.00327836	1.78005627	0.18214242	0.33335839	Epg5
ENSMUSG00000039841.15	192.823373	-0.0022186	0.00376581	0.3326279	0.56411576	0.74464039	Zfp800
ENSMUSG00000039842.16	63.2165764	0.00284535	0.00354854	0.65524735	0.41824284	0.60972097	Mcph1
ENSMUSG00000039844.21	839.916333	-0.0010624	0.00264655	0.16042317	0.68876721	0.8420289	Rapgef1
ENSMUSG00000039849.7	449.578454	0.00204473	0.00329916	0.38703488	0.53386213	0.71816986	Pcif1
ENSMUSG00000039850.17	119.738516	-2.49E-05	0.00293763	7.33E-05	0.9931688	1	Endov
ENSMUSG00000039852.18	645.46459	0.00045672	0.00396308	0.01335456	0.90799973	0.98000648	Rere
ENSMUSG00000039853.19	227.047901	0.00534691	0.00547576	0.94890576	0.32999798	0.51678212	Trim14
ENSMUSG00000039860.20	11.4459705	0.02420302	0.02090754	2.13006356	0.14443477	0.27950758	Srrm3
ENSMUSG00000039865.9	162.87731	0.00224158	0.00453627	0.24198902	0.62277321	0.79240346	Slc44a3
ENSMUSG00000039873.5	33.7124134	-0.0024149	0.00485824	0.24068731	0.62371015	0.79292466	Neurl2
ENSMUSG00000039878.14	18.1212978	-0.0549146	0.00827798	42.1101528	8.63E-11	1.40E-09	Slc39a5
ENSMUSG00000039879.10	729.174779	-0.005014	0.00260684	3.58334814	0.05836141	0.13683025	Heca
ENSMUSG00000039883.6	3.95530705	0.01447731	0.01041967	1.82112124	0.17717987	0.32643265	Lrrc17
ENSMUSG00000039886.9	1078.02324	-0.0033315	0.00472574	0.48642298	0.48552729	0.67430057	Tmem120a
ENSMUSG00000039887.12	271.178003	-0.0096971	0.00242678	15.9718794	6.43E-05	0.00038496	Alg14
ENSMUSG00000039891.7	189.125447	0.02825738	0.012925	1.78189902	0.18191631	0.33303597	Txlnb
ENSMUSG00000039899.6	173.711519	0.06243748	0.00660784	76.0926849	2.71E-18	1.14E-16	Fgl2
ENSMUSG00000039901.11	164.836466	0.00271323	0.00196252	1.89886445	0.16820548	0.31352097	Armh3
ENSMUSG00000039903.19	7.61416876	-0.0105762	0.00663835	2.49983457	0.11385826	0.23236661	Eva1c
ENSMUSG00000039904.10	6.73533646	0.01806992	0.02542741	0.6378974	0.42447321	0.61606209	Gpr37
ENSMUSG00000039908.15	117.668729	-0.0052985	0.00238993	4.94090447	0.02622831	0.07124506	Slc26a11
ENSMUSG00000039910.11	459.837324	-0.0060234	0.00343346	3.05151911	0.08066166	0.17730531	Cited2
ENSMUSG00000039911.14	41.6879775	0.01756177	0.0055786	9.01161423	0.00268269	0.01039494	Spsb1
ENSMUSG00000039913.13	4.84646416	0.02190061	0.01708204	2.04191229	0.15301718	0.29175935	Pak5
ENSMUSG00000039914.7	361.623245	-0.0132886	0.00333186	16.0833188	6.06E-05	0.00036462	Coq10a
ENSMUSG00000039917.11	311.525852	0.00160703	0.00310748	0.27255336	0.60162408	0.7752629	Rhbdd2
ENSMUSG00000039929.15	87.1357927	0.00015731	0.00411975	0.00143212	0.96981258	1	Urb1
ENSMUSG00000039934.13	734.494547	0.00016768	0.00312341	0.00289956	0.95705663	1	Gsap
ENSMUSG00000039936.19	130.133174	0.0663105	0.00576489	124.136333	7.86E-29	1.27E-26	Pik3cd
ENSMUSG00000039942.16	21.3313328	0.07285489	0.00758253	79.9111217	3.92E-19	1.81E-17	Ptger4
ENSMUSG00000039943.17	87.0581724	0.00275393	0.0045661	0.35346113	0.55216038	0.73444928	Plcb4
ENSMUSG00000039952.16	1769.34243	0.00200226	0.00339303	0.34972053	0.55427137	0.73646807	Dag1
ENSMUSG00000039953.14	152.543437	0.02862312	0.00498182	31.9528056	1.58E-08	1.86E-07	Clstn1
ENSMUSG00000039954.10	1.77934994	0.00070084	0.01590287	0.0066979	0.93477338	0.99434035	Stk32a
ENSMUSG00000039956.10	883.323317	-0.0108342	0.00341525	9.93512919	0.00162154	0.00671094	Mrap
ENSMUSG00000039958.18	1078.15649	-0.0242181	0.00404737	34.0973482	5.24E-09	6.60E-08	Etfbkmt
ENSMUSG00000039959.14	189.359803	0.02865835	0.00460797	37.1871069	1.07E-09	1.48E-08	Hip1
ENSMUSG00000039960.6	1394.44472	0.00386062	0.00347542	1.18421004	0.27650058	0.45509719	Rhou
ENSMUSG00000039963.21	5.12390145	0.00156902	0.01131153	0.00718018	0.93247134	0.99301377	Ccdc40
ENSMUSG00000039967.15	438.925266	0.00406072	0.00339488	1.4078459	0.23541432	0.40399316	Zfp292
ENSMUSG00000039968.10	410.628344	0.00818678	0.00342998	5.47508656	0.01928941	0.05522028	Rsbn1l
ENSMUSG00000039976.5	403.263098	-0.0072959	0.00469432	2.40545764	0.12091276	0.24370606	Tbc1d16
ENSMUSG00000039977.17	13.8950808	-0.0051666	0.00909172	0.30461609	0.58100291	0.75882024	Deup1
ENSMUSG00000039981.7	79.570441	0.03711791	0.00487921	54.7106389	1.40E-13	3.27E-12	Zc3h12d
ENSMUSG00000039982.9	288.888017	-0.0009187	0.00429594	0.04510731	0.83180682	0.93526223	Dtx4
ENSMUSG00000039983.14	144.201078	0.00225821	0.00259065	0.76740297	0.38102167	0.57247911	Ccdc32
ENSMUSG00000039985.15	72.8075566	0.00686975	0.00513671	1.73850667	0.18732815	0.34065704	Sinhcaf
ENSMUSG00000039987.16	191.196023	-0.0089782	0.00375537	5.64519328	0.01750329	0.05099846	Phtf2
ENSMUSG00000039988.16	654.312532	0.00504311	0.0027928	3.22415366	0.07255929	0.16295787	Ankrd13c
ENSMUSG00000039989.8	328.313886	-0.0014884	0.00356528	0.17413865	0.67645955	0.83279147	Cbx4
ENSMUSG00000039990.17	359.907152	-0.0031168	0.00262358	1.42706698	0.23224371	0.40006573	Edrf1
ENSMUSG00000039994.16	65.9831552	0.01172411	0.00557604	4.2822488	0.03851236	0.09745227	Timeless

ENSMUSG00000039997.17	370.002033	0.02237316	0.00480436	20.8986258	4.84E-06	3.67E-05	Ifi203
ENSMUSG00000040003.19	26.2352349	0.02806049	0.00857	9.67229871	0.00187067	0.00762164	Magi2
ENSMUSG00000040006.14	760.031899	-0.0099839	0.00253771	15.4246465	8.59E-05	0.00049806	Ginm1
ENSMUSG00000040007.9	232.352778	0.00960901	0.00246054	15.033193	0.00010564	0.00060081	Bahd1
ENSMUSG00000040009.7	7.75984146	0.05733173	0.01343234	12.1615371	0.00048785	0.00234252	Gnaz
ENSMUSG00000040010.11	77.5832167	0.02625516	0.00653788	15.8063831	7.02E-05	0.00041628	Slc7a5
ENSMUSG00000040016.17	7.1926697	-0.0157778	0.01054069	2.20322864	0.13772201	0.26939056	Ptger3
ENSMUSG00000040017.9	4840.02496	-0.013703	0.0064539	4.33861399	0.03725724	0.09483588	Saa4
ENSMUSG00000040018.10	504.264376	-0.0037778	0.00290194	1.72801901	0.18866409	0.34261096	Cox15
ENSMUSG00000040021.15	632.048113	-0.0008307	0.00249006	0.11067165	0.73938077	0.87628937	Lats1
ENSMUSG00000040022.15	189.716346	0.01460378	0.00347129	17.3994018	3.03E-05	0.00019426	Rab11fip2
ENSMUSG00000040025.18	564.690833	-0.0053384	0.00251197	4.39829713	0.03597483	0.09228872	Ythdf2
ENSMUSG00000040026.9	374.013833	-0.0007368	0.01103132	0.00293602	0.95678769	1	Saa3
ENSMUSG00000040028.11	637.072553	-0.0038849	0.00208053	3.45636533	0.06300868	0.14554134	Elavl1
ENSMUSG00000040029.16	947.997159	-0.0092049	0.00257562	12.5070435	0.00040542	0.00198675	Ipo8
ENSMUSG00000040033.18	1190.19041	-0.0022715	0.00441558	0.26731678	0.60513741	0.77801967	Stat2
ENSMUSG00000040034.16	61.6647359	-0.0046313	0.00289691	2.56329861	0.10937017	0.22529299	Nup43
ENSMUSG00000040035.15	68.6021821	0.00860975	0.00577148	2.15164395	0.14241733	0.27639719	Disp2
ENSMUSG00000040037.14	15.2143021	0.06709274	0.0132974	23.1193624	1.52E-06	1.28E-05	Negr1
ENSMUSG00000040040.17	100.983357	-0.0011211	0.00305878	0.13577763	0.71251524	0.85824955	Ift88
ENSMUSG00000040043.18	219.255251	-0.0012294	0.00340547	0.12970157	0.71874166	0.86231451	Rbms2
ENSMUSG00000040044.12	373.249685	0.00143077	0.00191012	0.55732295	0.45534088	0.64708502	Orc3
ENSMUSG00000040048.15	2247.58735	-0.0177876	0.0039751	20.3874892	6.32E-06	4.68E-05	Ndufb10
ENSMUSG00000040054.18	839.004718	-0.0019368	0.00396386	0.23499791	0.62784246	0.79648732	Baz2a
ENSMUSG00000040055.10	7.23011933	0.030581	0.02136454	2.41003497	0.12055967	0.24311905	Gjb6
ENSMUSG00000040061.18	43.3695126	0.04080914	0.00559721	49.7390554	1.76E-12	3.56E-11	Plcb2
ENSMUSG00000040084.10	29.349809	0.03028295	0.00817034	13.4477492	0.0002453	0.00127719	Bub1b
ENSMUSG00000040086.14	32.1473773	-0.0086303	0.05527458	0.15240546	0.69624713	0.8470899	Tnni3k
ENSMUSG00000040093.17	163.198626	0.00190017	0.00581789	0.1076019	0.74289103	0.87846324	Bmf
ENSMUSG00000040097.17	336.871586	-0.002027	0.00335355	0.36350912	0.54656379	0.72963853	Flywch1
ENSMUSG00000040102.11	264.135651	0.0019491	0.00313363	0.37678835	0.53932709	0.72318797	Klhl42
ENSMUSG00000040105.4	254.863583	0.00286971	0.00200588	2.03344587	0.15387139	0.29308008	Plpp6
ENSMUSG00000040111.18	90.4172368	0.04777178	0.00662999	48.2255883	3.80E-12	7.43E-11	Gramd1b
ENSMUSG00000040112.10	627.720473	-0.014869	0.00383753	15.4222506	8.60E-05	0.00049857	Mrps35
ENSMUSG00000040113.15	0.91960519	-0.0132361	0.06985101	-0.0021124	1	1	Ntmt2
ENSMUSG00000040118.17	78.8398551	0.0220011	0.0058329	13.2730576	0.00026925	0.00138748	Cacna2d1
ENSMUSG00000040121.6	7.27664483	-0.0060883	0.00839837	0.49958859	0.47968095	0.66945759	Rep15
ENSMUSG00000040123.18	681.972503	-0.005456	0.002684	4.08024826	0.04338728	0.10754312	Zmym5
ENSMUSG00000040124.6	92.1799305	0.00543193	0.00277714	3.7880391	0.05162009	0.12376857	Gorab
ENSMUSG00000040125.5	6.9170201	0.01980664	0.01969862	1.33501041	0.24791581	0.41919438	Gpr26
ENSMUSG00000040127.14	1099.34338	-0.0004482	0.00571854	0.00578617	0.93936589	0.99644182	Sdr9c7
ENSMUSG00000040128.10	1200.34047	0.00143275	0.00415187	0.1125509	0.73725845	0.87469707	Pnrc1
ENSMUSG00000040133.3	4.04752363	0.08127902	0.0153387	24.4417688	7.66E-07	6.70E-06	Gpr176
ENSMUSG00000040134.9	18978.2555	-0.0124296	0.00512512	5.89438151	0.01518926	0.04532565	Rdh7
ENSMUSG00000040136.11	39.4685787	-0.0021031	0.02488145	0.00829568	0.92742852	0.99042726	Abcc8
ENSMUSG00000040139.15	177.269922	0.0078912	0.00418343	3.51980799	0.06063947	0.14120274	9430038I01Rik
ENSMUSG00000040140.16	3.91692151	0.00656328	0.02989983	0.11350326	0.73619044	0.87395751	Tdrd6
ENSMUSG00000040146.10	380.604418	-0.0119621	0.00348689	11.9224512	0.00055461	0.0026199	Rgl3
ENSMUSG00000040147.15	3325.4646	0.00207262	0.00280849	0.54744034	0.45936546	0.65092482	Maob
ENSMUSG00000040151.10	258.285692	0.00970523	0.00246554	15.368298	8.85E-05	0.00051113	Hs2st1
ENSMUSG00000040152.9	83.813525	0.03998697	0.00779924	23.8187847	1.06E-06	9.06E-06	Thbs1
ENSMUSG00000040158.13	283.312384	0.01267472	0.00270589	21.8027481	3.02E-06	2.39E-05	Tax1bp3
ENSMUSG00000040163.15	2.30715524	-0.008366	0.02551081	0.05019742	0.82272011	0.92947629	1700034J05Rik
ENSMUSG00000040167.17	232.989931	-0.0043457	0.00403861	1.1459791	0.28439245	0.46442431	Ikzf5
ENSMUSG00000040170.14	473.337107	0.03241798	0.00621965	26.6882991	2.39E-07	2.32E-06	Fmo2
ENSMUSG00000040174.15	275.767423	-0.0037077	0.00251998	2.20995126	0.137123	0.26853065	Alkbh3
ENSMUSG00000040177.11	76.7023653	-0.016218	0.00578884	7.7365848	0.0054113	0.01891951	2310057M21Rik
ENSMUSG00000040181.15	3420.79594	0.00166784	0.00325128	0.2668885	0.60542667	0.77817948	Fmo1
ENSMUSG00000040183.14	11.2692538	0.0261784	0.00660393	15.3872068	8.76E-05	0.00050666	Ankrd6
ENSMUSG00000040187.16	22.8329289	-0.0083368	0.00821284	0.95281201	0.32900456	0.51591659	Bmal2
ENSMUSG00000040188.11	361.445574	0.00972088	0.00372231	6.68464324	0.0097247	0.03116819	Scamp2

ENSMUSG00000040189.19	7.80647298	0.00954793	0.00839632	1.25018097	0.26351791	0.43844057	Odad1
ENSMUSG00000040195.12	164.673106	-0.0112375	0.00406704	7.54928343	0.00600339	0.02066874	Nemp1
ENSMUSG00000040204.7	29.7728302	0.05829456	0.01047848	26.5492744	2.57E-07	2.48E-06	Pclaf
ENSMUSG00000040209.13	218.946752	0.02042023	0.00398468	26.1012106	3.24E-07	3.06E-06	Zfp704
ENSMUSG00000040212.13	60.6360788	0.04506143	0.00577599	56.5884089	5.37E-14	1.33E-12	Emp3
ENSMUSG00000040213.14	3503.33459	-0.0080435	0.00353961	5.07232137	0.02431081	0.06686818	Kyat3
ENSMUSG00000040219.5	15.1133512	0.01741265	0.00708046	5.8764085	0.01534509	0.04574435	Ttc12
ENSMUSG00000040220.11	53.2391588	0.00974335	0.00377237	6.53125664	0.0105995	0.03351655	Gas8
ENSMUSG00000040225.16	1767.72162	0.00296804	0.00355862	0.68362791	0.40834026	0.5999946	Prrc2c
ENSMUSG00000040229.12	6.58366119	0.04036815	0.00924591	17.9259595	2.30E-05	0.00015144	Gpr34
ENSMUSG00000040231.17	0.89920204	-0.0226838	0.01944427	1.46454578	0.22620822	0.39239149	Syngr4
ENSMUSG00000040234.17	1376.38535	0.0075194	0.00201045	13.8627394	0.00019666	0.00105108	Tm7sf3
ENSMUSG00000040236.5	408.370374	-0.0046873	0.00225278	4.3213484	0.03763709	0.09567885	Trappc5
ENSMUSG00000040242.15	1089.19394	0.00043929	0.00192165	0.052152	0.81936007	0.92754627	Fgfr1op2
ENSMUSG00000040247.7	26.8540338	0.06644345	0.00723429	73.1096508	1.23E-17	4.78E-16	Tbc1d10c
ENSMUSG00000040249.16	11434.4599	0.00054775	0.00322799	0.02855153	0.86581869	0.9563173	Lrp1
ENSMUSG00000040250.15	346.81654	-0.0009868	0.00226004	0.19248696	0.66085475	0.82159984	Ints13
ENSMUSG00000040253.16	493.873593	0.04164506	0.00464339	78.5929848	7.63E-19	3.44E-17	Gbp7
ENSMUSG00000040254.12	62.7046872	0.01815917	0.00406411	18.7936427	1.46E-05	0.00010019	Sema3d
ENSMUSG00000040260.9	74.8937606	0.01032979	0.00408328	6.28008978	0.01221019	0.03767525	Daam2
ENSMUSG00000040263.17	235.098411	-0.010472	0.00276	14.4378079	0.00014486	0.00079775	Klhdc4
ENSMUSG00000040264.11	85.37252	0.03969001	0.00740729	27.5593978	1.52E-07	1.54E-06	Gbp2b
ENSMUSG00000040265.17	20.6232384	0.0222032	0.00709844	9.16512808	0.0024667	0.00965477	Dnm3
ENSMUSG00000040268.18	330.117544	0.00933927	0.00462961	3.81368095	0.05083562	0.12220383	Plekha1
ENSMUSG00000040269.6	368.993469	-0.0164016	0.00347243	22.9645805	1.65E-06	1.37E-05	Mrps28
ENSMUSG00000040270.17	184.494882	-0.0300883	0.0061796	22.8649882	1.74E-06	1.44E-05	Bach2
ENSMUSG00000040272.15	60.6294202	0.0154581	0.00471196	10.5616427	0.00115459	0.00498208	Accs
ENSMUSG00000040274.12	564.502594	0.00664256	0.00429837	2.38914649	0.12218032	0.24565212	Cdk6
ENSMUSG00000040276.16	13.2014345	0.04090403	0.01010359	14.1981266	0.00016453	0.00089582	Pacsin1
ENSMUSG00000040280.11	26.0292488	-0.0043489	0.00534264	0.66470207	0.41490483	0.60624119	Ndufa4l2
ENSMUSG00000040282.14	70.4713938	0.01103044	0.0031882	11.9678045	0.00054128	0.00256717	Cdin1
ENSMUSG00000040283.15	137.423392	0.01869676	0.0057877	9.67434353	0.00186859	0.00761447	Btnl9
ENSMUSG00000040287.10	7.00951047	-0.0098338	0.00817786	1.44539744	0.22926791	0.39631918	Stac3
ENSMUSG00000040289.9	34.82226	0.04655161	0.00677449	44.0158967	3.26E-11	5.58E-10	Hey1
ENSMUSG00000040296.16	572.24921	0.01247267	0.00339076	13.6451318	0.00022081	0.00116512	Rigi
ENSMUSG00000040297.13	424.947892	0.01145916	0.0049317	5.16313147	0.02307126	0.06406391	Suco
ENSMUSG00000040302.18	121.58637	-0.0053638	0.00336942	2.50408019	0.11355177	0.23184141	Rbm48
ENSMUSG00000040312.15	48.8943739	0.00797505	0.00490706	2.6485271	0.10364589	0.21583234	Cchcr1
ENSMUSG00000040314.3	2.92767851	0.06936825	0.0339754	24.6980311	6.71E-07	5.95E-06	Ctsg
ENSMUSG00000040321.4	167.210004	0.00374565	0.00267672	1.91272464	0.16666092	0.31135559	Zfp770
ENSMUSG00000040322.11	53.7716872	0.03103789	0.00544733	30.8593115	2.77E-08	3.13E-07	Slc25a24
ENSMUSG00000040325.17	641.183877	-0.0044776	0.00388784	1.34391094	0.24634544	0.41754238	Dcaf1
ENSMUSG00000040327.17	146.108089	0.00211279	0.00469367	0.20919731	0.64739727	0.81156057	Cul9
ENSMUSG00000040328.18	5.68886651	0.04162561	0.01158728	11.8212955	0.00058557	0.00274578	Or2v1
ENSMUSG00000040329.15	12.0800313	0.07125863	0.00742734	85.6236769	2.18E-20	1.19E-18	Il7
ENSMUSG00000040331.17	644.895963	-0.0027996	0.00248295	1.26396478	0.26090194	0.43508631	Nsmce4a
ENSMUSG00000040339.11	75.4612451	0.03023357	0.0047712	38.3133226	6.02E-10	8.62E-09	Eeig2
ENSMUSG00000040345.11	62.8915623	0.04435159	0.00583204	54.7750244	1.35E-13	3.17E-12	Arhgap9
ENSMUSG00000040350.17	236.295272	-0.0082688	0.00436005	3.55375427	0.05941089	0.13878035	Trim7
ENSMUSG00000040351.12	469.263232	0.00277484	0.0028413	0.94704012	0.33047385	0.5173607	Ankib1
ENSMUSG00000040354.15	1073.86077	-0.0087109	0.00308882	7.96675444	0.00476443	0.01696465	Mars1
ENSMUSG00000040356.17	1042.41178	-0.0081453	0.00284116	8.18767432	0.0042176	0.01529806	Skic2
ENSMUSG00000040359.15	810.853599	0.00165322	0.00351689	0.2206631	0.63853513	0.80521275	Ufl1
ENSMUSG00000040363.16	265.329411	0.00345945	0.00273777	1.57866107	0.20895366	0.37049925	Bcor
ENSMUSG00000040365.15	442.438954	-0.0044831	0.00341144	1.66907717	0.19638219	0.35313338	Trim41
ENSMUSG00000040370.14	1078.75948	-0.013581	0.00288528	21.9377407	2.82E-06	2.24E-05	Etfrf1
ENSMUSG00000040372.3	3.43639856	0.03451829	0.01165291	8.17697445	0.00424255	0.01538037	Gpr63
ENSMUSG00000040374.14	943.452642	-0.0022072	0.00237964	0.86470195	0.35242622	0.54099088	Pex2
ENSMUSG00000040380.16	17.1025135	0.02411196	0.0052301	19.971425	7.86E-06	5.69E-05	Cbln3
ENSMUSG00000040383.17	417.614675	-0.0005272	0.00259323	0.04111946	0.8393075	0.94011049	Aqr
ENSMUSG00000040385.8	2627.70764	-0.0016308	0.00233866	0.49122507	0.48338134	0.67262727	Ppp1ca

ENSMUSG00000040387.17	5.03186473	0.05055911	0.01003532	23.4635032	1.27E-06	1.08E-05	Klhl32
ENSMUSG00000040389.16	48.8996813	0.01367814	0.00486228	7.64176522	0.00570321	0.01980213	Wdr47
ENSMUSG00000040390.14	234.367534	-0.0071543	0.00350877	4.23519438	0.03959409	0.09977945	Map3k10
ENSMUSG00000040396.13	426.06904	-0.0079505	0.00214086	13.6577998	0.00021933	0.00115781	Abhd13
ENSMUSG00000040405.14	8.10318025	0.02863265	0.01245789	5.03538944	0.0248345	0.06808335	Havcr1
ENSMUSG00000040407.19	1188.26136	-0.0009198	0.00417564	0.0478168	0.8269067	0.93204288	Akap9
ENSMUSG00000040410.15	363.150925	0.00727354	0.00266293	7.55128198	0.00599674	0.02065106	Fbxl4
ENSMUSG00000040412.18	31.5801408	0.03757528	0.00676646	31.414265	2.08E-08	2.40E-07	Elapor1
ENSMUSG00000040413.16	1434.16134	-0.0042204	0.00376146	1.27004881	0.25975752	0.43365239	Timd2
ENSMUSG00000040414.9	301.595013	-0.0055591	0.00277151	4.04991543	0.04417356	0.10898301	Slc25a28
ENSMUSG00000040415.16	85.1426121	0.01421257	0.00562421	6.32583668	0.01189913	0.03686012	Dtx3
ENSMUSG00000040420.16	17.6512945	0.07730425	0.00728068	97.8544602	4.50E-23	3.31E-21	Cdh18
ENSMUSG00000040423.12	693.256866	0.00106112	0.0032056	0.10725817	0.74328753	0.87866779	Rc3h1
ENSMUSG00000040428.20	17.9140052	0.0231984	0.00694433	10.74528	0.00104546	0.00456717	Plekha4
ENSMUSG00000040429.7	55.447414	-0.0047876	0.00413168	1.32220681	0.25019638	0.42187102	Mterf1a
ENSMUSG00000040430.19	757.253398	0.0113413	0.00396135	8.12176714	0.00437369	0.01579914	Pitpnc1
ENSMUSG00000040432.6	24.4842168	-0.020776	0.005023	16.9430086	3.85E-05	0.00024259	Ltb4r2
ENSMUSG00000040433.17	578.530328	0.00668829	0.00350076	3.55731857	0.05928343	0.1385375	Zbtb38
ENSMUSG00000040434.17	13.2293668	0.00873607	0.00607128	2.17913931	0.13989268	0.27273174	Large2
ENSMUSG00000040435.13	286.529771	-0.0052212	0.00571573	0.83296365	0.36141696	0.55102653	Ppp1r15a
ENSMUSG00000040441.13	75.0169838	-0.0179825	0.00543921	11.2725387	0.00078662	0.00356245	Slc26a10
ENSMUSG00000040446.5	440.889333	-0.0113215	0.00234709	22.2991477	2.33E-06	1.88E-05	Rprd1a
ENSMUSG00000040447.16	460.525591	0.0091855	0.00373129	5.93269836	0.01486247	0.04455264	Spns2
ENSMUSG00000040451.19	304.363544	0.00758662	0.00321323	5.51054073	0.0189022	0.0543162	Sgms1
ENSMUSG00000040452.10	1.41194645	0.0249228	0.02672309	1.55088459	0.2130049	0.37553217	Cdh12
ENSMUSG00000040455.18	351.8929	-0.0056178	0.00312167	3.22518622	0.07251355	0.16288615	Usp45
ENSMUSG00000040459.12	1875.89776	0.00209886	0.00301813	0.47775024	0.48944314	0.67737107	Arglu1
ENSMUSG00000040462.14	3571.77846	-0.0028872	0.00243829	1.40188173	0.23640876	0.40537528	Os9
ENSMUSG00000040463.17	910.631205	-0.0022671	0.003259	0.4879177	0.48485764	0.67393082	Mybbp1a
ENSMUSG00000040464.16	404.411995	-0.0060186	0.00217609	7.61223588	0.00579736	0.02006993	Gtpbp10
ENSMUSG00000040466.17	2109.62643	0.00195044	0.00362529	0.28124409	0.59588695	0.77087481	Blvrb
ENSMUSG00000040471.18	304.653658	0.00544789	0.00392714	1.89918575	0.16816949	0.31349933	Ggt6
ENSMUSG00000040472.16	486.261439	-0.0076296	0.00313842	5.95784398	0.01465195	0.04405671	Rabggta
ENSMUSG00000040473.16	13.8157725	0.00890102	0.01104619	0.69391681	0.40483532	0.59633061	Cfap69
ENSMUSG00000040478.15	17.0980701	0.01517341	0.04594364	0.24468371	0.62084357	0.79105486	Prdm13
ENSMUSG00000040479.13	675.720519	0.01005964	0.00299334	11.2025213	0.00081686	0.00367752	Dgkz
ENSMUSG00000040481.18	759.620232	0.00215255	0.00358554	0.35852433	0.54932693	0.73191871	Bptf
ENSMUSG00000040482.17	268.525962	-0.0046874	0.00320702	2.1400928	0.14349321	0.27805052	Dxo
ENSMUSG00000040483.16	155.27308	0.03477377	0.00522919	43.5863403	4.06E-11	6.86E-10	Xaf1
ENSMUSG00000040488.20	894.897359	0.00371176	0.0045193	0.65449765	0.41850923	0.60995852	Ltbp4
ENSMUSG00000040489.6	1.81391842	-0.0367886	0.02033642	3.52490139	0.06045342	0.14082512	Sox30
ENSMUSG00000040498.8	13.9135707	0.00875367	0.00595847	2.08763463	0.14849583	0.28526003	Igsf23
ENSMUSG00000040502.6	10.1131716	0.00273646	0.00739456	0.14395355	0.70438186	0.85289545	Marchf9
ENSMUSG00000040505.14	1166.40518	-0.0006718	0.00510922	0.01776187	0.89397696	0.97270566	Abcg5
ENSMUSG00000040506.17	485.579394	0.00678393	0.00348407	3.73063232	0.05342317	0.12732809	Ambra1
ENSMUSG00000040511.15	144.171963	0.00489286	0.00369755	1.72457388	0.18910535	0.34307671	Pvr
ENSMUSG00000040520.8	403.556032	0.00938625	0.002831	10.8016741	0.00101408	0.00444989	Manea
ENSMUSG00000040521.12	211.008702	-0.0091067	0.00329906	7.8124746	0.00518868	0.01826025	Tsfm
ENSMUSG00000040522.6	54.1187563	0.05754417	0.00812575	43.8327184	3.58E-11	6.10E-10	Tlr8
ENSMUSG00000040524.10	363.651293	-0.0065707	0.00407762	2.56474593	0.10927011	0.22512621	Zfp609
ENSMUSG00000040525.14	230.013779	-0.0079761	0.00445867	3.22928824	0.07233212	0.16256499	Cblc
ENSMUSG00000040528.16	22.8701589	0.05562409	0.00810035	42.2798971	7.91E-11	1.29E-09	Milr1
ENSMUSG00000040532.15	493.12814	-0.0067806	0.00223998	9.33466992	0.00224859	0.00894353	Abhd11
ENSMUSG00000040536.16	920.668902	0.00726083	0.00425806	2.80324665	0.09407364	0.20047171	Necab1
ENSMUSG00000040537.18	25.6209409	0.04281175	0.00851604	21.456821	3.62E-06	2.82E-05	Adam22
ENSMUSG00000040543.17	9.58498233	0.00019582	0.01033621	0.00019611	0.9888268	1	Pitpnm3
ENSMUSG00000040548.17	2763.02851	-0.0031515	0.0024852	1.56966698	0.21025538	0.37210801	Tex2
ENSMUSG00000040549.17	280.742153	0.00685374	0.00320715	4.51414037	0.03361577	0.08732918	Ckap5
ENSMUSG00000040550.18	721.599182	-0.0090799	0.00242581	13.8356468	0.00019951	0.00106489	Otud6b
ENSMUSG00000040552.9	114.740313	0.0588862	0.00553142	102.737589	3.83E-24	3.25E-22	C3ar1
ENSMUSG00000040557.15	251.917436	0.00578317	0.00379142	2.3030762	0.12911806	0.25639782	Mettl27

ENSMUSG00000040560.11	435.492155	-0.0009188	0.00305281	0.08982487	0.76439992	0.89301781	Wdr7
ENSMUSG00000040562.13	1146.68341	0.01825483	0.00374485	24.2003966	8.68E-07	7.53E-06	Gstm2
ENSMUSG00000040563.14	93.1030635	-0.0080058	0.00541379	2.21144645	0.13699017	0.26835685	Plppr2
ENSMUSG00000040564.15	67880.7443	-0.0013074	0.0056868	0.05341151	0.81722989	0.92605476	Apoc1
ENSMUSG00000040565.9	496.011518	0.00140314	0.00327774	0.1786154	0.67256585	0.83015618	Btaf1
ENSMUSG00000040570.15	14.1974624	0.04329144	0.00718598	35.5005585	2.55E-09	3.35E-08	Rundc3b
ENSMUSG00000040583.9	563.918932	0.28198714	0.01791703	149.661208	2.06E-34	7.35E-32	Cyp2b13
ENSMUSG00000040584.9	125.881185	0.0568624	0.00602418	84.7587288	3.37E-20	1.79E-18	Abcb1a
ENSMUSG00000040586.9	110.656668	0.00978877	0.00322329	9.10506449	0.00254903	0.00993906	Ofd1
ENSMUSG00000040591.19	177.049478	-0.0033732	0.00634455	0.27353077	0.60097308	0.77486202	Cstpp1
ENSMUSG00000040592.12	47.2749147	0.08088832	0.00673946	126.709649	2.15E-29	3.87E-27	Cd79b
ENSMUSG00000040594.20	6.78328986	-0.0099963	0.0090327	1.19912253	0.27349713	0.45122434	Ranbp17
ENSMUSG00000040596.16	199.907258	0.00121111	0.00334026	0.1251212	0.72354517	0.86583137	Pogk
ENSMUSG00000040599.14	376.752343	-0.0024536	0.00280051	0.77245455	0.37945879	0.57093104	Mis12
ENSMUSG00000040600.10	1.02869746	0.01131372	0.01938026	0.5937657	0.44096663	0.63295027	Eps8l3
ENSMUSG00000040605.8	151.369065	-0.0056907	0.0056335	1.04277214	0.30717762	0.49216027	Bace2
ENSMUSG00000040606.15	38.7581045	0.01429839	0.00585161	5.90412328	0.01510548	0.04513275	Kazn
ENSMUSG00000040612.14	634.665243	0.04950817	0.0049218	99.0268591	2.49E-23	1.92E-21	Ildr2
ENSMUSG00000040613.15	377.271697	0.01290319	0.00450848	7.64524756	0.00569221	0.0197785	Apobec1
ENSMUSG00000040616.4	162.652395	0.04254293	0.00389147	115.662608	5.63E-27	6.87E-25	Tmem51
ENSMUSG00000040618.8	110.618309	0.01578539	0.00462797	11.4457178	0.00071659	0.00327802	Pck2
ENSMUSG00000040620.16	405.547991	-0.0085734	0.00266887	10.332053	0.0013074	0.00555008	Dhx33
ENSMUSG00000040621.15	24.5147444	0.00975183	0.00442924	4.81779275	0.02816739	0.07552935	Gemin8
ENSMUSG00000040624.19	110.332573	0.00615897	0.00355583	2.9500537	0.0858747	0.1864875	Plekhg1
ENSMUSG00000040629.9	2.87012986	0.0493583	0.036245	-2.3745178	1	1	Mael
ENSMUSG00000040631.9	30.8192271	-0.0005288	0.00537297	0.00956726	0.92208128	0.98770194	Dok4
ENSMUSG00000040640.12	189.528995	-0.0002388	0.00403384	0.00352494	0.95265642	1	Erc2
ENSMUSG00000040648.16	959.276428	-0.0097338	0.00294061	10.9984482	0.00091188	0.00404507	Ppip5k2
ENSMUSG00000040649.16	31.5005745	-0.0210982	0.01499085	1.5383532	0.21486309	0.37785274	Rimklb
ENSMUSG00000040651.10	754.027619	0.00305702	0.00268021	1.27276585	0.25924845	0.43292509	Tasor
ENSMUSG00000040652.18	569.957586	-0.0018896	0.00213216	0.78823762	0.37463356	0.56558326	Oaz2
ENSMUSG00000040653.7	40.7826359	0.01183908	0.01732977	0.56197689	0.45346482	0.6451186	Ppp1r14c
ENSMUSG00000040658.8	130.543842	-0.0046231	0.00489588	0.88719952	0.34623734	0.53457827	Dnph1
ENSMUSG00000040659.4	836.292963	0.023577	0.0032472	51.9547419	5.68E-13	1.23E-11	Efhd2
ENSMUSG00000040660.7	1771.891	0.1875753	0.01493355	127.364221	1.55E-29	2.89E-27	Cyp2b9
ENSMUSG00000040661.7	631.693485	-0.0062027	0.00440421	1.98052497	0.15933511	0.3006165	Rad54l2
ENSMUSG00000040663.10	31.4050602	0.05675924	0.00786402	46.7294986	8.15E-12	1.53E-10	Clcf1
ENSMUSG00000040666.21	69.5802062	-0.0202893	0.01631281	2.88645146	0.08932748	0.19242651	Sh3bgr
ENSMUSG00000040667.12	650.746234	-0.0040411	0.0024394	2.76652541	0.09625489	0.20410572	Nup88
ENSMUSG00000040669.15	58.8341044	-0.0038147	0.00512111	0.53623644	0.46399667	0.65495387	Phc1
ENSMUSG00000040675.18	37.9352423	0.0467596	0.00826073	30.5306254	3.29E-08	3.66E-07	Mthfd1l
ENSMUSG00000040680.10	1.38926642	0.04870969	0.01753075	8.71361693	0.00315842	0.01193248	Kremen2
ENSMUSG00000040681.17	1320.88005	0.00175126	0.00225696	0.60222049	0.43773206	0.62949505	Hmgn1
ENSMUSG00000040687.17	387.170194	0.00110102	0.00348905	0.098279	0.75390476	0.88616719	Madd
ENSMUSG00000040688.17	324.899931	-0.0071518	0.00333691	4.62570239	0.03149636	0.08278061	Tbl3
ENSMUSG00000040690.16	68.6177461	0.01848503	0.00551635	11.28787	0.00078015	0.00353519	Col16a1
ENSMUSG00000040693.8	3.74101923	0.08660983	0.01751104	21.7920416	3.04E-06	2.40E-05	Slco4c1
ENSMUSG00000040694.5	35.3155213	0.09027859	0.02025086	12.936158	0.0003223	0.0016229	Apobec2
ENSMUSG00000040697.11	274.338469	-0.0018853	0.00238791	0.62416541	0.42950359	0.62101525	Dnajc16
ENSMUSG00000040699.14	257.71604	0.02681047	0.00435864	37.0082913	1.18E-09	1.61E-08	Limd2
ENSMUSG00000040701.18	149.869856	0.00584461	0.00502896	1.338619	0.24727766	0.41863703	Ap1g2
ENSMUSG00000040703.12	13.9138079	0.00920024	0.00662364	1.96356677	0.16113237	0.30318852	Cyp2s1
ENSMUSG00000040706.5	2182.84424	-0.0052868	0.00420336	1.58671358	0.2077963	0.36888627	Agmat
ENSMUSG00000040710.11	56.9998751	0.05587182	0.00604123	75.6004241	3.47E-18	1.43E-16	St8sia4
ENSMUSG00000040711.9	48.3660609	0.07071058	0.007085	92.5199715	6.66E-22	4.38E-20	Sh3pxd2b
ENSMUSG00000040712.17	443.414993	-0.0073497	0.00363761	4.1604003	0.04137962	0.10344113	Camta2
ENSMUSG00000040713.13	8886.72915	-0.0036949	0.00335106	1.19794714	0.27373237	0.45143937	Creg1
ENSMUSG00000040714.15	5.43224467	0.00812657	0.00873962	0.87310268	0.35009778	0.53846797	Klc3
ENSMUSG00000040715.13	267.411924	-0.0329279	0.01731755	-2.8683924	1	1	Rsc1a1
ENSMUSG00000040717.7	17.7173886	0.01918126	0.00588566	10.2006053	0.00140395	0.0059089	Il17rd
ENSMUSG00000040720.16	466.860667	0.00259051	0.00275298	0.86864565	0.35133052	0.53976562	Virma

ENSMUSG00000040721.10	270.518931	-0.0003211	0.00530488	0.00353764	0.95257135	1	Zfhx2
ENSMUSG00000040722.8	248.80019	-0.0015788	0.00391839	0.15978845	0.68935136	0.84228708	Scamp5
ENSMUSG00000040723.15	156.753789	0.01848268	0.0043972	16.9689387	3.80E-05	0.00023959	Rcsd1
ENSMUSG00000040724.6	142.860502	0.03415716	0.00588694	32.9472683	9.47E-09	1.15E-07	Kcna2
ENSMUSG00000040725.14	1191.08954	0.00754463	0.00185467	16.4744977	4.93E-05	0.00030279	Hnrnpul1
ENSMUSG00000040726.11	0.38500606	0.01661886	0.03449755	0.41909519	0.51738887	0.70367011	Hesx1
ENSMUSG00000040728.16	6.15364392	0.02798545	0.0086487	10.4763081	0.00120915	0.00519378	Esrp1
ENSMUSG00000040729.9	11.1887155	0.01130881	0.0085482	1.63048556	0.2016359	0.3605139	Cep126
ENSMUSG00000040731.14	2405.19749	-0.002962	0.00165937	3.20252061	0.07352487	0.16479688	Eif4h
ENSMUSG00000040732.21	88.0735988	0.00460626	0.0039653	1.32392027	0.24988969	0.42144428	Erg
ENSMUSG00000040734.15	56.8289974	-0.0077222	0.00523427	2.10385873	0.14692793	0.28314247	Ppp1r13l
ENSMUSG00000040738.11	359.155781	-0.0152516	0.00328645	21.2113744	4.11E-06	3.17E-05	Ints8
ENSMUSG00000040740.8	112.690905	-0.0173889	0.00518966	10.5060806	0.00118982	0.00511635	Slc25a34
ENSMUSG00000040746.16	700.208759	0.00197856	0.00257255	0.58730399	0.44346355	0.63560593	Rnf167
ENSMUSG00000040747.10	239.883066	0.06105138	0.00564449	104.845837	1.32E-24	1.18E-22	Cd53
ENSMUSG00000040749.12	19.220952	0.00894286	0.0048915	3.23666665	0.072007	0.16199515	Siah1b
ENSMUSG00000040751.13	20.9194958	0.06817608	0.00758276	71.4514051	2.84E-17	1.06E-15	Lat2
ENSMUSG00000040752.17	11823.7282	-0.0441893	0.03095866	2.32980544	0.12691774	0.25308436	Myh6
ENSMUSG00000040760.12	330.650623	-0.0167304	0.00394194	17.9284527	2.29E-05	0.00015128	Appl1
ENSMUSG00000040761.17	585.892823	-0.0011294	0.00409759	0.07539292	0.78364069	0.90580491	Spen
ENSMUSG00000040767.12	79.4972312	-0.0037	0.00709112	0.27003658	0.60330723	0.77648764	Snrnp25
ENSMUSG00000040771.15	416.749082	-0.0058132	0.00242943	5.83979094	0.01566769	0.04650033	Oard1
ENSMUSG00000040774.16	1244.99811	0.00499841	0.00237419	4.3127549	0.03782766	0.09604942	Cept1
ENSMUSG00000040782.13	906.895664	-0.0090194	0.0027267	10.7486591	0.00104355	0.00455968	Cop1
ENSMUSG00000040785.20	687.644289	0.0088809	0.00399929	4.84834559	0.02767266	0.07441425	Ttc3
ENSMUSG00000040794.6	9.99001531	0.01253498	0.00793757	2.38135087	0.12279132	0.24665533	C1qtnf4
ENSMUSG00000040795.13	154.313837	-0.0105924	0.00409993	6.63507272	0.00999901	0.03189568	Iqcc
ENSMUSG00000040797.17	6.70218874	0.01296884	0.01758723	0.62699696	0.42845899	0.61982543	Iqsec3
ENSMUSG00000040808.5	22.4194	0.0286347	0.01767271	2.37059286	0.12364009	0.24799516	S100g
ENSMUSG00000040809.11	31.5081315	0.09816881	0.01517966	28.4263987	9.73E-08	1.01E-06	Chil3
ENSMUSG00000040811.16	131.993062	-0.0035948	0.00386702	0.86637771	0.35196007	0.54044566	Eml2
ENSMUSG00000040812.15	2.75905978	-0.0066799	0.01173377	0.32033787	0.57140467	0.75087716	Agbl2
ENSMUSG00000040813.17	950.736975	0.00127998	0.00278927	0.21036401	0.64648224	0.81100929	Tex264
ENSMUSG00000040818.11	471.331352	0.00126558	0.00266226	0.22033735	0.63878299	0.80521275	Dennd6a
ENSMUSG00000040820.17	304.0221	0.0166588	0.00334327	24.7234906	6.62E-07	5.88E-06	Hlcs
ENSMUSG00000040822.8	444.215051	-0.0076292	0.00228682	11.3128282	0.00076973	0.00349134	1700123O20Rik
ENSMUSG00000040824.4	570.790059	-0.0070517	0.00439073	2.59768757	0.10701976	0.22128238	Snrpd2
ENSMUSG00000040828.11	16.8785523	-7.65E-06	0.00617828	1.57E-06	0.99899891	1	Catsperd
ENSMUSG00000040829.15	79.4149117	0.02746794	0.00417979	41.90689	9.57E-11	1.54E-09	Zmynd15
ENSMUSG00000040836.16	6.74145297	0.03695571	0.00900919	17.3579746	3.10E-05	0.000198	Gpr161
ENSMUSG00000040838.11	4.29520659	0.03744404	0.01336156	7.95071432	0.00480684	0.01708986	Gm11639
ENSMUSG00000040841.6	54.8021966	-0.0001334	0.0034847	0.00147008	0.96941527	1	Six5
ENSMUSG00000040842.18	1516.808	-0.0066267	0.00299444	4.80421911	0.02839013	0.07599701	Szrd1
ENSMUSG00000040843.11	379.413238	-0.0027297	0.00295269	0.85060581	0.35638101	0.54514577	Tiprl
ENSMUSG00000040848.12	1143.55622	0.00189001	0.00203547	0.85987312	0.3537742	0.54245991	Sft2d2
ENSMUSG00000040850.18	2021.83852	-0.0137815	0.00229656	35.5692206	2.46E-09	3.25E-08	Psme4
ENSMUSG00000040852.7	52.5819486	0.01491049	0.00498772	8.79148225	0.0030264	0.01151057	Plekhh2
ENSMUSG00000040855.16	605.767555	0.0060457	0.00423282	2.0003573	0.15726213	0.29778297	Reps2
ENSMUSG00000040856.18	2.16814865	0.07538052	0.016309	21.0969089	4.37E-06	3.34E-05	Dlk1
ENSMUSG00000040857.16	390.536949	0.00639469	0.00345933	3.349323	0.06723266	0.15328656	Erf
ENSMUSG00000040859.15	973.202716	-0.0067439	0.00193705	11.956248	0.00054464	0.00258107	Bsdc1
ENSMUSG00000040860.17	41.3913871	0.00994654	0.00699792	1.9664137	0.16082904	0.30279235	Crocc
ENSMUSG00000040865.17	394.577797	0.00363384	0.00313448	1.31106793	0.25220139	0.42422066	Ino80d
ENSMUSG00000040867.14	3.25993944	0.03467252	0.01765552	4.42178271	0.03548295	0.0912584	Begain
ENSMUSG00000040875.13	5.32357221	0.01923521	0.00726496	6.94103114	0.00842405	0.02758813	Osbpl10
ENSMUSG00000040877.17	61.7664978	0.00286443	0.00437934	0.41036138	0.52178614	0.70781894	Wdr25
ENSMUSG00000040883.19	5409.72391	-0.0124234	0.00414842	9.11250249	0.00253868	0.00990527	Tmem205
ENSMUSG00000040888.11	627.195775	-0.0083134	0.00297873	7.83966811	0.0051112	0.01802523	Gfer
ENSMUSG00000040891.7	990.153218	-0.0139345	0.00535386	6.51176462	0.01071631	0.03385413	Foxa3
ENSMUSG00000040896.17	18.0252174	0.01703821	0.01074511	2.49570806	0.11415702	0.23285545	Kcnd3
ENSMUSG00000040899.15	1.78353577	0.06687236	0.01500572	17.0043938	3.73E-05	0.00023563	Ccr6

ENSMUSG00000040904.11	78.5589055	-0.0111182	0.00443013	6.33212392	0.01185702	0.0367451	Gm21988
ENSMUSG00000040907.16	58.6270798	0.07468116	0.00935281	56.3056258	6.20E-14	1.52E-12	Atp1a3
ENSMUSG00000040913.12	155.831922	0.00299478	0.00329724	0.82106863	0.36486791	0.55463873	Fbxw4
ENSMUSG00000040918.13	2282.81057	-0.0039125	0.00345739	1.27972766	0.25794968	0.43142825	Slc19a2
ENSMUSG00000040919.14	2.88815634	0.04639432	0.06863196	0.12328842	0.72549494	0.86741641	4930505A04Rik
ENSMUSG00000040928.16	355.983521	0.00236384	0.00264258	0.79988119	0.37112889	0.5616584	S100pbp
ENSMUSG00000040929.18	70.7166245	0.00829427	0.00377197	4.64555119	0.03113414	0.08199135	Rfx3
ENSMUSG00000040936.16	203.049479	-0.0188588	0.01031093	2.93554598	0.08664936	0.18785843	Ulk4
ENSMUSG00000040938.17	185.748533	-0.0066036	0.0049548	1.83217252	0.17587115	0.3245534	Slc16a11
ENSMUSG00000040940.19	658.042625	0.00549059	0.00298451	3.39031309	0.0655805	0.15034647	Arhgef1
ENSMUSG00000040943.13	331.072457	0.0133292	0.00378309	12.1010813	0.00050393	0.00241301	Tet2
ENSMUSG00000040945.14	525.806185	-7.09E-05	0.00296282	0.00056606	0.9810185	1	Rcc2
ENSMUSG00000040950.14	10.2684103	-0.0033935	0.01040666	0.07229408	0.78802543	0.90891329	Mgl2
ENSMUSG00000040952.17	5222.42746	-0.0018561	0.00422872	0.19117586	0.66193976	0.82248888	Rps19
ENSMUSG00000040957.16	39.2933096	0.0175265	0.00515796	11.6728103	0.0006342	0.00294744	Cables1
ENSMUSG00000040963.16	2668.75109	0.00369389	0.0046574	0.63284598	0.42631331	0.61789625	Asgr2
ENSMUSG00000040964.17	445.644732	-0.0105041	0.003827	7.44597955	0.00635783	0.02172328	Arhgef10l
ENSMUSG00000040966.11	25.3206164	0.01123122	0.02698825	0.26429959	0.60718154	0.7800525	Slc22a2
ENSMUSG00000040969.16	3.48119238	-0.0074215	0.01442495	0.23516054	0.62772349	0.79637923	Arhgef38
ENSMUSG00000040972.9	3.62536359	0.03852411	0.01081359	11.4176957	0.00072748	0.00332356	Igsf21
ENSMUSG00000040978.5	23.2706134	0.0029969	0.00577666	0.26458304	0.60698889	0.77984747	Gm11992
ENSMUSG00000040985.14	7.30330833	-0.0062315	0.00793104	0.61210264	0.43399722	0.62561169	Sun3
ENSMUSG00000040987.16	32.1210435	0.01396022	0.00503503	7.37376595	0.00661822	0.02253137	Mill2
ENSMUSG00000040990.18	119.251193	0.05189238	0.00664508	56.0894554	6.92E-14	1.69E-12	Sh3kbp1
ENSMUSG00000040997.15	658.901559	-0.0017067	0.00178297	0.92077357	0.33727194	0.52459087	Abhd4
ENSMUSG00000040998.19	35.0530258	0.03145773	0.00966458	8.76621955	0.00306861	0.01163979	Npnt
ENSMUSG00000041000.9	25.2022134	0.02063802	0.00455386	20.1359909	7.21E-06	5.27E-05	Trim62
ENSMUSG00000041009.9	1.89960962	-0.1186787	0.20950031	0.04899394	0.82482324	0.93064872	Iqcf4
ENSMUSG00000041012.5	950.497263	-0.0110115	0.00469273	5.56328555	0.01834096	0.05298101	Cmtm8
ENSMUSG00000041014.18	2.83418099	-0.0038343	0.02051311	0.02561945	0.8728332	0.96024272	Nrg3
ENSMUSG00000041020.15	87.8494297	0.00011996	0.01661351	-0.0003333	1	1	Map7d2
ENSMUSG00000041025.16	356.973724	0.00523371	0.00377171	1.8756691	0.1708272	0.31730433	Iffo2
ENSMUSG00000041028.17	5843.79251	-0.0087445	0.0025987	11.1244516	0.00085197	0.00381157	Ghitm
ENSMUSG00000041035.10	123.391285	-0.0042469	0.004422	0.92587799	0.33593632	0.5232362	Dpcd
ENSMUSG00000041037.9	359.026026	-0.0082653	0.00326442	6.36389056	0.01164659	0.03617759	Irgq
ENSMUSG00000041040.13	173.341894	0.01265546	0.00302204	17.3154212	3.17E-05	0.00020204	Fam117b
ENSMUSG00000041044.10	222.563171	-0.014847	0.0050236	8.71509955	0.00315585	0.01192469	Lrit1
ENSMUSG00000041046.8	4.15325695	0.01764745	0.00976305	3.27312243	0.07042347	0.15896333	Ramp3
ENSMUSG00000041052.9	115.680208	0.02597712	0.01869768	2.33336564	0.12662783	0.25257032	Slc7a13
ENSMUSG00000041057.11	902.197417	-0.002586	0.00362702	0.50233871	0.47847431	0.66840175	Wdr43
ENSMUSG00000041058.16	1702.57513	-0.0015251	0.00280217	0.29178124	0.58908132	0.76574976	Wwp1
ENSMUSG00000041064.15	5.23975503	0.03388757	0.01351395	5.76247425	0.016372	0.0482386	Pif1
ENSMUSG00000041073.11	4.37826292	0.02497512	0.01443303	2.26210534	0.13257372	0.26186113	Nacad
ENSMUSG00000041075.9	372.96688	-0.0033411	0.00317469	1.06564935	0.30193023	0.48636295	Fzd7
ENSMUSG00000041078.8	34.9563953	0.06266431	0.00814255	45.1392294	1.84E-11	3.27E-10	Grid1
ENSMUSG00000041079.13	148.300083	0.00030922	0.00226543	0.01864653	0.89138469	0.97082561	Rwdd2b
ENSMUSG00000041084.9	1297.96584	-0.0031425	0.00318092	0.98782926	0.3202735	0.50636619	Ostc
ENSMUSG00000041096.14	178.788922	0.0072266	0.00459439	2.45437049	0.11719815	0.23770919	Tspyl2
ENSMUSG00000041112.18	89.239676	0.05656407	0.00572888	90.5380393	1.81E-21	1.11E-19	Elmo1
ENSMUSG00000041115.19	217.783464	0.00533582	0.00338592	2.50595374	0.1134168	0.23164606	Iqsec2
ENSMUSG00000041117.10	19.2228993	-0.0135862	0.00507251	7.3440899	0.00672836	0.02284697	Ccdc8
ENSMUSG00000041119.13	345.53263	-0.0165991	0.00428459	15.203745	9.65E-05	0.00055318	Pde9a
ENSMUSG00000041120.7	19.3021744	0.00824578	0.00706561	1.30139548	0.25395848	0.42623388	Nbl1
ENSMUSG00000041124.12	454.515641	-0.0009049	0.00204303	0.19298166	0.6604465	0.82153687	Msantd4
ENSMUSG00000041126.17	629.670372	-0.0078495	0.00374772	4.42633672	0.03538838	0.09107151	H2az2
ENSMUSG00000041130.11	821.764407	-0.0130984	0.00257931	25.5621084	4.28E-07	3.95E-06	Zfp598
ENSMUSG00000041132.12	808.511141	-0.0049662	0.00379133	1.77653764	0.18257506	0.33398187	N4bp2l1
ENSMUSG00000041133.12	1485.71681	0.00034833	0.00256894	0.01796136	0.89338677	0.97233263	Smc1a
ENSMUSG00000041134.16	157.622379	-0.0009078	0.00432105	0.04259953	0.83648117	0.93837075	Cyyr1
ENSMUSG00000041135.16	131.749953	0.0054763	0.00294279	3.37341311	0.06625636	0.15152938	Ripk2
ENSMUSG00000041141.8	9.79297883	0.02552443	0.0217899	1.78407254	0.18165004	0.33269124	Pnma8a

ENSMUSG00000041143.17	256.96404	0.0070419	0.00309528	5.22105895	0.02231496	0.06233072	Tmco4
ENSMUSG00000041144.13	8.32256515	0.04323801	0.01211568	10.9795243	0.00092124	0.00408352	Dnah7b
ENSMUSG00000041147.11	82.8023367	0.01115446	0.00561064	4.01938171	0.0449802	0.11062597	Brca2
ENSMUSG00000041153.10	45.7291343	0.00502219	0.00433052	1.31720662	0.25109399	0.42286332	Osgin2
ENSMUSG00000041161.9	78.9135921	-0.0087361	0.00280993	9.65703647	0.00188628	0.00767859	Otud3
ENSMUSG00000041164.16	422.72408	-0.0007429	0.00349461	0.04469219	0.83257104	0.93567544	Zmiz2
ENSMUSG00000041165.7	1.1981825	-0.0213766	0.04090786	0.3558428	0.55082415	0.73333362	Spem1
ENSMUSG00000041168.11	2229.12731	-0.0105007	0.00211444	24.5748767	7.15E-07	6.30E-06	Lonp1
ENSMUSG00000041180.14	75.4002988	-0.0068021	0.00444189	2.23257317	0.13512864	0.26551926	Hectd2
ENSMUSG00000041187.17	136.708179	0.01105077	0.00349109	9.73408346	0.00180882	0.00739263	Prkd2
ENSMUSG00000041189.10	12.8308188	0.01675703	0.00720875	5.09065685	0.02405508	0.06628848	Chrnb1
ENSMUSG00000041193.16	9.09867491	0.04446446	0.01531773	5.8558847	0.01552505	0.04616413	Pla2g5
ENSMUSG00000041199.4	31.6307867	0.0089988	0.00436295	4.30140868	0.03808082	0.0965571	Rpusd1
ENSMUSG00000041202.13	8.96671153	0.16094255	0.0160044	65.3213823	6.36E-16	2.01E-14	Pla2g2d
ENSMUSG00000041203.10	822.949086	-0.0087609	0.00289853	9.0625684	0.00260895	0.01014421	Trir
ENSMUSG00000041205.9	2.26127095	0.00597295	0.01724356	0.15284923	0.69582727	0.84679748	Map6d1
ENSMUSG00000041215.17	216.16593	-0.0014225	0.00400063	0.12545604	0.72319069	0.86571464	Yeats2
ENSMUSG00000041216.16	10.1331394	-0.0016448	0.01280506	-0.0029711	1	1	Clvs1
ENSMUSG00000041219.16	49.3825457	0.02335464	0.00667299	11.7310226	0.00061467	0.002864	Arhgap11a
ENSMUSG00000041220.11	4414.73256	0.00781464	0.00652097	1.39090342	0.23825259	0.4075234	Elovl6
ENSMUSG00000041225.17	538.019388	-0.0007424	0.00236881	0.09774221	0.75455608	0.88645993	Arhgap12
ENSMUSG00000041229.16	275.278892	-0.0119843	0.00322214	13.6557084	0.00021957	0.00115884	Phf8
ENSMUSG00000041231.16	491.004156	-0.0056538	0.00217285	6.79658041	0.00913326	0.02958621	Ublcp1
ENSMUSG00000041235.13	541.927227	-0.0025152	0.00421614	0.34752469	0.5555177	0.73770849	Chd7
ENSMUSG00000041236.9	941.230012	0.00157901	0.0021788	0.51213449	0.47421631	0.66501373	Vps41
ENSMUSG00000041237.13	4871.04904	-0.0062707	0.00575288	1.16825864	0.27975933	0.45909751	Pklr
ENSMUSG00000041238.17	160.775644	0.00984836	0.00411803	5.54765277	0.01850549	0.05338435	Rbbp8
ENSMUSG00000041241.13	719.556776	-0.0078139	0.00293768	7.03697111	0.00798439	0.02634951	Mul1
ENSMUSG00000041245.15	5.84529273	0.03284927	0.01506153	4.67013438	0.03069153	0.08103628	Wnk3
ENSMUSG00000041248.10	7.63889546	0.02557976	0.03062727	0.70287849	0.40181817	0.59351724	Kcnj1
ENSMUSG00000041258.19	412.052453	0.00602399	0.0058388	0.97029184	0.32460741	0.51133309	Zfp236
ENSMUSG00000041261.10	2195.08085	-0.0052774	0.00333182	2.52478888	0.11206978	0.22947112	Car8
ENSMUSG00000041263.15	105.602592	-0.0027127	0.00316987	0.73776587	0.39037749	0.58179221	Rusc1
ENSMUSG00000041264.17	357.8425	-0.0010568	0.00211847	0.2464809	0.61956396	0.79010736	Uspl1
ENSMUSG00000041268.18	567.349292	-0.0027796	0.00314843	0.78370256	0.37601115	0.56715814	Dmxl2
ENSMUSG00000041272.12	118.443329	0.03684422	0.00440561	69.4789938	7.72E-17	2.73E-15	Tox
ENSMUSG00000041278.11	370.827829	-0.0028112	0.00224969	1.55706178	0.21209595	0.37439732	Ttc1
ENSMUSG00000041293.6	183.143402	-0.0613294	0.00833849	49.5949554	1.89E-12	3.82E-11	Adgrf1
ENSMUSG00000041297.9	643.2036	0.00384329	0.00304564	1.57423956	0.20959239	0.37126896	Cdk13
ENSMUSG00000041298.17	143.450429	0.00308458	0.00389039	0.60484321	0.43673606	0.62825426	Katnal1
ENSMUSG00000041301.16	5.68119269	0.0327002	0.00949989	11.6057613	0.00065748	0.00304363	Cftr
ENSMUSG00000041303.8	307.119184	0.00095392	0.00210172	0.2027629	0.6524999	0.81445081	Gtf3c3
ENSMUSG00000041308.7	228.19154	0.01111883	0.00326112	11.3335867	0.00076118	0.00345585	Sntb2
ENSMUSG00000041309.18	1.55024437	-0.0080833	0.02057074	0.23245357	0.62971048	0.79821311	Nkx6-2
ENSMUSG00000041313.15	53.6046476	0.02998932	0.00783277	13.4303038	0.00024759	0.00128685	Slc7a1
ENSMUSG00000041319.15	92.0865068	-0.0036632	0.0028654	1.63468051	0.20105689	0.35971717	Thoc6
ENSMUSG00000041323.7	9.11480176	0.02637234	0.00720712	12.8881847	0.00033066	0.00165937	Ak7
ENSMUSG00000041324.15	365.236907	-0.0197127	0.00563463	11.9789889	0.00053804	0.00255438	Inhba
ENSMUSG00000041328.17	690.020541	-0.0001845	0.00344229	0.00281659	0.95767491	1	Pcf11
ENSMUSG00000041329.14	40.5762229	0.01451945	0.00697745	4.35866509	0.03682115	0.09403027	Atp1b2
ENSMUSG00000041333.7	28.0775407	-0.0758901	0.01226656	35.3418244	2.77E-09	3.63E-08	Mup4
ENSMUSG00000041341.11	565.95819	-0.0092826	0.0020429	20.6094084	5.63E-06	4.22E-05	Atg2b
ENSMUSG00000041343.20	20.9402428	0.00246302	0.00511399	0.22642149	0.63419003	0.80186587	Ankrd42
ENSMUSG00000041346.12	102.994879	-0.0089319	0.0036139	6.11864969	0.01337629	0.04068256	Wrap53
ENSMUSG00000041351.17	92.1332913	0.0225127	0.00475071	22.6770102	1.92E-06	1.58E-05	Rap1gap
ENSMUSG00000041353.13	233.070845	-0.0002107	0.00332671	0.003847	0.95054359	1	Tmem29
ENSMUSG00000041354.17	217.884129	0.007165	0.00360296	3.93158497	0.04738726	0.11551013	Rgl2
ENSMUSG00000041355.14	1150.70637	-0.0054142	0.00262974	4.29112891	0.03831172	0.09702783	Ssr2
ENSMUSG00000041360.9	633.121866	-0.0021817	0.00370643	0.34218682	0.55856966	0.74017776	Pum3
ENSMUSG00000041361.14	140.380247	-0.0071278	0.00706165	0.98662623	0.32056835	0.50665409	Myzap
ENSMUSG00000041362.18	690.200445	-0.0038364	0.00287732	1.76304965	0.18424458	0.33652852	Shtn1

ENSMUSG00000041372.11	27.9343919	-0.0129392	0.00611565	4.30013415	0.03810937	0.0966191	B4galnt3
ENSMUSG00000041375.20	310.160737	0.00425312	0.00342275	1.51326608	0.21864139	0.38302824	Ccdc9
ENSMUSG00000041378.3	128.727576	-0.008068	0.00400079	4.04460746	0.04431268	0.10922342	Cldn5
ENSMUSG00000041380.14	3.10464328	0.00416714	0.02141305	0.0242086	0.87635531	0.96183824	Htr2c
ENSMUSG00000041390.19	404.336155	0.0066775	0.00254416	6.64049099	0.00996865	0.03182034	Mdfic
ENSMUSG00000041396.14	55.7700221	-0.0031004	0.00284854	1.18871546	0.27558882	0.45400837	Mettl18
ENSMUSG00000041399.4	1.75921442	-0.0229423	0.06669042	0.12950927	0.71894139	0.86242843	1700013G24Rik
ENSMUSG00000041406.15	11.5409928	0.00366521	0.00958359	0.13531801	0.71298064	0.85846738	Firrm
ENSMUSG00000041408.19	1804.84042	0.00080755	0.00277654	0.08231455	0.77418497	0.89950378	Wapl
ENSMUSG00000041415.12	747.933709	-0.0002951	0.00347798	0.00732202	0.93180922	0.99271395	Dicer1
ENSMUSG00000041417.16	1475.65198	0.00973561	0.00349369	7.81443749	0.00518305	0.01824588	Pik3r1
ENSMUSG00000041420.19	25.612182	0.05166684	0.00640764	58.5600635	1.97E-14	5.19E-13	Meis3
ENSMUSG00000041423.17	2.64384975	0.01046174	0.00999662	1.06986751	0.30097537	0.48532202	Paqr6
ENSMUSG00000041426.13	1415.97	-0.0108013	0.00300458	12.8564933	0.00033631	0.00168484	Hibch
ENSMUSG00000041429.4	84.5619311	-0.0086116	0.00410953	4.37333734	0.03650543	0.09341619	Nthl1
ENSMUSG00000041431.17	29.3118592	0.02199344	0.01194383	2.98281747	0.08415267	0.1834803	Ccnb1
ENSMUSG00000041438.9	280.58113	-0.007269	0.00217022	11.2317833	0.00080408	0.00362899	Utp4
ENSMUSG00000041439.16	240.459248	-0.0089938	0.00292379	9.44935459	0.00211223	0.00847697	Mfsd6
ENSMUSG00000041440.19	10.8043475	0.02283561	0.00691255	10.6227047	0.00111708	0.00483881	Gk5
ENSMUSG00000041444.15	301.323043	-0.0049064	0.00487732	1.00390867	0.31636657	0.50190502	Arhgap32
ENSMUSG00000041445.10	232.472059	0.00146899	0.00330238	0.19477287	0.65897353	0.82029538	Mmrn2
ENSMUSG00000041453.13	4044.07903	0.00355879	0.00286675	1.52957169	0.21617674	0.37968599	Rpl21
ENSMUSG00000041459.16	2015.31667	-0.0080031	0.00258379	9.3565556	0.00222189	0.00885229	Tardbp
ENSMUSG00000041460.17	3.26836303	0.07763081	0.01588769	21.3211535	3.88E-06	3.01E-05	Cacna2d4
ENSMUSG00000041468.8	14.2920536	-0.0021863	0.00845486	0.06551851	0.79797722	0.91515563	Gpr12
ENSMUSG00000041471.10	712.280901	-0.0279805	0.00386478	51.44718	7.35E-13	1.57E-11	Shld2
ENSMUSG00000041476.13	54.9602476	-0.0434107	0.03093514	-8.17E-05	1	1	Smpx
ENSMUSG00000041477.15	54.1165298	0.00907442	0.0034542	6.9074853	0.00858356	0.02806375	Dcp1b
ENSMUSG00000041479.15	76.1214963	-0.0302456	0.00795506	14.788663	0.00012026	0.00067565	Syt15
ENSMUSG00000041481.18	217.69921	0.05158235	0.00597199	67.6116463	1.99E-16	6.63E-15	Serpina3g
ENSMUSG00000041482.18	34.7050938	-0.0087628	0.00594576	2.11263829	0.14608727	0.28182678	Piezo2
ENSMUSG00000041483.15	571.675024	0.00280436	0.0034225	0.66541994	0.414653	0.6059759	Zfp281
ENSMUSG00000041488.17	126.070916	0.03820172	0.00493526	55.8177065	7.95E-14	1.92E-12	Stx3
ENSMUSG00000041491.9	33.3841096	0.00394174	0.00358006	1.21611907	0.27012363	0.44687775	Cep78
ENSMUSG00000041498.15	6.92436438	0.04654441	0.01226482	12.5695034	0.00039209	0.00192946	Kif14
ENSMUSG00000041506.16	157.099799	-0.0032697	0.00512646	0.40937517	0.52228682	0.70812191	Rrp9
ENSMUSG00000041515.11	192.77268	0.04534252	0.0046781	87.4941583	8.45E-21	4.77E-19	Irf8
ENSMUSG00000041528.17	1150.11873	-0.0117565	0.00357238	10.6732582	0.00108695	0.0047257	Rnf123
ENSMUSG00000041530.18	486.558333	0.00157906	0.0030053	0.27521425	0.59985526	0.77401314	ago-01
ENSMUSG00000041538.17	43.5397396	0.06562191	0.00681726	78.0119047	1.02E-18	4.53E-17	H2-Ob
ENSMUSG00000041540.17	427.498083	0.00369906	0.00518905	0.49610117	0.48121826	0.67084425	Sox5
ENSMUSG00000041548.5	3272.84313	-0.0099369	0.00395305	6.04844902	0.01391852	0.04215147	Hspb8
ENSMUSG00000041550.5	3.78753781	-0.022445	0.01046326	4.26627994	0.03887595	0.09822477	Serpina5
ENSMUSG00000041552.8	1.37137678	0.06290338	0.01951887	12.6705362	0.00037146	0.00183714	Ptchd1
ENSMUSG00000041556.9	4.60377676	0.01000035	0.01041771	0.9417466	0.33182907	0.51886239	Fbxo2
ENSMUSG00000041559.8	56.1328855	-0.0068771	0.00728462	0.88497141	0.34684366	0.53520801	Fmod
ENSMUSG00000041560.13	1223.81322	0.00263649	0.00242771	1.17472345	0.27843282	0.45741565	Nop53
ENSMUSG00000041566.4	3.04627145	0.00533396	0.00929776	0.34100578	0.55924924	0.74074536	Tssk1
ENSMUSG00000041567.4	1782.18123	-0.0162697	0.00827163	3.49047838	0.06172275	0.14313172	Serpina12
ENSMUSG00000041570.15	420.729126	-0.0019659	0.00276952	0.49562693	0.48142794	0.67097793	Camsap2
ENSMUSG00000041571.11	1341.99619	-0.0005106	0.00359527	0.02048423	0.88619295	0.96758388	Selenow
ENSMUSG00000041577.6	996.962629	0.00919648	0.0042766	4.46304751	0.03463565	0.08956436	Prelp
ENSMUSG00000041592.17	967.008544	0.01438869	0.0083323	2.80008971	0.09425903	0.20079422	Sdk2
ENSMUSG00000041594.19	100.217013	-0.0026543	0.00332341	0.62917724	0.42765727	0.61896702	Tmtc4
ENSMUSG00000041598.8	777.474645	0.00043697	0.00262598	0.02721756	0.86896173	0.95812872	Cdc42ep4
ENSMUSG00000041605.17	5.95691692	0.03698737	0.01494866	5.26793323	0.02172179	0.06089511	Inava
ENSMUSG00000041607.18	175.76816	0.04369467	0.00758939	29.8745117	4.61E-08	5.02E-07	Mbp
ENSMUSG00000041608.9	2.77108249	0.04912079	0.016814	7.76183346	0.00533618	0.01869564	Entpd3
ENSMUSG00000041609.17	36.9702495	0.00155765	0.00404066	0.14786411	0.70058491	0.85056083	Bicdl1
ENSMUSG00000041616.10	246.94409	-0.1017352	0.04373621	2.94517282	0.08613449	0.18689678	Nppa
ENSMUSG00000041617.11	1.62325876	0.02118294	0.01682293	2.02232116	0.15500204	0.29452012	Ccdc74a

ENSMUSG00000041623.18	71.260895	0.00142468	0.00292983	0.2289586	0.63229709	0.8000936	D11Wsu47e
ENSMUSG00000041624.11	11.6253698	0.0182154	0.00850224	4.39572229	0.0360292	0.09241388	Gucy1a2
ENSMUSG00000041625.16	1184.71778	-0.0098747	0.00300835	10.6020971	0.00112959	0.00488766	Ggact
ENSMUSG00000041629.8	420.987404	-0.0006834	0.00247293	0.07577912	0.78310108	0.90541669	Fam104a
ENSMUSG00000041632.8	523.207385	-0.0129032	0.0026428	23.5461639	1.22E-06	1.03E-05	Mrps27
ENSMUSG00000041633.15	58.6976078	0.02169549	0.00479893	19.4858691	1.01E-05	7.18E-05	Kctd12b
ENSMUSG00000041638.19	867.592809	-0.0113877	0.00341656	11.1086561	0.00085926	0.00383616	Gcn1
ENSMUSG00000041642.19	69.0664797	0.06023554	0.00550481	112.005521	3.56E-26	3.91E-24	Kif21b
ENSMUSG00000041644.15	6.69326678	0.01078034	0.026608	0.25840455	0.61121837	0.78348894	Slc5a12
ENSMUSG00000041645.15	773.074518	0.00416503	0.00263299	2.49245893	0.11439288	0.23315006	Ddx24
ENSMUSG00000041649.14	1.93289504	0.05401799	0.01306135	17.450992	2.95E-05	0.00018978	Klf8
ENSMUSG00000041650.16	1542.3965	-0.019354	0.00306851	39.5102023	3.26E-10	4.83E-09	Pcca
ENSMUSG00000041653.6	126.129281	0.0246227	0.00996945	6.22721218	0.01258016	0.03859466	Pnpla3
ENSMUSG00000041654.16	425.376861	0.00668138	0.00316493	4.3575546	0.03684516	0.09407122	Slc39a11
ENSMUSG00000041658.13	8.64702947	0.01107152	0.01264459	0.68463965	0.40799364	0.59963472	Rragb
ENSMUSG00000041660.9	2999.57203	-0.0023491	0.00438769	0.28487252	0.59352523	0.76887385	Bbox1
ENSMUSG00000041670.20	10.661758	0.03356886	0.01570429	4.58068199	0.03233434	0.08459654	Rims1
ENSMUSG00000041671.14	193.338208	-0.0008668	0.00252339	0.11747286	0.73179171	0.87130397	Pyroxd1
ENSMUSG00000041673.13	9.56066594	0.00582523	0.00812915	0.49397725	0.48215849	0.67144064	Lrrc18
ENSMUSG00000041679.10	60.015128	-0.0059972	0.00374991	2.61692175	0.10572937	0.2191522	Fbxl9
ENSMUSG00000041684.12	310.819298	-0.0073181	0.0030667	5.75557545	0.01643641	0.04838003	Bivm
ENSMUSG00000041685.18	794.293921	0.00185953	0.00282851	0.42159249	0.51614349	0.70275128	Fcho2
ENSMUSG00000041688.17	154.827625	0.00767374	0.00340969	5.09989343	0.02392731	0.06602897	Amot
ENSMUSG00000041695.3	46.2681538	0.04458317	0.00665152	41.3712334	1.26E-10	1.99E-09	Kcnj2
ENSMUSG00000041696.15	4.68202836	0.02515192	0.00892561	7.91742566	0.00489609	0.01736268	Rasl12
ENSMUSG00000041697.10	3841.41586	-0.0141917	0.00358536	15.7489951	7.23E-05	0.00042765	Cox6a1
ENSMUSG00000041698.12	6536.59091	-0.1082468	0.01072294	84.8624929	3.20E-20	1.71E-18	Slco1a1
ENSMUSG00000041702.8	459.612733	-0.0015277	0.00272716	0.31033313	0.57747583	0.755846	Btbd7
ENSMUSG00000041703.8	9.45682952	0.01074236	0.02143877	0.26159744	0.60902481	0.78139908	Zic5
ENSMUSG00000041707.9	26.9126406	0.03799023	0.00746245	22.8979506	1.71E-06	1.42E-05	Tmem273
ENSMUSG00000041708.13	4.97145668	0.03799973	0.01664194	4.98845458	0.02551699	0.06968203	Mpped1
ENSMUSG00000041712.8	330.998043	-0.0030586	0.0021386	2.03041315	0.15417869	0.2934758	Ubr7
ENSMUSG00000041716.8	76.0978255	-0.0010812	0.00298502	0.13147206	0.71691062	0.86099362	Gm20604
ENSMUSG00000041718.17	178.139281	-0.0108567	0.00374753	8.27704445	0.00401494	0.01467707	Alg13
ENSMUSG00000041720.15	712.723834	0.00410401	0.00309442	1.73740046	0.18746854	0.3408335	Pi4ka
ENSMUSG00000041729.16	45.8296966	0.0078565	0.00501519	2.45468187	0.11717491	0.23770919	Coro2b
ENSMUSG00000041731.14	32.7873465	-0.0065977	0.00742167	0.73590469	0.39097592	0.58228837	Pgm5
ENSMUSG00000041733.11	471.339129	-0.0185212	0.00239893	59.9819631	9.57E-15	2.64E-13	Coq5
ENSMUSG00000041734.16	48.0331868	0.02369635	0.00532429	19.6404699	9.35E-06	6.67E-05	Kirrel
ENSMUSG00000041736.8	1247.11761	-0.0015548	0.00369922	0.17758729	0.67345494	0.83086917	Tspo
ENSMUSG00000041737.9	12.66535	0.14163011	0.00880004	219.086388	1.43E-49	2.11E-46	Tmem45b
ENSMUSG00000041740.17	2134.21978	-0.0060072	0.00236351	6.47651933	0.01093088	0.03439379	Rnf10
ENSMUSG00000041741.11	101.781553	0.01103735	0.00456779	5.53912602	0.01859587	0.05359919	Pde3a
ENSMUSG00000041747.4	180.866093	0.00240351	0.00225857	1.11682385	0.29060339	0.47204625	Utp15
ENSMUSG00000041750.15	6.88034251	0.00769085	0.0081647	1.00623071	0.31580754	0.50132162	Cd1d2
ENSMUSG00000041754.6	4.64642216	0.09123491	0.01417883	33.6079067	6.74E-09	8.36E-08	Trem3
ENSMUSG00000041757.18	1114.1445	0.00027962	0.0035731	0.00597969	0.93836224	0.99606163	Plekha6
ENSMUSG00000041762.17	578.882166	0.00043613	0.00308086	0.0197	0.88837818	0.96916225	Gpr155
ENSMUSG00000041763.15	816.389246	-0.0082357	0.00287534	8.08783931	0.00445633	0.01603462	Tpp2
ENSMUSG00000041765.7	460.323877	-0.002283	0.00173739	1.74819433	0.18610389	0.33900574	Ubac2
ENSMUSG00000041769.14	613.936115	0.00046151	0.00293682	0.02372414	0.87758889	0.96264588	Ppp2r2d
ENSMUSG00000041773.9	161.609295	0.04146986	0.00464786	73.9747943	7.91E-18	3.14E-16	Enc1
ENSMUSG00000041774.17	5.39904938	0.0420359	0.00962674	17.6942335	2.59E-05	0.00016883	Ydjc
ENSMUSG00000041775.8	142.40505	0.00041996	0.00322223	0.01666581	0.8972816	0.97418909	Mapk1ip1
ENSMUSG00000041777.13	496.605747	-0.007999	0.00306053	6.78652469	0.00918485	0.02973291	Cir1
ENSMUSG00000041779.6	243.951789	-0.0087324	0.00347434	6.2474119	0.01243749	0.03826646	Tram2
ENSMUSG00000041781.10	454.737548	-0.0072511	0.00204073	12.5879416	0.00038824	0.00191211	Cpsf2
ENSMUSG00000041782.15	113.483937	-0.0454956	0.00620123	44.461364	2.59E-11	4.50E-10	Lad1
ENSMUSG00000041794.14	9.09151687	0.01113038	0.00877572	1.55323512	0.21265848	0.3750664	Myrip
ENSMUSG00000041797.8	103.114635	0.01818868	0.00500903	12.6588042	0.0003738	0.00184682	Abca9
ENSMUSG00000041798.16	2481.26548	-0.00387	0.004583	0.70804388	0.40009396	0.5918229	Gck

ENSMUSG00000041801.6	29.4712099	0.03926305	0.00634076	35.8950169	2.08E-09	2.76E-08	Phlda3
ENSMUSG00000041809.6	3.96766204	0.01993796	0.00920837	4.34922163	0.03702586	0.09439935	Efhc1
ENSMUSG00000041815.15	661.759852	0.00159815	0.00177287	0.80314084	0.37015597	0.56072567	Poldip3
ENSMUSG00000041817.15	18.3623676	0.01965537	0.00881887	4.898802	0.02687533	0.07270118	Fam169a
ENSMUSG00000041827.16	299.547722	0.02819424	0.00491787	32.5665294	1.15E-08	1.38E-07	Oasl1
ENSMUSG00000041828.17	1648.85539	-0.0422316	0.00488998	72.6315456	1.56E-17	6.03E-16	Abca8a
ENSMUSG00000041831.18	3.9296871	0.08155212	0.01028866	55.8458828	7.84E-14	1.89E-12	Sytl3
ENSMUSG00000041836.11	58.4261914	0.04016653	0.00502606	60.230216	8.44E-15	2.35E-13	Ptpre
ENSMUSG00000041837.5	153.361655	0.00159271	0.00303695	0.27675635	0.59883515	0.77320446	Pdcd7
ENSMUSG00000041840.9	109.025371	-0.0091568	0.00363	6.39125797	0.01146837	0.03577004	Haus1
ENSMUSG00000041841.9	2395.7523	-0.0016853	0.00355381	0.22509078	0.63518808	0.80282654	Rpl37
ENSMUSG00000041842.16	12.4250163	0.02508941	0.00795197	8.53547789	0.0034829	0.01298161	Fhdc1
ENSMUSG00000041845.12	541.395431	0.00436685	0.00416128	1.06510918	0.30205278	0.48652715	Rhod
ENSMUSG00000041846.16	1432.24572	8.19E-05	0.00265759	0.00093316	0.97563022	1	Ppp4r3a
ENSMUSG00000041849.8	275.397953	-0.005175	0.00354917	2.09826151	0.14746673	0.28381479	Card6
ENSMUSG00000041852.16	727.801426	-0.0031939	0.00323023	0.95347917	0.32883528	0.51589124	Tcf20
ENSMUSG00000041857.11	0.77916556	0.0999489	0.02143361	24.360519	7.99E-07	6.97E-06	Oosp1
ENSMUSG00000041859.11	117.924141	0.02903261	0.00576165	24.1643937	8.85E-07	7.66E-06	Mcm3
ENSMUSG00000041870.18	427.457612	0.01210879	0.00240276	25.225338	5.10E-07	4.64E-06	Ankrd13a
ENSMUSG00000041879.14	515.825837	0.00671962	0.00276464	5.84624004	0.01561037	0.04637105	Ipo9
ENSMUSG00000041881.14	2416.9928	-0.0111122	0.00422095	6.98211622	0.00823282	0.02707464	Ndufa7
ENSMUSG00000041886.8	4.62647337	0.01587014	0.00956097	2.71802225	0.09922089	0.2088926	Macc1
ENSMUSG00000041889.8	9.93342895	0.0254425	0.01039671	6.03747562	0.0140053	0.04236616	Shisa4
ENSMUSG00000041890.18	310.516153	0.00325939	0.00326778	0.96987181	0.32471215	0.51139562	Git2
ENSMUSG00000041891.17	5029.89245	-0.0049091	0.00315754	2.41876122	0.1198897	0.24205774	Lman1
ENSMUSG00000041895.16	339.219925	-0.0198449	0.0036149	30.1443867	4.01E-08	4.41E-07	Wipi1
ENSMUSG00000041907.10	10.130918	0.04359561	0.01844901	6.29191065	0.01212902	0.03747382	Gpr45
ENSMUSG00000041911.4	4.44168584	0.05048455	0.02500642	4.13538873	0.04199542	0.10464913	Dlx1
ENSMUSG00000041912.13	71.1957037	-0.0060298	0.00400451	2.22659775	0.13565226	0.26633599	Tdrkh
ENSMUSG00000041915.10	777.908036	-0.0021187	0.00317504	0.44389441	0.50524875	0.69211274	Ammecr1l
ENSMUSG00000041920.15	150.209674	-0.014881	0.00539097	7.36306861	0.00665771	0.02263313	Slc16a6
ENSMUSG00000041921.17	664.898671	-0.0029476	0.00267715	1.22477917	0.26842477	0.44465983	Metap1d
ENSMUSG00000041923.16	5.91614544	0.02165949	0.02406704	1.0042527	0.31628366	0.50180724	Nol4
ENSMUSG00000041926.16	595.184749	0.00300214	0.00255884	1.38988274	0.2384249	0.40773563	Rnpep
ENSMUSG00000041930.8	57.5173465	-0.0391914	0.00673247	32.3156319	1.31E-08	1.56E-07	Fam222a
ENSMUSG00000041935.11	629.510476	0.0022748	0.00291159	0.59937705	0.43881581	0.63074591	Rimoc1
ENSMUSG00000041936.19	523.211582	-0.0064362	0.00247888	6.88304868	0.0087017	0.02837863	Agrn
ENSMUSG00000041939.15	387.909487	-8.93E-05	0.00459291	0.00037088	0.98463514	1	Mvk
ENSMUSG00000041945.13	50.2763159	0.01011247	0.00363417	7.692993	0.00554356	0.01931014	Mfsd9
ENSMUSG00000041949.7	119.225183	-0.0120705	0.00237282	25.9840729	3.44E-07	3.24E-06	Tango6
ENSMUSG00000041954.19	7.92150091	0.05481718	0.00832363	37.5751346	8.80E-10	1.23E-08	Tnfrsf18
ENSMUSG00000041957.16	614.26155	-0.0053818	0.00268001	3.9391609	0.0471743	0.11516956	Pkp2
ENSMUSG00000041958.11	414.823012	-0.0050024	0.00205296	6.00933626	0.01423038	0.04298639	Pigs
ENSMUSG00000041959.15	1988.36501	-0.0036716	0.00398416	0.81038876	0.36800534	0.55836436	S100a10
ENSMUSG00000041961.15	203.615483	-0.0027078	0.00320426	0.71893143	0.39649464	0.58834367	Znrf3
ENSMUSG00000041966.19	294.401763	-0.0125277	0.00370815	11.3071078	0.00077211	0.00350076	Dcaf17
ENSMUSG00000041974.11	89.8776357	0.00731176	0.00273231	7.04498142	0.00794876	0.02624895	Spidr
ENSMUSG00000041975.18	192.58088	-0.0042039	0.00273818	2.36733875	0.12389811	0.24829166	Mettl8
ENSMUSG00000041977.19	596.033314	-0.001211	0.00310071	0.15304195	0.69564515	0.8467506	Arhgef11
ENSMUSG00000041986.18	12.1555865	0.04775159	0.01950984	7.17934465	0.00737476	0.02465502	Elmod1
ENSMUSG00000041992.10	234.387431	0.01217217	0.00407638	8.60927416	0.00334456	0.01254927	Rapgef5
ENSMUSG00000041995.9	66.1214523	0.00266012	0.00322688	0.68038184	0.40945527	0.60122094	Zbed3
ENSMUSG00000041997.17	1070.87874	0.00327766	0.00329557	0.97261754	0.32402824	0.51072685	Tlk1
ENSMUSG00000042010.17	2501.83354	-0.0119932	0.00502835	5.43621792	0.01972333	0.05624405	Acacb
ENSMUSG00000042015.19	263.535874	0.00681982	0.00189767	12.7165579	0.00036243	0.00179965	Wdr41
ENSMUSG00000042029.8	56.8314003	0.05240144	0.00612345	69.3951783	8.06E-17	2.84E-15	Ncapg2
ENSMUSG00000042032.14	852.008159	0.00130051	0.00234857	0.30788936	0.57897825	0.75713669	Mat2b
ENSMUSG00000042035.12	22.8912148	0.00225964	0.00548717	0.16448156	0.68506353	0.83935108	Igsf3
ENSMUSG00000042041.8	345.805134	0.03810615	0.00625057	31.7534779	1.75E-08	2.04E-07	2010003K11Rik
ENSMUSG00000042042.15	288.378941	0.00306277	0.00252166	1.44147642	0.22990057	0.3971316	Csgalnact2
ENSMUSG00000042043.7	2202.15647	-0.0045538	0.00373498	1.48738432	0.22262274	0.38794089	Tbca

ENSMUSG00000042045.7	5.90598645	0.03460327	0.03616489	-1.0132692	1	1	Sln
ENSMUSG00000042046.16	284.208405	-2.30E-05	0.00316648	5.20E-05	0.99424592	1	Dstyk
ENSMUSG00000042050.9	170.418284	-0.0094083	0.00594156	2.43870496	0.11837392	0.23963327	Dync2i1
ENSMUSG00000042055.14	587.997537	-0.0028852	0.0016184	3.17970354	0.07455826	0.16660026	Wdr11
ENSMUSG00000042063.12	234.674662	0.00173462	0.00323554	0.28509932	0.59337825	0.76876789	Zfp386
ENSMUSG00000042064.14	1.70120303	0.11330405	0.01851179	35.9772946	2.00E-09	2.66E-08	Myo3b
ENSMUSG00000042066.17	78.3115418	0.00085049	0.00425795	0.03955152	0.84236003	0.94223647	Tmcc2
ENSMUSG00000042073.16	3281.61977	0.00284543	0.00311538	0.8332874	0.36132366	0.5509199	Abhd14b
ENSMUSG00000042078.15	1.70872594	0.01908608	0.02202989	1.26897318	0.2599594	0.43386657	Svop
ENSMUSG00000042079.14	3600.89475	0.003005	0.00159108	3.50794613	0.06107513	0.14200689	Hnrnpf
ENSMUSG00000042082.8	606.834728	0.00774331	0.00287511	7.17389827	0.00739719	0.02471947	Arsb
ENSMUSG00000042096.16	8.68914592	-0.0007455	0.03065147	-0.0131573	1	1	Dao
ENSMUSG00000042097.18	6.86015225	0.02454545	0.0103316	5.07422529	0.02428412	0.06681037	Zfp239
ENSMUSG00000042099.17	103.372248	-0.0020109	0.00400098	0.25493003	0.61362489	0.78537859	Kank3
ENSMUSG00000042102.8	7364.97386	0.0012466	0.004892	0.06330135	0.80135211	0.91653045	Dmgdh
ENSMUSG00000042104.19	59.4200029	-0.0034057	0.00528676	0.41463598	0.51962579	0.70565949	Uggt2
ENSMUSG00000042105.19	486.949671	0.00094396	0.00303242	0.0963569	0.75624609	0.88746728	Inpp5f
ENSMUSG00000042106.6	9.71089814	0.01075017	0.0064711	2.72416099	0.09884005	0.20827672	Inka1
ENSMUSG00000042109.9	15.4922484	0.01629819	0.00911186	3.1988995	0.07368784	0.16506804	Csdc2
ENSMUSG00000042111.10	292.956944	0.00671645	0.0031471	4.54181448	0.03307651	0.08616552	Ccdc115
ENSMUSG00000042115.5	53.3964016	-0.0091887	0.00515979	3.05666996	0.0804063	0.17684287	Klhdc8a
ENSMUSG00000042116.4	19.1278	0.01226216	0.00515563	5.52110168	0.01878842	0.0540551	Vwa1
ENSMUSG00000042118.8	3621.67495	-0.0005682	0.00392585	0.02103444	0.88468518	0.96658844	Bhmt2
ENSMUSG00000042121.17	234.522755	0.01184959	0.00307312	14.9696888	0.00010925	0.00061854	Ssh1
ENSMUSG00000042129.9	215.390159	0.05694932	0.00553941	97.5092423	5.36E-23	3.90E-21	Rassf4
ENSMUSG00000042133.17	1751.46687	0.00352508	0.00402201	0.75985799	0.38337297	0.57505546	Ppig
ENSMUSG00000042138.10	121.852753	-0.0041411	0.00472947	0.76751668	0.38098639	0.57247911	Msantd2
ENSMUSG00000042148.9	232.294546	-0.0151717	0.00277831	30.3107093	3.68E-08	4.07E-07	Cox10
ENSMUSG00000042155.4	40.6783648	0.0133352	0.0054112	5.81361516	0.01590257	0.04708019	Klhl23
ENSMUSG00000042156.17	27.646201	0.04696031	0.00643298	50.9242543	9.60E-13	2.02E-11	Dzip1
ENSMUSG00000042165.10	54.8411844	0.00578582	0.00610951	0.87704208	0.34901311	0.53707988	Adrm1b
ENSMUSG00000042167.7	505.105346	0.00759229	0.00223756	11.5088793	0.00069265	0.00318331	Tent2
ENSMUSG00000042178.7	159.941447	0.00010878	0.00274239	0.00154706	0.96862515	1	Armc5
ENSMUSG00000042182.17	9.92064464	0.03539001	0.00850107	15.6550249	7.60E-05	0.00044708	Bend6
ENSMUSG00000042185.15	243.724682	0.00515198	0.00278508	3.37819272	0.06606446	0.15119313	Nfrkb
ENSMUSG00000042190.13	70.1627134	0.04511965	0.00596882	54.6829561	1.42E-13	3.31E-12	Cmklr1
ENSMUSG00000042195.10	16.8641423	0.01708381	0.00889278	3.21676519	0.07288754	0.16358601	Slc35f2
ENSMUSG00000042197.14	281.909526	0.00240799	0.00254985	0.87963894	0.34830059	0.53630877	Zfp451
ENSMUSG00000042198.13	277.58207	-0.0302599	0.0043331	50.5293661	1.17E-12	2.43E-11	Chchd7
ENSMUSG00000042200.4	0.91703317	-0.0154893	0.06318953	0.0899299	0.76426629	0.89301781	Cdrt4
ENSMUSG00000042202.16	1209.08866	-0.0025312	0.0033226	0.58000623	0.44630988	0.63834791	Slc35e2
ENSMUSG00000042203.9	106.426339	0.00295363	0.00289827	1.03161371	0.30977995	0.49498476	Tbc1d22b
ENSMUSG00000042207.18	424.091394	-0.0191702	0.00372826	25.6347088	4.13E-07	3.82E-06	Kdm5b
ENSMUSG00000042208.17	429.842174	-0.000174	0.00371459	0.0021731	0.96281886	1	Sanbr
ENSMUSG00000042210.19	162.576842	-0.0206532	0.00292056	49.8184442	1.69E-12	3.43E-11	Abhd14a
ENSMUSG00000042211.8	501.58348	-0.0007847	0.00213014	0.13455135	0.71375894	0.85896158	Fbxo38
ENSMUSG00000042213.19	728.202412	0.00693997	0.00374004	3.33544459	0.06780208	0.15428653	Zfand4
ENSMUSG00000042215.9	72.850643	0.00230739	0.00504599	0.2049655	0.65074234	0.81359178	Bag2
ENSMUSG00000042216.14	72.8735727	-0.0068895	0.00538819	1.62685203	0.20213901	0.36118566	Sgsm1
ENSMUSG00000042225.4	80.2687044	0.00703857	0.00453573	2.33367535	0.12660265	0.25255558	Ammecr1
ENSMUSG00000042228.15	643.748862	0.0193983	0.00296727	41.398642	1.24E-10	1.97E-09	Lyn
ENSMUSG00000042229.10	279.223775	0.00110786	0.00154894	0.51127866	0.47458586	0.66524436	Rabif
ENSMUSG00000042246.6	110.533127	-0.0124456	0.00480855	6.73851508	0.00943531	0.03041012	Tmc7
ENSMUSG00000042248.5	5889.27794	0.02483621	0.00689737	12.1975102	0.00047853	0.00230247	Cyp2c37
ENSMUSG00000042249.12	147.000909	0.03398017	0.00551896	36.7593689	1.34E-09	1.82E-08	Grk3
ENSMUSG00000042251.13	2375.59083	-0.0006241	0.00468562	0.01760321	0.89444877	0.97287826	Pm20d1
ENSMUSG00000042254.15	8.89441326	0.01507495	0.01016136	2.00944421	0.15632258	0.29650376	Cilp
ENSMUSG00000042256.6	1.38099469	0.00066823	0.02526066	0.00180087	0.96615058	1	Ptchd4
ENSMUSG00000042265.14	3.50408575	0.10210736	0.01366462	45.672764	1.40E-11	2.52E-10	Trem1
ENSMUSG00000042271.14	974.575322	8.70E-05	0.00291461	0.00087611	0.97638675	1	Nxt2
ENSMUSG00000042272.18	88.6615901	0.02168892	0.00488695	19.3491357	1.09E-05	7.66E-05	Sestd1

ENSMUSG00000042275.10	204.400087	-0.0057755	0.00308724	3.47862064	0.06216654	0.14389205	Pelo
ENSMUSG00000042284.11	775.258469	0.0068265	0.0030509	4.93445458	0.02632637	0.07143739	Itga1
ENSMUSG00000042286.14	977.653072	0.00539423	0.00584349	0.86688761	0.35181839	0.54037436	Stab1
ENSMUSG00000042289.12	8575.61776	-0.0177053	0.00443549	15.8761702	6.76E-05	0.00040247	Hsd3b7
ENSMUSG00000042292.19	152.337787	0.01139991	0.00383717	8.76942989	0.00306321	0.01162867	Mrtfa
ENSMUSG00000042293.9	330.161698	-0.0067737	0.00459401	2.16684772	0.14101503	0.27435293	Gm5617
ENSMUSG00000042298.19	269.077211	-0.0054278	0.00241165	5.01926895	0.02506675	0.06860343	Ttc19
ENSMUSG00000042302.15	228.716298	-0.0088174	0.00496894	3.15892262	0.075513	0.1682364	Ehbp1
ENSMUSG00000042303.20	129.125302	-0.0101847	0.00370428	7.28455059	0.00695501	0.02351504	Sgsm3
ENSMUSG00000042305.13	2407.49151	-0.0093965	0.00231479	16.3843193	5.17E-05	0.00031574	Tmem183a
ENSMUSG00000042308.15	397.871069	-0.0064893	0.00414077	2.39041309	0.12208137	0.24555783	Setd1a
ENSMUSG00000042312.11	355.161315	-0.0034507	0.00413166	0.71929382	0.39637565	0.5882041	S100a13
ENSMUSG00000042320.18	36.1499254	0.00064673	0.00445693	0.02071841	0.88554872	0.96711168	Prox2
ENSMUSG00000042323.18	1079.35024	0.0032775	0.00360229	0.80370604	0.36998763	0.56050666	Pbrm1
ENSMUSG00000042328.14	198.26773	-0.0147198	0.00396935	14.0425109	0.00017872	0.00096728	Hps4
ENSMUSG00000042331.14	117.697538	0.01771225	0.00585113	8.99229684	0.0027112	0.01048645	Specc1
ENSMUSG00000042333.17	53.3441552	0.02702217	0.00502572	27.2936642	1.75E-07	1.74E-06	Tnfrsf14
ENSMUSG00000042340.7	81.6593812	0.00434898	0.00434667	1.0148642	0.31374034	0.49912502	Ctf1
ENSMUSG00000042345.16	10.8354408	0.07178529	0.01060259	40.4669028	2.00E-10	3.06E-09	Ubash3a
ENSMUSG00000042348.11	181.467956	0.00470902	0.0030583	2.30578664	0.12889301	0.25613174	Arl15
ENSMUSG00000042349.14	379.370288	0.01737101	0.00438494	15.4136002	8.64E-05	0.00050061	Ikbke
ENSMUSG00000042350.14	800.970225	-0.0001718	0.00310771	0.0029732	0.95651526	1	Arel1
ENSMUSG00000042351.10	22.953373	0.07704506	0.00811902	75.4370113	3.77E-18	1.55E-16	Grap2
ENSMUSG00000042354.8	771.504633	-0.0086848	0.00393907	4.79256573	0.02858282	0.07642198	Gnl3
ENSMUSG00000042359.19	18.3499786	0.02308849	0.01295236	1.94689235	0.16292212	0.30572688	Osbpl6
ENSMUSG00000042363.15	451.514616	0.0068907	0.00441078	2.33028372	0.12687875	0.253028	Lgalsl
ENSMUSG00000042364.12	454.363335	0.00144953	0.00185698	0.60010066	0.43853962	0.63046419	Snx18
ENSMUSG00000042369.9	166.18239	-0.0019358	0.00216551	0.78562517	0.37542626	0.5665222	Rbm45
ENSMUSG00000042371.13	3.36101108	-0.000559	0.03221436	-0.0041602	1	1	Slc5a10
ENSMUSG00000042377.9	56.1121563	-0.0068858	0.00367428	3.38892993	0.06563553	0.15040002	Fam83g
ENSMUSG00000042379.9	83.2204794	0.10344941	0.00910308	100.868592	9.83E-24	7.83E-22	Esm1
ENSMUSG00000042380.9	310.623569	-0.0008398	0.00289117	0.08519302	0.77037957	0.89702669	Smim12
ENSMUSG00000042385.15	5.47763714	0.20074045	0.01556387	124.455705	6.70E-29	1.09E-26	Gzmk
ENSMUSG00000042388.15	10.0231692	0.00955308	0.01072571	0.76659888	0.38127128	0.57274469	Dlgap3
ENSMUSG00000042389.14	110.172042	0.00181045	0.00353267	0.26734544	0.60511806	0.77801967	Tsen2
ENSMUSG00000042390.10	583.883921	0.00304415	0.00365658	0.67717038	0.4105628	0.60217239	Gatad2b
ENSMUSG00000042396.11	1290.24368	0.00090537	0.00214771	0.17656803	0.67433937	0.83155036	Rbm7
ENSMUSG00000042401.9	1.19641889	0.04044153	0.02364013	4.15303775	0.0415599	0.10380479	Crtac1
ENSMUSG00000042404.17	122.856206	0.00934154	0.00421478	4.8583666	0.02751237	0.07403386	Dennd4b
ENSMUSG00000042406.9	2146.81251	-0.0019418	0.00366585	0.28301963	0.59472884	0.76996786	Atf4
ENSMUSG00000042408.14	156.052571	-0.0001924	0.00393739	0.00228021	0.96191428	1	Zmym6
ENSMUSG00000042410.17	330.205586	0.00475019	0.00371368	1.58602363	0.20789517	0.36898302	Agps
ENSMUSG00000042417.6	9.04142452	0.00161426	0.00606849	0.0609209	0.8050463	0.91866186	Ccno
ENSMUSG00000042419.9	90.179342	0.00422975	0.00317956	1.76416789	0.1841055	0.33637867	Nfkbil1
ENSMUSG00000042423.10	415.513598	-0.0017445	0.00363372	0.22715933	0.63363819	0.80146889	Fbrs
ENSMUSG00000042425.19	2.54764231	0.05175536	0.02056401	7.1758162	0.00738928	0.02469656	Frmpd3
ENSMUSG00000042426.7	269.37616	-0.0093518	0.00392926	5.58478847	0.01811712	0.05248869	Dhx29
ENSMUSG00000042428.7	16.3599816	0.0413291	0.00890574	21.297997	3.93E-06	3.04E-05	Mgat3
ENSMUSG00000042429.10	383.557761	0.02184133	0.0047172	21.7189861	3.16E-06	2.49E-05	Adora1
ENSMUSG00000042436.13	25.5638515	-0.0129762	0.0074275	2.95983531	0.08535663	0.18555038	Mfap4
ENSMUSG00000042439.14	69.6544532	0.0087411	0.00555819	2.40797881	0.12071814	0.24333459	Zfp532
ENSMUSG00000042444.11	733.309044	0.01163046	0.00425302	7.46245215	0.00629991	0.02154411	Mindy2
ENSMUSG00000042446.17	235.952095	0.01111443	0.00335631	10.6446568	0.00110389	0.00478699	Zmym4
ENSMUSG00000042447.14	228.509446	-0.0001153	0.00221475	0.00270653	0.95850933	1	Mios
ENSMUSG00000042448.6	2.62032573	-0.007148	0.04411581	-9.61E-05	1	1	Hoxd1
ENSMUSG00000042451.13	1.20478057	0.02247315	0.02544374	1.66718326	0.19663624	0.35346866	Mybph
ENSMUSG00000042453.15	1036.2775	0.01668621	0.00388171	17.6175179	2.70E-05	0.00017549	Reln
ENSMUSG00000042460.6	1140.19818	0.00259215	0.00247464	1.08074787	0.29853031	0.4824348	C1galt1
ENSMUSG00000042462.5	500.975395	-0.0147115	0.00402787	13.7269636	0.0002114	0.00112096	Dctpp1
ENSMUSG00000042472.12	331.268083	-0.0058428	0.00198279	8.76880834	0.00306426	0.01163076	Zfp410
ENSMUSG00000042473.9	419.608042	0.00224824	0.00364709	0.3717405	0.54205707	0.72551118	Tbc1d8b

ENSMUSG00000042474.7	15.8780434	0.0818481	0.00805973	89.6484619	2.84E-21	1.70E-19	Fcmr
ENSMUSG00000042476.13	3880.81923	-0.0087591	0.00380666	5.08076859	0.02419264	0.06661308	Abcb4
ENSMUSG00000042485.8	104.717975	0.04513687	0.00398178	119.936099	6.53E-28	9.69E-26	Mustn1
ENSMUSG00000042487.7	325.627795	-0.0079934	0.00347578	5.17849643	0.0228681	0.06361211	Leo1
ENSMUSG00000042489.16	17.8903017	0.04562695	0.01128973	15.0221641	0.00010626	0.00060375	Clspn
ENSMUSG00000042492.13	223.089838	0.01079985	0.00305341	12.4403064	0.00042017	0.00205007	Tbc1d10b
ENSMUSG00000042496.19	141.961821	-0.0031476	0.00302955	1.06325127	0.30247481	0.48707387	Prdm10
ENSMUSG00000042498.17	8.55267871	0.07395791	0.01511795	22.7044968	1.89E-06	1.56E-05	Radx
ENSMUSG00000042499.14	4.12373919	-0.0037806	0.0610219	-0.0213923	1	1	Hoxd11
ENSMUSG00000042500.12	131.086941	-0.0006033	0.00396017	0.02260547	0.88048757	0.96408053	ago-04
ENSMUSG00000042501.13	1.27515708	0.00683829	0.02703361	0.20767786	0.64859359	0.81215377	Cpa6
ENSMUSG00000042502.11	438.283459	0.00199048	0.00208338	0.91565816	0.33861759	0.5263021	Cd2bp2
ENSMUSG00000042505.7	205.134119	-0.0098823	0.00288348	11.6474306	0.00064291	0.00298499	Sdhaf3
ENSMUSG00000042506.16	609.539066	0.01746789	0.00265764	42.8406012	5.94E-11	9.86E-10	Usp22
ENSMUSG00000042507.16	217.388846	-0.0002136	0.00314252	0.00447942	0.94663862	1	Mideas
ENSMUSG00000042508.17	401.12383	0.00109826	0.00252552	0.18467714	0.66738422	0.82620849	Dmtf1
ENSMUSG00000042510.8	41.1441437	-0.0026013	0.00729913	0.13282688	0.71551885	0.85997899	AA986860
ENSMUSG00000042514.12	2.27078236	0.09208878	0.01579514	33.8748282	5.88E-09	7.35E-08	Klhl14
ENSMUSG00000042515.14	8.85794809	-0.0022093	0.01006864	0.04324439	0.83526578	0.93759884	Pwwp3b
ENSMUSG00000042520.17	1069.11524	0.00148631	0.00297087	0.24981258	0.61720707	0.78846591	Ubap2l
ENSMUSG00000042523.13	35.5742559	0.00581869	0.00570306	0.98645638	0.32061001	0.50666049	Dnal1
ENSMUSG00000042524.15	1259.26385	-0.0053076	0.00271589	3.6390329	0.05643971	0.13342558	Sun2
ENSMUSG00000042529.15	6.35127142	0.01020513	0.02828757	0.52736547	0.46771658	0.65887223	Kcnj12
ENSMUSG00000042532.15	3.18152917	0.01284587	0.020017	0.60159519	0.43797003	0.62979888	Golga7b
ENSMUSG00000042535.10	323.86936	-0.0022081	0.00289917	0.58042637	0.44614525	0.63824721	Gtpbp1
ENSMUSG00000042541.11	1049.87898	-0.0049566	0.0035004	2.01358888	0.15589615	0.29590911	Sem1
ENSMUSG00000042548.15	433.002754	0.00696456	0.00229998	9.0417398	0.00263884	0.01024186	Asxl1
ENSMUSG00000042557.15	547.795443	0.00118734	0.00271676	0.1901838	0.66276369	0.82289764	Sin3a
ENSMUSG00000042558.17	244.561679	-0.0058308	0.00263864	4.87250875	0.0272878	0.07353023	Adprs
ENSMUSG00000042564.15	8.93604971	-0.0119904	0.00700442	2.75348199	0.09704288	0.20538778	Fam227a
ENSMUSG00000042567.21	2.31673015	0.01284688	0.01890312	0.88405669	0.34709299	0.53537747	Nek10
ENSMUSG00000042569.14	395.263583	-0.002292	0.00308373	0.54395079	0.46079999	0.65205649	Dhrs7b
ENSMUSG00000042570.16	141.565721	-0.0093411	0.00315532	8.61634455	0.0033316	0.01250662	Mier2
ENSMUSG00000042572.13	624.083255	0.00631704	0.00179237	12.4500117	0.00041799	0.00203987	Ube2q1
ENSMUSG00000042581.15	2.71540965	0.02292379	0.02081242	2.11388027	0.14596878	0.28166732	Thsd7b
ENSMUSG00000042589.19	149.253535	0.07919381	0.01218313	33.6220636	6.69E-09	8.31E-08	Cux2
ENSMUSG00000042590.18	323.330241	-0.0017306	0.00260447	0.43476974	0.50965774	0.69625181	Ipo11
ENSMUSG00000042594.17	357.760862	0.00516531	0.0027596	3.48636766	0.06187621	0.14343119	Sh2b3
ENSMUSG00000042595.8	216.563515	0.0018333	0.00331752	0.29871675	0.58468802	0.76179792	Fam199x
ENSMUSG00000042599.9	488.365714	0.00517739	0.00276384	3.3865474	0.06573045	0.15056021	Kdm7a
ENSMUSG00000042604.6	7.70863593	0.00409638	0.02333388	0.06011439	0.80631523	0.91939817	Kcna4
ENSMUSG00000042605.17	1354.59228	-0.0081515	0.00454844	3.09635035	0.07846802	0.17353463	Atxn2
ENSMUSG00000042606.10	78.7547471	0.003459	0.00568248	0.34997633	0.55412653	0.73644156	Hirip3
ENSMUSG00000042607.16	13.4276691	0.03466555	0.00826412	16.2158943	5.65E-05	0.00034295	Asb4
ENSMUSG00000042608.16	763.059997	-0.0061714	0.00419429	2.10209232	0.14709773	0.28334371	Stk40
ENSMUSG00000042613.10	320.802784	0.00931348	0.00280454	11.1637908	0.00083409	0.00374366	Pbxip1
ENSMUSG00000042616.9	10.4388859	0.01180434	0.01067616	1.0366186	0.3086092	0.49351527	Oscp1
ENSMUSG00000042622.15	73.6150136	0.01310296	0.00581355	4.71011875	0.02998558	0.07943838	Maff
ENSMUSG00000042625.17	1017.83271	-0.0056771	0.00438354	1.63638457	0.20082224	0.35940461	Safb2
ENSMUSG00000042626.14	1094.09778	0.0009657	0.00191793	0.25176959	0.6158318	0.78726335	Shc1
ENSMUSG00000042628.9	972.15586	-0.014975	0.00299423	24.7564325	6.51E-07	5.79E-06	Zfyve1
ENSMUSG00000042632.18	636.923178	-0.0003957	0.00308204	0.01574142	0.90015558	0.97587825	Pla2g6
ENSMUSG00000042638.15	30.4464701	0.01964324	0.011935	2.62466391	0.10521478	0.21831581	Gucy2c
ENSMUSG00000042642.14	684.861947	-0.0090421	0.00217247	17.3829029	3.06E-05	0.00019579	Flad1
ENSMUSG00000042644.10	57.620733	0.03918021	0.00575709	43.528401	4.18E-11	7.04E-10	Itpr3
ENSMUSG00000042647.14	191.989034	-0.0033204	0.00404464	0.65318137	0.41897755	0.61037705	Acad12
ENSMUSG00000042650.4	1404.60315	-0.0036927	0.00222514	2.72295639	0.09891465	0.2083967	Alkbh5
ENSMUSG00000042659.16	299.007123	0.01828047	0.00286906	40.7575798	1.72E-10	2.67E-09	Arrdc4
ENSMUSG00000042660.9	258.801513	-0.0031846	0.00203293	2.47147002	0.11592945	0.23567593	Wdr55
ENSMUSG00000042662.17	4.73195498	0.03655424	0.02502959	3.67429179	0.05525772	0.13105165	Dusp15
ENSMUSG00000042670.6	1004.50856	-0.018162	0.00444977	16.6426256	4.51E-05	0.00028023	Immp1l

ENSMUSG00000042671.13	6.55847844	0.04701451	0.01292973	11.4490146	0.00071532	0.00327412	Rgs8
ENSMUSG00000042672.16	1.64514879	0.04371017	0.01439028	9.41809595	0.00214855	0.00859345	Dcst1
ENSMUSG00000042675.16	697.703538	-0.0113825	0.00461269	5.89491016	0.0151847	0.04532001	Ypel3
ENSMUSG00000042677.8	64.4789355	0.00998413	0.00463143	4.48649746	0.0341636	0.0885281	Zc3h12a
ENSMUSG00000042678.18	8.69247676	0.00897839	0.0128314	0.63840416	0.42428926	0.61594673	Myo15a
ENSMUSG00000042680.9	523.665432	-0.0080784	0.00486707	2.65633472	0.10313815	0.21508111	Garem1
ENSMUSG00000042682.11	2124.86432	-0.004476	0.0028722	2.40322839	0.12108514	0.24400693	Selenok
ENSMUSG00000042684.9	29.5193144	0.02097857	0.00542472	14.402491	0.00014761	0.00081153	Npl
ENSMUSG00000042686.6	42.6998261	-0.0015092	0.00599581	0.05729529	0.81082308	0.92218936	Jph1
ENSMUSG00000042688.17	682.796291	0.00262971	0.00227334	1.33533156	0.24785894	0.41912825	Mapk6
ENSMUSG00000042694.18	52.9286779	0.00496871	0.00426893	1.32727928	0.24928981	0.42079364	Stn1
ENSMUSG00000042699.12	928.895452	0.00069161	0.00272903	0.06415792	0.80004092	0.91588202	Dhx9
ENSMUSG00000042700.17	512.657376	-0.0008999	0.00350409	0.06569901	0.79770515	0.91506608	Sipa1l1
ENSMUSG00000042705.10	180.43619	0.01605124	0.0027891	33.0389703	9.03E-09	1.10E-07	Commd10
ENSMUSG00000042707.7	2.77922467	-0.0261683	0.01198506	4.8388628	0.02782524	0.07479044	Dnali1
ENSMUSG00000042708.13	2.91516671	0.0143903	0.01685316	1.15398844	0.28271578	0.46274354	Shcbp1l
ENSMUSG00000042709.17	335.664778	-0.0084656	0.00323196	6.76920941	0.00927439	0.02998571	Atpaf2
ENSMUSG00000042712.11	385.562922	-0.0010003	0.00409279	0.06043329	0.80581241	0.91899963	Tceal9
ENSMUSG00000042717.6	57.9274983	0.07716057	0.01227219	28.8654262	7.76E-08	8.20E-07	Ppp1r3a
ENSMUSG00000042719.18	434.927617	-0.0088679	0.00286767	9.51279446	0.00204044	0.00822514	Naa25
ENSMUSG00000042724.9	4.65181448	0.0496524	0.00856653	33.7900041	6.14E-09	7.66E-08	Map3k9
ENSMUSG00000042726.15	835.631508	0.00885087	0.00243645	13.3541224	0.00025786	0.0013346	Trafd1
ENSMUSG00000042729.12	301.48825	-0.0047841	0.00340758	2.01505716	0.1557454	0.29571817	Wdr74
ENSMUSG00000042734.7	3.68977988	0.00872072	0.01223495	0.48154446	0.48772357	0.67607688	Ttc9
ENSMUSG00000042737.10	461.618698	-0.0113833	0.00471944	5.93590633	0.01483544	0.04448934	Dpm3
ENSMUSG00000042742.8	266.298931	0.00314987	0.00351928	0.77060374	0.38003035	0.57147239	Bmt2
ENSMUSG00000042743.14	55.8972969	-0.0009612	0.00504555	0.03550948	0.85053207	0.94795622	Sgtb
ENSMUSG00000042744.17	569.773859	-0.0079481	0.00459847	2.95772788	0.08546797	0.18574103	Hectd4
ENSMUSG00000042745.10	196.289229	0.02344359	0.00642733	12.2790926	0.00045806	0.00221118	Id1
ENSMUSG00000042747.13	590.945323	-0.0053164	0.00300396	3.17380533	0.07482792	0.16705075	Krtcap2
ENSMUSG00000042750.8	12.0902076	0.01840997	0.01406781	1.85639363	0.1730416	0.32058434	Bex2
ENSMUSG00000042751.15	14.6636753	0.02931258	0.00981682	8.40304202	0.00374594	0.01380495	Nmnat2
ENSMUSG00000042757.17	12.5739817	0.04638197	0.00646729	48.0551636	4.14E-12	8.05E-11	Tmem108
ENSMUSG00000042759.13	48.2316349	0.03944056	0.0061095	40.5125032	1.95E-10	3.00E-09	Apobr
ENSMUSG00000042763.10	2.18423992	0.03403278	0.01509028	5.9620682	0.01461689	0.04397371	Maneal
ENSMUSG00000042766.18	7.55268799	0.03859684	0.00939145	16.3765254	5.19E-05	0.00031678	Trim46
ENSMUSG00000042770.9	4056.22315	-0.0036598	0.0037807	0.94764551	0.33031934	0.51715313	Hebp1
ENSMUSG00000042772.16	740.912162	-0.0002942	0.00275727	0.01122269	0.91563218	0.9838322	Smg7
ENSMUSG00000042784.10	22.181919	0.02821076	0.00698002	16.7758792	4.21E-05	0.00026338	Muc1
ENSMUSG00000042787.16	41.6582804	-0.0018055	0.00366457	0.24254527	0.6223738	0.79231977	Exog
ENSMUSG00000042790.17	1436.18627	-0.004192	0.00429775	0.93613876	0.33327284	0.52015439	Rnf214
ENSMUSG00000042793.14	11.7312444	0.01652014	0.0129727	1.60663551	0.20496539	0.36515701	Lgr6
ENSMUSG00000042797.10	617.88785	-0.0076786	0.00310802	6.25183118	0.0124065	0.03818285	Aqp11
ENSMUSG00000042804.14	29.6851487	0.02343209	0.00706801	10.7528868	0.00104117	0.00455349	Gpr153
ENSMUSG00000042807.16	95.3403965	-0.0002829	0.00588728	0.00221348	0.96247526	1	Hecw2
ENSMUSG00000042808.11	3.74209465	0.06022746	0.01371297	18.5260468	1.68E-05	0.00011385	Gpx2
ENSMUSG00000042810.15	220.920979	-0.0006634	0.00323392	0.04222948	0.83718296	0.93874262	Krba1
ENSMUSG00000042812.6	50.0951482	0.02674016	0.00338539	60.1838198	8.64E-15	2.39E-13	Foxf1
ENSMUSG00000042814.9	73.8538523	-0.0093896	0.00287147	10.67024	0.00108873	0.00473167	Mcts2
ENSMUSG00000042817.16	12.7948282	0.07216672	0.00704596	98.6293928	3.04E-23	2.31E-21	Flt3
ENSMUSG00000042821.8	18.1313249	0.01395966	0.00600618	5.37068408	0.02047794	0.05812188	Snai1
ENSMUSG00000042826.14	10.1246071	4.29E-05	0.0072637	3.37E-05	0.9953708	1	Fgf11
ENSMUSG00000042828.13	41.8378853	-0.0123831	0.010854	0.21487476	0.64297316	0.80872253	Trim72
ENSMUSG00000042831.14	139.873564	-0.0087515	0.00330362	7.15338573	0.00748226	0.0249578	Alkbh6
ENSMUSG00000042834.16	402.669747	-0.0482798	0.00596676	65.1636546	6.89E-16	2.16E-14	Nrep
ENSMUSG00000042842.12	106.292468	0.03339627	0.00571355	32.4645893	1.21E-08	1.45E-07	Serpinb6b
ENSMUSG00000042845.8	3.38718455	0.03590744	0.01625538	4.47565421	0.03438104	0.08903285	Wfdc12
ENSMUSG00000042846.9	28.5975094	0.03820438	0.01437795	6.24167846	0.01247782	0.0383605	Lrrtm3
ENSMUSG00000042848.5	0.95218489	-0.0079796	0.03369323	0.13425535	0.71406011	0.85918895	Vmn1r226
ENSMUSG00000042851.18	119.567238	0.03740442	0.00627995	33.4864458	7.18E-09	8.87E-08	Zc3h6
ENSMUSG00000042854.10	28.9456572	0.00383016	0.00552143	0.4828104	0.48715207	0.67565382	Trp53rkb

ENSMUSG00000042870.16	432.389194	0.00290599	0.0029247	1.00212885	0.31679594	0.5022603	Tom1
ENSMUSG00000042873.12	14.2836194	0.07720984	0.01418258	26.6822678	2.40E-07	2.33E-06	Lhfpl4
ENSMUSG00000042888.11	1.82955148	0.03184362	0.06508325	-0.3164406	1	1	Prr30
ENSMUSG00000042895.7	19.059746	0.02537259	0.01980426	-4.8834384	1	1	Abra
ENSMUSG00000042901.11	259.321278	0.02184543	0.00354458	37.2482645	1.04E-09	1.44E-08	Aida
ENSMUSG00000042903.9	364.318173	-0.0096107	0.00288989	11.0214417	0.00090064	0.00399976	Foxo4
ENSMUSG00000042918.18	6.54222994	-0.0225053	0.00965901	5.16144523	0.02309367	0.06410349	Mamstr
ENSMUSG00000042942.19	25.2850028	0.00866989	0.00959078	0.82768098	0.36294396	0.55252421	Greb1l
ENSMUSG00000042961.14	16.5601716	-0.0112728	0.007346	2.28262254	0.13083048	0.25911225	Egflam
ENSMUSG00000042978.12	555.476178	-0.0152108	0.0054447	7.90470244	0.00493065	0.01747208	Sbk1
ENSMUSG00000042985.8	40.2649563	-0.0115951	0.00717878	2.53672946	0.11122496	0.22821678	Upk3b
ENSMUSG00000042988.11	483.961936	-0.0135063	0.00457326	8.88228165	0.00287951	0.01103432	Notum
ENSMUSG00000042992.16	136.082506	-0.0065597	0.00284177	5.40347212	0.02009673	0.05718441	Borcs5
ENSMUSG00000042993.7	0.91239327	-0.0058434	0.02998203	0.07620379	0.7825094	0.90508382	Ifnk
ENSMUSG00000042997.14	334.481625	0.0034543	0.00223992	2.35838024	0.12461152	0.24937105	Nhlrc3
ENSMUSG00000043003.15	4.37592099	0.01696142	0.00964535	2.97040535	0.0848006	0.18464799	Rasef
ENSMUSG00000043004.14	88.8818774	0.07066633	0.00742242	80.6489566	2.70E-19	1.28E-17	Gng2
ENSMUSG00000043008.10	66.3214056	0.03063807	0.00448277	43.5597531	4.11E-11	6.94E-10	Klhl6
ENSMUSG00000043013.11	726.780705	-0.043559	0.00858351	23.0740064	1.56E-06	1.30E-05	Onecut1
ENSMUSG00000043015.16	22.767332	0.03623961	0.00584845	35.7441606	2.25E-09	2.98E-08	Nemp2
ENSMUSG00000043017.10	8.27607477	0.04358135	0.0082309	25.257485	5.02E-07	4.56E-06	Ptgir
ENSMUSG00000043019.13	711.039067	-0.0045893	0.00302693	2.29366444	0.12990291	0.25766892	Edem3
ENSMUSG00000043020.14	3.12301219	-0.001466	0.01445368	0.00463821	0.94570252	1	Dnai3
ENSMUSG00000043029.3	2.53796143	-0.0034649	0.00855491	0.16336958	0.68607299	0.8400414	Trpv3
ENSMUSG00000043036.15	1.82222625	-0.0042469	0.01965706	-0.0913511	1	1	Ccdc63
ENSMUSG00000043050.9	5.95449169	-0.031268	0.04763994	0.976704	0.32301389	0.50953756	Tnp2
ENSMUSG00000043051.19	3.74206307	0.02370326	0.00998456	5.29089565	0.02143719	0.06026432	Disc1
ENSMUSG00000043059.17	148.884153	0.0073482	0.00312377	5.55426547	0.01843571	0.05320908	Zfp513
ENSMUSG00000043061.11	314.691957	-0.0034524	0.00219099	2.47084725	0.11597539	0.23570837	Tmem18
ENSMUSG00000043065.13	55.1517519	0.00343661	0.0047624	0.5148343	0.47305358	0.66373835	Spice1
ENSMUSG00000043067.16	1088.43443	-0.0070891	0.00285117	6.0118552	0.01421008	0.04293057	Dpy19l1
ENSMUSG00000043068.7	94.2505891	-0.0150609	0.00546141	7.08415645	0.00777684	0.02576912	Fam89a
ENSMUSG00000043079.18	183.288814	0.02317444	0.00467717	23.8531507	1.04E-06	8.92E-06	Synpo
ENSMUSG00000043085.15	343.204647	-0.0061019	0.00291346	4.36483099	0.03668812	0.09376163	Tmem82
ENSMUSG00000043088.17	10.5206927	0.01854885	0.00689906	7.29543774	0.00691299	0.02338973	Il17re
ENSMUSG00000043090.19	202.956463	0.00066788	0.00207577	0.10255109	0.74878944	0.88247378	Zfp866
ENSMUSG00000043091.10	2226.07534	0.00036737	0.00369843	0.00980685	0.92111481	0.98715985	Tuba1c
ENSMUSG00000043099.6	61.4402622	0.02201459	0.00426583	25.6866373	4.02E-07	3.73E-06	Hic1
ENSMUSG00000043102.3	1.57249709	0.04332186	0.01451958	8.67210573	0.00323118	0.01216643	Qrfp
ENSMUSG00000043110.3	12.7326646	0.00897558	0.00717592	1.51494812	0.2183856	0.38284842	Lrrn4
ENSMUSG00000043122.8	6.43731255	0.07725279	0.0126666	34.3238652	4.67E-09	5.93E-08	A530016L24Rik
ENSMUSG00000043131.15	997.904953	0.00758722	0.00258172	8.40836222	0.00373499	0.01376891	Mob1a
ENSMUSG00000043140.11	234.818115	-0.0090741	0.00178838	25.7682505	3.85E-07	3.59E-06	Tmem186
ENSMUSG00000043144.7	1.83910764	0.00275095	0.05106329	0.01677448	0.89694911	0.97391776	Aqp6
ENSMUSG00000043153.7	60.6572156	0.00936106	0.00309073	8.92603331	0.00281134	0.01081702	Crppa
ENSMUSG00000043154.16	461.163546	-0.0009112	0.00336298	0.0732346	0.78668415	0.90803231	Ppp2r3a
ENSMUSG00000043155.5	2.75269185	-0.0101364	0.00988016	1.02884353	0.31043044	0.49566267	Hpdl
ENSMUSG00000043157.9	12.4285229	0.08187858	0.00816401	93.4033601	4.27E-22	2.88E-20	Arl11
ENSMUSG00000043162.8	647.250511	-0.015045	0.00240704	38.7092766	4.92E-10	7.13E-09	Pyurf
ENSMUSG00000043165.8	9.4909364	-0.0043955	0.00670875	0.41366583	0.52011472	0.70599826	Lor
ENSMUSG00000043183.19	174.647438	-0.0115109	0.0050133	5.27363514	0.02165076	0.06074161	Simc1
ENSMUSG00000043190.15	184.43867	-0.0078814	0.00331575	5.51972084	0.01880326	0.05409119	Rfesd
ENSMUSG00000043207.11	1248.6929	-0.0027479	0.00259649	1.09796687	0.29471273	0.4773584	Zmpste24
ENSMUSG00000043230.3	1.75890903	0.02660333	0.02228741	1.93387444	0.16433518	0.30786415	Fam124b
ENSMUSG00000043241.15	834.686056	-0.0018041	0.00370774	0.23341105	0.62900604	0.79765764	Upf2
ENSMUSG00000043243.16	11.0481981	0.03972671	0.00885272	18.5732728	1.63E-05	0.00011136	Niban3
ENSMUSG00000043251.7	55.7867473	-0.0019382	0.00402027	0.23577744	0.62727265	0.79593569	Exoc3l
ENSMUSG00000043252.9	920.281433	-0.0008247	0.00267614	0.09243737	0.76110111	0.8911945	Tmem64
ENSMUSG00000043257.16	147.438572	0.00426467	0.00255903	2.78457015	0.09517623	0.20223662	Pigv
ENSMUSG00000043259.16	15.2473079	0.02543443	0.00727307	11.9620078	0.00054296	0.00257413	Fam13c
ENSMUSG00000043262.17	251.56883	0.00735188	0.00286656	6.4523219	0.01108074	0.03480032	Uevld

ENSMUSG00000043263.14	68.7916552	0.04596163	0.00578197	60.1857368	8.63E-15	2.39E-13	Ifi209
ENSMUSG00000043279.10	568.942974	0.0140387	0.00376122	13.7861318	0.00020484	0.00108914	Trim56
ENSMUSG00000043282.4	3.49327797	0.0272262	0.00824302	11.1796913	0.00082698	0.00371383	Teddm1b
ENSMUSG00000043284.13	442.995677	-0.0096505	0.00208566	21.4287116	3.67E-06	2.86E-05	Tmem11
ENSMUSG00000043286.13	1.77994756	0.03884103	0.01347227	9.16375691	0.00246855	0.00966041	Pnpla1
ENSMUSG00000043290.7	44.6338872	0.00371633	0.00315175	1.398533	0.23696935	0.40600994	Zfp784
ENSMUSG00000043298.9	0.84339447	0.02666954	0.0253525	1.46813738	0.2256398	0.39169456	Smco3
ENSMUSG00000043300.3	28.6281694	0.03375931	0.00631296	26.864167	2.18E-07	2.14E-06	B3galnt1
ENSMUSG00000043301.17	1.98704746	0.01449029	0.02704373	0.4240045	0.51494561	0.70148476	Kcnj6
ENSMUSG00000043308.3	2.59477084	0.00711511	0.02515381	0.33307934	0.56385146	0.74449983	Vmn1r75
ENSMUSG00000043311.8	140.655616	-0.0003675	0.00325048	0.01243013	0.91122742	0.9815139	D17H6S53E
ENSMUSG00000043313.7	5.52768149	0.01543426	0.00825186	3.50745209	0.06109334	0.14202125	Pcdhb19
ENSMUSG00000043319.6	3.53185392	-0.3917084	0.16399409	0.1321066	0.71625776	0.86050603	Cox8c
ENSMUSG00000043323.18	340.81508	-0.0074422	0.00296787	6.27107486	0.01227247	0.03785255	Fbrsl1
ENSMUSG00000043336.15	203.626381	0.04213036	0.00541202	55.788674	8.07E-14	1.94E-12	Filip1l
ENSMUSG00000043354.3	2.38847614	-0.0179497	0.03920991	0.34407309	0.55748754	0.73940849	Or51g2
ENSMUSG00000043366.17	1.03690991	0.01347122	0.02310663	0.25538251	0.61331033	0.78523165	Or51e2
ENSMUSG00000043372.13	24.0934412	0.00197871	0.00424892	0.21179921	0.64536084	0.81029975	Hexim2
ENSMUSG00000043384.16	197.330492	-1.70E-05	0.00365873	2.10E-05	0.99634554	1	Gprasp1
ENSMUSG00000043388.9	10.2362927	0.01943136	0.01565538	2.00342297	0.15694444	0.29727824	Tmem130
ENSMUSG00000043391.11	212.444476	0.02257306	0.00396099	30.5930372	3.18E-08	3.56E-07	2510009E07Rik
ENSMUSG00000043398.6	33.6791783	0.01147086	0.00469067	5.90910038	0.01506286	0.04502822	Gpr135
ENSMUSG00000043411.16	883.286294	-0.0056547	0.00375312	2.28765314	0.13040697	0.25846196	Usp48
ENSMUSG00000043415.6	121.627568	0.01206589	0.00287958	16.9431529	3.85E-05	0.00024259	Otud1
ENSMUSG00000043418.5	80.0771669	-0.010873	0.00530706	4.02353609	0.04486954	0.11039986	Lrit2
ENSMUSG00000043419.12	22.9397908	0.00914542	0.00677844	1.68349449	0.19446076	0.35025213	Rnf227
ENSMUSG00000043421.9	60.9001011	0.01174343	0.00521054	4.83312272	0.02791802	0.07498855	Hilpda
ENSMUSG00000043424.11	413.936505	-0.0121411	0.0041838	8.44148457	0.00366757	0.0135585	Eif3j2
ENSMUSG00000043432.8	83.389903	-0.0027216	0.00394613	0.45618107	0.49941418	0.68667267	Leng9
ENSMUSG00000043439.6	56.1507868	-0.0100994	0.00560462	3.09872481	0.07835365	0.17329793	Epop
ENSMUSG00000043445.8	466.617782	-0.0059189	0.00265036	4.98429836	0.02557836	0.06980362	Pgp
ENSMUSG00000043448.14	14.5493711	0.01604776	0.00472142	11.191294	0.00082182	0.0036949	Gjc2
ENSMUSG00000043456.18	4.44336403	0.0329564	0.01941411	3.57176811	0.0587697	0.13756166	Zfp536
ENSMUSG00000043458.6	4.65586318	0.0455583	0.00770123	35.4385152	2.63E-09	3.45E-08	Pcdhb12
ENSMUSG00000043460.8	5.71600891	0.03749665	0.0166937	5.18104074	0.02283464	0.06353402	Elfn2
ENSMUSG00000043461.14	0.97192767	-0.0009909	0.02093003	0.01199402	0.91279228	0.98234708	Sptssb
ENSMUSG00000043463.7	3.43773947	0.00535158	0.02583417	0.10913918	0.7411263	0.87738961	Rab9b
ENSMUSG00000043467.20	164.84176	0.00642577	0.0035289	3.28494116	0.06991813	0.15801927	Zbtb37
ENSMUSG00000043487.5	22.858763	0.01193552	0.00527829	5.11174846	0.02376436	0.06567383	Acot6
ENSMUSG00000043496.8	14.3590024	0.01640656	0.00659679	6.14313843	0.0131923	0.0402163	Tril
ENSMUSG00000043501.6	2.14123709	0.04099625	0.01199384	11.6016909	0.00065892	0.00304928	Lgals2
ENSMUSG00000043505.9	36.2005922	0.03107814	0.00576384	26.5583559	2.56E-07	2.46E-06	Gimap5
ENSMUSG00000043510.13	77.3797685	0.00673685	0.00419911	2.58197738	0.10808656	0.2230775	Hscb
ENSMUSG00000043518.8	67.5887505	0.0094892	0.00362472	6.4085494	0.01135722	0.03550315	Rai2
ENSMUSG00000043531.18	2.47237308	0.02494513	0.01978038	2.14575216	0.14296495	0.27723188	Sorcs1
ENSMUSG00000043535.14	576.241911	0.00799604	0.00380286	4.30647991	0.03796745	0.09633178	Setx
ENSMUSG00000043541.17	5.03209044	0.01691626	0.01053856	2.35401531	0.12496077	0.24996393	Dnai7
ENSMUSG00000043542.13	35.7475129	0.01341369	0.00584738	4.96218337	0.02590748	0.07054369	Zc2hc1a
ENSMUSG00000043556.11	26.7972399	0.0030507	0.00469363	0.41322188	0.52033872	0.70612958	Fbxl7
ENSMUSG00000043557.17	8.15022594	0.02414958	0.01179895	3.79826896	0.0513056	0.12311464	Mdga1
ENSMUSG00000043572.8	54.7029772	0.00448111	0.00398106	1.24847673	0.26384361	0.43882769	Pars2
ENSMUSG00000043587.16	125.60525	0.01183311	0.00323746	13.0735396	0.0002995	0.0015224	Pxylp1
ENSMUSG00000043592.16	2.94754717	0.06967347	0.01153229	32.3692115	1.27E-08	1.52E-07	Unc5cl
ENSMUSG00000043602.6	32.0646294	-0.0016773	0.00324702	0.26861121	0.60426491	0.77736403	Zfp3
ENSMUSG00000043613.9	7.40568195	0.0593199	0.01359276	15.0390357	0.00010531	0.00059909	Mmp3
ENSMUSG00000043614.14	7.50294449	0.01415402	0.00780756	3.369583	0.06641056	0.15180844	Vps37d
ENSMUSG00000043621.14	4.67857653	0.03460398	0.00966888	12.0667926	0.00051328	0.00245065	Ubxn10
ENSMUSG00000043629.13	6.26486381	0.03496977	0.01249923	6.95382257	0.00836403	0.02741822	1700019D03Rik
ENSMUSG00000043631.9	34.5651824	0.00145226	0.0057328	0.0619321	0.80346784	0.91788203	Ecm2
ENSMUSG00000043633.7	56.9986223	-0.0144952	0.00726526	3.87295409	0.04907008	0.11875302	Fam221b
ENSMUSG00000043635.13	5.26854257	0.01398914	0.00927852	2.09253939	0.14801985	0.28455426	Adamts3

ENSMUSG00000043639.16	90.4735552	-0.0036406	0.00525827	0.46556115	0.49503637	0.68232397	Rbm20
ENSMUSG00000043648.8	25.4490434	0.00279149	0.00506025	0.301445	0.58297795	0.7603892	Pld6
ENSMUSG00000043664.8	7.24541697	0.03683443	0.00693272	27.9502914	1.24E-07	1.27E-06	Tmem221
ENSMUSG00000043668.14	4.81245608	0.0345638	0.01028154	11.2196036	0.00080938	0.00365084	Tox3
ENSMUSG00000043670.5	4.72734907	0.01584677	0.02348179	0.74462921	0.388182	0.57976144	Diras1
ENSMUSG00000043671.15	370.95236	0.03379685	0.0061662	25.27136	4.98E-07	4.54E-06	Dpy19l3
ENSMUSG00000043673.12	4.77522373	0.01012626	0.00703687	2.03167941	0.1540503	0.29327874	Kcns3
ENSMUSG00000043681.6	361.309148	-0.0128145	0.00661385	3.63445228	0.05659522	0.13369941	Fam25a
ENSMUSG00000043683.5	625.889615	-0.0109392	0.00262379	17.3645375	3.09E-05	0.00019753	Fem1a
ENSMUSG00000043687.17	87.1757234	-0.0102016	0.00453933	5.09169464	0.02404069	0.06627205	1190005I06Rik
ENSMUSG00000043702.10	235.453606	-0.0080548	0.002358	11.4779626	0.00070426	0.00322789	Pde12
ENSMUSG00000043716.14	6596.19062	0.0016288	0.00298045	0.30222684	0.58248975	0.76011009	Rpl7
ENSMUSG00000043719.15	18.6324418	0.02391075	0.00690172	11.404699	0.00073259	0.00334083	Col6a6
ENSMUSG00000043733.15	1246.78566	-0.0066989	0.00310297	4.52999547	0.03330571	0.08663818	Ptpn11
ENSMUSG00000043740.16	76.245125	0.05491608	0.00607083	74.4891269	6.10E-18	2.45E-16	B430306N03Rik
ENSMUSG00000043753.3	45.83156	0.11650257	0.00742831	184.406444	5.29E-42	4.62E-39	Dmrta1
ENSMUSG00000043760.17	159.818258	0.00595914	0.0051655	1.33858053	0.24728445	0.41863703	Pkhd1
ENSMUSG00000043782.10	2.26428859	0.00067541	0.00979829	0.00670251	0.934751	0.99434035	Bicdl2
ENSMUSG00000043789.9	576.810124	0.00188472	0.00439507	0.18314583	0.66868359	0.82724732	Vwce
ENSMUSG00000043795.11	2.26144449	0.01241839	0.02064441	0.64540779	0.42175932	0.61344445	Prr33
ENSMUSG00000043807.7	3.03960505	0.02087807	0.00737893	8.0082069	0.00465658	0.0166233	Ly6g5b
ENSMUSG00000043811.6	2.09930472	0.02274458	0.02153234	1.39843696	0.23698545	0.40600994	Rtn4r
ENSMUSG00000043822.19	30.427207	0.01183132	0.00807632	1.98958663	0.15838411	0.29933073	Adamtsl5
ENSMUSG00000043831.13	181.508129	-0.0081715	0.00237591	11.7947606	0.00059398	0.00277857	Lysmd4
ENSMUSG00000043832.14	74.0917437	0.04775913	0.00638495	50.9082096	9.68E-13	2.03E-11	Clec4a3
ENSMUSG00000043857.17	1.95516346	0.00103321	0.03166802	0.02354236	0.8780551	0.96289089	Mgat5b
ENSMUSG00000043866.12	885.021903	-0.0064433	0.00349812	3.39947736	0.06521708	0.14966044	Taf10
ENSMUSG00000043872.15	79.1388385	-0.0046036	0.00462256	0.95407997	0.32868295	0.51575517	Zmym1
ENSMUSG00000043881.10	261.024285	-0.0022082	0.00310588	0.48715505	0.48519912	0.6741773	Kbtbd7
ENSMUSG00000043885.15	211.791729	0.01251688	0.00357847	11.8086344	0.00058957	0.00276122	Slc36a4
ENSMUSG00000043895.7	125.57864	0.01248843	0.00372592	10.6063658	0.00112699	0.00487728	S1pr2
ENSMUSG00000043897.14	0.7561547	-0.0042398	0.04558469	0.0107724	0.91733585	0.98479095	Vmn2r2
ENSMUSG00000043903.5	6.01397437	0.02299997	0.013965	2.74774318	0.09739181	0.20594035	Zfp469
ENSMUSG00000043909.17	201.585357	0.00223802	0.00398126	0.31417316	0.57513059	0.75386306	Trp53bp1
ENSMUSG00000043913.15	1.87693137	-0.0135553	0.02699227	0.24147533	0.62314258	0.79278498	Ccdc60
ENSMUSG00000043923.9	276.739619	-0.0111432	0.0037047	9.07951336	0.00258489	0.01006108	Cenatac
ENSMUSG00000043924.17	14.190633	0.02559058	0.00754335	10.402316	0.00125857	0.00538157	Ncmap
ENSMUSG00000043929.17	103.395962	-0.0035661	0.00362932	0.93981342	0.33232583	0.51929489	Klhl15
ENSMUSG00000043931.11	19.6485073	0.0906481	0.0077891	113.043196	2.11E-26	2.43E-24	Gimap7
ENSMUSG00000043932.6	3.80026341	0.05572752	0.0099231	28.8715198	7.73E-08	8.18E-07	Klri2
ENSMUSG00000043939.17	7.77801082	0.08178949	0.00878262	78.5359294	7.86E-19	3.52E-17	A530064D06Rik
ENSMUSG00000043940.16	1057.59726	0.0078696	0.0037128	4.42479153	0.03542044	0.09113412	Wdfy3
ENSMUSG00000043943.15	33.4132435	0.00571799	0.0052601	1.11979376	0.28996287	0.47139952	Naalad2
ENSMUSG00000043953.13	36.7673847	0.02237973	0.00498153	19.2823626	1.13E-05	7.90E-05	Ccrl2
ENSMUSG00000043962.17	1584.90696	0.00198223	0.00250253	0.62977696	0.42743714	0.61876735	Thrap3
ENSMUSG00000043964.15	351.875381	-0.0027467	0.00212607	1.67096402	0.19612946	0.35279969	Orai3
ENSMUSG00000043969.5	6.09049424	-0.0086416	0.03614977	0.05906887	0.80797373	0.92067471	Emx2
ENSMUSG00000043987.19	242.520158	-0.0037389	0.004566	0.65934837	0.41679009	0.60805412	Cep164
ENSMUSG00000043991.6	748.330734	-0.0031717	0.0031105	1.01387426	0.31397647	0.4993223	Pura
ENSMUSG00000043998.11	973.540538	-0.0009529	0.0024042	0.15816052	0.69085576	0.84318365	Mgat2
ENSMUSG00000044005.14	5774.2859	-0.0024416	0.00517619	0.22013413	0.63893774	0.80536477	Gls2
ENSMUSG00000044006.9	1.54136399	-0.0386862	0.02027361	3.74130384	0.05308301	0.12660692	Cilp2
ENSMUSG00000044014.10	0.71186292	0.00112861	0.03659859	0.00981198	0.92109425	0.98715985	Npy5r
ENSMUSG00000044017.17	21.7843809	0.0191679	0.00532047	12.7864763	0.00034913	0.0017439	Adgrd1
ENSMUSG00000044018.4	1331.37759	-0.0089606	0.00217683	16.8193013	4.11E-05	0.0002579	Mrpl50
ENSMUSG00000044022.6	3.11341179	0.03408447	0.01067576	10.2279134	0.00138331	0.00582725	Pcdhb21
ENSMUSG00000044024.17	7.91993727	0.03188926	0.0084929	12.5214098	0.00040232	0.00197441	Rell2
ENSMUSG00000044026.4	577.943955	-0.0114655	0.00362442	10.317223	0.00131794	0.00558682	Slc35g1
ENSMUSG00000044030.5	252.092643	-0.0023338	0.00253294	0.84216396	0.35877861	0.54774758	Irf2bp1
ENSMUSG00000044033.17	284.549311	-0.0058728	0.00438267	1.83572007	0.17545341	0.32398373	Ccdc141
ENSMUSG00000044034.12	3.25093916	-0.0258073	0.00791206	11.2113246	0.000813	0.00366388	Npb

ENSMUSG00000044037.16	554.763885	-0.0093561	0.00392434	5.55292643	0.01844982	0.05324328	Als2cl
ENSMUSG00000044042.20	264.476993	0.04315426	0.00498398	71.6288984	2.60E-17	9.75E-16	Fmn1
ENSMUSG00000044043.5	12.3361952	0.02665316	0.00579725	20.0858081	7.40E-06	5.40E-05	Pcdhb14
ENSMUSG00000044052.4	0.96081473	0.04093455	0.01580398	6.63828385	0.009981	0.03185116	Ccr10
ENSMUSG00000044066.15	254.497984	-0.0077577	0.00248404	9.70883142	0.00183385	0.00748582	Cep68
ENSMUSG00000044067.8	15.7838508	-0.0129249	0.01623026	0.55334055	0.45695595	0.64879383	Gpr22
ENSMUSG00000044071.10	70.48444	0.10597145	0.00839598	125.41505	4.13E-29	7.11E-27	Tafa2
ENSMUSG00000044072.15	37.9387127	0.04405529	0.00703778	33.8404257	5.98E-09	7.47E-08	Eml6
ENSMUSG00000044080.10	2404.33671	-0.0069348	0.00393438	3.11012592	0.07780694	0.17234735	S100a1
ENSMUSG00000044086.9	82.7943979	-0.004437	0.01162859	0.09780128	0.75448431	0.88645993	Lmod3
ENSMUSG00000044092.9	11.5639766	0.04301607	0.00647864	41.7189201	1.05E-10	1.68E-09	C130050O18Rik
ENSMUSG00000044098.15	213.177745	0.00439581	0.00347802	1.55678445	0.21213666	0.37442605	Rsbn1
ENSMUSG00000044103.5	3.90926504	0.06535019	0.0110402	30.925912	2.68E-08	3.03E-07	Il36g
ENSMUSG00000044106.6	0.99906414	-0.00781	0.0334729	0.00490777	0.94414951	0.99922419	Or7e174
ENSMUSG00000044117.13	6.24249414	0.01479429	0.01142184	1.62041198	0.20303435	0.36245597	Bmerb1
ENSMUSG00000044122.16	81.7442756	-0.0048994	0.00518595	0.78868628	0.37449766	0.56541428	Proca1
ENSMUSG00000044134.10	122.079326	-0.0038978	0.00345181	1.29131146	0.25580641	0.42873939	Pheta1
ENSMUSG00000044139.13	75.1400703	-0.0290372	0.00519579	30.0719656	4.16E-08	4.56E-07	Prss53
ENSMUSG00000044145.3	37.2983391	-0.0136588	0.00507229	7.20059479	0.00728794	0.02444445	1810024B03Rik
ENSMUSG00000044147.9	485.778501	0.00315477	0.00239836	1.69763677	0.19259733	0.34779924	Arf6
ENSMUSG00000044148.7	155.04965	0.01520697	0.00237697	40.0839061	2.43E-10	3.69E-09	1810030O07Rik
ENSMUSG00000044149.8	70.6164345	-0.0024577	0.00301132	0.65209865	0.41936336	0.61074189	Nkrf
ENSMUSG00000044150.13	62.2715119	0.007189	0.00407846	2.95657776	0.08552879	0.18583897	Bclaf3
ENSMUSG00000044155.12	125.291211	0.00291914	0.00335181	0.75107042	0.38613754	0.57766578	Lsm8
ENSMUSG00000044156.15	6.29250089	0.06154967	0.0090078	41.5355693	1.16E-10	1.84E-09	Hepacam2
ENSMUSG00000044162.13	9.51946829	0.10914296	0.01322766	54.0727715	1.93E-13	4.47E-12	Tnip3
ENSMUSG00000044164.3	2.00087517	0.01475763	0.023601	0.5258346	0.46836336	0.65951154	Rnf182
ENSMUSG00000044165.13	1.92685133	0.04804552	0.01361311	12.1216097	0.00049841	0.00238884	Bcl2l15
ENSMUSG00000044167.7	759.3909	-0.0057912	0.00349949	2.779544	0.09547535	0.20274436	Foxo1
ENSMUSG00000044176.13	2.97162352	0.01997021	0.01810465	1.74917363	0.18598065	0.33885977	Spink10
ENSMUSG00000044177.5	2.46936109	0.03119788	0.01226102	6.19859132	0.0127852	0.03915164	Wfikkn2
ENSMUSG00000044186.11	5.91412147	0.02684398	0.00831389	10.4279453	0.00124123	0.005318	Nkx2-6
ENSMUSG00000044197.9	1645.36243	-0.0123284	0.00333594	13.1451061	0.00028827	0.00147328	Gpr146
ENSMUSG00000044199.7	12.0728745	0.0419916	0.00561686	52.15354	5.13E-13	1.12E-11	S1pr4
ENSMUSG00000044201.11	5.85032317	0.02109308	0.01604968	1.23229297	0.26696157	0.4427332	Cdc25c
ENSMUSG00000044205.8	0.8574038	-0.0086011	0.03917835	0.07108775	0.78975955	0.91011232	Or8b57
ENSMUSG00000044206.4	886.978171	0.05639001	0.00610809	76.4687974	2.24E-18	9.47E-17	Vsig4
ENSMUSG00000044217.19	11.961937	0.00341096	0.01753859	0.0294811	0.86367291	0.95506537	Aqp5
ENSMUSG00000044221.15	1768.85983	-0.0124068	0.00262902	21.9772332	2.76E-06	2.20E-05	Grsf1
ENSMUSG00000044224.17	587.512567	-0.0073683	0.00258197	7.99140518	0.00469999	0.01675291	Dnajc21
ENSMUSG00000044229.10	28.7534276	0.03900745	0.00588045	40.8318689	1.66E-10	2.57E-09	Nxpe4
ENSMUSG00000044231.4	26.1429991	0.00287633	0.00410833	0.48327311	0.48694346	0.67549454	Nhlrc1
ENSMUSG00000044244.19	13.6449762	0.00863959	0.00571841	2.2273113	0.13558962	0.26623515	Il20rb
ENSMUSG00000044250.9	50.8215701	0.02705273	0.00454431	34.5617543	4.13E-09	5.28E-08	Pced1b
ENSMUSG00000044252.19	2042.0059	-0.007899	0.00244501	10.3988188	0.00126096	0.00538884	Osbpl1a
ENSMUSG00000044254.7	1187.81934	0.01559642	0.00502552	9.25913995	0.00234323	0.00925288	Pcsk9
ENSMUSG00000044258.11	126.50637	0.03644734	0.00651729	29.5781322	5.37E-08	5.80E-07	Ctla2a
ENSMUSG00000044279.16	195.73729	-0.0079443	0.0033609	5.71240182	0.01684548	0.04940747	Crb3
ENSMUSG00000044288.7	10.5987542	0.04589132	0.01511264	9.22010068	0.00239372	0.00941748	Cnr1
ENSMUSG00000044296.11	10.0385492	0.00871656	0.00527322	2.69282686	0.10080084	0.2114639	Zfp879
ENSMUSG00000044303.7	8.7338689	0.10206252	0.01216797	59.3322466	1.33E-14	3.60E-13	Cdkn2a
ENSMUSG00000044308.19	1986.62304	-0.0051275	0.00237168	4.63210989	0.03137895	0.082518	Ubr3
ENSMUSG00000044309.9	6.69406498	0.04621122	0.0093467	22.2885128	2.35E-06	1.89E-05	Apol7c
ENSMUSG00000044313.14	6.66459412	0.05533437	0.01087127	21.8503492	2.95E-06	2.34E-05	Mab21l3
ENSMUSG00000044317.8	22.8822095	0.00290011	0.0049534	0.33286787	0.56397524	0.74453824	Gpr4
ENSMUSG00000044322.8	1.82848707	0.00662412	0.03039461	0.13978061	0.70849921	0.85578915	Dsc1
ENSMUSG00000044328.14	36.7348079	0.00358414	0.00456951	0.61126687	0.43431121	0.62600935	Trp53i13
ENSMUSG00000044337.6	57.759499	0.00362301	0.00750992	0.22724673	0.6335729	0.80142928	Ackr3
ENSMUSG00000044338.10	52.4730239	0.02818451	0.01785109	1.95733081	0.16179907	0.30411874	Aplnr
ENSMUSG00000044339.13	73.9763679	0.00465888	0.00446935	1.07668312	0.29944075	0.48340928	Alkbh2
ENSMUSG00000044340.8	384.162211	-0.0021468	0.00368624	0.33103398	0.56505088	0.74535875	Phlpp1

ENSMUSG00000044345.10	566.2511	-0.006679	0.00340965	3.87718054	0.04894669	0.11853954	Marveld1
ENSMUSG00000044348.16	41.7959872	0.01128923	0.00635963	2.96874073	0.08488791	0.18480397	Slc25a53
ENSMUSG00000044349.17	431.497918	0.03862148	0.00756477	23.0253817	1.60E-06	1.33E-05	Snhg11
ENSMUSG00000044350.15	232.11533	0.03159204	0.00408799	57.0991824	4.14E-14	1.05E-12	Lacc1
ENSMUSG00000044352.7	9.16145399	0.03182917	0.01163577	6.98063825	0.00823962	0.02708568	Sowaha
ENSMUSG00000044359.2	168.932865	0.0045228	0.00574043	0.62386136	0.42961599	0.62111822	P2ry4
ENSMUSG00000044361.17	105.807037	0.00068073	0.00477587	0.02034361	0.88658159	0.967829	BC024139
ENSMUSG00000044362.9	3.04295423	0.00220888	0.00917154	0.058487	0.80890347	0.92104881	Ccdc89
ENSMUSG00000044365.17	18.0589464	0.00667786	0.0065386	1.01045774	0.3147932	0.50021643	Cxxc4
ENSMUSG00000044367.8	41.6644488	-0.0081079	0.00652692	1.50284159	0.22023465	0.38498036	Slc16a13
ENSMUSG00000044378.13	74.2424922	-0.0230508	0.00713953	9.04726096	0.00263089	0.01021602	Slc15a5
ENSMUSG00000044390.6	3.58506678	0.0102013	0.00740157	1.94855955	0.16274216	0.30546208	Tigd3
ENSMUSG00000044393.16	2467.60456	0.00626623	0.00349127	3.25190083	0.07134068	0.16072607	Dsg2
ENSMUSG00000044408.8	1997.82232	0.0066838	0.00322145	4.29040187	0.03832811	0.09704849	Sptssa
ENSMUSG00000044433.17	272.742603	-0.0041594	0.00451761	0.8357902	0.36060356	0.54996415	Camsap3
ENSMUSG00000044441.6	3.05651423	0.00699355	0.01112237	0.36100152	0.54795054	0.73066268	Or5b94
ENSMUSG00000044442.12	190.264879	0.00164662	0.00283839	0.33415089	0.56322505	0.74388096	N6amt1
ENSMUSG00000044447.6	409.55433	0.00018123	0.00321458	0.00317914	0.95503605	1	Dock5
ENSMUSG00000044452.11	360.2298	0.0083322	0.00494963	2.75334687	0.09705108	0.20538778	Zfp507
ENSMUSG00000044453.6	2.90102345	0.04335432	0.01031503	15.6346745	7.68E-05	0.0004509	Ffar1
ENSMUSG00000044456.17	350.606859	0.00473354	0.00271288	3.01108497	0.08269692	0.18085661	Rin3
ENSMUSG00000044461.7	13.2944148	0.0496028	0.00764138	39.7856728	2.83E-10	4.24E-09	Shisa2
ENSMUSG00000044465.20	379.366086	-0.0068091	0.00323704	4.44472495	0.03500923	0.09032243	Fhip1b
ENSMUSG00000044468.15	225.92487	0.00127144	0.00423953	0.08424764	0.77162159	0.8976752	Tent5c
ENSMUSG00000044469.9	505.720745	-0.0139138	0.00438697	9.57195939	0.00197572	0.00799988	Tnfaip8l1
ENSMUSG00000044475.15	231.37638	-0.003592	0.00243531	2.18530707	0.13933327	0.27177593	Ascc1
ENSMUSG00000044477.13	1147.31004	-0.0005255	0.00180726	0.08453678	0.77124093	0.8974886	Zfand3
ENSMUSG00000044496.7	426.81728	-0.0021927	0.00199542	1.20491532	0.27234147	0.44968609	2510039O18Rik
ENSMUSG00000044499.12	1.22977672	-0.0168811	0.04494507	0.20246093	0.65274175	0.81453403	Hs3st5
ENSMUSG00000044501.18	140.293862	-0.000166	0.00317688	0.00265428	0.95891138	1	Zfp758
ENSMUSG00000044502.17	464.0302	-0.0072104	0.00271626	7.03222772	0.00800557	0.02640831	Bod1
ENSMUSG00000044505.6	26.2400923	-0.012067	0.0072171	2.92783321	0.08706427	0.18853268	Lingo4
ENSMUSG00000044528.6	3.69772869	0.02098259	0.02236189	1.2764907	0.25855255	0.43203209	Tram1l1
ENSMUSG00000044533.16	10014.7276	-0.007503	0.00337299	5.05715703	0.02452444	0.06737721	Rps2
ENSMUSG00000044534.9	49.2107077	0.0019462	0.0053508	0.1225935	0.72623847	0.86777349	Ackr2
ENSMUSG00000044548.12	71.3865303	-0.0228165	0.00422394	29.0940186	6.89E-08	7.33E-07	Dact1
ENSMUSG00000044550.12	6.92233447	0.03897037	0.01426914	7.71099984	0.00548853	0.01916113	Tceal3
ENSMUSG00000044551.14	9.0408758	0.01581722	0.00572143	7.52938322	0.00607007	0.02087555	9930012K11Rik
ENSMUSG00000044562.13	132.132513	0.00022889	0.00303592	0.00565588	0.94005113	0.99698934	Rasip1
ENSMUSG00000044566.16	16.7279767	-0.0018161	0.00517071	0.12177395	0.72711841	0.86818816	Cage1
ENSMUSG00000044573.17	1470.41959	-0.0052172	0.00206599	6.36745687	0.01162321	0.03612875	Acp1
ENSMUSG00000044576.7	1.43875362	0.01712563	0.03534438	0.45434911	0.50027683	0.68733788	Garem2
ENSMUSG00000044581.9	3.00919512	-0.0056338	0.02542323	0.00937587	0.92286215	0.98807551	4932415D10Rik
ENSMUSG00000044583.14	75.8693595	0.05190787	0.00743571	45.0589169	1.91E-11	3.40E-10	Tlr7
ENSMUSG00000044595.11	8.50024792	-0.0236677	0.00568347	17.7116269	2.57E-05	0.00016753	Dnd1
ENSMUSG00000044600.15	1234.32888	-0.0113972	0.00277586	16.9947609	3.75E-05	0.00023664	Smim7
ENSMUSG00000044617.9	183.914627	-0.0006548	0.00286011	0.0520289	0.81956972	0.92756274	Zbtb39
ENSMUSG00000044626.14	3.18682775	0.04960758	0.01244669	13.7995631	0.00020338	0.00108285	Liph
ENSMUSG00000044627.12	1865.93089	-0.0045844	0.0026306	3.07525784	0.07949199	0.17522412	Swi5
ENSMUSG00000044628.6	1.52468708	0.03047546	0.01269354	6.28058784	0.01220676	0.03766959	Rnf208
ENSMUSG00000044629.6	25.0963455	0.01263902	0.00400663	9.85730079	0.00169159	0.00696541	Cnrip1
ENSMUSG00000044636.7	44.8351209	-0.0061446	0.00348821	3.13020513	0.07685407	0.17074709	Csrnp2
ENSMUSG00000044641.8	126.406891	-0.0005394	0.00284801	0.03520072	0.85117569	0.9483632	Pard6b
ENSMUSG00000044646.16	46.4208278	-0.0353322	0.00699449	23.3967665	1.32E-06	1.11E-05	Zbtb7c
ENSMUSG00000044647.17	9.28778258	0.02502167	0.01682889	2.54231789	0.11083198	0.22754901	Csrnp3
ENSMUSG00000044667.13	16.8229403	0.03616104	0.01681172	4.88854071	0.02703553	0.07302565	Plppr4
ENSMUSG00000044674.7	169.025399	0.01403494	0.00269196	27.1059867	1.93E-07	1.90E-06	Fzd1
ENSMUSG00000044676.11	219.397908	0.00739616	0.00399583	3.3646183	0.06661102	0.15215514	Zfp612
ENSMUSG00000044678.12	3.97314072	0.14294399	0.01778231	43.5656624	4.10E-11	6.92E-10	Ly6k
ENSMUSG00000044681.20	3.35520695	0.07708854	0.01940143	10.3789511	0.0012746	0.00543827	Cnpy1
ENSMUSG00000044700.16	309.93857	-0.0139457	0.00299723	21.6853065	3.21E-06	2.53E-05	Tmem201

ENSMUSG00000044701.6	1.79466486	0.04184517	0.01161683	12.3794335	0.00043409	0.00210927	Il27
ENSMUSG00000044702.14	19.7387962	0.02801821	0.00615318	20.0465543	7.56E-06	5.49E-05	Palb2
ENSMUSG00000044703.6	9.73766988	0.04291397	0.00741435	31.3413605	2.16E-08	2.48E-07	Phf11a
ENSMUSG00000044705.4	1.36839324	0.00926172	0.03089477	0.26843138	0.60438596	0.77745919	Or52e18
ENSMUSG00000044707.8	7.75586542	0.00397566	0.00790545	0.23027699	0.63131852	0.79942611	Ccnjl
ENSMUSG00000044708.6	47.6367601	0.06094872	0.00658396	81.2216299	2.02E-19	9.81E-18	Kcnj10
ENSMUSG00000044709.7	344.241226	-0.0048232	0.00293851	2.71195887	0.09959862	0.20955692	Gemin7
ENSMUSG00000044712.16	114.907511	0.00595916	0.00388436	2.31647643	0.1280097	0.25500311	Slc38a6
ENSMUSG00000044715.8	211.424071	0.00673349	0.00290816	5.26779262	0.02172354	0.06089511	Gskip
ENSMUSG00000044716.13	3.76875732	-0.0040636	0.01339593	0.0356943	0.85014819	0.94770749	Dok7
ENSMUSG00000044726.9	3.95981089	0.00977543	0.01245778	0.63171809	0.42672581	0.618317	Erich5
ENSMUSG00000044730.18	121.395843	-0.0049928	0.00353366	1.96557759	0.16091806	0.30280943	Airim
ENSMUSG00000044734.17	233.15724	0.08869947	0.0069129	137.534677	9.21E-32	2.44E-29	Serpinb1a
ENSMUSG00000044748.9	76.0288291	0.03807269	0.00748407	26.1224989	3.20E-07	3.03E-06	Defb1
ENSMUSG00000044749.14	2911.77749	0.00383013	0.00454039	0.70816008	0.4000553	0.59181025	Abca6
ENSMUSG00000044763.9	406.974363	-0.0138135	0.00244705	31.8863348	1.63E-08	1.91E-07	Trmt10c
ENSMUSG00000044768.17	303.150994	0.00801483	0.00327496	5.86735152	0.01542424	0.04592806	Macir
ENSMUSG00000044770.15	9.39813589	0.04540904	0.00964118	19.2471261	1.15E-05	8.04E-05	Scml4
ENSMUSG00000044783.17	1639.23944	0.00897397	0.00346009	6.59390828	0.01023283	0.03251839	Hjurp
ENSMUSG00000044786.7	1283.41894	-7.57E-05	0.00452743	0.00028099	0.98662587	1	Zfp36
ENSMUSG00000044788.11	2320.09808	0.0006743	0.00449384	0.02201783	0.88203966	0.9650389	Fads6
ENSMUSG00000044791.17	785.975751	0.0003716	0.00246548	0.02262888	0.88042618	0.96408053	Setd2
ENSMUSG00000044792.8	1153.02075	-0.0114221	0.00255175	19.9879392	7.79E-06	5.64E-05	Isca1
ENSMUSG00000044795.13	149.896982	-0.0099806	0.00393755	6.29527899	0.012106	0.03741738	Cyb5d1
ENSMUSG00000044807.14	23.7777223	0.020425	0.00583897	11.8239772	0.00058473	0.00274237	Zfp354c
ENSMUSG00000044811.15	137.082551	0.05267254	0.0060958	67.95014	1.68E-16	5.62E-15	Cd300c2
ENSMUSG00000044813.16	522.596477	-0.0154721	0.00338035	21.0845607	4.39E-06	3.36E-05	Shb
ENSMUSG00000044816.11	3.07609987	0.03551443	0.01649042	4.12321284	0.04229868	0.10531686	D630023F18Rik
ENSMUSG00000044820.7	2.75461087	-0.0057167	0.00987465	0.20403143	0.65148628	0.81397256	AY074887
ENSMUSG00000044827.11	52.1286994	0.08853352	0.00691701	143.327424	4.98E-33	1.57E-30	Tlr1
ENSMUSG00000044835.16	8.32852204	0.01179661	0.01585485	0.65562594	0.41810841	0.60956268	Ankrd45
ENSMUSG00000044847.14	73.8086768	0.00220282	0.00337834	0.42207785	0.51590205	0.70250366	Lsm11
ENSMUSG00000044854.8	15.08015	0.02641092	0.00554664	22.6906147	1.90E-06	1.57E-05	1700056E22Rik
ENSMUSG00000044857.9	337.583169	-0.0056622	0.00267936	4.42545057	0.03540676	0.09110887	Lemd2
ENSMUSG00000044864.17	213.130763	0.00995866	0.00416027	5.58249786	0.01814083	0.05255093	Ankrd50
ENSMUSG00000044876.16	259.21986	0.00855451	0.00363487	5.52305514	0.01876745	0.05400795	Zfp444
ENSMUSG00000044881.8	139.291694	-0.0094668	0.00345771	7.52759895	0.00607608	0.02088466	Coa4
ENSMUSG00000044894.15	6458.51709	-0.0114418	0.00427614	7.25493192	0.00707066	0.02381078	Uqcrq
ENSMUSG00000044906.7	78.9771497	0.0176033	0.0042646	16.772102	4.21E-05	0.00026376	4930503L19Rik
ENSMUSG00000044912.12	4.01407463	0.00205034	0.02248438	-0.0200227	1	1	Syt16
ENSMUSG00000044916.11	21.2335051	0.00099206	0.00617913	0.02472123	0.8750637	0.96150045	Frey1
ENSMUSG00000044921.6	28.853101	0.00081215	0.00779843	0.01034837	0.91897344	0.98598497	Rassf9
ENSMUSG00000044923.5	9.9449344	0.13663418	0.00806458	262.773233	4.27E-59	2.01E-55	Or5m5
ENSMUSG00000044927.8	5.71175296	-0.0032631	0.01252008	0.06122179	0.80457518	0.91828391	H1f10
ENSMUSG00000044934.14	317.921059	0.00176585	0.00289885	0.36393938	0.5463265	0.72940433	Zfp367
ENSMUSG00000044937.16	44.3755315	-0.0167992	0.00526753	9.75938232	0.0017841	0.00730424	Ttc41
ENSMUSG00000044938.9	21.1377525	-0.0029217	0.01594593	-1.5014079	1	1	Klhl31
ENSMUSG00000044948.18	6.80045565	0.00854836	0.01233307	0.49902761	0.4799277	0.66959921	Cfap43
ENSMUSG00000044949.5	13.371617	-0.0075956	0.00621388	1.52083741	0.21749281	0.38165207	Ubtd2
ENSMUSG00000044950.8	177.612744	-0.0022512	0.00346341	0.41958949	0.51714195	0.70346099	Pwwp2a
ENSMUSG00000044951.18	44.2239954	0.03245567	0.0260897	-7.1211275	1	1	Mylk4
ENSMUSG00000044952.6	164.316489	-0.0060961	0.00239443	6.41365641	0.0113246	0.03543406	Kctd21
ENSMUSG00000044957.4	5.27177661	-0.0247456	0.01371411	3.17437084	0.07480202	0.16700874	Pp2d1
ENSMUSG00000044966.5	1.81856478	0.03315571	0.01282109	6.58220605	0.01030032	0.03268441	Fbxo48
ENSMUSG00000044968.17	29.660646	0.02044375	0.00448831	20.0604911	7.50E-06	5.46E-05	Napepld
ENSMUSG00000044976.18	7.65307914	0.02732849	0.01301098	4.56095721	0.03270879	0.08539622	Wdr72
ENSMUSG00000044982.8	87.5994199	0.00011506	0.0028906	0.00154428	0.96865338	1	Sft2d3
ENSMUSG00000044986.11	3606.7946	-0.017309	0.00389319	19.947565	7.96E-06	5.75E-05	Tst
ENSMUSG00000044991.11	116.164744	-0.0103903	0.00398096	6.88283697	0.00870273	0.02837863	Shld1
ENSMUSG00000045004.4	2.06555913	-0.0155447	0.04125497	0.1557214	0.69312666	0.84495099	Spata21
ENSMUSG00000045005.10	143.174366	-0.0003351	0.00349475	0.00913969	0.9238369	0.98846022	Fzd5

ENSMUSG00000045007.10	3.99665588	0.04523693	0.00912091	24.5240421	7.34E-07	6.45E-06	Tubg2
ENSMUSG00000045009.10	2.59984272	0.01746852	0.01357994	1.8753403	0.1708647	0.317324	Prrt3
ENSMUSG00000045010.15	0.96161101	-0.0345533	0.02629609	1.28832627	0.25635663	0.42946427	Gm4779
ENSMUSG00000045013.8	0.84301069	0.00621093	0.03647772	0.10790539	0.74254154	0.8782261	Or6b6
ENSMUSG00000045030.9	1.36122877	0.0265075	0.01439956	3.50374658	0.06123016	0.14225082	Or5b95
ENSMUSG00000045031.19	17.4305769	0.0410277	0.00955907	17.7250151	2.55E-05	0.00016649	Cetn4
ENSMUSG00000045034.13	3.05202726	0.01060821	0.0206971	0.47097988	0.49253672	0.68023019	Ankrd34b
ENSMUSG00000045036.16	1.09378797	-0.0248166	0.0600741	0.3661422	0.54511465	0.72837764	Tmem232
ENSMUSG00000045038.15	340.962214	0.01264653	0.00310321	16.2968649	5.42E-05	0.0003297	Prkce
ENSMUSG00000045039.10	210.538983	-0.0031369	0.00357654	0.76210482	0.38267063	0.57433467	Megf8
ENSMUSG00000045045.8	10.8719383	0.01837602	0.00881487	4.15286403	0.04156417	0.10380479	Lrfn4
ENSMUSG00000045062.5	3.91513006	0.04327722	0.0085959	24.9341931	5.93E-07	5.34E-06	Pcdhb7
ENSMUSG00000045064.6	66.046958	-0.0073167	0.0047518	2.23662018	0.13477529	0.26498916	Zc2hc1c
ENSMUSG00000045071.15	110.70867	-0.0100107	0.00453215	4.8619513	0.02745527	0.07391393	E130308A19Rik
ENSMUSG00000045078.13	382.833663	0.00357271	0.00231196	2.38863846	0.12222004	0.24571103	Rnf216
ENSMUSG00000045083.15	3.24978744	0.01833907	0.02335788	0.89001275	0.34547385	0.53393266	Lingo2
ENSMUSG00000045087.9	112.432331	0.04157045	0.00432502	85.5471073	2.26E-20	1.23E-18	S1pr5
ENSMUSG00000045091.5	2.92275607	-0.0151392	0.08325173	-0.0011873	1	1	Aqp12
ENSMUSG00000045092.9	1233.67415	-0.0055866	0.00399022	1.93973793	0.16369699	0.30691234	S1pr1
ENSMUSG00000045094.9	245.123837	-0.0086065	0.00422233	4.22420292	0.03985133	0.10030237	Arhgef37
ENSMUSG00000045095.17	546.36324	-0.0030852	0.00363848	0.71520368	0.39772171	0.58942283	Magi1
ENSMUSG00000045098.20	519.39194	-0.0007844	0.0029381	0.0698426	0.79156609	0.91112102	Kmt5b
ENSMUSG00000045100.12	115.083326	-0.0150195	0.00359492	17.1034061	3.54E-05	0.00022432	Slc25a26
ENSMUSG00000045102.13	4.83222208	0.01489543	0.01406854	0.72038483	0.39601771	0.58782997	Poln
ENSMUSG00000045103.20	531.321512	9.13E-05	0.00366392	0.00060365	0.98039845	1	Dmd
ENSMUSG00000045106.13	25.7197744	-0.0017836	0.00888125	0.03871735	0.84400968	0.943272	Ccdc73
ENSMUSG00000045107.6	248.854842	-0.0129758	0.00390004	11.1093411	0.00085894	0.00383616	Saysd1
ENSMUSG00000045114.11	16.9623204	0.04443399	0.0205555	3.74757618	0.05288415	0.12622197	Prrt2
ENSMUSG00000045128.10	4531.28202	-0.0008062	0.00316199	0.06477492	0.79910221	0.91547812	Rpl18a
ENSMUSG00000045132.4	7.43813338	0.01042269	0.01644146	0.70468447	0.40121411	0.59286488	Or51v14
ENSMUSG00000045136.7	12.1078558	0.04378741	0.01216905	12.4120406	0.00042657	0.00207618	Tubb2b
ENSMUSG00000045140.9	70.0681723	-0.0093279	0.00261787	12.7206634	0.00036164	0.00179646	Pigw
ENSMUSG00000045160.15	1093.51981	-0.0091883	0.00311919	8.6818426	0.00321396	0.0121132	Bola3
ENSMUSG00000045165.7	16.5563866	0.05933569	0.00695055	63.6250219	1.51E-15	4.56E-14	AI467606
ENSMUSG00000045176.4	146.951925	0.00386923	0.00302121	1.62895828	0.20184719	0.3608009	Borcs6
ENSMUSG00000045180.14	343.751874	-0.0079779	0.00362368	4.79053929	0.02861647	0.07647603	Shroom2
ENSMUSG00000045193.14	338.200217	0.01535667	0.00413599	14.0208731	0.00018079	0.00097645	Cirbp
ENSMUSG00000045201.7	4.68843664	-0.0231875	0.02351963	0.77755735	0.37788923	0.56907773	Lrrc3b
ENSMUSG00000045205.17	283.528874	0.00169361	0.00305621	0.30192	0.58268125	0.76025447	Dpy19l4
ENSMUSG00000045210.9	840.608696	-0.0012435	0.00256516	0.22893661	0.63231344	0.8000936	Vcpip1
ENSMUSG00000045211.6	225.502909	0.01554532	0.00383851	16.2140739	5.66E-05	0.00034315	Nudt18
ENSMUSG00000045215.19	4.41923181	-0.0033626	0.01458141	0.06164514	0.80391438	0.91805563	Asxl3
ENSMUSG00000045216.8	2081.35403	-0.0027093	0.00275961	0.96668099	0.32550933	0.51227484	Hs6st1
ENSMUSG00000045225.5	2.00830449	-0.0110474	0.01927799	0.3189172	0.57225909	0.75147837	Or5w19
ENSMUSG00000045237.6	216.679749	0.00132313	0.00258538	0.26653028	0.60566884	0.77840591	Eola1
ENSMUSG00000045246.13	1.2572738	0.03212441	0.01424047	5.30539672	0.02125946	0.05985027	Kcng4
ENSMUSG00000045248.7	87.8051247	-0.0047461	0.00218717	4.69099892	0.03032102	0.08022487	Med26
ENSMUSG00000045251.14	62.6735853	0.00530175	0.00452194	1.33774382	0.24743224	0.41875266	Zfp688
ENSMUSG00000045252.12	258.646777	-0.0112599	0.00261163	18.3598958	1.83E-05	0.0001233	Zfp574
ENSMUSG00000045257.11	65.2450544	-0.0079985	0.00347543	5.43056467	0.01978728	0.05637869	Morn2
ENSMUSG00000045268.14	116.653037	-0.019224	0.00518765	13.4368486	0.00024673	0.00128321	Zfp691
ENSMUSG00000045273.9	5.61038604	0.05393627	0.01250772	17.820119	2.43E-05	0.00015925	Cenph
ENSMUSG00000045275.18	3.67517561	-0.0066411	0.01184871	0.29099887	0.58958114	0.76607871	Lca5l
ENSMUSG00000045282.8	875.756682	-0.0064475	0.003701	3.03984957	0.08124345	0.17835144	Tmem86b
ENSMUSG00000045284.10	84.7715185	0.00417111	0.0038179	1.17719509	0.27792776	0.45680865	Dcaf12l1
ENSMUSG00000045287.7	245.554384	0.00743533	0.00439629	2.86333567	0.0906194	0.1944282	Rtn4rl1
ENSMUSG00000045288.11	8.7052372	0.00261873	0.00480271	0.2903587	0.58999076	0.76627324	Ush1g
ENSMUSG00000045294.12	8100.28779	0.01327334	0.004207	9.78027117	0.00176394	0.00723302	Insig1
ENSMUSG00000045302.14	1559.69682	-0.0067257	0.00274377	6.02465608	0.01410738	0.04264761	Preb
ENSMUSG00000045306.4	1.16164748	0.01759377	0.03711992	0.57602572	0.44787434	0.639712	Or4m1
ENSMUSG00000045312.13	179.420234	0.00013881	0.0032041	0.00182192	0.96595343	1	Lhfpl2

ENSMUSG00000045314.6	132.046381	0.00602574	0.00427812	1.90626697	0.16737852	0.31232514	Sowahb
ENSMUSG00000045316.6	979.909034	-0.0084308	0.00281266	8.80285236	0.0030076	0.01145442	Fahd1
ENSMUSG00000045318.7	3.8035727	0.03293772	0.01432703	4.87562956	0.0272385	0.07343763	Adra2c
ENSMUSG00000045319.14	316.798098	-0.005694	0.00358548	2.51962699	0.11243718	0.23008357	Proser2
ENSMUSG00000045322.11	12.8394116	0.07675348	0.0082387	77.0468991	1.67E-18	7.16E-17	Tlr9
ENSMUSG00000045326.14	4.03515903	0.01787507	0.01066719	2.46066303	0.1167295	0.23705717	Fndc7
ENSMUSG00000045328.12	31.0010211	0.03804138	0.01180731	9.17758963	0.00244996	0.0096036	Cenpe
ENSMUSG00000045331.4	1.60900987	-0.0122307	0.06817086	0.05608111	0.8128007	0.92326471	2310079G19Rik
ENSMUSG00000045333.16	17.4972005	0.05440883	0.00833468	38.0058325	7.05E-10	1.00E-08	Zfp423
ENSMUSG00000045340.5	1.41905102	0.00159822	0.02641464	0.01878225	0.89099257	0.97055162	Vmn1r70
ENSMUSG00000045341.3	1.34667387	-0.0078793	0.03313964	0.08732707	0.76760314	0.89529659	Or2l5
ENSMUSG00000045348.17	8.5457017	0.02541521	0.00984187	6.63209224	0.01001576	0.03194046	Nyap1
ENSMUSG00000045349.16	6.68655225	0.01851639	0.00964525	3.60704317	0.05753533	0.13528594	Sh2d5
ENSMUSG00000045350.16	5.85640016	0.00712497	0.01528376	0.39983226	0.5271759	0.71209958	Fam186a
ENSMUSG00000045362.9	22.4328868	0.03439117	0.00483195	49.0815358	2.46E-12	4.91E-11	Tnfrsf26
ENSMUSG00000045374.19	706.201923	0.00093952	0.00305404	0.09284019	0.76059703	0.89078115	Wdr81
ENSMUSG00000045377.6	47.1087496	-0.0015809	0.00365215	0.19035748	0.66261926	0.82289764	Tmem88
ENSMUSG00000045381.7	1.19573407	0.03422254	0.01572854	4.87150415	0.02730369	0.07356464	Or10aa1
ENSMUSG00000045382.7	39.0173831	0.09352408	0.0078126	121.05101	3.72E-28	5.63E-26	Cxcr4
ENSMUSG00000045392.9	148.631122	0.00788992	0.00642331	1.55070273	0.21303173	0.37553217	Or5m3b
ENSMUSG00000045394.10	45.669507	0.01881151	0.0059759	9.53738192	0.00201329	0.0081241	Epcam
ENSMUSG00000045404.17	21.5601589	0.04996278	0.00639936	56.1333489	6.77E-14	1.65E-12	Kcnk13
ENSMUSG00000045409.17	267.688571	-0.0039391	0.00303753	1.67639309	0.19540442	0.35179059	Trim39
ENSMUSG00000045410.19	981.682256	-0.0138889	0.00340787	16.7453291	4.27E-05	0.00026687	Akr1e1
ENSMUSG00000045411.17	211.850145	0.01004233	0.00324857	9.56171827	0.00198677	0.00803773	2410002F23Rik
ENSMUSG00000045414.8	806.997486	0.00243412	0.00252021	0.90834279	0.34055451	0.52833774	Dipk2a
ENSMUSG00000045417.3	2.05778583	0.00495007	0.02647298	0.11677835	0.73255524	0.87173305	Vmn1r230
ENSMUSG00000045427.14	1100.99654	0.0007083	0.0019502	0.12936913	0.71908704	0.86246565	Hnrnph2
ENSMUSG00000045435.10	228.623918	-0.0043002	0.00306007	1.96573811	0.16090096	0.30280763	Tmem60
ENSMUSG00000045438.13	1012.50226	0.00122914	0.00358088	0.11182725	0.73807336	0.87553177	Cox19
ENSMUSG00000045441.6	805.461777	-0.0131871	0.00607117	4.48888924	0.03411584	0.08843347	Gprin3
ENSMUSG00000045466.19	69.522768	-0.0005011	0.00328502	0.0233939	0.8784372	0.96308371	Zfp956
ENSMUSG00000045467.14	13.9829952	0.00261901	0.00616896	0.17413973	0.6764586	0.83279147	Ttll13
ENSMUSG00000045482.17	654.719678	0.0027995	0.00317046	0.76254955	0.38253182	0.57423597	Trrap
ENSMUSG00000045498.2	6.3484498	0.02710849	0.01253214	4.54806507	0.03295597	0.08588945	Pcdhb3
ENSMUSG00000045502.7	21.6515167	0.06444012	0.00961552	36.0728388	1.90E-09	2.53E-08	Hcar2
ENSMUSG00000045503.17	556.732305	-0.0086994	0.00305022	8.13184197	0.00434945	0.01571721	Sys1
ENSMUSG00000045515.5	41.288786	0.01352806	0.01951191	0.67857743	0.41007701	0.60183522	Pou3f3
ENSMUSG00000045519.16	161.314399	-0.0055677	0.00459154	1.46813373	0.22564038	0.39169456	Zfp560
ENSMUSG00000045521.8	1.30916539	-0.0151435	0.03314351	0.24902385	0.61776325	0.78881789	Tssk2
ENSMUSG00000045532.6	2.41594616	0.00288395	0.02761861	0.00067822	0.97922329	1	C1ql1
ENSMUSG00000045534.5	3.34110508	0.01199445	0.01546678	0.69424618	0.40472384	0.59627813	Kcna5
ENSMUSG00000045538.7	230.789931	-0.009273	0.00233169	15.5110579	8.20E-05	0.00047851	Ddx28
ENSMUSG00000045545.9	1.41077497	0.03334167	0.02835205	1.53282942	0.2156883	0.37907781	Krt14
ENSMUSG00000045551.8	88.9997354	0.04604961	0.00652086	46.0637831	1.14E-11	2.09E-10	Fpr1
ENSMUSG00000045559.7	2.74555623	0.00058164	0.02562793	-0.0012422	1	1	Or9k7
ENSMUSG00000045573.10	13.0639787	0.07927954	0.01219898	35.9593794	2.01E-09	2.68E-08	Penk
ENSMUSG00000045576.17	486.828783	-0.0087287	0.00273681	10.108157	0.00147615	0.00618408	St7l
ENSMUSG00000045589.8	44.9304493	0.04042092	0.01663777	6.29820734	0.01208601	0.03736389	Frrs1l
ENSMUSG00000045594.11	248.618912	0.00456285	0.00294325	2.42139037	0.11968866	0.2417139	Glb1
ENSMUSG00000045598.11	118.188505	-0.003719	0.00320635	1.32072996	0.25046109	0.42216645	Zfp553
ENSMUSG00000045613.10	28.0764857	-0.0050372	0.01302543	0.13368423	0.71464228	0.85936335	Chrm2
ENSMUSG00000045624.16	589.717615	0.00128975	0.00347797	0.13392075	0.71440101	0.85920732	Esf1
ENSMUSG00000045625.6	7.54386151	0.05406749	0.00867236	34.746235	3.76E-09	4.84E-08	Pigz
ENSMUSG00000045629.9	18.8985509	0.00671217	0.00403924	2.75850778	0.09673843	0.2048364	Sh3tc2
ENSMUSG00000045636.17	3356.23407	-0.0047287	0.00280765	2.74709881	0.09743107	0.20598864	Mtus1
ENSMUSG00000045639.15	349.552891	-0.0087245	0.00571015	2.25226953	0.1334186	0.26300135	Zfp629
ENSMUSG00000045648.16	1.4253185	0.03999054	0.02747306	4.0524847	0.04410638	0.10889701	Vwc2l
ENSMUSG00000045657.4	2.05171391	0.02321294	0.01202714	3.58377296	0.05834649	0.13682246	Pcdhb10
ENSMUSG00000045658.17	1895.43953	-0.001755	0.00316553	0.30349687	0.58169846	0.75926618	Pid1
ENSMUSG00000045659.19	127.192257	0.00524511	0.00407959	1.69247355	0.19327522	0.34875327	Plekha7

ENSMUSG00000045664.6	70.3253881	0.01382408	0.00338865	16.614317	4.58E-05	0.000284	Cdc42ep2
ENSMUSG00000045665.6	294.109766	0.00127225	0.00252147	0.25688504	0.6122683	0.78432327	Mfsd5
ENSMUSG00000045667.15	17.955564	0.02998786	0.00652565	20.4598656	6.09E-06	4.53E-05	Smtnl2
ENSMUSG00000045671.18	187.032332	0.00049229	0.0036392	0.01797288	0.89335277	0.97233263	Spred2
ENSMUSG00000045672.16	738.346053	-0.0478072	0.00656448	51.1154386	8.71E-13	1.84E-11	Col27a1
ENSMUSG00000045678.9	5.03726373	0.01196016	0.0193464	0.54442661	0.46060396	0.65189349	Or5b124
ENSMUSG00000045679.15	18.2128474	0.05216523	0.0074738	43.4044895	4.45E-11	7.48E-10	Slc66a3
ENSMUSG00000045680.9	95.8354156	0.0231828	0.00386788	35.6283166	2.39E-09	3.15E-08	Tcf21
ENSMUSG00000045689.4	3.57117768	0.04747625	0.00836659	31.0471394	2.52E-08	2.86E-07	Pcdhb4
ENSMUSG00000045690.9	79.0613786	0.00298591	0.00601818	0.23278195	0.62946868	0.79802214	Wdr89
ENSMUSG00000045691.15	491.968188	-0.0008426	0.00233705	0.12814358	0.72036466	0.86355888	Thtpa
ENSMUSG00000045725.6	6.27413741	0.0267991	0.00798132	11.1458523	0.0008422	0.003775	Prr15
ENSMUSG00000045730.5	53.2100806	0.0472703	0.00605545	58.9860878	1.59E-14	4.24E-13	Adrb2
ENSMUSG00000045733.8	27.8114924	0.00613858	0.00590756	1.0528456	0.30485263	0.48964993	Sprn
ENSMUSG00000045744.12	1.55667293	-0.0147458	0.0141531	0.9481655	0.33018669	0.51697978	Bricd5
ENSMUSG00000045751.17	34.1157661	0.01597629	0.00679774	5.31169305	0.02118276	0.05967709	Mms22l
ENSMUSG00000045752.14	236.987764	-0.0072937	0.00393271	3.43656577	0.06376811	0.14693579	Tssc4
ENSMUSG00000045757.7	50.6624967	0.0097723	0.00361076	7.16150373	0.00744847	0.02486972	Zfp764
ENSMUSG00000045761.17	2.57837577	0.06601642	0.02032043	10.4655892	0.00121619	0.0052202	Togaram2
ENSMUSG00000045763.9	119.536106	0.07254323	0.0073658	88.8304839	4.30E-21	2.51E-19	Basp1
ENSMUSG00000045767.10	960.293725	-0.0041207	0.00255556	2.54593811	0.11057822	0.22713938	B230219D22Rik
ENSMUSG00000045775.16	355.09953	0.07693954	0.0070306	107.704979	3.12E-25	2.99E-23	Slc16a5
ENSMUSG00000045776.4	279.485966	0.05655703	0.00806017	47.9043128	4.48E-12	8.67E-11	Lrtm1
ENSMUSG00000045777.15	7.68498371	0.03159547	0.00753993	16.4883746	4.89E-05	0.00030105	Ifitm10
ENSMUSG00000045780.4	2.7607056	-0.0002116	0.01015217	0.00023648	0.98773079	1	Or51v8
ENSMUSG00000045790.11	53.0653914	-0.0120584	0.00473733	6.33144524	0.01186156	0.03675339	Ccdc149
ENSMUSG00000045795.11	705.334894	-0.0055197	0.00327237	2.83484288	0.09223982	0.19731258	Whamm
ENSMUSG00000045817.9	795.227515	0.00721537	0.00317464	4.97505346	0.02571541	0.07012645	Zfp36l2
ENSMUSG00000045822.5	39.7405242	-0.0025993	0.00376554	0.4624851	0.49646484	0.68361374	Zswim3
ENSMUSG00000045826.10	26.9296548	0.06988865	0.00715317	84.8572801	3.21E-20	1.71E-18	Ptprcap
ENSMUSG00000045827.11	257.148264	0.01828168	0.00308177	35.030019	3.25E-09	4.22E-08	Serpinb9
ENSMUSG00000045835.6	5.09890361	-0.0219595	0.03811611	0.3861903	0.53430877	0.71868872	Hdgfl1
ENSMUSG00000045838.10	55.2727338	-0.003367	0.00433313	0.60664901	0.43605231	0.62750731	Ccdc9b
ENSMUSG00000045854.5	188.901001	-0.0025985	0.00372314	0.4826485	0.4872251	0.67566387	Lyrm2
ENSMUSG00000045867.11	220.614443	0.0009182	0.00234459	0.15192317	0.69670424	0.84758716	Cradd
ENSMUSG00000045868.13	1189.04813	0.0606236	0.00659755	75.7728506	3.18E-18	1.32E-16	Gvin1
ENSMUSG00000045871.6	3.06307709	-0.0033053	0.02133702	-0.0081581	1	1	Slitrk6
ENSMUSG00000045875.14	87.6951485	0.00830314	0.00490686	2.73906317	0.09792216	0.20678812	Adra1a
ENSMUSG00000045876.5	4.69056268	0.04840225	0.01049894	18.715844	1.52E-05	0.00010408	Pcdhb8
ENSMUSG00000045886.8	31.6522417	-0.0014098	0.00883694	0.02394578	0.87702295	0.96235933	Pam16l
ENSMUSG00000045896.15	427.0409	-0.0069232	0.00305559	5.00905538	0.02521506	0.06896932	Paip2b
ENSMUSG00000045912.10	3.79699477	0.04133108	0.01234245	10.208102	0.00139825	0.00588598	C2cd4c
ENSMUSG00000045915.16	1.57998495	0.01098009	0.04609036	0.25567755	0.61310541	0.78505452	Ccdc42
ENSMUSG00000045917.18	197.112113	0.03985061	0.0049008	63.6003048	1.52E-15	4.60E-14	Tmem268
ENSMUSG00000045930.4	328.569091	0.00424457	0.00278617	2.29006659	0.13020434	0.25817566	Clec14a
ENSMUSG00000045932.13	133.39228	0.03470223	0.00532692	42.4083359	7.41E-11	1.21E-09	Ifit2
ENSMUSG00000045934.16	72.2230077	0.05653332	0.0076117	51.9557658	5.68E-13	1.23E-11	Mtmr11
ENSMUSG00000045942.14	5.62017759	-0.0185428	0.00793519	5.63677413	0.01758754	0.05119334	BC049762
ENSMUSG00000045948.9	533.293425	-0.0128169	0.00422746	9.22856927	0.00238268	0.00938351	Mrps12
ENSMUSG00000045954.8	440.15702	-0.0109032	0.0036762	8.70814926	0.00316791	0.01196641	Cavin2
ENSMUSG00000045962.17	2442.02401	0.00510461	0.0030885	2.64160119	0.10409858	0.21645168	Wnk1
ENSMUSG00000045967.12	6.64310524	0.01283885	0.01768067	0.65477446	0.41841084	0.6098528	Gpr158
ENSMUSG00000045968.8	21.6943419	-0.0216343	0.00585194	13.1409963	0.0002889	0.00147619	Teddm2
ENSMUSG00000045969.9	252.205768	-0.0018276	0.00245063	0.55256948	0.4572697	0.64899696	Ing1
ENSMUSG00000045973.19	1273.67601	-0.0201028	0.00342977	33.9237759	5.73E-09	7.18E-08	Slc25a51
ENSMUSG00000045975.9	2364.30178	-0.0071748	0.00333458	4.6175702	0.03164604	0.08313694	C2cd2
ENSMUSG00000045980.14	205.977963	0.00422286	0.00330469	1.61265385	0.20411915	0.3639518	Tmem104
ENSMUSG00000045983.17	7396.36768	-0.0075141	0.0022831	10.7509673	0.00104225	0.00455737	Eif4g1
ENSMUSG00000045991.20	1904.66074	-0.0023853	0.00576565	0.16551049	0.684133	0.83856504	Onecut2
ENSMUSG00000045994.18	8.18497052	0.03119219	0.01875735	2.84158145	0.09185377	0.19664728	B3gat1
ENSMUSG00000045996.13	506.414776	-0.0113579	0.00400296	8.15283717	0.00429938	0.01555534	Polr2k

ENSMUSG00000046006.7	2.9702457	0.05450206	0.01052432	25.810237	3.77E-07	3.53E-06	Gapt
ENSMUSG00000046008.9	2.11025906	-0.0431178	0.06007258	0.00687089	NA	NA	Pnlip
ENSMUSG00000046010.8	159.995864	-0.0088326	0.00239883	13.5468759	0.00023268	0.00121873	Zfp830
ENSMUSG00000046020.14	254.597208	0.00478829	0.00210449	5.16663893	0.02302472	0.06397987	Pofut1
ENSMUSG00000046027.18	1923.98465	-0.0026196	0.00305811	0.75126817	0.38607502	0.57764307	Stard5
ENSMUSG00000046031.8	45.836002	0.07034125	0.0062124	119.507117	8.11E-28	1.18E-25	Calhm6
ENSMUSG00000046032.17	200.619625	0.01572149	0.00306457	26.4264118	2.74E-07	2.62E-06	Snx12
ENSMUSG00000046034.9	612.378196	-0.0096408	0.00332733	8.25430131	0.00406556	0.0148322	Otulin
ENSMUSG00000046041.5	3.00485054	-0.0026754	0.05362172	-0.0058624	1	1	Or6c3b
ENSMUSG00000046056.16	13.4497655	0.01787023	0.00870032	4.08183188	0.04334664	0.10748253	Sbsn
ENSMUSG00000046058.8	10.1461384	0.0109019	0.00672571	2.53476182	0.11136369	0.2283854	Eid2
ENSMUSG00000046062.6	2053.47541	-0.0030095	0.00281601	1.12308053	0.2892561	0.47041263	Ppp1r15b
ENSMUSG00000046070.10	1665.78137	-0.0206529	0.0044447	21.4655344	3.60E-06	2.81E-05	Igfals
ENSMUSG00000046079.17	628.225933	-0.0025059	0.00275659	0.81588131	0.36638713	0.55626694	Lrrc8d
ENSMUSG00000046080.18	54.3211165	0.04213247	0.0057653	49.3776214	2.11E-12	4.25E-11	Clec9a
ENSMUSG00000046082.5	38.2995123	-0.0080249	0.03625705	0.05974999	0.80689152	0.91974554	Tmem174
ENSMUSG00000046093.10	25.2956136	0.00231559	0.01852765	0.0442042	0.83347416	0.9362443	Hpcal4
ENSMUSG00000046096.8	330.84833	-0.002497	0.00272243	0.83759	0.36008694	0.54935386	Mosmo
ENSMUSG00000046101.17	3.81278636	0.04671261	0.01110175	16.4496574	5.00E-05	0.0003063	Mcmdc2
ENSMUSG00000046110.16	5.04169771	0.00683985	0.01009635	0.46673895	0.49449124	0.6818914	Serinc4
ENSMUSG00000046111.19	157.176574	0.00563929	0.00494364	1.30154528	0.25393115	0.42621834	Cep295
ENSMUSG00000046130.8	3.82798998	0.07266954	0.0168627	20.8914345	4.86E-06	3.69E-05	Vmn1r184
ENSMUSG00000046138.16	499.362741	0.00146347	0.00375778	0.15117077	0.69741903	0.84824135	9930021J03Rik
ENSMUSG00000046139.9	321.860966	-0.0003085	0.00298158	0.01076621	0.91735954	0.98479095	Patl1
ENSMUSG00000046152.17	19.7961032	0.00880667	0.00481736	3.34365778	0.06746448	0.15369624	Fut10
ENSMUSG00000046157.14	51.025664	0.02613701	0.0057003	19.6425658	9.34E-06	6.67E-05	Tmem229b
ENSMUSG00000046159.17	59.8856036	0.06770025	0.00778001	59.7894007	1.06E-14	2.89E-13	Chrm3
ENSMUSG00000046160.7	106.758248	0.01961234	0.00641296	7.94735792	0.00481576	0.01711807	Olig1
ENSMUSG00000046167.6	31.4350232	0.133654	0.01360956	62.626459	2.50E-15	7.35E-14	Gldn
ENSMUSG00000046168.10	5.22431224	-0.014496	0.01006508	1.94159561	0.16349539	0.30663186	Kcnrg
ENSMUSG00000046169.11	36.9027583	0.03203291	0.00587388	29.2259876	6.44E-08	6.86E-07	Adamts6
ENSMUSG00000046173.4	3.83561089	-0.0283401	0.03833336	-0.2072435	1	1	Pabpc6
ENSMUSG00000046178.4	6.27636854	0.06252906	0.01084189	28.757893	8.20E-08	8.64E-07	Nxph1
ENSMUSG00000046179.18	150.31742	-0.0431519	0.00624981	42.6293749	6.62E-11	1.09E-09	E2f8
ENSMUSG00000046182.9	10.9815225	0.0517835	0.00689422	55.5591771	9.07E-14	2.16E-12	Gsg1l
ENSMUSG00000046185.11	149.54506	-0.0026892	0.00219841	1.46605621	0.22596897	0.39214952	Zfp84
ENSMUSG00000046186.9	4.10576509	0.09509042	0.02062582	16.6893955	4.40E-05	0.0002737	Cd109
ENSMUSG00000046191.8	6.71923821	0.01347276	0.00821472	2.60009991	0.10685698	0.22104269	Pcdhb20
ENSMUSG00000046192.5	1.8475624	-0.0198731	0.01541352	1.48342724	0.22323907	0.3887851	Iqub
ENSMUSG00000046196.5	2.22540811	0.02012199	0.02191949	-33.250489	1	1	Ttc39d
ENSMUSG00000046201.10	413.782368	0.00100796	0.00224098	0.20158631	0.65344346	0.81515403	Scaf8
ENSMUSG00000046204.15	3.5137929	0.03408283	0.01705984	3.93551154	0.04727676	0.11534806	Pnma2
ENSMUSG00000046207.15	25.4846629	0.02193136	0.00621624	11.6869629	0.0006294	0.00292742	Pik3r6
ENSMUSG00000046213.10	1.45378341	-0.0229563	0.01292671	3.32530985	0.06822116	0.15509053	Cym
ENSMUSG00000046215.4	3.43086858	0.01574938	0.04394524	0.33902748	0.56039112	0.74178867	Rprml
ENSMUSG00000046223.11	22.5947746	0.06030663	0.00888943	41.1098825	1.44E-10	2.26E-09	Plaur
ENSMUSG00000046229.11	220.512366	0.00869502	0.00523848	2.70162226	0.1002462	0.21060005	Scand1
ENSMUSG00000046230.12	413.030001	0.00391175	0.00326511	1.40736647	0.23549407	0.40407122	Vps13a
ENSMUSG00000046240.9	2.5134912	0.02876181	0.01843753	2.83082705	0.09247073	0.19768101	Hepacam
ENSMUSG00000046245.14	124.671974	0.05149465	0.00624197	62.4903412	2.68E-15	7.84E-14	Pilra
ENSMUSG00000046258.5	0.95135457	-0.0006534	0.03620665	-0.0026488	1	1	Adam29
ENSMUSG00000046269.18	17.2051132	0.01123157	0.00476376	5.41486324	0.01996602	0.05684675	Usp27x
ENSMUSG00000046275.3	7.47261756	0.01838022	0.00659484	7.63898851	0.00571199	0.01982972	Trarg1
ENSMUSG00000046280.10	89.5319993	-0.004624	0.00357833	1.66789967	0.19654009	0.35332276	She
ENSMUSG00000046287.8	0.96088151	0.01542842	0.05658346	0.33100008	0.5650708	0.74535875	Pnma3
ENSMUSG00000046295.14	7.15444804	0.01090913	0.0105287	1.05844006	0.30357124	0.48817286	Ankle1
ENSMUSG00000046311.15	397.52125	0.00850063	0.00347195	5.75722147	0.01642102	0.04834679	Zfp62
ENSMUSG00000046312.5	647.475131	-0.0013106	0.00321021	0.16872827	0.68124451	0.83643067	Myorg
ENSMUSG00000046314.17	287.117465	0.00802712	0.00375644	4.50834746	0.03372981	0.08760614	Stxbp6
ENSMUSG00000046318.17	99.164463	0.01608466	0.00383911	17.0687818	3.61E-05	0.0002282	Ccbe1
ENSMUSG00000046321.9	0.89366307	0.04774091	0.03461364	2.91855803	0.08756608	0.18937786	Hs3st2

ENSMUSG00000046323.9	1.46571411	0.0562131	0.01267586	20.4372725	6.16E-06	4.57E-05	Dppa3
ENSMUSG00000046324.13	704.856848	0.01490966	0.00304494	22.5045814	2.10E-06	1.71E-05	Ermp1
ENSMUSG00000046329.15	4146.31718	-0.0045667	0.00347169	1.71976573	0.1897232	0.34396545	Slc25a23
ENSMUSG00000046330.11	3468.35154	0.00174998	0.00413952	0.18095185	0.67055651	0.82877326	Rpl37a
ENSMUSG00000046338.5	43.4388422	0.02238766	0.00397393	30.6128584	3.15E-08	3.53E-07	Gpat2
ENSMUSG00000046345.6	3.31875805	-0.0208018	0.02527984	0.903268	0.34190695	0.52984345	Smco1
ENSMUSG00000046351.12	330.590055	-0.0102595	0.00321853	10.0971521	0.00148499	0.0062178	Zfp322a
ENSMUSG00000046352.8	5148.33416	0.00112922	0.00444536	0.06258244	0.80245988	0.91721424	Gjb2
ENSMUSG00000046364.15	8841.06778	-0.0110777	0.00377627	8.67913735	0.00321873	0.01212732	Rpl27a
ENSMUSG00000046380.4	27.6383906	0.01889946	0.00463688	16.1856212	5.74E-05	0.00034784	Jrk
ENSMUSG00000046387.6	11.0836463	0.01009039	0.00670628	2.27369005	0.13158626	0.26034675	Pcdhb17
ENSMUSG00000046402.11	468.341407	0.0303073	0.0060784	23.8829588	1.02E-06	8.79E-06	Rbp1
ENSMUSG00000046404.6	372.131517	-0.0070938	0.00338771	4.37554624	0.03645814	0.0933357	Yod1
ENSMUSG00000046408.16	8.78633559	0.00410341	0.00724549	0.33383591	0.56340905	0.74404064	1700067K01Rik
ENSMUSG00000046410.11	65.7341598	0.02146192	0.00521482	15.8533501	6.84E-05	0.00040694	Kcnk6
ENSMUSG00000046417.15	15.2989663	0.02417963	0.00680993	11.6911491	0.00062798	0.00292199	Lrrc75a
ENSMUSG00000046432.13	43.0613461	0.02086603	0.00736477	8.11787086	0.0043831	0.01582537	Bex3
ENSMUSG00000046434.17	1268.09955	0.00953199	0.00285982	10.8617054	0.00098173	0.0043216	Hnrnpa1
ENSMUSG00000046441.6	62.9200867	0.00132519	0.00297289	0.19685114	0.65727461	0.81906689	Cmtr2
ENSMUSG00000046442.5	23.6464755	0.03655297	0.00872164	16.2806523	5.46E-05	0.00033236	Ppm1e
ENSMUSG00000046447.4	1658.98189	0.00662438	0.00322925	4.13110887	0.04210175	0.10489194	Camk2n1
ENSMUSG00000046449.16	4.48749929	0.03937938	0.01430069	7.3215024	0.00681344	0.02308603	Nexmif
ENSMUSG00000046456.15	4.63052526	0.04841753	0.00870027	29.2816532	6.26E-08	6.69E-07	Tmem150b
ENSMUSG00000046470.6	100.262022	-0.0004605	0.00378771	0.01460597	0.90380577	0.97767212	Sox18
ENSMUSG00000046480.7	15.7653621	0.00212091	0.0120438	0.04996334	0.82312707	0.92955242	Scn4b
ENSMUSG00000046490.8	3.02654536	-0.0079938	0.00706961	1.27442031	0.25893907	0.43253094	Rnf222
ENSMUSG00000046491.11	19.124171	0.00435365	0.00499739	0.75301864	0.38552219	0.57717132	C1qtnf2
ENSMUSG00000046516.12	857.494973	-0.0129977	0.005543	5.50399655	0.01897306	0.05447999	Cox17
ENSMUSG00000046519.17	147.182412	0.00610369	0.00302047	4.08147881	0.0433557	0.10749369	Golph3l
ENSMUSG00000046523.6	4.74965062	0.02904569	0.01893792	2.87492789	0.089969	0.19350802	Kctd4
ENSMUSG00000046532.9	310.561584	0.05291307	0.00496361	94.2021044	2.85E-22	1.96E-20	Ar
ENSMUSG00000046541.10	63.0534905	-0.0011906	0.00359993	0.10824697	0.74214883	0.87800736	Zfp526
ENSMUSG00000046546.4	128.629108	0.00903328	0.00320855	7.78073802	0.00528062	0.01853956	Fam43a
ENSMUSG00000046550.9	4.14612187	0.01572196	0.00894745	3.12536209	0.07708275	0.17111281	Spin2c
ENSMUSG00000046556.8	132.897516	0.00073191	0.00233442	0.09822998	0.75396416	0.88618033	Zfp319
ENSMUSG00000046561.10	1.31463341	0.00061312	0.02223957	0.01269089	0.910305	0.98098286	Arsj
ENSMUSG00000046562.6	580.415621	0.00155502	0.00237518	0.42989816	0.51203892	0.69865498	Unc119b
ENSMUSG00000046567.11	110.490976	-0.0043718	0.00346576	1.58491504	0.20805414	0.36919709	Rlig1
ENSMUSG00000046572.11	36.5157442	-0.0048916	0.00521406	0.84614603	0.3576449	0.54644119	Zfp518b
ENSMUSG00000046573.8	193.103501	-0.0062005	0.00257117	5.85591524	0.01552478	0.04616413	Lyrm4
ENSMUSG00000046574.9	213.809264	0.00020225	0.00452008	0.0019828	0.96448303	1	Prr12
ENSMUSG00000046585.10	2.0049332	-0.0137691	0.04410055	-0.0285943	1	1	Cfap58
ENSMUSG00000046589.8	11.2570473	0.02678907	0.0083163	9.55142046	0.00199795	0.00807741	Lrrc8e
ENSMUSG00000046591.11	5.57092833	0.0378666	0.01327538	7.41654664	0.00646267	0.02204637	Ticrr
ENSMUSG00000046593.4	3.52937906	0.03413025	0.00929669	12.944454	0.00032087	0.00161642	Tmem215
ENSMUSG00000046598.16	3366.47872	-0.0086938	0.00322749	7.29143995	0.00692839	0.02343175	Bdh1
ENSMUSG00000046603.17	429.931679	-0.0079028	0.00215896	13.0561283	0.00030229	0.00153529	Tcaim
ENSMUSG00000046605.15	50.9961498	0.00530481	0.00342107	2.38465991	0.12253156	0.24623237	B3gntl1
ENSMUSG00000046607.7	6.12337708	0.02508899	0.01087233	5.169365	0.02298862	0.0638946	Hrk
ENSMUSG00000046618.8	7.86652685	-0.0035017	0.0086171	0.15848818	0.69055225	0.8431396	Olfml2a
ENSMUSG00000046637.4	0.44505794	0.0169902	0.03136197	0.67151422	0.41252418	0.60397183	Ttc34
ENSMUSG00000046650.5	1.89112733	-0.0339296	0.02616778	2.41162357	0.1204374	0.24291401	Or5an6
ENSMUSG00000046658.17	83.8727669	0.00721979	0.00344733	4.31568616	0.03776254	0.09591506	Zfp316
ENSMUSG00000046667.15	169.137903	-0.0086433	0.00416669	4.17559131	0.04101025	0.10272597	Rbm12b1
ENSMUSG00000046668.10	658.00407	-0.003469	0.00255193	1.84391618	0.17449264	0.32271232	Cxxc5
ENSMUSG00000046671.19	1264.08842	-0.0080105	0.00185744	18.7133974	1.52E-05	0.00010419	Mtfr1l
ENSMUSG00000046675.8	120.499451	-0.0014801	0.00285283	0.26828088	0.60448731	0.77752292	Lyset
ENSMUSG00000046679.17	215.44067	0.00081026	0.00268025	0.09209414	0.76153158	0.89131623	C87436
ENSMUSG00000046688.15	497.387701	0.03609498	0.00587841	32.3292961	1.30E-08	1.55E-07	Tifa
ENSMUSG00000046691.15	618.432859	0.00248194	0.0038443	0.40460337	0.52472141	0.71016693	Chtf8
ENSMUSG00000046694.7	3.10267	0.01932233	0.01040774	3.44536791	0.0634293	0.14633073	Tent5b

ENSMUSG00000046699.15	3.86960387	0.02719007	0.01693083	2.60609511	0.1064536	0.22036287	Slitrk4
ENSMUSG00000046707.10	528.870213	-0.0070679	0.00334899	4.37819617	0.0364015	0.09323117	Csnk2a2
ENSMUSG00000046709.19	87.4489932	0.02484983	0.01605931	2.50300503	0.1136293	0.23195954	Mapk10
ENSMUSG00000046711.17	347.734925	0.00713878	0.00399068	3.1890454	0.07413329	0.16578248	Hmga1
ENSMUSG00000046714.8	2.57612426	0.01486537	0.01228744	1.51617051	0.21819993	0.38255137	Foxc2
ENSMUSG00000046718.9	2268.38171	0.00493412	0.00463647	1.14563956	0.28446381	0.46447652	Bst2
ENSMUSG00000046719.8	3.29241115	0.02051309	0.01403964	2.32950241	0.12694245	0.25311223	Nxph3
ENSMUSG00000046722.15	409.500385	0.01800897	0.00328124	29.6708018	5.12E-08	5.54E-07	Cdc42se1
ENSMUSG00000046727.14	163.732695	-6.30E-05	0.00415872	0.00022534	0.98802303	1	Cystm1
ENSMUSG00000046731.6	46.8879776	0.01312487	0.00401892	10.3328365	0.00130684	0.00554973	Kctd11
ENSMUSG00000046733.8	6.85319637	0.00851633	0.01144697	0.54081354	0.46209576	0.65329921	Gprc5a
ENSMUSG00000046743.7	60.8212808	-0.0041605	0.00493208	0.69885752	0.40316785	0.59472868	Fat4
ENSMUSG00000046750.19	2.61869355	-0.0247643	0.06831642	0.28428385	0.59390708	0.76915728	Selenov
ENSMUSG00000046753.11	124.269043	0.00360892	0.00466234	0.5763513	0.44774606	0.63964372	Ccdc66
ENSMUSG00000046755.6	6.16415069	-0.0012693	0.05497707	0.01404373	0.90566653	0.97866278	Kif2b
ENSMUSG00000046756.11	1115.30209	-0.0136296	0.00222954	37.6054512	8.66E-10	1.21E-08	Mrps7
ENSMUSG00000046761.14	260.79632	0.00382705	0.00324907	1.3369518	0.24757224	0.41885366	Fam83h
ENSMUSG00000046768.14	83.3055844	0.00174138	0.00417745	0.16637118	0.6833572	0.83793412	Rhoj
ENSMUSG00000046782.15	29.9202995	-0.0093764	0.00552115	2.77874069	0.09552325	0.2027913	Ttc6
ENSMUSG00000046785.9	576.668511	-0.0025425	0.00305491	0.68444383	0.4080607	0.59967726	Epm2aip1
ENSMUSG00000046791.9	96.3965626	-0.002262	0.00256096	0.77816882	0.37770177	0.56885664	Riox1
ENSMUSG00000046792.9	484.901871	-0.0106904	0.00312995	11.8119588	0.00058851	0.00275794	Zfp787
ENSMUSG00000046793.9	1.2584966	0.01808557	0.02535598	0.79083618	0.3738474	0.56472171	Gpr61
ENSMUSG00000046794.10	5093.43149	-0.0124403	0.00553351	5.10684258	0.02383165	0.06580137	Ppp1r3b
ENSMUSG00000046798.15	1778.76837	-0.0043671	0.00262687	2.70567143	0.09999198	0.21018919	Cldn12
ENSMUSG00000046805.11	1951.18601	0.06842158	0.00479369	187.018368	1.42E-42	1.34E-39	Mpeg1
ENSMUSG00000046806.14	101.25303	-0.0088853	0.0029917	8.79792408	0.00301573	0.01147799	Cyren
ENSMUSG00000046807.11	31.3309955	0.04066394	0.01107973	13.1247843	0.00029141	0.00148676	Lrrc75b
ENSMUSG00000046808.19	64.6805468	0.0284148	0.0042966	43.0489136	5.34E-11	8.90E-10	Atp10d
ENSMUSG00000046811.4	938.716223	-0.0093716	0.00373408	6.18871717	0.01285674	0.03934591	Gltpd2
ENSMUSG00000046814.4	2394.64878	-0.0052694	0.00462117	1.30075076	0.25407613	0.426401	Gchfr
ENSMUSG00000046818.8	38.0525542	0.02762688	0.00629802	17.9323716	2.29E-05	0.00015101	Ddit4l
ENSMUSG00000046822.15	174.231923	0.002083	0.00305591	0.46634147	0.4946751	0.68210505	Slc39a3
ENSMUSG00000046826.8	88.0273017	-0.0063663	0.00434715	2.07141634	0.15008209	0.28758113	Fam187b
ENSMUSG00000046834.8	2.14114725	0.01800436	0.0288076	0.67784291	0.4103305	0.60190654	Krt1
ENSMUSG00000046836.14	607.565889	0.00396531	0.00213562	3.4155581	0.06458457	0.14846898	Brox
ENSMUSG00000046841.7	95.3490248	0.02374732	0.00528513	19.8916682	8.20E-06	5.91E-05	Ckap4
ENSMUSG00000046844.7	4.45171422	0.05953474	0.01964159	9.63475154	0.0019093	0.00776025	Vat1l
ENSMUSG00000046846.5	1.04358636	-0.029312	0.04277992	0.68771337	0.40694324	0.59853849	Spesp1
ENSMUSG00000046854.17	2.10451714	0.00334696	0.05581057	0.02580374	0.87238055	0.95996741	Pip5kl1
ENSMUSG00000046861.10	959.782933	-0.0098793	0.00229275	18.7176991	1.52E-05	0.00010401	Hectd3
ENSMUSG00000046862.15	139.87204	0.00785611	0.0027867	7.75236471	0.00536423	0.01877161	Pramel12
ENSMUSG00000046865.8	547.254014	0.00058542	0.00306714	0.0367481	0.84797882	0.94597326	Fbl
ENSMUSG00000046873.19	199.964114	0.00823621	0.00330327	6.03223266	0.01404695	0.04247039	Mbtps2
ENSMUSG00000046876.17	543.746411	-0.0216267	0.00487053	18.5080931	1.69E-05	0.00011486	Atxn1
ENSMUSG00000046879.8	1134.40914	0.02091068	0.00327023	40.8775857	1.62E-10	2.52E-09	Irgm1
ENSMUSG00000046881.8	4.3415948	0.00210926	0.01864813	0.03912139	0.84320836	0.94257911	Or1ab2
ENSMUSG00000046897.18	540.806626	-0.0028144	0.00308178	0.82513461	0.36368319	0.55330141	Zfp740
ENSMUSG00000046908.6	8.07555105	0.01207461	0.00645673	3.45710359	0.06298055	0.14550487	Ltb4r1
ENSMUSG00000046909.10	267.975224	-0.0149912	0.00304718	24.2452943	8.48E-07	7.37E-06	Tefm
ENSMUSG00000046916.10	89.1034996	-0.003842	0.00356426	1.13066334	0.28763389	0.46861558	Myct1
ENSMUSG00000046924.5	1.50500191	0.00319408	0.02617937	0.06953344	0.7920173	0.91118387	Vmn1r179
ENSMUSG00000046932.3	1.25344798	0.00633286	0.02845546	0.12277441	0.72604468	0.86776026	Vmn1r193
ENSMUSG00000046934.6	1.45541346	-0.014964	0.01377103	1.00775146	0.31544212	0.50084267	Csl
ENSMUSG00000046947.13	174.890383	-0.0009826	0.00331189	0.08801308	0.76671848	0.89479575	Adck2
ENSMUSG00000046949.17	2448.10875	-0.0025489	0.0026006	0.95933163	0.32735533	0.5142193	Nqo2
ENSMUSG00000046957.6	6.75134215	-0.0609682	0.05692525	0.11763159	0.73161755	0.87129167	Spz1
ENSMUSG00000046959.17	3249.98744	0.00033849	0.00414626	0.00675229	0.93450969	0.99423346	Slc26a1
ENSMUSG00000046962.17	192.977383	-0.0103574	0.00409284	6.23973496	0.01249152	0.03839261	Zbtb21
ENSMUSG00000046971.8	3.18511285	0.04703494	0.01483715	10.8518295	0.00098698	0.00434309	Pla2g4f
ENSMUSG00000046982.12	449.017958	-0.0098001	0.00383673	6.46333098	0.0110123	0.03463149	Tshz1

ENSMUSG00000046985.12	1192.96247	0.00098084	0.00231543	0.17977605	0.67156577	0.82938268	Tapt1
ENSMUSG00000046991.13	12.0368168	-0.006183	0.00811684	0.5779882	0.44710196	0.63890991	Wdr27
ENSMUSG00000046994.10	93.0289162	-0.0012373	0.00295378	0.1732817	0.67721158	0.83315347	Mars2
ENSMUSG00000046997.6	76.5389788	-0.0062322	0.0052923	1.26467972	0.26076714	0.43490693	Spsb4
ENSMUSG00000046999.3	3.06741272	0.0128769	0.02452261	0.39734717	0.52846248	0.71310236	1110032F04Rik
ENSMUSG00000047003.16	50.6898321	0.00804976	0.00422526	3.62439705	0.0569382	0.13415053	Zfp41
ENSMUSG00000047013.16	3.63673644	0.03383604	0.01991527	3.71869456	0.05380642	0.12811208	Fbxo41
ENSMUSG00000047021.15	2.61175728	-0.0016142	0.01968648	-0.0217395	1	1	Cfap65
ENSMUSG00000047022.19	52.9878832	-0.0001854	0.00402125	0.00210241	0.96342819	1	Mipol1
ENSMUSG00000047025.6	1.71756773	-0.0105606	0.06709837	0.01141644	0.91490978	0.98336958	Ccer1
ENSMUSG00000047030.16	203.466684	-0.007486	0.00315384	5.62251994	0.01773114	0.05154136	Spata2
ENSMUSG00000047031.6	2.65274252	-0.0083537	0.02149891	0.17926623	0.67200459	0.82966396	Vmn1r68
ENSMUSG00000047036.9	1903.37911	-0.0003268	0.0036513	0.00760484	0.93050796	0.99195303	Zfp445
ENSMUSG00000047037.6	33.9843981	0.00376249	0.00545783	0.4943685	0.48198506	0.67131804	Nipa1
ENSMUSG00000047040.10	13.5351067	0.01922055	0.00721128	7.27041164	0.00700997	0.02365001	Prr15l
ENSMUSG00000047044.8	165.214826	-0.0113187	0.00293948	14.6785961	0.00012749	0.00071101	D030056L22Rik
ENSMUSG00000047045.18	205.25265	0.01236781	0.00354052	11.9344305	0.00055106	0.00260571	Tmem164
ENSMUSG00000047048.5	1.30493564	-0.0073035	0.03194249	-0.0447758	1	1	Or10aa3
ENSMUSG00000047067.8	126.987681	-0.0037263	0.00289538	1.66693026	0.19667021	0.35350278	Dusp28
ENSMUSG00000047084.9	290.711802	-0.0113417	0.00226451	25.1830031	5.21E-07	4.73E-06	Ngrn
ENSMUSG00000047085.15	2.85077432	0.00540282	0.02002713	0.15568191	0.6931636	0.84495099	Lrrc4b
ENSMUSG00000047090.14	84.5533435	-0.0018617	0.00312441	0.36678585	0.54476149	0.7280733	Tmem198b
ENSMUSG00000047098.19	309.946376	0.00038822	0.00399263	0.0093386	0.92301513	0.98811936	Rnf31
ENSMUSG00000047108.5	3.49866717	0.01604082	0.01205344	1.78493802	0.18154413	0.33262642	Dnajb7
ENSMUSG00000047109.14	198.802278	-0.0058982	0.00531344	1.17950644	0.2774565	0.45628604	Cldn14
ENSMUSG00000047115.16	7.84485238	-0.0034734	0.00838766	0.1527997	0.69587409	0.84681077	Fam221a
ENSMUSG00000047117.14	0.65055588	0.00309568	0.02497998	0.02927063	0.86415567	0.95533047	Ankdd1b
ENSMUSG00000047123.9	258.152546	-6.91E-05	0.0025314	0.00074189	0.97827023	1	Ticam1
ENSMUSG00000047126.18	3644.92409	0.00623909	0.00233775	7.00127892	0.00814515	0.02681624	Cltc
ENSMUSG00000047139.10	119.991151	0.06437414	0.00704614	70.1087105	5.61E-17	2.02E-15	Cd24a
ENSMUSG00000047141.6	324.239476	-0.0030712	0.00281781	1.1809066	0.27717151	0.45594694	Zfp654
ENSMUSG00000047143.4	1.5921263	0.01357829	0.01430614	0.95994242	0.32720139	0.51404595	Dmrta2
ENSMUSG00000047146.18	25.8049956	0.01505239	0.01113322	1.39689755	0.23724371	0.40618686	Tet1
ENSMUSG00000047150.9	212.450635	0.00552928	0.0040892	1.87643732	0.17073962	0.31719161	1700001C19Rik
ENSMUSG00000047153.5	719.569567	-0.0061212	0.00363847	2.74883478	0.09732533	0.20582043	Khnyn
ENSMUSG00000047155.14	2.04448759	0.00337454	0.01903019	-0.0219916	1	1	Cyp4x1
ENSMUSG00000047161.13	1.19336346	0.04040378	0.01550348	6.71851358	0.00954171	0.03067341	Chst9
ENSMUSG00000047180.9	156.555007	0.03078711	0.00444549	45.2151264	1.77E-11	3.15E-10	Neurl3
ENSMUSG00000047181.13	17.5771795	0.03345318	0.00707744	21.5474608	3.45E-06	2.70E-05	Samd14
ENSMUSG00000047182.7	4.01676894	0.04636621	0.00841882	29.4502553	5.74E-08	6.17E-07	Irs3
ENSMUSG00000047187.10	1950.69803	-4.09E-05	0.00163432	0.00061982	0.9801378	1	Rab2a
ENSMUSG00000047193.17	137.977366	0.00151017	0.00425682	0.12532754	0.72332667	0.86573362	Dync2h1
ENSMUSG00000047205.13	17.0782704	0.0231034	0.01195016	3.54663431	0.05966636	0.13923226	Dusp18
ENSMUSG00000047213.15	1452.77478	-0.0025301	0.00213205	1.39493859	0.23757286	0.40666183	Ythdf3
ENSMUSG00000047215.15	8213.89899	-0.0014335	0.00440281	0.10786106	0.74259256	0.87824239	Rpl9
ENSMUSG00000047216.9	22.3079862	0.16052919	0.00888652	260.350043	1.44E-58	5.66E-55	Cdh19
ENSMUSG00000047220.6	2.45510962	0.02592258	0.01574431	2.67198676	0.10212846	0.21358414	Iho1
ENSMUSG00000047221.6	130.2669	-0.0114701	0.00291816	15.1727212	9.81E-05	0.00056139	Fam185a
ENSMUSG00000047227.6	16.0443781	-0.0048569	0.00627933	0.56412957	0.45260115	0.64439519	Gm527
ENSMUSG00000047228.10	388.258601	0.03201192	0.00626894	25.3604457	4.76E-07	4.35E-06	A2ml1
ENSMUSG00000047230.7	1607.02517	0.00706349	0.00446694	2.45864175	0.11687981	0.23728071	Cldn2
ENSMUSG00000047238.5	49.5237275	-0.0042901	0.00366815	1.38776335	0.23878318	0.40817076	Mageh1
ENSMUSG00000047242.15	25.782497	0.02232351	0.00475434	20.8888786	4.87E-06	3.69E-05	Taf9b
ENSMUSG00000047246.8	40.4079837	-0.0106176	0.00644983	2.37009914	0.1236792	0.24805251	H2bc6
ENSMUSG00000047248.21	223.140451	-0.0069144	0.00403923	2.86849983	0.09032903	0.19405233	C2cd3
ENSMUSG00000047250.14	292.238486	0.02812677	0.00418799	43.5768699	4.08E-11	6.89E-10	Ptgs1
ENSMUSG00000047260.5	1210.612	-0.011816	0.00290412	16.528501	4.79E-05	0.00029567	Emc6
ENSMUSG00000047261.10	34.4728796	0.02853325	0.02095458	2.59668153	0.10708773	0.22138643	Gap43
ENSMUSG00000047264.9	172.110995	0.00117134	0.0030037	0.15351855	0.69519533	0.84646519	Zfp358
ENSMUSG00000047281.4	14.4626068	0.01591812	0.00514571	9.55024283	0.00199923	0.00808121	Sfn
ENSMUSG00000047284.15	319.204906	-0.0068183	0.00360178	3.59071307	0.05810331	0.1364557	Neurl4

ENSMUSG00000047298.4	1.699347	-0.0008853	0.03614538	-0.0035969	1	1	Kcnv2
ENSMUSG00000047307.3	1.8413162	0.04277343	0.01145326	14.4008789	0.00014773	0.00081203	Pcdhb13
ENSMUSG00000047330.9	10.0425872	0.03488892	0.00705018	22.3811757	2.24E-06	1.81E-05	Kcne4
ENSMUSG00000047342.18	3.73968007	0.04162299	0.012792	9.8683671	0.00168145	0.00692485	Zfp286
ENSMUSG00000047343.5	2.34483867	-0.0162843	0.01831305	0.81953674	0.36531565	0.55506892	Mettl21c
ENSMUSG00000047344.10	2.48306334	0.03691078	0.01449542	6.41141755	0.01133889	0.03546465	Lancl3
ENSMUSG00000047361.17	4.12465247	0.03653548	0.02275664	2.39773432	0.12151112	0.24461944	Gm973
ENSMUSG00000047368.5	462.344318	-0.0120097	0.00321572	13.7965723	0.00020371	0.00108433	Abhd17b
ENSMUSG00000047369.18	13.4584196	-0.0116651	0.01103567	0.76994244	0.38023486	0.57169755	Dnah14
ENSMUSG00000047371.8	297.41515	-0.0068853	0.00242792	8.06365047	0.00451621	0.01621299	Zfp768
ENSMUSG00000047379.8	312.140526	-0.0029307	0.00239282	1.52056156	0.21753453	0.38166933	B4gat1
ENSMUSG00000047388.11	174.167271	0.00416179	0.00246822	2.80857147	0.09376183	0.19992142	Atmin
ENSMUSG00000047394.10	79.2188364	-0.0213907	0.00531705	16.1707831	5.79E-05	0.00035004	Odf3b
ENSMUSG00000047407.18	284.699394	0.0091339	0.00351366	6.63703361	0.00998801	0.0318649	Tgif1
ENSMUSG00000047409.14	110.942883	0.008436	0.00312295	7.13094299	0.00757649	0.02522928	Ctdspl
ENSMUSG00000047412.17	962.208685	0.00350824	0.00299487	1.32116802	0.25038254	0.42206421	Zbtb44
ENSMUSG00000047414.7	27.4859793	0.01955502	0.00748391	6.55946281	0.01043279	0.03304252	Flrt2
ENSMUSG00000047415.13	17.08274	0.06942244	0.00899128	50.6734143	1.09E-12	2.28E-11	Gpr68
ENSMUSG00000047417.19	450.022891	-0.0097367	0.00348554	7.71482318	0.00547692	0.01912625	Rexo1
ENSMUSG00000047419.6	170.931216	0.04185775	0.01534193	0.98257398	0.32156416	0.50776007	Cmya5
ENSMUSG00000047420.9	32.1105321	0.04325257	0.0056437	57.3964228	3.56E-14	9.09E-13	Fam180a
ENSMUSG00000047423.11	242.46742	-0.0004139	0.00198009	0.0436467	0.83451231	0.93700883	AI837181
ENSMUSG00000047434.15	57.9431168	0.01316057	0.00385262	11.4139732	0.00072894	0.00332805	Xxylt1
ENSMUSG00000047446.19	984.939055	-0.0152329	0.00375387	15.8840674	6.73E-05	0.0004009	Arl4a
ENSMUSG00000047454.13	1074.67734	-0.0067483	0.00280641	5.73998633	0.01658292	0.04874437	Gphn
ENSMUSG00000047459.15	1030.21639	-0.0074963	0.00256241	8.44392907	0.00366264	0.01354666	Dynlrb1
ENSMUSG00000047462.10	4.39704269	0.05209006	0.00785708	42.7133451	6.34E-11	1.05E-09	Gpr141b
ENSMUSG00000047466.11	237.65927	-0.0033931	0.00235903	2.02980362	0.15424054	0.29354614	Ark2n
ENSMUSG00000047473.15	61.0050369	-0.0052128	0.0037566	1.94050177	0.16361406	0.30680564	Zfp30
ENSMUSG00000047485.7	0.95913076	-0.0168826	0.05999344	0.06246206	0.80264604	0.91721448	Klhl34
ENSMUSG00000047492.6	936.761856	-0.0008412	0.00600501	0.01761334	0.89441856	0.97287826	Inhbe
ENSMUSG00000047495.16	3.32346208	0.00709435	0.02317572	0.19266942	0.66070411	0.82159984	Dlgap2
ENSMUSG00000047496.7	448.010425	-0.0043763	0.00518147	0.69936758	0.40299628	0.5946243	Rnf152
ENSMUSG00000047497.11	15.5955777	0.03172674	0.00828847	14.0669403	0.00017642	0.00095633	Adamts12
ENSMUSG00000047501.4	4.27452792	0.04495511	0.0124505	12.7662021	0.00035294	0.00175917	Cldn4
ENSMUSG00000047502.15	8.18549914	-0.0090153	0.00816873	1.1306604	0.28763452	0.46861558	Mroh7
ENSMUSG00000047507.14	8.77575022	0.0999853	0.00951974	90.8759994	1.53E-21	9.54E-20	Baiap3
ENSMUSG00000047511.7	6.1029144	-0.0135166	0.01099784	1.38707965	0.2388989	0.40830937	Or2v2
ENSMUSG00000047514.7	511.680212	-0.0003529	0.00188863	0.03439094	0.85287771	0.94931447	Tspyl1
ENSMUSG00000047515.9	10.3034701	-0.015729	0.00933584	2.64194048	0.10407635	0.21644871	BC049715
ENSMUSG00000047517.14	64.9072929	0.02009867	0.02349384	-1.4844453	1	1	Dmbt1
ENSMUSG00000047528.14	3.47774043	-0.028333	0.01226425	5.02662141	0.02496054	0.06834449	Flacc1
ENSMUSG00000047534.18	13.9580141	0.02775287	0.00997611	7.01357939	0.00808938	0.02665495	Mis18bp1
ENSMUSG00000047535.12	1.36992101	-0.0052609	0.04413218	-0.0230953	1	1	Or52z1
ENSMUSG00000047539.10	212.066974	-0.0056841	0.00273899	4.23658154	0.03956175	0.09971184	Fbxo28
ENSMUSG00000047547.15	939.46262	-0.0054633	0.00274076	3.92525338	0.04756602	0.11582616	Cltb
ENSMUSG00000047554.15	601.391717	-0.0039971	0.00240609	2.79171246	0.09475292	0.20159141	Tmem41b
ENSMUSG00000047557.3	21.5219174	0.01911897	0.00713571	7.09601325	0.00772556	0.02563521	Lxn
ENSMUSG00000047583.10	42.8677708	-0.0072509	0.00652035	1.14644702	0.28429415	0.4643924	Tyw3
ENSMUSG00000047586.5	2.30906793	0.03227152	0.01564692	5.14139615	0.02336185	0.06472603	Nccrp1
ENSMUSG00000047591.6	2.73339569	0.03135223	0.01628331	4.07688102	0.04347384	0.1076508	Mafa
ENSMUSG00000047592.18	3.49460056	0.10809064	0.01548568	39.0654123	4.10E-10	6.02E-09	Nxpe5
ENSMUSG00000047594.5	1.42070672	0.01139777	0.02716624	0.34956219	0.55436107	0.73654575	Or10ag57
ENSMUSG00000047603.10	103.989517	-0.0085665	0.0024118	12.6994378	0.00036577	0.00181209	Zfp235
ENSMUSG00000047604.4	31.2975822	-0.0137472	0.003654	14.2916481	0.00015656	0.00085615	Frat2
ENSMUSG00000047606.5	2.02705834	0.03879124	0.02210518	3.50363418	0.06123432	0.14225082	Ankrd34c
ENSMUSG00000047613.11	682.436781	-0.0082064	0.00341424	5.7878331	0.01613747	0.04765473	A430005L14Rik
ENSMUSG00000047617.12	294.00193	-0.0244528	0.00549627	20.0812481	7.42E-06	5.41E-05	Paxx
ENSMUSG00000047619.6	3.07109708	-0.0137206	0.01861853	0.58281901	0.44520948	0.63752721	Ddi1
ENSMUSG00000047631.5	9772.24744	-0.0067486	0.00515103	1.7462988	0.1863427	0.33933593	Apof
ENSMUSG00000047632.12	14.9853731	0.01551008	0.00607588	6.39616591	0.01143671	0.03569488	Fgfbp3

ENSMUSG00000047635.7	226.388241	-0.007219	0.0033193	4.69355377	0.03027597	0.08011769	Mtrfr
ENSMUSG00000047638.16	1906.92678	-0.0059268	0.0039832	2.21342476	0.13681465	0.2680826	Nr1h4
ENSMUSG00000047648.14	273.744361	-0.01112	0.00279679	15.6123892	7.77E-05	0.00045545	Fbxo30
ENSMUSG00000047649.10	122.704393	-0.0014639	0.00424213	0.11701551	0.73229423	0.87157259	Polr1g
ENSMUSG00000047654.7	2.21302395	-0.002851	0.0121989	0.06295419	0.80188621	0.91696345	Tssk6
ENSMUSG00000047655.3	1.55987941	-0.0106675	0.03324875	0.17287428	0.67756988	0.83348092	Vmn1r74
ENSMUSG00000047656.10	44.0775866	-0.0065976	0.00357282	3.52266727	0.06053495	0.1409872	Trpt1
ENSMUSG00000047658.7	2.3732897	0.02778095	0.02912331	1.2682205	0.26010078	0.4340411	Gal3st3
ENSMUSG00000047669.9	18.5306525	0.029329	0.00427373	44.509766	2.53E-11	4.40E-10	Msl3l2
ENSMUSG00000047671.9	1.02117607	0.02459685	0.01563892	2.67477325	0.10194985	0.21338087	Dynlt4
ENSMUSG00000047675.16	8710.22939	-0.0074026	0.0051789	2.04824931	0.15238133	0.29084283	Rps8
ENSMUSG00000047694.13	660.288597	0.00172927	0.00239351	0.51112483	0.47465233	0.66526918	Yipf6
ENSMUSG00000047696.17	2.2933725	-0.0042799	0.02573425	0.01572263	0.90021488	0.97587825	Ccdc144b
ENSMUSG00000047710.9	156.567837	0.00569327	0.00240822	5.46015361	0.01945494	0.05562072	Champ1
ENSMUSG00000047712.9	24.3519707	0.04350861	0.00545833	60.9075269	5.98E-15	1.69E-13	Ust
ENSMUSG00000047714.15	935.77317	-0.0032366	0.00232321	1.94109125	0.1635501	0.30671007	Ppp1r2
ENSMUSG00000047719.3	179.186698	-0.0054536	0.00231574	5.53014957	0.01869151	0.05383538	Ubiad1
ENSMUSG00000047720.8	2.9847456	0.00472417	0.08044743	-0.0069796	1	1	Clec2m
ENSMUSG00000047721.9	600.958184	-0.007776	0.0045057	2.98585207	0.08399507	0.18319842	Bola2
ENSMUSG00000047728.14	3329.39717	-0.0090911	0.00536315	2.82220138	0.09296882	0.19858382	Ly6g2
ENSMUSG00000047730.18	11.9379484	0.03712472	0.00979904	13.2895611	0.00026689	0.00137652	Fcgbp
ENSMUSG00000047731.18	4407.49517	-0.0065069	0.00262213	6.10270559	0.01349751	0.04100892	Wbp1l
ENSMUSG00000047735.15	487.435693	0.04572311	0.004792	88.4274774	5.27E-21	3.03E-19	Samd9l
ENSMUSG00000047746.15	72.1235091	0.09012865	0.00834875	91.7145472	1.00E-21	6.35E-20	Fbxo40
ENSMUSG00000047747.11	96.9031035	0.02414703	0.0054131	19.1096994	1.23E-05	8.59E-05	Rnf150
ENSMUSG00000047749.3	12.9131524	0.01358975	0.00609762	4.89248058	0.0269739	0.07287589	Zc3hav1l
ENSMUSG00000047757.18	18.2884525	0.01171068	0.00486311	5.66069276	0.01734927	0.05060597	Fancb
ENSMUSG00000047759.7	60.8137554	0.02076422	0.00459409	19.6913933	9.10E-06	6.51E-05	Hs3st3a1
ENSMUSG00000047766.16	17.2103398	0.02686433	0.00772804	11.4834651	0.00070218	0.00322086	Lrrc49
ENSMUSG00000047767.18	289.30808	-0.0129017	0.00475937	6.9298447	0.0084769	0.02774966	Atg16l2
ENSMUSG00000047777.10	110.656014	0.00159571	0.00250268	0.40776384	0.52310668	0.70883569	Phf13
ENSMUSG00000047786.13	11.3805123	0.02110313	0.00715713	8.29086394	0.0039845	0.01457031	Lix1
ENSMUSG00000047787.9	20.3693989	-0.0050737	0.00670006	0.57793427	0.44712316	0.63890991	Flrt1
ENSMUSG00000047789.6	216.40189	-0.00106	0.00288217	0.13368625	0.71464021	0.85936335	Slc38a9
ENSMUSG00000047793.14	261.843394	-0.0187461	0.00497287	14.0598539	0.00017708	0.00095928	Sned1
ENSMUSG00000047797.15	10159.5154	-0.0165352	0.00480735	11.7961993	0.00059352	0.00277752	Gjb1
ENSMUSG00000047798.16	69.6114172	0.07074042	0.00761568	74.290981	6.74E-18	2.69E-16	Cd300lf
ENSMUSG00000047804.16	272.1453	-0.00173	0.00263293	0.4269389	0.51349482	0.70015544	Akap10
ENSMUSG00000047810.10	55.7257602	0.04795496	0.00618008	56.3461627	6.08E-14	1.49E-12	Ccdc88b
ENSMUSG00000047821.17	12.3078117	0.02434738	0.0085184	7.80269307	0.00521684	0.01834568	Trim16
ENSMUSG00000047822.9	1114.61696	0.01465486	0.00756642	3.78403474	0.05174375	0.12398944	Angptl8
ENSMUSG00000047824.14	269.116693	-0.0011017	0.00277243	0.15876387	0.69029715	0.84302534	Pygo2
ENSMUSG00000047832.10	76.7326017	0.00894247	0.00343629	6.75203261	0.00936409	0.03023016	Cdca4
ENSMUSG00000047842.8	10.1510388	0.0967935	0.01264504	50.7092257	1.07E-12	2.24E-11	Diras2
ENSMUSG00000047843.17	991.4466	-0.0041367	0.0041222	1.01503235	0.31370025	0.4991186	Bri3
ENSMUSG00000047844.4	9.0742625	-0.0135825	0.01011441	1.89115491	0.16907172	0.3147873	Bex4
ENSMUSG00000047854.6	1.41062061	0.00701048	0.01764461	0.27433795	0.60043657	0.77438195	Stx19
ENSMUSG00000047866.21	5064.33547	-0.0196022	0.00276665	50.2270482	1.37E-12	2.82E-11	Lonp2
ENSMUSG00000047867.12	258.71596	0.01484838	0.0035726	16.5156412	4.83E-05	0.00029729	Gimap6
ENSMUSG00000047875.7	76.9837935	-0.0040118	0.00401342	0.98962761	0.31983341	0.50583992	Gpr157
ENSMUSG00000047878.14	15.3957114	0.06287334	0.00752164	59.9075373	9.94E-15	2.73E-13	A4galt
ENSMUSG00000047879.18	792.5743	-0.0095381	0.00185301	26.1470342	3.16E-07	2.99E-06	Usp14
ENSMUSG00000047880.16	13.4658571	0.11929399	0.00915529	132.688348	1.06E-30	2.47E-28	Cxcr5
ENSMUSG00000047881.15	302.773842	0.01164689	0.00316868	13.2188672	0.00027715	0.0014232	Rell1
ENSMUSG00000047888.11	1096.15686	-0.0044191	0.00523516	0.69635391	0.40401155	0.59561529	Tnrc6b
ENSMUSG00000047904.7	22.9757653	0.00174671	0.00618943	0.07688021	0.78157063	0.90441296	Sstr2
ENSMUSG00000047907.12	363.961828	0.00184273	0.0044301	0.16792624	0.68196143	0.83718016	Tshz2
ENSMUSG00000047909.12	271.094695	0.00139114	0.00374219	0.13972999	0.70854959	0.85578915	Ankrd16
ENSMUSG00000047910.7	9.48237642	0.02745543	0.00610932	20.2937518	6.64E-06	4.90E-05	Pcdhb16
ENSMUSG00000047911.7	1.83450907	0.00320474	0.0125591	0.07545819	0.78354939	0.90578817	Npm2
ENSMUSG00000047921.19	227.828653	-0.0023149	0.00252517	0.84184422	0.35886986	0.54785142	Trappc9

ENSMUSG00000047945.7	241.50009	0.02876409	0.00420356	44.9146838	2.06E-11	3.64E-10	Marcksl1
ENSMUSG00000047953.8	3.41463766	0.04476548	0.00956319	21.6546559	3.26E-06	2.57E-05	Gp5
ENSMUSG00000047959.5	2.48373413	0.07619061	0.01273668	33.6190537	6.70E-09	8.32E-08	Kcna3
ENSMUSG00000047963.8	1573.53681	0.00175703	0.00333523	0.27843871	0.59772637	0.7723332	Stbd1
ENSMUSG00000047976.5	6.27946897	0.00214012	0.01943619	0.02490184	0.87461189	0.96125797	Kcna1
ENSMUSG00000047986.13	46.9826169	0.02404367	0.01626261	2.84299807	0.09177284	0.19650248	Palm3
ENSMUSG00000047989.12	272.357438	0.0089796	0.00197705	20.2790797	6.69E-06	4.93E-05	Ino80c
ENSMUSG00000047996.17	35.1337488	0.01512478	0.00522521	8.30170151	0.0039608	0.01449035	Prrg1
ENSMUSG00000048000.16	975.15057	-0.0032883	0.00282288	1.32702461	0.24933523	0.42084019	Gigyf2
ENSMUSG00000048004.16	3.20539577	0.01447479	0.0279491	0.47498362	0.49070336	0.67833369	Tmem196
ENSMUSG00000048007.5	638.892784	-0.0122912	0.00363059	11.8273427	0.00058367	0.00273851	Timm8a1
ENSMUSG00000048012.20	4.51878762	-0.0053059	0.00895327	0.30631946	0.57994754	0.75789105	Zfp473
ENSMUSG00000048022.12	55.2153303	0.03395009	0.00669913	24.4093055	7.79E-07	6.80E-06	Tmem229a
ENSMUSG00000048027.10	91.6057223	0.01056641	0.00337262	9.62281812	0.00192175	0.00780546	Rgmb
ENSMUSG00000048029.12	1.84289643	0.01673309	0.01518208	1.53883089	0.21479191	0.37781206	Eno4
ENSMUSG00000048031.16	4.19441207	0.18270316	0.01888687	57.9228416	2.73E-14	7.07E-13	Fcrl5
ENSMUSG00000048038.9	2.18992212	0.00322006	0.01161857	0.07752822	0.78067546	0.90388353	Ccdc187
ENSMUSG00000048039.9	302.006592	-0.000305	0.00189131	0.02581217	0.87235988	0.95996741	Isg20l2
ENSMUSG00000048040.9	6.32769671	0.040781	0.01241662	10.6076472	0.00112621	0.0048748	Arxes2
ENSMUSG00000048047.4	406.729727	0.00047726	0.00285903	0.02740407	0.86851759	0.95800441	Zbtb33
ENSMUSG00000048058.18	177.691641	0.02210534	0.00349356	39.9570978	2.60E-10	3.92E-09	Ldlrad3
ENSMUSG00000048067.9	5.88664862	-0.0052675	0.03724891	-0.0062852	1	1	Or10am5
ENSMUSG00000048070.5	1.39167256	-0.015885	0.03591099	0.21597911	0.64212087	0.8081677	Pirt
ENSMUSG00000048076.15	4335.26885	0.00245527	0.00237222	1.0705085	0.30083062	0.48521743	Arf1
ENSMUSG00000048077.7	3.85322051	-0.0063532	0.0657789	0.01904949	0.89022471	0.9700998	H1f7
ENSMUSG00000048078.17	16.7291362	0.04487774	0.0112863	13.690754	0.00021551	0.00114022	Tenm4
ENSMUSG00000048087.7	2133.76073	-0.0017884	0.00390084	0.20925348	0.64735315	0.81156057	Ahcyl
ENSMUSG00000048096.8	17.2798633	0.00031276	0.00642213	0.00231602	0.9616166	1	Lmod1
ENSMUSG00000048100.14	346.56339	-0.0136015	0.00262712	26.2231723	3.04E-07	2.89E-06	Taf13
ENSMUSG00000048108.14	11.8090148	0.02972315	0.0108914	7.05591353	0.0079004	0.02611251	Tmem72
ENSMUSG00000048109.11	270.377897	-0.0032598	0.0026261	1.50184806	0.22038723	0.38512656	Rbm15
ENSMUSG00000048118.18	1099.26368	0.00420591	0.00409778	1.03774839	0.30834572	0.49327445	Arid4a
ENSMUSG00000048120.17	204.181843	0.04001703	0.00697245	30.5889946	3.19E-08	3.56E-07	Entpd1
ENSMUSG00000048126.17	269.916243	0.03820423	0.00615835	37.2147007	1.06E-09	1.46E-08	Col6a3
ENSMUSG00000048138.11	5.51807271	-0.0435298	0.00838956	25.8783792	3.64E-07	3.41E-06	Dmrt2
ENSMUSG00000048142.10	16.6682994	0.03910785	0.00967606	15.9156191	6.62E-05	0.00039537	Nat8l
ENSMUSG00000048148.19	3.64212082	0.04937909	0.01625319	10.3500575	0.00129471	0.00550711	Nwd1
ENSMUSG00000048154.18	1151.44427	9.80E-05	0.00408039	0.00055281	0.98124186	1	Kmt2d
ENSMUSG00000048163.14	83.3100098	0.05651085	0.005511	98.9040029	2.65E-23	2.03E-21	Selplg
ENSMUSG00000048170.15	562.321774	0.00097497	0.00311528	0.09719872	0.75521754	0.88681641	Mcmbp
ENSMUSG00000048174.3	33.8658201	-0.0028202	0.00342075	0.6732659	0.41191528	0.60340561	Tmem81
ENSMUSG00000048175.14	444.209547	-0.0113499	0.00191434	34.8138208	3.63E-09	4.68E-08	Asb8
ENSMUSG00000048186.15	160.327205	-0.0140222	0.00281699	25.0797228	5.50E-07	4.97E-06	Bend7
ENSMUSG00000048200.13	62.4253377	0.00662087	0.005356	1.5205584	0.21753501	0.38166933	Cracr2b
ENSMUSG00000048206.7	1.67073649	-0.033274	0.06323014	0.50092201	0.47909528	0.66907107	Dnajb8
ENSMUSG00000048216.14	5.11614355	0.08299301	0.01586963	24.410738	7.78E-07	6.80E-06	Gpr85
ENSMUSG00000048217.12	1085.86061	-0.0031875	0.00400291	0.62577895	0.42890786	0.62036064	Nags
ENSMUSG00000048218.7	134.319506	0.00603802	0.00320139	3.46201802	0.06279363	0.14517258	Amigo2
ENSMUSG00000048222.4	435.121002	-0.0038176	0.00245807	2.39472908	0.12174484	0.2450272	Mfap1b
ENSMUSG00000048230.7	37.415872	-0.0203959	0.01528196	2.23467548	0.13494495	0.26525641	Fbxo43
ENSMUSG00000048232.13	25.6310462	0.01439063	0.00500213	8.11493714	0.0043902	0.01584503	Fbxo10
ENSMUSG00000048234.14	1415.05017	0.00594853	0.00276084	4.60299601	0.03191613	0.08371596	Rnf149
ENSMUSG00000048240.16	43.6088617	0.02804161	0.00760721	13.1555765	0.00028666	0.00146634	Gng7
ENSMUSG00000048249.15	659.16461	0.00850713	0.00458014	3.35721421	0.06691117	0.152674	Crebrf
ENSMUSG00000048251.16	24.1230092	0.08953107	0.00831202	103.605442	2.47E-24	2.15E-22	Bcl11b
ENSMUSG00000048264.17	338.349343	-0.0016834	0.00320511	0.27676815	0.59882735	0.77320446	Dip2c
ENSMUSG00000048271.15	962.1846	-0.0109824	0.00311078	12.3537633	0.0004401	0.0021332	Rbm33
ENSMUSG00000048277.16	982.610307	-0.0014142	0.00243954	0.33698987	0.5615719	0.74269012	Syngr2
ENSMUSG00000048279.20	73.762856	0.00130759	0.00563691	0.0520703	0.81949918	0.92756274	Sacs
ENSMUSG00000048280.19	233.084403	-0.0085648	0.00410639	4.3198924	0.0376693	0.09572979	Zfp738
ENSMUSG00000048281.10	3.09693536	0.07487629	0.02280763	8.63482602	0.00329797	0.0123922	Dleu7

ENSMUSG00000048285.10	129.730107	0.00921361	0.003848	5.51304917	0.01887511	0.05425157	Frmd6
ENSMUSG00000048304.9	2.52982861	-0.0003158	0.02740936	-0.0001573	1	1	Slitrk3
ENSMUSG00000048307.9	881.986438	-0.0100735	0.0022505	19.7134333	9.00E-06	6.45E-05	Ankrd46
ENSMUSG00000048310.9	558.189493	-0.0031645	0.00208177	2.3045978	0.12899167	0.25622347	Pskh1
ENSMUSG00000048327.7	20.6327888	0.01486121	0.01045744	1.91224991	0.16671355	0.31137993	Ckap2l
ENSMUSG00000048329.8	1.34947738	0.02505284	0.01224785	4.43402101	0.03522942	0.09072181	Mfsd6l
ENSMUSG00000048330.15	28.3843754	0.05037754	0.00593148	62.1508667	3.18E-15	9.26E-14	Ric3
ENSMUSG00000048332.14	228.874555	0.01000878	0.00325563	9.27634675	0.00232132	0.00917557	Lhfpl6
ENSMUSG00000048347.6	4.33758585	0.01971297	0.00832313	5.51582572	0.01884518	0.05419855	Pcdhb18
ENSMUSG00000048349.10	5.66565515	-0.0026485	0.01242891	0.01296002	0.90936296	0.98051784	Pou4f1
ENSMUSG00000048351.15	454.848634	-0.0004118	0.00367718	0.01266169	0.9104078	0.98098286	Coa7
ENSMUSG00000048355.3	2.03658869	0.03484805	0.02909913	2.50632005	0.11339044	0.23161227	Arxes1
ENSMUSG00000048368.10	58.6849117	0.011079	0.00551353	3.65959489	0.05574719	0.13200011	Omd
ENSMUSG00000048371.9	831.040846	-0.0166262	0.00281696	33.6834237	6.49E-09	8.07E-08	Pdp2
ENSMUSG00000048376.7	677.829366	-0.0020365	0.00282739	0.49978279	0.47959558	0.66941303	F2r
ENSMUSG00000048379.10	521.646779	-0.0097293	0.00294051	10.6958186	0.00107378	0.00467531	Socs4
ENSMUSG00000048385.11	8.59056843	0.03174613	0.01933397	3.77657491	0.05197495	0.12436656	Scrt1
ENSMUSG00000048387.9	4.0875312	-0.0169029	0.01588703	1.27845887	0.25818578	0.43160889	Osr1
ENSMUSG00000048388.4	66.796139	0.05860228	0.00587276	90.294453	2.05E-21	1.24E-19	Fam171b
ENSMUSG00000048391.4	1.96487328	-0.0043671	0.02569152	-0.0073428	1	1	Or7g25
ENSMUSG00000048410.18	164.6715	0.00632948	0.00433763	2.0488595	0.15232026	0.29083021	Zfp407
ENSMUSG00000048416.16	44.5699071	0.00096349	0.01100553	-4.8740536	1	1	Mlf1
ENSMUSG00000048424.19	19.3883337	0.01389143	0.00882622	2.41805906	0.11994346	0.24212483	Ranbp3l
ENSMUSG00000048429.11	817.355769	-0.0070475	0.00178669	15.5007137	8.25E-05	0.0004807	Timm29
ENSMUSG00000048439.16	68.5256186	0.00367466	0.00328766	1.24327435	0.26484094	0.43995904	Nupl2
ENSMUSG00000048440.17	62.8249842	0.03529607	0.00583101	35.0696573	3.18E-09	4.14E-08	Cyp4f16
ENSMUSG00000048442.16	4.88074896	0.01637197	0.00938663	2.93883349	0.08647316	0.18754538	Smim5
ENSMUSG00000048445.7	44.6139299	0.00291919	0.004754	0.39081938	0.53186902	0.7164471	Ccdc57
ENSMUSG00000048450.11	5.69866189	-0.013018	0.00850294	2.20295366	0.13774658	0.26941627	Msx1
ENSMUSG00000048456.4	1.40920095	0.00054058	0.03323527	0.00711722	0.93276731	0.99323294	Or5b12
ENSMUSG00000048458.9	12.0764457	0.01910192	0.00935751	3.79543256	0.05139259	0.12328577	Inka2
ENSMUSG00000048478.8	20.0707021	-0.0313099	0.01088804	7.09945322	0.00771075	0.02558966	Spata33
ENSMUSG00000048481.16	34.8336272	-0.0044194	0.00430461	1.03385327	0.30925536	0.49434747	Mypop
ENSMUSG00000048482.15	45.9664936	0.01123552	0.00476636	5.2793811	0.02157942	0.0605775	Bdnf
ENSMUSG00000048483.7	8.06369842	-0.000132	0.02007252	0.00050214	0.98212205	1	Zdhhc22
ENSMUSG00000048485.13	4.1073404	0.07825745	0.01160592	39.8158658	2.79E-10	4.19E-09	Zbtb8b
ENSMUSG00000048486.7	1025.39401	-0.0220349	0.00365315	34.198648	4.98E-09	6.29E-08	Fitm2
ENSMUSG00000048489.13	1197.31547	-0.0004443	0.00601627	0.00525136	0.94223086	0.99817899	Depp1
ENSMUSG00000048490.15	454.554646	0.0151216	0.00397276	13.7432693	0.00020957	0.00111177	Nrip1
ENSMUSG00000048495.17	232.385378	-0.0150199	0.00395577	14.3024055	0.00015567	0.00085147	Tyw5
ENSMUSG00000048497.12	27.5790324	0.02907682	0.00630959	19.2555285	1.14E-05	8.01E-05	Mmgt2
ENSMUSG00000048498.9	68.2310859	0.06188976	0.00757023	59.7711175	1.07E-14	2.91E-13	Cd300e
ENSMUSG00000048503.8	16.5271435	0.00041522	0.0059572	0.00486903	0.94436999	0.99936785	Tlcd5
ENSMUSG00000048520.17	1.93446497	0.0201403	0.0112261	3.14800517	0.07601985	0.16921406	Fbxl13
ENSMUSG00000048521.9	31.5931859	0.04907089	0.00632823	57.7682691	2.95E-14	7.63E-13	Cxcr6
ENSMUSG00000048534.8	21.8889122	0.02467455	0.00640184	14.1808801	0.00016605	0.00090365	Jaml
ENSMUSG00000048537.17	138.289577	0.00698274	0.00539879	1.65667556	0.19805278	0.35552728	Phldb1
ENSMUSG00000048546.9	830.067595	-0.003621	0.00362929	1.01695596	0.31324215	0.4986925	Tob2
ENSMUSG00000048550.18	77.0809824	0.00526814	0.00347591	2.30012433	0.12936364	0.25678934	Thnsl1
ENSMUSG00000048562.8	3.3378889	0.00646073	0.03519378	0.06948468	0.79208857	0.91118387	Sp8
ENSMUSG00000048572.6	13.1699537	0.03654341	0.01051258	12.0823867	0.000509	0.0024327	Tmem252
ENSMUSG00000048578.12	2317.52825	-0.0031524	0.00272243	1.31662323	0.25119898	0.42295681	Mlec
ENSMUSG00000048581.13	118.239771	0.01110596	0.00284599	14.8687817	0.00011525	0.00064987	E130311K13Rik
ENSMUSG00000048582.8	7.21537269	0.14942009	0.0627623	-0.91914	1	1	Gja3
ENSMUSG00000048583.18	23.9692763	0.01438109	0.00911095	2.26754924	0.13210867	0.26118315	Igf2
ENSMUSG00000048602.10	1.32282485	0.01257777	0.02912078	0.35376866	0.5519875	0.73430215	Morc2b
ENSMUSG00000048612.17	112.41428	0.05919338	0.0066385	75.8775896	3.02E-18	1.26E-16	Myof
ENSMUSG00000048616.5	1.63494217	0.00685178	0.03060607	0.1618653	0.68744495	0.84108303	Nog
ENSMUSG00000048621.4	37.0723003	0.05150866	0.00646992	57.218937	3.90E-14	9.92E-13	Gm6377
ENSMUSG00000048644.9	18.3195573	-0.004598	0.00855417	0.28939751	0.59060688	0.76675767	Ctxn1
ENSMUSG00000048647.10	65.6917282	-0.0128735	0.0048574	6.85419113	0.00884336	0.02876958	Exd1

ENSMUSG00000048655.18	11.3285713	-0.0570271	0.01308576	16.7759886	4.21E-05	0.00026338	Ccdc169
ENSMUSG00000048661.15	202.753187	0.00614848	0.00217999	7.75420078	0.00535878	0.01876214	Lemd3
ENSMUSG00000048668.17	85.224388	0.00646214	0.00362584	3.20182447	0.07355617	0.16483571	Rhno1
ENSMUSG00000048677.10	59.4055294	0.02901385	0.00430333	44.4212575	2.65E-11	4.59E-10	Tpcn2
ENSMUSG00000048686.5	2.92867559	-0.176011	0.09709264	-0.0437951	1	1	Hmgb4
ENSMUSG00000048696.12	45.8111072	0.00969052	0.00456146	4.41030741	0.0357224	0.09173855	Mex3d
ENSMUSG00000048699.5	3.42860593	-0.0145214	0.08201977	0.03646442	0.84855961	0.94642866	Krt90
ENSMUSG00000048701.14	454.068877	0.00078723	0.0025923	0.09077283	0.76319695	0.8923196	Ccdc6
ENSMUSG00000048706.4	882.181765	-0.0087334	0.00314168	7.72739415	0.00543892	0.01900761	Lurap1l
ENSMUSG00000048707.10	66.1465532	0.00117631	0.00471892	0.06145962	0.80420365	0.91805563	Tprn
ENSMUSG00000048720.9	183.687121	-0.0052317	0.00319237	2.61544083	0.10582811	0.21924829	Tbc1d12
ENSMUSG00000048728.16	18.9056586	0.00057064	0.00486274	0.01386374	0.9062702	0.97905006	Zfp454
ENSMUSG00000048731.16	200.279659	-0.0212784	0.0059651	12.7264964	0.00036051	0.00179229	Ggnbp1
ENSMUSG00000048732.6	50.4918177	0.00726279	0.00561496	1.63557331	0.20093391	0.35957718	Klhl11
ENSMUSG00000048752.8	1.19246273	0.00071728	0.01360315	0.02027553	0.88677026	0.96789839	Prss50
ENSMUSG00000048755.10	399.595725	-0.0104881	0.00280788	14.0311034	0.00017981	0.00097227	Mcat
ENSMUSG00000048756.12	754.41071	-0.0032768	0.00277199	1.40375035	0.23609665	0.40489897	Foxo3
ENSMUSG00000048758.15	2908.54169	-0.0062596	0.00298518	4.41255873	0.03567529	0.09164987	Rpl29
ENSMUSG00000048763.12	16.6308191	0.03064152	0.00618056	23.5718082	1.20E-06	1.02E-05	Hoxb3
ENSMUSG00000048764.17	1.96303206	-0.0049532	0.02873821	-0.0695307	1	1	Tmprss11f
ENSMUSG00000048772.16	337.126484	0.00813854	0.00311513	6.89846991	0.00862696	0.02818218	Tmem53
ENSMUSG00000048776.9	0.82074784	0.0229508	0.02792819	1.47763079	0.22414558	0.3896544	Pthlh
ENSMUSG00000048779.6	42.5970143	0.04745252	0.00570935	65.2059754	6.75E-16	2.12E-14	P2ry6
ENSMUSG00000048782.17	363.962794	-0.0043445	0.00523153	0.67623644	0.41088571	0.60249614	Insc
ENSMUSG00000048787.14	219.061655	-0.0059764	0.00362227	2.68745815	0.10114104	0.21202672	Dcun1d3
ENSMUSG00000048794.15	7.45574071	-0.0208323	0.01087153	3.34697038	0.06732882	0.15341683	Cfap100
ENSMUSG00000048796.8	114.065066	0.00531188	0.00209717	6.38413393	0.01151449	0.03586207	Cyb561d1
ENSMUSG00000048799.9	312.503946	0.00700846	0.00197084	12.3948807	0.00043051	0.00209319	Cep120
ENSMUSG00000048807.3	29.498989	0.0049815	0.00755149	0.41830199	0.51778554	0.70405221	Slc35e4
ENSMUSG00000048814.12	13.153402	0.02533511	0.01564853	3.00202229	0.08316065	0.18163045	Lonrf2
ENSMUSG00000048826.8	186.049096	0.00842322	0.00410995	4.10063574	0.04286709	0.10647254	Dact2
ENSMUSG00000048827.13	2.09765068	0.03224724	0.01382313	5.11937842	0.02366009	0.06545237	Pkd1l3
ENSMUSG00000048832.10	249.736686	-0.0005735	0.00281559	0.04128624	0.8389864	0.93997403	Vps37c
ENSMUSG00000048833.10	950.004579	-0.0024602	0.00238935	1.03762293	0.30837496	0.49327445	Slc39a9
ENSMUSG00000048834.17	15.585686	0.03716562	0.02069002	3.83898767	0.05007375	0.12070425	Vstm2a
ENSMUSG00000048852.14	13.3826375	0.06583458	0.00969216	37.9591145	7.22E-10	1.02E-08	Gm12185
ENSMUSG00000048856.10	9483.43253	-0.0119273	0.00470492	6.17730978	0.01293989	0.03954907	Slc25a47
ENSMUSG00000048865.17	179.156399	0.05801446	0.00492914	127.165355	1.71E-29	3.15E-27	Arhgap30
ENSMUSG00000048874.16	1448.63031	-0.0033531	0.00390455	0.71828888	0.39670576	0.58848719	Phf3
ENSMUSG00000048878.7	616.828797	-0.004276	0.00236875	3.26627414	0.07071807	0.1595062	Hexim1
ENSMUSG00000048895.14	34.5666595	0.00272803	0.00756116	0.12132244	0.7276046	0.86844199	Cdk5r1
ENSMUSG00000048897.16	246.910088	0.00727485	0.00378448	3.58567468	0.05827975	0.13672032	Zfp710
ENSMUSG00000048904.6	2.01129159	-0.0109054	0.10475481	0.01005432	0.92012898	0.98664131	Neurog1
ENSMUSG00000048905.6	8.97245703	0.0594004	0.0103361	29.3248941	6.12E-08	6.55E-07	Bnip5
ENSMUSG00000048911.16	148.235417	0.01230799	0.00440838	7.54795003	0.00600784	0.02068022	Rnf24
ENSMUSG00000048915.13	117.652384	0.01386806	0.00382377	12.3533529	0.0004402	0.00213323	Efna5
ENSMUSG00000048920.9	243.060793	-0.0025485	0.00259444	0.96657838	0.32553501	0.51228107	Fkrp
ENSMUSG00000048921.12	33.2858872	-0.0034565	0.00370361	0.85745052	0.35445313	0.54304199	Zfp689
ENSMUSG00000048922.19	18.8914861	0.02560922	0.00948021	6.92183078	0.00851498	0.02785882	Cdca2
ENSMUSG00000048924.15	102.68264	-0.0132603	0.00416605	10.0162247	0.00155167	0.00645804	Ccdc125
ENSMUSG00000048930.13	182.175492	0.00396111	0.00269434	2.1419455	0.14332003	0.27784412	Tada3
ENSMUSG00000048938.13	64.619124	0.02582547	0.00448975	32.8143781	1.01E-08	1.22E-07	Nr1h5
ENSMUSG00000048960.14	249.252598	0.00494478	0.00398891	1.51306129	0.21867255	0.38302824	Prex2
ENSMUSG00000048965.10	26.2035769	0.01960027	0.00502156	15.5911132	7.86E-05	0.00046027	Mrgpre
ENSMUSG00000048967.17	3.71854848	-0.0211479	0.00931604	5.1207725	0.02364109	0.06540748	Yjefn3
ENSMUSG00000048970.10	365.039558	-0.004207	0.00199418	4.41025121	0.03572358	0.09173855	C1galt1c1
ENSMUSG00000048978.15	18.2076405	0.00966508	0.01967268	0.25470891	0.61377873	0.78553287	Nrsn1
ENSMUSG00000048988.9	6.74040173	0.04267082	0.01440275	8.35536566	0.0038455	0.01412332	Elfn1
ENSMUSG00000048992.10	1.13305683	-0.0140556	0.02810191	0.37866462	0.53831878	0.72231122	Prss32
ENSMUSG00000048997.18	24.9023901	0.00800495	0.00448436	3.16499651	0.0752326	0.16776443	Atxn7l2
ENSMUSG00000049001.6	6.33029208	0.07333082	0.01361183	25.7885233	3.81E-07	3.56E-06	Ndnf

ENSMUSG00000049011.3	1.35060546	0.02844552	0.02440043	2.62838009	0.10496876	0.21797791	Or4k5
ENSMUSG00000049015.4	0.9373825	-0.0014588	0.0412252	-0.0132782	1	1	Or5b113
ENSMUSG00000049028.9	1.36506566	0.03177111	0.02134006	3.36707504	0.06651174	0.1519661	Or7e177
ENSMUSG00000049037.9	80.7170307	0.06279214	0.00676771	80.6769437	2.66E-19	1.26E-17	Clec4a1
ENSMUSG00000049038.4	55.2032254	0.00258771	0.00638997	0.16027823	0.68890048	0.84206088	Mterf2
ENSMUSG00000049041.7	1.12491591	0.00454057	0.02749285	0.11339068	0.73631643	0.87406308	Or1r1
ENSMUSG00000049044.17	2802.60517	-0.0216125	0.00413458	26.5785793	2.53E-07	2.44E-06	Rapgef4
ENSMUSG00000049047.12	263.586943	-0.0003473	0.00305654	0.01265163	0.91044326	0.98098286	Armcx3
ENSMUSG00000049076.13	417.073299	0.0095548	0.00317086	9.06078863	0.00261149	0.01015241	Acap2
ENSMUSG00000049086.9	65.6282942	-0.0057931	0.00403071	1.95680591	0.16185533	0.30416062	Bmyc
ENSMUSG00000049090.4	1590.80179	-0.0091777	0.00239331	14.7085341	0.00012548	0.00070131	Ptgr3
ENSMUSG00000049091.8	15737.936	-0.0106274	0.00401126	7.00508034	0.00812787	0.02676309	Sephs2
ENSMUSG00000049092.12	20.2482285	0.00564737	0.00757822	0.52916091	0.46695984	0.65800662	Gpr137c
ENSMUSG00000049097.10	3.10869572	0.0101202	0.01540862	0.5233327	0.4694235	0.66049172	Ankrd34a
ENSMUSG00000049098.5	1.03630839	0.00283971	0.03696072	0.07051439	0.7905893	0.91057306	Or8b3
ENSMUSG00000049100.16	11.6162521	0.02681752	0.01551794	3.37376495	0.06624221	0.15152641	Pcdh10
ENSMUSG00000049103.15	85.6630333	0.08289313	0.00652937	137.994749	7.31E-32	2.00E-29	Ccr2
ENSMUSG00000049106.8	647.524102	-0.0076128	0.00254282	8.87248792	0.002895	0.01108467	Dcaf5
ENSMUSG00000049107.14	13.2357658	0.03233243	0.00651312	23.109267	1.53E-06	1.28E-05	Ntf3
ENSMUSG00000049109.16	29.3767285	0.0845305	0.00860588	78.7465054	7.06E-19	3.20E-17	Themis
ENSMUSG00000049112.10	0.9710313	0.02636874	0.02227411	1.97068382	0.1603753	0.30207728	Oxtr
ENSMUSG00000049115.16	1096.5991	0.00858235	0.00288399	8.90363029	0.00284604	0.0109238	Agtr1a
ENSMUSG00000049119.15	11.9599828	0.0095103	0.00792366	1.41113684	0.23486776	0.40328997	Fam110b
ENSMUSG00000049122.18	1.80911762	0.01830914	0.01892051	1.12767197	0.28827246	0.46935092	Frmd3
ENSMUSG00000049130.7	41.8087356	0.06139597	0.00695891	70.0626419	5.75E-17	2.07E-15	C5ar1
ENSMUSG00000049133.9	0.92819952	-0.0021608	0.0289309	-0.0164603	1	1	Flg2
ENSMUSG00000049134.16	90.7591022	0.00861035	0.01255586	-0.8618522	1	1	Nrap
ENSMUSG00000049148.9	2.53935587	0.07210378	0.01415529	26.2625291	2.98E-07	2.83E-06	Plcxd3
ENSMUSG00000049149.10	3.19354597	0.02445117	0.02547714	2.10255433	0.14705329	0.2832965	Or4c10
ENSMUSG00000049152.12	6300.36047	-0.0101301	0.00454842	4.82590516	0.02803514	0.07522603	Ugt3a2
ENSMUSG00000049164.8	201.367423	-0.0067111	0.00360112	3.34219729	0.06752438	0.153803	Zfp518a
ENSMUSG00000049176.17	2.49919489	0.00762278	0.01951026	0.18002986	0.67134758	0.82915662	Frmpd4
ENSMUSG00000049184.7	30.3165452	-0.0039944	0.00402079	0.97733735	0.32285706	0.5093583	Purg
ENSMUSG00000049191.13	28.4641912	0.04271578	0.00738764	32.4425727	1.23E-08	1.47E-07	Rtl5
ENSMUSG00000049225.16	48.456349	0.01345559	0.00535705	6.22378426	0.01260454	0.03865437	Pdp1
ENSMUSG00000049230.9	11.5150332	0.00611209	0.00744534	0.61673121	0.43226459	0.62383101	Myef2l
ENSMUSG00000049232.4	250.409865	0.00258382	0.00274558	0.87612613	0.3492649	0.53738837	Tigd2
ENSMUSG00000049235.9	5.31412371	0.00709847	0.0173417	0.16328191	0.68615274	0.84009544	Gm7324
ENSMUSG00000049241.7	1.37360478	0.02785179	0.01445145	4.22101988	0.03992616	0.10045853	Hcar1
ENSMUSG00000049252.18	4.99881898	0.02577421	0.01642753	2.63632275	0.10444504	0.21706236	Lrp1b
ENSMUSG00000049265.9	56.5954647	-0.0003536	0.0085937	0.00290026	0.95705143	1	Kcnk3
ENSMUSG00000049281.17	7.99111344	0.07134907	0.01121111	35.2309553	2.93E-09	3.83E-08	Scn3b
ENSMUSG00000049285.5	22.2068311	0.01150296	0.00423696	7.27797152	0.00698053	0.02358104	Mblac1
ENSMUSG00000049287.6	206.642334	-0.0132485	0.0025065	27.6204347	1.48E-07	1.49E-06	Iba57
ENSMUSG00000049288.5	94.1640339	0.01147546	0.0040618	7.78002427	0.00528271	0.01854412	Lix1l
ENSMUSG00000049295.18	341.010095	-0.0012276	0.00278894	0.19281554	0.66058352	0.82159984	Zfp219
ENSMUSG00000049299.15	380.600478	-0.0026902	0.00240828	1.27420424	0.25897945	0.43255636	Trappc1
ENSMUSG00000049300.12	114.36755	-0.0024434	0.00216725	1.26381005	0.26093113	0.43508631	Prmt6
ENSMUSG00000049303.11	41.1179844	0.03056877	0.00841645	10.7340927	0.0010518	0.00458976	Syt12
ENSMUSG00000049305.7	284.516742	0.00160531	0.00257104	0.38747696	0.53362861	0.7180605	Ccdc71
ENSMUSG00000049307.7	9.15327237	0.06332267	0.00708388	74.8135338	5.17E-18	2.08E-16	Fut4
ENSMUSG00000049313.9	173.476642	0.07682915	0.00703023	108.341186	2.26E-25	2.21E-23	Sorl1
ENSMUSG00000049321.18	150.448967	-0.0048961	0.00395015	1.51299237	0.21868304	0.38302824	Zfp2
ENSMUSG00000049323.9	554.847319	-0.0048286	0.00239816	3.97872927	0.04607831	0.11293807	Smcr8
ENSMUSG00000049327.18	783.403659	0.0016503	0.00349015	0.21979345	0.63919734	0.80547676	Kmt5a
ENSMUSG00000049336.17	5.43212652	0.04167398	0.01680532	5.96778146	0.0145696	0.04385942	Tenm2
ENSMUSG00000049339.17	2806.06015	-0.0019216	0.00364462	0.27194073	0.60203287	0.77546542	Retreg2
ENSMUSG00000049350.7	4.804813	-0.0548473	0.02252939	6.16892096	0.01300139	0.03970617	Zg16
ENSMUSG00000049353.18	40.9149286	0.00328249	0.00525534	0.36903136	0.54353273	0.72699121	Rd3
ENSMUSG00000049354.9	723.127181	0.00487939	0.00264585	3.4225923	0.06430995	0.14798191	Dcaf7
ENSMUSG00000049357.12	4.08033878	-0.0029605	0.00862711	0.11745191	0.7318147	0.87130397	Brd8dc

ENSMUSG00000049372.12	1.67174305	0.0016834	0.0251412	0.0427638	0.83617066	0.93806703	Or4c31
ENSMUSG00000049382.11	3734.22373	0.02068605	0.00466674	19.3798208	1.07E-05	7.55E-05	Krt8
ENSMUSG00000049387.11	0.92226986	-0.0127256	0.14031348	0.00164334	0.96766413	1	Cox7b2
ENSMUSG00000049396.7	139.45793	-0.0079257	0.00746825	1.02946967	0.31028326	0.49558328	Gemin4
ENSMUSG00000049401.11	618.680811	0.0006245	0.00262887	0.05732231	0.81077932	0.92218936	Ogfr
ENSMUSG00000049404.8	879.375889	-0.0286202	0.00549604	26.4445182	2.71E-07	2.60E-06	Rarres1
ENSMUSG00000049409.10	3.79876224	0.05403862	0.00940421	32.2935456	1.33E-08	1.57E-07	Prokr1
ENSMUSG00000049410.9	3.28043059	0.04253417	0.00831586	26.3673469	2.82E-07	2.69E-06	Zfp683
ENSMUSG00000049411.16	95.045181	0.01349081	0.00363144	13.9515744	0.00018758	0.00100827	Tmem241
ENSMUSG00000049420.10	5.04682619	0.07868867	0.01711926	17.3554736	3.10E-05	0.0001982	Tmem200a
ENSMUSG00000049421.14	518.437361	-0.0061713	0.00256963	5.77504981	0.01625526	0.04793062	Zfp260
ENSMUSG00000049422.8	6370.85021	-0.0167571	0.00338097	24.84762	6.20E-07	5.56E-06	Chchd10
ENSMUSG00000049436.6	8.7995373	-0.0045598	0.00805773	0.30984202	0.57777715	0.7560331	Upk1b
ENSMUSG00000049439.14	232.388134	0.00679299	0.00235742	8.16411586	0.00427273	0.01547315	Cyp20a1
ENSMUSG00000049470.14	1989.97017	0.00117813	0.00401062	0.08352688	0.7725736	0.89838391	Aff4
ENSMUSG00000049482.17	185.403051	-0.0017138	0.00481122	0.124094	0.72463595	0.86682881	Ctu2
ENSMUSG00000049488.15	61.565584	0.01163165	0.00319819	12.9884036	0.00031343	0.00158501	Tmem67
ENSMUSG00000049489.6	125.777149	0.00543798	0.00268428	4.12059917	0.04236407	0.10544523	Ccnq
ENSMUSG00000049493.14	34.6283515	0.01136206	0.00946606	1.50471349	0.21994754	0.38463451	Pls1
ENSMUSG00000049502.18	1159.93592	0.00608559	0.00353822	2.8487335	0.09144595	0.19595204	Dtx3l
ENSMUSG00000049504.13	204.501494	-0.0007281	0.00414609	0.03049223	0.86137803	0.95391409	Proser1
ENSMUSG00000049511.6	4.50342468	0.00925637	0.02412136	0.23047508	0.63117179	0.7993749	Htr1b
ENSMUSG00000049515.13	2.90493917	-0.0092033	0.01001438	0.83124973	0.3619114	0.55153081	Espnl
ENSMUSG00000049516.10	391.677047	-0.0039575	0.00289993	1.83603664	0.17541619	0.32396572	Spty2d1
ENSMUSG00000049517.9	5417.77342	0.002402	0.00409026	0.34620916	0.55626692	0.73845396	Rps23
ENSMUSG00000049521.3	460.635638	-0.0027178	0.0030393	0.80473964	0.36968006	0.56020538	Cdc42ep1
ENSMUSG00000049526.9	1.60631532	0.0454388	0.01318841	11.1987014	0.00081855	0.00368299	Tmem202
ENSMUSG00000049532.12	79.2407927	-0.0025771	0.00408468	0.38453612	0.53518553	0.71953975	Sall2
ENSMUSG00000049536.6	25.8708336	-0.0050627	0.00441318	1.29690096	0.25478008	0.42724798	Tceal1
ENSMUSG00000049537.11	9.41713234	-0.0693798	0.03484893	0.1876045	0.66491789	0.82462685	Tecrl
ENSMUSG00000049538.15	2.59139191	-0.0091969	0.01218745	0.57300832	0.44906596	0.64075614	Adamts16
ENSMUSG00000049539.4	6.88197367	0.01299052	0.01786931	0.54918446	0.45865111	0.65026119	H1f1
ENSMUSG00000049550.18	1094.38992	-0.0070076	0.00318383	4.80075355	0.02844729	0.07611547	Clip1
ENSMUSG00000049551.3	1.57679848	-0.0097659	0.01189878	0.63717038	0.4247373	0.61640746	Fzd9
ENSMUSG00000049553.12	386.524263	0.00272603	0.00314735	0.75288629	0.38556395	0.57717132	Polr1a
ENSMUSG00000049555.11	475.788586	6.40E-05	0.0057605	0.00010542	0.99180799	1	Tmie
ENSMUSG00000049556.6	8.42271653	0.04109243	0.00990734	16.5222648	4.81E-05	0.00029641	Lingo1
ENSMUSG00000049562.7	45.2882195	0.00667557	0.0037445	3.11157428	0.07773779	0.17224269	Ap5b1
ENSMUSG00000049571.19	1.72122326	-0.0013544	0.01285792	-0.0044863	1	1	Cfap46
ENSMUSG00000049573.4	1.05516863	-0.0004165	0.04185979	-0.0030485	1	1	Or6c68
ENSMUSG00000049577.16	705.847384	-0.0085725	0.0055755	2.25318901	0.13333936	0.26289398	Zfpm1
ENSMUSG00000049580.13	1282.10265	-0.0209836	0.00659378	9.56722541	0.00198082	0.00801916	Tsku
ENSMUSG00000049583.16	17.2693731	0.03244305	0.01662719	4.18971396	0.04066996	0.10205784	Grm5
ENSMUSG00000049588.14	17.7120524	0.04971396	0.00732913	40.3054777	2.17E-10	3.32E-09	Ccdc69
ENSMUSG00000049598.10	12.4041546	0.04427915	0.01793585	8.53049053	0.00349246	0.01301518	Vsig8
ENSMUSG00000049600.10	60.6380509	0.01602394	0.004567	12.2953828	0.00045408	0.00219331	Zbtb45
ENSMUSG00000049606.17	410.659235	0.00223924	0.00314474	0.48607572	0.48568308	0.67447722	Zfp644
ENSMUSG00000049608.10	9.09500772	0.06047821	0.00900616	37.939296	7.30E-10	1.03E-08	Gpr55
ENSMUSG00000049612.11	3.17870285	0.03943201	0.01730401	5.92285346	0.01494573	0.04472911	Omg
ENSMUSG00000049624.15	555.252248	-0.0045041	0.00287426	2.39942405	0.12137993	0.24441792	Slc17a5
ENSMUSG00000049625.7	65.6964718	0.05575619	0.00504552	115.837502	5.16E-27	6.34E-25	Tifab
ENSMUSG00000049630.7	13.9957883	0.01074032	0.01060842	1.00294333	0.31659936	0.50203795	C1ql3
ENSMUSG00000049643.16	101.23536	0.00148352	0.00433369	0.11596342	0.7334544	0.87229001	2310022A10Rik
ENSMUSG00000049649.9	1.23320919	0.00270463	0.02973466	0.07809875	0.77989065	0.90341834	Gpr3
ENSMUSG00000049657.10	208.312059	-0.0122644	0.00242847	25.4440935	4.55E-07	4.18E-06	Zbtb5
ENSMUSG00000049658.14	374.236559	0.00257326	0.00358038	0.50719757	0.47635453	0.66658546	Bdp1
ENSMUSG00000049659.16	756.615832	-0.0028452	0.00189588	2.20463278	0.13759666	0.26923391	Aftph
ENSMUSG00000049670.10	7.49601185	-0.0035217	0.00836854	0.17262628	0.67778822	0.8335756	Morn4
ENSMUSG00000049672.16	264.196122	0.01131039	0.00171009	42.9072105	5.74E-11	9.54E-10	Zbtb14
ENSMUSG00000049676.15	6.39503761	0.01061196	0.00869412	1.40250178	0.23630514	0.40522706	Catsperg1
ENSMUSG00000049680.19	511.124117	-0.0054971	0.00241304	5.16118286	0.02309716	0.06410563	Urgcp

ENSMUSG00000049685.9	48.4698645	0.0514347	0.00886653	32.1918935	1.40E-08	1.65E-07	Cyp2g1
ENSMUSG00000049686.16	191.319275	-0.0062762	0.00377089	2.75660151	0.09685379	0.20504384	Orai1
ENSMUSG00000049687.6	6.41945424	0.03406187	0.00836505	15.6610137	7.58E-05	0.00044611	Pheta2
ENSMUSG00000049690.16	18.12645	0.0035329	0.00799615	0.19294615	0.66047579	0.82153687	Nckap5
ENSMUSG00000049694.14	2.41294486	-0.0018341	0.01138792	0.02442643	0.87580478	0.96175849	BC048671
ENSMUSG00000049709.5	14.0618401	0.0255725	0.00949817	6.77187115	0.00926057	0.02994924	Nlrp10
ENSMUSG00000049717.10	104.908626	-0.0093931	0.00288203	10.5236152	0.00117859	0.0050708	Lig4
ENSMUSG00000049721.15	38.2322227	0.03836148	0.00643958	33.8113124	6.07E-09	7.58E-08	Gal3st1
ENSMUSG00000049723.15	63.8200508	0.14743013	0.01108908	142.158527	8.98E-33	2.72E-30	Mmp12
ENSMUSG00000049728.15	61.3030678	0.00014782	0.00430556	0.00113799	0.97308912	1	Zfp668
ENSMUSG00000049734.10	122.830841	0.02437181	0.00363974	44.5761206	2.45E-11	4.27E-10	Trex1
ENSMUSG00000049739.11	308.448697	0.0006768	0.00284467	0.05564361	0.81351882	0.92363796	Zfp646
ENSMUSG00000049744.16	49.2129139	0.06294937	0.00629956	88.0796045	6.29E-21	3.59E-19	Arhgap15
ENSMUSG00000049751.7	3871.46425	-0.0067542	0.00406524	2.79372942	0.09463375	0.20142872	Rpl36al
ENSMUSG00000049755.15	346.485876	-0.0091136	0.00320474	8.11885659	0.00438072	0.01582049	Zfp672
ENSMUSG00000049760.7	2148.48792	-0.0174257	0.0039047	20.1953994	6.99E-06	5.14E-05	Micos13
ENSMUSG00000049761.7	1.00248567	-0.0104026	0.05391425	-0.0860618	1	1	Pmis2
ENSMUSG00000049764.9	174.057363	-0.0052047	0.00289913	3.19562645	0.07383548	0.16528887	Zfp280b
ENSMUSG00000049775.17	6102.01106	0.05446441	0.00577462	79.9359165	3.87E-19	1.79E-17	Tmsb4x
ENSMUSG00000049791.5	550.379019	-0.0030887	0.00343806	0.80444968	0.36976631	0.56027934	Fzd4
ENSMUSG00000049792.7	294.156066	-0.0055919	0.00175106	10.1859965	0.00141511	0.00595271	Bag5
ENSMUSG00000049799.14	11.6808703	0.02139756	0.01321593	2.36659752	0.12395696	0.24837727	Lrrc19
ENSMUSG00000049800.14	350.576893	0.00559621	0.00276224	4.12148598	0.04234187	0.10540109	Sertad2
ENSMUSG00000049804.10	49.3891302	0.01453827	0.00580756	6.49459351	0.0108203	0.03410955	Armcx4
ENSMUSG00000049806.5	1.72852116	-0.0040223	0.02900763	-0.0111077	1	1	Or9s18
ENSMUSG00000049807.17	395.701183	0.00056902	0.00362558	0.02456825	0.8754477	0.96166318	Arhgap23
ENSMUSG00000049811.18	34.8731631	-0.002299	0.00433862	0.28684652	0.59224848	0.7678525	Fam161a
ENSMUSG00000049823.10	50.8454428	0.00161691	0.0042817	0.14202755	0.70627361	0.85422129	Zbtb12
ENSMUSG00000049858.9	1786.4903	-0.0032817	0.00314329	1.07448288	0.29993506	0.48400851	Suox
ENSMUSG00000049866.13	85.9436571	0.04258456	0.00477788	75.9444789	2.92E-18	1.22E-16	Arl4c
ENSMUSG00000049871.15	13.2362385	0.04128724	0.00851842	21.9698371	2.77E-06	2.21E-05	Nlrc3
ENSMUSG00000049872.9	13.2908611	0.03040378	0.00549866	29.5337523	5.50E-08	5.93E-07	Calhm5
ENSMUSG00000049878.14	372.989192	0.0038824	0.00282457	1.8365705	0.17535344	0.32387519	Rlf
ENSMUSG00000049882.11	47.3479618	0.01804705	0.00436844	16.7040197	4.37E-05	0.0002721	Vcpkmt
ENSMUSG00000049892.8	7.27185695	-0.0063882	0.00897156	0.5309552	0.46620555	0.65733645	Rasd1
ENSMUSG00000049897.15	2.70371952	-0.016721	0.00992184	2.81616074	0.09331937	0.19922436	Stkld1
ENSMUSG00000049904.8	26.7682548	0.00811941	0.00422383	3.62458721	0.05693169	0.13415053	Tmem17
ENSMUSG00000049907.9	79.3868408	0.00555572	0.00449874	1.5203653	0.21756422	0.38169218	Rasl11b
ENSMUSG00000049916.13	3.56826475	0.01043065	0.01992529	0.16865862	0.68130669	0.83646347	2610318N02Rik
ENSMUSG00000049922.9	294.203833	-0.0033956	0.00285408	1.40439778	0.23598862	0.40474314	Slc35c1
ENSMUSG00000049928.16	4.20748676	0.04783535	0.00791635	36.0037218	1.97E-09	2.62E-08	Glp2r
ENSMUSG00000049929.8	6.77859035	0.01856431	0.0096975	3.35540881	0.06698458	0.1527948	Lpar4
ENSMUSG00000049932.4	160.88892	-0.0047178	0.00359049	1.74344369	0.18670308	0.33983476	H2ax
ENSMUSG00000049939.7	8.25669579	0.03082921	0.01302234	6.00262164	0.01428464	0.04311715	Lrrc4
ENSMUSG00000049940.8	1222.83289	0.00512436	0.00285857	3.09125932	0.07871387	0.17395113	Pgrmc2
ENSMUSG00000049950.7	55.8149122	-0.0029515	0.00445677	0.43305709	0.51049268	0.69719052	Rpp38
ENSMUSG00000049957.15	261.179738	-0.0085355	0.00423119	4.01884344	0.04499456	0.11064974	Ccdc137
ENSMUSG00000049960.5	549.205594	-0.0126792	0.00388686	10.7970546	0.00101662	0.00445852	Mrps16
ENSMUSG00000049969.4	348.630084	0.00810109	0.0025491	9.89676894	0.00165569	0.00682832	Plekhf2
ENSMUSG00000049971.18	542.670109	0.01452495	0.00475097	9.25393295	0.0023499	0.00927301	Glt1d1
ENSMUSG00000049985.16	12.6801694	0.04314189	0.00856601	22.410162	2.20E-06	1.79E-05	Ankrd55
ENSMUSG00000049988.6	90.4383427	0.0551481	0.00580075	84.1680936	4.54E-20	2.39E-18	Lrrc25
ENSMUSG00000049999.5	14.1774722	0.02500608	0.00752832	10.3255163	0.00131204	0.00556777	Ppp1r3d
ENSMUSG00000050002.15	554.236411	-0.002055	0.00373131	0.30655661	0.57980092	0.75787987	Idnk
ENSMUSG00000050010.9	14.4728305	0.11465443	0.00713776	226.897071	2.83E-51	5.57E-48	Shisa3
ENSMUSG00000050014.9	22.3805505	0.07438506	0.00945069	51.504242	7.14E-13	1.53E-11	Apol10b
ENSMUSG00000050017.12	1348.56232	-0.0037059	0.00194625	3.6443267	0.05626055	0.13306452	Pitpnb
ENSMUSG00000050022.17	27.0485147	0.05809291	0.00697589	66.5989534	3.33E-16	1.08E-14	Amz1
ENSMUSG00000050029.8	682.819969	0.00578999	0.00213075	7.18964094	0.00733257	0.02456967	Rap2c
ENSMUSG00000050030.6	1.30392337	-0.0049686	0.02760124	0.04641728	0.82941906	0.93373481	Or4k1
ENSMUSG00000050035.8	5.13923264	-0.034609	0.0177692	1.22088525	0.26918699	0.44564079	Fhl4

ENSMUSG00000050043.17	995.15271	-0.0125975	0.00261928	23.1222273	1.52E-06	1.27E-05	Tmx2
ENSMUSG00000050050.18	22.9741905	0.0154913	0.00568305	7.04476707	0.00794972	0.02624895	Ccdc158
ENSMUSG00000050052.13	249.524032	-0.0157735	0.00314893	24.5763116	7.14E-07	6.30E-06	Tdrp
ENSMUSG00000050058.7	1.45075416	-0.0255813	0.20950022	0.04011076	0.84126418	0.94154371	Prm3
ENSMUSG00000050064.16	73.3526705	-0.0110665	0.00462055	5.80569927	0.01597431	0.04725576	Zfp697
ENSMUSG00000050069.4	180.831117	-0.0127929	0.00596131	4.8201402	0.02812906	0.07543513	Grem2
ENSMUSG00000050071.9	6.21814505	-0.0107625	0.01316362	0.65388242	0.41872802	0.61012659	Bex1
ENSMUSG00000050075.9	7.67074177	0.06049219	0.00787254	55.7998375	8.02E-14	1.93E-12	Gpr171
ENSMUSG00000050079.11	415.639082	-0.0025107	0.00191571	1.69762821	0.19259845	0.34779924	Rspry1
ENSMUSG00000050088.10	564.915192	-0.0043897	0.00177534	6.01416447	0.0141915	0.04287992	1600012H06Rik
ENSMUSG00000050089.14	5.72851526	-0.0294732	0.06249951	0.11309154	0.73665154	0.87437287	Akap4
ENSMUSG00000050097.7	14.1044062	-0.1417626	0.01148462	137.893025	7.69E-32	2.09E-29	Ces2b
ENSMUSG00000050100.15	0.71083979	0.02047342	0.03992147	0.61472944	0.43301263	0.62461231	Hmx2
ENSMUSG00000050103.19	1236.3163	0.00473197	0.00307084	2.34137482	0.12597833	0.25159397	Agmo
ENSMUSG00000050105.6	3.25466496	0.00346661	0.01165827	0.08093925	0.77602857	0.90084703	Fam110d
ENSMUSG00000050106.19	35.5542038	0.0307521	0.00472897	41.1515876	1.41E-10	2.21E-09	Tmc8
ENSMUSG00000050107.3	13.0891786	0.01515514	0.00748138	3.84843346	0.04979248	0.12015606	Haspin
ENSMUSG00000050114.8	2.98343596	-0.0272565	0.01665141	2.81510664	0.09338069	0.19928311	Prdx6b
ENSMUSG00000050122.21	15.9000305	-0.0017138	0.00840134	0.04018384	0.84112156	0.94147177	Vwa3b
ENSMUSG00000050132.14	10.6273878	0.01478723	0.00701244	4.23308287	0.03964337	0.09984806	Sarm1
ENSMUSG00000050141.14	1.23042476	-0.0220643	0.02750758	0.64753074	0.42099689	0.6125995	Spata31f3
ENSMUSG00000050144.14	1394.56167	-0.008316	0.00313064	7.0219841	0.0080515	0.0265444	Slc25a44
ENSMUSG00000050147.10	1.18171336	0.04775126	0.0139284	11.7759434	0.00060001	0.00280401	F2rl3
ENSMUSG00000050148.10	312.2892	0.00120816	0.00313388	0.14869473	0.69978586	0.85006478	Ubqln2
ENSMUSG00000050150.17	4.56768404	-0.0037574	0.01877493	-0.0044675	1	1	Slc9b1
ENSMUSG00000050164.12	2.1558181	-0.0103627	0.02442982	0.29862417	0.58474623	0.7618109	Mchr1
ENSMUSG00000050174.16	137.423877	-0.0064911	0.00329339	3.92996376	0.04743297	0.11559764	Nudt6
ENSMUSG00000050188.9	229.447265	0.00609084	0.00359352	2.89911556	0.08862817	0.1911764	Lsm10
ENSMUSG00000050192.9	22.6522549	0.05172019	0.00671796	53.6660635	2.38E-13	5.43E-12	Eif5a2
ENSMUSG00000050195.9	3.73836002	0.01199028	0.01316257	0.88307028	0.34736213	0.53552527	Scd4
ENSMUSG00000050198.5	1.12038639	0.00489959	0.03746352	0.13707438	0.71120697	0.85737427	Or6c38
ENSMUSG00000050199.14	1019.45734	-0.0058787	0.00314458	3.45278577	0.06314526	0.14579971	Lgr4
ENSMUSG00000050211.15	3.40206544	-0.007379	0.02859132	0.07703572	0.78135544	0.90431569	Pla2g4e
ENSMUSG00000050212.5	80.8543918	0.01032759	0.00357605	8.37678282	0.00380045	0.0139818	Eva1b
ENSMUSG00000050213.7	240.080716	-0.0068419	0.0024482	7.84056677	0.00510866	0.01801897	Snip1
ENSMUSG00000050222.12	2.26223641	0.00561197	0.01312463	0.22055846	0.63861473	0.80521275	Il17d
ENSMUSG00000050229.4	253.354951	0.00140427	0.00213909	0.42746479	0.51323557	0.69992334	Pigm
ENSMUSG00000050232.7	14.284005	0.06777716	0.00638119	106.455788	5.86E-25	5.46E-23	Cxcr3
ENSMUSG00000050234.8	69.2463348	-0.0036532	0.00390682	0.90738774	0.34080848	0.52849811	Gja4
ENSMUSG00000050240.17	56.9171094	0.00766037	0.00461475	2.73134349	0.09839648	0.20758313	Hic2
ENSMUSG00000050241.9	5.88573589	0.03675972	0.00891858	15.9804711	6.40E-05	0.00038361	Klre1
ENSMUSG00000050244.10	419.538415	0.00745856	0.00351563	4.39089935	0.03613125	0.09262953	Heatr1
ENSMUSG00000050248.12	34.0031304	0.02565634	0.0063278	15.6396433	7.66E-05	0.00044994	Evc2
ENSMUSG00000050270.13	304.472902	0.00173977	0.00374685	0.21352182	0.64402093	0.8095203	Tmem220
ENSMUSG00000050271.13	24.717873	0.0191116	0.00617282	9.43733898	0.00212612	0.00851964	Prag1
ENSMUSG00000050272.11	16.2096122	0.0552464	0.01514183	13.6850273	0.00021617	0.00114344	Dscam
ENSMUSG00000050288.7	7.09653148	0.00115286	0.01015213	0.0130233	0.90914293	0.98049924	Fzd2
ENSMUSG00000050295.5	15.8172534	0.01385877	0.00909422	2.25506409	0.13317794	0.26265866	Foxc1
ENSMUSG00000050296.15	2.8879981	0.00349095	0.02142566	0.10345013	0.74772798	0.88175152	Abca12
ENSMUSG00000050304.5	3.07551991	-0.025697	0.00856498	8.78041064	0.00304483	0.01156818	Slc25a2
ENSMUSG00000050310.10	463.143624	0.00352344	0.00322016	1.16863485	0.27968192	0.45905204	Rictor
ENSMUSG00000050312.13	276.749347	0.00225905	0.00340202	0.42631642	0.51380198	0.70045278	Nsun3
ENSMUSG00000050315.15	59.043436	0.04409863	0.00806424	25.4285944	4.59E-07	4.21E-06	Synpo2
ENSMUSG00000050321.4	9.30037194	0.04919628	0.01152554	15.4163379	8.62E-05	0.00050001	Neto1
ENSMUSG00000050323.15	344.904717	-0.0156414	0.00403657	15.0684126	0.00010368	0.00059084	Ndufaf6
ENSMUSG00000050328.3	6.03944847	0.0110204	0.04691756	0.18297267	0.66883092	0.82734809	Hoxc12
ENSMUSG00000050332.8	154.973125	0.00281718	0.00436608	0.41512575	0.51937927	0.70540594	Amer1
ENSMUSG00000050335.18	192.727032	0.07567724	0.0066117	117.484105	2.25E-27	2.96E-25	Lgals3
ENSMUSG00000050343.3	0.76483656	-0.0049531	0.04535147	-0.0065224	1	1	Or1ad6
ENSMUSG00000050345.10	1.2427667	0.00881576	0.02961297	0.15995576	0.68919725	0.84224904	4930486L24Rik
ENSMUSG00000050350.9	7.6549383	0.09637677	0.00829721	118.092971	1.65E-27	2.23E-25	Gpr18

ENSMUSG00000050357.10	12.3994851	0.04602061	0.00756142	34.2113158	4.94E-09	6.26E-08	Carmil2
ENSMUSG00000050359.8	6.08124773	0.07432085	0.02032946	12.5393279	0.00039848	0.00195923	Sprr1a
ENSMUSG00000050370.6	3.31500429	0.05868002	0.0118554	22.0714083	2.63E-06	2.10E-05	Ch25h
ENSMUSG00000050373.14	164.378882	-0.0026001	0.00325789	0.63799897	0.42443633	0.61606209	Snx21
ENSMUSG00000050377.9	2.45655594	0.04989539	0.02334143	7.48499753	0.00622152	0.02131619	Il31ra
ENSMUSG00000050379.16	58.8380341	0.06447349	0.00580096	111.937061	3.69E-26	4.03E-24	Septin6
ENSMUSG00000050382.15	12.1325982	0.01890141	0.00591259	9.96228113	0.0015978	0.00662431	Kif7
ENSMUSG00000050390.13	1252.84124	-0.0047454	0.0040822	1.31539706	0.25141981	0.423238	C77080
ENSMUSG00000050394.15	14.8978616	0.01533014	0.00511533	8.96168663	0.002757	0.01063048	Armcx6
ENSMUSG00000050395.10	5.382035	0.07437025	0.01175804	35.5169135	2.53E-09	3.32E-08	Tnfsf15
ENSMUSG00000050410.16	36.7234827	0.01422607	0.00512905	7.70210467	0.00551565	0.01924154	Tcf19
ENSMUSG00000050423.12	75.1064647	0.01920173	0.01081315	3.19071473	0.07405763	0.16566039	Ppp1r3g
ENSMUSG00000050428.8	103.069941	0.00585296	0.00236333	6.10810734	0.01345632	0.04089957	Fbxo46
ENSMUSG00000050440.9	15261.9069	0.01370464	0.00682348	3.95623935	0.04669792	0.11413649	Hamp
ENSMUSG00000050445.5	5390.50957	-0.0304412	0.00669536	19.3341098	1.10E-05	7.71E-05	Cyp8b1
ENSMUSG00000050447.16	7.19899242	0.02220917	0.00968637	4.49389093	0.03401617	0.08825272	Lypd6
ENSMUSG00000050471.18	97.7967297	-0.0083419	0.00331183	6.35318144	0.0117171	0.03637745	Fam118b
ENSMUSG00000050473.6	1.19856296	0.05074516	0.01895507	7.65958762	0.00564714	0.01963058	Slc35d3
ENSMUSG00000050493.9	89.6163414	0.00155014	0.00449577	0.11973013	0.72932737	0.86957413	Fam167b
ENSMUSG00000050503.10	22.6027311	-0.0344245	0.00762341	19.6394021	9.35E-06	6.67E-05	Fbxl22
ENSMUSG00000050505.8	8.19545349	0.01436415	0.01473452	0.72821367	0.39346282	0.58494889	Pcdh20
ENSMUSG00000050520.5	10.1342742	0.02487672	0.00809554	9.2995735	0.00229207	0.00907857	Cldn8
ENSMUSG00000050530.15	108.88116	-0.0018273	0.00287747	0.40106533	0.52653959	0.71148455	Fam171a1
ENSMUSG00000050538.9	7.15149263	0.02016303	0.00744055	6.87701211	0.00873114	0.02845553	B230217C12Rik
ENSMUSG00000050541.15	984.487104	-0.0055155	0.00347732	2.48895388	0.11464791	0.23355377	Adra1b
ENSMUSG00000050545.11	45.3372318	-0.0252922	0.00854114	8.39233227	0.00376807	0.01387568	Fam228b
ENSMUSG00000050549.9	79.0106996	0.01582264	0.00328626	22.2188641	2.43E-06	1.95E-05	Fam241a
ENSMUSG00000050552.9	703.824821	-0.0093096	0.00436289	4.63769396	0.031277	0.08232333	Lamtor4
ENSMUSG00000050553.4	1.48570347	-0.0137304	0.03136332	0.10587426	0.74489103	0.87971637	Gk2
ENSMUSG00000050555.10	35.1069823	0.00128177	0.00390443	0.108557	0.74179299	0.87777279	Hyls1
ENSMUSG00000050556.10	72.8443696	0.0078296	0.00534705	2.06547952	0.15066753	0.28844516	Kcnb1
ENSMUSG00000050558.14	1.48322344	0.01465604	0.02125294	0.95384865	0.32874159	0.51580172	Prokr2
ENSMUSG00000050565.17	2925.16035	0.00060734	0.00202277	0.0894999	0.76481389	0.89332444	Tor1aip2
ENSMUSG00000050567.17	279.085459	-0.0018828	0.00239572	0.60964881	0.43492007	0.62653912	Maml1
ENSMUSG00000050577.10	0.91971573	0.00904795	0.03613499	0.19884526	0.65565454	0.81735031	Or8u3-ps
ENSMUSG00000050578.11	12.7511761	0.08624363	0.010105	65.8400265	4.89E-16	1.56E-14	Mmp13
ENSMUSG00000050587.15	71.6878241	0.03811098	0.00635232	34.5246507	4.21E-09	5.38E-08	Lrrc4c
ENSMUSG00000050592.9	33.476435	0.04028534	0.00610916	40.5438397	1.92E-10	2.96E-09	Fam78a
ENSMUSG00000050600.6	9.34796046	0.07639355	0.00810653	81.9306252	1.41E-19	7.00E-18	Zfp831
ENSMUSG00000050605.18	16.5016858	0.03290272	0.00672521	23.1066528	1.53E-06	1.28E-05	Zfp61
ENSMUSG00000050608.12	1257.78272	-0.0150063	0.00461598	10.7197423	0.00105998	0.00462122	Micos10
ENSMUSG00000050612.7	3.44590222	0.02642305	0.04955202	-0.3485988	1	1	Txndc2
ENSMUSG00000050619.15	279.900891	-0.0028697	0.00245326	1.37570468	0.24083415	0.41066472	Zscan29
ENSMUSG00000050621.8	125.953886	0.01570084	0.00689187	4.96479404	0.0258684	0.07045404	Rps27rt
ENSMUSG00000050623.6	1.9706745	-0.0146717	0.02194239	0.66277302	0.41558267	0.60689324	Catsperz
ENSMUSG00000050627.14	606.557546	0.00255499	0.00239402	1.14996701	0.28355606	0.46361609	Gpd1l
ENSMUSG00000050628.7	417.329428	0.00612113	0.00382191	2.54443686	0.11068337	0.22730326	Ubald2
ENSMUSG00000050640.17	16.4239402	0.05704989	0.0089235	38.4884394	5.51E-10	7.92E-09	Tmem150c
ENSMUSG00000050663.9	48.2886505	-0.013898	0.0086598	1.94283247	0.16336132	0.30642914	Trhde
ENSMUSG00000050666.15	41.1437808	0.01189412	0.00430873	7.57694984	0.00591194	0.02040652	Vstm4
ENSMUSG00000050668.11	184.732385	0.00627302	0.00423235	2.18960877	0.1389446	0.27124213	Gpatch11
ENSMUSG00000050671.13	3.05711946	0.03695739	0.00910208	18.1303683	2.06E-05	0.00013771	Ism2
ENSMUSG00000050675.8	4.18151477	0.02563362	0.01097277	4.93589312	0.02630447	0.07141081	Gp1ba
ENSMUSG00000050677.3	5.53337103	-0.0403859	0.00870748	21.08044	4.40E-06	3.37E-05	Ccdc96
ENSMUSG00000050685.6	1.19403214	-0.0022716	0.08234894	-0.0041754	1	1	Ccdc54
ENSMUSG00000050697.10	280.396134	0.00399871	0.00234591	2.86373011	0.09059719	0.19439822	Prkaa1
ENSMUSG00000050705.16	353.597644	-0.017725	0.00339099	27.8900905	1.28E-07	1.31E-06	2310061I04Rik
ENSMUSG00000050708.17	58150.0289	-0.0010344	0.00334165	0.09551766	0.75727641	0.88825947	Ftl1
ENSMUSG00000050711.8	26.9782698	0.03670755	0.01500691	6.09400644	0.01356412	0.04117947	Scg2
ENSMUSG00000050714.10	99.2760675	0.00075905	0.00291301	0.06675653	0.79611917	0.91380518	Zbtb26
ENSMUSG00000050721.10	170.100331	0.03812901	0.00465748	63.3568231	1.72E-15	5.17E-14	Plekho2

ENSMUSG00000050730.18	1144.26417	-0.014662	0.00346604	17.5221306	2.84E-05	0.00018366	Arhgap42
ENSMUSG00000050732.10	2192.25882	4.23E-07	0.00404016	1.26E-08	0.99991059	1	Vamp8
ENSMUSG00000050737.14	20.8292905	0.05758228	0.00856214	40.4829806	1.98E-10	3.04E-09	Ptges
ENSMUSG00000050751.16	4.05229672	0.05171015	0.01802707	9.37119835	0.00220421	0.00879523	Pgbd5
ENSMUSG00000050761.5	6.99483296	0.07077539	0.02699937	7.2715454	0.00700555	0.02364476	Gp1bb
ENSMUSG00000050772.3	1.24675772	0.00167746	0.02736782	0.0419045	0.83780191	0.93909497	Or10ag58
ENSMUSG00000050777.8	1047.58463	-0.00027	0.00405256	0.00438741	0.9471887	1	Tmem37
ENSMUSG00000050786.9	37.5823893	0.00535505	0.00343711	2.36910458	0.12375802	0.2481262	Ccdc126
ENSMUSG00000050796.5	58.8081325	0.0023506	0.0034142	0.46087267	0.49721641	0.68428908	B3galt6
ENSMUSG00000050812.19	1680.71465	-0.0051609	0.00295707	3.06830414	0.07983271	0.17585497	Ecpas
ENSMUSG00000050821.15	16.1020268	0.01352634	0.00672376	3.97178501	0.0462687	0.11327523	Fam131a
ENSMUSG00000050822.12	1.21186446	0.01996263	0.01711147	1.65356786	0.19847402	0.35612083	Slc29a4
ENSMUSG00000050830.18	3.19557383	0.02578071	0.01165435	4.19662111	0.04050461	0.10168619	Vwc2
ENSMUSG00000050840.9	1.61255759	0.01042595	0.03156677	0.30989585	0.5777441	0.7560331	Cdh20
ENSMUSG00000050846.10	221.18799	-0.006869	0.0021391	10.2309907	0.001381	0.00581857	Zfp623
ENSMUSG00000050854.10	114.684802	-0.0068754	0.00358586	3.50989119	0.06100346	0.14188223	Tmem125
ENSMUSG00000050855.17	22.2378082	0.0069142	0.00551796	1.48387513	0.2231692	0.38869213	Zfp940
ENSMUSG00000050856.17	2435.46379	-0.023762	0.00484941	24.4096296	7.79E-07	6.80E-06	Atp5me
ENSMUSG00000050860.7	142.263864	-0.0222134	0.00763112	7.20026116	0.0072893	0.02444552	Phospho1
ENSMUSG00000050866.8	3.68964356	-0.0041242	0.02581455	0.02428847	0.87615317	0.96183824	Clrn3
ENSMUSG00000050875.12	4.88261467	0.03649692	0.01786223	4.81756428	0.02817113	0.07553077	Minar2
ENSMUSG00000050890.16	252.195119	-0.0010536	0.00298684	0.12248584	0.72635388	0.8678275	Pdik1l
ENSMUSG00000050891.11	191.912193	-0.0048061	0.00272745	3.09092363	0.07873011	0.17395113	Tatdn1
ENSMUSG00000050896.13	3.69867172	0.03749258	0.01008633	13.312911	0.00026358	0.00136157	Rtn4rl2
ENSMUSG00000050908.13	8.44182239	0.04047599	0.00844189	21.5190633	3.50E-06	2.74E-05	Tvp23a
ENSMUSG00000050910.6	49.9379964	0.01357222	0.00430698	9.99696846	0.00156798	0.00651787	Cdr2l
ENSMUSG00000050912.16	851.097403	0.01208954	0.00279062	18.2769435	1.91E-05	0.00012831	Tmem123
ENSMUSG00000050914.17	20.0828374	0.03681478	0.00845765	17.3905818	3.04E-05	0.00019511	Ankrd37
ENSMUSG00000050919.11	53.9800128	0.0163935	0.00501983	10.5888696	0.00113771	0.00491734	Zfp366
ENSMUSG00000050921.13	19.862773	0.07585278	0.00684386	110.325781	8.31E-26	8.68E-24	P2ry10
ENSMUSG00000050930.6	9.51004671	-0.0583115	0.0062229	86.0671843	1.74E-20	9.54E-19	Map10
ENSMUSG00000050931.8	999.065247	0.00736127	0.00404731	3.23158603	0.0722307	0.16238999	Sgms2
ENSMUSG00000050944.15	5.46149717	0.03063392	0.01635789	3.31010395	0.06885514	0.15614049	Efcab5
ENSMUSG00000050945.9	38.2101774	0.00891827	0.0033502	6.97407949	0.00826988	0.02714698	Zfp438
ENSMUSG00000050947.10	176.574891	-0.0058219	0.00371325	2.46177054	0.11664723	0.23691049	Amigo1
ENSMUSG00000050953.11	280.850737	0.00868211	0.00527555	2.5455767	0.11060352	0.22715906	Gja1
ENSMUSG00000050954.16	67.4123885	0.00438056	0.00545144	0.63055003	0.42715363	0.61865276	Zfp169
ENSMUSG00000050957.5	6.17412528	0.02865508	0.00812485	12.0054913	0.00053044	0.00252329	Insl6
ENSMUSG00000050963.8	2.08746157	0.01112117	0.03152	0.24816302	0.61837153	0.78926949	Kcns2
ENSMUSG00000050965.15	259.337339	0.024635	0.00434215	31.181825	2.35E-08	2.69E-07	Prkca
ENSMUSG00000050966.10	3.34667595	0.05902998	0.02647728	3.65326887	0.05595928	0.13242255	Lin28a
ENSMUSG00000050967.6	9.66876845	0.05357339	0.0153015	11.7094258	0.00062184	0.00289457	Creg2
ENSMUSG00000050973.12	79.5745346	-0.005785	0.0029187	3.9306428	0.04741382	0.11556291	Gdpgp1
ENSMUSG00000050982.14	0.92758903	0.01790339	0.01846229	0.93360122	0.3339289	0.52086813	Apol10a
ENSMUSG00000050989.11	64.7538473	0.02474901	0.00473307	26.4690466	2.68E-07	2.57E-06	Selenon
ENSMUSG00000050994.22	54.1892536	0.05851043	0.00647553	72.7840107	1.45E-17	5.60E-16	Adgb
ENSMUSG00000050996.8	2.56769291	-0.1533262	0.05872817	-0.2341973	1	1	Cetn1
ENSMUSG00000051000.18	109.815163	-0.0151709	0.00413728	13.192521	0.00028107	0.00144178	Fhip1a
ENSMUSG00000051007.16	323.260063	0.00489413	0.00357489	1.84239322	0.17467071	0.32289233	Gatd1
ENSMUSG00000051022.8	8.00890836	0.0128518	0.00747806	2.94138973	0.08633642	0.18728326	Hs3st1
ENSMUSG00000051029.9	1.9193197	0.05979971	0.01402317	17.848321	2.39E-05	0.00015704	Serpinb1b
ENSMUSG00000051034.8	36.4992749	0.00919894	0.00295801	9.41954209	0.00214685	0.00858959	Zfp11
ENSMUSG00000051037.16	31.0608895	0.01232119	0.00376358	10.3731899	0.00127858	0.00545132	Zfp455
ENSMUSG00000051041.9	315.821364	0.01105913	0.00298842	13.6599379	0.00021908	0.00115711	Olfml1
ENSMUSG00000051043.17	1212.15751	-0.0093257	0.00354587	6.7387886	0.00943387	0.03041012	Gprc5c
ENSMUSG00000051048.18	1.89682192	0.00936785	0.01939351	0.36258136	0.5470761	0.72978546	P4ha3
ENSMUSG00000051056.6	1.74276028	-0.0278149	0.0138289	4.06697321	0.04372957	0.10819318	Gja10
ENSMUSG00000051065.10	333.323457	-0.0002383	0.0025184	0.00875521	0.9254513	0.98916486	Mb21d2
ENSMUSG00000051067.9	1.8725202	-0.0043145	0.0304183	-0.0457773	1	1	Lingo3
ENSMUSG00000051076.9	11.2789705	0.01639007	0.00720295	4.94129534	0.02622238	0.07123716	Vtcn1
ENSMUSG00000051098.7	255.321817	0.01621309	0.00360045	19.7220033	8.96E-06	6.42E-05	Mblac2

ENSMUSG00000051111.17	8.00460378	0.0121604	0.01183758	0.90779487	0.34070018	0.52842471	Sv2c
ENSMUSG00000051113.10	11.5566815	0.01112377	0.00768376	2.04073396	0.15313574	0.29193559	Garin5a
ENSMUSG00000051118.10	18.6304187	0.02309432	0.00852983	6.74641182	0.00939364	0.0303131	Or7d10
ENSMUSG00000051124.7	260.516205	0.0026222	0.00345234	0.57343364	0.4488977	0.64073467	Gimap9
ENSMUSG00000051146.4	23.3429306	-0.0055934	0.0069726	0.62498125	0.42920222	0.62063415	Camk2n2
ENSMUSG00000051147.11	392.357251	0.00395416	0.00311896	1.61524472	0.20375612	0.36352435	Nat2
ENSMUSG00000051149.16	130.256198	0.0099621	0.00795876	1.43218968	0.23140741	0.39912068	Adnp
ENSMUSG00000051154.13	1454.27423	0.00107364	0.00236748	0.20384408	0.65163575	0.81397525	Commd3
ENSMUSG00000051166.11	146.376681	0.00507142	0.00514004	0.9181553	0.3379598	0.52548749	Eml5
ENSMUSG00000051169.15	218.8757	-0.0230619	0.00466468	24.9657014	5.84E-07	5.26E-06	Rpusd3
ENSMUSG00000051176.7	3.88056772	-0.0124137	0.05010712	0.03431175	0.85304527	0.94936657	Zfp42
ENSMUSG00000051177.17	480.809997	0.00146403	0.00454425	0.10238529	0.74898575	0.88261545	Plcb1
ENSMUSG00000051184.8	189.238775	-0.006228	0.00351768	3.13812504	0.07648168	0.1701002	Zfp524
ENSMUSG00000051185.10	374.310059	0.00023024	0.00374244	0.00363857	0.95190035	1	Fam174a
ENSMUSG00000051212.9	13.9904938	0.06936608	0.00779455	69.1144201	9.29E-17	3.24E-15	Gpr183
ENSMUSG00000051220.3	11.3501983	0.02435484	0.00866982	7.67631837	0.00559502	0.01947235	Ercc6l
ENSMUSG00000051223.15	3264.75838	-0.0017421	0.00197146	0.77332477	0.37919047	0.57060013	Bzw1
ENSMUSG00000051224.14	50.6007306	0.00911581	0.00350084	6.63748366	0.00998549	0.03186116	Tceanc
ENSMUSG00000051225.10	23.0581961	0.06674194	0.01071402	32.2849914	1.33E-08	1.58E-07	Fam83a
ENSMUSG00000051232.14	211.978921	-0.0066248	0.00283973	5.5009262	0.0190064	0.05454917	Tmem199
ENSMUSG00000051234.7	690.961625	-0.0001215	0.00359755	0.00111708	0.97333743	1	Rnf7
ENSMUSG00000051235.11	36.2806626	0.00928373	0.00628844	2.02235613	0.15499848	0.29452012	Gen1
ENSMUSG00000051236.14	304.388603	0.0074789	0.00372281	3.86753568	0.04922875	0.1190393	Msrb3
ENSMUSG00000051238.7	85.3283478	0.00147113	0.0026235	0.31331582	0.57565256	0.75421293	Swsap1
ENSMUSG00000051242.2	3.98556006	0.06962266	0.00848229	63.6194809	1.51E-15	4.56E-14	Pcdhb9
ENSMUSG00000051243.15	2.97978254	0.03315516	0.01733148	4.36667494	0.03664844	0.09367036	Islr2
ENSMUSG00000051246.4	6.23945977	0.00306091	0.01021799	0.061207	0.80459829	0.91828391	Msantd1
ENSMUSG00000051256.11	499.867458	-0.0130251	0.00244159	28.6772855	8.55E-08	8.98E-07	Jagn1
ENSMUSG00000051262.10	10.5519937	-0.0193363	0.01242434	2.35646198	0.12476487	0.24965676	Nat8f3
ENSMUSG00000051278.13	23.8566305	0.0319129	0.00749096	17.5513615	2.80E-05	0.00018125	Zgrf1
ENSMUSG00000051279.3	17.7099622	0.0406814	0.00505694	61.4476367	4.55E-15	1.29E-13	Gdf6
ENSMUSG00000051285.18	3001.75043	-0.0071586	0.00330751	4.59435368	0.03207744	0.08403625	Pcmtd1
ENSMUSG00000051306.8	200.901876	0.00188921	0.00404338	0.21512053	0.64278325	0.80864489	Usp42
ENSMUSG00000051314.12	2.74626864	0.06723912	0.0114673	29.252284	6.35E-08	6.78E-07	Ffar2
ENSMUSG00000051316.9	115.940817	0.00060956	0.00247569	0.05883645	0.80834452	0.92087104	Taf7
ENSMUSG00000051319.7	273.431023	-0.0229184	0.00458103	25.7846245	3.82E-07	3.57E-06	Mtln
ENSMUSG00000051323.17	10.556828	0.03253716	0.01825847	4.02658087	0.04478863	0.11023526	Pcdh19
ENSMUSG00000051329.14	180.877598	-0.0001374	0.00513648	0.00073198	0.97841582	1	Nup160
ENSMUSG00000051331.17	78.3829752	-0.008419	0.01050659	0.6228938	0.42997396	0.62144343	Cacna1c
ENSMUSG00000051335.7	70.1959	-0.0093842	0.00576442	2.55822796	0.10972151	0.22583921	Gfod1
ENSMUSG00000051339.11	1646.50985	0.00076195	0.0036317	0.04290489	0.83590446	0.93799146	2900026A02Rik
ENSMUSG00000051341.7	29.8045241	0.03671633	0.00590985	35.1622211	3.03E-09	3.96E-08	Zfp52
ENSMUSG00000051343.12	42.0065063	0.02918791	0.00598127	22.0029335	2.72E-06	2.17E-05	Rab11fip5
ENSMUSG00000051344.14	70.5322974	0.03519678	0.00535328	41.4609341	1.20E-10	1.91E-09	Plekhm3
ENSMUSG00000051346.10	503.15383	-0.0077408	0.00230719	11.5084546	0.0006928	0.00318341	Spryd4
ENSMUSG00000051351.15	108.316353	0.00316396	0.00359727	0.76343803	0.38225474	0.57392961	Zfp46
ENSMUSG00000051354.15	3.44607513	0.0702732	0.0108008	37.2225463	1.05E-09	1.46E-08	Samd3
ENSMUSG00000051355.19	842.455585	-0.0075565	0.003343	5.19856451	0.02260555	0.06301554	Commd1
ENSMUSG00000051359.16	286.139169	0.0038269	0.00388974	0.97426923	0.32361775	0.51031248	Ncald
ENSMUSG00000051361.8	2.87695634	-0.028021	0.01053395	6.735385	0.00945188	0.03045105	6030498E09Rik
ENSMUSG00000051367.9	2.39517267	0.04557284	0.03062274	4.10084985	0.04286166	0.10647027	Six1
ENSMUSG00000051373.6	12.3959975	0.0095184	0.00639124	2.09465548	0.14781503	0.28427636	Plpp7
ENSMUSG00000051375.16	1594.43464	-0.010756	0.00347443	9.29704256	0.00229524	0.00908772	Pcdh1
ENSMUSG00000051378.11	8.04456218	0.01321945	0.01267012	0.9925912	0.31910989	0.50506816	Kif18b
ENSMUSG00000051379.13	27.4122004	0.04508221	0.00980126	20.7838311	5.14E-06	3.88E-05	Flrt3
ENSMUSG00000051390.11	255.398075	0.01026089	0.00220363	21.5497015	3.45E-06	2.70E-05	Zbtb22
ENSMUSG00000051391.10	2137.5362	0.00086876	0.00270928	0.10177522	0.74970962	0.88320519	Ywhag
ENSMUSG00000051396.16	20.1012193	0.00415101	0.00424338	0.94874661	0.33003854	0.51678212	Msantd7
ENSMUSG00000051397.6	3.57979186	0.05612036	0.01617438	11.8392932	0.00057994	0.0027237	Tacstd2
ENSMUSG00000051401.8	1.54245694	0.01478565	0.03905029	0.48490533	0.48620877	0.67492908	Kctd16
ENSMUSG00000051403.10	1051.23514	-0.0172476	0.00307812	31.040698	2.53E-08	2.87E-07	Ppp1r37

ENSMUSG00000051413.18	109.242142	0.01327705	0.00350137	14.24136	0.0001608	0.00087771	Plagl2
ENSMUSG00000051427.15	258.911264	-0.0128365	0.00314121	16.7149383	4.34E-05	0.00027075	Ccdc157
ENSMUSG00000051435.12	44.7522353	-0.0097589	0.00631399	2.34761586	0.12547478	0.25080069	Fhad1
ENSMUSG00000051439.8	79.1745989	0.04285059	0.0063027	42.6283852	6.62E-11	1.09E-09	Cd14
ENSMUSG00000051444.10	12.3000775	0.03995785	0.00626925	38.4031682	5.75E-10	8.25E-09	Bbs12
ENSMUSG00000051451.7	451.838655	-0.0006364	0.00297419	0.04484442	0.83229037	0.93549374	Crebzf
ENSMUSG00000051452.9	93.4229825	0.00734644	0.0052561	1.9552699	0.1620201	0.30442174	Gm11437
ENSMUSG00000051455.14	18.0281222	-0.0126913	0.01818142	0.44055125	0.50685651	0.69371077	Meioc
ENSMUSG00000051456.5	1.17123781	-0.0175042	0.09622816	0.0551418	0.81434619	0.92404355	Hspb3
ENSMUSG00000051457.8	39.8381356	0.09240654	0.00804607	112.016034	3.54E-26	3.91E-24	Spn
ENSMUSG00000051469.15	530.403886	-0.0045009	0.00267556	2.81885915	0.0931626	0.1989617	Zfp24
ENSMUSG00000051483.10	1460.04227	0.00620198	0.00373109	2.88116182	0.08962134	0.19293619	Cbr1
ENSMUSG00000051486.6	4.5490558	0.03544535	0.00818976	18.6876063	1.54E-05	0.00010551	Pcdhb11
ENSMUSG00000051495.9	899.947604	-0.0051933	0.00399914	1.6391241	0.20044569	0.35897585	Irf2bp2
ENSMUSG00000051497.16	61.1440449	0.02216204	0.00721013	8.74978245	0.00309639	0.01173008	Kcnj16
ENSMUSG00000051498.8	20.4281384	0.05847352	0.00736581	55.7360048	8.29E-14	1.98E-12	Tlr6
ENSMUSG00000051499.8	7.26070449	0.01481049	0.00688063	4.42903554	0.03533247	0.09095739	Zfp786
ENSMUSG00000051502.8	35.4703323	-0.0084379	0.00362136	5.47985949	0.01923681	0.05510314	Ufsp1
ENSMUSG00000051503.3	21.228007	0.01023004	0.01405075	0.66078648	0.41628242	0.60750138	Ccdc121rt3
ENSMUSG00000051504.19	13.4324663	-0.0038385	0.00727408	0.2925526	0.58858939	0.76542258	Siglech
ENSMUSG00000051506.18	126.917581	0.06911127	0.00672346	92.6733956	6.17E-22	4.07E-20	Wdfy4
ENSMUSG00000051510.14	1270.64613	-0.0078351	0.00357298	4.78332267	0.02873662	0.07673293	Mafg
ENSMUSG00000051515.12	3.49797929	0.02525785	0.01271866	3.77982806	0.05187399	0.12420058	Fam181b
ENSMUSG00000051517.15	12.2559019	0.00907519	0.00861971	1.04278303	0.3071751	0.49216027	Arhgef39
ENSMUSG00000051518.9	196.609382	-0.0105284	0.00346795	9.23049509	0.00238017	0.00937678	Rps19bp1
ENSMUSG00000051527.12	5.94311303	0.02659404	0.01537767	2.9236983	0.0872876	0.18893633	Usp29
ENSMUSG00000051550.11	102.910499	0.00251096	0.00501929	0.25434756	0.61403033	0.78576959	Zfp579
ENSMUSG00000051557.16	86.1176912	-0.0059848	0.0030002	4.04048901	0.04442095	0.10944453	Pusl1
ENSMUSG00000051579.11	308.77925	-0.0086826	0.0049129	3.31810421	0.0685208	0.15563678	Tceal8
ENSMUSG00000051586.18	550.203917	-0.0146092	0.00392193	13.925354	0.00019022	0.00102079	Mical3
ENSMUSG00000051590.11	1.72432304	0.02579283	0.01212235	4.14710988	0.04170565	0.10409201	Map3k19
ENSMUSG00000051615.15	434.594536	-0.0161546	0.00255518	39.8410079	2.75E-10	4.15E-09	Rap2a
ENSMUSG00000051618.6	1.20164705	-0.0184634	0.04579775	0.13183144	0.71654064	0.86076844	Ubqln3
ENSMUSG00000051627.4	174.468299	0.0039593	0.00932162	0.15295617	0.69572619	0.84675595	H1f4
ENSMUSG00000051648.14	0.9870964	-7.21E-05	0.03358008	-0.0012025	1	1	Kctd19
ENSMUSG00000051650.12	198.105247	-0.0052794	0.00719425	0.50780333	0.47609133	0.66641484	B3gnt2
ENSMUSG00000051652.5	1588.20557	-0.0072512	0.00414968	3.00980128	0.08276244	0.18092825	Lrrc3
ENSMUSG00000051669.7	36.3296199	0.0209857	0.00392987	27.9063819	1.27E-07	1.30E-06	AU021092
ENSMUSG00000051671.8	524.738111	-0.012197	0.00361944	11.6172116	0.00065344	0.00302792	Coa6
ENSMUSG00000051674.16	634.644982	-0.0007638	0.00334355	0.052028	0.81957125	0.92756274	Dcun1d4
ENSMUSG00000051675.14	156.591898	0.00923703	0.0032684	7.80707441	0.00520421	0.01830944	Trim32
ENSMUSG00000051682.16	126.317243	0.05914874	0.00537711	110.221362	8.76E-26	9.11E-24	Treml4
ENSMUSG00000051687.3	2.0872776	-0.012731	0.02603584	0.3035624	0.58165769	0.75926618	Vmn1r73
ENSMUSG00000051695.7	2683.02833	-0.0016311	0.00300829	0.29164916	0.58916565	0.76574976	Pcbp1
ENSMUSG00000051703.15	2.89475243	0.02879137	0.01169594	5.86194259	0.01547171	0.04604615	Tmem198
ENSMUSG00000051705.14	59.1153616	0.00197307	0.00410575	0.22488439	0.63534319	0.802947	Senp8
ENSMUSG00000051716.7	5578.77694	0.00024726	0.00514787	0.00231405	0.9616329	1	Apon
ENSMUSG00000051721.7	189.607362	-0.0031519	0.00347098	0.80258924	0.37032035	0.56090264	Wdcp
ENSMUSG00000051726.7	21.7936305	0.01713912	0.01994004	0.8542514	0.35535241	0.54388888	Kcnf1
ENSMUSG00000051727.15	3.2886398	0.02181089	0.00934939	5.14424986	0.02332349	0.06464088	Kctd14
ENSMUSG00000051728.8	1.6400489	1.66E-06	0.02499875	1.85E-05	0.99656527	1	Fam243
ENSMUSG00000051730.16	269.04981	-0.0091898	0.00295348	9.7549771	0.00178838	0.00731923	Mettl5
ENSMUSG00000051732.3	3.32928566	-0.0494288	0.03488233	0.9620183	0.32667889	0.51353295	Pabpc2
ENSMUSG00000051735.8	47.6576825	0.05017459	0.00587416	69.0641845	9.53E-17	3.32E-15	Rinl
ENSMUSG00000051736.9	12.4886123	0.00101656	0.00886766	0.01366199	0.90695158	0.97941233	Fam229b
ENSMUSG00000051747.17	1150.16264	0.0556083	0.01476073	6.23417222	0.01253082	0.0384786	Ttn
ENSMUSG00000051748.3	10485.1719	0.00083415	0.00601068	0.0194272	0.88914869	0.96954458	Wfdc21
ENSMUSG00000051768.10	191.340775	-0.0005345	0.0034532	0.0243244	0.87606234	0.96183824	Xrcc1
ENSMUSG00000051786.16	207.792777	-0.0069167	0.00357969	3.72677164	0.05354679	0.12758407	Tubgcp6
ENSMUSG00000051790.16	53.9850523	0.01332309	0.00555381	5.71723956	0.01679912	0.04929386	Nlgn2
ENSMUSG00000051804.4	1.6156055	0.0295075	0.02688074	1.91818637	0.16605673	0.31049739	Adam6b

ENSMUSG00000051811.15	3.77936215	-0.0167828	0.00961737	3.02636889	0.08192116	0.17949376	Cox6b2
ENSMUSG00000051817.10	70.3961137	-0.0071688	0.0052887	1.88203835	0.17010266	0.31630726	Sox12
ENSMUSG00000051839.8	6.95109445	0.05997142	0.01584818	7.95082947	0.00480653	0.01708986	Gypa
ENSMUSG00000051851.6	5.83076783	0.01602527	0.00660457	5.78697471	0.01614535	0.0476601	Rtl8c
ENSMUSG00000051853.11	1282.97068	0.0049215	0.00322721	2.31474878	0.128152	0.25522192	Arf3
ENSMUSG00000051855.16	57.4376456	0.0118704	0.00535635	4.83247776	0.02792847	0.07500806	Mest
ENSMUSG00000051864.11	299.745624	0.00991905	0.00206354	22.688526	1.91E-06	1.57E-05	Tbc1d22a
ENSMUSG00000051877.10	0.8737648	0.0054176	0.03901466	0.06861609	0.79336251	0.91207587	Vmn2r72
ENSMUSG00000051879.5	0.34711143	0.00698577	0.07667954	0.04744951	0.82756229	0.93249935	Krt71
ENSMUSG00000051890.14	36.5600779	0.02632553	0.01187365	4.28531711	0.03844291	0.09730784	Klhdc1
ENSMUSG00000051906.16	29.379075	-0.0092855	0.01679367	-0.0219691	1	1	Cd209f
ENSMUSG00000051910.14	323.394332	-0.006145	0.00442228	1.89422653	0.16872598	0.3143421	Sox6
ENSMUSG00000051920.8	2.5225505	0.00750367	0.02313605	0.13166682	0.71671005	0.86088425	Rspo2
ENSMUSG00000051934.6	178.057394	0.00799489	0.00426675	3.45946391	0.0628907	0.14534001	Spats2
ENSMUSG00000051940.3	0.69602067	-0.0260186	0.08126437	0.12873104	0.71975138	0.86299914	5031410I06Rik
ENSMUSG00000051950.11	49.4574743	0.01467111	0.00410204	12.2359219	0.00046878	0.00225831	B3glct
ENSMUSG00000051951.6	4.22586025	0.03010392	0.01608337	4.05100987	0.04414493	0.10894656	Xkr4
ENSMUSG00000051956.5	1.51457925	-0.0197007	0.02584841	0.6274887	0.42827798	0.61960157	Rnf133
ENSMUSG00000051969.8	8.59414313	0.05169937	0.00695549	53.2407789	2.95E-13	6.67E-12	Tlr11
ENSMUSG00000051977.17	98.2042092	0.00163418	0.00389451	0.17383727	0.67672378	0.8328736	Prdm9
ENSMUSG00000051978.10	57.4034407	0.00594622	0.0034138	2.97094282	0.08477244	0.18462799	Erich1
ENSMUSG00000051980.14	1.49668614	0.03519478	0.03568498	1.70929222	0.19107723	0.34582268	Casr
ENSMUSG00000051984.14	111.506795	-0.0011779	0.00448708	0.06964772	0.79185039	0.91118387	Sec31b
ENSMUSG00000051989.12	315.063028	-0.00895	0.00361611	6.20128415	0.01276577	0.03911319	Smim11
ENSMUSG00000051998.15	15.1192936	0.09474555	0.00767005	131.08164	2.38E-30	5.32E-28	Lax1
ENSMUSG00000052013.16	27.1259025	0.06867419	0.00671488	92.0797729	8.33E-22	5.34E-20	Btla
ENSMUSG00000052026.9	4.87791202	0.02830815	0.01048627	6.64748564	0.00992959	0.03171427	Slc6a7
ENSMUSG00000052031.9	232.919997	-0.0022096	0.00301643	0.54982326	0.45838992	0.6501077	Tagap1
ENSMUSG00000052033.14	145.823379	-0.0060628	0.0042267	2.08281545	0.14896519	0.28597534	Pfdn4
ENSMUSG00000052040.11	935.513997	0.00059972	0.00445683	0.01832744	0.89231232	0.97160722	Klf13
ENSMUSG00000052056.15	217.365325	0.0068639	0.00341019	3.99570947	0.04561624	0.11198009	Zfp217
ENSMUSG00000052062.15	342.668847	-0.0049054	0.00489089	0.95733371	0.32785956	0.51490849	Pard3b
ENSMUSG00000052075.8	1.33507966	-0.0284853	0.08864935	0.17280762	0.67762854	0.83350961	1700029F12Rik
ENSMUSG00000052085.8	623.312732	0.00327569	0.00338428	0.91277633	0.33937885	0.52703381	Dock8
ENSMUSG00000052087.15	11.9041062	0.05623497	0.00722814	55.362578	1.00E-13	2.37E-12	Rgs14
ENSMUSG00000052102.16	428.830756	0.0069251	0.00346571	3.97090563	0.04629287	0.11332264	Gnpda1
ENSMUSG00000052105.18	19.1349321	0.02268539	0.00902913	5.94724503	0.01474031	0.04424343	Mtcl1
ENSMUSG00000052117.6	259.278953	-0.0012874	0.00433389	0.08564096	0.76979369	0.89665436	D630039A03Rik
ENSMUSG00000052131.8	19.3395284	0.0774616	0.01062496	48.6637588	3.04E-12	6.03E-11	Akr1b7
ENSMUSG00000052133.17	45.0766578	0.02813877	0.00831763	9.00467738	0.0026929	0.01042591	Sema5b
ENSMUSG00000052135.9	42.0755745	0.00742933	0.01472689	0.2504393	0.61676592	0.78813993	Foxo6
ENSMUSG00000052137.12	133.275099	-0.0004537	0.00343743	0.01685098	0.89671572	0.97379762	Rbm12b2
ENSMUSG00000052139.19	401.589905	0.00169665	0.00236241	0.51386304	0.47347134	0.66420597	Babam2
ENSMUSG00000052142.16	36.4432811	0.0680197	0.00836414	57.6798352	3.08E-14	7.95E-13	Rasal3
ENSMUSG00000052144.10	1012.60379	0.00269888	0.00242257	1.22852364	0.26769435	0.44369915	Ppp4r2
ENSMUSG00000052146.17	5348.33501	0.00103692	0.00418736	0.06126508	0.80450749	0.91828391	Rps10
ENSMUSG00000052151.13	349.825832	0.01139831	0.00331706	11.8359557	0.00058098	0.00272696	Plpp2
ENSMUSG00000052155.6	482.857599	-0.0069087	0.00267561	6.57813957	0.01032387	0.03275036	Acvr2a
ENSMUSG00000052160.8	220.907119	0.04955078	0.00503173	90.7317838	1.65E-21	1.02E-19	Pld4
ENSMUSG00000052212.7	6.36883081	0.07750979	0.0139016	24.7720128	6.45E-07	5.75E-06	Cd177
ENSMUSG00000052214.10	889.550102	-0.0073354	0.00268629	7.32846115	0.00678711	0.02300674	Opa3
ENSMUSG00000052221.9	2.95852472	0.03072811	0.01356817	5.21033103	0.02245306	0.06265894	Ppp1r36
ENSMUSG00000052229.6	6.89518984	0.00578012	0.01170475	0.29816794	0.58503325	0.76201646	Gpr17
ENSMUSG00000052253.7	365.473499	-0.0021122	0.00230336	0.84461151	0.3580812	0.54693066	Zfp622
ENSMUSG00000052270.8	47.0252495	0.05898958	0.00825733	47.8363968	4.63E-12	8.94E-11	Fpr2
ENSMUSG00000052271.8	10.4626732	-0.0043445	0.0112054	0.14541787	0.70295324	0.85173405	Bhlha15
ENSMUSG00000052293.15	102.453829	-0.0010588	0.00754383	0.01924881	0.88965555	0.96970934	Taf9
ENSMUSG00000052296.8	1341.26502	-0.0032296	0.00177696	3.31329703	0.06872149	0.15591345	Ppp6r1
ENSMUSG00000052298.13	313.237595	0.02004527	0.0035854	30.6727792	3.05E-08	3.43E-07	Cdc42se2
ENSMUSG00000052299.10	665.699941	-0.0027204	0.00259974	1.08528934	0.2975173	0.48102845	Ltn1
ENSMUSG00000052301.15	5.43483486	-0.0027015	0.02223561	-0.0098637	1	1	Doc2a

ENSMUSG00000052302.7	164.168529	-0.0293965	0.00553968	27.077945	1.95E-07	1.93E-06	Tbc1d30
ENSMUSG00000052305.7	5717.98855	0.01691554	0.00780678	4.11540453	0.04249436	0.10569145	Hbb-bs
ENSMUSG00000052310.10	1454.30224	0.00375314	0.00229557	2.63143327	0.1047671	0.21757831	Slc39a1
ENSMUSG00000052316.10	5.78442147	0.01328271	0.00975048	1.91789003	0.16608945	0.31053395	Lrrc15
ENSMUSG00000052331.15	125.975937	0.02809384	0.00366137	56.9943887	4.37E-14	1.10E-12	Ankrd44
ENSMUSG00000052336.9	59.8693147	0.10054425	0.0075725	148.136404	4.43E-34	1.52E-31	Cx3cr1
ENSMUSG00000052337.16	1987.28724	-0.0099477	0.00211212	22.1673708	2.50E-06	2.01E-05	Immt
ENSMUSG00000052353.14	3.10041508	0.03708259	0.01978361	3.13129985	0.07680248	0.17068283	Cemip
ENSMUSG00000052364.5	18.0113396	0.01018282	0.00499138	4.04247194	0.04436879	0.10935028	B630019K06Rik
ENSMUSG00000052369.9	268.626618	0.00685301	0.00212037	10.3620983	0.00128629	0.00547922	Tmem106c
ENSMUSG00000052372.11	2.5520373	0.02582366	0.02193022	2.05344975	0.15186175	0.2902132	Il1rapl1
ENSMUSG00000052373.15	4.66645193	0.01577088	0.00981731	2.477461	0.11548855	0.2349416	Mpp3
ENSMUSG00000052374.17	98.8329648	0.06041016	0.02014913	1.33237631	0.24838291	0.41977352	Actn2
ENSMUSG00000052384.15	121.036824	0.03562747	0.00463156	57.084768	4.17E-14	1.05E-12	Nrros
ENSMUSG00000052387.17	11.8118651	0.0408375	0.01260859	9.74751748	0.00179565	0.00734467	Trpm3
ENSMUSG00000052392.6	1464.08832	0.02486598	0.00544944	20.1736383	7.07E-06	5.19E-05	Acot4
ENSMUSG00000052395.18	292.527147	-0.0046268	0.00289653	2.55222368	0.11013914	0.22645963	Rft1
ENSMUSG00000052396.8	5.16142589	0.01949812	0.00899265	4.78820021	0.02865535	0.07654192	Mogat2
ENSMUSG00000052397.9	259.595462	0.03634662	0.00509322	48.288585	3.68E-12	7.22E-11	Ezr
ENSMUSG00000052406.15	367.354683	0.00753784	0.00252955	8.83260371	0.00295896	0.01129288	Rexo4
ENSMUSG00000052407.18	122.544056	0.01503328	0.00425315	11.8544284	0.00057524	0.00270381	Ccdc171
ENSMUSG00000052415.6	26.7616564	-0.0047948	0.00826092	0.28752026	0.59181401	0.76762188	Tchh
ENSMUSG00000052417.7	1.06241436	-0.0107456	0.0322865	0.1415816	0.70671372	0.85451121	Or2t6
ENSMUSG00000052419.8	318.884962	0.00800006	0.00314572	6.37871904	0.01154968	0.03594282	Smim30
ENSMUSG00000052423.16	183.577928	-0.0023385	0.00335302	0.4870035	0.48526703	0.6741773	B4galt3
ENSMUSG00000052428.11	1797.22129	-0.0082108	0.00304539	7.16530411	0.00743271	0.0248206	Tmco1
ENSMUSG00000052430.16	4.27069854	0.06358921	0.01494915	17.2964557	3.20E-05	0.0002039	Bmpr1b
ENSMUSG00000052435.8	83.6958198	0.01160694	0.00429531	6.9472296	0.00839491	0.02750034	Cebpe
ENSMUSG00000052446.18	186.448443	0.0001343	0.00293138	0.0020757	0.96366109	1	Zfp961
ENSMUSG00000052456.10	496.450883	-0.0022402	0.00217116	1.06711832	0.30159726	0.48595935	Get3
ENSMUSG00000052459.14	1044.40711	0.00990132	0.00288263	11.6564431	0.00063981	0.00297173	Atp6v1a
ENSMUSG00000052469.10	1.80223644	-0.0462653	0.05019829	1.17941865	0.27747438	0.45628604	Tcp10c
ENSMUSG00000052477.16	48.8010753	0.17957182	0.00997049	218.196485	2.24E-49	3.10E-46	Sp140l2
ENSMUSG00000052485.8	8.57790086	0.02548481	0.01098355	4.73118881	0.02962041	0.07865648	Tmem171
ENSMUSG00000052488.8	461.216655	-0.0022351	0.00348337	0.40727557	0.52335557	0.70892775	Cherp
ENSMUSG00000052496.6	5.90875304	-0.0385457	0.01327838	8.01242704	0.00464574	0.01658963	Pkdrej
ENSMUSG00000052504.8	27.4940469	0.0096578	0.00530524	3.30318433	0.06914572	0.15663378	Epha3
ENSMUSG00000052512.19	924.716607	0.0109162	0.004831	5.00386013	0.02529085	0.06912853	Nav2
ENSMUSG00000052516.20	89.0895717	0.04518768	0.00519924	70.5461286	4.50E-17	1.64E-15	Robo2
ENSMUSG00000052520.8	9790.29225	-0.011879	0.00461856	6.46008017	0.01103247	0.03467976	Cyp2j5
ENSMUSG00000052525.15	5.35751102	-0.0052062	0.01008127	0.23908182	0.62487006	0.79382808	Spdya
ENSMUSG00000052533.15	160.253978	-0.0044611	0.00379688	1.36146061	0.24328451	0.4136468	Nup188
ENSMUSG00000052534.16	442.822187	0.0237245	0.00353203	44.2692563	2.86E-11	4.94E-10	Pbx1
ENSMUSG00000052539.16	557.986141	2.37E-05	0.00296637	6.16E-05	0.99373653	1	Magi3
ENSMUSG00000052544.10	41.6109647	-0.0025974	0.0083239	0.09081709	0.76314095	0.8923196	St6galnac3
ENSMUSG00000052551.17	3.55230094	0.03951172	0.0133764	8.33337981	0.00389232	0.01427748	Adarb2
ENSMUSG00000052557.12	217.828591	0.00396217	0.00431602	0.80763483	0.36882044	0.55938517	Gan
ENSMUSG00000052560.16	99.9390668	0.00803943	0.00581938	1.7245403	0.18910966	0.34307671	Cpne8
ENSMUSG00000052562.16	3540.1512	-0.0316585	0.00490072	40.3842352	2.09E-10	3.19E-09	Slc22a30
ENSMUSG00000052565.8	37.8300051	0.01338783	0.01036397	1.5440315	0.21401872	0.37692993	H1f3
ENSMUSG00000052566.9	412.420546	-0.0044161	0.00366578	1.44769761	0.22889775	0.39589223	Hook2
ENSMUSG00000052572.20	32.5109803	0.02108919	0.01017234	4.11026483	0.04262369	0.10595724	Dlg2
ENSMUSG00000052581.14	2.95081332	0.02732018	0.02394566	1.30219503	0.25381267	0.42611044	Lrrtm4
ENSMUSG00000052584.11	6.63621929	-0.0004565	0.01390084	-0.0025752	1	1	Serp2
ENSMUSG00000052593.17	432.483567	0.00925284	0.00298311	9.31885991	0.00226807	0.0090104	Adam17
ENSMUSG00000052595.9	1900.04061	-0.0107866	0.00525165	4.12900712	0.04215408	0.10498902	A1cf
ENSMUSG00000052605.13	232.2747	-0.0148062	0.00388282	14.2299219	0.00016178	0.00088244	Isoc2b
ENSMUSG00000052609.10	619.608175	-0.0033476	0.00303138	1.22818354	0.26776059	0.44377776	Plekhg3
ENSMUSG00000052613.18	10.9769792	0.00740399	0.0118198	0.24374552	0.6215139	0.79165236	Pcdh15
ENSMUSG00000052625.9	1.1217979	-0.0085655	0.03227103	0.04621908	0.82977806	0.93400521	Or7g32
ENSMUSG00000052632.17	300.678852	-0.0150462	0.00605156	5.72650682	0.01671069	0.04906838	Asap2

ENSMUSG00000052656.14	1858.46631	-0.0009693	0.00276998	0.12016722	0.72885319	0.86918452	Rnf103
ENSMUSG00000052675.12	34.4587859	-0.0117669	0.00444265	6.81087084	0.00906046	0.02938668	Zfp112
ENSMUSG00000052676.17	152.783369	0.01095859	0.00379238	8.03561211	0.00458665	0.01641044	Zmat1
ENSMUSG00000052681.9	1886.1961	0.01778296	0.00306345	32.2574666	1.35E-08	1.60E-07	Rap1b
ENSMUSG00000052684.6	626.688065	0.01887016	0.00572773	9.92679349	0.0016289	0.00673904	Jun
ENSMUSG00000052688.13	43.5371465	0.05929547	0.00709618	63.9443456	1.28E-15	3.90E-14	Rab7b
ENSMUSG00000052698.16	213.473096	0.00080246	0.00454963	0.02961546	0.86336565	0.95486666	Tln2
ENSMUSG00000052707.9	1953.59563	0.00358213	0.00500623	0.50120682	0.47897033	0.66893619	Tnrc6a
ENSMUSG00000052712.18	1523.19413	-0.0030681	0.00272266	1.26371806	0.26094848	0.43508631	BC004004
ENSMUSG00000052713.10	73.4633306	0.03522373	0.00414648	70.2461673	5.23E-17	1.89E-15	Zfp608
ENSMUSG00000052726.17	39.0388157	0.00676422	0.00665114	0.99183658	0.31929391	0.50517585	Kcnt2
ENSMUSG00000052727.7	174.443424	0.025841	0.00876398	8.00045043	0.00467657	0.01668708	Map1b
ENSMUSG00000052736.16	8.31658557	0.03803663	0.00873205	17.3585521	3.10E-05	0.00019799	Klrc2
ENSMUSG00000052738.15	3382.95837	-0.0113471	0.00267315	18.3367207	1.85E-05	0.00012478	Suclg1
ENSMUSG00000052748.15	318.661334	-0.0114108	0.00391097	8.38475663	0.00378381	0.01392494	Swt1
ENSMUSG00000052749.11	16.6135345	0.03791727	0.00736413	23.3807778	1.33E-06	1.12E-05	Trim30b
ENSMUSG00000052751.17	822.59071	-0.0010024	0.00284821	0.12123286	0.72770118	0.86851331	Repin1
ENSMUSG00000052752.16	254.987213	0.00202971	0.00410809	0.24117174	0.6233611	0.79279322	Traf7
ENSMUSG00000052760.17	20.5939243	0.06539117	0.00735518	69.981576	5.99E-17	2.14E-15	A630001G21Rik
ENSMUSG00000052763.8	130.029532	0.00346033	0.00174474	3.92410931	0.0475984	0.11589228	Zfp212
ENSMUSG00000052776.11	123.132167	0.04731105	0.00609138	58.3807217	2.16E-14	5.66E-13	Oas1a
ENSMUSG00000052783.17	34.798971	-0.0144002	0.0076529	3.24145115	0.07179702	0.16159982	Grk4
ENSMUSG00000052794.14	84.6029695	-0.0082565	0.00622123	1.61391019	0.20394302	0.36372023	1700030K09Rik
ENSMUSG00000052798.15	90.716555	0.00893387	0.00365614	5.71681967	0.01680314	0.04929952	Nup107
ENSMUSG00000052812.6	473.397446	-0.0053593	0.00417602	1.62724592	0.2020844	0.36112676	Atad2b
ENSMUSG00000052821.4	32.0893813	0.05967505	0.00740621	61.7268497	3.95E-15	1.13E-13	Cysltr1
ENSMUSG00000052833.10	379.017516	0.00202544	0.00348281	0.33453296	0.56300203	0.74379465	Sae1
ENSMUSG00000052837.8	544.230424	0.01728483	0.0056856	8.24310176	0.00409072	0.01490095	Junb
ENSMUSG00000052852.9	20.1306311	-0.0129013	0.00651103	4.08048932	0.04338109	0.10754312	Reep1
ENSMUSG00000052861.14	1.7151746	-0.0053236	0.01787617	0.08020662	0.7770176	0.90149203	Dnah6
ENSMUSG00000052889.12	124.600178	0.05583104	0.00596113	81.5103951	1.74E-19	8.52E-18	Prkcb
ENSMUSG00000052906.8	518.99071	-0.0031159	0.00293458	1.12867307	0.28805855	0.46911199	Ubxn8
ENSMUSG00000052911.10	173.135765	-0.0081291	0.00583492	1.87746881	0.17062211	0.31707321	Lamb2
ENSMUSG00000052914.5	1268.84393	-0.015074	0.00301038	24.9045809	6.02E-07	5.41E-06	Cyp2j6
ENSMUSG00000052915.15	1021.71855	-0.0050332	0.00279801	3.21426415	0.07299901	0.16378944	Msl1
ENSMUSG00000052917.16	160.688968	0.01031943	0.00323155	9.77565185	0.00176838	0.00724613	Senp7
ENSMUSG00000052920.17	65.7105597	0.0145247	0.00584682	5.8135688	0.01590299	0.04708019	Prkg1
ENSMUSG00000052921.14	134.806404	-0.0143017	0.00352118	16.6902886	4.40E-05	0.0002737	Arhgef15
ENSMUSG00000052926.17	344.098877	-0.007191	0.00403837	3.18500777	0.07431664	0.1661295	Rnaseh2a
ENSMUSG00000052928.10	555.182333	-0.0057235	0.00338099	2.76046393	0.09662021	0.20467796	Ctif
ENSMUSG00000052934.15	782.918032	-0.0244088	0.00345273	49.5319714	1.95E-12	3.94E-11	Fbxo31
ENSMUSG00000052942.14	31.3897164	0.02835602	0.00784686	11.9384034	0.00054989	0.00260173	Glis3
ENSMUSG00000052949.15	60.3214902	0.0281839	0.00692141	15.5067027	8.22E-05	0.00047938	Rnf157
ENSMUSG00000052957.9	296.432236	-0.0018061	0.00385097	0.21627584	0.64189231	0.80805252	Gas1
ENSMUSG00000052962.4	412.267025	-0.010866	0.00292469	13.7180338	0.00021241	0.00112554	Mrpl35
ENSMUSG00000052974.9	15750.8452	-0.0286351	0.00493322	32.9352873	9.53E-09	1.15E-07	Cyp2f2
ENSMUSG00000052981.7	5.4468608	-0.0019924	0.01036061	0.0287818	0.86528383	0.95599514	Ube2ql1
ENSMUSG00000052997.16	583.710395	0.00233258	0.00188474	1.51302084	0.21867871	0.38302824	Uba2
ENSMUSG00000053004.10	3.19255596	0.07820422	0.01005073	55.8030968	8.01E-14	1.93E-12	Hrh1
ENSMUSG00000053007.10	23.9581889	0.06554408	0.00950504	44.6723667	2.33E-11	4.09E-10	Creb5
ENSMUSG00000053012.18	2993.54916	0.00158345	0.00369236	0.1845497	0.66749211	0.82629235	Krcc1
ENSMUSG00000053024.15	13.9980561	0.03030254	0.00692835	18.0750403	2.12E-05	0.00014129	Cntn2
ENSMUSG00000053025.14	18.7132882	0.02429835	0.01533885	3.16170669	0.07538433	0.16804227	Sv2b
ENSMUSG00000053030.12	0.6958066	-0.0085957	0.05328797	0.07614247	0.78259473	0.90508382	Spink2
ENSMUSG00000053040.14	38.8311231	0.00183293	0.00807776	0.05046044	0.82226401	0.92917429	Aph1c
ENSMUSG00000053044.9	10.6808063	0.12870037	0.00992982	136.758326	1.36E-31	3.49E-29	Cd8b1
ENSMUSG00000053046.17	11.3027085	0.12972498	0.01133488	113.635975	1.57E-26	1.84E-24	Brsk2
ENSMUSG00000053062.17	268.003079	-0.0048361	0.00447492	1.16261556	0.28092376	0.46051346	Jam2
ENSMUSG00000053063.12	220.838149	0.07288987	0.00649706	109.564782	1.22E-25	1.25E-23	Clec12a
ENSMUSG00000053070.6	4.10078902	0.00555066	0.00845341	0.44770347	0.50342756	0.69033749	Cfap300
ENSMUSG00000053080.11	62.7774434	0.00086319	0.00377386	0.05184368	0.81988563	0.92769597	Zfta

ENSMUSG00000053091.17	69.3028008	-0.0044617	0.00357511	1.51321484	0.21864918	0.38302824	Lins1
ENSMUSG00000053093.17	124.866656	0.00698767	0.02349219	-0.3324389	1	1	Myh7
ENSMUSG00000053094.16	1176.082	0.00481931	0.00241258	3.97678984	0.0461314	0.11303296	Tmem248
ENSMUSG00000053101.4	12.9701554	0.08783197	0.01160027	48.2139986	3.82E-12	7.46E-11	Gpr141
ENSMUSG00000053110.14	1080.5928	-0.0078469	0.00220764	12.4926241	0.00040856	0.0020009	Yap1
ENSMUSG00000053111.14	2.04347081	-0.0202988	0.01881636	1.28684995	0.25662928	0.42979892	Fank1
ENSMUSG00000053113.4	213.287653	0.00482112	0.00690209	0.43062685	0.51168152	0.6984123	Socs3
ENSMUSG00000053119.12	2215.13836	-9.86E-05	0.00309523	0.00101246	0.9746162	1	Chmp3
ENSMUSG00000053128.16	226.666001	-0.0049837	0.00253142	3.88097371	0.04883623	0.11829632	Rnf26
ENSMUSG00000053129.6	6.00367317	0.00726169	0.10652343	0.0262461	0.87130074	0.95940269	Gsx1
ENSMUSG00000053134.14	180.224048	-0.00163	0.00263543	0.37479187	0.5404038	0.72412022	Supt7l
ENSMUSG00000053137.8	25.8020275	0.01935635	0.00489815	15.4769292	8.35E-05	0.00048627	Mapk11
ENSMUSG00000053141.17	18.5976067	-0.0255138	0.01033064	4.96911973	0.02580378	0.07031052	Ptprt
ENSMUSG00000053146.3	1.22355608	0.00694583	0.03083689	0.21599781	0.64210645	0.8081677	Or1j20
ENSMUSG00000053153.15	2.11007092	-0.009345	0.02059913	0.16933554	0.68070301	0.83605273	Spag16
ENSMUSG00000053158.11	142.329436	0.03060805	0.00477231	40.7733346	1.71E-10	2.65E-09	Fes
ENSMUSG00000053164.7	11.1511183	0.00723189	0.01371143	0.2700171	0.6033203	0.77648764	Gpr21
ENSMUSG00000053166.15	1.98166742	0.00916316	0.02538575	0.29306808	0.58826111	0.76503785	Cdh22
ENSMUSG00000053175.18	385.178311	-0.0085997	0.00483506	2.96598277	0.08503277	0.18501202	Bcl3
ENSMUSG00000053178.6	18.6222275	-0.0051238	0.00552097	0.85501528	0.3551374	0.54383038	Mterf1b
ENSMUSG00000053182.12	7.68507761	0.01883075	0.00908566	4.35273966	0.03694946	0.09422532	Cd200l1
ENSMUSG00000053192.10	36.1382639	0.02247811	0.0066452	11.1512538	0.00083975	0.00376689	Mllt11
ENSMUSG00000053198.15	5.66769026	0.01448839	0.00679122	4.49143641	0.03406504	0.08833174	Prx
ENSMUSG00000053199.14	12.5294932	0.03457498	0.01329594	6.09168687	0.01358194	0.04122256	Arhgap20
ENSMUSG00000053205.10	292.004804	0.00220986	0.00373477	0.34874009	0.5548272	0.73704052	Styx
ENSMUSG00000053219.16	197.043571	0.06316462	0.0063571	87.4178877	8.79E-21	4.92E-19	Raet1e
ENSMUSG00000053226.8	60.1874209	-0.0080924	0.0028404	8.24987405	0.00407548	0.01485923	Dand5
ENSMUSG00000053253.17	806.75476	-0.0024208	0.00206873	1.37363445	0.24118842	0.41109047	Ndfip2
ENSMUSG00000053279.9	18518.6587	0.00174827	0.00448244	0.14114057	0.70714975	0.85475498	Aldh1a1
ENSMUSG00000053286.12	384.458189	-0.0053012	0.00205835	6.58284499	0.01029662	0.03267708	Trmt1l
ENSMUSG00000053287.5	1.90552866	-0.0004548	0.0357642	-0.0068968	1	1	Or9g19
ENSMUSG00000053289.9	300.250507	-0.0008006	0.0028025	0.0810343	0.77590062	0.90074283	Ddx10
ENSMUSG00000053291.16	227.746712	0.00657057	0.00306962	4.57494439	0.0324428	0.08485206	Rab4b
ENSMUSG00000053293.10	308.920819	-0.0001149	0.00343343	0.00111886	0.97331628	1	Pom121
ENSMUSG00000053297.15	3.812468	0.06621565	0.01062808	37.0918605	1.13E-09	1.55E-08	AI854703
ENSMUSG00000053303.15	680.302608	0.17387572	0.01110659	188.794417	5.83E-43	5.73E-40	Slc22a26
ENSMUSG00000053310.12	58.0457758	0.05869396	0.01042319	29.7579878	4.89E-08	5.33E-07	Nrgn
ENSMUSG00000053317.12	1269.71407	-0.0124106	0.00403777	9.54124849	0.00200905	0.00811395	Sec61b
ENSMUSG00000053318.8	30.2617325	0.0879399	0.00704811	142.125502	9.13E-33	2.73E-30	Slamf8
ENSMUSG00000053329.8	1505.38722	-0.007686	0.00325079	5.62125742	0.01774391	0.05155944	Gatd3a
ENSMUSG00000053333.16	259.842128	-0.0039551	0.00211779	3.5057667	0.06115553	0.1421503	Dis3l2
ENSMUSG00000053334.7	169.261812	-0.0049291	0.00327383	2.31324377	0.12827611	0.25532833	Ficd
ENSMUSG00000053338.10	1.02218905	0.09310739	0.01923123	24.3747411	7.93E-07	6.92E-06	Tarm1
ENSMUSG00000053347.16	315.617965	-0.0022224	0.00280768	0.61765558	0.43191982	0.62345538	Zfp943
ENSMUSG00000053390.17	118.119026	0.0006076	0.00278955	0.04703616	0.82830325	0.93287941	Zfp952
ENSMUSG00000053395.16	2.01988434	0.05898086	0.0189744	11.6015804	0.00065896	0.00304928	Cacng8
ENSMUSG00000053398.12	22.4483857	0.08151494	0.01174314	36.7486336	1.34E-09	1.83E-08	Phgdh
ENSMUSG00000053399.10	1.60336464	0.00533166	0.02058992	0.17641758	0.67447017	0.83155036	Adamts18
ENSMUSG00000053411.17	204.240454	0.01072722	0.00348377	9.45054235	0.00211087	0.00847292	Cbx7
ENSMUSG00000053414.9	67.3538938	0.01340531	0.00488505	7.20525703	0.00726903	0.02439491	Hunk
ENSMUSG00000053436.16	1473.82109	-0.0053698	0.00216691	6.07826087	0.01368554	0.04150537	Mapk14
ENSMUSG00000053441.6	2.87082927	0.01120067	0.01856736	0.64802387	0.42082008	0.61241825	Adamts19
ENSMUSG00000053453.10	840.018943	-0.0077976	0.00290854	7.25863266	0.0070561	0.02377163	Thoc7
ENSMUSG00000053460.9	1019.90243	-0.0096672	0.0025962	13.8673706	0.00019617	0.00104873	Ggcx
ENSMUSG00000053461.10	35.8846365	0.09307802	0.00973524	73.2861405	1.12E-17	4.40E-16	Hhipl2
ENSMUSG00000053469.15	2.28897837	0.10714757	0.01974831	23.7652458	1.09E-06	9.30E-06	Tg
ENSMUSG00000053470.14	368.357442	0.00735311	0.00302122	5.84054798	0.01566095	0.04648617	Kdm3a
ENSMUSG00000053475.6	3.48507364	0.00744821	0.01160634	0.39414634	0.53012792	0.71465461	Tnfaip6
ENSMUSG00000053477.19	734.155983	0.02509223	0.00361283	46.7128852	8.22E-12	1.54E-10	Tcf4
ENSMUSG00000053483.16	263.777929	0.00781903	0.00331602	5.51892926	0.0188117	0.05410907	Usp21
ENSMUSG00000053510.13	1989.90841	0.00082465	0.00373216	0.04976503	0.82347264	0.92974752	Nrdc

ENSMUSG00000053519.18	1.70378657	0.02146164	0.02591611	1.18000948	0.27735406	0.45618361	Kcnip1
ENSMUSG00000053536.8	543.504251	0.00377577	0.00217053	2.9582274	0.08544156	0.18570076	Cstf2t
ENSMUSG00000053550.14	9.66764862	-0.0420458	0.0122449	10.7488423	0.00104345	0.00455968	Shisa7
ENSMUSG00000053552.15	5.63706481	0.00734415	0.0076047	0.95379042	0.32875635	0.51580172	Ebf4
ENSMUSG00000053553.12	107.889579	-0.0281374	0.00584072	22.0927855	2.60E-06	2.08E-05	3110082I17Rik
ENSMUSG00000053559.14	392.265742	-0.0105255	0.00361493	8.48269597	0.0035854	0.0133069	Smagp
ENSMUSG00000053560.6	462.34995	-0.0008004	0.00443116	0.03143316	0.85927747	0.95283438	Ier2
ENSMUSG00000053565.11	776.576729	0.00050638	0.00293294	0.02989143	0.86273681	0.9546284	Eif3k
ENSMUSG00000053574.6	4.82870605	0.0012287	0.00708171	0.02901257	0.86475002	0.95571876	4930563E22Rik
ENSMUSG00000053580.17	186.775171	0.01828275	0.00535817	11.1067471	0.00086014	0.00383793	Tanc2
ENSMUSG00000053581.14	230.176457	0.00721624	0.004062	3.04743054	0.08086499	0.17766945	Zfand2a
ENSMUSG00000053600.9	75.782181	0.00644064	0.00371656	2.92019995	0.08747702	0.18930481	Zfp472
ENSMUSG00000053604.6	106.142182	0.0042692	0.00399619	1.13951037	0.28575581	0.46626334	Rpia
ENSMUSG00000053617.13	617.113118	-0.0126098	0.00441076	7.86319007	0.00504513	0.01782955	Sh3pxd2a
ENSMUSG00000053624.5	39.6289071	-0.0071816	0.02752476	-0.0198073	1	1	Gykl1
ENSMUSG00000053626.6	2.16723593	0.02246487	0.01389004	2.83723218	0.09210274	0.19712661	Tll1
ENSMUSG00000053641.10	656.466628	0.00459251	0.00312432	2.09304338	0.14797104	0.28448361	Dennd4a
ENSMUSG00000053644.14	7547.01906	-0.0011274	0.0033665	0.1100427	0.74009553	0.87693558	Aldh7a1
ENSMUSG00000053646.14	906.108679	0.00289175	0.00386256	0.54921727	0.45863769	0.65026119	Plxnb1
ENSMUSG00000053684.9	81.7254511	0.00172134	0.00284416	0.3601802	0.54840618	0.73114624	Kics2
ENSMUSG00000053687.16	11.2393079	0.08273001	0.01099245	47.9638906	4.34E-12	8.41E-11	Dpep2
ENSMUSG00000053693.17	14.6482117	-0.0087551	0.0075147	1.29991758	0.25422828	0.42653494	Mast1
ENSMUSG00000053702.17	285.270931	0.11165824	0.00854281	135.012088	3.28E-31	7.98E-29	Nebl
ENSMUSG00000053716.10	109.719855	0.00682244	0.00452231	2.25621345	0.1330791	0.26256896	Dusp7
ENSMUSG00000053730.16	52.6628971	-0.0015306	0.00386274	0.15476624	0.69402155	0.84563839	Tmem39b
ENSMUSG00000053746.9	13.605196	0.00032681	0.00576432	0.00313565	0.95534428	1	Ptrh1
ENSMUSG00000053754.15	845.967394	-0.0029212	0.00429835	0.45391662	0.50048086	0.68749805	Chd8
ENSMUSG00000053768.14	1677.26065	-0.0142291	0.00279351	26.1092398	3.23E-07	3.05E-06	Chchd3
ENSMUSG00000053769.6	198.751894	-0.0056946	0.00194007	8.58798861	0.00338388	0.01267449	Lysmd1
ENSMUSG00000053774.10	630.05752	-0.0065195	0.00306839	4.40274302	0.03588117	0.09208851	Ubxn7
ENSMUSG00000053783.6	4.20819513	-0.0183319	0.02903096	0.41559123	0.51914517	0.70520838	Liat1
ENSMUSG00000053799.9	449.213922	0.00731742	0.00211188	11.8426803	0.00057888	0.00271929	Exoc6
ENSMUSG00000053801.19	165.780876	-0.0090152	0.00324621	7.92396382	0.00487843	0.01731306	Grwd1
ENSMUSG00000053819.17	301.663493	0.02344762	0.00412818	31.2121055	2.31E-08	2.65E-07	Camk2d
ENSMUSG00000053825.16	34.2679588	0.00318423	0.00772755	0.15534104	0.69348264	0.84516517	Ppfia2
ENSMUSG00000053835.18	69.7570294	0.01679952	0.00464552	12.8680003	0.00033425	0.00167522	H2-T24
ENSMUSG00000053838.15	564.018959	0.00245997	0.00194991	1.6097813	0.20452255	0.36451295	Nudcd3
ENSMUSG00000053841.16	556.405722	-0.0078108	0.00273692	8.05786069	0.00453066	0.0162508	Txlna
ENSMUSG00000053846.6	309.292958	-0.0078277	0.00537911	2.0757845	0.14965298	0.28703872	Lipg
ENSMUSG00000053852.12	2.48728359	0.01763675	0.04071184	0.8546169	0.35524951	0.54383819	Adgrg4
ENSMUSG00000053862.7	13.9450082	0.05068383	0.00910311	30.964714	2.63E-08	2.98E-07	Slc51b
ENSMUSG00000053870.8	334.918274	0.00494311	0.00281313	2.99662525	0.08343815	0.18215219	Fpgt
ENSMUSG00000053877.14	799.924062	-0.001736	0.00363053	0.22539688	0.63495818	0.80262198	Srcap
ENSMUSG00000053886.5	131.299387	0.01063053	0.00392615	7.59299984	0.00585954	0.02025546	Sh2d4a
ENSMUSG00000053896.15	1.25550256	-0.0195152	0.18483702	0.01636854	0.89819678	0.97482373	4933409G03Rik
ENSMUSG00000053897.16	553.906598	-0.0021354	0.00251115	0.71690058	0.39716246	0.58900112	Slc39a8
ENSMUSG00000053898.13	4787.47215	-0.0202504	0.00379882	27.8748617	1.29E-07	1.32E-06	Ech1
ENSMUSG00000053907.11	1997.70808	0.00331681	0.00356659	0.8559141	0.35488463	0.54359715	Mat2a
ENSMUSG00000053916.5	260.7829	-0.0029649	0.00315643	0.86640013	0.35195384	0.54044566	Nanp
ENSMUSG00000053929.19	358.39164	-0.011123	0.00266289	17.4967142	2.88E-05	0.00018583	Zftraf1
ENSMUSG00000053930.14	6.75314382	0.04806804	0.01291009	12.1050304	0.00050286	0.0024087	Shisa6
ENSMUSG00000053931.12	2427.78089	0.00320296	0.00352744	0.82903214	0.36255254	0.55207982	Cnn3
ENSMUSG00000053950.11	170.292687	0.00421138	0.00243417	2.89886798	0.08864178	0.1911764	Adnp2
ENSMUSG00000053963.8	17.6265722	0.02441372	0.01014691	5.03128771	0.02489338	0.06819226	Stum
ENSMUSG00000053964.18	218.920348	-0.0204127	0.00545507	13.5078451	0.00023757	0.00124131	Lgals4
ENSMUSG00000053965.11	36.3944933	0.00866939	0.00419567	4.21829695	0.03999028	0.10058769	Pde5a
ENSMUSG00000053977.14	21.0106033	0.14487062	0.00920125	196.02839	1.54E-44	1.73E-41	Cd8a
ENSMUSG00000053985.11	30.4734653	-0.005367	0.00432287	1.56103218	0.21151415	0.37373913	Zfp14
ENSMUSG00000054000.6	41.729883	0.02241893	0.0033298	45.0118379	1.96E-11	3.48E-10	Tusc1
ENSMUSG00000054008.10	2839.34951	-0.0019052	0.00298831	0.40175854	0.52618247	0.71124822	Ndst1
ENSMUSG00000054013.7	2.105194	0.01144036	0.01911952	0.75545812	0.38475364	0.57643393	Tmem179

ENSMUSG00000054021.7	234.418879	-0.0049876	0.0031166	2.63295064	0.10466704	0.21744709	Sirt5
ENSMUSG00000054027.9	268.113773	-0.0048866	0.00343153	2.00684544	0.15659063	0.29691668	Nt5dc3
ENSMUSG00000054034.11	3.87869313	0.06377087	0.01858473	12.7699807	0.00035223	0.00175704	Tceal5
ENSMUSG00000054051.8	242.682084	0.00539789	0.00359	2.27195038	0.13173403	0.26056164	Ercc6
ENSMUSG00000054052.6	0.95486098	0.01824017	0.01531194	2.01401648	0.15585223	0.29584956	Rdh19
ENSMUSG00000054065.13	24.886142	0.05500282	0.00643256	65.4350416	6.01E-16	1.90E-14	Pkp3
ENSMUSG00000054072.13	7172.11411	0.01273257	0.00447914	7.98444252	0.0047181	0.01681238	Iigp1
ENSMUSG00000054074.11	15.63639	0.01708604	0.00668296	6.10516861	0.01347871	0.04096166	Skida1
ENSMUSG00000054079.13	251.371818	0.00020754	0.00216672	0.0090727	0.92411568	0.98864086	Utp18
ENSMUSG00000054083.9	7.73823176	0.03638757	0.01525568	-4.6036304	1	1	Capn12
ENSMUSG00000054091.10	504.071661	-0.0065277	0.00448461	2.10028999	0.1472712	0.28353103	1810037I17Rik
ENSMUSG00000054099.12	97.6352232	0.00783536	0.00290303	7.15003113	0.00749627	0.02499745	Slc25a40
ENSMUSG00000054106.8	3.84746979	-0.3345649	0.11782505	0.14550158	NA	NA	Try4
ENSMUSG00000054115.13	56.239026	0.00537477	0.00494567	1.16925694	0.27955397	0.45893577	Skp2
ENSMUSG00000054134.16	1.42062029	0.05516299	0.01641799	9.73269384	0.00181019	0.00739694	Umodl1
ENSMUSG00000054141.6	1.06691697	0.0019861	0.02895468	0.01462848	0.90373202	0.97767212	Or1m1
ENSMUSG00000054142.6	2.28809341	-0.0121726	0.0226219	0.27336398	0.60108406	0.77489467	Vmn1r236
ENSMUSG00000054146.9	0.746092	0.00329736	0.06452255	0.00792938	0.92904452	0.99119297	Krt15
ENSMUSG00000054150.13	78.3052971	0.00371905	0.00466879	0.61213879	0.43398365	0.62561169	Syne3
ENSMUSG00000054161.13	2.85981396	0.00514696	0.01446441	0.13581464	0.71247779	0.85824827	Fam83e
ENSMUSG00000054162.16	15.7130968	0.02556788	0.0126878	3.45323782	0.06312799	0.14577412	Spock3
ENSMUSG00000054169.8	0.63129674	-0.0025813	0.02658663	0.04216595	0.83730377	0.93875775	Ceacam10
ENSMUSG00000054191.10	46.2412951	0.00633659	0.00448531	2.0285859	0.15436418	0.29371772	Klf1
ENSMUSG00000054196.8	1.39754241	0.02004601	0.01778556	1.63002006	0.20170027	0.36059294	Cthrc1
ENSMUSG00000054199.19	430.247823	-0.0001398	0.00372742	0.00140389	0.97011148	1	Gon4l
ENSMUSG00000054200.8	3.80510142	0.02043119	0.00949804	4.61495959	0.03169424	0.08324503	Ffar4
ENSMUSG00000054203.8	69.8572586	0.05630506	0.00720341	54.4922027	1.56E-13	3.63E-12	Ifi205
ENSMUSG00000054204.7	0.80497357	-0.0260349	0.04672005	0.41099599	0.52146441	0.70746389	Alkal2
ENSMUSG00000054206.3	2.86855057	0.03631045	0.01030396	11.8109288	0.00058884	0.00275892	Gzmm
ENSMUSG00000054226.14	2280.62035	-0.004613	0.00328084	1.96198007	0.16130171	0.30345874	Tprkb
ENSMUSG00000054236.7	1.30341875	-0.0050795	0.03280468	-0.0255597	1	1	Or5p4
ENSMUSG00000054237.7	244.392925	-0.0068323	0.00220351	9.64137518	0.00190243	0.00774258	Fra10ac1
ENSMUSG00000054252.19	581.770039	0.00516882	0.00386454	1.74173089	0.18691966	0.34009775	Fgfr3
ENSMUSG00000054256.12	5.32860863	-0.0091761	0.00989874	0.72676978	0.39393224	0.58530427	Msi1
ENSMUSG00000054263.13	5438.4935	-0.0477066	0.0048508	93.8362044	3.43E-22	2.35E-20	Lifr
ENSMUSG00000054277.9	186.341725	0.01279639	0.00416339	9.46061261	0.00209931	0.00843227	Arfgap3
ENSMUSG00000054280.15	521.711691	0.00448711	0.00658302	0.43933072	0.50744566	0.69427539	Prr14l
ENSMUSG00000054293.11	23.2068846	0.04615427	0.00733204	35.2985758	2.83E-09	3.70E-08	P2ry10b
ENSMUSG00000054302.17	564.546036	-0.0068169	0.00249653	7.39211369	0.00655105	0.02231879	Eapp
ENSMUSG00000054309.10	684.588749	-0.0020578	0.00280856	0.52776953	0.46754611	0.65867534	Cpsf3
ENSMUSG00000054312.7	964.052381	-0.0129121	0.00351673	13.5192559	0.00023613	0.00123488	Mrps21
ENSMUSG00000054321.8	51.2059762	0.00163922	0.00364415	0.19641776	0.657628	0.81933411	Taf4b
ENSMUSG00000054342.9	13.715554	0.05706925	0.0082426	40.5266378	1.94E-10	2.98E-09	Kcnn4
ENSMUSG00000054364.6	522.316702	0.01642212	0.00324176	24.4888726	7.47E-07	6.56E-06	Rhob
ENSMUSG00000054381.6	80.9456867	-0.0006849	0.00294618	0.05334095	0.81734853	0.92606919	Zfp747
ENSMUSG00000054383.3	1.9782964	-0.0135939	0.01262933	1.10896291	0.2923075	0.47423062	Pnma1
ENSMUSG00000054385.15	29.3692409	0.06255951	0.00954383	39.0110341	4.21E-10	6.17E-09	Ceacam2
ENSMUSG00000054387.14	862.181855	0.00371501	0.00328612	1.2690019	0.25995401	0.43386657	Mdm4
ENSMUSG00000054400.17	40.6410981	0.02965757	0.00605203	22.7128671	1.88E-06	1.55E-05	Cklf
ENSMUSG00000054404.14	362.577107	0.02070089	0.00403687	25.4468256	4.55E-07	4.18E-06	Slfn5
ENSMUSG00000054405.15	846.838725	0.00431805	0.00282994	2.30835299	0.12868033	0.25590678	Dnajc8
ENSMUSG00000054408.10	1717.38854	-0.0002996	0.00224281	0.01754143	0.89463306	0.97287826	Spcs3
ENSMUSG00000054414.5	476.081249	0.00539983	0.00389462	1.86237805	0.17235059	0.31960539	Slc30a7
ENSMUSG00000054417.6	904.016658	0.05613064	0.01009978	31.7184047	1.78E-08	2.07E-07	Cyp3a44
ENSMUSG00000054422.8	63168.6558	-0.0318903	0.0065452	22.7694945	1.83E-06	1.51E-05	Fabp1
ENSMUSG00000054423.16	15.0867241	0.02819188	0.01671986	3.14309438	0.07624902	0.16966126	Cadps
ENSMUSG00000054428.13	443.664196	0.00561594	0.00439323	1.62803972	0.2019744	0.36100092	Atp5if1
ENSMUSG00000054434.15	69.1730244	-0.0139113	0.00410086	11.410413	0.00073034	0.00333376	Tmem120b
ENSMUSG00000054435.17	290.241435	0.02591822	0.00370043	46.9020482	7.46E-12	1.40E-10	Gimap4
ENSMUSG00000054452.11	7203.74344	-0.0071438	0.00281338	6.34186599	0.01179208	0.0365621	Tle5
ENSMUSG00000054453.12	22.400489	0.0612216	0.00909363	41.178522	1.39E-10	2.19E-09	Sytl5

ENSMUSG00000054455.6	1332.53231	-0.0044914	0.0034755	1.64455651	0.19970144	0.35788756	Vapb
ENSMUSG00000054459.9	32.852882	0.01943471	0.01694053	1.64866233	0.19914109	0.35704613	Vsnl1
ENSMUSG00000054469.15	868.539056	0.00122252	0.00364343	0.10892908	0.74136667	0.87749657	Lclat1
ENSMUSG00000054474.16	784.009892	-0.0110648	0.00310058	12.9664072	0.00031713	0.00160031	Thnsl2
ENSMUSG00000054477.18	417.97027	-0.0019652	0.00322394	0.37697669	0.53922572	0.72317591	Kcnn2
ENSMUSG00000054484.15	254.239867	-0.006087	0.00371296	2.59883378	0.10694238	0.22118055	Tmem62
ENSMUSG00000054498.7	3.29106729	-0.0018643	0.03678776	-0.0215222	1	1	Or6f1
ENSMUSG00000054499.10	308.980984	-0.0064759	0.00302873	4.59884695	0.03199347	0.08387217	Dedd2
ENSMUSG00000054509.8	283.643169	0.0110718	0.00417412	7.02958116	0.00801741	0.02644366	Parp4
ENSMUSG00000054517.10	194.065147	0.00168734	0.00267457	0.39023423	0.5321763	0.7167196	Trim65
ENSMUSG00000054519.9	115.87354	-0.0067562	0.00305863	4.82510989	0.02804808	0.07525218	Zfp867
ENSMUSG00000054520.16	316.945795	0.01098274	0.00365849	8.90050585	0.00285092	0.01093717	Sh3bp2
ENSMUSG00000054523.14	0.76630964	-0.0213235	0.18442659	0.02461953	0.87531883	0.96163165	Ms4a5
ENSMUSG00000054537.9	1.76437661	-0.0016913	0.01427615	0.02743146	0.86845251	0.95800441	Tmprss11e
ENSMUSG00000054545.18	2381.8948	-0.0112686	0.0038683	8.36429701	0.00382665	0.01406722	Ugt1a6a
ENSMUSG00000054555.12	114.493184	0.02453037	0.00791556	9.30944809	0.00227975	0.00904613	Adam12
ENSMUSG00000054580.15	113.855496	0.0138785	0.00433831	9.9835377	0.00157946	0.00655403	Pla2r1
ENSMUSG00000054582.5	1.77979093	0.025901	0.01621208	2.50842401	0.11323913	0.23132323	Pabpc1l
ENSMUSG00000054604.8	1006.45554	-0.000346	0.00256599	0.01789417	0.89358518	0.97245882	Cggbp1
ENSMUSG00000054611.18	1177.72461	-0.001409	0.0036323	0.14759465	0.70084467	0.85062771	Kdm2a
ENSMUSG00000054612.5	320.18418	0.00363105	0.0034986	1.0446792	0.30673571	0.49179881	Mgmt
ENSMUSG00000054619.8	4506.56673	-0.0094932	0.00283953	11.0648782	0.00087978	0.00391964	Tmt1a
ENSMUSG00000054626.12	23.5410033	0.00738537	0.00888273	0.63661892	0.42493779	0.61656893	Xlr
ENSMUSG00000054630.8	17096.8212	-0.0246185	0.00526181	21.3098759	3.91E-06	3.02E-05	Ugt2b5
ENSMUSG00000054640.17	255.841263	0.05270371	0.0074531	43.3738417	4.52E-11	7.59E-10	Slc8a1
ENSMUSG00000054641.11	18.1390956	0.00169883	0.00790914	0.04323879	0.8352763	0.93759884	Mmrn1
ENSMUSG00000054648.9	460.009023	-0.0007036	0.00248766	0.07946573	0.77802275	0.90218508	Zfp869
ENSMUSG00000054659.14	28.5761087	-0.005146	0.00566041	0.78151762	0.3766774	0.5677603	Pm20d2
ENSMUSG00000054662.9	2.79068203	0.01646487	0.01132398	2.03236291	0.15398105	0.29319547	Ano9
ENSMUSG00000054666.8	1.97337595	-0.0034898	0.02365198	0.00976883	0.92126736	0.98718877	Or10h28
ENSMUSG00000054667.8	1.63260527	0.00217524	0.038355	0.0116107	0.91419168	0.98312004	Irs4
ENSMUSG00000054672.14	1.65114467	0.08859676	0.01645414	28.3676936	1.00E-07	1.04E-06	Scart2
ENSMUSG00000054675.6	21.472174	0.01305166	0.00703553	3.32084326	0.06840673	0.15542252	Tmem119
ENSMUSG00000054676.18	2629.38827	-0.0074167	0.00225587	10.7393185	0.00104883	0.00457936	1600014C10Rik
ENSMUSG00000054690.18	83.1063562	0.00868063	0.00464627	3.31363461	0.06870737	0.15591034	Emcn
ENSMUSG00000054693.15	679.949353	0.01442859	0.00275033	26.7466416	2.32E-07	2.26E-06	Adam10
ENSMUSG00000054702.15	14.9355216	0.02203283	0.00646069	11.3719849	0.0007456	0.00339493	Ap1s3
ENSMUSG00000054708.18	89.189374	-0.0017873	0.00445244	0.16106808	0.68817506	0.8415667	Ankrd24
ENSMUSG00000054715.12	98.4942228	-0.0001969	0.00278597	0.00505461	0.94332151	0.99877588	Zscan22
ENSMUSG00000054716.5	211.537621	-0.0055947	0.0044951	1.52438658	0.21695688	0.38083019	Zfp771
ENSMUSG00000054717.8	242.416496	0.02983358	0.00632646	20.9953441	4.60E-06	3.50E-05	Hmgb2
ENSMUSG00000054720.13	129.632354	0.01866314	0.00501762	13.0320442	0.00030621	0.00155349	Lrrc8c
ENSMUSG00000054723.7	219.716893	0.01188635	0.0030408	15.1217874	0.00010079	0.00057562	Vmac
ENSMUSG00000054728.18	69.7271632	0.01844974	0.0072465	6.17777407	0.01293649	0.03954382	Phactr1
ENSMUSG00000054733.10	2402.71939	-0.0028533	0.00257084	1.22903145	0.26759548	0.44359762	Msra
ENSMUSG00000054737.12	92.495955	0.01415498	0.00339657	17.1072831	3.53E-05	0.00022392	Zfp182
ENSMUSG00000054752.18	34.4700444	-0.0050874	0.0070326	0.49539446	0.48153078	0.6710816	Fsd1l
ENSMUSG00000054757.15	2091.90341	0.00205164	0.00464278	0.19483215	0.65892492	0.82029538	Akr1c20
ENSMUSG00000054764.10	44.1234838	0.02482348	0.00680312	11.6107799	0.00065571	0.00303662	Mtnr1a
ENSMUSG00000054766.14	1900.89635	0.00421908	0.00176289	5.63523622	0.01760297	0.05122563	Set
ENSMUSG00000054770.17	103.76111	0.0110386	0.00271434	16.3881817	5.16E-05	0.00031525	Kctd18
ENSMUSG00000054792.16	137.415945	0.00022283	0.00229538	0.00933849	0.92301559	0.98811936	Klhl18
ENSMUSG00000054793.9	115.830712	-0.009819	0.00574848	2.76437927	0.09638407	0.20426944	Cadm4
ENSMUSG00000054808.16	2952.17457	0.00296223	0.00310078	0.90799849	0.34064604	0.52841022	Actn4
ENSMUSG00000054814.15	289.703433	-0.0066928	0.003185	4.44221774	0.03506068	0.09039583	Usp46
ENSMUSG00000054823.18	1235.22104	-0.0187159	0.00285324	41.9936345	9.16E-11	1.48E-09	Nsd3
ENSMUSG00000054827.13	11263.8966	-0.0018759	0.00655851	0.07876632	0.77897627	0.90280253	Cyp2c50
ENSMUSG00000054836.13	69.9841067	-0.0150398	0.00358227	17.2585654	3.26E-05	0.00020779	Elp6
ENSMUSG00000054843.10	473.750612	-0.0101752	0.00278818	13.2708058	0.00026957	0.00138854	Atrnl1
ENSMUSG00000054850.5	2.01147087	0.03597882	0.01083307	10.9722165	0.00092488	0.00409734	Smim10l2a
ENSMUSG00000054855.14	165.944434	-0.0082216	0.00455551	3.18830068	0.07416707	0.16582589	Rnd1

ENSMUSG00000054863.10	24.0563214	0.02795331	0.00628509	17.4140544	3.01E-05	0.00019318	Tafa5
ENSMUSG00000054871.6	18.0014425	0.00493841	0.00685339	0.52694888	0.46789244	0.65896645	Tmem158
ENSMUSG00000054874.13	535.860395	-0.0053033	0.00366708	2.04485181	0.15272185	0.29133757	Pcnx3
ENSMUSG00000054889.11	1378.18574	0.00367982	0.00483962	0.56602077	0.4518445	0.64362873	Dsp
ENSMUSG00000054890.3	0.91493609	0.01103681	0.02966998	0.37491237	0.54033871	0.72408108	Or2b7
ENSMUSG00000054892.15	8.51984831	0.05171671	0.00711031	50.4196263	1.24E-12	2.56E-11	Txk
ENSMUSG00000054893.16	56.2751339	0.0028653	0.00334216	0.71870739	0.39656824	0.58841586	Zfp667
ENSMUSG00000054894.7	107.739584	-0.0010031	0.00352459	0.08064924	0.7764195	0.90104078	Dmac2l
ENSMUSG00000054920.13	510.663987	0.01019615	0.00216721	21.8058618	3.02E-06	2.39E-05	Klhl5
ENSMUSG00000054931.10	14.872309	0.02024571	0.00468128	18.5989862	1.61E-05	0.00011005	Zkscan4
ENSMUSG00000054932.7	28.3987688	-0.001866	0.00602771	0.09417892	0.75893027	0.88949162	Afp
ENSMUSG00000054934.11	2.33739258	0.03768819	0.01669102	5.02407271	0.02499731	0.06842926	Kcnmb4
ENSMUSG00000054939.7	48.6341448	-0.0080967	0.00343923	5.53818027	0.01860592	0.05361506	Zfp174
ENSMUSG00000054942.14	55.5243065	0.01361174	0.00462054	8.57302992	0.00341179	0.01275054	Miga1
ENSMUSG00000054951.4	7.34584235	0.03287707	0.00916398	12.1648774	0.00048698	0.00233881	9130008F23Rik
ENSMUSG00000054966.14	4.67075295	0.00818882	0.02484912	-0.0828921	1	1	Lmntd1
ENSMUSG00000054967.8	12.0138511	0.00366543	0.00477622	0.57860244	0.44686064	0.63868956	Zfp647
ENSMUSG00000054976.15	3.38767581	0.02979825	0.02011117	2.88450677	0.08943539	0.19258862	Nyap2
ENSMUSG00000054978.4	2.9152988	0.0346097	0.01160028	8.55551051	0.00344478	0.01285375	Kbtbd13
ENSMUSG00000054986.14	2.45486832	-0.0095143	0.03724812	0.06479008	0.79907921	0.91547812	Sec14l3
ENSMUSG00000054994.3	0.53653445	-0.0101165	0.07953319	0.01725137	0.89550281	0.97320224	Pramel3e
ENSMUSG00000055003.15	15.7129736	0.13594969	0.01735916	36.8743536	1.26E-09	1.72E-08	Lrtm2
ENSMUSG00000055013.17	216.926884	-0.0009219	0.00335345	0.07471277	0.78459466	0.9065966	Agap1
ENSMUSG00000055022.15	19.2872424	0.04616653	0.0143831	11.1901402	0.00082233	0.00369544	Cntn1
ENSMUSG00000055024.13	737.026357	0.00103322	0.00387265	0.07041158	0.79073846	0.91057306	Ep300
ENSMUSG00000055026.14	5.38036834	0.00762477	0.02146313	0.33484199	0.56282176	0.7436398	Gabrg3
ENSMUSG00000055027.18	125.448764	-0.0048455	0.00633972	0.55738045	0.45531763	0.64708502	Smyd1
ENSMUSG00000055041.9	246.203478	-0.0011387	0.0030706	0.13938936	0.70888883	0.85602354	Commd5
ENSMUSG00000055044.13	512.133652	0.00608192	0.00350673	2.96135091	0.08527667	0.18541071	Pdlim1
ENSMUSG00000055053.19	2281.14354	-0.0050104	0.00402995	1.49498651	0.22144438	0.38660157	Nfic
ENSMUSG00000055065.8	2783.88203	-0.0012888	0.00250477	0.26121559	0.60928625	0.78164896	Ddx17
ENSMUSG00000055067.16	70.5037569	0.00708156	0.0043403	2.65531121	0.10320456	0.21518588	Smyd3
ENSMUSG00000055069.4	22.5210579	0.04620025	0.00683766	41.2392533	1.35E-10	2.12E-09	Rab39
ENSMUSG00000055078.8	2.40833356	0.03840893	0.02699316	2.41222584	0.12039108	0.24284135	Gabra5
ENSMUSG00000055088.6	3.94245044	-0.00416	0.02531412	0.00170297	0.96708304	1	Or1n2
ENSMUSG00000055114.11	22.6459338	0.03315711	0.0066791	24.5318988	7.31E-07	6.43E-06	Anxa13
ENSMUSG00000055116.9	263.721356	-0.010681	0.0080296	1.61350498	0.20399981	0.363794	Bmal1
ENSMUSG00000055128.16	584.898887	-0.0108717	0.0022938	22.6096072	1.98E-06	1.63E-05	Cgrrf1
ENSMUSG00000055137.8	1061.08266	-0.0088339	0.00408842	4.51800472	0.03353992	0.08718976	Sugct
ENSMUSG00000055148.8	158.022784	0.01119474	0.00480694	5.29805826	0.02134921	0.0600671	Klf2
ENSMUSG00000055150.16	22.7497818	-0.0082034	0.00502225	2.64533771	0.10385409	0.21617708	Zfp78
ENSMUSG00000055170.5	1.96259408	0.10491983	0.01441716	45.286506	1.70E-11	3.05E-10	Ifng
ENSMUSG00000055172.11	5936.37252	-0.0044719	0.0033091	1.84006225	0.17494365	0.32329554	C1ra
ENSMUSG00000055184.5	19.0569	0.01127218	0.00467328	5.83459335	0.01571404	0.04662616	Fam72a
ENSMUSG00000055197.5	2.88606755	-0.0115634	0.01539603	0.3629793	0.54685625	0.72969854	Fev
ENSMUSG00000055200.8	107.522222	0.00072299	0.00396557	0.03297956	0.8558945	0.95083263	Sertad3
ENSMUSG00000055202.12	8.04691362	0.05937828	0.01066917	26.4426942	2.71E-07	2.60E-06	Zfp811
ENSMUSG00000055204.17	5485.00336	-0.0006233	0.00463096	0.0178484	0.89372055	0.97247153	Ankrd17
ENSMUSG00000055210.5	2.76953851	0.00915008	0.01174587	0.63047236	0.4271821	0.61865276	Foxd2
ENSMUSG00000055214.16	2.1365812	0.01499015	0.02051467	0.90252821	0.34210471	0.53001062	Pld5
ENSMUSG00000055228.9	2.72946849	0.00051871	0.06676942	-9.44E-06	1	1	Gm49359
ENSMUSG00000055235.12	11.4433096	0.02844496	0.0069408	16.1748073	5.78E-05	0.00034948	Wdr86
ENSMUSG00000055239.11	1342.11849	-0.0108096	0.0021877	23.9929122	9.67E-07	8.34E-06	Kcmf1
ENSMUSG00000055240.18	136.099772	-0.0110654	0.00412272	7.06003578	0.00788224	0.02606498	Zfp101
ENSMUSG00000055254.16	252.277929	0.15681495	0.00850489	264.948677	1.43E-59	8.44E-56	Ntrk2
ENSMUSG00000055296.15	785.91389	-0.0024279	0.00341658	0.49709249	0.48078045	0.6705035	Tmem245
ENSMUSG00000055298.7	0.77803639	-0.0104958	0.02598768	0.15346943	0.69524165	0.84647788	Ctsj
ENSMUSG00000055301.9	293.803117	-0.0160059	0.00584073	7.45328056	0.00633209	0.02164475	Adh7
ENSMUSG00000055302.6	2672.77784	0.00035542	0.00205751	0.02964637	0.86329508	0.95486666	Mrfap1
ENSMUSG00000055305.16	85.329452	-0.0101859	0.00495611	4.07974659	0.04340017	0.10754742	Zfp93
ENSMUSG00000055312.3	331.081726	0.00745939	0.00429481	2.84889423	0.09143681	0.19595026	Them7

ENSMUSG00000055313.16	14.4662088	0.00590922	0.00471485	1.59997394	0.2059069	0.36644686	Pgbd1
ENSMUSG00000055319.9	458.28679	-0.0053412	0.0020777	6.56710972	0.01038805	0.03291853	Sec23ip
ENSMUSG00000055320.19	275.505133	0.00727918	0.00487317	2.23722058	0.13472295	0.26490834	Tead1
ENSMUSG00000055322.17	2194.48298	-0.0058037	0.00375972	2.34563831	0.12563409	0.25107657	Tns1
ENSMUSG00000055334.5	108.762763	0.00131786	0.00278676	0.22296557	0.63678954	0.80373039	Snupn
ENSMUSG00000055341.11	4.47325425	-0.0131706	0.00810482	2.52705887	0.11190863	0.22924066	Zfp457
ENSMUSG00000055371.18	325.876037	-0.0021144	0.00278934	0.57055409	0.45003882	0.64152155	Stam2
ENSMUSG00000055373.9	42.5499439	0.01882287	0.0158463	1.51326948	0.21864087	0.38302824	Fut9
ENSMUSG00000055401.15	602.811218	0.00029089	0.00295409	0.00961193	0.92190017	0.98764251	Fbxo6
ENSMUSG00000055407.15	11.363093	0.04641697	0.01485408	9.18181686	0.00244431	0.00958782	Map6
ENSMUSG00000055409.15	4.32436154	0.0090534	0.02058035	0.24078694	0.62363832	0.79291214	Nell1
ENSMUSG00000055413.13	261.990913	0.01863214	0.00994525	3.00121039	0.08320234	0.18170466	H2-Q5
ENSMUSG00000055415.9	1.50540339	0.02568485	0.01536561	3.09071346	0.07874028	0.1739573	Atp10b
ENSMUSG00000055421.9	13.5329495	0.04087443	0.00992983	14.8576912	0.00011593	0.00065308	Pcdh9
ENSMUSG00000055430.5	22.0660523	0.05703507	0.00882583	38.9248262	4.40E-10	6.43E-09	Nap1l5
ENSMUSG00000055435.7	968.661512	0.0169988	0.00375632	19.4483876	1.03E-05	7.32E-05	Maf
ENSMUSG00000055436.19	4045.87683	0.00123242	0.00401217	0.09410336	0.75902399	0.88955726	Srsf11
ENSMUSG00000055447.20	1977.6331	0.00936313	0.00261991	12.6851823	0.00036856	0.00182434	Cd47
ENSMUSG00000055471.8	2.66939309	0.02677071	0.02234649	2.78431821	0.0951912	0.20225021	Alk
ENSMUSG00000055480.8	34.96078	0.00664325	0.00398503	2.73714546	0.09803976	0.20696014	Zfp458
ENSMUSG00000055485.7	55.0268655	0.01569127	0.00525698	8.57120935	0.0034152	0.01275844	Soga1
ENSMUSG00000055489.9	6.70300931	0.01287504	0.00971961	1.78231685	0.18186509	0.33298168	Ano5
ENSMUSG00000055491.15	255.860542	-0.0014079	0.00520138	0.07242019	0.78784504	0.90888301	Pprc1
ENSMUSG00000055493.5	93.8154787	0.0021433	0.00418944	0.25586687	0.612974	0.78493124	Epm2a
ENSMUSG00000055523.8	1.2679933	0.05599611	0.0139135	15.5435215	8.06E-05	0.00047095	Gucy2g
ENSMUSG00000055531.13	671.317381	-0.001539	0.00250423	0.37872422	0.53828681	0.72231122	Cpsf6
ENSMUSG00000055538.8	1911.48861	-0.0025868	0.00275738	0.88111943	0.34789526	0.5360443	Zcchc24
ENSMUSG00000055540.16	1.40635018	0.02532169	0.04059486	1.26884995	0.25998254	0.43387449	Epha6
ENSMUSG00000055541.20	162.213902	0.0673976	0.00627708	98.1507472	3.88E-23	2.89E-21	Lair1
ENSMUSG00000055546.7	199.18763	0.03428622	0.00661249	24.2716627	8.37E-07	7.29E-06	Timd4
ENSMUSG00000055547.5	1.5040295	-0.0063768	0.05780493	-0.0437313	1	1	Apobec4
ENSMUSG00000055553.17	626.193433	-0.0020661	0.00219317	0.89999785	0.34278229	0.53078145	Kxd1
ENSMUSG00000055555.3	1.93039218	0.12981124	0.03416242	0.95174119	0.32927649	0.51620232	Ct55
ENSMUSG00000055560.9	3.45978733	0.0357746	0.01138996	8.90851806	0.00283844	0.01089992	Zfp459
ENSMUSG00000055561.10	15.2061358	0.01596555	0.00788229	3.78034065	0.0518581	0.12418068	Spink5
ENSMUSG00000055567.19	39.7678759	0.00408794	0.0157289	0.05485691	0.81481766	0.92431172	Unc80
ENSMUSG00000055602.18	5.68723995	-0.0157301	0.00885284	3.07664297	0.07942431	0.1751404	Tcp10b
ENSMUSG00000055610.6	0.81362279	-0.0140052	0.03415099	0.2592412	0.61064194	0.78292025	Or14a260
ENSMUSG00000055612.16	38.5044578	-0.0085796	0.00521141	2.57819385	0.10834522	0.22350964	Cdca7
ENSMUSG00000055629.6	5.14409405	0.03787861	0.0123171	7.62612095	0.00575289	0.0199482	B4galnt4
ENSMUSG00000055632.19	18.8588503	0.00911605	0.0073307	1.49368387	0.22164577	0.38677394	Hmcn2
ENSMUSG00000055633.7	10.8950664	0.0160323	0.00603188	7.23384804	0.00715417	0.02405735	Zfp580
ENSMUSG00000055639.17	16.033678	0.02207063	0.00819027	7.06391941	0.00786517	0.02601795	Dach1
ENSMUSG00000055652.15	293.17052	-0.0018661	0.003599	0.27038475	0.6030738	0.77633984	Klhl25
ENSMUSG00000055653.14	46.5733607	0.01525038	0.00600643	6.25174821	0.01240708	0.03818285	Gpc3
ENSMUSG00000055660.10	315.6342	0.00421599	0.00283536	2.16128754	0.14152606	0.27505221	Mettl4
ENSMUSG00000055670.15	729.111514	-0.0033042	0.0024683	1.8007481	0.17962208	0.32997635	Zzef1
ENSMUSG00000055675.7	60.5419889	0.06411116	0.01059099	28.8630188	7.77E-08	8.21E-07	Kbtbd11
ENSMUSG00000055681.15	1608.19921	-0.0077419	0.00269753	8.35900299	0.00383781	0.01409771	Cope
ENSMUSG00000055691.6	2.57659143	-0.0113417	0.00935617	1.46115066	0.22674713	0.39303724	Gja6
ENSMUSG00000055692.22	22.2904694	0.01628457	0.00628396	6.78355186	0.00920016	0.02977021	Tmem191
ENSMUSG00000055707.14	98.8865382	0.01078923	0.00298182	13.0785789	0.00029869	0.00151864	Klhl26
ENSMUSG00000055717.14	10.3628332	0.06428924	0.00866017	48.6817025	3.01E-12	5.98E-11	Slain1
ENSMUSG00000055720.11	474.695453	-0.0070312	0.00231266	9.30330343	0.00228741	0.00906715	Ubl7
ENSMUSG00000055723.7	322.869243	-0.0016274	0.00238742	0.46382819	0.49584028	0.68308417	Rras2
ENSMUSG00000055725.12	101.962286	-0.0038306	0.00362871	1.0870631	0.29712285	0.48052246	Paqr3
ENSMUSG00000055730.17	5601.14387	-0.0237761	0.00541572	19.3039117	1.11E-05	7.82E-05	Ces2a
ENSMUSG00000055733.7	31.4222983	0.02837164	0.00448632	39.2530987	3.72E-10	5.48E-09	Nap1l3
ENSMUSG00000055737.13	9977.94047	-0.0134746	0.00293449	20.9647251	4.68E-06	3.56E-05	Ghr
ENSMUSG00000055745.5	9.78578091	0.0413092	0.00655539	38.1203912	6.65E-10	9.48E-09	Rtl6
ENSMUSG00000055748.13	1.20925625	0.01452852	0.0211472	0.64124509	0.42326029	0.61498289	Gsdmc4

ENSMUSG00000055760.13	79.5834637	-0.0082099	0.00298204	7.61124537	0.00580054	0.0200749	Gemin6
ENSMUSG00000055761.15	1.34022192	0.03428641	0.02363515	3.44356257	0.06349863	0.14641497	Nkain3
ENSMUSG00000055762.18	1456.67328	-0.0059417	0.00272706	4.79458004	0.02854942	0.07635388	Eef1d
ENSMUSG00000055775.17	2.24848378	-0.0168141	0.02559187	0.54121468	0.46192976	0.65316751	Myh8
ENSMUSG00000055782.10	741.469985	0.06462514	0.00562137	127.576231	1.39E-29	2.62E-27	Abcd2
ENSMUSG00000055799.14	150.469087	-0.008906	0.00483674	3.35772857	0.06689027	0.1526535	Tcf7l1
ENSMUSG00000055805.16	67.855603	0.0589675	0.00662115	70.5500129	4.49E-17	1.64E-15	Fmnl1
ENSMUSG00000055809.8	8.50725923	0.05986458	0.0084558	45.086477	1.89E-11	3.36E-10	Dnaaf3
ENSMUSG00000055817.19	220.249409	0.01134587	0.00266619	18.3197029	1.87E-05	0.00012575	Mta3
ENSMUSG00000055835.11	100.176337	0.00291598	0.00294544	0.96543649	0.32582095	0.51259424	Zfp1
ENSMUSG00000055839.7	1113.70002	-0.0097077	0.00338902	8.25323436	0.00406795	0.01483863	Elob
ENSMUSG00000055850.13	1367.52285	-0.0031771	0.00187135	2.90967934	0.08804938	0.1901944	Rnf181
ENSMUSG00000055862.8	153.658719	0.00787098	0.00481182	2.67704708	0.10180435	0.213152	Izumo4
ENSMUSG00000055865.9	6.34530805	0.02186814	0.01062954	3.63182095	0.05668476	0.13375573	Tafa3
ENSMUSG00000055866.10	427.099715	0.00934909	0.00630983	2.12870416	0.14456292	0.27970969	Per2
ENSMUSG00000055884.9	118.968234	0.00187199	0.00587818	0.10486283	0.74607029	0.88036892	Fancm
ENSMUSG00000055900.16	162.818305	-0.0091022	0.00355914	6.43835411	0.01116821	0.03500975	Tmem69
ENSMUSG00000055912.9	1426.16851	-0.0133433	0.00302918	19.8134754	8.54E-06	6.13E-05	Tmem150a
ENSMUSG00000055917.16	305.54907	0.00038982	0.00231789	0.02827691	0.86645946	0.95662186	Zfp277
ENSMUSG00000055923.17	171.923758	-0.0136794	0.00379154	13.0461182	0.00030391	0.00154252	Aasdh
ENSMUSG00000055926.4	2.63594887	0.05817699	0.01025184	30.502088	3.34E-08	3.71E-07	Gm14137
ENSMUSG00000055932.16	679.042004	0.00687512	0.00194856	12.5065179	0.00040553	0.0019869	Fto
ENSMUSG00000055943.6	1377.81878	-0.0065757	0.00181061	13.2223461	0.00027663	0.00142087	Emc7
ENSMUSG00000055945.9	8.79639972	0.01239339	0.0104276	1.37182834	0.241498	0.41149919	Prr18
ENSMUSG00000055963.13	13.6596726	0.01097405	0.00972896	1.16503299	0.28042418	0.45988887	Triqk
ENSMUSG00000055976.7	2.75371948	0.00162852	0.01200044	0.01103999	0.91631918	0.98421167	Cldn23
ENSMUSG00000055978.6	4.26506729	0.00424426	0.01134061	0.13975033	0.70852935	0.85578915	Fut2
ENSMUSG00000055980.3	533.854233	0.0002203	0.00341621	0.00412978	0.94876049	1	Irs1
ENSMUSG00000055991.16	250.344478	-0.0004605	0.00246684	0.03412484	0.85344152	0.94949395	Zkscan5
ENSMUSG00000055994.16	6.92430167	0.04756712	0.00794536	33.953559	5.64E-09	7.08E-08	Nod2
ENSMUSG00000056004.17	15.4728757	0.01945103	0.00646604	8.60528801	0.00335188	0.01257276	Elapor2
ENSMUSG00000056014.17	92.4459818	-0.0003639	0.00306075	0.01384701	0.90632651	0.97905006	A430033K04Rik
ENSMUSG00000056018.15	3.11803081	-0.0594935	0.11354942	-0.1576281	1	1	Ccdc7b
ENSMUSG00000056019.13	110.63956	0.01130928	0.00405207	7.40596587	0.00650079	0.02216359	Zfp709
ENSMUSG00000056025.13	153.987091	0.04920164	0.00487607	98.3162054	3.57E-23	2.67E-21	Clca3a1
ENSMUSG00000056035.9	82621.0222	-0.0055541	0.0068721	0.63953776	0.42387823	0.61550157	Cyp3a11
ENSMUSG00000056043.6	2.37916872	0.01796656	0.0101767	3.12543325	0.07707938	0.17111281	Rgs9bp
ENSMUSG00000056050.16	3778.69837	0.00044509	0.00278421	0.0252859	0.87365673	0.96079021	Mia3
ENSMUSG00000056054.10	116.117258	0.09106504	0.01227295	33.4412792	7.34E-09	9.07E-08	S100a8
ENSMUSG00000056055.15	3.05107261	0.0400316	0.01191441	10.2471159	0.00136898	0.00577722	Sag
ENSMUSG00000056069.11	102.170205	0.06105239	0.00626252	86.5759365	1.34E-20	7.43E-19	Otulinl
ENSMUSG00000056071.13	178.882648	0.08595653	0.01189363	31.4778152	2.02E-08	2.33E-07	S100a9
ENSMUSG00000056073.17	6.30851752	0.01780767	0.01744924	1.12996134	0.28778359	0.46879368	Grik2
ENSMUSG00000056076.14	1668.58288	-0.0035575	0.00268512	1.75094654	0.18575778	0.33855512	Eif3b
ENSMUSG00000056091.13	2752.49873	-0.0054108	0.00473946	1.32759647	0.24923326	0.42074534	St3gal5
ENSMUSG00000056116.19	1929.73793	0.01791051	0.00292151	38.0724723	6.82E-10	9.69E-09	H2-T22
ENSMUSG00000056121.17	583.14686	0.00425409	0.00206275	4.17407065	0.04104707	0.10278917	Fez2
ENSMUSG00000056124.6	85.7937605	0.04471257	0.00510734	71.951123	2.21E-17	8.39E-16	B4galt6
ENSMUSG00000056130.11	17.6798389	0.05753304	0.00690604	61.8562333	3.69E-15	1.07E-13	Ticam2
ENSMUSG00000056131.14	324.694093	0.00136529	0.00317816	0.18023843	0.67116842	0.82902217	Pgm3
ENSMUSG00000056133.6	74.5429394	0.0053146	0.00548114	0.9494732	0.32985343	0.51666376	Unc93a2
ENSMUSG00000056144.15	169.640516	0.0112961	0.00341224	10.8157058	0.00100643	0.00442041	Trim34a
ENSMUSG00000056148.9	1311.03362	0.02127179	0.00508871	17.4122641	3.01E-05	0.00019326	Rdh9
ENSMUSG00000056153.16	287.981352	0.00597273	0.00278512	4.53167116	0.03327311	0.08657249	Socs6
ENSMUSG00000056158.15	4.4386585	0.01538625	0.01826753	0.57762896	0.4472432	0.639004	Car10
ENSMUSG00000056162.15	8.26971561	0.00890146	0.01585297	0.43062303	0.51168339	0.6984123	Cndp1
ENSMUSG00000056167.9	381.576668	0.00216928	0.00154795	1.97713443	0.15969261	0.30115216	Cnot10
ENSMUSG00000056174.9	6.79108888	0.00280835	0.00848745	0.10953639	0.74067256	0.87726802	Col8a2
ENSMUSG00000056185.6	64.9566356	-0.0002048	0.00326571	0.00397154	0.94975048	1	Snx32
ENSMUSG00000056201.10	3097.2485	0.00832951	0.00240076	11.9296197	0.00055248	0.00261088	Cfl1
ENSMUSG00000056204.9	1034.60509	-0.0029883	0.0030838	0.93680012	0.33310213	0.51999119	Pgpep1

ENSMUSG00000056209.8	278.626604	-0.011893	0.00342843	12.33834	0.00044375	0.00214781	Npm3
ENSMUSG00000056211.14	492.478341	-0.0059712	0.00276346	4.64824659	0.03108528	0.08188268	R3hdm1
ENSMUSG00000056214.10	20.4105466	0.0168476	0.00449347	13.8876913	0.00019407	0.00103792	Pard6g
ENSMUSG00000056215.15	3.35493673	0.05008372	0.0137947	12.2548864	0.00046404	0.00223867	Lrguk
ENSMUSG00000056216.10	1284.12162	-0.0050432	0.00261227	3.61338614	0.05731631	0.134885	Cebpg
ENSMUSG00000056220.15	61.0929079	0.05227134	0.00615178	65.7951835	5.00E-16	1.59E-14	Pla2g4a
ENSMUSG00000056222.16	14.939754	0.01444511	0.01902019	0.70945997	0.39962315	0.5914232	Spock1
ENSMUSG00000056223.9	1.75432618	-0.0258655	0.05919855	0.28793608	0.59154618	0.76749042	Spata31
ENSMUSG00000056228.11	250.027403	-0.0063001	0.00329422	3.62781476	0.05682138	0.1340055	Cars2
ENSMUSG00000056234.16	3423.64041	0.00342903	0.0027238	1.56745302	0.21057728	0.37255571	Ncoa4
ENSMUSG00000056258.10	14.1536015	0.04151219	0.01473538	8.43200214	0.00368674	0.01361768	Kcnq3
ENSMUSG00000056260.16	301.135503	0.01186896	0.00384331	9.11021216	0.00254186	0.00991604	Lrif1
ENSMUSG00000056267.15	73.4503528	0.01001118	0.0036514	7.37112022	0.00662796	0.02255803	Cep70
ENSMUSG00000056268.16	427.546788	0.00172449	0.00294677	0.32997339	0.56567476	0.74586342	Dennd1b
ENSMUSG00000056290.17	53.5605531	0.08148534	0.00839463	78.6822334	7.29E-19	3.29E-17	Ms4a4b
ENSMUSG00000056293.13	0.90499671	-0.0014178	0.03214543	0.00755447	0.93073794	0.99206576	Gsdmc2
ENSMUSG00000056296.18	14.7995998	0.02176598	0.01928696	1.60767942	0.20481831	0.36495011	Synpr
ENSMUSG00000056300.15	2.40973633	0.00898611	0.01455592	0.46951546	0.49321016	0.68072892	Zfp981
ENSMUSG00000056305.5	348.437873	-0.0071498	0.00181576	15.6157359	7.76E-05	0.00045487	Usp39
ENSMUSG00000056306.7	2.57299004	0.02763366	0.02940887	1.41349158	0.23447764	0.40284548	Sertm1
ENSMUSG00000056310.15	299.268635	-0.0014004	0.00187164	0.55251895	0.45729027	0.64899696	Tyw1
ENSMUSG00000056313.6	503.655642	-0.0171886	0.00481918	13.1116356	0.00029347	0.00149594	Tcim
ENSMUSG00000056328.15	4.57923174	-0.0022411	0.02570311	-2.5848751	1	1	Myh1
ENSMUSG00000056342.17	1174.07285	-0.0040701	0.00361838	1.23016924	0.26737413	0.44326184	Usp34
ENSMUSG00000056367.15	16.3834733	0.02961741	0.00731205	15.6406362	7.66E-05	0.00044982	Actr3b
ENSMUSG00000056370.18	1.0706295	0.00437535	0.03551017	0.09980118	0.75206836	0.88496729	Sftpb
ENSMUSG00000056383.11	440.399401	0.00190016	0.00320305	0.34519077	0.55684823	0.73894192	AI987944
ENSMUSG00000056394.21	98.0798695	0.04106672	0.00606726	44.9136185	2.06E-11	3.64E-10	Lig1
ENSMUSG00000056399.4	0.67689512	0.05305183	0.02955172	3.10269096	0.078163	0.17300604	Prss34
ENSMUSG00000056413.17	34.2669383	0.05678272	0.00621275	75.6324111	3.42E-18	1.41E-16	Adap1
ENSMUSG00000056427.11	8.85314213	0.00775935	0.01027461	0.5844771	0.44456277	0.63683313	Slit3
ENSMUSG00000056429.5	4076.96674	-0.0062855	0.00266326	5.34319682	0.02080329	0.05886113	Tgoln1
ENSMUSG00000056458.17	3.64639441	0.01969268	0.01120559	2.96672929	0.08499354	0.18494857	Mok
ENSMUSG00000056459.15	38.5538609	0.0184897	0.00371839	24.0551276	9.36E-07	8.08E-06	Zbtb25
ENSMUSG00000056476.14	58.0373437	-0.0391398	0.00533711	53.3227275	2.83E-13	6.42E-12	Med12l
ENSMUSG00000056481.8	18.5191278	-0.0044484	0.00683674	0.44545332	0.50450204	0.69129061	Cd248
ENSMUSG00000056486.19	30.3593924	-0.004003	0.01042398	0.12905126	0.71941776	0.86268683	Chn1
ENSMUSG00000056487.9	6.61045573	0.02615159	0.0134893	3.37869143	0.06604447	0.15116205	Tmt1a2
ENSMUSG00000056492.7	808.150094	0.0002795	0.0035422	0.00605948	0.93795319	0.99588386	Adgrf5
ENSMUSG00000056493.10	425.211914	-0.0005055	0.00322485	0.02441165	0.87584205	0.96175849	Foxk1
ENSMUSG00000056494.8	2.22622644	-0.0205551	0.02182464	0.83151846	0.36183382	0.5514482	Cngb3
ENSMUSG00000056498.14	14.5518342	0.08050948	0.00892312	61.8877951	3.64E-15	1.05E-13	Tmem154
ENSMUSG00000056501.4	1422.97803	-0.0112776	0.00543002	4.203084	0.04035053	0.10136412	Cebpb
ENSMUSG00000056515.10	175.147544	0.03147361	0.00497748	38.0951244	6.74E-10	9.59E-09	Rab31
ENSMUSG00000056529.8	36.2227111	0.0545245	0.00519924	101.995856	5.56E-24	4.62E-22	Ptafr
ENSMUSG00000056531.12	13.4499666	-0.0035148	0.00864756	0.1455403	0.70283417	0.85165054	Ccdc18
ENSMUSG00000056536.15	255.968212	0.01306326	0.00402881	10.2453236	0.00137032	0.00578077	Pign
ENSMUSG00000056537.12	784.989925	0.00333969	0.00258275	1.62553259	0.20232207	0.36140324	Rlim
ENSMUSG00000056553.15	3.67585906	0.02828089	0.01448226	4.1822516	0.0408494	0.1023884	Ptprn2
ENSMUSG00000056564.8	30.9962435	0.01579652	0.03024668	0.830655	0.3620832	0.5516722	Or2ab1
ENSMUSG00000056569.12	5.34013237	0.00376627	0.00924264	0.16897846	0.68102128	0.83624365	Mpz
ENSMUSG00000056579.19	877.581193	0.00484571	0.00283563	2.88498175	0.08940902	0.19254941	Tug1
ENSMUSG00000056592.15	25.5715278	0.01702139	0.00448805	14.2053387	0.0001639	0.00089281	Zfp658
ENSMUSG00000056596.9	3.15255825	0.03933588	0.01213097	10.0380383	0.0015334	0.00639556	Trnp1
ENSMUSG00000056598.18	7.63910213	0.0001015	0.0060007	0.00028643	0.98649707	1	Drc3
ENSMUSG00000056602.14	55.5719192	0.02375192	0.00534862	19.3491547	1.09E-05	7.66E-05	Fry
ENSMUSG00000056608.15	2177.49676	0.00819865	0.00687908	1.24211198	0.26506441	0.44023725	Chd9
ENSMUSG00000056612.8	231.576237	0.00483193	0.00386254	1.59533583	0.20656545	0.36723092	Ppp1r14b
ENSMUSG00000056629.17	1013.72032	-0.002923	0.00367804	0.63456848	0.4256845	0.61728833	Fkbp2
ENSMUSG00000056643.6	105.216312	0.00178849	0.00434037	0.16727316	0.68254669	0.83759346	Chst13
ENSMUSG00000056648.7	7.05374765	0.03538612	0.01468142	5.94499375	0.01475914	0.04429433	Hoxb8

ENSMUSG00000056656.7	1.60914833	0.04996023	0.02474656	2.68991971	0.1009849	0.21174598	Apol8
ENSMUSG00000056665.3	48.5777979	0.01448767	0.00407243	12.6993457	0.00036578	0.00181209	Them6
ENSMUSG00000056666.14	5357.34812	-0.0231755	0.00440151	27.0025021	2.03E-07	2.00E-06	Retsat
ENSMUSG00000056671.8	28.1041702	0.07843363	0.00716887	105.220791	1.09E-24	9.95E-23	Prelid2
ENSMUSG00000056673.15	290.255911	-0.001196	0.00319516	0.13788385	0.71039388	0.85686965	Kdm5d
ENSMUSG00000056679.14	2.63601746	0.01454953	0.01411583	0.8789195	0.34849779	0.53647126	Gpr173
ENSMUSG00000056692.14	4251.40128	-0.0032882	0.00217598	2.28871805	0.13031752	0.25837836	Ilrun
ENSMUSG00000056698.12	318.029497	-0.0122623	0.00316677	14.7399531	0.0001234	0.00069086	Elmod3
ENSMUSG00000056708.6	142.279602	0.03167041	0.00407439	56.317633	6.17E-14	1.51E-12	Ier5
ENSMUSG00000056718.3	2.64422757	-0.0001824	0.00841993	0.00029806	0.98622566	1	Gm13199
ENSMUSG00000056724.16	168.415561	0.00106346	0.00476238	0.04947802	0.82397406	0.93004653	Nbeal2
ENSMUSG00000056737.15	120.437579	0.08733546	0.0068575	143.199109	5.32E-33	1.65E-30	Capg
ENSMUSG00000056749.8	500.135858	-0.0241946	0.00538948	18.8862623	1.39E-05	9.59E-05	Nfil3
ENSMUSG00000056755.14	4.75415069	0.0119805	0.02067122	0.53133888	0.46604451	0.65718796	Grm7
ENSMUSG00000056758.15	4.86378603	-0.0067433	0.01663987	0.18335596	0.66850491	0.82707494	Hmga2
ENSMUSG00000056763.17	447.536542	-0.0106365	0.00413076	6.69975154	0.00964263	0.03095144	Cspp1
ENSMUSG00000056770.16	1540.91222	0.00195814	0.00169544	1.33971976	0.2470834	0.41844683	Setd3
ENSMUSG00000056812.15	32.6443494	0.00895689	0.00895436	0.95011339	0.32969044	0.5164969	St8sia3
ENSMUSG00000056820.8	779.976311	0.00489739	0.00188334	6.73235146	0.00946797	0.03049038	Tsnax
ENSMUSG00000056822.5	1.49721135	-0.0057525	0.02443408	0.01785255	0.89370826	0.97247153	Or2l13
ENSMUSG00000056832.15	11.8788147	0.01700391	0.0064375	6.63465887	0.01000133	0.03189878	Ift56
ENSMUSG00000056851.15	2738.89052	0.00281491	0.00275064	1.03919279	0.30800928	0.49296549	Pcbp2
ENSMUSG00000056856.15	2.83643215	0.03743611	0.01740245	4.83223035	0.02793247	0.07501028	Jakmip3
ENSMUSG00000056863.6	0.609524	0.06589165	0.0344917	5.50702087	0.01894028	0.05440573	Or13n4
ENSMUSG00000056870.10	26.5485757	0.00055814	0.00743504	0.00426417	0.94793463	1	Gulp1
ENSMUSG00000056880.15	3.83576837	-0.0044016	0.01136049	0.08938309	0.7649629	0.89336576	Gadl1
ENSMUSG00000056888.12	29.4837287	0.07701452	0.00799691	81.9530747	1.39E-19	6.93E-18	Glipr1
ENSMUSG00000056895.5	1.33714597	0.04026874	0.0142324	7.45746347	0.0063174	0.02159764	H2bc27
ENSMUSG00000056899.11	556.703974	-0.008156	0.00416838	3.81612951	0.05076137	0.1220944	Immp2l
ENSMUSG00000056900.14	70.1178441	-0.0129684	0.01424909	0.90908091	0.3403584	0.52813771	Usp13
ENSMUSG00000056910.6	1.09811948	-0.0082823	0.03321015	0.02908047	0.86459338	0.95561795	Vmn2r107
ENSMUSG00000056912.13	0.92296916	-0.0114453	0.20950063	-0.0004345	1	1	1700017N19Rik
ENSMUSG00000056917.14	252.244298	0.01436612	0.00610936	5.43346118	0.01975449	0.05629886	Sipa1
ENSMUSG00000056919.10	103.589612	0.0024298	0.00510355	0.22758198	0.63332258	0.80119858	Cep162
ENSMUSG00000056921.3	1.00805689	-0.0055492	0.03385797	0.00737762	0.9315514	0.99255044	Or1e34
ENSMUSG00000056938.18	788.01876	-0.0070487	0.0030057	5.47909531	0.01924522	0.05512054	Acbd4
ENSMUSG00000056941.18	454.240061	-0.0019103	0.00271614	0.4970408	0.48080326	0.6705035	Commd7
ENSMUSG00000056952.14	428.990698	0.00212287	0.00274077	0.59303794	0.44124676	0.63308268	Tatdn2
ENSMUSG00000056962.12	306.307352	-0.0053524	0.00262979	4.16250887	0.04132814	0.10334665	Jmjd6
ENSMUSG00000056966.8	34.7038725	-0.0085714	0.00670281	1.56176566	0.21140688	0.37358528	Gjc3
ENSMUSG00000056973.7	12683.3493	-0.0102128	0.00483518	4.45151064	0.03487039	0.09001346	Ces1d
ENSMUSG00000056978.9	1858.03414	-0.0274873	0.00961751	8.86358124	0.00290916	0.01112624	Hamp2
ENSMUSG00000056987.9	4.49196511	-0.0046891	0.01584325	0.08329551	0.77288014	0.89851888	Garin2
ENSMUSG00000056995.4	1.89723458	0.00987122	0.02148514	0.47165662	0.49222603	0.67996036	Or4p7
ENSMUSG00000056999.17	2143.80289	-0.0047077	0.00272645	2.9520252	0.08577001	0.18627731	Ide
ENSMUSG00000057003.13	5.86956979	0.00718121	0.02796291	-0.9687946	1	1	Myh4
ENSMUSG00000057037.9	3817.61909	-0.0050606	0.00474528	1.12514236	0.28881386	0.4700175	Cfhr1
ENSMUSG00000057054.12	217.000313	-0.0105545	0.00389554	7.50012671	0.00616947	0.02117169	Inca1
ENSMUSG00000057058.17	10.3523454	0.0662674	0.00748169	69.6636353	7.03E-17	2.49E-15	Skap1
ENSMUSG00000057060.9	10.4992137	0.03638006	0.02307322	3.52940546	0.0602894	0.14049855	Slc35f3
ENSMUSG00000057068.16	396.073166	0.01094422	0.00558029	3.59379559	0.05799564	0.13624354	Fam47e
ENSMUSG00000057069.8	1249.70646	-0.0108871	0.00396141	7.25615066	0.00706586	0.0238011	Ero1b
ENSMUSG00000057074.7	3761.75049	0.01663016	0.0048983	11.5116958	0.0006916	0.00317973	Ces1g
ENSMUSG00000057092.13	15.2500167	0.01889471	0.00899144	4.31922874	0.037684	0.09575682	Fxyd3
ENSMUSG00000057093.15	27.3445622	0.01733295	0.00838215	4.17400936	0.04104855	0.10278917	Zfp607b
ENSMUSG00000057098.15	198.864245	0.0508689	0.00883155	27.5834275	1.50E-07	1.52E-06	Ebf1
ENSMUSG00000057101.9	303.52887	-0.013363	0.00293275	20.4707218	6.06E-06	4.51E-05	Zfp180
ENSMUSG00000057103.12	3462.85421	-0.0221394	0.00460194	23.1578005	1.49E-06	1.25E-05	Nat8f1
ENSMUSG00000057110.16	425.005788	-0.0098246	0.00405649	5.67320702	0.01722594	0.0503209	Cntrl
ENSMUSG00000057113.14	2961.61734	0.00394021	0.00252878	2.3901591	0.1221012	0.24557678	Npm1
ENSMUSG00000057123.15	23.9846764	-0.0123886	0.00569775	4.68724912	0.03038726	0.08034909	Gja5

ENSMUSG00000057130.13	345.8436	-0.0180529	0.00401469	19.6231712	9.43E-06	6.72E-05	Txnl4a
ENSMUSG00000057132.17	52.2113476	0.00018774	0.00668643	-7.90E-05	1	1	Rpgrip1
ENSMUSG00000057133.15	604.161579	0.0006884	0.00451421	0.02326618	0.87876692	0.9632329	Chd6
ENSMUSG00000057134.8	373.645254	0.00058121	0.00230269	0.06245939	0.80265017	0.91721448	Ado
ENSMUSG00000057135.13	22.0028506	0.04661806	0.00594972	58.6100133	1.92E-14	5.08E-13	Scimp
ENSMUSG00000057137.10	478.102475	0.0070596	0.00336126	4.2437681	0.03939464	0.09931877	Tmem140
ENSMUSG00000057143.16	257.757131	0.01537685	0.00417517	13.0597196	0.00030171	0.00153268	Trim12c
ENSMUSG00000057147.14	114.006137	0.0019464	0.00306672	0.40137904	0.52637792	0.7113476	Dph6
ENSMUSG00000057149.5	1.25861915	0.00201306	0.03851909	0.03583925	0.84984783	0.94743445	Or4k51
ENSMUSG00000057156.11	95.3025692	0.00316153	0.00432071	0.52840457	0.46727839	0.65837682	Homez
ENSMUSG00000057163.4	2.83800982	-0.0571895	0.0510939	0.35298811	NA	NA	Prss2
ENSMUSG00000057176.5	19.2275279	-0.0051225	0.00566934	0.80873894	0.36849335	0.55899692	Cfap119
ENSMUSG00000057177.13	690.60938	-0.0008516	0.00246788	0.11860123	0.73055655	0.87054925	Gsk3a
ENSMUSG00000057181.17	385.11942	-0.0020158	0.00278697	0.51069841	0.47483668	0.66544848	5730455P16Rik
ENSMUSG00000057182.17	15.7982919	0.00462417	0.00928781	0.28494833	0.5934761	0.76885242	Scn3a
ENSMUSG00000057191.15	87.8306114	0.05682443	0.0064106	69.7149146	6.85E-17	2.43E-15	AB124611
ENSMUSG00000057193.9	203.708262	0.01987335	0.00402771	23.9354565	9.96E-07	8.58E-06	Slc44a2
ENSMUSG00000057203.4	0.74545374	-0.0018426	0.03296665	0.0136968	0.90683363	0.97933049	Vmn1r234
ENSMUSG00000057219.12	53.1372408	0.01699219	0.0036166	21.8719168	2.91E-06	2.31E-05	Armc7
ENSMUSG00000057228.7	1502.10182	-0.0208102	0.00484185	17.8174757	2.43E-05	0.00015943	Aadat
ENSMUSG00000057229.12	521.260362	-0.013156	0.00281797	21.8712691	2.92E-06	2.31E-05	Dmac2
ENSMUSG00000057230.15	494.853648	0.00486135	0.00576123	0.71600994	0.39745585	0.58921393	Aak1
ENSMUSG00000057234.10	57.4690282	0.01073178	0.00362844	8.94051796	0.00278914	0.01073858	Mettl15
ENSMUSG00000057236.11	2447.90565	-0.0130934	0.0032928	15.826155	6.94E-05	0.00041232	Rbbp4
ENSMUSG00000057246.17	8.13419276	0.05153032	0.01279287	13.4478676	0.00024528	0.00127719	Tsbp1
ENSMUSG00000057265.14	46.6303373	0.00131881	0.00676873	0.03758689	0.84627502	0.94463997	Bbof1
ENSMUSG00000057270.4	0.9392384	0.00648567	0.03707932	0.11088113	0.7391432	0.87615953	Or9i2
ENSMUSG00000057278.9	654.417363	-0.0093105	0.00452371	4.1648775	0.0412704	0.10322413	Snrpg
ENSMUSG00000057286.7	32.8211807	0.01809454	0.00607309	8.1716136	0.0042551	0.01541879	St6galnac2
ENSMUSG00000057315.15	188.870162	0.00766768	0.00329738	5.37594511	0.02041628	0.05795382	Arhgap24
ENSMUSG00000057322.13	2605.53104	-0.000803	0.00450194	0.03202967	0.85796255	0.95209865	Rpl38
ENSMUSG00000057329.8	82.5009352	0.04979224	0.00491605	94.9393357	1.96E-22	1.37E-20	Bcl2
ENSMUSG00000057335.12	194.785195	0.01216073	0.00399124	8.86638116	0.0029047	0.01111459	Cep170
ENSMUSG00000057337.15	11.2286027	0.06295526	0.00804405	54.0345785	1.97E-13	4.55E-12	Chst3
ENSMUSG00000057342.16	784.415079	-0.0034783	0.00324733	1.11024691	0.29202828	0.47397324	Sphk2
ENSMUSG00000057346.13	1421.7613	0.03686514	0.00566836	41.8487107	9.86E-11	1.58E-09	Apol9a
ENSMUSG00000057363.13	238.114176	0.00211307	0.00311565	0.45461645	0.50015079	0.68724477	Uxs1
ENSMUSG00000057367.13	440.786489	0.00347111	0.00210684	2.71206509	0.09959199	0.20955692	Birc2
ENSMUSG00000057375.14	610.230128	-0.0019752	0.00312436	0.39908882	0.5275602	0.7123739	Yipf1
ENSMUSG00000057378.16	5.71650935	0.04159584	0.0177103	6.90153656	0.00861217	0.02814524	Ryr3
ENSMUSG00000057388.10	1104.36181	-0.0037347	0.00358235	1.0922072	0.29598268	0.47897746	Mrpl18
ENSMUSG00000057396.8	75.7747577	0.0034656	0.00381845	0.80039802	0.37097439	0.56163424	Zfp759
ENSMUSG00000057400.15	39854.7393	-0.0026098	0.00544954	0.2294295	0.63194717	0.79997348	Ces1c
ENSMUSG00000057406.17	371.169421	-0.003896	0.00337605	1.29576604	0.25498805	0.42756635	Nsd2
ENSMUSG00000057409.8	125.136198	0.0146001	0.00275858	27.0893764	1.94E-07	1.92E-06	Zfp53
ENSMUSG00000057411.10	444.740943	-0.0091897	0.003981	5.4592274	0.01946525	0.05564264	Antkmt
ENSMUSG00000057421.13	709.55071	-0.0053419	0.0025027	4.56395539	0.03265158	0.08529407	Las1l
ENSMUSG00000057425.4	90.992423	-0.0470965	0.0087693	24.2279058	8.56E-07	7.43E-06	Ugt2b37
ENSMUSG00000057440.9	120.328294	-0.0091186	0.00366403	6.03882026	0.01399463	0.04234476	Mpp7
ENSMUSG00000057447.3	1.67773939	-0.0045917	0.03191122	-0.0120338	1	1	Or4c11c
ENSMUSG00000057455.13	7.1611594	0.01431978	0.02447168	0.44190149	0.50620611	0.6930619	Rit2
ENSMUSG00000057461.4	4.913687	0.00637322	0.03072867	0.19992052	0.65478501	0.81652528	Or52s1b
ENSMUSG00000057465.6	6876.70789	-0.0212112	0.01464735	-1.0877315	1	1	Saa2
ENSMUSG00000057469.9	319.917291	-0.014729	0.00321272	20.4861673	6.01E-06	4.47E-05	E2f6
ENSMUSG00000057497.9	1290.63958	-0.0064226	0.00219213	8.5968195	0.00336751	0.01262132	Fam136a
ENSMUSG00000057503.7	1.78419224	0.01644466	0.01987318	1.35656921	0.24413295	0.41467088	Or5b106
ENSMUSG00000057506.6	339.174664	-0.0059501	0.0036666	2.62660175	0.10508641	0.21814541	Bloc1s2
ENSMUSG00000057522.16	2549.2196	-0.0092344	0.00219403	17.4928734	2.88E-05	0.00018615	Spop
ENSMUSG00000057530.15	4544.76924	-0.0069391	0.00445654	2.36726816	0.12390371	0.24829166	Ece1
ENSMUSG00000057531.16	535.245269	-0.0018595	0.00255889	0.53830306	0.46313685	0.6542189	Dtnbp1
ENSMUSG00000057541.15	234.993347	-0.0062706	0.00322684	3.75631179	0.0526085	0.1256658	Pus7

ENSMUSG00000057551.11	263.377076	0.00045961	0.00258598	0.03119404	0.85980818	0.95324542	Zfp317
ENSMUSG00000057554.14	2423.2349	-0.0015401	0.00220763	0.47571653	0.49036898	0.67803042	Lgals8
ENSMUSG00000057561.12	1310.43785	-0.0014005	0.00348859	0.15768511	0.69129678	0.84354731	Eif1a
ENSMUSG00000057564.7	16.1045515	-0.0077064	0.02589428	0.03413531	0.85341929	0.94949395	Or4e1
ENSMUSG00000057572.16	455.659916	-0.0114072	0.00315105	13.2464642	0.00027309	0.00140515	Zbtb8os
ENSMUSG00000057594.13	89.3060508	-0.0048876	0.00254892	3.71747136	0.05384586	0.1281801	Arl16
ENSMUSG00000057596.14	196.669537	0.04368852	0.004368	93.395625	4.28E-22	2.88E-20	Trim30d
ENSMUSG00000057604.10	39.2856792	0.00600816	0.00652633	0.83052374	0.36212113	0.5516722	Lmcd1
ENSMUSG00000057606.15	3.60148137	0.03872088	0.01682684	3.32447336	0.06825587	0.15513954	Colq
ENSMUSG00000057614.7	78.3836765	0.0280158	0.00576262	18.5951022	1.62E-05	0.00011025	Gnai1
ENSMUSG00000057637.14	568.442155	-0.019202	0.00369554	26.511281	2.62E-07	2.52E-06	Prdm2
ENSMUSG00000057649.7	1090.759	0.00509513	0.00392559	1.7084283	0.19118942	0.34597077	Brd9
ENSMUSG00000057666.19	15919.0224	-0.0070881	0.00243977	8.46153608	0.00362735	0.01343511	Gapdh
ENSMUSG00000057667.8	96.895951	0.00746555	0.00260583	8.09208721	0.00444589	0.01600299	Bloc1s3
ENSMUSG00000057672.17	549.230552	0.00686542	0.00284957	5.81324417	0.01590593	0.04708298	Pkn1
ENSMUSG00000057691.9	323.642943	0.00377783	0.00204943	3.31904465	0.06848161	0.15557767	Zfp746
ENSMUSG00000057697.3	2.11933035	-0.0051533	0.01038066	0.20509554	0.65063893	0.81357294	Vmn1r53
ENSMUSG00000057706.8	14.088501	0.0170464	0.00581223	8.48517903	0.00358051	0.01329084	Mex3b
ENSMUSG00000057715.14	7.27334217	0.01701389	0.01594991	1.30296223	0.25367285	0.42596666	A830018L16Rik
ENSMUSG00000057716.8	8.76344577	0.02182087	0.01382409	2.87591744	0.08991372	0.1934108	Tmem178b
ENSMUSG00000057719.12	10.4808995	-0.0042678	0.01334096	0.18902174	0.66373207	0.82363259	Sh3rf2
ENSMUSG00000057722.18	286.6787	-0.0061734	0.00766393	0.6191318	0.4313701	0.62285247	Lepr
ENSMUSG00000057729.13	55.5165892	0.049325	0.01427446	8.58184019	0.00339532	0.01270942	Prtn3
ENSMUSG00000057735.7	0.86206467	-0.0004274	0.03961621	-0.0036102	1	1	Or4c99
ENSMUSG00000057738.15	1782.00328	0.00872248	0.00317419	7.63106225	0.00573715	0.01990533	Sptan1
ENSMUSG00000057751.15	20.69663	0.00422456	0.00821138	0.26173701	0.60892931	0.78137266	Megf6
ENSMUSG00000057762.7	1.54690844	0.01723454	0.02745903	0.29068463	0.58978214	0.76618284	Gm6169
ENSMUSG00000057766.15	13.3376562	0.00774497	0.00613682	1.50956429	0.21920558	0.383744	Ankrd29
ENSMUSG00000057777.6	11.5720058	-0.00635	0.00720027	0.727512	0.39369084	0.58510347	Mab21l2
ENSMUSG00000057778.15	247.860562	-0.0086551	0.00267862	10.1289267	0.0014596	0.00612348	Cyb5d2
ENSMUSG00000057788.14	312.031055	-0.0083175	0.00309704	7.36467249	0.00665177	0.02262202	Ddx49
ENSMUSG00000057789.15	216.97879	0.01337273	0.00365181	13.2322101	0.00027518	0.00141433	Bak1
ENSMUSG00000057835.8	86.7759703	-0.0041543	0.00281152	2.14813461	0.14274323	0.27693306	Zfp119a
ENSMUSG00000057836.13	205.950103	-0.0048017	0.00725142	0.39671631	0.52878998	0.71334022	Xlr3a
ENSMUSG00000057841.6	6391.81173	-0.0003895	0.00387549	0.0101368	0.91980315	0.98644397	Rpl32
ENSMUSG00000057842.15	122.544612	-0.0044019	0.00306274	2.06175529	0.1510361	0.28889283	Zfp595
ENSMUSG00000057858.9	132.880432	0.00685305	0.00291344	5.46101834	0.01944531	0.0556066	Fam204a
ENSMUSG00000057863.7	3967.52186	4.60E-05	0.00416265	0.00012554	0.9910604	1	Rpl36
ENSMUSG00000057880.13	5704.30958	-0.0069165	0.00395543	3.01364377	0.08256649	0.18068411	Abat
ENSMUSG00000057894.11	149.794811	-0.0036377	0.00446253	0.63610011	0.42512654	0.61670672	Zfp329
ENSMUSG00000057895.12	148.575039	0.0074431	0.00521389	1.95027209	0.16255753	0.30518841	Zfp105
ENSMUSG00000057897.16	169.513918	0.02688502	0.00565145	21.8454453	2.96E-06	2.34E-05	Camk2b
ENSMUSG00000057899.8	1.29619568	0.01832799	0.01365616	1.8792166	0.17042321	0.31682435	Adgrf2
ENSMUSG00000057914.16	25.2208671	0.01503948	0.00917995	2.47983601	0.11531428	0.23468828	Cacnb2
ENSMUSG00000057933.11	715.876906	0.04826161	0.00693128	45.6474544	1.42E-11	2.55E-10	Gsta2
ENSMUSG00000057948.13	28.1397778	0.04153366	0.0063913	38.6806412	4.99E-10	7.22E-09	Unc13d
ENSMUSG00000057963.10	326.376611	0.00605713	0.00246645	6.04577388	0.01393962	0.04219996	Itpk1
ENSMUSG00000057967.13	1.62893714	0.00118964	0.01536651	0.00185848	0.96561376	1	Fgf18
ENSMUSG00000057969.16	22.53932	0.02724784	0.00822145	10.2116565	0.00139556	0.00587569	Sema3b
ENSMUSG00000057982.9	590.596287	-0.0018962	0.00307217	0.36926833	0.54340336	0.72685939	Zfp809
ENSMUSG00000058006.13	323.20789	-0.0023218	0.00552643	0.17723153	0.6737633	0.83118265	Mdn1
ENSMUSG00000058013.12	395.410076	0.01741565	0.00414986	16.8682508	4.01E-05	0.0002518	Septin11
ENSMUSG00000058014.5	0.94229881	0.0009316	0.0418663	0.01924313	0.88967173	0.96970934	Or5p76
ENSMUSG00000058022.16	2499.90459	-0.0053258	0.00492516	1.10483587	0.29320728	0.47530599	Adtrp
ENSMUSG00000058030.6	0.78875851	-0.011297	0.04548892	0.05858585	0.80874519	0.92098111	Vmn1r237
ENSMUSG00000058046.2	1.31746671	0.00305829	0.01005427	0.09655072	0.75600884	0.88732041	4933430I17Rik
ENSMUSG00000058056.17	1186.05788	-0.0097721	0.00379977	6.57950437	0.01031596	0.03272966	Palld
ENSMUSG00000058070.15	78.6833846	0.00822242	0.00497788	2.67801727	0.10174233	0.21307891	Eml1
ENSMUSG00000058076.13	3039.22606	-0.0121259	0.00262498	21.6763328	3.23E-06	2.54E-05	Sdhc
ENSMUSG00000058093.14	230.652325	-1.47E-05	0.00344589	1.93E-05	0.99649929	1	Zfp729b
ENSMUSG00000058099.17	169.923134	0.04850978	0.00496156	91.851502	9.34E-22	5.94E-20	Nfam1

ENSMUSG00000058114.4	1.25164955	-0.0014032	0.04124862	-0.0069925	1	1	Or14j6
ENSMUSG00000058126.8	200.428429	0.0170927	0.00526613	10.5524045	0.00116037	0.00500521	Tpm3-rs7
ENSMUSG00000058132.6	1.38105437	-0.0009832	0.03891559	-0.0017977	1	1	Vmn1r82
ENSMUSG00000058135.13	25951.3585	0.00928687	0.00459504	4.1399984	0.04188121	0.10444176	Gstm1
ENSMUSG00000058145.17	3.20273815	-0.0153799	0.01549969	0.96467227	0.3260125	0.51279299	Adamts17
ENSMUSG00000058152.9	109.269336	-0.0036624	0.00382194	0.92118669	0.33716358	0.52447556	Chsy3
ENSMUSG00000058153.16	10.7239675	0.01885136	0.01605875	1.73088311	0.18829816	0.34205178	Sez6l
ENSMUSG00000058163.15	32.9755754	0.03913608	0.00639538	36.3294751	1.67E-09	2.24E-08	Gm5431
ENSMUSG00000058173.13	118.687788	0.0069931	0.00412749	2.89579561	0.08881092	0.19145365	Smco4
ENSMUSG00000058174.8	18.4364364	0.02444324	0.00862177	8.10476338	0.00441491	0.01591472	Gm5148
ENSMUSG00000058186.14	2.74015301	0.01198538	0.01466833	0.67291973	0.41203551	0.60350677	Zfp980
ENSMUSG00000058192.17	68.9317064	-0.0023954	0.00318689	0.55994422	0.45428272	0.64601243	Zfp846
ENSMUSG00000058207.7	159845.795	-0.0520785	0.00663262	58.5774622	1.95E-14	5.16E-13	Serpina3k
ENSMUSG00000058216.14	297.668509	0.01900669	0.00468629	16.7661695	4.23E-05	0.00026445	Gstp3
ENSMUSG00000058230.14	1780.5313	-0.0034386	0.00302066	1.27405007	0.25900826	0.43255636	Arhgap35
ENSMUSG00000058239.14	1254.29383	-0.0009269	0.00270742	0.11559839	0.73385831	0.8725582	Usf2
ENSMUSG00000058240.15	316.321799	-0.0007303	0.00260822	0.07804602	0.77996306	0.90343314	Cryzl1
ENSMUSG00000058246.10	6.15335122	-0.0063688	0.00674536	0.88599326	0.34656541	0.53498866	Gm10037
ENSMUSG00000058248.13	3.65992555	0.00746175	0.02005388	0.23712574	0.6262898	0.79505422	Kcnh1
ENSMUSG00000058254.13	400.597292	-0.0084686	0.0032797	6.85665892	0.00883115	0.0287378	Tspan7
ENSMUSG00000058258.16	6167.74607	0.00838607	0.00486214	2.77697456	0.09562866	0.20296028	Idi1
ENSMUSG00000058260.3	26.1006811	-0.1079454	0.00961334	122.937335	1.44E-28	2.26E-26	Serpina9
ENSMUSG00000058267.14	573.931526	-0.0143156	0.00361806	15.8539788	6.84E-05	0.00040691	Mrps14
ENSMUSG00000058270.4	1.56218862	-0.001286	0.03172122	-0.0323476	1	1	Or8c9
ENSMUSG00000058290.5	19.3548807	0.04686033	0.01226826	12.4219136	0.00042433	0.00206737	Espl1
ENSMUSG00000058291.15	401.032614	-0.000685	0.00247943	0.07636089	0.78229097	0.90495438	Zfp68
ENSMUSG00000058297.17	67.9502779	0.0266189	0.0069801	13.9069602	0.00019209	0.00102897	Spock2
ENSMUSG00000058298.11	134.60741	0.00230091	0.00337567	0.46383422	0.49583747	0.68308417	Mcm9
ENSMUSG00000058301.9	918.728988	-0.00272	0.00234064	1.32623299	0.24947648	0.42098821	Upf1
ENSMUSG00000058317.13	325.31591	0.00758988	0.00298804	6.39072816	0.0114718	0.03577126	Ube2e2
ENSMUSG00000058318.17	338.716708	0.00849409	0.00422457	3.95102215	0.04684291	0.11446714	Phf21a
ENSMUSG00000058325.7	351.956521	0.00099028	0.00293892	0.11021843	0.7398956	0.87676736	Dock1
ENSMUSG00000058331.16	68.0502907	0.0068312	0.00376279	3.25160586	0.07135352	0.16073965	Zfp85
ENSMUSG00000058351.12	231.909863	-0.0137459	0.00542594	6.48462723	0.01088114	0.03427841	Uqcc5
ENSMUSG00000058355.9	1534.14894	-0.0081844	0.00163949	24.7460667	6.54E-07	5.82E-06	Abce1
ENSMUSG00000058385.9	19.0838022	0.01344464	0.01276096	0.96105001	0.32692247	0.51374465	H2bc8
ENSMUSG00000058388.15	119.440286	0.01074994	0.00259134	17.191867	3.38E-05	0.00021475	Phtf1
ENSMUSG00000058392.14	109.329018	0.00392772	0.00475842	0.64690554	0.42122121	0.61279899	Rrp1b
ENSMUSG00000058396.8	477.567686	-0.0015043	0.00291419	0.26471775	0.60689737	0.77977236	Gpr182
ENSMUSG00000058402.15	62.9621496	-0.0008325	0.00297798	0.07788876	0.78017915	0.9034863	Zfp420
ENSMUSG00000058407.13	896.11754	-0.0025013	0.00261856	0.91434589	0.33896395	0.52659753	Txndc9
ENSMUSG00000058420.9	3.59498298	0.04421757	0.01392241	10.0064726	0.00155991	0.0064889	Syt17
ENSMUSG00000058427.11	6.61418752	0.05546357	0.01016852	26.8129766	2.24E-07	2.19E-06	Cxcl2
ENSMUSG00000058435.7	0.63172429	0.04969663	0.03086087	2.6521675	0.10340881	0.21549746	Btnl4
ENSMUSG00000058440.15	207.02232	-0.0023883	0.0024909	0.90780712	0.34069692	0.52842471	Nrf1
ENSMUSG00000058441.8	4.34314506	0.01502793	0.01590809	0.9049297	0.34146331	0.52930536	Panx2
ENSMUSG00000058443.6	858.527474	0.0107645	0.00546602	3.82802423	0.05040231	0.12135173	Rpl10-ps3
ENSMUSG00000058444.9	309.328563	-0.0054987	0.00190567	8.43009607	0.00369061	0.0136266	Map2k5
ENSMUSG00000058446.15	1184.2758	-0.0020327	0.00247318	0.66161226	0.41599133	0.60722686	Znrf2
ENSMUSG00000058447.9	1.19319554	-0.0354141	0.04100763	1.11233181	0.29157563	0.47327113	Gm26920
ENSMUSG00000058454.16	2079.2358	0.01542278	0.00462932	10.8136098	0.00100757	0.00442459	Dhcr7
ENSMUSG00000058470.11	10.6520407	0.07647873	0.00974696	51.0377345	9.06E-13	1.91E-11	Gm8369
ENSMUSG00000058486.12	281.322804	-0.0100247	0.00353888	8.05345353	0.0045417	0.01628219	Wdr91
ENSMUSG00000058488.8	34.1475806	0.00675265	0.01476466	0.14749404	0.70094174	0.85063224	Kl
ENSMUSG00000058498.13	6.73721832	0.0060013	0.01777466	0.19512353	0.65868613	0.82008926	Rnf207
ENSMUSG00000058503.12	389.226551	0.00306901	0.0036196	0.70523892	0.40102892	0.5927598	Fam133b
ENSMUSG00000058513.8	1.09364403	-0.0062369	0.03985879	0.01503766	0.90240158	0.97691499	Or6c211
ENSMUSG00000058515.5	1.44965407	-0.0059081	0.04084246	-0.0067505	1	1	Or8d1b
ENSMUSG00000058523.3	59.9816501	-0.1055723	0.0114699	71.2153639	3.20E-17	1.20E-15	Mup5
ENSMUSG00000058546.9	4083.00942	-0.0015939	0.00303629	0.27646669	0.59902648	0.77331403	Rpl23a
ENSMUSG00000058558.13	8727.98121	-0.0028768	0.00293633	0.94090295	0.33204573	0.51899474	Rpl5

ENSMUSG00000058569.9	934.766032	-0.0111553	0.00244397	20.9001532	4.84E-06	3.67E-05	Tmed9
ENSMUSG00000058571.12	33.5662271	0.01183116	0.0061744	3.70073263	0.05438858	0.12935279	Gpc6
ENSMUSG00000058579.6	19.6706656	-0.0842923	0.01273185	36.1743945	1.80E-09	2.42E-08	Cela2a
ENSMUSG00000058586.13	479.00373	-0.0007887	0.0039188	0.04080083	0.83992286	0.94039782	Serhl
ENSMUSG00000058587.10	513.950155	0.00643393	0.00284609	4.95734175	0.02598012	0.07070117	Tmod3
ENSMUSG00000058589.16	15.69651	0.04203875	0.01583211	6.96708166	0.00830228	0.02723092	Anks1b
ENSMUSG00000058594.16	521.042319	-0.0056829	0.00286972	3.93754266	0.04721971	0.1152484	Fbh1
ENSMUSG00000058600.14	2907.47204	-0.0009125	0.00298348	0.09295697	0.76045111	0.89065466	Rpl30
ENSMUSG00000058620.6	117.634058	0.03246303	0.00393891	65.9625371	4.60E-16	1.47E-14	Adra2b
ENSMUSG00000058624.14	549.787091	0.01416961	0.00385269	12.7475679	0.00035647	0.00177454	Gda
ENSMUSG00000058626.17	13.7080411	0.0015866	0.01369888	0.00978157	0.92121623	0.98718877	Capn11
ENSMUSG00000058638.15	611.889125	-0.000892	0.00213753	0.174605	0.67605121	0.83261156	Zfp110
ENSMUSG00000058655.10	4193.37543	-0.0017966	0.00225554	0.62662832	0.42859477	0.61998383	Eif4b
ENSMUSG00000058656.14	23.618361	0.02707718	0.00770404	10.4793096	0.00120719	0.00518629	Samd12
ENSMUSG00000058659.7	0.67528285	-0.0112542	0.04998598	0.06168103	0.80385847	0.91805563	Or7e165
ENSMUSG00000058665.9	2.5673151	0.00661198	0.05888721	0.088496	0.76609796	0.89425983	En1
ENSMUSG00000058672.8	2120.34194	0.00922632	0.00597836	2.20686606	0.13739754	0.26897271	Tubb2a
ENSMUSG00000058690.15	939.90229	0.01091369	0.00363667	8.7836455	0.00303943	0.01154955	Ccser2
ENSMUSG00000058692.5	1.11410674	0.00714022	0.03515134	0.10411746	0.74694338	0.88117874	Or7g28
ENSMUSG00000058704.10	658.57128	-0.0135842	0.00231113	34.3901458	4.51E-09	5.73E-08	Memo1
ENSMUSG00000058706.6	701.94283	-0.0086561	0.0038981	4.8341357	0.02790162	0.07496158	0610030E20Rik
ENSMUSG00000058709.12	1074.20703	-0.0023496	0.00225923	1.08587741	0.29738645	0.48088282	Egln2
ENSMUSG00000058715.12	274.391774	0.05834817	0.0054527	106.722286	5.12E-25	4.79E-23	Fcer1g
ENSMUSG00000058728.17	1.21087985	0.02098808	0.01351211	2.51497866	0.11276916	0.23056288	Cd300c
ENSMUSG00000058729.14	66.1376537	-0.0063355	0.00391752	2.60835307	0.10630211	0.22008792	Lin9
ENSMUSG00000058740.15	5.87411608	0.03886476	0.01273531	8.65997968	0.00325275	0.01223837	Kcnt1
ENSMUSG00000058743.7	0.95760405	0.01909352	0.01800092	1.68018576	0.19489977	0.35096244	Kcnj14
ENSMUSG00000058748.10	233.799191	0.00748678	0.00349567	4.39421979	0.03606096	0.09247947	Zfp958
ENSMUSG00000058755.4	1.68519689	0.0822317	0.0134874	32.772523	1.04E-08	1.25E-07	Osm
ENSMUSG00000058756.14	230.893476	0.00318197	0.00330487	0.92598146	0.33590932	0.52323057	Thra
ENSMUSG00000058761.6	866.089262	-0.0093957	0.0039053	5.77627489	0.01624393	0.04790914	Rnf169
ENSMUSG00000058773.3	3.02981558	-0.0089196	0.02301536	0.10646404	0.74420626	0.87935719	H1f5
ENSMUSG00000058793.15	1917.77937	0.00396279	0.00299166	1.72763028	0.18871382	0.34264893	Cds2
ENSMUSG00000058794.13	33.523346	0.06307035	0.00721453	68.7139829	1.14E-16	3.91E-15	Nfe2
ENSMUSG00000058799.17	1776.89694	0.00296532	0.00280366	1.0789986	0.29892168	0.48280263	Nap1l1
ENSMUSG00000058806.16	70.22986	-0.0190497	0.0050106	14.6200506	0.00013151	0.00073085	Col13a1
ENSMUSG00000058818.14	197.509482	0.06001334	0.00550508	107.585779	3.31E-25	3.16E-23	Pirb
ENSMUSG00000058833.12	447.106946	0.00085652	0.00381299	0.04994456	0.82315978	0.92955242	Rex1bd
ENSMUSG00000058835.15	762.087876	0.006044	0.00261719	5.16361225	0.02306488	0.06406391	Abi1
ENSMUSG00000058881.14	113.724085	0.00725714	0.00475728	2.33361852	0.12660727	0.25255558	Zfp516
ENSMUSG00000058883.17	40.1973774	0.00682736	0.01491917	0.19921862	0.65535229	0.81710309	Zfp708
ENSMUSG00000058884.8	0.9041108	0.00347051	0.04017799	0.04101432	0.83951028	0.94029297	Or5m13
ENSMUSG00000058886.10	197.322302	0.00090402	0.00315519	0.0820645	0.77451892	0.89969972	Deaf1
ENSMUSG00000058897.19	31.1598961	0.04121187	0.00853167	21.2941835	3.94E-06	3.05E-05	Col25a1
ENSMUSG00000058900.7	64.3047793	0.01197297	0.00385542	9.17122343	0.0024585	0.00963226	Rsl1
ENSMUSG00000058914.13	8.43936162	-0.0466094	0.01135615	16.4792618	4.92E-05	0.00030234	C1qtnf3
ENSMUSG00000058921.4	893.817357	-0.0145087	0.00686024	4.36339186	0.03671913	0.09382053	Slc10a5
ENSMUSG00000058927.7	833.075362	-0.0147456	0.00442164	11.1846786	0.00082476	0.00370457	Gm10053
ENSMUSG00000058952.13	11061.8783	0.00993861	0.00507376	3.8154233	0.05078277	0.12212097	Cfi
ENSMUSG00000058966.14	6.022634	0.00797698	0.00746572	1.11612312	0.29075478	0.47221279	Tlcd3b
ENSMUSG00000058975.8	13.4615218	0.06525735	0.01478393	19.3753494	1.07E-05	7.57E-05	Kcnc1
ENSMUSG00000058979.8	335.181762	-0.0151376	0.00278883	29.529106	5.51E-08	5.94E-07	Hdhd5
ENSMUSG00000058981.5	1.46176238	-0.0070329	0.0336479	-0.0102701	1	1	Or10j7
ENSMUSG00000058997.9	2920.75931	-0.0200559	0.00282719	49.3753922	2.11E-12	4.25E-11	Vwa8
ENSMUSG00000059003.13	25.6199724	0.01651053	0.01609936	1.05279849	0.30486345	0.48964993	Grin2a
ENSMUSG00000059005.14	4367.85844	0.00698565	0.00314313	4.84962068	0.02765221	0.07436774	Hnrnpa3
ENSMUSG00000059013.13	130.920721	0.01031822	0.00265743	15.0877248	0.00010263	0.00058525	Sh2d3c
ENSMUSG00000059022.16	102.571972	0.00271058	0.00513642	0.26237816	0.60849101	0.78110668	Kcp
ENSMUSG00000059030.6	1.21091944	-0.0021192	0.03363354	-0.0172964	1	1	Or14j7
ENSMUSG00000059031.4	2.65616318	0.01790956	0.0113869	2.45944088	0.11682036	0.23718042	Or5p58
ENSMUSG00000059040.6	171.292055	-0.001518	0.00592638	0.06674188	0.79614104	0.91380518	Eno1b

ENSMUSG00000059049.16	128.376115	0.00710047	0.00759789	0.82905728	0.36254527	0.55207982	Frem1
ENSMUSG00000059060.16	68.0471479	0.06592757	0.0093958	46.938863	7.32E-12	1.38E-10	Rad51b
ENSMUSG00000059070.17	3592.89345	-0.0044532	0.00446385	0.98735556	0.32038956	0.50643689	Rpl18
ENSMUSG00000059087.5	1.29754984	0.04562046	0.02938373	4.18056116	0.04089016	0.10246869	Or2ag16
ENSMUSG00000059089.5	176.151789	0.0765501	0.00646873	125.319126	4.33E-29	7.25E-27	Fcgr4
ENSMUSG00000059108.5	15.3367377	0.07326957	0.00934432	49.7585455	1.74E-12	3.53E-11	Ifitm6
ENSMUSG00000059112.7	1.96494757	-0.0045491	0.025533	-0.0112299	1	1	Or4c52
ENSMUSG00000059119.10	915.723106	0.00626467	0.00295345	4.4432425	0.03503964	0.09036077	Nap1l4
ENSMUSG00000059136.5	4.74330595	0.00798479	0.01470728	0.47673791	0.48990364	0.67777325	Or13a25
ENSMUSG00000059142.17	258.347198	0.00882157	0.00296184	8.68811056	0.00320292	0.01207548	Zfp945
ENSMUSG00000059146.13	57.9490373	0.02824571	0.01084925	5.90766654	0.01507513	0.04505918	Ntrk3
ENSMUSG00000059149.18	102.323353	0.00942648	0.00786575	1.31789766	0.2509697	0.42272166	Mfsd4a
ENSMUSG00000059173.21	85.2231123	0.03458316	0.0041741	66.8417823	2.94E-16	9.61E-15	Pde1a
ENSMUSG00000059182.9	604.430652	0.01432893	0.00286029	24.4469393	7.64E-07	6.69E-06	Skap2
ENSMUSG00000059183.13	146.046123	-0.0034819	0.00189781	3.39703755	0.06531362	0.14983825	Mtfmt
ENSMUSG00000059187.13	3.82548894	0.00501732	0.01531061	0.11475261	0.7347969	0.87327017	Tafa1
ENSMUSG00000059201.13	3.40589565	0.02672633	0.03092349	0.87854976	0.34859919	0.53651293	Lep
ENSMUSG00000059203.11	2.37224514	0.01165623	0.03686172	0.30156623	0.5829022	0.76037449	Il1rapl2
ENSMUSG00000059206.5	2.4206886	-0.0042156	0.02470676	0.02354154	0.87805721	0.96289089	Vmn1r71
ENSMUSG00000059208.16	1807.01649	0.0005732	0.00285503	0.03956756	0.84232848	0.94223647	Hnrnpm
ENSMUSG00000059213.8	47.017581	0.01287675	0.01536718	0.67391952	0.41168841	0.60327141	Ddn
ENSMUSG00000059248.14	1266.4934	0.01285021	0.00293409	18.7831803	1.46E-05	0.00010062	Septin9
ENSMUSG00000059263.10	1793.24207	-0.0080697	0.00247888	10.4221229	0.00124515	0.00533092	Usp47
ENSMUSG00000059273.10	483.067709	0.00354846	0.00347853	1.01559553	0.31356605	0.49897239	Zc3h4
ENSMUSG00000059278.10	532.606294	-0.011643	0.00382468	9.29142261	0.00230229	0.00911105	Naa38
ENSMUSG00000059288.15	91.7019144	0.00601881	0.00314462	3.61459031	0.05727483	0.13484568	Cdyl
ENSMUSG00000059291.16	6109.99556	-0.0037385	0.00319795	1.38359101	0.23949041	0.40905358	Rpl11
ENSMUSG00000059303.7	1.01607605	-0.0101125	0.03878714	0.0188946	0.89066908	0.97031506	Or7g21
ENSMUSG00000059305.13	2.9498509	-0.016044	0.00965307	2.61525073	0.10584079	0.21924829	Vpreb1a
ENSMUSG00000059316.3	557.239791	-0.0083137	0.0029257	8.02792387	0.00460616	0.01646823	Slc27a4
ENSMUSG00000059319.4	2.08364194	0.01281491	0.02565371	0.82525935	0.36364693	0.55328193	Or14c41
ENSMUSG00000059323.15	27.5345987	0.01854475	0.00688286	7.21666696	0.00722297	0.02426103	Tonsl
ENSMUSG00000059325.15	947.553526	-0.030535	0.00463599	42.8763683	5.83E-11	9.69E-10	Hopx
ENSMUSG00000059326.8	105.832577	0.04444056	0.00554066	62.5015525	2.66E-15	7.81E-14	Csf2ra
ENSMUSG00000059327.10	29.6403329	0.01966327	0.00471461	17.4542473	2.94E-05	0.0001895	Eda
ENSMUSG00000059336.15	8.32020378	0.05378792	0.00914427	31.7951804	1.71E-08	2.00E-07	Slc14a1
ENSMUSG00000059343.5	1.84535508	0.0031855	0.01626507	0.0332571	0.85529605	0.95052597	Aldoart1
ENSMUSG00000059355.14	472.46139	-0.001677	0.00310008	0.29842667	0.58487044	0.76188856	Wdr83os
ENSMUSG00000059361.7	2.16021021	0.01666556	0.0263365	0.52071179	0.47053821	0.66146825	Nrsn2
ENSMUSG00000059363.11	326.56495	-0.0093109	0.00323534	8.23607304	0.00410659	0.01494311	Fxn
ENSMUSG00000059366.4	1.85578109	0.00148358	0.0261645	0.00494874	0.94391728	0.99904273	Or10d1
ENSMUSG00000059395.5	12.0281989	0.00763017	0.00713329	1.10018174	0.29422622	0.47665497	Nkapl
ENSMUSG00000059401.15	21.8259978	0.01969392	0.00566815	12.0746131	0.00051113	0.00244138	Mamld1
ENSMUSG00000059406.9	3.66170587	-0.0149129	0.00955345	2.50192097	0.11370753	0.23209915	Tmprss9
ENSMUSG00000059408.4	0.96374732	-0.0306778	0.04674545	0.68019548	0.40951942	0.60124028	Mrgprh
ENSMUSG00000059409.10	523.671869	-0.0053657	0.00225167	5.70403524	0.01692596	0.04960432	Ppp2r5d
ENSMUSG00000059412.8	76.3885896	0.02547809	0.01303824	3.30966428	0.06887356	0.15616726	Fxyd2
ENSMUSG00000059423.14	145.241448	-0.0069495	0.00340207	4.13866527	0.04191421	0.10449091	Zfp933
ENSMUSG00000059430.15	8.47343566	-0.0030675	0.00950204	0.09198224	0.76167212	0.89131623	Actg2
ENSMUSG00000059434.10	3323.0762	-0.0033068	0.00526078	0.39757249	0.52834559	0.71302621	Gckr
ENSMUSG00000059436.14	447.19463	-0.0055324	0.00436765	1.55607481	0.21224087	0.37449912	Max
ENSMUSG00000059439.16	400.475238	-0.0112498	0.00381463	8.66750286	0.00323935	0.01219331	Bcas3
ENSMUSG00000059447.14	4162.1942	-0.0199064	0.00375283	28.0519785	1.18E-07	1.21E-06	Hadhb
ENSMUSG00000059456.14	771.618615	0.00499091	0.00330426	2.23950739	0.13452383	0.26458298	Ptk2b
ENSMUSG00000059474.14	359.526764	0.00531165	0.00300893	3.03605312	0.08143369	0.17868593	Mbtd1
ENSMUSG00000059475.14	193.067085	-0.0015992	0.00259121	0.37112031	0.54239424	0.72588008	Zfp426
ENSMUSG00000059479.8	23.1092842	0.02338356	0.00623913	13.3925713	0.00025262	0.00130982	B3gnt8
ENSMUSG00000059481.6	37170.0135	-0.0014618	0.00536319	0.07439009	0.78504888	0.90694371	Plg
ENSMUSG00000059482.16	89.8894821	-0.0069906	0.00262805	7.0556263	0.00790167	0.02611304	Cfap418
ENSMUSG00000059486.12	374.344639	0.0031527	0.00225622	1.91540754	0.16636382	0.31089905	Kbtbd2
ENSMUSG00000059493.14	11.4702717	0.04256068	0.01023194	16.4763487	4.93E-05	0.00030273	Nhs

ENSMUSG00000059495.15	2786.00955	-0.0024487	0.00318678	0.58352293	0.44493475	0.63724988	Arhgef12
ENSMUSG00000059498.14	271.887118	0.05431829	0.00608281	71.1706209	3.28E-17	1.22E-15	Fcgr3
ENSMUSG00000059518.15	583.970713	-0.0111109	0.00293069	14.3799153	0.00014939	0.00082055	Znhit1
ENSMUSG00000059534.9	2620.21298	-0.018388	0.00414087	20.1563795	7.14E-06	5.23E-05	Uqcr10
ENSMUSG00000059540.16	17.724141	0.01794357	0.00588932	9.04617325	0.00263245	0.01021873	Tcea2
ENSMUSG00000059552.14	328.933572	0.00342154	0.0029427	1.32150572	0.250322	0.42199233	Trp53
ENSMUSG00000059554.13	195.217293	-0.0134254	0.00362727	13.4448251	0.00024568	0.00127833	Ccdc28a
ENSMUSG00000059555.7	122.067993	0.03080903	0.0033776	80.4753927	2.94E-19	1.38E-17	Tor4a
ENSMUSG00000059586.11	188.320929	0.00687225	0.00272534	6.40544265	0.01137711	0.03555119	Nsmce2
ENSMUSG00000059588.15	427.629852	0.00637731	0.00392264	2.54736561	0.11047833	0.22704033	Calcrl
ENSMUSG00000059602.15	31.4290802	-0.003007	0.00504483	0.36659172	0.54486796	0.72811647	Syn3
ENSMUSG00000059654.8	2.13429602	-0.3161293	0.06705897	2.67842341	0.10171638	0.21304348	Reg1
ENSMUSG00000059657.6	1.45254368	0.09560258	0.017387	27.522493	1.55E-07	1.56E-06	Stfa2l1
ENSMUSG00000059668.7	2.21816228	-9.09E-05	0.01183308	0.00026059	0.98712046	1	Krt4
ENSMUSG00000059669.9	101.173832	0.01274335	0.0031178	16.5295642	4.79E-05	0.00029558	Taf1b
ENSMUSG00000059674.8	8.98253301	-0.0092698	0.00901868	1.02819766	0.31058236	0.4957631	Cdh24
ENSMUSG00000059689.16	272.611324	-0.0069047	0.00268053	6.64259734	0.00995687	0.03178704	Zfp637
ENSMUSG00000059708.13	321.490819	-0.0051849	0.00489217	1.12869182	0.28805455	0.46911199	Akap17b
ENSMUSG00000059713.13	52.7439697	0.04925517	0.00506013	92.9822027	5.28E-22	3.52E-20	Rcan3
ENSMUSG00000059714.14	406.524636	0.01093495	0.00373105	8.53926812	0.00347565	0.0129587	Flot1
ENSMUSG00000059729.3	1.43152072	-0.0017709	0.01992673	0.02631884	0.87112408	0.9593469	Or2y1
ENSMUSG00000059734.8	1365.46013	-0.0150044	0.00325833	21.410512	3.71E-06	2.88E-05	Ndufs8
ENSMUSG00000059741.14	797.966338	-0.0348222	0.02290209	2.66315961	0.10269655	0.21441098	Myl3
ENSMUSG00000059742.11	14.3673233	0.02965046	0.01130183	6.49336321	0.01082779	0.0341286	Kcnh7
ENSMUSG00000059743.13	5880.11502	0.00955777	0.00499835	3.43944181	0.0636572	0.14673755	Fdps
ENSMUSG00000059772.13	166.947624	0.00984008	0.00344839	8.15292751	0.00429917	0.01555534	Slx1b
ENSMUSG00000059791.15	19.3057208	0.03045304	0.00784958	14.0663734	0.00017647	0.0009564	Nrm
ENSMUSG00000059796.17	4338.00446	-0.003511	0.00178624	3.87421697	0.04903318	0.11870025	Eif4a1
ENSMUSG00000059810.19	322.434146	0.00317493	0.00549647	0.31695883	0.57344103	0.75252769	Rgs3
ENSMUSG00000059811.14	2077.88439	-0.0096318	0.00241211	15.6831582	7.49E-05	0.00044116	Atl2
ENSMUSG00000059820.15	275.121868	0.00018383	0.00332763	0.00297963	0.95646829	1	Nkapd1
ENSMUSG00000059824.13	926.992966	0.01314333	0.01128212	1.20138761	0.27304452	0.45057217	Dbp
ENSMUSG00000059834.13	139.700454	-0.0003251	0.00294023	0.01221231	0.91200546	0.98198164	Sclt1
ENSMUSG00000059839.10	177.438781	-0.0037945	0.00342609	1.20140892	0.27304026	0.45057217	Zfp874b
ENSMUSG00000059842.13	36.3726793	-0.0116098	0.0037481	9.27334441	0.00232513	0.00918908	Zfp341
ENSMUSG00000059851.16	202.524605	-0.011575	0.00371147	9.81201837	0.00173375	0.00711913	Kmt5c
ENSMUSG00000059852.8	10.859146	0.00198347	0.01039389	0.03940486	0.84264875	0.94228681	Kcng2
ENSMUSG00000059854.9	3.77681772	0.02865808	0.01686852	3.16130506	0.07540288	0.16806215	Hydin
ENSMUSG00000059857.17	4.86290021	0.03937301	0.01890027	5.10782766	0.02381813	0.06578924	Ntng1
ENSMUSG00000059864.3	4.36590587	-0.0157172	0.00961558	2.53019789	0.11168621	0.22884466	Or2y1g
ENSMUSG00000059866.14	109.657797	0.01414697	0.00335931	17.3014041	3.19E-05	0.00020348	Tnip2
ENSMUSG00000059873.5	15.0272287	0.11976898	0.00887702	153.730322	2.65E-35	1.03E-32	Or5m11b
ENSMUSG00000059878.13	296.500675	-0.0040637	0.00182515	4.90758857	0.02673895	0.07239867	Zfp422
ENSMUSG00000059883.17	210.185854	0.01175481	0.00257918	20.4496638	6.12E-06	4.55E-05	Irak4
ENSMUSG00000059890.17	1398.58295	-0.0017831	0.00279695	0.40491012	0.5245643	0.71007653	Ube4a
ENSMUSG00000059891.15	1.73509248	-0.0051917	0.0191345	0.00674056	0.93456645	0.99423346	Tsks
ENSMUSG00000059895.14	180.449024	0.01561529	0.00496171	9.62175365	0.00192286	0.00780864	Ptp4a3
ENSMUSG00000059897.6	207.454286	-0.0006175	0.00335921	0.03391289	0.85389224	0.9497266	Zfp930
ENSMUSG00000059898.10	1.67916741	0.06681577	0.02046276	11.0416156	0.00089089	0.00396239	Dsc3
ENSMUSG00000059900.15	2.08310481	0.07451015	0.01699217	15.0153869	0.00010664	0.00060563	Tmem40
ENSMUSG00000059901.13	21.7204646	0.0148733	0.00416494	12.7097785	0.00036375	0.00180466	Adamts14
ENSMUSG00000059908.10	49764.3707	-0.0242938	0.00621066	15.2797732	9.27E-05	0.00053395	Mug1
ENSMUSG00000059910.5	1.83414408	0.01673679	0.03080245	0.64859614	0.42061504	0.61223318	Or4x11
ENSMUSG00000059920.10	581.761015	-0.0054198	0.00259441	4.40915578	0.03574653	0.09178289	4930453N24Rik
ENSMUSG00000059921.16	8.57067609	0.02920358	0.014937	3.96778143	0.04637884	0.11347421	Unc5c
ENSMUSG00000059923.14	910.628781	0.01014861	0.00269619	13.9302802	0.00018972	0.00101859	Grb2
ENSMUSG00000059939.14	76.1845404	-0.0030396	0.00347689	0.75573046	0.38466798	0.5763422	9430015G10Rik
ENSMUSG00000059970.8	77.2996735	0.02890306	0.00468405	38.5155081	5.43E-10	7.82E-09	Hspa2
ENSMUSG00000059974.11	116.16314	-0.0005428	0.00629112	0.00747106	0.93112041	0.99233681	Ntm
ENSMUSG00000059975.16	88.3388419	-0.0045798	0.00339419	1.79297243	0.18056442	0.3313204	Zfp74
ENSMUSG00000059981.13	775.33733	-0.0065057	0.00344082	3.53978095	0.05991337	0.13976034	Taok2

ENSMUSG00000059991.8	6.84156242	0.01625092	0.02299122	0.78132458	0.37673634	0.56781286	Nptx2
ENSMUSG00000059994.11	13.3027375	0.07197847	0.00745541	78.4589009	8.17E-19	3.65E-17	Fcrl1
ENSMUSG00000059995.13	437.149095	-0.0001217	0.00316941	0.00145013	0.96962346	1	Atxn7l3
ENSMUSG00000060002.15	5393.84329	-0.0009697	0.0033276	0.07925776	0.7783058	0.9023802	Chpt1
ENSMUSG00000060012.10	854.308794	-0.0042346	0.0033836	1.56257951	0.21128793	0.37345902	Kif13b
ENSMUSG00000060032.7	787.350633	-0.001855	0.004544	0.15915747	0.6899334	0.84275567	H2aj
ENSMUSG00000060036.15	6414.14545	0.00166497	0.00288686	0.33585426	0.56223205	0.74332221	Rpl3
ENSMUSG00000060038.15	197.159306	-0.0023112	0.00400626	0.3421905	0.55856754	0.74017776	Dhps
ENSMUSG00000060044.9	50.2150201	0.06060162	0.00588688	98.0947367	3.99E-23	2.96E-21	Tmem26
ENSMUSG00000060057.5	1.6244413	-0.0021529	0.04097722	-0.0225432	1	1	Or5h25
ENSMUSG00000060063.10	91.6846012	0.04377963	0.00748081	30.5708106	3.22E-08	3.59E-07	Alox5ap
ENSMUSG00000060073.10	4433.30104	-0.0041574	0.00293232	1.99891753	0.15741159	0.29800805	Psma3
ENSMUSG00000060090.17	278.277889	0.01138603	0.00343086	10.6378095	0.00110799	0.00480386	Rp2
ENSMUSG00000060093.7	5.98801601	0.02397224	0.01847942	1.63794874	0.20060714	0.3591014	H4c1
ENSMUSG00000060098.12	239.957188	-0.0031001	0.00289342	1.15140739	0.28325473	0.46327203	Prmt7
ENSMUSG00000060121.16	240.527019	-0.0136316	0.00311249	19.5734786	9.68E-06	6.88E-05	Gemin2
ENSMUSG00000060126.15	20117.0925	-0.0009603	0.0034024	0.07979058	0.77758141	0.90185066	Tpt1
ENSMUSG00000060131.12	52.4004466	0.02910407	0.00569434	24.5251914	7.33E-07	6.45E-06	Atp8b4
ENSMUSG00000060147.16	515.008986	0.02747565	0.00522652	25.2157027	5.13E-07	4.66E-06	Serpinb6a
ENSMUSG00000060152.15	553.568294	-0.0080284	0.00407987	3.93194792	0.04737704	0.11550907	Pop5
ENSMUSG00000060166.7	129.893265	0.00452935	0.00387235	1.35758574	0.24395633	0.41445226	Zdhhc8
ENSMUSG00000060176.5	5.57535326	0.0132562	0.01230961	1.0519698	0.30505386	0.4899224	Kif27
ENSMUSG00000060181.7	363.325341	-0.0080438	0.00328319	5.64628004	0.01749244	0.05097946	Slc35e3
ENSMUSG00000060183.8	80.2504295	0.01688011	0.00658795	6.28138193	0.01220129	0.03765764	Cxcl11
ENSMUSG00000060187.5	4.9091398	0.06993506	0.04929271	-0.6933678	1	1	Lrrc10
ENSMUSG00000060206.12	37.6177993	0.01622597	0.00735572	4.54090603	0.03309407	0.08619243	Zfp462
ENSMUSG00000060212.14	3.74106563	0.03377971	0.00974797	11.8246856	0.00058451	0.00274188	Pcnx2
ENSMUSG00000060216.16	300.10924	0.02385875	0.00405591	33.7150313	6.38E-09	7.94E-08	Arrb2
ENSMUSG00000060224.5	417.509394	0.01104384	0.00333863	10.6635775	0.00109266	0.00474525	Pyroxd2
ENSMUSG00000060227.16	120.826363	-0.0092349	0.00496186	3.19979445	0.07364753	0.1649934	Golm2
ENSMUSG00000060240.14	9.29544318	0.01381785	0.0107724	1.56405168	0.21107296	0.37318557	Cend1
ENSMUSG00000060244.3	73.8216192	0.0002385	0.00334035	0.0049773	0.94375591	0.99894211	Alyref2
ENSMUSG00000060245.6	1.07128761	0.00185913	0.04287424	0.02524389	0.87376085	0.96081512	Vmn1r228
ENSMUSG00000060257.3	7.1359749	-0.0086202	0.03825491	0.04121995	0.83911395	0.94004359	Scrt2
ENSMUSG00000060260.14	51.0603709	0.0121281	0.00411766	8.41574168	0.00371986	0.01372386	Pwwp2b
ENSMUSG00000060261.17	1513.23763	-0.0009101	0.00237695	0.14756671	0.70087163	0.85062771	Gtf2i
ENSMUSG00000060275.15	2.22449157	0.04259607	0.01547946	7.93427683	0.0048507	0.01723022	Nrg2
ENSMUSG00000060279.16	535.997598	-0.0014906	0.00274938	0.29434156	0.5874517	0.76423804	Ap2a1
ENSMUSG00000060288.14	154.760633	-0.0124084	0.00364166	11.6358983	0.00064691	0.00300119	Ppih
ENSMUSG00000060301.7	195.601355	0.00722703	0.0026502	7.3224518	0.00680984	0.02307715	2610008E11Rik
ENSMUSG00000060314.14	34.1822518	0.02269223	0.00740006	8.46104311	0.00362834	0.01343664	Zfp941
ENSMUSG00000060317.10	718.662384	0.05595311	0.00730725	50.9358702	9.54E-13	2.01E-11	Acnat2
ENSMUSG00000060336.7	39.8612656	0.01351423	0.00381928	12.1866497	0.00048133	0.00231355	Zfp937
ENSMUSG00000060371.13	84.6342994	-0.0051113	0.00552041	0.86980434	0.35100948	0.53944808	Caln1
ENSMUSG00000060373.17	2179.64519	-0.005818	0.00235351	5.92725244	0.01490847	0.04465157	Hnrnpc
ENSMUSG00000060376.8	3457.00309	-0.0148428	0.00288753	26.6045753	2.50E-07	2.41E-06	Bckdha
ENSMUSG00000060397.7	39.73512	0.01404289	0.00302787	21.4378332	3.65E-06	2.85E-05	Zfp128
ENSMUSG00000060402.9	6.48946908	-0.0036561	0.00968474	0.11462855	0.7349349	0.8733788	Chst8
ENSMUSG00000060407.7	8960.80457	-0.0070785	0.00518852	1.8897959	0.16922494	0.31499799	Cyp2a12
ENSMUSG00000060427.16	126.472265	0.00420424	0.00240977	3.02243033	0.08212032	0.17979468	Zfp868
ENSMUSG00000060429.13	1726.55441	0.00651999	0.00413311	2.36032762	0.12445605	0.24912335	Sntb1
ENSMUSG00000060441.17	72.9050803	0.03466966	0.00505161	44.6154803	2.40E-11	4.19E-10	Trim5
ENSMUSG00000060445.12	3.72043688	0.04629289	0.01810109	5.19193079	0.02269199	0.06320422	Sycp2
ENSMUSG00000060450.15	1167.39386	0.00367033	0.00192355	3.66102826	0.05569925	0.13191309	Rnf14
ENSMUSG00000060459.15	5284.22102	0.01407481	0.00482626	8.57391398	0.00341013	0.01274868	Kng2
ENSMUSG00000060461.6	0.632568	0.02415783	0.03755474	1.00914394	0.31510801	0.5005323	Dppa5a
ENSMUSG00000060470.8	85.1933497	-0.001319	0.00327804	0.16241228	0.68694523	0.84067297	Adgrg3
ENSMUSG00000060475.14	797.91606	0.00609741	0.00217495	7.69910089	0.00552483	0.01926219	Wtap
ENSMUSG00000060477.15	474.702907	-6.87E-05	0.00348281	0.00034895	0.98509623	1	Irak2
ENSMUSG00000060487.8	42.5122617	0.00733325	0.00556024	1.67989741	0.19493809	0.35100463	Samd5
ENSMUSG00000060503.7	0.83170651	0.00902001	0.03391132	0.21848989	0.64019297	0.80654664	Or2d2

ENSMUSG00000060509.6	14.7209685	0.0651598	0.00766426	67.9492726	1.68E-16	5.62E-15	Xcr1
ENSMUSG00000060510.15	529.729794	0.00382747	0.00305902	1.50663773	0.21965286	0.38435583	Zfp266
ENSMUSG00000060512.8	591.617965	-0.0015549	0.00267329	0.34490979	0.55700881	0.73900864	0610040J01Rik
ENSMUSG00000060519.12	201.831989	0.01419019	0.00506668	7.56671793	0.0059456	0.02049888	Tor3a
ENSMUSG00000060534.17	4.14513613	0.02204913	0.01859719	1.49203892	0.22190038	0.38709754	Dcc
ENSMUSG00000060538.15	562.24055	-0.0019335	0.00392187	0.24306212	0.62200319	0.79201896	Tmem219
ENSMUSG00000060548.14	127.538488	0.03674894	0.00415985	77.0307028	1.68E-18	7.21E-17	Tnfrsf19
ENSMUSG00000060550.18	1554.31557	0.02629324	0.00350249	54.4807462	1.57E-13	3.65E-12	H2-Q7
ENSMUSG00000060560.9	38.4290964	-0.1377017	0.00834044	248.405617	5.78E-56	1.70E-52	Ces4a
ENSMUSG00000060568.15	7.06167709	0.04174174	0.009054	19.3958585	1.06E-05	7.50E-05	Fam78b
ENSMUSG00000060572.18	3.49900298	0.01206343	0.01196096	1.092814	0.29584856	0.47885562	Mfap2
ENSMUSG00000060579.14	53.1477639	-0.0066916	0.00430163	2.48044667	0.11526951	0.23461743	Fhit
ENSMUSG00000060583.5	1.78597651	-0.0003114	0.02707328	-0.0041579	1	1	Or8b35
ENSMUSG00000060586.12	862.913248	0.08165496	0.00614167	156.823394	5.59E-36	2.31E-33	H2-Eb1
ENSMUSG00000060591.10	2595.42658	0.00318587	0.00322354	0.98345818	0.32134653	0.50763762	Ifitm2
ENSMUSG00000060594.7	18.6507882	0.00474491	0.00607912	0.59831504	0.43922164	0.63121384	Layn
ENSMUSG00000060600.16	201.598654	-0.0043957	0.00628533	0.4775666	0.48952662	0.67738192	Eno3
ENSMUSG00000060601.14	548.519041	-0.0034047	0.00261125	1.70677648	0.19140414	0.34620077	Nr1h2
ENSMUSG00000060613.11	10081.612	-0.008307	0.00618001	1.01641301	0.31337137	0.49876355	Cyp2c70
ENSMUSG00000060630.8	1.22535212	-0.002795	0.03919108	0.0306534	0.86101589	0.95374443	Zfp735
ENSMUSG00000060636.15	6518.50995	-0.010453	0.00417611	6.29164815	0.01213082	0.03747446	Rpl35a
ENSMUSG00000060639.6	44.214808	0.01722682	0.00525932	9.9243356	0.00163108	0.00674686	H4c9
ENSMUSG00000060657.9	1363.16755	-0.0034418	0.00291089	1.3791601	0.24024426	0.40988184	Marf1
ENSMUSG00000060671.13	59.4769653	0.02727871	0.00644919	16.8180426	4.11E-05	0.000258	Atp8b2
ENSMUSG00000060675.14	221.141238	0.00082799	0.0048079	0.02942415	0.86380335	0.95512006	Plaat3
ENSMUSG00000060678.6	51.1397251	-0.0177493	0.01202639	1.97454996	0.15996574	0.30145029	H4c3
ENSMUSG00000060679.15	377.433712	-0.0070198	0.00279108	6.42278322	0.01126655	0.0352805	Mrps9
ENSMUSG00000060681.16	290.727642	0.01039212	0.00331493	9.5081639	0.0020456	0.00824039	Slc9a6
ENSMUSG00000060703.13	6630.65192	0.00410095	0.00364506	1.25443443	0.26270721	0.4373694	Cd302
ENSMUSG00000060708.5	187.176686	-0.0100911	0.00280661	12.7992962	0.00034675	0.00173309	Bloc1s4
ENSMUSG00000060716.8	10.3750203	0.02715758	0.00894418	9.24993918	0.00235503	0.00929015	Plekhh1
ENSMUSG00000060724.3	6.69959496	-0.016887	0.02692086	0.48473231	0.48628656	0.67495762	Vmn1r47
ENSMUSG00000060733.14	1203.76814	-0.0041421	0.0028547	2.08923158	0.14834067	0.28503161	Ipmk
ENSMUSG00000060739.9	2684.38346	0.00137728	0.0032922	0.17409017	0.67650203	0.83279147	Nsa2
ENSMUSG00000060743.13	4311.11446	-0.0052343	0.00276057	3.5282845	0.06033018	0.1405658	H3f3a
ENSMUSG00000060771.16	46.2161482	-0.000325	0.0037161	0.00745093	0.93121302	0.99234583	Tsga10
ENSMUSG00000060780.3	1.44238539	0.0044544	0.02919277	0.03962493	0.84221573	0.94223647	Lrrtm1
ENSMUSG00000060791.16	134.533787	0.02169897	0.00499036	18.394715	1.80E-05	0.00012128	Gmfg
ENSMUSG00000060798.8	72.4168115	-0.0022253	0.00382574	0.34383094	0.55762623	0.73945536	Intu
ENSMUSG00000060802.9	29220.2909	0.01633398	0.00274022	35.2739709	2.86E-09	3.74E-08	B2m
ENSMUSG00000060803.7	25016.3094	-0.0544973	0.00498338	117.425179	2.32E-27	3.04E-25	Gstp1
ENSMUSG00000060807.8	4847.86717	0.02405602	0.00567057	17.8646602	2.37E-05	0.00015592	Serpina6
ENSMUSG00000060843.12	6.48555166	-0.0085408	0.01620998	0.17139992	0.67887064	0.83429778	Ctnna3
ENSMUSG00000060860.9	371.969412	-0.0149999	0.00394838	14.4461136	0.00014423	0.00079443	Ube2s
ENSMUSG00000060862.11	167.542402	-0.0101042	0.0042534	5.4823097	0.01920986	0.05503933	Zbtb40
ENSMUSG00000060882.6	9.19169435	0.03397989	0.01334335	7.50227582	0.00616211	0.02115206	Kcnd2
ENSMUSG00000060904.15	2789.58088	-0.0031201	0.0023928	1.67266034	0.19590258	0.35249911	Arl1
ENSMUSG00000060913.7	16.808938	0.01197378	0.04821617	-0.0727542	1	1	Trim55
ENSMUSG00000060923.6	93.3534111	-0.007375	0.00698494	1.03906412	0.30803923	0.49296549	Acyp2
ENSMUSG00000060924.16	9.04167569	0.04959904	0.01149131	16.8207925	4.11E-05	0.00025776	Csmd1
ENSMUSG00000060935.7	239.895544	-0.0026143	0.0026768	0.92937691	0.33502489	0.52209428	Tmem263
ENSMUSG00000060938.15	6468.25985	0.00168854	0.00358025	0.22292404	0.63682093	0.80373039	Rpl26
ENSMUSG00000060950.13	96.443021	-0.0132949	0.00431904	9.56663817	0.00198145	0.00802035	Trmt61a
ENSMUSG00000060961.17	1919.30244	0.01681278	0.00473296	11.9972482	0.00053279	0.00253253	Slc4a4
ENSMUSG00000060962.14	1.22591744	0.04231228	0.01717926	6.44459181	0.01112906	0.03493055	Dmkn
ENSMUSG00000060969.10	21.1296745	0.04678327	0.00859682	24.6820487	6.76E-07	6.00E-06	Irx1
ENSMUSG00000060981.8	109.584044	-0.0101106	0.00864211	1.26595207	0.26052744	0.43459936	H4c8
ENSMUSG00000060985.16	4.34841542	0.01551638	0.0244191	0.83154004	0.36182759	0.5514482	Tdrd5
ENSMUSG00000060988.13	3.90627283	0.03711306	0.02051064	4.33774376	0.03727629	0.09486389	Galnt13
ENSMUSG00000060992.10	2476.39417	0.00134241	0.00246977	0.29635927	0.58617389	0.76312289	Copz1
ENSMUSG00000061013.7	21.5000404	0.0157723	0.00805417	3.86273185	0.04936988	0.11934386	Mkx

ENSMUSG00000061024.9	349.641108	-0.0038176	0.00256578	2.18422143	0.13943156	0.27194515	Rrs1
ENSMUSG00000061028.8	181.520803	-0.0065978	0.00367893	3.23927326	0.07189252	0.1617839	Clasrp
ENSMUSG00000061032.10	1201.82388	0.00066546	0.00179632	0.13730604	0.710974	0.85726062	Rrp1
ENSMUSG00000061039.6	7.33295031	0.03009142	0.00785047	13.5845547	0.00022805	0.00119797	Or8b53
ENSMUSG00000061046.10	80.0965788	0.00941455	0.00449113	4.46633188	0.03456913	0.08943155	Haghl
ENSMUSG00000061048.9	11.9999666	0.02806584	0.0064974	18.3121156	1.88E-05	0.00012611	Cdh3
ENSMUSG00000061068.8	1.94866466	0.05022217	0.02250808	3.9269452	0.04751819	0.11574553	Mcpt4
ENSMUSG00000061079.15	131.061125	0.00490004	0.00257542	3.51433519	0.06084006	0.14155802	Zfp143
ENSMUSG00000061080.12	25.8151938	0.00337511	0.01165004	0.04463877	0.83266965	0.93574168	Lsamp
ENSMUSG00000061086.13	82.1849071	-0.009488	0.00848102	1.20484717	0.27235503	0.44968609	Myl4
ENSMUSG00000061099.13	6.6172401	0.03049968	0.01076591	7.69642698	0.00553302	0.0192822	Gapdhs
ENSMUSG00000061100.6	3.56142974	0.03731454	0.01574456	5.25361311	0.02190125	0.06134951	Retnla
ENSMUSG00000061104.6	272.921743	-0.0055222	0.00389148	2.06261494	0.15095094	0.28877677	Sap18b
ENSMUSG00000061111.9	344.540372	0.00485676	0.00482636	1.0148379	0.31374661	0.49912502	Mcrip1
ENSMUSG00000061118.9	316.569127	-0.0035405	0.0023238	2.34545591	0.1256488	0.25108468	Dnajc30
ENSMUSG00000061119.8	168.48482	0.02112228	0.00343474	36.8174193	1.30E-09	1.77E-08	Prcp
ENSMUSG00000061126.6	7.17361652	-0.0312742	0.00848305	12.3994097	0.00042947	0.00208855	Cyp4f39
ENSMUSG00000061130.14	2487.77776	-0.0057117	0.00204168	7.71590323	0.00547365	0.01911764	Ppm1b
ENSMUSG00000061132.14	59.9672358	0.06611917	0.00580091	114.987236	7.92E-27	9.50E-25	Blnk
ENSMUSG00000061136.17	2628.07512	0.00659639	0.00416754	2.4795504	0.11533522	0.23471065	Prpf40a
ENSMUSG00000061143.16	74.4743971	0.00376933	0.00447272	0.71286445	0.39849452	0.5902671	Maml3
ENSMUSG00000061171.16	2.33198167	-0.001339	0.01306756	0.00178336	0.96631543	1	Slc38a11
ENSMUSG00000061175.13	654.7864	0.01124374	0.00413819	7.3798301	0.00659594	0.022462	Fnip2
ENSMUSG00000061186.16	13.3725844	0.02276951	0.00918459	5.84961255	0.01558048	0.04629976	Sfmbt2
ENSMUSG00000061207.12	182.000333	-0.0073156	0.00376797	3.63402128	0.05660988	0.13370725	Stk19
ENSMUSG00000061232.17	20858.0881	0.01366797	0.00322701	17.7898312	2.47E-05	0.00016149	H2-K1
ENSMUSG00000061244.15	543.417841	-0.003621	0.00289584	1.54666663	0.21362821	0.3764108	Exoc5
ENSMUSG00000061273.4	498.937047	-0.0053124	0.0022691	5.37766253	0.02039619	0.05791771	Mmgt1
ENSMUSG00000061286.8	416.279672	-0.0136457	0.00312493	19.4524244	1.03E-05	7.31E-05	Exosc5
ENSMUSG00000061288.14	1077.62197	0.00045782	0.00387471	0.01373406	0.90670758	0.97928404	Taok3
ENSMUSG00000061292.11	667.541618	0.01909693	0.00535865	13.279196	0.00026837	0.00138354	Cyp3a59
ENSMUSG00000061298.15	1.56128192	0.0034202	0.02641567	0.01110633	0.91606906	0.98407746	Agbl4
ENSMUSG00000061306.17	1754.60559	-0.0051903	0.00279077	3.49550579	0.06153561	0.14281008	Slc38a10
ENSMUSG00000061311.7	1.01576046	0.01970085	0.0247337	1.18145989	0.27705899	0.45579364	Rag1
ENSMUSG00000061313.11	439.185359	-0.0055054	0.00331861	2.67369111	0.10201917	0.21348808	Ddhd2
ENSMUSG00000061315.15	5524.04133	-0.003062	0.00295503	1.07711104	0.29934474	0.48328735	Naca
ENSMUSG00000061322.16	11.7165684	-0.0712545	0.01156112	37.6002614	8.68E-10	1.21E-08	Dnai1
ENSMUSG00000061353.12	5936.94311	0.0055315	0.00228957	5.82735752	0.01577881	0.04678302	Cxcl12
ENSMUSG00000061356.14	1.13576163	0.01852104	0.01314697	2.04727428	0.15247896	0.2909919	Nuggc
ENSMUSG00000061360.9	418.641017	-0.0116627	0.00301635	14.9704693	0.00010921	0.00061843	Phf5a
ENSMUSG00000061371.8	97.4897498	-0.003362	0.00324117	1.06695198	0.30163494	0.48597182	Zfp873
ENSMUSG00000061374.15	250.870459	-0.0041561	0.00214557	3.7818516	0.0518113	0.12412608	Fiz1
ENSMUSG00000061376.3	1.4859277	-0.000389	0.03279903	-0.0029731	1	1	Vmn1r219
ENSMUSG00000061388.12	1.15945576	-0.0013485	0.03915042	-0.005519	1	1	Csn1s2b
ENSMUSG00000061393.15	83.6482251	0.00382152	0.00584986	0.3955884	0.52937643	0.71394707	Acvr2b
ENSMUSG00000061397.9	2.25372004	0.01041905	0.01079918	0.88525747	0.34676573	0.53520801	Krt79
ENSMUSG00000061410.7	588.227688	-0.0011619	0.00370957	0.09736739	0.75501204	0.88675002	Zcchc14
ENSMUSG00000061411.13	37.4949049	0.01295924	0.00528681	5.92493743	0.01492807	0.04469325	Nol4l
ENSMUSG00000061414.9	13.8641344	0.04827773	0.0068397	46.3171008	1.01E-11	1.86E-10	Cracr2a
ENSMUSG00000061436.16	766.757387	-0.0036486	0.00486204	0.5579285	0.45509609	0.64689304	Hipk2
ENSMUSG00000061451.14	13.1634904	0.01134041	0.00962275	1.37910656	0.24025339	0.40988184	Tmem151a
ENSMUSG00000061455.14	544.05836	-0.0027255	0.00272875	1.00564353	0.31594878	0.50147833	Stx17
ENSMUSG00000061458.10	114.835018	-0.0059886	0.00281585	4.53030798	0.03329962	0.08663191	Nol10
ENSMUSG00000061461.12	571.305875	-0.011428	0.00375141	9.29152686	0.00230216	0.00911105	Smim20
ENSMUSG00000061462.19	91.49119	0.00348685	0.0150337	0.01082553	0.91713299	0.98472703	Obscn
ENSMUSG00000061474.12	247.027692	-0.0129749	0.00349294	14.038107	0.00017914	0.00096932	Mrps36
ENSMUSG00000061477.5	6504.22177	-0.00141	0.00362126	0.15296959	0.69571351	0.84675595	Rps7
ENSMUSG00000061479.16	447.876751	-0.0021934	0.00379307	0.32825784	0.56668674	0.74657172	Snrpa
ENSMUSG00000061482.8	58.1495226	0.01500887	0.0117859	1.54646688	0.21365778	0.37642041	H4c4
ENSMUSG00000061489.4	1.06089541	0.00807438	0.0276884	0.180719	0.67075607	0.82877326	Or13a19
ENSMUSG00000061517.9	4.76743742	0.03302484	0.03819053	1.04765021	0.30604889	0.49101891	Sox21

ENSMUSG00000061518.12	3230.09311	-0.0182015	0.00389608	21.7635241	3.08E-06	2.44E-05	Cox5b
ENSMUSG00000061524.9	1.81915754	-0.0078455	0.03458233	0.07238945	0.78788899	0.90888925	Zic2
ENSMUSG00000061527.8	1.54127981	0.04159755	0.04076422	0.27495066	0.60003	0.77411156	Krt5
ENSMUSG00000061533.17	42.7031075	0.01244254	0.00685033	3.23164734	0.072228	0.16238999	Cep128
ENSMUSG00000061535.12	27.7952807	0.03134774	0.0060654	25.117037	5.40E-07	4.88E-06	C1qtnf7
ENSMUSG00000061536.14	215.632824	0.00240908	0.00437398	0.29899238	0.5845148	0.76167768	Sec22c
ENSMUSG00000061540.4	1529.39267	0.04666132	0.01267784	8.64055445	0.00328761	0.01235867	Orm2
ENSMUSG00000061544.14	123.998215	0.0121199	0.00382367	9.6387716	0.00190513	0.00774649	Zfp229
ENSMUSG00000061559.16	915.200244	-0.0052114	0.00197076	7.06758357	0.0078491	0.02597572	Skic8
ENSMUSG00000061576.16	28.4923278	0.0173382	0.01393232	1.65529088	0.19824034	0.35575566	Dpp6
ENSMUSG00000061577.13	8.50477103	0.06944807	0.00859742	59.0109548	1.57E-14	4.20E-13	Adgrg5
ENSMUSG00000061578.10	33.6125503	0.04705591	0.00742427	38.1416495	6.58E-10	9.39E-09	Ksr2
ENSMUSG00000061589.15	370.276276	-0.0140574	0.00347633	16.3834574	5.17E-05	0.00031579	Dot1l
ENSMUSG00000061601.16	72.7718416	0.04367249	0.00653039	37.6215259	8.59E-10	1.20E-08	Pclo
ENSMUSG00000061602.4	4.39967161	-0.0010384	0.01808974	-0.011547	1	1	Vmn1r78
ENSMUSG00000061603.9	69.0665448	0.02628342	0.00953595	7.03318264	0.0080013	0.02639792	Akap6
ENSMUSG00000061607.16	245.995006	0.00826605	0.0038242	4.54554492	0.03300452	0.08598746	Mdc1
ENSMUSG00000061613.14	776.124667	-0.0039707	0.00308773	1.64487798	0.1996575	0.35783601	U2af1
ENSMUSG00000061615.3	5.19124164	0.00912128	0.02513425	0.22005081	0.6390012	0.80540172	H2ac4
ENSMUSG00000061650.7	128.565175	-0.0006698	0.0022952	0.08452123	0.77126138	0.8974886	Med9
ENSMUSG00000061653.5	2.34151183	0.00170455	0.01937195	0.04176534	0.83806774	0.93917284	Vmn1r46
ENSMUSG00000061665.8	878.049079	0.01266637	0.00278041	20.3104183	6.58E-06	4.86E-05	Cd2ap
ENSMUSG00000061666.7	34.6703567	0.03249038	0.00618163	26.6927224	2.39E-07	2.32E-06	Gdpd1
ENSMUSG00000061689.16	308.818726	0.0089343	0.00333755	7.18539712	0.00734993	0.02460338	Dlgap4
ENSMUSG00000061702.11	4.54311366	0.01035812	0.01316188	0.67548355	0.4111463	0.6026909	Tmem91
ENSMUSG00000061718.13	99.0564708	0.00133203	0.00539875	0.06001245	0.80647627	0.91944843	Ppp1r1b
ENSMUSG00000061723.20	3.43537164	0.02642238	0.02093172	1.98861737	0.15848552	0.29940244	Tnnt3
ENSMUSG00000061731.10	705.7322	-0.000721	0.0028166	0.06549025	0.79801984	0.91515701	Ext1
ENSMUSG00000061740.9	3297.01839	-0.006654	0.00364346	3.34844291	0.06726862	0.15332408	Cyp2d22
ENSMUSG00000061742.13	13.0323413	0.03998697	0.02369484	4.15622406	0.04148178	0.10364293	Slc22a12
ENSMUSG00000061751.17	501.174905	0.00214556	0.00291	0.55580697	0.45595463	0.64768405	Kalrn
ENSMUSG00000061755.11	1405.91821	0.00284541	0.0041357	0.46507211	0.49526301	0.6825167	Bod1l
ENSMUSG00000061758.14	48.2829286	0.03403858	0.00554532	35.5601551	2.47E-09	3.26E-08	Akr1b10
ENSMUSG00000061759.16	282.835903	-0.0060046	0.00227852	6.97722663	0.00825534	0.02711846	Armt1
ENSMUSG00000061762.13	8.58103097	0.01722741	0.01924365	1.06765076	0.30147669	0.48589785	Tac1
ENSMUSG00000061778.11	646.339225	0.01401257	0.00257958	28.6624381	8.62E-08	9.05E-07	Mospd2
ENSMUSG00000061780.7	46.5526309	0.01738565	0.01432957	0.7668423	0.38119569	0.57266761	Cfd
ENSMUSG00000061787.16	4033.53553	-0.0024453	0.00321953	0.58185997	0.44558419	0.63779274	Rps17
ENSMUSG00000061808.5	87409.9663	-0.0105527	0.00577403	3.37785486	0.06607801	0.15120945	Ttr
ENSMUSG00000061815.12	9.46083693	-0.010979	0.00780343	1.84416379	0.17446371	0.32268672	Rufy4
ENSMUSG00000061816.16	9.15649418	-0.001397	0.0240746	0.19024777	0.66271048	0.82289764	Myl1
ENSMUSG00000061825.11	906.656882	-0.0478171	0.00609792	56.3751067	5.99E-14	1.47E-12	Ces2c
ENSMUSG00000061829.5	1.1625787	0.00543087	0.03345375	0.10386113	0.74724442	0.88135755	Vmn1r214
ENSMUSG00000061838.8	4837.39841	0.00379992	0.00224736	2.82877746	0.09258882	0.19786173	Suclg2
ENSMUSG00000061859.18	221.635249	0.00494534	0.00351319	1.95975405	0.16153962	0.30373673	Patj
ENSMUSG00000061878.17	12.6358539	0.03335206	0.008421	14.5572522	0.00013596	0.00075208	Sphk1
ENSMUSG00000061882.14	51.080313	-0.0135097	0.00468572	8.03581324	0.00458614	0.01641044	Ccdc62
ENSMUSG00000061887.15	848.87972	0.00047923	0.00424119	0.01278954	0.90995855	0.98081708	Ssbp3
ENSMUSG00000061894.16	49.4142027	-0.0046248	0.00404533	1.27846738	0.25818419	0.43160889	Zscan20
ENSMUSG00000061898.11	87.5475732	0.01013539	0.00276605	13.4094264	0.00025036	0.00130014	Rbak
ENSMUSG00000061904.13	4810.38285	-0.0128762	0.00258496	24.902804	6.03E-07	5.42E-06	Slc25a3
ENSMUSG00000061906.6	347.077512	-0.132706	0.00950762	155.037838	1.37E-35	5.40E-33	Ugt2b38
ENSMUSG00000061911.16	15.0906523	0.00514368	0.01790886	0.22994094	0.63156762	0.79957992	Myt1l
ENSMUSG00000061923.5	3.7837133	-0.0642673	0.06432719	0.29970304	0.58406865	0.76134869	Odf1
ENSMUSG00000061928.7	1.04080392	0.02807819	0.01521416	3.4943681	0.0615779	0.14286607	Dsg1b
ENSMUSG00000061947.11	4475.78946	0.00201063	0.00543709	0.13271133	0.71563723	0.86003362	Serpina10
ENSMUSG00000061950.18	396.285139	-0.0028016	0.00271132	1.05951154	0.30332662	0.4880097	Ppp4r1
ENSMUSG00000061959.14	2795.56419	-0.0060737	0.00520341	1.31710726	0.25111187	0.42286332	Ces1e
ENSMUSG00000061972.4	1.20044206	0.02837709	0.01585354	4.29056452	0.03832444	0.09704849	Or1o4
ENSMUSG00000061979.9	273.538699	-0.008049	0.0025928	9.58681466	0.0019598	0.00794223	Rcc1l
ENSMUSG00000061981.15	778.400582	0.00149699	0.00219246	0.46932143	0.49329951	0.68080528	Flot2

ENSMUSG00000061983.8	8514.63736	-0.0009934	0.00444863	0.04967016	0.82363822	0.92984544	Rps12
ENSMUSG00000061991.3	2.17285917	0.02880737	0.02475344	2.6101504	0.10618169	0.21987721	H2ac10
ENSMUSG00000062006.13	5613.05214	-0.0055469	0.00431304	1.65098973	0.19882428	0.35658653	Rpl34
ENSMUSG00000062007.14	2.51744097	0.06171805	0.01027291	33.6770746	6.51E-09	8.09E-08	Hsh2d
ENSMUSG00000062012.15	18.2024692	0.0128165	0.00639759	4.31703918	0.03773252	0.09584914	Zfp13
ENSMUSG00000062014.15	829.916384	0.00389914	0.00273204	1.98817833	0.15853148	0.29946528	Gmfb
ENSMUSG00000062017.16	12.5008056	0.01065624	0.01090668	0.83936734	0.35957777	0.54879005	Abca14
ENSMUSG00000062028.9	2.38407444	-0.0369641	0.02736343	1.70354238	0.19182536	0.34680308	Irgc1
ENSMUSG00000062031.14	504.921144	-0.0070498	0.00241361	8.59576407	0.00336946	0.01262663	Pgghg
ENSMUSG00000062040.15	102.2309	-0.0044885	0.00327385	1.89226192	0.16894702	0.31467933	Zfp27
ENSMUSG00000062044.17	4.5398653	0.02189277	0.01204116	3.59044478	0.05811269	0.13646414	Lmtk3
ENSMUSG00000062054.8	1608.44826	-0.0001654	0.00341644	0.00234963	0.96133931	1	Iah1
ENSMUSG00000062061.4	17.1349262	-0.0583856	0.01054557	21.9471095	2.80E-06	2.23E-05	Obp2a
ENSMUSG00000062070.13	5789.53674	-0.0084422	0.00244203	12.1264769	0.00049711	0.00238358	Pgk1
ENSMUSG00000062074.8	4.27244983	-0.0131013	0.00785132	2.6678885	0.10239179	0.21392231	Ccn6
ENSMUSG00000062075.14	150.229697	-0.0081065	0.00292102	7.8363249	0.00512066	0.01804985	Lmnb2
ENSMUSG00000062077.15	25.7186922	-0.082819	0.03871783	0.92530705	0.33608536	0.52336652	Trim54
ENSMUSG00000062078.16	1603.44591	0.00392607	0.00309311	1.57249721	0.20984473	0.37157641	Qki
ENSMUSG00000062082.17	9.73152325	0.07614053	0.00803089	82.1471209	1.26E-19	6.35E-18	Cd200r4
ENSMUSG00000062098.12	146.264853	0.00961048	0.00258416	13.7260173	0.0002115	0.00112127	Btbd3
ENSMUSG00000062101.13	96.2853923	0.0006384	0.0027105	0.05550115	0.8137533	0.92377077	Zfp119b
ENSMUSG00000062110.15	231.828034	-0.0049268	0.00416235	1.3887798	0.23861127	0.40799517	Scfd2
ENSMUSG00000062115.16	202.466581	-0.0068211	0.00315007	4.59586809	0.03204911	0.08399003	Rai1
ENSMUSG00000062116.11	121.637988	0.0004936	0.00264515	0.03495305	0.85169408	0.94848938	Zfp954
ENSMUSG00000062127.12	132.671912	0.02560254	0.00429696	33.5726497	6.87E-09	8.50E-08	Cttnbp2nl
ENSMUSG00000062128.9	1.20394895	0.00629587	0.02872556	0.08418442	0.77170491	0.89772784	Or1e1
ENSMUSG00000062151.14	13.0971469	0.01628096	0.02025588	0.83816525	0.35992204	0.54920885	Unc13c
ENSMUSG00000062154.15	1.37705816	-0.0223427	0.06653202	0.20393328	0.65156457	0.81397256	Tex33
ENSMUSG00000062157.7	4.75678599	0.0496787	0.00899838	28.2289335	1.08E-07	1.11E-06	Ifnlr1
ENSMUSG00000062169.14	379.354021	-0.0030309	0.00378295	0.63657676	0.42495313	0.61656893	Cnih4
ENSMUSG00000062170.13	13.2938206	0.00375217	0.01467399	0.06204655	0.80329004	0.91779337	Fmr1nb
ENSMUSG00000062175.14	12.6531603	0.03323905	0.00637728	25.2769041	4.97E-07	4.53E-06	Tgif2
ENSMUSG00000062181.15	6428.90414	-0.0547079	0.00594872	80.0246869	3.70E-19	1.72E-17	Ces3b
ENSMUSG00000062184.12	8.52605908	0.05314378	0.01642318	9.26861197	0.00233114	0.00920823	Hs6st2
ENSMUSG00000062186.3	1.32720481	0.00581154	0.0343061	0.06522567	0.79841945	0.91521751	Or1e35
ENSMUSG00000062190.13	129.726978	0.00075204	0.00235839	0.09986553	0.75199106	0.88492047	Lancl2
ENSMUSG00000062198.6	124.858314	0.0015431	0.00264081	0.34391742	0.55757669	0.73944357	2700097O09Rik
ENSMUSG00000062200.15	1.42205321	0.00838277	0.03199023	0.13493923	0.71336486	0.85867547	Vmn2r7
ENSMUSG00000062202.15	344.267248	0.00793213	0.00400674	3.78424089	0.05173737	0.12398676	Btbd9
ENSMUSG00000062203.16	2043.39477	-0.0107953	0.00385809	7.73596595	0.00541316	0.01892319	Gspt1
ENSMUSG00000062209.16	75.7315037	-0.0003146	0.00666804	0.00210243	0.96342798	1	Erbb4
ENSMUSG00000062210.15	141.170046	0.05732815	0.0057661	89.1578193	3.65E-21	2.15E-19	Tnfaip8
ENSMUSG00000062232.15	376.971519	0.01597192	0.00300839	27.8767397	1.29E-07	1.32E-06	Rapgef2
ENSMUSG00000062234.15	1115.3497	-0.0019224	0.00219963	0.75693687	0.38428883	0.57592046	Gak
ENSMUSG00000062248.6	362.194623	-0.0089041	0.00357508	6.2231316	0.01260919	0.03866359	Cks2
ENSMUSG00000062257.14	17.9918916	0.02849566	0.01388512	3.92637374	0.04753434	0.11576096	Opcml
ENSMUSG00000062270.15	3827.8072	0.00148267	0.00215833	0.47085813	0.49259265	0.6802676	Morf4l1
ENSMUSG00000062296.9	21.9680839	0.03598118	0.01330999	6.59247226	0.01024109	0.03253586	Trank1
ENSMUSG00000062300.15	855.988731	-0.0221671	0.00276852	63.5539571	1.56E-15	4.69E-14	Nectin2
ENSMUSG00000062309.9	2.15951918	0.03340231	0.01211321	7.70722305	0.00550003	0.01919274	Rpp25
ENSMUSG00000062310.8	1.80313117	0.03732681	0.01367559	7.08818039	0.0077594	0.02572101	Glrp1
ENSMUSG00000062312.6	31.0395562	0.01268273	0.00490728	6.77028768	0.00926879	0.02997171	Erbb2
ENSMUSG00000062328.9	11626.265	-0.0016015	0.00393258	0.16519678	0.68441635	0.83870962	Rpl17
ENSMUSG00000062329.5	2.97488092	0.00959877	0.019778	-0.4317578	1	1	Cytl1
ENSMUSG00000062345.11	2.72505947	0.08651843	0.01566677	26.1196002	3.21E-07	3.03E-06	Serpinb2
ENSMUSG00000062352.16	396.522632	-0.0058903	0.00367729	2.55233175	0.11013161	0.22645963	Itgb1bp1
ENSMUSG00000062373.9	289.72939	0.01821512	0.00433901	16.5927921	4.63E-05	0.00028716	Tmem65
ENSMUSG00000062376.8	222.040337	0.00582562	0.00466967	1.54443686	0.21395859	0.37685217	Borcs7
ENSMUSG00000062380.5	9.82011906	0.05112504	0.01418684	12.5584565	0.00039442	0.00194049	Tubb3
ENSMUSG00000062382.10	915.771974	0.01165291	0.00534318	4.65554389	0.03095342	0.08162651	Ftl1-ps1
ENSMUSG00000062397.9	2068.77596	-0.0089618	0.0022597	15.5428347	8.07E-05	0.000471	Zfp706

ENSMUSG00000062410.15	3369.59628	-0.0278636	0.00521138	28.7177685	8.37E-08	8.81E-07	Hsd3b3
ENSMUSG00000062421.14	202.8341	0.00729605	0.0034484	4.42311228	0.03545531	0.09120395	Arf2
ENSMUSG00000062432.5	2.94677005	0.00568326	0.01198527	0.32002336	0.57159361	0.75098976	Cyp26c1
ENSMUSG00000062438.5	1.10196366	-0.0217421	0.05290238	0.13885308	0.70942388	0.85624353	Adam1b
ENSMUSG00000062444.16	4.10554808	0.02097106	0.02100896	1.53718037	0.21503799	0.37807574	Ap3b2
ENSMUSG00000062456.5	235.189877	0.01165657	0.00654927	3.12098728	0.07728994	0.17142755	Rpl9-ps6
ENSMUSG00000062480.13	4799.02482	-0.002774	0.00396133	0.48402749	0.48660367	0.67509437	Acat3
ENSMUSG00000062488.10	82.6992559	0.02538693	0.0051137	25.6687948	4.05E-07	3.76E-06	Ifit3b
ENSMUSG00000062510.13	15.8236266	0.02849449	0.00991644	7.27999957	0.00697266	0.02355781	Nsl1
ENSMUSG00000062515.4	888.911316	0.01270389	0.00640648	3.86880433	0.04919155	0.11898595	Fabp4
ENSMUSG00000062519.14	192.72944	-0.0002122	0.00356108	0.00353782	0.9525701	1	Zfp398
ENSMUSG00000062524.5	4.48506678	0.05638893	0.0085773	39.375444	3.50E-10	5.16E-09	Ncr1
ENSMUSG00000062526.5	224.627279	0.00482048	0.00268082	3.22651436	0.07245475	0.16280059	Mppe1
ENSMUSG00000062542.12	40.9744147	0.02512925	0.00617903	16.4429661	5.01E-05	0.00030714	Syt9
ENSMUSG00000062545.5	174.200652	0.03130578	0.00476595	40.8904	1.61E-10	2.50E-09	Tlr12
ENSMUSG00000062563.16	78.5640864	-0.004674	0.00457385	1.01285871	0.31421895	0.49957319	Cys1
ENSMUSG00000062580.10	673.734511	-0.0176495	0.00306746	33.3662872	7.63E-09	9.40E-08	Timm17a
ENSMUSG00000062585.12	72.5158447	0.01470574	0.00320078	20.4597567	6.09E-06	4.53E-05	Cnr2
ENSMUSG00000062590.14	96.0139224	0.00303021	0.00413515	0.52498343	0.4687236	0.65982183	Armc9
ENSMUSG00000062591.6	33.0752405	-0.0152163	0.00873983	2.95564955	0.08557791	0.18591145	Tubb4a
ENSMUSG00000062593.18	81.8875303	0.07719626	0.0096404	57.1450641	4.05E-14	1.02E-12	Gm49339
ENSMUSG00000062604.12	434.532621	0.0058473	0.00303756	3.67185589	0.05533853	0.13120372	Srpk2
ENSMUSG00000062609.15	76.4453502	0.01192556	0.00780131	2.15788159	0.14184012	0.27550367	Kcnj15
ENSMUSG00000062619.7	587.319807	0.00247234	0.00356571	0.47946136	0.4886664	0.67691923	2310039H08Rik
ENSMUSG00000062621.4	0.98183119	-0.0038419	0.03519099	-0.0257683	1	1	Or8b47
ENSMUSG00000062624.6	5637.23516	-0.0102092	0.00534744	3.41502984	0.06460525	0.14848756	Cyp2c67
ENSMUSG00000062627.10	370.72336	0.00317971	0.00313549	1.0033145	0.31650982	0.50196347	Mysm1
ENSMUSG00000062646.13	400.953566	-0.0001366	0.00298689	0.0020469	0.96391389	1	Ganc
ENSMUSG00000062647.17	7131.06498	-0.0030663	0.00265678	1.34779069	0.24566472	0.4166739	Rpl7a
ENSMUSG00000062649.7	1.29452108	-0.0096833	0.03612346	0.02101715	0.88473223	0.96658844	Or8g20
ENSMUSG00000062661.7	21.2555796	0.04954148	0.00917515	26.7718015	2.29E-07	2.24E-06	Ncs1
ENSMUSG00000062683.12	1600.26343	-0.0119626	0.00388144	9.62475138	0.00191972	0.00779859	Atp5mc2
ENSMUSG00000062691.12	410.043645	-0.0042533	0.00355134	1.4546852	0.22777766	0.39424406	Cebpzos
ENSMUSG00000062694.8	3.67770659	-0.0108236	0.02520735	0.18050492	0.67093968	0.82882644	Cav3
ENSMUSG00000062713.9	168.02517	0.03977721	0.03778099	-0.3196338	1	1	Sim2
ENSMUSG00000062727.5	6.35849497	0.00859809	0.01765939	0.21343548	0.64408793	0.8095203	H2bc12
ENSMUSG00000062729.13	372.707485	-0.0066565	0.00280267	5.66854592	0.01727177	0.05042355	Ppox
ENSMUSG00000062732.11	1.04240161	-0.0225517	0.12103782	0.04083628	0.83985427	0.94036567	Lypd4
ENSMUSG00000062743.10	25.7272084	0.05686672	0.00445891	151.586048	7.80E-35	2.97E-32	Zfp677
ENSMUSG00000062753.15	76.2996996	0.02828439	0.00528364	28.1236694	1.14E-07	1.17E-06	Smim29
ENSMUSG00000062757.3	1.12700439	-0.008559	0.04255064	-0.0229894	1	1	Or4c11
ENSMUSG00000062760.12	4.48902688	0.01648298	0.01055222	2.41851002	0.11990893	0.24207584	Shisal1
ENSMUSG00000062761.8	230.861529	0.00621335	0.00292388	4.45713943	0.03475565	0.08981388	Zfp512
ENSMUSG00000062762.18	3456.46647	-0.0029629	0.0019208	2.38045365	0.12286186	0.24674897	Ei24
ENSMUSG00000062782.5	2.32664471	0.00128426	0.04179091	0.01532383	0.90148198	0.97646956	Or12j2
ENSMUSG00000062783.3	85.4831461	0.1066252	0.00468716	426.308514	1.03E-94	2.44E-90	Csprs
ENSMUSG00000062785.15	97.9433136	-0.0287229	0.00566593	25.5893178	4.22E-07	3.91E-06	Kcnc3
ENSMUSG00000062794.9	6.02808787	-0.0009618	0.00793544	0.01490418	0.90283355	0.97702372	Zfp599
ENSMUSG00000062797.15	319.189428	-0.0217596	0.00341192	39.5001898	3.28E-10	4.85E-09	Hikeshi
ENSMUSG00000062818.8	29.2190158	0.01072004	0.01119344	1.19675108	0.273972	0.45169185	Vmn1r51
ENSMUSG00000062822.9	470.256347	5.99E-05	0.00200289	0.00089634	0.97611576	1	4833420G17Rik
ENSMUSG00000062825.16	3091.99633	0.03190926	0.00417011	55.5254755	9.23E-14	2.20E-12	Actg1
ENSMUSG00000062859.16	2.56002227	-0.0294947	0.01868244	2.2402597	0.1344584	0.26449099	Tcp11
ENSMUSG00000062861.9	39.758062	0.02332619	0.00549312	17.9709714	2.24E-05	0.00014823	Zfp28
ENSMUSG00000062866.17	249.337756	0.0078602	0.00390142	4.00904095	0.04525689	0.11121368	Phactr2
ENSMUSG00000062867.14	663.257591	-0.0013249	0.00330231	0.16149167	0.68778687	0.8413393	Impdh2
ENSMUSG00000062892.5	1.22372891	-0.0011018	0.03912723	3.14E-05	0.99552735	1	Or5b98
ENSMUSG00000062901.4	1570.62806	-0.0082815	0.00370479	4.82992064	0.02796992	0.07508519	Klhl24
ENSMUSG00000062906.13	161.877087	-0.0007861	0.00267936	0.08667167	0.76845186	0.89579922	Hdac10
ENSMUSG00000062908.13	8755.3309	-0.002132	0.00445207	0.21699752	0.64133724	0.80765552	Acadm
ENSMUSG00000062914.4	1.12998853	-0.001499	0.03882731	-0.0098069	1	1	Or6c207

ENSMUSG00000062929.9	1175.62797	-0.0018339	0.00307524	0.34976762	0.5542447	0.73646807	Cfl2
ENSMUSG00000062931.16	121.218595	-0.0028956	0.0029288	0.95717749	0.32789903	0.51493619	Zfp938
ENSMUSG00000062937.8	370.606767	-0.0020127	0.00195278	1.07617263	0.29955535	0.48356118	Mtap
ENSMUSG00000062939.12	14.166346	0.0612156	0.00830698	46.8720166	7.58E-12	1.43E-10	Stat4
ENSMUSG00000062944.5	73.6967749	0.0034568	0.00292884	1.38063448	0.2399931	0.40961555	9130023H24Rik
ENSMUSG00000062949.14	3623.90829	-0.004988	0.00420271	1.3998822	0.2367433	0.40581899	Atp11c
ENSMUSG00000062960.11	933.43532	-0.0048504	0.00361369	1.75539314	0.18520017	0.33777693	Kdr
ENSMUSG00000062963.11	1636.99375	-0.0044139	0.00279313	2.51400641	0.11283873	0.23065537	Ufc1
ENSMUSG00000062980.16	933.42164	-0.0071109	0.00319688	4.95484762	0.02601762	0.07078503	Cped1
ENSMUSG00000062981.6	869.162775	-0.012038	0.00361911	11.068021	0.00087829	0.00391448	Mrpl42
ENSMUSG00000062991.10	32.6205342	0.06479965	0.00913982	44.3501265	2.75E-11	4.75E-10	Nrg1
ENSMUSG00000062995.13	35.633739	0.00177388	0.00503813	0.12053726	0.72845252	0.86883849	Ica1
ENSMUSG00000062997.7	8005.02405	-0.0070316	0.00440945	2.51336079	0.11288496	0.23069965	Rpl35
ENSMUSG00000063011.8	31.6161979	-0.0211308	0.00867151	5.94190262	0.01478505	0.04436079	Msln
ENSMUSG00000063015.13	2724.01292	0.00501476	0.0021086	5.55152849	0.01846456	0.0532793	Ccni
ENSMUSG00000063019.6	138.85126	-0.0006488	0.00286984	0.05149187	0.82048735	0.92810967	Manbal
ENSMUSG00000063021.4	2.32861883	-0.0024727	0.03980421	0.00395036	0.94988442	1	H2ac15
ENSMUSG00000063047.10	161.132276	-0.0051885	0.00361274	2.03651865	0.15356074	0.29260653	Zfp780b
ENSMUSG00000063049.5	168.544777	-0.0010353	0.0028505	0.13311447	0.71522446	0.85984354	Ing2
ENSMUSG00000063052.10	248.147059	0.00442224	0.00281967	2.45432241	0.11720173	0.23770919	Lrrc40
ENSMUSG00000063060.7	43.9323145	0.00733356	0.00468096	2.37467232	0.12331747	0.24749539	Sox7
ENSMUSG00000063063.13	5.81466052	0.03332539	0.0150227	4.73415652	0.02956934	0.07853858	Ctnna2
ENSMUSG00000063065.14	504.056148	0.01055683	0.00312259	11.4413819	0.00071826	0.00328504	Mapk3
ENSMUSG00000063077.16	3335.36062	0.00026334	0.00364988	0.00508857	0.94313176	0.99876665	Kif1b
ENSMUSG00000063108.16	264.791487	0.01105477	0.00420163	6.91512744	0.00854695	0.02795181	Zfp26
ENSMUSG00000063116.5	1.01333924	0.00075258	0.03381608	0.01013266	0.91981948	0.98644397	Or3a1
ENSMUSG00000063130.5	0.90216518	0.0068714	0.02917426	0.19250655	0.66083858	0.82159984	Calml3
ENSMUSG00000063142.17	25.0848382	0.04598268	0.01074998	17.1733058	3.41E-05	0.00021668	Kcnma1
ENSMUSG00000063145.11	55.4702989	-0.0046565	0.00407741	1.32524253	0.24965334	0.42113601	Bbs5
ENSMUSG00000063146.12	70.3179165	0.03417029	0.00519965	41.3035291	1.30E-10	2.06E-09	Clip2
ENSMUSG00000063157.10	2.09013685	-0.0001503	0.02841429	-0.0023039	1	1	Csn2
ENSMUSG00000063160.13	27.3658371	0.02623814	0.00654594	15.434123	8.54E-05	0.00049618	Numbl
ENSMUSG00000063163.14	6.42798316	-0.0218884	0.14932072	0.17619854	0.67466072	0.83155036	Speer2
ENSMUSG00000063171.7	254.616678	-0.0365102	0.00385684	89.5825068	2.94E-21	1.75E-19	Rps4l
ENSMUSG00000063172.14	105.780334	-0.0053329	0.00370999	2.05791674	0.15141706	0.28945718	Ift25
ENSMUSG00000063179.14	260.487585	-0.0117127	0.0036815	10.2906876	0.00133703	0.00566264	Pstk
ENSMUSG00000063193.10	37.1262849	0.06722251	0.00659678	91.6271682	1.05E-21	6.62E-20	Cd300lb
ENSMUSG00000063200.5	1314.63835	-0.0033452	0.00326906	1.03361863	0.30931027	0.49439914	Nol7
ENSMUSG00000063229.16	13846.7294	-0.000282	0.00296266	0.00897691	0.92451613	0.98882811	Ldha
ENSMUSG00000063232.17	1171.67643	-0.0131602	0.00505704	6.68937511	0.00969892	0.03111517	Serpina11
ENSMUSG00000063234.6	7.09191627	0.04817816	0.00998734	20.4312881	6.18E-06	4.59E-05	Gpr84
ENSMUSG00000063235.19	1194.69533	-0.0080458	0.00316446	6.50472978	0.01075879	0.03395195	Ptpmt1
ENSMUSG00000063236.7	130.27791	-0.0015311	0.00300499	0.25824466	0.61132867	0.78358774	1110038F14Rik
ENSMUSG00000063239.18	3.60419019	0.01530443	0.02230697	0.69998376	0.40278915	0.59446749	Grm4
ENSMUSG00000063245.12	181.521962	0.03713565	0.00758985	18.6821093	1.54E-05	0.00010573	Zfp993
ENSMUSG00000063253.12	305.81105	-0.0013767	0.00314163	0.18994023	0.66296637	0.82289889	Scoc
ENSMUSG00000063260.3	3.52506927	0.00030291	0.06814547	0.00074422	0.97823616	1	Syt10
ENSMUSG00000063268.13	385.535567	0.01340476	0.00419819	10.3478081	0.00129629	0.00551194	Parp10
ENSMUSG00000063273.12	3860.87171	0.00163365	0.00476636	0.11364475	0.73603219	0.87394559	Naa15
ENSMUSG00000063275.17	130.088077	-0.0204925	0.0036187	32.0029903	1.54E-08	1.81E-07	Hacd1
ENSMUSG00000063281.10	223.919211	-0.0028441	0.00236269	1.44753731	0.22892352	0.39590777	Zfp35
ENSMUSG00000063296.6	8.76263509	0.03563274	0.01008812	11.2448081	0.00079846	0.00360637	Tmem117
ENSMUSG00000063297.8	21.5345533	0.03158292	0.01777211	3.61380702	0.05730181	0.13488227	Luzp2
ENSMUSG00000063316.14	3464.68168	0.0002943	0.00394315	0.00562368	0.9402217	0.99699614	Rpl27
ENSMUSG00000063317.8	157.498736	-0.0019771	0.0036639	0.28228299	0.59520875	0.77012418	Usp31
ENSMUSG00000063320.12	173.031625	-0.016325	0.00346722	22.2490739	2.39E-06	1.93E-05	Uqcc6
ENSMUSG00000063334.17	386.207172	-0.0051356	0.00216264	5.59566523	0.01800497	0.05219583	Krr1
ENSMUSG00000063354.8	639.86792	-0.0410597	0.00622805	47.8828913	4.52E-12	8.75E-11	Slc39a4
ENSMUSG00000063358.18	1716.34177	0.00017879	0.0019434	0.00840196	0.92696643	0.99001224	Mapk1
ENSMUSG00000063362.14	679.539505	-0.0049009	0.00262389	3.42452795	0.0642346	0.1478518	Alg11
ENSMUSG00000063364.11	9.0636735	-0.0056123	0.00615594	0.8060081	0.3693031	0.55986902	3300002I08Rik

ENSMUSG00000063382.7	738.807466	-0.0107305	0.00417878	6.43166672	0.01121033	0.03512313	Bcl9l
ENSMUSG00000063383.6	34.0785314	0.02685173	0.00369192	51.0046152	9.21E-13	1.94E-11	Zfp947
ENSMUSG00000063386.3	0.89695254	0.01086106	0.03860513	0.26349962	0.607726	0.78049689	Or2y15
ENSMUSG00000063406.12	2209.58574	-0.0084757	0.00312176	7.14585454	0.00751375	0.02505219	Tmed5
ENSMUSG00000063410.9	401.849383	0.00716698	0.00254999	7.73822702	0.00540639	0.01890512	Stk24
ENSMUSG00000063412.5	230.125856	0.00152133	0.00738005	0.03856775	0.84430748	0.94351541	Erhrt-ps
ENSMUSG00000063415.13	170.659425	0.00376917	0.00787497	0.20388982	0.65159925	0.81397277	Cyp26b1
ENSMUSG00000063428.9	366.945496	-0.0079104	0.0027292	8.40261637	0.00374681	0.01380602	Ddo
ENSMUSG00000063430.10	4.91789269	0.05056759	0.01217045	15.9488043	6.51E-05	0.00038909	Wscd2
ENSMUSG00000063434.7	4.96667769	0.03315922	0.0137656	6.00720851	0.01424755	0.04302656	Sorcs3
ENSMUSG00000063439.8	95.9100102	-0.0030043	0.00425896	0.50018933	0.47941694	0.66926906	B9d2
ENSMUSG00000063445.15	90.4399419	-0.0069707	0.00381305	3.42821123	0.06409148	0.14759439	Nmral1
ENSMUSG00000063446.5	7.96350235	0.05567819	0.01201938	20.4042822	6.27E-06	4.65E-05	Plppr1
ENSMUSG00000063447.8	4.51266991	-0.0115519	0.01319534	0.64454272	0.42207058	0.6137458	Ube2d2b
ENSMUSG00000063450.15	774.852501	-0.0012285	0.00310015	0.15668042	0.69223134	0.8443819	Syne2
ENSMUSG00000063455.17	88.6674703	0.00682365	0.0050869	1.69233976	0.19329282	0.34875327	D630045J12Rik
ENSMUSG00000063457.15	5716.51349	-0.0025174	0.00422875	0.35753256	0.54987979	0.73248982	Rps15
ENSMUSG00000063458.14	14.8216473	0.05332306	0.00591182	77.6220001	1.25E-18	5.43E-17	Lrmda
ENSMUSG00000063480.9	698.817341	-0.0053684	0.00305321	3.06251517	0.08011757	0.17635585	Snu13
ENSMUSG00000063488.7	376.304142	0.01187879	0.00417213	7.22994615	0.00716974	0.02410282	Zkscan7
ENSMUSG00000063506.15	14.3526942	0.06884078	0.01057748	40.122743	2.38E-10	3.62E-09	Arhgap22
ENSMUSG00000063511.12	1584.38413	-0.0012198	0.00455763	0.07149832	0.78916759	0.9097357	Snrnp70
ENSMUSG00000063522.6	1.71624281	0.05328837	0.01335608	15.1468606	9.95E-05	0.00056871	Ly6m
ENSMUSG00000063524.14	8086.10967	-0.0072856	0.00249311	8.5812866	0.00339635	0.01271126	Eno1
ENSMUSG00000063531.8	8.86801745	0.01981068	0.01294381	2.44419291	0.11796054	0.23894	Sema3e
ENSMUSG00000063535.8	24.6109612	0.03677897	0.00526191	45.8047161	1.31E-11	2.37E-10	Zfp773
ENSMUSG00000063550.9	470.100221	0.00193421	0.00255376	0.57188357	0.4495114	0.64104398	Nup98
ENSMUSG00000063558.5	1526.91316	-0.0051994	0.00359058	1.96043378	0.16146693	0.3036969	Aox1
ENSMUSG00000063564.14	9.7274266	0.01969182	0.00607538	9.95874542	0.00160087	0.00663238	Col23a1
ENSMUSG00000063568.12	14.1619633	-0.00983	0.00784971	1.630572	0.20162395	0.3605139	Jazf1
ENSMUSG00000063576.13	824.702157	-0.0087422	0.00237741	13.5135667	0.00023684	0.00123781	Klhdc3
ENSMUSG00000063590.4	313.296547	-0.0749874	0.00814087	71.0081072	3.56E-17	1.32E-15	Slc22a28
ENSMUSG00000063594.6	1.96273656	0.03751108	0.01327364	7.89427401	0.00495915	0.01756255	Gng8
ENSMUSG00000063600.15	13.5726653	-0.0064462	0.00687318	0.84539973	0.357857	0.54665901	Egfem1
ENSMUSG00000063605.6	18.3288556	0.01623501	0.00553671	8.49220278	0.00356672	0.01325214	Ccdc102a
ENSMUSG00000063615.7	1.60792381	0.00134789	0.02621848	0.02354805	0.87804047	0.96289089	Or51b17
ENSMUSG00000063626.7	5.04774345	0.02552698	0.0171666	2.71156152	0.09962343	0.20957173	Unc5d
ENSMUSG00000063632.7	7.07195154	-0.0024991	0.02092688	-0.0813916	1	1	Sox11
ENSMUSG00000063646.19	12.9331808	0.06466569	0.00990403	36.7184524	1.36E-09	1.85E-08	Jakmip1
ENSMUSG00000063652.11	13.2598895	0.02580394	0.00759319	10.6791418	0.0010835	0.00471417	Slc22a21
ENSMUSG00000063659.12	538.02117	0.01172711	0.00344399	11.4166899	0.00072787	0.00332383	Zbtb18
ENSMUSG00000063663.12	230.802804	-0.0103711	0.00422339	5.9353063	0.01484049	0.04449883	Brwd3
ENSMUSG00000063681.15	2.09188432	-0.000491	0.02425158	-0.0053801	1	1	Crb1
ENSMUSG00000063683.13	5028.14965	-0.0169154	0.00433783	14.8904844	0.00011394	0.00064326	Glyat
ENSMUSG00000063687.4	2.49427859	0.0301197	0.01005177	8.71600945	0.00315428	0.01192065	Pcdhb5
ENSMUSG00000063689.5	0.94015439	-0.0002161	0.03465872	-0.0013968	1	1	H2ac21
ENSMUSG00000063694.6	3291.92433	-0.0229612	0.00352431	42.9962196	5.48E-11	9.14E-10	Cycs
ENSMUSG00000063698.10	9.79750299	0.0244595	0.00790248	9.21818426	0.00239623	0.0094243	Sfxn4
ENSMUSG00000063704.14	195.748055	-0.0022575	0.00657915	0.10728908	0.74325184	0.87866779	Mapk15
ENSMUSG00000063727.4	92.4645721	0.01767275	0.00406036	18.7265431	1.51E-05	0.00010359	Tnfrsf11b
ENSMUSG00000063730.14	734.028488	-0.0280501	0.00576338	23.4593931	1.28E-06	1.08E-05	Hsd3b2
ENSMUSG00000063760.10	453.659511	-0.0125209	0.00297847	17.3501395	3.11E-05	0.00019871	Rnf217
ENSMUSG00000063765.13	9.55528525	-0.0274067	0.0074967	13.0275374	0.00030694	0.00155656	Chadl
ENSMUSG00000063767.5	5.27313556	-0.0017737	0.02238031	-0.0370178	1	1	S100a7a
ENSMUSG00000063777.4	1.19208664	0.00814392	0.04447464	0.10856606	0.7417826	0.87777279	Or5b3
ENSMUSG00000063779.7	1.09917699	0.00065798	0.03725885	0.0045857	0.94601028	1	Chil4
ENSMUSG00000063785.13	251.48217	-0.0056659	0.00263592	4.5497064	0.03292439	0.08582612	Utp14a
ENSMUSG00000063787.5	895.831108	-0.0129175	0.00410485	9.93622585	0.00162057	0.00670986	Chchd1
ENSMUSG00000063796.10	17.4141128	0.02130649	0.02159678	1.33457636	0.24799271	0.41926428	Slc22a8
ENSMUSG00000063800.6	221.732502	-0.0035372	0.00259746	1.85880995	0.17276221	0.32028835	Prpf38a
ENSMUSG00000063801.8	577.596302	-0.0018322	0.00227104	0.64402137	0.42225834	0.61390527	Ap3s2

ENSMUSG00000063802.6	274.21427	-0.0115973	0.003356	11.9476016	0.00054718	0.00259047	Hspbp1
ENSMUSG00000063804.9	3.58445164	0.00180391	0.02130175	0.01942704	0.88914916	0.96954458	Lin28b
ENSMUSG00000063808.15	227.998847	0.00269108	0.00318984	0.71564741	0.39757536	0.58931702	Gpatch1
ENSMUSG00000063810.8	60.6639025	0.00557344	0.0043985	1.55785207	0.21198	0.3743176	Alms1
ENSMUSG00000063823.3	0.87314437	0.00032277	0.03386934	0.00430713	0.94767342	1	Or13a21
ENSMUSG00000063838.7	59.8035789	0.02111541	0.00650864	9.44795075	0.00211385	0.00847972	Cdc42ep5
ENSMUSG00000063842.6	1.28835632	0.01405469	0.03333064	0.37223303	0.54178958	0.72535898	Or7e170
ENSMUSG00000063849.7	266.918915	0.00288008	0.00302463	0.90846739	0.34052139	0.52832112	Ppcdc
ENSMUSG00000063851.13	0.97000819	-0.0001283	0.04551621	-0.0036956	1	1	Rnf183
ENSMUSG00000063856.9	32563.2745	-0.0111709	0.00389585	8.2360122	0.00410673	0.01494311	Gpx1
ENSMUSG00000063870.13	1933.26205	-0.0014875	0.00260764	0.32721299	0.56730481	0.7471773	Chd4
ENSMUSG00000063873.11	18.5289063	0.03469669	0.00794282	18.2352609	1.95E-05	0.00013085	Slc24a3
ENSMUSG00000063882.13	5499.89343	-0.0175957	0.00471232	14.2138116	0.00016317	0.00088921	Uqcrh
ENSMUSG00000063884.7	786.927235	-0.0143336	0.00242578	34.9112997	3.45E-09	4.47E-08	Ptcd3
ENSMUSG00000063887.14	10.1946887	0.03142875	0.01584034	4.11350275	0.04254217	0.10578804	Nlgn1
ENSMUSG00000063888.8	1396.03305	-0.009436	0.00256168	13.5036267	0.0002381	0.00124383	Rpl7l1
ENSMUSG00000063889.17	603.683994	-0.0127156	0.00331462	13.9874879	0.00018403	0.00099145	Crem
ENSMUSG00000063894.16	189.461744	0.00301718	0.00382781	0.60852929	0.4353421	0.62690124	Zkscan8
ENSMUSG00000063903.6	20.2940363	-0.0217138	0.01363256	2.42994731	0.1190369	0.24076874	Klk1
ENSMUSG00000063904.5	981.194924	-0.0099705	0.00236104	17.9953424	2.21E-05	0.00014651	Dpp3
ENSMUSG00000063919.12	107.611204	0.00790329	0.00572687	1.79667898	0.18011451	0.33072642	Srrm4
ENSMUSG00000063929.7	273.86175	-0.0268567	0.00516387	26.6924585	2.39E-07	2.32E-06	Cyp4a32
ENSMUSG00000063931.15	685.215416	-0.0047168	0.00227419	4.3028226	0.03804917	0.09648724	Pepd
ENSMUSG00000063952.17	519.363088	-0.0069693	0.00397255	3.05853524	0.08031404	0.17670587	Brpf3
ENSMUSG00000063953.4	40.8467355	0.01629471	0.00802978	3.94321732	0.04706069	0.11492788	Amd2
ENSMUSG00000063954.8	357.565479	0.00490349	0.01537647	-9.8746319	1	1	H2ac19
ENSMUSG00000063972.14	205.039747	0.0043766	0.00533395	0.67141738	0.41255788	0.60397183	Nr6a1
ENSMUSG00000063998.5	0.9294402	0.00052052	0.04007927	0.00886335	0.92499369	0.98907054	Vmn1r211
ENSMUSG00000064006.7	1.84618609	0.00462345	0.03010492	0.05611858	0.81273933	0.92324182	Or5h22
ENSMUSG00000064023.5	7.47347679	0.03861634	0.00918664	16.090869	6.04E-05	0.00036336	Klk8
ENSMUSG00000064030.16	162.820158	-0.0009315	0.0027763	0.11354748	0.73614098	0.87395751	Pym1
ENSMUSG00000064037.14	241.543784	-0.0024495	0.00231394	1.10796551	0.29252463	0.47445234	Gpn1
ENSMUSG00000064039.5	0.8781765	0.0491464	0.0185552	7.47715263	0.00624868	0.02139373	Ccr1l1
ENSMUSG00000064043.16	28.7535578	0.04722216	0.00596692	59.7107986	1.10E-14	3.00E-13	Trerf1
ENSMUSG00000064057.13	5.05093463	0.00944532	0.01423117	0.52633957	0.46814986	0.65925026	Scgb3a1
ENSMUSG00000064061.14	80.1583999	0.00107001	0.00491529	0.04375539	0.83430937	0.93682555	Dzip3
ENSMUSG00000064065.16	35.2606381	0.07250393	0.00837351	63.4004994	1.69E-15	5.06E-14	Ipcef1
ENSMUSG00000064068.16	476.239682	-0.0021522	0.00277028	0.6198786	0.4310924	0.62260397	Mtx1
ENSMUSG00000064080.13	55.0210992	-0.0020585	0.00676773	0.09197305	0.76168366	0.89131623	Fbln2
ENSMUSG00000064090.15	128.066285	0.01326455	0.00403876	10.3547008	0.00129145	0.00549824	Vrk2
ENSMUSG00000064105.14	121.510454	0.00020453	0.00277359	0.00541636	0.94133193	0.99771963	Cnnm2
ENSMUSG00000064109.9	9.29389935	0.05681519	0.00853545	40.9072468	1.60E-10	2.48E-09	Hcst
ENSMUSG00000064115.14	13.9186846	0.03627668	0.01369519	6.40789266	0.01136142	0.03551158	Cadm2
ENSMUSG00000064120.15	1459.35765	-0.0097663	0.00344374	8.08248104	0.00446952	0.01607471	Mocs1
ENSMUSG00000064125.15	16.9476755	0.01868901	0.00605686	9.34967596	0.00223025	0.00888108	Prr36
ENSMUSG00000064127.14	347.378705	0.00668553	0.00271041	5.95157389	0.01470415	0.04417426	Med14
ENSMUSG00000064128.9	77.5721767	0.00505798	0.0048362	1.06159931	0.3028507	0.48751274	Cenpj
ENSMUSG00000064137.7	4.57584523	-0.0046597	0.05312918	-0.013783	1	1	Rhox8
ENSMUSG00000064138.15	236.447078	0.01410634	0.00234968	35.5308821	2.51E-09	3.30E-08	Fam172a
ENSMUSG00000064141.15	21.574992	0.01846723	0.00500818	12.8734768	0.00033327	0.00167175	Zfp69
ENSMUSG00000064145.13	794.654606	-0.0029687	0.00258961	1.30547389	0.25321577	0.42541115	Arih2
ENSMUSG00000064147.8	4.69784831	0.06393072	0.01329491	18.3704828	1.82E-05	0.00012269	Rab44
ENSMUSG00000064158.7	2.26637687	-0.0079885	0.01107107	0.51546307	0.47278345	0.66347775	Izumo1
ENSMUSG00000064179.15	31.0313985	-0.0135792	0.00593067	4.9744792	0.02572395	0.07013352	Tnnt1
ENSMUSG00000064181.7	455.974459	-0.0148363	0.00239874	38.5823384	5.25E-10	7.57E-09	Rab3ip
ENSMUSG00000064202.17	46.6745328	-0.0165876	0.00670444	5.90132851	0.01512947	0.04518152	Spata6l
ENSMUSG00000064210.8	283.873701	0.03169154	0.00494957	38.4249333	5.69E-10	8.17E-09	Ano6
ENSMUSG00000064213.6	0.60925227	-0.0576408	0.11211406	0.3188502	0.57229945	0.75148953	Defa24
ENSMUSG00000064215.14	1793.87923	0.04355188	0.00381706	127.229099	1.66E-29	3.07E-27	Ifi27
ENSMUSG00000064220.7	455.777796	-0.0381484	0.00520332	53.055991	3.24E-13	7.27E-12	H2ac18
ENSMUSG00000064225.7	6679.30831	-0.0194737	0.00410461	21.0864492	4.39E-06	3.36E-05	Paqr9

ENSMUSG00000064246.11	10.5336211	0.09577359	0.01163743	59.9526193	9.72E-15	2.68E-13	Chi3l1
ENSMUSG00000064247.15	36.1338496	0.01530316	0.00673899	5.05243999	0.02459129	0.06751526	Plcxd1
ENSMUSG00000064254.7	842.156585	-0.0160135	0.00395163	16.483131	4.91E-05	0.0003018	Ethe1
ENSMUSG00000064259.3	4.04612863	-0.0059364	0.01948143	0.03598013	0.84955654	0.94727181	Vmn1r13
ENSMUSG00000064262.8	123.248357	0.03092804	0.00382254	62.6646026	2.45E-15	7.23E-14	Gimap8
ENSMUSG00000064264.15	19.414396	-0.0201308	0.00565441	12.3732187	0.00043554	0.002115	Zfp428
ENSMUSG00000064267.14	47.6496934	0.06852982	0.00568523	131.044274	2.42E-30	5.34E-28	Hvcn1
ENSMUSG00000064280.15	7.56958289	0.00304553	0.01779338	0.05883379	0.80834877	0.92087104	Ccdc146
ENSMUSG00000064284.13	89.616268	-0.000708	0.00350686	0.04046588	0.84057243	0.94094643	Cdpf1
ENSMUSG00000064288.5	3.57900583	-0.0065967	0.02322557	0.07545977	0.78354719	0.90578817	H4c12
ENSMUSG00000064289.16	406.60009	-0.0034077	0.00244641	1.89573936	0.168556	0.31405022	Tank
ENSMUSG00000064293.15	5.40737493	0.00560486	0.02553109	0.04829253	0.82606148	0.93133299	Cntn4
ENSMUSG00000064294.13	8496.46823	-0.0363589	0.00551822	42.1560761	8.43E-11	1.37E-09	Aox3
ENSMUSG00000064302.15	416.929675	-0.0063691	0.0041612	2.30647194	0.12883618	0.2561077	Clasp1
ENSMUSG00000064307.14	29.6214517	-0.0136624	0.00454587	9.29896354	0.00229284	0.00907972	Lrrc51
ENSMUSG00000064325.5	58.8159826	0.00198813	0.00541359	0.12900138	0.7194697	0.86270526	Hhip
ENSMUSG00000064326.14	117.201246	0.01283145	0.00470417	7.43661966	0.00639098	0.02181443	Siva1
ENSMUSG00000064329.15	19.1761368	0.01710429	0.01703518	1.11400258	0.29121353	0.47281354	Scn1a
ENSMUSG00000064341.1	130674.939	-0.005771	0.00410374	1.94745577	0.16286128	0.30566135	mt-Nd1
ENSMUSG00000064345.1	152693.845	-0.0117432	0.00505711	5.21552515	0.02238608	0.06250719	mt-Nd2
ENSMUSG00000064351.1	522057.219	-0.0071543	0.00381683	3.49507997	0.06155144	0.14283276	mt-Co1
ENSMUSG00000064354.1	81814.2135	0.00107399	0.00600765	0.03065282	0.8610172	0.95374443	mt-Co2
ENSMUSG00000064356.1	59030.8496	-0.0091104	0.00598329	2.14131944	0.14337853	0.27789678	mt-Atp8
ENSMUSG00000064357.1	136553.416	0.0026982	0.00561307	0.21613127	0.64200364	0.80814954	mt-Atp6
ENSMUSG00000064358.1	157321.546	-0.0048227	0.00497413	0.91082874	0.33989463	0.52762634	mt-Co3
ENSMUSG00000064360.1	12841.0192	-0.0196638	0.00782494	6.38882435	0.01148411	0.03580018	mt-Nd3
ENSMUSG00000064363.1	170834.322	-0.0146103	0.00528615	7.30628769	0.00687137	0.02327229	mt-Nd4
ENSMUSG00000064367.1	105227.74	-0.0103882	0.00545783	3.51700141	0.06074225	0.14135834	mt-Nd5
ENSMUSG00000064368.1	11323.644	-0.0112107	0.00729606	2.2874335	0.13042543	0.25846196	mt-Nd6
ENSMUSG00000064370.1	199845.051	-0.0134971	0.00441658	9.1663254	0.00246509	0.00965166	mt-Cytb
ENSMUSG00000064373.14	169124.873	-0.001298	0.00306408	0.17830589	0.67283319	0.83025237	Selenop
ENSMUSG00000065947.1	11375.8785	0.00728614	0.01070306	0.41081866	0.52155428	0.7075451	mt-Nd4l
ENSMUSG00000065952.14	92.7400682	0.00070863	0.00617269	0.01327659	0.90826752	0.9800179	Rps23rg1
ENSMUSG00000065954.12	1139.62195	-0.0087665	0.00398902	4.76559538	0.02903402	0.07732569	Tacc1
ENSMUSG00000065979.13	1165.24311	0.00132596	0.00179489	0.54745164	0.45936083	0.65092482	Cpped1
ENSMUSG00000065987.13	7.81013301	0.00332274	0.00685895	0.22035212	0.63877175	0.80521275	Cd209b
ENSMUSG00000065990.13	918.799525	-0.012257	0.00283294	18.9249202	1.36E-05	9.41E-05	Aurkaip1
ENSMUSG00000065999.14	5.72545164	0.01459397	0.01634989	0.88462205	0.34693885	0.53521978	Zfp985
ENSMUSG00000066000.13	97.6731183	0.05709132	0.00828285	32.8982675	9.71E-09	1.17E-07	Zfp979
ENSMUSG00000066007.16	4.47728964	0.00682907	0.01896395	0.19719952	0.65699087	0.81875656	Zfp600
ENSMUSG00000066009.9	11.5440084	0.03788994	0.01302702	8.60163409	0.00335862	0.012592	Zfp987
ENSMUSG00000066026.15	2263.33287	-0.0051534	0.0032735	2.40989278	0.12057062	0.24312035	Dhrs3
ENSMUSG00000066036.15	2563.56939	-0.0095147	0.00393481	5.67465255	0.01721175	0.05029957	Ubr4
ENSMUSG00000066037.15	712.671663	0.00442303	0.00262078	2.75433588	0.09699108	0.20529765	Hnrnpr
ENSMUSG00000066042.5	38.9953793	0.00664378	0.0031789	4.30385515	0.03802608	0.09644942	Med18
ENSMUSG00000066043.14	742.693369	-0.0077084	0.00400266	3.63192913	0.05668108	0.13375573	Phactr4
ENSMUSG00000066071.7	3905.69579	-0.0727793	0.00834886	65.7670131	5.08E-16	1.61E-14	Cyp4a12a
ENSMUSG00000066072.14	5638.00536	-0.0106876	0.0076042	1.93747744	0.16394269	0.30720206	Cyp4a10
ENSMUSG00000066097.7	5.6287317	0.0236854	0.02665512	1.29266863	0.25555674	0.42836765	Cyp2j11
ENSMUSG00000066113.17	35.8733675	0.04015384	0.00762187	25.1886024	5.20E-07	4.72E-06	Adamtsl1
ENSMUSG00000066122.7	3.52077976	0.00191339	0.01694927	0.03812621	0.84518992	0.94418839	Or13a17
ENSMUSG00000066129.18	6.79114995	0.02775553	0.01486263	4.27004861	0.03878982	0.09804916	Kndc1
ENSMUSG00000066148.4	243.195914	-0.0072657	0.00227945	10.1156544	0.00147015	0.00616301	Prpf4
ENSMUSG00000066149.12	673.202833	-0.0072004	0.00506226	2.00115348	0.15717955	0.2976505	Cdc26
ENSMUSG00000066150.13	3801.67029	-0.0066209	0.00249438	6.99382493	0.00817914	0.02691311	Slc31a1
ENSMUSG00000066151.13	589.907772	0.00245671	0.00209953	1.37827602	0.24039502	0.41009381	Fkbp15
ENSMUSG00000066152.12	191.159571	0.00741595	0.00288342	6.60367061	0.01017688	0.03236675	Slc31a2
ENSMUSG00000066153.3	2777.21241	-0.0655996	0.00777565	64.0457512	1.22E-15	3.71E-14	Mup21
ENSMUSG00000066154.13	163766.627	-0.0699975	0.00772367	75.0823373	4.51E-18	1.84E-16	Mup3
ENSMUSG00000066189.10	6.21085259	-0.0022846	0.02363942	0.02132186	0.88390555	0.96617695	Cacng3
ENSMUSG00000066191.13	14.1626531	0.03700311	0.00709557	27.3512318	1.70E-07	1.69E-06	Anks6

ENSMUSG00000066196.12	6.04475445	-0.0187166	0.00845791	5.08959599	0.0240698	0.06632129	Spag8
ENSMUSG00000066232.5	1761.96496	-0.0025019	0.00235346	1.09774156	0.29476227	0.47735945	Ipo7
ENSMUSG00000066233.7	145.411804	-0.0082468	0.00294353	7.8009747	0.00522181	0.01835766	Tmem42
ENSMUSG00000066235.8	58.2838868	-0.0029533	0.00352179	0.70527648	0.40101638	0.5927598	Pomgnt2
ENSMUSG00000066241.3	5.99951108	-0.0097674	0.18238195	-0.010724	1	1	Or10a48
ENSMUSG00000066258.14	187.873517	0.02655945	0.00433129	37.468416	9.29E-10	1.29E-08	Trim12a
ENSMUSG00000066262.6	1.24563706	0.00355052	0.04066531	0.04210878	0.83741255	0.93883508	Or51i1
ENSMUSG00000066263.11	1.86797392	-0.0051865	0.01965697	-0.0053116	1	1	Or51k1
ENSMUSG00000066268.7	6.89637119	-0.0043527	0.03785151	-0.0139872	1	1	Or51a5
ENSMUSG00000066278.7	294.581345	-0.0052598	0.00297928	3.02447009	0.08201711	0.17963186	Vps37b
ENSMUSG00000066306.14	1029.27962	0.0005718	0.00228383	0.06179893	0.80367493	0.91798522	Numa1
ENSMUSG00000066319.7	986.455494	-0.0164438	0.00423567	14.7190025	0.00012478	0.00069792	Rtp3
ENSMUSG00000066324.3	1140.53017	0.00195049	0.00250245	0.58983459	0.44248309	0.63439342	Bpnt2
ENSMUSG00000066357.7	319.35647	-0.0024946	0.00332102	0.58605012	0.44395059	0.63622667	Wdr6
ENSMUSG00000066361.4	21.9234769	-0.0390541	0.00892001	18.6375012	1.58E-05	0.00010804	Serpina3c
ENSMUSG00000066363.13	83.4679774	0.04159388	0.00555741	53.2350624	2.96E-13	6.69E-12	Serpina3f
ENSMUSG00000066364.3	1.36846552	0.01267833	0.01198407	1.2581204	0.26200717	0.43654289	Serpina3b
ENSMUSG00000066366.15	71482.3415	-0.0135076	0.00580065	5.35143694	0.0207052	0.05864274	Serpina1a
ENSMUSG00000066372.7	1.68172809	0.00702721	0.01976804	0.23719516	0.6262393	0.79505422	Vmn2r65
ENSMUSG00000066392.13	21.3745935	-0.0067043	0.00980937	0.41670891	0.51858382	0.70465019	Nrxn3
ENSMUSG00000066406.17	3090.54784	0.00284624	0.00578923	0.23937187	0.62466015	0.79368822	Akap13
ENSMUSG00000066415.5	527.11147	0.00042875	0.00239467	0.03143992	0.85926251	0.95283438	Msl2
ENSMUSG00000066438.7	8.57134417	-0.0108604	0.01401468	0.63293945	0.42627915	0.61789625	Plekhd1
ENSMUSG00000066440.6	545.392686	-0.006234	0.00330772	3.5470513	0.05965137	0.139218	Zfyve26
ENSMUSG00000066441.15	1651.21303	0.004495	0.00425344	1.05879443	0.30349031	0.48814256	Rdh11
ENSMUSG00000066442.18	395.387873	0.00079438	0.00339642	0.05587558	0.81313769	0.92338303	Mthfs
ENSMUSG00000066456.15	43.208713	0.02583398	0.00727469	12.2153223	0.00047399	0.00228152	Hmgn3
ENSMUSG00000066500.6	0.83279473	-0.0161203	0.20950078	0.00720698	0.93234573	0.99297137	Izumo2
ENSMUSG00000066513.5	55.4828257	-0.0030897	0.00439403	0.49714956	0.48075527	0.6705035	Klk1b4
ENSMUSG00000066537.6	18.6984899	-0.0075032	0.00490208	2.2788078	0.13115266	0.25959773	Vmn2r57
ENSMUSG00000066551.13	5074.53847	0.001104	0.00300872	0.13466697	0.7136414	0.85890423	Hmgb1
ENSMUSG00000066568.13	655.269987	0.00017138	0.00219498	0.00606114	0.93794473	0.99588386	Lsm14a
ENSMUSG00000066571.14	311.48053	-0.0015483	0.0037468	0.1675606	0.68228894	0.83734655	Garre1
ENSMUSG00000066595.10	86.4667506	0.01021174	0.00373771	7.29509016	0.00691433	0.0233909	Flvcr1
ENSMUSG00000066607.6	8.54949437	0.02628843	0.01320296	3.88629509	0.0486817	0.11801898	Insyn1
ENSMUSG00000066613.15	222.367274	-0.0119149	0.00396236	8.97020768	0.00274417	0.01058967	Zfp932
ENSMUSG00000066621.13	287.136833	-0.000676	0.00405668	0.02731247	0.86873552	0.95806749	Tecpr1
ENSMUSG00000066637.3	294.37584	-0.011574	0.00363258	10.0238189	0.00154529	0.00643816	Ttc32
ENSMUSG00000066640.12	176.864993	-0.0087489	0.00369353	5.51204554	0.01888595	0.0542761	Fbxl18
ENSMUSG00000066643.13	89.0796099	0.00178601	0.00376163	0.22393625	0.63605693	0.80335577	Wdr35
ENSMUSG00000066677.13	38.1995183	0.01957181	0.00510228	14.5260458	0.00013824	0.00076338	Ifi208
ENSMUSG00000066682.12	42.1574725	0.04451113	0.00659507	42.9488619	5.62E-11	9.35E-10	Pilrb2
ENSMUSG00000066684.11	55.2343235	0.03698785	0.00628189	32.8337975	1.00E-08	1.21E-07	Pilrb1
ENSMUSG00000066687.6	409.345098	-0.0279585	0.00794103	12.3350224	0.00044454	0.00215119	Zbtb16
ENSMUSG00000066705.8	16.4422353	0.00397198	0.00708221	0.30858517	0.57854968	0.75666368	Fxyd6
ENSMUSG00000066735.9	1013.9269	0.00188765	0.00243497	0.59118994	0.44195935	0.63383516	Vkorc1l1
ENSMUSG00000066747.7	2.22721291	0.00259035	0.0363435	0.03094323	0.86036709	0.95360048	Or8b4
ENSMUSG00000066748.5	1.91057966	0.00175482	0.02998719	0.02455005	0.87549346	0.96166318	Or8b8
ENSMUSG00000066755.3	0.960176	0.07118114	0.01911434	13.9371982	0.00018902	0.00101554	Tnfsf18
ENSMUSG00000066760.13	2.17839061	0.0147144	0.01324836	1.46524914	0.22609677	0.3922274	Psg16
ENSMUSG00000066798.4	193.728491	0.00430177	0.00251437	2.86865748	0.09032018	0.194051	Zbtb6
ENSMUSG00000066800.12	81.1572379	0.03000516	0.00454954	41.7811371	1.02E-10	1.63E-09	Rnasel
ENSMUSG00000066820.8	0.77804124	-0.0106957	0.02866564	0.11820136	0.7309935	0.87076832	Vmn2r28
ENSMUSG00000066829.8	147.457022	0.01644279	0.00372052	19.3034923	1.12E-05	7.82E-05	Zfp810
ENSMUSG00000066838.8	48.7942914	0.01057317	0.00303718	11.8748155	0.00056898	0.00267757	Zfp772
ENSMUSG00000066839.13	661.015199	-0.0171135	0.00229784	56.0211829	7.17E-14	1.74E-12	Ecsit
ENSMUSG00000066842.19	98.4138142	0.01642577	0.0082021	3.77149193	0.05213311	0.12471971	Hmcn1
ENSMUSG00000066861.15	47.8636694	0.04004908	0.00759077	27.7870564	1.35E-07	1.38E-06	Oas1g
ENSMUSG00000066877.12	66.4568426	0.00211945	0.00388769	0.29381103	0.58778862	0.76463418	Nck2
ENSMUSG00000066878.6	26.3934593	0.00835782	0.00806426	1.01437721	0.31385647	0.49919879	Kpna2rt
ENSMUSG00000066880.16	470.757329	0.00379889	0.00281717	1.76437061	0.1840803	0.33635868	Zfp617

ENSMUSG00000066892.15	115.462382	-0.002212	0.00316493	0.50008209	0.47946406	0.66926906	Fbxl12
ENSMUSG00000066894.15	34.2877818	0.00549782	0.00551194	0.98921963	0.31993318	0.50596379	Vsig10
ENSMUSG00000066900.12	1639.40452	-0.0102064	0.00265491	14.8044638	0.00011925	0.00067033	Suds3
ENSMUSG00000066952.12	15.3160403	0.02045874	0.01142511	2.98972319	0.08379449	0.18279475	Myo1h
ENSMUSG00000066975.13	2.73041445	0.02322588	0.01332845	2.38999625	0.12211392	0.24558143	Cryba4
ENSMUSG00000066979.7	466.802338	0.00340414	0.00199562	2.91890094	0.08754748	0.18937786	Bub3
ENSMUSG00000067001.12	2.75545931	0.01245	0.01289725	0.76067759	0.38311655	0.57480736	Serpinb7
ENSMUSG00000067006.13	0.76838596	0.01010745	0.02211224	0.33272879	0.56405668	0.74460407	Serpinb5
ENSMUSG00000067010.5	1.40304792	-0.0112418	0.02227283	0.17800973	0.67308926	0.83052489	Vmn2r10
ENSMUSG00000067028.12	1.50608159	-0.0080397	0.03074557	0.09379572	0.75940603	0.88987235	Cntnap5b
ENSMUSG00000067049.9	3.44252489	-0.0148617	0.01146537	1.60017605	0.20587826	0.36644686	Unc93a
ENSMUSG00000067071.9	1373.06281	-0.0319425	0.00467225	46.9169626	7.41E-12	1.40E-10	Hes6
ENSMUSG00000067081.14	5.43345563	-0.001731	0.02717658	-0.0083843	1	1	Asb18
ENSMUSG00000067144.8	444.520735	-0.0492043	0.00728205	40.9952151	1.53E-10	2.38E-09	Slc22a7
ENSMUSG00000067148.16	300.741829	-0.0069467	0.00287285	5.82529193	0.01579735	0.04682033	Polr1c
ENSMUSG00000067149.7	227.97673	0.15181878	0.00815123	234.163766	7.37E-53	1.58E-49	Jchain
ENSMUSG00000067150.7	346.840685	-0.004525	0.00240852	3.50859657	0.06105115	0.14196514	Xpo5
ENSMUSG00000067158.10	31.2824724	0.02742936	0.00635375	17.667269	2.63E-05	0.00017101	Col4a4
ENSMUSG00000067194.7	1315.82347	-0.0059093	0.00308587	3.61429882	0.05728486	0.13485586	Eif1ax
ENSMUSG00000067199.5	116.1192	0.00015784	0.00372195	0.00175013	0.96663059	1	Frat1
ENSMUSG00000067206.4	1.84515202	0.05548124	0.01464684	15.2848884	9.25E-05	0.00053276	Lrrc66
ENSMUSG00000067212.9	3726.46355	0.01539308	0.00321971	23.1251907	1.52E-06	1.27E-05	H2-T23
ENSMUSG00000067219.10	249.254193	0.06651074	0.00794711	65.3123839	6.39E-16	2.02E-14	Nipal1
ENSMUSG00000067220.13	8.55008613	0.01055985	0.01636593	0.45696268	0.4990469	0.68628769	Cnga1
ENSMUSG00000067225.3	3942.0832	-0.0079351	0.00773697	0.99576638	0.31833709	0.50408182	Cyp2c54
ENSMUSG00000067231.5	0.87155201	0.02176194	0.01782265	1.63364737	0.20119931	0.35991279	Cyp2c65
ENSMUSG00000067235.15	40196.8097	-0.004451	0.00525422	0.70605572	0.40075634	0.59246828	H2-Q10
ENSMUSG00000067242.12	9.8955539	0.02207873	0.0143022	2.65844322	0.1030015	0.21487647	Lgi1
ENSMUSG00000067274.11	10639.7873	-0.0050656	0.00301548	2.80476788	0.09398445	0.20031783	Rplp0
ENSMUSG00000067276.6	31.166728	0.04893993	0.00633289	58.5729453	1.96E-14	5.16E-13	Capn6
ENSMUSG00000067279.4	1609.73451	-0.0157126	0.0057212	7.48783902	0.00621171	0.02129275	Ppp1r3c
ENSMUSG00000067288.14	2107.62983	0.00192011	0.00374544	0.26339059	0.60780028	0.78051174	Rps28
ENSMUSG00000067297.6	4.6608562	0.03801145	0.01093608	10.9581002	0.00093195	0.0041248	Ifit1bl2
ENSMUSG00000067336.7	904.714288	0.00352759	0.0033133	1.10433625	0.29331644	0.47540968	Bmpr2
ENSMUSG00000067338.7	3.07217283	-0.0145556	0.06076341	0.13933013	0.70894787	0.85603439	Tuba3b
ENSMUSG00000067341.7	6.18089384	0.10052046	0.00972441	87.9557833	6.69E-21	3.81E-19	H2-Eb2
ENSMUSG00000067365.12	300.47165	0.00234076	0.00270977	0.74982524	0.38653157	0.57799616	Tmem128
ENSMUSG00000067367.10	303.920518	-0.0042944	0.00380854	1.26153064	0.26136156	0.43571344	Lyar
ENSMUSG00000067369.13	576.689794	-0.0089805	0.00194348	21.6061181	3.35E-06	2.63E-05	Trmt2b
ENSMUSG00000067370.5	8.18029164	0.02890555	0.00669388	17.7958745	2.46E-05	0.00016107	B3galt4
ENSMUSG00000067377.13	15.7478423	0.0211257	0.00723466	8.36962365	0.00381545	0.01403261	Tspan6
ENSMUSG00000067399.11	1.43788786	0.00043322	0.01847241	0.00589972	0.93877501	0.99612864	Trim43c
ENSMUSG00000067424.14	112.853847	0.01048766	0.00399484	6.79615158	0.00913546	0.02958925	Zfp563
ENSMUSG00000067430.8	64.6119501	0.00409581	0.00369686	1.2194919	0.26946039	0.44599949	Zfp763
ENSMUSG00000067455.6	5.25040855	-0.0253823	0.0183499	2.25112149	0.13351761	0.263096	H4c11
ENSMUSG00000067525.4	0.7204278	-0.0063619	0.04891174	0.00549919	0.94088585	0.99742612	Or4z4
ENSMUSG00000067567.13	69.7755011	0.00364652	0.00306255	1.41379697	0.2344271	0.40279722	Hdac8
ENSMUSG00000067578.4	5.07555829	0.01186233	0.02560675	0.42729071	0.51332136	0.69999986	Cbln4
ENSMUSG00000067586.5	51.9686112	-0.0150409	0.00496605	8.72903735	0.00313182	0.01184526	S1pr3
ENSMUSG00000067591.13	2.43729727	0.13131027	0.01435118	76.7849494	1.91E-18	8.12E-17	Klra3
ENSMUSG00000067594.7	1.40344609	0.01655954	0.04524694	0.38453926	0.53518386	0.71953975	Krt77
ENSMUSG00000067599.14	1.83251142	0.05056466	0.01493913	10.0856944	0.00149425	0.00625215	Klra7
ENSMUSG00000067629.14	37.7736515	0.01183555	0.00722674	2.5624709	0.10942743	0.22537159	Syngap1
ENSMUSG00000067649.4	8.285175	-0.0042913	0.05612325	-0.0174489	1	1	Mageb18
ENSMUSG00000067653.13	211.49502	-0.0187234	0.0080622	6.0433726	0.01395859	0.04225197	Ankrd23
ENSMUSG00000067656.14	203.689499	0.15192602	0.00927094	223.961951	1.24E-50	2.24E-47	Slc22a27
ENSMUSG00000067702.6	2.82917714	-0.0864752	0.04318135	1.57084706	0.21008404	0.37191523	Tuba3a
ENSMUSG00000067713.8	572.222055	-0.0031335	0.00210077	2.25700429	0.13301114	0.26248334	Prkag1
ENSMUSG00000067714.15	3.61127257	0.05614677	0.00878175	38.9178663	4.42E-10	6.44E-09	Lpar5
ENSMUSG00000067722.5	493.411907	-0.0149179	0.00215728	48.2191056	3.81E-12	7.45E-11	Uqcc4
ENSMUSG00000067768.13	17.3606032	0.04928125	0.01209667	13.2035688	0.00027942	0.00143392	Xlr4b

ENSMUSG00000067771.11	0.53449254	-0.0513183	0.04786833	1.5301145	0.21609526	0.37962333	Pwwp4a
ENSMUSG00000067780.4	7.09922441	-0.0099404	0.00913594	1.11728904	0.29050294	0.47195223	Pi15
ENSMUSG00000067786.17	59.6526635	0.02121117	0.01079307	3.55702777	0.05929382	0.13854805	Nnat
ENSMUSG00000067787.11	469.199407	-0.0060976	0.00238553	6.49758596	0.01080211	0.03407455	Blcap
ENSMUSG00000067813.4	373.263956	-0.0131827	0.00403243	10.7122491	0.00106429	0.0046374	Xkr9
ENSMUSG00000067818.7	62.8129884	-0.0041864	0.00529689	0.62191818	0.43033537	0.62177714	Myl9
ENSMUSG00000067825.12	434.541176	-0.0136705	0.00324094	17.5632412	2.78E-05	0.00018022	Pex26
ENSMUSG00000067847.14	788.915485	-0.0068628	0.0042149	2.66101799	0.1028349	0.21461696	Romo1
ENSMUSG00000067851.12	1676.49819	-0.0051783	0.00358877	2.01755079	0.15548976	0.29528032	Arfgef1
ENSMUSG00000067873.12	796.877985	0.00139389	0.00201316	0.47838671	0.48915398	0.67722386	Htatsf1
ENSMUSG00000067878.14	5.58339726	0.06514919	0.01012377	38.9832139	4.27E-10	6.25E-09	Map7d3
ENSMUSG00000067879.4	17.2696825	0.06822585	0.01087079	34.4201591	4.44E-09	5.65E-08	Vxn
ENSMUSG00000067882.16	1.43586173	-0.014666	0.01110745	1.66902012	0.19638983	0.35313338	Casp16
ENSMUSG00000067889.8	1463.80043	-0.0065076	0.00472954	1.86692217	0.171828	0.31886194	Sptbn2
ENSMUSG00000067916.10	132.210912	0.05607704	0.0081601	38.8102298	4.67E-10	6.79E-09	Zfp991
ENSMUSG00000067919.9	2.26657332	-0.0014399	0.02533285	-0.0070744	1	1	Rex2
ENSMUSG00000067924.5	107.457493	0.00965564	0.00344137	7.87291453	0.00501807	0.01774722	Rtl8b
ENSMUSG00000067925.5	90.5775134	0.00486769	0.00371053	1.75963272	0.18467033	0.33704515	Rtl8a
ENSMUSG00000067928.7	123.268265	0.0087767	0.00301922	8.32992085	0.00389974	0.01429802	Zfp760
ENSMUSG00000067931.7	95.6480998	0.00985521	0.00315354	9.49670808	0.00205841	0.00828351	Zfp948
ENSMUSG00000067942.7	299.519681	0.00126978	0.00401463	0.09837981	0.75378266	0.88616719	Zfp160
ENSMUSG00000067951.6	0.98813368	-0.0135489	0.02957448	0.35383681	0.5519492	0.73429262	Vmn1r227
ENSMUSG00000067995.5	169.918263	-0.0028652	0.00226705	1.60558748	0.20511318	0.36530993	Gtf2f2
ENSMUSG00000068011.5	357.407796	0.01091997	0.00358564	9.3648779	0.00221183	0.00882113	Mkrn2os
ENSMUSG00000068015.9	227.414063	0.00774911	0.00491709	2.45724228	0.11698401	0.23745136	Lrch1
ENSMUSG00000068036.17	3073.12596	-0.0030498	0.00443425	0.46131676	0.49700922	0.68420356	Afdn
ENSMUSG00000068037.12	3.42794225	0.015928	0.0118671	1.4000898	0.23670854	0.40581899	Mas1
ENSMUSG00000068039.14	2637.70958	-0.0085056	0.00204369	17.0809643	3.58E-05	0.0002268	Tcp1
ENSMUSG00000068040.11	856.270734	0.00038441	0.00239855	0.02566152	0.87272971	0.96017366	Tm9sf4
ENSMUSG00000068079.6	7.07977797	-0.0350476	0.01919681	4.46134264	0.03467024	0.08962431	Tcf15
ENSMUSG00000068083.3	1105.66864	-0.0366345	0.00526732	47.8939933	4.50E-12	8.70E-11	Cyp2d40
ENSMUSG00000068085.7	6.91845571	-0.0429483	0.00637511	46.8478892	7.67E-12	1.44E-10	Cyp2d11
ENSMUSG00000068086.7	14378.3366	-0.058213	0.0066143	71.9593835	2.20E-17	8.37E-16	Cyp2d9
ENSMUSG00000068101.12	29.2047608	0.01061449	0.00753198	1.90483612	0.167538	0.31252383	Cenpm
ENSMUSG00000068105.12	15.1985685	0.10005035	0.00983106	79.6335535	4.51E-19	2.06E-17	Tnfrsf13c
ENSMUSG00000068114.8	77.2870554	0.00611428	0.00490201	1.55524357	0.21236301	0.37465735	Ccdc134
ENSMUSG00000068115.14	32.9247517	0.0185684	0.00680416	7.00569714	0.00812507	0.0267576	Ninl
ENSMUSG00000068129.6	5.066934	0.14229691	0.0121623	107.204854	4.01E-25	3.79E-23	Cst7
ENSMUSG00000068130.13	27.9597592	0.00772025	0.0053344	2.00869774	0.15639952	0.29662585	Zfp442
ENSMUSG00000068134.15	307.715719	0.0049256	0.00287422	2.82786817	0.09264126	0.19795586	Zfp120
ENSMUSG00000068154.6	11.1487393	0.03049668	0.01956817	3.31312753	0.06872857	0.15591345	Insm1
ENSMUSG00000068167.7	1.26549123	-0.008563	0.04658397	0.0149297	0.90275079	0.97702372	Csnka2ip
ENSMUSG00000068184.8	771.659255	-0.0150892	0.00355692	18.0242611	2.18E-05	0.00014466	Ndufaf2
ENSMUSG00000068196.6	29.9213953	-0.0105354	0.00739846	2.02035285	0.15520307	0.29478335	Col8a1
ENSMUSG00000068205.15	80.5733396	-0.0094655	0.00370341	6.59365291	0.0102343	0.03251867	Macrod2
ENSMUSG00000068206.14	275.850216	-0.0125733	0.0023142	29.2568235	6.34E-08	6.76E-07	Pick1
ENSMUSG00000068220.7	631.529049	0.04334806	0.00750697	27.4085705	1.65E-07	1.65E-06	Lgals1
ENSMUSG00000068227.10	58.7939829	0.05392568	0.00542954	92.094672	8.26E-22	5.32E-20	Il2rb
ENSMUSG00000068231.6	2.86377919	-0.0015275	0.01798805	-0.0060669	1	1	Vmn1r43
ENSMUSG00000068240.6	644.50045	0.02100477	0.00539047	15.0244775	0.00010613	0.00060315	Uba52rt
ENSMUSG00000068245.15	90.8326603	0.02417383	0.00503385	22.6320466	1.96E-06	1.61E-05	Phf11d
ENSMUSG00000068246.7	2189.09673	0.02051236	0.00527774	15.4312159	8.56E-05	0.00049658	Apol9b
ENSMUSG00000068250.14	432.782492	0.00660771	0.00421512	2.48784639	0.11472862	0.23365765	Amn1
ENSMUSG00000068252.7	2.93462258	-0.0052848	0.01456961	0.13884291	0.70943404	0.85624353	Apol7b
ENSMUSG00000068263.12	12.2984913	0.00658493	0.00534064	1.49444203	0.22152853	0.386634	Efcc1
ENSMUSG00000068264.12	267.931804	0.00451834	0.00296673	2.30732145	0.12876577	0.25601193	Ap5s1
ENSMUSG00000068267.6	1282.45201	0.0065153	0.00220514	8.62551466	0.00331487	0.01244976	Cenpb
ENSMUSG00000068270.16	29.140327	0.0045476	0.00530099	0.71825737	0.39671612	0.58848719	Shroom4
ENSMUSG00000068284.15	927.368553	-0.0113752	0.00367329	9.36436335	0.00221245	0.00882211	Usf3
ENSMUSG00000068290.12	2126.86769	0.00422704	0.00369992	1.30302598	0.25366123	0.42596666	Ddrgk1
ENSMUSG00000068299.12	130.64069	0.01000957	0.00312992	10.3181093	0.00131731	0.00558514	Nat8f4

ENSMUSG00000068323.13	3.97718088	0.01459353	0.0115759	1.42636404	0.23235875	0.40020545	Slc4a5
ENSMUSG00000068328.11	2065.47241	-0.0070112	0.0031326	5.03522136	0.02483691	0.06808335	Aup1
ENSMUSG00000068329.13	213.261208	-8.84E-05	0.00284865	0.00097625	0.9750742	1	Htra2
ENSMUSG00000068335.7	43.3502858	0.03795552	0.0057719	40.4648098	2.00E-10	3.07E-09	Dok1
ENSMUSG00000068373.16	6.84968083	0.02531075	0.01488253	3.12627704	0.07703949	0.17108119	D430041D05Rik
ENSMUSG00000068391.9	305.134929	-0.0029524	0.00303199	0.93792156	0.33281294	0.51974614	Chrac1
ENSMUSG00000068392.4	6.54697167	-0.0598359	0.00838807	47.8101089	4.70E-12	9.05E-11	Rnase13
ENSMUSG00000068394.5	64.0511706	0.00766415	0.00401816	3.73792856	0.05319035	0.12679882	Cep152
ENSMUSG00000068417.9	12.7683629	0.01256612	0.0071068	3.08114176	0.07920491	0.17475462	Pnp2
ENSMUSG00000068452.9	2.00812295	0.03390677	0.01966303	3.82792927	0.05040517	0.12135173	Duox2
ENSMUSG00000068457.15	280.548018	-0.0037547	0.00347304	1.15106163	0.28332702	0.46335815	Uty
ENSMUSG00000068479.6	374.790082	0.00048878	0.00207241	0.05452465	0.81536918	0.92471498	Mfap1a
ENSMUSG00000068522.5	22.9028083	0.00593678	0.00362795	2.66180784	0.10278385	0.21455527	Aard
ENSMUSG00000068523.13	1431.92568	-0.0015478	0.00236953	0.42451459	0.5146929	0.70123446	Gng5
ENSMUSG00000068551.13	364.645218	-0.0119484	0.00357714	11.2638245	0.00079032	0.00357509	Zfp467
ENSMUSG00000068566.14	700.405341	0.00642161	0.00366661	2.98876187	0.08384425	0.18288639	Myadm
ENSMUSG00000068580.12	330.467353	-0.003822	0.00393566	0.94498729	0.33099853	0.51790708	Zfyve19
ENSMUSG00000068587.11	2644.52938	-0.0042776	0.00510342	0.69250737	0.40531284	0.59695945	Mgam
ENSMUSG00000068606.7	24.6327832	0.05244192	0.00881984	32.0253269	1.52E-08	1.79E-07	Gm4841
ENSMUSG00000068614.8	1046.90396	0.00673911	0.01660326	-2.2278953	1	1	Actc1
ENSMUSG00000068647.3	1.02551313	0.00589733	0.04129951	0.05274259	0.81835788	0.92676761	Or4f54
ENSMUSG00000068663.15	329.337982	-0.0124693	0.0033711	13.4480603	0.00024526	0.00127719	Clec16a
ENSMUSG00000068686.14	73.4311306	-0.0168461	0.0046643	12.3773529	0.00043457	0.00211119	Cd59b
ENSMUSG00000068696.7	8.64612841	0.0436149	0.014927	9.05292923	0.00262274	0.01018943	Gpr88
ENSMUSG00000068697.8	1.33226706	0.03550063	0.01866524	3.85613429	0.0495644	0.11969141	Myoz1
ENSMUSG00000068699.13	36.3943913	-0.01246	0.01307092	0.80472039	0.36968579	0.56020538	Flnc
ENSMUSG00000068732.6	582.088331	0.0035503	0.00173364	4.1584726	0.04142675	0.10352736	Tmem167b
ENSMUSG00000068735.15	166.932852	0.01837621	0.00352642	26.7173831	2.35E-07	2.29E-06	Trp53i11
ENSMUSG00000068739.14	986.648723	-0.0033836	0.00276925	1.4822633	0.22342074	0.38894957	Sars
ENSMUSG00000068740.15	39.1292348	0.00214051	0.00992885	0.05927073	0.80765232	0.9204333	Celsr2
ENSMUSG00000068742.12	577.852832	0.00853525	0.00366939	5.45147976	0.01955176	0.05583579	Cry2
ENSMUSG00000068744.13	6.84209873	0.05055594	0.01145349	16.7193907	4.33E-05	0.00027019	Psrc1
ENSMUSG00000068745.7	2.63530386	-0.0206185	0.04530859	0.19482602	0.65892995	0.82029538	Mybphl
ENSMUSG00000068747.15	346.886549	-0.0215781	0.00448611	22.911	1.70E-06	1.41E-05	Sort1
ENSMUSG00000068748.8	17.0132539	0.02236627	0.01226057	3.41280022	0.06469258	0.14864483	Ptprz1
ENSMUSG00000068749.10	1559.88375	-0.0110329	0.00253195	19.0100615	1.30E-05	9.02E-05	Psma5
ENSMUSG00000068758.9	38.7225801	0.01329662	0.00515433	6.74871979	0.0093815	0.03027805	Il3ra
ENSMUSG00000068762.12	1658.62113	-0.0017277	0.00435117	0.15644163	0.69245398	0.84452244	Gstm6
ENSMUSG00000068794.8	3.84134985	-0.0093286	0.0160611	0.29992741	0.58392793	0.7612494	Col28a1
ENSMUSG00000068798.11	1573.74967	-0.0014617	0.002436	0.35290784	0.55247167	0.73469761	Rap1a
ENSMUSG00000068823.11	5266.8186	0.00018476	0.00276364	0.00434218	0.94746125	1	Csde1
ENSMUSG00000068854.8	57.7614737	-0.0117773	0.00364801	10.4594649	0.00122023	0.00523563	H2bc21
ENSMUSG00000068855.4	17.6680028	-0.0072722	0.01756016	0.12691573	0.7216516	0.86435479	H2ac20
ENSMUSG00000068856.4	277.787404	-0.0004824	0.00307871	0.02442415	0.87581053	0.96175849	Sf3b4
ENSMUSG00000068859.6	2.06343579	0.01443775	0.01839536	1.07092917	0.30073566	0.48513449	Sp9
ENSMUSG00000068874.14	5359.21723	0.00670654	0.00257637	6.90162074	0.00861176	0.02814524	Selenbp1
ENSMUSG00000068876.17	597.621019	-0.0106041	0.00408591	6.55565237	0.01045515	0.03310002	Cgn
ENSMUSG00000068877.13	5694.45767	-0.0966532	0.00864002	116.507523	3.68E-27	4.67E-25	Selenbp2
ENSMUSG00000068882.14	1319.40823	-0.000276	0.00262329	0.01095188	0.91665254	0.98437449	Ssb
ENSMUSG00000068893.6	14.454353	0.04441405	0.01296978	10.8504606	0.00098771	0.00434549	Sprr2a2
ENSMUSG00000068917.13	252.201244	-0.0014194	0.00285267	0.24798981	0.61849408	0.7893832	Clk2
ENSMUSG00000068921.15	566.147738	-0.0006888	0.0027213	0.06462515	0.79932964	0.91550472	Dap3
ENSMUSG00000068922.15	126.220297	0.00265189	0.00241413	1.22401578	0.26857399	0.4448133	Msto1
ENSMUSG00000068923.15	88.1856649	0.00630773	0.00576121	1.12318727	0.28923319	0.4704078	Syt11
ENSMUSG00000068959.7	31.1834037	0.01198808	0.00401191	8.74243579	0.00310889	0.01176798	Zfp619
ENSMUSG00000068962.8	3.60580648	-0.001738	0.00930322	0.03507877	0.8514307	0.94841998	Zfp114
ENSMUSG00000068966.11	93.5217703	0.0048432	0.00379246	1.61465365	0.20383887	0.36358948	Zbtb34
ENSMUSG00000069020.10	272.516703	-0.0021946	0.00330376	0.44662329	0.50394288	0.69092581	Urm1
ENSMUSG00000069041.9	2.08214227	0.00397322	0.01397902	0.07880681	0.77892094	0.90278277	Slc25a31
ENSMUSG00000069045.12	1273.43485	-0.0062562	0.0035498	3.04690345	0.08089124	0.17771057	Ddx3y
ENSMUSG00000069049.12	868.602185	-0.0092771	0.00270451	11.5330301	0.0006837	0.00314897	Eif2s3y

ENSMUSG00000069072.10	12.0870227	0.01452933	0.01405931	1.01463896	0.31379405	0.49913315	Slc7a14
ENSMUSG00000069089.9	264.30871	-0.0105292	0.00235042	20.1666017	7.10E-06	5.21E-05	Cdk7
ENSMUSG00000069094.13	151.268894	0.01525336	0.0048549	9.45979361	0.00210025	0.0084346	Pde7a
ENSMUSG00000069114.9	96.7530947	-0.0052806	0.00335764	2.45056761	0.11748238	0.23814294	Zbtb10
ENSMUSG00000069117.5	45.6482474	-0.0009147	0.01060963	-0.0005429	1	1	Rps18-ps6
ENSMUSG00000069135.12	343.837327	-0.0093997	0.00203653	21.3117072	3.90E-06	3.02E-05	Cep43
ENSMUSG00000069170.15	57.9278425	-0.0047474	0.00542468	0.68784369	0.40689879	0.59851044	Adgrv1
ENSMUSG00000069171.15	60.1829842	0.0129756	0.00687853	3.26784238	0.07065049	0.1593995	Nr2f1
ENSMUSG00000069184.5	71.3851833	0.00729417	0.00368217	3.81190668	0.05088949	0.12230278	Zfp72
ENSMUSG00000069206.15	628.171279	-0.0070196	0.00285803	5.92254317	0.01494837	0.04473131	Zfp874a
ENSMUSG00000069208.10	356.996337	-0.0090141	0.00275872	10.5005632	0.00119338	0.00512978	Zfp825
ENSMUSG00000069227.5	6.41454583	0.01651399	0.02071794	0.87518115	0.34952493	0.53769208	Gprin1
ENSMUSG00000069237.9	1284.2195	0.00094027	0.00227022	0.16600922	0.68368317	0.8382031	Fam8a1
ENSMUSG00000069255.14	378.850301	-0.0020287	0.00258518	0.60885016	0.43522107	0.62683619	Dusp22
ENSMUSG00000069265.3	4.9640413	-0.0009771	0.01756613	-0.01188	1	1	H3c1
ENSMUSG00000069266.6	7.80504365	-0.002415	0.01777759	-0.0006259	1	1	H4c2
ENSMUSG00000069267.3	7.14736495	-0.0120287	0.01998626	0.33421142	0.56318971	0.74387593	H3c2
ENSMUSG00000069268.5	1.82654742	-0.0078783	0.02528143	0.18077488	0.67070816	0.82877326	H2bc7
ENSMUSG00000069270.7	10.9077402	0.01824033	0.00758318	5.1505555	0.02323894	0.06443846	H2ac6
ENSMUSG00000069272.7	5.08934241	-0.0142224	0.00978236	1.95395895	0.16216087	0.30455218	H2ac8
ENSMUSG00000069273.3	15.8681225	-0.0103185	0.01555581	0.385769	0.53453182	0.71886575	H3c6
ENSMUSG00000069274.4	8.42623699	0.00423524	0.02675759	0.09565421	0.75710843	0.88815077	H4c6
ENSMUSG00000069280.3	1.32311934	0.00103814	0.04073443	0.0132883	0.90822725	0.9800179	Vmn1r223
ENSMUSG00000069289.3	3.91255084	0.00553161	0.01614496	0.21981473	0.63918112	0.80547676	Vmn1r203
ENSMUSG00000069292.5	0.7113899	-0.0050107	0.05205089	-0.0207277	1	1	Vmn1r199
ENSMUSG00000069295.3	1.71913194	-0.0041785	0.03319	0.00931161	0.92312612	0.98816701	Vmn1r196
ENSMUSG00000069299.3	1.29714005	-0.0098848	0.02656133	0.21847913	0.6402012	0.80654664	Vmn1r188
ENSMUSG00000069300.4	3.18050201	0.01856358	0.01828473	0.93456311	0.33368001	0.52054875	H2bc11
ENSMUSG00000069301.6	1.42827176	0.02298776	0.03920594	0.79744438	0.37185856	0.56243786	H2ac11
ENSMUSG00000069302.6	0.9107546	0.00156635	0.03867918	0.00436472	0.94732525	1	H2ac12
ENSMUSG00000069303.8	2.38884444	-0.0011113	0.02550539	-0.0225428	1	1	H2bc24
ENSMUSG00000069305.4	6.1555742	-0.0435825	0.02273099	4.41628056	0.03559755	0.09149945	H4c18
ENSMUSG00000069306.6	13.8325714	-0.038696	0.01881744	1.60642568	0.20499497	0.36518213	H4c17
ENSMUSG00000069307.8	2.84969808	0.01458462	0.0168429	0.91241263	0.33947509	0.52711385	H2bc23
ENSMUSG00000069309.3	0.64469179	0.0002548	0.0712165	-0.0005633	1	1	H2ac22
ENSMUSG00000069310.5	3.49854368	0.02922171	0.02176585	2.20557226	0.13751285	0.26911535	H3c3
ENSMUSG00000069324.7	975.267694	-0.0085304	0.00699456	1.42279693	0.23294359	0.40094925	Bhmt1b
ENSMUSG00000069378.15	7.90869365	-0.0103196	0.01108093	0.87125252	0.35060879	0.53900788	Prdm6
ENSMUSG00000069441.4	1.90741572	0.03556577	0.01412066	7.26391961	0.00703536	0.0237153	Dsg1a
ENSMUSG00000069456.5	2410.79658	0.01506687	0.00564258	6.92466574	0.00850149	0.02782241	Rdh16
ENSMUSG00000069476.14	0.99057039	0.00692325	0.03820048	-0.0211963	1	1	Zfp616
ENSMUSG00000069495.13	280.777578	-0.0008129	0.00285762	0.07884067	0.77887469	0.90278089	Epc2
ENSMUSG00000069515.7	11.1069934	0.05234473	0.01075073	21.9314166	2.83E-06	2.25E-05	Lyz1
ENSMUSG00000069516.9	3691.04418	0.06361865	0.00585038	107.040191	4.36E-25	4.10E-23	Lyz2
ENSMUSG00000069520.7	2168.71589	0.01073092	0.00325451	10.3982838	0.00126132	0.00538942	Tmem19
ENSMUSG00000069539.12	613.295299	-0.0032775	0.00205302	2.52841153	0.11181273	0.2290641	Scyl2
ENSMUSG00000069565.13	574.55811	-0.0018318	0.00259027	0.50516262	0.47724045	0.66728444	Dazap1
ENSMUSG00000069581.13	1.97658653	0.01111021	0.01021951	1.20413395	0.272497	0.44982602	Tspear
ENSMUSG00000069601.15	546.268564	0.02022955	0.00441554	21.0630041	4.44E-06	3.40E-05	Ank3
ENSMUSG00000069607.5	23.780678	0.04459852	0.01092943	13.1864372	0.00028198	0.00144552	Cd300ld3
ENSMUSG00000069609.8	1.33750086	0.05276832	0.03518975	2.01338267	0.15591734	0.29592551	Cd300ld4
ENSMUSG00000069631.15	58.9697424	0.01736008	0.0037465	21.0082061	4.57E-06	3.48E-05	Strada
ENSMUSG00000069633.13	741.688729	-0.0086191	0.00374923	5.28196852	0.02154737	0.06052338	Pex11g
ENSMUSG00000069662.6	639.828747	0.03495051	0.00415189	66.6543644	3.24E-16	1.05E-14	Marcks
ENSMUSG00000069668.5	204.375439	0.15742976	0.01746218	62.6634434	2.45E-15	7.23E-14	Sult3a1
ENSMUSG00000069670.10	9.1816416	-0.0038608	0.00874955	0.18402522	0.66793661	0.82650191	Nkain2
ENSMUSG00000069678.11	79.4658102	-0.0003128	0.00380451	0.00688144	0.93388778	0.99389393	Pcgf1
ENSMUSG00000069727.6	97.1465511	-0.0017173	0.00297723	0.33512107	0.56265907	0.74359146	Zfp975
ENSMUSG00000069729.15	504.519456	-0.0025337	0.00350187	0.51740389	0.47195122	0.66271964	Arid1b
ENSMUSG00000069733.12	62.5399366	-0.0100491	0.00670992	2.17895239	0.13990967	0.27274233	Ube2u
ENSMUSG00000069743.6	77.4560375	0.02570015	0.0039868	40.644882	1.83E-10	2.81E-09	Zfp820

ENSMUSG00000069744.8	1365.6807	-0.0082216	0.00295203	7.84798856	0.00508773	0.01795934	Psmb3
ENSMUSG00000069763.4	17.7465403	0.01105959	0.00752903	2.21133441	0.13700012	0.26835685	Tmem100
ENSMUSG00000069769.14	487.265288	0.00319201	0.00280982	1.25086232	0.26338784	0.43825507	Msi2
ENSMUSG00000069792.6	811.496384	0.06851507	0.00670805	92.5628772	6.52E-22	4.30E-20	Wfdc17
ENSMUSG00000069793.13	106.493396	0.02650783	0.0060703	19.038665	1.28E-05	8.89E-05	Slfn9
ENSMUSG00000069804.3	6.72348467	-0.002732	0.01351545	-0.0208633	1	1	Gm10277
ENSMUSG00000069805.11	15893.6906	-0.0023841	0.00436169	0.2986914	0.58470396	0.76179792	Fbp1
ENSMUSG00000069806.6	23.193653	0.02130683	0.00804958	6.56148739	0.01042092	0.03301825	Cacng7
ENSMUSG00000069808.14	62.0311288	0.00048849	0.00282565	0.03011997	0.8622183	0.95426216	Tlcd3a
ENSMUSG00000069814.7	3.22325832	0.03456437	0.00983977	11.8200574	0.00058596	0.00274706	Ccdc92b
ENSMUSG00000069818.7	0.98818916	-0.0049502	0.03656156	-0.0525593	1	1	Or1e30
ENSMUSG00000069825.13	6.34040043	-0.0345663	0.00918246	13.772653	0.00020632	0.00109649	Gm49340
ENSMUSG00000069830.11	4.072638	0.04596451	0.0092445	23.5447565	1.22E-06	1.04E-05	Nlrp1a
ENSMUSG00000069833.14	926.732718	0.00155532	0.0046873	0.11086652	0.73915977	0.87615953	Ahnak
ENSMUSG00000069835.11	146.828958	-0.0024949	0.00293258	0.73604889	0.39092951	0.582287	Sat2
ENSMUSG00000069844.13	228.107946	-0.0051743	0.00222233	5.34070428	0.02083305	0.05891708	Sco1
ENSMUSG00000069855.10	2.34000016	0.05066728	0.01032369	20.8718433	4.91E-06	3.72E-05	Slc47a2
ENSMUSG00000069874.8	1217.47463	0.03208718	0.00392149	65.8488281	4.87E-16	1.56E-14	Irgm2
ENSMUSG00000069892.10	147.152043	0.04952273	0.00618258	57.6040425	3.21E-14	8.24E-13	9930111J21Rik2
ENSMUSG00000069893.11	41.6539225	0.05390257	0.00830973	39.1372069	3.95E-10	5.81E-09	9930111J21Rik1
ENSMUSG00000069895.5	414.382111	-0.0037798	0.00492873	0.57410493	0.44863232	0.64052312	Atxn1l
ENSMUSG00000069899.4	38.1540557	0.00916324	0.00635057	2.11779068	0.14559644	0.28117888	Sft2d1rt
ENSMUSG00000069910.3	9.70018814	0.02600579	0.00969125	6.75931164	0.00932597	0.03012771	Spdl1
ENSMUSG00000069911.13	1.63424729	0.02680714	0.01620985	3.56341898	0.05906596	0.13812454	Insyn2b
ENSMUSG00000069917.8	3090.06206	0.01158114	0.00720765	2.34883117	0.12537698	0.25064757	Hba-a2
ENSMUSG00000069919.8	2582.42427	0.01108763	0.00722534	2.13079933	0.14436546	0.27944224	Hba-a1
ENSMUSG00000069920.11	19.836819	0.01638518	0.0051048	9.98450752	0.00157863	0.00655403	B3gnt9
ENSMUSG00000069922.13	25935.1929	-0.043435	0.00588837	52.3781276	4.58E-13	1.01E-11	Ces3a
ENSMUSG00000069971.6	1.60582115	-0.0099701	0.12943702	0.00209923	0.96345584	1	4933402J07Rik
ENSMUSG00000070000.14	16.0795425	0.08630766	0.00928011	69.3426064	8.28E-17	2.91E-15	Fcho1
ENSMUSG00000070002.8	497.187738	-0.0021955	0.00265584	0.66063311	0.41633652	0.60753133	Ell
ENSMUSG00000070003.8	100.684367	0.03840757	0.0058403	41.4239338	1.23E-10	1.94E-09	Ssbp4
ENSMUSG00000070031.12	137.902661	0.05322674	0.00558201	81.7270613	1.56E-19	7.73E-18	Sp140
ENSMUSG00000070034.14	95.1735828	0.05502865	0.00501299	111.494451	4.61E-26	4.99E-24	Sp110
ENSMUSG00000070044.16	492.619796	-0.0040301	0.00269399	2.25163434	0.13347337	0.26306534	Fam149a
ENSMUSG00000070047.15	811.06099	-0.001528	0.00394833	0.14568664	0.70269192	0.85165054	Fat1
ENSMUSG00000070056.7	508.872769	-0.0159464	0.0035929	19.6233765	9.43E-06	6.72E-05	Mfhas1
ENSMUSG00000070283.5	177.3137	-0.0057381	0.00346684	2.80236206	0.09412555	0.2005642	Ndufaf3
ENSMUSG00000070284.11	366.461614	-0.0037266	0.004929	0.56159494	0.45361833	0.64529806	Gmppb
ENSMUSG00000070287.4	13.9234894	0.00915866	0.00716991	1.57759551	0.20910738	0.37065507	Slc35g2
ENSMUSG00000070304.14	21.3950444	0.05500901	0.0120679	18.7038528	1.53E-05	0.00010468	Scn2b
ENSMUSG00000070305.11	169.775606	-0.0040272	0.0025286	2.51375826	0.1128565	0.23066147	Mpzl3
ENSMUSG00000070306.4	0.92055795	-0.0144631	0.04228082	0.06655627	0.79641847	0.91399017	Ccdc153
ENSMUSG00000070319.7	894.514107	-0.0045928	0.00214955	4.62246088	0.03155593	0.0829187	Eif3g
ENSMUSG00000070323.12	29.9865984	0.08746437	0.00809234	99.6297249	1.84E-23	1.43E-21	Mmp27
ENSMUSG00000070327.16	1716.92934	0.01957626	0.00438301	20.2394225	6.83E-06	5.03E-05	Rnf213
ENSMUSG00000070331.15	2.60096002	0.01311798	0.01919371	-0.2730299	1	1	Qrich2
ENSMUSG00000070332.5	19.1531214	0.02975561	0.00620492	22.3979248	2.22E-06	1.80E-05	Trim80
ENSMUSG00000070336.4	8.7424722	0.01328636	0.00583694	4.96505413	0.02586451	0.07045158	Fbxo47
ENSMUSG00000070337.5	2.12868793	-0.0006174	0.01688298	0.00253713	0.95982762	1	Gpr179
ENSMUSG00000070345.4	4.4953375	-0.0146335	0.00912303	2.45215186	0.11736388	0.23795732	Hsf5
ENSMUSG00000070348.6	995.146036	0.0417009	0.00574693	50.369684	1.27E-12	2.63E-11	Ccnd1
ENSMUSG00000070354.15	15.1691906	0.05451581	0.00783205	44.5401307	2.49E-11	4.33E-10	Evi2
ENSMUSG00000070360.6	2.63023035	-0.0403685	0.09592737	0.27237875	0.60174053	0.77530954	Amy2a1
ENSMUSG00000070368.10	62.054375	-0.0507352	0.00714716	48.417875	3.44E-12	6.80E-11	Prok1
ENSMUSG00000070369.14	3.73161363	0.11126237	0.01739941	38.3042974	6.05E-10	8.66E-09	Itgad
ENSMUSG00000070371.12	97.9158929	-0.0249927	0.00580962	17.9695497	2.24E-05	0.0001483	Prss36
ENSMUSG00000070372.12	891.465712	0.00307106	0.0030693	0.98527357	0.32090031	0.50701733	Capza1
ENSMUSG00000070390.13	19.2501863	0.06020841	0.0074866	60.3752475	7.84E-15	2.19E-13	Nlrp1b
ENSMUSG00000070394.11	2206.72273	-0.0145743	0.00626675	5.44994954	0.01956889	0.05587795	Tmem256
ENSMUSG00000070407.6	1754.70021	-0.0041383	0.00409725	1.00456893	0.31620748	0.50172012	Hs3st3b1

ENSMUSG00000070419.12	1.68305772	0.02328359	0.01260897	3.66695421	0.05550152	0.13149763	Cyp3a57
ENSMUSG00000070420.5	55.7681162	-0.0004177	0.00336237	0.01508725	0.90224158	0.97685334	Zscan25
ENSMUSG00000070423.7	3.02879964	-0.0047463	0.0224292	0.01122741	0.9156145	0.9838322	Or51e1
ENSMUSG00000070424.13	5.48913599	-0.0093325	0.02978747	-0.1308607	1	1	Art5
ENSMUSG00000070425.14	13.8726366	-0.0095764	0.00808555	1.42708305	0.23224108	0.40006573	Xntrpc
ENSMUSG00000070426.13	372.572536	-0.0072453	0.00223275	10.4670749	0.00121521	0.0052179	Rnf121
ENSMUSG00000070427.5	306.054501	0.04666775	0.00386616	140.326198	2.26E-32	6.42E-30	Il18bp
ENSMUSG00000070436.13	381.770565	0.00189453	0.00545316	0.11426065	0.73534462	0.87356897	Serpinh1
ENSMUSG00000070458.6	1.49446617	-0.0129953	0.0225006	0.36996574	0.54302294	0.72651535	Vmn2r73
ENSMUSG00000070459.7	1.66531623	0.005797	0.03883918	0.07720813	0.78111714	0.90419025	Or5ae1
ENSMUSG00000070462.5	118.21918	0.0135018	0.00311085	18.8380999	1.42E-05	9.81E-05	Tlnrd1
ENSMUSG00000070469.13	18.5753011	0.04538477	0.0105963	15.8058237	7.02E-05	0.00041628	Adamtsl3
ENSMUSG00000070473.5	1332.75715	0.00017203	0.00404106	0.0018307	0.96587154	1	Cldn3
ENSMUSG00000070476.11	27.0393869	0.04451894	0.00580624	56.3784038	5.98E-14	1.47E-12	Fam217b
ENSMUSG00000070493.4	9682.63051	-0.0204408	0.00497834	16.9999556	3.74E-05	0.00023612	Chchd2
ENSMUSG00000070495.13	12.411764	0.0209215	0.00614043	11.1283781	0.00085017	0.00380567	Ctcfl
ENSMUSG00000070498.4	12.4999586	0.02061907	0.01642249	1.50090421	0.22053229	0.38532295	Tmem132b
ENSMUSG00000070501.15	4.98182545	0.05096855	0.01076387	20.5707427	5.75E-06	4.29E-05	Ifi214
ENSMUSG00000070509.16	13.9840021	0.03144857	0.00818251	14.0042827	0.00018239	0.00098398	Rgma
ENSMUSG00000070520.5	280.26511	-0.0027289	0.00284681	0.91295788	0.33933082	0.52699393	Nsmce3
ENSMUSG00000070526.3	4.21409481	0.02000627	0.00628408	10.0256658	0.00154374	0.00643297	Peg12
ENSMUSG00000070527.4	9.29461951	0.02516742	0.00529465	22.1535651	2.52E-06	2.02E-05	Mkrn3
ENSMUSG00000070532.6	1.74592585	0.0586902	0.01143553	25.451613	4.54E-07	4.17E-06	Ccdc190
ENSMUSG00000070544.7	4024.34266	-0.0035666	0.00383192	0.84326501	0.35846464	0.54732879	Top1
ENSMUSG00000070547.5	1.61872341	0.06013463	0.02744604	4.45324408	0.03483501	0.08996152	Mrgprb1
ENSMUSG00000070565.12	288.07728	-0.0115991	0.00385645	9.09718703	0.00256003	0.00997866	Rasal2
ENSMUSG00000070568.13	6.3827809	-0.0202839	0.01225113	2.35126091	0.12518172	0.25031006	Slc6a21
ENSMUSG00000070570.6	22.4243851	0.03791383	0.01371328	7.69693321	0.00553147	0.01927964	Slc17a7
ENSMUSG00000070576.5	307.033233	-0.0107974	0.00451219	5.66925949	0.01726474	0.05040929	Mn1
ENSMUSG00000070583.2	26.5791205	-0.0064458	0.00497739	1.62386153	0.20255419	0.36173568	Fv1
ENSMUSG00000070594.11	5441.28373	-0.0025572	0.00552038	0.21157803	0.64553336	0.81043008	Cfhr4
ENSMUSG00000070601.6	1.7830469	0.02797893	0.02055056	2.81052785	0.09364756	0.19972613	Vmn2r84
ENSMUSG00000070604.6	20.079722	-0.0008363	0.00659107	0.01398825	0.90585219	0.97876356	Vsig10l
ENSMUSG00000070605.5	202.249944	0.03701542	0.00626398	28.3207984	1.03E-07	1.07E-06	Zfp992
ENSMUSG00000070639.6	45.6730308	0.01068517	0.00577265	3.39535639	0.06538023	0.14994731	Lrrc8b
ENSMUSG00000070643.12	119.873285	-0.0089151	0.00327736	7.26819909	0.00701861	0.02366562	Sox13
ENSMUSG00000070644.14	4555.34854	-0.0053984	0.00496004	1.19649359	0.27402362	0.45174538	Etnk2
ENSMUSG00000070645.6	1.29871056	-0.0036201	0.02258494	-0.0173692	1	1	Ren1
ENSMUSG00000070661.8	140.580505	0.00598426	0.0049895	1.36079715	0.24339938	0.41378788	Rnf186
ENSMUSG00000070687.12	1.08696504	-0.0074094	0.02342896	0.23286959	0.62940419	0.79802214	Htr1d
ENSMUSG00000070690.8	673.43234	-0.0038116	0.00453383	0.70204595	0.40209708	0.59372583	Albfm1
ENSMUSG00000070691.11	21.9331481	0.08362114	0.01006494	60.1248991	8.90E-15	2.46E-13	Runx3
ENSMUSG00000070695.5	6.87177572	0.02405114	0.0188705	1.94933392	0.16265864	0.30532963	Cntnap5a
ENSMUSG00000070697.5	563.346284	-0.0075381	0.00225703	11.1174493	0.00085519	0.00382308	Utp3
ENSMUSG00000070699.6	141.607306	-0.0134273	0.00229257	34.6815172	3.88E-09	4.99E-08	Sars2
ENSMUSG00000070704.11	14984.2276	-0.0126333	0.00478879	6.90131008	0.00861326	0.02814524	Ugt2b36
ENSMUSG00000070705.4	63.1861983	0.0095515	0.00301904	9.6890451	0.0018537	0.00756163	Eid2b
ENSMUSG00000070709.13	70.573536	0.00676189	0.00379303	3.08783814	0.07887954	0.1741834	Zfp974
ENSMUSG00000070713.6	17.2920675	0.01621771	0.0069159	5.34024121	0.02083859	0.0589186	Hmgn2-ps
ENSMUSG00000070720.5	24.9313285	0.02012513	0.00521679	14.796185	0.00011978	0.00067312	Tmem200b
ENSMUSG00000070730.12	1116.13615	-0.0053404	0.00221427	5.7494748	0.01649359	0.0485241	Rmdn3
ENSMUSG00000070733.14	401.92909	0.00575139	0.00316291	3.26121052	0.07093674	0.15993825	Fryl
ENSMUSG00000070737.11	113.962896	0.00481119	0.00243904	3.80279625	0.05116707	0.12288223	Tmem35b
ENSMUSG00000070738.11	382.96331	0.0019283	0.00270097	0.50599785	0.47687651	0.66700254	Dgkd
ENSMUSG00000070780.12	1315.21885	-0.0029102	0.00421962	0.45545997	0.49975344	0.68698749	Rbm47
ENSMUSG00000070802.6	24.9075771	0.01882707	0.00934229	4.06936555	0.04366767	0.10806271	Pnma8b
ENSMUSG00000070803.7	7.72853624	0.0010574	0.00778008	0.02030772	0.886681	0.96789273	Cited4
ENSMUSG00000070806.6	2.50185107	0.02435634	0.00924773	7.0086553	0.00811166	0.02672089	Zmynd12
ENSMUSG00000070808.5	126.313243	0.00623757	0.00405284	2.31610456	0.12804032	0.25504256	Bicra
ENSMUSG00000070810.11	5.11078992	0.08743646	0.05912973	2.83895275	0.09200416	0.19693349	Sult2a6
ENSMUSG00000070811.7	824.743134	0.15979579	0.02033369	29.7314142	4.96E-08	5.39E-07	Sult2a2

ENSMUSG00000070814.18	84.9962857	0.01303864	0.00426889	9.03467263	0.00264906	0.01027308	Zswim9
ENSMUSG00000070817.4	1.1331135	0.0033288	0.02857858	0.0207949	0.88533909	0.96700971	Vmn1r85
ENSMUSG00000070822.6	5.78899341	0.03699258	0.00955116	12.8721183	0.00033351	0.00167241	Zscan18
ENSMUSG00000070847.14	1.50820001	0.01832687	0.01799221	1.36178478	0.2432284	0.41358122	Vmn2r30
ENSMUSG00000070855.9	2.11710006	0.00738926	0.02403423	0.25543243	0.61327565	0.78522987	Or10ag54
ENSMUSG00000070858.13	5.38054648	0.00015827	0.01100425	0.00015265	0.99014214	1	Gm1673
ENSMUSG00000070866.5	1.32608536	0.01765301	0.02535025	1.06291984	0.30255018	0.48716197	Zfp804a
ENSMUSG00000070867.5	40.8825245	0.00863752	0.00663603	1.66494229	0.19693734	0.35392673	Trabd2b
ENSMUSG00000070868.12	4.77229312	0.04178117	0.01041813	14.6960293	0.00012631	0.00070547	Skint3
ENSMUSG00000070871.11	110.839138	-0.0112658	0.00413021	7.41347025	0.00647373	0.02207771	Ccnyl1
ENSMUSG00000070873.6	104.123857	0.03551214	0.00481878	50.6036951	1.13E-12	2.35E-11	Lilra5
ENSMUSG00000070880.11	17.1309154	0.02158335	0.01614386	1.70385345	0.1917848	0.34675633	Gad1
ENSMUSG00000070883.4	18.9643754	0.00990966	0.00681951	2.04019841	0.15318966	0.29197022	Cfap210
ENSMUSG00000070923.5	1188.48079	0.00079579	0.00216109	0.13152072	0.71686048	0.86097726	Klhl9
ENSMUSG00000070934.7	576.125036	-0.0016036	0.00191263	0.69741234	0.40365455	0.59526049	Rraga
ENSMUSG00000070939.10	718.794499	-0.0023088	0.00256384	0.8116906	0.3676209	0.55792462	Tgfbrap1
ENSMUSG00000070942.9	10.790884	0.03368775	0.00712168	21.2132878	4.11E-06	3.17E-05	Il1rl2
ENSMUSG00000070953.14	188.173199	-0.0109003	0.00346073	10.048531	0.00152469	0.00636486	Rabepk
ENSMUSG00000070972.14	589.916867	-0.002623	0.003534	0.54978627	0.45840504	0.6501077	Dnajc25
ENSMUSG00000070985.4	482.812106	-0.0175428	0.0047059	13.6774926	0.00021704	0.00114753	Acnat1
ENSMUSG00000071001.5	4.46549844	0.01032425	0.00797142	1.616962	0.20351591	0.36320569	Hrct1
ENSMUSG00000071005.8	23.9759361	0.05200696	0.00758402	40.9911606	1.53E-10	2.39E-09	Ccl19
ENSMUSG00000071014.11	1894.68105	-0.0209674	0.00411237	26.1629654	3.14E-07	2.97E-06	Ndufb6
ENSMUSG00000071037.7	18.8896739	0.00493054	0.00487454	1.03906269	0.30803957	0.49296549	Camkmt
ENSMUSG00000071042.13	169.711921	0.00163207	0.00477275	0.11391316	0.7357323	0.87376546	Rasgrp3
ENSMUSG00000071047.6	3.08828024	-0.0162444	0.00945592	2.89900334	0.08863434	0.1911764	Ces1a
ENSMUSG00000071054.13	870.158516	-0.00928	0.00338409	7.5624965	0.00595954	0.02054094	Safb
ENSMUSG00000071064.16	18.7070748	0.0236078	0.00904782	6.32488366	0.01190553	0.03687509	Zfp827
ENSMUSG00000071068.8	12.6220866	0.05401683	0.0090361	29.2013509	6.52E-08	6.95E-07	Treml2
ENSMUSG00000071072.13	2300.08636	-0.0065349	0.00256273	6.24404977	0.01246112	0.03832416	Ptges3
ENSMUSG00000071073.6	2.17343157	-0.004212	0.00971235	0.19243038	0.66090149	0.82159984	Lrrc73
ENSMUSG00000071074.10	933.486961	-0.0131431	0.00418058	9.89498272	0.0016573	0.00683376	Yipf3
ENSMUSG00000071076.9	1169.65321	0.01154549	0.00346129	10.6687605	0.0010896	0.00473459	Jund
ENSMUSG00000071078.7	177.475446	-0.00392	0.00388275	1.01840785	0.31289697	0.49827749	Nr2c2ap
ENSMUSG00000071103.11	15.3027526	-0.0059774	0.00525213	1.29126549	0.25581487	0.42873939	1700029J07Rik
ENSMUSG00000071104.11	1.6059521	-0.0228479	0.06122696	0.15991055	0.68923889	0.84224904	Ccdc110
ENSMUSG00000071172.14	1667.87167	-0.0003762	0.00312428	0.01440842	0.90445536	0.97793089	Srsf3
ENSMUSG00000071176.11	119.200197	-0.0004973	0.00372791	0.01745675	0.89488622	0.97287826	Arhgef10
ENSMUSG00000071177.5	57544.0734	-0.0095857	0.00566364	2.83120057	0.09244922	0.19765295	Serpina1d
ENSMUSG00000071178.12	88220.2574	-0.0014158	0.00525989	0.07166623	0.78892602	0.90959619	Serpina1b
ENSMUSG00000071180.6	554.290687	-0.0020455	0.00306225	0.43944356	0.50739115	0.69424107	Smim15
ENSMUSG00000071192.7	6.74515767	-0.0036631	0.007038	0.26718071	0.60522928	0.77809538	Wfikkn1
ENSMUSG00000071203.7	74.9089074	0.05875095	0.00550936	108.211564	2.42E-25	2.35E-23	Naip5
ENSMUSG00000071226.12	398.886622	-0.0102566	0.00448393	5.23800924	0.02209855	0.06182141	Cecr2
ENSMUSG00000071252.7	79.4632964	-0.011633	0.00491641	5.38796879	0.02027606	0.0576321	2210408I21Rik
ENSMUSG00000071253.9	1288.28382	-0.0031222	0.00259322	1.43113971	0.23157853	0.39924063	Slc25a16
ENSMUSG00000071256.5	102.262866	-0.0012617	0.00248833	0.25788881	0.6115743	0.78377909	Zfp213
ENSMUSG00000071266.14	332.89105	0.00273295	0.0026293	1.06991965	0.3009636	0.48532202	Zfp946
ENSMUSG00000071267.12	253.154718	0.00044687	0.00267486	0.02724562	0.86889481	0.95812872	Zfp942
ENSMUSG00000071281.13	141.810176	-0.0007459	0.00327315	0.05061046	0.8220044	0.92896218	Zfp65
ENSMUSG00000071291.11	92.1998933	-0.0024146	0.00280091	0.73824834	0.39022258	0.5816716	Zfp58
ENSMUSG00000071302.11	179.067639	0.00219462	0.00401843	0.29210258	0.58887629	0.76566032	Zfp1006
ENSMUSG00000071311.3	0.72517362	0.08728782	0.02298771	16.0207959	6.27E-05	0.00037629	Gpr31b
ENSMUSG00000071317.5	10.1205952	0.05726507	0.01630124	8.56935026	0.00341869	0.01276853	Bves
ENSMUSG00000071322.13	1.55609127	0.00421672	0.03404974	0.03793176	0.84558023	0.94426255	Tcp10a
ENSMUSG00000071324.12	12.9802008	0.02174878	0.01118871	3.462966	0.06275765	0.1451036	Armc2
ENSMUSG00000071337.12	311.270945	0.01087075	0.00351458	9.65978806	0.00188345	0.00766964	Tia1
ENSMUSG00000071341.5	2.64515835	0.0061002	0.05273121	0.0594256	0.80740611	0.92019718	Egr4
ENSMUSG00000071342.6	18.7508848	-0.0207893	0.02282556	-0.6705642	1	1	Lsmem1
ENSMUSG00000071347.4	20.1171563	0.00282028	0.00787946	0.11492716	0.73460288	0.87312757	C1qtnf9
ENSMUSG00000071350.13	233.164825	-0.0028047	0.00294697	0.91013806	0.34007779	0.52780646	Setdb2

ENSMUSG00000071356.8	1.34675486	-0.0453816	0.03128221	0.71185022	0.39883027	0.59050914	Reg3b
ENSMUSG00000071359.14	205.936855	-0.007282	0.00315456	5.19715718	0.02262386	0.06305168	Tbpl1
ENSMUSG00000071369.12	603.289931	-0.0068493	0.0035232	3.67360095	0.05528063	0.13109279	Map3k5
ENSMUSG00000071379.3	184.722921	0.02603942	0.00309802	69.8898255	6.27E-17	2.24E-15	Hpcal1
ENSMUSG00000071398.9	1.41236933	0.00078419	0.01514424	0.00345	0.95316186	1	2410004P03Rik
ENSMUSG00000071415.7	4699.30955	-0.0006733	0.00267227	0.06324603	0.80143713	0.91658324	Rpl23
ENSMUSG00000071424.11	4.28804397	0.01799558	0.01829892	1.45902701	0.227085	0.39339161	Grid2
ENSMUSG00000071451.11	145.325926	0.00051845	0.00391876	0.01754544	0.89462109	0.97287826	Psmg4
ENSMUSG00000071454.14	378.286569	-0.0054863	0.00375433	2.12494776	0.14491772	0.28023525	Dtnb
ENSMUSG00000071470.5	12.8297119	0.00645448	0.02988955	0.0523069	0.81909663	0.92733845	Ccnb1ip1
ENSMUSG00000071477.11	140.477263	0.00418689	0.003112	1.79048562	0.18086701	0.33174657	Zfp777
ENSMUSG00000071478.7	3.04172151	-0.0054383	0.0209612	0.15473572	0.6940502	0.84563839	H2ac7
ENSMUSG00000071491.3	1.35210098	0.00751308	0.02695187	0.12607612	0.72253566	0.86519399	Vmn1r209
ENSMUSG00000071497.5	133.816234	0.01829202	0.00844905	4.61549745	0.0316843	0.0832282	Nutf2-ps1
ENSMUSG00000071506.5	4.79669738	-0.0007268	0.01043263	0.00461772	0.94582239	1	Tmem139
ENSMUSG00000071516.3	3.35532214	-0.0945475	0.0436597	-2.2268103	1	1	H2ac13
ENSMUSG00000071528.5	5431.0223	-0.0224879	0.00455943	24.4650798	7.57E-07	6.63E-06	Atp5mk
ENSMUSG00000071531.4	3.87932521	0.03540014	0.01270199	6.35271386	0.01172019	0.03638225	Gprin2
ENSMUSG00000071533.12	1487.88152	0.00145729	0.00164604	0.76844586	0.38069827	0.57221191	Pcnp
ENSMUSG00000071537.7	5.29839317	0.01861761	0.01270404	2.16427693	0.14125105	0.27469882	Klrg2
ENSMUSG00000071540.5	12.1873787	0.08664021	0.02417864	2.86238267	0.0906731	0.19449033	3425401B19Rik
ENSMUSG00000071547.5	95.4739508	-0.0057623	0.0040894	1.99528787	0.15778908	0.29851715	Nt5dc2
ENSMUSG00000071550.15	3.76818607	0.0370615	0.01682051	4.20722338	0.04025217	0.10114935	Cfap44
ENSMUSG00000071551.14	1894.31999	-0.009917	0.00479379	4.13493463	0.04200669	0.10466615	Akr1c19
ENSMUSG00000071552.6	3.27522724	0.16012325	0.01613092	78.2654303	9.01E-19	4.02E-17	Tigit
ENSMUSG00000071573.16	7.08239878	0.00675583	0.00637209	1.1231898	0.28923264	0.4704078	Rnls
ENSMUSG00000071604.6	23.7902127	-0.0112834	0.00556127	4.06397961	0.04380714	0.1083283	Entrep1
ENSMUSG00000071629.4	0.95750087	-0.003591	0.03872245	-0.0239352	1	1	Or5b120
ENSMUSG00000071632.11	427.262278	-0.0082684	0.00356835	5.28820516	0.02147034	0.06033592	2510002D24Rik
ENSMUSG00000071633.12	2045.58989	-0.0230221	0.00563266	16.1584174	5.83E-05	0.00035197	Gm4952
ENSMUSG00000071636.9	1.74186886	-0.0107129	0.02646528	0.04570497	0.830713	0.93462054	Rimbp3
ENSMUSG00000071637.6	124.479139	0.01013159	0.00488597	4.14267123	0.04181513	0.10431008	Cebpd
ENSMUSG00000071644.11	3269.57409	-0.0098028	0.00280396	12.2870381	0.00045612	0.00220269	Eef1g
ENSMUSG00000071645.7	240.295032	-0.0117438	0.00251142	21.8418321	2.96E-06	2.35E-05	Tut1
ENSMUSG00000071646.10	842.312123	0.00466343	0.00163297	8.14244112	0.0043241	0.01563758	Mta2
ENSMUSG00000071647.6	340.282043	-0.0028854	0.00278996	1.0778183	0.29918613	0.4831807	Eml3
ENSMUSG00000071648.5	39.8900035	0.00235715	0.00353943	0.43594365	0.50908681	0.69575394	Rom1
ENSMUSG00000071649.7	641.053651	-0.012431	0.00336609	13.5367387	0.00023394	0.00122506	B3gat3
ENSMUSG00000071650.7	3024.32408	-0.0102754	0.00223813	21.1904611	4.16E-06	3.20E-05	Ganab
ENSMUSG00000071652.9	217.692663	-0.0053714	0.00237387	5.21292916	0.02241953	0.06258576	Ints5
ENSMUSG00000071653.10	324.013392	-0.0104862	0.0031136	11.2822551	0.00078251	0.00354521	1810009A15Rik
ENSMUSG00000071654.7	333.935951	-0.0093765	0.00342649	7.65116134	0.00567358	0.01971667	Uqcc3
ENSMUSG00000071655.13	1777.99826	-0.0107212	0.00289562	13.8282305	0.0002003	0.00106862	Ubxn1
ENSMUSG00000071656.8	4.13660125	-0.0018363	0.01028557	0.02715325	0.86911524	0.95820882	Lrrn4cl
ENSMUSG00000071657.13	662.249823	-0.0082457	0.00244485	11.355404	0.00075229	0.00341878	Bscl2
ENSMUSG00000071658.6	6.32616111	0.0492812	0.01436462	11.1459256	0.00084216	0.003775	Gng3
ENSMUSG00000071659.5	1484.4574	0.00285413	0.00237457	1.43060407	0.23166588	0.39930367	Hnrnpul2
ENSMUSG00000071660.11	367.237569	0.00219811	0.00195328	1.24028152	0.2654168	0.44079149	Ttc9c
ENSMUSG00000071661.8	5.34159558	0.01679607	0.00705631	5.45847759	0.01947361	0.0556574	Zbtb3
ENSMUSG00000071662.5	228.754922	0.00186406	0.00407967	0.21292395	0.64448523	0.80984702	Polr2g
ENSMUSG00000071669.15	69.2680221	-0.0270697	0.00489659	30.8962528	2.72E-08	3.08E-07	Snx29
ENSMUSG00000071691.12	8.16338318	-0.0297093	0.00876074	10.4697374	0.00121346	0.00521134	Gm960
ENSMUSG00000071708.12	235.373261	0.0005514	0.00406597	0.01726361	0.89546595	0.97320224	Sms
ENSMUSG00000071711.13	1157.91916	-0.0179597	0.00365901	23.9122917	1.01E-06	8.67E-06	Mpst
ENSMUSG00000071713.7	198.403386	0.04252316	0.00564387	54.4876004	1.56E-13	3.64E-12	Csf2rb
ENSMUSG00000071714.8	58.4214545	0.06683705	0.00629872	103.716915	2.33E-24	2.05E-22	Csf2rb2
ENSMUSG00000071715.13	57.0492515	0.07170157	0.00711602	87.9044628	6.87E-21	3.90E-19	Ncf4
ENSMUSG00000071716.6	14.490568	0.09702659	0.00793334	128.057768	1.09E-29	2.11E-27	Apol7e
ENSMUSG00000071719.3	31.6671772	0.03945677	0.00974469	10.8805723	0.00097178	0.004285	Nalf2
ENSMUSG00000071722.5	11.9361784	-0.0016455	0.00551128	0.0871651	0.76781257	0.89540802	Spin4
ENSMUSG00000071723.8	11.9435394	-0.0085558	0.00707122	1.50465538	0.21995644	0.38463451	Gspt2

ENSMUSG00000071724.6	6.07209966	0.04003953	0.00948954	16.5530107	4.73E-05	0.00029248	Smpd5
ENSMUSG00000071745.2	1.8503164	0.00989231	0.01982644	0.31572622	0.57418742	0.75308817	Pwwp4b
ENSMUSG00000071748.3	16.0755445	0.0067526	0.01174726	0.3161514	0.57392974	0.75283393	Styx-ps
ENSMUSG00000071757.11	332.261011	-0.0078089	0.00304205	6.6060266	0.01016342	0.03233703	Zhx2
ENSMUSG00000071847.15	9.28619055	0.0257755	0.01214299	4.26544603	0.03889504	0.09826246	Apcdd1
ENSMUSG00000071855.6	11.166709	0.04293988	0.00989649	16.1874091	5.74E-05	0.00034761	Ccdc112
ENSMUSG00000071856.11	684.159891	-0.0085021	0.00415533	4.2269855	0.03978604	0.10017012	Mcc
ENSMUSG00000071862.4	7.88789532	0.00545642	0.01351659	0.18784244	0.66471843	0.82450954	Lrrtm2
ENSMUSG00000071866.13	13869.0055	-0.0009959	0.00265011	0.14295509	0.70536075	0.85373011	Ppia
ENSMUSG00000071893.8	8.19318853	-0.0111092	0.00612287	3.19182931	0.07400715	0.16557889	Vmn1r4
ENSMUSG00000071984.13	10.0025649	0.00188482	0.00966254	0.03705331	0.84735656	0.94548925	Fndc1
ENSMUSG00000072049.7	0.62637502	0.01230718	0.04795685	0.2701776	0.60321266	0.77647622	Vmn2r121
ENSMUSG00000072066.8	13.101054	0.02306322	0.00742859	8.83120283	0.00296123	0.01129968	Zfp1008
ENSMUSG00000072082.8	259.986541	-0.0216794	0.00395188	29.6481913	5.18E-08	5.60E-07	Ccnf
ENSMUSG00000072115.12	3558.93576	0.0113036	0.00500604	4.97950675	0.0256493	0.06997042	Ang
ENSMUSG00000072214.9	35.1400199	0.01690722	0.00710764	5.27815804	0.02159458	0.06061285	Septin5
ENSMUSG00000072235.7	293.317574	0.01758958	0.00621084	8.02613955	0.0046107	0.01647946	Tuba1a
ENSMUSG00000072244.12	7.97322102	0.01413035	0.00709952	3.83541908	0.05018045	0.12090673	Trim6
ENSMUSG00000072258.12	101.541026	-0.0063675	0.00251525	6.38998565	0.0114766	0.03578149	Taf1a
ENSMUSG00000072294.6	484.630189	0.00531056	0.00557972	0.8871374	0.34625423	0.53457827	Klf12
ENSMUSG00000072295.8	3.25606481	0.04514183	0.02872133	-0.9936153	1	1	C2cd6
ENSMUSG00000072437.5	7.69176149	0.02923886	0.01128534	6.44694986	0.0111143	0.03489641	Nanos1
ENSMUSG00000072494.8	63.7489227	0.00342452	0.00443964	0.58874828	0.44290356	0.63495766	Ppp1r3e
ENSMUSG00000072501.6	884.511736	-0.0073501	0.0028967	6.34603315	0.01176441	0.0364907	Phf20l1
ENSMUSG00000072568.5	251.284458	0.01545662	0.00421362	13.8679925	0.00019611	0.00104862	Lratd2
ENSMUSG00000072571.4	4.78680255	-0.0117437	0.01252578	0.8624867	0.35304374	0.5417014	Tmem253
ENSMUSG00000072572.10	24.20228	-0.0233388	0.00651998	13.0359672	0.00030557	0.00155057	Slc39a2
ENSMUSG00000072582.10	350.839646	-0.0064132	0.0027372	5.48481399	0.01918236	0.05496722	Ptrh2
ENSMUSG00000072596.5	65.0101305	0.07383919	0.0059554	140.46096	2.11E-32	6.07E-30	Ear2
ENSMUSG00000072620.4	146.243213	0.05750087	0.00618328	78.4831137	8.07E-19	3.61E-17	Slfn2
ENSMUSG00000072623.7	74.4623155	-0.0201016	0.00616961	10.3248156	0.00131253	0.00556888	Zfp9
ENSMUSG00000072625.2	316.708834	0.01265927	0.00340534	13.8342	0.00019967	0.00106547	Gdf2
ENSMUSG00000072640.12	620.269798	-0.0186901	0.00629661	8.49977773	0.0035519	0.01320332	Lyrm9
ENSMUSG00000072647.7	18.3581666	0.00475872	0.0055949	0.73010057	0.39285059	0.58418599	Adam1a
ENSMUSG00000072662.3	4.25163969	0.02542259	0.00891793	7.61605926	0.00578508	0.0200333	Mansc4
ENSMUSG00000072663.13	4.46178127	0.02614718	0.01001499	6.60177795	0.0101877	0.0323968	Spef2
ENSMUSG00000072664.12	1870.89733	0.00114113	0.00507968	0.04614418	0.82991392	0.93411356	Ugt3a1
ENSMUSG00000072676.13	3652.54842	-0.0074156	0.00382111	3.61536329	0.05724822	0.13479649	Tmem254
ENSMUSG00000072694.9	44.061036	0.0467772	0.00657772	45.2656855	1.72E-11	3.08E-10	1500011B03Rik
ENSMUSG00000072704.7	1793.76365	-0.0064694	0.0018665	11.8662254	0.00057161	0.00268888	Smim10l1
ENSMUSG00000072720.10	35.9990599	0.013383	0.01229189	1.31914539	0.25074548	0.4224345	Myo18b
ENSMUSG00000072762.10	64.0915882	0.00704104	0.00359927	3.80199578	0.05119153	0.12290345	4930522L14Rik
ENSMUSG00000072763.3	66.4907657	0.01177743	0.00484099	5.66122774	0.01734398	0.05059679	Zfp1007
ENSMUSG00000072770.10	54.873584	-0.0139072	0.00599269	5.36032444	0.02059994	0.05841893	Acrbp
ENSMUSG00000072772.4	1303.49484	-0.0090792	0.00513624	3.11275205	0.0776816	0.17215054	Grcc10
ENSMUSG00000072774.11	79.3613681	0.02451418	0.0037953	39.8779709	2.70E-10	4.07E-09	Zfp951
ENSMUSG00000072780.4	1.5252499	0.00538302	0.01979538	0.11853849	0.73062505	0.87054925	Vmn2r24
ENSMUSG00000072825.13	696.889879	0.00299383	0.00423079	0.48665239	0.48542441	0.67423709	Cep170b
ENSMUSG00000072845.4	1.41504454	0.02436197	0.02590566	1.25447627	0.26269925	0.4373694	Tmprss11a
ENSMUSG00000072849.11	90230.9562	-0.0900973	0.0094603	78.5535939	7.79E-19	3.50E-17	Serpina1e
ENSMUSG00000072872.4	316.177152	0.00042979	0.00279727	0.02298441	0.87949763	0.96361922	Rybp
ENSMUSG00000072875.3	9.81366429	-0.0017438	0.01126825	0.02427839	0.87617866	0.96183824	Gpr27
ENSMUSG00000072878.5	2.58599672	0.00482104	0.0663447	-0.1113264	1	1	Nup50l
ENSMUSG00000072889.10	252.257901	0.00610681	0.00370974	2.66995797	0.10225872	0.21373306	Nfxl1
ENSMUSG00000072915.4	50.5610797	0.00717106	0.00484066	2.15960829	0.1416808	0.27530758	Gm12258
ENSMUSG00000072919.5	5.13634232	-0.0268372	0.00915533	8.95872207	0.00276148	0.01064601	Noxred1
ENSMUSG00000072941.6	696.079119	0.02211177	0.00378637	33.8529529	5.94E-09	7.43E-08	Sod3
ENSMUSG00000072944.12	20.4145546	-0.0057654	0.00885507	0.4019736	0.52607176	0.71121911	Nup62cl
ENSMUSG00000072945.4	5.70674627	0.00963933	0.00713798	1.79139485	0.18075631	0.3315951	Ripply1
ENSMUSG00000072946.13	1464.22311	-0.0089556	0.00300991	8.78708029	0.00303371	0.01153225	Ptgr2
ENSMUSG00000072949.7	741.817628	-0.0535174	0.00685625	53.6772091	2.36E-13	5.40E-12	Acot1

ENSMUSG00000072955.15	12.9663647	0.0290023	0.00884668	10.8714	0.00097661	0.00430225	Tmsb15l
ENSMUSG00000072964.15	48.3685554	-0.0072508	0.00518799	2.02176633	0.15505868	0.29455654	Bhlhb9
ENSMUSG00000072966.12	12.9295219	0.00903943	0.00986022	0.74096023	0.38935345	0.58085329	Gprasp2
ENSMUSG00000072969.3	57.0966175	0.00469093	0.00311968	2.20072658	0.1379457	0.26978336	Armcx5
ENSMUSG00000072972.4	11.8705323	-0.0076497	0.00619345	1.48990811	0.22223072	0.38748678	Adam4
ENSMUSG00000072974.5	13.3201328	-0.014861	0.00586391	6.45967143	0.011035	0.03467976	Gm4787
ENSMUSG00000072980.4	8.98487513	-0.0034248	0.00963134	0.11664636	0.73270064	0.87175964	Oip5
ENSMUSG00000073001.5	3.26671646	0.07247748	0.07366097	-0.2628289	1	1	Cylc1
ENSMUSG00000073002.10	213.095673	-0.0100819	0.00438945	5.32833665	0.0209814	0.05923008	Vamp5
ENSMUSG00000073008.12	12.4248997	0.08466396	0.00842893	85.0046421	2.98E-20	1.60E-18	Gpr174
ENSMUSG00000073016.4	26.6480208	0.00496672	0.00587539	0.69520504	0.40439958	0.59598653	Uprt
ENSMUSG00000073062.4	83.0935762	-0.0002519	0.00351646	0.00484299	0.94451871	0.9994266	Zxdb
ENSMUSG00000073077.8	4.13054569	0.08240244	0.01413292	27.6426141	1.46E-07	1.48E-06	Cfap47
ENSMUSG00000073079.7	1028.87817	0.00445355	0.00365147	1.44604615	0.22916344	0.39617745	Srp54a
ENSMUSG00000073096.12	99.3230269	0.00299395	0.00401219	0.53484021	0.46457901	0.65563098	Lrrc61
ENSMUSG00000073102.8	21.2977692	-0.0005613	0.00742372	0.00592741	0.93863178	0.99608629	Drc1
ENSMUSG00000073111.4	1.32513893	0.00521723	0.02516542	0.11555949	0.7339014	0.8725582	Or2a12
ENSMUSG00000073125.11	17.7305139	0.00869081	0.01156729	0.45296563	0.50092998	0.68782008	Xlr3b
ENSMUSG00000073131.13	888.927283	-0.0035282	0.0028137	1.53971527	0.21466019	0.37766487	Vma21
ENSMUSG00000073139.10	325.161938	0.01193869	0.00251787	22.4089439	2.20E-06	1.79E-05	Tmem185a
ENSMUSG00000073155.13	980.304331	0.00633909	0.00357707	3.16824599	0.07508304	0.16754102	Stmp1
ENSMUSG00000073176.5	17.1891059	0.00597414	0.00641342	0.84712561	0.35736676	0.54619314	Zfp449
ENSMUSG00000073197.10	9.97684938	0.02740591	0.00746083	12.3708766	0.00043608	0.00211678	5730507C01Rik
ENSMUSG00000073207.2	6.57378873	0.00699861	0.00665067	1.10958477	0.29217223	0.47407637	Ccdc160
ENSMUSG00000073209.5	1.62133547	0.00463783	0.02741145	0.11360908	0.73607207	0.87394895	Klf14
ENSMUSG00000073293.5	3.29533897	0.02622575	0.01062962	5.34540607	0.02077694	0.05879364	Nudt10
ENSMUSG00000073295.5	8.78094797	0.02982437	0.0071969	14.8464315	0.00011663	0.00065667	Nudt11
ENSMUSG00000073399.10	0.87095623	0.0727089	0.02744118	6.1049708	0.01348022	0.04096166	Trim40
ENSMUSG00000073400.7	3.58157655	0.04886966	0.01659019	5.61810849	0.01777582	0.05163308	Trim10
ENSMUSG00000073402.12	2.1811892	-0.0011295	0.01124378	-0.006075	1	1	Gm8909
ENSMUSG00000073406.11	9.04933022	-0.0319582	0.00853248	13.4511572	0.00024486	0.00127599	H2-Bl
ENSMUSG00000073409.13	667.696919	0.03814829	0.00337073	121.924833	2.40E-28	3.72E-26	H2-Q6
ENSMUSG00000073411.13	11115.5855	0.02717815	0.00267928	101.147104	8.54E-24	6.92E-22	H2-D1
ENSMUSG00000073412.7	37.1364215	0.05297569	0.00651737	61.9875072	3.46E-15	1.00E-13	Lst1
ENSMUSG00000073413.10	2.01534278	0.02175496	0.01134934	3.38487559	0.06579714	0.1506837	Ly6g6d
ENSMUSG00000073414.9	4.72083831	0.07317166	0.01423443	23.7942486	1.07E-06	9.17E-06	Mpig6b
ENSMUSG00000073418.5	25291.9419	0.00281827	0.00508989	0.30942336	0.57803425	0.75627664	C4b
ENSMUSG00000073421.7	1064.74204	0.07278474	0.00591412	136.826375	1.32E-31	3.41E-29	H2-Ab1
ENSMUSG00000073422.12	996.063806	-0.0070668	0.00320532	4.89029546	0.02700806	0.07295982	Hsd17b8
ENSMUSG00000073423.11	79.0970997	0.01156553	0.0030749	14.079314	0.00017526	0.00095071	Zfp414
ENSMUSG00000073424.10	1970.59367	-0.0056399	0.00440092	1.61426635	0.20389312	0.36365874	Cyp4f15
ENSMUSG00000073427.5	33.4714042	0.01175925	0.00537911	4.48121541	0.03426934	0.08877284	Gm4924
ENSMUSG00000073434.13	85.6151056	-0.0002875	0.00479219	0.00360886	0.95209685	1	Wdr90
ENSMUSG00000073435.11	331.541128	-0.0089065	0.00355707	6.35996716	0.01167237	0.03624813	Nme3
ENSMUSG00000073436.12	215.153371	-0.0099952	0.00319791	9.91535236	0.00163906	0.00677156	Eme2
ENSMUSG00000073448.5	100.117432	0.00795181	0.00381007	4.28419596	0.03846827	0.09736159	Gm10509
ENSMUSG00000073460.6	36.9116559	0.02032449	0.00928712	3.99428607	0.04565479	0.11206305	Pnldc1
ENSMUSG00000073468.13	518.299913	0.00212835	0.00331355	0.41757617	0.51814898	0.7042216	Sft2d1
ENSMUSG00000073471.4	225.091872	-0.0052258	0.00393126	1.79674547	0.18010645	0.33072642	Rsph3a
ENSMUSG00000073481.10	6270.96017	-0.0093236	0.00382338	6.04825731	0.01392003	0.04215147	Mtarc2
ENSMUSG00000073489.7	247.310153	0.03850094	0.00469538	64.1940749	1.13E-15	3.46E-14	Ifi204
ENSMUSG00000073490.12	162.811369	0.034195	0.00482388	47.8135244	4.69E-12	9.04E-11	Ifi207
ENSMUSG00000073491.12	61.8303969	0.03532166	0.00619444	31.8993678	1.62E-08	1.90E-07	Ifi213
ENSMUSG00000073494.6	1.45198677	0.03380418	0.02700121	2.66129968	0.10281669	0.21460482	Sh2d1b2
ENSMUSG00000073514.5	8.57553523	0.03244289	0.01780596	3.8213965	0.05060205	0.12174843	Dok6
ENSMUSG00000073530.12	3.56842051	-0.0076301	0.02223934	-0.1361581	1	1	Pappa2
ENSMUSG00000073542.4	106.380965	-0.0037118	0.00307954	1.42210542	0.23305717	0.40105694	Cep76
ENSMUSG00000073555.2	1899.08163	-0.0107678	0.00471713	5.07243255	0.02430925	0.06686818	Iigp1c
ENSMUSG00000073557.13	182.475573	0.00130093	0.00483687	0.07186473	0.78864083	0.90940074	Ppp1r12b
ENSMUSG00000073563.4	640.56683	0.0017255	0.00251331	0.47069091	0.49266949	0.68029404	Csnk1g3
ENSMUSG00000073565.6	718.419954	-0.0041689	0.00420546	0.97779941	0.3227427	0.50921195	Prr16

ENSMUSG00000073591.5	12.4783515	0.02319395	0.00764962	8.76950798	0.00306308	0.01162867	Pcdhb22
ENSMUSG00000073599.11	52.5625747	0.00986144	0.00448914	4.70191496	0.03012903	0.0797557	Ecscr
ENSMUSG00000073600.6	238.315855	0.00038516	0.00465453	0.00677084	0.93442001	0.99423346	Prob1
ENSMUSG00000073608.5	3.3835804	0.04440601	0.01544606	8.4818175	0.00358714	0.01330704	Gal3st2c
ENSMUSG00000073609.10	604.618757	-0.0106401	0.00279197	14.5727908	0.00013485	0.00074695	D2hgdh
ENSMUSG00000073616.11	1462.97516	-0.0114951	0.0053595	4.63131346	0.03139352	0.08254711	Cops9
ENSMUSG00000073633.10	74.5576545	-0.0025462	0.00320286	0.65083864	0.419813	0.61125437	Fbxo36
ENSMUSG00000073639.7	1933.1599	-0.0038694	0.00325771	1.38911127	0.23855524	0.40792894	Rab18
ENSMUSG00000073643.12	267.242651	-0.0057072	0.00329584	2.9381403	0.08651028	0.18760863	Wdfy1
ENSMUSG00000073650.10	1.83960933	0.03830645	0.01107535	11.5486864	0.00067797	0.00312806	Catip
ENSMUSG00000073664.12	835.484341	-0.0027149	0.00387445	0.48420451	0.48652399	0.67509437	Nbeal1
ENSMUSG00000073676.5	4229.26293	-0.0165448	0.00369944	19.4130911	1.05E-05	7.44E-05	Hspe1
ENSMUSG00000073678.5	407.836817	0.00515637	0.00417233	1.48455218	0.22306365	0.3885374	Pgap1
ENSMUSG00000073680.3	61.2861847	-0.0074986	0.00523364	2.04050173	0.15315912	0.29193559	Tmem88b
ENSMUSG00000073684.14	163.556548	0.00671925	0.00313301	4.65852156	0.03089979	0.08152152	Faap20
ENSMUSG00000073700.4	665.410271	-0.0169686	0.00347306	23.6838776	1.14E-06	9.68E-06	Klhl21
ENSMUSG00000073702.12	3633.50149	-0.0015145	0.00332502	0.20808585	0.64827185	0.81215377	Rpl31
ENSMUSG00000073705.11	6.85810891	0.0275895	0.01153189	-17.319929	1	1	Cenps
ENSMUSG00000073725.9	1333.20213	0.00281618	0.00226374	1.526325	0.21666484	0.38042558	Lmbrd1
ENSMUSG00000073730.3	1.25069185	-0.0218422	0.05702854	0.11728678	0.73199603	0.8714344	Ppp1r14bl
ENSMUSG00000073733.5	3.56071693	0.00734653	0.00852435	0.76645529	0.38131587	0.5727752	Cplane2
ENSMUSG00000073755.5	130.138551	0.00484893	0.00241533	4.02001982	0.04496319	0.11059565	5730409E04Rik
ENSMUSG00000073758.11	23.2798811	0.00212366	0.00479209	0.19443005	0.65925482	0.82049413	Sh3d21
ENSMUSG00000073771.12	28.9824543	-0.0133583	0.00662751	3.91751452	0.04778548	0.11625253	Btbd19
ENSMUSG00000073775.5	267.563269	-0.0155921	0.00263912	35.0507827	3.21E-09	4.18E-08	Kti12
ENSMUSG00000073791.14	9.20768582	0.01283803	0.0066164	3.45976887	0.0628791	0.14532744	Efcab7
ENSMUSG00000073792.12	179.689558	0.0027148	0.00288229	0.87640188	0.34918907	0.5373156	Alg6
ENSMUSG00000073802.6	8.85083742	0.04290522	0.00847197	24.7829384	6.42E-07	5.72E-06	Cdkn2b
ENSMUSG00000073805.6	10.0812971	0.00643608	0.01270983	0.36551484	0.54545928	0.72856369	Insyn2a
ENSMUSG00000073830.12	43636.008	-0.1092428	0.0105367	84.7032103	3.47E-20	1.84E-18	Mup14
ENSMUSG00000073834.11	51872.2493	-0.1165581	0.01017398	103.454593	2.66E-24	2.29E-22	Mup11
ENSMUSG00000073838.11	1418.4526	-0.0162689	0.00259205	39.6329847	3.06E-10	4.56E-09	Tufm
ENSMUSG00000073842.11	115535.571	-0.1212186	0.01246113	68.3753046	1.35E-16	4.59E-15	Mup7
ENSMUSG00000073856.12	6.00835067	0.02408856	0.00730292	10.4263937	0.00124227	0.00532054	Iqck
ENSMUSG00000073876.4	11.4113926	0.01088623	0.01255544	0.68179774	0.40896837	0.60065558	Gm13305
ENSMUSG00000073877.11	1.64305663	0.01056132	0.01781122	0.54042852	0.46225519	0.65348543	Gm13306
ENSMUSG00000073878.4	4.56725933	-0.0024945	0.03200578	-0.0050903	1	1	Gm13304
ENSMUSG00000073888.14	190.324274	-0.003364	0.00435757	0.59612486	0.4400604	0.6320238	Ccl27a
ENSMUSG00000073889.11	967.501951	-0.0037188	0.00454901	0.67636805	0.41084019	0.60246684	Il11ra1
ENSMUSG00000073894.2	10.3157193	0.00641884	0.02326302	0.21756448	0.64090196	0.80715045	Rbmxl2
ENSMUSG00000073897.6	1.34902497	-0.0010374	0.04755841	-0.0007147	1	1	Or10a4
ENSMUSG00000073898.4	1.91862109	-0.0094455	0.03691872	0.06813088	0.7940779	0.91250287	Or10a5
ENSMUSG00000073900.6	1.63879827	0.00901632	0.02988414	0.2086916	0.64779485	0.81190258	Or2ag20
ENSMUSG00000073901.5	1.99003474	0.14883356	0.01513466	92.5101117	6.70E-22	4.39E-20	Or2ag19
ENSMUSG00000073907.4	0.97620233	-0.007579	0.04358117	0.05626183	0.81250493	0.92302	Or56b35
ENSMUSG00000073910.11	724.32023	-0.0039224	0.0037942	1.05129016	0.30521014	0.49007327	Mob3b
ENSMUSG00000073922.3	1.42967479	-0.0024761	0.03449038	-0.04449	1	1	Or52n20
ENSMUSG00000073927.7	2.06854855	-0.0085326	0.02662075	0.07524309	0.78385045	0.90595857	Or52h7
ENSMUSG00000073940.4	1265.55253	0.0102182	0.00827038	1.30916953	0.25254507	0.4246776	Hbb-bt
ENSMUSG00000073945.3	1.23500139	0.00826225	0.04129404	0.20623203	0.64973687	0.81314428	Or52z13
ENSMUSG00000073946.6	0.87051293	-0.0035416	0.05869356	-0.0073846	1	1	Or52z12
ENSMUSG00000073947.5	0.85831659	-0.001436	0.04836773	-0.0083074	1	1	Or51ah3
ENSMUSG00000073956.4	1.3720125	0.00719284	0.029991	0.27257029	0.60161279	0.7752629	Or52j3
ENSMUSG00000073967.4	0.83434853	-0.0108698	0.04475495	0.01455143	0.90398466	0.97773111	Or51d1
ENSMUSG00000073968.5	41.4304445	-0.0015192	0.0048332	0.09685699	0.75563447	0.88699521	Trim68
ENSMUSG00000073969.4	0.94026683	-0.0070079	0.03342135	-0.0107183	1	1	Or52i2
ENSMUSG00000073972.3	4.329505	-0.0034403	0.03399722	-0.0164585	1	1	Or52m2
ENSMUSG00000073974.5	0.9436877	-0.0057579	0.03615167	-0.0068561	1	1	Or52p2
ENSMUSG00000073978.10	0.9520248	-0.0043801	0.04374054	-0.0290374	1	1	Or52b4i
ENSMUSG00000073982.12	268.760234	0.02042271	0.00334473	36.8741824	1.26E-09	1.72E-08	Rhog
ENSMUSG00000073987.5	359.302068	0.01007157	0.00332963	9.06697331	0.00260268	0.01012147	Ggh

ENSMUSG00000073988.14	8165.2395	0.00551088	0.00433068	1.57063435	0.21011492	0.37191523	Ttpa
ENSMUSG00000074001.4	3.8007591	-0.0098403	0.0372937	0.08452706	0.77125371	0.8974886	Klhl40
ENSMUSG00000074006.4	3.99295101	0.04977783	0.00920986	28.5142571	9.30E-08	9.73E-07	Omp
ENSMUSG00000074030.4	191.696425	0.00081332	0.00252481	0.10193253	0.74952274	0.88311736	Exoc8
ENSMUSG00000074063.11	3001.90264	-0.0191836	0.00614514	9.30988577	0.00227921	0.00904613	Osgin1
ENSMUSG00000074064.7	1671.63088	-0.0131709	0.00286117	21.0436486	4.49E-06	3.43E-05	Mlycd
ENSMUSG00000074071.13	386.273389	0.00338727	0.00325864	1.08942034	0.29659968	0.47985314	Fam169b
ENSMUSG00000074088.7	152.130734	0.00253363	0.00251017	1.01563046	0.31355772	0.49897239	Snrnp40
ENSMUSG00000074093.5	682.237318	-0.0060105	0.00349117	2.92505579	0.08721421	0.18880536	Svip
ENSMUSG00000074102.5	313.278223	-0.0033722	0.00355561	0.90269964	0.34205887	0.52997441	Rbm15b
ENSMUSG00000074109.5	1.06048536	0.01204251	0.02855751	0.19914555	0.65541142	0.81713361	Mrgprx2
ENSMUSG00000074115.6	7031.26009	-0.0197478	0.0131601	0.47968334	0.48856578	0.67684656	Saa1
ENSMUSG00000074121.4	1.39757123	0.05169066	0.01403011	13.7395076	0.00020999	0.00111375	Ntf5
ENSMUSG00000074127.7	0.98194211	-0.0339988	0.1144818	0.14824561	0.70021759	0.85042905	Cmtm2a
ENSMUSG00000074129.15	18412.0189	-0.0016267	0.00445244	0.13304378	0.71529679	0.85984354	Rpl13a
ENSMUSG00000074141.14	18.3404439	0.03567904	0.00632503	30.1777152	3.94E-08	4.34E-07	Il4i1
ENSMUSG00000074151.15	416.233772	0.02927932	0.00471001	37.6224272	8.59E-10	1.20E-08	Nlrc5
ENSMUSG00000074158.10	93.5148496	-0.0164401	0.00372987	18.9632709	1.33E-05	9.24E-05	Zfp976
ENSMUSG00000074165.11	80.3335928	0.02623406	0.00358422	51.3466786	7.74E-13	1.65E-11	Zfp788
ENSMUSG00000074166.8	125.448456	-0.0044304	0.00261945	2.80852495	0.09376455	0.19992142	AW146154
ENSMUSG00000074170.6	186.70735	-0.002465	0.00342898	0.51731249	0.47199035	0.66271964	Plekhf1
ENSMUSG00000074179.12	7.70007477	0.04856284	0.01559726	9.00593742	0.00269104	0.01042043	Gsta5
ENSMUSG00000074182.9	130.490643	0.00153416	0.00298515	0.26421401	0.60723974	0.78008478	Znhit6
ENSMUSG00000074183.4	252.679827	0.06935175	0.01203442	29.8390954	4.69E-08	5.11E-07	Gsta1
ENSMUSG00000074194.5	22.7548407	-0.0030343	0.00428501	0.51647923	0.47234742	0.6630631	Zfp791
ENSMUSG00000074195.4	0.4753554	-0.0269007	0.04698908	0.46302347	0.49621433	0.68339023	Clca4b
ENSMUSG00000074199.8	4.24442527	-0.0019799	0.07997957	-0.0031493	1	1	Krtdap
ENSMUSG00000074206.8	3.89467915	0.00247315	0.00889133	0.07324289	0.78667236	0.90803231	Adh6b
ENSMUSG00000074207.11	34240.3075	0.00193089	0.00454132	0.18235873	0.66935398	0.82771622	Adh1
ENSMUSG00000074211.5	244.249867	-0.0049228	0.00335892	2.21273845	0.13687551	0.26815731	Sdhaf1
ENSMUSG00000074212.8	614.469107	0.00081254	0.00333595	0.05796216	0.80974631	0.92161736	Dnajb14
ENSMUSG00000074215.3	7.82661728	0.00730223	0.00623935	1.36542771	0.24259902	0.412898	Gm10643
ENSMUSG00000074217.9	3.94937251	0.01019714	0.01155832	0.84845089	0.35699094	0.54579269	Misp3
ENSMUSG00000074218.4	77.2906275	-0.0167303	0.00830918	2.67108594	0.10218628	0.21366108	Cox7a1
ENSMUSG00000074220.11	6.11644923	0.05089215	0.01107327	19.5772038	9.66E-06	6.87E-05	Zfp382
ENSMUSG00000074221.13	262.948335	-0.01403	0.00395099	12.4581983	0.00041616	0.00203305	Zfp568
ENSMUSG00000074227.13	1225.94208	0.00094842	0.0035792	0.06952886	0.79202399	0.91118387	Spint2
ENSMUSG00000074238.7	527.04806	0.01105051	0.00402804	7.33093813	0.00677777	0.02298827	Ap1ar
ENSMUSG00000074240.6	281.700606	0.01334863	0.00945652	1.98803185	0.15854682	0.29947027	Cib3
ENSMUSG00000074247.11	294.196044	-9.16E-05	0.00270377	0.00116951	0.97271914	1	Dda1
ENSMUSG00000074254.5	4125.76997	0.08544065	0.01574098	19.4483402	1.03E-05	7.32E-05	Cyp2a4
ENSMUSG00000074259.11	2.62133329	0.06036738	0.01240889	19.1727413	1.19E-05	8.33E-05	Gramd2a
ENSMUSG00000074261.4	8.83919883	0.01938539	0.01883406	1.4626631	0.22650687	0.39276511	Erich4
ENSMUSG00000074264.13	4045.17975	0.0165606	0.0033222	23.671728	1.14E-06	9.74E-06	Amy1
ENSMUSG00000074268.5	13.673446	-0.1261548	0.0302277	10.2329342	0.00137955	0.00581556	Amy2a5
ENSMUSG00000074269.11	21.6027251	-0.0065564	0.00921845	0.48133339	0.48781897	0.67614645	Rec114
ENSMUSG00000074272.11	3112.78603	-0.0113539	0.00406435	7.79212997	0.00524743	0.01843126	Ceacam1
ENSMUSG00000074277.5	100.783392	-0.010977	0.00456572	5.73678338	0.01661319	0.04882725	Phldb3
ENSMUSG00000074282.11	37.0037387	0.00143529	0.00398173	0.1302259	0.71819793	0.86190861	Zfp94
ENSMUSG00000074283.6	43.710262	-0.0047889	0.00435841	1.21155009	0.27102535	0.44796103	Zfp109
ENSMUSG00000074291.4	2.09288613	-0.0036998	0.02771309	-0.0064397	1	1	Vmn1r168
ENSMUSG00000074305.10	364.658104	0.00551696	0.00356139	2.37980054	0.12291324	0.24678758	Peak1
ENSMUSG00000074336.6	16691.5546	-0.0128995	0.00587066	4.89996918	0.02685717	0.07266873	Apoc4
ENSMUSG00000074340.10	35.5207124	-0.0029528	0.00787242	0.14124242	0.70704899	0.85467699	Ovgp1
ENSMUSG00000074342.5	21.1958195	0.0439514	0.00687196	37.8014764	7.83E-10	1.10E-08	I830077J02Rik
ENSMUSG00000074345.5	28.2712354	0.0524538	0.00659676	59.3470311	1.32E-14	3.58E-13	Tnfaip8l3
ENSMUSG00000074358.10	70.445871	0.00685337	0.00419013	2.66791631	0.10239	0.21392231	Ccdc61
ENSMUSG00000074361.12	47.2877127	0.01330853	0.00383413	11.5845606	0.00066502	0.0030749	C5ar2
ENSMUSG00000074364.7	108.551344	0.00904823	0.00556679	2.59157619	0.10743338	0.22200137	Ehd2
ENSMUSG00000074375.4	67.978677	0.10021935	0.0265197	20.1388418	7.20E-06	5.27E-05	Sult2a3
ENSMUSG00000074377.13	1.74779237	0.08802023	0.04365956	4.73562512	0.02954411	0.07848923	Sult2a4

ENSMUSG00000074384.5	12.7750088	0.01618528	0.00595604	7.14506242	0.00751707	0.02505972	AI429214
ENSMUSG00000074403.3	10.1558938	-0.0023664	0.0236309	0.03078414	0.86072284	0.95372587	H3c13
ENSMUSG00000074405.9	362.166088	0.00317929	0.00673735	0.21254495	0.64477997	0.81004476	Zfp865
ENSMUSG00000074406.6	82.7403872	-0.0072977	0.00299338	5.87127011	0.01538994	0.04584909	Zfp628
ENSMUSG00000074417.10	6.17600949	0.09084888	0.01266487	46.3422862	9.93E-12	1.84E-10	Pira12
ENSMUSG00000074419.13	3.38980133	0.05418085	0.01037775	24.9054128	6.02E-07	5.41E-06	Pira13
ENSMUSG00000074435.11	12.7982656	-0.0629495	0.02512939	0.05818198	0.80939282	0.92139288	Smcp
ENSMUSG00000074445.5	1.2307561	0.00599676	0.02090666	-0.0009264	1	1	Sprr2a3
ENSMUSG00000074449.4	3.49869745	0.02193496	0.02698847	1.10979989	0.29212545	0.47403309	Potefam3a
ENSMUSG00000074457.11	479.999462	0.00023832	0.00467081	0.00261854	0.95918875	1	S100a16
ENSMUSG00000074472.11	29.6707878	0.00407329	0.00614576	0.38421067	0.53535833	0.71973105	Zfp872
ENSMUSG00000074476.7	272.298841	-0.0007987	0.0047623	0.02614045	0.8715578	0.95955572	Spc24
ENSMUSG00000074480.5	6.53234596	0.03470449	0.01113723	9.53584973	0.00201497	0.0081295	Mex3a
ENSMUSG00000074489.10	9.10972342	0.00140577	0.00892116	0.02426098	0.87622269	0.96183824	Bglap3
ENSMUSG00000074491.11	985.104012	-0.0042553	0.00390758	1.18232268	0.27688365	0.45560052	Clec4g
ENSMUSG00000074500.12	8.9869538	0.00547739	0.00640357	0.68498527	0.40787533	0.5994982	Zfp558
ENSMUSG00000074502.5	1.18495627	0.01399136	0.01838673	0.96454729	0.32604384	0.51280809	Ubtfl1
ENSMUSG00000074505.7	12.5260985	0.01976511	0.0150814	1.79719745	0.18005168	0.33066253	Fat3
ENSMUSG00000074513.10	412.578186	0.00283446	0.00181548	2.41355934	0.12028859	0.24269689	Arfip1
ENSMUSG00000074519.12	144.033116	-0.0041715	0.00423711	0.95162174	0.32930684	0.51620232	Zfp971
ENSMUSG00000074521.11	2638.62077	0.00317511	0.0053302	0.33531646	0.56254521	0.74355204	Gm14327
ENSMUSG00000074527.12	328.048572	-0.0022991	0.00725855	0.09827084	0.75391465	0.88616719	Gm14296
ENSMUSG00000074529.12	9.49627008	0.00809422	0.00569258	2.04061264	0.15314796	0.29193559	Zfp972
ENSMUSG00000074569.5	2.74760225	-0.0002746	0.01106363	-0.0084303	1	1	Gcnt7
ENSMUSG00000074570.15	3.6278797	0.05611543	0.00995833	30.7277846	2.97E-08	3.34E-07	Cass4
ENSMUSG00000074576.5	102.940174	-0.0048084	0.00250244	3.63335621	0.0566325	0.13373668	Mocs3
ENSMUSG00000074577.10	28.2114556	0.01299369	0.00507338	6.40084465	0.01140661	0.03561744	Ripor3
ENSMUSG00000074579.15	15.1375227	0.00797834	0.00758151	1.03808638	0.30826695	0.49323548	Lekr1
ENSMUSG00000074582.11	1057.17809	0.00110835	0.00299752	0.13669951	0.71158444	0.85765398	Arfgef2
ENSMUSG00000074591.9	7.46854375	0.0159042	0.00703318	5.06099457	0.02447019	0.06724385	Ankub1
ENSMUSG00000074604.10	8.43064356	0.06165926	0.01345313	17.7951672	2.46E-05	0.00016109	Mgst2
ENSMUSG00000074607.12	15.3597154	0.03922945	0.00933846	14.9682998	0.00010933	0.00061884	Tox2
ENSMUSG00000074619.6	5.48460078	-0.0372906	0.01512983	6.14501762	0.01317829	0.04018406	Fundc2b
ENSMUSG00000074622.5	889.918992	-0.0026155	0.00491985	0.29112973	0.58949748	0.76605441	Mafb
ENSMUSG00000074623.5	23.6010853	-0.0231055	0.00581434	15.8956198	6.69E-05	0.00039886	Gm826
ENSMUSG00000074627.12	2.32213772	-0.0294808	0.01146395	6.45555044	0.01106063	0.0347464	Mroh8
ENSMUSG00000074634.13	197.134653	-0.0018166	0.0039565	0.2150174	0.64286292	0.80864489	Tmem267
ENSMUSG00000074637.8	7.9379239	0.0157183	0.03834761	0.43629483	0.50891622	0.69565567	Sox2
ENSMUSG00000074639.7	2320.02543	0.01016012	0.00544618	3.5286529	0.06031678	0.14054845	Rdh16f2
ENSMUSG00000074643.13	442.305534	0.00371254	0.00282558	1.72110192	0.18955127	0.34374507	Cpne1
ENSMUSG00000074646.12	29.7675531	-0.0100788	0.00547614	3.37452258	0.06621176	0.15148004	6430550D23Rik
ENSMUSG00000074647.5	2.2772127	0.00702704	0.0140903	0.32866027	0.56644904	0.74642373	Fam83c
ENSMUSG00000074649.7	229.641553	0.00925725	0.00275606	11.3487922	0.00075497	0.00342965	Mmp24os1
ENSMUSG00000074652.4	32.5044326	0.00431616	0.01795032	0.03796177	0.84551992	0.94426255	Myh7b
ENSMUSG00000074653.12	2.21205103	0.01614525	0.01363776	1.44229081	0.229769	0.39702063	Lrrc31
ENSMUSG00000074656.14	2401.54562	-0.0050746	0.00295007	2.9171872	0.08764051	0.1895195	Eif2s2
ENSMUSG00000074657.6	87.9339489	0.00602982	0.00891423	0.44378401	0.50530171	0.69214507	Kif5a
ENSMUSG00000074671.7	30.4238246	0.04719793	0.00567968	68.4982423	1.27E-16	4.32E-15	Tspyl3
ENSMUSG00000074676.3	8.53870606	0.02229264	0.00759425	8.11236743	0.00439643	0.01586265	Foxs1
ENSMUSG00000074677.12	24.8567778	0.10728441	0.00909269	119.120618	9.86E-28	1.38E-25	Sirpb1c
ENSMUSG00000074682.5	14.4953765	-0.0103234	0.00529393	3.95823776	0.04664251	0.1140174	Zcchc3
ENSMUSG00000074698.11	1926.743	-0.0028812	0.0019911	2.06469086	0.1507455	0.28850898	Csnk2a1
ENSMUSG00000074715.4	6.48001135	0.00983871	0.00801822	1.48903075	0.22236691	0.38763827	Ccl28
ENSMUSG00000074733.15	1331.23082	-0.006633	0.00557095	1.42530078	0.23253289	0.40041782	Zfp950
ENSMUSG00000074738.3	11.2428035	0.04050562	0.00750952	27.4856327	1.58E-07	1.59E-06	Fndc10
ENSMUSG00000074743.5	109.456926	-0.0077579	0.00469161	2.72318351	0.09890058	0.20838567	Thbd
ENSMUSG00000074746.4	665.665168	-0.0009114	0.00288144	0.09717719	0.75524378	0.88681641	Pdzd8
ENSMUSG00000074748.4	2110.70682	-0.0012687	0.0028742	0.19362863	0.65991354	0.82105401	Atxn7l3b
ENSMUSG00000074749.11	295.31218	0.00119229	0.00404187	0.0864337	0.76876089	0.89593806	Kiz
ENSMUSG00000074754.5	398.3874	-0.0160321	0.00432078	13.9249543	0.00019026	0.00102079	Smim26
ENSMUSG00000074766.11	27.5192192	0.01319643	0.01036529	1.62256676	0.20273426	0.36197504	Ism1

ENSMUSG00000074768.7	51543.3606	-0.0050101	0.00653339	0.55888195	0.45471107	0.64646265	Bhmt
ENSMUSG00000074771.11	4.33359011	-0.0005645	0.01855068	-0.0039825	1	1	Ankef1
ENSMUSG00000074781.6	1049.68166	0.00351646	0.00264774	1.74258446	0.18681169	0.33995375	Ube2n
ENSMUSG00000074785.6	358.294102	-0.0053611	0.00354084	2.25106601	0.13352239	0.263096	Plxnc1
ENSMUSG00000074793.11	25.7370194	-0.0081267	0.00522671	2.44802992	0.11767247	0.23845888	Hspa12b
ENSMUSG00000074794.11	3068.87982	0.00113161	0.00587412	0.03449098	0.85266633	0.94925088	Arrdc3
ENSMUSG00000074796.11	4.45746223	0.01065284	0.00942598	1.17611635	0.27814805	0.4570602	Slc4a11
ENSMUSG00000074797.12	357.739605	-0.0101101	0.00254486	16.0017315	6.33E-05	0.00037961	Itpa
ENSMUSG00000074802.12	91.767152	0.01962279	0.00579582	10.7573193	0.00103868	0.00454661	Gas2l3
ENSMUSG00000074811.4	72.0068426	-0.0032274	0.00236299	1.85745782	0.17291849	0.32043177	Hps6
ENSMUSG00000074817.3	2.18901692	-0.0255426	0.02712461	1.0132254	0.31413137	0.49950128	Papolb
ENSMUSG00000074818.14	1.83075683	0.00140137	0.01331468	0.01347801	0.90757738	0.97977482	Pdzd7
ENSMUSG00000074824.4	26.7872047	-0.0065474	0.00493662	1.69710849	0.19266656	0.34787058	Rslcan18
ENSMUSG00000074825.5	132.040777	0.01138355	0.00312312	13.2564738	0.00027164	0.00139797	Itpripl1
ENSMUSG00000074826.11	20.8992031	-0.0053754	0.0047458	1.32802254	0.24915732	0.42069042	Ramacl
ENSMUSG00000074829.11	445.851682	-0.0054559	0.00235948	5.33755749	0.0208707	0.05897403	2010315B03Rik
ENSMUSG00000074832.12	18.9168832	0.00559829	0.06240667	0.06725251	0.79537995	0.91328716	Zfp998
ENSMUSG00000074862.12	19.1154151	-0.0001285	0.00493751	0.00066683	0.97939842	1	BC025920
ENSMUSG00000074863.6	21.5167449	0.02453413	0.00583867	16.4143276	5.09E-05	0.00031133	Platr25
ENSMUSG00000074865.13	12.5174944	0.01940809	0.00662772	8.27465464	0.00402023	0.01469184	Zfp934
ENSMUSG00000074867.5	6.68223233	0.01249687	0.00804189	2.27333441	0.13161646	0.26036281	Zfp808
ENSMUSG00000074874.11	147.7799	0.06115667	0.00739169	56.6079866	5.32E-14	1.32E-12	Ctla2b
ENSMUSG00000074882.4	4981.79792	0.00336106	0.00498012	0.46381037	0.49584856	0.68308417	Cyp2c68
ENSMUSG00000074884.13	4233.95997	-0.0107909	0.00370354	8.4933772	0.00356442	0.01324568	Serf2
ENSMUSG00000074886.12	319.829513	0.01049608	0.00251132	17.2624231	3.26E-05	0.00020742	Grk6
ENSMUSG00000074890.5	157.796873	-0.0074092	0.00229485	10.3841242	0.00127103	0.005426	Lcmt2
ENSMUSG00000074892.10	6.5433001	0.04880881	0.0135501	10.9398388	0.00094118	0.00416175	B3galt5
ENSMUSG00000074896.4	337.787422	0.02905697	0.00439555	45.0036852	1.97E-11	3.49E-10	Ifit3
ENSMUSG00000074899.7	4.02267141	0.01767729	0.0115057	2.44631606	0.11780105	0.23867842	Sptbn5
ENSMUSG00000074909.6	333.478615	-0.0071898	0.00235443	9.16652219	0.00246483	0.00965166	Ranbp6
ENSMUSG00000074916.6	37.9947838	0.00938947	0.00414597	5.09448087	0.0240021	0.06619662	Chst14
ENSMUSG00000074918.5	228.988379	-0.0025911	0.00232748	1.23706488	0.26603747	0.44166677	Inafm2
ENSMUSG00000074922.2	85.8812355	-0.0022309	0.00249193	0.78813994	0.37466316	0.56559174	Pabir1
ENSMUSG00000074923.11	14.1123629	0.04049997	0.00849516	22.3603777	2.26E-06	1.83E-05	Pak6
ENSMUSG00000074925.5	323.339277	-8.58E-05	0.00360797	0.00054466	0.98138068	1	Ptar1
ENSMUSG00000074934.4	5.55893819	0.07306867	0.02569188	1.02169063	0.31211832	0.49730614	Grem1
ENSMUSG00000074968.12	9.11154577	0.0309631	0.01941906	3.01128961	0.08268648	0.18085661	Ano3
ENSMUSG00000074971.5	14.6309057	-0.002264	0.00588796	0.14662784	0.70177897	0.85122956	Fibin
ENSMUSG00000074994.7	412.200651	0.00073445	0.00302144	0.05827527	0.80924301	0.92131127	Qser1
ENSMUSG00000074995.3	1.09834818	0.00215398	0.04092963	0.03238191	0.85719202	0.95169135	Or5ac19
ENSMUSG00000075000.12	477.679347	-0.0073484	0.00227923	10.1577276	0.00143698	0.00603714	Nrbf2
ENSMUSG00000075012.5	3.53652194	0.0068881	0.01113411	0.40117606	0.52648251	0.71144818	Fjx1
ENSMUSG00000075028.13	15.8280202	0.01831478	0.00535372	11.5110188	0.00069185	0.00318026	Prdm11
ENSMUSG00000075031.5	10.3995517	0.00177115	0.01856385	0.00875315	0.92546006	0.98916486	H2bc3
ENSMUSG00000075033.5	20.5163202	0.05144716	0.00599607	69.8256844	6.48E-17	2.31E-15	Nxpe3
ENSMUSG00000075040.10	110.069459	0.00388596	0.00414093	0.86889534	0.35126131	0.53969443	Zfp408
ENSMUSG00000075044.15	36.7484311	0.1081518	0.00829548	143.587442	4.37E-33	1.39E-30	Slc22a29
ENSMUSG00000075054.6	605.952744	-0.0157203	0.00341455	20.7767928	5.16E-06	3.89E-05	Yae1d1
ENSMUSG00000075062.5	2.83072233	0.0017924	0.02318863	0.02128129	0.88401527	0.96623496	Or4b12
ENSMUSG00000075065.6	1.12764476	0.00364049	0.04644411	0.03357578	0.85461211	0.95029689	Or4b1
ENSMUSG00000075069.4	0.98997346	-0.0163429	0.03609795	0.17572858	0.67507002	0.83175059	Or4c3
ENSMUSG00000075073.4	1.63131177	0.0007641	0.03389619	-0.0073107	1	1	Or4a47
ENSMUSG00000075075.3	1.23722659	-0.0066827	0.03235502	0.08343561	0.77269446	0.89845362	Or4a80
ENSMUSG00000075084.4	6.11149646	-0.000706	0.04067833	-0.0081312	1	1	Or4a71
ENSMUSG00000075090.5	0.87209755	-0.0057563	0.04888476	-0.0006861	1	1	Or4a15
ENSMUSG00000075091.6	1.73421931	-0.0120724	0.02968671	0.17848973	0.67267437	0.83015618	Or4c125
ENSMUSG00000075093.4	0.937318	0.00574626	0.03890753	0.09043701	0.76362232	0.89268418	Or4c1
ENSMUSG00000075097.4	1.76083324	0.00321059	0.03000452	0.06533668	0.79825168	0.91521751	Or4c121
ENSMUSG00000075104.4	1.55170792	-0.0063512	0.03494677	-0.0139365	1	1	Or4c114
ENSMUSG00000075105.4	0.77941929	0.0051313	0.04633911	0.04601744	0.8301441	0.93432805	Or4c113
ENSMUSG00000075107.4	0.88828367	-0.0058214	0.04565185	0.01421778	0.90508653	0.97833964	Or4c111

ENSMUSG00000075113.3	1.53017912	0.00435763	0.02895207	0.09077339	0.76319624	0.8923196	Or4c29
ENSMUSG00000075115.4	1.73957898	-0.0007819	0.03595692	-0.0059113	1	1	Or4a67
ENSMUSG00000075120.5	1.19415707	-0.0019916	0.04251494	-0.0095037	1	1	Or4a66
ENSMUSG00000075122.6	14.321601	0.05956696	0.00740375	57.5362606	3.32E-14	8.50E-13	Cd80
ENSMUSG00000075125.3	1.65428603	0.010259	0.02884793	0.4521399	0.5013205	0.68801031	Or4p20
ENSMUSG00000075132.3	1.51325741	-0.0010215	0.02071563	-0.0165273	1	1	Or5d43
ENSMUSG00000075133.3	1.24181073	-0.0152147	0.03326659	0.40148444	0.52632362	0.7113476	Or5d41
ENSMUSG00000075141.6	1.29241718	0.00807172	0.03102671	0.23434321	0.62832194	0.79696699	Or9m1b
ENSMUSG00000075143.6	0.98824729	-0.0046632	0.03828986	-0.0151991	1	1	Or5l14
ENSMUSG00000075159.6	0.97157988	-0.0159417	0.04325151	0.2210198	0.63826398	0.80503178	Or5aq1
ENSMUSG00000075160.4	2.00668077	0.00673409	0.02861089	0.20860297	0.64786459	0.81193062	Or5aq7
ENSMUSG00000075165.4	0.68352975	-0.0136365	0.05532612	0.02512728	0.87405032	0.96088988	Or5be3
ENSMUSG00000075170.5	0.73929402	-0.0013054	0.04609962	-0.0018185	1	1	Or8h7
ENSMUSG00000075188.5	0.48112569	-0.009733	0.07915661	0.00986929	0.9208649	0.98698171	Or8k23
ENSMUSG00000075202.6	1.09176906	-0.000567	0.03029355	-0.0013386	1	1	Or5al1
ENSMUSG00000075224.11	3.32456018	0.09735332	0.01760927	27.6268785	1.47E-07	1.49E-06	Lrrc55
ENSMUSG00000075225.17	204.377655	-0.0191415	0.00433906	19.3181478	1.11E-05	7.77E-05	Ccdc162
ENSMUSG00000075227.7	239.219811	-0.0098378	0.00335334	8.70334871	0.00317626	0.01199205	Znhit2
ENSMUSG00000075229.12	802.099813	-0.0161822	0.00325774	24.7971586	6.37E-07	5.69E-06	Mix23
ENSMUSG00000075232.7	1296.39598	-0.0038123	0.0027918	1.89060598	0.16913359	0.31487764	Amd1
ENSMUSG00000075249.13	9.97691582	0.01148202	0.02396123	-0.4057731	1	1	Fsip2
ENSMUSG00000075254.13	407.073302	0.01912213	0.00386059	24.1575735	8.88E-07	7.69E-06	Heg1
ENSMUSG00000075256.10	69.9255752	0.01101806	0.00611933	2.85031673	0.09135594	0.19584813	Cerkl
ENSMUSG00000075266.6	35.4610256	-0.0071395	0.00496999	2.06035072	0.15117537	0.28906545	Cenpw
ENSMUSG00000075270.12	54.7678711	-0.0018624	0.0168242	0.00377093	0.95103437	1	Pde11a
ENSMUSG00000075271.5	42.7856476	-0.0037826	0.0034226	1.17667424	0.2780341	0.45695158	Ift70a1
ENSMUSG00000075272.5	5.61250668	-0.0217001	0.00973869	4.94996073	0.02609126	0.07094633	Ift70a2
ENSMUSG00000075273.5	123.116217	-0.0021722	0.00282499	0.57279926	0.4491487	0.64075614	Ift70b
ENSMUSG00000075284.11	155.837612	0.04454173	0.00452027	91.5441394	1.09E-21	6.88E-20	Wipf1
ENSMUSG00000075289.6	34.7115209	-0.0026203	0.00510326	0.24957382	0.61737533	0.78855272	Carns1
ENSMUSG00000075296.6	0.98480722	0.05224198	0.01734423	10.9336553	0.00094433	0.00417253	Aldh3b2
ENSMUSG00000075302.11	3.38990528	0.01198983	0.01727187	0.68029425	0.40948542	0.60122778	Erich2
ENSMUSG00000075304.3	30.4434025	0.00130253	0.00536297	0.05408735	0.81609775	0.92527431	Sp5
ENSMUSG00000075307.4	29.2145011	-0.0010855	0.01301523	0.30092249	0.58330468	0.76069546	Klhl41
ENSMUSG00000075316.14	5.97191825	0.03783755	0.01720874	6.56895003	0.01037732	0.03289334	Scn9a
ENSMUSG00000075318.14	22.8139459	0.04548336	0.01413879	8.81056359	0.00299492	0.01140796	Scn2a
ENSMUSG00000075324.14	186.889839	0.01760016	0.00486504	12.5168442	0.0004033	0.00197759	Fign
ENSMUSG00000075327.11	116.237739	-0.0052647	0.00251814	4.36230296	0.0367426	0.09386018	Zbtb2
ENSMUSG00000075334.3	3.78669263	0.03256238	0.02654098	2.37255679	0.12348466	0.24778455	Rprm
ENSMUSG00000075376.11	485.515777	0.00614405	0.00279621	4.75869379	0.02915066	0.07758379	Rc3h2
ENSMUSG00000075377.3	0.91464631	-0.0148139	0.04742265	0.24735609	0.61894292	0.78964854	Or1b1
ENSMUSG00000075380.3	0.86405078	0.00659468	0.03849985	0.1050262	0.74587939	0.88032745	Or1l8
ENSMUSG00000075382.4	1.11614893	0.00163884	0.03837568	0.02131606	0.88392123	0.96617695	Or1n1b
ENSMUSG00000075387.3	2.35793189	-0.0047601	0.02548454	0.01361834	0.90709965	0.97942966	Or1j13
ENSMUSG00000075394.5	1.64008388	0.03560848	0.01870234	4.87327145	0.02727575	0.07350614	Hoxc4
ENSMUSG00000075408.7	0.97746038	-0.0002953	0.02986327	-0.0028411	1	1	Smim41
ENSMUSG00000075410.15	2.42725617	0.01308985	0.00923941	1.95585502	0.16195731	0.30432801	Prcd
ENSMUSG00000075415.14	506.967834	0.01710019	0.00370386	20.4800992	6.03E-06	4.49E-05	Fnbp1
ENSMUSG00000075419.5	248.279775	-0.0115688	0.00210722	30.5257151	3.29E-08	3.67E-07	Dolk
ENSMUSG00000075420.7	20.1404007	-0.0108886	0.00614089	3.23883163	0.0719119	0.16181207	Smim6
ENSMUSG00000075467.5	381.2518	-0.0083319	0.00232669	12.8470656	0.00033801	0.00169263	Dnlz
ENSMUSG00000075470.3	781.072416	-0.0013152	0.00265053	0.24133357	0.6232446	0.79279322	Alg10b
ENSMUSG00000075478.4	8.32645116	0.02778018	0.02248844	2.07164649	0.15005944	0.28757609	Slitrk1
ENSMUSG00000075486.11	872.132236	-0.0062867	0.00318	3.8682789	0.04920696	0.1189988	Commd6
ENSMUSG00000075502.3	2.1268303	-0.0056067	0.01073671	0.23895671	0.62496065	0.79389896	Kbtbd6
ENSMUSG00000075520.6	1.08432943	0.03527496	0.0211323	4.09634097	0.04297613	0.10670964	Malrd1
ENSMUSG00000075524.4	2.02542154	0.0245892	0.03668802	-31.701515	1	1	4930407I10Rik
ENSMUSG00000075528.14	331.975864	-0.0087317	0.00256795	11.682018	0.00063107	0.00293463	Aarsd1
ENSMUSG00000075543.5	329.366688	-0.010317	0.00404457	6.44420567	0.01113148	0.03493176	Urad
ENSMUSG00000075551.5	7664.77795	0.10923525	0.01288329	50.8195786	1.01E-12	2.12E-11	Cyp3a41a
ENSMUSG00000075552.5	1105.54316	0.05600884	0.0296992	3.33752007	0.0677166	0.15413662	Cyp3a41b

ENSMUSG00000075569.11	3.23187949	0.01632486	0.01335857	1.47298144	0.22487588	0.39071252	Rsph10b
ENSMUSG00000075588.7	21.4902002	-0.0013456	0.00586067	0.04871436	0.82531567	0.93098166	Hoxb2
ENSMUSG00000075590.5	800.582337	-0.0020071	0.00369337	0.29579057	0.58653347	0.76333822	Nrbp2
ENSMUSG00000075592.12	149.260652	0.00023249	0.0045484	0.00257181	0.95955418	1	Nynrin
ENSMUSG00000075593.11	1.04244192	0.02894382	0.01973384	2.70456289	0.1000615	0.21030571	Gal3st4
ENSMUSG00000075595.10	940.975214	-0.0007623	0.00291967	0.06787999	0.7944489	0.91279578	Zfp652
ENSMUSG00000075598.6	0.84283293	-0.0087512	0.03354403	-0.0198211	1	1	Smok3c
ENSMUSG00000075600.4	93.2519344	-0.00385	0.00360624	1.12600657	0.28862875	0.46981351	Zc3h3
ENSMUSG00000075602.11	304.128327	0.05479113	0.00607948	81.713585	1.57E-19	7.76E-18	Ly6a
ENSMUSG00000075700.11	3736.54589	-0.0021808	0.00221755	0.95831003	0.32761303	0.51458984	Selenot
ENSMUSG00000075701.11	1417.1133	-0.0031182	0.00411991	0.57510096	0.44823902	0.64015535	Selenos
ENSMUSG00000075702.11	43.8549785	0.01595868	0.00611858	6.71140819	0.0095798	0.03077909	Selenom
ENSMUSG00000075703.17	1193.53631	-0.0054462	0.00332066	2.61634125	0.10576806	0.21919388	Selenoi
ENSMUSG00000075704.18	557.605179	-0.0075474	0.00324032	5.42047332	0.01990196	0.05667122	Txnrd2
ENSMUSG00000075705.14	4451.82943	-0.0099611	0.00316497	10.0029215	0.00156292	0.00650027	Msrb1
ENSMUSG00000075706.12	10247.9606	0.00584509	0.00666773	0.7423084	0.38892242	0.58046726	Gpx4
ENSMUSG00000075707.7	2.64569082	0.01137137	0.01194669	1.02169513	0.31211726	0.49730614	Dio3
ENSMUSG00000076431.5	178.399047	0.02712053	0.00587212	20.3519471	6.44E-06	4.76E-05	Sox4
ENSMUSG00000076432.14	1655.87548	-0.0007219	0.00199902	0.12944034	0.71901302	0.86242843	Ywhaq
ENSMUSG00000076433.6	2.46296216	0.00804194	0.0190953	0.20792581	0.64839801	0.81215377	Cep295nl
ENSMUSG00000076434.10	5.25581366	0.01569212	0.01377289	1.01240979	0.31432622	0.49965077	Wfdc3
ENSMUSG00000076435.4	2717.49184	-0.0131199	0.00362979	12.7261852	0.00036057	0.00179229	Acsf2
ENSMUSG00000076437.12	117.602703	0.0009345	0.00442687	0.04575922	0.83061409	0.93462054	Selenoh
ENSMUSG00000076441.10	52906.4064	-0.0021628	0.00514571	0.17633386	0.67454298	0.83155036	Ass1
ENSMUSG00000077450.14	1135.38548	-0.0043236	0.00206313	4.37458344	0.03647875	0.09335804	Rab11b
ENSMUSG00000078137.2	2.3752551	0.01901287	0.01914171	1.43514732	0.23092623	0.39852389	Ankrd63
ENSMUSG00000078139.4	6.07925932	0.00027226	0.00853415	0.00044219	0.98322298	1	AK157302
ENSMUSG00000078153.4	57.5998475	0.01538931	0.00462356	10.9387851	0.00094172	0.00416274	Psme2b
ENSMUSG00000078154.4	32.8892581	0.01479376	0.00682019	4.47427481	0.0344088	0.08909496	Cdk2ap1rt
ENSMUSG00000078157.2	2.09361568	0.02308652	0.01459813	3.10928907	0.07784693	0.17241974	Fem1al
ENSMUSG00000078161.9	18.3675566	0.05343432	0.01333852	13.7079659	0.00021355	0.00113058	Erich3
ENSMUSG00000078173.4	2.02539269	0.00105435	0.03321159	0.01809423	0.8929955	0.97206943	Lenep
ENSMUSG00000078184.2	2.22649423	-0.0020715	0.01002778	0.03590606	0.84970962	0.94739771	Rbm8a2
ENSMUSG00000078185.4	66.7043733	0.00842417	0.0040868	4.1003653	0.04287395	0.10647836	Chml
ENSMUSG00000078193.3	1628.12101	0.00282248	0.00639101	0.1980248	0.65631991	0.8180933	Gm2000
ENSMUSG00000078201.6	117.433619	-0.003822	0.00325265	1.40530201	0.23583785	0.40454339	Tmem203
ENSMUSG00000078202.4	39.3211909	0.01947744	0.00519247	13.635855	0.00022191	0.00116932	Nrarp
ENSMUSG00000078234.7	557.995226	0.00834004	0.00441677	3.42551915	0.06419606	0.14779024	Klhdc7a
ENSMUSG00000078240.3	3.95414315	0.0069514	0.0119584	0.316747	0.57356917	0.75256607	Rpl27rt
ENSMUSG00000078249.6	79.9911866	0.02993354	0.01679214	1.02622704	0.31104648	0.49624607	Hmga1b
ENSMUSG00000078302.6	2.03565125	0.0064687	0.04488678	0.06431443	0.79980236	0.91577933	Foxd1
ENSMUSG00000078307.4	18.4471347	0.01870219	0.01611655	1.6272421	0.20208493	0.36112676	AI593442
ENSMUSG00000078317.7	113.071553	0.00154963	0.00259304	0.35460151	0.55151981	0.73384555	F8a
ENSMUSG00000078348.5	329.521061	-0.0029252	0.0038495	0.58086561	0.44597323	0.63815596	Sf3b5
ENSMUSG00000078350.12	171.438769	-0.0087838	0.00546416	2.64149846	0.10410531	0.21645168	Smim1
ENSMUSG00000078427.4	663.872432	-0.0049115	0.00297953	2.72737832	0.09864109	0.20796895	Sarnp
ENSMUSG00000078429.10	960.516372	0.00159432	0.00284893	0.31346201	0.57556349	0.75420025	Ctdsp2
ENSMUSG00000078435.6	77.8946519	0.00161008	0.00368681	0.18621934	0.66608203	0.82535189	AU041133
ENSMUSG00000078439.10	20.2054807	0.05439039	0.0054489	95.8513681	1.24E-22	8.80E-21	Smim24
ENSMUSG00000078440.11	367.265433	-0.0124005	0.0029373	18.0017424	2.21E-05	0.00014614	Dohh
ENSMUSG00000078451.6	6.81736295	0.00441297	0.00769094	0.33149868	0.56477794	0.74522252	Ppil6
ENSMUSG00000078452.11	491.730327	0.05281294	0.00672641	56.9215898	4.54E-14	1.14E-12	Raet1d
ENSMUSG00000078453.4	251.932197	0.02376532	0.00421864	30.4496643	3.43E-08	3.81E-07	Abracl
ENSMUSG00000078485.5	22.9295758	0.02358373	0.0069266	11.1087567	0.00085921	0.00383616	Plekhn1
ENSMUSG00000078486.4	43.1082239	0.01131425	0.0122512	0.61630776	0.43242266	0.6239521	Perm1
ENSMUSG00000078490.11	6.41849261	0.02286487	0.00829541	7.89857526	0.00494738	0.0175261	Cfap74
ENSMUSG00000078495.11	82.2085605	0.02663889	0.00458722	31.7102679	1.79E-08	2.08E-07	Zfp984
ENSMUSG00000078496.10	205.189672	0.00690971	0.00592175	1.15903628	0.2816655	0.46144075	Zfp982
ENSMUSG00000078498.6	4.23378788	0.03748176	0.01821979	4.4977725	0.03393904	0.08807197	Zfp988
ENSMUSG00000078502.11	22.869928	0.03603924	0.00846756	15.3810038	8.79E-05	0.00050807	Zfp268
ENSMUSG00000078503.10	2.04948586	0.01260941	0.01704716	0.70712304	0.40040055	0.59214046	Zfp990

ENSMUSG00000078515.5	4497.89644	-0.0076751	0.00352074	4.59899657	0.03199067	0.08387217	Ddi2
ENSMUSG00000078517.14	638.468215	-0.003167	0.00232681	1.84953191	0.17383785	0.32180672	Emc1
ENSMUSG00000078521.3	2.5473113	0.03509233	0.01617503	4.71850574	0.02983966	0.07913354	Aunip
ENSMUSG00000078532.10	3.25303721	0.08112751	0.01338677	35.7240052	2.27E-09	3.01E-08	Nkain1
ENSMUSG00000078546.9	57.0706169	0.01330352	0.00435575	9.16678207	0.00246448	0.00965166	Zfp995
ENSMUSG00000078552.11	6.88581374	-0.022485	0.00669151	11.2050232	0.00081576	0.00367397	Dcdc2b
ENSMUSG00000078566.9	5798.90643	0.002022	0.00315009	0.40913642	0.52240815	0.70813776	Bnip3
ENSMUSG00000078570.11	308.550003	-0.0082583	0.00372722	4.86313458	0.02743644	0.07387169	1110065P20Rik
ENSMUSG00000078572.4	278.498804	-0.0125348	0.00418415	9.04898343	0.00262841	0.01020921	Ndufaf8
ENSMUSG00000078578.10	5989.26939	-0.0010117	0.0020517	0.23996337	0.62423257	0.79336661	Ube2d3
ENSMUSG00000078580.4	79.0908239	-0.0002724	0.00290749	0.00877549	0.92536527	0.98916486	E430018J23Rik
ENSMUSG00000078584.10	1457.54161	-0.0161049	0.00391624	16.7933838	4.17E-05	0.00026123	AU022252
ENSMUSG00000078588.11	9.42276241	0.0045699	0.01179973	0.15880396	0.69026008	0.84302373	Ccdc24
ENSMUSG00000078591.2	28.2136584	0.01591456	0.01812014	1.0584468	0.3035697	0.48817286	Hs3st4
ENSMUSG00000078597.3	1239.86784	-0.0804105	0.00846837	76.0389183	2.78E-18	1.16E-16	Cyp4a12b
ENSMUSG00000078606.9	682.542819	0.06536878	0.00990988	38.756322	4.80E-10	6.96E-09	Gvin2
ENSMUSG00000078607.7	46.0310225	0.01904665	0.00378508	25.7066127	3.97E-07	3.70E-06	1810010H24Rik
ENSMUSG00000078611.4	2.85670867	0.00533253	0.02615821	-0.0335764	1	1	Gm5901
ENSMUSG00000078612.10	151.774161	0.01984265	0.00442876	19.3118928	1.11E-05	7.80E-05	Fyb2
ENSMUSG00000078616.3	7.80514961	0.03313322	0.00855554	14.0707679	0.00017606	0.00095482	Trim30c
ENSMUSG00000078619.11	945.50956	0.00054744	0.00240701	0.05142599	0.82060026	0.92814836	Smarcd2
ENSMUSG00000078622.9	2240.29118	-0.0071915	0.00298555	5.75769387	0.01641661	0.04833983	Ccdc47
ENSMUSG00000078624.5	1.18150144	-0.0038366	0.02670415	-0.0122696	1	1	Or51ab3
ENSMUSG00000078627.10	40.5262972	-0.0176977	0.04141469	0.28308294	0.59468763	0.76995677	Marchf10
ENSMUSG00000078630.9	4.8873627	-0.0036684	0.00990085	0.12213568	0.72672962	0.86810052	Tomt
ENSMUSG00000078632.5	1.17598736	-0.0078019	0.03612077	0.04806686	0.82646189	0.93169535	Lrrc37a
ENSMUSG00000078650.3	5785.58068	-0.0384259	0.00643938	34.6522794	3.94E-09	5.06E-08	G6pc
ENSMUSG00000078651.9	15.2685764	-0.0270162	0.00633256	18.4508234	1.74E-05	0.00011806	Aoc2
ENSMUSG00000078652.10	1003.99745	-0.0040949	0.00204398	3.9914979	0.04573039	0.11219025	Psme3
ENSMUSG00000078653.5	11.1056299	-0.008782	0.00967098	0.80122038	0.37072875	0.56130493	Cntd1
ENSMUSG00000078656.12	974.899817	-0.0070093	0.00250081	7.92550141	0.00487428	0.01730319	Vps25
ENSMUSG00000078664.3	1.35216633	0.02611375	0.03091861	1.01650176	0.31335024	0.49876355	Sprr2a1
ENSMUSG00000078670.4	135.348315	0.00436487	0.00516846	0.68217356	0.40883927	0.60054076	Fam174b
ENSMUSG00000078671.12	2835.25624	0.00680113	0.00517593	1.70127885	0.19212082	0.34720417	Chd2
ENSMUSG00000078672.3	285069.426	-0.0404497	0.00756337	25.138544	5.34E-07	4.83E-06	Mup20
ENSMUSG00000078673.11	65840.8672	-0.0905445	0.01092708	50.6486923	1.10E-12	2.30E-11	Mup19
ENSMUSG00000078674.3	9080.13157	-0.0252408	0.01100134	1.62898187	0.20184393	0.3608009	Mup18
ENSMUSG00000078675.10	16877.4593	-0.1022599	0.01003234	87.4870592	8.48E-21	4.78E-19	Mup16
ENSMUSG00000078676.10	386.498937	0.00187023	0.00225196	0.68821718	0.40677145	0.59840326	Casc3
ENSMUSG00000078680.4	19747.1298	-0.0558964	0.00856341	37.7926418	7.87E-10	1.10E-08	Mup10
ENSMUSG00000078681.11	429.882396	-0.0085829	0.00334744	6.60501183	0.01016921	0.0323511	Tm2d3
ENSMUSG00000078683.10	10610.5715	-0.0922638	0.01247917	42.0357001	8.96E-11	1.45E-09	Mup1
ENSMUSG00000078686.12	6500.87925	-0.0829986	0.01195512	37.4904416	9.19E-10	1.28E-08	Mup9
ENSMUSG00000078687.10	244.819307	-0.1159936	0.01312328	62.0664887	3.32E-15	9.65E-14	Mup8
ENSMUSG00000078688.12	3096.13887	-0.0675399	0.01163755	26.2900951	2.94E-07	2.80E-06	Mup2
ENSMUSG00000078689.9	186.253072	-0.121977	0.01027291	119.181091	9.56E-28	1.34E-25	Mup6
ENSMUSG00000078695.9	4007.5712	0.00620013	0.00515451	1.46579403	0.22601047	0.39217816	Cisd3
ENSMUSG00000078713.9	668.981482	-0.0134709	0.00433293	9.73669044	0.00180626	0.00738471	Tomm5
ENSMUSG00000078716.11	49.9999127	-0.0025121	0.00534432	0.20787104	0.6484412	0.81215377	Tmem8b
ENSMUSG00000078719.3	12.0302884	0.0563133	0.00998162	30.6426712	3.10E-08	3.48E-07	Msmp
ENSMUSG00000078722.4	2.53429458	0.0277085	0.0125423	5.9969537	0.0143306	0.04323377	Gm12394
ENSMUSG00000078735.5	2.31684701	-1.57E-05	0.02060576	-0.000118	1	1	Il11ra2
ENSMUSG00000078747.10	8.25166799	0.01530463	0.01045814	2.03574743	0.15363864	0.29270768	Gm20878
ENSMUSG00000078762.11	89.0404631	-0.0029364	0.00360856	0.66058225	0.41635446	0.60753133	Haus5
ENSMUSG00000078763.3	18.444613	0.05567203	0.00754625	50.9846941	9.31E-13	1.96E-11	Slfn1
ENSMUSG00000078765.12	181.947819	-0.0072071	0.00294905	5.97020953	0.01454955	0.04380466	U2af1l4
ENSMUSG00000078768.3	14.3710987	0.00573722	0.00572988	0.9935994	0.31886424	0.50471323	Zfp566
ENSMUSG00000078771.12	71.1154836	0.06388878	0.00648039	85.5687916	2.24E-20	1.22E-18	Evi2a
ENSMUSG00000078773.12	21.0339692	0.01569118	0.00862598	3.05684384	0.08039769	0.17684044	Rad54b
ENSMUSG00000078776.10	7.07483912	-0.0181702	0.01532331	1.42457879	0.23265122	0.40058732	Fcgbpl1
ENSMUSG00000078779.5	53.1524362	0.00619941	0.00320823	3.74883195	0.05284443	0.1261527	Zfp59

ENSMUSG00000078780.8	26.6221916	0.06102344	0.00758982	59.2501246	1.39E-14	3.75E-13	Gm5150
ENSMUSG00000078783.2	1.34928886	0.10733313	0.01295976	66.6264461	3.28E-16	1.06E-14	Sirpd
ENSMUSG00000078784.9	716.689899	-0.000893	0.00412162	0.0466964	0.82891488	0.93343453	Rbis
ENSMUSG00000078786.10	212.46525	-0.0079996	0.00378809	4.53609693	0.03318718	0.08637749	Actmap
ENSMUSG00000078789.10	222.941613	-0.0118626	0.00253852	22.1450501	2.53E-06	2.03E-05	Dph1
ENSMUSG00000078794.5	19.0682511	0.03309296	0.00650869	25.579478	4.25E-07	3.93E-06	Dact3
ENSMUSG00000078798.5	854.782244	0.11534385	0.02229605	7.62188235	0.00576643	0.01998339	Sult2a1
ENSMUSG00000078799.6	58.2082153	0.10186655	0.02656216	13.4261252	0.00024814	0.00128943	Sult2a5
ENSMUSG00000078808.6	2.21445455	0.00802365	0.01804693	0.38384321	0.53555356	0.71991145	Vmn1r58
ENSMUSG00000078810.5	2.99960555	0.06415511	0.01508796	14.4603945	0.00014314	0.0007888	Gp6
ENSMUSG00000078812.11	5589.56246	-0.0054419	0.00281199	3.78026608	0.05186041	0.12418068	Eif5a
ENSMUSG00000078813.4	202.03322	-0.0062376	0.00250936	6.1712187	0.01298451	0.03965977	Leng1
ENSMUSG00000078815.9	1.43855443	-0.0058782	0.03836195	-0.034619	1	1	Cacng6
ENSMUSG00000078816.11	13.8123522	0.02282555	0.00993498	4.98643765	0.02554675	0.06975523	Prkcg
ENSMUSG00000078817.5	823.899972	-0.0311341	0.00511411	34.8990204	3.47E-09	4.49E-08	Nlrp12
ENSMUSG00000078851.5	86.1356498	0.00354752	0.00376167	0.86630819	0.35197939	0.54044566	H2ac25
ENSMUSG00000078853.9	868.646605	0.05253102	0.00475727	119.896453	6.67E-28	9.83E-26	Igtp
ENSMUSG00000078861.9	223.743123	-0.0146083	0.0037392	14.8414471	0.00011694	0.00065825	Zfp931
ENSMUSG00000078862.11	173.812728	-0.0028742	0.00456259	0.39048829	0.53204285	0.7166217	Gm14326
ENSMUSG00000078864.11	19.6346815	0.02269385	0.00789568	7.95846876	0.00478629	0.01702449	Gm14322
ENSMUSG00000078866.11	550.046374	-0.0012022	0.0050909	0.05596769	0.81298659	0.92330035	Zfp970
ENSMUSG00000078867.11	209.671643	0.00455409	0.00471729	0.93794027	0.33280811	0.51974614	Gm14418
ENSMUSG00000078868.4	6.2060659	0.00466258	0.00766177	0.3480948	0.55519361	0.73744418	Gm14412
ENSMUSG00000078869.4	2.11348027	0.0060687	0.0176984	0.37490277	0.54034389	0.72408108	Gm14409
ENSMUSG00000078870.11	94.2323282	0.00048558	0.00462309	0.0111926	0.91574492	0.9838637	Gm14410
ENSMUSG00000078872.11	2.05571566	-0.0143994	0.01315217	1.00332014	0.31650846	0.50196347	Gm14401
ENSMUSG00000078875.12	44.5592864	0.00458955	0.01697576	0.06748109	0.79504025	0.91318787	Gm14419
ENSMUSG00000078876.12	7.9761416	-0.0107452	0.01082861	0.96795523	0.32519067	0.51190997	Gm14408
ENSMUSG00000078877.12	148.973042	-0.0022093	0.00801446	0.07618125	0.78254076	0.90508382	Gm14295
ENSMUSG00000078878.10	127.781158	0.00424064	0.00475691	0.79565554	0.37239547	0.5631055	Gm14305
ENSMUSG00000078879.11	107.111818	-0.0108738	0.01004058	1.11797791	0.29035428	0.47182547	Zfp973
ENSMUSG00000078880.11	13.1162729	-0.0054086	0.01282642	0.16666902	0.68308928	0.83793412	Gm14308
ENSMUSG00000078881.11	39.9257563	-0.0149259	0.02426466	0.40414709	0.52495526	0.71030504	Gm14434
ENSMUSG00000078886.13	95.6924646	0.02135885	0.00887335	5.69880598	0.01697646	0.04970292	Gm2026
ENSMUSG00000078887.11	185.831158	-0.0120807	0.0063643	3.5922759	0.05804869	0.13634101	Gm6710
ENSMUSG00000078889.13	220.90724	-0.0147451	0.00783982	3.50908142	0.06103329	0.1419376	Gm14288
ENSMUSG00000078894.10	677.297445	-0.009097	0.01400845	-2.8996638	1	1	2210418O10Rik
ENSMUSG00000078896.12	64.0835592	-0.0138235	0.0170494	0.65147487	0.41958587	0.61096137	Zfp965
ENSMUSG00000078897.11	81.2968309	-0.0224951	0.00669787	11.131768	0.00084862	0.00380017	Gm4724
ENSMUSG00000078898.11	197.338532	-0.0053223	0.01990905	-0.1062826	1	1	Zfp968
ENSMUSG00000078899.10	510.623127	-0.0059378	0.00322582	3.39126683	0.06554257	0.1502758	Gm4631
ENSMUSG00000078901.10	1393.88482	-0.0260475	0.00477747	29.293868	6.22E-08	6.65E-07	Gm14440
ENSMUSG00000078902.4	7.90621479	0.00858255	0.00787529	1.15337995	0.28284272	0.46285496	Gm14443
ENSMUSG00000078903.11	97.5331503	0.00029124	0.00630856	0.00215345	0.96298725	1	Gm14391
ENSMUSG00000078905.9	4.59754559	0.00990517	0.01218081	0.69825969	0.40336908	0.59498832	Gm14393
ENSMUSG00000078908.11	236.430295	0.00016808	0.00242806	0.00467141	0.94550885	0.99995047	Mon1b
ENSMUSG00000078919.11	719.012112	-0.0129633	0.00339881	14.538741	0.00013731	0.00075915	Dpm1
ENSMUSG00000078920.4	677.184277	0.02134731	0.0046366	20.142505	7.19E-06	5.26E-05	Ifi47
ENSMUSG00000078921.4	200.788146	0.06323233	0.00713347	69.5576375	7.42E-17	2.62E-15	Tgtp2
ENSMUSG00000078922.10	346.95161	0.08361263	0.00863219	87.7352932	7.48E-21	4.24E-19	Tgtp1
ENSMUSG00000078923.11	1256.70774	-0.0009033	0.00240838	0.14023596	0.70804655	0.85548069	Ube2v1
ENSMUSG00000078931.5	370.608016	-0.005668	0.00304181	3.49786586	0.06144797	0.14264879	Pdf
ENSMUSG00000078937.9	85.2390732	-0.0097195	0.0088557	1.11286609	0.29145977	0.4731482	Cpt1b
ENSMUSG00000078941.10	676.047171	-0.013233	0.00337075	15.4043861	8.68E-05	0.00050257	Ak6
ENSMUSG00000078942.11	56.5864578	0.05080633	0.00516751	93.4096705	4.25E-22	2.88E-20	Naip6
ENSMUSG00000078945.11	160.118949	0.05105592	0.00356293	194.247154	3.76E-44	3.86E-41	Naip2
ENSMUSG00000078949.3	0.87699465	-0.0220441	0.02144711	1.71394486	0.19047434	0.34496874	R3hdml
ENSMUSG00000078954.11	3.90404136	0.05834634	0.0145635	11.2195794	0.00080939	0.00365084	Arhgap8
ENSMUSG00000078958.10	1.96017861	0.00949966	0.06609211	0.09392725	0.75924261	0.88976926	Atp6ap1l
ENSMUSG00000078963.4	50.5861243	-0.0368592	0.00561295	45.1896623	1.79E-11	3.19E-10	Hsbp1l1
ENSMUSG00000078964.3	1135.4262	-0.0104612	0.00477497	4.94254946	0.02620336	0.07120189	Ces1b

ENSMUSG00000078970.6	182.900922	-0.0098766	0.00248161	15.9681369	6.44E-05	0.00038552	Dnaaf10
ENSMUSG00000078974.11	1026.32866	-0.0112975	0.00485037	5.56159381	0.01835869	0.05301924	Sec61g
ENSMUSG00000078994.10	33.2101196	0.03086824	0.0048131	40.0293169	2.50E-10	3.79E-09	Zfp429
ENSMUSG00000078995.11	31.5132621	0.01656647	0.00510089	10.0334041	0.00153727	0.00641053	Zfp456
ENSMUSG00000079003.4	152.091612	-0.0011414	0.00351469	0.1060002	0.74474463	0.87968842	Samd1
ENSMUSG00000079012.12	6325.29108	0.01988589	0.00513106	14.8332327	0.00011745	0.00066081	Serpina3m
ENSMUSG00000079014.5	5.19010253	0.01900591	0.00934982	3.89488626	0.04843332	0.11757392	Serpina3i
ENSMUSG00000079015.3	100222.068	-0.0225517	0.00599261	13.8074224	0.00020253	0.00107857	Serpina1c
ENSMUSG00000079017.4	180.614029	0.02708611	0.00681112	15.6989997	7.43E-05	0.00043811	Ifi27l2a
ENSMUSG00000079018.11	23.4813548	0.02115897	0.01032299	4.22263767	0.03988811	0.10037351	Ly6c1
ENSMUSG00000079019.3	4.79390897	-0.0322211	0.0200648	2.37377865	0.12338807	0.24761601	Insl3
ENSMUSG00000079020.10	176.050787	0.01139474	0.00370861	9.22942171	0.00238157	0.00938071	Slc45a4
ENSMUSG00000079022.11	149.944648	0.04079973	0.01631755	6.23136382	0.0125507	0.0385143	Col22a1
ENSMUSG00000079036.11	176.285699	-0.0062867	0.00459838	1.82111184	0.17718099	0.32643265	Alkbh1
ENSMUSG00000079037.10	652.761162	0.00998372	0.00246442	16.1534112	5.84E-05	0.00035272	Prnp
ENSMUSG00000079038.4	9.3067921	0.03917629	0.00813	21.1948641	4.15E-06	3.19E-05	D130040H23Rik
ENSMUSG00000079042.11	2.91031253	0.10105833	0.02840692	14.5720642	0.0001349	0.00074706	Apela
ENSMUSG00000079043.4	187.405326	-0.0111619	0.00210483	27.9807247	1.23E-07	1.26E-06	Fastkd5
ENSMUSG00000079049.10	1.6640396	-0.0111284	0.01457895	0.56384005	0.45271715	0.64448254	Serpinb1c
ENSMUSG00000079051.6	1.75411508	0.00348302	0.00996402	0.16650669	0.68323527	0.83793412	Vinac1
ENSMUSG00000079055.11	10.3409723	0.04138237	0.00968488	16.4024534	5.12E-05	0.00031321	Slc8a3
ENSMUSG00000079056.13	13.5200106	0.03881933	0.00578203	44.0275446	3.24E-11	5.55E-10	Kcnip3
ENSMUSG00000079057.5	6365.38368	-0.0222259	0.00391251	31.5717166	1.92E-08	2.22E-07	Cyp4v3
ENSMUSG00000079071.3	1.05463578	-0.002977	0.01856234	0.01205669	0.91256566	0.98233956	Slc28a2b
ENSMUSG00000079083.4	82.3158166	0.00847987	0.00287424	8.57107774	0.00341545	0.01275844	Jrkl
ENSMUSG00000079084.13	176.718659	0.02202963	0.00303187	51.7648253	6.26E-13	1.35E-11	Ccdc82
ENSMUSG00000079104.5	402.549106	-0.0029904	0.0027965	1.12482175	0.28888257	0.47003203	Prps1l3
ENSMUSG00000079105.5	25.2009632	0.02600475	0.00678828	13.6402551	0.00022139	0.00116737	C7
ENSMUSG00000079108.7	552.259271	0.00611476	0.00273644	4.94856341	0.02611236	0.07097097	Srp54c
ENSMUSG00000079109.12	61.0862275	0.01529099	0.00466966	10.3397475	0.00130196	0.00553397	Pms2
ENSMUSG00000079110.13	26.466489	-0.0466532	0.00706313	40.5829728	1.88E-10	2.90E-09	Capn3
ENSMUSG00000079111.4	1505.58391	-0.0020873	0.0021969	0.90155234	0.34236581	0.53031064	Kdelr2
ENSMUSG00000079144.8	166.419368	-0.0013182	0.002627	0.24940366	0.61749529	0.78862053	A130010J15Rik
ENSMUSG00000079157.5	5.77627401	0.02838307	0.01877868	2.52357313	0.11215619	0.22962812	Nalf1
ENSMUSG00000079164.11	148.552835	-0.0022512	0.00549238	0.15783037	0.69116195	0.84343751	Tlr5
ENSMUSG00000079165.11	20.3953388	0.00591324	0.00962881	0.38756986	0.53357956	0.71803546	Sap25
ENSMUSG00000079168.5	11.1971039	0.01328427	0.02151001	0.63619482	0.42509208	0.61669466	Cd209g
ENSMUSG00000079173.12	3.79732405	0.01668501	0.01554914	1.00039514	0.31721491	0.50270987	Zan
ENSMUSG00000079177.5	6.71609479	-0.0219553	0.00853381	6.29300604	0.01212153	0.03746048	Fam228a
ENSMUSG00000079184.11	493.63169	-0.0055762	0.0035636	2.41070877	0.12050779	0.24303521	Mphosph8
ENSMUSG00000079186.4	0.68483148	0.06734011	0.0189767	12.9549457	0.00031908	0.00160841	Gzmc
ENSMUSG00000079190.4	16.2702182	0.25487212	0.01565459	160.264951	9.90E-37	4.67E-34	ENSMUSG00000079190
ENSMUSG00000079192.3	1.41028998	0.05781479	0.04508968	2.75089073	0.09720027	0.20564814	ENSMUSG00000079192
ENSMUSG00000079197.11	2313.75082	0.00178769	0.0039174	0.21143851	0.64564224	0.81052363	Psme2
ENSMUSG00000079215.9	480.484506	-0.0008441	0.002668	0.09956919	0.75234723	0.88503052	Zfp664
ENSMUSG00000079222.3	1.58650503	0.12918793	0.01979955	39.8156118	2.79E-10	4.19E-09	ENSMUSG00000079222
ENSMUSG00000079227.11	252.923779	0.05532264	0.00808911	41.2768413	1.32E-10	2.08E-09	Ccr5
ENSMUSG00000079243.6	48.9571617	-0.0184414	0.01085451	1.90140045	0.16792165	0.31316518	Xirp1
ENSMUSG00000079244.4	2.18069416	0.06366438	0.01304088	24.2696438	8.37E-07	7.29E-06	Gm5622
ENSMUSG00000079259.3	1.97922313	0.04336709	0.01404126	10.030802	0.00153944	0.00641733	Trim71
ENSMUSG00000079260.4	43.0272349	0.00366523	0.00380915	0.93297212	0.33409181	0.52101887	Tmppe
ENSMUSG00000079262.4	10.0007999	0.01057406	0.01937186	0.49102199	0.48347177	0.67271342	Slco1a6
ENSMUSG00000079263.9	3.71202724	-0.0486081	0.00989804	24.4825582	7.50E-07	6.58E-06	Gm6614
ENSMUSG00000079283.3	81.4300158	-0.0018919	0.00403405	0.22287586	0.63685734	0.80373039	2310009B15Rik
ENSMUSG00000079293.13	401.20336	0.08860308	0.00756276	117.512637	2.22E-27	2.94E-25	Clec7a
ENSMUSG00000079298.10	5.41999588	0.07374258	0.01089947	40.8384602	1.65E-10	2.57E-09	Klrb1b
ENSMUSG00000079316.11	1426.96391	-0.0046404	0.00252295	3.35037031	0.0671899	0.15320388	Rab9
ENSMUSG00000079317.11	125.225027	0.00249333	0.00275185	0.82019114	0.36512429	0.5548854	Trappc2
ENSMUSG00000079324.12	1.74295445	0.00278766	0.06488012	0.02736934	0.86860017	0.95804356	4932414N04Rik
ENSMUSG00000079334.9	638.576583	-0.0134824	0.00380937	12.2540247	0.00046426	0.00223925	Naa80
ENSMUSG00000079339.8	14.4360599	0.05325127	0.00824314	37.7958832	7.85E-10	1.10E-08	Ifit1bl1

ENSMUSG00000079343.5	9.01562728	0.00981255	0.01178772	0.79834191	0.37158957	0.56214939	C1s2
ENSMUSG00000079355.6	35.5243679	-0.0107481	0.00484764	4.81346663	0.02823818	0.07566756	Ackr4
ENSMUSG00000079357.10	18.8420248	0.01348405	0.01377302	1.04893252	0.30575307	0.49067781	Gm11100
ENSMUSG00000079362.6	78.6025255	0.0095193	0.02016223	-2.0858013	1	1	Gm43302
ENSMUSG00000079363.8	149.47408	0.04160551	0.00557973	53.044888	3.26E-13	7.31E-12	Gbp4
ENSMUSG00000079410.5	1.16832502	0.03371647	0.01834682	3.19205949	0.07399673	0.16557129	Gm2897
ENSMUSG00000079415.3	12.7960069	-0.0318199	0.007274	18.8139019	1.44E-05	9.92E-05	Cntf
ENSMUSG00000079418.6	320.473208	-0.0042065	0.00278752	2.25623277	0.13307744	0.26256896	Atg4a
ENSMUSG00000079419.5	155.203148	0.07935073	0.00769637	92.5055769	6.71E-22	4.39E-20	Ms4a6c
ENSMUSG00000079424.10	1.3482713	0.05306921	0.02460357	4.77720877	0.02883883	0.07693616	Pramel47
ENSMUSG00000079426.14	1200.05384	0.01018756	0.00413812	5.96099457	0.01462579	0.04398927	Arpc4
ENSMUSG00000079427.11	455.708498	0.00075591	0.00302729	0.06201439	0.80333999	0.91779337	Mthfsl
ENSMUSG00000079429.11	49.9718433	-0.0066339	0.00675637	0.95106987	0.32944712	0.51633596	Mroh2a
ENSMUSG00000079434.9	31.0641408	-0.0052131	0.00440753	1.36472902	0.24271958	0.4129542	Neu2
ENSMUSG00000079435.10	2111.88044	0.00349869	0.00368266	0.91103458	0.33984006	0.52757636	Rpl36a
ENSMUSG00000079436.4	1.97793583	0.00163707	0.02605565	0.04119445	0.83916305	0.94004359	Kcnj13
ENSMUSG00000079442.13	44.8422993	0.05344109	0.00636201	68.5264662	1.25E-16	4.27E-15	St6galnac4
ENSMUSG00000079444.10	12.4577763	-0.0097991	0.00584223	2.72216041	0.09896399	0.20848201	Gm21981
ENSMUSG00000079445.4	2.46220952	0.02247424	0.01283039	2.91324712	0.08785483	0.18989589	B3gnt7
ENSMUSG00000079450.13	16.2381048	-0.0052947	0.006723	0.63284704	0.42631292	0.61789625	Cldn34c1
ENSMUSG00000079457.12	44.6441627	0.06429434	0.00480162	156.247202	7.48E-36	3.04E-33	Gm7609
ENSMUSG00000079465.9	52.5878426	0.03407401	0.00745894	19.0655136	1.26E-05	8.78E-05	Col4a3
ENSMUSG00000079469.12	194.643545	-0.0054091	0.00273265	3.8708727	0.04913097	0.11887598	Pigb
ENSMUSG00000079470.9	259.436917	0.02513129	0.00397791	39.6380643	3.06E-10	4.55E-09	Utp14b
ENSMUSG00000079477.10	3763.76484	0.00245977	0.00186848	1.73133146	0.18824095	0.3419742	Rab7
ENSMUSG00000079478.10	133.550244	-0.008117	0.00464784	3.11056817	0.07778582	0.17231674	Znrd2
ENSMUSG00000079480.4	309.918607	-0.0047548	0.00335387	2.01611942	0.15563645	0.29553508	Pin4
ENSMUSG00000079481.12	44.8400609	0.0226722	0.00515442	18.7223904	1.51E-05	0.00010379	Nhsl2
ENSMUSG00000079484.13	994.84532	-0.0146452	0.00263296	31.0195042	2.55E-08	2.90E-07	Phyhd1
ENSMUSG00000079487.12	392.389493	0.00324616	0.00451842	0.50709303	0.47639997	0.6666095	Med12
ENSMUSG00000079491.11	56.0853914	0.04416573	0.00463475	88.8276331	4.31E-21	2.51E-19	H2-T10
ENSMUSG00000079492.3	2.81615716	0.06116257	0.0247547	6.4203894	0.01128174	0.03531871	Gm11127
ENSMUSG00000079494.3	186.120491	-0.0594912	0.00764512	52.3185269	4.72E-13	1.04E-11	Nat8f5
ENSMUSG00000079495.3	16.4226853	-0.0671642	0.01145987	29.314234	6.15E-08	6.58E-07	Nat8f6
ENSMUSG00000079497.5	2.44240837	0.00476174	0.00956959	0.28619042	0.59267221	0.76819073	Gm13420
ENSMUSG00000079502.9	1.90650544	0.02486239	0.01012436	5.87472376	0.01535978	0.04577079	Cfap77
ENSMUSG00000079507.11	258.68345	0.07898933	0.00550511	185.36094	3.27E-42	2.97E-39	H2-Q1
ENSMUSG00000079508.9	502.361886	-0.0218215	0.0032259	46.1613611	1.09E-11	2.00E-10	Apoo
ENSMUSG00000079509.11	366.768377	0.0031947	0.00281775	1.28191103	0.25754401	0.4310854	Zfx
ENSMUSG00000079523.9	320.715014	0.05267145	0.00575926	78.7201809	7.16E-19	3.23E-17	Tmsb10
ENSMUSG00000079531.2	1.21147212	0.01372259	0.02381203	0.36126756	0.54780311	0.73058999	Pwwp4d
ENSMUSG00000079534.2	2.9159262	0.02763882	0.0151578	3.92790626	0.04749104	0.11571525	Pwwp4c
ENSMUSG00000079547.5	134.52111	0.07290848	0.0063586	118.401448	1.42E-27	1.94E-25	H2-DMb1
ENSMUSG00000079550.10	40.2712574	-0.0395602	0.00679158	32.725343	1.06E-08	1.28E-07	Mpp4
ENSMUSG00000079553.13	11.2061089	0.03250113	0.01069301	8.89912224	0.00285308	0.0109419	Kifc1
ENSMUSG00000079554.3	2.15329231	0.04634861	0.01152923	15.80566	7.02E-05	0.00041628	Aox2
ENSMUSG00000079555.3	265.628174	0.00460202	0.0046654	0.94056172	0.33213342	0.51907459	Haus3
ENSMUSG00000079557.12	950.665498	-0.0084548	0.0020255	17.33746	3.13E-05	0.00019988	Marchf2
ENSMUSG00000079559.6	2.69806162	0.03686424	0.00968794	14.8593684	0.00011583	0.00065265	Pou2af3
ENSMUSG00000079560.14	10.4361114	0.01609937	0.0064884	5.89382494	0.01519406	0.04533424	Hoxa3
ENSMUSG00000079562.8	542.933365	0.00037759	0.00202464	0.03455241	0.85253668	0.94920341	Maea
ENSMUSG00000079563.11	2166.01653	0.00716588	0.00421793	2.82097279	0.09304	0.19871787	Pglyrp2
ENSMUSG00000079579.4	1.44580318	-0.0192576	0.184706	0.02207072	0.88189912	0.9650389	Gm6760
ENSMUSG00000079588.4	54.7142931	0.02149624	0.03149026	0.3287789	0.566379	0.74642373	Tmem182
ENSMUSG00000079592.10	43.0959237	0.01242067	0.00449008	7.52586796	0.00608192	0.02090169	C1qtnf5
ENSMUSG00000079605.6	73.6111045	0.00867685	0.00885797	0.90490478	0.34146995	0.52930536	Zbtb9
ENSMUSG00000079608.11	4.61654558	0.00116234	0.01040889	0.01160268	0.91422118	0.98312004	Stard6
ENSMUSG00000079610.10	80.703985	-0.0041135	0.00434468	0.89668928	0.34367099	0.53177354	Ankrd39
ENSMUSG00000079614.5	853.866542	0.00049051	0.00149174	0.10729649	0.74324329	0.87866779	Seh1l
ENSMUSG00000079641.4	2748.81026	0.00119676	0.0045487	0.0703451	0.79083498	0.91063974	Rpl39
ENSMUSG00000079652.9	1.33108079	-0.01946	0.01462158	2.02484643	0.15474457	0.29418628	Garin1a

ENSMUSG00000079657.11	2.30405041	0.02730322	0.01240891	4.35729793	0.03685071	0.09407521	Rab26
ENSMUSG00000079658.10	1048.71427	-0.0118935	0.00318201	13.6717845	0.0002177	0.00115025	Eloc
ENSMUSG00000079659.6	1578.05778	-0.0088875	0.00311817	7.9861561	0.00471364	0.01679901	Tmem243
ENSMUSG00000079677.3	267.680992	-0.0083544	0.00338058	6.17604524	0.01294914	0.03956197	Fdx2
ENSMUSG00000079685.11	20.7515925	0.01666883	0.00804075	3.97974795	0.04605045	0.11288152	Ulbp1
ENSMUSG00000079698.8	2.67414779	0.00923776	0.01826667	0.45285637	0.50098162	0.68782008	Vmn2r93
ENSMUSG00000079707.11	3.78928985	0.03510986	0.02199284	2.35909305	0.12455458	0.24928817	Dynlt2a1
ENSMUSG00000079710.12	21.8068766	-0.0080923	0.006444	1.51036371	0.2190836	0.38358739	Dynlt2a2
ENSMUSG00000079800.3	2.14673665	0.09328811	0.01908684	21.1334996	4.28E-06	3.29E-05	ENSMUSG00000079800
ENSMUSG00000079808.4	15.283528	0.23701756	0.01486089	163.808806	1.67E-37	8.73E-35	ENSMUSG00000079808
ENSMUSG00000079845.9	17.1989576	0.04560775	0.0165642	5.84672769	0.01560604	0.04636405	Xlr4a
ENSMUSG00000079852.5	1.2944334	0.01626215	0.01646398	0.88720668	0.3462354	0.53457827	Klra4
ENSMUSG00000079853.6	1.25889646	0.03407999	0.01590301	4.71846816	0.02984031	0.07913354	Klra1
ENSMUSG00000079941.3	59.3476652	-0.0168113	0.00551461	9.30240018	0.00228854	0.00906879	Cox5b-ps
ENSMUSG00000080115.4	2.20690856	0.02412181	0.01268685	3.69736144	0.05449859	0.12956392	Eef1akmt3
ENSMUSG00000080268.5	286.660277	-0.0006423	0.00311199	0.04256054	0.83655497	0.9384089	Brms1
ENSMUSG00000080316.12	88.8024295	0.02263788	0.00613061	13.6908013	0.00021551	0.00114022	Spaca6
ENSMUSG00000080717.12	132.181188	0.00101386	0.00342093	0.08622624	0.76903068	0.89618877	B230307C23Rik
ENSMUSG00000081058.6	20.7246658	-0.0249739	0.008776	7.32958706	0.00678286	0.02299831	H3c15
ENSMUSG00000081534.4	822.218658	0.00573425	0.00312998	3.34126761	0.06756254	0.15384535	Slc48a1
ENSMUSG00000081665.3	29.0295476	0.06626882	0.01106982	30.692059	3.02E-08	3.40E-07	Pira1
ENSMUSG00000081724.5	2.48914753	0.01608918	0.03609619	0.37680381	0.53931876	0.72318797	Or10al7
ENSMUSG00000081984.4	5.87413597	-0.0112896	0.01685298	0.31965032	0.57181787	0.75110805	Dnajb3
ENSMUSG00000082229.2	13.8044429	0.00356656	0.02312836	0.03079676	0.86069458	0.95372587	Nap1l2
ENSMUSG00000082361.7	51.4805634	0.02318739	0.0047032	23.4118299	1.31E-06	1.10E-05	Btc
ENSMUSG00000082932.4	1.93782028	0.0018786	0.01091998	0.01945394	0.88907294	0.96954458	Cyp2j8
ENSMUSG00000083012.10	38.7182265	0.0089227	0.0120664	0.52006739	0.47081293	0.66169669	Fam220a
ENSMUSG00000083282.4	406.080778	-0.0133443	0.00323351	17.1346726	3.48E-05	0.00022083	Ctsf
ENSMUSG00000083649.6	23.1729139	0.01176682	0.00559298	4.41820393	0.03555744	0.09142678	Rasl2-9
ENSMUSG00000083695.7	1.85281599	0.06723463	0.02571457	7.07149139	0.007832	0.02592641	Rnf138rt1
ENSMUSG00000083855.7	1.5584657	0.01803566	0.02043275	1.2999491	0.25422252	0.42653494	Or5d45
ENSMUSG00000084128.11	1356.44872	-0.0072798	0.00374663	3.6877573	0.05481327	0.13013201	Esrp2
ENSMUSG00000084174.4	1.91769399	-0.0453873	0.02959706	2.07060643	0.1501618	0.28768713	Sycn
ENSMUSG00000084262.6	1.06867045	0.00595943	0.03631139	0.15721492	0.69173372	0.84394949	Or51p1
ENSMUSG00000084786.10	850.298624	-0.0037382	0.00323562	1.33705535	0.24755393	0.41885366	Ubl5
ENSMUSG00000084845.11	4.82330295	0.03131999	0.00974761	9.80186505	0.00174335	0.00715729	Tmem240
ENSMUSG00000084883.3	238.160353	0.00198613	0.00292889	0.45532302	0.49981792	0.68698749	Ccdc85c
ENSMUSG00000084957.4	383.812923	-0.0028156	0.00364392	0.59607376	0.44008	0.6320238	Bbip1
ENSMUSG00000085007.4	2.10538232	0.02396822	0.02015919	1.45845078	0.22717678	0.3934928	Smim43
ENSMUSG00000085139.2	1.86548199	0.0111285	0.01198238	0.88138819	0.34782175	0.5360443	Sertm2
ENSMUSG00000085272.8	7.09267434	0.0527872	0.02121102	0.85870154	0.35410231	0.54271604	Sbk3
ENSMUSG00000085486.3	1.11950041	-0.017251	0.04355936	0.15445554	0.69431334	0.84580005	Ccdc200
ENSMUSG00000085793.4	169.59311	-0.0074525	0.00307426	5.94966465	0.01472009	0.04421087	Lin52
ENSMUSG00000085795.9	125.643547	0.01294315	0.00462969	7.55523063	0.00598361	0.02061489	Zfp703
ENSMUSG00000085925.4	1.61690804	0.01470609	0.02445515	0.87008559	0.35093161	0.53936355	Rtl1
ENSMUSG00000086040.9	172.496193	0.00817729	0.00526425	2.41626698	0.12008078	0.24236054	Wipf3
ENSMUSG00000086158.4	75.6806538	-0.0116047	0.00258001	20.2581423	6.77E-06	4.98E-05	Pierce2
ENSMUSG00000086316.9	39.7919179	0.00700828	0.00674381	1.07571104	0.29965901	0.48366232	Nbdy
ENSMUSG00000086322.8	8.66318702	-0.0127594	0.01177486	1.08293499	0.29804191	0.48174454	Zfp593os
ENSMUSG00000086338.3	1.13524171	0.00506919	0.03436679	0.11256343	0.73724437	0.87469707	Or8h6
ENSMUSG00000086564.10	3.31728049	0.09301802	0.01116041	57.606723	3.20E-14	8.23E-13	Cd101
ENSMUSG00000086587.9	48.7185039	-0.0148207	0.00516447	8.47125841	0.00360802	0.01337609	Gm11837
ENSMUSG00000086596.3	2.00064408	0.00225437	0.0207739	0.03606845	0.8493742	0.94715805	Susd5
ENSMUSG00000086727.10	4.19596087	-0.0088476	0.00876344	0.97265408	0.32401915	0.51072685	4931428L18Rik
ENSMUSG00000086784.3	1222.5502	-0.0148801	0.00332999	19.9623867	7.90E-06	5.71E-05	Isoc2a
ENSMUSG00000087006.4	103.399437	0.00546445	0.0054245	0.99220156	0.31920489	0.50517585	Gm13889
ENSMUSG00000087119.5	3.16403313	-0.0040859	0.00871662	0.20768473	0.64858817	0.81215377	Atg4a-ps
ENSMUSG00000087141.2	1411.41928	-0.0324372	0.00492826	42.0770744	8.77E-11	1.42E-09	Plcxd2
ENSMUSG00000087260.6	629.610409	-0.009439	0.0030898	9.35211831	0.00222728	0.00887075	Lamtor5
ENSMUSG00000087370.4	558.813167	0.00847909	0.00303505	7.56527546	0.00595036	0.02051229	Tmem170b
ENSMUSG00000087385.9	70.5568144	-0.0046692	0.00324664	2.03235646	0.1539817	0.29319547	Frg2f1

ENSMUSG00000087403.10	57.9852134	-0.0002528	0.00687661	0.0013251	0.97096184	1	Kantr
ENSMUSG00000087408.11	1.82777294	0.04430999	0.01848279	6.74239119	0.00941483	0.03036487	Cers1
ENSMUSG00000087598.10	94.8398447	0.00524318	0.00338554	2.36989975	0.12369499	0.2480631	Zfp111
ENSMUSG00000087651.4	2.79411805	0.03609871	0.01805393	4.15855769	0.04142466	0.10352736	1500009L16Rik
ENSMUSG00000087679.3	1093.34938	-0.0058264	0.0020129	8.275815	0.00401766	0.01468473	Tmem250-ps
ENSMUSG00000087687.4	713.706885	-0.0098155	0.00619242	2.49636839	0.11410916	0.23277794	Pet100
ENSMUSG00000089661.10	3.9103076	0.00317679	0.01210705	0.06854015	0.79347429	0.91207587	Mia
ENSMUSG00000089665.3	17.5281902	0.00912302	0.00671501	1.70717198	0.1913527	0.34613428	Fcor
ENSMUSG00000089669.8	42.3861778	0.05178194	0.00725755	46.5178003	9.08E-12	1.69E-10	Tnfsf13
ENSMUSG00000089675.2	18.7130649	0.06402886	0.0292599	1.30754175	0.25284022	0.42499457	Ugt1a8
ENSMUSG00000089678.9	2727.53662	0.001525	0.00408261	0.13913147	0.70914598	0.85615534	Agxt2
ENSMUSG00000089682.11	368.472109	-0.007216	0.00276027	6.8554268	0.00883724	0.02875366	Bcl2l2
ENSMUSG00000089694.3	21.616167	-0.0203544	0.01526463	1.53975023	0.21465499	0.37766487	Nat8f7
ENSMUSG00000089704.10	748.864665	0.00166351	0.00322742	0.26207516	0.60869806	0.78119232	Galnt2
ENSMUSG00000089715.13	180.298468	0.01650452	0.00499464	10.8105706	0.00100922	0.00443103	Cbx6
ENSMUSG00000089722.3	3.0974714	0.07824543	0.03464206	2.6444254	0.10391372	0.21619346	Cd300ld5
ENSMUSG00000089727.3	1.56333345	0.03512651	0.01636246	4.55162171	0.03288758	0.08576808	Klra8
ENSMUSG00000089736.3	56.9133027	0.00198044	0.00688065	0.08263628	0.77375609	0.89913896	Tgfbr3l
ENSMUSG00000089739.3	11.2231613	-0.0509718	0.05045054	-0.4151662	1	1	Gm20431
ENSMUSG00000089762.4	16.4233244	0.00972723	0.00643232	2.35119673	0.12518687	0.25031006	Ier5l
ENSMUSG00000089768.3	16.1089175	0.006941	0.00931434	0.73531337	0.39116632	0.58248935	Tmsb15b1
ENSMUSG00000089774.3	187.172993	-0.0145802	0.00501598	7.8694259	0.00502776	0.01777616	Slc5a3
ENSMUSG00000089789.2	6.19761061	0.00711115	0.00676862	1.09337898	0.29572375	0.47875217	Rdh1
ENSMUSG00000089798.11	4.48142875	0.03133554	0.01337164	4.10615101	0.0427275	0.10619292	1700028K03Rik
ENSMUSG00000089809.10	318.949556	0.03226287	0.00489286	42.0538409	8.88E-11	1.43E-09	Rasgef1b
ENSMUSG00000089824.11	192.702055	0.00558147	0.00391921	1.97605631	0.15980648	0.30131871	Rbm12
ENSMUSG00000089832.9	125.371198	0.00927656	0.00432473	4.6453952	0.03113697	0.08199135	Shkbp1
ENSMUSG00000089844.8	9.52675939	0.1410864	0.01307674	89.0611089	3.83E-21	2.26E-19	Sp140l1
ENSMUSG00000089847.9	484.99176	-0.0063598	0.0042371	2.2835586	0.13075156	0.25897769	Timm10b
ENSMUSG00000089857.10	42.658847	0.00788389	0.00429725	3.23623097	0.07202616	0.16201904	Zfp882
ENSMUSG00000089862.9	336.410103	0.00497857	0.00250387	3.93818667	0.04720163	0.11522435	Umad1
ENSMUSG00000089865.3	14.455886	-0.0229544	0.02507583	0.66703541	0.41408711	0.60545897	Gm44503
ENSMUSG00000089872.11	183.659865	0.00576505	0.00318002	3.24764388	0.07152621	0.16108251	Rps6kc1
ENSMUSG00000089873.9	861.173248	-0.1137367	0.01152766	76.3326797	2.40E-18	1.01E-16	Mup13
ENSMUSG00000089876.4	162.470244	-0.0007536	0.00409566	0.0326884	0.85652508	0.95130912	Tmem102
ENSMUSG00000089901.2	13.4462744	0.03201128	0.00849318	13.4944386	0.00023927	0.00124938	Gm8113
ENSMUSG00000089911.5	686.684452	-0.0012186	0.00170985	0.50198023	0.47863132	0.66856204	Mfsd14a
ENSMUSG00000089917.8	287.381739	-0.008175	0.00280916	8.55380949	0.003448	0.0128617	Uckl1
ENSMUSG00000089929.2	78.1735198	0.1098583	0.00872011	125.550301	3.86E-29	6.69E-27	Bcl2a1b
ENSMUSG00000089942.10	138.779891	0.05458374	0.00695431	53.5859637	2.48E-13	5.65E-12	Pira2
ENSMUSG00000089943.2	2555.79859	0.02832516	0.00658199	13.3232079	0.00026214	0.00135441	Ugt1a5
ENSMUSG00000089945.14	106.202034	0.01419345	0.01828169	0.47682883	0.48986225	0.67776682	Pakap
ENSMUSG00000089951.10	75.2630162	-0.0127327	0.0093522	1.60974598	0.20452751	0.36451295	Zfp968-ps
ENSMUSG00000089960.2	9789.34716	0.01220252	0.00565901	4.68472558	0.03043192	0.08044915	Ugt1a1
ENSMUSG00000089984.8	1.325594	-0.0118981	0.01267737	0.9155332	0.33865055	0.52631865	Fbxo24
ENSMUSG00000089989.11	10.6599543	0.01714451	0.02360817	0.5356142	0.46425605	0.65523777	Gm45713
ENSMUSG00000089996.8	2.74926237	-0.0182362	0.02043183	0.81697595	0.36606581	0.55592224	Tmsb15b2
ENSMUSG00000090000.3	598.355389	-0.0113629	0.00374879	9.01042299	0.00268444	0.01040001	Ier3ip1
ENSMUSG00000090015.10	175.837373	-0.0068524	0.00461701	2.1469176	0.14285644	0.27708981	Gm15446
ENSMUSG00000090019.6	60.9182758	0.02279368	0.00462103	23.2744811	1.40E-06	1.18E-05	Gimap1
ENSMUSG00000090035.4	287.578929	-0.0055426	0.00262716	4.34843778	0.03704291	0.09440574	Galnt4
ENSMUSG00000090053.10	3.52865648	0.02946813	0.01554988	3.95081149	0.04684877	0.11446961	Pakap
ENSMUSG00000090061.10	7.71327686	0.00842542	0.01046265	0.63088517	0.42703081	0.61861091	Nwd2
ENSMUSG00000090066.3	19.498139	0.01235676	0.01479457	0.79170596	0.37358478	0.56443345	1110002E22Rik
ENSMUSG00000090071.5	15.1136631	0.00504464	0.02535107	0.0499968	0.82306884	0.92955242	Cdk5r2
ENSMUSG00000090077.10	84.2677347	0.00425533	0.00551269	0.58414581	0.44469187	0.63697938	Lime1
ENSMUSG00000090083.12	220.516198	-0.006071	0.00206503	8.7041582	0.00317485	0.01199071	Rnf8
ENSMUSG00000090084.8	7.80354896	0.0029827	0.00862916	0.11714695	0.73214969	0.87157259	Srpx
ENSMUSG00000090093.10	182.49224	-0.0059823	0.00370401	2.58421203	0.10793411	0.22284085	Gm14399
ENSMUSG00000090097.9	0.83911229	-0.0066579	0.04704504	-0.0160468	1	1	Or4p21
ENSMUSG00000090100.8	158.132325	0.0039242	0.00463169	0.70870207	0.39987503	0.59166468	Ttbk2

ENSMUSG00000090110.9	72.5241398	0.00397691	0.00356934	1.21456686	0.27042955	0.44725835	Cmc4
ENSMUSG00000090112.9	391.628217	0.00247804	0.00318038	0.59485315	0.44054855	0.63254265	Shprh
ENSMUSG00000090115.8	87.7922114	0.00749932	0.00507123	2.1181714	0.14556024	0.281132	Usp49
ENSMUSG00000090121.11	3.01657542	0.00020437	0.03115059	-0.0004933	1	1	Abhd12b
ENSMUSG00000090124.8	214.559749	0.01184676	0.00542983	4.75194954	0.02926512	0.07785328	Ugt1a7c
ENSMUSG00000090125.4	221.388113	0.0611166	0.01495703	18.1378552	2.05E-05	0.00013721	Pou3f1
ENSMUSG00000090137.9	6424.59615	-0.0039925	0.00300557	1.76088514	0.18451415	0.33688913	Uba52
ENSMUSG00000090145.8	2396.71553	-0.0015415	0.00495883	-5.8104013	1	1	Ugt1a6b
ENSMUSG00000090150.11	6060.29931	-0.0107444	0.00265287	16.2848952	5.45E-05	0.00033171	Acad11
ENSMUSG00000090164.9	16.619034	0.04485684	0.00753131	31.2594719	2.26E-08	2.58E-07	BC035044
ENSMUSG00000090165.9	45.6718794	-0.0037693	0.00913488	-1.7489843	1	1	Ugt1a10
ENSMUSG00000090171.2	11.7123921	0.00553459	0.01307223	0.18310963	0.66871438	0.82724732	Ugt1a2
ENSMUSG00000090173.9	2.58355693	0.02969982	0.01250056	4.88786238	0.02704615	0.07304598	Fbxw10
ENSMUSG00000090175.2	1469.5272	0.00051053	0.00695112	-1.1262894	1	1	Ugt1a9
ENSMUSG00000090176.2	0.8445207	0.08683783	0.01797873	24.503454	7.42E-07	6.51E-06	Cd200r2
ENSMUSG00000090202.2	1.2244968	-0.0152963	0.07289726	0.05547422	0.81379766	0.92377667	4930503B20Rik
ENSMUSG00000090210.8	23.3306634	-0.0411434	0.00673483	37.0303554	1.16E-09	1.59E-08	Itga10
ENSMUSG00000090213.2	364.732664	0.0206951	0.00367593	30.1616602	3.97E-08	4.37E-07	Peds1
ENSMUSG00000090215.11	1.78939041	0.04791976	0.01705786	8.10402536	0.00441671	0.01591877	Trim34b
ENSMUSG00000090223.3	7.25353744	-0.0034769	0.01523216	0.07616157	0.78256815	0.90508382	Pcp4
ENSMUSG00000090231.11	19989.854	0.00464137	0.00440547	1.11808324	0.29033156	0.47182547	Cfb
ENSMUSG00000090236.4	7.28957075	-0.0168012	0.00805258	4.33608016	0.03731273	0.09492922	Car15
ENSMUSG00000090247.8	909.501916	-0.0055329	0.00322542	2.97087929	0.08477576	0.18462799	Bloc1s1
ENSMUSG00000090258.8	858.660467	-0.0070339	0.0043386	2.62260081	0.10535164	0.2185421	Churc1
ENSMUSG00000090264.2	500.506794	-0.0602805	0.00739509	64.379274	1.03E-15	3.17E-14	Eif4ebp3
ENSMUSG00000090266.11	706.270988	-0.0095536	0.00334405	8.08747278	0.00445723	0.01603541	Mettl23
ENSMUSG00000090272.10	565.272761	0.03028401	0.00513283	33.3616428	7.65E-09	9.41E-08	Mndal
ENSMUSG00000090273.4	3.24027278	-0.0068938	0.00826205	0.70378654	0.40151428	0.59310552	Prr22
ENSMUSG00000090290.3	91.5091968	-0.0127649	0.00333227	14.8899496	0.00011397	0.00064326	Tarbp1
ENSMUSG00000090298.3	16.655078	0.03739795	0.02147019	3.5818959	0.05841245	0.13692268	Sult3a2
ENSMUSG00000090304.5	1.88438314	0.01344416	0.02533619	0.44042087	0.50691939	0.69375657	Vmn2r99
ENSMUSG00000090315.3	2.39235427	-0.0132267	0.02856585	0.20349999	0.65191047	0.81405974	Vmn2r104
ENSMUSG00000090346.3	1.41551111	0.01314591	0.01920903	0.70212158	0.40207173	0.59372583	Vmn1r5
ENSMUSG00000090362.3	2.51512114	0.00568215	0.01756548	0.14803893	0.70041652	0.85049547	Vmn2r79
ENSMUSG00000090369.4	41.5237675	-0.0117445	0.00655994	3.11458731	0.07759413	0.17198903	4933411K16Rik
ENSMUSG00000090383.3	0.93380878	-0.0030429	0.03382099	-0.0116237	1	1	Vmn2r58
ENSMUSG00000090394.9	291.713796	0.01112587	0.0058825	3.53122732	0.06022319	0.14037635	4930523C07Rik
ENSMUSG00000090411.3	2.19612633	0.0110567	0.02131968	0.49324802	0.482482	0.67165322	Vmn1r174
ENSMUSG00000090417.5	1.54475495	0.01409056	0.02976654	0.34578572	0.55650848	0.73860835	Vmn2r94
ENSMUSG00000090451.2	1.89700489	-0.0052038	0.01811759	0.05558142	0.81362114	0.92370197	Gm6133
ENSMUSG00000090486.3	12.7086792	0.04645225	0.01451706	8.13773973	0.00433533	0.01567337	BC035947
ENSMUSG00000090523.3	177.288857	0.01976394	0.00351992	32.5874302	1.14E-08	1.37E-07	Gypc
ENSMUSG00000090553.9	624.259813	-0.0048596	0.00424917	1.3218332	0.25026331	0.42195371	Snrpe
ENSMUSG00000090619.3	2.78017336	-0.0005637	0.01548896	-0.0063492	1	1	Vmn2r60
ENSMUSG00000090626.10	36.3491542	0.01843566	0.0066923	7.24144163	0.00712398	0.02396949	Tex9
ENSMUSG00000090634.2	1.15837484	-0.0038115	0.04234493	0.00898483	0.92448295	0.98882811	Gm8126
ENSMUSG00000090641.3	21.3740429	0.01605263	0.00422018	14.1232832	0.00017121	0.00093024	Zfp712
ENSMUSG00000090655.4	3.40054913	0.00730971	0.01686138	0.32860874	0.56647946	0.74642373	Vmn2r120
ENSMUSG00000090659.10	32.4395965	-0.0028189	0.00554278	0.24273592	0.62223704	0.79218843	Zfp493
ENSMUSG00000090667.3	9.97353505	0.0524752	0.00650114	63.7012767	1.45E-15	4.40E-14	Mdfic2
ENSMUSG00000090675.3	0.80150173	0.10348184	0.02164488	23.9891408	9.69E-07	8.35E-06	Or5v1b
ENSMUSG00000090698.3	33.5674686	0.01338001	0.00494604	6.9971758	0.00816384	0.02686652	Apold1
ENSMUSG00000090733.7	5641.38269	0.00203203	0.00461618	0.19607598	0.65790702	0.81946531	Rps27
ENSMUSG00000090751.3	1.03764011	-0.0045228	0.02335351	0.00947385	0.92246137	0.98788476	Vmn2r63
ENSMUSG00000090762.3	1.25279194	-0.018403	0.02707212	0.58049397	0.44611876	0.63824721	Vmn2r56
ENSMUSG00000090774.3	2.11504226	0.01195114	0.02649002	0.41804634	0.51791349	0.70414501	Vmn2r74
ENSMUSG00000090799.3	13.619587	-0.0036506	0.01075795	0.10922634	0.74102666	0.87738276	Klhl33
ENSMUSG00000090806.4	3.02592171	0.00328959	0.01860104	0.08824539	0.76641974	0.89453563	Vmn2r70
ENSMUSG00000090812.9	2.35895001	0.00841967	0.01419275	0.42515846	0.51437423	0.70103697	Samd15
ENSMUSG00000090817.8	4.02319969	-0.0660679	0.02444125	-2.1007255	1	1	Hsd3b9
ENSMUSG00000090841.3	3672.36399	0.00050041	0.00366951	0.01850833	0.89178545	0.97112755	Myl6

ENSMUSG00000090862.4	4993.58758	-0.0050691	0.00433473	1.37057978	0.24171229	0.41177509	Rps13
ENSMUSG00000090877.4	297.169656	-0.0538396	0.00833394	35.7641099	2.23E-09	2.95E-08	Hspa1b
ENSMUSG00000090919.7	13.5589679	-0.0041854	0.00720079	0.31561246	0.5742564	0.75313676	Pabpc4l
ENSMUSG00000090935.11	1637.12762	-0.0063994	0.0025569	6.20955531	0.01270625	0.03894603	Synj2bp
ENSMUSG00000090942.2	367.273064	-0.0058278	0.00597876	0.94287439	0.33153972	0.51858185	F830016B08Rik
ENSMUSG00000090946.4	141.457264	0.03059881	0.00285128	109.674432	1.15E-25	1.19E-23	Ccdc71l
ENSMUSG00000090949.3	1.08525477	0.00785835	0.03057761	0.19014773	0.66279369	0.82289764	Vmn2r77
ENSMUSG00000090958.3	252.325071	0.01970153	0.00374838	27.3330962	1.71E-07	1.71E-06	Lrrc32
ENSMUSG00000090961.3	1.23665111	0.00048472	0.03190102	0.00515672	0.94275287	0.99850775	Vmn2r8
ENSMUSG00000090963.3	28.4297987	0.01788247	0.00623257	7.61633423	0.00578419	0.02003319	Gm17655
ENSMUSG00000090966.3	1.44902159	-0.0036884	0.03417668	0.00090735	0.97596957	1	Vmn2r116
ENSMUSG00000090996.2	40.5355658	-0.0036755	0.0249478	0.02023188	0.88689138	0.96789839	Gm20458
ENSMUSG00000091006.4	2.8539383	-0.0068669	0.02075453	0.05323329	0.81752969	0.92611878	Vmn2r69
ENSMUSG00000091076.3	2.76308406	-0.014967	0.01972115	0.50991575	0.47517533	0.66568575	Vmn2r115
ENSMUSG00000091119.3	1689.90862	0.00086196	0.00889435	0.00848851	0.9265923	0.98974711	Ccdc152
ENSMUSG00000091144.3	81.1570156	-0.0077096	0.00441034	3.10488248	0.07805786	0.17278956	Phf11c
ENSMUSG00000091151.3	2.05562722	-0.0035361	0.02775895	-0.0009813	1	1	Vmn1r224
ENSMUSG00000091183.6	7.65231878	0.02901341	0.00855715	10.4398852	0.00123323	0.00528661	Gm5141
ENSMUSG00000091228.2	644.091334	-0.0241582	0.00584092	17.4994004	2.87E-05	0.00018566	Gm20390
ENSMUSG00000091239.4	6.60806073	-0.0003521	0.01597605	-0.0065966	1	1	Vmn2r76
ENSMUSG00000091243.3	19.1051501	0.03221116	0.00900896	11.8930665	0.00056343	0.0026557	Vgll3
ENSMUSG00000091259.3	1.62730355	0.00584829	0.02285361	0.18669452	0.66568209	0.82501066	Vmn2r110
ENSMUSG00000091264.4	591.830179	-0.0033503	0.00281276	1.39158766	0.23813717	0.40737989	Smim13
ENSMUSG00000091318.3	2.24547496	-0.0089592	0.02408636	0.08393701	0.77203134	0.8979203	Semp2l1
ENSMUSG00000091337.4	195.489421	0.01484649	0.00424462	12.0616042	0.00051471	0.00245648	Eid1
ENSMUSG00000091345.11	9.17329944	0.0028727	0.00998987	0.0895364	0.76476735	0.89331432	Col6a5
ENSMUSG00000091382.3	4.2293656	-0.005327	0.02092394	0.03330091	0.85520183	0.95052597	Vmn1r18
ENSMUSG00000091387.3	130.31223	-0.0157072	0.00411556	14.4927454	0.0001407	0.00077627	Gcnt4
ENSMUSG00000091405.3	18.2875695	-0.0464082	0.01112372	16.5692012	4.69E-05	0.0002903	H4c14
ENSMUSG00000091415.6	2.40520757	-0.0147429	0.01716971	0.58224958	0.44543191	0.63769086	Ak9
ENSMUSG00000091450.4	1.12801399	0.0035033	0.03165965	0.02319332	0.87895542	0.96325074	Vmn2r11
ENSMUSG00000091455.5	5.25175399	-0.0026499	0.03123368	0.0020388	0.96398525	1	Otogl
ENSMUSG00000091468.4	0.9803258	-0.0010552	0.03784412	-0.0147358	1	1	Vmn2r82
ENSMUSG00000091471.3	2.08750131	-0.0079564	0.02277382	0.24077637	0.62364594	0.79291214	Gm20538
ENSMUSG00000091472.4	0.93365775	0.04168124	0.02633017	2.77985328	0.09545691	0.20272346	Gm3739
ENSMUSG00000091474.3	178.221862	-0.0001951	0.00377703	0.00270578	0.95851506	1	2610021A01Rik
ENSMUSG00000091476.9	5.29767427	0.02854513	0.00793492	12.8147266	0.0003439	0.00171958	Catspere2
ENSMUSG00000091491.4	3.65528609	0.04377691	0.0124558	13.5837003	0.00022816	0.00119825	Vmn2r97
ENSMUSG00000091504.4	1.41413309	0.00235462	0.04082511	0.03313258	0.85556423	0.95064484	Vmn2r118
ENSMUSG00000091512.6	352.515107	-0.0047924	0.00327969	2.13144303	0.14430485	0.27937078	Lamtor3
ENSMUSG00000091537.3	2700.02242	-0.0132303	0.0033354	15.6430568	7.65E-05	0.00044958	Tma7
ENSMUSG00000091541.3	1.57376726	0.01353004	0.01858679	0.82025553	0.36510546	0.5548854	Vmn1r9
ENSMUSG00000091572.10	7.44614955	0.08834361	0.02078833	-2.7621852	1	1	Vmn2r3
ENSMUSG00000091586.3	231.701232	-0.0214532	0.004052	26.7605772	2.30E-07	2.25E-06	Cyp4f17
ENSMUSG00000091601.6	3.89104583	0.00537915	0.0434682	0.06861295	0.79336713	0.91207587	Or2h1b
ENSMUSG00000091620.4	3.40313056	0.0039163	0.01876839	0.11558913	0.73386857	0.8725582	Vmn2r23
ENSMUSG00000091624.3	1.9160587	-0.0006217	0.02634099	-0.0040659	1	1	Vmn2r9
ENSMUSG00000091625.9	217.433058	0.00496386	0.00535798	0.84912923	0.35679879	0.54558726	Lsm5
ENSMUSG00000091648.3	15.8012434	-0.0265874	0.00684111	14.3301213	0.00015339	0.00084039	C2cd4d
ENSMUSG00000091649.4	31.0133863	0.046034	0.00594252	57.2173952	3.90E-14	9.92E-13	Phf11b
ENSMUSG00000091650.3	0.50105179	0.08275147	0.03280225	8.90205136	0.0028485	0.01092969	Apol11a
ENSMUSG00000091670.4	1.5441761	0.0034477	0.02356181	0.0701976	0.7910493	0.91082276	Vmn2r105
ENSMUSG00000091679.10	3.50319033	0.08436877	0.01420343	32.4222856	1.24E-08	1.48E-07	Vmn2r96
ENSMUSG00000091680.4	6.87182266	-0.0393167	0.01154503	10.0865187	0.00149358	0.00625157	Klhdc7b
ENSMUSG00000091685.7	2.10050324	0.0326612	0.01620276	4.11404525	0.04252852	0.10576526	Gm17359
ENSMUSG00000091694.10	7.48745407	0.10912041	0.01306338	53.2186897	2.98E-13	6.73E-12	Apol11b
ENSMUSG00000091705.9	14.3207983	0.02083358	0.00618039	11.370651	0.00074614	0.00339639	H2-Q2
ENSMUSG00000091712.3	3.2734527	0.02921379	0.01087587	6.98128688	0.00823664	0.02707963	Sec14l5
ENSMUSG00000091721.5	70.958509	0.01868969	0.00527367	12.0402294	0.00052065	0.0024813	Gimd1
ENSMUSG00000091736.4	13.0043041	0.01337015	0.01274945	1.00878904	0.31519312	0.50054841	Yy2
ENSMUSG00000091756.3	4.36094343	0.03556727	0.01473382	6.10966028	0.0134445	0.04086891	Gm3095

ENSMUSG00000091764.5	35.9205444	0.00962935	0.00347073	7.49309527	0.0061936	0.02124215	Zfp964
ENSMUSG00000091780.4	675.432539	-0.0245382	0.00421726	33.3780096	7.59E-09	9.35E-08	Sco2
ENSMUSG00000091803.8	1086.96963	-0.0092958	0.00385143	5.81931924	0.01585108	0.0469554	Cox16
ENSMUSG00000091805.3	1.16123086	-0.0014201	0.02489306	0.00409373	0.94898432	1	Vmn2r108
ENSMUSG00000091811.3	73.1133726	0.00567672	0.00348859	2.63244359	0.10470046	0.21745907	Inafm1
ENSMUSG00000091813.5	14.3956207	0.01912374	0.00753511	6.36446275	0.01164283	0.03617069	Ces2h
ENSMUSG00000091844.3	10.7795117	-0.023655	0.00918953	6.2891291	0.01214807	0.03752285	Ccdc168
ENSMUSG00000091859.3	1.86556459	0.01260896	0.01810502	0.67919688	0.40986341	0.60159552	Vmn2r100
ENSMUSG00000091867.3	399.392311	0.0851837	0.00941246	80.7611197	2.55E-19	1.22E-17	Cyp2a22
ENSMUSG00000091873.3	1.32966542	-0.0031455	0.03392912	-0.0712478	1	1	Or4n4
ENSMUSG00000091879.3	1.93750502	0.01469777	0.02112886	0.77093304	0.37992856	0.57147239	Vmn2r17
ENSMUSG00000091896.10	1509.19195	-0.0026277	0.00218135	1.44361457	0.22955532	0.39668047	Ube2d2a
ENSMUSG00000091898.9	1216.52066	-0.038701	0.01078363	9.71499005	0.00182771	0.00746265	Tnnc1
ENSMUSG00000091924.3	2.37525357	0.00724556	0.0254567	0.15097384	0.69760646	0.84829658	Vmn1r185
ENSMUSG00000091930.3	1.91251426	0.03023229	0.01847891	3.71392146	0.05396048	0.12843998	Vmn2r52
ENSMUSG00000091931.6	83.6629703	-0.0119819	0.002906	16.9577469	3.82E-05	0.00024091	Gon7
ENSMUSG00000091945.3	2.08857078	0.01256444	0.01631311	0.9225221	0.33681362	0.5241952	Vmn2r114
ENSMUSG00000091955.3	4.28844341	0.04686997	0.01378582	11.1958147	0.00081982	0.00368732	Tmsb10b
ENSMUSG00000091962.4	0.98346806	0.00111158	0.03563278	0.0092927	0.92320397	0.98818647	Vmn2r78
ENSMUSG00000091971.4	32.6708988	-0.0566858	0.00984707	30.7250158	2.97E-08	3.35E-07	Hspa1a
ENSMUSG00000091983.4	1.45112546	0.00518914	0.02717059	0.11258041	0.73722528	0.87469707	Or2r3
ENSMUSG00000091996.9	4.63738832	-0.0002938	0.01017691	0.00071744	0.97863119	1	Smim35
ENSMUSG00000092008.3	2451.8123	-0.0048242	0.01202015	0.11755221	0.73170463	0.87130397	Cyp2c69
ENSMUSG00000092021.10	188.507876	0.00074914	0.00781304	0.00862813	0.92599277	0.98947002	Gbp11
ENSMUSG00000092032.3	2.95296414	0.02390028	0.01781967	2.72954508	0.09850734	0.20774268	Vmn2r59
ENSMUSG00000092035.10	48.4844861	0.01217607	0.00668604	2.74004586	0.09786196	0.20669581	Peg10
ENSMUSG00000092049.11	1.8289248	0.00956475	0.02235609	0.31739789	0.57317563	0.75222126	Vmn2r4
ENSMUSG00000092060.5	26.9931196	0.00842653	0.00534216	2.440458	0.1182417	0.23944781	Bend4
ENSMUSG00000092074.3	32.3671143	0.03032685	0.00739445	15.5000362	8.25E-05	0.00048071	Dynlt1a
ENSMUSG00000092080.4	1.57642197	0.0005116	0.03499932	0.00186448	0.96555834	1	Vmn2r16
ENSMUSG00000092083.5	4.80301906	0.0061993	0.0177018	0.13891871	0.70935833	0.85624353	Kcnb2
ENSMUSG00000092094.6	1.48869963	-0.0019828	0.01420774	0.00930729	0.9231439	0.98816701	Zfp804b
ENSMUSG00000092111.3	5.25663647	0.00585516	0.01874335	0.23738973	0.62609778	0.79500093	Vmn2r113
ENSMUSG00000092118.2	54.6116359	0.00575337	0.00237022	5.90282094	0.01511665	0.04515767	Fancf
ENSMUSG00000092162.4	1.75652783	-0.0093066	0.01747934	0.31002135	0.57766708	0.75601242	Vmn2r86
ENSMUSG00000092192.8	3.97871252	0.00154561	0.00954959	0.02686971	0.86979425	0.9584194	Dnaaf4
ENSMUSG00000092212.5	3.5467043	-0.0220098	0.0088108	6.29259453	0.01212434	0.03746427	Slc22a13b
ENSMUSG00000092232.2	1.08314338	0.04747866	0.03089166	2.05939838	0.15126988	0.2892006	Gm20521
ENSMUSG00000092260.8	81.7882283	-0.0055263	0.00255017	4.67367982	0.03062823	0.08090464	Zfp963
ENSMUSG00000092277.2	2.897892	0.04262746	0.00887208	22.9926869	1.63E-06	1.36E-05	Gm19684
ENSMUSG00000092300.8	1.9443636	-0.0498627	0.01137782	19.2015601	1.18E-05	8.22E-05	Cdk3
ENSMUSG00000092305.2	1.44935007	-0.0055453	0.02452626	0.01336578	0.90796128	0.98000648	Prps1l1
ENSMUSG00000092310.4	0.65525393	0.0102095	0.02973318	0.16757667	0.68227454	0.83734655	Gm20509
ENSMUSG00000092335.3	26.5303669	-0.0118809	0.00484354	5.8420812	0.01564731	0.04645153	Zfp977
ENSMUSG00000092349.2	1.28293272	-0.0090838	0.01234045	0.48686145	0.48533069	0.67418633	Smim40
ENSMUSG00000092360.2	0.9967033	0.05233796	0.0265544	3.96319944	0.04650523	0.11372446	Gm20441
ENSMUSG00000092367.2	1.27955363	-0.0039183	0.03963222	0.00569449	0.93984726	0.99681795	Gm20479
ENSMUSG00000092416.3	192.215652	-0.0204315	0.00324914	38.5758524	5.27E-10	7.59E-09	Zfp141
ENSMUSG00000092417.4	157.69466	-0.0068832	0.00391068	3.13596477	0.07658306	0.17025998	Gpank1
ENSMUSG00000092470.2	5.24842098	-0.0108686	0.01020335	1.11867408	0.29020414	0.47169422	Gm20518
ENSMUSG00000092511.8	109.853302	-0.0205721	0.02626165	-0.0575168	1	1	Gm20547
ENSMUSG00000092517.3	3.46420291	0.07154635	0.0127117	28.4985578	9.38E-08	9.80E-07	Art2a
ENSMUSG00000092541.8	2.15000029	-0.0451985	0.02696556	3.66709994	0.05549667	0.13149763	Gm20537
ENSMUSG00000092544.9	1.75330169	0.01230128	0.02857695	0.16234328	0.68700821	0.84070643	Gm20422
ENSMUSG00000092558.4	305.869826	-0.0078043	0.00188816	17.2232477	3.32E-05	0.00021149	Med20
ENSMUSG00000092572.9	2.65626524	0.0870894	0.01231926	41.3067196	1.30E-10	2.06E-09	Serpinb10
ENSMUSG00000092592.2	2.06127912	-0.0037585	0.01191788	0.09029844	0.7637981	0.89280137	Gm20449
ENSMUSG00000092607.10	171.027657	-0.0018158	0.00324636	0.31156811	0.57671952	0.75519135	Scnm1
ENSMUSG00000092618.3	0.58161462	0.05624867	0.0344399	3.08873354	0.07883614	0.17412184	Btnl6
ENSMUSG00000093445.8	199.928554	0.01031576	0.00518945	3.71121794	0.05404794	0.12863519	Lrch4
ENSMUSG00000093456.8	3.44324974	0.04086647	0.01791624	4.5485009	0.03294758	0.08587707	Gm20662

ENSMUSG00000093485.2	207.43248	-0.0261882	0.01447829	-0.1753872	1	1	Gm20708
ENSMUSG00000093536.8	1.3717759	0.00228762	0.02181879	-0.0392626	1	1	Smim17
ENSMUSG00000093575.2	25.5709211	-0.0167595	0.00644564	6.51975961	0.01066824	0.03372034	Gm20695
ENSMUSG00000093593.2	43.4116681	-0.0101245	0.00686382	2.14075928	0.14343089	0.27797542	Gm20683
ENSMUSG00000093661.2	201.878315	0.03540476	0.00502425	47.1846608	6.46E-12	1.23E-10	Eif4e3
ENSMUSG00000093668.4	3.14141269	-0.0047965	0.01272373	0.11277806	0.73700325	0.87459669	Pou5f2
ENSMUSG00000093674.8	10634.2923	-0.0057644	0.0050118	1.3363488	0.24767889	0.41893373	Rpl41
ENSMUSG00000093701.9	27.2777078	0.05958182	0.03634224	0.30467909	0.58096381	0.75881119	Gm21970
ENSMUSG00000093752.2	846.928282	-0.0007889	0.00230213	0.11440609	0.73518257	0.87346444	Gm20716
ENSMUSG00000093769.5	4.64311046	0.03579748	0.08895805	-1.819286	1	1	H3c14
ENSMUSG00000093789.4	3.861819	0.01938968	0.01180063	2.6894064	0.10101744	0.21178643	Methig1
ENSMUSG00000093803.6	113.851779	-0.0069237	0.0038859	3.1471995	0.07605739	0.16926681	Ppp2r3d
ENSMUSG00000093805.8	1.26375609	0.0079084	0.01612824	0.30632818	0.57994215	0.75789105	Gal3st2b
ENSMUSG00000093904.3	2663.04516	-0.0022432	0.00357204	0.39340692	0.53051399	0.7150116	Tomm20
ENSMUSG00000093930.3	9243.44166	0.02171361	0.00536301	15.0972887	0.00010211	0.00058257	Hmgcs1
ENSMUSG00000093931.3	5.68347316	-0.1421646	0.12216242	-0.0106291	NA	NA	Amy2a3
ENSMUSG00000093938.3	69.1679795	0.063491	0.00634945	90.733483	1.64E-21	1.02E-19	Evi2b
ENSMUSG00000093942.4	3.29887447	0.00152728	0.01602012	0.04788103	0.82679233	0.93197878	Or13a18
ENSMUSG00000093954.9	88.5065357	0.01746401	0.00781679	4.70798377	0.03002284	0.07949361	Gm16867
ENSMUSG00000093979.9	1.38661248	0.06647109	0.01546326	16.4452904	5.01E-05	0.00030693	Gm2237
ENSMUSG00000093989.2	812.581359	-0.00452	0.00320652	2.02312793	0.15491973	0.29443488	Rnasek
ENSMUSG00000093996.3	0.55203391	0.042641	0.05108903	1.00806545	0.31536674	0.50079041	Spata31f1c
ENSMUSG00000094021.9	0.99541606	-0.0115239	0.04218555	0.1024766	0.74887762	0.8825336	Gm3685
ENSMUSG00000094027.3	1.7892565	0.00430446	0.01745338	0.08013404	0.77711585	0.90149234	Gm21762
ENSMUSG00000094065.2	3.64720438	-0.0704161	0.03688862	3.50560653	0.06116145	0.1421503	Ccl21d
ENSMUSG00000094066.2	2.39846695	-0.0026908	0.01278324	0.03733029	0.84679413	0.94504052	Spata31f1b
ENSMUSG00000094076.8	18.4454243	0.02918717	0.0259416	1.13819359	0.28603436	0.46658872	Gm4767
ENSMUSG00000094091.4	6.15797481	0.01178409	0.00704389	2.61527745	0.10583901	0.21924829	Ankrd40cl
ENSMUSG00000094103.3	27.4245	-0.0314235	0.04088261	-0.6085238	1	1	Fam177a2
ENSMUSG00000094132.9	1.08792108	0.00909331	0.03654406	0.07883602	0.77888105	0.90278089	Gm3264
ENSMUSG00000094145.3	22.4516149	-0.0171334	0.00922415	3.12202767	0.07724061	0.17133425	Vmn2r20
ENSMUSG00000094152.6	17.0828887	-0.015199	0.00841224	3.10039646	0.07827323	0.17320128	Slc6a16
ENSMUSG00000094156.3	129.32092	0.10356762	0.00969956	95.7280906	1.32E-22	9.31E-21	Sult2a7
ENSMUSG00000094248.2	2.08880807	-0.0076607	0.03768242	0.18730325	0.66517064	0.82467887	H2ac23
ENSMUSG00000094295.4	1.2489076	-0.011076	0.04308197	0.0737842	0.78590463	0.90757112	Or10u4
ENSMUSG00000094338.2	2.47002432	0.04977757	0.03497278	-1.0931621	1	1	H2bc13
ENSMUSG00000094379.3	1.30797975	-0.0039851	0.02663852	-0.0362997	1	1	Vmn1r202
ENSMUSG00000094380.4	1.0374487	0.01070455	0.03182694	0.25411554	0.61419199	0.78581865	Or8b1b
ENSMUSG00000094396.4	3.46976817	0.00948106	0.01751882	0.60367901	0.43717775	0.62881293	Vmn2r124
ENSMUSG00000094410.9	805.012552	-0.0045931	0.00646074	0.50389194	0.47779501	0.66784836	Zbed6
ENSMUSG00000094439.2	4.53766863	0.00091921	0.00859993	0.01710368	0.89594851	0.97341387	Gm21969
ENSMUSG00000094441.3	186.644117	-0.0050216	0.00288321	2.99974067	0.08327785	0.18181906	Zfp955a
ENSMUSG00000094443.4	0.90895256	-0.0027439	0.04672835	-0.0133798	1	1	Sgo2b
ENSMUSG00000094475.9	23.16053	-0.0024219	0.02232555	0.00755404	0.9307399	0.99206576	Gm11007
ENSMUSG00000094483.3	907.104395	0.00366182	0.002902	1.55754154	0.21202555	0.37437001	Purb
ENSMUSG00000094520.5	2.22307155	0.002159	0.01747895	0.05598165	0.81296369	0.92330035	Or51q1
ENSMUSG00000094531.4	1.06753067	0.00027674	0.02705157	0.00185258	0.9656684	1	Or52e8
ENSMUSG00000094539.4	0.81449806	0.00259799	0.04744405	0.01586878	0.89975461	0.9756166	Or5h23
ENSMUSG00000094559.3	1.39374704	-0.0226064	0.01278022	3.29229679	0.06960559	0.1574789	Cyp2d34
ENSMUSG00000094595.2	10.3689545	0.01176758	0.0077523	2.2325009	0.13513496	0.26551926	Fsbp
ENSMUSG00000094622.4	47.2312475	0.00200479	0.00814329	0.05556174	0.81365353	0.92370197	Gm3055
ENSMUSG00000094632.5	2.01225494	0.01372832	0.0274288	0.40422825	0.52491365	0.71030484	Gm10310
ENSMUSG00000094638.2	16.6608	0.00745577	0.01137803	0.3892841	0.53267595	0.71722869	Gm21972
ENSMUSG00000094651.3	2.58887545	0.07450089	0.01823102	19.3912536	1.06E-05	7.51E-05	Gal3st2
ENSMUSG00000094672.3	1.7938964	0.01454041	0.02106946	0.66860593	0.41353807	0.6049562	Vmn2r25
ENSMUSG00000094686.2	26.2982758	-0.0568708	0.00815664	47.6958564	4.98E-12	9.55E-11	Ccl21a
ENSMUSG00000094724.9	1781.06734	3.29E-05	0.00288915	0.00013213	0.99082881	1	Rnaset2b
ENSMUSG00000094747.4	3.59121555	0.03245092	0.01554249	5.45261431	0.01953907	0.0558063	Or4f14b
ENSMUSG00000094777.3	23.2027063	0.01842363	0.01621405	1.73063875	0.18832934	0.34208209	H2ac24
ENSMUSG00000094786.3	347.466802	-0.0085074	0.00498893	2.94541897	0.08612136	0.1868855	Gm14403
ENSMUSG00000094793.2	3389.00472	-0.0748459	0.01210966	26.6483845	2.44E-07	2.36E-06	Mup12

ENSMUSG00000094796.3	8.68635139	0.07378061	0.00995838	50.8660555	9.89E-13	2.07E-11	BC147527
ENSMUSG00000094806.3	11785.8619	-0.0175753	0.00498806	12.2180682	0.00047329	0.00227909	Cyp2d10
ENSMUSG00000094814.2	12.1301392	0.0085665	0.015539	0.38308497	0.53595682	0.72027161	Gm21973
ENSMUSG00000094822.3	1.2974931	0.011956	0.02943524	0.5026039	0.47835822	0.66832178	Or52a20
ENSMUSG00000094858.4	1.48295911	0.00388533	0.03832715	0.04084633	0.83983484	0.94036567	Or4k47
ENSMUSG00000094870.9	271.023133	-0.004735	0.00280344	2.83306601	0.09234191	0.19745932	Zfp131
ENSMUSG00000094878.3	0.85878863	0.00611345	0.03786737	0.08575509	0.76964468	0.89652506	Or2g7
ENSMUSG00000094887.2	2.78845734	0.07536129	0.02604932	7.11614299	0.00763929	0.02540142	ENSMUSG00000094887
ENSMUSG00000094892.3	2.08757467	0.00925103	0.02674365	0.20056939	0.65426164	0.81600207	Vmn2r101
ENSMUSG00000094898.3	1.17730849	-0.0105926	0.02613239	0.15352352	0.69519064	0.84646519	Vmn1r201
ENSMUSG00000094910.2	31.1035033	0.01387335	0.00604402	5.08853523	0.02408453	0.06635412	D430019H16Rik
ENSMUSG00000094915.2	4.89058749	0.15501047	0.016625	68.8959117	1.04E-16	3.60E-15	ENSMUSG00000094915
ENSMUSG00000094921.3	1.86502636	-0.0067113	0.02038132	0.0719633	0.78849938	0.90928207	Vmn2r112
ENSMUSG00000094932.9	94.9987035	-0.0094112	0.01797796	0.25611214	0.61280384	0.78483058	Gm2007
ENSMUSG00000094936.9	326.349877	-0.0162295	0.00261089	38.3298828	5.97E-10	8.56E-09	Rbm4
ENSMUSG00000094942.8	6.25197903	0.00456851	0.00769252	0.34480385	0.55706939	0.73902011	Gm3604
ENSMUSG00000094950.3	2.60001416	0.01157528	0.02140976	0.5037178	0.47787109	0.66791511	Vmn2r66
ENSMUSG00000094970.4	2.48101978	0.0071757	0.02220864	0.30983892	0.57777905	0.7560331	Or8g26
ENSMUSG00000094986.4	0.90147536	5.80E-05	0.03722808	0.00043121	0.98343257	1	Or5b102
ENSMUSG00000095002.4	1.4048709	-0.0086559	0.03186285	0.03858684	0.84426944	0.94351541	Or6c66
ENSMUSG00000095026.3	6.1743498	0.00412908	0.00778965	0.2739192	0.60071477	0.77461367	Gm3336
ENSMUSG00000095028.7	17.2653612	0.08193442	0.00961993	63.9470846	1.28E-15	3.90E-14	Sirpb1b
ENSMUSG00000095041.8	2673.67652	0.01711198	0.00472725	12.4848191	0.00041027	0.00200803	ENSMUSG00000095041
ENSMUSG00000095061.2	11.5096471	-0.0213725	0.01239533	2.67291427	0.10206897	0.21353546	E030018B13Rik
ENSMUSG00000095071.3	0.87335108	0.11847377	0.04143571	8.12158268	0.00437414	0.01579914	AF067061
ENSMUSG00000095098.3	184.080425	-0.0007193	0.00477746	0.02294378	0.87960336	0.9636572	Ccdc85b
ENSMUSG00000095105.3	5.07352511	0.02660775	0.0072349	12.9112375	0.00032662	0.00164045	Edaradd
ENSMUSG00000095115.3	188.471586	0.02906209	0.00447622	39.6176524	3.09E-10	4.59E-09	Itpripl2
ENSMUSG00000095125.3	1.4635577	0.0120848	0.02820692	0.48965293	0.48408216	0.67321939	Vmn1r198
ENSMUSG00000095139.3	5.87595736	0.01268853	0.02163121	0.60694905	0.43593886	0.6274136	Pou3f2
ENSMUSG00000095143.6	5.21441581	-0.0634084	0.02415721	3.02476622	0.08200214	0.17961575	Hsd3b4
ENSMUSG00000095180.11	7.20274716	0.04620421	0.00969302	21.0476787	4.48E-06	3.42E-05	Rhox5
ENSMUSG00000095190.4	0.55914088	0.00602436	0.1526615	0.00766934	0.93021467	0.99177898	Gm10668
ENSMUSG00000095199.9	180.607222	-0.0185909	0.01780584	0.93823949	0.33273101	0.51969244	Zfp967
ENSMUSG00000095217.2	2.54707263	-0.0082771	0.02999745	0.11322232	0.73650497	0.87424289	H2bc15
ENSMUSG00000095248.4	1.35043761	0.00371063	0.03664384	0.01516436	0.90199333	0.97674187	Or56a3b
ENSMUSG00000095253.5	128.25836	0.006882	0.00419499	2.67204626	0.10212464	0.21358414	Zfp799
ENSMUSG00000095294.3	1.63104746	0.03556831	0.02547268	2.45598723	0.11707754	0.23760031	Potefam3b
ENSMUSG00000095304.10	318.330142	0.00685793	0.00727245	0.88571375	0.34664149	0.53507109	Plac9
ENSMUSG00000095315.4	37.4816827	0.00313114	0.00376901	0.67798275	0.41028222	0.60188609	Zfp1001
ENSMUSG00000095325.4	148.765948	-0.0041007	0.00381762	1.14578679	0.28443286	0.46445815	Zfp870
ENSMUSG00000095362.8	585.33746	-0.0140855	0.00522062	7.2281696	0.00717683	0.02411981	Gm14325
ENSMUSG00000095384.2	1.09277896	-0.0192873	0.2095006	0.01625014	0.89856365	0.97504154	1700001F09Rik
ENSMUSG00000095388.9	7.69013518	-0.0416963	0.02856087	-0.0768872	1	1	Hsd3b8
ENSMUSG00000095407.3	1.88943199	0.01882733	0.02070025	0.7811843	0.37677918	0.56784114	Tmem200c
ENSMUSG00000095432.10	144.10994	0.00051581	0.0033838	0.02264906	0.88037327	0.96408053	Zfp748
ENSMUSG00000095440.3	129.207342	-0.0005461	0.00346178	0.02422981	0.8763016	0.96183824	Fignl2
ENSMUSG00000095448.4	2.01921675	0.00971303	0.02522	0.31406934	0.57519374	0.75386306	Or7e168
ENSMUSG00000095450.2	0.20910382	0.06023979	0.11916826	0.46950427	0.49321532	0.68072892	ENSMUSG00000095450
ENSMUSG00000095463.9	494.474168	-0.0055017	0.00363149	2.2841826	0.13069898	0.25891703	Entpd4
ENSMUSG00000095486.3	1.2948475	0.00960562	0.02658265	0.4154377	0.51922237	0.70523344	Vmn2r22
ENSMUSG00000095538.2	4.00607656	-0.0085076	0.02227917	0.13567913	0.7126149	0.85832576	Gm21983
ENSMUSG00000095545.9	233.819526	0.00639935	0.01635851	-0.932461	1	1	Zfp969
ENSMUSG00000095567.9	543.10181	-0.0029009	0.00243429	1.41175553	0.23476518	0.4032019	Noc2l
ENSMUSG00000095595.3	85.1470833	0.04193394	0.01370213	9.19748368	0.00242348	0.00951718	Fam177a
ENSMUSG00000095609.4	19.9737785	0.14774209	0.01313078	101.057311	8.94E-24	7.17E-22	Gm21188
ENSMUSG00000095629.3	0.98552152	-0.0014401	0.0400849	-0.0119931	1	1	Vmn1r89
ENSMUSG00000095648.9	48.6027725	-0.0014633	0.01348604	0.01193786	0.91299587	0.98246232	Gm2004
ENSMUSG00000095658.3	2.56841825	0.00661731	0.02543475	0.15551638	0.69331848	0.84505244	Vmn2r130
ENSMUSG00000095664.3	1.70937059	-0.0040528	0.02439171	-0.0062775	1	1	Vmn2r67
ENSMUSG00000095675.3	5.96421767	-0.0422679	0.03353315	0.89818981	0.34326755	0.53132357	Ccl21b

ENSMUSG00000095677.3	106.734767	0.01455495	0.00659436	4.58966472	0.03216531	0.08423839	Dynlt1f
ENSMUSG00000095687.3	568.153026	0.00137469	0.00362427	0.14047972	0.70780457	0.85532732	Rnaset2a
ENSMUSG00000095730.4	10.2813347	0.0244403	0.01235072	3.48019653	0.06210736	0.1437833	Vmn2r29
ENSMUSG00000095788.7	12.7160357	0.11065407	0.0098705	111.304371	5.07E-26	5.44E-24	Sirpb1a
ENSMUSG00000095789.7	13.2845248	0.01105639	0.00880638	1.46524771	0.226097	0.3922274	Nupr1l
ENSMUSG00000095864.3	1.87279605	0.00225258	0.02986494	0.05036402	0.82243105	0.92926094	Vmn1r77
ENSMUSG00000095903.4	1.13871936	-0.0051451	0.03511219	-0.0083031	1	1	Or8g53
ENSMUSG00000095909.4	1.24105107	-0.0010679	0.03631804	0.00301287	0.95622638	1	Zfp997
ENSMUSG00000095916.3	1.14233968	0.01311159	0.03410662	0.29156414	0.58921993	0.76577812	Vmn1r191
ENSMUSG00000095930.2	52.5177733	-0.0056593	0.00390672	2.13018553	0.14442327	0.27950758	Nim1k
ENSMUSG00000095932.4	1.62579057	-0.0060518	0.03178105	0.07363037	0.7861225	0.90769497	Vmn1r49
ENSMUSG00000095936.5	1.41069319	0.01635356	0.01665069	1.24690336	0.26414474	0.43917369	Zscan4e
ENSMUSG00000095954.6	1.20639763	-0.0030791	0.03403708	-0.0076841	1	1	Pramel56
ENSMUSG00000095961.3	4.5447346	-0.0051064	0.01846306	0.07299172	0.78702963	0.90820886	Vmn2r102
ENSMUSG00000095975.4	48.3993703	-0.0049488	0.00563948	0.75031897	0.38637526	0.57782275	Cphx1
ENSMUSG00000095990.2	156.657629	0.00296692	0.00412003	0.51677955	0.47221868	0.66295542	Zfp97
ENSMUSG00000095996.6	1.61956167	0.01428095	0.06483019	0.11124478	0.7387314	0.87592122	Pramel60
ENSMUSG00000096002.3	5.04449277	0.01375021	0.01371885	1.12346295	0.28917401	0.47037643	Vmn2r53
ENSMUSG00000096010.3	9.39234189	-0.0050512	0.01330629	0.13029211	0.71812936	0.86190861	H4c16
ENSMUSG00000096014.2	2.36570021	0.02042889	0.03461378	0.95292005	0.32897714	0.51591659	Sox1
ENSMUSG00000096029.5	1.3884065	-0.0130319	0.04261746	-0.0098838	1	1	Or56a3
ENSMUSG00000096039.9	2.39573954	0.00399032	0.01105948	0.12050023	0.72849258	0.86884233	D830030K20Rik
ENSMUSG00000096045.8	0.70473136	0.00968767	0.08149007	0.1582723	0.69075218	0.8431396	Gm21698
ENSMUSG00000096054.4	600.574842	-4.72E-05	0.0052894	7.93E-05	0.99289677	1	Syne1
ENSMUSG00000096099.3	2.05295261	0.00253019	0.0287494	0.04567252	0.83077219	0.93462054	Vmn1r220
ENSMUSG00000096100.2	1.23426052	0.08644202	0.07802431	1.5438921	0.2140394	0.3769382	ENSMUSG00000096100
ENSMUSG00000096140.3	1.33008798	0.06721393	0.0191577	13.0830342	0.00029798	0.00151568	Ankrd66
ENSMUSG00000096145.3	2426.64188	-0.00145	0.00351988	0.16990031	0.68020042	0.8355837	Vkorc1
ENSMUSG00000096146.3	8.05205083	0.02893528	0.0227782	-30.351046	1	1	Kcnj11
ENSMUSG00000096167.5	1.99503091	-0.0093283	0.02696481	0.04501786	0.8319712	0.93532402	Or8b39
ENSMUSG00000096188.2	878.450984	-0.0036287	0.00288293	1.52576433	0.21674926	0.38054548	Cmtm4
ENSMUSG00000096199.3	99.056089	0.00621691	0.00290658	4.55926948	0.03274104	0.08544258	Ptrhd1
ENSMUSG00000096210.3	2138.35531	-0.0006361	0.00200156	0.10101737	0.75061215	0.88395936	H1f0
ENSMUSG00000096215.9	62.3168127	0.00468884	0.00670366	0.47765821	0.48948497	0.67737107	Smim22
ENSMUSG00000096225.8	1.19848506	0.10965853	0.04677311	7.72267776	0.00545315	0.01904886	Lhx8
ENSMUSG00000096255.3	290.282	0.00092241	0.00468119	0.03834584	0.84475032	0.94387612	Dynlt1b
ENSMUSG00000096265.3	2.19818022	0.00481714	0.02593842	0.08906085	0.76537451	0.89366946	Potefam3e
ENSMUSG00000096351.3	1.84579078	0.03875024	0.01912079	3.62554029	0.05689909	0.13409493	Samd11
ENSMUSG00000096370.9	106.804063	-0.0157417	0.00797816	4.16683482	0.04122274	0.10314869	Gm21992
ENSMUSG00000096373.4	1.2582154	0.01305967	0.02433575	0.45251856	0.50114135	0.68785612	Vmn2r31
ENSMUSG00000096409.4	1.62797101	-0.0077606	0.03542689	0.08044178	0.77669961	0.9012268	Or8b41
ENSMUSG00000096433.3	111.564974	0.01068106	0.00293284	12.9207378	0.00032496	0.00163319	Zfp994
ENSMUSG00000096458.8	27.3122465	-0.0028794	0.00721824	0.15851324	0.69052905	0.8431396	Moap1
ENSMUSG00000096472.3	56.8670036	0.01073584	0.00456202	5.45834049	0.01947513	0.0556574	Cdkn2d
ENSMUSG00000096497.3	2.72860201	0.0080252	0.02699831	0.29915907	0.58441009	0.76163918	Or6c5c
ENSMUSG00000096546.3	491.501	-0.0013007	0.0039549	0.10952108	0.74069004	0.87726802	Smlr1
ENSMUSG00000096549.10	6.61563202	-0.0124493	0.00715178	3.13007763	0.07686008	0.17074709	Prickle4
ENSMUSG00000096569.3	11.367051	-0.2573038	0.09873196	1.41841627	0.23366425	0.40177919	Amy2a2
ENSMUSG00000096584.3	0.79775145	-0.0006621	0.06052287	-0.0035391	1	1	Or51aa5
ENSMUSG00000096596.3	4.05106455	-0.0357307	0.03629422	1.11790372	0.29037029	0.47182547	Gm10591
ENSMUSG00000096606.3	169.058738	-0.0026266	0.00393202	0.44641376	0.50404294	0.69102283	Tpbgl
ENSMUSG00000096674.2	30979.2901	-0.1812947	0.01677592	75.3677703	3.91E-18	1.60E-16	Mup15
ENSMUSG00000096687.9	90.8225409	0.01170037	0.00301049	15.0525974	0.00010456	0.00059524	Mfsd4b4
ENSMUSG00000096688.2	17052.4022	-0.1906689	0.0131094	132.590062	1.11E-30	2.57E-28	Mup17
ENSMUSG00000096695.4	1.77734586	0.00325581	0.04737625	0.02722352	0.86894752	0.95812872	Or5h26
ENSMUSG00000096696.2	125.014087	0.00549424	0.0037589	2.16036235	0.14161129	0.27519518	Zfp960
ENSMUSG00000096714.3	1.15450488	0.01905957	0.02747161	1.281296	0.2576582	0.43120326	Or2ag17
ENSMUSG00000096718.3	21.5283331	0.02099081	0.01237356	2.61593408	0.10579521	0.21922742	Zfp781
ENSMUSG00000096719.2	1.17009184	0.09270564	0.01950407	22.3100507	2.32E-06	1.87E-05	Mrgpra2b
ENSMUSG00000096727.4	386.019839	0.03151249	0.00519912	36.2255019	1.76E-09	2.36E-08	Psmb9
ENSMUSG00000096735.4	2.10481511	0.00768185	0.0256175	0.1744805	0.67616016	0.83267936	Vmn1r79

ENSMUSG00000096740.9	175.459978	-0.0159952	0.00773597	3.92719746	0.04751106	0.11574012	Lbhd1
ENSMUSG00000096757.3	1.59414068	0.00710976	0.03117061	0.16584623	0.68383009	0.83826645	Or8c11
ENSMUSG00000096764.2	5.10705406	-0.0618302	0.03781642	2.28007317	0.13104569	0.25945133	Gm21985
ENSMUSG00000096794.3	1.25193401	-0.0011803	0.02399267	-0.0129762	1	1	Or8b1c
ENSMUSG00000096795.5	94.6436384	0.00623911	0.00521914	1.39082252	0.23826625	0.4075234	Zfp433
ENSMUSG00000096847.3	6.32461908	0.07676562	0.01743426	19.5659456	9.72E-06	6.90E-05	Tmem151b
ENSMUSG00000096852.3	12.7741144	0.03497315	0.00812918	17.3849263	3.05E-05	0.00019563	Cyp2d12
ENSMUSG00000096856.8	72.1144008	-0.0073747	0.00823797	0.79398597	0.37289757	0.56364792	Gm10778
ENSMUSG00000096861.3	1.07837877	0.00050949	0.03499379	0.0020873	0.96355973	1	Vmn2r68
ENSMUSG00000096883.3	1.48563437	0.0568455	0.0160983	13.1639229	0.00028539	0.00146077	Shisa8
ENSMUSG00000096887.3	23.6441808	-0.0175136	0.01880969	1.05959729	0.30330705	0.4880097	Gm20594
ENSMUSG00000096910.3	228.250528	-0.0031954	0.00405931	0.60770802	0.43565209	0.62715394	Zfp955b
ENSMUSG00000096914.5	2.3643017	0.01812341	0.02219283	1.4588954	0.22710596	0.39339902	Galntl6
ENSMUSG00000096916.8	11.4378738	0.02914637	0.00738829	14.3612285	0.00015088	0.00082777	Zfp850
ENSMUSG00000096968.2	5.72470943	0.0240274	0.00661952	13.0005517	0.0003114	0.00157577	Gm26620
ENSMUSG00000097075.4	4.98128474	0.01175827	0.00991075	1.36678558	0.24236493	0.41258891	Cdiptos
ENSMUSG00000097148.3	162.061928	0.01360953	0.00562801	5.77438962	0.01626137	0.04794257	Gapdhrt
ENSMUSG00000097221.2	10.2591968	-0.0128102	0.01068872	1.35786626	0.24390762	0.41443737	1810049J17Rik
ENSMUSG00000097239.8	36.4298148	-0.0092374	0.00950846	0.91199666	0.3395852	0.52725012	Gm27029
ENSMUSG00000097328.8	156.068641	0.00941144	0.00331999	8.19043286	0.00421119	0.01528078	Tnfsf12
ENSMUSG00000097333.8	187.707859	-0.0044655	0.00478281	0.86141382	0.35334335	0.54197543	Zfp87
ENSMUSG00000097392.5	713.054943	-0.0087355	0.00437351	3.90768236	0.04806584	0.11683818	Thoc2l
ENSMUSG00000097487.8	30.9264037	-0.0160172	0.00876228	3.1590567	0.0755068	0.1682364	Ptges3l
ENSMUSG00000097565.2	0.54643928	-0.0305026	0.11315401	0.12342889	0.72534493	0.86733236	Gm26965
ENSMUSG00000097638.2	1.09771612	0.03393745	0.03690295	1.51719414	0.2180446	0.38230746	Carlr
ENSMUSG00000097789.3	10.0534121	0.00337699	0.01516469	0.05425923	0.81581102	0.92512711	Ctxnd1
ENSMUSG00000097993.9	5.40570968	0.08083097	0.01422719	23.8378667	1.05E-06	8.98E-06	Ptprv
ENSMUSG00000098022.8	7.20509563	0.01811545	0.00900112	3.88842094	0.04862012	0.11794241	Zfp82
ENSMUSG00000098112.9	91.6030795	0.0509536	0.00592735	68.5758623	1.22E-16	4.17E-15	Bin2
ENSMUSG00000098132.2	5.82493838	0.01108931	0.00842651	1.75272178	0.18553493	0.33823055	Rassf10
ENSMUSG00000098134.2	143.217203	-0.0077971	0.00216347	12.7573007	0.00035462	0.00176645	Rnf113a2
ENSMUSG00000098176.3	54.2722052	0.00875813	0.00303757	8.24905163	0.00407733	0.01486137	Ccdc166
ENSMUSG00000098188.2	194.779894	0.01963793	0.00362892	27.4460359	1.62E-07	1.62E-06	Sowahc
ENSMUSG00000098274.8	4240.22056	-0.0011069	0.00397833	0.07828703	0.77963233	0.90327065	Rpl24
ENSMUSG00000098306.10	68.8557097	-0.0226328	0.01119874	3.89322007	0.04848139	0.1176785	Gm28040
ENSMUSG00000098332.3	155.405963	-0.0030885	0.00389341	0.64227808	0.42288707	0.61462993	Pigbos1
ENSMUSG00000098374.2	87.1448474	-0.0005009	0.00568704	0.00775457	0.92982898	0.99172227	Gm28043
ENSMUSG00000098387.3	9.43941318	-0.003993	0.03661245	-0.0149975	1	1	Pet117
ENSMUSG00000098470.2	5.14395652	-0.0079825	0.01168526	0.40542349	0.52430155	0.70981008	C1rb
ENSMUSG00000098488.8	13.0652537	-0.0167587	0.00943927	2.9856656	0.08400474	0.18320258	Pla2g4b
ENSMUSG00000098557.2	377.249327	0.04349057	0.00434051	92.3334996	7.32E-22	4.75E-20	Kctd12
ENSMUSG00000098650.2	299.282551	0.01237298	0.00953136	1.69983593	0.19230943	0.34743855	Commd1b
ENSMUSG00000098678.2	21.3160126	-0.0130718	0.00891456	2.07414425	0.14981394	0.28730073	Mroh6
ENSMUSG00000098692.2	9.02093288	-0.0072385	0.01066967	0.47481085	0.49078224	0.67836321	Gm28041
ENSMUSG00000098715.7	2.88795636	0.01725978	0.01632078	0.9515775	0.32931808	0.51620232	Gm28053
ENSMUSG00000098781.2	4.20432645	-0.0262682	0.01641662	3.0128976	0.0826045	0.18073375	Gm28044
ENSMUSG00000098789.8	52.2934557	-0.0032731	0.00368863	0.80475187	0.36967643	0.56020538	Jmjd7
ENSMUSG00000098905.3	94.2209036	0.00105853	0.00302421	0.11991759	0.72912389	0.86946338	Zfp953
ENSMUSG00000098923.2	170.090089	0.00406336	0.0023371	3.02519163	0.08198064	0.17958533	Tmem185b
ENSMUSG00000098950.8	89.0097928	0.00645261	0.01019305	0.26124466	0.60926634	0.78164896	Gm28036
ENSMUSG00000099009.2	5.761708	0.0122222	0.03808487	0.01988441	0.8878604	0.96868802	Rdh16f1
ENSMUSG00000099032.3	40.3037602	-0.0667429	0.01132304	34.2280001	4.90E-09	6.21E-08	Tcf24
ENSMUSG00000099041.2	82.2468003	-0.0128892	0.0055201	5.35017367	0.02072021	0.05866828	Gm28035
ENSMUSG00000099083.8	549.54358	-0.0027107	0.00522066	0.27701869	0.59866196	0.77310781	Atf7
ENSMUSG00000099216.3	0.70335973	0.04870093	0.02326351	6.68287272	0.00973436	0.0311924	Obox8
ENSMUSG00000099398.2	9.63744041	0.09734554	0.01263837	51.2135702	8.28E-13	1.76E-11	Ms4a14
ENSMUSG00000099481.7	85.1733939	0.00561581	0.00333182	2.82494832	0.09280988	0.19829036	Xndc1
ENSMUSG00000099517.3	1.68066219	0.00216062	0.03924211	0.02211735	0.88177534	0.9650389	H3c8
ENSMUSG00000099583.3	3.36986119	0.00419478	0.01303602	0.12264132	0.72618723	0.86776026	H3c4
ENSMUSG00000099611.3	2.63331739	-0.0025268	0.03765673	-0.0388247	1	1	Vmn1r189
ENSMUSG00000099689.7	57.6431758	-0.0031978	0.00307218	1.10378942	0.29343599	0.47553808	Zfp383

ENSMUSG00000099787.4	1.01643599	-0.0053131	0.05239437	0.00889448	0.92486248	0.98901915	Vmn1r192
ENSMUSG00000099908.2	7.83095322	-0.0098295	0.0466768	0.01069384	0.91763675	0.98495405	Gm28539
ENSMUSG00000099913.2	3.27862475	-0.0245014	0.01684391	1.95713409	0.16182015	0.30411874	Gm28551
ENSMUSG00000099974.2	20.9250814	0.1075963	0.009694	105.934327	7.62E-25	7.02E-23	Bcl2a1d
ENSMUSG00000100200.2	1.90331676	-0.0305481	0.01105591	7.89150155	0.00496676	0.01758421	H2al1m
ENSMUSG00000100210.3	10.9101977	0.02976441	0.02002815	2.36937292	0.12373675	0.24812574	H3c7
ENSMUSG00000100235.9	129.286418	-0.0064385	0.00315426	4.04134452	0.04439844	0.10940049	Gm28557
ENSMUSG00000100254.2	2.05676401	-0.0125239	0.04544403	0.04518135	0.83167092	0.93522524	Trpc2
ENSMUSG00000100512.2	4.91473746	0.00352542	0.00982407	0.12054117	0.72844828	0.86883849	Ovol3
ENSMUSG00000100586.3	25.0459877	-0.0156326	0.01045975	2.15721193	0.14190196	0.27560109	Vmn1r90
ENSMUSG00000100621.3	1.84308559	0.00186286	0.01255498	0.04769976	0.82711533	0.93220921	Gm28374
ENSMUSG00000100679.3	13.4004733	0.03036048	0.0338714	0.74643028	0.38760878	0.57920304	Gm28778
ENSMUSG00000100838.2	2.46607558	0.05062869	0.03536764	2.39861062	0.12144307	0.2445033	Gm29094
ENSMUSG00000100937.2	3.42617407	0.00400722	0.00938315	0.18447301	0.66755706	0.82629235	Nscme3l
ENSMUSG00000101073.3	1.29950261	0.01782589	0.03628518	0.60663612	0.43605719	0.62750731	Vmn1r200
ENSMUSG00000101152.2	2.55807652	-0.0061201	0.06796131	0.01178239	0.91356202	0.98282712	Gm28305
ENSMUSG00000101337.7	3.20343177	-0.0199484	0.01886748	1.23599046	0.26624519	0.44186809	Dnah7c
ENSMUSG00000101355.2	3.1880445	-0.0092031	0.04570325	-0.0243102	1	1	H3c10
ENSMUSG00000101389.2	6.81503634	0.10047617	0.01273811	57.8715368	2.80E-14	7.25E-13	Ms4a4a
ENSMUSG00000101391.4	3.79788461	-0.0073787	0.02841624	-0.0171593	1	1	Or4c112
ENSMUSG00000101645.2	4.60575325	0.07237825	0.12014825	-0.0197184	1	1	Gm28635
ENSMUSG00000101678.4	64.4927023	0.01912485	0.00909191	4.22999081	0.03971566	0.10001425	Gm29609
ENSMUSG00000101892.2	245.393311	-0.0012209	0.00244855	0.24731814	0.61896982	0.78964854	9130401M01Rik
ENSMUSG00000101904.2	7.5427403	-0.0068891	0.01195309	0.35529262	0.55113229	0.73353683	Gm29427
ENSMUSG00000101918.3	184.643196	-0.0063381	0.03097587	0.01034881	0.9189717	0.98598497	Or4c120
ENSMUSG00000101959.2	4.58725111	-0.0246635	0.0140844	1.32997241	0.24881013	0.4202792	Ldhal6b
ENSMUSG00000101972.2	3.66558692	-0.0070624	0.03300788	-0.0440484	1	1	H3c11
ENSMUSG00000102018.9	7.37711199	0.01830265	0.00675239	7.05289053	0.00791374	0.02614928	Iqank1
ENSMUSG00000102037.2	18.9501199	0.09918934	0.00989573	82.9519196	8.41E-20	4.29E-18	Bcl2a1a
ENSMUSG00000102091.3	60.7681023	0.0024914	0.00616601	0.21084002	0.6461098	0.8108085	Or5m9
ENSMUSG00000102222.2	6.9752267	0.02614203	0.01027387	6.23283959	0.01254025	0.03849724	Pcdhga10
ENSMUSG00000102252.6	204.453603	0.01291427	0.00328226	15.6957836	7.44E-05	0.00043875	Snrpn
ENSMUSG00000102418.2	7.40890518	0.11236027	0.01202559	73.4499153	1.03E-17	4.07E-16	Sh2d1b1
ENSMUSG00000102428.2	14.0789407	0.00661199	0.00621623	1.08090775	0.29849458	0.4824101	Pcdhga12
ENSMUSG00000102439.5	1.30870709	0.01121524	0.02462106	0.50551647	0.47708621	0.66717392	Flg
ENSMUSG00000102440.2	4.65217991	0.00119058	0.01021016	0.01630956	0.89837936	0.97497703	Pcdhga9
ENSMUSG00000102543.6	12.742402	0.02849278	0.01176149	5.10416411	0.02386848	0.06588203	Pcdhgc5
ENSMUSG00000102627.2	6.73920567	0.00380781	0.02660015	0.03038883	0.86161089	0.95394374	Snurf
ENSMUSG00000102692.2	16.7036059	0.0078655	0.01205003	0.47636104	0.49007525	0.67778322	Dchs2
ENSMUSG00000102697.2	6.76988575	0.10756099	0.01844238	26.6743685	2.41E-07	2.34E-06	Pcdhac2
ENSMUSG00000102742.2	4.62464513	0.03336295	0.01053402	9.91552162	0.00163891	0.00677156	Pcdhga11
ENSMUSG00000102748.2	20.8688094	0.01520677	0.00614043	5.85859835	0.01550113	0.04612182	Pcdhgb2
ENSMUSG00000102752.2	148.097408	0.01381221	0.00513036	6.72199776	0.00952309	0.03063442	Gm7694
ENSMUSG00000102758.4	172.699352	0.00673188	0.00466699	2.04887775	0.15231843	0.29083021	Naaladl2
ENSMUSG00000102805.6	17.8030225	-0.0088504	0.01174659	0.48747486	0.48505588	0.67416193	Gm37240
ENSMUSG00000102918.2	163.709131	0.01733664	0.00508281	11.3852298	0.0007403	0.00337211	Pcdhgc3
ENSMUSG00000102976.7	1287.92798	-0.0007626	0.00253548	0.09020529	0.76391634	0.89280137	Zc3h11a
ENSMUSG00000103034.2	47.038908	-0.0147065	0.0096982	2.14237534	0.14327989	0.27779692	Gm8797
ENSMUSG00000103037.2	9.39920109	0.03031551	0.00853362	12.1748058	0.00048439	0.00232686	Pcdhgb1
ENSMUSG00000103081.4	2.77103222	0.03783382	0.01306135	8.08220804	0.00447019	0.01607471	Pcdhgb8
ENSMUSG00000103088.2	21.046578	0.00306035	0.00734569	0.17621483	0.67464654	0.83155036	Pcdhgb6
ENSMUSG00000103144.2	5.90051783	0.01644495	0.00834273	3.88672393	0.04866927	0.11800097	Pcdhga1
ENSMUSG00000103332.2	4.07499531	0.01147452	0.01093498	1.19335119	0.27465462	0.45265907	Pcdhga2
ENSMUSG00000103392.2	23.4493833	-0.0040494	0.00718601	0.30146412	0.582966	0.7603892	Gm38303
ENSMUSG00000103409.3	3.79630544	0.00614812	0.0139437	0.23171958	0.6302517	0.79872746	Lsmem2
ENSMUSG00000103421.2	240.466695	-0.0025705	0.00481892	0.29322676	0.58816013	0.76494871	Golt1a
ENSMUSG00000103472.2	6.26054876	0.02156019	0.00843277	6.73004535	0.00948022	0.03052566	Pcdhga7
ENSMUSG00000103476.2	1.52695378	-0.0162541	0.20950059	0.00783376	0.92947249	0.99147656	Strit1
ENSMUSG00000103567.2	10.5759889	0.00775014	0.00797345	0.89200216	0.34493532	0.53334498	Pcdhga5
ENSMUSG00000103585.2	9.41360247	-0.0038814	0.00804234	0.20430915	0.65126488	0.81396145	Pcdhgb4
ENSMUSG00000103677.2	6.71247956	0.02129325	0.00967482	4.26146518	0.03898628	0.09845339	Pcdhga4

ENSMUSG00000103711.2	352.925742	0.02669714	0.0045426	33.7218533	6.36E-09	7.92E-08	Tstd1
ENSMUSG00000103749.2	5.94506217	0.00912921	0.00905153	1.02507249	0.31131881	0.49640113	Pcdhgb5
ENSMUSG00000103766.6	11.9479628	-0.0345978	0.02607801	1.31765696	0.25101299	0.42276437	Gm38392
ENSMUSG00000103793.2	7.91974105	0.01857399	0.0089386	4.04544153	0.04429079	0.10919229	Pcdhga6
ENSMUSG00000103897.2	5.29945423	0.0123148	0.01252195	1.04317926	0.30708321	0.49214689	Pcdhga8
ENSMUSG00000103906.2	41.3363258	-0.0059256	0.00307379	3.73806056	0.05318615	0.12679882	Tigd5
ENSMUSG00000104063.2	9.76969314	0.02821295	0.00921449	8.9800758	0.00272939	0.01053953	Pcdhgb7
ENSMUSG00000104217.2	14.6638568	-0.0099931	0.01180513	0.70200615	0.40211042	0.59372583	Gm37988
ENSMUSG00000104252.6	3.57313437	0.00733224	0.03236138	-0.3522088	1	1	Pcdha4
ENSMUSG00000104346.2	7.64241698	0.00765563	0.01035261	0.55251562	0.45729163	0.64899696	Pcdhga3
ENSMUSG00000104445.2	122.192249	-0.0128323	0.0052448	6.22592427	0.01258932	0.03861772	Rhbg
ENSMUSG00000104713.5	533.208467	0.04345915	0.00579159	54.5597074	1.51E-13	3.52E-12	Gbp6
ENSMUSG00000105053.2	3.14109121	-0.0119696	0.02153682	0.27477083	0.60014926	0.77422308	Gm43064
ENSMUSG00000105096.2	359.12539	0.04093826	0.00789205	25.74175	3.90E-07	3.64E-06	Gbp10
ENSMUSG00000105103.2	1.33589289	0.13992473	0.07701987	-0.0818593	1	1	Gm43191
ENSMUSG00000105340.6	19.0743049	-0.0026888	0.005935	0.20360284	0.65182832	0.81405974	Gm42878
ENSMUSG00000105504.5	56.2726967	0.05484812	0.00595881	77.8296117	1.12E-18	4.93E-17	Gbp5
ENSMUSG00000105518.2	3.37900912	0.02278718	0.02822556	0.76615302	0.38140979	0.57287978	Gm42674
ENSMUSG00000105827.2	3.87947461	0.00545747	0.03136346	35.7092757	2.29E-09	3.03E-08	H2bc18
ENSMUSG00000105835.2	3.81991558	0.00556378	0.05102446	0.06963926	0.79186275	0.91118387	Gm43552
ENSMUSG00000105867.3	1.86810264	0.00967904	0.01455038	0.485448	0.4859649	0.67481115	Gm42517
ENSMUSG00000106379.2	2.4765727	0.01292857	0.02126867	0.51851418	0.47147618	0.66231311	Lhfpl3
ENSMUSG00000106447.2	1.7133763	-0.0300796	0.01597697	3.38834543	0.06565881	0.1504107	Gm42957
ENSMUSG00000106631.3	3.61867171	0.00785675	0.01291026	0.49014362	0.48386325	0.67309929	Gm42669
ENSMUSG00000106715.4	18.9899665	-0.0247843	0.0141185	2.96364122	0.08515597	0.18523368	Tmem265
ENSMUSG00000106864.4	885.463178	-0.0041744	0.00249128	2.81054475	0.09364657	0.19972613	Gtf3c2
ENSMUSG00000106918.4	852.793695	-0.0098373	0.00486567	4.11952568	0.04239096	0.10547877	Mrpl33
ENSMUSG00000107002.2	408.735618	-0.0065933	0.00277192	5.69087998	0.0170533	0.04988455	Ncbp2as2
ENSMUSG00000107023.3	377.656802	0.00031048	0.00536899	0.00331411	0.95409249	1	Gm42715
ENSMUSG00000107068.2	210.689866	-0.0249374	0.00795829	9.10895595	0.00254361	0.00992122	Gm42742
ENSMUSG00000107071.2	4.1895397	-0.0377186	0.02549755	2.55532238	0.10992339	0.22612251	Gm42420
ENSMUSG00000107283.4	728.628381	0.0012629	0.00325223	0.14647262	0.7019293	0.85128057	Mpv17
ENSMUSG00000107392.3	0.65044288	-0.0004762	0.07082755	-0.0021514	1	1	Pramel35
ENSMUSG00000107478.2	33.0432018	-0.0187756	0.03930186	0.24124265	0.62331005	0.79279322	Gm45234
ENSMUSG00000107482.3	3.1184255	-0.0205483	0.01257037	2.34409432	0.12575863	0.25126159	Etfbl
ENSMUSG00000107499.2	38.0153559	0.00228514	0.00515633	0.19416154	0.65947534	0.82068197	Ccdc142
ENSMUSG00000107504.4	1.94295685	-0.0077775	0.01309588	0.29637461	0.58616419	0.76312289	Gm35549
ENSMUSG00000107539.2	0.44680399	0.00307418	0.04674213	0.00660056	0.93524804	0.99453383	Gm44790
ENSMUSG00000107573.3	1.01512964	-0.0014052	0.03990956	-0.0022415	1	1	Or2y1d
ENSMUSG00000107705.2	80.0785634	-0.009283	0.01337209	0.45245361	0.50117207	0.68785612	Gm45062
ENSMUSG00000107877.3	27.7881247	-0.0043768	0.01536421	0.06856555	0.7934369	0.91207587	4933427D14Rik
ENSMUSG00000107928.2	127.57463	0.00405743	0.01304904	0.0935109	0.75976034	0.89011064	Gm45140
ENSMUSG00000108022.2	6.03466959	-0.0286116	0.0083926	12.2679615	0.0004608	0.0022235	Gm7298
ENSMUSG00000108265.3	0.96672356	0.00178628	0.03510802	0.01827189	0.89247466	0.97160892	Or2ak5
ENSMUSG00000108348.3	13.807729	0.03642247	0.01057807	11.1918851	0.00082156	0.00369443	Pnma8c
ENSMUSG00000108596.2	3.39835306	0.04272126	0.02484386	4.00604498	0.04533738	0.11138829	Gm49368
ENSMUSG00000108763.2	84.9677457	-0.0218775	0.00400199	28.8195772	7.94E-08	8.38E-07	Btnl12
ENSMUSG00000108815.2	42.6203293	-0.0188327	0.01959873	0.88739528	0.34618414	0.53457827	Gm49388
ENSMUSG00000108891.6	1.52235198	0.00077408	0.0313167	0.01326692	0.90830078	0.9800179	Or4f4-ps1
ENSMUSG00000108919.4	1.00511697	-0.014432	0.03607966	0.13794198	0.7103356	0.85686747	Or4k45
ENSMUSG00000108929.3	6.04697831	0.01687852	0.01262837	1.22881766	0.2676371	0.44363544	Cc2d2b
ENSMUSG00000108948.4	1.88116076	0.04026036	0.02025998	5.61283371	0.01782941	0.05177597	Or2ag13
ENSMUSG00000109061.2	28.0999855	0.00304814	0.01097262	0.07380823	0.78587062	0.90757112	Gm49320
ENSMUSG00000109129.2	4.50234118	0.03864628	0.04401058	0.6340162	0.42588596	0.61748739	Gm44973
ENSMUSG00000109179.3	29.7049901	-0.0140886	0.005317	7.0384131	0.00797797	0.02633199	Wdr97
ENSMUSG00000109324.3	246.066335	0.00317057	0.00340297	0.87282025	0.35017573	0.53851762	Prmt1
ENSMUSG00000109336.3	393.275748	-0.0018321	0.00418572	0.18497588	0.66713146	0.82615576	Samd4b
ENSMUSG00000109350.2	1188.73245	-0.0166567	0.00505449	3.13092901	0.07681995	0.1706901	Apoc2l
ENSMUSG00000109372.2	2.3638	-0.0124752	0.03551211	0.09261634	0.76087701	0.89102056	Gm19410
ENSMUSG00000109378.2	72.2033759	-0.0025859	0.00397715	0.41950011	0.51718658	0.70348113	Gm49396
ENSMUSG00000109396.2	0.54850048	0.01191089	0.13027651	0.03446517	0.85272083	0.94925088	Gm4565

ENSMUSG00000109398.3	2.79403756	-0.0042343	0.01191564	0.10309427	0.74814752	0.88197206	Gm3854
ENSMUSG00000109449.4	1.53914655	-0.0034556	0.02945002	0.00305524	0.95591997	1	Or4k36
ENSMUSG00000109482.3	1291.36655	0.01670253	0.00524143	9.69825248	0.00184443	0.00752644	Dhrs7l
ENSMUSG00000109493.2	1.99827341	-0.0032524	0.02064298	0.01936973	0.88931172	0.96954715	Gm45208
ENSMUSG00000109510.2	18.2774647	-0.0254734	0.02897496	0.51158258	0.47445457	0.66518691	Gm42417
ENSMUSG00000109511.2	225.858344	-0.0088612	0.00372555	5.55642148	0.01841301	0.05315009	Nup62
ENSMUSG00000109564.2	20.4746838	0.02237156	0.01045626	4.02877451	0.04473042	0.11011499	Muc16
ENSMUSG00000109713.2	4.95553281	0.03933178	0.00871417	18.6509663	1.57E-05	0.00010737	Pvrig
ENSMUSG00000109737.2	1.15842468	0.00532067	0.03862354	0.04851883	0.82566096	0.93114849	Spem3
ENSMUSG00000109764.2	5297.45638	-0.0128542	0.00515449	6.09461012	0.01355948	0.041176	Klkb1
ENSMUSG00000109771.3	88.6446262	0.00245534	0.00321562	0.57959353	0.44647169	0.63840983	Gm35315
ENSMUSG00000109801.3	1.34114215	-0.0120926	0.03174329	0.1973084	0.65690225	0.81868938	Or4f14d
ENSMUSG00000109835.3	1.15081004	0.01768069	0.04056677	0.49158125	0.48322279	0.67244633	Or4k2
ENSMUSG00000109850.2	0.68073671	-0.0447323	0.06015033	-0.5081833	1	1	Gm45692
ENSMUSG00000109864.2	7.50941055	0.01285481	0.01001662	1.54056918	0.2145331	0.37758211	Eid3
ENSMUSG00000109865.2	272.267144	-0.0021969	0.0017874	1.51904789	0.21776363	0.3819568	Hspa14
ENSMUSG00000109901.3	605.387989	0.00387053	0.00162853	5.5622162	0.01835217	0.05300689	Chmp1b
ENSMUSG00000109926.2	28.8241105	-0.0033555	0.0162945	0.04225347	0.83713738	0.93874262	Gm45808
ENSMUSG00000109946.2	10.5974063	0.02497574	0.00566217	19.0381344	1.28E-05	8.89E-05	Brd3os
ENSMUSG00000110040.2	100.452035	0.00282527	0.01620704	-0.218782	1	1	Gm49369
ENSMUSG00000110105.2	42.4639093	-0.0003404	0.00527543	0.00394187	0.94993823	1	Gm45844
ENSMUSG00000110170.2	5.96881556	0.00881123	0.01188214	0.59778257	0.43942533	0.63139116	St6galnac2
ENSMUSG00000110171.3	1.43558997	0.00832115	0.03150242	0.16272315	0.68666166	0.84051254	Or5p60
ENSMUSG00000110185.3	263.123728	-0.0067227	0.00308956	4.6562929	0.03093992	0.08160915	Igip
ENSMUSG00000110195.3	312.760571	0.00145406	0.00470331	0.09696566	0.7555018	0.88694202	Pde2a
ENSMUSG00000110206.2	106.857979	0.00523152	0.00411058	1.61064466	0.20440121	0.36439961	Flt3l
ENSMUSG00000110218.3	3.58286947	0.01889358	0.00892554	4.25361859	0.03916679	0.09883256	Tincr
ENSMUSG00000110221.2	1.77617928	-0.0386003	0.01336605	9.30181718	0.00228927	0.00907016	Gm36210
ENSMUSG00000110234.3	73.1830443	-0.0022472	0.00782032	0.08649669	0.76867905	0.89588694	Gm45799
ENSMUSG00000110277.3	233.33369	0.01512401	0.00339882	19.5446105	9.83E-06	6.97E-05	Gm45871
ENSMUSG00000110332.2	2.90728716	0.03453304	0.01219164	8.02762988	0.00460691	0.01646841	Gm19935
ENSMUSG00000110333.2	3.2056872	-0.0187836	0.04159052	0.09456142	0.75845642	0.88922599	Gm45861
ENSMUSG00000110344.2	129.940833	-0.016843	0.00452431	13.8100029	0.00020226	0.00107741	Smim36
ENSMUSG00000110439.2	94155.1829	-0.0835332	0.01683036	-5.5209255	1	1	Mup22
ENSMUSG00000110444.4	577.45222	-0.0114792	0.00497679	5.11822395	0.02367584	0.06548057	Gm10033
ENSMUSG00000110469.2	1.87041281	-0.0140001	0.0133498	1.13013878	0.28774574	0.4687644	Gm10358
ENSMUSG00000110519.3	1.46187052	0.02927933	0.01666776	4.28053563	0.0385512	0.09753321	Or7g23
ENSMUSG00000110622.2	1.6933131	-0.0183596	0.01801981	0.75395734	0.3852262	0.57686585	Iqcn
ENSMUSG00000110949.2	270.809407	-0.019011	0.00419196	20.4613779	6.08E-06	4.52E-05	Nudt8
ENSMUSG00000111021.3	1.66194077	-0.005305	0.0270849	0.02166976	0.88296902	0.96558361	Or1j21
ENSMUSG00000111174.2	1.21715327	-0.0124731	0.03588482	-0.0269291	1	1	Or4a68
ENSMUSG00000111259.3	1.41952363	-0.0043436	0.02830495	-0.0299779	1	1	Or10ak7
ENSMUSG00000111375.6	73.2535681	0.02364787	0.00429419	29.4607056	5.71E-08	6.14E-07	Btbd8
ENSMUSG00000111410.2	2.60299041	0.00267219	0.0614126	0.0174457	0.8949193	0.97287826	Gm49337
ENSMUSG00000111448.3	1.97322675	-0.008515	0.01541238	0.27841941	0.59773907	0.7723332	Or8b48
ENSMUSG00000111497.2	34.3763383	-0.0002786	0.00915566	0.00096182	0.97525905	1	Gm38431
ENSMUSG00000111517.2	1.45141066	-0.0074829	0.03034514	-0.0011077	1	1	Or4a39
ENSMUSG00000111590.3	1.47969995	0.0267438	0.02682622	1.76617637	0.18385599	0.33605296	Or4p4
ENSMUSG00000111611.2	1.25677992	-0.0053752	0.0264513	0.14528775	0.70307985	0.8518437	Or13c7c
ENSMUSG00000111692.2	9.39666741	0.00806732	0.01146138	0.48725839	0.48515283	0.6741773	Gm49373
ENSMUSG00000111709.2	191.369684	0.07091275	0.01289848	26.9424144	2.10E-07	2.06E-06	Gm3776
ENSMUSG00000111869.2	1.18914985	-0.0020931	0.04422099	-0.0106977	1	1	Or1j14
ENSMUSG00000112023.2	199.279199	0.08501216	0.00599342	171.336816	3.78E-39	2.34E-36	Lilrb4b
ENSMUSG00000112129.2	3755.86791	-0.0058421	0.00480254	1.49570152	0.22133394	0.38649458	Pbld1
ENSMUSG00000112148.2	263.701986	0.05875982	0.00587597	90.6300086	1.73E-21	1.06E-19	Lilrb4a
ENSMUSG00000112160.2	46.2760825	0.00316634	0.00475965	0.42452618	0.51468717	0.70123446	BC024063
ENSMUSG00000112241.2	35.4032925	0.00728809	0.01430721	0.21348062	0.6440529	0.8095203	Gm49325
ENSMUSG00000112449.2	212.962279	0.01005403	0.0070223	1.93150496	0.16459389	0.30825087	Srp54b
ENSMUSG00000112640.2	106.121675	0.00045625	0.00620094	0.00530518	0.94193608	0.99809086	Gm32687
ENSMUSG00000112743.2	6.80417374	-0.0632911	0.03572337	-6.4067795	1	1	Gm49358
ENSMUSG00000112920.2	77.4722418	-0.0066843	0.00477093	1.87893365	0.1704554	0.31683828	Spata22

ENSMUSG00000113149.2	21.1772246	-0.0082569	0.00899663	0.87234791	0.35030613	0.53868303	Gm49383
ENSMUSG00000113255.2	16.09082	-0.0044692	0.00621436	0.50616792	0.47680246	0.66697484	Tes3-ps
ENSMUSG00000113262.2	6.42345647	0.00832011	0.00806153	1.05643315	0.30403011	0.48866427	Gm48551
ENSMUSG00000113450.2	86.847438	0.00413121	0.00370095	1.21924425	0.26950902	0.44604868	Zfp935
ENSMUSG00000113475.3	2.77093193	0.02060876	0.07008841	0.24687637	0.61928316	0.7899201	Gm49366
ENSMUSG00000113640.2	8.22151795	0.00480156	0.00892824	0.3157668	0.57416282	0.75308817	Adat3
ENSMUSG00000113786.2	20.0226768	-0.003741	0.00898431	0.21231629	0.64495795	0.8101389	Gm49384
ENSMUSG00000113902.2	2205.09319	-0.0246548	0.00518918	22.8084703	1.79E-06	1.48E-05	Ndufb1
ENSMUSG00000113949.2	395.142787	-0.0040925	0.00333993	1.51392201	0.2185416	0.38300803	Scamp4
ENSMUSG00000114004.2	1.56320744	-0.0094053	0.02143501	0.34169186	0.55885427	0.74042415	Gm48552
ENSMUSG00000114133.2	209.431706	0.00584742	0.00722159	0.65980499	0.4166288	0.60785642	Btf3l4b
ENSMUSG00000114278.3	3.40196699	0.01042071	0.0151141	0.38146933	0.53681792	0.72120002	Gm49027
ENSMUSG00000114279.2	2.49605228	0.01244155	0.02884732	0.64220541	0.42291331	0.61463019	H2bc14
ENSMUSG00000114378.2	1.97947584	0.00186409	0.01130161	0.02450928	0.87559604	0.96170911	Gm49355
ENSMUSG00000114432.2	35.0893582	0.03154747	0.01566694	3.97433544	0.04619868	0.11315637	Gm49391
ENSMUSG00000114456.2	4.75206519	0.00650752	0.01055565	0.35665131	0.55037192	0.73290118	H2bc9
ENSMUSG00000114470.2	16.9571094	0.00450162	0.00892997	0.23965273	0.62445705	0.79353713	Gm49395
ENSMUSG00000114635.2	5.59200936	0.05481792	0.01340711	16.1257807	5.93E-05	0.00035745	Gm49392
ENSMUSG00000114797.2	439.514864	-0.0021605	0.00314191	0.4657322	0.49495714	0.68229451	Gm49336
ENSMUSG00000114942.2	1.07777736	-0.01938	0.20944645	0.00591559	0.93869287	0.99608629	Gm49361
ENSMUSG00000114982.2	1.04561367	0.00805089	0.03072202	0.15627352	0.69261083	0.84453906	Vmn1r14
ENSMUSG00000115018.3	33.3969154	0.00197229	0.01448643	0.0186088	0.89149395	0.97089977	Ndor1
ENSMUSG00000115020.2	2.02037466	-0.018869	0.02128491	0.75988442	0.38336469	0.57505546	Vmn1r218
ENSMUSG00000115021.2	1.86239705	-0.0059025	0.03303859	-0.04698	1	1	Vmn1r173
ENSMUSG00000115022.2	157.946823	-0.0148299	0.03229032	-1.6274967	1	1	Gm49387
ENSMUSG00000115027.2	3.60931113	-0.0015812	0.01815702	-0.0246346	1	1	Vmn1r81
ENSMUSG00000115074.2	4.21221227	-0.0139858	0.02311414	0.32273673	0.5699676	0.74962474	Ndor1
ENSMUSG00000115219.3	141.90994	-0.0068984	0.00338731	4.17774224	0.04095823	0.10260656	Eef1akmt4
ENSMUSG00000115232.2	18.1886021	-0.0075253	0.01891517	0.14031206	0.70797098	0.85544805	Gm49378
ENSMUSG00000115267.2	2.19742611	-0.0061791	0.02189657	-0.000106	1	1	Vmn1r76
ENSMUSG00000115338.4	1181.0829	-0.0066091	0.00357085	3.38989726	0.06559704	0.15034647	Pnp
ENSMUSG00000115423.3	293.957913	-0.0129386	0.00643054	4.06792148	0.04370502	0.10814379	Gm56450
ENSMUSG00000115463.2	4.27911426	-0.0059366	0.01345578	0.07267266	0.78748441	0.90855929	Gm49356
ENSMUSG00000115697.3	1.40081718	0.00797641	0.02983021	0.2663245	0.60580806	0.77854241	Vmn1r216
ENSMUSG00000115701.2	1.99828456	0.00342151	0.01926861	0.07975073	0.7776355	0.90186905	Vmn1r6
ENSMUSG00000115705.2	1.88065955	-0.0050023	0.02827739	0.01297467	0.909312	0.98051784	Vmn1r28
ENSMUSG00000115744.2	3.08898192	-0.0057141	0.0190306	0.07968935	0.77771884	0.90192135	Vmn1r80
ENSMUSG00000115791.2	7.924853	-0.0115281	0.02582302	0.15476226	0.69402529	0.84563839	Vmn1r217
ENSMUSG00000115792.2	2.65895371	-0.0096165	0.02557787	0.18742189	0.66507107	0.82467887	Vmn1r16
ENSMUSG00000115987.2	708.072368	-0.0057582	0.00268826	4.71547226	0.02989235	0.07922591	Vps28
ENSMUSG00000116016.2	1.47377381	0.00654267	0.13715232	0.00652109	0.93563819	0.99465354	Gm49496
ENSMUSG00000116024.2	1.42206078	-0.0074154	0.04588877	0.02746862	0.86836424	0.95799033	Gm49527
ENSMUSG00000116048.2	1594.53954	0.00219173	0.00253325	0.73711108	0.39058788	0.5819219	Septin2
ENSMUSG00000116069.2	1.99570804	-0.0154636	0.03007387	0.25236907	0.61541186	0.78689712	Gm49510
ENSMUSG00000116079.2	1.56335359	0.06861314	0.01445779	22.8513966	1.75E-06	1.45E-05	Gm36245
ENSMUSG00000116121.2	1.13621185	-0.0364971	0.02476627	3.16387172	0.07528444	0.16784751	Gm49486
ENSMUSG00000116138.2	357.459776	-0.010374	0.00254353	16.5700621	4.69E-05	0.00029025	C030006K11Rik
ENSMUSG00000116165.2	16.9416174	0.02536037	0.01049229	5.51418798	0.01886283	0.05422423	Pdxp
ENSMUSG00000116207.2	605.167362	-0.0009467	0.01232403	-8.0512533	1	1	Nnt
ENSMUSG00000116347.2	1.82986225	0.00618882	0.09492579	0.01707106	0.89604723	0.97347628	Gm49416
ENSMUSG00000116358.2	133.947036	0.14759453	0.04162443	1.36377917	0.24288359	0.41316185	Gm49450
ENSMUSG00000116378.2	57.5298009	-0.0895031	0.02678702	-3.507225	1	1	Gcat
ENSMUSG00000116461.2	17.5082107	0.00449281	0.01702102	0.07046132	0.79066628	0.91057306	Gm44502
ENSMUSG00000116563.2	13.286775	-0.0042747	0.01982712	-0.0070408	1	1	Gm56451
ENSMUSG00000116564.2	457.181038	-0.0004345	0.00268004	0.026231	0.87133745	0.95940269	Riok2
ENSMUSG00000116594.3	0.75229165	0.02632072	0.03428164	0.88296881	0.34738983	0.53552527	Gm49601
ENSMUSG00000116652.3	12.7642297	0.00301589	0.08372259	0.00657722	0.93536237	0.99456572	Fam246a
ENSMUSG00000116673.2	40.9585933	-0.008923	0.00517645	2.88747701	0.08927062	0.19232813	A630089N07Rik
ENSMUSG00000116780.2	25.4437893	0.01471441	0.02050236	-1.6677903	1	1	Dynlt2a3
ENSMUSG00000116876.2	0.75725538	0.0338184	0.02700545	1.59682048	0.20635438	0.36702786	Gm49721
ENSMUSG00000116895.2	42.8842655	0.00471425	0.00538414	0.76394365	0.38209718	0.57372957	Ermardl1

ENSMUSG00000116925.2	0.96404279	0.00173077	0.09569003	0.00535899	0.94164291	0.99791471	Gm49776
ENSMUSG00000116953.2	116.786817	0.00637465	0.00361096	3.11122826	0.0777543	0.17226311	Ermardl2
ENSMUSG00000117081.2	4.75623216	-0.0181046	0.03147796	0.44057644	0.50684436	0.69371077	Gm49948
ENSMUSG00000117098.2	463.279907	3.44E-06	0.0093238	-3.09E-06	1	1	Gm49909
ENSMUSG00000117145.2	5.02477799	0.02154264	0.0098153	4.81583804	0.02819936	0.07558068	Gng5c
ENSMUSG00000117148.2	3.6276	0.01541198	0.01758989	1.30228349	0.25379654	0.42611044	Vmn1r229
ENSMUSG00000117284.2	244.006777	0.00493549	0.00311283	2.4483065	0.11765174	0.23843735	Gm7072
ENSMUSG00000117286.2	2.14857082	0.04942712	0.01614461	8.51717849	0.0035181	0.01309418	Gm1043
ENSMUSG00000117310.3	4386.57049	0.00147384	0.00382871	0.14345598	0.70486919	0.85334525	Ptp4a1
ENSMUSG00000117338.2	22.5812806	-0.0246481	0.03172671	1.35877373	0.24375012	0.41422943	Gm49804
ENSMUSG00000117406.2	108.173196	-0.0016453	0.00472917	0.11377208	0.73588987	0.87386459	Ntn3
ENSMUSG00000117477.2	1.06725698	-0.0265404	0.0355055	0.45971158	0.4977588	0.68487569	Gm50092
ENSMUSG00000117621.2	68.114827	-0.0346564	0.00555229	39.1859604	3.85E-10	5.67E-09	Hspe1-rs1
ENSMUSG00000117679.2	239.659501	9.31E-05	0.00480338	0.00037803	0.98448766	1	Apbb3
ENSMUSG00000117694.2	7.43198611	-0.0028828	0.01591301	0.0731787	0.78676361	0.90807959	Snhg4
ENSMUSG00000117748.2	49.4954294	0.00901085	0.01536409	0.20432211	0.65125455	0.81396145	Derpc
ENSMUSG00000117789.2	35.5550018	0.00545151	0.01189375	0.21101898	0.64596991	0.81071922	Gm50388
ENSMUSG00000117809.2	11.5022271	0.00881906	0.02808679	0.20142662	0.65357177	0.81527099	Asdurf
ENSMUSG00000117819.2	135.952927	-0.0114106	0.00490385	5.36400197	0.02055655	0.0583169	Gm50253
ENSMUSG00000117863.2	1.92888028	0.00106417	0.02426233	0.01020879	0.91951985	0.98639174	Vmn1r61
ENSMUSG00000117873.2	1.58282731	0.00452861	0.05682501	0.05466448	0.81513687	0.92462936	Gm45928
ENSMUSG00000117874.2	1.38567496	-0.0120923	0.03689293	0.0714106	0.78929391	0.90976723	Gm50318
ENSMUSG00000117924.2	397.806239	-0.009254	0.00348928	7.09276819	0.00773956	0.02567083	Tmem223
ENSMUSG00000117942.2	147.919947	0.00025642	0.00371569	0.00481636	0.94467126	0.9994266	Maskbp3
ENSMUSG00000117975.2	33.1364213	0.01757283	0.00589668	8.57613135	0.00340598	0.0127372	Itprip
ENSMUSG00000118124.2	8.17370904	0.01332129	0.03054553	0.42104436	0.51641639	0.70282266	Gm50139
ENSMUSG00000118125.2	54.8776074	-0.0040278	0.00432255	0.86971798	0.3510334	0.53944968	Gm50387
ENSMUSG00000118215.2	3.14648893	0.00611819	0.01756658	0.27463359	0.60024032	0.77426898	Vmn1r55
ENSMUSG00000118298.2	1.51626485	0.00159996	0.0304497	0.010986	0.91652332	0.98434129	Vmn1r60
ENSMUSG00000118318.2	1.33035246	0.00255855	0.02114412	0.05455777	0.81531412	0.92471498	Vmn1r183
ENSMUSG00000118332.2	165.370151	-0.0008146	0.00399323	0.04059394	0.84032376	0.94071271	Fam220a
ENSMUSG00000118346.2	174.098565	0.00381002	0.00299025	1.66804345	0.1965208	0.35331501	Tmem179b
ENSMUSG00000118401.2	3.08704757	0.03106871	0.01186737	6.74470996	0.00940261	0.03033788	Gpr52
ENSMUSG00000118471.2	0.62955331	0.00546595	0.11204995	0.0024469	0.96054779	1	Gm50461
ENSMUSG00000118491.2	619.658872	0.01917145	0.01946156	0.98886538	0.32001984	0.50606692	Gm44505
ENSMUSG00000118504.2	135.492114	-0.0067707	0.00423324	2.53110886	0.11162175	0.22875234	Gm53012
ENSMUSG00000118506.3	2.48070959	0.01248723	0.04414958	0.1390979	0.70917947	0.85615534	Cfap141
ENSMUSG00000118516.2	2453.28357	-0.0294982	0.00607474	23.0638517	1.57E-06	1.31E-05	Cyp2d13
ENSMUSG00000118537.2	71.5312804	-0.0032767	0.00413315	0.62255604	0.43009903	0.62154989	Shld3
ENSMUSG00000118552.2	26.9117068	-0.0003305	0.01281968	0.00516089	0.94272978	0.99850775	Rplp2-ps1
ENSMUSG00000118559.3	5.56698069	0.00720048	0.00763653	0.9059946	0.34117941	0.52899405	A930007A09Rik
ENSMUSG00000118590.2	14.1863934	0.11086711	0.00768289	174.03657	9.72E-40	6.55E-37	Gm15433
ENSMUSG00000118591.2	4.14849259	0.08730551	0.01375965	33.9850732	5.55E-09	6.97E-08	Gm52955
ENSMUSG00000118623.2	4.01159292	0.01476111	0.02429427	0.33293311	0.56393705	0.74452949	Gm52968
ENSMUSG00000118640.2	1.34333006	0.0842395	0.02073181	18.1267733	2.07E-05	0.0001379	Gm7582
ENSMUSG00000118653.2	27.6109532	-0.0052527	0.01255587	0.16427371	0.68525191	0.83942825	Gm2296
ENSMUSG00000118661.1	5.0822294	-0.0054273	0.01647504	0.12063897	0.72834251	0.86880397	Muc6
ENSMUSG00000118662.2	28.5361179	-0.0044045	0.00447447	0.98277586	0.32151445	0.50774957	Tctn2
ENSMUSG00000118663.1	112.898213	-0.010625	0.00271785	15.1282833	0.00010045	0.00057378	Afg2b
ENSMUSG00000118664.1	229.464713	0.0108328	0.00330177	10.5394439	0.00116854	0.00503491	Tusc3
ENSMUSG00000118665.1	175.634386	-0.0004778	0.00280885	0.02833198	0.86633071	0.95656927	Lin54
ENSMUSG00000118667.1	29.6904058	0.0478443	0.00714344	42.4947124	7.09E-11	1.16E-09	Ahnak2
ENSMUSG00000118668.1	160.743468	0.01207335	0.00355986	11.4386203	0.00071933	0.00328802	Rps6ka4
ENSMUSG00000118669.2	267.365477	0.00977149	0.0031094	10.0673629	0.00150919	0.00630794	Arvcf
ENSMUSG00000118671.1	342.978772	0.00531698	0.00519702	1.05809881	0.3036492	0.48821774	Eppk1
ENSMUSG00000118672.1	3.73777615	0.00410279	0.01629467	0.03072243	0.86086108	0.95374443	Muc4
ENSMUSG00000120166.1	116.356051	0.00170708	0.00207211	0.67347483	0.41184274	0.60336639	Zfp33b
ENSMUSG00000121513.1	671.363847	-0.0003386	0.00364861	0.00857946	0.92620117	0.9895534	Ptprg
ENSMUSG00000121514.1	1.25904806	-0.0138868	0.05358829	0.06121365	0.80458789	0.91828391	ENSMUSG00000121514
ENSMUSG00000121515.1	1.60883873	-0.0040573	0.04076164	0.00197859	0.96452071	1	ENSMUSG00000121515
ENSMUSG00000121521.1	0.78115463	0.00757463	0.209441	0.00593137	0.93861132	0.99608629	ENSMUSG00000121521

ENSMUSG00000121543.1	0.51650909	0.00493967	0.11258565	0.01181699	0.91343569	0.98278149	ENSMUSG00000121543
ENSMUSG00000121556.1	0.61043058	-0.0003447	0.06829234	-0.0013657	1	1	ENSMUSG00000121556
ENSMUSG00000121570.1	10.4169639	-0.0800666	0.07742498	0.17065346	0.67953171	0.83497968	ENSMUSG00000121570
ENSMUSG00000121571.1	3.05347611	-0.0244971	0.0136822	2.91069333	0.08799404	0.1901271	ENSMUSG00000121571
ENSMUSG00000121572.1	4.78036946	-0.0085274	0.02742986	-0.0119352	1	1	ENSMUSG00000121572
ENSMUSG00000121577.1	1.74971583	0.00230113	0.20947866	0.00099861	0.97479045	1	ENSMUSG00000121577
ENSMUSG00000121583.1	458.429407	-0.0111456	0.00210607	27.7688091	1.37E-07	1.39E-06	Zftraf1
ENSMUSG00000121584.1	217.241265	-0.0048232	0.00296032	2.6899763	0.10098132	0.21174598	Tmem276
ENSMUSG00000121596.1	64.5124023	0.00319009	0.00360304	0.76746818	0.38100143	0.57247911	ENSMUSG00000121596
ENSMUSG00000121597.1	1.2016458	0.6485795	0.20941289	-0.0728976	1	1	ENSMUSG00000121597
ENSMUSG00000121605.1	4.81919112	0.09340783	0.0238598	2.19473952	0.13848262	0.27063145	Ahsp
ENSMUSG00000121606.1	228.184756	0.00278722	0.00465426	0.33310266	0.56383781	0.74449983	AKAP17A
ENSMUSG00000121607.1	93.7191332	-0.0060907	0.00443653	1.84259287	0.17464735	0.32288869	ENSMUSG00000121607
ENSMUSG00000121608.1	31.4509907	-0.0084519	0.00576321	2.01437294	0.15581563	0.2958277	ENSMUSG00000121608
ENSMUSG00000121914.1	93.7530953	-0.0081462	0.00544009	2.11752255	0.14562194	0.28120509	Sts
ENSMUSG00001074846.1	5.97416836	-0.1426263	0.15091441	0.05846772	0.80893437	0.92104881	Iqcf3
ENSMUSG00002076083.1	133.889849	0.00072863	0.00415064	0.03142494	0.85929567	0.95283438	Klhl17
