# Supplementary material for: LivAge: An Online Aging Clock for Murine Transcriptomic Age Estimation
Source: Aging Cell. 2026 Aug 21;25(9):e70677. doi: 10.1111/acel.70677 (PMC13498547; doi:10.1111/acel.70677)
Supplement: Supplementary file 5 [file ACEL-25-e70677-s005.pdf]

Supplementary Data 4. Estimated liver transcriptomic ages by LivAge for all samples used in this study. For each sample, the table reports the sample identifier, the sample set (Training, Optimization, Validation or Intervention), sex, chronological age and the estimated transcriptomic age in months.

Set	Sample	Sex	C. Age	Est. Age
Intervention	SRR4473784	m	22	10.8670005
Intervention	SRR4473785	m	22	7.548134813
Intervention	SRR4473786	m	22	7.837085949
Intervention	SRR4473787	m	22	9.696209415
Intervention	SRR4473792	m	2	2.646675302
Intervention	SRR4473793	m	2	2.309599876
Intervention	SRR4473794	m	2	2.566339959
Intervention	SRR4473795	m	2	2.419001082
Intervention	SRR9137545	m	4	5.358089611
Intervention	SRR9137546	m	4	6.47548349
Intervention	SRR9137547	m	4	4.729995036
Intervention	SRR9137548	m	4	3.639028187
Intervention	SRR9137549	m	4	5.472817726
Intervention	SRR9137550	m	4	3.118467028
Intervention	SRR9137551	m	4	4.559693728
Intervention	SRR9137552	m	4	5.898171874
Intervention	SRR9137553	m	4	3.282248071
Intervention	SRR9137554	m	4	4.475875198
Intervention	SRR9137555	m	4	3.552898196
Intervention	SRR9137556	m	4	3.708651737
Intervention	SRR9137557	m	4	4.757479972
Intervention	SRR9137558	m	4	3.75548844
Intervention	SRR9137559	m	4	3.469088049
Intervention	SRR9137560	m	4	3.655985345
Intervention	SRR7295411	m	6	3.964605239
Intervention	SRR7295412	m	6	4.617833417
Intervention	SRR7295413	m	6	4.482219736
Intervention	SRR7295414	m	6	5.648456975
Intervention	SRR7295415	m	6	5.052823016
Intervention	SRR7295416	m	6	5.446117594
Intervention	SRR7295417	m	6	5.268557328
Intervention	SRR7295418	m	6	4.5947863
Intervention	SRR5068696	m	3	2.692545645
Intervention	SRR5068697	m	3	3.952745383
Intervention	SRR5068698	m	3	2.57558835
Intervention	SRR4309641	m	15.5	16.32280504
Intervention	SRR4309642	m	15.5	13.25316957
Intervention	SRR4309643	m	15.5	13.10526173
Intervention	SRR4309644	m	15.5	11.25143218
Intervention	SRR4309645	m	15.5	10.75983353
Intervention	SRR4309646	m	15.5	8.91861852
Intervention	SRR4309647	m	15.5	12.99628836
Intervention	SRR4309648	m	15.5	14.63214539
Intervention	SRR4309649	m	15.5	14.25167728
Intervention	SRR36865167	m	21	14.15061056
Intervention	SRR36865170	m	21	16.57816872
Intervention	SRR36865175	m	21	15.44959744

Intervention	SRR36865178	m	21	13.56446613
Intervention	SRR36865179	m	21	15.09397108
Intervention	SRR29789945	m	24	19.80840479
Intervention	SRR29789946	m	24	20.92327849
Intervention	SRR29789947	m	24	21.36461706
Intervention	SRR29789948	f	24	15.73143839
Intervention	SRR29789949	f	24	13.50018704
Intervention	SRR29789950	f	24	16.10966376
Intervention	SRR29789951	f	24	15.35237415
Intervention	SRR29789952	f	24	12.68143186
Intervention	SRR29789953	f	24	17.80065476
Intervention	SRR29789954	f	24	15.21489849
Intervention	SRR29789955	f	24	12.91250986
Intervention	SRR29789956	f	24	14.39651581
Intervention	SRR29789957	f	24	15.65935215
Intervention	SRR29789958	m	24	18.14007676
Intervention	SRR29789959	m	24	15.89119729
Intervention	SRR29789960	m	24	14.8380058
Intervention	SRR29789961	m	24	17.76587961
Intervention	SRR29789962	m	24	21.68707717
Intervention	SRR29789963	m	24	22.84310208
Intervention	SRR29789964	m	24	19.61114115
Intervention	ERS29382556	f	5.1	6.049782099
Intervention	ERS29382557	f	5.1	6.437633561
Intervention	ERS29382558	f	5.1	5.617798152
Intervention	ERS29382559	f	5.1	5.401449559
Intervention	ERS29382560	f	5.2	6.24244202
Intervention	ERS29382561	m	5	7.508670562
Intervention	ERS29382562	m	5	6.955145958
Intervention	ERS29382562	m	5.2	5.133799453
Intervention	ERS29382563	m	5	6.321586832
Intervention	ERS29382564	m	5	6.416330646
Intervention	ERS29382565	f	4.9	5.043728484
Intervention	ERS29382566	f	4.9	5.541373443
Intervention	ERS29382567	f	4.9	5.241043905
Intervention	ERS29382568	f	4.8	4.243839365
Intervention	ERS29382569	f	4.8	4.704825565
Intervention	ERS29382570	m	4.8	4.74460215
Intervention	ERS29382571	m	4.9	4.934145225
Intervention	ERS29382572	m	4.7	5.854714548
Intervention	ERS29382573	m	4.9	4.595345682
Intervention	ERS29382574	m	4.7	5.252786604
Intervention	SRR36865181	m	21	12.09475651
Intervention	SRR36865183	m	21	14.08285758
Intervention	SRR36865167	m	21	14.15061056
Intervention	SRR36865170	m	21	16.57816872
Intervention	SRR36865175	m	21	15.44959744
Intervention	SRR36865178	m	21	13.56446613

Intervention	SRR36865179	m	21	15.09397108
Intervention	SRR36865181	m	21	12.09475651
Intervention	SRR36865183	m	21	14.08285758
Intervention	SRR29099965	m	6	8.552696534
Intervention	SRR29099966	m	6	7.045033253
Intervention	SRR29099968	m	6	8.080127471
Intervention	SRR29099969	m	6	8.027898188
Intervention	SRR9137561	m	4	3.996136419
Intervention	SRR9137562	m	4	4.879932247
Intervention	SRR9137563	m	4	4.037494761
Intervention	SRR9137564	m	4	5.010742617
Intervention	SRR4309632	m	15.5	11.17367594
Intervention	SRR4309633	m	15.5	11.1538288
Intervention	SRR4309634	m	15.5	10.68117633
Intervention	SRR4309635	m	15.5	6.414609639
Intervention	SRR4309636	m	15.5	8.278308631
Intervention	SRR4309637	m	15.5	9.117542341
Intervention	SRR4309638	m	15.5	9.183600005
Intervention	SRR4309639	m	15.5	10.15538901
Intervention	SRR4309640	m	15.5	12.20152586
Intervention	SRR7295403	m	6	3.720156235
Intervention	SRR7295404	m	6	4.202994671
Intervention	SRR7295405	m	6	3.781315844
Intervention	SRR7295406	m	6	3.996104238
Intervention	SRR7295407	m	6	5.031108937
Intervention	SRR7295408	m	6	4.190468378
Intervention	SRR7295409	m	6	4.103172776
Intervention	SRR7295410	m	6	3.496396603
Intervention	SRR5068699	m	3	2.307255883
Intervention	SRR5068700	m	3	2.273791995
Intervention	SRR5068701	m	3	3.16800751
Optimization	SRR6436158	m	4	3.642143055
Optimization	SRR6436159	m	4	3.826299988
Optimization	SRR6436160	m	4	4.368795846
Optimization	SRR11869343	m	11	11.47203032
Optimization	SRR11869345	m	11	11.57559158
Optimization	SRR11869347	m	11	13.53401345
Optimization	SRR11869349	m	1	0.577162043
Optimization	SRR11869351	m	1	0.434089777
Optimization	SRR11869353	m	1	0.52021007
Optimization	SRR11869355	m	20	21.08783494
Optimization	SRR11869357	m	20	21.33760483
Optimization	SRR11869359	m	20	23.55360698
Optimization	SRR11869361	m	26	21.1570056
Optimization	SRR11869363	m	26	22.65474564
Optimization	SRR11869365	m	26	22.78946969
Optimization	SRR11869367	m	32	29.10475928
Optimization	SRR11869369	m	32	33.74183435

Optimization	SRR11869371	m	32	24.71707653
Optimization	SRR11869373	m	3	3.187048816
Optimization	SRR11869375	m	3	3.195363709
Optimization	SRR11869377	m	3	3.9474427
Optimization	SRR9029885	m	2.5	6.051958513
Optimization	SRR9029886	m	2.5	5.859239825
Optimization	SRR9029887	m	2.5	7.00240398
Optimization	SRR9987187	m	3.5	3.720823233
Optimization	SRR9987188	m	3.5	3.825495457
Optimization	SRR9987189	m	3.5	3.693395318
Optimization	SRR11902465	m	6.5	5.722301903
Optimization	SRR11902470	m	6.5	5.43764328
Optimization	SRR11902475	m	6.5	4.944146307
Optimization	SRR18939772	m	12	14.32984223
Optimization	SRR18939773	m	12	13.33694728
Intervention	SRR8646175	m	2.5	4.830005531
Intervention	SRR8646177	m	2.5	5.654699778
Intervention	SRR8646178	m	2.5	6.184992979
Intervention	SRR8646180	m	2.5	6.749477846
Intervention	SRR8646181	m	2.5	4.864940827
Intervention	SRR8646186	m	2.5	6.303470496
Intervention	SRR9119201	f	12	10.30308007
Intervention	SRR9119202	f	12	8.487322818
Intervention	SRR9119203	f	12	9.578264045
Intervention	SRR9119204	m	12	10.77754685
Intervention	SRR9119205	m	12	12.43991083
Intervention	SRR9119206	m	12	12.36454855
Intervention	SRR9119207	f	12	8.158923811
Intervention	SRR9119208	f	12	7.120105759
Intervention	SRR9119209	f	12	8.957323678
Intervention	SRR9119210	m	12	8.972965652
Intervention	SRR9119211	m	12	10.17604528
Intervention	SRR9119212	m	12	8.810028593
Intervention	SRR9119213	f	12	11.24003729
Intervention	SRR9119214	f	12	9.70095004
Intervention	SRR9119215	f	12	9.326255838
Intervention	SRR9119216	m	12	12.19691453
Intervention	SRR9119217	m	12	11.97686895
Intervention	SRR9119218	m	12	6.320580638
Intervention	SRR9119219	m	5	4.278273344
Intervention	SRR9119220	m	5	4.463294993
Intervention	SRR9119221	m	5	2.896822448
Intervention	SRR9119222	m	5	4.63027672
Intervention	SRR9119223	m	5	4.19203776
Intervention	SRR9119224	m	5	5.434842052
Intervention	SRR9119225	f	12	8.824134333
Intervention	SRR9119226	f	12	7.720611708
Intervention	SRR9119227	f	12	10.34802015

Intervention	SRR9119228	m	12	7.509737931
Intervention	SRR9119229	m	12	11.40594467
Intervention	SRR9119230	m	12	10.99006532
Intervention	SRR9119231	m	5	3.143196967
Intervention	SRR9119232	m	5	3.22469203
Intervention	SRR9119233	m	5	3.085553146
Intervention	SRR9119234	m	5	5.52369863
Intervention	SRR9119235	m	5	5.132598242
Intervention	SRR9119236	m	5	5.167392027
Intervention	SRR9119237	f	6	5.470210879
Intervention	SRR9119238	f	6	4.770885256
Intervention	SRR9119239	f	6	5.536593674
Intervention	SRR9119240	m	6	5.205816919
Intervention	SRR9119241	m	6	4.996361792
Intervention	SRR9119242	m	6	3.816417156
Intervention	SRR9119243	f	6	5.865490069
Intervention	SRR9119244	f	6	6.095689344
Intervention	SRR9119245	f	6	5.87964064
Intervention	SRR9119246	m	6	6.234767812
Intervention	SRR9119247	m	6	5.551496081
Intervention	SRR9119248	m	6	5.369246799
Intervention	SRR9119249	f	6	5.539237762
Intervention	SRR9119250	f	6	7.827928759
Intervention	SRR9119251	f	6	6.365256312
Intervention	SRR9119252	m	6	6.117426277
Intervention	SRR9119253	m	6	8.195835648
Intervention	SRR9119254	m	6	6.912270034
Intervention	SRR9119255	f	6	5.482038916
Intervention	SRR9119256	f	6	6.248113822
Intervention	SRR9119257	f	6	5.509140849
Intervention	SRR9119258	m	6	4.915910654
Intervention	SRR9119259	m	6	5.172302177
Intervention	SRR9119260	m	6	5.328031703
Intervention	SRR9119261	f	6	6.409942687
Intervention	SRR9119262	f	6	6.402981183
Intervention	SRR9119263	f	6	6.902606619
Intervention	SRR9119264	m	6	5.513603641
Intervention	SRR9119265	m	6	7.285378616
Intervention	SRR9119266	m	6	6.175729569
Intervention	SRR9119267	m	14	10.79917839
Intervention	SRR9119268	m	14	10.33928599
Intervention	SRR9119269	m	14	11.41846047
Intervention	SRR9119270	m	14	7.057087537
Intervention	SRR9119271	m	14	7.239948447
Intervention	SRR9119272	m	14	7.796077484
Intervention	SRR9119273	f	6	5.80474485
Intervention	SRR9119274	f	6	5.365986642
Intervention	SRR9119275	f	6	5.191504906

Intervention	SRR9119276	m	6	5.529078839
Intervention	SRR9119277	m	6	4.998551679
Intervention	SRR9119278	m	6	6.030029966
Intervention	ERS29374298	m	4	6.065101508
Intervention	ERS29380426	m	4	5.683856579
Intervention	ERS29374310	m	4	4.623466935
Intervention	ERS29374311	m	4	4.539302143
Intervention	ERS29374298	m	4	5.744325058
Intervention	ERS29380427	m	4	5.329798309
Intervention	ERS29374299	m	4	6.181505771
Intervention	ERS29380428	m	4	5.353341489
Intervention	ERS29374300	m	4	5.71782609
Intervention	ERS29380432	m	4	5.598991256
Intervention	ERS29380433	m	4	5.650646681
Intervention	ERS29380434	m	4	6.519197972
Optimization	SRR18939774	m	12	12.76742926
Optimization	SRR1292215	m	21	17.9049605
Optimization	SRR1292216	m	21	18.79330979
Optimization	SRR1292217	m	21	19.05028133
Optimization	SRR2086344	m	5	5.22031652
Optimization	SRR2086348	m	5	6.105264878
Optimization	SRR2086349	m	5	5.470515414
Intervention	SRR36865169	m	21	13.30329291
Intervention	SRR36865172	m	21	14.94110206
Intervention	SRR36865174	m	21	12.38596026
Intervention	SRR36865177	m	21	12.87382706
Intervention	SRR36865180	m	21	12.10997156
Intervention	SRR36865182	m	21	13.29059643
Intervention	SRR36865184	m	21	12.55428149
Intervention	SRR36865185	m	21	11.48773184
Intervention	SRR36865162	m	21	7.558960409
Intervention	SRR36865163	m	21	10.1757786
Intervention	SRR36865164	m	21	9.992733788
Intervention	SRR36865165	m	21	10.4594785
Intervention	SRR36865166	m	21	8.854900864
Intervention	SRR36865168	m	21	9.845512041
Intervention	SRR36865171	m	21	11.36640168
Intervention	SRR36865173	m	21	10.67644158
Intervention	SRR36865176	m	21	9.779491787
Intervention	SRR9137565	m	4	3.439308637
Intervention	SRR9137566	m	4	4.588904855
Intervention	SRR9137567	m	4	5.077808569
Intervention	SRR9137568	m	4	6.156651723
Intervention	SRR4473788	m	22	18.31064361
Intervention	SRR4473789	m	22	17.99927653
Intervention	SRR4473790	m	22	16.32290466
Intervention	SRR4473791	m	22	14.2487883
Intervention	SRR4473796	m	2	2.456374275

Intervention	SRR4473797	m	2	3.303741772
Intervention	SRR4473798	m	2	2.435552615
Intervention	SRR4473799	m	2	3.619805903
Intervention	SRR29099967	m	6	6.459385404
Intervention	SRR29099970	m	6	6.59094109
Intervention	SRR29099971	m	6	6.158033295
Training	SRR5850781	m	12	13.5182204
Training	SRR5850782	m	12	14.6927918
Training	SRR5850783	m	12	14.33645285
Training	SRR5850784	m	12	14.93413856
Training	SRR5850785	m	12	13.84622841
Training	SRR5850786	m	12	13.08498259
Training	SRR5850787	m	12	14.57263586
Training	SRR5850788	m	12	14.57970509
Training	SRR5850789	m	12	15.16812035
Training	SRR5850790	m	12	16.03999732
Training	SRR8145413	f	1.75	2.734140286
Training	SRR8145414	f	1.75	2.28437746
Training	SRR8145415	f	1.75	2.553072447
Training	SRR8145416	f	1.75	2.474991377
Training	SRR8145417	f	1.75	2.188927477
Training	SRR8145418	f	1.75	2.240660921
Training	SRR9126652	f	21	18.99470003
Training	SRR9126667	f	12	9.112160138
Training	SRR9126676	m	3	3.748490143
Training	SRR9126677	m	15	9.551638352
Training	SRR9126682	m	27	18.03664391
Training	SRR9126712	m	21	16.88263983
Training	SRR9126727	m	9	7.597635475
Training	SRR9126770	m	18	16.62799017
Training	SRR9126772	m	1	1.647402507
Training	SRR9126806	m	27	21.95176618
Training	SRR9126831	m	6	5.062162631
Training	SRR9126838	f	12	9.077773952
Training	SRR9126867	f	18	14.4251367
Training	SRR9126881	m	24	22.28923489
Training	SRR9126899	f	1	1.338361856
Training	SRR9126914	m	9	5.567029883
Training	SRR9126918	m	18	14.25405051
Training	SRR9126980	f	15	15.43899548
Training	SRR9127012	f	6	8.122475284
Training	SRR9127017	m	1	1.304398562
Training	SRR9127018	m	27	17.25101936
Training	SRR9127050	m	3	2.33718695
Training	SRR9127056	f	15	12.40246651
Training	SRR9127081	f	9	7.875767811
Training	SRR9127087	m	24	17.883314
Training	SRR9127099	m	1	2.195481295

Training	SRR9127110	m	6	6.081052592
Training	SRR9127113	m	21	18.00673433
Training	SRR9127116	m	6	6.301516445
Training	SRR9127121	m	9	6.891945624
Training	SRR9127212	m	12	8.25952116
Training	SRR9127256	f	3	3.04708171
Training	SRR9127280	m	12	10.84251625
Training	SRR9127285	m	21	15.12976688
Training	SRR9127297	m	15	12.69245065
Training	SRR9127299	f	18	13.82542548
Training	SRR9127306	m	21	18.28128517
Training	SRR9127313	m	15	13.96651778
Training	SRR9127318	m	24	20.53421733
Training	SRR9127340	m	12	11.78247713
Training	SRR9127346	m	15	14.6814625
Training	SRR9127348	f	21	15.70487702
Training	SRR9127356	m	18	17.09460112
Training	SRR9127436	m	3	3.789512343
Training	SRR9127488	f	1	1.12881935
Training	SRR9127492	m	27	22.92893109
Training	SRR9127503	m	6	5.102333218
Training	SRR9127505	m	1	2.227140112
Training	SRR9127512	m	3	2.132109511
Training	SRR9127536	m	12	9.722053735
Training	SRR9127585	f	6	6.276100223
Training	SRR10573512	m	24	19.60063251
Training	SRR10573519	m	9	6.470525823
Training	SRR10573536	m	9	7.485556672
Training	SRR10573553	m	4	2.657725906
Training	SRR10573569	m	4	4.410212252
Training	SRR10573586	m	4	2.966848153
Training	SRR10573637	m	24	19.14365652
Training	SRR10573654	m	24	18.24469928
Training	SRR10573669	m	18	11.78403798
Training	SRR10573684	m	18	17.4235033
Training	SRR10573700	m	18	15.51417765
Training	SRR10573717	m	12	11.46474719
Training	SRR10573733	m	12	9.930448906
Training	SRR10573748	m	12	12.15169002
Training	SRR10573763	m	9	6.970014944
Training	SRR10573779	m	9	7.744897635
Training	SRR10573796	m	9	6.120372248
Training	SRR10573813	m	4	3.744376229
Training	SRR10573830	m	4	4.043590298
Training	SRR10573847	m	4	3.732459369
Training	SRR10573864	m	24	20.58652119
Training	SRR10573881	m	24	23.11169697
Training	SRR10573897	m	24	20.62250881

Training	SRR10573912	m	18	15.14631094
Training	SRR10573929	m	18	13.03518948
Training	SRR10573945	m	18	19.08069249
Training	SRR10573961	m	12	10.71018129
Training	SRR10573977	m	12	13.08347295
Training	SRR10573994	m	12	9.97667313
Training	SRR10574011	m	9	9.421792394
Training	SRR10851479	m	10	9.758385936
Training	SRR10851480	m	5	4.972739893
Training	SRR10851482	m	10	9.862424555
Training	SRR10851484	m	5	4.255880239
Training	SRR10851487	m	5	5.128664645
Training	SRR10851489	m	10	9.371340887
Training	SRR10851491	m	5	4.647894244
Training	SRR10851493	m	10	10.42996877
Training	SRR10851495	m	5	5.037657106
Training	SRR10851497	m	10	9.08898197
Training	SRR10851499	m	5	6.268604734
Training	SRR10851501	m	10	10.08875273
Training	SRR11250383	f	5	5.209083022
Training	SRR11250384	f	5	5.686831248
Training	SRR11250385	f	5	5.06035216
Training	SRR11250386	f	5	4.892877617
Training	SRR11250387	f	5	4.929729758
Training	SRR11250388	f	5	9.423510841
Training	SRR11250389	m	5	5.788129728
Training	SRR11250390	m	5	5.905514023
Training	SRR11250391	m	5	6.371333337
Training	SRR11250392	m	5	7.162037991
Training	SRR11250393	m	5	6.278937178
Training	SRR11250394	m	5	7.930682338
Training	SRR11250697	f	5	4.471340121
Training	SRR11250698	f	5	3.928391435
Training	SRR11250699	f	5	4.391388582
Training	SRR11250700	f	5	6.537899986
Training	SRR11250701	m	5	6.302542398
Training	SRR11250702	m	5	5.041312381
Training	SRR11250703	m	5	4.356432302
Training	SRR11250704	m	5	5.012265023
Training	SRR11250705	m	5	5.007063069
Training	SRR11250706	m	5	5.191995283
Training	SRR11250781	m	5	6.535212282
Training	SRR11250782	m	5	7.797383142
Training	SRR11250783	f	5	8.970822048
Training	SRR11250784	f	5	5.591106317
Training	SRR11250785	f	5	5.061805859
Training	SRR11250786	f	5	4.496803699
Training	SRR11250787	m	5	10.25875101

Training	SRR11250788	m	5	5.376563057
Training	SRR11250789	m	5	8.864436156
Training	SRR11250790	m	5	6.914950829
Training	SRR11250791	m	5	6.150172613
Training	SRR11250792	m	5	7.557516719
Training	SRR11250793	m	5	5.765420568
Training	SRR11251016	f	5	3.78495685
Training	SRR11251017	f	5	4.02476147
Training	SRR11902426	m	6.5	5.706179734
Training	SRR11902427	m	6.5	6.370848071
Training	SRR11902428	m	6.5	5.714112916
Training	SRR11902429	m	6.5	5.961560471
Training	SRR11902430	m	6.5	5.882497326
Training	SRR11902431	m	6.5	5.913295592
Training	SRR11902432	m	6.5	5.407108165
Training	SRR11902433	m	6.5	6.853895881
Training	SRR11902434	m	6.5	5.127411214
Training	SRR11902435	m	6.5	6.269483154
Training	SRR11902436	m	6.5	5.689794973
Training	SRR11902437	m	6.5	5.186309224
Training	SRR15881309	f	2.5	2.911179046
Training	SRR15881310	f	2.5	2.612343285
Training	SRR15881311	f	2.5	2.660152296
Training	SRR15881312	f	2.5	2.637731426
Training	SRR15881313	f	2.5	2.310090057
Training	SRR16296667	m	18	19.71817973
Training	SRR16296668	f	18	22.53224124
Training	SRR16296669	f	18	23.73448755
Training	SRR16296685	m	4	3.423778394
Training	SRR16296686	f	4	3.583231427
Training	SRR16296687	f	4	3.365618327
Training	SRR17260234	m	6	4.510202125
Training	SRR17260235	m	6	3.854678858
Training	SRR17260236	m	6	5.086724283
Training	SRR17260237	m	6	4.914447957
Training	SRR17260238	m	6	4.88138865
Training	SRR17260239	m	6	5.134928913
Training	SRR17260240	m	6	3.55722982
Training	SRR17260241	m	6	4.297422122
Training	SRR17260242	m	6	4.687880781
Training	SRR17260243	m	6	4.419184636
Training	SRR17260244	m	6	4.883120651
Training	SRR17260245	m	6	4.017333892
Training	SRR17260246	m	6	3.538373272
Training	SRR17260247	m	6	4.566608524
Training	SRR17260248	m	6	4.557586294
Training	SRR17260249	m	6	5.31510737
Training	SRR17260250	m	6	4.498689373

Training	SRR17260251	m	6	3.638348033
Training	SRR17260252	m	6	4.285008539
Training	SRR17260253	m	6	4.096657171
Training	SRR17260254	m	6	3.735042947
Training	SRR17260255	m	6	3.370358535
Training	SRR17260256	m	6	3.533847194
Training	SRR17260257	m	6	3.418625155
Training	SRR17260378	m	19	14.20498596
Training	SRR17260379	m	19	17.61371793
Training	SRR17260380	m	19	17.9572761
Training	SRR17260381	m	19	15.64117842
Training	SRR17260382	m	19	13.47007699
Training	SRR17260383	m	19	14.9942306
Training	SRR17260384	m	19	13.92267168
Training	SRR17260385	m	19	13.139687
Training	SRR17260386	m	19	15.50374816
Training	SRR17260387	m	19	13.19028204
Training	SRR17260388	m	19	13.28012975
Training	SRR17260389	m	19	11.66550131
Training	SRR17260390	m	19	15.26257771
Training	SRR17260391	m	19	17.46892843
Training	SRR17260392	m	19	17.99191533
Training	SRR17260393	m	19	14.06527689
Training	SRR17260394	m	19	14.64250166
Training	SRR17260395	m	19	13.7033834
Training	SRR17260396	m	19	13.48259623
Training	SRR17260397	m	19	16.31591385
Training	SRR17260398	m	19	14.19291028
Training	SRR17260399	m	19	17.9631814
Training	SRR17260400	m	19	14.95097082
Training	SRR17260401	m	19	15.68742423
Training	SRR17261649	m	22	20.44568457
Training	SRR17261650	m	22	20.18849139
Training	SRR17261651	f	22	16.84783483
Training	SRR17261652	f	22	15.97698119
Training	SRR17261653	f	22	20.85169003
Training	SRR17261796	m	25	23.81090118
Training	SRR17261797	m	25	19.07382271
Training	SRR21769374	m	2	1.463134622
Training	SRR21769376	m	2	1.857315499
Training	SRR21769522	m	2	1.909046337
Training	SRR21769524	m	2	1.649333057
Training	SRR21769526	m	2	1.687697333
Training	SRR21769528	m	2	1.994708622
Training	SRR21769530	m	2	1.60118868
Training	SRR21769532	m	2	1.771328325
Training	SRR21769534	m	2	1.486738001
Training	SRR21769536	m	2	1.666105363

Training	SRR21769538	m	2	2.550459852
Training	SRR21769540	m	2	1.316717713
Training	SRR21769542	m	2	1.65946816
Training	SRR21769544	m	2	3.749304062
Training	SRR21769546	m	2	1.449436907
Training	SRR21769548	m	2	2.047725298
Training	SRR21769550	m	2	2.199812032
Training	SRR21769552	m	2	1.942669902
Training	SRR21769554	m	2	1.35955068
Training	SRR21769556	m	2	1.974166517
Training	SRR21769558	m	2	1.827377146
Training	SRR21769560	m	2	1.397808813
Training	SRR21769562	m	2	1.498359576
Training	SRR21769564	m	2	1.168589322
Training	SRR23517674	m	30	26.04611757
Training	SRR23517675	m	30	26.76801842
Training	SRR23517676	m	30	27.43272723
Training	SRR23517677	m	30	27.42774622
Training	SRR23517678	m	24	24.56953067
Training	SRR23517679	m	24	27.60462498
Training	SRR23517680	m	24	28.61065618
Training	SRR23517681	m	24	23.29374361
Training	SRR23517682	m	15	15.78090559
Training	SRR23517683	m	15	15.55671865
Training	SRR23517684	m	15	16.19793669
Training	SRR23517685	m	15	13.87285086
Training	SRR23517686	m	6	5.153903025
Training	SRR23517687	m	6	5.07007622
Training	SRR23517688	m	6	5.365339354
Training	SRR23517689	m	6	5.606629607
Training	SRR23730067	f	2.5	4.349053997
Training	SRR23730068	f	2.5	4.246151461
Training	SRR23730069	f	2.5	4.249289413
Training	SRR23730070	f	2.5	4.806472549
Training	SRR23730071	f	2.5	4.693497026
Training	SRR23730072	f	2.5	4.2920415
Training	SRR24886776	m	24	28.39720563
Training	SRR24886777	m	24	30.79826979
Training	SRR24886778	m	24	23.4944944
Training	SRR24886814	f	24	23.10450881
Training	SRR24886815	f	24	22.63268918
Training	SRR24886827	m	6	7.083769874
Training	SRR24886828	m	6	5.561971385
Training	SRR24886829	m	6	5.441065321
Training	SRR28424098	m	15	15.11531055
Training	SRR28424099	m	15	14.31676484
Training	SRR28424100	m	15	16.12648552
Training	SRR28424101	m	15	17.39897785

Training	SRR28424102	m	15	16.66318931
Training	SRR28424103	m	15	15.57014094
Training	SRR28424104	m	15	14.68256432
Training	SRR28424105	m	15	16.05287363
Training	SRR28424106	m	9	9.142233802
Training	SRR28424107	m	9	10.39093096
Training	SRR28424108	m	9	10.20005828
Training	SRR28424109	m	9	8.694148353
Training	SRR28424110	m	9	9.864665266
Training	SRR28424111	m	9	10.96432662
Training	SRR28424112	m	9	8.93049428
Training	SRR28424113	m	9	9.230180817
Training	SRR28424130	m	9	11.52388734
Training	SRR28424131	m	9	10.41526174
Training	SRR28424132	m	2	2.729629762
Training	SRR28424133	m	2	2.305582688
Training	SRR28424134	m	2	2.523878701
Training	SRR28424135	m	2	2.532764327
Training	SRR28424136	m	2	2.265294032
Training	SRR28424137	m	2	2.279053912
Training	SRR28424138	m	2	1.904559757
Training	SRR28424139	m	2	1.976206682
Training	SRR28424140	m	2	2.945876098
Training	SRR28424141	m	2	3.02772795
Training	SRR28424154	m	30	26.24201999
Training	SRR28424155	m	30	27.63375919
Training	SRR28424156	m	30	25.56160828
Training	SRR28424157	m	30	26.43688532
Training	SRR28424158	m	30	25.67387265
Training	SRR28424159	m	30	26.35814017
Training	SRR28424160	m	30	25.88086421
Training	SRR28424161	m	30	23.47885415
Training	SRR28424190	m	30	26.86616394
Training	SRR28424191	m	30	24.95232471
Training	SRR28424192	m	30	25.48941063
Training	SRR28424193	m	30	27.76343656
Training	SRR28424194	m	24	29.36391903
Training	SRR28424195	m	24	23.56183086
Training	SRR28424196	m	24	23.11327383
Training	SRR28424197	m	24	21.73200751
Training	SRR28424198	m	24	20.96868012
Training	SRR28424199	m	24	23.44463622
Training	SRR28424200	m	24	22.78820745
Training	SRR28424201	m	24	24.55133667
Training	SRR28424202	m	24	23.82770751
Training	SRR28424203	m	24	24.22421889
Training	SRR28424204	m	15	17.29439241
Training	SRR28424205	m	15	15.39898695

Training	SRR30855989	f	3	3.425263467
Training	SRR30856001	f	3	2.989873923
Training	SRR30856011	f	2.75	3.345010694
Training	SRR30856023	f	3	3.325099766
Training	SRR30856036	f	2.75	2.975501615
Training	SRR30856047	f	2.75	3.062908228
Training	SRR30856072	f	2.75	2.243086477
Training	SRR30856083	f	3	3.36404472
Training	SRR32613941	m	14	4.500324843
Training	SRR32613944	m	20	10.95624736
Training	SRR32613945	m	14	4.251728045
Training	SRR32613949	m	20	18.25813812
Training	SRR32613950	m	14	9.450269695
Training	SRR32613954	m	26	15.66791657
Training	SRR32613955	m	20	18.88883447
Training	SRR32613977	m	14	11.43760503
Training	SRR32613981	m	26	18.22867891
Training	SRR32613982	m	20	12.26830655
Training	SRR32613983	m	14	4.422136703
Training	SRR32613984	m	14	6.319094737
Training	SRR32613989	m	26	6.90037696
Training	SRR32613990	m	20	11.95778185
Training	SRR32613991	m	14	6.633323944
Training	SRR32613995	m	26	8.14759733
Training	SRR32613996	m	20	8.025453574
Training	SRR32613997	m	14	4.259271074
Training	SRR32614109	m	26	11.52623049
Training	SRR32614110	m	20	7.650389792
Training	SRR32614111	m	14	5.387391639
Training	SRR1158577	m	1.5	3.089123509
Training	SRR1158578	m	1.5	2.623909897
Training	SRR1158579	m	1.5	3.254039375
Training	SRR1158580	m	1.5	2.866390527
Training	SRR1158581	m	1.5	2.937047198
Training	SRR1158582	m	1.5	3.000839075
Training	SRR1158583	m	1.5	3.53353755
Training	SRR1158584	m	1.5	3.516150864
Training	SRR1166896	m	27	27.93636468
Training	SRR1166897	f	27	23.5127753
Training	SRR1166898	f	27	27.2628453
Training	SRR1166899	f	27	20.33441394
Training	SRR1166900	m	27	26.73141331
Training	SRR1166901	m	27	26.99720811
Training	SRR2925235	m	2	2.032510873
Training	SRR2925236	m	2	2.06744566
Training	SRR2925237	m	2	2.459818293
Training	SRR2925238	m	2	2.166999965
Training	SRR2925239	m	9	11.20250601

Training	SRR2925240	m	9	7.923978914
Training	SRR2925241	m	9	9.323801002
Training	SRR2925242	m	9	7.738656633
Training	SRR2925243	m	9	10.04410311
Training	SRR2925244	m	15	16.38641908
Training	SRR2925245	m	15	12.52847447
Training	SRR2925246	m	15	14.93894629
Training	SRR2925247	m	15	17.99762283
Training	SRR2925248	m	15	16.54704143
Training	SRR2925249	m	24	24.09893458
Training	SRR2925250	m	24	22.40035146
Training	SRR2925251	m	24	21.7816449
Training	SRR2925252	m	24	21.56660583
Training	SRR2925253	m	24	20.61892355
Training	SRR2925254	m	30	24.72483413
Training	SRR2925255	m	30	35.23080175
Training	SRR2925256	m	30	25.38834247
Training	SRR2925257	m	30	20.35998671
Training	SRR2925258	m	30	27.20377102
Training	SRR6081860	m	2	3.646928963
Training	CRR117136	m	2	1.589370189
Training	CRR117137	m	2	1.478416704
Training	CRR117138	m	2	1.666302272
Training	CRR117139	m	2	1.388443448
Training	CRR117140	m	2	1.771228927
Training	CRR117141	m	6	5.171075253
Training	CRR117142	m	6	4.375493776
Training	CRR117143	m	6	4.8906953
Training	CRR117144	m	6	4.889790342
Training	CRR117145	m	6	4.540285711
Training	CRR117146	m	15	11.95120437
Training	CRR117147	m	15	12.83168097
Training	CRR117148	m	15	10.79780067
Training	CRR117149	m	15	10.72099969
Training	CRR117150	m	15	12.45705483
Training	CRR117151	m	19	19.34923771
Training	CRR117152	m	19	18.98311596
Training	CRR117153	m	19	17.74483023
Training	CRR117154	m	19	16.89466474
Training	CRR117155	m	19	19.09573314
Training	CRR117156	m	26	15.11830841
Training	CRR117157	m	26	13.08421231
Training	CRR117158	m	26	15.68608685
Training	CRR117159	m	26	16.79783658
Training	CRR117160	m	26	15.65113317
Training	SRR5642546	m	29	21.93537336
Training	SRR5642547	m	29	21.51299274
Training	SRR5642548	m	29	23.11335538

Training	SRR5642552	m	12	11.4683255
Training	SRR5642556	m	12	7.002158213
Training	SRR5642557	m	12	10.34722547
Training	SRR5642558	m	3	4.548074781
Training	SRR5642559	m	3	2.913478399
Training	SRR5642560	m	3	4.783029202
Validation	SRR28812615	f	12	13.26987502
Validation	SRR28812616	f	12	11.25162411
Validation	SRR28812617	f	12	11.63142384
Validation	SRR28812618	f	12	15.04984941
Validation	SRR28812619	f	12	10.87688567
Validation	SRR28812654	f	18	16.01588875
Validation	SRR28812655	f	18	17.60412556
Validation	SRR28812656	f	18	15.33007292
Validation	SRR28812657	f	18	17.58937382
Validation	SRR28812658	f	18	13.94987672
Validation	SRR28812670	f	7	7.367639349
Validation	SRR28812671	f	7	8.714851507
Validation	SRR28812672	f	7	8.165136168
Validation	SRR28812673	f	7	8.869511379
Validation	SRR28812674	f	7	8.720250539
Validation	SRR28812675	f	7	9.5526876
Validation	SRR32335461	f	10	9.949981154
Validation	SRR32335462	f	10	10.54353554
Validation	SRR32335463	f	10	9.231676433
Validation	SRR32335464	f	10	10.04078884
Validation	SRR32335465	f	10	9.545312767
Validation	SRR32335466	f	10	10.42174417
Validation	SRR32335467	f	10	10.43993732
Validation	SRR32335468	f	10	11.10163171
Validation	SRR32335469	f	10	10.22031964
Validation	SRR32335470	f	10	10.24312264
Validation	SRR32335471	f	10	9.121252836
Validation	SRR32335472	f	10	8.719131353
Validation	SRR36039785	f	2	2.431490328
Validation	SRR36039786	f	2	2.212721893
Validation	SRR36039787	f	2	2.240050015
Validation	SRR36039788	f	2	2.113409958
Validation	SRR36039789	f	2	2.088373772
Validation	SRR36039790	f	2	2.077957503
Validation	SRR33828225	m	24	22.47964933
Validation	SRR33828226	m	24	24.42271892
Validation	SRR33828227	f	21	16.68061044
Validation	SRR33828228	m	24	23.87763903
Validation	SRR33828229	f	24	15.80169324
Validation	SRR33828230	f	24	18.8630077
Validation	SRR33828231	f	24	18.97237595
Validation	SRR33828232	f	24	18.40428949

Validation	SRR33828233	f	24	19.69516944
Validation	SRR33828234	f	21	15.87041146
Validation	SRR33828235	m	24	21.18707749
Validation	SRR33828236	m	24	21.19175471
Validation	SRR33828237	m	24	21.55488159
Validation	SRR33828239	m	24	20.93190507
Validation	SRR33828240	m	24	22.83986634
Validation	SRR33828241	f	27	20.55591757
Validation	SRR33828242	f	21	18.14726753
Validation	SRR33828243	f	21	17.30583758
Validation	SRR33828244	f	21	17.25613333
Validation	SRR33828245	f	24	16.63519835
Validation	SRR33828396	m	6	5.567259059
Validation	SRR33828397	m	6	5.725906931
Validation	SRR33828399	f	12	13.76377262
Validation	SRR33828400	f	12	12.5627127
Validation	SRR33828401	f	12	14.60414578
Validation	SRR33828402	m	12	13.15510765
Validation	SRR33828403	f	12	13.5603203
Validation	SRR33828404	m	6	4.981203287
Validation	SRR33828405	f	12	12.99284741
Validation	SRR33828406	f	6	5.686579964
Validation	SRR33828407	f	6	5.981938697
Validation	SRR33828412	f	18	14.81874155
Validation	SRR33828413	m	12	13.97269525
Validation	SRR33828415	f	24	18.59725311
Validation	SRR33828416	f	24	18.28648293
Validation	SRR33828417	m	24	24.56146188
Validation	SRR33828418	m	27	27.18825078
Validation	SRR33828419	m	27	22.4896078
Validation	SRR33828421	f	27	19.24730634
Validation	SRR33828422	f	27	21.31875859
Validation	SRR33828423	m	27	21.23851273
Validation	SRR33828425	f	27	19.05778532
Validation	SRR33828426	f	27	18.87357334
Validation	SRR33828427	f	27	20.97909193
Validation	SRR33828428	m	24	23.57602637
Validation	SRR33828429	m	27	27.1583045
Validation	SRR33828430	m	27	24.14166372
Validation	SRR33828431	f	27	22.33606472
Validation	SRR33828432	m	27	20.76288718
Validation	SRR33828434	m	27	24.99946504
Validation	SRR33828435	m	27	25.10786831
Validation	SRR33828437	m	27	23.7563329
Validation	SRR33828510	m	12	14.73489152
Validation	SRR33828511	m	12	12.78241252
Validation	SRR33828512	f	18	18.93938916
Validation	SRR33828514	f	18	16.20076331

Validation	SRR33828515	f	12	12.43250228
Validation	SRR33828517	m	12	13.0600211
Validation	SRR33828518	m	12	13.37422999
Validation	SRR33828519	f	6	6.293343002
Validation	SRR33828520	m	12	12.27373386
Validation	SRR33828521	m	12	12.49340313
Validation	SRR33828522	f	12	11.43968524
Validation	SRR33828523	m	6	5.758707867
Validation	SRR33828524	m	6	7.09873413
Validation	SRR33828525	f	6	6.625032789
Validation	SRR33828526	f	6	6.59981471
Validation	SRR33828527	f	6	6.584857286
Validation	SRR33828528	f	6	6.464265787
Validation	SRR33828529	m	6	5.813577345
Validation	SRR33828530	m	6	5.346005148
Validation	SRR33828531	m	6	5.643322363
Validation	SRR33828532	f	6	6.634668762
Validation	SRR33828533	m	21	18.76559841
Validation	SRR33828534	f	24	19.19591258
Validation	SRR33828535	f	24	18.30365023
Validation	SRR33828536	f	27	19.26348538
Validation	SRR33828537	f	27	19.11996193
Validation	SRR33828631	m	18	17.73370725
Validation	SRR33828632	m	18	19.52588737
Validation	SRR33828633	m	21	18.35477629
Validation	SRR33828634	m	21	19.33447855
Validation	SRR33828635	m	21	20.87976566
Validation	SRR33828636	m	18	16.22146364
Validation	SRR33828637	m	18	18.49014126
Validation	SRR33828638	f	21	18.8680325
Validation	SRR33828639	f	21	17.6152913
Validation	SRR33828640	f	21	19.27557781
Validation	SRR33828642	m	21	23.13886311
Validation	SRR33828643	m	21	23.81520435
Validation	SRR33828645	f	18	16.39325567
Validation	SRR33828646	f	18	15.88121471
Validation	SRR33828647	f	18	17.77211898
Validation	SRR33828648	m	18	19.40965899
Validation	SRR33828649	m	18	19.3382027
Validation	SRR33828650	m	18	18.38056892
Validation	SRR33828651	f	18	16.39120499
Validation	SRR33828652	m	21	19.82151233
Validation	SRR33828653	f	18	13.25781146
Validation	SRR33828654	m	18	17.96832487
