# Supplementary material for: LivAge: An Online Aging Clock for Murine Transcriptomic Age Estimation
Source: Aging Cell. 2026 Aug 21;25(9):e70677. doi: 10.1111/acel.70677 (PMC13498547; doi:10.1111/acel.70677)
Supplement: Supplementary file 6 [file ACEL-25-e70677-s006.pdf]

Table S3. Summary of RNA-seq datasets used in the evaluation of genetic and environment interventions in estimated transcriptomic age. For each study, the table reports the accession number (from NCBI or EMBL-EBI), the number of samples, the genetic background and sex of the mice, the first author, the year of publication, and the intervention evaluated.

Accession	N	Strain	Sex	Reference	Intervention
PRJEB108956	20	C57BL/6N	M	Unpublished	*Lmna*$^{G609G/G609G}$
PRJEB108934	6	C57BL/6N	M	Unpublished	*Zmpste24*$^{-/-}$
PRJEB108947	6	C57BL/6N	M	Unpublished	*Zmpste24*$^{-/-}$
PRJNA524859	6	C57BL/6	M	Ferrara-Romeo I et al., 2020	*Terc*$^{-/-}$
PRJNA1114029	7	C57BL/6J	M	Terzié J et al., 2024	*Sprtn*$^{Y118C/Y118C}$
PRJNA545276	6	(PW/JxC3H/HeJ)/F2	M	Tyshkovskiy A et al., 2019	Snell Dwarf
PRJNA545276	6	(C57BL6/JxBALB/cBy J)/F2	M	Tyshkovskiy A et al., 2019	GHRKO
PRJNA1134780	20	C57BL/6J	F & M	Poudel SB et al., 2025	Induced GHRKO
PRJNA351224	16	Heterogeneous	F & M	Cole JJ et al., 2017	Ames Dwarf
PRJNA545276	24	UM-HET3	F & M	Tyshkovskiy A et al., 2019	Caloric Restriction
PRJNA344979	18	C57BL/6J	M	Green CD et al. 2017	High- or low-fat diets
PRJNA475758	16	C57BL/6J	M	Pak HH et al., 2019	Protein restriction
PRJNA545276	6	C57BL/6J	M	Tyshkovskiy A et al., 2019	Methionine restriction
PRJNA545276	48	UM-HET3	F & M	Tyshkovskiy A et al., 2019	Longevity drugs
PRJNA355861	6	C57BL/6	M	Meng Y et al., 2020	Metformin
PRJNA1404104	16	C57BL/6	M	Havas A et al., 2026	Rapamycin in H-F diet
PRJNA545143	24	UM-HET3	M	Tyshkovskiy A et al., 2019	Longevity drugs
