# Supplementary material for: LivAge: An Online Aging Clock for Murine Transcriptomic Age Estimation
Source: Aging Cell. 2026 Aug 21;25(9):e70677. doi: 10.1111/acel.70677 (PMC13498547; doi:10.1111/acel.70677)
Supplement: Supplementary file 7 [file ACEL-25-e70677-s007.pdf]

Table S2. List of gene predictors employed by LivAge. For each gene included in the transcriptomic aging clock, the table reports the Ensembl gene identifier, the gene symbol and the regression coefficient used in the model.

Ensembl gene ID	Gene symbol	Coefficient
ENSMUSG00000000037	*Scml2*	-0.429362027
ENSMUSG00000000167	*Pih1d2*	-0.047672236
ENSMUSG00000000303	*Cdh1*	-0.233998964
ENSMUSG00000000359	*Rem1*	0.226603516
ENSMUSG00000001095	*Slc13a2*	-0.000672974
ENSMUSG00000001143	*Lman2l*	-0.030233018
ENSMUSG00000001227	*Sema6b*	0.335958183
ENSMUSG00000001773	*Folh1*	0.480351767
ENSMUSG00000002007	*Srpk3*	0.077295726
ENSMUSG00000002076	*Hsf2bp*	0.226271552
ENSMUSG00000002910	*Arrdc2*	0.053565774
ENSMUSG00000003271	*Sult2b1*	-0.043310352
ENSMUSG00000003518	*Dusp3*	0.217324363
ENSMUSG00000004341	*Gpx6*	0.172927443
ENSMUSG00000005364	*Il5ra*	0.055384928
ENSMUSG00000005540	*Fcer2a*	-0.289281466
ENSMUSG00000005611	*Irag1*	-0.12146769
ENSMUSG00000006411	*Nectin4*	0.17139664
ENSMUSG00000006675	*P4htm*	0.10175301
ENSMUSG00000006777	*Krt23*	-0.024358136
ENSMUSG00000006782	*Cnp*	0.178596165
ENSMUSG00000008845	*Cd163*	-0.148342695
ENSMUSG00000013483	*Card14*	-0.007143318
ENSMUSG00000015027	*Galns*	0.036864233
ENSMUSG00000015405	*Ace2*	-0.212853334
ENSMUSG00000016283	*H2-M2*	0.189599341
ENSMUSG00000018341	*Il12rb2*	-0.028934663
ENSMUSG00000019194	*Scn1b*	0.432707566
ENSMUSG00000020151	*Ptprr*	0.002220076
ENSMUSG00000020212	*Mdm1*	-0.05328551
ENSMUSG00000020399	*Havcr2*	0.025209208
ENSMUSG00000020467	*Efemp1*	0.349557963
ENSMUSG00000020684	*Rasl10b*	0.041272107
ENSMUSG00000020787	*P2rx1*	-0.271983306
ENSMUSG00000020793	*Galr2*	-0.036491908
ENSMUSG00000020810	*Cygb*	0.420747208
ENSMUSG00000020811	*Wscd1*	0.103837834
ENSMUSG00000020871	*Dlx4*	0.320990096

ENSMUSG00000020961	*Ston2*	-0.054291071
ENSMUSG00000021003	*Galc*	0.067771198
ENSMUSG00000021208	*Ifi27l2b*	0.309461972
ENSMUSG00000021259	*Cyp46a1*	0.087192626
ENSMUSG00000021288	*Klc1*	0.242276522
ENSMUSG00000021553	*Slc28a3*	-0.008107898
ENSMUSG00000021747	*Cfap20dc*	-0.377414924
ENSMUSG00000022074	*Tnfrsf10b*	-0.166267662
ENSMUSG00000022129	*Dct*	-0.022843215
ENSMUSG00000022178	*Ajuba*	-0.18543158
ENSMUSG00000022404	*Slc25a17*	-0.194714033
ENSMUSG00000023259	*Slc26a6*	-0.018245083
ENSMUSG00000023439	*Gnb3*	0.101671693
ENSMUSG00000023992	*Trem2*	0.007886053
ENSMUSG00000024048	*Myl12a*	1.445128209
ENSMUSG00000024063	*Lbh*	0.106870468
ENSMUSG00000024112	*Cacna1h*	0.032625068
ENSMUSG00000024132	*Eci1*	-0.001212491
ENSMUSG00000024197	*Plin3*	0.070393107
ENSMUSG00000024485	*Slc4a9*	0.142482734
ENSMUSG00000024526	*Cidea*	0.086436595
ENSMUSG00000024697	*Gna14*	-0.087335066
ENSMUSG00000024937	*Ehbp1l1*	0.613119076
ENSMUSG00000024972	*Lgals12*	0.946232415
ENSMUSG00000025008	*Tctn3*	-0.064736698
ENSMUSG00000025165	*Sectm1a*	-0.190125317
ENSMUSG00000025200	*Cwf19l1*	0.161693496
ENSMUSG00000025229	*Pitx3*	-0.081524079
ENSMUSG00000025277	*Abhd6*	-0.082728974
ENSMUSG00000025330	*Padi4*	0.051428185
ENSMUSG00000025742	*Prps2*	0.033020128
ENSMUSG00000025916	*Ppp1r42*	-0.242518104
ENSMUSG00000025969	*Nrp2*	0.035082331
ENSMUSG00000026249	*Serpine2*	0.213855308
ENSMUSG00000026303	*Mlph*	0.108200802
ENSMUSG00000026525	*Opn3*	-0.408444844
ENSMUSG00000026616	*Cr2*	-0.088224203
ENSMUSG00000026622	*Nek2*	-0.329049571
ENSMUSG00000026728	*Vim*	0.17444916
ENSMUSG00000026879	*Gsn*	0.053305703
ENSMUSG00000026895	*Ndufa8*	0.578024141
ENSMUSG00000026970	*Rbms1*	0.328385721
ENSMUSG00000027230	*Creb3l1*	0.002483472
ENSMUSG00000027408	*Cpxm1*	-0.002683351
ENSMUSG00000027559	*Car3*	-0.310257836
ENSMUSG00000027612	*Mmp24*	0.024840874
ENSMUSG00000027624	*Epb41l1*	0.030511041
ENSMUSG00000027863	*Cd2*	0.151038584

ENSMUSG00000027965	*Olfm3*	0.187385655
ENSMUSG00000028008	*Asic5*	-0.05483031
ENSMUSG00000028439	*Fam219a*	0.001528654
ENSMUSG00000028475	*Spaar*	0.001246039
ENSMUSG00000028478	*Clta*	0.282979913
ENSMUSG00000028551	*Cdkn2c*	-0.916811083
ENSMUSG00000028587	*Orc1*	-0.077821308
ENSMUSG00000028712	*Cyp4a31*	-0.039889024
ENSMUSG00000029049	*Morn1*	-0.146737159
ENSMUSG00000029121	*Crmp1*	0.002210393
ENSMUSG00000029272	*Sult1e1*	0.17074289
ENSMUSG00000029814	*Igf2bp3*	-0.457049582
ENSMUSG00000029816	*Gpnmb*	0.182083903
ENSMUSG00000029832	*Nfe2l3*	-0.440324562
ENSMUSG00000029851	*Tcaf2*	0.080903792
ENSMUSG00000029862	*Clcn1*	0.038250176
ENSMUSG00000029882	*Prss3b*	-0.075816669
ENSMUSG00000030117	*Gdf3*	0.248518371
ENSMUSG00000030142	*Clec4e*	0.013239416
ENSMUSG00000030403	*Vasp*	0.005187533
ENSMUSG00000030519	*Apba2*	0.547462415
ENSMUSG00000030605	*Mfge8*	0.023281451
ENSMUSG00000030701	*Plekhb1*	0.576347672
ENSMUSG00000030707	*Coro1a*	0.012490047
ENSMUSG00000030772	*Dkk3*	0.045448486
ENSMUSG00000030869	*Ndufab1*	-0.072373091
ENSMUSG00000031132	*Cd40lg*	-0.000571459
ENSMUSG00000031271	*Serpina7*	0.143121161
ENSMUSG00000031290	*Lrch2*	-0.033947166
ENSMUSG00000031562	*Dctd*	-0.061549766
ENSMUSG00000031595	*Pdgfrl*	-0.443795575
ENSMUSG00000031703	*Itfg1*	-0.208887035
ENSMUSG00000031896	*Ctrl*	-0.094991414
ENSMUSG00000032131	*Abcg4*	-0.011245608
ENSMUSG00000032265	*Tent5a*	-0.232321768
ENSMUSG00000032315	*Cyp1a1*	-0.183864195
ENSMUSG00000032446	*Eomes*	0.004294841
ENSMUSG00000033083	*Tbc1d4*	0.078260415
ENSMUSG00000033327	*Tnxb*	0.038789241
ENSMUSG00000033389	*Arhgap44*	-0.174810492
ENSMUSG00000033906	*Zdhhc15*	-0.014793293
ENSMUSG00000034220	*Gpc1*	0.126930192
ENSMUSG00000034457	*Eda2r*	0.217241789
ENSMUSG00000034533	*Scn10a*	0.373274281
ENSMUSG00000034785	*Dio1*	-0.217372911
ENSMUSG00000035275	*Raver2*	-0.013456324
ENSMUSG00000035745	*Grin3b*	-0.001443674
ENSMUSG00000035914	*Cd276*	-0.021673881

ENSMUSG00000035919	*Bbs9*	-0.041788043
ENSMUSG00000036091	*Hyal3*	-0.003495348
ENSMUSG00000036223	*Ska1*	0.104891436
ENSMUSG00000036264	*Fstl4*	0.015813418
ENSMUSG00000036744	*Or2ag2b*	0.521417823
ENSMUSG00000036928	*Stag3*	-0.012770573
ENSMUSG00000036944	*Tmem71*	0.186652336
ENSMUSG00000037188	*Grhl3*	-0.044464823
ENSMUSG00000037337	*Map4k1*	0.007661988
ENSMUSG00000037685	*Atp8a1*	0.045632649
ENSMUSG00000037818	*Abhd18*	-0.006027612
ENSMUSG00000038151	*Prdm1*	0.00421095
ENSMUSG00000038155	*Gstp2*	3.47E-05
ENSMUSG00000038526	*Car14*	-0.073807677
ENSMUSG00000038560	*Sp6*	-0.001016855
ENSMUSG00000038602	*Slc35f1*	-0.080742123
ENSMUSG00000039621	*Prex1*	0.100864443
ENSMUSG00000039748	*Exo1*	-0.012911018
ENSMUSG00000039801	*Cplane1*	0.732563564
ENSMUSG00000039878	*Slc39a5*	-0.387012044
ENSMUSG00000039883	*Lrrc17*	-0.207142453
ENSMUSG00000040016	*Ptger3*	-0.066471545
ENSMUSG00000040420	*Cdh18*	0.002067112
ENSMUSG00000040782	*Cop1*	-0.225815692
ENSMUSG00000041471	*Shld2*	-0.02632291
ENSMUSG00000041698	*Slco1a1*	-0.070853447
ENSMUSG00000041737	*Tmem45b*	0.346066477
ENSMUSG00000042379	*Esm1*	0.369226449
ENSMUSG00000042401	*Crtac1*	0.545454949
ENSMUSG00000042436	*Mfap4*	-0.573549449
ENSMUSG00000042474	*Fcmr*	0.189867626
ENSMUSG00000042485	*Mustn1*	0.390513645
ENSMUSG00000042918	*Mamstr*	-0.019315362
ENSMUSG00000043458	*Pcdhb12*	0.23700838
ENSMUSG00000043681	*Fam25a*	-0.05250773
ENSMUSG00000043753	*Dmrta1*	0.643376667
ENSMUSG00000043807	*Ly6g5b*	-0.034050894
ENSMUSG00000044303	*Cdkn2a*	0.337847384
ENSMUSG00000044309	*Apol7c*	-0.013793666
ENSMUSG00000044703	*Phf11a*	-0.14802585
ENSMUSG00000044734	*Serpinb1a*	0.2495453
ENSMUSG00000044923	*Or5m5*	0.579880913
ENSMUSG00000044949	*Ubtd2*	-0.562697466
ENSMUSG00000045062	*Pcdhb7*	0.184327159
ENSMUSG00000045087	*S1pr5*	0.267965558
ENSMUSG00000045246	*Kcng4*	0.133414871
ENSMUSG00000045917	*Tmem268*	0.001085509
ENSMUSG00000046159	*Chrm3*	0.025172428

ENSMUSG00000046532	*Ar*	0.661425545
ENSMUSG00000047216	*Cdh19*	0.707038549
ENSMUSG00000047507	*Baiap3*	0.019601739
ENSMUSG00000047528	*Flacc1*	-0.037680328
ENSMUSG00000047746	*Fbxo40*	0.08922928
ENSMUSG00000048138	*Dmrt2*	-0.04173344
ENSMUSG00000048251	*Bcl11b*	0.001728942
ENSMUSG00000048330	*Ric3*	0.24495439
ENSMUSG00000048677	*Tpcn2*	0.000435749
ENSMUSG00000049488	*Tmem67*	-0.159499433
ENSMUSG00000049892	*Rasd1*	-0.089021464
ENSMUSG00000050010	*Shisa3*	0.6670659
ENSMUSG00000050538	*B230217C12Rik*	-0.079997289
ENSMUSG00000050737	*Ptges*	0.2008302
ENSMUSG00000050930	*Map10*	-0.272709412
ENSMUSG00000051056	*Gja10*	-0.039707655
ENSMUSG00000051579	*Tceal8*	-0.237694979
ENSMUSG00000051811	*Cox6b2*	-0.199469473
ENSMUSG00000052477	*Sp140l2*	0.111093619
ENSMUSG00000053004	*Hrh1*	0.146887155
ENSMUSG00000053044	*Cd8b1*	0.09907586
ENSMUSG00000053046	*Brsk2*	0.163455658
ENSMUSG00000053141	*Ptprt*	-0.122843934
ENSMUSG00000053461	*Hhipl2*	0.349843608
ENSMUSG00000053536	*Cstf2t*	0.083066623
ENSMUSG00000053702	*Nebl*	0.045523991
ENSMUSG00000053977	*Cd8a*	0.185455498
ENSMUSG00000054277	*Arfgap3*	0.079615646
ENSMUSG00000054364	*Rhob*	0.0020649
ENSMUSG00000055184	*Fam72a*	-0.004542717
ENSMUSG00000055254	*Ntrk2*	0.149264806
ENSMUSG00000056978	*Hamp2*	-0.082979101
ENSMUSG00000057594	*Arl16*	-0.003781022
ENSMUSG00000058620	*Adra2b*	0.018894497
ENSMUSG00000059119	*Nap1l4*	0.293341143
ENSMUSG00000059873	*Or5m11b*	0.795313269
ENSMUSG00000060586	*H2-Eb1*	0.02489071
ENSMUSG00000060969	*Irx1*	0.341263068
ENSMUSG00000061100	*Retnla*	0.105836414
ENSMUSG00000061577	*Adgrg5*	0.240031553
ENSMUSG00000061825	*Ces2c*	-0.069213595
ENSMUSG00000062783	*Csprs*	0.724520366
ENSMUSG00000063458	*Lrmda*	0.013648119
ENSMUSG00000063506	*Arhgap22*	0.166639262
ENSMUSG00000063568	*Jazf1*	-0.027575121
ENSMUSG00000064147	*Rab44*	0.043405629
ENSMUSG00000064215	*Ifi27*	0.360143035
ENSMUSG00000064345	*mt-Nd2*	0.132224884

ENSMUSG00000067149	*Jchain*	0.250033711
ENSMUSG00000067768	*Xlr4b*	0.024429302
ENSMUSG00000068392	*Rnase13*	-0.164575244
ENSMUSG00000070532	*Ccdc190*	0.048518879
ENSMUSG00000071661	*Zbtb3*	-0.041052502
ENSMUSG00000072214	*Septin5*	0.039473715
ENSMUSG00000072244	*Trim6*	-0.33917067
ENSMUSG00000072919	*Noxred1*	-0.055094583
ENSMUSG00000072964	*Bhlhb9*	-0.294906021
ENSMUSG00000073791	*Efcab7*	-0.032622307
ENSMUSG00000073901	*Or2ag19*	0.355101267
ENSMUSG00000078439	*Smim24*	0.382920259
ENSMUSG00000078687	*Mup8*	-0.020427641
ENSMUSG00000078719	*Msmp*	0.086098527
ENSMUSG00000079055	*Slc8a3*	0.134022989
ENSMUSG00000079190		0.522171322
ENSMUSG00000079244	*Gm5622*	0.107076873
ENSMUSG00000079457	*Gm7609*	0.170324439
ENSMUSG00000079808		0.118209776
ENSMUSG00000081058	*H3c15*	-0.03055286
ENSMUSG00000089873	*Mup13*	-4.91E-05
ENSMUSG00000090164	*BC035044*	-0.012578298
ENSMUSG00000091183	*Gm5141*	-0.01667043
ENSMUSG00000091572	*Vmn2r3*	-0.021196075
ENSMUSG00000091971	*Hspa1a*	-0.114436741
ENSMUSG00000092416	*Zfp141*	-0.14872432
ENSMUSG00000094686	*Ccl21a*	-0.549232004
ENSMUSG00000095071	*Tcstv2a*	0.180196428
ENSMUSG00000095180	*Rhox5*	0.105471398
ENSMUSG00000095595	*Fam177a*	0.272014607
ENSMUSG00000096674	*Mup15*	-0.040022918
ENSMUSG00000096688	*Mup17*	-0.052438993
ENSMUSG00000098022	*Zfp82*	-0.013237323
ENSMUSG00000099032	*Tcf24*	-0.35620235
ENSMUSG00000102222	*Pcdhga10*	0.040165871
ENSMUSG00000104445	*Rhbg*	-0.298095053
ENSMUSG00000106715	*Tmem265*	-0.105508287
ENSMUSG00000118401	*Gpr52*	-0.010050348
Intercept		2.982407584
