# Supplementary material for: LivAge: An Online Aging Clock for Murine Transcriptomic Age Estimation
Source: Aging Cell. 2026 Aug 21;25(9):e70677. doi: 10.1111/acel.70677 (PMC13498547; doi:10.1111/acel.70677)
Supplement: Supplementary file 8 [file ACEL-25-e70677-s008.pdf]

Supplementary Data 5. Estimated transcriptomic age by the TACO clock and by RAPToR in the validation cohort. For each sample, the table reports the sample identifier, chronological age in months, sex of the animal, and the estimated age from each clock.

Sample	C. Age	Sex	TACO_EN_liver_scaled	TACO_EN_liver_yugene	TACO_EN_multi_scaled	TACO_EN_multi_yugene	TACO_BR_liver_scaled	TACO_BR_liver_yugene	TACO_BR_multi_scaled	TACO_BR_multi_yugene	RAPToR
SRR36039785	2	Female	-21.97397329	0.588574576	-33.21638067	-9.986184292	-10.82563827	2.736162176	-35.9640563	-8.954024108	2.165829146
SRR36039786	2	Female	-23.29360905	-1.302566416	-34.39952576	-11.07424213	-11.79080072	0.988464279	-37.21719153	-10.18167258	2.165829146
SRR36039787	2	Female	-20.91283273	0.183975032	-33.76734491	-10.27358959	-10.55441953	2.305890946	-36.43922043	-9.186382937	1.874371859
SRR36039788	2	Female	-21.44629119	-0.186780492	-34.18329572	-10.95200332	-11.24835577	2.09504347	-37.01409557	-9.958267226	2.165829146
SRR36039789	2	Female	-23.07738693	-1.204302851	-33.25301989	-10.3595729	-11.99524815	1.074741612	-36.01481984	-9.475969073	2.603015075
SRR36039790	2	Female	-23.42031532	-0.397782429	-33.48669539	-9.650490117	-11.95360095	1.853120938	-36.10217525	-8.855948019	2.020100503
SRR33828396	6	Male	-15.96294243	7.533673716	-27.69632522	-0.470573164	-6.010199459	9.177376056	-30.33855925	0.10261513	9.743718593
SRR33828397	6	Male	-16.12649498	6.666680149	-26.86126405	-0.436499262	-6.513021355	8.724330343	-28.9801253	0.418014737	9.889447236
SRR33828404	6	Male	-17.02662251	6.751273716	-28.38914492	0.046765569	-7.458477088	8.717667188	-30.32791424	0.634662845	9.306532663
SRR33828406	6	Female	-19.75787146	6.372956319	-30.50686501	-0.823725492	-8.811393541	8.35385886	-32.90397505	0.495990489	8.140703518
SRR33828407	6	Female	-17.27308309	7.159412057	-30.87588215	-1.257902161	-7.40288142	9.152796938	-33.34235786	-0.251576921	7.557788945
SRR33828519	6	Female	-16.2953534	8.415747311	-27.31695788	1.385282024	-5.541682937	10.41159428	-29.63199817	2.421703118	7.557788945
SRR33828523	6	Male	-17.6201864	7.118837653	-28.29338085	-0.025530515	-6.753114853	9.403740348	-30.41510644	0.781617244	9.015075377
SRR33828524	6	Male	-16.07059052	9.07437204	-27.909208	0.561929868	-4.809541311	10.9203145	-30.11908336	1.438695468	9.889447236
SRR33828525	6	Female	-14.40010811	8.97983017	-26.84699273	0.715554962	-3.949367293	10.82649319	-29.33933536	1.595725309	7.994974874
SRR33828526	6	Female	-16.33181738	9.511294481	-27.8136278	0.7817964	-5.121342575	11.74983124	-29.94816306	1.45168414	7.849246231
SRR33828527	6	Female	-18.10939619	7.122116828	-28.04060982	0.016259903	-6.894907825	8.894345686	-30.55163311	0.699962443	7.994974874
SRR33828528	6	Female	-18.4886927	6.807545675	-29.50381297	-0.631060902	-7.609738174	8.862928111	-31.87343053	0.156667562	7.557788945
SRR33828529	6	Male	-17.19352027	7.143797209	-27.95946329	-0.356899112	-6.654182986	8.892222577	-30.22461158	0.73862046	9.452261307
SRR33828530	6	Male	-17.97550509	6.931121164	-27.72984709	0.045551164	-7.181672639	8.672002558	-29.93902466	0.558725186	9.743718593
SRR33828531	6	Male	-18.39634141	7.360492501	-28.55586588	-0.157517101	-7.138613996	9.053028722	-30.95934036	0.574476124	9.743718593
SRR33828532	6	Female	-17.45843983	8.152477002	-29.96597298	1.141327185	-6.424725822	10.21167598	-32.1257659	2.088798358	7.849246231
SRR28812670	7	Female	-12.03710073	6.177903071	-21.24276659	-0.881942908	-2.967075628	8.986625994	-23.43010013	-0.42099197	2.457286432
SRR28812671	7	Female	-11.34609779	8.722523328	-22.22987774	1.268987427	-2.483955017	10.69047305	-24.32836603	1.38652352	1.291457286
SRR28812672	7	Female	-8.158688254	9.795888751	-21.1138909	1.963731641	0.234733822	11.97340938	-23.7086184	1.938277162	1.728643216
SRR28812673	7	Female	-11.29481656	9.336740257	-21.63388333	2.300431148	-0.692265934	11.47347795	-24.34044894	3.182794941	1
SRR28812674	7	Female	-10.62925548	8.389513863	-21.4122214	0.285997993	-2.197410226	10.77097405	-23.86127414	0.653705483	1.43718593
SRR28812675	7	Female	-12.67597179	8.152312809	-23.75438679	-1.240132781	-1.745451285	10.24934052	-25.76814379	-0.811061274	1
SRR32335461	10	Female	-12.82780847	10.31160399	-23.87058449	0.78154479	-1.542803484	12.78551666	-26.56500631	0.843719599	1
SRR32335462	10	Female	-14.27994172	10.74874304	-23.26480034	1.745363391	-1.210078787	13.30646403	-24.86650445	1.944147593	1.291457286
SRR32335463	10	Female	-10.66851906	15.10343397	-18.98406383	7.315221966	2.628421074	17.11166869	-20.64856931	7.733038587	1
SRR32335464	10	Female	-9.808982636	14.58325208	-18.05808855	6.010212	2.923917669	16.50313867	-19.92732257	6.534223133	1
SRR32335465	10	Female	-12.23863456	12.10899754	-20.64461353	4.185681766	-0.241762934	14.31589426	-22.77410587	4.505193351	1
SRR32335466	10	Female	-12.72208654	12.56811071	-20.9943267	4.562532588	-0.142005681	14.93545815	-23.06544462	4.770391352	1
SRR32335467	10	Female	-11.49637825	14.00217452	-18.07091758	7.604116044	1.449653328	16.25257698	-20.22409057	8.294786233	1
SRR32335468	10	Female	-11.5334387	13.68102831	-19.47462044	6.040963362	0.923762057	15.60206275	-21.57880035	6.598563118	1
SRR32335469	10	Female	-12.19106605	12.13548511	-22.49620256	3.056020472	0.074165709	14.38873056	-24.29637778	3.553044363	1
SRR32335470	10	Female	-10.33238256	11.93812384	-22.76164298	2.847826629	-0.13914015	14.2084825	-24.75001927	3.103062545	1
SRR32335471	10	Female	-12.09689686	11.46144948	-20.69405171	3.489610001	-0.050334405	13.81715164	-22.59793963	3.668048417	1.291457286
SRR32335472	10	Female	-10.8049522	10.75696535	-20.38016698	2.749706866	-0.998755266	13.10689083	-21.98271178	2.669915293	1.291457286
SRR28812615	12	Female	-3.180861113	14.6683754	-16.50154651	6.156440635	4.443963191	17.19968574	-18.19728449	6.539920275	28.25125628
SRR28812616	12	Female	-9.167232312	9.282362384	-21.66909355	2.28625479	-0.20659983	12.15539925	-23.9732593	2.639979667	1.874371859
SRR28812617	12	Female	-8.13821016	11.27465549	-18.70413683	4.854363718	0.355836725	14.13453248	-20.74065243	5.322295672	1.874371859
SRR28812618	12	Female	-5.152234502	14.09839383	-17.51085214	7.198848789	4.38204803	16.7308111	-19.33762265	7.450368972	1.43718593
SRR28812619	12	Female	-9.173210802	13.07319516	-16.38381672	6.806143229	1.796672866	15.85252671	-18.44953176	7.449439534	2.020100503
SRR33828399	12	Female	-7.643556345	16.14300552	-19.27187114	9.855500829	2.921579638	18.28024716	-21.35707127	10.45994301	10.32663317
SRR33828400	12	Female	-11.42592908	13.73271478	-23.10613255	7.385650594	-0.869913242	16.22157673	-25.50516274	8.479833123	8.286432161
SRR33828401	12	Female	-8.807964208	14.6305359	-22.79601813	8.231262119	0.775135966	16.79573526	-25.13208624	9.480183247	8.869346734
SRR33828402	12	Male	-8.791013925	14.10103695	-19.91717755	7.113081498	1.065988502	16.1248411	-22.39690409	7.920111335	9.889447236
SRR33828403	12	Female	-8.791397345	14.0305846	-21.91491562	7.029268739	0.161719889	15.92761515	-23.96323101	8.028202772	8.869346734
SRR33828405	12	Female	-5.995472779	16.32840958	-20.78989691	9.275255199	3.823790701	18.73693027	-23.09450758	9.754032724	10.32663317
SRR33828413	12	Male	-8.557716918	14.08352159	-19.02512166	8.784645438	0.811188756	16.22886109	-21.2607815	9.55748121	10.03517588
SRR33828510	12	Male	-9.15996971	14.12166421	-20.28298346	7.949848895	0.243589594	16.12090754	-22.86734583	8.608014229	9.889447236
SRR33828511	12	Male	-11.10240897	13.02555884	-20.81239326	7.045901891	-0.243006219	14.91024167	-23.26235501	7.69943806	9.889447236
SRR33828515	12	Female	-8.016803288	15.06595937	-19.83811942	9.111129208	2.151449058	17.41368096	-21.96282998	10.06409977	10.32663317

SRR33828517	12	Male	-10.2510091	14.35025834	-22.26840569	7.723467729	0.46667654	16.36918386	-24.58882472	8.254749498	9.889447236
SRR33828518	12	Male	-10.47409218	13.24989822	-21.58378303	7.316991445	-1.087496509	14.81803276	-24.28458247	8.28378741	9.889447236
SRR33828520	12	Male	-10.28533479	13.14203366	-21.0278188	6.809231872	-0.52351868	15.25394856	-23.38825827	7.828835593	9.889447236
SRR33828521	12	Male	-10.17917256	12.86329922	-20.31325457	6.245329875	-0.395746346	14.72220269	-22.4492542	6.551575961	10.32663317
SRR33828522	12	Female	-7.754199293	14.5157818	-19.57212533	8.048593186	2.274270549	16.63792542	-21.95751114	9.120053715	9.015075377
SRR28812654	18	Female	1.393361783	20.66841286	-9.775784989	16.13823057	9.169571431	23.12195994	-11.69088081	15.15882273	2.020100503
SRR28812655	18	Female	-4.24997681	16.03910537	-15.76005653	10.48768442	4.595067728	18.73135018	-18.02223937	10.573004	30
SRR28812656	18	Female	-1.450978402	17.81942918	-14.22221503	11.28311716	7.073792329	20.4084106	-15.76598257	11.30783658	28.68844221
SRR28812657	18	Female	-2.542088855	17.64058134	-11.44126708	10.26588228	8.385564125	20.24241943	-13.13072149	10.18462957	1
SRR28812658	18	Female	4.424892564	19.58981411	-11.59280563	13.21586282	8.614604538	22.19604819	-14.1493126	13.37186596	29.85427136
SRR33828412	18	Female	-2.372865632	19.6876224	-16.47245664	12.28736182	6.091902663	21.74334592	-19.21247846	13.37047046	8.72361809
SRR33828512	18	Female	-2.54215453	18.52091562	-16.25012808	11.98567291	5.855848298	20.79279483	-18.4860745	13.12596486	9.743718593
SRR33828514	18	Female	-4.863454884	18.11530137	-17.2640189	11.89333748	5.014184912	20.48053158	-19.36608553	13.06739946	10.03517588
SRR33828631	18	Male	-5.882938462	16.51602634	-17.2327087	10.36181666	3.360739437	18.91805663	-19.13563618	11.3223912	10.7638191
SRR33828632	18	Male	-4.43852968	16.6405336	-19.24429006	9.101811941	2.498060057	18.00881739	-22.14515337	10.2129051	10.90954774
SRR33828636	18	Male	-8.458719377	15.00561927	-18.64912135	8.307501167	1.467094995	16.97969797	-21.09247404	9.398808116	9.889447236
SRR33828637	18	Male	-7.970079847	14.75338471	-18.13988206	8.365959872	2.287425785	16.49347506	-20.76447551	9.240473818	9.889447236
SRR33828645	18	Female	-5.646243284	16.13890226	-17.07864819	9.926552124	4.647881604	18.20716374	-19.47082937	10.90075019	10.03517588
SRR33828646	18	Female	-1.950497831	19.22360385	-16.68654011	12.50235546	5.836984812	21.42017278	-19.19738723	13.73176185	9.015075377
SRR33828647	18	Female	-2.057323902	19.89765961	-15.01431696	14.3289597	6.768098734	22.03681954	-17.54104316	15.04408183	11.7839196
SRR33828648	18	Male	-5.240278165	16.37541336	-15.57671596	12.32252639	3.79487855	18.83031989	-17.58646353	13.36442717	10.32663317
SRR33828649	18	Male	-7.487399496	16.48724705	-18.61356763	11.59258944	2.346745419	18.86060604	-21.27704503	12.26289871	9.889447236
SRR33828650	18	Male	-4.344961866	17.50115445	-17.4046778	11.79370314	3.981754401	19.51537574	-19.61497061	12.35329761	10.61809045
SRR33828651	18	Female	-4.544950642	18.37865028	-16.0788991	12.19216992	5.423673875	20.53369886	-18.34449878	13.2564117	9.743718593
SRR33828653	18	Female	-6.619630474	16.70415362	-16.78292756	10.57975489	3.721232015	19.3958126	-18.99472052	11.40606602	7.849246231
SRR33828654	18	Male	-5.686460472	15.92686255	-15.99535233	9.392081538	3.027889868	18.03958635	-18.25050179	10.16786818	10.47236181
SRR33828227	21	Female	-1.241307225	19.66031007	-14.64649375	12.411498	7.928157673	21.75769776	-16.98567339	13.39977133	10.03517588
SRR33828234	21	Female	-1.702813982	18.10484301	-15.33762973	11.66299997	6.671460216	20.0841663	-17.39852586	12.15329833	8.72361809
SRR33828242	21	Female	-2.786936873	19.51179573	-14.68949541	12.16448928	7.430098643	21.5851563	-16.89370146	13.40236535	11.7839196
SRR33828243	21	Female	-2.791887785	19.6627479	-14.29278412	13.09905793	6.614783314	22.30690833	-16.9301675	13.90035808	8.432160804
SRR33828244	21	Female	1.643191145	22.53706751	-12.83426117	15.35278813	9.203409153	24.48003093	-15.25588263	15.90178317	14.11557789
SRR33828533	21	Male	-3.51340357	15.99103073	-15.1146246	11.01619269	3.884917949	17.68460792	-17.92503921	12.21092896	10.90954774
SRR33828633	21	Male	-1.128408943	17.50922105	-15.8099972	11.42541692	5.896037278	19.56915052	-18.4591819	12.93439893	12.22110553
SRR33828634	21	Male	-2.874943823	17.42114191	-14.38894	11.56257786	4.706841271	19.25648454	-17.45406551	12.94865678	10.32663317
SRR33828635	21	Male	-2.016218659	19.76497509	-14.1510719	12.8239124	5.535755279	21.28487714	-17.17327026	14.01963754	10.7638191
SRR33828638	21	Female	0.685640995	20.62087224	-14.01552233	12.89369164	8.69441249	22.73103132	-16.53549857	14.02124877	11.7839196
SRR33828639	21	Female	-2.093585782	19.86911891	-14.12069611	14.48701743	6.60737022	22.14014995	-16.24950919	15.86477263	10.7638191
SRR33828640	21	Female	0.421520388	20.92572706	-13.59131392	13.23954518	8.686965879	23.35510786	-16.06932709	14.36124455	13.8241206
SRR33828642	21	Male	-2.174497597	19.34804297	-14.64454386	13.3904286	5.69842748	21.0389034	-16.85621596	14.50349345	11.05527638
SRR33828643	21	Male	-1.032090796	19.83649126	-13.77569087	13.24225597	5.842002211	21.61003703	-16.35980522	14.45713571	11.7839196
SRR33828652	21	Male	-1.026522471	17.75671948	-13.56774914	12.13958204	5.869049206	19.56737649	-16.45212898	13.45793988	13.24120603
SRR33828225	24	Male	-0.127696567	20.86018501	-12.6523035	15.73512582	7.450041256	22.86727923	-15.14240735	17.00900026	11.92964824
SRR33828226	24	Male	-2.643586464	19.84304173	-14.12248495	13.69028007	5.317248187	21.91921701	-16.65197691	14.67831022	11.7839196
SRR33828228	24	Male	-1.948247285	19.69871806	-14.62450914	14.51939876	5.784406218	21.53171623	-17.04225928	15.2735698	10.7638191
SRR33828229	24	Female	-1.691426192	19.79124278	-15.15916907	12.46886717	7.517885073	22.21750533	-16.90504205	13.44731878	10.7638191
SRR33828230	24	Female	-1.493128152	21.51951176	-11.84066254	16.1245182	8.728454664	23.0068417	-13.37621732	17.52136231	30
SRR33828231	24	Female	3.558589996	22.77772536	-9.774649025	16.17217515	10.96084526	24.86512635	-11.66664232	17.27418748	28.25125628
SRR33828232	24	Female	1.616677497	20.36179514	-11.33243252	14.01028296	8.701352216	22.56083759	-13.23131753	15.49736587	30
SRR33828233	24	Female	-0.920223056	21.93615982	-14.28803212	15.94349615	9.020573402	24.57200282	-16.36334118	17.04654769	10.32663317
SRR33828235	24	Male	-1.85577279	18.02562976	-15.46915712	10.9780049	5.654585267	19.8599925	-18.37632899	12.53644213	10.90954774
SRR33828236	24	Male	0.208391389	24.78630558	-12.59914476	16.91177362	10.30255508	27.0856207	-14.9284565	18.64523392	30
SRR33828237	24	Male	-3.516633836	17.2417002	-16.24642584	11.56224434	4.54718526	18.91818347	-18.80878479	12.02918919	10.90954774
SRR33828239	24	Male	-2.941561694	19.13193891	-15.0448141	14.3249647	5.765264579	21.30001381	-17.28212268	15.54774827	11.05527638
SRR33828240	24	Male	-4.6407149	18.81890224	-16.44259592	13.1268757	4.610050781	20.80181267	-18.87055961	13.75193645	10.32663317
SRR33828245	24	Female	-1.407925545	20.50404499	-13.7309457	13.83957786	7.877518429	22.97616131	-15.92147673	14.51202826	10.03517588
SRR33828415	24	Female	-2.727664247	19.54954015	-13.90621071	14.24532793	6.86661139	21.91712912	-16.17261058	15.40382344	9.743718593
SRR33828416	24	Female	-1.766997516	19.73347241	-14.19420993	13.33052935	6.693839149	21.96885216	-16.84113264	14.54143901	9.743718593

SRR33828417	24	Male	-1.389576304	21.12696636	-14.13897165	15.68925983	6.711863543	23.12959994	-16.74317353	16.41150612	11.05527638
SRR33828428	24	Male	0.418126635	25.47546993	-9.596567587	19.81592482	10.89675088	27.18783317	-11.84609767	20.76412807	14.11557789
SRR33828534	24	Female	-1.067543664	20.4712424	-14.15505067	14.41550818	6.975525285	22.93874045	-16.07466158	15.55816543	9.743718593
SRR33828535	24	Female	-0.637067087	20.21704822	-13.47921306	13.90198924	7.930655838	22.16454873	-15.64562713	15.20560322	10.7638191
SRR33828241	27	Female	-3.232994024	21.73993224	-12.5786138	16.06888435	9.450704636	24.12786723	-14.48984933	17.18759272	26.21105528
SRR33828418	27	Male	1.047318882	22.9244353	-11.68882808	16.78733806	8.863724689	24.60787418	-14.46523799	18.15288056	12.36683417
SRR33828419	27	Male	0.974133545	24.71234597	-9.973613872	19.04788121	11.88807119	26.94499411	-11.83641683	20.90769513	30
SRR33828421	27	Female	3.010506375	22.84653517	-14.28232157	16.88983733	9.599264207	25.13497221	-16.56613202	17.72286663	28.68844221
SRR33828422	27	Female	1.839817811	21.12937089	-12.52955183	15.41543867	9.025407663	23.53087656	-14.8770396	16.11696348	13.53266332
SRR33828423	27	Male	-2.185225386	22.58757128	-12.43766495	16.30264015	8.255557255	24.86276868	-14.47277646	17.62941628	11.49246231
SRR33828425	27	Female	2.525077815	23.98483089	-11.41507173	15.93663124	10.19678894	26.10873465	-13.83991838	16.92855716	9.16080402
SRR33828426	27	Female	3.433459255	23.26758411	-11.14109709	16.66813743	11.47365908	25.76236405	-13.44330659	18.08815274	13.8241206
SRR33828427	27	Female	5.133074237	24.49875049	-8.225757227	18.62280936	12.45868635	26.55310947	-10.43808202	20.22270182	27.95979899
SRR33828429	27	Male	4.635035751	26.93870993	-5.996299507	20.03430326	13.80231593	29.15331579	-9.000750573	21.07202309	28.83417085
SRR33828430	27	Male	0.824024504	23.83833578	-10.41961403	16.7656917	10.39503928	25.4415175	-12.74535804	18.27268287	28.83417085
SRR33828431	27	Female	3.17090622	23.84339444	-9.839035115	18.09250401	11.34473723	26.11296296	-12.33905044	18.96840394	26.79396985
SRR33828432	27	Male	-3.525153628	19.11171626	-16.32277331	8.910047521	5.050411891	20.72801224	-19.45122823	10.21630676	30
SRR33828434	27	Male	-0.068315077	23.21882033	-14.63466414	14.99595532	8.392297716	24.93330723	-17.07451479	16.6080981	28.68844221
SRR33828435	27	Male	-2.417048822	21.09560365	-11.20846044	15.31226407	8.436586044	23.21662144	-13.53815037	16.49173903	11.49246231
SRR33828437	27	Male	-3.01899466	19.84170144	-14.99771688	13.10072579	5.714093457	21.73951782	-18.0813883	14.06072217	12.94974874
SRR33828536	27	Female	2.254652867	23.31412551	-12.27209826	16.81265283	9.366437513	25.52530432	-14.52299931	17.82559924	10.7638191
SRR33828537	27	Female	-1.217462614	20.49138495	-14.29260765	15.8388491	7.004804426	22.68416259	-16.4312986	16.46287395	10.32663317
