# Supplementary material for: LivAge: An Online Aging Clock for Murine Transcriptomic Age Estimation
Source: Aging Cell. 2026 Aug 21;25(9):e70677. doi: 10.1111/acel.70677 (PMC13498547; doi:10.1111/acel.70677)
Supplement: Supplementary file 9 [file ACEL-25-e70677-s009.pdf]

**Supplementary Data 2. Gene set enrichment analysis results.** For each pathway, the table reports the nominal P value, the adjusted P value (FDR), the standard error of the $\log_2$ enrichment score, the enrichment score (ES), the normalized enrichment score (NES) and the number of genes in the pathway.

| Pathway | P-val | Adj. P | Log2err | ES | NES | Size |
|---|---|---|---|---|---|---|
| HALLMARK_OXIDATIVE_PHOSPHORYLATION | 5.94E-37 | 2.97E-35 | 1.58292822 | -0.8029837 | -2.9039976 | 185 |
| HALLMARK_ADIPOGENESIS | 5.36E-10 | 4.17E-09 | 0.80121557 | -0.5331098 | -1.9708045 | 197 |
| HALLMARK_FATTY_ACID_METABOLISM | 5.23E-08 | 2.01E-07 | 0.71951283 | -0.5422263 | -1.9204394 | 153 |
| HALLMARK_MYC_TARGETS_V1 | 5.28E-09 | 2.64E-08 | 0.7614608 | -0.504626 | -1.8144813 | 201 |
| HALLMARK_MYC_TARGETS_V2 | 0.00226377 | 0.00665813 | 0.4317077 | -0.5105206 | -1.6105012 | 58 |
| HALLMARK_BILE_ACID_METABOLISM | 0.00154505 | 0.00482828 | 0.45505987 | -0.4225392 | -1.4766199 | 111 |
| HALLMARK_UNFOLDED_PROTEIN_RESPONSE | 0.01324276 | 0.03484937 | 0.3807304 | -0.3735293 | -1.3020914 | 112 |
| HALLMARK_PEROXISOME | 0.02383805 | 0.05417739 | 0.35248786 | -0.37882 | -1.2942317 | 98 |
| HALLMARK_HEME_METABOLISM | 0.45945946 | 0.60455192 | 0.35481951 | -0.2772463 | -1.003477 | 188 |
| HALLMARK_DNA_REPAIR | 0.99979853 | 0.99979853 | 0.00126246 | 0.2275845 | 0.52197771 | 150 |
| HALLMARK_XENOBIOTIC_METABOLISM | 0.99338545 | 0.99979853 | 0.00136706 | 0.28004476 | 0.64591869 | 201 |
| HALLMARK_E2F_TARGETS | 0.98827068 | 0.99979853 | 0.00173759 | 0.29414714 | 0.67820575 | 200 |
| HALLMARK_SPERMATOGENESIS | 0.96527214 | 0.99979853 | 0.00329214 | 0.29957661 | 0.68178029 | 120 |
| HALLMARK_PROTEIN_SECRETION | 0.88137336 | 0.95801452 | 0.00588568 | 0.344588 | 0.77454952 | 95 |
| HALLMARK_TGF_BETA_SIGNALING | 0.79348757 | 0.88494293 | 0.0085355 | 0.37577767 | 0.8139587 | 53 |
| HALLMARK_PANCREAS_BETA_CELLS | 0.68083874 | 0.79167295 | 0.01208547 | 0.43174263 | 0.87984733 | 29 |
| HALLMARK_GLYCOLYSIS | 0.79644864 | 0.88494293 | 0.00735256 | 0.38361383 | 0.88412048 | 194 |
| HALLMARK_WNT_BETA_CATENIN_SIGNALING | 0.60615453 | 0.72161253 | 0.01311478 | 0.44361625 | 0.93561837 | 40 |
| HALLMARK_MTORC1_SIGNALING | 0.55117794 | 0.67216823 | 0.01305666 | 0.42800569 | 0.98682061 | 199 |
| HALLMARK_NOTCH_SIGNALING | 0.52505721 | 0.65632151 | 0.01566777 | 0.48008219 | 0.98808056 | 32 |
| HALLMARK_G2M_CHECKPOINT | 0.49373056 | 0.6329879 | 0.01465561 | 0.43706884 | 1.00740629 | 197 |
| HALLMARK_ESTROGEN_RESPONSE_LATE | 0.40832915 | 0.55179615 | 0.01741996 | 0.45103529 | 1.0391629 | 188 |
| HALLMARK_ANDROGEN_RESPONSE | 0.37202625 | 0.51670313 | 0.01912194 | 0.47324523 | 1.06608133 | 99 |
| HALLMARK_MYOGENESIS | 0.30597913 | 0.46360475 | 0.02178076 | 0.46677109 | 1.07569403 | 195 |
| HALLMARK_PI3K_AKT_MTOR_SIGNALING | 0.33363989 | 0.4906469 | 0.02072318 | 0.48031196 | 1.08484157 | 102 |
| HALLMARK_REACTIVE_OXYGEN_SPECIES_PATHWAY | 0.34673312 | 0.49533303 | 0.02103338 | 0.50968024 | 1.09432287 | 48 |
| HALLMARK_UV_RESPONSE_UP | 0.19061525 | 0.30744394 | 0.02986088 | 0.49538659 | 1.13664461 | 155 |
| HALLMARK_MITOTIC_SPINDLE | 0.13413534 | 0.2235589 | 0.03671563 | 0.49879517 | 1.15003459 | 199 |
| HALLMARK_HEDGEHOG_SIGNALING | 0.24303912 | 0.37974862 | 0.02752239 | 0.56378953 | 1.17215183 | 35 |
| HALLMARK_UV_RESPONSE_DN | 0.10575177 | 0.18233063 | 0.04217654 | 0.52351159 | 1.19818336 | 143 |
| HALLMARK_KRAS_SIGNALING_DN | 0.08394565 | 0.14990294 | 0.04784367 | 0.52486448 | 1.20527052 | 161 |
| HALLMARK_HYPOXIA | 0.04684522 | 0.09369044 | 0.06521918 | 0.52925892 | 1.21996251 | 196 |
| HALLMARK_COAGULATION | 0.05962607 | 0.11466553 | 0.05764208 | 0.54438331 | 1.24478048 | 136 |
| HALLMARK_APICAL_JUNCTION | 0.01825476 | 0.04346372 | 0.10611052 | 0.5509019 | 1.2697683 | 192 |
| HALLMARK_P53_PATHWAY | 0.01544789 | 0.03861972 | 0.11554753 | 0.55305993 | 1.27482479 | 196 |
| HALLMARK_CHOLESTEROL_HOMEOSTASIS | 0.06734183 | 0.12470708 | 0.0549664 | 0.58046528 | 1.28670497 | 75 |
| HALLMARK_ESTROGEN_RESPONSE_EARLY | 0.00822798 | 0.02285549 | 0.15915346 | 0.56880001 | 1.31051891 | 187 |
| HALLMARK_APICAL_SURFACE | 0.03367561 | 0.07015752 | 0.08140782 | 0.65764998 | 1.39108262 | 41 |
| HALLMARK_EPITHELIAL_MESENCHYMAL_TRANSITION | 0.00031318 | 0.00104395 | 0.49849311 | 0.61089111 | 1.40792978 | 194 |
| HALLMARK_ANGIOGENESIS | 0.02781516 | 0.06046775 | 0.09056177 | 0.67677718 | 1.41257391 | 36 |
| HALLMARK_APOPTOSIS | 2.68E-06 | 9.59E-06 | 0.62725674 | 0.66759796 | 1.53192483 | 158 |
| HALLMARK_IL2_STAT5_SIGNALING | 1.02E-08 | 4.24E-08 | 0.74773966 | 0.68817896 | 1.5862224 | 193 |
| HALLMARK_KRAS_SIGNALING_UP | 3.77E-09 | 2.09E-08 | 0.7614608 | 0.69944281 | 1.6126528 | 199 |
| HALLMARK_COMPLEMENT | 1.33E-09 | 8.33E-09 | 0.78818681 | 0.70415723 | 1.62289635 | 191 |
| HALLMARK_TNFA_SIGNALING_VIA_NFKB | 5.84E-10 | 4.17E-09 | 0.80121557 | 0.70619399 | 1.62771674 | 197 |
| HALLMARK_IL6_JAK_STAT3_SIGNALING | 6.28E-09 | 2.85E-08 | 0.7614608 | 0.78025407 | 1.7443795 | 85 |
| HALLMARK_INTERFERON_ALPHA_RESPONSE | 5.31E-10 | 4.17E-09 | 0.80121557 | 0.78376711 | 1.76552555 | 98 |
| HALLMARK_INFLAMMATORY_RESPONSE | 1.46E-15 | 1.82E-14 | 1.0175448 | 0.76858817 | 1.76987044 | 184 |
| HALLMARK_INTERFERON_GAMMA_RESPONSE | 4.59E-21 | 7.66E-20 | 1.18665095 | 0.79267353 | 1.82828864 | 201 |
| HALLMARK_ALLOGRAFT_REJECTION | 1.21E-25 | 3.02E-24 | 1.31101476 | 0.82360506 | 1.89803324 | 195 |
