# Supplementary material for: LivAge: An Online Aging Clock for Murine Transcriptomic Age Estimation
Source: Aging Cell. 2026 Aug 21;25(9):e70677. doi: 10.1111/acel.70677 (PMC13498547; doi:10.1111/acel.70677)
Supplement: Supplementary file 10 [file ACEL-25-e70677-s010.pdf]

Categoy	Intervention	Bilateral FDR	Unilateral FDR
Genetic	Snell Dwarf	0.000342382	0.000171191
Genetic	Ames Dwarf	0.007976655	0.003988327
Genetic	iGHRKO	0.011324657	0.005662329
Genetic	GHRKO	0.228016108	0.114008054
Lifestyle	Methionine restriction	0.008111222	0.00365005
Lifestyle	Protein restriction	0.033935931	0.015271169
Lifestyle	TreatmentLF	0.064080254	0.028836114
Lifestyle	TreatmentHF + CR	0.064080254	0.028836114
Lifestyle	TreatmentLF + CR	0.064080254	0.028836114
Lifestyle	Normal vs HF diet	0.064080254	0.028836114
Lifestyle	CR (short, 6m)	0.202373627	1
Lifestyle	CR (long, 12m)	0.243768994	0.125366911
Lifestyle	TreatmentLF + Ex	0.685498847	0.347033791
Lifestyle	TreatmentHF + Ex	0.829130986	0.414565493
Pharmacological	Rapamycin (HF diet)	4.99E-05	6.80E-06
Pharmacological	17-alpha-estradiol	0.070373824	1
Pharmacological	Rapamycin (short, 6m)	0.070373824	1
Pharmacological	Ascorbyl-palmitate	0.106893462	1
Pharmacological	AZD8055	0.195513522	1
Pharmacological	Rilmenidine	0.195513522	1
Pharmacological	KU-0063794	0.395887699	1
Pharmacological	Protandim	0.461523508	1
Pharmacological	Metformin	0.496613685	0.294496695
Pharmacological	Rapamycin (long, 12m)	0.647892729	0.294496695
Pharmacological	Acarbose	0.992491536	1
Progeroid	LmnaG609G	0.007771621	0.00388581
Progeroid	Zmpste24-/-	0.007771621	0.00388581
Progeroid	Sprtn Y118C	0.015058089	0.007529045
Progeroid	Terc-/-	0.015380231	0.007690115
