# Supplementary material for: LivAge: An Online Aging Clock for Murine Transcriptomic Age Estimation
Source: Aging Cell. 2026 Aug 21;25(9):e70677. doi: 10.1111/acel.70677 (PMC13498547; doi:10.1111/acel.70677)
Supplement: Supplementary file 11 [file ACEL-25-e70677-s011.pdf]

Supplementary Data 6. Estimated cell-type proportions in liver RNA-seq samples by cellular deconvolution for the external validation set. For each sample, the table reports the sample identifier, chronological age in months, LivAge-estimated transcriptomic age, sex and the estimated proportion of each hepatic cell type (natural killer cell, hepatocyte, endothelial cell of the hepatic sinusoid, Kupffer cell, B cell) as inferred by reference-based deconvolution of the bulk RNA-seq profile.

Sample	C. Age	Sex	Est. Age	NK cell	Hepatocyte	Endothelial cell of hepatic sinusoid	Kupffer cell	B cell
SRR33828225	24	Male	22.47964933	0.028014449	0.675114738	0.248125475	0.022274219	0.026471119
SRR33828226	24	Male	24.42271892	0.026388301	0.681074702	0.242145808	0.021972582	0.028418607
SRR33828227	21	Female	16.68061044	0.019200128	0.664641455	0.265998155	0.02697218	0.023188082
SRR33828228	24	Male	23.87763903	0.025836055	0.719170362	0.214903996	0.015919648	0.024169939
SRR33828229	24	Female	15.80169324	0.017047722	0.619209584	0.272734272	0.065198771	0.02580965
SRR33828230	24	Female	18.8630077	0.037052688	0.466207297	0.264455114	0.168547504	0.063737397
SRR33828231	24	Female	18.97237595	0.023583642	0.631755536	0.25257511	0.060571268	0.031514445
SRR33828232	24	Female	18.40428949	0.054264363	0.558511764	0.256766086	0.049558556	0.080899231
SRR33828233	24	Female	19.69516944	0.024262341	0.654488149	0.263153024	0.036527717	0.021568769
SRR33828234	21	Female	15.87041146	0.021754687	0.643781185	0.270160633	0.042332118	0.021971377
SRR33828235	24	Male	21.18707749	0.042789604	0.661522885	0.24499252	0.016206063	0.034488929
SRR33828236	24	Male	21.19175471	0.096453715	0.191175492	0.221464109	0.341108686	0.149797999
SRR33828237	24	Male	21.55488159	0.052650626	0.66619608	0.235562249	0.020662694	0.02492835
SRR33828240	24	Male	22.83986634	0.022037524	0.705641235	0.233795496	0.016991291	0.021534453
SRR33828241	27	Female	20.55591757	0.045170435	0.618085149	0.28050016	0.042222884	0.014021373
SRR33828242	21	Female	18.14726753	0.017897376	0.670308376	0.254201457	0.027978478	0.029614312
SRR33828244	21	Female	17.25613333	0.027495019	0.628343706	0.265767837	0.057924144	0.020469294
SRR33828245	24	Female	16.63519835	0.010438729	0.68082603	0.253848848	0.034548919	0.020337473
SRR33828239	24	Male	20.93190507	0.024123031	0.693392212	0.228579037	0.027208025	0.026697695
SRR33828243	21	Female	17.30583758	0.007603208	0.703868478	0.247447106	0.020928049	0.020153158
SRR33828396	6	Male	5.567259059	0.016977843	0.747904458	0.195872725	0.014164985	0.025079988
SRR33828397	6	Male	5.725906931	0.020794681	0.735600485	0.207947263	0.015012932	0.02064464
SRR33828399	12	Female	13.76377262	0.020172159	0.65836238	0.257505843	0.033611382	0.030348236
SRR33828400	12	Female	12.5627127	0.021254896	0.673148649	0.250535058	0.023329417	0.03173198
SRR33828401	12	Female	14.60414578	0.02084315	0.678818272	0.252843849	0.026821492	0.020673237
SRR33828402	12	Male	13.15510765	0.014916305	0.742651085	0.21006418	0.011757652	0.020610777
SRR33828404	6	Male	4.981203287	0.010317622	0.74360976	0.216549067	0.012664372	0.016859179
SRR33828405	12	Female	12.99284741	0.012378146	0.686453737	0.237059486	0.031456643	0.032651988
SRR33828407	6	Female	5.981938697	0.021424526	0.702722807	0.232486599	0.020547766	0.022818302
SRR33828413	12	Male	13.97269525	0.019032427	0.743268389	0.205823976	0.012025048	0.01985016

SRR33828415	24	Female	18.59725311	0.012867477	0.666224737	0.264664149	0.030976726	0.025266912
SRR33828416	24	Female	18.28648293	0.017540198	0.688697453	0.243762796	0.020319418	0.029680136
SRR33828417	24	Male	24.56146188	0.016472624	0.708418566	0.226926048	0.017147398	0.031035364
SRR33828419	27	Male	22.4896078	0.066700953	0.472726473	0.256623208	0.127123096	0.076826269
SRR33828422	27	Female	21.31875859	0.029351181	0.618350147	0.268748333	0.052074635	0.031475704
SRR33828423	27	Male	21.23851273	0.016243987	0.709039668	0.224800847	0.027696575	0.022218923
SRR33828425	27	Female	19.05778532	0.038978677	0.632949101	0.275004665	0.031488331	0.021579226
SRR33828426	27	Female	18.87357334	0.022593246	0.639851705	0.265436118	0.029879593	0.042239338
SRR33828427	27	Female	20.97909193	0.022279586	0.601838759	0.27673589	0.055613859	0.043531906
SRR33828428	24	Male	23.57602637	0.040977536	0.634780456	0.238930263	0.05895479	0.026356955
SRR33828429	27	Male	27.1583045	0.037246688	0.635498044	0.21890071	0.063005647	0.045348911
SRR33828430	27	Male	24.14166372	0.036413496	0.507209939	0.285568438	0.098501575	0.072306552
SRR33828431	27	Female	22.33606472	0.029399686	0.613976711	0.265029078	0.060103253	0.031491272
SRR33828432	27	Male	20.76288718	0.088805749	0.53586131	0.247311206	0.045680623	0.082341111
SRR33828403	12	Female	13.5603203	0.026215209	0.683331887	0.239056598	0.025170539	0.026225768
SRR33828406	6	Female	5.686579964	0.014058288	0.692633149	0.251472875	0.019555819	0.022279869
SRR33828412	18	Female	14.81874155	0.021337962	0.669103963	0.25605581	0.022590652	0.030911613
SRR33828418	27	Male	27.18825078	0.041669518	0.607597386	0.269686723	0.044794654	0.036251718
SRR33828421	27	Female	19.24730634	0.02142167	0.603109301	0.256051546	0.072466837	0.046950646
SRR33828434	27	Male	24.99946504	0.045940173	0.514740304	0.233873934	0.083787635	0.121657954
SRR33828435	27	Male	25.10786831	0.0527564	0.63959121	0.249937549	0.021643256	0.036071585
SRR33828437	27	Male	23.7563329	0.075936889	0.564117178	0.268276472	0.052571682	0.039097779
SRR33828510	12	Male	14.73489152	0.018900918	0.738997711	0.209765166	0.011475892	0.020860313
SRR33828511	12	Male	12.78241252	0.014374735	0.742735825	0.21059447	0.01252773	0.01976724
SRR33828512	18	Female	18.93938916	0.014823461	0.679814877	0.260110439	0.025553578	0.019697646
SRR33828514	18	Female	16.20076331	0.025254482	0.668716111	0.254316087	0.023617925	0.028095394
SRR33828515	12	Female	12.43250228	0.013055503	0.664181231	0.268287211	0.031090207	0.023385849
SRR33828517	12	Male	13.0600211	0.024018098	0.73399151	0.210159309	0.008498645	0.023332438
SRR33828518	12	Male	13.37422999	0.021757447	0.736087825	0.209252223	0.010780309	0.022122197
SRR33828519	6	Female	6.293343002	0.011096661	0.695649631	0.253717052	0.021704272	0.017832384
SRR33828520	12	Male	12.27373386	0.020083771	0.732399659	0.206370002	0.012653704	0.028492865

SRR33828521	12	Male	12.49340313	0.015250717	0.734796714	0.215955819	0.013877714	0.020119035
SRR33828522	12	Female	11.43968524	0.018892109	0.690668586	0.246392771	0.023892696	0.020153838
SRR33828523	6	Male	5.758707867	0.019714444	0.742778211	0.20488253	0.008275293	0.024349523
SRR33828524	6	Male	7.09873413	0.017862969	0.741807973	0.209431496	0.010622154	0.020275408
SRR33828525	6	Female	6.625032789	0.017061918	0.69195936	0.252767435	0.017444868	0.02076642
SRR33828526	6	Female	6.59981471	0.016316009	0.703488479	0.235922528	0.024678837	0.019594147
SRR33828527	6	Female	6.584857286	0.015236261	0.686068492	0.259108137	0.022338509	0.0172486
SRR33828528	6	Female	6.464265787	0.009966946	0.715969942	0.240976336	0.017268523	0.015818252
SRR33828529	6	Male	5.813577345	0.024547357	0.729010369	0.202358386	0.018352746	0.025731141
SRR33828530	6	Male	5.346005148	0.009693007	0.739967798	0.217563321	0.012008603	0.020767272
SRR33828531	6	Male	5.643322363	0.011327899	0.738882575	0.215904911	0.015342623	0.018541992
SRR33828532	6	Female	6.634668762	0.021248765	0.694831463	0.234902574	0.025675532	0.023341666
SRR33828533	21	Male	18.76559841	0.047734705	0.687218531	0.221235111	0.011493633	0.03231802
SRR33828534	24	Female	19.19591258	0.007751551	0.688841802	0.256616954	0.029116262	0.01767343
SRR33828535	24	Female	18.30365023	0.011587862	0.686688374	0.253542819	0.032006391	0.016174555
SRR33828536	27	Female	19.26348538	0.017947351	0.647988776	0.27700846	0.035603593	0.021451819
SRR33828537	27	Female	19.11996193	0.016405584	0.670349083	0.250772049	0.034765557	0.027707727
SRR33828631	18	Male	17.73370725	0.019651316	0.713717258	0.221191522	0.024325744	0.02111416
SRR33828632	18	Male	19.52588737	0.031303398	0.689069043	0.226129982	0.028210779	0.025286798
SRR33828633	21	Male	18.35477629	0.042168912	0.611742973	0.246951589	0.042927887	0.05620864
SRR33828634	21	Male	19.33447855	0.052738004	0.673847569	0.227790886	0.012625834	0.032997707
SRR33828635	21	Male	20.87976566	0.039322442	0.704233804	0.21760256	0.010876851	0.027964343
SRR33828636	18	Male	16.22146364	0.037069002	0.70091436	0.219080821	0.011182006	0.031753811
SRR33828637	18	Male	18.49014126	0.049496307	0.735160074	0.185477157	0.008051215	0.021815247
SRR33828638	21	Female	18.8680325	0.019778437	0.672279217	0.244553683	0.031601121	0.031787541
SRR33828639	21	Female	17.6152913	0.022133245	0.667337701	0.248753974	0.038915914	0.022859166
SRR33828640	21	Female	19.27557781	0.024649277	0.625511735	0.269213079	0.033044421	0.047581488
SRR33828642	21	Male	23.13886311	0.030509985	0.685985981	0.233848732	0.022376904	0.027278398
SRR33828643	21	Male	23.81520435	0.036183515	0.678096066	0.227705516	0.020990328	0.037024575
SRR33828645	18	Female	16.39325567	0.018298953	0.683354787	0.255149272	0.019967698	0.02322929
SRR33828646	18	Female	15.88121471	0.021942853	0.688547069	0.243660067	0.02411288	0.021737131

SRR33828647	18	Female	17.77211898	0.018279269	0.663193827	0.269017371	0.025498257	0.024011276
SRR33828648	18	Male	19.40965899	0.030612865	0.729935212	0.196119664	0.015966813	0.027365447
SRR33828649	18	Male	19.3382027	0.014732793	0.75240134	0.199734008	0.012055408	0.021076451
SRR33828650	18	Male	18.38056892	0.018564312	0.707791762	0.233571234	0.014319922	0.025752771
SRR33828651	18	Female	16.39120499	0.01675077	0.678848671	0.254773421	0.027148544	0.022478594
SRR33828652	21	Male	19.82151233	0.075068226	0.595996456	0.254303628	0.024741792	0.049889898
SRR33828653	18	Female	13.25781146	0.008531207	0.696697292	0.255216716	0.019979089	0.019575696
SRR33828654	18	Male	17.96832487	0.020145218	0.715142701	0.21974695	0.024635011	0.02033012
SRR28812615	12	Female	13.26987502	0.012070187	0.783502203	0.113641263	0.087383173	0.003403174
SRR28812616	12	Female	11.25162411	0.020388701	0.81863033	0.115486317	0.036521398	0.008973256
SRR28812617	12	Female	11.63142384	0.019109104	0.786698075	0.130660301	0.042710766	0.020821753
SRR28812618	12	Female	15.04984941	0.018587592	0.791071981	0.123155553	0.059893861	0.007291014
SRR36039785	2	Female	2.431490328	0.023246732	0.734365884	0.191429442	0.022036485	0.028921456
SRR36039786	2	Female	2.212721893	0.018159062	0.783819522	0.16275064	0.02175098	0.013519795
SRR36039787	2	Female	2.240050015	0.024128819	0.772505822	0.15919913	0.030183802	0.013982427
SRR36039788	2	Female	2.113409958	0.023591906	0.749305861	0.179003411	0.026690435	0.021408388
SRR36039789	2	Female	2.088373772	0.020432894	0.750377238	0.175301462	0.036673901	0.017214505
SRR36039790	2	Female	2.077957503	0.020595607	0.771948677	0.161356935	0.031502321	0.01459646
SRR28812619	12	Female	10.87688567	0.011583097	0.831461236	0.103302367	0.046691804	0.006961496
SRR28812654	18	Female	16.01588875	0.010013396	0.828792031	0.112635295	0.044255452	0.004303825
SRR28812655	18	Female	17.60412556	0.007877704	0.742389819	0.113479519	0.095488944	0.040764014
SRR28812656	18	Female	15.33007292	0.011316022	0.785385192	0.09418721	0.102036182	0.007075394
SRR28812657	18	Female	17.58937382	0.008338616	0.820388286	0.065214845	0.102791762	0.003266492
SRR28812658	18	Female	13.94987672	0.013450556	0.792217737	0.09545925	0.072616919	0.026255538
SRR28812670	7	Female	7.367639349	0.012958945	0.819640127	0.120300925	0.039561245	0.007538758
SRR28812671	7	Female	8.714851507	0.016112363	0.826508521	0.105715998	0.038826638	0.01283648
SRR28812672	7	Female	8.165136168	0.015592787	0.83363434	0.103741051	0.036138488	0.010893334
SRR28812673	7	Female	8.869511379	0.020088545	0.823520719	0.103122477	0.038529022	0.014739236
SRR28812674	7	Female	8.720250539	0.018649465	0.861085092	0.07926374	0.022735396	0.018266308
SRR28812675	7	Female	9.5526876	0.026764347	0.823147858	0.086376923	0.046994214	0.016716658
SRR32335461	10	Female	9.949981154	0.012276189	0.862172327	0.100515381	0.015105785	0.009930318

SRR32335462	10	Female	10.54353554	0.012301302	0.863282191	0.09958065	0.015535164	0.009300692
SRR32335463	10	Female	9.231676433	0.014502761	0.846962033	0.101725853	0.028189643	0.00861971
SRR32335464	10	Female	10.04078884	0.013461994	0.847774007	0.102563414	0.027464534	0.008736051
SRR32335465	10	Female	9.545312767	0.016561975	0.837357093	0.114584592	0.020466449	0.011029891
SRR32335467	10	Female	10.43993732	0.012663647	0.853740159	0.10241832	0.023034376	0.008143498
SRR32335469	10	Female	10.22031964	0.014056081	0.832915882	0.107861524	0.036846119	0.008320394
SRR32335471	10	Female	9.121252836	0.018941194	0.840950892	0.105590031	0.023690034	0.010827849
SRR32335466	10	Female	10.42174417	0.016692233	0.839856167	0.110925569	0.020550122	0.011975909
SRR32335468	10	Female	11.10163171	0.012109351	0.851263024	0.102750746	0.023368107	0.010508772
SRR32335470	10	Female	10.24312264	0.014154882	0.832166027	0.108703247	0.037171283	0.007804561
SRR32335472	10	Female	8.719131353	0.017872107	0.838233306	0.106182278	0.024590313	0.013121996
