# Supplementary material for: LivAge: An Online Aging Clock for Murine Transcriptomic Age Estimation
Source: Aging Cell. 2026 Aug 21;25(9):e70677. doi: 10.1111/acel.70677 (PMC13498547; doi:10.1111/acel.70677)
Supplement: Supplementary file 13 [file ACEL-25-e70677-s013.pdf]

Table S1. Summary of RNA-seq datasets used in the training and validation of LivAge. For each study, the table reports the accession number (from NCBI or CNCB), the number of samples employed, the genetic background of the mice, the first author, and the year of publication. The role of each dataset is also indicated, distinguishing between datasets employed for repeated cross-validation (RCV) during model training and testing, those used for optimization of the predictive model through the evaluation of gene expression and chronological age transformations, and those used for external validation of the transcriptomic aging clock.

Accession	N	Strain	Reference	Purpose
PRJNA1090973	52	C57BL/6J/Ukj	Best et al., 2025	Train and Test (RCV)
PRJNA544748	51	C57BL/6JN	Schaum et al., 2020	Train and Test (RCV)
PRJNA788968	48	C57BL/6JN	Acosta-Rodríguez et al., 2022	Train and Test (RCV)
PRJNA610793	37	C57BL/6	Wang et al., 2018	Train and Test (RCV)
PRJNA592965	30	C57BL/6J	Stoeger T et al., 2021	Train and Test (RCV)
PRJCA002140	25	C57BL/6J	Zhou et al., 2020	Train and Test (RCV)
PRJNA302755	25	C57BL/6	Aramillo Irizar et al., 2018	Train and Test (RCV)
PRJNA885815	24	C57BL/6J	Koritala et al., 2023	Train and Test (RCV)
PRJNA1233337	21	C57BL/6J	Morsy S et al., 2026	Train and Test (RCV)
PRJNA936435	16	C57BL/6 J	Takasugi etal., 2024	Train and Test (RCV)
PRJNA636375	12	C57BL/6	Aronson JK, 2020	Train and Test (RCV)
PRJNA599950	12	C57BL/6	Katz et al., 2020	Train and Test (RCV)
PRJNA395093	10	C57BL/6	Newman JC, 2017	Train and Test (RCV)
PRJNA281127	9	C57BL/6	Benayoun etal., 2019	Train and Test (RCV)
PRJNA1167759	8	C57BL/6J	Best et al., 2025	Train and Test (RCV)
PRJNA237291	8	C57BL/6	R. Zhang et al., 2014	Train and Test (RCV)
PRJNA982056	8	C57BL	Z. Zhang etal., 2023	Train and Test (RCV)
PRJNA789246	7	C57BL/6	Browder et al., 2022	Train and Test (RCV)
PRJNA941888	6	C57BL/6N	Zhen Y et al., 2023	Train and Test (RCV)
PRJNA237825	6	C57BL/6	Weimer et al., 2014	Train and Test (RCV)
PRJNA503413	6	C57BL/6	Guerrero et al., 2019	Train and Test (RCV)
PRJNA770539	6	C57BL/6JN	Yang et al., 2023	Train and Test (RCV)
PRJNA762912	5	C57BL/6J	Graca FA et al., 2023	Train and Test (RCV)
PRJNA510416	18	C57BL/6	Anisimova AS et al., 2020	Optimization
PRJNA636378	3	C57BL/6J	Morton J et al. 2020	Optimization
PRJNA248136	3	C57BL/6	Bochkis IM et al., 2014	Optimization
PRJNA831822	3	C57BL/6	Wang Set al., 2022	Optimization
PRJNA288820	3	C57BL/6	Yang G et al., 2016	Optimization
PRJNA428303	3	C57BL/6J	Quagliarini Fetal., 2019	Optimization
PRJNA541932	3	C57BL/6	Pan Y et al. 2020	Optimization
PRJNA560507	3	C57BL/6	Weger BD et al., 2021	Optimization
PRJNA1250527	100	C57BL/6J	Shavlakadze T et al., 2026	External validation
PRJNA1104985	16	C57BL/6	Batista MN et al., 2026	External validation
PRJNA1223435	12	C57BL/6J	Picard E et al., 2025	External validation
PRJNA1365013	6	C57BL/6	Lee HK et al, 2025	External validation
